# Adaptive mutations of neuraminidase stalk truncation and deglycosylation confer enhanced pathogenicity of influenza A viruses

**DOI:** 10.1038/s41598-017-11348-0

**Published:** 2017-09-07

**Authors:** Sehee Park, Jin Il Kim, Ilseob Lee, Joon-Yong Bae, Kirim Yoo, Misun Nam, Juwon Kim, Mee Sook Park, Ki-Joon Song, Jin-Won Song, Sun-Ho Kee, Man-Seong Park

**Affiliations:** Department of Microbiology, and the Institute for Viral Diseases, College of Medicine, Korea University, Seoul, 02841 Republic of Korea

## Abstract

It has been noticed that neuraminidase (NA) stalk truncation has arisen from evolutionary adaptation of avian influenza A viruses (IAVs) from wild aquatic birds to domestic poultry. We identified this molecular alteration after the adaptation of a 2009 pandemic H1N1 virus (pH1N1) in BALB/c mice. The mouse-adapted pH1N1 lost its eight consecutive amino acids including one potential *N*-linked glycosite from the NA stalk region. To explore the relationship of NA stalk truncation or deglycosylation with viral pathogenicity changes, we generated NA stalk mutant viruses on the pH1N1 backbone by reverse genetics. Intriguingly, either NA stalk truncation or deglycosylation changed pH1N1 into a lethal virus to mice by resulting in extensive pathologic transformation in the mouse lungs and systemic infection affecting beyond the respiratory organs in mice. The increased pathogenicity of these NA stalk mutants was also reproduced in ferrets. In further investigation using a human-infecting H7N9 avian IAV strain, NA stalk truncation or deglycosylation enhanced the replication property and pathogenicity of H7N9 NA stalk mutant viruses in the same mouse model. Taken together, our results suggest that NA stalk truncation or deglycosylation can be the pathogenic determinants of seasonal influenza viruses associated with the evolutionary adaptation of IAVs.

## Introduction

Influenza virus circulates seasonally among humans^1, 2^. Successfully transmitting from person to person, the virus causes approximately three to five million human infections per year, which of almost 10% would be the fatal cases^3, 4^. Two kinds of medical countermeasures, such as vaccine and antiviral drug, are currently available for human use against influenza^5, 6^. However, some drawbacks of the vaccines and antivirals should be resolved for their continued use^6–9^.

When new approaches of vaccine and antiviral developments have been sought for, their effectiveness and safety should be assessed in an animal model, mainly in mice, prior to clinical trials in humans^10^. However, most human influenza virus strains are often sub-lethal to mice. Due to this, preparing a properly pathogenic virus to mice is one of the indispensable prerequisites for the development of new medical intervention methods^11^. Several strategies have been suggested^12–14^, and of these, adaptation of a certain virus strain by multiple lung-to-lung passages in mice may be the most frequently chosen method^15^. After the serial adaptation in mice, a more virulent strain can be recovered, and molecular transformations arisen from the mouse-adapted strain would be indicators associated with the host adaptation of IAVs^16–18^.

NA stalk truncation has been suggested for its association with the adaptation of avian IAVs from wild aquatic birds to domestic poultry species^19, 20^. It was reported previously in the NAs of avian influenza H2N2, H5N1, H6N1, H7N1, H7N3, and H9N2 subtype viruses^20–26^. More recently, the NA stalk truncation was also recognized in H7N9 human strains, which have been posing severe public health threats in China since 2013^27^. Intriguingly, the NA stalk truncation appears related with the pathogenicity increases of avian IAVs^19, 20, 24, 25, 28–31^, and some of these studies noted the pathogenic contribution of *N*-linked glycosylation (NLG) pattern changes in the globular head region of hemagglutinin (HA) protein^20, 24^. In fact, the NLG pattern changes in the HA of IAVs have been suggested for their differential effects on viral antigenicity, pathogenicity, transmissibility, and/or immune evasion from host immunity^14, 32, 33^. Given that the NA stalk truncation also includes at least one NLG site^21, 24, 28^ and that the functional balance between the HA and NA proteins is of great importance in terms of viral fitness^34, 35^, genetic alterations affecting the NA stalk truncation and the number of NLG in the truncated NA stalk region may be closely associated with the host adaptation of IAVs.

In this study, we adapted the K/09 virus in mice by multiple rounds of lung-to-lung passage after initial intranasal infection. We then recovered a mouse-adapted (*ma)* virus and identified its amino acid mutations. Of these amino acid mutations, we focused on the truncation of eight consecutive amino acids including one potential NLG site in the NA stalk region because this adaptive mutation has been reported mostly in the NAs of avian IAVs, not often in those of human seasonal strains previously. We then investigated the effects of NA stalk truncation or deglycosylation on viral pathogenicity in mice and ferret models. We also explored the effects of NA stalk truncation or deglycosylation on the pathogenicity of the H7N9 human strain in mice.

## Results

### Pathogenicity and genetic mutations of the mouse-adapted K/09 virus

**Figure 1:**
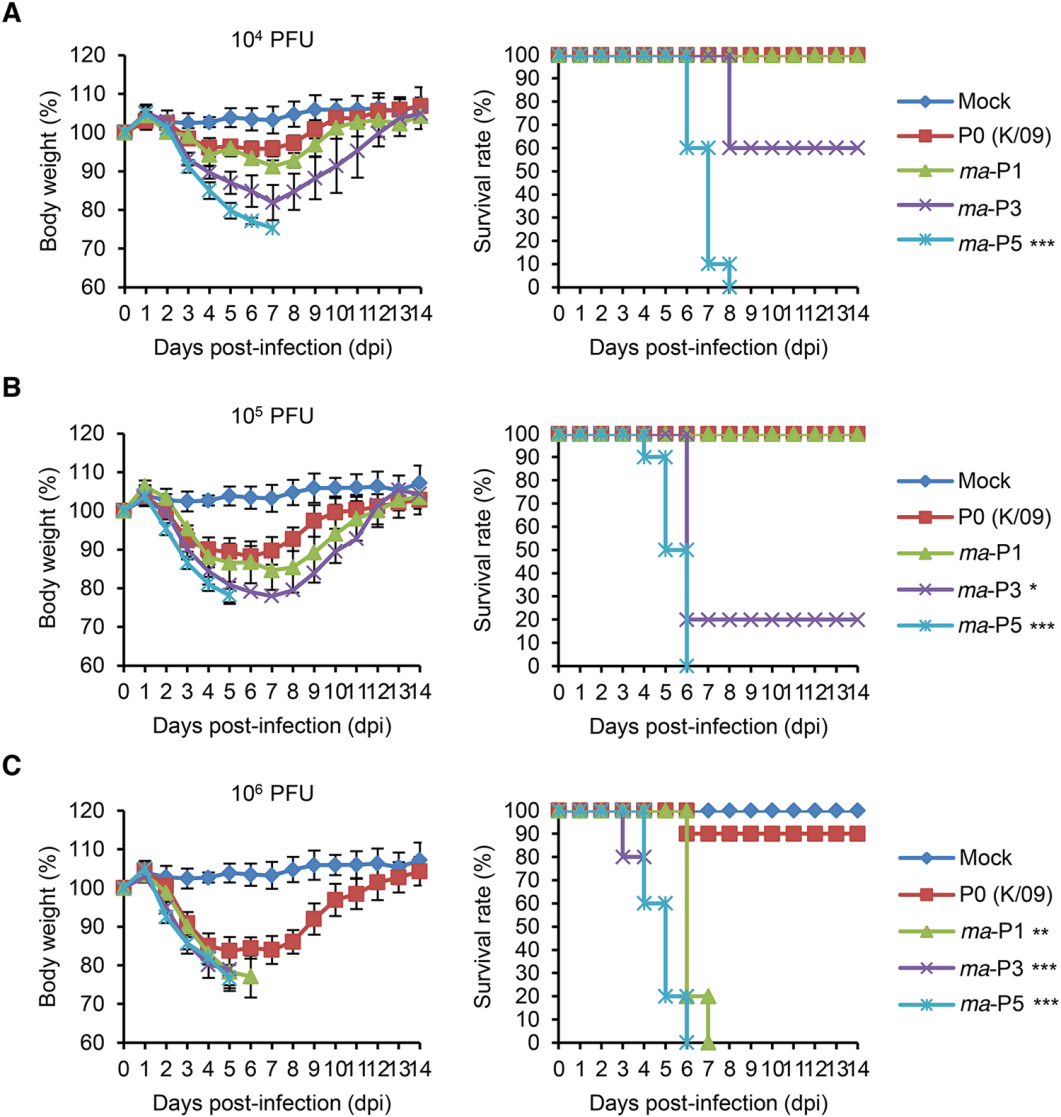
Pathogenicity of the K/09 and its mouse-adapted viruses in mice. Averaged body weight changes and survival rates of BALB/c mice (n = 10 per group) after the intranasal infection (10^4^, 10^5^, and 10^6^ PFU) with the wild-type K/09 (P0) and its mouse-adapted (*ma*-P1, -P3, and -P5) viruses were represented in average. Mice in the mock group were infected with PBS. Error bars denote standard deviation (SD). ^*^ *P < *0.05, ^**^ *P* *<* 0.01, and ^***^ *P* *<* 0.001 (compared with the K/09 infection group).

After the first, third, and fifth rounds of lung-to-lung passage in BALB/c mice, we recovered the *ma*-P1, -P3, and -P5 viruses from the lung homogenates, respectively. In the same mouse model, the *ma*-P1, -P3, and -P5 viruses exhibited increased pathogenicity (Fig. 1) by resulting in approximately 15.38%, 22.03%, and 21.87% weight losses in maximum at 7, 7, and 5 days post-infection (dpi), respectively, with a 10^5^ plaque forming unit (PFU) titer whereas the parental K/09 virus only caused an averaged 11.79% weight loss (Table 1). The *ma*-P3 and -P5 viruses killed more than 40% and 80% of the infected mice with 10^4^ and 10^5^ PFU titers, respectively (Fig. 1A and B), and their mean day of death (MDD) was lower than 8 with a 10^5^ PFU titer (Table 1). The infected mice with a 10^6^ PFU titer of *ma*-P1, *ma*-P3, and *ma*-P5 were all dead before 8 dpi (Fig. 1C). Based on the body weight changes and survival rates of the infected mice (Fig. 1A to C), 50% mouse lethal dose (MLD_50_) titers of the *ma*-P1, -P3, and -P5 viruses were determined as 10^5.5^, 10^4.1^, and 10^2.7^ PFU, respectively (Table 1).

**Table 1:** Pathological findings from the mice infected with the K/09 and *ma* viruses.

Virus	MLD_50_	% of body weight loss^a^ (dpi)	MDD^b^
MOCK	—	—	—
P0 (K/09)	>10^6.5^	11.79 ± 2.71 (6)	14.0 ± 0
*ma*-P1	10^5.5^	15.38 ± 5.08 (7)	14.0 ± 0
*ma*-P3	10^4.1^	22.03 (7)	7.6 ± 3.6
*ma*-P5	10^2.7^	21.87 ± 1.74 (5)	5.4 ± 0.7

^a^The mean maximum body weight losses and ^*b*^mean day of death (MDD) were obtained from the mice infected with 10^5^ PFU. Each result was expressed in average with ± standard deviations (SD).

To identify molecular alterations that transformed the K/09 virus into a lethal, *ma* virus, we compared the genetic sequences of eight genetic segments between K/09 and *ma*-P5. It was then revealed that *ma*-P5 harbored amino acid substitutions in the five viral protein coding regions (based on the numbering of K/09 amino acid residues; PB1, D193E and G710R; PA, M21I and L511V; HA, D144E and I283V; NA, truncation of the eight amino acids between the residues 53 and 60; and M1, A206S) (Table 2), and the amino acid substitutions in the PB1 and PA proteins appeared to be novel mutations that have been never reported in the human H1N1 strains (Table S1). Of these, it was the NA stalk truncation including one potential NLG site that we paid our attention to because it has been considered as the evolutionary adaptive molecular alteration of avian IAVs from migratory birds to domestic poultry^19, 20^.

**Table 2:** Amino acid changes between the K/09 and *ma*-P5 viruses.

Protein coding region	Residue^a^	Amino acid signature	
		P0 (K/09)	*ma*-P5
PB1	193	D	E
	710	G	R
PA	21	M	I
	511	L	V
HA	144	D	E
	283	I	V
NA	53–60	VITYENNT	Truncated
M1	206	A	S

^a^Based on K/09 numbering.

### Effects of NA stalk mutations on enzymatic activity and viral replication property in cells

**Figure 2:**
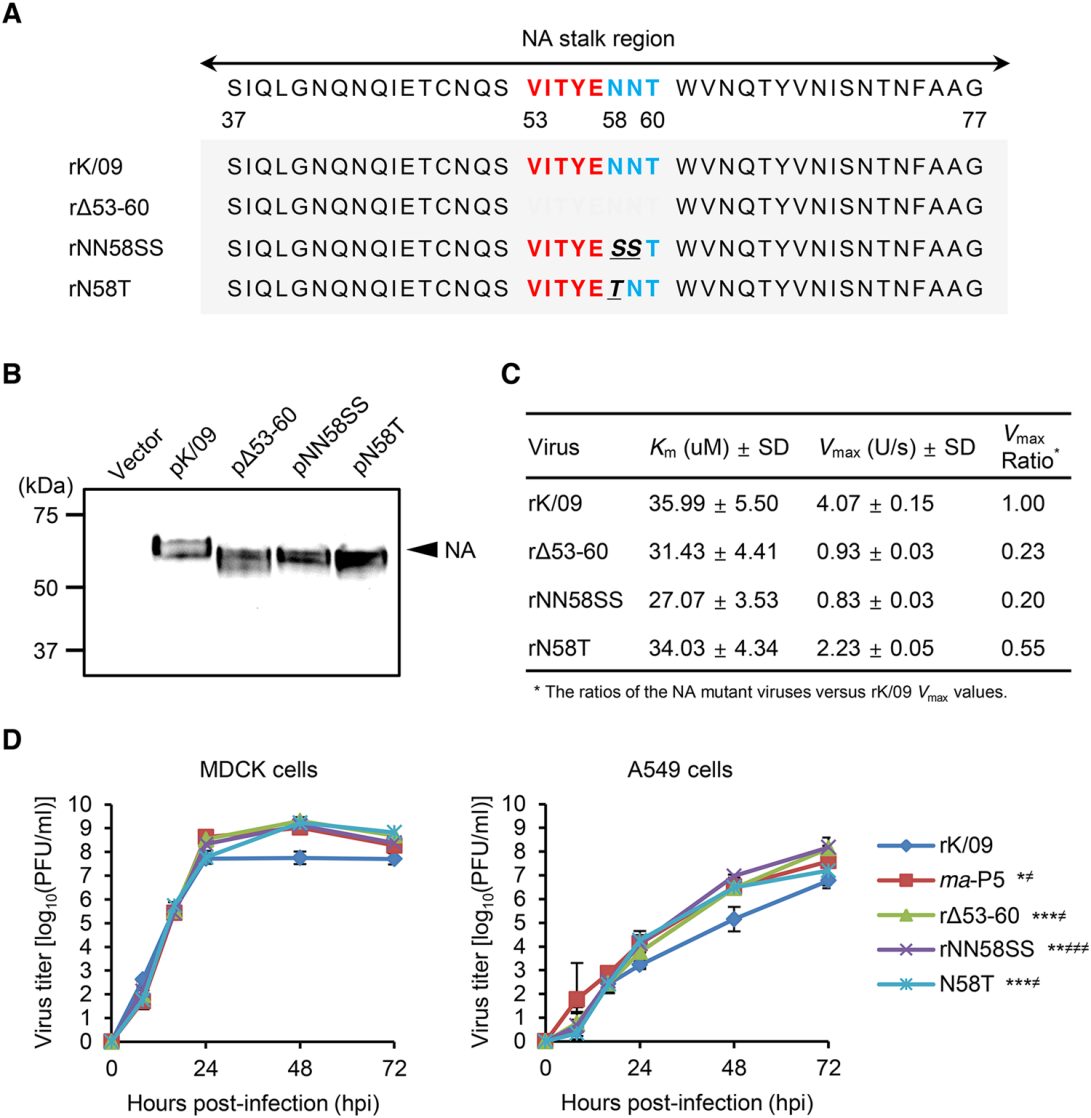
*In vitro* evaluation of the rK/09 and its NA stalk mutant viruses. (**A**) rK/09 and its NA stalk mutant viruses (rΔ53–60, rNN58SS, and rN58T) were generated by reverse genetics based on the NA stalk truncation or deglycosylation strategy. (**B**) In the western blotting assay, NA stalk mutations (truncation and deglycosylation) were confirmed based on the mobility shift of NA proteins. (**C**) The enzyme kinetics data were fit to the Michaelis-Menten equation by nonlinear regression to determine the Michaelis constant (*K*_*m*_) and maximum velocity (*V*_*max*_) of substrate conversion. Results were given as the means ± SD from three independent determinations on triplicate samples where the *R* ^2^ was > 0.99. (**D**) Replication properties of each virus were evaluated in MDCK and A549 cells at a multiplicity of infection (MOI) of 0.001. The results were represented as the mean values from three independent experiments. Error bars denote SD. ^*^ *P* < 0.05, ^**^ *P* < 0.01, and ^***^ *P* < 0.001 for the results assessed in the MDCK cells and ^≠^ *P* < 0.05 and ^≠≠≠^ *P* < 0.001 in the A549 cells (compared with the rK/09 replication titers).

In the K/09 NA stalk region, there are several potential NLG sites, and asparagine at NA residue 58 is one of them (Fig. 2A). However, it was removed from *ma*-P5 by the NA stalk truncation (Table 2). To investigate the effects of NA stalk truncation or deglycosylation on the pathogenicity of IAVs to mice, we generated a stalk-truncated (rΔ53–60) or deglycosylated (rN58T) mutant virus on the K/09 backbone. Based on the NA sequences of human H1N1 strains of A/Melborne/1935 (GenBank accession no.: CY009326) and A/Melbourne/1946 (CY045774), which appeared to contain no NLG at NA residue 58, we additionally generated another deglycosylated mutant virus, rNN58SS (Fig. 2A). After confirming the mobility shift of the NA proteins of rΔ53–60, rNN58SS, and rN58T, compared with that of rK/09, in a western blotting assay (Fig. 2B), we examined the NA enzymatic dynamics of these viruses. The relative enzymatic activity (*V*_max_ value) of the NA stalk mutant viruses decreased to almost a half (rN58T, 2.23 ± 0.05) or more than three fourths (rΔ53–60, 0.93 ± 0.03 and rNN58SS, 0.83 ± 0.03) of the rK/09 NA activity (4.07 ± 0.15) with similar ranges of the relative affinity (*K*_m_ values, 27.07 ± 3.53 to 35.99 ± 5.50) for substrates (Fig. 2C). Compared with those of rK/09, however, all the NA mutant and *ma*-P5 viruses demonstrated their enhanced replication properties in both Madin-Darby canine kidney (MDCK) and human lung epithelial (A549) cells (Fig. 2D). These suggest NA stalk truncation or deglycosylation may be the molecular alterations to increase the replication property of the pH1N1 virus in cells regardless of its negative effects on NA enzymatic activity.

### Pathogenicity of the NA stalk mutant viruses in mice

**Figure 3:**
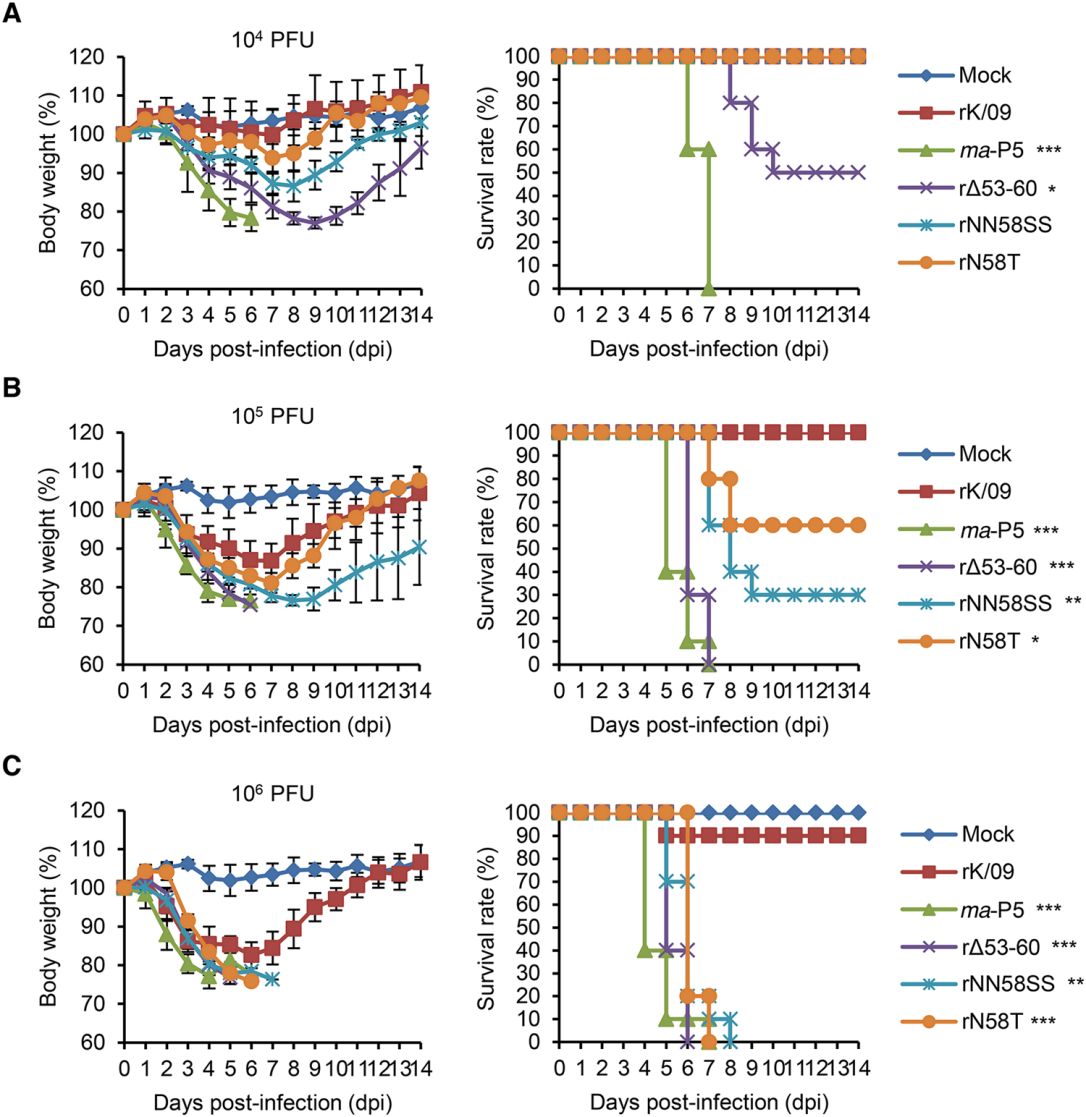
Body weight changes and survival rates of the mice infected with the rK/09 and its NA stalk mutant viruses. Ten mice per group were intranasally infected with (**A**) 10^4^, (**B**) 10^5^, and (**C**) 10^6^ PFU of the rK/09, *ma*-P5, rΔ53–60, rNN58SS, and rN58T viruses, respectively. Body weight changes and survival rates of the infected mice were represented in average. Mice in the mock group were infected with PBS. Error bars denote SD. (^*^ *P* < 0.05, ^**^ *P* < 0.01, and ^***^ *P* < 0.001 compared with the results of rK/09 infection group).

We observed that the NA stalk truncation or the removal of a potential NLG site increased the replication property of rK/09 in cells. Similarly, the NA stalk mutations resulted in the increases of viral pathogenicity in mice (Fig. 3A to C). Given the number of experimental death of the infected mice, rΔ53–60 appeared to be the most pathogenic virus among the NA stalk mutant viruses. It resulted in more than 24% body weight loss from the infected mice at 6 dpi (Table 3) and killed them even with a 10^4^ PFU infection titer (Fig. 3A). Based on the body weight changes and survival rates of the infected mice, the MLD_50_ (rΔ53–60, 10^3.9^; rNN58SS, 10^4.7^; and N58T, 10^5.1^ PFU) and the MDD (rΔ53–60, 6.3 ± 0.48; rNN58SS, 9.5 ± 3.17; and N58T, 11.4 ± 3.57) values of the NA stalk mutant viruses were determined (Table 3).

**Table 3:** Pathological findings from the mice infected with the K/09 and its NA stalk mutant viruses.

Virus	MLD_50_	% of body weight loss^a^ (dpi)	MDD^b^
MOCK	—	—	—
rK/09	>10^6.5^	12.70 ± 4.98 (6)	14.0 ± 0
rΔ53–60	10^3.9^	24.45 ± 0.17 (6)	6.3 ± 0.48
rNN58SS	10^4.7^	23.40 ± 1.32 (8)	9.5 ± 3.17
rN58T	10^5.1^	18.90 ± 3.23 (7)	11.4 ± 3.57

^a,b^Please see footnotes in Table 1.

**Figure 4:**
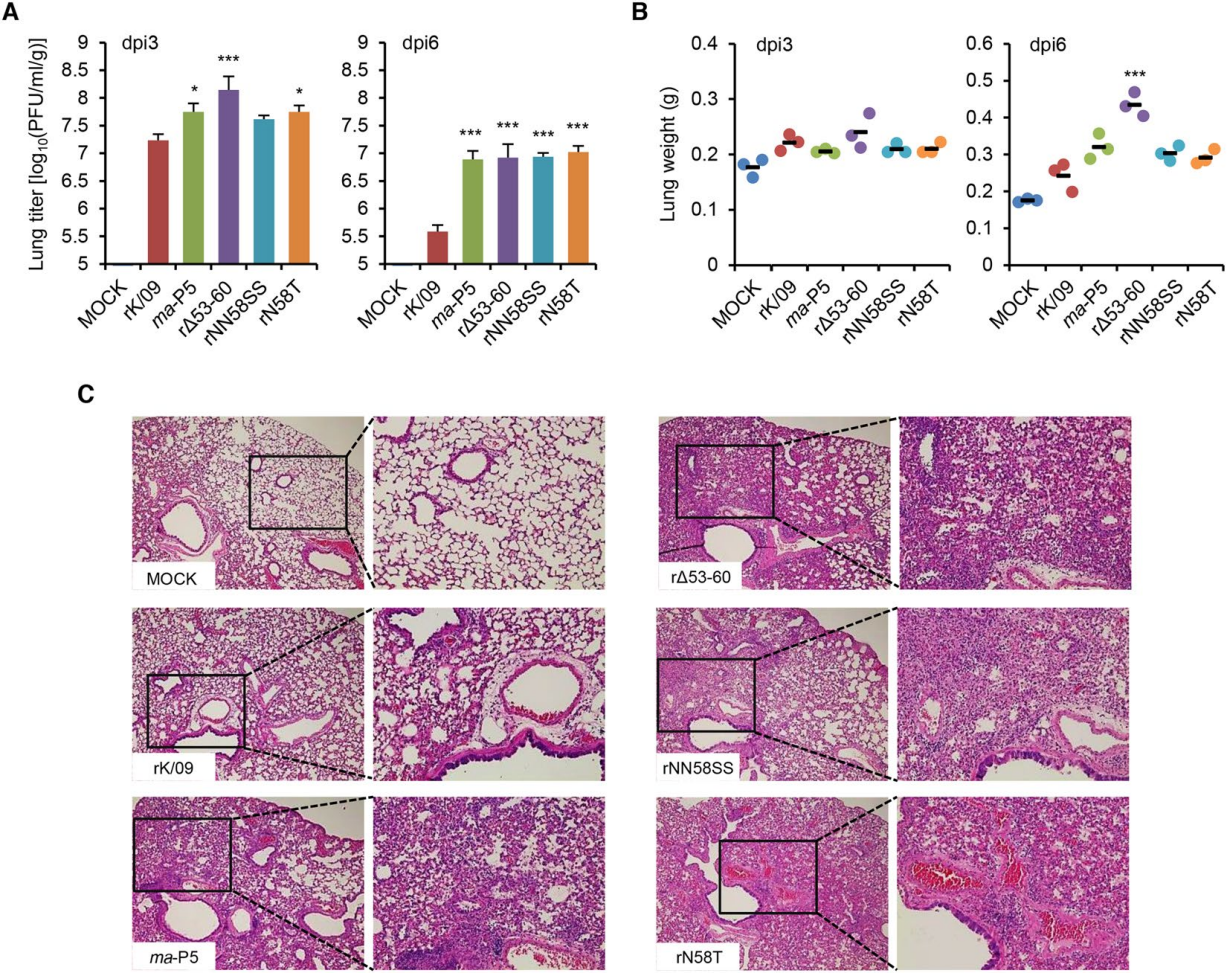
Virus replication and histopathological examination of the lungs of the infected mice. Three mice per group were infected intranasally with 10^5^ PFU of each virus and euthanized at 3 and 6 dpi, respectively, for internal body organ collection from the infected mice. Viral titers in the lungs (**A**) and lung weights (**B**) of the infected mice were represented in average. Error bars denote SD (^*^ *P* < 0.05 and ^***^ *P* < 0.001 compared with the rK/09 results). (**C**) Lung histopathology of the mice infected with rK/09, *ma*-P5, rΔ53–60, rNN58SS, and rN58T were evaluated using the H&E slides.

Viral replication property was evaluated in mice. In the lungs of the infected mice, the parental rK/09 virus replicated up to 10^7.2^ PFU/ml/g at 3 dpi (Fig. 4A). All of the NA stalk mutant viruses and *ma*-P5 showed higher replication properties than rK/09 at 3 and 6 dpi (Fig. 4A). Of the NA stalk mutant viruses, rΔ53–60 and rN58T exhibited the highest replication titers at 3 and 6 dpi, respectively (Fig. 4A). In addition, the lung weights of mice infected with the NA stalk mutant viruses and *ma*-P5 all increased, compared with those infected with rK/09 (Fig. 4B). To confirm the pathogenicity increases of the NA stalk mutant viruses, we then investigated the histopathologic changes in the infected mouse lungs. Compared with rK/09, *ma*-P5 that killed all of the infected mice only with a 10^4^ PFU titer (Fig. 1A) severely destructed alveoli and bronchioles by causing extensive hemorrhage and inflammation in most parts of the infected mouse lungs (Fig. 4C). Similarly, rΔ53–60, rNN58SS, and rN58T caused severe hemorrhage and inflammation, and the normal histologic structures were barely noticeable in the infected mouse lungs (Fig. 4C).

**Figure 5:**
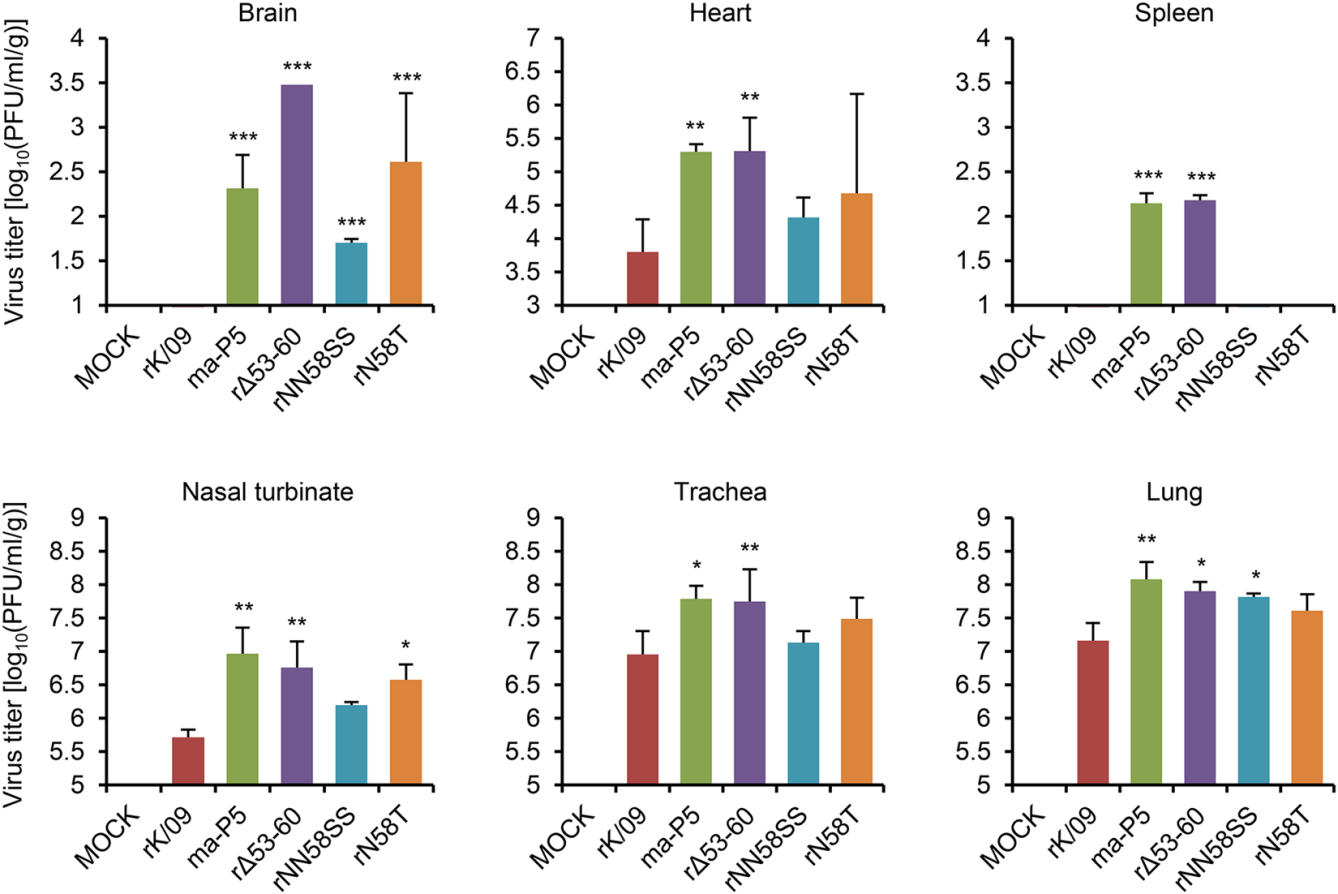
Viral replication in the respiratory and other internal body organs in the infected mice. After 10^6^ PFU of intranasal infection, viral replication property was investigated in the respiratory (nasal turbinate, trachea, and lung) and other internal body organs (brain, heart, and spleen) of the infected mice. The results were represented in average, and error bars denote SD (^*^ *P* < 0.05, ^**^ *P* < 0.01, and ^***^ *P* < 0.001 compared with the rK/09 results).

**Figure 6:**
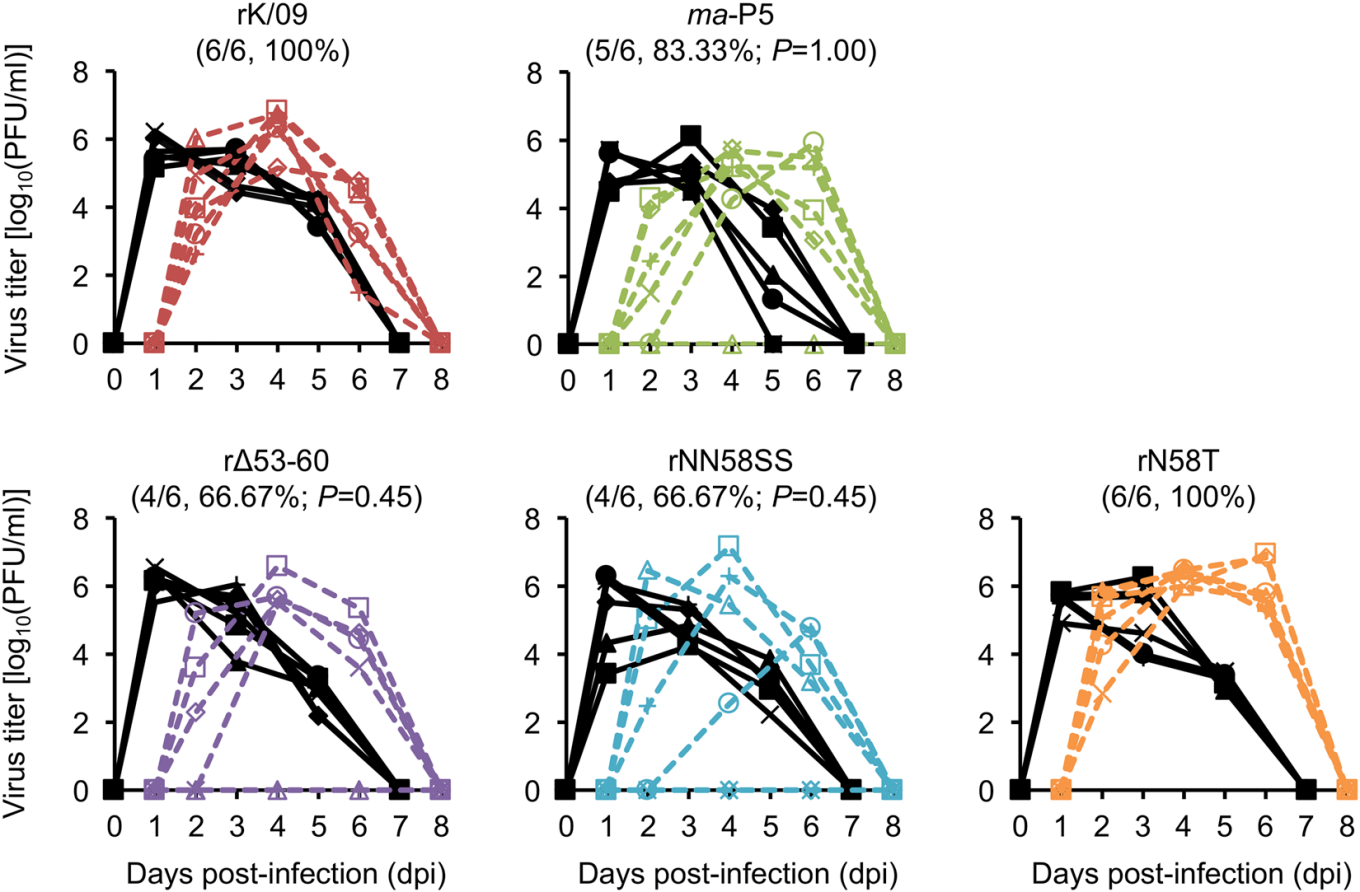
Transmissibility of the rK/09 and its NA stalk mutant viruses in guinea pigs. Transmission efficiency of rK/09, *ma*-P5, rΔ53–60, rNN58SS, and rN58T was examined in guinea pigs. Solid lines represent the nasal wash titers of infected guinea pigs, and dotted lines represent those of contacts. Transmission efficiency of each virus group is indicated in parenthesis as the ratio of the number of contact guinea pigs versus the number of infected guinea pigs.

We also evaluated whether the NA stalk mutant viruses could extend their pathogenicity to other internal body organs of the infected mice. In this systemic infection analysis, the parental rK/09 virus replicated only in the respiratory organs (nasal turbinate, trachea, and lung) and the heart. In contrast, the NA stalk mutant viruses were isolated from the brain of the infected mice, and rΔ53–60 exhibited its replication capacity in the spleen as *ma*-P5 did (Fig. 5). In contrast to their systemic infectivity, both rΔ53–60 and rNN58SS viruses exhibited reduced transmissibility in guinea pigs by resulting in only 66.67% contact transmission rate whereas another NA stalk deglycosylation mutant rN58T showed 100% transmission (Fig. 6). Considered together, our results suggest that NA stalk truncation or deglycosylation, which affects viral transmissibility, may substantially increase the pathogenicity of the pH1N1 virus in mice.

### Pathogenicity of the NA stalk mutant viruses in ferrets

**Figure 7:**
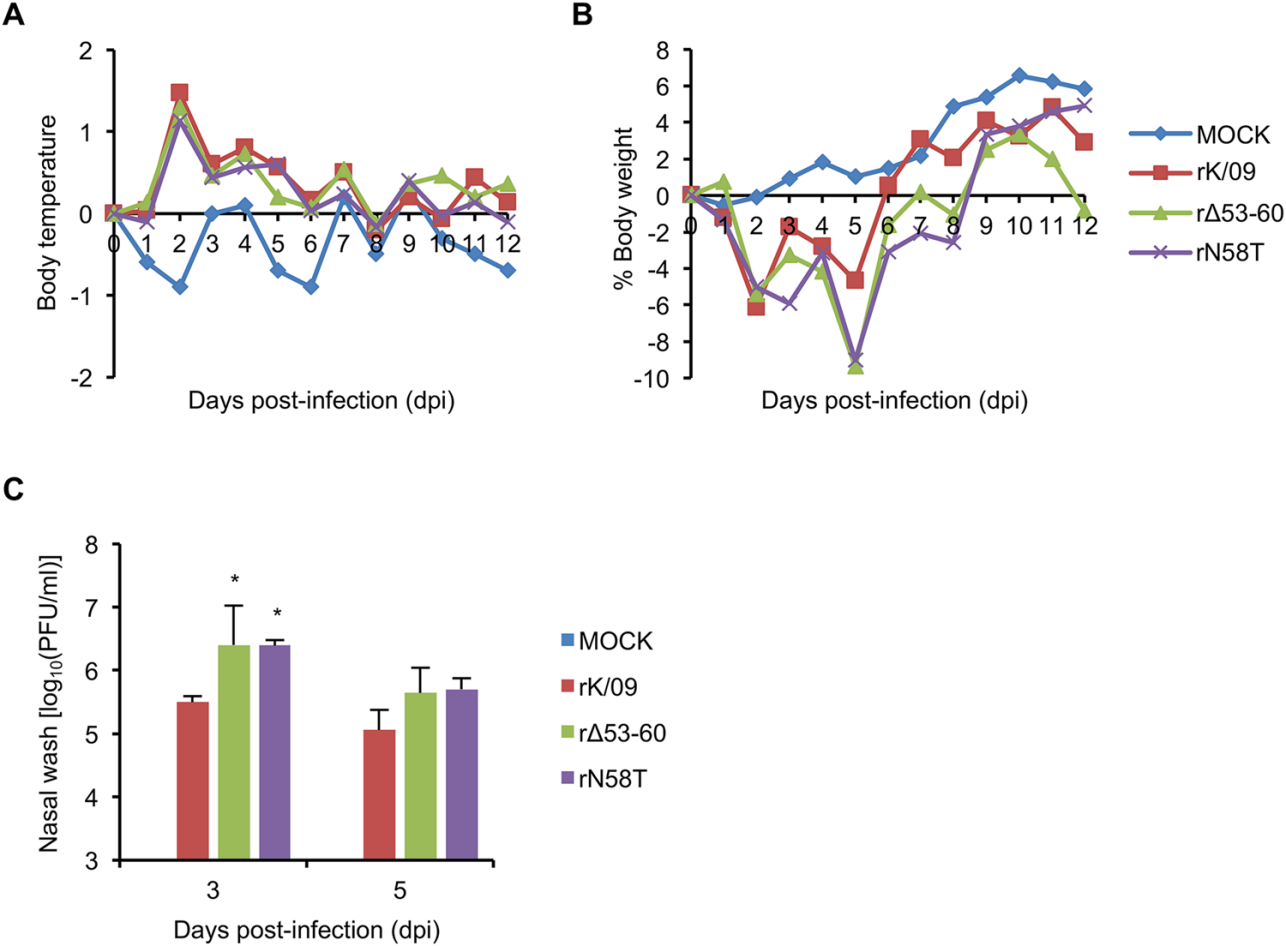
Pathogenicity of the rK/09 and its NA stalk mutant viruses in ferrets. Four groups of ferrets (n = 3 per group) were intranasally infected with 10^6^ PFU of each virus, and their rectal body temperatures (**A**) and body weight changes (**B**) were represented in average. (**C**) Viral titers in nasal wash samples collected at 3 and 5 dpi were determined by the plaque assay in MDCK cells. The results were represented in average, and error bars denote SD (^*^ *P < *0.05 compared with the rK/09 results).

To further investigate the effects of NA stalk truncation or deglycosylation on viral pathogenicity, we infected ferrets with the rK/09, rΔ53–60, and rN58T, respectively, and determined rectal body temperatures, body weight changes, and viral replication in the upper respiratory tracts of the infected ferrets. During the 12 days of observation, body temperatures of the infected ferrets all increased, compared with those of the ferrets in the mock group (Fig. 7A). The ferrets infected with rK/09 showed approximately 6% weight loss in maximum at 2 dpi and regained their weights afterwards (Fig. 7B). However, the ferrets infected with rΔ53–60 or rN58T lost approximately 9% of their body weights at 5 dpi and needed 4 more days to recover to their original body weights (Fig. 7B). Consistently, rΔ53–60 and rN58T resulted in higher nasal wash titers than rK/09 (Fig. 7C). These results indicate that NA stalk truncation or deglycosylation may be one of the mechanistic alterations to increase the pathogenicity of pH1N1 virus.

### NA stalk truncation or deglycosylation of H7N9 vaccine virus

**Figure 8:**
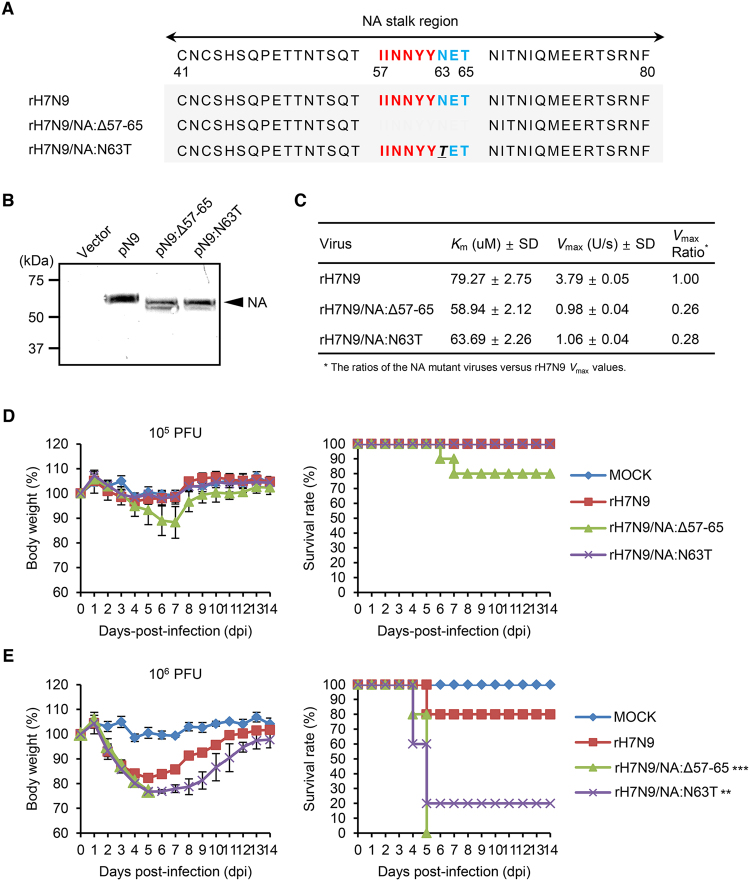
*In vitro* and *in vivo* evaluation of the H7N9 vaccine and its NA stalk mutant viruses. (**A**) The H7N9 vaccine (rH7N9) and its NA stalk mutant viruses (rH7N9/NA:Δ57–65 and rH7N9/NA:N63T) were generated by reverse genetics based on the NA stalk truncation or deglycosylation strategy. (**B**) In the western blotting assay, the NA stalk mutations (truncation and deglycosylation) were confirmed based on the mobility shift of NA proteins. (**C**) The enzyme kinetics data were fit to the Michaelis-Menten equation by nonlinear regression to determine the Michaelis constant (*Km*) and maximum velocity (*Vmax*) of substrate conversion. Results are given as the means ± SD from three independent determinations on triplicate samples where the *R* ^2^ was > 0.99. After 10^5^ (**D**) and 10^6^ (**E**) PFU intranasal infection of each virus, body weight changes and survival rates of the infected mice were represented in average (^**^ *P* < 0.01 and ^***^ *P* < 0.001 compared with the rH7N9 results).

**Figure 9:**
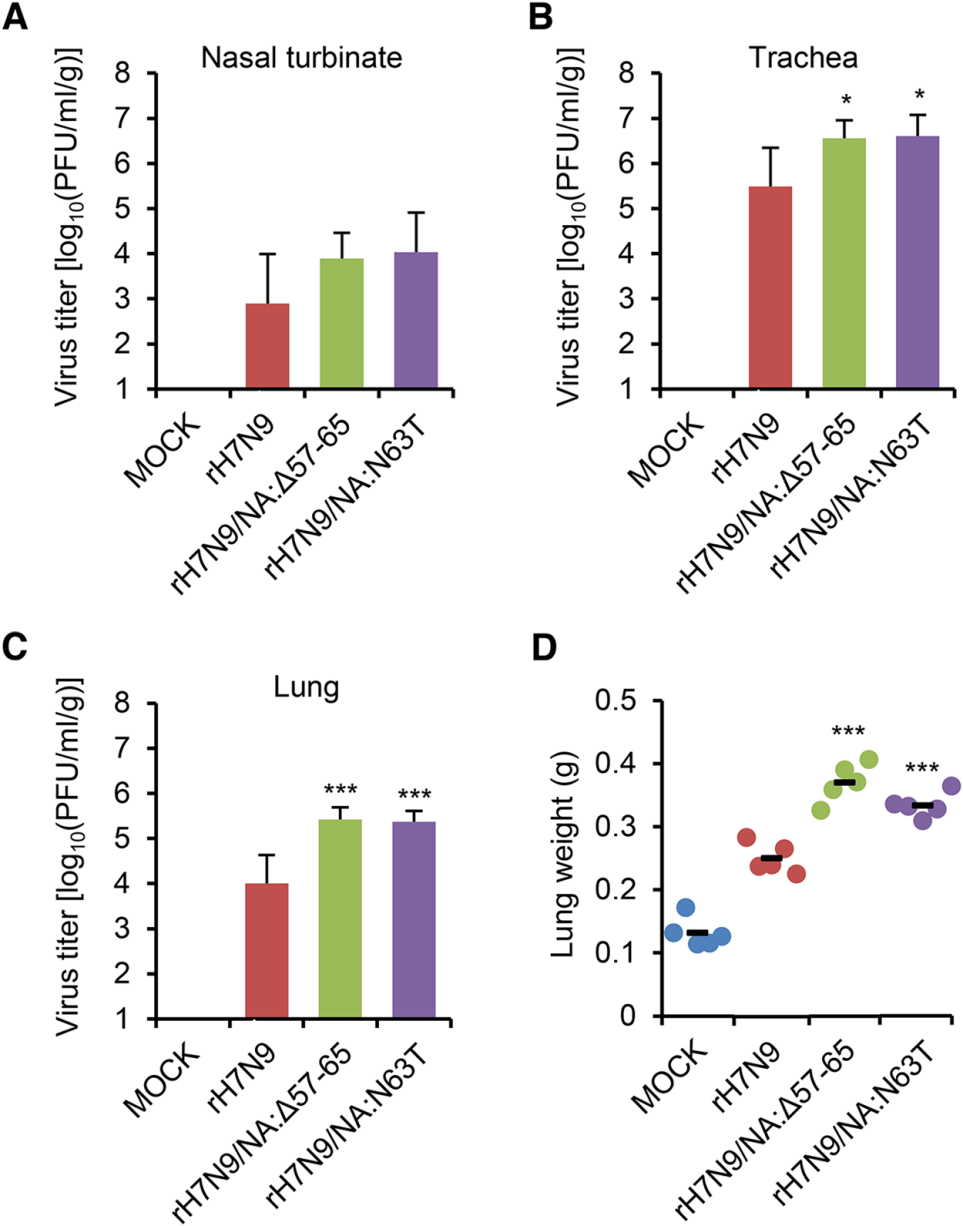
Pathogenicity of the H7N9 NA stalk mutant viruses in mice. Viral replication in the nasal turbinate (**A**), trachea (**B**), and lung (**C**) and lung weights (**D**) of the infected mice (n = 5) were investigated with 10^6^ PFU intranasal infection. The results were represented in average, and error bars denote SD (^*^ *P* < 0.05 and ^***^ *P* < 0.001 compared with the rH7N9 results).

We extended our findings regarding the NA stalk truncation or deglycosylation to the NA of a human-infecting avian influenza H7N9 strain. Using the H7N9 vaccine virus (rH7N9)^36^, we generated NA stalk-truncated (rH7N9/NA:Δ57–65) or -deglycosylated (rH7N9/NA:N63T) viruses (Fig. 8A). After assessing the mobility shift of the NA proteins of the H7N9 NA mutant viruses in the western blotting assay (Fig. 8B), we examined their NA enzymatic activity. In the same NA enzymatic activity assay, as we did for the K/09 NA stalk mutant viruses (Fig. 2C), the NA stalk mutations decreased the NA enzymatic activity of rH7N9/NA:Δ57–65 and rH7N9/NA:N63T (Fig. 8C). However, both rH7N9/NA:Δ57–65 and rH7N9/NA:N63T exhibited increased viral pathogenicity in mice. Even though only two mice infected with rH7N9/NA:Δ57–65 were dead at a 10^5^ PFU infection titer (Fig 8D), 10^6^ PFU of rH7N9/NA:Δ57–65 and rH7N9/NA:N63T killed ten and eight mice out of the ten infected mice, respectively (Fig. 8E). Based on these, the MLD_50_ values of rH7N9/NA:Δ57–65 and rH7N9/NA:N63T were determined as 10^5.3^ and 10^5.7^ PFU, respectively. The MDD values of rH7N9/NA:Δ57–65 and rH7N9/NA:N63T were determined as 5.8 ± 0.42 and 7.2 ± 3.61, respectively (Table 4). The H7N9 NA stalk mutant viruses also exhibited higher replication properties in the respiratory tracts of mice. Compared with the limited replication of rH7N9, both rH7N9/NA:Δ57–65 and rH7N9/NA:N63T viruses efficiently replicated in the nasal turbinate, trachea, and lung of the infected mice (Fig. 9A to C). Consistently, rH7N9/NA:Δ57–65 and rH7N9/NA:N63T resulted in the increases of lung weights of the infected mice (Fig. 7F). Taken all together, our results suggest that NA stalk truncation or deglycosylation may be one of the adaptive determinants that increase the pathogenicity of IAVs.

**Table 4:** Pathological findings from the mice infected with the rH7N9 and its NA stalk mutant viruses.

Virus	MLD_50_	% of body weight loss^a^ (dpi)	MDD^b^
MOCK	—	—	—
rH7N9	>10^6.5^	17.72 ± 4.68 (5)	12.4 ± 3.37
rH7N9/NA:Δ57–65	10^5.3^	23.08 ± 1.18 (5)	5.8 ± 0.42
rH7N9/NA:N63T	10^5.7^	23.23 ± 0.65 (5)	7.2 ± 3.61

^a^The mean maximum body weight losses and ^b^MDD were obtained from the mice infected with 10^6^ PFU. Each result was expressed in average with ± SD.

## Discussion

Through the events of host crossover or adaptation of avian IAVs from wild aquatic birds to poultry, swine, and mammalian hosts, zoonotic cases of human infection with avian IAVs can happen at any time^37, 38^. Regardless of explanation, it can instigate a public health threat of pandemic influenza. As well as the molecular adjustment of a shift of HA receptor binding specificity^39^, NA stalk truncation is considered as one way of host adaptation of avian IAVs in new hosts^21, 28^.

Previously, NA stalk truncation was reported only in the NAs of avian-origin IAVs^20–26, 28^. For the human H1N1 and H3N2 viruses, only a few strains appeared to retain stalk-truncated NAs (Table S2), and these stalk truncation regions did not include potential NLG sites (Data S1). For the first time, we here report the removal of the eight consecutive amino acids including one potential NLG site from the NA stalk of a human H1N1 virus strain (Fig. 2A and Table 2) and provide the experimental evidences of their pathogenic contribution to influenza animal models (Figs 3, 4, 5 and 7). Even though this evolutionary, adaptive mutation resulted from viral adaption in mice, it gives us useful information regarding the host adaptation of IAVs. At first, NA stalk truncation may alter NA enzymatic function^19, 30^. It can be appreciated that the short stalk NA may not properly promote the release of progeny virions due to a limited accessibility to its substrate, compared with the full-length stalk NA^19^, and it might be reflected in the reduced transmissibility of rΔ53–60 whereas the two NA stalk deglycosylation mutants rNN58SS and rN58T exhibited different transmission efficiency in guinea pigs (Fig. 6). As Blumenkrantz *et al*. reported the reduced transmissibility of a short-stalk NA mutant virus in a direct contact transmission experiment setting^19^, the NA stalk truncation in our study negatively affected viral transmissibility (Fig. 6).

Given that K/09 might be already equipped with a functionally-balanced HA and NA set^35^, the change of viral fitness caused by the NA stalk truncation or deglycosylation need to be compensated for with other molecular alterations in the NA protein *per se* or in any other viral proteins. Some or all the paralleled mutations identified in the other viral proteins might be first considered for their repayment role of fitness costs^40^. Even so, the NA stalk mutant viruses harboring the stalk-truncated or -deglycosylated mutation, exhibited increased viral pathogenicity in mice and ferrets, compared with their patental rK/09 virus (Figs 3 to 7 and Table 3). Just focusing on the changes of NA enzymatic activity due to the NA stalk mutations (Figs 2C and 8C), the pathogenicity increases of the NA stalk mutant viruses might be fortuitous indicators or aftermaths limited in our study. However, several research papers provided similar observations^19, 20, 24, 28–30^. Of these studies, some presented the increases of NA enzymatic activity by NA stalk truncation^20, 24, 29^ whereas the others reported its decreases^19, 28, 30^. Hence, the functional balance between HA and NA proteins should be considered^41, 42^. Even though NA stalk truncation or deglycosylation reduced NA enzymatic activity, their impacts might be neutral or even beneficial for HA receptor binding activity. Then, NA stalk truncation or deglycosylation may be a way of fine-tuning for a virus to secure the HA-NA functional balance, as demonstrated for the swine-origin 2009 pandemic H1N1 and avian-origin H7N9 viruses in our study and in the natural adaptation of avian IAVs from migratory waterfowls to terrestrial birds^19, 20^ and then to swine and humans.

Recently, the effects of NA stalk truncation were explained based on the functional flexibility changes of NA enzymatic pocket. Based on Durrant *et al*.^43^, NA stalk truncation might reduce the structural flexibility of NA enzymatic pocket, and, as a result, it would restrain NA affinity for its substrate. Similar to a limited access theory of short stalk NAs^34, 44, 45^, the flexibility constraint of NA enzymatic pocket caused by NA stalk truncation^43^ and the reduction of NA enzymatic activity due to antiviral treatment^46^ also suggested the increase of viral pathogenicity. When the NA stalk mutations applied to the NA of H7N9 vaccine virus, based on the sequence alignment with the N1 subtype NA protein, the rH7N9/NA:Δ57–65 and rH7N9/NA:N63T viruses also exhibited enhanced viral pathogenicity in mice (Figs 8 and 9). Given that N1 and N9 subtype NA proteins are classified into the two different NA groups (group I NA protein: N1, N4, N5, and N8 subtypes and group II NA protein: N2, N3, N6, N7, and N9 subtypes)^47^, the pathogenic contribution of NA stalk truncation or deglycosylation can be remarked as the molecular adaptive alterations of IAVs. However, considering the viral penetration of mucous layers in the respiratory tracts of animal models or the terminal phase (budding-out) of viral replication in host cells, the exact mechanisms of enhanced pathogenicity and systemic viral infection in mice, which resulted from the NA stalk truncation or deglycosylation (Fig. 5), needs further elucidation.

In this study, we explored the relationship of NA stalk truncation or deglycosylation with the subsequent changes of NA enzymatic activity, viral replication property in cells, and viral pathogenicity in animal models. Based on our results, we suggest that NA stalk truncation or deglycosylation may be an evolutionary, adaptive molecular alteration for adjusting NA enzymatic activity not only of avian IAVs but also of human seasonal viruses. Given the recent seasonal virus-like epidemic of the H7N9 virus in China, sustained surveillance should be wide open on further molecular changes of the NA stalk region of IAVs.

## Methods

### Ethics statement

This study was conducted in strict accordance with the recommendations in the Guide for the Care and Use of Laboratory Animals of the Animal, Plant, and Fisheries Quarantine and Inspection Agency of Korea. The experimental protocols were approved by the Institutional Animal Care and Use Committee (IACUC) of Korea University College of Medicine (permit number: KUIACUC-2014-225, 249). Given the biosafety issues, all the procedures regarding the generation and experiments of the NA stalk mutants of 2009 pandemic H1N1 and avian-origin H7N9 viruses were reviewed by the IACUC of Korea University College of Medicine (permit number: KUIBC-15–7-A-1) and conducted inside the BL3 facility of Korea University College of Medicine (national permit number of BL3 facility: KCDC-14–3–05, Korea Centers for Disease Control and Prevention, Osong, Republic of Korea).

### Cells and viruses

Madin-Darby canine kidney (MDCK), human embryonic kidney (293 T), and human lung epithelial (A549) cells were obtained from the American Type Culture Collection (ATCC, Manassas, VA, USA) and maintained with EMEM (for MDCK cells; Lonza, Basel, Switzerland) and DMEM (for 293 T and A549 cells; Thermo Fisher Scientific, Waltham, MA, USA) at 37 °C, 5% CO_2_. The p H1N1 A/Korea/01/2009 (K/09; National Center for Biotechnology Information (NCBI) taxonomy ID: 644289) virus was obtained from the Korea Centers for Disease Control and Prevention (KCDC; Osong, Republic of Korea). An avian influenza A(H7N9) vaccine virus (rH7N9) harboring the HA and NA genes of A/Anhui/01/2013 on the backbone of a vaccine donor, A/Puerto Rico/8/1934, was the virus used in the study of Lee *et al*.^36^.

### Reverse genetics

The NA stalk mutant viruses were generated by plasmid-based reverse genetics. Briefly, the eight gene segments (PB2, PB1, PA, HA, NP, NA, M, and NS) of the K/09 and rH7N9 viruses were first cloned into an ambisense pDZ plasmid. After modifying the NA stalk region (truncation: K/09 and rH7N9 NAs, removal of the amino acid residues of 53–60 and 57–65, respectively; deglycosylation: amino acid substitution of NN58–59SS and N58T for K/09 NA and N63T for rH7N9 NA), the eight plasmids of PB2, PB1, PA, HA, NP, NA, M, and NS genes were transfected into co-cultured 293 T and MDCK cells. After 48 hours, the cell supernatants were inoculated into the allantoic cavity of 10 days old embryonated chicken eggs and propagated for 48–72 h at 37 °C, 5% CO_2_ incubator. The allantoic fluids were tested by a hemagglutination (HA) assay using 0.5% (v/v) turkey RBC. Each virus (rK/09, rΔ53–60, rNN58SS, rN58T, rH7N9, rH7N9/NA:Δ57–65, and rH7N9/NA:N63T) (Table S3) was then purified by a plaque assay in MDCK cells and confirmed by sequence analysis after reverse transcription PCR before use.

### Western blot analysis

The NA gene of rK/09, rΔ53–60, rNN58SS, rN58T, rH7N9, rH7N9/NA:Δ57–65, and rH7N9/NA:N63T was first cloned into the HA-tagged pCAGGSII expression plasmid. Each pCAGGSII-NA plasmid (1 μg) was transfected into 293 T cells. Approximately 20 h later, the 293 T cells were harvested and treated with IP lysis buffer (Thermo Fisher Scientific) for SDS-PAGE. Each NA expression was then primarily detected with mouse monoclonal anti-HA antibody (Sigma-Aldrich, St. Louis, MO, USA) and secondarily with anti-mouse IgG-HRP antibody (KPL, Milford, MA, USA).

### Growth kinetics in cells

Confluent MDCK or A549 cells were inoculated with rK/09, *ma*-P5, rΔ53–60, rNN58SS, and rN58T at a multiplicity of infection (MOI) of 0.001 for 1 h. The cells were then washed five times and maintained with media containing 0.3% bovine serum albumin (BSA) in the presence of tosyl phenylalanyl chloromethyl ketone (TPCK) treated trypsin (1 µg/ml; Sigma-Aldrich). At each time point after virus inoculation (8, 16, 24, 48, and 72 hpi), the cell supernatants were collected and used for the viral titration by the plaque assay in MDCK cells. Limit of detection was 10^1^ PFU in the plaque assay.

### NA enzymatic assay

For a fluorometric-based assay, 2′-(4-Methylumbelliferyl)-α-D-*N*-acetylneuraminic acid (MU-NANA) (Sigma-Aldrich) was serially two-fold diluted in MES buffer (Sigma-Aldrich) with a final substrate concentration of 0 to 1,000 µM. Each virus was standardized to an equivalent dose of 10^6^ PFU and incubated at 37 °C in 50 µl reaction mixtures with the diluted concentrations of MU-NANA. Fluorescence expression was measured every 90 s for 45 m (30 measurements) at an excitation wavelength of 360 nm and emission of 448 nm. The reaction rate was then plotted against the MU-NANA concentration, and the *K*_m_ and *V*_max_ values were determined by fitting Michaelis-Menten kinetics curves in GraphPad Prism 5 (La Jolla, CA, USA).

### K/09 adaptation in mice

For the adaptation of K/09 in mice, a serial lung-to-lung passage was performed five times. Briefly, BALB/c mice (female, 5 weeks old; NaraBiotech, Seoul, Republic of Korea) were anesthetized and intranasally infected with 10^5^ PFU of K/09 (10^5^ PFU in 30 μl inoculum per mouse). After 48 h, the mouse lungs were harvested and homogenized in media with TissueLyzer II (Qiagen, Germany). 30 µl of the centrifuged lung homogenate was used as the inoculum for the next passage. After the first, third, and fifth rounds of lung-to-lung passage, mouse-adapted (*ma*) viruses (*ma*-P1, *ma*-P3, and *ma*-P5, respectively) were purified by the plaque assay in MDCK cells and propagated in embryonated chicken eggs.

### Determination of MLD50 titers of the viruses

The MLD_50_ titers of *ma*-P1, *ma*-P3, *ma*-P5, rK/09, rΔ53–60, rNN58SS, and rN58T were determined based on the body weight changes and survival rates of the infected mice. Briefly, 10 BALB/c mice per group were intranasally infected with 10^4^, 10^5^, and 10^6^ PFU of each virus. Body weight changes and survival rates of the infected mice were monitored daily for 14 dpi. The infected mice showing severe clinical symptoms (such as extreme shivering and ruffled feather) and lost more than 25% of initial body weight were considered experimental death and humanely euthanized. The MLD_50_ titers were then determined using the Reed and Muench method^48^. For the determination of MLD_50_ titers of rH7N9, rH7N9/NA:Δ57–65, rH7N9/NA:N63T, 5 BALB/c mice per group were intranasally infected with 10^5^ and 10^6^ PFU of each virus. Mean day of death (MDD) of the infected mice were determined based on the humane endpoint of body weight loss defined above.

### Replication property of the viruses in the respiratory and other internal body organs of the infected mice

To assess the replication property of rK/09, *ma*-P5, rΔ53–60, rNN58SS, and rN58T in the mouse lungs, six BALB/c mice per group were anesthetized and infected intranasally with 10^5^ PFU of each virus. At 3 and 6 dpi, three mice per group were sacrificed for lung collection. The collected lungs were then homogenized in media. Viral titers were determined by the plaque assay in MDCK cells. To assess the systemic infection property of rK/09, *ma*-P5, rΔ53–60, rNN58SS, and rN58T, three BALB/c mice per group were anesthetized and intranasally infected with 10^6^ PFU of each virus. At 3 dpi, the infected mice were sacrificed for organ collection (brain, heart, nasal turbinate, trachea, lung, and spleen), and each mouse organ was homogenized with media in separate tubes. Viral titers of these organs were determined by the plaque assay in MDCK cells. For the replication property analysis of rH7N9, rH7N9/NA:Δ57–65, rH7N9/NA:N63T, five BALB/c mice per group were infected intranasally with 10^6^ PFU of each virus, and their nasal turbinate, trachea, and lungs were collected at 3 dpi.

### Ferret experiment

Viral pathogenicity was also assessed in ferrets (male, 13 weeks old; Marshall BioResources, North Rose, NY, USA). Briefly, ferrets were intranasally infected with 10^6^ PFU of rK/09, rΔ53–60, and rN58T (10^6^ PFU in 100 μl inoculum per ferret). Body weight changes and rectal body temperature were monitored daily for 12 dpi. Nasal wash samples were collected at 3 and 5 dpi for the titration of viral replication in the upper respiratory tract of ferrets by the plaque assay in MDCK cells.

### Transmission study

For a direct contact transmission study, six guinea pigs (Hartley strain, female, 5–6 weeks old; Charles River Laboratories, Wilmington, MA, USA) were anesthetized and intranasally infected with 10^5^ PFU of rK/09, ma-P5, rΔ53–60, rNN58SS, and rN58T. Next day, six naïve guinea pigs were co-caged with each infected guinea pig. Nasal wash samples were collected using 1 ml of PBS (supplemented with 0.3% BSA and penicillin-streptomycin) at 1, 3, 5 and 7 dpi from the inoculated guinea pigs and at 2, 4, 6 and 8 days post-contact (dpc) from the contact group. Viral titers in the nasal wash samples were determined by the plaque assay in MDCK cells.

### Histophathology

For the lung histopathology, three mice per group were anesthetized and intranasally infected with 10^5^ PFU of each virus. At 6 dpi, the infected mice were sacrificed, and their lungs were preserved in 10% formalin solution (Sigma-Aldrich). The formalin-fixed lungs were embedded in paraffin and cut into 5 µm thick sections. The lung specimen was then stained with hematoxylin-and-eosin (H&E).

### Sequence analysis

To investigate polymorphic variations of the amino acid substitutions of *ma*-P5, PB1 (n = 6,158), PA (n = 6,397), HA (n = 10,169), and M (4,569) gene sequences of the human H1N1 strains were downloaded from the NCBI database of Influenza Virus Resource (https://www.ncbi.nlm.nih.gov/genomes/FLU/Database/nph-select.cgi?go=database) (Table S1). Each sequence set was aligned using MAFFT (v7.310)^49^, and polymorphic signatures were analyzed using AliView (v1.19). For the investigation of the natural prevalence of NA stalk-truncated strains, NA sequences (N1, n = 8,586; N2, 8,306; N3, n = 2; N6, n = 3; N7, n = 4; N8, n = 2; and N9, n = 79) were also downloaded from the NCBI database (Table S2).

### Statistical analysis

Differences of viral replication kinetics in cells, the mean days of death, viral titers in the lungs and other internal organs of the infected mice, and nasal wash titers in ferrets were evaluated using one-way ANOVA test and confirmed by Tukey’s multiple comparison test. Survival graphs were prepared by Kaplan-Meier method and statistically analyzed with the Mantel-Cox log-rank test followed by the Gehan-Breslow-Wilcoxon test. Differences of viral transmission efficiency in guinea pigs were evaluated using Fisher’s exact test.

## Electronic supplementary material

Supplementary Information

Dataset 1
