# Supplementary material for: Adaptive mutations of neuraminidase stalk truncation and deglycosylation confer enhanced pathogenicity of influenza A viruses
Source: Sci Rep. 2017 Sep 7;7:10928. doi: 10.1038/s41598-017-11348-0 (PMC5589767; doi:10.1038/s41598-017-11348-0)
Supplement: Supplementary file 1 — Supplementary Information [file 41598_2017_11348_MOESM1_ESM.pdf]

Supplementary information

Adaptive mutations of neuraminidase stalk truncation and deglycosylation confer enhanced pathogenicity of influenza A viruses

Authors: Sehee Park, Jin Il Kim, Ilseob Lee, Joon-Yong Bae, Kirim Yoo, Misun Nam, Juwon Kim, Mee Sook Park, Ki-Joon Song, Jin-Won Song, Sun-Ho Kee, Man-Seong Park

Table S1 Page 2

Table S2 Page 3

Table S3 Page 4

Table S1. Polymorphic amino acid variations of the substituted residues in *ma*-P5 virus.

Protein coding region	n of sequence	Amino acid residue	Polymorphism at a given residue (%)[§]
PB1	6,158	193	D193 = 6,158 (100)
		710	G710 = 6,158 (100)
PA	6,397	21	M21 = 6,397 (100)
		511	L511 = 6,397 (100)
HA	10,169	144	D144 = 10,166 (99.48); E144 = 5 (0.49); N144 = 2 (0.02); G144 = 1 (0.01)
		283	I283 = 10,127 (99.58); V283 = 26 (0.26); F283 = 15 (0.15); L283 = 1 (0.01)
M1	4,569	206	A206 = 4,567 (99.96); V206 = 2 (0.04)

[§], Constitution rate (%) of each polymorphic amino acid was indicated in parenthesis.

Table S2. Prevalence of stalk truncation in the NAs of human influenza A isolates.

NA subtype	Virus subtype	n of NA sequence[§]	n of stalk-truncated NA (%)[¶]
N1	H1N1	8,343	26 (0.31)
	H3N1	1	0
	H5N1	242	241 (99.59)
N2	H1N2	54	0
	H2N2	127	1 (0.79)
	H3N2	8,113	5 (0.06)
	H7N2	1	1 (100)
	H9N2	11	4 (36.36)
N3	H7N3	2	0
N6	H5N6	3	3 (100)
N7	H7N7	4	0
N8	H10N8	2	0
N9	H7N9	79	79 (100)

[§], Unique NA sequences were downloaded from the NCBI database; [¶], NA stalk truncation was determined based on the sequence removal between the amino acid residues 31 and 100 of each NA subtypes.

Table S3. HA and PFU titers of the viruses.

Virus	HA titer	PFU/ml
rK/09	2^6	4.26×10^7
ma-P5	2^7	4.85×10^8
rΔ53-60	2^7	2.70×10^8
rNN58SS	2^7	1.80×10^8
rN58T	2^6	4.25×10^8
rH7N9	2^7	4.30×10^7
rH7N9/NA:rΔ57-65	2^7	1.80×10^7
rH7N9/NA:N63T	2^7	9.40×10^7
