# Supplementary material for: Adaptive mutations of neuraminidase stalk truncation and deglycosylation confer enhanced pathogenicity of influenza A viruses
Source: Sci Rep. 2017 Sep 7;7:10928. doi: 10.1038/s41598-017-11348-0 (PMC5589767; doi:10.1038/s41598-017-11348-0)
Supplement: Supplementary file 2 — Dataset 1 [file 41598_2017_11348_MOESM2_ESM.doc]

>H1N1_PB1_CY049322_A/Singapore/ON478/2009

atggatgtcaatccgactctacttttcctaaaaattccagcgcaaaatgccataagcaccacattcccttatactggagatcctccatacagccatggaacaggaacaggatacaccatggacacagtaaacagaacacaccaatactcagaaaagggaaagtggacgacaaacacagagactggtgcaccccagctcaacccgattgatggaccactacctgaggataatgaaccaagtgggtatgcacaaacagactgtgttctagaggctatggctttccttgaagaatcccacccaggaatatttgagaattcatgccttgaaacaatggaagttgttcaacaaacaagggtagataaactaactcaaggtcgccagacttatgattggacattaaacagaaatcaaccggcagcaactgcattggccaacaccatagaagtctttagatcgaatggcctaacagctaatgagtcaggaaggctaatagatttcttaaaggatgtaatggaatcaatgaacaaagaggaaatagagataacaacccactttcaaagaaaaaggagagtaagagacaacatgaccaagaagatggtcacgcaaagaacaatagggaagaaaaaacaaagactgaataagagaggctatctaataagagcactgacattaaatacgatgaccaaagatgcagagagaggcaagttaaaaagaagggctatcgcaacacctgggatgcagattagaggtttcgtatactttgttgaaactttagctaggagcatttgcgaaaagcttgaacagtctgggctcccagtagggggcaatgaaaagaaggccaaactggcaaatgttgtgagaaagatgatgactaattcacaagacacagagatttctttcacaatcactggggacaacactaagtggaatgaaaatcaaaatcctcgaatgttcctggcgatgattacatatatcaccagaaatcaacccgagtggttcagaaacatcctgagcatggcacccataatgttctcaaacaaaatggcaagactagggaaagggtacatgttcgagagtaaaagaatgaagattcgaacacaaataccagcagaaatgctagcaagcattgacctgaagtacttcaatgaatcaacaaagaagaaaattgagaaaataaggcctcttctaatagatggcacagcatcactgagtcctgggatgatgatgggcatgttcaacatgctaagtacggtcttgggagtctcgatactgaatcttggacaaaagaaatacaccaagacaatatactggtgggatgggctccaatcatccgacgattttgctctcatagtgaatgcaccaaaccatgagggaatacaagcaggagtggacagattctacaggacctgcaagttagtgggaatcaacatgagcaaaaagaagtcctatataaataagacagggacatttgaattcacaagctttttttatcgctatggatttgtggctaattttagcatggagctacccagctttggagtgtctggagtaaatgaatcagctgacatgagtattggagtaacagtgataaagaacaacatgataaacaatgaccttggacctgcaacggcccagatggctcttcaattgttcatcaaagactacagatacacatataggtgccataggggagacacacaaattcagacgagaagatcatttgagttaaagaagctgtgggatcaaacccaatcaaaggtagggctattagtatcagatggaggaccaaacttatacaatatacggaatcttcacattcctgaagtctgcttaaaatgggagctaatggatgatgattatcggggaagactttgtaatcccctgaatccctttgtcagtcataaagagattgattctgtaaacaatgctgtggtaatgccagcccatggtccagccaaaagcatggaatatgatgccgttgcaactacacattcctggattcccaagaggaatcgttctattctcaacacaagccaaaggggaattcttgaggatgaacagatgtaccagaagtgctgcaatctattcgagaaatttttccctagcagttcatataggagaccggttggaatttctagcatggtggaggccatggtgtctagggcccggattgatgccagggtcgacttcgagtctggacggatcaagaaagaagagttctctgagatcatgaagatctgttccaccattgaagaactcagacggcaaaaataa

>H1N1_PB1_KY925004_A/Viamao/LACENRS-877/2012

atggatgtcaatccgactctacttttcctaaaaattccagcgcaaaatgccataagcaccacattcccttatactggagatcctccatacagccatggaacaggaacaggatacaccatggacacagtaaacagaacacaccaatactcagaaaagggaaagtggacgacaaacacagagactggtgcaccccagctcaacccgattgatggaccactacctgaggataatgaaccaagtgggtatgcacaaacagactgtgttctagaggctatggctttccttgaagaatcccacccaggaatatttgagaattcatgccttgaaacaatggaagttgttcaacaaacaagggtagataaactaactcaaggtcgccagacttatgattggacactaaacagaaatcaaccggcagcaactgcattggccaacaccatagaagtctttagatcgaatggcctaacagctaatgagtcaggaaggttaatagatttcttaaaggatgtaatggaatcaatgaacaaagaggaaatagagataacaacccactttcaaagaaaaaggagagtgagagacaacatgaccaagaaaatggtcacgcaaagaacaatagggaagaaaaaacaaagactgaataagagaggctatctaataagagcactgacattaaatacgatgaccaaagatgcagagagaggcaagttaaaaagaagggctatcgcaacacctgggatgcagattagaggtttcgtatattttgttgaaactttagctaggagcatttgcgaaaagcttgaacagtctgggctcccagtagggggcaatgaaaagaaggccaaactggcaaatgttgtgaggaagatgatgactaattcacaagacacagagatgtctttcacaatcactggggacaacactaagtggaatgaaaaccaaaatcctcgaatgttcctggcgatgattacatatatcaccagaaatcaacccgagtggttcagaaacatcctgagcatggcacccataatgttctcaaacaaaatggcaagactagggaaagggtacatgttcgagagtaaaagaatgaagattcgaacgcaaataccagcagaaatgctagcaagcattgacctgaagtacttcaatgaatcaacaaagaagaaaattgagaaaataagacctcttttaatagatggcacagcatcactgagtcctgggatgatgatgggcatgttcaacatgctaagtacggtcttgggagtctcgatactgaatcttgggcaaaagaaatacaccaagacaatatactggtgggatgggctccaatcatccgacgattttgctctcatagtgaatgcaccaaaccatgagggaatacaagcaggagtggacagattttacaggacctgcaagttagtgggaatcaacatgagcaaaaagaagtcctatataaataagacagggacatttgaattcacaagctttttttatcgctatggatttgtggctaattttagcatggagctacccagctttggagtgtctggagtaaatgaatcagctgacatgagtattggagtaacagtgataaagaacaacatgataaacaatgaccttggacctgcaacggcccagatggctcttcaattgttcatcaaagactacagatacacatataggtgccataggggagacacacaaattcagacgagaagatcatttgagttaaagaagctgtgggatcaaacccaatcaaaggtagggctattagtatcagatggaggaccaaacttatacaatatacggaatcttcacattcctgaagtctgcttaaaatgggagctaatggatgatgattatcggggaagactttgtaatcccctgaatccctttgtaagtcataaagagattgattctgtaaacaatgctgtggtaatgccagctcatggtccagccaaaagcatggaatatgatgccgttgcaactacacattcctggattcccaagaggaatcgctctattctcaacacaagccaaaggggaattcttgaggatgaacagatgtaccagaagtgctgcaatctattcgagaaatttttccctagcagttcatataggagaccggttggaatttctagcatggtggaggccatggtgtctagggcccggattgatgccagggtcgactttgagtctggacggatcaagaaagaagagttctctgagatcatgaagatctgttccaccattgaagaactcagacggcaaaaataa

>H1N1_PB1_CY027537_A/Memphis/44/1983

atggatgtcaatccgaccttacttttcttaaaagtgccagcacaaaatgctataagcacaactttcccttatactggagaccctccttacagccatgggacaggaacagggtacaccatggatacagtcaacaggacacatcagtactcagaaaggggaagatggacaaaaaacaccgaaactggagcaccgcaactcaacccgattgatgggccactaccaaaagacaatgaaccaagtggttatgcccaaacagattgtgtattagaagcaatggctttccttgaggaatcccatcccggtatttttgaaaactcgtgtattgaaacgatggaggttgttcagcaaacacgtgtagacaaactgacacaaggcagacagacctatgactggactctaaatagaaaccagcctgctgctacagcattggccaacaccatagaagtgttcagatcaaacggcctcatggcaaatgaatctggaaggctaatagacttccttaaagatgtaatggattcaatggatagagaagaaatggagatcacaactcattttcagagaaagagaagagtgagggacaatgtaactaagaaaatggtgacacaaagaacaataggcaaaaagaaacatagattaaacaaaaggagttatctaattagggcattaaccctgaacacaatgaccaaagatgctgagagggggaaactaaaacgtagagcaattgcaaccccagggatgcaaataagggggtttgtatactttgttgagacactggcaagaagtatatgtgaaaaacttgaacaatcagggttgccagttggaggaaatgaaaagaaagcaaagttagcaaatgttgtaaggaaaatgatgagcaattctcaggacactgaaatttctttcaccatcactggagataacacaaaatggaacgaaaatcagaaccctagaatgtttttggccatgatcacatatataaccagaaatcagcccgaatggttcagaaatattctaagtattgctccaataatgttctcaaacaaaatggcgagactaggtaaggggtacatgtttgaaagcaagagtatgaaacttagaactcaaatacctgcagaaatgctagcaaacatcgatttgaggtatttcaatgattcaacaagaaagaaaattgaaaaaatcaggccactcttaatagatggaactgcatcactgagccctggaatgatgatgggcatgttcaatatgttaagcactgtattgggcgtctccatcctgaatcttggacaaaagagatacaccaagactacttactggtgggatggtcttcaatcgtctgatgattttgctctgattgtgaatgcacccaactatgcaggaattcaagctggagttgacaggttttatcgaacctgtaagctgctcggaatcaacatgagcaagaaaaagtcctacataaacagaacaggtacttttgaattcacgagctttttttatcgttatgggtttgttgccaatttcagcatggagcttcccagttttggggtgtctgggatcaacgagtctgcagacatgagtattggagtcactgtcatcaaaaacaatatgataaacaatgatcttggtccagcaaccgctcaaatggcccttcagttattcatcaaagattacaggtacacatatcgatgccacagaggtgacacacaaatacaaacccgaagatcatttgaaataaagaaactgtgggatcaaacccgttccaaaactgggctgctggtctctgatggaggtcctaatttgtacaacattagaaatctccacattcctgaagtctgcttgaaatgggatttgatggatgaggattaccaggggcgtttatgcaacccattgaacccatttgtcagtcataaagagattgaatcagtgaacaatgcagtgatgatgccggcacatggtccagccaaaattatggagtatgatgctgttgcaacaacacactcctgggtccccaaaaggaatcgatccatcttgaatacgagccaaaggggaatacttgaggatgaacaaatgtatcagaggtgctgcaatttatttgaaaaattcttccccagcagttcatacagaagaccagtcggaatatccagtatggtagaggctatgatttccagagcccgaattgatgcacggattgatttcgaatctggaaggataaagaaagaggagttcactgagatcatgaagacctgttccaccattgaagaactcaaacggcaaaaatag

>H1N1_PB1_CY027769_A/Ohio/UR06-0112/2007

atggatgtcaatccgacattacttttcttaaaagtgccagcacaaaatgctataagcacaacttttccttatactggtgaccctccttacagccatgggacaggaacagggtacaccatggatacagtcaacaggacacaccagtactcagaaagaggaagatggacaaaaaataccgaaactggagcaccgcaactcaacccaattgatgggccactaccagaagacaatgaaccgagtggctatgcccaaacagattgtgtattagaagcaatggctttccttgaagaatcccatcctggtatctttgaaaactcttgtattgagacaatggaggttgttcagcaaacaagggtggacaaactgacacagggcagacagacctatgactggactctaaataggaaccagcctgctgccacagcattggcaaacactatagaagtattcagatcaaacggcctcatagcaaatgaatctgggaggctaatagacttccttaaagatgtaatggagtcgatggacatagacgaagtagaggtcacaactcattttcaaagaaagaggagagtgagagacaatgtaactaaaaaaatggtgacccaaagaacaataggcaaaaagaaacataaattagacaaaagaagttacctaattagggcattaaccctgaacacaatgaccaaagatgctgagagagggaaactaaaacgcagagcaattgcaaccccaggaatgcaaataagggggtttgtatactttgttgagacactggcaagaagcatatgtgaaaagcttgaacaatcaggattgccagttggaggaaatgagaagaaagcaaagttagcaaatgttgtaaggaagatgatgaccaactcccaggacactgaaatttctttcaccatcactggagataacacaaaatggaacgaaaatcaaaaccctagaatgttcttggccatgatcacatatataaccaaaaatcagcctgaatggttcagaaatattctaagtattgccccaataatgttttcaaacaagatggcgagactaggtaaggggtacatgtttgaaagcaagagtatgaaactgagaactcaaatacctgcagagatgctagccaacatagatttgaaatatttcaatgattcaactaaaaagaaaattgaaaaaatccgaccattattaatagatggaactgcatcattgagtcctggaatgatgatgggcatgttcaatatgttgagcaccgtcttgggcgtctccattctgaatcttgggcaaaagagatacaccaagactacttactggtgggatggtcttcaatcgtctgatgattttgctttgattgtgaatgcacccaactatgcaggaattcaagctggagttgacaggttttatcgaacctgtaagctgctcggaattaatatgagcaaaaagaagtcttacataaacagaacaggtacctttgaattcacgagctttttctatcgttatgggtttgttgccaatttcagcatggagcttcctagttttggggtgtctggggtcaatgaatctgcagacatgagtattggagtcactgtcatcaaaaacaatatgataaacaatgaccttggcccagcaactgctcaaatggcccttcagttatttataaaagattacaggtacacttatcgatgccacagaggtgacacacaaatacaaacccggagatcatttgagataaagaaactatgggaccaaacccgctctaaagctgggctgttggtctctgatggaggccccaatttatataacattagaaatctccatattcctgaagtttgcttgaaatgggagttgatggatgaggattatcaggggcgtttatgcaacccattaaacccgtttgtcagccataaagagattgaatcagtaaacaatgcagtgataatgccggcacatggtccagccaaaaatatggagtatgacgctgttgcaacaacacactcctgggtccccaaaagaaatcgatccattttgaacacgagccaacgggggatacttgaagatgagcaaatgtatcagaggtgctgcaatttatttgaaaaattcttcccaagtagctcatacagaagaccagttggaatatccagtatggtagaggctatggtttcaagagcccgaattgatgcacggattgatttcgaatctggaaggataaagagagaggaatttgctgagatcatgaagatctgttccaccattgaagacctcagacggcaaaaatag

>H1N1_PB1_CY083803_A/Berlin/INS362/2009

atggatgtcaatccgactctacttttcctaaaaattccagcgcaaaatgccataagcaccacattcccttatactggagatcctccatacagccatggaacaggaacaggatacaccatggacacagtaaacagaacacaccagtactcagaaaagggaaagtggacgacaaacacagagactggtgcaccccagctcaacccgattgatggaccactacctgaggataatgaaccaagtgggtatgcacaaacagactgtgttctagaggctatggctttccttgaagaatcccacccaggaatatttgagaattcatgccttgaaacaatggaagttgttcaacaaacaagggtagataaactaactcaaggtcgccagacttatgattggacattaaacagaaatcaaccggcagcaactgcattggccaacaccatagaagtctttagatcgaatggcctaacagctaatgagtcaggaaggctaatagatttcttaaaggatgtaatggaatcaatgaacagagaggaaatagagataacaacccactttcagagaaaaaggagagtaagagacaacatgaccaagaagatggtcacgcaaagaacaatagggaagaaaaaacaaagactgaataagagaggctatctaataagagcactgacattaaatacgatgaccaaagatgcagagagaggcaagttaaaaagaagggctatcgcaacacctgggatgcagattagaggtttcgtatactttgttgaaactttagctaggagcatttgcgaaaagcttgaacagtctgggctcccagtagggggcaatgaaaagaaggccaaactggcaaatgttgtgagaaagatgatgactaattcacaagacacagagatttctttcacaatcactggggacaacactaagtggaatgaaaatcaaaatcctcgaatgttcctggcgatgattacatatatcaccagaaatcaacccgagtggttcagaaacatcctgagcatggcacccataatgttctcaaacaaaatggcaagactagggaaagggtacatgttcgagagtaaaagaatgaagattcgaacacaaataccagcagaaatgctagcaagcattgacctgaagtacttcaatgaatcaacaaagaagaaaattgagaaaataaggcctcttctaatagatggcacagcatcactgagtcctgggatgatgatgggcatgttcaacatgctaagtacggtcttgggagtctcgatactgaatcttggacaaaagaaatacaccaagacaatatactggtgggatgggctccaatcatccgacgattttgctctcatagtgaatgcaccaaaccatgagggaatacaagcaggagtggacagattctacaggacctgcaagttagtgggaatcaacatgagcaaaaagaagtcctatataaataagacagggacatttgaattcacaagctttttttatcgctatggatttgtggctaattttagcatggagctacccagctttggagtgtctggagtaaatgaatcagctgacatgagtattggagtaacagtgataaagaacaacatgataaacaatgaccttggacctgcaacggcccagatggctcttcaattgttcatcaaagactacagatacacatataggtgccataggggagacacacaaattcagacgagaagatcatttgagttaaagaagctgtgggatcaaacccaatcaaaggtagggctattagtatcagatggaggaccaaacttatacaatatacggaatcttcacattcctgaagtctgcttaaaatgggagctaatggatgatgattatcggggaagactttgtaatcccctgaatccctttgtcagtcataaagagattgattctgtaaacaatgctgtggtaatgccagcccatggtccagtcaaaagcatggaatatgatgccgttgcaactacacattcctggattcccaagaggaatcgttctattctcaacacaagccaaaggggaattcttgaggatgaacagatgtaccagaagtgctgcaatctattcgagaaatttttccctagcagttcatataggagaccggttggaatttctagcatggtggaggccatggtgtctagggcccggattgatgccagggtcgacttcgagtctggacggatcaagaaagaagagttctctgagatcatgaagatctgttccaccattgaagaactcagacggcaaaaataa

>H1N1_PB1_KX005680_A/Florida/04/2016

atggatgtcaatccgactctacttttcctaaaaattccagcacaaaatgccataagcaccacattcccttatactggagatcctccatacagccatggaacaggaacaggatacaccatggacacagtaaacagaacacaccaatactcagaaaaggggaagtggacaacaaacacagagactggtgcaccccagctcaacccgattgacggaccactacctgaagataatgaaccaagtgggtatgcacaaacagactgtgttctagaggctatggcttttcttgaagaatcccatccaggaatatttgagaattcatgccttgaaacaatggaagttgttcaacaaacaagggtagataaactgactcaaggtcgccagacttatgattggacattaaacagaaatcaaccggcagcaactgcattggccaacaccatagaagtttttagatcgaatgacctaacagctaacgagtcaggaaggctaatagatttcttaaaggatgtgatggaatcaatgaacaaagaggaaatagagataacaacccactttcaaagaaagaggagagtaagagacaacatgaccaagaagatggtcacgcaaagaacaatagggaagaaaaaacagagactgaataagagaggctatctaataagagcactgacattaaatacgatgaccaaagatgcagagagaggcaagttaaaaagaagggctatcgcaacacctgggatgcagattagaggtttcgtatactttgttgaaactttagctaggagcatttgcgaaaagcttgaacagtctgggctcccagtagggggcaatgaaaagaaggccaaattggcaaatgttgtgagaaagatgatgactaattcacaagatacagagatttctttcacaatcactggggacaacactaagtggaatgaaaatcaaaatcctcgaatgttcctggcgatgattacatatatcaccagaaatcaacccgagtggtttagaaacatcctgagcatggcacccataatgttctcaaataaaatggcaagactaggaaaagggtacatgttcgagagtaaaagaatgaagattcgaacacaaataccagcagaaatgctaacaagcattgatctgaagtatttcaatgaatcaacaaagaagaaaattgagaaaataaggcctcttttaatggatggcacagcatcactgagccctgggatgatgatgggcatgttcaacatgctaagtacggtcttgggagtctcgatactgaatcttggacaaaagaaatacaccaagacaacatactggtgggatgggctccaatcatccgacgattttgctctcatagtgaatgcaccaaaccatgaaggaatacaagcaggagtggacagattctacaggacctgcaagttagtgggaatcaacatgagcaaaaagaagtcctatataaataagacagggacatttgaattcacaagctttttttatcgctatggatttgtggctaattttagcatggagctacccagctttggagtgtctggagtaaatgaatcagctgacatgagtattggagtaacagtgataaagaacaacatgataaacaatgaccttggacctgcaacggctcagatggctcttcaattgttcatcaaagactacagatacacatataggtgccataggggagacacacaaattcagacgagaagatcatttgagttgaagaagctatgggatcaaacccaatcaaaggtagggctattagtatcagatggaggaccaaacttatacaacatacggaatcttcacattcctgaagtctgcttaaaatgggagctaatggatgatgattatcggggaagactttgtaatcccctgaatccctttgtgagtcataaggagattgattctgtaaacaatgctgtggtaatgccagcccatggcccagccaaaagcatggaatatgatgccgttgctactacacattcctggattcccaagaggaatcgttctattctcaacacaagccaaaggggaattcttgaggatgaacagatgtaccagaagtgttgcaatttattcgagaaatttttccctagcagttcatataggagaccggttggaatttctagcatggtggaggccatggtgtctagggcccggattgatgccagagtcgactttgagtctggacggatcaagaaagaagagttctctgagatcatgaagatctgttccaccattgaagaactcagacggcaaaaataa

>H1N1_PB1_CY031146_A/Tennessee/UR06-0236/2007

atggatgtcaatccgacattacttttcttaaaagtgccagcacaaaatgctataagcacaacttttccttatactggtgaccctccttacagccatggaacaggaacagggtacaccatggatacagtcaacaggacacatcagtactcagaaagaggaagatggacaaaaaataccgaaactggagcaccgcaactcaacccgattgatgggccactaccagaagacaatgaaccaagtggctatgcccaaacagattgtgtattagaagcaatggctttccttgaagaatcccatcctggtatttttgaaaattcttgtattgaaacaatggaggttgttcagcaaacaagggtggacaaactgacacaaggcagacaaacctatgactggactctaaataggaaccagcctgctgccacagcattggcaaacactatagaagtattcagatcaaatggcctcatagcaaatgaatctgggaggctaatagacttccttaaagatgtaatggagtcgatggacagagacgaagtagaggtcacaactcattttcaaagaaagaggagagtgagagacaatgtaactaaaaaaatggtaacccaaagaacaataggaaaaaagaaacataaattagacaaaagaagttacctaattagggcattaaccctgaacacaatgaccaaagatgctgagagggggaaactaaaacgcagagcaattgcaaccccaggaatgcaaataagggggtttgtatactttgttgagacactggcaagaagcatatgtgaaaagcttgaacaatcagggttgccagttggaggaaatgagaagaaagcaaagttagcaaatgttgtaaggaagatgatgaccaactcccaggacactgaaatctctttcaccatcactggagataacacaaaatggaacgaaaatcaaaaccctagaatgttcttggccatgatcacatatataaccaaaaatcagcctgaatggttcagaaatattctaagtattgctccaataatgttttcaaacaaaatggcgagactaggtagggggtatatgtttgaaagcaagagtatgaaactgagaacccaaatacctgcagagatgctagccaacatagatttgaaatatttcaatgattcaactaaaaagaaaattgaaaaaattcgaccattattaatagatggaactgcatcattgagtcctggaatgatgatgggcatgttcaatatgttaagcaccgtcttgggcgtctccattctgaatcttgggcaaaagagatacaccaagactacttactggtgggatggtcttcaatcgtctgatgattttgctttgattgtgaatgcacccaattatgcaggaattcaagctggagttgacaggttttatcgaacctgtaagctgctcggaattaatatgagcaaaaagaagtcttacataaacagaacaggtacctttgaattcacgagctttttctatcgttatgggtttgttgccaatttcagcatggagcttcctagttttggggtgtctggggtcaatgaatctgcagacatgagtattggagtcactgtcatcaaaaacaatatgataaacaatgaccttggcccagcaactgctcaaatggcccttcagttatttataaaagattacaggtacacttatcgatgccacagaggtgacacacaaatacaaacccggagatcatttgaaataaagaaactatgggaccaaacccgctccaaagctgggctgttggtttctgatggaggccccaatttatataacattaggaatctacatattcctgaagtctgcttgaaatgggagttgatggatgaggattaccaggggcgtttatgcaacccattgaacccgtttgtcagccataaagaaattgaatcagtgaacaatgcagtgataatgccggcacatggtccagccaaaaatatggagtatgacgctgttgcaacaacacactcctgggtacccaaaagaaatcgatccattttgaacacgagccaaagagggatacttgaagatgagcaaatgtatcagaggtgctgcaatttatttgaaaaattcttcccaagtagctcatacagaagaccagttggaatatccagtatggtagaggctatggtttcaagagcccgaattgatgcacggattgatttcgaatctggaaggataaagaaagaggaattcgctgagatcatgaagacctgttccaccattgaagacctcagacggcaaaaatag

>H1N1_PB1_CY037477_A/Virginia/UR06-0065/2007

atggatgtcaatccgactttacttttcttaaaagtgccagcacaaaatgctataagcacaacttttccttatactggtgaccctccttacagccatgggacaggaacagggtacaccatggatacagtcaacaggacacaccagtactcagaaagaggaagatggacaaaaaataccgaaactggagcaccgcaactcaacccaattgatgggccactaccagaagacaatgaaccaagtggctatgcccaaacagattgtgtattagaagcaatggctttccttgaagaatcccatcctggtatctttgaaaactcttgtattgaaacaatggaggttgttcagcaaacaagggtggacaaactgacacaaggcagacagacctatgactggactctaaataggaaccagcctgctgcaacagcattggcaaacactatagaagtattcagatcaaacggcctcatagcaaatgaatctggaaggctaatagacttccttaaagatgtaatggagtcgatggacagagacgaagtagaggtcacaactcattttcaaagaaagaggagagtaagagacaatgtaactaaaaaaatggtgactcaaagaacaataggcaaaaagaaacataaattagacaaaagaagttacctaattagggcattaaccctgaacacaatgaccaaagatgctgagagggggaaactaaaacgcagagcaattgcaaccccaggaatgcaaataagagggtttgtatactttgttgagacactggcaagaagcatatgtgaaaagcttgaacaatcaggattgccagttggaggaaatgagaagaaagcaaagttagcaaatgttgtaaggaagatgatgaccaactcccaggacactgaaatttctttcaccatcactggagataacacaaaatggaacgaaaatcaaaaccctagaatgttcttggccatgatcacatatataaccaaaaatcagcctgaatggttcagaaatattctaagtattgctccaataatgttttcaaacaagatggcgagactaggtaaggggtacatgtttgaaagcaagagtatgaaactgagaactcaaatacctgcagagatgctagtcaacatagatttgaaatatttcaatgattcaactaaaaagaaaattgaaaaaatccgaccattattaatagatggaactgcatcattgagtcctggaatgatgatgggcatgttcaatatgttgagcaccgtcttgggcgtctccattctgaatcttgggcaaaagagatacaccaagactacttactggtgggatggtcttcaatcgtctgatgattttgctttgattgtgaatgcacccaactatgcaggaattcaagctggagttgacaggttttatcgaacctgtaagctgctcggaattaatatgagcaaaaagaagtcttacataaacagaacaggtacctttgaattcacgagctttttctatcgttatgggtttgttgccaatttcagcatggagcttcctagttttggggtgtctggggtcaatgaatctgcagacatgagtattggagtcactgtcatcaaaaacaatatgataaacaatgaccttggcccagcaactgctcaaatggcccttcagttatttataaaagattacaggtacacttatcgatgccacagaggtgacacacaaatacaaacccggagatcatttgagataaagaaactatgggaccaaacccgctccaaagctgggctgttggtctctgatggaggccccaatttatataacattagaaatctccatattcctgaagtttgcttgaaatgggagttgatggatgaggattaccaggggcgtttatgcaacccattaaacccgtttgtcagccataaagagattgaatcagtgaacaatgcagtgataatgccggcacatggcccagccaaaaatatggagtatgacgctgttgcaacaacacactcctgggtccccaaaagaaatcgatccattttgaacacgagccaaagggggatacttgaagatgagcaaatgtatcagaggtgctgcaatttatttgaaaaattcttcccaagtagctcatacagaagaccagttggaatatccagtatggtagaggctatggtttcaagagcccgaattgatgcacggattgatttcgaatctggaaggataaagagggaggaatttgctgagatcatgaagatctgttccaccattgaagacctcagacggcaaaaatag

>H1N1_PB1_HM241709_A/Ytml/NIV9438/2009

atggatgtcaatccgactctacttttcctaaaaattccagcgcaaaatgccataagcaccacattcccttatactggagatcctccatacagccatggaacaggaacaggatacaccatggacacagtaaacagaacacaccaatactcagaaaagggaaagtggacgacaaacacagagactggtgcaccccagctcaacccgattgatggaccactacctgaggataatgaaccaagtgggtatgcacaaacagactgtgttctagaggctatggctttccttgaagaatcccacccaggaatatttgagaattcatgccttgaaacaatggaagttgttcaacaaacaagggtagataaactaactcaaggtcgccagacttatgattggacattaaacagaaatcaaccggcagcaactgcattggccaacaccatagaagtctttagatcgaatggcctaacagctaatgagtcaggaaggctaatagatttcttaaaggatgtaatggaatcaatgaacaaagaggaaatagagataacaacccactttcaaagaaaaaggagagtaagagacaacatgaccaagaagatggtcacgcaaagaacaatagggaagaaaaaacaaagactgaataagagaggctatctaataagagcactgacattaaatacgatgaccaaagatgcagagagaggcaagttaaaaagaagggctatcgcaacacctgggatgcagattagaggtttcgtatactttgttgaaactttagctaggagcatttgcgaaaagcttgaacagtctgggctcccagtagggggcaatgaaaagaaggccaaactggcaaatgttgtgagaaagatgatgactaattcacaagacacagagatttctttcacaatcactggggacaacactaagtggaatgaaaatcaaaatcctcgaatgttcctggcgatgattacatatatcaccagaaatcaacccgagtggttcagaaacatcctgagcatggcacccataatgttctcaaacaaaatggcaagactagggaaagggtacatgttcgagagtaaaagaatgaagattcgaacacaaataccagcagaaatgctagcaagcattgacctgaagtacttcaatgaatcaacaaagaaaaaaattgagaaaataaggcctcttctaatagatggcacagcatcactgagtcctgggatgatgatgggcatgttcaacatgctaagtacggtcttgggagtctcgatactgaatcttggacaaaagaaatacaccaagacaatatactggtgggatgggctccaatcatccgacgattttgctctcatagtgaatgcaccaaaccatgagggaatacaagcaggagtggacagattctacaggacctgcaagttagtgggaatcaacatgagcaaaaagaagtcctatataaataagacagggacatttgaattcacaagctttttttatcgctatggatttgtggccgatttcagcatggagctacccagctttggagtgtctggagtaaatgaatcagctgacatgagtattggagtaacagtgataaagaacaacatgataaacaatgaccttggacctgcaacggcccagatggctcttcaattgttcatcaaagactacagatacacatataggtgccataggggagacacacaaattcagacgagaagatcatttgagttaaagaagctgtgggatcaaacccaatcaaaggtagggctattagtatcagatggaggaccaaacttatacaatatacggaatcttcacattcctgaaatctgcttaaaatgggagctaatggatgatgattatcggggaagactttgtaatcccctgaatccctttgtcagtcataaagagattgattctgtaaacaatgctgtggtaatgccagcccatggtccagccaaaagcatggaatatgatgcagttgcaactacacattcctggattcccaagaggaatcgttctattctcaacacaagccaaaggggaattcttgaggatgaacagatgtaccagaagtgctgcaatctattcgagaaatttttccctagcagttcatataggagaccggttggaatttctagcatggtggaggccatggtgtctagggcccgaattgatgccagggtcgacttcgagtctggacggatcaagaaagaagagttctctgagatcatgaagatctgttccaccattgaagaactcagacggcaaaaataa

>H1N1_PB1_CY122693_A/Singapore/GP1142/2009

atggatgtcaatccgactctacttttcctaaaaattccagcgcaaaatgccataagcaccacattcccttatactggagatcctccatacagccatggaacaggaacaggatacaccatggacacagtaaacagaacacaccaatactcagaaaagggaaagtggacgacaaacacagagactggtgcaccccagctcaacccgattgatggaccactacctgaggataatgaaccaagtgggtatgcacaaacagactgtgttctagaggctatggctttccttgaagaatcccacccaggaatatttgagaattcatgccttgaaacaatggaagttgttcaacaaacaagggtagataaactaactcaaggtcgccagacttatgattggacattaaacagaaatcaaccggcagcaactgcattggccaacaccatagaagtctttagatcgaatggcctaacagctaatgagtcaggaaggctgatagatttcttaaaggatgtaatggaatcaatgaacaaagaggaaatagagataacaacccactttcaaagaaaaaggagagtaagagacaacatgaccaagaagatggtcacacaaagaacaatagggaagaaaaaacaaagactgaataagagaggctatctaataagagcactgacattaaatacgatgaccaaagatgcagagagaggcaagttaaaaagaagggctatcgcaacacctgggatgcagattagaggtttcgtatactttgttgaaactttagctaggagcatttgcgaaaagcttgaacagtctgggctcccagtagggggcaatgaaaagaaggccaaactggcaaatgttgtgagaaagatgatgactaattcacaagacacagagatttctttcacaatcactggggacaacactaagtggaatgaaaatcaaaatcctcgaatgttcctggcgatgattacatatatcaccagaaatcaacccgagtggttcagaaacatcctgagcatggcacccataatgttctcaaacaaaatggcaagactagggaaagggtacatgttcgagagtaaaagaatgaagattcgaacacaaataccagcagaaatgctagcaagcattgacctgaagtacttcaatgaatcaacaaagaagaaaattgagaaaataaggcctcttctaatagatggcacagcatcactgagtcctgggatgatgatgggcatgttcaacatgctaagtacggtcttgggagtctcgatactgaatcttggacaaaagaaatacaccaagacaatatactggtgggatgggctccaatcatccgacgattttgctctcatagtgaatgcaccaaaccatgagggaatacaagcaggagtggacagattctacaggacctgcaagttagtgggaatcaacatgagcaaaaagaagtcctatataaataagacagggacatttgaattcacaagctttttttatcgctatggatttgtggctaattttagcatggagctacccagctttggagtgtctggagtaaatgaatcagctgacatgagtattggagtaacagtgataaagaacaacatgataaacaatgaccttggacctgcaacggcccagatggctcttcaattgttcatcaaagactacagatacacatataggtgccatagaggagacacacaaattcagacgagaagatcatttgagttaaagaagctgtgggatcaaacccaatcaaaggtagggctattagtatcagatggaggaccaaacttatacaatatacggaatcttcacattcctgaagtctgcttaaaatgggagctaatggatgatgattatcggggaagactttgtaatcccctgaatccctttgtcagtcataaagagattgattctgtaaacaatgctgtggtaatgccagcccatggtccagccaaaagcatggaatatgatgccgttgcaactacacattcctggattcccaagaggaatcgttctattctcaacacaagccaaaggggaattcttgaggatgaacagatgtaccagaagtgttgcaatctattcgagaaatttttccctagcagttcatataggagaccggttggaatttctagcatggtggaggccatggtgtctagggcccggattgatgccagggtcgacttcgagtctggacggatcaagaaagaagagttctctgagatcatgaagatctgttccaccattgaagaactcagacggcaaaaataa

>H1N1_PB1_JX625892_A/England/05080815/2010

atggatgtcaatccgactctacttttcctaaaaattccagcgcaaaatgccataagcaccacattcccttatactggagatcctccatacagccatggaacaggaacaggatacaccatggacacagtaaacagaacacaccaatactcagaaaagggaaagtggacgacaaacacagagactggtgcaccccagctcaacccgattgatggaccactacctgaggataatgaaccaagtgggtatgcacaaacagactgtgttctagaggctatggctttccttgaagaatcccacccaggaatatttgagaattcatgccttgaaacaatggaagttgttcaacaaacaagggtagataaactaactcaaggtcgccagacttatgattggacattaaacagaaatcaaccggcagcaactgcattggccaacaccatagaagtctttagatcgaatggcctaacagctaatgagtcaggaaggctaatagatttcttaaaggatgtaatggaatcaatgaacaaagaggaaatagagataacaacccactttcaaagaaaaaggagagtaagagacaacatgaccaagaagatggtcacgcaaagaacaatagggaagaaaaaacaaagactgaataagagaggctatctaataagagcactgacattaaatacgatgaccaaagatgcagagagaggaaagttaaaaagaagggctatcgcaacacctgggatgcagattagaggcttcgtatactttgttgaaactttagctaggagcatttgcgaaaagcttgaacagtctgggctcccagtagggggcaatgaaaagaaggccaaactggcaaatgttgtgagaaagatgatgactaattcacaagacacagagatttctttcacaatcactggggacaacactaagtggaatgaaaaccaaaatcctcgaatgttcctggcgatgattacatatatcaccagaaatcaacccgagtggttcagaaacatcctgagcatggcacccataatgttctcaaacaaaatggcaagactagggaaagggtacatgttcgagagtaaaagaatgaagattcgaacgcaaataccagcagaaatgctagcaagcattgacctgaagtacttcaatgaatcaacaaagaagaaaattgagaaaataaggcctcttttaatagatggcacagcatcactgagtcctgggatgatgatgggcatgttcaacatgctaagtacggttttgggagtctcgatactgaatcttggacaaaagaaatacaccaagacaatatactggtgggatgggctccaatcatccgacgattttgctctcatagtgaatgcaccaaaccatgagggaatacaagcaggagtggacagattttacaggacctgcaagttagtgggaatcaacatgagcaaaaagaagtcctatataaataagacagggacatttgaattcacaagctttttttatcgctatggatttgtggctaattttagcatggagctacccagctttggagtgtctggagtaaatgaatcagctgacatgagtattggagtaacagtgataaagaacaacatgataaacaatgaccttggacctgcaacggcccagatggctcttcaattgttcatcaaagactacagatacacatataggtgccataggggagacacacaaattcagacgagaagatcatttgagttaaagaagctgtgggatcaaacccaatcaaaggtagggctattagtatcagatggaggaccaaacttatacaatatacggaatcttcacattcctgaagtctgcttaaaatgggagctaatggatgatgactatcggggaagactttgtaatcccctgaatccctttgtaagtcataaagagattgattctgtaaacaatgctgtgataatgccagctcatggtccagccaaaagcatggaatatgatgccgttgcaactacacattcctggattcccaagaggaatcgttctattctcaacacaagccaaaggggaattcttgaggatgaacagatgtaccagaagtgctgcaatctattcgagaaatttttccctagcagttcatataggagaccggttggaatttctagcatggtggaggccatggtgtctagggcccggattgatgccagggtcgactttgagtctggacggatcaagaaagaagagttctctgagatcatgaagatctgttccaccattgaagaactcagacggcaaaaataa

>H1N1_PB1_KM437656_A/Finland/110/2014

atggatgtcaatccgactctacttttcctaaaaattccagcacaaaatgccataagcaccacattcccttatactggagatcctccatacagccatggaacaggaacaggatacaccatggacacagtaaacagaacacaccaatactcagaaaagggaaagtggacaacaaacacagagactggtgcaccccagctcaacccgattgatggaccactacctgaggataatgaaccaagtgggtatgcacaaacagactgtgttctagaggctatggctttccttgaagaatcccacccaggaatatttgagaattcatgccttgaaacaatggaagttgttcaacaaacaagggtagataaactgactcaaggtcgccagacttatgattggacattaaacagaaatcaaccggcagcaactgcattggccaacaccatagaagtctttagatcgaatgacctaacagctaacgagtcaggaaggctaatagatttcttaaaggatgtaatggaatcaatgaacaaagaggaaatagagataacaacccactttcaaagaaaaagaagagtaagagacaacatgaccaagaagatggtcacgcaaagaacaataggtaagaaaaaacagagactgaataagagaggctatctaataagagcactgacattaaatacgatgaccaaagatgcagagagaggcaagttaaaaagaagggctatcgcaacacctgggatgcagattagaggtttcgtatactttgttgaaactttagctaggagcatttgcgaaaagcttgaacagtctgggctcccagtagggggcaatgaaaagaaggccaaattggcaaatgttgtgagaaagatgatgactaattcacaagatacagagatttctttcacaatcactggggacaacactaagtggaatgaaaatcaaaatcctcgaatgttcctggcgatgattacatatatcaccagaaatcaacccgagtggtttagaaacatcctgagcatggcacccataatgttctcaaataaaatggcaagactagggaaagggtacatgttcgagagtaaaagaatgaagattcgaacacaaataccagcagaaatgctagcaaacattgatctgaagtacttcaatgaatcaacaaagaagaaaattgagaaaataaggcctcttttaatggatggcacagcatcactgagtcctgggatgatgatgggcatgttcaacatgctaagtacggtcttgggagtctcgatactgaatcttggacaaaagaaatacaccaagacaacatactggtgggatgggctccaatcatccgacgattttgctctcatagtgaatgcaccaaaccatgaaggaatacaagcaggagtggacagattctacaggacctgcaagttagtgggaatcaacatgagcaaaaagaaatcctatataaataagacagggacatttgaattcacaagctttttttatcgctatggatttgtggctaattttagcatggagctacccagctttggagtgtctggagtaaatgaatcagctgacatgagtattggaataacagtgataaagaacaacatgataaacaatgaccttggacctgcaacggcccagatggctcttcaattgttcatcaaagactacagatacacatataggtgccataggggagacacacaaattcagacaagaagatcatttgagttaaagaagctatgggatcaaacccaatcaaaggtagggctattagtatcagatggaggaccaaacttatacaacatacggaatcttcacattcctgaagtctgcttaaaatgggagctaatggatgatgattatcggggaagactttgtaatcccctgaatccctttgtgagtcataaggagattgattctataaacaatgctgtggtaatcccagcccatggtccagccaaaagcatggaatatgatgccgttgcaactacacattcctggattcccaagaggaatcgttctattctcaacacaagccaaaggggaattcttgaggatgaacagatgtaccagaagtgttgcaatctattcgagaaatttttccctagcagttcatataggagaccggttggaatttctagcatggtggaggccatggtgtctagggcccggattgatgccagggtcgactttgagtctggacggatcaagaaagaagagttctctgagatcatgaagatctgttccaccattgaagaactcagacggcaaaaataa

>H1N1_PB1_LN866282_A/England/12240612/2010

atggatgtcaatccgactctacttttcctaaaaattccagcgcaaaatgccataagcaccacattcccttatactggagatcctccatacagccatggaacaggaacaggatacaccatggacacagtgaacagaacacaccaatactcagaaaagggaaagtggacgacaaacacagagactggtgcaccccagctcaacccgattgatggaccactacctgaggataatgaaccaagtgggtatgcacaaacagactgtgttctagaggctatggctttccttgaagaatcccacccagggatatttgagaattcatgccttgaaacaatggaagttgttcaacaaacaagggtagataaactaactcaaggtcgccagacttatgattggacattaaacagaaatcaaccggcagcaactgcattggccaacaccatagaagtctttagatcgaatggactaacagctaatgagtcaggcaggctaatagatttcttaaaggatgtaatggaatcaatgaacaaagaggaaatagagataacaacccactttcaaagaaaaaggagagtaagagacaacatgaccaagaagatggtcacgcaaagaacaatagggaagaaaaaacaaagactgaataagagaggctatctaataagagcactgacattaaatacgatgaccaaagatgcagagagaggcaagttaaaaagaagggctatcgcaacacctgggatgcagattagaggtttcgtatactttgttgaaactttagctaggagcatttgcgaaaagcttgaacagtctgggctcccagtagggggcaatgaaaagaaggccaaactggcaaatgttgtgagaaagatgatgactaattcacaagacacagagatttctttcacaatcactggggacaacactaagtggaatgaaaatcaaaatcctcgaatgttcctggcgatgattacatatatcaccagaaatcaacccgagtggttcagaaacatcctgagcatggcacccataatgttctcaaacaaaatggcaagactagggaaagggtacatgttcgagagtaaaagaatgaagattcgaacacaaataccagcagaaatgctagcaagcattgacctgaagtacttcaatgaatcaacaaagaagaaaattgagaaaataaggcctcttttaatagatggcacagcatcactgagtcctgggatgatgatgggcatgttcaacatgctaagtacggtcttgggagtctcgatactgaatcttggacaaaagaaatataccaagacaatatactggtgggatgggctccaatcatccgacgattttgctctcatagtgaatgcaccaaaccatgagggaatacaagcaggagtggacagattctacaggacctgcaagttagtgggaatcaacatgagcaaaaagaagtcctatataaataagacagggacatttgaattcacaagctttttttatcgctatggatttgtggctaattttagcatggagctacccagctttggagtgtctggagtaaatgaatcagctgacatgagtattggagtaacagtgataaagaacaacatgataaacaatgaccttggacctgcaacggcccagatggctcttcaattgttcatcaaagactacagatacacatataggtgccataggggagacacacaaattcagacgagaagatcatttgagttaaagaagctgtgggatcaaacccaatcaaaggtagggctattagtatcagatggaggaccaaacttatacaatatacggaatcttcacattcctgaagtctgcttaaaatgggagctaatggatgatgattatcggggaagactttgtaatcccctgaatccctttgtaagtcataaagagattgattctgtaaacaatgctgtggtaatgccagcccatggtccagccaaaagcatggaatatgatgccgttgcaactacacattcctggattcccaagaggaatcgttctattctcaacacaagccaaaggggaattcttgaggatgaacagatgtaccagaagtgctgcaatctattcgagaaatttttccctagcagttcatataggagaccggttggaatttctagcatggtggaggccatggtgtctagggcccggattgatgccagggtcgattttgagtctggacggatcaagaaagaagagttctctgagatcatgaagatctgttccaccattgaagaactcagacggcaaaaataa

>H1N1_PB1_CY062152_A/New York/2963/2010

atggatgtcaatccgactctacttttcctaaaaattccagcgcaaaatgccataagcaccacattcccttatactggagatcctccatacagccatggaacaggaacaggatacaccatggacacagtaaacagaacacaccaatactcagaaaagggaaagtggacgacaaacacagagactggtgcaccccagctcaacccgattgatggaccactacctgaggataatgaaccaagtgggtatgcacaaacagactgtgttctagaggctatggctttccttgaagaatcccacccaggaatatttgagaattcatgccttgaaacaatggaagttgttcaacaaacaagggtagataaactaactcaaggtcgccagacttatgattggacattaaacagaaatcaaccggcagcaactgcattggccaacaccatagaagtctttagatcgaatggcctaacagctaatgagtcaggaaggctaatagatttcttaaaggatgtaatggaatcaatgaacaaagaggaaatagagataacaacccactttcaaagaaaaaggagagtaagagacaacatgaccaagaagatggtcacgcaaagaacaatagggaagaaaaaacaaagactgaataagagaggctatctaataagagcactgacattaaatacgatgaccaaagatgcagagagaggcaagttaaaaagaagggctatcgcaacacctgggatgcagattagaggtttcgtatactttgttgaaactttagctaggagcatttgcgaaaagcttgaacagtctgggctcccagtagggggcaatgaaaagaaggccaaactggcaaatgttgtgagaaagatgatgactaattcacaagacacagagatttctttcacaatcactggggacaacactaagtggaatgaaaatcaaaatcctcgaatgttcctggcgatgattacatatatcaccagaaatcaacccgagtggttcagaaacatcctgagcatggcacccataatgttctcaaacaaaatggcaagactagggaaagggtacatgttcgagagtaaaagaatgaagattcgaacacaaataccagcagaaatgctagcaagcattgacctgaagtacttcaatgaatcaacaaagaagaaaattgagaaaataaggcctcttctaatagatggcacagcatcactgagtcctgggatgatgatgggcatgttcaacatgctaagtacggtcttgggagtctcgatactgaatcttggacaaaagaaatacaccaagacaatatactggtgggatgggctccaatcatccgacgattttgctctcatagtgaatgcaccaaaccatgagggaatacaagcaggagtggacagattctacaggacctgcaagttagtgggaatcaacatgagcaaaaagaagtcctatataaataagacagggacatttgaattcacaagctttttttatcgctatgggtttgtggctaactttagcatggagctacccagctttggagtgtctggagcaaatgaatcagctgacatgagtattggagtaacagtgataaaaaacaacatgataaacaatgaccttggacctgcaacggcccagatggctcttcaattgttcatcaaagactacagatacacatataggtgccataagggagacacacaaattcagacgagaagatcatttgagttaaagaagctgtgggatcaaacccaatcaaaggtagggctattagtatcagatggaggaccaaacttatacaatatacggaatcttcacattcctgaagtctgcttaaaatgggagctaatggatgatgattatcggggaagactttgtaatcccctgaatccctttgtcagtcataaagagattgattctgtaaacaatgctgtggtaatgccagcccatggtccagccaaaagcatggaatatgatgccgttgcaactacacattcctggattcccaagaggaatcgttctattctcaacacaagccaaaggggaattcttgaagatgaacagatgtaccagaagtgctgcaatctattcgagaaatttttccctagcagttcatataggagaccggttggaatttctagcatggtggaggccatggtgtctagggcccggattgatgccagggtcgacttcgagtctggacggatcaagaaagaagagttctctgagatcatgaagatctgttccaccattgaagaactcagacggcaaaaataa

>H1N1_PB1_CY071317_A/Athens/INS420/2010

atggatgtcaatccgactctacttttcctaaaaattccagcgcaaaatgccataagcaccacattcccttatactggagatcctccatacagccatggaacaggaacaggatacaccatggacacagtaaacagaacacaccaatactcagaaaagggaaagtggacgacaaacacagagactggtgcaccccagctcaacccgattgatggaccactacctgaggataatgaaccaagtgggtatgcacaaacagactgtgttctagaggctatggctttccttgaagaatcccacccaggaatatttgagaattcatgccttgaaacaatggaagttgttcaacaaacaagggtagataaactaactcaaggtcgccagacttatgattggacattaaacagaaatcaaccggcagcaactgcgttggccaacaccatagaagtctttagatcgaatggcctaacagctaatgagtcaggaaggctaatagatttcttaaaggatgtaatggaatcaatgaacaaagaggaaatagagataacaacccactttcaaagaaaaaggagagtaagagacaacatgaccaagaagatggtcacgcaaagaacaatagggaagaaaaaacaaagactgaataagagaggctatctaataagagcactgacattaaatacgatgaccaaagatgcagagagaggcaagttaaaaagaagggctatcgcaacacctgggatgcagattagaggtttcgtatactttgttgaaactttagctaggagcatttgcgaaaagcttgaacagtctgggctcccagtagggggcaatgaaaagaaggccaaactggcaaatgttgtgagaaagatgatgactaattcacaagacacagagatttctttcacaatcactggggacaacactaagtggaatgaaaatcaaaatcctcgaatgttcctggcgatgattacatatatcaccagaaatcaacccgagtggttcagaaacatcctgagcatggcacccataatgttctcaaacaaaatggcaagactagggaaagggtacatgttcgagagtaaaaaaatgaagattcgaacacaaataccagcagaaatgctagcaagcattgacctgaagtacttcaatgaatcaacaaagaagaaaattgagaaaataaggcctcttctaatagatggcacagcatcactgagtcctgggatgatgatgggcatgttcaacatgctaagtacggtcttgggagtctcgatactgaatcttggacaaaagaaatacaccaagacaatatactggtgggatgggctccaatcatccgacgattttgctctcatagtgaatgcaccaaaccatgagggaatacaggcaggagtggacagattctacaggacctgcaagttagtgggaatcaacatgagcaaaaagaagtcctatataaataagacagggacatttgaattcacaagctttttttatcgctatggatttgtggctaattttagcatggagctacccagctttggagtgtctggagtaaatgaatcagctgacatgagtattggagtaacagtgataaagaacaacatgataaacaatgaccttggacctgcaacggcccagatggctcttcaactgttcatcaaagactacagatacacatataggtgccataggggagacacacaaattcagacgagaagatcatttgagttaaagaagctgtgggatcaaacccaatcaaaggtagggctattagtatcagatggaggaccaaacttatacaatatacggaatcttcacattcctgaagtctgcttaaaatgggagctaatggatgatgattatcggggaagactttgtaatcccctgaatccctttgtcagtcataaagagattgattctgtaaataatgctgtggtaatgccagcccatggtccagccaaaagcatggaatatgatgccgttgcaactacacattcctggattcccaagaggaatcgttccattctcaacacaagccaaaggggaattcttgaggatgaacagatgtaccagaagtgctgcaatctattcgagaaatttttccctagcagttcatataggagaccggttggaatttctagcatggtggaggccatggtgtctagggcccggattgatgccagggtcgacttcgagtctggacggatcgggaaggaagagttctctgagatcatgaagatctgttccaccattgaagaactcagacggcaaaaataa

>H1N1_PB1_CY083294_A/San Diego/WRAIR1647P/2009

atggatgttaatccgactctacttttcctaaaaattccagcgcaaaatgccataagcaccacattcccttatactggagatcctccatacagccatggaacaggaacaggatacaccatggacacagtaaacagaacacaccaatactcagaaaagggaaagtggacgacaaacacagagactggtgcaccccagctcaacccgattgatggaccactacctgaggataatgaaccaagtgggtatgcacaaacagactgtgttctagaggctatggctttccttgaagaatcccacccaggaatatttgagaattcatgccttgaaacaatggaagttgttcaacaaacaagggtagataaactaactcaaggtcgccagacttatgattggacattaaacagaaatcaaccggcagcaactgcattggccaacaccatagaagtctttagatcgaatggcctaacagctaatgagtcaggaaggctaatagatttcttaaaggatgtaatggaatcaatgaacaaagaggaaatagagataacaacccactttcaaagaaaaaggagagtaagagacaacatgaccaagaagatggtcacgcaaagaacaatagggaagaaaaaacaaagactgaataagagaggctatctaataagagcactgacattaaatacgatgaccaaagatgcagagagaggcaagttaaaaagaagggctatcgcaacacctgggatgcagattagaggtttcgtacactttgttgaaactttagctaggagcatttgcgaaaagcttgaacagtctgggctcccagtagggggcaatgaaaagaaggccaaactggcaaatgttgtgagaaagatgatgactaattcacaagacacagagatttctttcacaatcactggggacaacactaagtggaatgaaaatcaaaatcctcgaatgttcctggcgatgattacatatatcaccagaaatcaacccgagtggttcagaaacatcctgagcatggcacccataatgttctcaaacaaaatggcaagactagggaaagggtacatgttcgagagtaaaagaatgaagattcgaacacaaataccagcagaaatgctagcaagcattgacctgaagtacttcaatgaatcaacaaagaagaaaattgagaaaataaggcctcttctaatagatggcacagcatcactgagtcctgggatgatgatgggcatgttcaacatgctaagtacggtcttgggagtctcgatactgaatcttggacaaaagaaatacaccaagacaatatactggtgggatgggctccaatcatccgacgattttgctctcatagtgaatgcaccaaaccatgagggaatacaagcaggagtggacagattctacaggacctgcaagttagtgggaatcaacatgagcaaaaagaagtcctatataaataagacagggacatttgaattcacaagctttttttatcgctatggatttgtggctaattttagcatggagctacccagctttggagtgtctggagtaaatgaatcagctgacatgagtattggagtaacagtgataaagaacaacatgataaacaatgaccttggacctgcaacggcccagatggctcttcaattgttcatcaaagactacagatacacatataggtgccataggggagacacacaaattcagacgagaagatcatttgagttaaagaagctgtgggatcaaacccaatcaaaggtagggctattagtatcagatggaggaccaaacttatacaatatacggaatcttcacattcctgaagtctgcttaaaatgggagctaatggatgatgattatcggggaagactttgtaatcccctgaatccctttgtcagtcataaagagattgattctgtaaacaatgctgtggtaatgccagcccatggtccagccaaaagcatggaatatgatgccgttgcaactacacattcctggattcccaagaggaatcgttctattctcaacacaagccaaaggggaattcttgaggatgaacagatgtaccagaagtgctgcaatctattcgagaaatttttccctagcagttcatataggagaccggttggaatttctagcatggtggaggccatggtgtctagggcccggattgatgccagggtcgacttcgagtctggacggatcaagaaagaagagttctctgagatcatgaagatctgttccaccattgaagaactcagacggcaaaaataa

>H1N1_PB1_JN714498_A/Joshkar-Ola/CRIE-BLP/2011

atggatgtcaatccgactctacttttcctaaaaattccagcgcaaaatgccataagcaccacattcccttatactggagatcctccatacagccatggaacaggaacaggatacaccatggacacagtaaacagaacacaccaatactcagaaaagggaaagtggacgacaaacacagagactggtgcaccccagctcaacccgattgatggaccactacctgaggataatgaaccaagtgggtatgcacaaacagactgtgttctagaggctatggctttccttgaagaatcccacccaggaatatttgagaattcatgccttgaaacaatggaagttgttcaacaaacaagggtagataaactgactcaaggtcgccagacttatgattggacattaaacagaaatcaaccggcagcaactgcattggccaacaccatagaagtctttagatcgaatggcctaacagctaatgagtcaggaaggctaatagatttcttaaaggatgtaatggaatcaatgaacaaagaggaaatagagataacaacccactttcaaagaaaaaggagagtaagagacaacatgaccaagaagatggtcacgcaaagaacaatagggaagaaaaaacagagactgaataagagaggctatctaataagagcactgacattaaatacgatgaccaaagatgcagagagaggcaagttaaaaagaagggctatcgcaacacctgggatgcagattagaggtttcgtatactttgttgaaactttagctaggagcatttgcgaaaagcttgaacagtctgggctcccagtagggggcaatgaaaagaaggccaaactggcaaatgttgtgagaaagatgatgactaattcacaagacacagagatttctttcacaatcactggggacaacactaagtggaatgaaaatcaaaatcctcgaatgttcctggcgatgattacatatatcaccagaaatcaacccgagtggtttagaaacatcctgagcatggcacccataatgttctcaaacaaaatggcaagactagggaaagggtacatgttcgagagtaaaagaatgaagattcgaacacaaataccagcagaaatgctagcaagcattgacctgaagtacttcaatgaatcaacaaagaagaaaattgagaaaataaggcctcttttaatggatggcacagcatcactgagtcctgggatgatgatgggtatgttcaacatgctaagtacggtcttgggagtctcgatactgaatcttggacaaaagaaatacaccaagacaacatactggtgggatgggctccaatcatccgacgattttgctctcatagtgaatgcaccaaaccatgagggaatacaagcaggagtggacagattctacaggacctgcaagttagtgggaatcaacatgagcaaaaagaagtcctatataaataagacagggacatttgaattcacaagctttttttatcgctatggatttgtggctaattttagcatggagctacccagctttggagtgtctggagtaaatgaatcagctgacatgagtattggagtaacagtgataaagaacaacatgataaacaatgaccttggacctgcaacggcccagatggctcttcaattgttcatcaaagactacagatacacatataggtgccataggggagacacacaaattcagacgagaagatcatttgagttaaagaagctgtgggatcaaacccaatcaaaggtagggctattagtatcagatggaggaccaaacttatacaatatacggaatcttcacattcctgaagtctgcttaaaatgggagctaatggatgatgattatcggggaagactttgtaatcccctgaatccctttgtaagtcataaggagattgattctgtaaacaatgctgtggtaatgccagcccatggtccagccaaaagcatggaatatgatgccgttgcaactacacattcctggattcccaagaggaatcgttctattctcaacacaagccaaaggggaattcttgaggatgaacagatgtaccagaagtgctgcaatctattcgagaaatttttccctagcagttcatataggagaccggttggaatttctagcatggtggaggccatggtgtctagggcccggattgatgccagggtcgactttgagtctggacggatcaagaaagaagagttctctgagatcatgaagatctgttccaccattgaagaactcagacggcaaaaataa

>H1N1_PB1_CY065628_A/England/398/2009

atggatgtcaatccgactctacttttcctaaaaattccagcgcaaaatgccataagcaccacattcccttatactggagatcctccatacagccatggaacaggaacaggatacaccatggacacagtaaacagaacacaccaatactcagaaaagggaaagtggacgacaaacacagagactggtgcaccccagctcaacccgattgatggaccactacctgaggataatgaaccaagtgggtatgcacaaacagactgtgttctagaggctatggctttccttgaagaatcccacccaggaatatttgagaattcatgccttgaaacaatggaagttgttcaacaaacaagggtagataaactaactcaaggtcgccagacttatgattggacattaaacagaaatcaaccggcagcaactgcattggccaacaccatagaagtctttagatcgaatggcctaacagctaatgagtcaggaaggctaatagatttcttaaaggatgtaatggaatcaatgaacaaagaggaaatagagataacaacccactttcaaagaaaaaggagagtaagagacaacatgaccaagaagatggtcacgcaaagaacaatagggaagaaaaaacaaagactgaataagagaggctatctaataagagcactgacattaaatacgatgaccaaagatgcagagagaggcaagttaaaaagaagggctatcgcaacacctgggatgcagattagaggtttcgtatactttgttgaaactttagctaggagcatttgcgaaaagcttgaacagtctgggctcccagtagggggcaatgaaaagaaggccaaactggcaaatgttgtgagaaaaatgatgactaattcacaagacacagagatttctttcacaatcactggggacaacactaagtggaatgaaaatcaaaatcctcgaatgttcctggcgatgattacatatatcaccagaaatcaacccgagtggttcagaaacatcctgagcatggcacccataatgttctcaaacaaaatggcaagactagggaaagggtacatgttcgagagtaaaagaatgaagattcgaacacaaataccagcagaaatgctagcaagcattgacctgaagtacttcaatgaatcaacaaagaagaaaattgagaaaataaggcctcttctaatagatggcacagcatcactgagtcctgggatgatgatgggcatgttcaacatgctaagtacggtcttgggagtctcgatactgaatcttggacaaaagaaatacaccaagacaatatactggtgggatgggctccaatcatccgacgattttgctctcatagtgaatgcaccaaaccatgagggaatacaagcaggagtggacagattctacaggacctgcaagttagtgggaatcaacatgagcaaaaagaagtcctatataaataagacagggacatttgaattcacaagctttttttatcgctatggatttgtggctaattttagcatggagctacccagctttggagtgtctggagtaaatgaatcagctgacatgagtattggagtaacagtgataaagaacaacatgataaacaatgaccttggacctgcaacggcccagatggctcttcaattgttcatcaaagactacagatacacatataggtgccataggggagacacacaaattcagacgagaagatcatttgagttaaagaagctgtgggatcaaacccaatcaaaggtagggctattagtatcagatggaggaccaaacttatacaacatacggaatcttcacattcctgaagtctgcttaaaatgggagctaatggatgatgattatcggggaagactttgtaatcccctgaatccctttgtcagtcataaagagattgattctgtaaacaatgctgtggtaatgccagcccatggtccagccaaaagcatggaatatgatgccgttgcaactacacattcctggattcccaagaggaatcgttctattctcaacacaagccaaaggggaattcttgaggatgaacagatgtaccagaagtgctgcaatctattcgagaaatttttccctagcagttcatataggagaccagttggaatttctagcatggtggaggccatggtgtctagggcccggattgatgccagggtcgacttcgagtctggacggatcaagaaagaagagttctctgagatcatgaagatctgttccaccattgaagagctcagacggcaaaaataa

>H1N1_PB1_JQ431370_A/Reunion/0215-4-M1E/2009

atggatgtcaatccgactctacttttcctaaaaattccagcgcaaaatgccataagcaccacattcccttatactggagatcctccatacagccatggaacaggaacaggatacaccatggacacagtaaacagaacacaccaatactcagaaaagggaaagtggacgacaaacacagagactggtgcaccccagctcaacccgattgatggaccactacctgaggataatgaaccaagtgggtatgcacaaacagactgtgttctagaggctatggctttccttgaagaatcccacccaggaatatttgagaattcatgccttgaaacaatggaagttgttcaacaaacaagggtagataaactaactcaaggtcgccagacttatgattggacattaaacagaaatcaaccggcagcaactgcattggccaacaccatagaagtctttagatcgaatggcctaacagctaatgagtcaggaaggctaatagatttcttaaaggatgtaatggaatcaatgaacaaagaggaaatagagataacaacccactttcaaagaaaaaggagagtaagagacaacatgaccaagaagatggtcacgcaaagaacaatagggaagaaaaaacaaagactgaataagagaggctatctaataagagcactgacattaaatacgatgaccaaagatgcagagagaggcaagttaaaaaggagggctatcgcaacacctgggatgcagattagaggtttcgtatactttgttgaaactttagctaggagcatttgcgaaaagcttgaacagtctgggctcccagtagggggcaatgaaaagaaggccaaactggcaaatgttgtgagaaagatgatgactaattcacaagacacagagatttctttcacaatcactggggacaacactaagtggaatgaaaatcaaaatcctcgaatgttcctggcgatgattacatatatcaccagaaatcaacccgagtggttcagaaacatcctgagcatggcacccataatgttctcaaacaaaatggcaagactagggaaagggtacatgttcgagagtaaaagaatgaagattcgaacacaaataccagcagaaatgctagcaagcattgacctgaagtacttcaatgaatcaacaaagaagaaaattgagaaaataaggcctcttctaatagatggcacagcatcactgagtcctgggatgatgatgggcatgttcaacatgctaagtacggtcttgggagtctcgatactgaatcttggacaaaagaaatacaccaagacaacatactggtgggatgggctccaatcatccgacgattttgctctcatagtgaatgcaccaaaccatgagggaatacaagcaggagtggacagattctacaggacctgcaagttagtgggaatcaacatgagcaaaaagaagtcctatataaataagacagggacatttgaattcacaagctttttttatcgctatggatttgtggctaattttagcatggagctacccagctttggagtgtctggagtaaatgaatcagctgacatgagtattggagtaacagtgataaagaacaacatgataaacaatgaccttggacctgcaacggcccagatggctcttcaattgttcatcaaagactacagatacacatataggtgccataggggagacacacaaattcagacgagaagatcatttgagttaaagaagctgtgggatcaaacccaatcaaaggtagggctattagtatcagatggaggaccaaacttatacaatatacggaatcttcacattcctgaagtctgcttaaaatgggagctaatggatgatgattatcggggaagactttgtaatcccctgaatccctttgtcagtcataaagagattgattctgtaaacaatgctgtggtaatgccagcccatggtccagccaaaagcatggaatatgatgccgttgcaactacacattcctggattcccaagaggaatcgttctattctcaacacaagccaaaggggaattcttgaggatgaacagatgtaccagaagtgctgcaatctattcgagaaatttttccctagcagttcatataggagaccggttggaatttctagcatggtggaggccatggtgtctagggcccggattgatgccagggtcgacttcgagtctggacggatcaagaaagaagagttctctgagatcatgaagatctgttccaccattgaagaactcagacggcaaaaataa

>H1N1_PB1_CY129457_A/Athens/INS559/2011

atggatgtcaatccgactctactcttcctaaaaactccagcgcaaaatgccataagcaccacattcccttatactggagatcctccatacagccatggaacaggaacaggatacaccatggacacagtgaacagaacacaccaatactcagaaaagggaaagtggacgacaaacacagagactggtgcaccccagctcaacccgattgatggaccactacctgaggataatgaaccaagtgggtatgcacaaacagactgtgttctagaggctatggctttccttgaagaatcccacccagggatatttgagaattcatgccttgaaacaatggaagttgttcaacaaacaagggtagataaactaactcaaggtcgccagacttatgattggacattaaacagaaatcaaccggcagcaactgcattggccaacaccatagaagtctttagatcgaatggcctaacagctaatgagtcaggcaggctaatagatttcttaaaggatgtaatggaatcaatgaacaaagaggaaatagagataacaacccactttcaaagaaaaaggagagtaagagacaacatgaccaagaagatggtcacgcaaagaacaatagggaagaaaaaacaaagactgaataagagaggctatctaataagagcactgacattaaatacgatgaccaaagatgcagagagaggcaagttaaaaagaagggctatcgcaacacctgggatgcagattagaggtttcgtatactttgttgaaactttagctaggagcatttgcgaaaagcttgaacagtctgggctcccagtagggggcaatgaaaagaaggccaaactggcaaatgttgtgagaaagatgatgactaattcacaagacacagagatttctttcacaatcactggggacaacactaagtggaatgaaaatcaaaatcctcgaatgttcctggcgatgattacatatatcaccagaaatcaacccgagtggttcagaaacatcctgagcatggcacccataatgttctcaaacaaaatggcaagactagggaaagggtacatgttcgagagtaaaagaatgaagattcgaacacaaataccagcagaaatgctagcaagcattgacctgaagtacttcaatgaatcaacaaagaagaaaattgagaaaataaggcctcttttaatagatggcacagcatcactgagtcctgggatgatgatgggcatgttcaacatgctaagtacggtcttgggagtctcgatactgaatcttggacaaaagaaatacaccaagacaatatactggtgggatgggctccaatcatccgacgattttgctctcatagtgaatgcaccaaaccatgagggaatacaagcaggagtggacagattctacaggacctgcaagttagtgggaatcaacatgagcaaaaagaagtcctatataaataagacagggacatttgaattcacaagctttttttatcgctatggatttgtggctaattttagcatggagctacccagctttggagtgtctggagtaaatgaatcagctgacatgagtattggagtaacagtgataaagaacaacatgataaacaatgaccttggacctgcaacggcccagatggctcttcaattgttcatcaaagactacagatacacatataggtgccataggggagacacacaaattcagacgagaagatcatttgagttaaagaagctgtgggatcaaacccaatcaaaggtagggctattagtatcagatggaggaccaaacttatacaatatacggaatcttcacattcctgaagtctgcttaaaatgggagctaatggatgatgattatcggggaagactttgtaatcccctgaatccctttgtaggtcataaagagattgattctgtaaacaatgctgtggtaatgccagcccatggtccagccaaaagcatggaatatgatgccgttgcaactacacattcctggattcccaagaggaatcgttctattctcaacacaagccaaaggggaattcttgaggatgaacagatgtaccagaagtgctgcaatctattcgagaaatttttccctagcagttcatataggagaccggttggaatttctagcatggtggaggccatggtgtctagggcccggattgatgccagggtcgactttgagtctggacggatcaagaaagaagagttctctgagatcatgaagatctgttccaccattgaagaactcagacggcaaaaataa

>H1N1_PB1_CY181767_A/Nicaragua/AGA2-27/2011

atggatgtcaatccgactctacttttcctaaaaattccagcgcaaaatgccataagcaccacattcccttatactggagatcctccatacagccatggaacaggaacaggatacaccatggacacagtaaacagaacacaccaatactcagaaaagggaaagtggacgacaaacacagagactggtgcaccccagctcaacccgattgatggaccattacctgaggataatgaaccaagtgggtatgcacaaacagactgtgttctagaggctatggctttccttgaagaatcccacccaggaatatttgagaattcatgccttgaaacaatggaagttgttcaacaaacaagggtagataaactaactcaaggtcgccagacttatgattggacattaaacagaaatcaaccggcagcaactgcattggccaacaccatagaagtctttagatcgaatggcctaacagctaatgagtcaggaaggctaatagatttcttaaaggatgtaatggaatcaatgaacaaagaggaaatagagataacaacccactttcaaagaaaaaggagagtgagagacaacatgaccaagaagatggtcacgcaaagaacaatagggaagaaaaaacaaagactgaataagagaggctatctaataagagcactgacattaaatacgatgaccaaagatgcagagagaggcaagttaaaaagaagggctatcgcaacacctgggatgcagattagaggtttcgtatactttgttgaaactttagctaggagcatttgcgaaaagcttgaacagtctgggctcccagtagggggcaatgaaaaaaaggccaaactggcaaatgttgtgagaaagatgatgactaattcacaagacacagagatttctttcacaatcactggggacaacactaagtggaatgaaaaccaaaatcctcgaatgttcctggcgatgattacatatatcaccagaaatcaacccgagtggttcagaaacatcctgagcatggcacccataatgttctcaaacaaaatggcaagactagggaaagggtacatgttcgagagtaaaagaatgaagattcgaacgcaaataccagcagaaatgctagcaagcattgacctgaagtacttcaatgagtcaacaaagaagaaaattgagaaaataaggcctcttttaatagatggcacagcatcactgagtcctgggatgatgatgggcatgttcaacatgctaagtacggtcttgggagtctcgatactgaatcttggacaaaagaaatacaccaagacaatatactggtgggatgggctccaatcatccgacgattttgctctcatagtgaatgcaccaaaccatgagggaatacaagcaggagtggacagattttacaggacctgcaagttagtgggaatcaacatgagcaaaaagaagtcctatatcaataagacagggacatttgaattcacaagcttcttttatcgctatggatttgtggctaattttagcatggagctacccagctttggagtggctggagtaaatgaatcagctgacatgagtattggagtaacagtgataaagaacaacatgataaacaatgaccttggacctgcaacggcccagatggctcttcaattgttcatcaaagactacagatacacatataggtgccataggggagacacacaaattcagacgagaagatcatttgagttaaagaagctgtgggatcaaacccaatcaaaggtagggctattagtatcagatggaggaccaaacttatacaatatacggaatcttcacattcctgaagtctgcttaaaatgggagctaatggatgatgattatcggggaagactttgtaatcccctgaatccctttgtaagtcataaagagattgattctgtaaacaatgctgtggtaatgccagcccatggtccagccaaaagcatggaatatgatgccgttgcaactacacattcctggattcccaagaggaatcgttctattctcaacacaagccaaaggggaattcttgaggatgaacagatgtaccagaagtgctgcaatctattcgagagatttttccctagcagttcatataggagaccggttggaatttctagcatggtggaggccatggtgtctagggcccggattgatgccagggtcgactttgagtctggacggatcaagaaagaagagttctctgagatcatgaagatctgttccaccattgaagaactcagacggcaaaaataa

>H1N1_PB1_KM366677_A/Helsinki/723M/2014

atggatgtcaatccgactctacttttcctaaaaattccagcacaaaatgccataagcaccacattcccttatactggagatcctccatacagccatggaacaggaacaggatacaccatggacacagtaaacagaacacaccaatactcagaaaagggaaagtggacaacaaacacagagactggtgcaccccagctcaacccgattgatggaccactacctgaggataatgaaccaagtgggtatgcacaaacagactgtgttctagaggctatggctttccttgaagaatcccacccaggaatatttgagaattcatgccttgaaacaatggaagttgttcaacaaacaagggtagataaactgactcaaggtcgccagacttatgattggacattaaacagaaatcaaccggcagcaactgcattggccaacaccatagaagtctttagatcgaatgacctaacagctaacgagtcaggaaggctaatagatttcttaaaggatgtaatggaatcaatgaacaaagaggaaatagagataacaacccattttcaaagaaaaagaagagtaagagacaacatgaccaagaagatggtcacgcaaagaacaataggtaagaaaaaacagagactgaataagagaggctatctaataagagcactgacattaaatacgatgaccaaagatgcagagagaggcaagttaaaaagaagggctatcgcaacacctgggatgcagattagaggtttcgtatactttgttgaaactttagctaggagcatttgcgaaaagcttgaacagtctgggctcccagtagggggcaatgaaaagaaggccaaattggcaaatgttgtgagaaagatgatgactaattcacaagatacagagatttctttcacaatcactggggacaacactaagtggaatgaaaatcaaaatcctcgaatgttcctggcgatgattacatatatcaccagaaatcaacccgagtggtttagaaacatcctgagcatggcacccataatgttctcaaataaaatggcaagactagggaaagggtacatgttcgagagtaaaagaatgaagattcgaacacaaataccagcagaaatgctagcaaacattgatctgaagtacttcaatgaatcaacaaagaagaaaattgagaaaataaggcctcttttaatggatggcacagcatcactgagtcctgggatgatgatgggcatgttcaacatgctaagtacggtcttgggagtctcgatactgaatcttggacaaaagaaatacaccaagacaacatactggtgggatgggctccaatcatccgacgattttgctctcatagtgaatgcaccaaaccatgaaggaatacaagcaggagtggacagattctacaggacctgcaagttagtgggaatcaacatgagcaaaaagaaatcctatataaataagacagggacatttgaattcacaagctttttttatcgctatggatttgtggctaattttagcatggagctacccagctttggagtgtctggagtaaatgaatcagctgacatgagtattggagtaacagtgataaagaacaacatgataaacaatgaccttggacctgcaacggcccagatggctcttcaattgttcatcaaagactacagatacacatataggtgccataggggagacacacaaattcagacaagaagatcatttgagttaaagaagctatgggatcaaacccaatcaaaggtagggctattagtatcagatggaggaccaaacttatacaacatacggaatcttcacattcctgaagtctgcttaaaatgggagctaatggatgatgattatcggggaaggctttgtaatcccctgaatccctttgtaagtcataaggagattgattctgtaaacaatgctgtggtaatgccagcccatggtccagccaaaagcatggaatatgatgccgttgcaactacacattcctggattcccaagaggaatcgttctattctcaacacaagccaaaggggaattcttgaggatgaacagatgtaccagaagtgttgcaatctattcgagaaatttttccctagcagttcatataggagaccggttggaatttctagcatggtggaggccatggtgtctagggcccggattgatgccagggtcgactttgagtctggacggatcaagaaagaagagttctctgagatcatgaagatctgttccaccattgaagaactcagacggcaaaaataa

>H1N1_PB1_KM366637_A/Helsinki/447M/2014

atggatgtcaatccgactctacttttcctaaaaattccagcacaaaatgccataagcaccacattcccttatactggagatcctccatacagccatggaacaggaacaggatacaccatggacacagtaaacagaacacaccaatactcagaaaagggaaagtggacaacaaacacagagactggtgcaccccagctcaacccgattgatggaccactacctgaggacaatgaaccaagtgggtatgcacaaacagactgtgttctagaggctatggctttccttgaagaatcccacccaggaatatttgagaattcatgccttgaaacaatggaagttgttcaacaaacaagggtagataaactgactcaaggtcgccagacttatgattggacattaaacagaaatcaaccggcagcaactgcattggccaacaccatagaagtctttagatcgaatgacctaacagctaacgagtcaggaaggctaatagatttcttaaaggatgtaatggaatcaatgaacaaagaggaaatagagataacaacccattttcaaagaaaaagaagagtaagagacaacatgaccaagaagatgatcacgcaaagaacaataggtaagaaaaaacagagactgaataagagaggctatctaataagagcactgacattaaatacgatgaccaaagatgcagagagaggcaagttaaaaagaagggctatcgcaacacctgggatgcagattagaggtttcgtatactttgttgaaactttagctaggagcatttgcgaaaagcttgaacagtctgggctcccagtagggggcaatgaaaagaaggccaaattggcaaatgttgtgagaaagatgatgactaattcacaagatacagagatttctttcacaatcactggggacaacactaagtggaatgaaaatcaaaatcctcgaatgttcctggcgatgattacatatatcaccagaaatcaacccgagtggtttagaaacatcctgagcatggcacccataatgttctcaaataaaatggcaagactagggaaagggtacatgttcgagagtaaaagaatgaagattcgaacacaaataccagcagaaatgctagcaaacattgatctgaagtacttcaatgaatcaacaaagaagaaaattgagaaaataaggcctcttttaatggatggcacagcatcactgagtcctgggatgatgatgggcatgttcaacatgctaagtacggtcttgggagtctcgatactgaatcttggacaaaagaaatacaccaagacaacatactggtgggatgggctccaatcatccgacgattttgctctcatagtgaatgcaccaaaccatgaaggaatacaagcaggagtggacagattctacaggacctgcaagttagtgggaatcaacatgagcaaaaagaaatcctatataaataagacagggacatttgaattcacaagctttttttatcgctatggatttgtggctaattttagcatggagctacccagctttggagtgtctggagtaaatgaatcagctgacatgagtattggagtaacagtgataaagaacaacatgataaacaatgaccttggacctgcaacggcccagatggctcttcaattgttcatcaaagactacagatacacatataggtgccataggggagacacacaaattcagacaagaagatcatttgagttaaagaagctatgggatcaaacccaatcaaaggtagggctattagtatcagatggaggaccaaacttatacaacatacggaatcttcacattcctgaagtctgcttaaaatgggagctaatggatgatgattatcggggaaggctttgtaatcccctgaatccctttgtgagtcataaggagattgattctgtaaacaatgctgtggtaatcccagcccatggtccagccaaaagcatggaatatgatgccgttgcaactacacattcctggattcccaagaggaatcgttctattctcaacacaagccaaaggggaattcttgaggatgaacagatgtaccagaagtgttgcaatctattcgagaaatttttccctagcagttcatataggagaccggttggaatttctagcatggtggaggccatggtgtctagggcccggattgatgccagggtcgactttgagtctggacggatcaagaaagaagagttctctgagatcatgaagatctgttccaccattgaagaactcagacggcaaaaataa

>H1N1_PB1_KM366405_A/Helsinki/1198/2013

atggatgtcaatccgactctacttttcctaaaaattccagcacaaaatgctataagcaccacattcccttatactggagatcctccatacagccatggaacaggaacaggatacaccatggacacagtaaacagaacacaccaatactcagaaaagggaaagtggacaacaaacacagagactggtgcaccccagctcaacccgattgatggaccactacctgaggataatgaaccaagtgggtatgcacaaacagactgtgttctagaggctatggctttccttgaagaatcccacccaggaatatttgagaattcatgccttgaaacaatggaagttgttcaacaaacaagggtagataaactgactcaaggtcgccagacttatgattggacattaaacagaaatcaaccggcagcaactgcattggccaacaccatagaagtctttagatcgaatgacctaacagctaacgagtcaggaaggctaatagatttcttaaaggatgtaatggaatcaatgaacaaagaggaaatagagataacaacccactttcaaagaaaaaggagagtaagagacaacatgaccaagaagatggtcacacaaagaacaatagggaagaaaaaacagagactgaataagagaggctatctaataagagcactgacattaaatacgatgaccaaagatgcagagagaggcaagttaaaaagaagggctatcgcaacacctgggatgcagattagaggtttcgtatactttgttgaaactttagctaggagcatttgcgaaaagcttgaacagtctggactcccagtagggggcaatgaaaagaaggccaaactggcaaatgttgtgagaaagatgatgactaattcacaagatacagagatttctttcacaatcactggggacaacactaagtggaatgaaaatcaaaatcctcgaatgttcctggcgatgattacatatatcaccagaaatcaacccgagtggtttagaaacatcctgagcatggcacccataatgttctcaaataaaatggcaagactagggaaagggtacatgttcgagagtaaaagaatgaagatccgaacacaaataccagcagaaatgctagcaagcattgatctgaagtacttcaatgaatcaacaaagaagaaaattgagaaaataaggcctcttttaatggatggcacagcatcactgagtcctgggatgatgatgggcatgttcaacatgctaagtacggtcttgggagtctcgatactgaatcttggacaaaagaaatacaccaagacaacatactggtgggatgggctccaatcatccgacgattttgctctcatagtgaatgcaccaaaccatgagggaatacaagcaggagtggacagattctacaggacctgcaagttagtgggaatcaacatgagcaaaaagaagtcctatataaataagacagggacatttgaattcacaagctttttttatcgctatggatttgtggctaattttagcatggagctacccagctttggagtgtctggagtaaatgaatcagctgacatgagtattggagtaacagtgataaagaacaacatgataaacaatgaccttggacctgcaacggcccagatggctcttcaattgttcatcaaagactacagatacacatataggtgccataggggagacacacaaattcagacgagaagatcatttgagttaaagaagctgtgggatcaaacccaatcaaaggtagggctattagtatcagatggaggaccaaacttatacaatatacggaatcttcacattcctgaagtctgcttaaaatgggagctaatggatgatgattatcggggaagactttgtaatcccctgaatccctttgtaagtcataaggagattgattctgtaaacaatgctgtggtaatgccagcccatggtccagccaaaagcatggaatatgatgccgttgcaactacacattcctggattcccaagaggaaccgttctattctcaacacaagccaaaggggaattcttgaggatgaacagatgtaccagaagtgttgcaatctattcgagaaatttttccctagcagttcatataggagaccggttggaatttctagcatggtggaggccatggtatctagggcccggattgatgccagggtcgactttgagtctgggcggatcaagaaagaagagttctctgagatcatgaagatctgttccaccattgaagaactcagacggcaaaaataa

>H1N1_PB1_KT836761_A/Montana/08/2015

atggatgtcaatccgactctacttttcctaaaaattccagcacaaaatgccataagcaccacattcccttatactggagatcctccatacagccatggaacaggaacaggatacaccatggacacagtaaacagaacacaccaatactcagaaaaggggaagtggacaacaaacacagagactggtgcaccccagctcaacccgattgatggaccactacctgaggataatgaaccaagtgggtatgcacagacagactgtgtcctagaggctatggctttccttgaagaatcccacccaggaatatttgagaattcatgccttgaaacaatggaagttgttcaacaaacaagggtagataaactgactcaaggtcgccagacttatgattggacattaaacagaaatcaaccagcagcaactgcattggccaacaccatagaagtctttagatcgaatgacctaacagctaacgagtcaggaaggctaatagatttcttaaaggatgtaatggaatcaatggacaaagaggaaatagagataacaacccactttcaaagaaaaaggagagtaagagacaacatgaccaagaagatggtcacgcaaagaacaatagggaagaaaaaacagaaactgaataagagaggctatctaataagagcactgacattaaatacgatgaccaaagatgcagagagaggcaagttaaaaagaagggctatcgcaacacctgggatgcagattagaggtttcgtatactttgttgaaactttagctaggagcatttgcgaaaagcttgaacagtctgggctcccagtagggggcaatgaaaagaaggccaaattggcaaatgttgtgagaaagatgatgactaattcacaagatacagagatttctttcacaatcactggggacaacactaaatggaatgaaaatcaaaatcctcgaatgttcctggcaatgattacatatatcaccagaaatcaacccgagtggtttagaaacatcctgagcatggcacccataatgttctcaaataaaatggcaagactggggaaagggtacatgttcgagagtaaaagaatgaaaattcgaacacaaataccagcagaaatgctagcaagcattgatctgaagtacttcaatgaatcaacaaagaagaaaattgagaaaataaggcctcttttaatggatggcacagcatcactgagtcctgggatgatgatgggcatgttcaacatgctaagtacggtcttgggagtctcgatactgaatcttggacaaaagaaatacaccaagacaacatactggtgggatgggctccaatcatccgacgattttgctctcatagtgaatgcaccaaaccacgaaggaatacaagcaggagtggacagattctacaggacctgcaagttagtgggaatcaacatgagcaaaaagaagtcttatataaataagacagggacatttgaattcacaagctttttttatcgctatggatttgtggctaattttagcatggagctacccagctttggagtgtctggagtaaatgaatcagctgacatgagtattggagtaacagtgataaagaacaacatgataaacaatgaccttggacctgcaacggcccagatggctcttcaattgttcatcaaagactacagatacacatataggtgccacaggggagacacacaaattcagacgagaagatcatttgagttaaagaagctatgggatcaaacccaatcaaaggtagggctattagtatcagatggaggaccaaacttatacaacatacggaatcttcacattcctgaagtctgcttaaaatgggagctaatggatgatgattatcggggaagactttgtaatcccctgaatccctttgtgagtcataaggagattgattctgtaaacaatgccgtggtaatgccagcccatggtccagccaaaagcatggaatatgatgccgttgcaactacacattcctggattcccaagaggaatcgttctatcctcaacacaagccaaaggggaattcttgaggatgaacagatgtaccagaagtgttgcaatctattcgagaaatttttccctagcagttcatataggagaccggttggaatttctagcatggtggaggccatggtgtctagggcccggattgatgccagggtcgactttgagtctggacggatcaagaaagaggagttctctgagatcatgaagatctgttccaccattgaagaactcagacggcaaaaataa

>H1N1_PB1_KX005936_A/Nebraska/04/2016

atggatgtcaatccgactctacttttcctaaaaattccagcacaaaatgccataagcaccacattcccttatactggagatcctccatatagccatggaacaggaacaggatacaccatggacacagtaaacagaacacaccaatactcagaaaaggggaagtggacaacaaacacagagactggtgcaccccagctcaacccgattgatggaccactacctgaggataatgaaccaagtgggtatgcacaaacagactgtgttctagaggctatggctttccttgaagaatcccacccaggaatatttgagaattcatgccttgaaacaatggaagttgttcaacaaacaagggtagataaactgactcaaggtcgccagacttatgattggacattaaacagaaatcaaccggcagcaactgcattggccaacaccatagaagtctttagatcgaatgacctaacagctaacgagtcaggaaggctaatagatttcttaaaggatgtaatggaatcaatgaacaaagaggaaatagagataacaacccactttcaaagaaaaaggagagtaagagacaacatgaccaagaagatggtcacgcaaagaacaatagggaagaaaaaacagagactgaataagagaggctatctaataagagcactgacattaaatacgatgaccaaagatgcagagagaggcaagttaaaaagaagggctatcgcaacacctgggatgcagattagaggtttcgtatactttgttgaaactttagctaggagcatttgcgaaaagcttgaacagtctgggctcccagtagggggcaatgaaaagaaggccaaattggcaaatgttgtgagaaagatgatgactaattcacaagatacagagatttctttcacaatcactggggacaacactaagtggaatgaaaatcaaaatcctcgaatgttcctggcgatgattacatatatcaccagaaatcaacccgagtggtttagaaacatcctgagcatggcacccataatgttctcaaataaaatggcaagactggggaaagggtacatgttcgagagtaaaagaatgaagattcgaacacaaataccagcagaaatgctagcaagcattgatctgaagtacttcaatgaatcaacaaagaagaaaattgagaaaataaggcctcttttaatggatggcacagcatcactgagtcctgggatgatgatgggaatgttcaacatgctaagtacggtattgggagtctcgatactgaatcttggacaaaagaaatacaccaagacaacatactggtgggatgggctccaatcatccgacgattttgccctcatagtgaatgcaccaaaccacgaaggaatacaagcaggagtggacaggttctacaggacctgcaagttagtgggaatcaacatgagcaaaaagaagtcctatataaataagacagggacatttgaattcacaagctttttttatcgctatggatttgtggctaattttagcatggagctacccagctttggagtgtctggagtgaatgaatcagctgacatgagtattggagtaacagtgataaagaacaacatgataaacaatgaccttggacctgcgacggcccagatggctcttcaattgttcatcaaagactacagatacacatataggtgccataggggagacacacaaattcagacgagaagatcctttgagttaaagaagctatgggatcaaacccaatcaaaggtagggctattagtatcagatggagggccaaacttatacaacatacggaatcttcacattcctgaagtctgcttaaaatgggagctaatggatgatgattatcggggaagactttgtaatcccctgaatccctttgtgagtcataaggagattgattctgtaaacaatgccgtggtaatgccagcccatggtccagccaaaagcatggaatatgatgccgttgcaactacacattcctggattcccaagaggaatcgttctattctcaacacaagccaaaggggaattcttgaggatgaacagatgtaccagaagtgttgcaatctattcgagaaatttttccctagcagttcatataggagaccggttggaatttctagcatggtggaggccatggtgtctagggcccggattgatgccagagtcgactttgagtctggacggatcaagaaagaagagttctctgagatcatgaagatctgttccaccattgaagaactcagacggcaaaaataa

>H1N1_PB1_KX410313_A/Montana/34/2016

atggatgtcaatccgactctacttttcctaaaaattccagcacaaaatgccataagcaccacattcccttatactggagatcctccatacagccatggaacaggaacaggatacaccatggacacagtaaacagaacacaccaatactcagaaaaggggaagtggacaacaaacacagagactggtgcaccccagctcaacccgattgacggaccactacctgaagataatgaaccaagtgggtatgcacaaacagactgtgttctagaggctatggcttttcttgaagaatcccatccaggaatatttgagaattcatgccttgaaacaatggaagttgttcaacaaacaagggtagataaactgactcaaggtcgccagacttatgattggacattaaacagaaatcaaccggcagcaactgcattggccaacaccatagaagtttttagatcgaatgacctaacagctaacgagtcaggaaggctaatagatttcttaaaggatgtgatggaatcaatgaacaaagaggaaatagagataacaacccactttcaaagaaaaaggagagtaagagacaacatgaccaagaagatggtcacgcaaagaacaatagggaagaaaaaacagagactgaataagagaggctatctaataagagcactgacattaaatacgatgaccaaagatgcagaaagaggcaaattaaaaagaagggctatcgcaacacctgggatgcagattagaggtttcgtatactttgttgaaactttagctaggagcatttgcgaaaagcttgaacagtctgggctcccagtagggggcaatgaaaagaaggccaaattggcaaatgttgtgagaaagatgatgactaattcacaagatacagagatttctttcacaatcactggggacaacactaagtggaatgaaaatcaaaatcctcgaatgttcctggcgatgattacatatatcaccagaaatcaacccgagtggtttagaaacatcctgagcatggcacccataatgttctcaaataaaatggcaagactaggaaaagggtacatgttcgagagtaaaagaatgaagattcgaacacaaataccagcagaaatgctagcaagcattgatctgaagtatttcaatgaatcaacaaagaagaaaattgagaaaataaggcctcttttaatggatggcacagcatcactgagtcctgggatgatgatgggcatgttcaacatgctaagtacggtcttgggagtctcgatactgaatcttggacaaaagaaatacaccaagacaacatactggtgggatgggctccaatcatccgacgattttgctctcatagtgaatgcaccaaaccatgaaggaatacaagcaggagtggacagattctacaggacctgcaagttagtgggaatcaacatgagcaaaaagaagtcctatataaataagacagggacatttgaattcacaagctttttttatcgctatggatttgtggctaattttagcatggagctacccagctttggagtgtctggagtaaatgaatcagctgacatgagtattggagtaacagtgataaagaacaacatgataaacaatgaccttggacctgcaacggctcagatggctcttcaattgttcatcaaagactacagatacacatataggtgccataggggagacacacaaattcagacgagaagatcatttgagttgaagaagctatgggatcaaacccaatcaaaggtagggctattagtatcagatggaggaccaaacttatacaacatacggaatcttcacattcctgaagtctgcttaaaatgggagctaatggatgatgattatcggggaagactttgtaatcccctgaatccctttgtgagtcataaggagattgattctgtaaacaatgctgtggtagtgccagcccatggcccagccaaaagcatggaatatgatgccgttgctactacacattcctggattcccaagaggaatcgttctattctcaacacaagccaaaggggaattcttgaggatgaacagatgtaccagaagtgttgcaatttattcgagaaatttttccctagcagttcatataggagaccggttggaatttctagcatggtggaggccatggtgtctagggcccggattgatgccagagtcgactttgagtctggacggatcaagaaagaagagttctctgagatcatgaagatctgttccaccattgaagaactcagacggcaaaaataa

>H1N1_PB1_KX411889_A/Iowa/17/2016

atggatgtcaatccgactctacttttcctaaaaattccagcacaaaatgccataagcaccacattcccttatactggagatcctccatacagccatggaacaggaacaggatacaccatggacacagtaaacagaacacaccaatactcagaaaaggggaagtggacaacaaacacagagactggtgcaccccagctcaacccgattgacggaccactacctgaagataatgaaccaagtgggtatgcacaaacagactgtgttctagaggctatggcttttcttgaagaatcccatccaggaatatttgagaattcatgccttgaaacaatggaagttgttcaacaaacaagggtagataaactgactcaaggtcgccagacttatgattggacattaaacagaaatcaaccggcagcaactgcattggccaacaccatagaagtttttagatcgaatgacctaacagctaacgagtcaggaaggctaatagatttcttaaaggatgtgatggaatcaatgaacaaagaagaaatagagataacaacccactttcaaagaaaaaggagagtaagagacaacatgaccaagaagatggtcacgcaaagaacaatagggaagaaaaaacagagactgaataagagaggctatctaataagagcactgacattaaatacgatgaccaaagatgcagagagaggcaagttaaaaagaagggctatcgcaacacctgggatgcagattagaggtttcgtatactttgttgaaactttagctaggagcatttgcgaaaagcttgaacagtctgggctcccagtagggggcaatgaaaagaaggccaaattggcaaatgttgtgagaaagatgatgactaattcacaagatacagagatttctttcacaatcactggggacaacactaagtggaatgaaaatcaaaatcctcgaatgttcctggcgatgattacatatatcaccagaaatcaacccgagtggtttagaaacatcctgagcatggcacccataatgttctcaaataaaatggcaagactaggaaaagggtacatgttcgagagtaaaagaatgaagattcgaacacaaataccagcagaaatgctagcaagcattgatctgaagtatttcaatgaatcaacaaagaagaaaattgagaaaataaggcctcttttaatggatggcacagcatcactgagtcctgggatgatgatgggcatgttcaacatgctaagtacggtcttgggagtctcgatactgaatcttggacaaaagaaatacaccaagacaacatactggtgggatgggctccaatcatccgacgattttgctctcatagtgaatgcaccaaaccatgaaggaatacaagcaggagtggacagattctacaggacttgcaagttagtgggaatcaacatgagcaaaaagaagtcctatataaataagacagggacatttgaattcacaagctttttttatcgctatggatttgtggctaattttagcatggagctacctagctttggagtgtctggagtaaatgaatcagctgacatgagtattggagtaacagtgataaagaacaacatgataaacaatgaccttggacctgcaacggctcagatggctcttcaattgttcatcaaagactacagatacacatataggtgccataggggagacacacaaattcagacgagaagatcatttgagttgaagaagctatgggatcaaacccaatcaaaggtagggctattagtatcagatggaggaccaaacttatacaacatacggaatcttcacattcctgaagtctgcttaaaatgggagctaatggatgatgattatcggggaagactttgtaatcccctgaatccctttgtgagtcataaggagattgattctgtaaacaatgctgtggtaatgccagcccatggcccagccaaaagcatggaatatgatgccgttgctactacacattcctggattcccaagaggaatcgctctattctcaacacaagccaaaggggaattcttgaggatgaacagatgtaccagaagtgttgcaatttattcgagaaatttttccctagcagttcatataggagaccggttggaatttctagcatggtggaggccatggtgtctagggcccggattgatgccagagtcgactttgagtctggacggatcaagaaagaagagttctctgagatcatgaagatctgttccaccattgaagaactcagacggcaaaaataa

>H1N1_PB1_KX949238_A/Virginia/59/2016

atggatgtcaatccgactctacttttcctaaaaattccagcacaaaatgccataagcaccacattcccttatactggagatcctccatacagccatggaacaggaacaggatacaccatggacacagtaaacagaacacaccaatactcagaaaaggggaagtggacaacaaacacagagactggtgcaccccagctcaacccgattgacggaccactacctgaagataatgaaccaagtgggtatgcacaaacagactgtgttctagaggctatggcttttcttgaagaatcccatccaggaatatttgagaattcatgccttgaaacaatggaagttgttcaacagacaagggtagataaactgactcaaggtcgccagacttatgattggacattaaacagaaatcaaccggcagcaactgcattggccaacaccatagaagtttttagatcgaatgacctaacagctaacgagtcaggaaggctaatagatttcttaaaggatgtgatggaatcaatgaacaaagaggaaatagagataacaacccactttcaaagaaaaaggagagtaagagacaacatgaccaagaagatggtcacgcaaagaacaatagggaagaaaaaacagagactgaataagagaggctatctaataagagcactgacattaaatacgatgaccaaagatgcagagagaggcaagttaaaaagaagggctatcgcaacacctgggatgcagattagaggtttcgtatactttgttgaaactttagctaggagcatttgcgaaaagcttgaacagtctgggctcccagtagggggcaatgaaaagaaggccaaattggcaaatgttgtgagaaagatgatgactaattcacaagatacagagatttctttcacaatcactggggacaacactaagtggaatgaaaatcaaaatcctcgaatgttcctggcgatgattacatatatcaccagaaatcaacccgagtggtttagaaacatcctgagcatggcacccataatgttctcaaataaaatggcaagactaggaaaagggtacatgttcgagagtaaaagaatgaagattcgaacacaaataccagcagaaatgctagcaagcattgatctgaagtatttcaatgaatcaacaaagaagaaaattgagaaaataaggcctcttttaatggatggcacagcatcactgagtcctgggatgatgatgggcatgttcaacatgctaagtacggtcttgggagtctcgatactgaatcttggacaaaagaaatacaccaagacaacatactggtgggatgggctccaatcatccgacgattttgctctcataatgaatgcaccaaaccatgaaggaatacaagcaggagtggacagattctacaggacctgcaagttagtgggaatcaacatgagcaaaaagaagtcctatataaataagacagggacatttgaattcacaagctttttttatcgctatggatttgtggctaattttagcatggagctacccagctttggagtgtctggagtaaatgaatcagctgacatgagtattggagtaacagtgataaagaacaacatgataaacaatgaccttggacctgcaacggctcagatggctcttcaattgttcatcaaagactacagatacacatataggtgccataggggagacacacaaattcagacgagaagatcatttgagttgaagaagctatgggatcaaacccaatcaaaggtagggctattagtatcagatggaggaccaaacttatacaacatacggaatcttcacattcctgaagtctgcttaaaatgggagctaatggatgatgattatcggggaagactttgtaatcccctgaatccctttgtgagtcataaggagattgattctgtaaacaatgctgtggtaatgccagcccatggcccagccaaaagcatggaatatgatgccgttgctactacacattcctggattcccaagaggaatcgttctattctcaacacaagccaaaggggaattcttgaggatgaacagatgtaccagaagtgttgcaatttattcgagaaatttttccctagcagttcatataggagaccggttggaatttctagcatggtggaggccatggtgtctagggcccggattgatgccagagtcgactttgagtctggacggatcaagaaagaagagttctctgagatcatgaagatctgttccaccattgaagaactcagacggcaaaaataa

>H1N1_PB1_CY009290_A/USSR/92/1977

atggatgtcaatccgaccttacttttcttaaaagtgccagcacaaaatgctataagcacaactttcccttatactggagaccctccttacagccatgggacaggaacagggtacaccatggatacagtcaacaggacacatcagtactcagaaaggggaagatggacaaaaaacaccgaaactggagcaccgcaactcaacccgattgatgggccactaccaaaagacaatgaaccaagtggttatgcccaaacagattgtgtattagaagcaatggctttccttgaggaatcccatcctggtatttttgaaaactcgtgtattgaaacgatggaggttgttcagcaaacacgagtggacaaactgacacaaggcagacagacctatgactggactctaaatagaaaccagcctgctgctacagcattggccaacaccatagaagtgttcagatcaaatggcctcatggcaaatgaatctggaaggctaatagacttccttaaagatgtaatggagtcaatggacagagaagaagtggagatcacaactcattttcagagaaagagaagagtgagggacaatgtgactaagaaaatggtgacacagagaacaataggcaaaaagaaacagagattaaacaaaaggagttatctaattagggcattaaccctgaacacaatgaccaaagatgctgagagagggaaactaaaacgtagagcaattgcaaccccagggatgcaaataagggggtttgtatactttgttgagacactggcaagaagtatatgtgaaaaacttgaacaatcagggttgccagttggaggaaatgaaaagaaagcaaagttggcaaatgttgtaaggaagatgatgaccaattctcaggacactgaaatttctttcaccatcactggagataacaccaaatggaacgaaaatcagaaccctagaatgtttttggccatgatcacatatataaccagaaatcagcccgaatggttcagaaatattctaagtattgctccaataatgttctcaaacaaaatggcgagactaggtaaggggtacatgtttgaaagcaagagtatgaaacttagaactcaaatacctgcagaaatgctagcaaacatcgatttgaagtatttcaatgattcaacaagaaagaaaattgaaaaaatccggccactcttaatagatgggactgcatcactgagccctggaatgatgatgggcatgttcaatatgttaagcactgtattgggcgtctccatcctgaatcttggacaaaagagatacaccaagactacttactggtgggatggtcttcaatcgtctgatgattttgctctgattgtgaatgcacccaaccatgcaggaattcaagctggagttgacaggttttatcgaacctgtaagctgctcggaatcaatatgagcaagaaaaagtcttacataaacagaacaggtacatttgaattcacgagctttttctatcgttatgggtttgttgccaatttcagcatggagcttcccagttttggggtgtctgggatcaacgagtctgcagacatgagtattggagtcactgtcatcaaaaacaatatgataaacaatgatcttggtccagcaaccgctcaaatggcccttcagttattcatcaaagattacaggtacacgtatcgatgccacagaggtgacacacaaatacaaacccgaagatcatttgaaataaagaaactgtgggagcaaacccgttccaaagctgggctgctggtctccgatggaggtcctaatttatacaacattagaaatctccacattcctgaagtctgcttgaaatgggaattgatggatgaggattaccaggggcgtttatgcaacccattgaacccatttgtcagtcataaagagattgaatcagtgaacaatgcagtgatgatgccggcacatggtccagccaaaaatatggagtatgatgctgttgcaacaacacactcctgggtccccaaaaggaatcgatccatcttgaatacgagccaaagaggaatacttgaagatgaacaaatgtatcaaaggtgctgcaatttatttgaaaaattcttccccagcagttcatacagaaggccagtcggaatatccagtatggtagaggctatggtttccagagcccgaattgatgcacggattgatttcgaatctggaaggataaagaaagaggagttcactgagatcatgaagacctgttccaccattgaagaactcagacggcaaaaatag

>H1N1_PB1_EU097786_A/Denmark/16/2004

atggatgtcaatccgacattacttttcttaaaagtgccagcacaaaatgctataagcacaactttcccttatactggtgaccctccttacagccatgggacaggaacagggtacaccatggatacagtcaacaggacacatcagtactcagaaagaggaagatggacaaaaaataccgaaactggagcaccgcaactcaacccgattgatgggccactaccagaagacaatgaaccaagtggctatgcccaaacagattgtgtattagaagcaatggctttccttgaagaatcccatcctggtatttttgaaaactcttgtattgaaacaatggaggttgttcagcaaacaagggtggacaaactgacacaaggcagacaaacctatgactggactttaaataggaaccagcctgctgccacagctttggcaaacactatagaagtattcagatcaaacggcctcatagcaaatgaatctgggaggctaatagacttccttaaagatgtaatggagtcgatggacagagacgaagtagaggtcacaactcattttcaaagaaagaggagagtgagagacaatgtaactaaaaaaatggtgacccaaagaacaataggcaaaaagaaacataaattagacaaaagaagttacctaattagggcattaaccctgaacacaatgaccaaagatgctgagggggggaaactaaaacgcagagcaattgcaaccccaggaatgcaaataagggggttcgtatactttgttgagacactggcaagaagcatatgtgaaaagcttgaacaatcagggttgccagttggaggaaatgagaagaaagcaaagttagcaaatgttgtaagaaagatgatgaccaactcccaggacactgaaatttctttcaccatcactggagataacacaaaatggaacgaaaatcaaaaccctagaatgttcttggccatgatcacatatataaccaaaaatcaacctgaatggttcagaaatattctaagtattgctccaataatgttttcaaacaagatggcgagactaggtaaggggtacatgtttgaaagcaagagtatgaaactgagaactcaagtacctgcagagatgctagccaacatagatttgaaatatttcaatgattcaactaaaaagaaaattgaaaaaatccgaccattattaatagatggaactgcatcattgagtcctggaatgatgatgggcatgttcaatatgttaagcaccgtcttgggcgtctccattctgaatcttgggcaaaagagatacaccaagactacttactggtgggatggtcttcaatcgtctgatgattttgctttgattgtgaatgcacccaactatgcaggaattcaagctggagttgacagattttatcgaacctgtaagctgctaggaattaatatgagcaaaaagaagtcttacataaacagaacaggtacctttgaattcacgagctttttctatcgttatgggtttgttgccaatttcagcatggagcttcctagttttggggtgtctggggtcaatgaatctgcagacatgagtattggagtcactgttatcaaaaacaatatgataaacaatgaccttggcccagcaactgctcaaatggcccttcagttatttataaaagattacaggtacacttatcgatgccacagaggtgacacacaaatacaaacccggagatcatttgagataaagaaactatgggaccaaacccgctccaaagctgggctgttggtctctgatggagggcccaatttatataacattagaaatctccatattcctgaagtctgcttgaagtgggagttgatggatgaggattaccaggggcgtttatgcaacccattgaacccgtttgtcagccataaagagattgaatcagtgaacaatgcagtgataatgccggcacatggtccagccaaaaatatggagtatgacgctgttgcaacaacacactcctgggtccccaaaagaaaccgatccattttgaacacgagccaaagggggatacttgaagatgagcaaatgtatcagaggtgctgcaatttatttgaaaaattcttcccaagtagctcatacagaagaccagttggaatatccagtatggtagaggctatggtttcaagagcccgaattgatgcacggattgatttcgaatctggaaggataaagaaggaggaattcgctgagatcatgaagacctgttccaccattgaagacctcagacggcaaaaatag

>H1N1_PB1_CY027561_A/Texas/UR06-0204/2007

atggatgtcaatccgacattacttttcttaaaagtgccagcacaaaatgctataagcacaacttttccttatactggtgaccctccttacagccatgggacaggaacagggtacaccatggatacagtcaacaggacacaccagtactcagaaagaggaagatggacaaaaaataccgaaactggagcaccgcaactcaacccaattgatgggccactaccagaagacaatgaaccaagtggctatgcccaaacagattgtgtattagaagcaatggctttccttgaagaatcccatcctggtatctttgaaaactcttgtattgaaacaatggaggttgttcagcaaacaagggtggacaaactgacacaaggcagacagacctatgactggactctaaataggaaccagcctgctgcaacagcattggcaaacactatagaagtattcagatcgaacggcctcatagcaaatgaatctggaaggctaatagacttccttaaagatgtaatggagtcgatggacagagacgaagtagaggtcacaactcattttcaaagaaagaggagagtaagagacaatgtaactaaaaaaatggtgactcaaagaacaataggcaaaaagaaacataaattagacaaaagaagttacctaattagggcattaaccctgaacacaatgaccaaagatgctgagagggggaaactaaaacgcagagcaattgcaaccccaggaatgcaaataagagggtttgtatactttgttgagacactggcaagaagcatatgtgaaaagcttgaacaatcaggattgccagttggaggaaatgagaagaaagcaaagttagcaaatgttgtaaggaagatgatgaccaactcccaggacactgaaatttctttcaccatcactggagataacacaaaatggaacgaaaatcaaaaccctagaatgttcttggccatgatcacatatataaccaaaaatcagcctgaatggttcagaaatattctaagtattgctccaataatgttttcaaacaagatggcgagactaggtaaggggtacatgtttgaaagcaagagtatgaaactgagaactcaaatacctgcagagatgctagccaacatagatttgaaatatttcaatgattcaactaaaaagaaaattgaaaaaatccgaccattattaatagatggaactgcatcattgagtcctggaatgatgatgggcatgttcaatatgttgagcaccgtcttgggcgtctccattctgaatcttgggcaaaagagatacaccaagactacttactggtgggatggtcttcaatcgtctgatgattttgctttgattgtgaatgcacccaactatgcaggaattcaagctggagttgacaggttttatcgaacctgtaagctgctcggaattaatatgagcaaaaagaagtcttacataaacagaacaggtacctttgaattcacgagctttttctatcgttatgggtttgttgccaatttcagcatggagcttcctagttttggggtgtctggggtcaatgaatctgcagacatgagtattggagtcactgtcatcaaaaacaatatgataaacaatgaccttggcccagcaactgctcaaatggcccttcagttatttataaaagattacaggtacacttatcgatgccacagaggtgacacacaaatacaaacccggagatcatttgagataaagaaactatgggaccaaacccgctccaaagctgggctgttggtctctgatggaggccccaatttatataacattagaaatctccatattcctgaagtttgcttgaaatgggagttgatggatgaggattaccaggggcgtttatgcaacccattaaacccgtttgtcagccataaagagattgaatcagtgaacaatgcagtgataatgccggcacatggcccagccaaaaatatggagtatgacgctgttgcaacaacacactcctgggtccccaaaagaaatcgatccattttgaacacgagccaaagggggatacttgaagatgaacaaatgtatcagaggtgctgcaatttatttgaaaaattcttcccaagtagctcatacagaagaccagttggaatatccagtatggtagaggctatggtttcaagagcccgaattgatgcacggattgatttcgaatctggaaggataaagagggaggaatttgctgagatcatgaagatctgttccaccattgaagacctcagacggcaaaaatag

>H1N1_PB1_GQ496138_A/Vladivostok/IIV17/2009

atggatgtcaatccgactctacttttcctaaaaattccagcgcaaaatgccataagcaccacattcccttatactggagatcctccatacagccatggaacaggaacaggatacaccatggacacagtaaacagaacacaccaatactcagaaaagggaaagtggacgacaaacacagagactggtgcaccccagctcaacccgattgatggaccactacctgaggataatgaaccaagtgggtatgcacaaacagactgtgttctagaggctatggctttccttgaagaatcccacccgggaatatttgagaattcatgccttgaaacaatggaagttgttcaacaaacaagggtagataaactaactcaaggtcgccagacttatgattggacattaaacagaaatcaaccggcagcaactgcattggccaacaccatagaagtctttagatcgaatggcctgacagctaatgagtcaggaaggctaatagatttcttaaaggatgtaatggaatcaatgaacaaagaggaaatagagataacaacccactttcaaagaaaaaggagagtaagagacaacatgaccaagaagatggtcacgcaaagaacaatagggaagaaaaaacaaagactgaataagagaggctatctaataagagcactgacattaaatacgatgaccaaagatgcagagagaggcaagttaaaaagaagggctatcgcaacacctgggatgcagattagaggtttcgtatactttgttgaaactttagctaggagcatttgcgaaaagcttgaacagtctgggctcccagtagggggcaatgaaaagaaggccaaactggcaaatgttgtgagaaagatgatgactaattcacaagacacagagatttctttcacaatcactggggacaacactaagtggaatgaaaatcaaaatcctcgaatgttcctggcgatgattacatatatcaccagaaatcaacccgagtggttcagaaacatcctgagcatggcacccataatgttctcaaacaaaatggcaagactagggagagggtacatgttcgagagtaaaagaatgaagattcgaacacaaataccagcagaaatgctagcaagcattgacctgaagtacttcaatgaatcaacaaagaagaaaattgagaaaataaggcctcttctaatagatggcacagcatcactgagtcctgggatgatgatgggcatgttcaacatgctaagtacggtcttgggagtctcgatactgaatcttggacaaaagaaatacaccaagacaatatactggtgggatgggctccaatcatccgacgattttgctctcatagtgaatgcaccaaaccatgagggaatacaagcaggagtggacagattctacaggacctgcaagttagtgggaatcaacatgagcaaaaagaagtcctatataaataagacagggacatttgaattcacaagctttttttatcgctatggatttgtggctaattttagcatggagctacccagctttggagtgtctggagtaaatgaatcagctgacatgagtattggagtaacagtgataaagaacaacatgataaacaatgaccttggacctgcaacggcccagatggctcttcaattgttcatcaaagactacagatacacatataggtgccataggggagacgcacaaattcagacgagaagatcatttgagttaaagaagctgtgggatcaaacccaatcaaaggtagggctattagtatcagatggaggaccaaacttatacaatatacggaatcttcacattcctgaagtctgcttaaaatgggagctaatggatgatgattatcggggaagactttgtaatcccctgaatccctttgtcagtcataaagagattgattctgtaaacaatgctgtggtaatgccagcccatggtccagccaaaagcatggaatatgatgccgttgcaactacacattcctggattcccaagaggaatcgttctattctcaacacaagccaaaggggaattcttgaggatgaacagatgtaccagaagtgctgcaatctattcgagaaatttttccctagcagttcatataggagaccggttggaatttctagcatggtggaggccatggtgtctagggcccggattgatgccagggtcgacttcgagtctggacggatcaagaaagaagagttctctgagatcatgaagatctgttccaccattgaagaactcagacggcaaaaataa

>H1N1_PB1_GU136048_A/Niigata/717/2009

atggatgtcaatccgactctacttttcctaaaaattccagcgcaaaatgccataagcaccacattcccttatactggagatcctccatacagccatggaacaggaacaggatacaccatggacacagtaaacagaacacaccaatactcagaaaagggaaagtggacgacaaacacagagactggtgcaccccagctcaacccgattgatggaccactacctgaggataatgaaccaagtgggtatgcacaaacagactgtgttctagaggctatggctttccttgaagaatcccacccaggaatatttgagaattcatgccttgaaacaatggaagttgttcaacaaacaagagtagataaactaactcaaggtcgccagacttatgattggacattaaacagaaatcaaccggcagcaactgcattggccaacaccatagaagtctttagatcgaatggcctaacagctaatgagtcaggaagactaatagatttcttaaaggatgtaatggaatcaatgaacaaagaggaaatagagataacaacccactttcaaagaaaaaggagagtaagagacaacatgaccaagaagatggtcacgcaaagaacaatagggaagaaaaaacaaagactgaataagagaggctatctaataagagcactgacattaaatacgatgaccaaagatgcagagagaggcaagttaaaaagaagggctatcgcaacacctgggatgcagattagaggtttcgtatactttgttgaaactttagctaggagcatttgcgaaaagcttgaacagtctgggctcccagtagggggcaatgaaaagaaggccaaactggcaaatgttgtgagaaagatgatgactaattcacaagacacagagatttctttcacaatcactggggacaacactaagtggaatgaaaatcaaaatcctcgaatgttcctggcgatgattacatatatcaccagaaatcaacccgagtggttcagaaacatcctgagcatggcacccataatgttctcaaacaaaatggcaagactagggaaagggtacatgttcgagagtaaaagaatgaagattcgaacacaaataccagcagaaatgctagcaagcattgacctgaagtacttcaatgaatcaacaaagaaaaaaattgagaaaataaggcctcttctaatagatggcacagcatcactgagtcctgggatgatgatgggcatgttcaacatgctaagtacggtcttgggagtctcgatactgaatcttggacaaaagaaatacaccaagacaatatactggtgggatgggctccaatcatccgacgattttgctctcatagtgaatgcaccaaaccatgagggaatacaagcaggagtggacagattctacaggacctgcaagttagtgggaatcaacatgagcaaaaagaagtcctatataaataagacagggacatttgaattcacaagctttttttatcgctatggatttgtggctaattttagcatggagctacccagctttggagtgtctggagtaaatgaatcagctgacatgagtattggagtaacagtgataaagaacaacatgataaacaatgaccttggacctgcaacggcccagatggctcttcaattgttcatcaaagactacagatacacatataggtgccataggggagacacacaaattcagacgagaagatcatttgagttaaagaagctgtgggatcaaacccaatcaaaggtagggctattagtatcagatggaggaccaaacttatacaatatacggaatcttcacattcctgaagtctgcttaaaatgggagctaatggatgatgattatcggggaagactttgtaatcccctgaatccctttgtcagtcataaagagattgattctgtaaacaatgctgtggtaatgccagcccatggtccagccaaaagcatggaatatgatgccgttgcaactacacattcctggattcccaagaggaatcgttctattctcaacacaagccaaaggggaattcttgaggatgaacagatgtaccagaagtgctgcaatctattcgagaaattttttcctagcagttcatataggagaccggttggaatttctagcatggtggaggccatggtgtctagggcccggattgatgccagggtcgacttcgagtctggacggatcaagaaagaagagttctctgagatcatgaagatctgttccaccattgaagaactcagacggcaaaaataa

>H1N1_PB1_CY049649_A/Singapore/ON2081/2009

atggatgtcaatccgactctacttttcctaaaaattccagcgcaaaatgccataagcactacattccctcannctggagatcctccatacagccatggaacaggaacaggatacaccatggacacagtaaacagaacacaccaatactcagaaaagggaaagtggacgacaaacacagagactggtgcaccccagctcaacccgattgatggaccactacctgaggataatgaaccaagtgggtatgcacaaacagactgtgttctagaggctatggctttccttgaagaatcccacccaggaatatttgagaattcatgccttgaaacaatggaagttgttcaacaaacaagggtagataaactaactcaaggtcgccagacttatgattggacattaaacagaaatcaaccggcagcaactgcattggccaacaccatagaagtctttagatcgaatggcctaacagctaatgagtcaggaaggctaatagatttcttaaaggatgtaatggaatcaatgaacaaagaggaaatagagataacaacccactttcaaagaaaaaggagagtaagagacaacatgaccaagaagatggtcacgcaaagaacaatagggnagaaaaaacaaagactgaataagagaggctatctaataagagcactgacattaaatacgatgaccaaagatgcagagagaggcaagttaaaaagaagggctatcgcaacacctgggatgcagattagaggtttcgtatactttgttgaaactttagctaggagcatttgcgaaaagcttgaacagtctgggtncncagtngggggcaatgaaaagaaggccaaactggcaaatgttgtgagaaagatgatgactaattcacaagacacagagatttctttcacaatcactggggacaacactaagtggaatgaaaatcaaaatcctcgaatgttcctggcgatgattacatatatcaccagaaatcaacccgagtggttcagaaacatcctgagcatggcacccataatgttctcaaacaaaatggcaagactagggaaagggnncnnatttgagagtaaaagnatgaagattcgaacacaaataccagcagaaatgctagcaagcattgacctgaagtacttcaatgaatcaacaaagaagaaaattgagaaaataaggcctcttctaatagatggcacagcatcactgagtcctgggatgatgatgggcatgttcaacatgctaagtacggtcttgggagtctcgatactgaatcttggacaaaagaaatacaccaagacaatatactggtgggatgggctccaatcatccgacgattttgctctcatagtgaatgcnccaaaccntgagggaatacaagcaggagtggacagattctacaggacctgcaagttagtgggaatcaacatgagcaaaaagaagtcctatntaaataagacagggacatttgaattcacaagctttttttatcgctatggatttgtggctaattttagcatggagctacccagctttggagtgtctggagtaaatgaatcagctgacatgagtattggagtaacagtgataaagaacaacatgataaacaatgaccttggacctgcaacggcccagatggctcttcaattgttcatcaaagactacagatacacatataggtgccataggggagacacacaaattcagacgagaagatcatttgagttaaagaagctgtgggatcaaacccaatcaaaggtagggctattagtatcagatggaggaccaaacttatacaatatacggaatcttcacattcctgaagtctgcttaaaatgggagctaatggatgatgattatcggggaagactttgtaatcccctgaatccctttgtcagtcataaagagattgattctgtaaacaatgctgtggtaatgccagcccatggtccagccaaaagcatggaatatgatgccgttgcaactacacattcctggattcccaacangnatcgttctattctcaacacaagccaaaggggaattcttgaggatgaacagatgtaccagaagtgctgcaatctattcgagaaatttttccctagcagttcatataggagaccggttggaatttctagcatggtggaggccatggtgtctagggcccggattgatgccagggtcgacttcgagtctggacggatcaagaaagaagagttctctgagatcatgaagatctgttccaccattgaagaactcagacggcaaaaataa

>H1N1_PB1_CY052517_A/Texas/45103737/2009

atggatgtcaatccgactctacttttcctaaaaattccagcgcaaaatgccataagcaccacattcccttatactggagatcctccatacagccatggaacaggaacaggatacaccatggacacagtaaacagaacacaccaatactcagaaaagggaaagtggacgacaaacacagagactggtgcaccccagctcaacccgattgatggaccactacctgaggataatgaaccaagtgggtatgcacaaacagactgtgttctagaggctatggctttccttgaagaatcccacccaggaatatttgagaattcatgccttgagacaatggaagttgttcaacaaacaagggtagataaactaactcaaggtcgccagacttatgattggacattaaacagaaatcaaccggcagcaactgcattggccaacaccatagaagtctttagatcgaatggcctaacagctaatgagtcaggaaggctaatagatttcttaaaggatgtaatggaatcaatgaacaaagaggaaatagagataacaacccactttcaaagaaaaaggagagtaagagacaacatgaccaagaagatggtcacgcaaagaacaatagggaagaaaaaacaaagactgaataagagaggctatctaataagagcactgacattaaatacgatgaccaaagatgcagagagaggcaagttaaaaagaagggctatcgcaacacctgggatgcagattagaggtttcgtatactttgttgaaactttagctaggagcatttgcgaaaagcttgaacagtctgggctcccagtagggggcaatgaaaagaaggccaaactggcaaatgttgtgagaaagatgatgactaattcacaagacacagagatttctttcacaatcactggggacaacactaagtggaatgaaaatcaaaatcctcgaatgttcctggcgatgattacatatatcaccagaaatcaacccgagtggttcagaaacatcctgagcatggcacccataatgttctcaaacaaaatggcaagactagggaaagggtacatgttcgagagtaaaagaatgaagattcgaacacaaataccagcagaaatgctagcaagcattgacctgaagtacttcaatgaatcaacaaagaagaaaattgagaaaataaggcctcttctaatagatggcacagcatcactgagtcctgggatgatgatgggcatgttcaacatgctaagtacggtcttgggagtctcgatactgaatcttggacaaaagaaatacaccaagacaatatactggtgggatgggctccaatcatccgacgattttgctctcatagtgaatgcaccaaaccatgagggaatacaagcaggagtggacagattctacaggacctgcaagttagtgggaatcaacatgagcaaaaagaagtcctatataaataagacagggacatttgaattcacaagctttttttatcgctatggatttgtggctaattttagcatggagctacccagctttggagtgtctggagtaaatgaatcagctgacatgagtattggaataacagtgataaagaacaacatgataaacaatgaccttggacctgcaacggcccagatggctcttcaattgttcatcaaagactacagatacacatataggtgccataggggagacacacaaattcaaacgagaagatcatttgagttaaaaaggctgtgggatcaaacccaatcaaaggcagggctattagtatcagatggaggaccaaacttatacaatatacggaatcttcacattcctgaagtctgcttaaaatgggagctaatggatgatgattatcggggaagactttgtaatcccctgaatccctttgtcagtcataaagagattgattctgtaaacaatgctgtggtaatgccagcccatggtccagccaaaagcatggaatatgatgccgttgcaactacacattcctggattcccaagaggaatcgttctattctcaacacaagccaaaggggaattcttgaggatgaacagatgtaccagaagtgctgcaatctattcgagaaatttttccctagcagttcatataggagaccggttggaatttctagcatggtggaggccatggtgtctagggcccggattgatgccagggtcgacttcgagtctggacggatcaagaaagaagagttctctgagatcatgaagatctgttccaccattgaagaactcagacggcaaaaataa

>H1N1_PB1_CY058714_A/Texas/46193311/2009

atggatgtcaatccgactctacttttcctaaaaattccagcgcaaaatgccataagcaccacattcccttatactggagatcctccatacagccatggaacaggaacaggatacaccatggacacagtaaacagaacacaccaatactcagaaaagggaaagtggacgacaaacacagagactggtgcaccccagctcaacccgattgatggaccactacctgaggataatgaaccaagtgggtatgcacaaacagactgtgttctagaggctatggctttccttgaagaatcccacccaggaatatttgagaattcatgccttgaaacaatggaagttgttcaacaaacaagggtagataaactaactcaaggtcgccagacttatgattggacattaaacagaaatcaaccggcagcaactgcattggccaacaccatagaagtctttagatcgaatggcctaacagctaatgagtcaggaaggctaatagatttcttaaaggatgtaatggaatcaatgaacaaagaggaaatagagataacaacccactttcaaagaaaaaggagagtaagagacaacatgaccaagaagatggtcacgcaaagaacaatagggaagaaaaaacaaagactgaataagagaggctatctaataagagcactgacattaaatacgatgaccaaagatgcagagagaggcaagttaaaaagaagggctatcgcaacacctgggatgcagattagaggtttcgtatactttgttgaaactttagctaggagcatttgcgaaaagcttgaacagtctgggctcccagtagggggcaatgaaaagaaggccaaactggcaaatgttgtgagaaagatgatgactaattcacaagacacagagatttctttcacaatcactggggacaacactaagtggaatgaaaatcaaaatcctcgaatgttcctggcgatgattacatatatcaccagaaatcaacccgagtggttcagaaacatcctgagcatggcacccataatgttctcaaacaaaatggcaagactagggaaagggtacatgttcgagagtaaaagaatgaagattcgaacacaaataccagcagaaatgctagcaagcattgacctgaagtacttcaatgaatcaacaaagaagaaaattgagaaaataaggcctcttctaatagatggcacagcatcactgagtcctgggatgatgatgggcatgttcaacatgctaagtacggtcttgggagtctcgatactgaatcttggacaaaagaaatacaccaagacaatatactggtgggatgggctccaatcatccgacgattttgctctcatagtgaatgcaccaaaccatgagggaatacaagcaggagtggacagattctacaggacctgcaagttagtgggaatcaacatgagcaaaaagaagtcctatataaataagacagggacatttgaattcacaagctttttttatcgctatggatttgtggctaattttagcatggagctacccagctttggagtgtctggagtaaatgaatcagctgacatgagtattggagtaacagtgataaagaacaacatgataaacaatgaccttggacctgcaacggcccagatggctcttcaactgttcatcaaagactacagatacacatataggtgccataggggagacacacaaattcagacgagaagatcatttgagttaaagaagctgtgggatcaaacccaatcaaaggtagggctattagtatcagatggaggaccaaacttatacaatatacggaatcttcacattcctgaagtctgcttaaaatgggagctaatggatgatgattatcggggaagactttgtaatcccctgaatccctttgtcagtcataaagagattgattctgtaaacaatgctgtggtaatgccagcccatggtccagccaaaagcatggaatatgatgccgttgcaactacacattcctggattcccaagaggaatcgttctattctcaacacaagccaaaggggaattcttgaggatgaacagatgtaccagaagtgctgcaatttattcgagaaatttttccctagcagttcatataggagaccggttggaatttctagcatggtggaggccatggtgtctagggcccggattgatgccagggtcgacttcgagtctggacggatcgggaaggaagagttctctgagatcatgaagatctgttccaccattgaagaactcagacggcaaaaataa

>H1N1_PB1_HM568118_A/England/941/2009

atggatgtcaatccgactctacttttcctaaaaattccagcgcaaaatgccataagcaccacattcccttatactggagatcctccatacagccatggaacaggaacaggatacaccatggacacagtaaacagaacacaccaatactcagaaaagggaaagtggacgacaaacacagagactggtgcaccccagctcaacccgattgatggaccactacctgaggataatgaaccaagtgggtatgcacaaacagactgtgttctagaggctatggctttccttgaagaatcccacccaggaatatttgagaattcatgccttgaaacaatggaagttgttcaacaaacaagggtagataaactaactcaaggtcgccagacttatgattggacattaaacagaaatcaaccggcagcaactgcattggccaacaccatagaagtctttagatcgaatggcctaacagctaatgagtcaggaaggctaatagatttcttaaaggatgtaatggaatcaatgaacaaagaggaaatagagataacaacccactttcaaagaaaaaggagagtaagagacaacatgaccaagaagatggtcacgcaaagaacaatagggaagaaaaaacaaagactgaataagagaggctatctaataagagcactgacattaaatacgatgaccaaagatgcagagagaggcaagttaaaaagaagggctatcgcaacacctgggatgcagattagaggtttcgtatactttgttgaaactttagctaggagcatttgcgaaaagcttgaacagtctgggctcccagtagggggcaatgaaaagaaggccaaactggcaaatgttgtgagaaagatgatgactaattcacaagacacagagatttctttcacaatcactggggacaacactaagtggaatgaaaatcaaaatcctcgaatgttcctggcgatgattacatatatcaccagaaatcaacccgagtggttcagaaacatcctgagcatggcacccataatgttctcaaacaaaatggcaagactagggaaagggtacatgttcgagagtaaaagaatgaagattcgaacacaaataccagcagaaatgctagcaagcattgacctgaagtacttcaatgaatcaacaaagaagaaaattgagaaaataaggcctcttttaatagatggcacagcatcactgagtcctgggatgatgatgggcatgttcaacatgctaagtacggtcttgggagtctcgatactgaatcttggacaaaagaaatacaccaagacaatatactggtgggatgggctccaatcatccgacgattttgctctcatagtgaatgcaccaaaccatgagggaatacaagcaggagtggacagattctacaggacctgcaagttagtgggaatcaacatgagcaaaaagaagtcctatataaataagacagggacatttgaattcacaagctttttttatcgctatggatttgtggctaattttagcatggagctacccagctttggagtgtctggagtaaatgaatcagctgacatgagtattggagtaacagtgataaagaacaacatgataaacaatgaccttggacctgcaacggcccagatggctcttcaattgttcatcaaagactacagatacacatataggtgccataggggagacacacaaattcagacgagaagatcatttgagttaaagaagctgtgggatcaaacccaatcaaaggtagggctattagtatcagatggaggaccaaacttgtacaatatacggaatcttcacattcctgaagtctgcttaaaatgggagctaatggatgatgattatcggggaagactttgtaatcccctgaatccctttgtcagtcataaagagattgattctgtaaacaatgctgtggtaatgccagcccatggtccagccaaaagcatggaatatgatgccgttgcaactacacattcctggattcccaagaggaatcgttctattctcaacacaagccaaaggggaattcttgaggatgaacagatgtaccagaagtgctgcaatctattcgagaaatttttccctagcagttcatataggagaccggttggaatttctagcatggtggaggccatggtgtctagggcccggattgatgccagggtcgactttgagtctggacggatcaagaaagaagagttctctgagatcatgaagatctgttccaccattgaagaactcagacggcaaaaataa

>H1N1_PB1_CY066613_A/Athens/INS273/2009

atggatgtcaatccgactctacttttcctaaaaattccagcgcaaaatgccataagcaccacattcccttatactggagatcctccatacagccatggaacaggaacaggatacaccatggacacagtaaacagaacacaccaatactcagaaaagggaaagtggacgacaaacacagagactggtgcaccccagctcaacccgattgatggaccactacctgaggataatgaaccaagtgggtatgcacaaacagactgtgttctagaggctatggctttccttgaagaatcccacccaggaatatttgagaattcatgccttgaaacaatggaagttgttcaacaaacaagggtagataaactaactcaaggtcgccagacttatgattggacattaaacagaaatcaaccggcagcaactgcattggccaacaccatagaagtctttagatcgaatggcctaacagcgaatgagtcaggaaggctaatagatttcttaaaggatgtaatggaatcaatgaacaaagaggaaatagagataacaacccactttcaaagaaaaaggagagtaagagacaacatgaccaagaagatggtcacgcaaagaacaatagggaagaaaaaacaaagactgaataagagaggctatctaataagagcactgacattaaatacgatgaccaaagatgcagagagaggcaagttaaaaagaagggctatcgcaacacctgggatgcagattagaggtttcgtatactttgttgaaactttagctaggagcatttgcgaaaagcttgaacagtctgggctcccagtagggggcaatgaaaagaaggccaaactggcaaatgttgtgagaaagatgatgactaattcacaagacacagagatttctttcacaatcactggggacaacactaagtggaatgaaaatcaaaatcctcgaatgttcctggcaatgattacatatatcaccagaaatcaacccgagtggttcagaaacatcctgagcatggcacccataatgttctcaaacaaaatggcaagactagggaaagggtacatgttcgagagtaaaagaatgaagattcgaacacaaataccagcagaaatgctagcaagcattgacctgaagtacttcaatgaatcaacaaagaagaaaattgagaaaataaggcctcttctaatagatggcacagcatcactgagtcctgggatgatgatgggcatgttcaacatgctaagtacggtcttgggagtctcgatactgaatcttggacaaaagaaatacaccaagacaatatactggtgggatgggctccaatcatccgacgattttgctctcatagtgaatgcaccaaaccatgagggaatacaagcaggagtggacagattctacaggacctgcaagttagtgggaatcaacatgagcaaaaagaagtcctatataaataagacagggacatttgaattcacaagctttttttatcgctatggatttgtggctaattttagcatggagctacccagctttggagtgtctggagtaaatgaatcagctgacatgagtattggagtaacagtgataaagaacaacatgataaacaatgaccttggacctgcaacggcccagatggctcttcaattgttcatcaaagactacagatacacatataggtgccataggggagacacacaaattcagacgagaagatcatttgagttaaagaagctgtgggatcaaacccaatcaaaggtagggctattagtatcagatggaggaccaaacttatacaatatacggaatcttcacattcctgaagtctgcttaaaatgggagctaatggatgatgattatcggggaagactttgtaatcccctgaatccctttgtcagtcataaagagattgattctgtaaacaatgctgtggtaatgccagcccatggtccagccaaaagcatggaatatgatgccgttgcaactacacattcctggattcccaagaggaatcgttctattctcaacacaagccaaaggggaattcttgaggatgaacagatgtaccagaagtgctgcaatctattcgagaaatttttccctagcagttcatataggagaccggttggaatttctagcatggtggaggccatggtgtctagggcccggattgatgccagggtcgacttcgagtctggacggatcaagaaagaagagttctctgagatcatgaagatctgttccaccattgaagaactcagacggcaaaaataa

>H1N1_PB1_CY066949_A/Aarhus/INS237/2009

atggatgtcaatccgactctacttttcctaaaaattccagcgcaaaatgccataagcaccacattcccttatactggagatcctccatacagccatggaacaggaacaggatacaccatggacacagtaaacagaacacaccaatactcagaaaagggaaagtggacgacaaacacagagactggtgcaccccagctcaacccgattgatggaccactacctgaggataatgaaccaagtgggtatgcacaaacagactgtgttctagaggctatggctttccttgaagaatcccacccaggaatatttgagaattcatgccttgaaacaatggaagttgttcaacaaacaagggtagataaactaactcaaggtcgccagacttatgattggacattaaacagaaatcaaccggcagcaactgcattggccaacaccatagaagtctttagatcgaatggcctaacagctaatgagtcaggaaggctaatagatttcttaaaggatgtaatggaatcaatgaacaaagaggaaatagagataacaacccactttcaaagaaaaaggagagtaagagacaacatgaccaagaagatggtcacgcaaagaacaatagggaagaaaaaacaaagactgaataagagaggctatctaataagagcactgacattaaatacgatgaccaaagatgcagagagaggcaagttaaaaagaagggctatcgcaacacctgggatgcagattagaggtttcgtatactttgttgaaactttagctaggagcatttgcgaaaagcttgaacagtctgggctcccagtagggggcaatgaaaagaaggccaaactggcaaatgttgtgagaaagatgatgactaattcacaagacacagagatttctttcacaatcactggggacaacactaagtggaatgaaaatcaaaatcctcgaatgttcctggcgatgattacatatatcaccagaaatcaacccgagtggttcagaaacatcctgagcatggcacccataatgttctcaaacaaaatggcaagactagggaaagggtacatgttcgagagtaaaagaatgaagattcgaacacaaataccagcagaaatgctatcaagcattgacctgaagtacttcaatgaatcaacaaagaagaaaattgagaaaataaggcctcttctaatagatggcacagcatcactgagtcctgggatgatgatgggcatgttcaacatgctaagtacggtcttgggagtctcgatactgaatcttggacaaaagaaatacaccaagacaatatactggtgggatgggctccaatcatccgacgattttgctctcatagtgaatgcaccaaaccatgagggaatacaagcaggagtggacagattctacaggacctgcaagttagtgggaatcaacatgagcaaaaagaagtcctatataaataagacagggacatttgaattcacaagctttttttatcgctatggatttgtagctaattttagcatggagctacccagctttggagtgtctggagtaaatgaatcagctgacatgagtattggagtaacagtgataaagaacaacatgataaacaatgaccttggacctgcaacggcccagatggctcttcaattgttcatcaaagactacagatacacatataggtgccataggggagacacacaaattcagacgagaagatcatttgagttaaagaagctgtgggatcaaacccaatcaaagggagggctattagtatcagatggaggaccaaacttatacaatatacggaatcttcacattcctgaagtctgcttaaaatgggagctaatggatgatgattatcggggaagactttgtaatcccctgaatccctttgtcagtcataaagagattgattctgtaaacaatgctgtggtaatgccagcccatggtccagtcaaaagcatggaatatgatgccgttgcaactacacattcctggattcccaagaggaatcgttctattctcaacacaagccaaaggggaattcttgaggatgaacagatgtaccagaagtgctgcaatctattcgagaaatttttccctagcagttcatataggagaccggttggaatttctagcatggtggaggccatggtgtctagggcccggattgatgccagggtcgacttcgagtctggacggatcaagaaagaagagttctctgagatcatgaagatctgctccaccattgaagaactcagacggcaaaaataa

>H1N1_PB1_CY071093_A/Bochum/INS376/2009

atggatgtcaatccgactctacttttcctaaaaattccagcgcaaaatgccataagcaccacattcccttatactggagatcctccatacagccatggaacaggaacaggatacaccatggacacagtaaacagaacacaccaatactcagaaaagggaaagtggacgacaaacacagagactggtgcaccccagctcaacccgattgatggaccactacctgaggataatgaaccaagtgggtatgcacaaacagactgtgttctagaggctatggctttccttgaagaatcccacccaggaatatttgagaattcatgccttgaaacaatggaagttgttcaacaaacaagggtagataaactaactcaaggtcgccagacttatgattggacattaaacagaaatcaaccggcagcaactgcattggccaacaccatagaagtctttagatcgaatggcctaacagctaatgagtcaggaaggctaatagatttcttaaaggatgtaatggaatcaatgaacaaagaggaaatagagataacaacccactttcaaagaaaaaggagagtaagagacaacatgaccaagaagatggtcacgcaaagaacaatagggaagaaaaaacaaagactgaataagagaggctatctaataagagcactgacattaaatacgatgaccaaagatgcagagagaggcaagttaaaaagaagggctatcgcaacacctgggatgcagattagaggtttcgtatactttgttgaaactttagctaggagcatttgcgaaaagcttgaacagtctgggctcccagtagggggcaatgaaaagaaggccaaactggcaaatgttgtgagaaagatgatgactaattcacaagacacagagatttctttcacaatcactggggacaacactaagtggaatgaaaatcaaaatcctcgaatgttcctggcgatgattacatatatcaccagaaatcaacccgagtggttcagaaacatcctgagcatggcacccataatgttctcaaacaaaatggcaagactagggaaagggtacatgttcgagagtaaaagaatgaagattcgaacacaaataccagcagaaatgctagcaagcattgacctgaagtatttcaatgaatcaacaaagaagaaaattgagaaaataaggcctcttctaatagatggcacagcatcactgagtcctgggatgatgatgggcatgttcaacatgctaagtacggtattgggagtctcgatactgaatcttggacaaaagaaatacaccaagacaatatactggtgggatgggctccaatcatccgacgattttgctctcatagtgaatgcaccaaaccatgagggaatacaagcaggagtggacagattctacaggacctgcaagttagtgggaatcaacatgagcaaaaagaagtcctatataaataagacagggacatttgaattcacaagctttttttatcgctatggatttgtggctaattttagcatggagctacccagctttggagtgtctggagtaaatgaatcagctgacatgagtattggagtaacagtgataaagaacaacatgataaacaatgaccttggacctgcaacggcccagatggctcttcaattgttcatcaaagactacagatacacatataggtgccataggggagacacacaaattcagacgagaagatcatttgagttaaagaagctgtgggatcaaacccaatcaaaggtagggctattagtatcagatggaggaccaaacttatacaatatacggaatcttcacattcctgaagtctgcttaaaatgggagctaatggatgatgattatcggggaagactttgtaatcccctgaatccctttgtcagtcataaagagattgattctgtaaacaatgctgtggtaatgccagcccatggtccagccaaaagcatggaatatgatgccgttgcaactacacattcctggattcccaagaggaatcgttctattctcaacacaagccaaaggggaattcttgaggatgaacagatgtaccagaagtgctgcaatctattcgagaaatttttccccagcagttcatataggagaccggttggaatttctagcatggtggaggccatggtgtctagggcccggattgatgccagggtcgacttcgagtctggacggatcaagaaagaagagttctctgagatcatgaagatctgttccaccattgaagaactcagacggcaaaaataa

>H1N1_PB1_CY075570_A/Boston/655/2009

atggatgtcaatccgactctacttttcctaaaaattccagcgcaaaatgccataagcaccacattcccttatactggagatcctccatacagccatggaacaggaacaggatacaccatggacacagtaaacagaacacaccaatactcagaaaagggaaagtggacgacaaacacagagactggtgcaccccagctcaacccgattgatggaccactacctgaggataatgaaccaagtgggtatgcacaaacagactgtgttctagaggctatggctttccttgaagaatcccacccaggaatatttgagaattcatgccttgaaacaatggaagttgttcaacaaacaagggtagataaactaactcaaggtcgccagacttatgattggacattaaacagaaatcaaccggcagcaactgcattggccaacaccatagaagtctttagatcgaatggcctaacagctaatgagtcaggaaggctaatagatttcttaaaggatgtaatggaatcaatgaacaaagaggaaatagagataacaacccactttcaaagaaaaaggagagtaagagacaacatgaccaagaagatggtcacgcaaagaacaatagggaagaaaaaacaaagactgaataagagaggctatctaataagagcactgacattaaatacgatgaccaaagatgcagagagaggcaagttaaaaagaagggctatcgcaacacctgggatgcagattagaggtttcgtatactttgttgaaactttagctaggagcatttgcgaaaagcttgaacagtctgggctcccagtagggggcaatgaaaagaaggccaaactggcaaatgttgtgagaaagatgatgactaattcacaagacacagagatttctttcacaatcactggggacaacactaagtggaatgaaaatcaaaatcctcgaatgttcctggcgatgattacatatatcaccagaaatcaacccgagtggttcagaaacatcctgagcatggcacccataatgttctcaaacaaaatggcaagactagggaaagggtacatgttcgagagtaaaagaatgaagattcgaacacaaataccagcagaaatgctagcaagcattgacctgaagtacttcaatgaatcaacaaagaagaaaattgagaaaataaggcctcttctaatagatggcacagcatcactgagtcctgggatgatgatgggcatgttcaacatgctaagtacggtcttgggagtctcgatactgaatcttggacaaaagaaatacaccaagacaatatactggtgggatgggctccaatcatccgacgattttgctctcatagtgaatgcaccaaaccatgagggaatacaagcaggagtggacagattctacaggacctgcaagttagtgggaatcaacatgagcaaaaagaagtcctatataaataagacagggacatttgaattcacaagctttttttatcgctatgggtttgtggctaattttagcatggagctacccagctttggagtgtctggagtaaatgaatcagctgacatgagtattggagtaacagtgataaagaacaacatgataaacaatgaccttggacctgcaacggcccagatggctcttcaattgttcatcaaagactacagatacacatataggtgccataagggagacacacaaattcagacgagaagatcatttgagttaaagaagctgtgggatcaaacccgatcaaaggtagggctattagtatcagatggaggaccaaacttatacaatatacggaatcttcacattcctgaagtctgcttaaaatgggagctaatggatgatgattatcggggaagactttgtaatcccctgaatccctttgtcagtcataaagagattgattctgtaaacaatgctgtggtaatgccagcccatggtccagccaaaagcatggaatatgatgccgttgcaactacacattcctggattcccaagaggaatcgttctattctcaacacaagccaaaggggaattcttgaggatgaacagatgtaccagaagtgctgcaatctattcgagaaatttttccctagcagttcatataggagaccggttggaatttctagcatggtggaggccatggtgtctagggcccggattgatgccagggtcgacttcgagtctggacggatcaagaaagaagagttctctgagatcatgaagatctgttccaccattgaagaactcagacggcaaaaataa

>H1N1_PB1_CY075610_A/Boston/681/2009

atggatgtcaatccgactctacttttcctaaaaattccagcgcaaaatgctataagcaccacattcccttatactggagatcctccatacagccatggaacaggaacaggatacaccatggacacagtaaacagaacacaccaatactcagaaaagggaaagtggacgacaaacacagagactggtgcaccccagctcaacccgattgatggaccactacctgaggataatgaaccaagtgggtatgcacaaacagactgtgttctagaggctatggctttccttgaagaatcccacccaggaatatttgagaattcatgccttgaaacaatggaagttgttcaacaaacaagggtagataaactgactcaaggtcgccagacttatgattggacattaaacagaaatcaaccggcagcaactgcattggccaacaccatagaagtctttagatcgaatggcctaacagctaatgagtcaggaaggctaatagatttcttaaaggatgtaatggaatcaatgaacaaagaggaaatagagataacaacccactttcaaagaaaaaggagagtaagagacaacatgaccaagaagatggtcacgcaaagaacaatagggaagaaaaaacaaagactgaataaaagaggctatctaataagagcactgacattaaatacgatgaccaaagatgcagagagaggcaagttaaaaagaagggctatcgcaacacctgggatgcagattagaggtttcgtatactttgttgaaactttagctaggagcatttgcgaaaagcttgaacagtctgggctcccagtagggggcaatgaaaagaaggccaaattggcaaatgttgtgagaaagatgatgactaattcacaagacacagagatttctttcacaatcactggggacaacactaagtggaatgaaaatcaaaatcctcgaatgttcctggcgatgattacatatatcaccagaaatcaacccgagtggttcagaaacatcctgagcatggcacccataatgttctcaaacaaaatggcaagactagggaaagggtacatgttcgagagtaaaagaatgaagattcgaacacaaataccagcagaaatgctagcaagcattgacctgaagtacttcaatgaatcaacaaagaagaaaattgagaaaataaggcctcttctaatagatggcacagcatcactgagtcctgggatgatgatgggcatgttcaacatgctaagtacggtcttgggagtctcgatactgaatcttggacaaaagaaatacaccaagacaatatactggtgggatgggctccaatcatccgacgattttgctctcatagtgaatgcaccaaaccatgagggaatacaagcaggagtggacagattctacaggacctgcaagttagtgggaatcaacatgagcaaaaagaagtcctatataaataagacagggacatttgaattcacaagctttttttatcgctatggatttgtggctaattttagcatggagctacccagctttggagtgtctggagtaaatgaatcagctgacatgagtattggagtaacagtgataaagaacaacatgataaacaatgaccttggacctgcaacggcccagatggctcttcaattgttcatcaaagactacagatacacatataggtgccataggggagacacacaaattcagacgagaagatcatttgagttaaagaagctgtgggatcaaacccaatcaaaggtagggctattagtatcagatggaggaccaaacttatacaatatacggaatcttcacattcctgaagtctgcttaaaatgggagctaatggatgatgattatcggggaagactttgtaatcccctgaatccctttgtcagtcataaagagattgattctgtaaacaatgctgtggtaatgccagcccatggtccagccaaaagcatggaatatgatgccgttgcaactacacattcctggattcccaagaggaatcgttctattctcaacacaagccaaaggggaattcttgaggatgaacagatgtaccagaagtgctgcaatctattcgagaaatttttccctagcagttcatataggagaccggttggaatttctagcatggtggaggccatggtgtctagggcccggattgatgccagggtcgacttcgagtctggacggatcaagaaagaagagttctctgagatcatgaagatctgttccaccattgaagaactcagacggcaaaaataa

>H1N1_PB1_CY092950_A/Chile/6/2010

atggatgtcaatccgactctacttttcctaaaaattccagcgcaaaatgccataagcaccacattcccttatactggagatcctccatacagccatggaacaggaacaggatacaccatggacacagtaaacagaacacaccaatactcagaaaagggaaagtggacgacaaacacagagactggtgcaccccagctcaacccgattgatggaccactacctgaggataatgaaccaagtgggtatgcacaaacagactgtgttctagaggctatggctttccttgaagaatcccacccaggaatatttgagaattcatgccttgaaacaatggaagttgttcaacaaacaagggtagataaactaactcaaggtcgccagacttatgattggacattaaacagaaatcagccggcagcaactgcattggccaacaccatagaagtctttagatcgaatggcctaacagctaatgagtcaggaaggctaatagatttcttaaaggatgtaatggaatcaatgaacaaagaggaaatagagataacaacccactttcaaagaaaaaggagagtaagagacaacatgaccaagaagatggtcacgcaaagaacaatagggaagaaaaaacaaagactgaataagagaggctatctaataagagcactgacattaaatacgatgaccaaagatgcagagagaggcaagttaaaaagaagggctatcgcaacacctgggatgcagattagaggtttcgtatactttgttgaaactttagctaggagcatttgcgaaaagcttgaacagtctgggctcccagtagggggcaatgaaaagaaggccaaactggcaaatgttgtgagaaagatgatgactaattcacaagacacagagatttctttcacaatcactggggacaacactaagtggaatgaaaatcaaaatcctcgaatgttcctggcgatgattacatatatcaccagaaatcaacccgagtggttcagaaacatcctgagcatggcacccataatgttctcaaacaaaatggcaagactagggaaagggtacatgttcgagagtaaaagaatgaagattcgaacacaaataccagcagaaatgctagcaagcattgacctgaagtacttcaatgaatcaacaaagaagaaaattgagaaaataaggcctcttctaatagatggcacagcatcactgagtcctgggatgatgatgggcatgttcaacatgctaagtacggtcttgggagtctcgatactgaatcttggacaaaagaagtacaccaagacaatatactggtgggatgggctccaatcatccgacgattttgctctcatagtgaatgcaccaaaccatgagggaatacaagcaggagtggacagattctacaggacctgcaagttagtgggaatcaacatgagcaaaaagaagtcctatataaataagacagggacatttgaattcacaagctttttttatcgctatggatttgtggctaattttagcatggagctacccagctttggagtgtctggagtaaatgaatcagctgacatgagtattggagtaacagtgataaagaacaacatgataaacaatgaccttggacctgcaacggcccagatggctcttcaactgttcatcaaagactacagatacacatataggtgccataggggagacacacaaattcagacgagaagatcatttgagttaaagaagctgtgggatcaaaccaaatcaaaggtagggctattagtatcagatggaggaccaaacttatacaatatacggaatcttcacattcctgaagtctgcttaaaatgggagctaatggatgatgattatcggggaagactttgtaatcccctgaatccctttgtcagtcataaagagattgattctgtaaacaatgctgtggtaatgccagcccatggtccagccaaaagcatggaatatgatgccgttgcaactacacattcctggattcccaagaggaatcgttctattctcaacacaagccaaaggggaattcttgaggatgaacagatgtaccagaagtgctgcaatctattcgagaaatttttccctagcagttcatataggagaccggttggaatttctagcatggtggaggccatggtgtctagggcccggattgatgccagggtcgacttcgagtctggacggatcgggaaggaagagttctctgagatcatgaagatctgttccaccattgaagagctcagacggcaaaaataa

>H1N1_PB1_CY096264_A/North Fitzroy/INS473/2010

atggatgtcaatccgactctacttttcctaaaaattccagcgcaaaatgccataagcaccacattcccttatactggagatcctccatacagccatggaacaggaacaggatacaccatggacacagtaaacagaacacaccaatactcagaaaagggaaagtggacgacaaacacagagactggtgcaccccagctcaacccgattgatggaccactacctgaggataatgaaccaagtgggtatgcacaaacagactgtgttctagaggctatggctttccttgaagaatcccacccaggaatatttgagaattcatgccttgaaacaatggaagttgttcaacaaacaagggtagataaactaactcaaggtcgccagacttatgattggacattaaacagaaatcaaccggcagcaactgcattggccaacaccatagaagtctttagatcgaatggcctaacagctaatgagtcaggaaggctaatagatttcttaaaggatgtaatggaatcaatgaacaaagaggaaatagagataacaacccactttcaaagaaaaaggagagtaagagacaacatgaccaagaagatggtcacgcaaagaacaatagggaagaaaaaacaaagactgaataagagaggctatctaataagagcactgacattaaatacgatgaccaaagatgcagagagaggcaagttaaaaagaagggctatcgcaacacctgggatgcagattagaggtttcgtatactttgttgaagctttagctaggagcatttgcgaaaagcttgaacagtctgggctcccagtagggggcaatgaaaagaaggccaaactggcaaatgttgtgagaaagatgatgactaattcacaagacacagagatttctttcacaatcactggggacaacactaagtggaatgaaaatcaaaatcctcgaatgttcctggcaatgattacatatatcaccagaaatcaacccgagtggttcagaaacatcctgagcatggcacccataatgttctcaaacaaaatggcaagactagggaaagggtacatgttcgagagtaaaagaatgaagattcgaacacaaataccagcagaaatgctagcaagcattgacctgaagtacttcaatgaatcaacgaagaagaaaattgagaaaataaggcctcttctaatagatggcacagcatcactgagtcctgggatgatgatgggcatgttcaacatgctaagtacggtcttgggagtctcgatactgaatcttggacaaaagaaatacaccaagacaatatactggtgggatgggctccaatcatccgacgattttgctctcatagtgaatgcaccaaaccatgagggaatacaagcaggagtggacagattctacaggacctgcaagttagtgggaatcaacatgagcaaaaagaagtcctatataaataagacagggacatttgaattcacaagctttttttatcgctatggatttgtggctaattttagcatggagctacccagctttggagtgtctggagtaaatgaatcagctgacatgagtattggagtaacagtgataaagaacaacatgataaacaatgaccttggacctgcaacggcccagatggctcttcaattgttcatcaaagactacagatacacatataggtgccataggggagacacacaaattcagacgagaagatcatttgagttaaagaagctgtgggaccaaacccaatcaaaggtagggctattagtatcagatggaggaccaaacttatacaatatacggaatcttcacattcctgaagtctgcttaaaatgggagctaatggatgatgattatcggggaagactttgtaatcccctgaatccctttgtcagtcataaagagattgattctgtaaacaatgctgtggtaatgccagcccatggtccagtcaaaagcatggaatatgatgccgttgcaactacacattcctggattcccaagaggaatcgttctattctcaacacaagccaaaggggaattcttgaggatgaacagatgtaccagaagtgctgcaatctattcgagaaatttttccctagcagttcatataggagaccggttggaatttctagcatggtggaggccatggtgtctagggcccggattgatgccagggtcgacttcgagtctggacggatcaagaaagaagagttctctgagatcatgaagatctgttccaccattgaagaactcagacggcaaaaataa

>H1N1_PB1_CY106574_A/Merida/2189-CIR/2009

atggatgtcaatccgactctacttttcctaaaaattccagcgcaaaatgccataagcaccacattcccttatactggagatcctccatacagccatggaacaggaacaggatacaccatggacacagtaaacagaacacaccaatactcagaaaagggaaagtggacgacaaacacagagactggtgcaccccagctcaacccgattgatggaccactacctgaggataatgaaccaagtgggtatgcacaaacagactgtgttctagaggctatggctttccttgaagaatcccacccaggaatatttgagaattcatgccttgaaacaatggaagttgttcaacaaacaagggtagataaactaactcaaggtcgccagacttatgattggacattaaacagaaatcaaccggcagcaactgcattggccaacaccatagaagtctttagatcgaatggcctaacagctaatgagtcaggaaggctaatagatttcttaaaggatgtaatggaatcaatgaacaaagaggaaatagagataacaacccactttcaaagaaaaaggagagtaagagacaacatgaccaagaagatggtcacgcaaagaacaatagggaagaaaaaacaaagactgaataagagaggctatctaataagagcactgacattaaatacgatgaccaaagatgcagagagaggcaagttaaaaagaagggctatcgcaacacctgggatgcagattagaggtttcgtatactttgttgaaactttagctaggagcatttgcgaaaagcttgaacagtctgggctcccagtagggggcaatgaaaagaaggccaaactggcaaatgttgtgagaaagatgatgactaattcacaagacacagagatttctttcacaatcactggggacaacactaagtggaatgaaaatcaaaatcctcgaatgttcctggcgatgattacatatatcaccagaaatcaacccgagtggttcagaaacatcctgagcatggcacccataatgttctcaaacaaaatggcaagactagggaaagggtacatgttcgagagtaaaagaatgaagattcgaacacaaataccagcagaaatgctagcaagcattgacctgaagtacttcaatgaatcaacaaagaagaaaattgagaaaataaggcctcttctaatagatggcacagcatcactgagtcctgggatgatgatgggcatgttcaacatgctaagtacggtcttgggagtctcgatactgaatcttggacaaaagaaatacaccaagacaatatactggtgggatgggctccaatcatccgacgattttgctctcatagtgaatgcaccaaaccatgagggaatacaagcaggagtggacagattctacaggacctgcaagttagtgggaatcaacatgagcaaaaagaagtcctatataaataagacagggacatttgaattcacaagctttttttatcgctatggatttgtggctaattttagcatggagctacccagctttggagtgtctggagtaaatgaatcagctgacatgagtattggagtaacagtgataaagaacaacatgataaacaatgaccttggacctgcaacggcccagatggctcttcaattgttcatcaaagactacagatacacatataggtgccataggggagacacacaaattcagacgagaagatcatttgagttgaagaagctgtgggatcaaacccaatcaaaggtagggctattagtatcagatggaggaccaaacttatacaatatacggaatcttcacattcctgaagtctgcttaaaatgggagctaatggatgatgattatcggggaagactttgtaatcccctgaatccctttgtcagtcataaagagattgattctgtaaacaatgctgtggtaatgccagcccatggtccagccaaaagcatggaatatgatgccgttgcaactacacattcctggattcccaagaggaatcgttctattctcaacacaagccaaaggggaattcttgaggatgaacagatgtaccagaagtgctgcaatctattcgagaaatttttccctagcagttcatataggagaccggttggaatttctagcatggtggaggccatggtgtctagggcccggattgatgccagggtcgacttcgagtctggacggatcaagaaagaagagttctctgagatcatgaagatctgttccaccattgaagaactcagacggcaaaaataa

>H1N1_PB1_CY123554_A/Singapore/ON1852/2009

atggatgtcaatccgactctacttttcctaaaaattccagcgcaaaatgccataagcaccacattcccttatactggagatcctccatacagccatggaacaggaacaggatacaccatggacacagtaaacagaacacaccaatactcagaaaagggaaagtggacgacaaacacagagactggtgcaccccagctcaacccgattgatggaccactacctgaggataatgaaccaagtgggtatgcacaaacagactgtgttctagaggctatggctttccttgaagaatcccacccaggaatatttgagaattcatgccttgaaacaatggaagttgttcaacaaacaagggtagataaactaactcaaggtcgccagacttatgattggacattaaacagaaatcaaccggcagcaactgcattggccaacaccatagaagtctttagatcgaatggcctaacagctaatgagtcaggaaggctaatagatttcttaaaggatgtaatggaatcaatgaacaaagaggaaatagagataacaacccactttcaaagaaaaaggagagtaagagacaacatgaccaagaagatggtcacgcaaagaacaatagggaagaaaaaacaaagactgaataagagaggctatctaataagagcactgacattaaatacgatgaccaaagatgcagagagaggcaagttaaaaagaagggctatcgcaacacctgggatgcagattagaggtttcgtatactttgttgaaactttagctaggagcatttgcgaaaagcttgaacagtctgggctcccagtagggggcaatgaaaagaaggccaaactggcaaatgttgtgagaaagatgatgactaattcacaagacacagagatttctttcacaatcactggggacaacactaagtggaatgaaaatcaaaatcctcgaatgttcctggcgatgattacatatatcaccagaaatcaacccgagtggttcagaaacatcctgagcatggcacccataatgttctcaaacaaaatggcaagactagggaaagggtacatgtttgagagtaaaagaatgaagattcgaacacaaataccagcagaaatgctagcaagcattgacctgaagtacttcaatgaatcaacaaagaagaaaattgagaaaataaggcctcttctaatagatggcacagcatcactgagtcctgggatgatgatgggcatgttcaacatgctaagtacggtcttgggagtctcgatactgaatcttggacaaaagaaatacaccaagacaatatactggtgggatgggctccaatcatccgacgattttgctctcatagtgaatgcaccaaaccatgagggaatacaagcaggagtggacagattctacaggacctgcaagttagtgggaatcaacatgagcaaaaagaagtcctatataaataagacaggtacatttgaattcacaagctttttttatcgctatggatttgtggctaattttagcatggagctacccagctttggagtgtctggagtaaatgaatcagctgacatgagtattggagtaacagtgataaagaacaacatgataaacaatgaccttggacctgcaacggcccagatggctcttcaattgttcatcaaagactacagatacacatataggtgccataggggagacacacaaattcagacgagaagatcatttgagttaaagaagctgtgggatcaaacccaatcaaaggtagggctattagtatcagatggaggaccaaacttatacaatatacggaatcttcacattcctgaagtctgcttaaagtgggagctaatggatgatgattatcggggaagactttgtaatcccctgaatccctttgtcagtcataaagagattgattctgtaaacaatgctgtggtaatgccagcccatggtccagccaaaagcatggaatatgatgccgttgcaactacacattcctggattcccaagaggaaccgttctattctcaacacaagccaaaggggaattcttgaggatgaacagatgtaccagaagtgctgcaatctattcgagaaatttttccctagcagttcatataggagaccggttggaatttctagcatggtggaggccatggtgtctagggcccggattgatgccagggtcgacttcgagtctggacggatcaaaaaagaagagttctctgagatcatgaagatctgttccaccattgaagaactcagacggcaaaaataa

>H1N1_PB1_JX262206_A/India/GWL02/2011

atggatgtcaatccgactctacttttcctaaaaattccagcgcaaaatgccataagcaccacattcccttatactggagatcctccatacagccatggaacaggaacaggatacaccatggacacagtaaacagaacacaccaatactcagaaaagggaaagtggacgacaaacacagagactggtgcaccccagctcaacccgattgatggaccactacctgaggataatgaaccaagtgggtatgcacaaacagactgtgttctagaggctatggctttccttgaagaatcccacccaggaatatttgagaattcatgccttgaaacaatggaagttgttcaacaaacaagggtagataaactaactcaaggtcgccagacttatgattggacattaaacagaaatcaaccggcagcaactgcattggccaacaccatagaagtctttagatcgaatggcctaacagctaatgagtcaggaaggctaatagatttcttaaaggatgtaatggaatcaatgaacaaagaggaaatagagataacaacccactttcaaagaaaaaggagagtaagagacaacatgaccaagaagatggtcacgcaaagaacaatagggaagaaaaaacaaagactgaataagagaggctatctaatacgagcactgacattaaatacgatgaccaaagatgcagagagaggcaagttaaaaagaagggctatcgcaacacctgggatgcagattagaggtttcgtatactttgttgaaactttagctaggagcatttgcgaaaagcttgaacagtctgggctcccagtagggggcaatgaaaagaaggccaaactggcaaatgttgtgagaaagatgatgactaattcacaagacacagagatttctttcacaatcactggggacaacactaagtggaatgaaaatcaaaatcctcgaatgttcctggcgatgattacatatatcaccagaaatcaacccgagtggttcagaaacatcctgagcatggcacccataatgttctcaaacaaaatggcaagactagggaaagggtacatgttcgagagtaaaagaatgaagattcgaacacaaataccagcagaaatgctagcaagcattgacctgaagtacttcaatgaatcaacaaagaagaaaattgagaaaataaggcctcttctaatagatggcacagcatcactgagtcctgggatgatgatgggcatgttcaacatgctaagtacggtcttgggagtctcgatactgaatcttggacaaaagaaatacaccaagacaacatactggtgggatgggctccaatcatccgacgattttgctctcatagtgaatgcaccaaaccatgagggaatacaagcaggagtggacagattctacaggacctgcaagttagtgggaatcaacatgagcaaaaagaagtcctatataaataagacagggacatttgaattcacaagctttttttatcgctatggatttgtggctaattttagcatggagctacccagctttggagtgtctggagtaaatgaatcagctgacatgagtattggagtaacagtgataaagaacaacatgataaacaatgaccttggacctgcaacggcccagatggctcttcaattgttcatcaaagactacagatacacatataggtgccataggggagacacacaaattcagacgagaagatcatttgagttaaagaagctgtgggatcaaacccaatcaaaggtagggctattagtatcagatggaggaccaaacttatacaatatacggaatcttcacattcctgaagtctgcttaaaatgggagctaatggatgatgattatcggggaagactttgtaatcccctgaatccctttgtcagtcataaagagattgattctgtaaacaatgctgtggtaatgccagcccatggtccagccaaaagcatggaatatgatgccgttgcaactacacattcctggattcccaagaggaatcgttctattctcaacacaagccaaaggggaattcttgaggatgaacagatgtaccagaagtgctgcaatctattcgagaaatttttccctagcagttcatataggagaccggttggaatttctagcatggtggaggccatggtgtctagggcccggattgatgccagggtcgacttcgagtctggacggatcaaaaaagaagagttctctgagatcatgaagatctgttccaccattgaagaactcagacggcaaaaataa

>H1N1_PB1_JX308999_A/Singapore/GP220/2010

atggatgtcaatccgactctacttttcctaaaaattccagcgcaaaatgccataagcaccacattcccttatactggagatcctccatacagccatggaacaggaacaggatacaccatggacacagtaaacagaacacaccaatactcagaaaagggaaagtggacgacaaacacagagactggtgcaccccagctcaacccgattgatggaccactacctgaggataatgaaccaagtgggtatgcacaaacagactgtgttctagaggctatggctttccttgaagaatcccacccaggaatatttgagaattcatgccttgaaacaatggaagttgttcaacaaacaagggtagataaactaactcaaggtcgccagacttatgattggacattaaacagaaatcaaccggcagcaactgcattggccaacaccatagaagtctttagatcgaatggcctaacagctaatgagtcaggaaggctaatagatttcttaaaggatgtaatggaatcaatgaacaaagaggaaatagagataacaacccactttcaaagaaaaaggagagtaagagacaacatgaccaagaagatggtcacgcaaagaacaatagggaagaaaaaacaaagactgaataagagaggctatctaataagagcactgacattaaatacgatgaccaaagatgcagagagaggcaagttaaaaagaagggctatcgcaacacctgggatgcagattagaggtttcgtatactttgttgaagctttagctaggagcatttgcgaaaagcttgaacagtctgggctcccagtagggggcaatgaaaagaaggccaaactggcaaatgttgtgagaaagatgatgactaattcacaagacacagagatttctttcacaatcactggggacaacactaagtggaatgaaaatcaaaatcctcgaatgttcctggcaatgattacatatatcaccagaaatcaacccgagtggttcagaaacatcctgagcatggcacccataatgttctcaaacaaaatggcaagactagggaaagggtacatgttcgagagtaaaagaatgaagattcgaacacaaataccagcagaaatgctagcaagcattgacctgaagtacttcaatgaatcaacaaagaagaaaattgagaaaataaggcctcttctaatagatggcacagcatcactgagtcctgggatgatgatgggcatgttcaacatgctaagtacggtcttgggagtctcgatactgaatcttggacaaaagaaatacaccaagacaatatactggtgggatgggctccaatcatccgacgattttgctctcatagtgaatgcaccaaaccatgagggaatacaagcaggagtggacagattctacaggacctgcaagttagtgggaatcaacatgagcaaaaagaagtcctatataaataagacagggacatttgaattcacaagctttttttatcgctatggatttgtggctaattttagcatggagctacccagctttggagtgtctggagtaaatgaatcagctgacatgagtattggagtaacagtgataaagaacaacatgataaacaatgaccttggacctgcaacggcccagatggctcttcaattgttcatcaaagactacagatacacatataggtgccataggggagacacacaaattcagacgagaagatcatttgagttaaagaagctgtgggatcaaacccaatcaaaggtagggctattagtatcagatggaggaccaaacttatacaatatacggaatcttcacattcctgaagtctgcttaaaatgggagctaatggatgatgattatcggggaagactttgtaatcccctgaatccctttgtcggtcataaagagattgattctgtaaacaatgctgtggtaatgccagcccatggtccagccaaaagcatggaatatgatgccgttgcaactacacattcctggattcccaagaggaatcgttctattctcaacacaagccaaaggggaattcttgaggatgaacagatgtaccagaagtgctgcaatctattcgagaaatttttccctagcagttcatataggagaccggttggaatttctagcatggtggaggccatggtgtctagggcccagattgatgccagggtcgacttcgagtctggacggatcaagaaagaagagttctctgagatcatgaagatctgttccaccattgaagaactcagacggcaaaaataa

>H1N1_PB1_JX309148_A/Singapore/GP737/2010

atggatgtcaatccgactctacttttcctaaaaattccagcgcaaaatgccataagcaccacattcccttatactggagatcctccatacagccatggaacaggaacaggatacaccatggacacagtaaacagaacacaccaatactcagaaaagggaaagtggacgacaaacacagagactggtgcaccccagctcaacccgattgatggaccactacctgaggataatgaaccaagtgggtatgcacaaacagactgtgttctagaggctatggctttccttgaagaatcccacccaggaatatttgagaattcatgccttgaaacaatggaagttgttcaacaaacaagggtagataaactaactcaaggtcgccagacttatgattggacattaaacagaaatcaaccggcagcaactgcattggccaacaccatagaagtctttagatcgaatggcctaacagctaatgagtcaggaaggctaatagatttcttaaaggatgtaatggaatcaatgaacaaagaggaaatagagataacaacccactttcaaagaaaaaggagagtaagagacaacatgaccaagaagatggtcacgcaaagaacaatagggaagaaaaaacaaagactgaataagagaggctatctaataagagcactgacattaaatacgatgaccaaagatgcagagagaggcaagttaaaaagaagggctatcgcaacacctgggatgcagattagaggtttcgtatactttgttgaagctttagctaggagcatttgcgaaaagcttgaacagtctgggctcccagtagggggcaatgaaaagaaggccaaactggcaaatgttgtgagaaagatgatgactaattcacaagacacagagatttctttcacaatcactggggacaacactaagtggaatgaaaatcaaaatcctcgaatgttcctggcaatgattacatatatcaccagaaatcaacccgagtggttcagaaacatcctgagcatggcacccataatgttctcaaacaaaatggcaagactagggaaagggtacatgttcgagagtaaaagaatgaagattcgaacacaaataccagcagaaatgctagcaagcattgacctgaagtacttcaatgaatcaacaaagaagaaaattgagaaaataaggcctcttctaatagatggcacagcatcactgagtcctgggatgatgatgggcatgttcaacatgctaagtacggtcttgggagtctcgatactgaatcttggacaaaagaaatacaccaagacaatatactggtgggatgggctccaatcatccgacgattttgctctcatagtgaatgcaccaaaccatgagggaatacaagcaggagtggacagattctacaggacctgcaagttagtgggaatcaacatgagcaaaaagaagtcctatataaataagacagggacatttgaattcacaagctttttttatcgctatggatttgtggctaattttagcatggagctacccagctttggagtgtctggagtaaatgaatcagctgacatgagtattggagtaacagtgataaagaacaacatgataaacaatgaccttggacctgcaacggcccagatggctcttcaattgttcatcaaagactacagatacacatataggtgccataggggagacacacaaattcagacgagaagatcatttgagttaaagaagctgtgggatcaaacccaatcaaaggtagggctattagtatcagatggaggaccaaacttatacaatatacggaatcttcacattcctgaagtctgcttaaaatgggagctaatggatgatgattatcggggaagactttgtaatcccctgaatccctttgtcggtcataaagagattgattctgtaaacaatgctgtggtaatgccagcccatggtccagccaaaagcatggaatatgatgccgttgcaactacacattcctggattcccaagaggaatcgttctattctcaacacaagccaaaggggaattcttgaggatgaacagatgtaccagaagtgctgcaatctattcgagaaatttttccctagcagttcatataggagaccggttggaatttctagcatggtggaggccatggtgtctagggcccagattgatgccagggtcgatttcgagtctggacggatcaagaaagaagagttctctgagatcatgaagatctgttccaccattgaagaactcagacggcaaaaataa

>H1N1_PB1_KC781961_A/Mississippi/05/2009

atggatgtcaatccgactctacttttcctaaaaattccagcgcaaaatgccataagcaccacattcccttatactggagatcctccatacagccatggaacaggaacaggatacaccatggacacagtaaacagaacacaccaatactcagaaaagggaaagtggacgacaaacacagagactggtgcatcccagctcaacccgattgatggaccactacctgaggataatgaaccaagtgggtatgcacaaacagactgtgttctagaggctatggctttccttgaagaatcccacccaggaatatttgagaattcatgccttgaaacaatggaagttgttcaacaaacaagggtagataaactaactcaaggtcgccagacttatgattggacattaaacagaaatcaaccggcagcaactgcattggccaacaccatagaagtctttagatcgaatggcctaacagctaatgagtcaggaaggctaatagatttcttaaaggatgtaatggaatcaatgaacaaagaggaaatagagataacaacccactttcaaagaaaaaggagagtaagagacaacatgaccaagaagatggtcacgcaaagaacaatagggaagaaaaaacaaagactgaataagagaggctatctaataagagcactgacattaaatacgatgaccaaagatgcagagagaggcaagttaaaaagaagggctatcgcaacacctgggatgcagattagaggtttcgtatactttgttgaaactttagctaggagcatttgcgaaaagcttgaacagtctgggctcccagtagggggcaatgaaaagaaggccaaactggcaaatgttgtgagaaagatgatgactaattcacaagacacagagatttctttcacaatcacaggggacaacactaagtggaatgaaaatcaaaatcctcgaatgttcctggcgatgattacatatatcaccagaaatcaacccgagtggttcagaaacatcctgagcatggcacccataatgttctcaaacaaaatggcaagactagggaaagggtacatgttcgagagtaaaagaatgaagattcgaacacaaataccagcagaaatgctagcaagcattgacctgaagtacttcaatgaatcaacaaagaagaaaattgagaaaataaggcctcttctaatagatggcacagcatcactgagtcctgggatgatgatgggcatgttcaacatgctaagtacggtcttgggagtctcgatactgaatcttggacaaaagaaatacaccaagacagtatactggtgggatgggctccaatcatccgacgattttgctctcatagtgaatgcaccaaaccatgagggaatacaagcaggagtggacagattctacaggacctgcaagttagtgggaatcaacatgagcaaaaagaagtcctatataaataagacagggacatttgaattcacaagctttttttatcgctatggatttgtggctaattttagcatggagctacccagctttggagtgtctggagtaaatgaatcagctgacatgagtattggagtaacagtgataaagaacaacatgataaacaatgaccttggacctgcaacggcccagatggctcttcaattgttcatcaaagactacagatacacatataggtgccataggggagacacacaaattcagacgagaagatcatttgagttaaagaagctgtgggatcaaacccaatcaaaggtagggctattagtatcagatggaggaccaaacttatacaatatacggaatcttcacattcctgaagtctgcttaaaatgggagctaatggatgatgattatcggggaagactttgtaatcccctgaatccctttgtcagtcataaagagattgattctgtaaacaatgctgtggtaatgccagcccatggtccagccaaaagcatggaatatgatgccgttgcaactacacattcctggattcccaagaggaatcgttctattctcaacacaagccaaaggggaattcttgaggatgaacagatgtaccagaagtgctgcaatctattcgagaaatttttccctagcagttcatataggagaccggttggaatttctagcatggtggaggccatggtgtctagggcccggattgatgccagggtcgacttcgagtctggacggatcaagaaagaagagttctctgagatcatgaagatctgttccaccattgaagaactcagacggcaaaaataa

>H1N1_PB1_CY172709_A/New York/1073/2007

atggatgtcaatccgacattacttttcttaaaagtgccagcacaaaatgctataagcacaacttttccttatactggtgaccctccttacagccatgggacaggaacagggtacaccatggatacagtcaacaggacacaccagtactcagaaagaggaagatggacaaaaaataccgaaactggagcaccgcaactcaacccaattgatgggccactaccagaagacaatgaaccgagtggctatgcccaaacagattgtgtattagaagcaatggctttccttgaagaatcccatcctggtatctttgaaaactcttgtattgagacaatggaggttgttcaacaaacaagggtggacaaactgacacagggcagacagacctatgactggactctaaataggaaccagcctgctgccacagcattggcaaacactatagaagtattcagatcaaacggcctcatagcaaatgaatctgggaggctaatagacttccttaaagatgtaatggagtcgatggacatagacgaagtagaggtcacaactcattttcaaagaaagaggagagtgagagacaatgtaactaaaaaaatggtgacccaaagaacaataggcaaaaagaaacataaattagacaaaagaagttacctaattagggcattaaccctgaacacaatgaccaaagatgctgagagagggaaactaaaacgcagagcaattgcaaccccaggaatgcaaataagggggtttgtatactttgttgagacactggcaagaagcatatgtgaaaagcttgaacaatcaggattgccagttggaggaaatgagaagaaagcaaagttagcaaatgttgtaaggaagatgatgaccaactcccaggacactgaaatttctttcaccatcactggagataacacaaaatggaacgaaaatcaaaaccctagaatgttcttggccatgatcacatatataaccaaaaatcagcctgaatggttcagaaatattctaagtattgccccaataatgttttcaaacaagatggcgagactaggtaaggggtacatgtttgaaagcaagagtatgaaactgagaactcaaatacctgcagagatgctagccaacatagatttgaaatatttcaatgattcaactaaaaagaaaattgaaaaaatccgaccattattaatagatggaactgcatcattgagtcctggaatgatgatgggcatgttcaatatgttgagcaccgtcttgggcgtctccattctgaatcttgggcaaaagagatacaccaagactacttactggtgggatggtcttcaatcgtctgatgattttgctttgattgtgaatgcacccaactatgcaggaattcaagctggagttgacaggttttatcgaacctgtaagctgctcggaattaatatgagcaaaaagaagtcttacataaacagaacaggtacctttgaattcacgagctttttctatcgttatgggtttgttgccaatttcagcatggagcttcctagttttggggtgtctggggtcaatgaatctgcagacatgagtattggagtcactgtcatcaaaaacaatatgataaacaatgaccttggcccagcaactgctcaaatggcccttcagttatttataaaagattacaggtacacttatcgatgccacagaggtgacacacaaatacaaacccggagatcatttgagataaagaaactatgggaccaaacccgctctaaagctgggctgttggtctctgatggaggccccaatttatataacattagaaatctccatattcctgaagtttgcttgaaatgggagttgatggatgaggattatcaggggcgtttatgcaacccattaaacccgtttgtcagccataaagagattgaatcagtaaacaatgcagtgataatgccggcacatggtccagccaaaaatatggagtatgacgctgttgcaacaacacactcctgggtccccaaaagaaatcgatccattttgaacacgagccaacgggggatacttgaagatgagcaaatgtatcagaggtgctgcaatttatttgaaaaattcttcccaagtagctcatacagaagaccagttggaatatccagtatggtagaggctatggtttcaagagcccgaattgatgcacggattgatttcgaatctggaaggataaagaaagaggaatttgctgagatcatgaagatctgttccaccattgaagacctcagacggcaaaaatag

>H1N1_PB1_KP637532_A/Thailand/SN08294/2009

atggatgtcaatccgacattacttttcttaaaagtgccagcacaaaatgctataagcacaacttttccttatactggtgaccctccttacagccatgggacaggaacagggtacaccatggatacagtcaacaggacacaccagtactcagaaaggggaagatggacaaaaaataccgaaacgggagcaccgcaacttaacccaattgatggtcccttaccggaagacaatgaaccaagtggctatgcccaaacagattgtgtattagaggcaatggctttccttgaagaatcccatcccggtatctttgaaaactcttgtattgaaacaatggaggttgttcaacaaacaagggtggacaaactgacacaaggcagacagacctatgactggactctaaataggaaccagcctgctgccacagcattggcaaacactatagaagtattcagatcaaacggcctcatagcaaatgaatctgggaggctaatagacttccttaaagatgtaatggagtcgatggacagaggcgaagtagaggtcacaacccattttcaaagaaagaggagagtgagagacaatgtaactaaaaaaatggtgacccaaagaacaataggcaaaaggaaacataaattagacaaaagaagttacctaattagggcattaaccctgaacacaatgaccaaagatgctgagagggggaaactaaaacgcagagcaattgcaaccccaggaatgcaaataagggggtttgtatactttgttgagacactggcaagaagcatatgtgagaagcttgaacaatcaggattgccagttggaggaaatgagaagaaagcaaagttagcaaatgttgtaaggaagatgatgaccaactcccaggacactgaaatttctttcaccataaccggagataacacaaaatggaacgaaaatcaaaaccctagaatgttcttggccatgatcacatatataaccaaaaatcagcctgaatggttcagaaatattctaagtattgctccaataatgttttcaaacaagatggcgagactaggcaaggggtacatgtttgaaagcaagagtatgaaactgagaactcaaatacctgcagagatgctagccaacatagacttgaaatatttcaatgattcaactaaaaagaaaattgaaaaaatccgaccattattaatagatggaactgcatcattgagtcctggaatgatgatgggcatgttcaatatgttgagcaccgtcttgggcgtctccattctgaatcttgggcaaaaaagatacaccaagactacttactggtgggatggtcttcaatcgtctgatgattttgctttgattgtgaacgcacccaactatgcaggaattcaagctggagttgacaggttttatcgaacctgtaagctgctcggaattaatatgagcaaaaggaagtcttacataaacagaacaggtacctttgaattcacgagctttttctatcgttatgggtttgttgccaatttcagcatggagcttcctagttttggggtgtctggggtcaatgaatctgcagacatgagtattggagtcactgtcatcaaaaacaatatgataaacaatgaccttggcccagcaactgctcaaatggcccttcagttatttatcaaagattacaggtacacttatcgatgccaccgaggtgacacacaaatacaaacccggagatcatttgagataaagaaactatgggaccaaacccgctcaaaagctggactgttggtctctgatggaggccccaatttgtataacattagaaatctccatattcctgaagtttgcttgaaatgggagttgatggatgaggattaccaggggcgtttatgcaacccattaaacccgtttgtcagccacaaagagattgaatctgtgaacagtgcagtgataatgccggcacatggtccagccaaaaatatggagtatgacgctgttgcaacaacacactcctgggtccccaaaagaaatcgatccattttgaacacgagccaaagggggatacttgaagatgagcaaatgtatcagaggtgctgcaatttatttgaaaaattcttcccaagtagctcatacagaagaccagttggaatatccagtatggtagaggctatggtctcaagagcccgaattgatgcacggattgatttcgaatctggaaggataaagaaagaggaatttgctgagatcatgaagatctgttccaccattgaagacctcagacggcaaaaatga

>H1N1_PB1_KT180506_A/Singapore/DMS67/2010

atggatgtcaatccgactctacttttcctaaaaattccagcgcaaaatgccataagcaccacattcccttatactggagatcctccatacagccatggaacaggaacaggatacaccatggacacagtaaacagaacacaccaatactcagaaaagggaaagtggacgacaaacacagagactggtgcaccccagctcaacccgattgatggaccactacctgaggataatgaaccaagtgggtatgcacaaacagactgtgttctagaggctatggctttccttgaagaatcccacccaggaatatttgagaattcatgccttgaaacaatggaagttgttcaacaaacaagggtagataaactaactcaaggtcgccagacttatgattggacattaaacagaaatcaaccggcagcaactgcattggccaacaccatagaagtctttagatcgaatggcctaacagctaatgagtcaggaaggctaatagatttcttaaaggatgtaatggaatcaatgaacaaagaggaaatagagataacaacccactttcaaagaaaaaggagagtaagagacaacatgaccaagaagatggtcacgcaaagaacaatagggaagaaaaaacaaagactgaataagagaggctatctaataagagcactgacattaaatacgatgaccaaagatgcagagagaggcaagttaaaaagaagggctatcgcaacacctgggatgcagattagaggtttcgtatactttgttgaaactttagctaggagcatttgcgaaaagcttgaacagtctgggctcccagtagggggcaatgaaaagaaggccaaactggcaaatgttgtgagaaagatgatgactaattcacaagacacagagatttctttcacaatcactggggacaacactaagtggaatgaaaatcaaaatcctcgaatgttcctggcaatgattacatatatcaccagaaatcaacccgagtggttcagaaacatcctgagcatggcacccataatgttctcaaacaaaatggcaagactagggaaagggtacatgttcgagagtaaaagaatgaagattcgaacacaaataccagcagaaatgctagcaagcattgacctgaagtacttcaatgaatcaacaaagaaaaaaattgagaaaataaggcctcttctaatagatggcacagcatcactgagtcctgggatgatgatgggcatgttcaacatgctaagtacggtcttgggagtctcgatactgaatcttggacaaaagaaatacaccaagacaatatactggtgggatgggctccaatcatccgacgattttgctctcatagtgaatgcaccaaaccatgagggaatacaggcaggagtggacagattctacaggacctgcaagttagtgggaatcaacatgagcaaaaagaagtcctatataaataagacagggacatttgaattcacaagctttttttatcgctatggatttgtggctaattttagcatggagctacccagctttggagtgtctggagtaaatgaatcagctgacatgagtattggagtaacagtgataaagaacaacatgataaacaatgaccttggacctgcaacggcccagatggctcttcaattgttcatcaaagactacagatacacatataggtgccataggggagacacacaaattcagacgagaagatcatttgagttaaagaagctgtgggatcaaacccaatcaaaggtagggctattagtatcagatggaggaccaaacttatacaatatacggaatcttcacattcctgaagtctgcttaaaatgggagctaatggatgatgattatcggggaagactttgtaatcccctgaatccctttgtcagtcataaagagattgattctgtaaacaatgctgtggtaatgccagcccatggtccagccaaaagcatggaatatgatgccgttgcaactacacattcctggattcccaagaggaatcgttctattctcaacacaagccaaaggggaattcttgaggatgaacagatgtaccagaagtgctgcaatctattcgagaaatttttccctagcagttcatataggagaccggttggaatttctagcatggtggaggccatggtgtctagggcccggattgatgccagggtcgacttcgagtctggacggatcaagaaagaagagttctctgagatcatgaagatctgttccaccattgaagaactcagacggcaaaaataa

>H1N1_PB1_KT180366_A/Singapore/DMS1166/2011

atggatgtcaatccgactctacttttcctaaaaattccagcgcaaaatgccataagcaccacattcccttatactggagatcctccatacagccatggaacaggaacaggatacaccatggacacagtaaacagaacacaccaatactcagaaaaggggaagtggacgacaaacacagagactggtgcaccccagctcaacccgattgatggaccactacctgaggataatgaaccaagtgggtatgcacaaacagactgtgttctagaggctatggctttccttgaagaatcccacccaggaatatttgagaattcatgccttgaaacaatggaagttgttcaacaaacaagggtagataaactgactcaaggtcgccagacttatgattggacattaaacagaaatcaaccggcagcaactgcattggccaacaccatagaagtctttagatcgaatggcctaacagctaatgagtcaggaaggctaatagatttcttaaaggatgtaatggaatcaatgaacaaagaggaaatagagataacaacccactttcaaagaaaaaggagagtaagagacaacatgaccaagaagatggtcacgcaaagaacaatagggaagaaaaaacagagactgaataagagaaactatctaataagagcactgacattaaatacgatgaccaaagatgcagagagaggcaagttaaaaagaagggctatcgcaacacctgggatgcagattagaggtttcgtatactttgttgaaactttagctaggagcatttgcgaaaagcttgaacagtctgggctcccagtagggggcaatgaaaagaaggccaaactggcaaatgttgtgagaaagatgatgactaattcacaagacacagagatttctttcacaatcactggggacaacactaagtggaatgaaaatcaaaatcctagaatgttcctggcgatgattacatatatcaccagaaatcaacccgagtggtttagaaacatcctgagcatggcacccataatgttctcaaacaaaatggcaagactagggaaagggtacatgttcgagagtaaaagaatgaagattcgaacacaaataccagcagaaatgctagcaagcattgacctgaagtacttcaatgaatcaacaaagaagaaaattgagaaaataaggcctcttttaatggatggcacagcatcactgagtcctgggatgatgatgggcatgttcaacatgctaagtacggtcttgggagtctcgatactgaatcttggacaaaagaaatacaccaagacaacatactggtgggatgggctccaatcatccgacgattttgctctcatagtgaatgcaccaaaccatgaaggaatacaagcaggagtggacagattctacaggacctgcaagttagtgggaatcaacatgagcaaaaagaagtcctatataaataagacagggacatttgaattcacaagctttttttatcgctatggatttgtggctaattttagcatggagctacccagctttggagtgtctggagtaaatgaatcagctgacatgagtattggagtaacagtgataaagaacaacatgataaacaatgaccttggacctgcaacggcccagatggctcttcaattgttcatcaaagactacagatacacatataggtgccataggggagacacacaaattcagacgagaagatcatttgagttaaagaagctgtgggatcaaacccaatcaaaggtagggctattagtatcagatggaggaccaaacttatacaatatacggaatcttcacattcctgaagtctgcttaaaatgggagctaatggatgatgattatcggggaagactttgtaatcccctgaatccctttgtaagtcataaggagattgattctgtaaacaatgctgtggtaatgccagcccatggtccagccaaaagcatggaatatgatgccgttgcaactacacattcctggattcccaagaggaatcgttctattctcaacacaagccaaaggggaattcttgaggatgaacagatgtaccagaagtgctgcaatctattcgagaaattttttcctagcagttcatataggagaccggttggaatttctagcatggtggaggccatggtgtctagggcccggattgatgccagggtcgactttgagtctggacggatcaagaaagaagagttctctgagatcatgaagatctgttccaccattgaagagctcagacggcaaaaataa

>H1N1_PB1_KX006243_A/Wisconsin/19/2016

atggatgtcaatccgactctacttttcctaaaaattccagcacaaaatgccataagcaccacattcccttatactggagatcctccatacagccatggaacaggaacaggatacaccatggacacagtaaacagaacacaccaatactcagaaaaggggaagtggacaacaaacacagagactggtgcaccccagcttaacccgattgacggaccactacctgaagataatgaaccaagtgggtatgcacaaacagactgtgttttagaggctatggcttttcttgaagaatcccatccaggaatatttgagaattcatgccttgaaacaatggaagttgttcaacaaacaagggtagataaactgactcaaggtcgccagacttatgattggacattaaacagaaatcaaccggcagcaactgcattggccaacaccatagaagtttttagatcgaatgacctaacagctaacgagtcaggaaggctaatagatttcttaaaggatgtgatggaatcaatgaacaaagaggaaatagagataacaacccactttcaaagaaaaaggagagtaagagacaacatgaccaagaagatggtcacgcaaagaacaatagggaagaaaaaacagagactgaataagagaggctatctaataagagcactgacattaaatacgatgaccaaagatgcagagagaggcaagttaaaaagaagggctatcgcaacacctgggatgcagattagaggtttcgtatactttgttgaaactttagctaggagcatttgcgaaaagcttgaacagtctgggctcccagtagggggcaatgaaaagaaggccaaattggcaaatgttgtgagaaagatgatgactaattcacaagatacagagatttctttcacaatcactggggacaacactaagtggaatgaaaatcaaaaccctcgaatgttcctggcgatgattacatatatcaccagaaatcaacccgagtggtttagaaacatcctgagcatggcacccataatgttctcaaataaaatggcaagactaggaaaagggtacatgttcgagagtaaaagaatgaagattcgaacacaaataccagcagaaatgctagcaagcattgatctgaagtatttcaatgaatcaacaaagaagaaaattgagaaaataaggcctcttttaatggatggcacagcatcactgagtcctgggatgatgatgggcatgttcaacatgctaagtacggtcttgggagtctcgatactgaatcttggacaaaagaaatacaccaagacaacatactggtgggatgggctccaatcatccgacgattttgctctcatagtgaatgcaccaaaccatgaaggaatacaagcaggagtggacagattctacaggacctgcaagttagtgggaatcaacatgagcaaaaagaagtcctatataaataagacagggacatttgaattcacaagctttttttatcgctatggatttgtggctaattttagcatggagctacccagctttggagtgtctggagtaaatgaatcagctgacatgagtattggagtaacagtgataaagaacaacatgataaacaatgaccttggacctgcaacggctcagatggctcttcaattgttcatcaaagactacagatacacatataggtgccataggggagacacacaaattcagacgagaagatcatttgagttgaagaagctatgggatcaaacccaatcaaaggtagggctattagtatcagatggaggaccaaacttatacaacatacggaatcttcacattcctgaagtctgcttaaaatgggagctaatggatgatgattatcggggaagactttgtaatcccctgaatccctttgtgagtcataaggagattgattctgtaaacaatgctgtggtaatgccagcccatggcccagccaaaagcatggaatatgatgccgttgctactacacattcctggattcccaagaggaatcgttctattctcaacacaagccaaaggggaattcttgaggatgaacagatgtaccagaagtgttgcaatttattcgagaaatttttccctagcagttcatataggagaccggttggaatttctagcatggtggaggccatggtgtctagggcccggattgatgccagagtcgactttgagtctggacggatcaagaaagaagagttctctgagatcatgaagatctgttccaccattgaagaactcagacggcaaaaataa

>H1N1_PB1_KX406313_A/Massachusetts/04/2016

atggatgtcaatccgactctacttttcctaaaaattccagcacaaaatgccataagcaccacattcccttatactggagatcctccatacagccatggaacaggaacaggatacaccatggacacagtaaacagaacacaccaatactcagaaaaggggaagtggacaacaaacacagagactggtgcaccccagctcaacccgattgacggaccactacctgaagataatgaaccaagtgggtatgcacaaacagactgtgttctagaggctatggcttttcttgaagaatcccatccaggaatatttgagaattcatgccttgaaacaatggaagttgttcaacaaacaagggtagataaactgactcaaggtcgccagacttatgattggacattaaacagaaatcaaccggcagcaactgcattggccaacaccatagaagtttttagatcgaatgacctaacagctaacgagtcaggaaggctaatagatttcttaaaggatgtgatggaatcaatgaacaaagaggaaatagagataacaacccactttcaaagaaaaaggagagtaagagacaacatgaccaagaagatggtcacgcaaagaacaatagggaagaaaaaacagagactgaataagagaggctatctaataagagcactgacattaaatacgatgaccaaagatgcagagagaggcaagttaaaaagaagggctatcgcaacacctgggatgcagattagaggtttcgtatactttgttgaaactttagctaggagcatttgcgaaaagcttgaacagtctgggctcccagtagggggcaatgaaaagaaggccaaattggcaaatgttgtgagaaagatgatgactaattcacaagatacagagatttctttcacaatcactggggacaacactaagtggaatgaaaatcaaaatcctcgaatgttcctggcgatgattacatatatcaccagaaatcaacccgagtggtttagaaacatcctgagcatggcacccataatgttctcaaataaaatggcaagactaggaaaagggtacatgttcgagagtaaaagaatgaagattcgaacacaaataccagcagaaatgctagcaagcattgatctgaagtatttcaatgaatcaacaaagaagaaaattgagaaaataaggcctcttttaatggatggcacagcatcactgagtcctgggatgatgatgggcatgttcaacatgctaagtacggtcttgggagtctcgatactgaatcttggacaaaagaaatacaccaagacaacatactggtgggatgggctccaatcatccgacgattttgctctcatagtgaatgcaccaaaccatgaaggaatacaagcaggagtggacagattctacaggacctgcaagttagtgggaatcaacatgagcaaaaagaagtcctatataaataagacagggacatttgaattcacaagctttttttatcgctatggatttgtggctaattttagcatggagctacccagctttggagtgtctggagtaaatgaatcagctgacatgagtattggagtaacagtgataaagaacaacatgataaacaatgaccttggacctgcaacggctcagatggctcttcaattgttcatcaaagactacagatacacatataggtgccataggggagacacacaaattcagacgagaagatcatttgagttgaagaagctatgggatcaaacccaatcaaaggtagggctattagtatcagatggaggaccaaacttatacaacatacggaatcttcacattcctgaagtctgcttaaaatgggagctaatggatgatgattatcggggaagactttgtaatcccctgaatccctttgtgagtcataaggagattgattctgtaaacaatgctgtggtaatgccagcccatggcccagccaaaagcatggaatatgatgccgttgctactacacattcctggattcccaagaggaatcgttctattctcaacacaagccaaaggggaattcttgaggatgaacagatgtaccagaagtgttgcaatttattcgagaaatttttccctagcagttcatataggagaccgattggaatttctagcatggtggaggccatggtgtctagggcccggattgatgccagagtcgactttgagtctggacggatcaagaaagaagagttctctgagatcatgaagatctgttccaccattgaagaactcagacggcaaaaataa

>H1N1_PB1_KY003258_A/Colorado/30/2016

atggatgtcaatccgactctacttttcctaaaaattccagcacaaaatgccataagcaccacattcccttatactggagatcctccatacagccatggaacaggaacaggatacaccatggacacagtaaacagaacacaccaatactcagaaaaggggaagtggacaacaaacacagagactggtgcaccccagctcaacccgattgacggaccactacctgaagataatgaaccaagtgggtatgcacaaacagactgtgttctagaggctatggcttttcttgaagaatcccatccaggaatatttgagaattcatgccttgaaacaatggaagttgttcaacaaacaagggtagataaactgactcaaggtcgccagacttatgattggacattaaacagaaatcaaccggcagcaactgcattggccaacaccatagaagtttttagatcgaatgacctaacagctaacgagtcaggaaggctaatagatttcttaaaggatgtgatggaatcaatgaacaaagaggaaatagagataacaacccactttcaaagaaaaaggagagtaagagacaacatgaccaagaagatggtcacgcaaagaacaatagggaagaaaaaacagagactgaataagagaggctatctaataagagcactgacattaaatacgatgaccaaagatgcagagagaggcaagttaaaaagaagggctatcgcaacacctgggatgcagattagaggtttcgtatactttgttgaaactttagctaggagcatttgcgaaaagcttgaacagtctgggctcccagtagggggcaatgaaaagaaggccaaattggcaaatgttgtgagaaagatgatgactaattcacaagatacagagatttctttcacaatcactggggacaacactaagtggaatgaaaatcaaaatcctcgaatgttcctggcgatgattacatatatcaccagaaatcaacccgagtggtttagaaacatcctgagcatggcacccataatgttctcaaataaaatggcaagactaggaaaagggtacatgttcgagagtaaaagaatgaagattcgaacacaaataccagcagaaatgctagcaagcattgatctgaagtatttcaatgaatcaacaaagaagaaaattgagaaaataaggcctcttttaatggatggcacagcatcactgagtcctgggatgatgatgggcatgttcaacatgctaagtacggtcttgggagtctcgatactgaatcttggacaaaagaactacaccaagacaacatactggtgggatgggctccaatcatccgacgattttgctctcatagtgaatgcaccaaaccatgaaggaatacaagcaggagtggacagattctacaggacctgcaagttagtgggaatcaacatgagcaaaaagaagtcctatataaataagacagggacatttgaattcacaagctttttttatcgctatggatttgtggctaattttagcatggagctacccagctttggagtgtctggagtaaatgaatcagctgacatgagtattggagtaacagtgataaagaacaacatgataaacaatgaccttggacctgcaacggctcagatggctcttcaattgttcatcaaagactacagatacacatataggtgccataggggagacacacaaattcagacgagaagatcatttgagttgaagaagctatgggatcaaacccaatcaaaggtagggctattagtatcagatggaggaccaaacttatacaacatacggaatcttcacattcctgaagtctgcttaaaatgggagctaatggatgatgattatcggggaagactttgtaatcccctgaatccctttgtgagtcataaggagattgattctgtaaacaatgctgtggtaatgccagcccatggcccagccaaaagcatggaatatgatgccgttgctactacacattcctggattcccaagaggaatcgttctattctcaacacaagccaaaggggaattcttgaggatgaacagatgtaccagaagtgttgcaatttattcgagaaatttttccctagcagttcatataggagaccggttggaatttctagcatggtggaggccatggtgtctagggcccggattgatgccagagtcgactttgagtctggacggatcaagaaagaagagttctctgagatcatgaagatctgttccaccattgaagaactcagacggcaaaaataa

>H1N1_PB1_CY209526_A/Oklahoma/12/2016

atggatgtcaatccgactctacttttcctaaaaattccagcacaaaatgccataagcaccacattcccttatactggagatcctccatacagccatggaacaggaacaggatacaccatggacacagtaaacagaacacaccaatactcagaaaaggggaagtggacaacaaacacagagactggtgcaccccagctcaacccgattgacggaccactacctgaagataatgaaccaagtgggtatgcacaaacagactgtgttctagaggctatggcttttcttgaagaatcccatccaggaatatttgagaattcatgccttgaaacaatggaagttgttcaacaaacaagggtagataaactgactcaaggtcgccagacttatgattggacattaaacagaaatcaaccggcagcaactgcattggccaacaccatagaagtttttagatcgaatgacctaacagctaacgagtcaggaaggctaatagatttcttaaaggatgtgatggaatcaatgaacaaagaggaaatagagataacaacccactttcaaagaaaaaggagagtaagagacaacatgaccaagaagatggtcacgcaaagaacaatagggaagaaaaaacagagactgaataagagaggctatctaataagagcactgacattaaatacgatgaccaaagatgcagagagaggcaagttaaaaagaagggctatcgcaacacctgggatgcagattagaggtttcgtatactttgttgaaactttagctaggagcatttgcgaaaagcttgaacagtctgggctcccagtagggggcaatgaaaagaaagccaaattggcaaatgttgtgagaaagatgatgactaattcacaagatacagagatttctttcacaatcactggggacaacactaagtggaatgaaaatcaaaatcctcgaatgttcctggcgatgattacatatatcaccagaaatcaacccgagtggtttagaaacatcctgagcatggcacccataatgttctcaaataaaatggcaagactaggaaaagggtacatgttcgagagtaaaagaatgaagattcgaacacaaataccagcagaaatgctggcaagcattgatctgaagtatttcaatgaatcaacaaagaagaaaattgagaaaataaggcctcttttaatggatggcacagcatcactgagtcctgggatgatgatgggcatgttcaacatgctaagtacggtcttgggagtctcgatactaaatcttggacaaaagaaatacaccaagacaacatactggtgggatgggctccaatcatccgacgattttgctctcatagtgaatgcaccaaaccatgaaggaatacaagcaggagtggacagattctacaggacctgcaagttagtgggaatcaacatgagcaaaaagaagtcctatataaataagacagggacatttgaattcacaagctttttttatcgctatggatttgtggctaattttagcatggagctacccagctttggagtgtctggagtaaatgaatcagctgacatgagtattggagtaacagtgataaagaacaacatgataaacaatgaccttggacctgcaacggctcagatggctcttcaattgttcatcaaagactacagatacacatataggtgccataggggagacacacaaattcagacgagaagatcatttgagttgaagaagctatgggatcaaacccaatcaaaggtagggctattagtatcagatggaggaccaaacttatacaacatacggaatcttcacattcctgaagtctgcttaaaatgggagctaatggatgatgattatcggggaagactttgtaatcccctgaatccctttgtgagtcataaggagattgattctgtaaacaatgctgtggtaatgccagcccatggcccagccaaaagcatggaatatgatgccgttgctactacacattcctggattcccaagaggaatcgttctattctcaacacaagccaaaggggaattcttgaggatgaacagatgtaccagaagtgttgcaatttattcgagaaatttttccctagcagttcatataggagaccggttggaatttctagcatggtggaggccatggtgtctagggcccgaattgatgccagagtcgactttgagtctggacggatcaaaaaagaagagttctctgagatcatgaagatctgttccaccattgaagaactcagacggcaaaaataa

>H1N1_PB1_CY233233_A/Tennessee/24/2017

atggatgtcaatccgactctacttttcttaaaaattccagcacaaaatgccataagcaccacattcccttatactggagatcctccatatagccatggaacaggaacaggatacaccatggacacagtaaacagaacacaccaatactcagaaaagggaaagtggacaacaaacacagagactggtgcaccccagctcaacccgattgatggaccactacctgaggataatgaaccaagtgggtatgcacaaacagactgtgttctagaggctatggctttccttgaagaatcccacccaggaatatttgagaattcatgccttgaaacaatggaagttgttcaacaaacaagggtagataaactgactcaaggtcgccagacttatgattggacattaaacagaaatcaaccggcagcaactgcattggccaacaccatagaagtctttagatcgaatgacctaacagctaacgagtcaggaaggctaatagatttcttaaaggatgtaatggaatcaatgaacaaagaggaaatagagataacaacccactttcaaagaaaaaggagagtaagagacaacatgaccaagaagatggtcacgcaaagaacaatagggaagaaaaaacagagactgaataagagaggctatctaataagagcactgacattaaatacgatgaccaaagatgcagagagaggcaagttaaaaagaagggctatcgcaacacctgggatgcagattagaggtttcgtatactttgttgaaactttagctaggagcatttgcgaaaagcttgaacagtctgggctcccagtagggggcaatgaaaagaaggccaaattggcaaatgttgtgagaaagatgatgactaattcacaagatacagagatttctttcacaatcaccggggacaacactaagtggaatgaaaatcaaaatcctcgaatgttcctggcgatgattacatatatcaccagaaatcagcccgagtggtttagaaacatcctgagcatggcacccataatgttctcaaataaaatggcaagactggggaaagggtacatgttcgagagtaaaagaatgaagattcgaacacaaataccagcagaaatgctagcaagcattgatctgaagtacttcaatgaatcaacaaagaagaaaattgagaaaataaggcctcttttaatggatggcacagcatcactgagtcctgggatgatgatgggcatgttcaacatgctaagtacggtattgggagtctcgatactgaatcttggacaaaagaaatacaccaagacaacatactggtgggatgggctccaatcatccgacgattttgctctcatagtgaatgcaccaaaccacgaaggaatacaagcaggagtggacaggttctacaggacctgcaagttagtgggaatcaacatgagcaaaaagaagtcctatataaataagacagggacatttgaattcacaagctttttttatcgctatggatttgtggctaattttagcatggagctacccagctttggagtgtctggagtaaatgaatcagctgacatgagtattggagtaacagtgataaagaacaacatgataaacaatgaccttggacctgcaacggcccagatggctcttcaattgttcatcaaagactacagatacacatataggtgccataggggagacacacaaattcagacgagaagatcttttgagttaaagaagctatgggatcaaacccaatcaaaggtagggctattagtatcagatggaggaccaaacttatacaacatacggaatcttcacattcctgaagtctgcttaaaatgggagctaatggatgatgattatcggggaagactttgtaatcccctgaatccctttgtgagtcataaggagattgattctgtaaacaatgccgtggtaatgccagcccatggtccagccaaaagcatggaatatgatgccgttgcaactacacattcctggattcccaagaggaatcgttctattctcaacacaagccaaaggggaattcttgaggatgaacagatgtaccagaagtgttgcaatctattcgagaaatttttccctagcagttcatataggagaccggttggaatttctagcatggtggaggccatggtgtctagggcccggattgatgccagagtcgactttgagtctggacggatcaagaaagaagagttctctgagatcatgaagatctgttccaccattgaagaactcagacggcaaaaataa

>H1N1_PB1_CY233934_A/New York/23/2017

atggatgtcaatccgactctacttttcttaaaaattccagcacaaaatgccataagcaccacattcccttatactggagatcctccatatagccatggaacaggaacaggatacaccatggacacagtaaacagaacacaccaatactcagaaaagggaaagtggacaacaaacacagagactggtgcacctcagctcaacccgattgatggaccactacctgaggataatgaaccaagtgggtatgcacaaacagactgtgttctagaggctatggctttccttgaagaatcccacccaggaatatttgagaattcatgccttgaaacaatggaagttgttcaacaaacaagggtagataaactgactcaaggtcgccagacttatgattggacattaaacagaaatcaaccggcagcaactgcattggccaacaccatagaagtctttagatcgaatgacctaacagctaacgagtcaggaaggctaatagatttcttaaaggatgtaatggaatcaatgaacaaagaggaaatagagataacaacccactttcaaagaaaaaggagagtaagagacaacatgaccaagaagatggtcacgcaaagaacaatagggaagaaaaaacagagactgaataagagaggctatctaataagagcactgacattaaatacgatgaccaaagatgcagagagaggcaagttaaaaagaagggctatcgcaacacctgggatgcagattagaggtttcgtatactttgttgaaactttagctaggagcatttgcgaaaagcttgaacagtctgggctcccagtagggggcaatgaaaagaaggccaaattggcaaatgttgtgagaaagatgatgactaattcacaagatacagagatttctttcacaatcaccggggacaacactaagtggaatgaaaatcaaaatcctcgaatgttcctggcgatgattacatatatcaccagaaatcaacccgagtggtttagaaacatcctgagcatggcacccataatgttctcaaataaaatggcaagactggggaaagggtacatgttcgagagtaaaagaatgaagattcgaacacaaataccagcagaaatgctagcaagcattgatctgaagtacttcaatgaatcaacaaagaagaaaattgagaaaataaggcctcttttaatggatggcacagcatcactgagtcctgggatgatgatgggcatgttcaacatgctaagtacggtattgggagtctcgatactgaatcttggacaaaagaaatacaccaagacaacatactggtgggatgggctccaatcatccgacgattttgctctcatagtgaatgcaccaaaccacgaaggaatacaagcaggagtggacaggttctacaggacctgcaagttggtgggaatcaacatgagcaaaaagaagtcctatataaataagacagggacatttgaattcacaagctttttttatcgctatggatttgtggctaattttagcatggagctacccagctttggagtgtctggagtaaatgaatcagctgacatgagtattggagtaacagtgataaagaacaacatgataaacaatgaccttggaccagcaacggcccagatggctcttcaattgttcatcaaagactacagatacacatataggtgccataggggagacacacaaattcagacgagaagatcttttgagttaaagaagctatgggatcaaacccaatcaaaggtagggctattagtatcagatggaggaccaaacttatacaacatacggaatcttcacattcctgaagtctgcttaaaatgggagctaatggatgatgattatcggggaagactttgtaatcccctgaatccctttgtgagtcataaggagattgattctgtaaacaatgccgtggtaatgccagcccatggtccagccaaaagcatggaatatgatgccgttgcaactacacattcctggattcccaagaggaatcgttctattctcaacacaagccaaaggggaattcttgaggatgaacagatgtaccaraagtgttgcaatctattcgagaaatttttccctagcagttcatataggagaccggttggaatttctagcatggtggaggccatggtgtctagggcccggattgatgccagagtcgactttgagtctggacggatcaagaaagaagagttctctgagatcatgaagatctgttccaccattgaagaactcagacggcaaaaataa

>H1N1_PB1_CY009002_A/New York/484/2003

atggatgtcaatccgacattacttttcttaaaagtgccagcacaaaatgctataagcacaacttttccttatactggtgaccctccttacagccatgggacaggaacagggtacaccatggatacagtcaacaggacacatcagtactcagaaagaggaagatggacaaaaaataccgaaactggagcaccgcaactcaacccaattgatgggccactaccagaagacaatgaaccaagtggctatgcccaaacagattgtgtattagaagcaatggctttccttgaagaatcccatcctggtatttttgaaaactcttgtattgaaacaatggaggttgttcagcaaacaagggtggacaaactgacacaaggcagacagacctatgactggactctaaataggaaccagcctgctgccacagcattggcaaacactatagaagtattcagatcaaacggcctcatagcaaatgaatctgggaggctaatagacttccttaaagatgtaatggagtcgatggacagagacgaagtagaggtcacaactcattttcaaagaaagaggagagtgagagacaatgtaactaaaaaaatggtgacccaaagaacaataggcaaaaagaaacataaattagacaaaagaagttacctaattagggcattaaccctgaacacaatgaccaaagatgctgagagggggaaactaaaacgcagagcaattgcaaccccaggaatgcaaataagggggtttgtatactttgttgagacactggcaagaagcatatgtgaaaagcttgaacaatcagggttgccagttggaggaaatgagaagaaagcaaagttagcaaatgttgtaaggaagatgatgaccaactcccaggacactgaaatttctttcaccatcactggagataacacaaaatggaacgaaaatcaaaaccctagaatgttcttggccatgatcacatatataaccaaaaatcagcctgaatggttcagaaatattctaagtattgctccaataatgttttcaaacaagatggcgagactaggtaaggggtacatgtttgaaagcaagagtatgaaactgagaactcaaatacctgcagagatgctagccaacatagatttgaaatatttcaatgattcaactaaaaagaaaattgaaaaaatccgaccattattaatagatggaactgcatcattgagtcctggaatgatgatgggcatgttcaatatgttaagcaccgtcttgggcgtctccattctgaatcttgggcaaaagagatacaccaagactacttactggtgggatggtcttcaatcgtctgatgattttgctttgattgtgaatgcacccaactatgcaggaattcaagctggagtcgacaggttttatcgaacctgtaagctgctcggaattaatatgagcaaaaagaagtcttacataaacagaacaggtacctttgaattcacgagctttttctatcgttatgggtttgttgccaatttcagcatggagcttcctagttttggggtgtctggggtcaatgaatctgcagacatgagtattggagtcactgtcatcaaaaacaatatgataaacaatgaccttggcccagcaactgctcaaatggcccttcagttatttataaaagattacaggtacacttatcgatgccacagaggtgacacacaaatacaaacccggagatcatttgagataaagaaactatgggaccaaactcgctccaaagctgggctgttggtatctgatggaggccccaatttatataacattagaaatctccatattcctgaagtctgcttgaaatgggagttgatggatgaggattaccaggggcgtttatgcaacccattgaacccgtttgtcagtcataaagagattgaatcagtgaacaatgcagtgataatgccggcacatggtccagccaaaaatatggagtatgacgctgttgcaacaacacactcctgggtccccaaaaggaatcgatccattttgaacacgagccaaagggggatacttgaagatgagcaaatgtatcagaggtgctgcaatttatttgaaaaattcttcccaagtagctcatacagaagaccagttggaatatccagtatggtagaggctatggtttcaagagcccgaattgatgcacggattgattttgaatctggaaggataaagaaagaggaattcgctgagatcatgaagacctgttccaccattgaagacctcagacggcaaaaatag

>H1N1_PB1_CY010898_A/Memphis/15/1978

atggatgtcaatccgaccttacttttcttaaaagtgccagcacaaaatgctataagcacaactttcccttatactggagaccctccttacagccatgggacaggaacagggtacaccatggatacagtcaacaggacacatcagtactcagaaaggggaagatggacaaaaaacaccgaaactggagcaccgcaactcaacccgattgatgggccactaccaaaagacaatgaaccaagtggttatgcccaaacagattgtgtattagaagcaatggctttccttgaggaatcccatcctggtatttttgaaaactcgtgtattgaaacgatggaggttgttcagcaaacacgagtggacaaactgacacaaggcagacagacctatgactggactctaaatagaaaccagcctgctgctacagcattggccaacaccatagaagtgttcagattaaatggcctcatggcaaatgaatctggaaggctaatagacttccttaaagatgtaatggagtcaatggacagagaagaagtggagatcacaactcattttcagagaaagagaagagtgagggacaatgtgactaagaaaatggtgacacagagaacaataggcaaaaagaaacagagattaaacaaaaggagttatctaattagggcattaaccctgaacacaatgaccaaagatgctgagagagggaaactaaaacgtagagcaattgcaaccccagggatgcaaataagggggtttgtatactttgttgagacactggcaaggagtatatgtgaaaaacttgaacaatcagggttgccagttggaggaaatgaaaagaaagcaaagttggcaaatgttgtaaggaagatgatgaccaattctcaggacactgaaatttctttcaccatcactggagataacaccaaatggaacgaaaatcagaaccctagaatgtttttggccatgatcacatatataaccagaaatcagcccgaatggttcagaaatattctaagtattgctccaataatgttctcaaacaaaatggcgagactaggtaaggggtacatgtttgaaagcaagagtatgaaacttagaactcaaatacctgcagaaatgctagcaaacatcgatttgaagtatttcaatgattcaacaagaaagaaaattgaaaaaatccggccactcttaatagatgggactgcatcactgagccctggaatgatgatgggcatgttcaatatgttaagcactgtattgggcgtctccatcctgaatcttggacaaaagagatacaccaagactacttactggtgggatggtcttcaatcgtctgatgattttgctctgattgtgaatgcacccaaccatgcaggaattcaagctggagttgacaggttttatcgaacctgtaagctgctcggaatcaatatgagcaagaaaaagtcttacataaacagaacaggtacatttgaattcacgagctttttctatcgttatgggtttgttgccaatttcagcatggagcttcccagttttggggtgtctgggatcaacgagtctgcagacatgagtattggagtcactgtcatcaaaaacaatatgataaacaatgatcttggtccagcaaccgctcaaatggcccttcagttattcatcaaagattacaggtacacgtatcgatgccacagaggtgacacacaaatacaaacccgaagatcatttgaaataaagaaactgtgggagcaaacccgttccaaagctgggctgctggtctccgatggaggtcctaatttatacaacattagaaatctccacattcctgaagtctgcttgaaatgggaattgatggatgaggattaccaggggcgtttatgcaacccattgaacccatttgtcagtcataaagagattgaatcagtgaacaatgcagtgatgatgccggcacatggtccagccaaaaatatggagtatgatgctgttgcaacaacacactcctgggtccccaaaaggaatcgatccatcttgaatacgagccaaagaggaatacttgaagatgaacaaatgtatcaaaggtgctgcaatttgtttgaaaaattcttccccagcagttcatacagaaggccagtcggaatatccagtatggtagaggctatggtttccagagcccgaattgatgcacggattgatttcgaatctggaaggataaagaaagaggagttcactgagatcatgaagacctgttccaccattgaagaactcagacggcaaaaatag

>H1N1_PB1_CY016058_A/Nanchang/21/1996

atggatgtcaatccgaccttacttttcttaaaagtgccagcacaaaatgctataagcacaacttttccttatactggagaccctccttacagccatgggacaggaacagggtacaccatggatacagtcaacaggacacatcagtactcagaaagaggaagatggacaaaaaataccgaaactggagcaccgcaactcaacccaattgatgggccactaccaaaagacaatgaaccaagtggctatgcccaaacagattgtgtattagaagcaatggctttccttgaggaatcccatcctggtatttttgaaaactcttgtattgaaacaatggaggttgttcagcaaacaagggtggacaaactgacacaaggcagacagacctatgactggactctaaatagaaaccagcctgctgccacagcattggccaacactatagaagtgttcagatcaaacggcctcatagcaaatgaatctgggaggctaatagacttccttaaagatgtaatggagtcgatggacagagacgaagtagagatcacaactcattttcagagaaagaggagagtgagagacaatgtaactaaaaaaatggtgacccaaagaacaataggcaaaaagaaacataaattagacaaaagaagttacctaattagggcattaaccctgaacacaatgaccaaagatgctgagagggggaaactaaaacgtagagcaattgcaaccccaggaatgcaaataagggggtttgtatactttgttgagacactggcaaggagcatatgtgaaaagcttgaacaatcagggttgccagttggaggaaatgaaaagaaagcaaagttagcaaatgttgtaaggaagatgatgaccaactcccaggacactgaaatttctttcaccatcactggagataacacaaaatggaacgaaaatcaaaaccctagaatgttcttggccatgatcacatatataaccaaaaatcagcctgaatggttcagaaatattctaagtattgctccaataatgttttcaaacaaaatggcgagactaggtaaggggtacatgtttgaaagcaagagtatgaaactgagaactcaaatacctgcagagatgctagccaacatagatttgaaatatttcaatgattcaacaaaaaagaaaattgaaaaaatccggccattattaatagatggaactgcatcattgagtcctggaatgatgatgggcatgttcaatatgttaagcactgtcttgggcgtctccattctgaatcttgggcaaaagagatacaccaagactacttactggtgggatggtcttcaatcgtctgatgattttgctctgattgtgaatgcacccaactatgcaggaattcaagctggagttgacaggttttatcgaacctgtaagctgctcggaattaatatgagcaaaaagaagtcttacataaacagaacaggtacctttgaattcacgagctttttctatcgttatgggtttgttgccaatttcagcatggagcttcctagttttggggtgtctggggtcaatgagtctgcagacatgagtattggagtcactgtcatcaaaaacaatatgataaacaatgatcttggcccagcaactgctcaaatggcccttcagttatttatcaaagattacaggtacacgtatcgatgccacagaggtgacacacaaatacaaacccggagatcatttgagataaagaaactatgggaccaaacccgctccaaagctgggctgttggtctctgatggaggccccaatttatataacattagaaatctccacattcctgaagtctgcttgaaatgggagttgatggatgaggattaccaggggcgtttatgcaacccattgaacccgtttgtcagtcataaagagattgaatcagtgaacaatgcagtgatgatgccggcacatggtccagccaaaaatatggagtatgacgctgttgcaacaacacactcctgggttcccaaaaggaatcgatccattttgaatacgagccaaagggggatacttgaggatgagcaaatgtatcagaggtgctgcaatttatttgaaaaattcttcccaagtagctcatacagaagaccagttggaatatccagtatggtagaggctatggtttccagagcccgaattgatgcacggattgatttcgaatctggaaggataaagaaagaggagttcgctgagatcatgaagacctgttccaccattgaagacctcagacggcaaaaatag

>H1N1_PB1_CY021827_A/Albany/12/1951

atggatgtcaatccgaccttacttttcttaaaagtgccagcacaaaatgctataagcacaactttcccttatactggagaccctccttacagccatgggacaggaacagggtacaccatggatactgtcaacaggacacatcagtactcagaaaggggaagatggacaaaaaacaccgaaactggagcaccacaactcaacccaattgatgggccactaccagaagacaatgaaccaagtggttatgcccaaacagattgtgtattagaagcaatggctttccttgaggaatcccatcctggtatttttgaaaactcatgtattgaaacgatggaggttgttcagcaaacacgagtggacaagctgacacaaggccgacagacctatgactggactctgaatagaaaccagcctgctgctacagcattggccaacacaatagaagtgttcagatcaaatggcctcatggcaaatgaatctggaaggctaatagactttcttaaggatgtaatggagtcaatggacaaagaagaaatggaggtcacaactcattttcagagaaagagacgagtgagggacaatgtgactaagaaaatggtgacacaaagaacaataggcaaaaagaaacagagattaaacaaaaggagttatctaattagggcattgaccctgaacacaatgaccaaagatgctgagagagggaaactaaaacgaagagcaattgcaaccccagggatgcaaataagggggtttgtatactttgttgagacactggcaagaagtatatgtgaaaaacttgaacaatcaggattgccagttggaggaaatgaaaagaaagcaaagttggcaaatgttgtaaggaagatgatgaccaattctcaggacactgaaatttctttcaccatcactggagataacaccaaatggaacgaaaatcagaaccctagaatgtttttggccatgatcacatatataaccagaaatcagcccgaatggttcagaaatattctaagtattgctccaataatgttctcaaacaaaatggcgagactaggtaagggttacatgtttgagagcaagagtatgaaacttagaactcaaatacctgcagaaatgatagcaaacattgatttgaagtatttcaatgattcaacaagaaagaaaattgaaaaaatccggccgctcttaatagatgggactgcatcactgagccctggaatgatgatgggcatgttcaatatgttaagcactgtattgggcgtctccatcctgaatcttggacaaaagagatacaccaagactacttactggtgggatggtcttcaatcatctgatgattttgctctgattgtgaatgcacccaatcatgcaggaattcaagctggagttgacaggttttatcgaacctgtaagctgctcggaatcaatatgagcaagaaaaagtcttacataaacagaacaggtacatttgaattcacgagctttttctatcgttatgggtttgttgccaatttcagcatggagcttcccagttttggagtgtctgggatcaacgagtctgcagacatgagtattggggtcactgtcatcaaaaacaatatgataaacaatgatcttggtccagcaaccgctcaaatggcccttcagttattcatcaaagattacaggtacacgtatcgatgccacagaggtgacacacaaatacaaacccgaagatcatttgaaataaagaaactatgggagcaaacccgttccaaagctgggctgctggtctccgatggaggtcctaatttatataacattagaaatctccacattcctgaagtctgcttgaaatgggaattgatggatgaggattaccaggggcgtttatgcaacccattgaacccatttgtcagtcataaagagattgaatcagtgaacaatgcagtgatgatgccggcacatggtccagccaaaaacatggagtatgatgctgtagcaacaacacactcctgggtccccaaaagaaatcgatccatcttgaatacgagccaaaggggaatacttgaagatgaacaaatgtatcaaaggtgctgcaatttatttgaaaaattcttccccagcagttcatacagaaggccagtcggaatatccagtatggtggaggctatggtttccagagcccgaattgatgcacggattgatttcgaatctggaaggataaagaaagaggatttcactgagatcatgaagatctgttccaccattgaagaactcagacggcaaaaatag

>H1N1_PB1_CY023024_A/Auckland/595/2000

atggatgtcaatccgacattacttttcttaaaagtgccagcacaaaatgctataagcacaacttttccttatactggtgaccctccttacagccatgggacaggaacagggtacaccatggatacagtcaacaggacacatcagtactcagaaagaggaagatggacaaaaaataccgaaactggagcaccgcaactcaacccaattgatgggccactaccaaaagacaatgaaccaagtggctatgcccaaacagattgtgtattagaagcaatggctttccttgaggaatcccatcctggtatttttgaaaactcttgtattgaaacaatggaggttgttcagcaaacaagggtggacaaactgacacaaggcagacagacctatgactggactctaaataggaaccagcctgctgccacagcattggccaacactatagaagtgttcagatcaaacggcctcatagcaaatgaatctgggaggctaatagacttccttaaagatgtaatggagtcgatggacagagacgaaatagaggtcacaactcattttcagagaaagaggagagtgagagacaatgtaactaaaaaaatggtgacccaaagaacaataggcaaaaagaaacataaattagataaaagaagttacctaattagggcattaaccctgaacacaatgaccaaagatgctgagagggggaaactaaaacgcagagcaattgcaaccccaggaatgcaaataagggggtttgtatactttgttgagacactggcaagaagcatatgtgaaaagcttgaacaatcagggttgccagttggaggaaatgaaaagaaagcaaagttagcaaatgttgtaaggaagatgatgaccaactcccaggacactgaaatttctttcaccatcactggagataacacaaaatggaacgaaaatcaaaaccctagaatgttcttggccatgatcacatatataaccaaaaatcagcctgaatggttcagaaatattctaagtattgctccaataatgttttcaaacaaaatggcgagactaggtaaggggtacatgtttgaaagcaagagtatgaaactgagaactcaaatacctgcagagatgctagccaacatagatttgaaatatttcaatgattcaactaaaaagaaaattgaaaaaatccggccattattaatagatggaactgcatcattgagtcctggaatgatgatgggcatgttcaatatgttaagcaccgtcttgggcgtctccattctgaatcttgggcaaaagagatacaccaagactacttactggtgggatggtcttcaatcgtctgatgattttgctctgattgtgaatgcacccaactatgcaggaattcaagctggagttgacaggttttatcgaacctgtaagctgctcggaattaatatgagcaaaaagaagtcttacataaacagaacaggtacctttgaattcacgagctttttctatcgttatgggtttgttgccaatttcagcatggagcttcctagttttggggtgtctggggtcaatgaatctgcagacatgagtattggagtcactgtcatcaaaaacaatatgataaacaatgaccttggcccagcaactgctcaaatggcccttcagttatttataaaagattacaggtacacgtatcgatgccacagaggtgacacacaaatacaaacccggagatcatttgagataaagaaactatgggaccaaacccgctccaaagctgggctgttggtctctgatggaggccccaatttatataacattagaaatctccatattcctgaagtctgcttgaaatgggagttgatggatgaggattaccaagggcgtttatgcaacccattgaacccgtttgtcagtcataaagagattgaatcagtgaacaatgcagtgatgatgccggcacatggtccagccaaaaatatggagtatgacgctgttgcaacaacacactcctgggttcccaaaaggaatcgatccattttgaatacgagccaaagggggatacttgaggatgagcaaatgtatcagaggtgctgcaatttatttgaaaaattcttcccaagtagctcatacagaagaccagttggaatatccagtatggtagaggctatggtttccagagcccgaattgatgcacggattgatttcgaatctggaaggataaagaaagaggaattcgctgagatcatgaagacctgttccaccattgaagacctcagacggcaaaaatag

>H1N1_PB1_CY025475_A/Vermont/UR06-0576/2007

atggatgtcaatccgacattacttttcttaaaagtgccagcacaaaatgctataagcacaacttttccttatactggtgaccctccttacagccatgggacaggaacagggtacaccatggatacagtcaacaggacacaccagtactcagaaagaggaagatggacaaaaaatacagaaactggagcaccgcaactcaacccaattgatgggccactaccagaagacaatgaaccaagtggctatgcccaaacagattgtgtattagaagcaatggctttccttgaagaatcccatcctggtatctttgaaaactcttgtattgaaacaatggaggttgttcagcaaacaagggtggacaaactgacacaaggcagacagacctatgactggactctaaataggaaccagcctgctgcaacagcattggcaaacactatagaagtattcagatcaaacggcctcatagcaaatgaatctggaaggctaatagacttccttaaagatgtaatggagtcgatggacagagacgaagtagaggtcacaacccattttcaaagaaagagaagagtgagagacaatgtgactaaaaaaatggtgactcaaagaacaataggcaaaaagaaacataaattagacaaaagaagttacctaattagggcattaaccctgaacacaatgaccaaagatgctgagagggggaaactaaaacgcagagcaattgcaaccccaggaatgcaaataagagggtttgtatactttgttgagacactggcaagaagcatatgtgaaaagcttgaacaatcaggattgccagttggaggaaatgagaagaaagcaaagttagcaaatgttgtaaggaagatgatgaccaactcccaggacactgaaatttctttcaccatcactggagataacacaaaatggaacgaaaatcaaaaccctagaatgttcttggccatgatcacatatataaccaaaaatcagcctgaatggttcagaaatattctaagtattgctccaataatgttttcaaacaagatggcgagactaggtaaggggtacatgtttgaaagcaagagtatgaaactgagaactcaaatacctgcagagatgctagccaacatagatttgaaatatttcaatgattcaactaaaaagaaaattgaaaaaatccgaccattattaatagatggaactgcatcattgagtcctggaatgatgatgggcatgttcaatatgttgagcaccgtcttgggcgtctccattctgaatcttgggcaaaagagatacaccaagactacttactggtgggatggtcttcaatcgtctgatgattttgctttgattgtgaatgcacccaactatgcaggaattcaagctggagttgacaggttttatcgaacctgtaagctgctcggaattaatatgagcaaaaagaagtcttacataaacagaacaggtacctttgaattcacgagctttttctatcgttatgggtttgttgccaatttcagcatggagcttcctagttttggggtgtctggggtcaatgaatctgcagacatgagtattggagtcactgtcatcaaaaacaatatgataaacaatgaccttggcccagcaactgctcaaatggcccttcagttatttataaaagattacaggtacacttatcgatgccacagaggtgacacacaaatacaaacccggagatcatttgagataaagaaactatgggaccaaacccgctccaaagctggactgttggtctctgatggaggccccaatttatataacattagaaatctccatattcctgaagtttgcttgaaatgggagttgatggatgaggattaccaggggcgtttatgcaacccattaaacccgtttgtcagccataaagagattgaatcagtgaacaatgcagtgataatgccggcacatggcccagccaaaaatatggagtatgacgctgttgcaacaacacactcctgggtccccaaaagaaatcgatccattttgaacacgagccaaagggggatacttgaagatgagcaaatgtatcagaggtgctgcaatttatttgaaaaattcttcccaagtagctcatacagaagaccagttggaatatccagtatggtagaggctatggtttcaagagcccgaattgatgcacggattgatttcgaatctggaaggataaagagggaggaatttgctgagatcatgaagatctgttccaccattgaagacctcagacggcaaaaatag

>H1N1_PB1_CY027937_A/Oregon/UR06-0291/2007

atggatgtcaatccgactttacttttcttaaaagtgccagcacaaaatgctataagcacaacttttccttatactggtgaccctccttacagccatgggacaggaacagggtacaccatggatacagtcaacaggacacaccagtactcagaaagaggaagatggacaaaaaataccgaaactggagcaccgcaactcaacccaattgatgggccactaccagaagacaatgaaccaagtggctatgcccaaacagattgtgtattagaagcaatggctttccttgaagaatcccatcctggtatctttgaaaactcttgtattgaaacaatggaggttgttcagcaaacaagggtggacaaactgacacaaggcagacagacctatgactggactctaaataggaaccagcctgctgcaacagcattggcaaacactatagaagtattcagatcaaacggcctcatagcaaatgaatctggaaggctaatagacttccttaaagatgtaatggagtcgatggacagagacgaagtagaggtcacaactcattttcaaagaaagaggagagtaagagacaatgtaactaaaaaaatggtgactcaaagaacaataggcaaaaagaaacataaattagacaaaagaagttacctaattagggcattaaccctgaacacaatgaccaaagatgctgagagggggaaactaaaacgcagagcaattgcaaccccaggaatgcaaataagagggtttgtatactttgttgagacactggcaagaagcatatgtgaaaagcttgaacaatcaggattgccagttggaggaaatgagaagaaagcaaagttagcaaatgttgtaaggaagatgatgaccaactcccaggacactgaaatttctttcaccatcactggagataacacaaaatggaacgaaaatcaaaaccctagaatgttcttggccatgatcacatatataaccaaaaatcagcctgaatggttcagaaatattctaagtattgctccaataatgttttcaaacaagatggcgagactaggtaaggggtacatgtttgaaagcaagagtatgaaactgagaactcaaatacctgcagagatgctagtcaacatagatttgaaatatttcaatgattcaactaaaaagaaaattgaaaaaatccgaccattattaatagatggaactgcatcattgagtcctggaatgatgatgggcatgttcaatatgttgagcaccgtcttgggcgtctccattctgaatcttgggcaaaagagatacaccaagactacttactggtgggatggtcttcaatcgtctgatgattttgctttgattgtgaatgcacccaactatgcaggaattcaagctggagttgacaggttttatcgaacctgtaagctgctcggaattaatatgagcaaaaagaagtcttacataaacagaacaggtacctttgaattcacgagctttttctatcgttatgggtttgttgccaatttcagcatggagcttcctagttttggggtgtctggggtcaatgaatctgcagacatgagtattggagtcactgtcatcaaaaacaatatgataaacaatgaccttggcccagcaactgctcaaatggcccttcagttatttataaaagattacaggtacacttatcgatgccacagaggtgacacacaaatacaaacccggagatcatttgagataaagaaactatgggaccaaacccgctccaaagctgggctgttggtctctgatggaggccccaatttatataacattagaaatctccatattcctgaagtttgcttgaaatgggagttgatggatgaggattaccaggggcgtttatgcaacccattaaacccgtttgtcagccataaagagattgaatcagtgaacaatgcagtgataatgccggcacatggcccagccaaaaatatggagtatgacgctgttgcaacaacacactcctgggtccccaaaagaaatcgatccattttgaacacgagccaaagggggatacttgaagatgagcaaatgtatcagaggtgctgcaatttatttgaaaaattcttcccaagtagctcatacagaagaccagttggaatatccagtatggtagaggctatggtttcaagagcccgaattgatgcacggattgatttcgaatctggaaggataaagagggaggaatttgctgagatcatgaagatctgttccaccattgaagacctcagacggcaaaaatag

>H1N1_PB1_CY028113_A/Tennessee/UR06-0076/2007

atggatgtcaatccgacattacttttcttaaaagtgccagcacaaaatgctataagcacaacttttccttatactggtgaccctccttacagccatgggacaggaacagggtacaccatggatacagtcaacaggacacaccagtactcagaaagaggaagatggacaaaaaataccgaaactggagcaccgcaactcaacccaattgatgggccactaccagaagacaatgaaccaagtggctatgcccaaacagattgtgtattagaagcaatggctttccttgaagaatcccatcctggtatctttgaaaactcttgtattgaaacaatggaggttgttcagcaaacaagggtggacaaactgacacaaggcagacagacctatgactggactctaaataggaaccagcctgctgcaacagcattggcaaacactatagaagtattcagatcaaacggcctcatagcaaatgaatctggaaggctaatagacttccttaaagatgtaatggagtcgatggacagagacgaagtagaggtcacaactcattttcaaagaaagaggagagtgagagacaatgtaactaaaaaaatggtgactcaaagaacaataggcaaaaagaaacataaattagacaaaagaagttacctaattagggcattaaccctgaacacaatgaccaaagatgctgagagggggaaactaaaacgcagagcaattgcaaccccaggaatgcaaataagagggtttgtatactttgttgagacactggcaagaagcatatgtgaaaagcttgaacaatcaggattgccagttggaggaaatgagaagaaagcaaagttagcaaatgttgtaaggaagatgatgaccaactcccaggacactgaaatttctttcaccatcactggagataacacaaaatggaacgaaaatcaaaaccctagaatgttcttggccatgatcacatatataaccaaaaatcagcctgaatggttcagaaatattctaagtattgctccaataatgttttcaaacaagatggcgagactaggtaaggggtacatgtttgaaagcaagagtatgaaactgagaactcaaatacctgcagagatgctagccaacatagatttgaaatatttcaatgattcaactaaaaagaaaattgaaaaaatccgaccattattaatagatggaactgcatcattgagtcctggaatgatgatgggcatgttcaatatgttgagcaccgtcttgggcgtctccattctgaatcttgggcaaaagagatacaccaagactacttactggtgggatggtcttcaatcgtctgatgattttgctttgattgtgaatgcacccaactatgcaggaattcaagctggagttgacaggttttatcgaacctgtaagctgctcggaattaatatgagcaaaaagaagtcttacataaacagaacaggtacctttgaattcacgagctttttctatcgttatgggtttgttgccaatttcagcatggagcttcctagttttggggtgtctggggtcaatgaatctgcagacatgagtattggagtcactgtcatcaaaaacaatatgataaacaatgaccttggcccagcaactgctcaaatggcccttcagttatttataaaagattacagatacacttatcgatgccacagaggtgacacacaaatacaaacccggagatcatttgagataaagaaactatgggaccaaacccgctccaaagctgggctgttggtctctgatggaggccccaatttatataacattagaaatctccatattcctgaagtttgcttgaaatgggagttgatggatgaggattaccaggggcgtttatgcaacccattaaacccgtttgtcagccataaagagattgaatcagtgaacaatgcagtgataatgccggcacatggcccagccaaaactatggagtatgacgctgttgcaacaacacactcctgggtccccaaaagaaatcgatccattttgaacacgagccaaagggggatacttgaagatgagcaaatgtatcagaggtgctgcaatttatttgaaaaattcttcccaagtagctcatacagaagaccagttggaatatccagtatggtagaggctatggtttcaagagcccgaattgatgcacggattgatttcgaatctggaaggataaagagggaggaatttgctgagatcatgaagatctgttccaccattgaagacctcagacggcaaaaatag

>H1N1_PB1_CY028145_A/Kansas/UR06-0191/2007

atggatgtcaatccgacattacttttcttaaaagtgccagcacaaaatgctataagcacaacttttccttatactggtgaccctccttacagccatgggacaggaacagggtacaccatggatacagtcaacaggacacaccagtactcagaaagaggaagatggacaaaaaataccgaaactggagcaccgcaactcaacccaattgatgggccactaccagaagacaatgaaccgagtggctatgcccaaacagattgtgtattagaagcaatggctttccttgaagaatcccatcctggtatctttgaaaactcttgtattgagacaatggaggttgttcagcaaacaagagtggacaaactgacacagggcagacagacctatgactggactctaaataggaaccagcctgctgccacagcattggcaaacactatagaagtattcagatcaaacggcctcatagcaaatgaatctgggaggctaatagacttccttaaagatgtaatggagtcgatggacatagacgaagtagaggtcacaactcattttcaaagaaagaggagagtgagagacaatgtaactaaaaaaatggtgacccaaagaacaataggcaaaaagaaacataaattagacaaaagaagttacctaattagggcattaaccctgaacacaatgaccaaagatgctgagagagggaaactaaaacgcagagcaattgcaaccccaggaatgcaaataagggggtttgtatactttgttgagacactggcaagaagcatatgtgaaaagcttgaacaatcaggattgccagttggaggaaatgagaagaaagcaaagttagcaaatgttgtaaggaagatgatgaccaactcccaggacactgaaatttctttcaccatcactggagataacacaaaatggaacgaaaatcaaaaccctagaatgttcttggccatgatcacatatataaccaaaaatcagcctgaatggttcagaaatattctaagtattgccccaataatgttttcaaacaagatggcgagactaggtaaggggtacatgtttgaaagcaagagtatgaaactgagaactcaaatacctgcagagatgctagccaacatagatttgaaatatttcaatgattcaactaaaaagaaaattgaaaaaatccgaccattattaatagatggaactgcatcattgagtcctggaatgatgatgggcatgttcaatatgttgagcaccgtcttgggcgtctccattctgaatcttgggcaaaagagatacaccaagactacttactggtgggatggtcttcaatcgtctgatgattttgctttgattgtgaatgcacccaactatgcaggaattcaagctggagttgacaggttttatcgaacctgtaagctgctcggaattaatatgagcaaaaagaagtcttacataaacagaacaggtacctttgaattcacgagctttttctatcgttatgggtttgttgccaatttcagcatggagcttcctagttttggggtgtctggggtcaatgaatctgcagacatgagtattggagtcactgtcatcaaaaacaatatgataaacaatgaccttggcccagcaactgctcaaatggcccttcagttatttataaaagattacaggtacacttatcgatgccacagaggtgacacacaaatacaaacccggagatcatttgagataaagaaactatgggaccaaacccgctctaaagctgggctgttggtctctgatggaggccccaatttatataacattagaaatctccatattcctgaagtttgcttgaaatgggagttgatggatgaggattatcaggggcgtttatgcaacccattaaacccgtttgtcagccataaagagattgaatcagtaaacaatgcagtgataatgccggcacatggtccagccaaaaatatggagtatgacgctgttgcaacaacacactcctgggtccccaaaagaaatcgatccattttgaacacgagccaacgggggatacttgaagatgagcaaatgtatcagaggtgctgcaatttatttgaaaaattcttcccaagtagctcatacagaagaccagttggaatatccagtatggtagaggctatggtttcaagagcccgaattgatgcacggattgatttcgaatctggaaggataaagaaagaggaatttgctgagatcatgaagatctgttccaccattgaagacctcagacggcaaaaatag

>H1N1_PB1_CY038933_A/Managua/4537.03/2008

atggatgtcaatccgacattacttttcttaaaagtgccagcacaaaatgctataagcacaacttttccttatactggtgaccctccttacagccatgggacaggaacagggtacaccatggatacagtcaacaggacacaccagtactcagaaagaggaagatggacaaaaaataccgaaactggagcaccgcaacttaacccaattgatggtccactaccggaagacaatgaaccaagtggctatgcccaaacagattgtgtattagaagcaatggctttccttgaagaatcccatcccggtatctttgaaaactcttgtattgaaacaatggaggttgttcagcaaacaagggtggacaaactgacacaaggcagacagacctatgactggactctaaataggaaccagcctgctgccacagcattggcaaacactatagaagtattcagatcaaacggcctcatagccaatgaatctggaaggctaatagacttccttaaagatgtaatggagtcgatggacagaggcgaagtagaggtcacaactcattttcaaagaaagaggagagtgagagacaatgtaactaaaaaaatggtgacccaaagaacaataggcaaaaagaaacataaattagacaaaagaagttacctaattagggcattaaccctgaacacaatgaccaaagatgctgagagggggaaactaaaacgcagagcaattgcaaccccaggaatgcaaataagggggtttgtatactttgttgagacactggcaagaagcatatgtgaaaagcttgaacaatcaggattgccagttggagggaatgagaagaaagcaaagttagcaaatgttgtaaggaagatgatgaccaactcccaggacactgaaatttctttcaccataaccggagataacacaaaatggaacgaaaatcaaaaccctagaatgttcttggccatgatcacatatataaccaaaaatcagcctgaatggttcagaaatattctaagtattgctccaataatgttttcaaacaagatggcgagactaggtaaggggtacatgtttgaaagcaagagtatgaaactgagaactcaaatacctgcagagatgctagccaacatagatttgaaatatttcaatgattcaactaaaaagaaaattgaaaaaatccgaccattattaatagatggaactgcatcattgagtcctggaatgatgatgggcatgttcaatatgttgagcaccgtcttgggcgtctccattctgaatcttgggcaaaagagatacaccaagactacttactggtgggatggtcttcaatcgtctgatgattttgctttgattgtgaatgcacccaactatgcaggaattcaagctggagttgacaggttttatcgaacctgtaagctgctcggaattaatatgagcaaaaagaagtcttacataaacagaacaggtacctttgaattcacgagctttttctatcgttatgggtttgttgccaatttcagcatggagcttcctagttttggggtgtctggggtcaatgaatctgcagacatgagtattggagtcactgtcatcaaaaacaatatgataaacaatgaccttggcccagcaactgctcaaatggcccttcagttatttataaaagattacaggtacacttatcgatgccaccggggtgacacacaaatacaaacccggagatcgtttgagataaagaaactatgggaccaaacccgctcaaaagctgggctgttggtctctgatggaggccccaatttatataacattagaaatctccatattcctgaagtttgcctgaaatgggagttgatggatgaggattaccaggggcgtttatgcaacccattaaacccgtttgtcagccataaagagattgaatcagtgaacaatgcagtgataatgccggcacatggtccagccaaaaatatggagtatgacgctgttgcaacaacacactcctgggtccccaaaagaaatcgatccattttgaacacgagccaaagggggatacttgaagatgagcaaatgtatcagaggtgctgcaatttatttgaaaaattcttcccaagtagctcatacagaagaccagttggaatatccagtatggtagaggctatggtctcaagagcccgaattgatgcacggattgatttcgaatctggaaggataaagaaagaggaatttgctgagatcatgaagatctgttccaccattgaagacctcagacggcaaaaatga

>H1N1_PB1_CY040272_A/Managua/309.01/2008

atggatgtcaatccgacattacttttcttaaaagtgccagcacaaaatgctataagcacaacttttccttatactggtgaccctccttacagccatgggacaggaacagggtacaccatggatacagtcaacaggacacaccagtactcagaaagaggaagatggacaaaaaataccgaaactggagcaccgcaacttaacccaattgatggtccactaccggaagacaatgaaccaagtggctatgcccaaacagattgtgtattagaagcaatggctttccttgaagaatcccatcccggtatctttgaaaactcttgtattgaaacaatggaggttgttcagcaaacaagggtggacaaactgacacaaggcagacagacctatgactggactctaaataggaaccagcctgctgccacagcattggcaaacactatagaagtattcagatcaaacggcctcatagccaatgaatctggaaggctaatagacttccttaaagatgtaatggagtcgatggacagaggcgaagtagaggtcacaactcattttcaaagaaagaggagagtgagagacaatgtaactaagaaaatggtgacccaaagaacaataggcaaaaagaaacataaattagacaaaagaagttacctaattagggcattaaccctgaacacaatgaccaaagatgctgagagggggaaactaaaacgcagagcaattgcaaccccaggaatgcaaataagggggtttgtatactttgttgagacactggcaagaagcatatgtgaaaagcttgaacaatcaggattgccagttggagggaatgagaagaaagcaaagttagcaaatgttgtaaggaagatgatgaccaactcccaggacactgaaatttctttcaccataaccggagataacacaaaatggaacgaaaatcaaaaccctagaatgttcttggccatgatcacatatataaccaaaaatcagcctgaatggttcagaaatattctaagtattgctccaataatgttttcaaacaagatggcgagactaggtaaggggtacatgtttgaaagcaagagtatgaaactgagaactcaaatacctgcagagatgctagccaacatagatttgaaatatttcaatgattcaactaaaaagaaaattgaaaaaatccgaccattattaatagatggaactgcatcattgagtcctggaatgatgatgggcatgttcaatatgttgagcaccgtcttgggcgtctccattctgaatcttgggcaaaagagatacaccaagactacttactggtgggatggtcttcaatcgtctgatgattttgctttgattgtgaatgcacccaactatgcaggaattcaagctggagttgacaggttttatcgaacctgtaagctgctcggaattaatatgagcaaaaagaagtcttacataaacagaacaggtacctttgaattcacgagctttttctatcgttatgggtttgttgccaatttcagcatggagcttcctagttttggggtgtctggggtcaatgaatctgcagacatgagtattggagtcactgtcatcaaaaacaatatgataaacaatgaccttggcccagcaactgctcaaatggcccttcagttatttataaaagattacaggtacacttatcgatgccaccggggtgacacacaaatacaaacccggagatcgtttgagataaagaaactatgggaccaaacccgctcaaaagctgggctgttggtctctgatggaggccccaatttatataacattagaaatctccatattcctgaagtttgcctgaaatgggagttgatggatgaggattaccaggggcgtttatgcaacccattaaacccgtttgtcagccataaagagattgaatcagtgaacaatgcagtgataatgccggcacatggtccagccaaaaatatggagtatgacgctgttgcaacaacacactcctgggtccccaaaagaaatcgatccattttgaacacgagccaaagggggatacttgaagatgagcaaatgtatcagaggtgctgcaatttatttgaaaaattcttcccaagtagctcatacagaagaccagttggaatatccagtatggtagaggctatggtctcaagagcccgaattgatgcacggattgatttcgaatctggaaggataaagaaagaggaatttgctgagatcatgaagatctgttccaccattgaagacctcagacggcaaaaatga

>H1N1_PB1_GQ222421_A/Paris/2580/2009

atggatgtcaatccgactttacttttcctaaaaattccagcgcaaaatgccataagcaccacattcccttatactggagatcctccatacagccatggaacaggaacaggatacaccatggacacagtaaacagaacacaccaatactcagaaaagggaaagtggacgacaaacacagagactggtgcaccccagctcaacccgattgatggaccactacctgaggataatgaaccaagtgggtatgcacaaacagactgtgttctagaggctatggctttccttgaagaatcccacccgggaatatttgagaattcatgccttgaaacaatggaacttgttcaacaaacaagggtagataaactaactcaaggtcgccagacttatgattggacattaaacagaaatcaaccggcagcaactgcattggccaacaccatagaagtctttagatcgaatggcctaacagctaatgagtcaggaaggctaatagatttcttaaaggatgtaatggaatcaatgaacaaagaggaaatagagataacaacccactttcaaagaaaaaggagagtaagagacaacatgaccaagaagatggtcacgcaaagaacaatagggaagaaaaaacaaagactgaataagagaggctatctaataagagcactgacattaaatacgatgaccaaagatgcagagagaggcaagttaaaaagaagggctatcgcaacacctgggatgcagattagaggtttcgtatactttgttgaaactttagctaggagcatttgcgaaaagcttgaacagtctgggctcccagtagggggcaatgaaaagaaggccaaactggcaaatgttgtgagaaagatgatgactaattcacaagacacagagatttctttcacaatcactggggacaacactaagtggaatgaaaatcaaaatcctcgaatgttcctggcgatgattacatatatcaccagaaatcaacccgagtggttcagaaacatcctgagcatggcacccataatgttctcaaacaaaatggcaagactagggagagggtacatgttcgagagtaaaagaatgaagattcgaacacaaataccagcagaaatgctagcaagcattgacctgaagtacttcaatgaatcaacaaagaagaaaattgagaaaataaggcctcttctaatagatggcacagcatcactgagtcctgggatgatgatgggcatgttcaacatgctaagtacggtcttgggagtctcgatactgaatcttggacaaaagaaatacaccaagacaatatactggtgggatgggctccaatcatccgacgattttgctctcatagtgaatgcaccaaaccatgagggaatacaagcaggagtggacagattctacaggacctgcaagttagtgggaatcaacatgagcaaaaagaagtcctatataaataagacagggacatttgaattcacaagctttttttatcgctatggatttgtggctaattttagcatggagctacccagctttggagtgtctggagtaaatgaatcagctgacatgagtattggagtaacagtgataaagaacaacatgataaacaatgaccttggacctgcaacggcccagatggctcttcaattgttcatcaaagactacagatacacatataggtgccataggggagacacacaaattcagacgagaagatcatttgagttaaagaagctgtgggatcaaacccaatcaaaggtagggctattagtatcagatggaggaccaaacttatacaatatacggaatcttcacattcctgaagtctgcttaaaatgggagctaatggatgatgattatcggggaagactttgtaatcccctgaatccctttgtcagtcataaagagattgattctgtaaacaatgctgtggtaatgccagcccatggtccagccaaaagcatggaatatgatgccgttgcaactacacattcctggattcccaagaggaatcgttctattctcaacacaagccaaaggggaattcttgaggatgaacagatgtaccagaagtgctgcaatctattcgagaaatttttccctagcagttcatataggagaccggttggaatttctagcatggtggaggccatggtgtctagggcccggattgatgccagggtcgacttcgagtctggacggatcaagaaagaagagttctctgagatcatgaagatctgttccaccattgaagaactcagacggcaaaaataa

>H1N1_PB1_CY040756_A/New York/3243/2009

atggatgtcaatccgactctacttttcctaaaaattccagcgcaaaatgccataagcaccacattcccttatactggagatcctccatacagccatggaacaggaacaggatacaccatggacacagtaaacagaacacaccaatactcagaaaagggaaagtggacgacaaacacagagactggtgcaccccagctcaacccgattgatggaccactacctgaggataatgaaccaagtgggtatgcacaaacagactgtgttctagaggctatggctttccttgaagaatcccacccaggaatatttgagaattcatgccttgaaacaatggaagttgttcaacaaacaagggtagataaactaactcaaggtcgccagacttatgattggacattaaacagaaatcaaccggcagcaactgcattggccaacaccatagaagtctttagatcgaatggcctaacagctaatgagtcaggaaggctaatagatttcttaaaggatgtaatggaatcaatgaacaaagaggaaatagagataacaacccactttcaaagaaaaaggagagtaagagacaacatgaccaagaagatggtcacgcaaagaacaatagggaagaaaaaacaaagactgaataagagaggctatctaataagagcactgacattaaatacgatgaccaaagatgcagagagaggcaagttaaaaagaagggctatcgcaacacctgggatgcagattagaggtttcgtatactttgttgaaactttagctaggagcatttgcgaaaagcttgaacagtctgggctcccagtagggggcaatgaaaagaaggccaaactggcaaatgttgtgagaaagatgatgactaattcacaagacacagagatttctttcacaatcactggggacaacactaagtggaatgaaaatcaaaatcctcgaatgttcctggcgatgattacatatatcaccagaaatcaacccgagtggttcagaaacatcctgagcatggcacccataatgttctcaaacaaaatggcaagactagggaaagggtacatgttcgagagtaaaagaatgaagattcgaacacaaataccagcagaaatgctagcaagcattgacctgaagtacttcaatgaatcaacaaagaagaaaattgagaaaataaggcctcttctaatagatggcacagcatcactgagtcctgggatgatgatgggcatgttcaacatgctaagtacggtcttgggagtctcgatactgaatcttggacaaaagaaatacaccaagacaatatactggtgggatgggctccaatcatccgacgattttgctctcatagtgaatgcaccaaaccatgagggaatacaagcaggagtggacagattctacaggacctgcaagttagtgggaatcaacatgagcaaaaagaagtcctatataaataagacagggacatttgaattcacaagctttttttatcgctatggatttgtggctaattttagcatggagctacccagctttggagtgtctggagtaaatgaatcagctgacatgagtattggagtaacagtgataaagaacaacatgataaacaatgaccttggacctgcaacggcccagatggctcttcaattgttcatcaaagactacagatacacatataggtgccataggggagacacacaaattcagacgagaagatcatttgagttaaagaagctgtgggatcaaacccaatcaaaggtagggctattagtatcagatggaggaccaaacttatacaatatacggaatcttcacattcctgaagtctgcttaaaatgggagctaatggatgatgattatcggggaagactttgtaatcccctgaatccctttgtcagtcataaagagattgattctgtaaacaatgctgtggtaatgccagcccatggtccagccaaaagcatggaatatgatgccgttgcaactacacattcctggattcccaagaggaatcgttctattctcaacacaagccaaaggggaattcttgaggatgaacagatgtaccagaagtgctgcaatctattcgagaaatttttccctagcagttcatataggagaccggttggaatttctagcatggtggaggccatggtgtctagggcccggattgatgccagggtcgacttcgagtctggacggatcaagaaagaagagttctctgagatcatgaagatctgttccaccattgaagaactcagacggcaaaaataa

>H1N1_PB1_CY041144_A/New York/3258/2009

atggatgtcaatccgactctacttttcctaaaaattccagcgcaaaatgccataagcaccacattcccttatactggagatcctccatacagccatggaacaggaacaggatacaccatggacacagtaaacagaacacaccaatactcagaaaagggaaagtggacgacaaacacagagactggtgcaccccagctcaacccgattgatggaccactacctgaggataatgaaccaagtgggtatgcacaaacagactgtgttctagaggctatggctttccttgaagaatcccacccaggaatatttgagaattcatgccttgaaacaatggaagttgttcaacaaacaagggtagataaactaactcaaggtcgccagacttatgattggacattaaacagaaatcaaccggcagcaactgcattggccaacaccatagaagtctttagatcgaatggcctaacagctaatgagtcaggaaggctaatagatttcttaaaggatgtaatggaatcaatgaacaaagaggaaatagagataacaacccactttcaaagaaaaaggagagtaagagacaacatgaccaagaagatggtcacgcaaagaacaatagggaagaaaaaacaaagactgaataagagaggctatctaataagagcactgacattaaatacgatgaccaaagatgcagagagaggcaagttaaaaagaagggctatcgcaacacctgggatgcagattagaggtttcgtatactttgttgaaactttagctaggagcatttgygaaaagcttgaacagtctgggctcccagtagggggcaatgaaaagaaggccaaactggcaaatgttgtgagaaagatgatgactaattcacaagacacagagatttctttcacaatcactggggacaacactaagtggaatgaaaatcaaaatcctcgaatgttcctggcgatgattacatatatcaccagaaatcaacccgagtggttcagaaacatcctgagcatggcacccataatgttctcaaacaaaatggcaagactagggaaagggtacatgttcgagagtaaaagaatgaagattcgaacacaaataccagcagaaatgctagcaagcattgacctgaagtacttcaatgaatcaacaaagaagaaaattgagaaaataaggcctcttctaatagatggcacagcatcactgagtcctgggatgatgatgggcatgttcaacatgctaagtacggtcttgggagtctcgatactgaatcttggacaaaagaaatacaccaagacaatatactggtgggatgggctccaatcatccgacgattttgctctcatagtgaatgcaccaaaccatgagggaatacaagcaggagtggacagattctacaggacctgcaagttagtgggaatcaacatgagcaaaaagaagtcctatataaataagacagggacatttgaattcacaagctttttttatcgctatggatttgtggctaattttagcatggagctacccagctttggagtgtctggagtaaatgaatcagctgacatgagtattggagtaacagtgataaagaacaacatgataaacaatgaccttggacctgcaacggcccagatggctcttcaattgttcatcaaagactacagatacacatataggtgccataggggagacacacaaattcagacgagaagatcatttgagttaaagaagctgtgggatcaaacccaatcaaaggtagggctattagtatcagatggaggaccaaacttatacaatatacggaatcttcacattcctgaagtctgcttaaaatgggagctaatggatgatgattatcggggaagactttgtaatcccctgaatccctttgtcagtcataaagagattgattctgtaaacaatgctgtggtaatgccagcccatggtccagccaaaagcatggaatatgatgccgttgcaactacacattcctggattcccaagaggaatcgttctattctcaacacaagccaaaggggaattcttgaggatgaacagatgtaccagaagtgctgcaatctattcgagaaatttttccctagcagttcatataggagaccggttggaatttctagcatggtggaggccatggtgtctagggcccggattgatgccagggtcgacttcgagtctggacggatcaagaaagaagagttctctgagatcatgaagatctgttccaccattgaagaactcagacggcaaaaataa

>H1N1_PB1_GQ385298_A/Toronto/0462/2009

atggatgtcaatccgactctacttttcctaaaaattccagcgcaaaatgccataagcaccacattcccttatactggagatcctccatacagccatggaacaggaacaggatacaccatggacacagtaaacagaacacaccaatactcagaaaagggaaagtggacgacaaacacagagactggtgcaccccagctcaacccgattgatggaccactacctgaggataatgaaccaagtgggtatgcacaaacagactgtgttctagaggctatggctttccttgaagaatcccacccaggaatatttgagaattcatgccttgaaacaatggaagttgttcaacaaacaagggtagataaactaactcaaggtcgccagacttatgattggacattaaacagaaatcaaccggcagcaactgcattggccaacaccatagaagtctttagatcgaatggcctaacagctaatgagtcaggaaggctaatagatttcttaaaggatgtaatggaatcaatgaacaaagaggaaatagagataacaacccactttcaaagaaaaaggagagtaagagacaacatgaccaagaagatggtcacgcaaagaacaatagggaagaaaaaacaaagactgaataagagaggctatctaataagagcactgacattaaatacgatgaccaaagatgcagagagaggcaagttaaaaagaagggctatcgcaacacctgggatgcagattagaggtttcgtatactttgttgaaactttagctaggagcatttgcgaaaagcttgaacagtctgggctcccagtagggggcaatgaaaagaaggccaaactggcaaatgttgtgagaaagatgatgactaattcacaagacacagagatttctttcacaatcactggggacaacactaagtggaatgaaaatcaaaatcctcgaatgttcctggcgatgattacatatatcaccagaaatcaacccgagtggttcagaaacatcctgagcatggcacccataatgttctcaaacaaaatggcaagactagggagagggtacatgttcgagagtaaaagaatgaagattcgaacacaaataccagcagaaatgctagcaagcattgacctgaagtacttcaatgaatcaacaaagaagaaaattgagaaaataaggcctcttctaatagatggcacagcatcactgagtcctgggatgatgatgggcatgttcaacatgctaagtacggtcttgggagtctcgatactgaatcttggacaaaagaaatacaccaagacaatatactggtgggatgggctccaatcatccgacgattttgctctcatagtgaatgcaccaaaccatgagggaatacaagcaggagtggacagattctacaggacctgcaagttagtgggaatcaacatgagcaaaaagaagtcctatataaataagacagggacatttgaattcacaagctttttttatcgctatggatttgtggctaattttagcatggagctacccagctttggagtgtctggagtaaatgaatcagctgacatgagtattggagtaacagtgataaagaacaacatgataaacaatgaccttggacctgcaacggcccagatggctcttcaattgttcatcaaagactacagatacacatataggtgccataggggagacacacaaattcagacgagaagatcatttgagttaaagaagctgtgggatcaaacccaatcaaaggtagggctattagtatcagatggaggaccaaacttatacaatatacggaatcttcacattcctgaagtctgcttaaaatgggagctaatggatgatgattatcggggaagactttgtaatcccctgaatccctttgtcagtcataaagagattgattctgtaaacaatgctgtggtaatgccagcccatggtccagccaaaagcatggaatatgatgccgttgcaactacacattcctggattcccaagaggaatcgttctattctcaacacaagccaaaggggaattcttgaggatgaacagatgtaccagaagtgctgcaatctattcgagaaatttttccctagcagttcatataggagaccggttggaatttctagcatggtggaggccatggtgtctagggcccggattgatgccagggtcgacttcgagtctggacggatcaagaaagaagagttctctgagatcatgaagatctgttccaccattgaagaactcagacggcaaaaataa

>H1N1_PB1_CY052613_A/Texas/45034157/2009

atggatgtcaatccgactctacttttcctaaaaattccagcgcaaaatgccataagcaccacattcccttatactggagatcctccatacagccatggaacaggaacaggatacaccatggacacagtaaacagaacacaccaatactcagaaaagggaaagtggacgacaaacacagagactggtgcaccccagctcaacccgattgatggaccactacctgaggataatgaaccaagtgggtatgcacaaacagactgtgttctagaggctatggctttccttgaagaatcccacccaggaatatttgagaattcatgccttgaaacaatggaagttgttcaacaaacaagggtagataaactaactcaaggtcgccagacttatgattggacattaaacagaaatcaaccggcagcaactgcattggccaacaccatagaagtctttagatcgaatggcctaacagctaatgagtcaggaaggctaatagatttcttaaaggatgtaatggaatcaatgaataaagaggaaatagagataacaacccactttcaaagaaaaaggagagtaagagacaacatgaccaagaagatggtcacgcaaagaacaatagggaagaaaaaacaaagactgaataagagaggctatctaataagagcactgacattaaatacgatgaccaaagatgcagagagaggcaagttaaaaagaagggctatcgcaacacctgggatgcagattagaggtttcgtatactttgttgaaactttagctaggagcatttgcgaaaagcttgaacagtctgggctcccagtagggggcaatgaaaagaaggccaaactggcaaatgttgtgagaaagatgatgactaattcacaagacacagagatttctttcacaatcactggggacaacactaagtggaatgaaaatcaaaatcctcgaatgttcctggcgatgattacatatatcaccagaaatcaacccgagtggttcagaaacatcctgagcatggcacccataatgttctcaaacaaaatggcaagactagggaaagggtacatgttcgagagtaaaagaatgaagattcgaacacaaataccagcagaaatgctagcaagcattgacctgaagtacttcaatgaatcaacaaagaagaaaattgagaaaataaggcctcttctaatagatggcacagcatcactgagtcctgggatgatgatgggcatgttcaacatgctaagtacggtcttgggagtctcgatactgaatcttggacaaaagaaatacaccaagacaatatactggtgggatgggctccaatcatccgacgattttgctctcatagtgaatgcaccaaaccatgagggaatacaagcaggagtggacagattctacaggacctgcaagttagtgggaatcaacatgagcaaaaagaagtcctatataaataagacagggacatttgaattcacaagctttttttatcgctatggatttgtggctaattttagcatggagctacccagctttggagtgtctggagtaaatgaatcagctgacatgagtattggagtaacagtgataaagaacaacatgataaacaatgaccttggacctgcaacggcccagatggctcttcaattgttcatcaaagactacagatacacatataggtgccataggggagacacacaaattcagacgagaagatcatttgagttaaagaagctgtgggatcaaacccaatcaaaggtagggctattagtatcagatggaggaccaaacttatacaatatacggaatcttcacattcctgaagtctgcttaaaatgggagctaatggatgatgattatcggggaagactttgtaatcccctgaatccctttgtcagtcataaagagattgattctgtaaacaatgctgtggtaatgccagcccatggtccagccaaaagcatggaatatgatgccgttgcaactacacattcctggattcccaagaggaatcgttctattctcaacacaagccaaaggggaattcttgaggatgaacagatgtaccagaagtgctgcaatctattcgagaaatttttccctagcagttcatataggagaccggttggaatttctagcatggtggaggccatggtgtctagggcccggattgatgccagggtcgacttcgagtctggacggatcaagaaagaagagttctctgagatcatgaagatctgttccaccattgaagaactcagacggcaaaaataa

>H1N1_PB1_CY055516_A/California/VRDL76/2009

atggatgtcaatccgactctacttttcctaaaaattccagcgcaaaatgccataagcaccacattcccttatactggagatcctccatacagccatggaacaggaacaggatacaccatggacacagtaaacagaacacaccaatactcagaaaagggaaagtggacgacaaacacagagactggtgcaccccagctcaacccgattgatggaccactacctgaggataatgaaccaagtgggtatgcacaaacagactgtgttctagaggctatggctttccttgaagaatcccacccaggaatatttgagaattcatgccttgaaacaatggaagttgttcaacaaacaagggtagataaactaactcaaggtcgccagacttatgattggacattaaacagaaatcaaccggcagcaactgcattggccaacaccatagaagtctttagatcgaatggcctaacagctaatgagtcaggaaggctaatagatttcttaaaggatgtaatggaatcaatgaacaaagaggaaatagagataacaacccactttcaaagaaaaaggagagtaagagacaacatgaccaagaagatggtcacgcaaagaacaatagggaagaaaaaacaaagactgaataagagaggctatctaataagagcactgacattaaatacgatgaccaaagatgcagagagaggcaagttaaaaagaagggctatcgcaacacctgggatgcagattagaggtttcgtatactttgttgaaactttagctaggagcatttgcgaaaagcttgaacagtctgggctcccagtagggggcaatgaaaagaaggccaaactggcaaatgttgtgagaaagatgatgactaattcacaagacacagagatttctttcacaatcactggggacaacactaagtggaatgaaaatcaaaatcctcgaatgttcctggcgatgattacatatatcaccagaaatcaacccgagtggttcagaaacatcctgagcatggcacccataatgttctcaaacaaaatggcaagactagggaaagggtacatgttcgagagtaaaagaatgaagattcgaacacaaataccagcagaaatgctagcaagcattgacctgaagtacttcaatgaatcaacaaagaagaaaattgagaaaataaggcctcttctaatggatggcacagcatcactgagtcctgggatgatgatgggcatgttcaacatgctaagtacggtcttgggagtctcgatactgaatcttggacaaaagaaatacaccaagacaatatactggtgggatgggctccaatcatccgacgattttgctctcatagtgaatgcaccaaaccatgagggaatacaagcaggagtggacagattctacaggacctgcaagttagtgggaatcaacatgagcaaaaagaagtcctatataaataagacagggacatttgaattcacaagctttttttatcgctatggatttgtggctaattttagcatggagctacccagctttggagtgtctggagtaaatgaatcagctgacatgagtattggagtaacagtgataaagaacaacatgataaacaatgaccttggacctgcaacggcccagatggctcttcaactgttcatcaaagactacagatacacatataggtgccataggggagacacacaaattcagacgagaagatcatttgagttaaagaagctgtgggatcaaacccaatcaaaggtagggctattagtatcagatggaggaccaaacttatacaatatacggaatcttcacattcctgaagtctgcttaaaatgggagctaatggatgatgattatcggggaagactttgtaatcccctgaatccctttgtcagtcataaagagattgattctgtaaacaatgctgtggtaatgccagcccatggtccagccaaaagcatggaatatgatgccgttgcaactacacattcctggattcccaagaggaatcgttctattctcaacacaagccaaaggggaattcttgaggatgaacagatgtaccagaagtgctgcaatctattcgagaaatttttccctagcagttcatataggagaccggttggaatttctagcatggtggaggccatggtgtctagggcccggattgatgccagggtcgacttcgagtctggacggatcgggaaggaagagttctctgagatcatgaagatctgttccaccattgaagaactcagacggcaaaaataa

>H1N1_PB1_CY055682_A/Australia/31/2009

atggatgtcaatccgactctacttttcctaaaaattccagcgcaaaatgccataagcaccacattcccttatactggagatcctccatacagccatggaacaggaacaggatacaccatggacacagtaaacagaacacaccaatactcagaaaagggaaagtggacgacaaacacagagactggtgcaccccagctcaacccgattgatggaccactacctgaggataatgaaccaagtgggtatgcacaaacagactgtgttctagaggctatggctttccttgaagaatcccacccaggaatatttgagaattcatgccttgaaacaatggaagttgttcaacaaacaagggtagataaactaactcaaggtcgccagacttatgattggacattaaacagaaatcaaccggcagcaactgcattggccaacaccatagaagtttttagatcgaatggcctaacagctaatgagtcaggaaggctaatagatttcttaaaggatgtaatggaatcaatgaacaaagaggaaatagagataacaacccactttcaaagaaaaaggagagtaagagacaacatgaccaagaagatggtcacgcaaaggacaatagggaagaaaaaacaaagactgaataagagaggctatctaataagagcactgacattaaatacgatgaccaaagatgcagagagaggcaagttaaaaagaagggctatcgcaacacctgggatgcagattagaggtttcgtatactttgttgaaactttagctaggagcatttgcgaaaagcttgaacagtctgggctcccagtagggggcaatgaaaagaaggccaaactggcaaatgttgtgagaaagatgatgactaattcacaagacacagagatttctttcacaatcactggggacaacactaagtggaatgaaaatcaaaatcctcgaatgttcctggcgatgattacatatatcaccagaaatcaacccgagtggttcagaaacatcctgagcatggcacccataatgttctcaaacaaaatggcaagactagggaaagggtacatgttcgagagtaaaagaatgaagattcgaacacaaataccagcagaaatgctagcaagcattgacctgaagtacttcaatgaatcaacaaagaagaaaattgagaaaataaggcctcttctaatagatggcacagcatcactgagtcctgggatgatgatgggcatgttcaacatgctaagtacggtcttgggagtctcgatactgaatcttggacaaaagaaatacaccaagacaatatactggtgggatgggctccaatcatccgacgattttgctctcatagtgaatgcaccaaaccatgagggaatacaagcaggagtggacagattctacaggacctgcaagttagtgggaatcaacatgagcaaaaagaagtcctatataaataagacagggacatttgaattcacaagctttttttatcgctatggatttgtggctaattttagcatggagctacccagctttggagtgtctggagtaaatgaatcagctgacatgagtattggagtaacagtgataaagaacaacatgataaacaatgaccttggacctgcaacggcccagatggctcttcaattgttcatcaaagactacagatacacatataggtgccataggggagacacacaaattcagacgagaagatcatttgagttaaagaagctgtgggatcaaacccaatcaaaggtagggctattagtatcagatggaggaccaaacttatacaatatacggaatcttcacattcctgaagtctgcttaaaatgggagctaatggatgatgattatcggggaagactttgtaatcccctgaatccctttgtcagtcataaagagattgattctgtaaacaatgctgtggtaatgccagcccatggtccagccaaaagcatggaatatgatgccgttgcaactacacattcctggattcccaagaggaatcgttctattctcaacacaagccaaaggggaattcttgaggatgaacagatgtaccagaagtgctgcaatctattcgagaaatttttccctagcagttcatataggagaccggttggaatttctagcatggtggaggccatggtgtctagggcccggattgatgccaggatcgacttcgagtctggacggatcaagaaagaagagttctctgagatcatgaagatctgttccaccattgaagaactcagacggcaaaaataa

>H1N1_PB1_CY055730_A/Australia/40/2009

atggatgtcaatccgactctacttttcctaaaaattccagcgcaaaatgccataagcaccacattcccttatactggagatcctccatacagccatggaacaggaacaggatacaccatggacacagtaaacagaacacaccaatactcagaaaagggaaagtggacgacaaacacagagactggtgcactccagctcaacccgattgatggaccactacctgaggataatgaaccaagtgggtatgcacaaacagactgtgttctagaggctatggctttccttgaagaatcccacccaggaatatttgagaattcatgccttgaaacaatggaagttgttcaacaaacaagggtagataaactaactcaaggtcgccagacttatgattggacattaaacagaaatcaaccggcagcaactgcattggccaacaccatagaagtttttagatcgaatggcctaacagctaatgagtcaggaaggctaatagatttcttaaaggatgtaatggaatcaatgaacaaagaggaaatagagataacaacccactttcaaagaaaaaggagagtaagagacaacatgaccaagaagatggtcacgcaaagaacaatagggaagaaaaaacaaagactgaataagagaggctatctaataagagcactgacattaaatacgatgaccaaagatgcagagagaggcaagttaaaaagaagggctatcgcaacacctgggatgcagattagaggtttcgtatactttgttgaaactttagctaggagcatttgcgaaaagcttgaacagtctgggctcccagtagggggcaatgaaaagaaggccaaactggcaaatgttgtgagaaagatgatgactaattcacaagacacagagatttctttcacaatcactggggacaacactaagtggaatgaaaatcaaaatcctcgaatgttcctggcgatgattacatatatcaccagaaatcaacccgagtggttcagaaacatcctgagcatggcacccataatgttctcaaacaaaatggcaagactagggaaagggtacatgttcgagagtaaaagaatgaagattcgaacacaaataccagcagaaatgctagcaagcattgacctgaagtacttcaatgaatcaacaaagaagaaaattgagaaaataaggcctcttctaatagatggcacagcatcactgagtcctgggatgatgatgggcatgttcaacatgctaagtacggtcttgggagtctcgatactgaatcttggacaaaagaaatacaccaagacaatatactggtgggatgggctccaatcatccgacgattttgctctcatagtgaatgcaccaaaccatgagggaatacaagcaggagtggacagattctacaggacctgcaagttagtgggaatcaacatgagcaaaaagaagtcctatataaataagacagggacatttgaattcacaagctttttttatcgctatggatttgtggctaattttagcatggagctacccagctttggagtgtctggagtaaatgaatcagctgacatgagtattggagtaacagtgataaagaacaacatgataaacaatgaccttggacctgcaacggcccagatggctcttcaattgttcatcaaagactacagatacacatataggtgccataggggagacacacaaattcagacgagaagatcatttgagttaaagaagctgtgggatcaaacccaatcaaaggtagggctattagtatcagatggaggaccaaacttatacaatatacggaatcttcacattcctgaagtctgcttaaaatgggagctaatggatgatgattatcggggaagactttgtaatcccctgaatccctttgtcagtcataaagagattgattctgtaaacaatgctgtggtaatgccagcccatggtccagccaaaagcatggaatatgatgccgttgcaactacacattcctggattcccaagaggaatcgttctattctcaacacaagccaaaggggaattcttgaggatgaacagatgtaccagaagtgctgcaatctattcgagaaatttttccctagcagttcatataggagaccggttggaatttctagcatggtggaggccatggtgtctagggcccggattgatgccagggtcgacttcgagtctggacggatcaagaaagaagagttctctgagatcatgaagatctgttccaccattgaagaactcagacggcaaaaataa

>H1N1_PB1_CY069632_A/Singapore/478/2009

atggatgtcaatccgactctacttttcctaaaaattccagcgcaaaatgccataagcaccacattcccttatactggagatcctccatacagccatggaacaggaacaggatacaccatggacacagtaaacagaacacaccaatactcagaaaagggaaagtggacgacaaacacagagactggtgcaccccagctcaacccgattgatggaccactacctgaggataatgaaccaagtgggtatgcacaaacagactgtgttctagaggctatggctttccttgaagaatcccacccaggaatatttgagaattcatgccttgaaacaatggaagttgttcaacaaacaagggtagataaactaactcaaggtcgccagacttatgattggacattaaacagaaatcaaccggcagcaactgcattggccaacaccatagaagtctttagatcgaatggcctaacagctaatgagtcaggaaggctaatagatttcttaaaggatgtaatggaatcaatgaacaaagaggaaatagagataacaacccactttcaaagaaaaaggagagtaagagacaacatgaccaagaagatggtcacgcaaagaacaatagggaagaaaaaacaaagactgaataagagaggctatctaataagagcactgacattaaatacgatgaccaaagatgcagagagaggcaagttaaaaagaagggctatcgcaacacctgggatgcagattagaggtttcgtatactttgttgaaactttagctaggagcatttgcgaaaagcttgaacagtctgggctcccagtagggggcaatgaaaagaaggccaaactggcaaatgttgtgagaaagatgatgactaattcacaagacacagagatttctttcacaatcactggggacaacactaagtggaatgaaaatcaaaatcctcgaatgttcctggcgatgattacatatatcaccagaaatcaacccgagtggttcagaaacatcctgagcatggcacccataatgttctcaaacaaaatggcaagactagggaaagggtacatgttcgagagtaaaagaatgaagattcgaacacaaataccagcagaaatgctagcaagcattgacctgaagtacttcaatgaatcaacaaagaagaaaattgagaaaataaggcctcttctaatagatggcacagcatcactgagtcctgggatgatgatgggcatgttcaacatgctaagtacggtcttgggagtctcgatactgaatcttggacaaaagaaatacaccaagacaatatactggtgggatgggctccaatcatccgacgattttgctctcatagtgaatgcaccaaaccatgagggaatacaagcaggagtggacagattctacaggacctgcaagttagtgggaatcaacatgagcaaaaagaagtcctatataaataagacagggacatttgaattcacaagctttttttatcgctatggatttgtggctaattttagcatggagctacccagctttggagtgtctggagtaaatgaatcagctgacatgagtattggagtaacagtgataaagaacaacatgataaacaatgaccttggacctgcaacggcccagatggctcttcaattgttcatcaaagactacagatacacatataggtgccataggggagacacacaaattcagacgagaagatcatttgagttaaagaagctgtgggatcaaacccaatcaaaggtagggctattagtatcagatggaggaccaaacttatacaatatacggaatcttcacattcctgaagtctgcttaaaatgggagctaatggatgatgattatcggggaagactttgtaatcccctgaatccctttgtcagtcataaagagattgattctgtaaacaatgctgtggtaatgccagcccatggtccagccaaaagcatggaatatgatgccgttgcaactacacattcctggattcccaagaggaatcgttctattctcaacacaagccaaaggggaattcttgaggatgaacagatgtaccagaagtgctgcaatctattcgagaaatttttccctagcagttcatataggagaccggttggaatttctagcatggtggaggccatggtgtctagggcccggattgatgccagggtcgacttcgagtctggacggatcaagaaagaagagttctctgagatcatgaagatctgttccaccattgaagaactcagacggcaaaaataa

>H1N1_PB1_CY073440_A/San Diego/WR1651P/2009

atggatgtcaatccgactctacttttcctaaaaattccagcgcaaaatgccataagcaccacattcccttatactggagatcctccatacagccatggaacaggaacaggatacaccatggacacagtaaacagaacacaccaatactcagaaaagggaaagtggacgacaaacacagagactggtgcaccccagctcaacccgattgatggaccattacctgaggataatgaaccaagtgggtatgcacaaacagactgtgttctagaggctatggctttccttgaagaatcccacccaggaatatttgagaattcatgccttgaaacaatggaagttgttcaacaaacaagggtagataaactaactcaaggtcgccagacttatgattggacattaaacagaaatcaaccggcagcaactgcattggccaacaccatagaagtctttagatcgaatggcctaacagctaatgagtcaggaaggctaatagatttcttaaaggatgtaatggaatcaatgaacaaagaggaaatagagataacaacccactttcaaagaaaaaggagagtaagagacaacatgaccaagaagatggtcacgcaaagaacaatagggaagaaaaaacaaagactgaataagagaggctatctaataagagcactgacattaaatacgatgaccaaagatgcagagagaggcaagttaaaaagaagggctatcgcaacacctgggatgcagattagaggtttcgtatactttgttgaaactttagctaggagcatttgcgaaaagcttgaacagtctgggctcccagtagggggcaatgaaaagaaggccaaactggcaaatgttgtgagaaagatgatgactaattcacaagacacagagatttctttcacaatcactggggacaacactaagtggaatgaaaatcaaaatcctcgaatgttcctggcgatgattacatatatcaccagaaatcaacccgagtggttcagaaacatcctgagcatggcacccataatgttctcaaacaaaatggcaagactagggaaagggtacatgttcgagagtaaaagaatgaagattcgaacacaaataccagcagaaatgctagcaagcattgacctgaagtacttcaatgaatcaacaaagaagaaaattgagaaaataaggcctcttctaatagatggcacagcatcactgagtcctgggatgatgatgggcatgttcaacatgctaagtacagtcttgggagtctcgatactgaatcttggacaaaagaaatacaccaagacaatatactggtgggatgggctccaatcatccgacgattttgctctcatagtgaatgcaccaaaccatgagggaatacaagcaggagtggacagattctacaggacctgcaagttagtgggaatcaacatgagcaaaaagaagtcctatataaataagacagggacatttgaattcacaagctttttttatcgctatggatttgtggctaattttagcatggagctacccagctttggagtgtctggagtaaatgaatcagctgacatgagtattggagtaacagtgataaagaacaacatgataaacaatgaccttggacctgcaacggcccagatggctcttcaattgttcatcaaagactacagatacacatataggtgccataggggagacacacaaattcagacgagaagatcatttgagttaaagaagctgtgggatcaaacccaatcaaaggtagggctattagtatcagatggaggaccaaacttatacaatatacggaatcttcacattcctgaagtctgcttaaaatgggagctaatggatgatgattatcggggaagactttgtaatcccctgaatccctttgtcagtcataaagagattgattctgtaaacaatgctgtggtaatgccagcccatggtccagccaaaagcatggaatatgatgccgttgcaactacacattcctggattcccaagaggaatcgttctattctcaacacaagccaaaggggaattcttgaggatgaacagatgtaccagaagtgctgcaatctattcgagaaatttttccctagcagttcatataggagaccggttggaatttctagcatggtggaggccatggtgtctagggcccggattgatgccagggtcgacttcgagtctggacggatcaagaaagaagagttctctgagatcatgaagatctgttccaccattgaagaactcagacggcaaaaataa

>H1N1_PB1_CY075578_A/Boston/658/2009

atggatgtcaatccgactctacttttcctaaaaattccagcgcaaaatgccataagcaccacattcccttatactggagatcctccatacagccatggaacaggaacaggatacaccatggacacagtaaacagaacacaccaatactcagaaaagggaaagtggacgacaaacacagagactggtgcaccccagctcaacccgattgatggaccactacctgaggataatgaaccaagtgggtatgcacaaacagactgtgttctagaggctatggctttccttgaagaatcccacccaggaatatttgagaattcatgccttgaaacaatggaagttgttcaacaaacaagggtagacaaactaactcaaggtcgccagacttatgattggacattaaacagaaatcaaccggcagcaactgcattggccaacaccatagaagtttttagatcgaatggcctaacagctaatgagtcaggaaggctaatagatttcttaaaggatgtaatggaatcaatgaacaaagaggaaatagagataacaacccactttcaaagaaaaaggagagtaagagacaacatgaccaagaagatggtcacgcaaagaacaatagggaagaaaaaacaaagactgaataagagaggctatctaataagagcactgacattaaatacgatgaccaaagatgcagagagaggcaagttaaaaagaagggctatcgcaacacctgggatgcagattagaggtttcgtatactttgttgaaactttagctaggagcatttgcgaaaagcttgaacagtctgggctcccagtagggggcaatgaaaagaaggccaaactggcaaatgttgtgagaaagatgatgactaattcacaagacacagagatttctttcacaatcactggggacaacactaagtggaatgaaaatcaaaatcctcgaatgttcctggcgatgattacatatatcaccagaaatcaacccgagtggttcagaaacatcctgagcatggcacccataatgttctcaaacaaaatggcaagactagggaaagggtacatgttcgagagtaaaagaatgaagattcgaacacaaataccagcagaaatgctagcaagcattgacctgaagtacttcaatgaatcaacaaagaagaaaattgagaaaataaggcctcttctaatagatggcacagcatcactgagtcctgggatgatgatgggcatgttcaacatgctaagtacggtcttgggagtctcgatactgaatcttggacaaaagaaatacaccaagacaatatactggtgggatgggctccaatcatccgacgattttgctctcatagtgaatgcaccaaaccatgagggaatacaagcaggagtggacagattctacaggacctgcaagttagtgggaatcaacatgagcaaaaagaagtcctatataaataagacagggacatttgaattcacaagctttttttatcgctatggatttgtggctaattttagcatggagctacccagctttggagtgtctggagtaaatgaatcagctgacatgagtattggagtaacagtgataaagaacaacatgataaacaatgaccttggacctgcaacggcccagatggctcttcaattgttcatcaaagactacagatacacatataggtgccataggggagacacacaaattcagacgagaagatcatttgagttaaagaagctgtgggatcaaacccaatcaaagggagggctattagtatcagatggaggaccaaacttatacaatatacggaatcttcacattcctgaagtctgcttaaaatgggagctaatggatgatgattatcggggaagactttgtaatcccctgaatccctttgtcagtcataaagagattgattctgtaaacaatgctgtggtaatgccagcccatggtccagtcaaaagcatggaatatgatgccgttgcaactacacattcctggattcccaagagaaatcgttctattctcaacacaagccaaaggggaattcttgaggatgaacagatgtaccagaagtgctgcaatctattcgagaaatttttccctagcagttcatataggagaccggttggaatttctagcatggtggaggccatggtgtctagggcccggattgatgccagggtcgatttcgagtctggacggatcaagaaagaagagttctctgagatcatgaagatctgctccaccattgaagaaatcagacggcaaaaataa

>H1N1_PB1_CY080783_A/Boston/38/2009

atggatgtcaatccgacattacttttcttaaaagtgccagcacaaaatgctataagcacaacttttccttatactggtgaccctccttacagccatgggacaggaacagggtacaccatggatacagtcaacaggacacaccagtactcagaaagaggaagatggacaaaaaataccgaaacgggagcaccgcaacttaacccaattgatggtcccttaccggaagacaatgaaccaagtggctatgcccaaacagattgtgtattagaagcaatggctttccttgaagaatcccatcccggtatctttgaaaactcttgtattgaaacaatggaggttgttcaacaaacaagggtggacaaactgacacaaggcagacagacctatgactggactctaaataggaaccagcctgctgccacagcattggcaaacactatagaagtattcagatcaaacggcctcatagcaaatgaatctggaaggctaatagacttccttaaagatgtaatggagtcgatggacagaggcgaagtagaggtcacaactcattttcaaagaaagaggagagtgagagacaatgtaactaaaaaaatggtgacccaaagaacaataggcaaaaagaaacataaattagacaaaagaagttacctaattagggcattaaccctgaacacaatgaccaaagatgctgagagggggaaactaaaacgcagagcaattgcaaccccaggaatgcaaataagggggtttgtatactttgttgagacactggcaagaagcatatgtgaaaagcttgaacaatcaggattgccagttggaggaaatgagaagaaagcaaagttagcaaatgttgtaaggaagatgatgaccaactcccaggacactgaaatttctttcaccataaccggagacaacacaaaatggaacgaaaatcaaaaccctagaatgttcttggccatgatcacatatataaccaaaaatcagcctgaatggttcagaaatattctaagtattgctccaataatgttttcaaacaagatggcgagactaggtaaggggtacatgtttgaaagcaagagtatgaaactgagaactcaaatacctgcagagatgctagccaacatagacttgaaatatttcaatgattcaactaaaaagaaaattgaaaaaatccgaccattattaatagatggaactgcatcattgagtcctggaatgatgatgggcatgttcaatatgttgagcaccgtattgggcgtctccattctgaatcttgggcaaaagagatacaccaagactacttactggtgggatggtcttcaatcgtctgatgattttgctttaattgtgaacgcacccaactatgcaggaattcaagctggagttgacaggttttatcgaacctgtaagctgctcggaattaatatgagcaaaaagaagtcttacataaacagaacaggtacctttgaattcacgagctttttctatcgttatgggtttgttgccaatttcagcatggagcttcctagttttggggtgtctggggtcaatgaatctgcagacatgagtattggagtcactgtcatcaaaaacaatatgataaacaatgaccttggcccagcaactgctcaaatggcccttcagttatttataaaagattacaggtacacttatcgatgccaccgaggtgacacacaaatacaaacccgaagatcatttgagataaagaaactatgggaccaaacccgctcaaaagctggactgttggtctctgatggaggccccaatttgtataacattagaaatctccatattcctgaagtttgcttgaaatgggagttgatggatgaggattaccaggggcgtttatgcaacccattaaacccgtttgtcagccataaagagattgaatcagtgaacagtgcagtgataatgccggcacatggtccagccaaaaatatggagtatgacgctgttgcaacaacacactcctgggtccccaaaagaaatcgatccattttgaacacgagccaaagggggatacttgaagatgagcaaatgtatcagaggtgctgcaatttatttgaaaaattcttcccaagtagctcatacagaagaccagttggaatatccagtatggtagaggctatggtctcaagagcccgaattgatgcacggattgatttcgaatctgaaaggataaagaaagaggaatttgctgagatcatgaagatctgttccaccattgaagacctcagacggcaaaaatga

>H1N1_PB1_CY083924_A/Warsaw/INS147/2009

atggatgtcaatccgactctacttttcctaaaaattccagcgcaaaatgccataagcaccacattcccttatactggagatcctccatacagccatggaacaggaacaggatacaccatggacacagtaaacagaacacaccaatactcagaaaaaggaaagtggacgacaaacacagagactggtgcaccccagctcaacccgattgatggaccactacctgaggataatgaaccaagtgggtatgcacaaacagactgtgttctagaggctatggctttccttgaagaatcccacccaggaatatttgagaattcatgccttgaaacaatggaagttgttcaacaaacaagggtagataaactaactcaaggtcgccagacttatgattggacattaaacagaaatcaaccggcagcaactgcattggccaacaccatagaagtctttagatcgaatggcctaacagctaatgagtcaggaaggttaatagatttcttaaaggatgtaatggaatcaatgaacaaagaggaaatagagataacaacccactttcaaagaaaaaggagagtaagagacaacatgaccaagaagatggtcacgcaaagaacaatagggaagaaaaaacaaagactgaataagagaggctatctaataagagcactgacattaaatacgatgaccaaagatgcagagagaggcaagttaaaaagaagggctatcgcaacacctgggatgcagattagaggtttcgtatactttgttgaaactttggctaggagcatttgcgaaaagcttgaacagtctgggctcccagtagggggcaatgaaaagaaggccaaactggcaaatgttgtgagaaagatgatgactaattcacaagacacagagatttctttcacaatcactggggacaacactaagtggaatgaaaatcaaaatcctcgaatgttcctggcgatgattacatatatcaccagaaatcaacccgagtggttcagaaacatcctgagcatggcacccataatgttctcaaacaaaatggcaagactagggaaagggtacatgttcgagagtaaaagaatgaagattcgaacacaaataccagcagaaatgctagcaagcatagacctgaagtacttcaatgaatcaacaaagaagaaaattgagaaaataaggcctcttctaatagatggcacagcatcactgagtcctgggatgatgatgggcatgttcaacatgctaagtacggtcttgggagtctcgatactgaatcttggacaaaagaaatacaccaagacaatatactggtgggatgggctccaatcatccgacgattttgctctcatagtgaatgcaccaaaccatgagggaatacaagcaggagtggacagattctacaggacctgcaagttagtgggaatcaacatgagcaaaaagaagtcctatataaataagacagggacatttgaattcacaagctttttttatcgctatggatttgtggctaattttagcatggagctacccagctttggagtgtctggagtaaatgaatcagctgacatgagtattggagtaacagtgataaagaacaacatgataaacaatgaccttggacctgcaacggcccagatggctcttcaattgttcatcaaagactacagatacacatataggtgccataggggagacacacaaattcagacgagaagatcatttgagttaaagaagctgtgggatcaaacccaatcaaaggtagggctattagtatcagatggaggaccaaacttatacaatatacggaatcttcacattcctgaagtctgcttaaaatgggagctaatggatgatgattatcggggaagactttgtaatcccctgaatccctttgtcagtcataaagagattgattctgtaaacaatgctgtggtaatgccagcccatggtccagccaaaagcatggaatatgatgccgttgcaactacacattcctggattcccaagaggaatcgttctattctcaacacaagccaaaggggaattcttgaggatgaacagatgtaccagaagtgctgcaatctattcgagaagtttttccctagcagttcatataggagaccggttggaatttctagcatggtggaggccatggtgtctagggcccggattgatgccagggtcgacttcgagtctggacggatcaagaaagaagagttctctgagatcatgaagatctgttccaccattgaagaactcagacggcaaaaataa

>H1N1_PB1_JF714140_A/Korea/NAP-1/2009

atggatgtcaatccgactctacttttcctaaaaattccagcgcaaaatgccataagcaccacattcccttatactggagatcctccatacagccatggaacaggaacaggatacaccatggacacagtaaacagaacacaccaatactcagaaaagggaaagtggacgacaaacacagagactggtgcaccccagctcaacccgattgatggaccactacctgaggataatgaaccaagtgggtatgcacaaacagactgtgttctagaggctatggctttccttgaagaatcccacccaggaatatttgagaattcatgccttgaaacaatggaagttgttcaacaaacaagggtagataaactaactcaaggtcgccagacttatgattggacattaaacagaaatcaaccggcagcaactgcattggccaacaccatagaagtctttagatcgaatggcctaacagctaatgagtcaggaaggctaatagatttcttaaaggatgtaatggaatcaatgaacaaagaggaaatagagataacaacccactttcaaagaaaaaggagagtaagagacaacatgaccaagaagatggtcacgcaaagaacaatagggaagaaaaaacaaagactgaataagagaggctatctaataagagcactgacattaaatacgatgaccaaagatgcagagagaggaaagttaaaaagaagggctatcgcaacacctgggatgcagattagaggtttcgtatactttgttgaaactttagctaggagcatttgcgaaaagcttgaacagtctgggctcccagtagggggcaatgaaaagaaggccaaactggcaaatgttgtgagaaagatgatgactaattcacaagacacagagatttctttcacaatcactggggacaacactaagtggaatgaaaatcaaaatcctcgaatgttcctggcgatgattacatatatcaccagaaatcaacccgagtggttcagaaacatcctgagcatggcacccataatgttctcaaacaaaatggcaagactagggaaagggtacatgttcgagagtaaaagaatgaagattcgaacacaaataccagcagaaatgctagcaagcattgacctgaagtacttcaatgaatcaacaaagaagaaaattgagaaaataaggcctcttctaatagatggcacagcatcactgagtcctgggatgatgatgggcatgttcaacatgctaagtacggtcttgggagtctcgatactgaatcttggacaaaagaaatacaccaagacaatatactggtgggatgggctccaatcatccgacgattttgctctcatagtgaatgcaccaaaccatgagggaatacaagcaggagtggacagattctacaggacctgcaagttagtgggaatcaacatgagcaaaaagaagtcctatataaataagacagggacatttgaattcacaagctttttttatcgctatggatttgtggctaattttagcatggagctacccagctttggagtgtctggagtaaatgaatcagctgacatgagtattggagtaacagtgataaagaacaacatgataaacaatgaccttggacctgcaacggcccagatggctcttcaactgttcatcaaagactacagatacacatataggtgccataggggagacacacaaattcagacgagaagatcatttgagttaaagaagctgtgggatcaaacccaatcaaaggtagggctattagtatcagatggaggaccaaacttatacaatatacggaatcttcacattcctgaagtctgcttaaaatgggagctaatggatgatgattatcggggaagactttgtaatcccctgaatccctttgtcagtcataaagagattgattctgtaaacaatgctgtggtaatgccagcccatggtccagccaaaagcatggaatatgatgccgttgcaactacacattcctggattcccaagaggaatcgttctattctcaacacaagccaaaggggaattcttgaggatgaacagatgtaccagaagtgctgcaatctattcgagaaatttttccctggcagttcatataggagaccggttggaatttctagcatggtggaggccatggtgtctagggcccggattgatgccagggtcgacttcgagtctggacggatcaagaaagaagagttctctgagatcatgaagatctgttccaccattgaagaactcagacggcaaaaataa

>H1N1_PB1_CY092644_A/Sydney/DD3-37/2010

atggatgtcaatccgactctacttttcctaaaaattccagcgcaaaatgccataagcaccacattcccttatactggagatcctccatacagccatggaacaggaacaggatacaccatggacacagtaaacagaacacaccaatactcagaaaagggaaagtggacgacaaacacagagactggtgcaccccagctcaacccgattgatggaccactacctgaggataatgaaccaagtgggtatgcacaaacagactgtgttctagaggctatggctttccttgaagaatcccacccaggaatatttgagaattcatgccttgaaacaatggaagttgttcaacaaacaagggtagataaactaactcaaggtcgccagacttatgattggacattaaacagaaatcaaccggcagcaactgcattggccaacaccatagaagtctttagatcgaatggcctaacagctaatgagtcaggaaggctaatagatttcttaaaggatgtaatggaatcaatgaacaaagaggaaatagagataacaacccactttcaaagaaaaaggagagtaagagacaacatgaccaagaagatggtcacgcaaagaacaatagggaagaaaaaacaaaggctgaataagagaggctatctaataagagcactgacattaaatacgatgaccaaagatgcagagagaggcaagttaaaaagaagggctatcgcaacacctgggatgcagattagaggtttcgtatactttgttgaaactttagctaggagcatttgcgaaaagcttgaacagtctgggctcccagtagggggcaatgaaaagaaggccaaactggcaaatgttgtgagaaagatgatgactaattcacaagacacagagatttctttcacaatcactggggacaacactaagtggaatgaaaatcaaaatcctcgaatgttcctggcgatgattacatatatcaccagaaatcaacccgagtggttcagaaacatcctgagcatggcacccataatgttctcaaacaaaatggcaagactagggaaagggtacatgttcgagagtaaaagaatgaagatccgaacacaaataccagcagaaatgctagcaagcattgacctgaagtacttcaatgaatcaacaaagaagaaaattgagaaaataaggcctcttctaatagatggcacagcatcactgagtcctggaatgatgatgggcatgttcaacatgctaagtacggtcttgggagtctcgatactgaatcttggacaaaagaaatacaccaagacaatatactggtgggatgggctccaatcatcagacgattttgctctcatagtgaatgcaccaaaccatgagggaatacaagcaggagtggacagattctacaggacctgcaagttagtgggaatcaacatgagcaaaaagaagtcctatataaataagacagggacatttgaattcacaagctttttttatcgctatggatttgtggctaattttagcatggagctacccagctttggagtgtctggagtaaatgaatcagcagacatgagtattggagtaacagtgataaagaacaacatgataaacaatgaccttggacctgcaacggcccagatggctcttcaattgttcatcaaagactacagatacacatataggtgccataggggagacacacaaattcagacgagaagatcatttgagttaaagaagctgtgggatcaaacccaatcaaaggtagggctattagtatcagatggaggaccaaacttatacaatatacggaatcttcacatccctgaagtctgcttaaaatgggagctaatggatgatgattatcggggaagactttgtaatcccctgaatccctttgtcagtcataaagagattgattctgtaaacaatgctgtggtaatgccagcccatggtccagtcaaaagcatggaatatgatgccgttgcaactacacattcctggattcccaagaggaatcgttctattctcaacacaagccaaaggggaattcttgaggatgaacagatgtaccagaagtgctgcaatctattcgagaaatttttccctagcagttcatataggagaccggttggaatttctagcatggtggaggccatggtgtctagggcccggattgatgccagggtcgacttcgagtctggacggatcaagaaagaagagttctctgagatcatgaagatctgttccaccattgaagaactcagacggcaaaaataa

>H1N1_PB1_CY104250_A/TayNguyen/TN371/2005

atggatgtcaatccgacattacttttcttaaaagtgccggcacaaaatgctataagcacaacttttccttatactggtgaccctccttacagccatgggacaggaacagggtacaccatggatacagtcaacaggacacatcagtactcagaaagaggaagatggacaaaaaacaccgaaactggagcaccgcaactcaacccaattgatgggccactaccagaagacaacgaaccaagtggctatgcccaaacagattgtgtattagaagcaatggctttccttgaagaatcccatcctggtatttttgaaagctcttgtattgaaacaatggaggttgttcagcaaacaagggtggacaaactgacacaaggcagacagacctatgactggactctaaataggaaccagcctgctgccacagcattggcaaacactatagaagtattcagatcaaacggcctcatagcaaatgaatctggaaggctaatagacttccttaaagatgtaatggattcgatggacagagacgaagtagaggtcacaactcattttcaaagaaagaggagagtgagagacaatgtaactaaaaaaatggtgacccaaagaacaataggcaaaaagaaacataaattagacaaaagaagttacctaattagggcattaaccctgaacacaatgaccaaagatgctgagagggggaaactaaaacgcagagcaattgcaaccccaggaatgcaaataagggggtttgtatactttgttgagacactggcaagaagcatatgtgaaaagcttgaacaatcagggttgccagttggaggaaatgagaagaaagcaaagttagcaaatgttgtaaggaagatgatgaccaactcccaggacactgaaatttctttcaccatcactggagataacacaaaatggaacgaaaatcaaaaccctagaatgttcttggccatgatcacatatataaccaaaaatcagcctgaatggttcagaaatattctaagtattgctccaataatgttttcaaataagatggcgagactaggtaaggggtacatgtttgaaagcaagaatatgaaactgagaactcaaatacctgcagagatgctagccaacatagatttgaaatatttcaatgattcaactaaaaagaaaattgaaaaaatccgaccattattaatagatggaactgcatcattgagtcctggaatgatgatgggcatgttcaatatgttaagcaccgtcttgggcgtctccattctgaatcttgggcaaaagagatacaccaagactacttactggtgggatggtcttcaatcgtctgatgattttgctttgattgtgaatgcacccaactatgcaggaattcaagctggagttgacaggttttatcgaacctgtaagctgctcggaattaacatgagcaaaaagaagtcttacataaacagaacaggtacctttgaattcacgagctttttctatcggtatggatttgttgccaatttcagcatggagcttcctagttttggggtgtctgggatcaatgaatctgcagacatgagtattggagtcactgtcatcaaaaacaatatgataaataatgaccttggcccagcaactgctcaaatggcccttcagttatttataaaagattacaggtacacttatcgatgccacagaggtgacacacaaatacaaacccggagatcatttgagataaagaaactatgggaccaaacccgctccaaagctgggctgttagtctctgatggagggcccaatttatataacattagaaatctccatattcctgaagtctgtttgaaatgggagttgatggatgaggattaccaggggcgtttatgcaacccattgaatccgttcgtcagccataaagagattgaatcagtgaacaatgcagtgataatgccggcacatggtccagccaaaaatatggagtatgacgctgttgcaacaacacactcctgggtccccaaaagaaatcgatccattttgaacacgagccaaagggggatacttgaagatgagcaaatgtatcagaggtgctgcaatttatttgaaaaattcttcccaagtagctcatacagaagaccagttggaatatccagtatggtagaggctatggtttcaagagcccgaattgatgcacggattgatttcgaatctggaaggataaagaaagaggaattcgctgagatcatgaagacctgttccaccattgaagacctcagacggcaaaaatag

>H1N1_PB1_CY111969_A/Hong Kong/H090-761-V10/2009

atggatgtcaatccgactctacttttcctaaaaattccagcgcaaaatgccataagcaccacattcccttatactggagatcctccatacagccatggaacaggaacaggatacaccatggacacagtaaacagaacacaccaatactcagaaaagggaaagtggacgacaaacacagagactggtgcaccccagctcaacccgattgatggaccactacctgaggataatgaaccaagtgggtatgcacaaacagactgtgttctagaggctatggctttccttgaagaatcccacccaggaatatttgagaattcatgccttgaaacaatggaagttgttcagcaaacaagggtagataaactaactcaaggtcgccagacttatgattggacattaaacagaaatcaaccggcagcaactgcattggccaacaccatagaagtctttagatcgaatggcctaacagctaatgagtcaggaaggctaatagatttcttaaaggatgtaatggaatcaatgaacaaagaggaaatagagataacaacccactttcaaagaaaaaggagagtaagagacaacatgaccaagaagatggtcacgcaaagaacaatagggaagaaaaaacaaagactgaataagagaggctatctaataagagcactgacattaaatacgatgaccaaagatgcagagagaggcaagttaaaaagaagggctatcgcaacacctgggatgcagattagaggtttcgtatactttgttgaaactttagctaggagcatttgcgaaaagcttgaacagtctgggctcccagtagggggcaatgaaaagaaggccaaactggcaaatgttgtgagaaagatgatgactaattcacaagacacagagatttctttcacaatcactggggacaacactaagtggaatgaaaatcaaaatcctcgaatgttcctggcgatgattacatatatcaccagaaatcaacccgagtggttcagaaacatcctgagcatggcacccataatgttctcaaacaaaatggcaagactagggaaagggtacatgttcgagagtaaaagaatgaagattcgaacacaaataccagcagaaatgctagcaagcattgacctgaagtacttcaatgaatcaacaaagaagaaaattgagaaaataaggcctcttctaatagatggcacagcatcactgagtcctgggatgatgatgggcatgttcaacatgctaagtacggtcttgggagtctcgatactgaatcttggacaaaagaaatacaccaagacaatatactggtgggatgggctccaatcatccgacgattttgctctcatagtgaatgcaccaaaccatgagggaatacaagcaggagtggacagattctacaggacctgcaagttagtgggaatcaacatgagcaaaaagaagtcctatataaataagacagggacatttgaattcacaagctttttttatcgctatggatttgtggctaattttagcatggagctacccagctttggagtgtctggagtaaatgaatcagctgacatgagtattggagtaacagtgataaagaacaacatgataaacaatgaccttggacctgcaacggcccagatggctcttcaattgttcatcaaagactacagatacacatataggtgccataggggagacacacaaattcagacgagaagatcatttgagttaaagaagctgtgggatcaaacccaatcaaaggtagggctattagtatcagatggaggaccaaacttatacaatatacggaatcttcacattcctgaagtctgcttaaaatgggagctaatggatgatgattatcggggaagactttgtaatcccctgaatccctttgtcagtcataaagagattgattctgtaaacaatgctgtggtaatgccagcccatggtccagccaaaagcatggaatatgatgccgttgcaactacacattcctggattcccaagaggaatcgttctattctcaacacaagccaaaggggaattcttgaggatgaacagatgtaccagaagtgctgcaatctattcgagaaatttttccctagcagttcatataggagaccggttggaatttctagcatggtggaggccatggtgtctagggcccggattgatgccagggtcgacttcgagtctggacggatcaagaaagaagagttctctgagatcatgaagatctgttccaccattgaagaactcagacggcaaaaataa

>H1N1_PB1_CY107817_A/Scotland/StAndrews_17693/2009

atggatgtcaatccgactctacttttcctaaaaattccagcgcaaaatgccataagcaccacattcccttatactggagatcctccatacagccatggaacaggaacaggatacaccatggacacagtaaacagaacacaccaatactcagaaaagggaaagtggacgacaaacacagagactggtgcaccccagctcaacccgattgatggaccactacctgaggataatgaaccaagtgggtatgcacaaacagactgtgttctagaggctatggctttccttgaagaatcccacccaggaatatttgagaattcatgccttgaaacaatggaagttgttcaacaaacaagggtagataaactaactcaaggtcgccagacttatgattggacattaaacagaaatcaaccagcagcaactgcattggccaacaccatagaagtctttagatcgaatggcctaacagctaatgagtcaggaaggctaatagatttcttaaaggatgtaatggaatcaatgaacaaagaggaaatagagataacaacccactttcaaagaaaaaggagagtaagagacaacatgaccaagaagatggtcacgcaaagaacaatagggaagaaaaaacaaagactgaataagagaagctatctaataagagcactgacattaaatacgatgaccaaagatgcagagagaggcaagttaaaaagaagggctatcgcaacacctgggatgcagattagaggtttcgtatactttgttgaaactttagctaggagcatttgcgaaaagcttgaacagtctgggctcccagtagggggcaatgaaaagaaggccaaactggcaaatgttgtgagaaagatgatgactaattcacaagacacagagatttctttcacaatcactggggacaacactaagtggaatgaaaatcaaaatcctcgaatgttcctggcgatgattacatatatcaccagaaatcaacccgagtggttcagaaacatcctgagcatggcacccataatgttctcaaacaaaatggcaagactagggaaagggtacatgttcgagagtaaaagaatgaagattcgaacacaaataccagcagaaatgctagcaagcattgacctgaagtacttcaatgaatcaacaaagaagaaaattgagaaaataaggcctcttctaatagatggcacagcatcactgagtcctgggatgatgatgggcatgttcaacatgctaagtacggtcttgggagtctcgatactgaatcttggacaaaagaaatacaccaagacaatatactggtgggatgggctccaatcatccgacgattttgctctcatagtgaatgcaccaaaccatgagggaatacaagcaggagtggacagattctacaggacctgcaagttagtgggaatcaacatgagcaaaaagaagtcctatataaataagacagggacatttgaattcacaagctttttttatcgctatggatttgtggctaattttagcatggagctacccagctttggagtgtctggagtaaatgaatcagctgacatgagtattggagtaacagtgataaagaacaacatgataaacaatgaccttggacctgcaacggcccagatggctcttcaattgttcatcaaagactacagatacacatataggtgccataggggtgacacacaaattcagacgagaagatcatttgagttaaagaagctgtgggatcaaacccaatcaaaggtagggctattagtatcagatggaggaccaaacttatacaatatacggaatcttcacattcctgaagtctgcttaaaatgggagctaatggatgatgattatcggggaagactttgtaatcccctgaatccctttgtcagtcataaagagattgattctgtaaacaatgctgtggtaatgccagcccatggtccagccaaaagcatggaatatgatgccgttgcaactacacattcctggattcccaagaggaatcgttctattctcaacacaagccaaaggggaattcttgaggatgaacagatgtaccagaagtgctgcaatctattcgagaaatttttccctagcagttcatataggagaccggttggaatttctagcatggtggaggccatggtgtctagggcccggattgatgccagggtcgacttcgagtctggacggatcaagaaagaagagttctctgagatcatgaagatctgttccaccattgaagaactcagacggcaaaaataa

>H1N1_PB1_CY122793_A/Singapore/GP2215/2009

atggatgtcaatccgactctacttttcctaaaaattccagcgcaaaatgccataagcaccacattcccttatactggagatcctccatacagccatggaacaggaacaggatacaccatggacacagtaaacagaacacaccaatactcagaaaagggaaagtggacgacaaacacagagactggtgcaccccagctcaacccgattgatggaccactacctgaggataatgaaccaagtgggtatgcacaaacagactgtgttctagaggctatggctttccttgaagaatcccacccaggaatatttgagaattcatgccttgaaacaatggaagttgttcaacaaacaagggtagataaactaactcaaggtcgccagacttatgattggacattaaacagaaatcaaccggcagcaactgcattggccaacaccatagaagtctttagatcgaatggcctaacagctaatgagtcaggaaggctaatagatttcttaaaggatgtaatggaatcaatgaacaaagaggaaatagagataacaacccactttcaaagaaaaaggagagtaagagacaacatgaccaagaagatggtcacgcaaagaacaatagggaagaaaaaacaaagactgaataagagaggctatctaataagagcactgacattaaatacgatgaccaaagatgcagagagaggcaagttaaaaagaagggctatcgcaacacctgggatgcagattagaggtttcgtatactttgttgaaactttagctaggagcatttgcgaaaagcttgaacagtctgggctcccagtagggggcaatgaaaagaaggccaaactggcaaatgttgtgagaaagatgatgactaactcacaagacacagagatttctttcacaatcactggggacaacactaagtggaatgaaaatcaaaatcctcgaatgttcctggcgatgattacatatatcaccagaaatcaacccgagtggttcagaaacatcctgagcatggcacccataatgttctcaaacaaaatggcaagactagggaaagggtacatgttcgagagtaaaagaatgaagattcgaacacaaataccagcagaaatgctagcaagcattgacctgaagtacttcaatgaatcaacaaagaagaaaattgagaaaataaggcctcttctaatagatggcacagcatcactgagtcctgggatgatgatgggcatgttcaacatgctaagtacggtcttgggagtctcgatactgaatcttggacaaaagaaatacaccaagacaatatactggtgggatgggctccaatcatccgacgattttgctctcatagtgaatgcaccaaaccatgagggaatacaagcaggagtggacagattctacaggacctgcaagttagtgggaatcaacatgagcaaaaagaagtcctatataaataagacagggacatttgaattcacaagctttttttatcgctatggatttgtggctaattttagcatggagctacccagctttggagtgtctggagtaaatgaatcagctgacatgagtattggagtaacagtgataaagaacaacatgataaacaatgaccttggacctgcaacggcccagatggctcttcaattgttcatcaaagactacagatacacatataggtgccataggggagacacacaaattcagacgagaagatcatttgagttaaagaagctgtgggatcaaacccaatcaaaggtagggctattagtatcagatggaggaccaaacttatacaatatacggaatcttcacatccctgaagtctgcttaaaatgggagctaatggatgatgattatcggggaagactttgtaatcccctgaatccctttgtcagtcataaagagattgattctgtaaacaatgctgtggtaatgccagcccatggtccagccaaaagcatggaatatgatgccgttgcaactacacattcctggattcccaagaggaatcgttctattctcaacacaagccaaaggggaattcttgaggatgaacagatgtatcagaagtgctgcaatctattcgagaaatttttccctagcagttcatataggagaccggttggaatttctagcatggtggaggccatggtgtctagggcccggattgatgccagggtcgacttcgagtctggacggatcaagaaagaagagttctctgagatcatgaagatctgttccaccattgaagaactcagacggcaaaaataa

>H1N1_PB1_CY123893_A/Singapore/ON2416/2009

atggatgtcaatccgactctacttttcctaaaaattccagcgcaaaatgccataagcaccacattcccttatactggagatcctccatacagccatggaacaggaacaggatacaccatggacacagtaaacagaacacaccaatactcagaaaaggggaagtggacgacaaacacagagactggtgcaccccagctcaacccgattgatggaccactacctgaggataatgaaccaagtgggtatgcacaaacagactgtgttctagaggctatggctttccttgaagaatcccacccaggaatatttgagaattcatgccttgaaacaatggaagttgttcaacaaacaagggtagataaactaactcaaggtcgccagacttatgattggacattaaacagaaatcaaccggcagcaactgcattggccaacaccatagaagtctttagatcgaatggcctaacagctaatgagtcaggaaggctaatagatttcttaaaggatgtaatggaatcaatgaacaaagaggaaatagagataacaacccactttcaaagaaaaaggagagtaagagacaacatgaccaagaagatggtcacgcaaagaacaatagggaagaaaaaacaaagactgaataagagaggctatctaataagagcactgacattaaatacgatgaccaaagatgcagagagaggcaagttaaaaagaagggctatcgcaacacctgggatgcagattagaggtttcgtatactttgttgaaactttagctaggagcatttgcgaaaagcttgaacagtctgggctcccagtagggggcaatgaaaagaaggccaaactggcaaatgttgtgagaaagatgatgactaattcacaagacacagagatttctttcacaatcactggggacaacactaagtggaatgaaaatcaaaatcctcgaatgttcctggcgatgattacatatatcaccagaaatcaacccgagtggttcagaaacatcctgagcatggcacccataatgttctcaaacaaaatggcaagactagggaaagggtacatgttcgagagtaaaagaatgaagattcgaacacaaataccagcagaaatgctagcaagcattgacctgaagtacttcaatgaatcaacaaagaagaaaattgagaaaataaggcctcttctaatagatggcacagcatcactgagtcctgggatgatgatgggcatgttcaacatgctaagtacggtcttgggagtctcgatactgaatcttggacaaaagaaatacaccaagacaatatactggtgggatgggctccaatcatccgacgattttgctctcatagtgaatgcaccaaaccatgagggaatacaagcaggagtggacagattctacaggacctgcaagttagtgggaatcaacatgagcaaaaagaagtcctatataaataagacagggacatttgaattcacaagctttttttatcgctatggatttgtggctaattttagcatggagctacccagctttggagtgtctggagtaaatgaatcagctgacatgagtattggagtaacagtgataaagaacaacatgataaacaatgaccttggacctgcaacggcccagatggctcttcaattgttcatcaaagactacagatacacatataggtgccataggggagacacacaaattcagacgagaagatcatttgagttaaagaagctgtgggatcaaacccaatcaaaggtagggctattagtatcagatggaggaccaaacttatacaatatacggaatcttcacattcctgaagtctgcttaaaatgggagctaatggatgatgattatcggggaagactttgtaatcccctgaatccctttgtcagtcataaagagattgattctgtaaacaatgctgtggtaatgccagcccatggtccagccaaaagcatggaatatgatgccgttgcaactacacattcctggattcccaagaggaatcgttctattctcaacacaagccaaaggggaattcttgaggatgaacagatgtaccagaagtgctgcaatctattcgagaaatttttccctagcagttcatataggagaccggttggaatttctagcatggtggaggccatggtgtctagggcccggattgatgccagggtcgacttcgagtctggacggatcaagaaagaagagttctctgagatcatgaagatctgttccaccattgaagaactcagacggcaaaaataa

>H1N1_PB1_CY124010_A/Singapore/ON520/2009

atggatgtcaatccgactctacttttcctaaaaattccagcgcaaaatgccataagcaccacattcccttatactggagatcctccatacagccatggaacaggaacaggatacaccatggacacagtaaacagaacacaccaatactcagaaaagggaaagtggacgacaaacacagagactggtgcaccccagctcaacccgattgatggaccactacctgaggataatgaaccaagtgggtatgcacaaacagactgtgttctagaggctatggctttccttgaagaatcccacccaggaatatttgagaattcatgccttgaaacaatggaagttgttcaacaaacaagggtagataaactaactcaaggtcgccagacttatgattggacattaaacagaaatcaaccggcagcaactgcattggccaacaccatagaagtctttagatcgaatggcctaacagctaatgagtcaggaaggctaatagatttcttaaaggatgtaatggaatcaatgaacaaagaggaaatagagataacaacccactttcaaagaaaaaggagagtaagagacaacatgaccaagaagatggtcacgcaaagaacaatagggaagaaaaaacaaagactgaataagagaggctatctaataagagcactgacattaaatacgatgaccaaagatgcagagagaggcaagttaaaaagaagggctatcgcaacacctgggatgcagattagaggtttcgtatactttgttgaaactttagctaggagcatttgcgaaaagcttgaacagtctgggctcccagtagggggcaatgaaaagaaggccaaactggcaaatgttgtgagaaagatgatgactaattcacaagacacagagatttctttcacaatcactggggacaacactaagtggaatgaaaatcaaaatcctcgaatgttcctggcgatgattacatatatcaccagaaatcaacccgagtggttcagaaacatcctgagcatggcacccataatgttctcaaacaaaatggcaagactagggaaagggtacatgttcgagagtaaaagaatgaagattcgaacacaaataccagcagaaatgctagcaagcattgacctgaagtacttcaatgaatcaacaaagaagaaaattgagaaaataaggcctcttctaatagatggcacagcatcactgagtcctgggatgatgatgggcatgttcaacatgctaagtacggtcttgggagtctcgatactgaatcttggacaaaagaaatacaccaagacaatatactggtgggatgggctccaatcatccgacgattttgctctcatagtgaatgcaccaaaccatgagggaatacaagcaggagtggacagattctacaggacctgcaagttagtgggaatcaacatgagcaaaaagaagtcctatataaataagacagggacatttgaattcacaagctttttttatcgctatggatttgtggctaattttagcatggagctacccagctttggagtgtctggagtaaatgaatcagctgacatgagtattggagtaacagtgataaagaacaacatgataaacaatgaccttggacctgcaacggcccagatggctcttcaattgttcatcaaagactacagatacacatataggtgccataggggagacacacaaattcagacgagaagatcatttgagttaaagaagctgtgggatcaaacccaatcaaaggtagggctattagtatcagatggaggaccaaacttatacaatatacggaatcttcacattcctgaagtctgcttaaaatgggagctaatggatgatgattatcggggaagactttgtaatcccctgaatccctttgtcagtcataaagagattgattctgtaaacaatgctgtggtaatgccagcccatggtccagccaaaagcatggaatatgatgccgttgcaactacacattcctggattcccaagaggaatcgttctattctcaacacaagccaaaggggaattcttgaggatgaacagatgtaccagaagtgctgcaatctattcgagaaatttttccctagcagttcatataggagaccggttggaatttctagcatggtggaggccatggtgtctagggcccggattgatgccagggtcgacttcgagtctggacggatcaagaaagaagagttctctgagatcatgaagatctgttccaccattgaagaactcagacggcaaaaataa

>H1N1_PB1_AB704468_A/Tochigi/445/2009

atggatgtcaatccgactctacttttcctaaaaattccagcgcaaaatgccataagcaccacattcccttatactggagatcctccatacagccatggaacaggaacaggatacaccatggacacagtaaacagaacacaccaatactcagaaaagggaaagtggacgacaaacacagagactggtgcaccccagctcaacccgattgatggaccactacctgaggataatgaaccaagtgggtatgcacaaacagactgtgttctagaggctatggctttccttgaagaatcccacccaggaatatttgagaattcatgccttgaaacaatggaagttgttcaacaaacaagggtagataaactaactcaaggtcgccagacttatgattggacattaaacagaaatcaaccggcagcaactgcattggccaacaccatagaagtctttagatcgaatggcctaacagctaatgagtcaggaaggctaatagatttcttaaaggatgtaatggaatcaatgaacaaagaggaaatagagataacaacccactttcaaagaaaaaggagagtaagagacaacatgaccaagaagatggtcacgcaaagaacaatagggaagaaaaaacaaagactgaataagagaggctatctaataagagcactgacattaaatacgatgaccaaagatgcagagagaggcaagttaaaaagaagggctattgcaacacctgggatgcagattagaggtttcgtatactttgttgaaactttagctaggagcatttgcgaaaagcttgaacagtctgggctcccagtagggggcaatgaaaagaaggccaaactggcaaatgttgtgagaaaaatgatgactaattcacaagacacagagatttctttcacaatcactggggacaacactaagtggaatgaaaatcaaaatcctcgaatgttcctggcgatgattacatatatcaccagaaatcaacccgagtggttcagaaacatcctgagcatggcacccataatgttctcaaacaaaatggcaagactagggaaagggtacatgttcgagagtaaaagaatgaagattcgaacacaaataccagcagaaatgctagcaagcattgacctgaagtacttcaatgaatcaacaaagaagaaaattgagaaaataaggcctcttctaatagatggcacagcatcactgagtcctgggatgatgatgggcatgttcaacatgctaagtacggtcttgggagtctcgatactgaatcttggacaaaagaaatacaccaagacagtatactggtgggatgggctccaatcatccgacgattttgctctcatagtgaatgcaccaaaccatgagggaatacaagcaggagtggacagattctacaggacctgcaagttagtgggaatcaacatgagcaaaaagaagtcctatataaataagacagggacatttgaattcacaagctttttttatcgctatggatttgtggctaattttagcatggagctacccagctttggagtgtctggagtaaatgaatcagctgacatgagtattggagtaacagtgataaagaacaacatgataaacagtgaccttggacctgcaacggcccagatggctcttcaattgttcataaaagactacagatacacatataggtgccataggggagacacacaaattcagacgagaagatcatttgagttaaagaagctgtgggatcaaacccaatcaaaggtagggctattagtatcagatggaggaccaaacttatacaatatacggaatcttcacattcctgaagtctgcttaaaatgggagctaatggatgatgattatcggggaagactttgtaatcccctgaatccctttgtcagtcataaagagattgattctgtaaacaatgctgtggtaatgccagcccatggtccagccaaaagcatggaatatgatgccgttgcaactacacattcctggattcccaagaggaatcgttctattctcaacacaagccaaaggggaattcttgaggatgaacagatgtaccagaagtgctgcaatctattcgagaaatttttccctagcagttcatataggagaccggttggaatttctagcatggtggaggccatggtgtctagggcccggattgatgccagggtcgacttcgaatctggacggatcaagaaagaagagttctctgagatcatgaagatctgttccaccattgaagaactcagacggcaaaaataa

>H1N1_PB1_JX875050_A/Kentucky/110/2009

atggatgtcaatccgactctacttttcctaaaaattccagcgcaaaatgccataagcaccacattcccttatactggagatcctccatacagccatggaacaggaacaggatacaccatggacacagtaaacagaacacaccaatactcagaaaagggaaagtggacgacaaacacagagactggtgcaccccagctcaacccgattgatggaccactacctgaggataatgaaccaagtgggtatgcacaaacagactgtgttctagaggctatggctttccttgaagaatcccacccaggaatatttgagaattcatgccttgaaacaatggaagttgttcaacaaacaagggtagataaactaactcaaggtcgccagacttatgattggacattaaacagaaatcaaccggcagcaactgcattggccaacaccatagaagtctttagatcgaatggcctaacagctaatgagtcaggaaggctaatagatttcttaaaggatgtaatggaatcaatgaacaaagaggaaatagagataacaacccactttcaaagaaaaaggagagtaagagacaacatgaccaagaagatggtcacgcaaagaacaatagggaagaaaaaacaaagactgaataagagaggctatctaataagagcactgacattaaatacgatgaccaaagatgcagagagaggcaagttgaaaagaagggccatcgcaacacctgggatgcagattagaggtttcgtatactttgttgaaactttagctaggagcatttgcgaaaagcttgaacagtctgggctcccagtagggggcaatgaaaagaaggccaaactggcaaatgttgtgagaaagatgatgactaattcacaagacacagagatttctttcacaatcactggggacaacactaagtggaatgaaaatcaaaatcctcgaatgttcctggcgatgattacatatatcaccagaaatcaacccgagtggttcagaaacatcctgagcatggcacccataatgttctcaaacaaaatggcaagactagggaaagggtacatgttcgagagtaaaagaatgaagattcgaacacaaataccagcagaaatgctagcaagcattgacctgaagtacttcaatgaatcaacaaagaagaaaattgagaaaataaggcctcttctaatagatggcacagcatcattgagtcctgggatgatgatgggcatgttcaacatgctaagtacggtcttgggagtctcgatactgaatcttggacaaaagaaatacaccaagacaatatactggtgggatgggctccaatcatccgacgattttgctctcatagtgaatgcaccaaaccatgagggaatacaagcaggagtggacagattctacaggacctgcaagttagtgggaatcaacatgagcaaaaagaagtcctatataaataagacagggacatttgaattcacaagctttttttatcgctatggatttgtggctaattttagcatggagctacccagctttggagtgtctggagtaaatgaatcagctgacatgagtattggagtaacagtgataaagaacaacatgataaacaatgaccttggacctgcaacggcccagatggctcttcaattgttcatcaaagactacagatacacatataggtgccataggggagacacacaaattcagacgagaagatcatttgagttaaagaagctgtgggatcaaacccaatcaaaggtagggctattagtatcagatggaggaccaaacttatacaatatacggaatcttcacattcctgaagtctgcttaaaatgggagctaatggatgatgattatcggggaagactttgtaatcccctgaatccctttgtcagtcataaagagattgattctgtaaacaatgctgtggtaatgccagcccatggtccagccaaaagcatggaatatgatgccgttgcaaatacacattcctggattcccaagaggaatcgttctattctcaacacaagccaaaggggaattcttgaggatgaacagatgtaccagaagtgctgcaatctattcgagaaatttttccctagcagttcatataggagaccggttggaatttctagcatggtggaggccatggtgtctagggcccggattgatgccagggtagacttcgagtctggacggatcaagaaagaagagttctctgagatcatgaagatctgttccaccattgaagaactcagacggcaaaaataa

>H1N1_PB1_CY128465_A/Viet Nam/001-1889/2009

atggatgtcaatccgactctacttttcctaaaaattccagcgcaaaatgccataagcaccacattcccttatactggagatcctccatacagccatggaacaggaacaggatacaccatggacacagtaaacagaacacaccaatactcagaaaagggaaagtggacgacaaacacagagactggtgcaccccagctcaacccgattgatggaccactacctgaggataatgaaccaagtgggtatgcacaaacagactgtgttctagaggctatggctttccttgaagaatcccacccaggaatatttgagaattcatgccttgaaacaatggaagttgttcaacaaacaagggtagataaactaactcaaggtcgccagacttatgattggacattaaacagaaatcaaccggcagcaactgcattggccaacaccatagaagtctttagatcgaatggcctaacagctaatgagtcaggaaggctaatagatttcttaaaggatgtaatggaatcaatgaacaaagaggaaatagagataacaacccactttcaaagaaaaaggagagtaagagacaacatgaccaagaagatggtcacgcaaagaacaatagggaagaaaaaacaaagactgaataagagaggctatctaataagagcactgacattaaatacgatgaccaaagatgcagagagaggcaagttaaaaagaagggctatcgcaacacctgggatgcagattagaggtttcgtatactttgttgaaactttagctaggagcatttgcgaaaagcttgaacagtctgggctcccagtagggggcaatgaaaagaaggccaaactggcaaatgttgtaagaaagatgatgactaattcacaagacacagagatttctttcacaatcactggggacaacactaagtggaatgaaaatcaaaatcctcgaatgttcctggcgatgattacatatatcaccagaaatcaacccgagtggttcagaaacatcctgagcatggcacccataatgttctcaaacaaaatggcaagactagggaaagggtacatgttcgagagtaaaagaatgaagattcgaacacaaataccagcagaaatgctagcaagcattgacctgaagtacttcaatgaatcaacaaagaagaaaattgagaaaataaggcctcttctaatagatggcacagcatcactgagtcctgggatgatgatgggcatgttcaacatgctaagtacggtcttgggagtctcgatactgaatcttggacaaaagaaatacaccaagacagtatactggtgggatgggctccaatcatccgacgattttgctctcatagtgaatgcaccaaaccatgagggaatacaagcaggagtggacagattctacaggacctgcaagttagtgggaatcaacatgagcaaaaagaagtcctatataaataagacagggacatttgaattcacaagctttttttatcgctatggatttgtggctaattttagcatggagctacccagctttggagtgtctggagtaaatgaatcagctgacatgagtattggagtaacagtgataaagaacaacatgataaacaatgaccttggacctgcaacggcccagatggctcttcaattgttcatcaaagactacagatacacatataggtgccataggggagacacacaaattcagacgagaagatcatttgagttaaagaagctgtgggatcaaacccaatcaaaggtagggctattagtatcagatggaggaccaaacttatacaatatacggaatcttcacattcctgaagtctgcttaaaatgggagctaatggatgatgattatcggggaagactttgtaatcccctgaatccctttgtcagtcataaagagattgattctgtaaacaatgctgtggtaatgccagcccatggtccagccaaaagcatggaatatgatgccgttgcaactacacattcctggattcccaagaggaatcgttctattctcaacacaagccaaaggggaattcttgaggatgaacagatgtaccagaagtgctgcaatctattcgagaaatttttccctagcagttcatataggagaccggttggaatttctagcatggtggaggccatggtgtctagggcccggattgatgccagggtcgacttcgagtctggacggatcaagaaagaagagttctctgagatcatgaagatctgttccaccattgaagaactcagacggcaaaaataa

>H1N1_PB1_CY129940_A/Aarhus/INS613/2011

atggatgtcaatccgactctacttttcctaaaaattccagcgcaaaatgccataagcaccacattcccttatactggagatcctccatacagccatggaacaggaacaggatacaccatggacacagtaaacagaacacaccaatactcagaaaagggaaagtggacgacaaacacagagactggtgcaccccagctcaacccgattgatggaccactacctgaggataatgaaccaagtgggtatgcacaaacagactgtgttctagaggctatggctttccttgaagaatcccacccaggaatatttgagaattcatgccttgaaacaatggaagttgttcaacaaacaagggtagataaactaactcaaggtcgccagacttatgattggacattaaacagaaatcaaccggcagcaactgcattggccaacaccatagaagtctttagatcgaatggcctaacagctaatgagtcaggaaggctaatagatttcttaaaggatgtaatggaatcaatgaacaaagaggaaatagagataacaacccactttcaaagaaaaaggagagtaagagacaacatgaccaagaagatggtcacacaaagaacaatagggaagaaaaaacaaagactgaataagagaggctatctaataagagcactgacattaaatacgatgaccaaagatgcagagagaggcaagttaaaaagaagggctatcgcaacacctgggatgcagattagaggtttcgtatactttgttgaaactttagctaggagcatttgcgaaaagcttgaacagtctgggctcccagtagggggcaatgaaaagaaggccaaactggcaaatgttgtgagaaagatgatgactaattcacaagacacagagatttctttcacaatcactggggacaacactaagtggaatgaaaaccaaaatcctcgaatgttcctggcgatgattacatatatcaccagaaatcaacccgagtggttcagaaacatcctgagcatggcacccataatgttctcaaacaaaatggcaagactagggaaagggtacatgttcgagagtaaaagaatgaagattcgaacgcaaataccagcagaaatgctagcaagcattgacctgaagtacttcaatgaatcaacaaagaagaaaattgagaaaataaggcctcttttaatagatggcacagcatcactgagtcctgggatgatgatgggcatgttcaacatgctaagtacggtcttgggagtctcgatactgaatcttggacaaaagaaatacaccaagacaatatactggtgggatgggctccaatcatccgacgattttgctctcatagtgaatgcaccaaaccatgagggaatacaagcaggagtggacagattttacaggacctgcaagttagtgggaatcaacatgagcaaaaagaagtcctatataaataagacagggacatttgaattcacaagctttttttatcgctatggatttgtggctaattttagcatggagctacccagctttggagtgtctggagtaaatgaatcagctgacatgagtattggagtaacagtgataaagaacaacatgataaacaatgaccttggacctgcaacggcccagatggctcttcaattgttcatcaaagactacagatacacatataggtgccataggggagacacacaaattcagacgagaagatcatttgagttaaagaagctgtgggatcaaacccaatcaaaggtagggctattagtatcagatggaggaccaaacttatacaatatacggaatcttcacattcctgaagtctgcttaaaatgggagctaatggatgatgattatcggggaagactttgtaatcccctgaatccctttgtaagtcataaagagattgattctgtaaacaatgctgtggtaatgccagcccatggtccagccaaaagcatggaatatgatgccgttgcaactacacattcctggattcccaagaggaatcgttctattctcaacacaagccaaaggggaattcttgaggatgaacagatgtaccagaagtgctgcaatctattcgagaaatttttccctagcagttcatataggagaccggttggaatttctagcatggtggaggccatggtgtctagggcccggattgatgccagggtcgactttgagtctggacggatcaagaaagaagagttctctgagatcatgaagatctgttccaccattgaagaactcagacggcaaaaataa

>H1N1_PB1_CY129996_A/Kowloon/INS629/2011

atggatgtcaatccgactctacttttcctaaaaattccagcacaaaatgccataagcaccacattcccttatactggagatcctccatacagccatggaacaggaacaggatacaccatggacacagtaaacagaacacaccaatactcagaaaagggaaagtggacaacgaacacagagactggtgcaccccagctcaacccgattgatggaccactacctgaggataatgaaccaagtgggtatgcacaaacagactgtgttctagaggctatggctttccttgaagaatcccacccaggaatatttgagaattcatgccttgaaacaatggaagttgttcaacaaacaagggtagataaactgactcaaggtcgccagacttatgattggacattaaacagaaatcaaccggcagcaactgcattggccaacaccatagaagtctttagatcgaatgacctaacaactaatgagtcaggaaggctaatagatttcttaaaggatgtaatggaatcaatgaacaaagaggaaatagagataacaacccactttcaaagaaaaaggagagtaagagacaacatgaccaagaagatggtcacgcaaagaacaataggaaagaaaaaacagagactgaataagagaggctatctaataagagcactgacattaaatacgatgaccaaagatgcagagagaggcaagttaaaaagaagggctatcgcaacacctgggatgcagattagaggtttcgtatactttgttgaaactttagctaggagcatttgcgaaaagcttgaacagtctgggctcccagtagggggcaatgaaaagaaggccaaactggcaaatgttgtgagaaagatgatgactaattcacaagacacagagatttctttcacaatcactggggacaacactaagtggaatgaaaatcaaaatcctcgaatgttcctggcgatgattacatatatcaccagaaatcaacccgagtggtttagaaacatcctgagcatggcacccataatgttctcaaacaaaatggcaagactagggaaagggtacatgttcgagagtaaaagaatgaagattcgaacacaaataccagcagaaatgctagcaagcattgacctgaagtacttcaatgaatcaacaaagaagaaaattgagaaaataaggcctcttttaatggatggcacagcatcactgagtcctgggatgatgatgggcatgttcaacatgctaagtacggtcttgggagtctcgatactgaatcttggacaaaagaaatacaccaagacaacatactggtgggatgggctccaatcatccgacgattttgctctcatagtgaatgcaccaaaccatgagggaatacaagcaggagtggacagattctacaggacctgcaagttagtgggaatcaacatgagcaaaaagaagtcctatataaataagacagggacatttgaattcacaagctttttttatcgctatggatttgtggctaattttagcatggagctacccagctttggagtgtctggagtaaatgaatcagctgacatgagtattggagtaacagtgataaagaacaacatgataaacaatgaccttggacctgcaacggcccagatggctcttcaattgttcatcaaagactacagatacacatataggtgccataggggagacacacaaattcagacgagaagatcatttgagttaaagaagctgtgggatcaaacccaatcaaaggtagggctattagtatcagatggaggaccaaacttatacaatatacggaatcttcacattcctgaagtctgcttaaaatgggagctaatggatgatgattatcggggaagactttgtaatcccctgaatccctttgtaagtcataaggagattgattctgtaaacaatgctgtggtaatgccagcccatggtccagccaaaggcatggaatatgatgccgttgcaactacacattcctggattcccaagaggaatcgttctattctcaacacaagccaaaggggaattcttgaggatgaacagatgtaccagaagtgctgcaatctattcgagaaatttttccctagcagttcatataggagaccggttggaatttctagcatggtggaggccatggtgtctagggcccggattgatgccagggtcgactttgagtctggacggatcaagaaagaagagttctctgagatcatgaagatctgttccaccattgaagaactcagacggcaaaaataa

>H1N1_PB1_KC882425_A/Delaware/04/2011

atggatgtcaatccgactctacttttcctaaaaattccagcgcaaaatgccataagcaccacattcccttatactggagatcctccatacagccatggaacaggaacaggatacaccatggacacagtgaacagaacacaccaatactcagaaaagggaaagtggacgacaaacacagagactggtgcaccccagctcaacccgattgatggaccactacctgaggataatgaaccaagtgggtatgcacaaacagactgtgttctagaggctatggctttccttgaagaatcccacccagggatatttgagaattcatgccttgaaacaatggaagttgttcaacaaacaagggtagataaactaactcaaggtcgccagacttatgattggacattaaacagaaatcaaccggcagcaactgcattggccaacaccatagaagtctttagatcgaatggcctaacagctaatgagtcaggcaggctaatagatttcttaaaggatgtaatggaatcaatgaacaaagaggaaatagagataacaacccactttcaaagaaaaaggagagtaagagacaacatgaccaagaagatggtcacgcaaagaacaatagggaagaaaaaacaaagactgaataagagaggctatctaataagagcactgacattaaatacgatgaccaaagatgcagagagaggcaagttaaaaagaagggctatcgcaacacctgggatgcagattagaggtttcgtatactttgttgaaactttagctaggagcatttgcgaaaaacttgaacagtctgggctcccagtagggggcaatgaaaagaaggccaaactggcaaatgttgtgagaaagatgatgactaattcacaagacacagagatttctttcacaatcactggggacaacactaagtggaatgaaaatcaaaatcctcgaatgttcctggcgatgattacatatatcaccagaaatcaacccgagtggttcagaaacatcctgagcatggcacccataatgttctcaaacaaaatggcaagactagggaaagggtacatgttcgagagtaaaagaatgaagattcgaacacaaataccagcagaaatgctagcaagcattgacctgaagtacttcaatgaatcaacaaagaagaaaattgagaaaataaggcctcttttaatagatggcacagcatcactgagtcctgggatgatgatgggcatgttcaacatgctaagtacggtcttgggagtctcgatactgaatcttggacaaaagaaatacaccaagacaatatactggtgggatgggctccaatcatccgacgattttgctctcatagtgaatgcaccaaaccatgagggaatacaagcaggagtggacagattctacaggacctgcaagttagtgggaatcaacatgagcaaaaagaagtcctatataaataagacagggacatttgaattcacaagctttttttatcgctatggatttgtggctaattttagcatggagctacccagctttggagtgtctggagtaaatgaatcagctgacatgagtattggagtaacagtgataaagaacaacatgataaacaatgaccttggacctgcaacggcccagatggctcttcaattgttcatcaaagactacagatacacatataggtgccataggggagacacacaaattcagacgagaagatcatttgagttaaagaagctgtgggatcaaacccaatcaaaggtagggctattagtatcagatggaggaccaaacttatacaatatacggaatcttcacattcctgaagtctgcttaaaatgggagctaatggatgatgattatcggggaagactttgtaatcccctgaatccctttgtaagtcataaagagattgattctgtaaacaatgctgtggtaatgccagcccatggtccagccaaaagcatggaatatgatgccgttgcaactacacattcctggattcccaagaggaatcgttctattctcaacacaagccaaaggggaattcttgaggatgaacagatgtaccagaagtgctgcaatctattcgagaaatttttccctagcagttcatataggagaccggttggaatttctagcatggtggaggccatggtgtctagggcccggattgatgccagggtcgactttgagtctggacggatcaagaaagaagagttctctgagatcatgaagatctgttccaccattgaagaactcagacggcaaaaataa

>H1N1_PB1_CY147577_A/Mexico/24001/2009

atggatgtcaatccgacattacttttcttaaaagtgccagcacaaaatgctataagcacaacttttccttatactggtgaccctccttacagccatgggacaggaacagggtacaccatggatacagtcaataggacacaccagtactcagaaagaggaagatggacaaaaaataccgaaacgggagcaccgcaacttaacccaattgatggtcccttaccggaagacaatgaaccaagtggctatgcccaaacagattgtgtattagaagcaatggctttccttgaagaatcccatcccggtatctttgaaaactcttgtattgaaacaatggaggttgttcaacaaacaagggtggacaaactgacacaaggcagacagacctatgactggactctaaataggaaccagcctgctgccacagcattggcaaacactatagaagtattcagatcaaacggcctcatagcaaatgaatctggaaggctaatagacttccttaaagatgtaatggagtcgatggacagaggtgaagtagaggtcacaactcattttcaaagaaagaggagagtgagagacaatgtaactaaaaaaatggtgacccaaagaacaataggcaaaaagaaacataaattagacaaaagaagttacctaattagggcattaaccctgaacacaatgaccaaagatgctgagagggggaaactaaaacgcagagcaattgcaaccccaggaatgcaaataagggggtttgtatactttgttgagacactggcaagaagcatatgtgaaaagcttgaacaatcaggattgccagttggaggaaatgagaagaaagcaaagttagcaaatgttgtaaggaagatgatgaccaactcccaggacactgaaatttctttcaccataaccggagacaacacaaaatggaacgaaaatcaaaaccctagaatgttcttggccatgatcacatatataaccaaaaatcagcctgaatggttcagaaatattctaagtattgctccaataatgttttcaaacaagatggcgagactaggtaaggggtacatgtttgaaagcaagagtatgaaactgagaactcaaatacctgcagagatgctagccaacatagacttgaaatatttcaatgattcaactaagaagaaaattgaaaaaatccgaccattattaatagatggaactgcatcattgagtcctggaatgatgatgggcatgttcaatatgttgagcaccgtcttgggcgtctccattctgaatcttgggcaaaagagatacaccaagactacttactggtgggatggtcttcaatcgtctgatgattttgctttgattgtgaacgcacccaactatgcaggaattcaagctggagtcgacaggttctatcgaacctgtaagctgctcggaattaatatgagcaaaaagaagtcttacataaacagaacaggtacctttgaattcacgagctttttctatcgttatgggtttgttgccaatttcagcatggagcttcctagttttggggtgtctggggtcaatgaatctgcagacatgagtattggagtcactgtcatcaaaaacaatatgataaacaatgaccttggcccagcaactgctcaaatggcccttcagttatttataaaagattacaggtacacttatcgatgccaccgaggtgacacacaaatacaaacccgaagatcatttgagataaagaaactatgggaccaaacccgctcaaaagctggactgttggtctctgatggaggccccaatttgtataacattagaaatctccatattcctgaagtttgcttgaaatgggagttgatggatgaggattaccaggggcgtttatgcaacccattaaacccgtttgtcagccataaagagattgaatcagtgaacagtgcagtgataatgccggcacatggtccagccaaaaatatggagtatgacgctgttgcaacaacacactcctgggtccccaaaagaaatcgatccattttgaacacgagccaaaggggggtacttgaagatgagcaaatgtatcagaggtgctgcaatttatttgaaaaattcttcccaagtagctcatacagaagaccagttggaatatccagtatggtagaggctatggtctcaagagcccgaattgatgcacggattgatttcgaatctggaaggataaagaaagaggaatttgctgagatcatgaagatctgttccaccattgaagacctcagacggcaaaaatga

>H1N1_PB1_KF411321_A/Qingdao/FF85/2009

atggatgtcaatccgactctacttttcctaaaaattccagcacaaaatgccataagcaccacattcccttatactggagatcctccatatagccatggaacaggaacaggatacaccatggacacagtaaacagaacacaccaatactcagaaaagggaaagtggacaacaaacacagagactggtgcaccccagctcaacccgattgatggaccactacctgaggataatgaaccaagtgggtatgcacaaacagactgtgttctagaggctatggctttccttgaagaatcccacccaggaatatttgagaattcatgccttgaaacaatggaagttgttcaacaaacaagggtagataaactgactcaaggtcgccagacttatgattggacattaaacagaaatcaaccggcagcaactgcattggccaacaccatagaagtcttcagatcgaatgacctaacggctaatgagtcaggaaggctaatagatttcttaaaggatgtaatggaatcaatgaacaaagaggaaatagagataacaacccactttcaaagaaaaaggagagtaagagacaacatgaccaagaagatggtcacgcaaagaacaatagggaagaaaaaacagagactgaataagagaggctatctaataagagcactgacattaaatacgatgaccaaagatgcagagagaggcaagttaaaaagaagggctatcgcaacacctgggatgcagattagaggtttcgtatactttgttgaaactttagctaggagcatttgcgaaaagcttgaacagtctgggctcccagtagggggcaatgaaaagaaggccaaactggcaaatgttgtgagaaagatgatgactaattcacaagacacagagatttctttcacaatcactggggacaacactaagtggaatgaaaatcaaaatcctcgaatgttcctggcgatgattacatatatcaccagaaatcaacccgagtggtttagaaacatcctgagcatggcacccataatgttctcaaacaaaatggcaagactagggaaagggtacatgttcgagagtaaaagaatgaagattcgaacacaaataccagcagaaatgctagcaagcattgacctgaagtacttcaatgaatcaacaaagaagaaaattgagaaaataaggcctcttttaatggatggcacagcatcactgagtcctgggatgatgatgggcatgttcaacatgctaagtacggtcttgggagtctcgatactgaatcttggacaaaagaaatacaccaagacaacatactggtgggatgggctccaatcatccgacgattttgctctcatagtgaatgcaccaaaccatgagggaatacaagcaggagtggacagattctacaggacctgcaagttagtgggaatcaacatgagcaaaaagaagtcctatataaataagacagggacatttgaattcacaagctttttttatcgctatggatttgtggctaattttagcatggagctacccagctttggagtgtctggagtaaatgaatcagctgacatgagtattggagtaacagtgataaagaacaacatgataaacaatgaccttggacctgcaacggcccagatggctcttcaattgttcatcaaagactacagatacacatataggtgccataggggagacacacaaattcagacgagaagatcatttgagttaaagaagctgtgggatcaaacccaatcaaaggtagggctattagtatcagatggaggaccaaacttatacaatatacggaatctccacattcctgaagtctgcttaaaatgggagctaatggatgatgactatcggggaagactttgtaatcccctgaatccctttgtcagtcataaagagattgattctgtaaacaatgctgtggtaatgccagcccatggtccagccaaaagcatggaatatgatgccgttgcaactacacattcctggattcccaagaggaatcgttctattctcaacacaagccaaaggggaattcttgaggatgaacagatgtaccagaagtgctgcaatctattcgagaaatttttccctagcagttcatataggagaccggttggaatttctagcatggtggaggccatggtgtctagggcccggattgatgccagggtcgacttcgagtctggacggatcaagaaagaagagttctctgagatcatgaagatctgttccaccattgaagaactcagacggcaaaaataa

>H1N1_PB1_KF612051_A/Santa Cruz/111/2012

atggatgtcaatccgactttacttttcctaaaaattccagcgcaaaatgccataagcaccacattcccttatactggagatcctccatacagccatggaacaggaacaggatacaccatggacacagtaaacagaacacaccaatactcagaaaagggaaagtggacgacaaacacagagactggtgcaccccagctcaacccgattgatggaccactacctgaggataatgaaccaagtgggtatgcacaaacagactgtgttctagaggctatggctttccttgaagaatcccacccaggaatatttgagaattcatgccttgaaacaatggaggttgttcaacaaacaagggtagataaactaactcaaggtcgccagacttatgattggacattaaacagaaatcaaccggcagcaactgcattggccaacaccatagaagtctttagatcgaatggcctaacagctaatgagtcaggaaggctaatagatttcttaaaggatgtaatggaatcaatgaacaaagaggaaatagagataacaacccactttcaaagaaaaaggagagtaagagacaacatgaccaagaagatggtcacgcaaagaacaatagggaagaaaaaacaaagactgaataagagaggctatctaataagagcactgacattaaatacgatgaccaaagatgcagagagaggcaagttaaaaagaagggctatcgcaacacctgggatgcagattagaggtttcgtatactttgttgaaactttagctaggagcatttgcgaaaagcttgaacagtctgggctcccagtagggggcaatgaaaagaaggccaaactggccaatgttgtgagaaagatgatgactaattcacaagacacagagatttctttcacaatcactggggacaacactaagtggaatgaaaaccaaaatcctcgaatgttcctggcgatgattacatatatcaccagaaatcaacccgagtggttcagaaacatcctgagcatggcacccataatgttctcaaacaaaatggcaagactagggaaagggtacatgttcgagagtaaaagaatgaagattcgaacgcaaataccagcagaaatgctagcaagcattgacctgaagtacttcaatgaatcaacaaagaagaaaattgagaaaataaggcctcttttaatagatggcacagcatcactgagccctgggatgatgatgggcatgttcaacatgctaagtacggtcttgggagtctcgatactgaatcttggacaaaagaaatacaccaagacaatatactggtgggatgggctccaatcatccgacgattttgctctcatagtgaatgcaccaaaccatgagggaatacaagcaggagtggacagattttacaggacctgcaagttagtgggaatcaacatgagcaaaaagaagtcctatataaataagacagggacatttgaattcacaagctttttttatcgctatggatttgtggctaattttagcatggagctacccagctttggagtgtctggagtaaatgaatcagctgacatgagtattggagtaacagtgataaagaacaacatgataaacaatgaccttggacctgcaactgcccagatggctcttcaattgttcatcaaagactacagatacacatataggtgccataggggagacacacaaattcagacgagaagatcatttgagttaaagaagctgtgggatcaaacccaatcaaaggtagggctattagtatcagatggaggaccaaacttatacaatatacggaatcttcacattcctgaagtctgcttaaaatgggagctaatggatgatgattatcggggaagactttgtaatcccctgaatccctttgtaagtcataaagagattgattctgtaaacaatgctgtgataatgccagctcatggtccagccaaaagcatggaatatgatgccgttgcaactacacattcctggattcccaagaggaatcgttctattctcaacacaagccaaaggggaattcttgaggatgaacagatgtaccagaagtgctgcaatctattcgagaaatttttccctagcagttcatataggagaccggttggaatttctagcatggtggaggccatggtgtctagggcccggattgatgccagggtcgactttgagtctggacggatcaagaaagaagagttctctgagatcatgaagatctgttccaccattgaagaactcagacggcaaaaataa

>H1N1_PB1_CY167474_A/Tennessee/F2005A/2010

atggatgtcaatccgactctacttttcctaaaaattccagcgcaaaatgccataagcaccacattcccttatactggagatcctccatacagccatggaacaggaacaggatacaccatggacacagtaaacagaacacaccaatactcagaaaagggaaagtggacgacaaacacagagactggtgcaccccagctcaacccgattgatggaccactacctgaggataatgaaccaagtgggtatgcacaaacagactgtgttctagaggctatggctttccttgaagaatctcacccaggaatatttgagaattcatgccttgaaacaatggaagttgttcaacaaacaagggtagacaaactgactcaaggtcgccagacttatgattggacattaaacagaaatcaaccggcagcaactgcattggccaacaccatagaagtctttagatcgaatggcctaacagctaatgagtcaggaaggctaatagatttcttaaaggatgtaatggaatcaatgaacaaagaggaaatagagataacaacccactttcaaagaaaaaggagagtaagagacaacatgaccaagaagatggtcacgcaaagaacaatagggaagaaaaaacagagactgaataagagaggctatctaataagagcactgacattaaatacgatgaccaaagatgcagagagaggcaagttaaaaagaagggctatcgcaacacctgggatgcagattagaggtttcgtatactttgttgaaactttagctaggagcatttgcgaaaagcttgaacagtctgggctcccagtagggggcaatgaaaagaaggccaaactggcaaatgttgtgagaaagatgatgactaattcacaagacacagagatttctttcacaatcactggggacaacactaagtggaatgaaaatcaaaatcctcgaatgttcctggcgatgattacatatatcaccagaaatcaacccgagtggtttagaaacatcctgagcatggcacccataatgttctcaaacaaaatggcaagaatagggaaagggtacatgttcgagagtaaaagaatgaagattcgaacacaaataccagcagaaatgctagcaagcattgacctgaagtacttcaatgaatcaacaaagaagaaaattgagaaaataaggcctcttttaatggatggcacagcatcactgagtcctgggatgatgatgggcatgttcaacatgctaagtacggtcttgggagtctcgatactgaatcttggacaaaagaaatacaccaagacaacatactggtgggatgggctccaatcatccgacgattttgctctcatagtgaatgcacaaaaccatgagggaatacaagcaggagtggacagattctacaggacctgcaaattagtgggaatcaacatgagcaaaaagaagtcctatataaataagacagggacatttgaattcacaagctttttttatcgctatggatttgtggctaattttagcatggagctacccagctttggagtgtctggagtaaatgaatcagctgacatgagtattggagtaacagtgataaagaacaacatgataaacaatgaccttggacctgcaacggcccagatggctcttcaattgttcatcaaagactacagatacacatataggtgccataggggagacacacaaattcagacgagaagatcatttgagttaaagaagctgtgggatcaaacccaatcaaaggtagggctattagtatcagatggaggaccaaacttatacaatatacggaatcttcacattcctgaagtctgcttaaaatgggagctaatggatgatgattatcggggaagactttgtaatcccctgaatccctttgtaagtcataaggagattgattctgtaaacaatgctgtggtaatgccagcccatggtccagccaaaagcatggaatatgatgccgttgcaactacacattcctggattcccaagaggaatcgttccattctcaacacaagccaaaggggaattcttgaggatgaacagatgtaccagaagtgctgcaatctattcgagaaatttttccctagcagttcatataggagaccggttggaatttctagcatggtggaggccatggtgtctagggcccggattgatgccagggtcgactttgagtctggacggatcaagaaagaagagttctctgagatcatgaagatctgttccaccattgaagaactcagacggcaaaaataa

>H1N1_PB1_CY173453_A/New York/1179/2009

atggatgtcaatccgacattacttttcttaaaagtgccagcacaaaatgctataagcacaacttttccttatactggtgaccctccttacagccatgggacaggaacagggtacaccatggatacagtcaataggacacaccagtactcagaaagaggaagatggacaaaaaataccgaaacgggagcaccgcaacttaacccaattgatggccccttaccggaagacaatgaaccaagtggctatgcccaaacagattgtgtattagaagcaatggctttccttgaagaatcccatcccggtatctttgaaaactcttgtattgaaacaatggaggttgttcaacaaacaagggtggacaaactgacacaaggcagacagacctatgactggactctaaataggaaccagcctgctgccacagcattggcaaacactatagaagtattcagatcaaacggcctcatagcaaatgaatctggaaggctaatagacttccttaaagatgtaatggagtcgatggacagaggcgaagtagaggtcacaactcattttcaaagaaagaggagagtgagagacaatgtaactaaaaaaatggtgacccaaagaacaataggcaaaaagaaacataaattagacaaaagaagttacctaattagggcattaaccctgaacacaatgaccaaagatgctgagagggggaaactaaaacgcagagcaattgcaaccccaggaatgcaaataagggggtttgtatactttgttgagacactggcaagaagcatatgtgaaaagcttgaacaatcaggattgccagttggaggaaatgagaagaaagcaaagttagcaaatgttgtaaggaagatgatgaccaactcccaggacactgaaatttctttcaccataaccggagacaacacaaaatggaacgaaaatcaaaaccctagaatgttcttggccatgatcacatatataaccaaaaatcagcctaaatggttcagaaatattctaagtattgctccaataatgttttcaaacaagatggcgagactaggtaaggggtacatgtttgaaagcaagagtatgaaactgagaactcaaatacctgcagagatgctagccaacatagacttgaaatatttcaatgattcaactaaaaagaaaattgaaaaaatccgaccattattaatagatggaactgcatcattgagtcctggaatgatgatgggcatgttcaatatgttgagcaccgtcctgggcgtctccattctgaatcttgggcaaaagagatacaccaagactacttactggtgggatggtcttcaatcgtctgatgattttgctttgattgtgaacgcacccaactatgcaggaattcaagctggagttgacaggttctatcgaacctgtaagctactcggaattaatatgagcaaaaagaagtcttacataaacagaacaggtacctttgaattcacgagctttttctatcgttatgggtttgttgccaatttcagcatggagcttcctagttttggggtgtctggggtcaatgaatctgcagacatgagtattggagtcactgtcatcaaaaacaatatgataaacaatgaccttggcccagcaactgctcaaatggcccttcagttatttataaaagattacaggtacacttatcgatgccaccgaggtgacacacaaatacaaacccgaagatcatttgagataaagaaactatgggaccaaacccgctcaaaagctggactgttggtctctgatggaggccccaatttgtataacattagaaatctccatattcctgaagtttgcttgaaatgggagttgatggatgaggattaccaggggcgtttatgcaacccattaaacccgtttgtcagccataaagagattgaatcagtgaacagtgcagtgataatgccggcacatggtccagccaaaaatatggagtatgacgctgttgcaacaacacactcctgggtccccaaaagaaatcgatccattttgaacacgagccaaagggggatacttgaagatgagcaaatgtatcagaggtgctgcaatttatttgaaaaattcttcccaagtagctcatacagaagaccagttggaatatccagtatggtagaggctatggtctcaagagcccgaattgatgcacggattgatttcgaatctggaaggataaagaaagaggaatttgctgagatcatgaagatctgttccaccattgaagacctcagacggcaaaaatga

>H1N1_PB1_CY173493_A/New York/1184/2009

atggatgtcaatccgacattacttttcttaaaagtgccagcacaaaatgctataagcacaacttttccttatactggtgaccctccttacagccatgggacaggaacagggtacaccatggatacagtcaacaggacacaccagtactcagaaagaggaagatggacaaaaaacaccgaaacgggagcaccgcaacttaacccaattgatggtcccttaccggaagacaatgaaccaagtggctatgcccaaacagattgtgtattagaagcaatggctttccttgaagaatcccatcccggtatctttgaaaactcttgtattgaaacaatggaggttgttcaacaaacaagggtggacaaactgacacaaggcagacagacctatgactggactctaaataggaaccagcctgctgccacagcattggcaaacactatagaagtattcagatcaaacggcctcatagcaaatgaatctgggaggctaatagacttccttaaagatgtaatggagtcgatggacagaggcgaagtagagatcacaactcattttcaaagaaagaggagagtgagagacaatgtaactaaaaaaatggtgacccaaagaacaataggcaaaaagaaacataaattagacaaaagaagttacctgattagggcattaaccctgaacacaatgaccaaagatgctgagagggggaaactaaaacgcagagcaattgcaaccccaggaatgcaaataagggggtttgtgtactttgttgagacactggcaagaaacatatgtgaaaagcttgaacaatcaggattgccagttggaggaaatgagaagaaagcaaagttagcaaatgttgtaaggaagatgatgaccaactcccaggacactgaaatttctttcaccataaccggagataacacaaaatggaacgaaaatcaaaaccctagaatgttcttggccatgatcacatatataaccaaaaatcagcctgaatggttcagaaatattctaagtattgctccaataatgttttcaaacaagatggcgagactaggtaaggggtacatgtttgaaagcaagagtatgaaactgagaactcaaatacctgcagagatgctagccaacatagacttgaaatatttcaatgattcaactaaaaagaaaattgaaaaaatccgaccattattaatagatggaactgcatcattgagtcctggaatgatgatgggcatgttcaatatgttgagcaccgtcttgggcgtctccattctgaatcttgggcaaaagagatacaccaagactacttactggtgggatggtcttcaatcgtctgatgattttgctttgattgtgaacgcacccaactatgcaggaattcaagctggagttgataggttttatcgaacctgtaagctgctcggaattaatatgagcaaaaagaagtcttacataaacagaacaggtacctttgaattcacgagctttttctatcgttatgggtttgttgccaatttcagcatggagcttcctagttttggggtgtctggggtcaatgaatctgcagacatgagtattggagtcactgtcatcaaaaacaacatgataaacaatgaccttggcccagcaactgctcaaatggcccttcagttatttataaaagattacaggtacacttatcgatgccaccgaggtgacacacaaatacaaacccggagatcatttgagataaagaaactatgggaccaaacccgctcaaaagctgggctgttggtctctgatggaggccccaatttgtataacattagaaatctccatattcctgaagtttgcttgaaatgggagttgatggatgaggattaccaggggcgtttatgcaacccattaaacccgtttgtcagccataaagagattgaatcagtgaacaatgcagtgataatgccggcacatggtccagccaaaaatatggagtatgacgctgttgcaacaacacactcctgggtccccaaaagaaatcgatccattttgaacacgagccaaagggggatacttgaagatgagcaaatgtatcagaggtgctgcaatttatttgaaaaattcttcccaagtagctcatacagaagaccagttggaatatccagtatggtagaggctatggtctcaagagcccgaattgatgcacggattgatttcgaatctggaaggataaagaaagaggaatttgctgagatcatgaagatctgttccaccattgaagacctcagacggcagaaatga

>H1N1_PB1_KJ856065_A/Sri Lanka/2005/2009

atggatgtcaatccgactctacttttcctaaaaattccagcgcaaaatgccataagcaccacattcccttatactggagatcctccatacagccatggaacaggaacaggatacaccatggacacagtaaacagaacacaccaatactcagaaaagggaaagtggacgacaaacacagagactggtgcaccccagctcaacccgattgatggaccactacctgaggataatgaaccaagtgggtatgcacaaacagactgtgttctagaggctatggctttccttgaagaatcccacccaggaatatttgagaattcatgccttgaaacaatggaagttgttcaacaaacaagggtagataaactaactcaaggtcgccagacttatgattggacattaaacagaaatcaaccggcagcaactgcattggccaacaccatagaagtctttagatcgaatggcctaacagctaatgagtcaggaaggctaatagatttcttaaaggatgtaatggaatcaatgaacaaagaggaaatagagataacaacccactttcaaagaaaaaggagagtaagagacaacatgaccaagaagatggtcacgcaaagaacaatagggaagaaaaaacaaagactgaataagagaggctatctaataagagcactgacattaaatacgatgaccaaagatgcagagagaggcaagttaaaaagaagggctatcgcaacacctgggatgcagattagaggtttcgtatactttgttgaaactttagctaggagcatttgcgaaaagcttgaacagtctgggctcccagtagggggcaatgaaaagaaggccaaactggcaaatgttgtgagaaagatgatgactaattcacaagacacagagatttctttcacaatcactggggacaacactaagtggaatgaaaatcaaaatcctcgaatgttcctggcgatgattacatatatcaccagaaatcaacccgagtggttcagaaacatcctgagcatggcacccataatgttctcaaacaaaatggcaagactagggaaagggtacatgttcgagagtaaaagaatgaagattcgaacacaaataccggcagagatgctagcaagcattgacctgaagtacttcaatgaatcaacaaagaagaaaattgagaaaataaggcctcttctaatagatggcacagcatcactgagtcctgggatgatgatgggcatgttcaacatgctaagtacggtcttgggagtctcgatactgaatcttggacaaaagaaatacaccaagacaatatactggtgggatgggctccaatcatccgacgattttgctctcatagtgaatgcaccaaaccatgagggaatacaagcaggagtggacagattctacaggacctgcaagttagtgggaatcaacatgagcaaaaagaagtcctatataaataagacagggacatttgaattcacaagctttttttatcgctatggatttgtggctaattttagcatggagctacccagctttggagtgtctggagtaaatgaatcagctgacatgagtattggagtaacagtgataaagaacaacatgataaacaatgaccttggacctgcaacggcccagatggctcttcaattgttcatcaaagactacagatacacatataggtgccataggggagacacacaaattcagacgagaagatcatttgagttaaagaagctgtgggatcaaacccaatcaaaggtagggctattagtatcagatggaggaccaaacttatacaatatacggaatcttcacattcctgaagtctgcttaaaatgggagctaatggatgatgattatcggggaagactttgtaatcccctgaatccctttgtcagtcataaagagattgattctgtaaacaatgctgtggtaatgccagcccatggtccagccaaaagcatggaatatgatgccgttgcaactacacattcctggattcccaagaggaatcgttctattctcaacacaagccaaaggggaattcttgaggatgaacagatgtaccagaagtgctgcaatctattcgagaaatttttccctagcagttcatataggagaccggttggaatttctagcatggtggaggccatggtgtctagggcccggattgatgccagggtcgacttcgagtctggacggatcaagaaagaagagttctctgagatcatgaagatctgttccaccattgaagaactcagacggcaaaaataa

>H1N1_PB1_CY189055_A/New York/WC-LVD-14-001/2014

atggatgtcaatccgactctacttttcctaaaaattccagcacaaaatgccataagcaccacattcccttatactggagatcctccatacagccatggaacaggaacaggatacaccatggacacagtaaacagaacacaccaatactcagaaaaggggaagtggacaacaaacacagagactggtgcaccccagctcaacccgattgatggaccactacctgaggataatgaaccaagtgggtatgcacaaacagactgtgttctagaggctatggctttccttgaagaatcccacccaggaatatttgagaattcatgccttgaaacaatggaaattgttcaacaaacaagggtagataaactgactcaaggtcgccagacttatgattggacattaaacagaaatcaaccggcagcaactgcattggccaacaccatagaagtctttagatcgaatgacctaacagctaacgagtcaggaaggctaatagatttcttaaaggatgtaatggaatcaatgaacaaagaggaaatagagataacaacccactttcaaagaaaaaggagagtaagagacaacatgaccaagaagatggtcactcaaagaacaatagggaagaaaaaacagagactgaataagagaggctatctaataagagcactgacattaaatacgatgaccaaagatgcagagagaggcaagttaaaaagaagggctatcgcaacacctgggatgcagattagaggtttcgtatactttgttgaaactttagctaggagcatttgcgaaaagcttgaacagtctgggctcccagtagggggcaatgaaaagaaggccaaattggcaaatgttgtgagaaagatgatgactaattcacaagatacagagatttctttcacaatcactggggacaacactaagtggaatgaaaatcaaaatcctcgaatgttcctggcgatgattacatatatcaccagaaatcaacccgagtggtttagaaacatcctgagcatggcacccataatgttctcaaataaaatggcaagactagggaaagggtacatgttcgagagtaaaagaatgaagattcgaacacaaataccagcagaaatgctagcaagcattgatctgaagtacttcaatgaatcaacaaagaagaaaattgagaaaataaggccccttttaatggatggcacagcatcactgagtcctgggatgatgatgggcatgttcaacatgctaagtacggtcttgggagtctcgatactgaatcttggacaaaagaaatacaccaagacaacatactggtgggatgggctccaatcatccgacgattttgctctcatagtgaatgcaccaaaccatgaaggaatacaagcaggagtggacagattctacaggacctgcaagttagtgggaatcaacatgagcaaaaagaagtcctatataaataagacagggacatttgaattcacaagctttttttatcgctatggatttgtggctaattttagcatggagctacccagctttggagtgtctggagtaaatgaatcagctgatatgagtattggagtaacagtgataaagaacaacatgataaacaatgacctcggacctgcaacggcccagatggctcttcaattgttcatcaaagattacagatacacatataggtgccataggggagacacacaaattcagacgagaagatcatttgagttaaggaagctatgggatcaaacccaatcaaaggtagggctattagtatcagatggaggaccaaacttatacaacatacggaatcttcacattcctgaagtctgcttaaaatgggagctaatggatgatgattatcggggaagactttgtaatcccctgaatccctttgtgagtcataaggagattgattctgtaaacaatgctgtggtaatgccagcccatggtccagccaaaagcatggaatatgatgccgttgcaactacacattcttggattcccaagaggaatcgttctattctcaacacaagccaaaggggaattcttgaggatgaacagatgtaccagaagtgttgcaatctattcgagaaatttttccctagcagttcatataggagaccagttggaatttctagcatggtggaggccatggtgtctagggcccggattgatgccagggtcgactttgagtctggacggatcaagaaagaagagttctctgagatcatgaagatctgttccaccattgaagaactcagacggcaaaaataa

>H1N1_PB1_CY189287_A/New York/WC-LVD-14-030/2014

atggatgtcaatccgactctacttttcctaaagattccagcacaaaatgccataagcaccacattcccttatactggagatcctccatacagccatggaacaggaacaggatacaccatggacacagtaaacagaacacaccaatactcagaaaaggggaagtggacaacaaacacagagactggtgcaccccagctcaacccgattgatggaccactacctgaggataatgaaccaagtgggtatgcacaaacagactgtgttctagaggctatggctttccttgaagaatcccacccaggaatatttgagaattcatgccttgaaacaatggaaattgttcaacaaacaagggtagataaactgactcaaggtcgccagacttatgattggacattaaacagaaatcaaccggcagcaactgcattggccaacaccatagaagtctttagatcgaatgacctaacagctaacgagtcaggaaggctaatagatttcttaaaggatgtaatggaatcaatgaacaaagaggaaatagagataacaacccactttcaaagaaaaaggagagtaagagacaacatgaccaagaagatggtcactcaaagaacaatagggaagaaaaaacagagactgaataagagaggctatctaataagagcactgacattaaatacgatgaccaaagatgcagagagaggcaagttaaaaagaagagctatcgcaacacctgggatgcaaattagaggtttcgtatactttgttgaaactttagctaggagcatttgcgaaaagcttgaacagtctgggctcccagtagggggcaatgaaaagaaggccaaattggcaaatgttgtgagaaagatgatgactaattcacaagatacagagatttctttcacaatcactggggacaacactaagtggaatgaaaatcaaaatcctcgaatgttcctggcgatgattacatatatcaccagaaatcaacccgagtggtttagaaacatcctgagcatggcacccataatgttctcaaataaaatggcaagactagggaaagggtacatgttcgagagtaaaagaatgaagattcgaacacaaataccagcagaaatgctagcaagcattgatctgaagtacttcaatgaatcaacaaagaagaaaattgagaaaataaggcctcttttaatggatggcacagcatcactgagtcctgggatgatgatgggcatgttcaacatgctaagtacggtcttgggagtctcgatactgaatcttggacaaaagaaatacaccaagacaacatactggtgggatgggctccaatcatccgacgattttgctctcatagtgaatgcaccaaaccatgaaggaatacaagcaggagtggacagattctacaggacctgcaagttagtgggaatcaacatgagcaaaaagaagtcctatataaataagacagggacatttgaattcacaagctttttttatcgctatggatttgtggctaattttagcatggagctacccagctttggagtgtctggggtaaatgaatcagctgacatgagtattggagtaacagtgataaagaacaacatgataaacaatgacctcggacctgcaacggcccagatggctcttcaattgttcatcaaagactacagatacacatataggtgccataggggagacacacaaattcagacgagaagatcatttgagttaaagaagctatgggatcaaacccaatcaaaggtagggctattagtatcagatggaggaccaaacttatacaacatacggaatcttcacattcctgaagtctgcttaaaatgggagctaatggatgatgattatcggggaagactttgtaatcccctgaatccctttgtgagtcataaggagattgattctgtaaacaatgctgtggtaatgccagcccatggtccagccaaaagcatggaatatgatgccgttgcaactacacattcctggattcccaagaggaatcgttctattctcaacacaagccaaaggggaattcttgaggatgaacagatgtaccagaagtgttgcaatctattcgagaaatttttccctagcagttcatataggagaccggttggaatttctagcatggtggaggccatggtgtctagggccaggattgatgccagggtcgactttgagtctggacggatcaagaaagaagagttctctgagatcatgaagatctgttccaccattgaagaactcagacggcaaaaataa

>H1N1_PB1_KP637444_A/Thailand/MR11669/2012

atggatgtcaatccgactctacttttcctaaaaattccagcgcaaaatgccataagcaccacattcccttatactggagatcctccatacagccatggaacaggaacaggatacaccatggacacagtaaacagaacacaccaatactcagaaaagggaaagtggacgacaaacacagagactggtgcaccccagctcaacccgattgatggaccactacctgaggataatgaaccaagtgggtatgcacaaacagactgtgttctagaggctatggctttccttgaagaatcccacccaggaatatttgagaattcatgccttgaaacaatggaagttgttcaacaaacaagggtagacaaactaactcaaggtcgccagacttatgattggacattaaacagaaatcaaccggcagcaactgcattggccaacaccatagaagtctttagatcgaatggcctaacagctaatgagtcaggaaggctaatagatttcttaaaggatgtaatggaatcaatgaacaaagaggaaatagagataataacccactttcaaagaaaaaggagagtaagagacaacatgaccaagaagatggtcacgcaaagaacaatagggaagaaaaaacaaagactgaataagagaggctatctaataagagcactgacattaaatacgatgaccaaagatgcagagagaggcaagttaaaaagaagggctatcgcaacacctggaatgcagattagaggtttcgtatactttgttgaaactttagctaggagcatttgcgaaaagcttgaacagtctgggctcccagtagggggcaatgaaaagaaggccaaactggcaaatgttgtgagaaagatgatgactaattcacaagacacagagatctctttcacaatcactggggacaacactaagtggaatgaaaaccaaaatcctcgaatgttcctggcgatgattacatatatcaccagaaatcaacccgagtggttcagaaacatcctgagcatggcacccataatgttctcaaacaaaatggcaagactagggaaagggtacatgttcgagagtaaaagaatgaagattcgaacgcaaataccagcagaaatgctagcaagcattgacctgaagtacttcaatgaatcaacaaagaagaaaattgagaaaataaggcctcttttaatagatggcacagcatcactgagtcctgggatgatgatgggcatgttcaacatgctaagtacggtcttgggagtctcgatactgaatcttggacaaaagaaatacaccaagacaatatactggtgggatgggctccaatcatccgacgattttgctctcatagtgaatgcaccaaaccatgagggaatacaagcaggagtggacagattttacaggacctgcaagttagtgggaatcaacatgagcaaaaagaagtcctatataaataagacagggacatttgaattcacaagctttttttatcgctatggatttgtggctaattttagcatggagctacccagctttggagtgtctggagtaaatgaatcagctgacatgagtattggagtaacagtgataaagaacaacatgataaacaatgaccttggacctgcaacggcccagatggctcttcaattgttcatcaaagactacagatacacatataggtgccataggggagacacacaaattcagacgagaagatcatttgagttaaagaagctgtgggatcaaacccaatcaaaagtagggctattagtatcagatggaggaccaaacttatacaatatacggaatcttcacattcctgaagtctgcttaaaatgggagctaatggatgatgattatcggggaagactttgtaatcccctgaatccctttgtaagtcataaagagattgattctgtaaacaatgctgtggtaatgccagcccatggtccagccaaaagcatggaatatgatgccgttgcaactacacattcctggattcccaagaggaatcgttctattctcaacacaagccaaaggggaattcttgaggatgaacagatgtaccagaagtgctgcaatctattcgagaaatttttccctagcagttcatataggagaccggttggaatttctagcatggtggaggccatggtgtctagggcccggattgatgccagggtcgactttgagtctggacggatcaagaaagaagagttctctgagatcatgaagatctgttccaccattgaagaactcagacggcagaaataa

>H1N1_PB1_KT180439_A/Singapore/DMS141/2010

atggatgtcaatccgactctacttttcctaaaaattccagcgcaaaatgccataagcaccacattcccttatactggagatcctccatacagccatggaacaggaacaggatacaccatggacacagtaaacagaacacaccaatactcagaaaagggaaagtggacgacaaacacagagactggtgcaccccagctcaacccgattgatggaccactacctgaggataatgaaccaagtgggtatgcacaaacagactgtgttctagaggctatggctttccttgaagaatcccacccaggaatatttgagaattcatgccttgaaacaatggaagttgttcaacaaacaagggtagataaactaactcaaggtcgccagacttatgattggacattaaacagaaatcaaccggcagcaactgcattggccaacaccatagaagtctttagatcgaatggcctaacagctaatgagtcaggaaggctaatagatttcttaaaggatgtaatggaatcaatgaacaaagaggaaatagagataacaacccactttcaaagaaaaaggagagtaagagacaacatgaccaagaagatggtcacgcaaagaacaatagggaagaaaaaacaaagactgaataagagaggctatctaataagagcactgacattaaatacgatgaccaaagatgcagagagaggcaagttaaaaagaagggctatcgcaacacctgggatgcagattagaggtttcgtatactttgttgaaactttagctaggagcatttgcgaaaagcttgaacagtctgggctcccagtagggggcaatgaaaagaaggccaaactggcaaatgttgtgagaaagatgatgactaattcacaagacacagagatttctttcacaatcactggggacaacactaagtggaatgaaaatcaaaatcctcgaatgttcctggcgatgattacatatatcaccagaaatcaacccgagtggttcagaaacatcctgagcatggcacccataatgttctcaaacaaaatggcaagactagggaaagggtacatgttcgagagtaaaagaatgaagattcgaacacaaataccagcagaaatgctagcaagcattgacctgaagtacttcaatgaatcaacaaagaagaaaattgagaaaataaggcctcttttaatagatggcacagcatcactgagtcctgggatgatgatgggcatgttcaacatgctaagtacggtcttgggagtctcgatactgaatcttggacaaaagaaatacaccaagacaatatactggtgggatgggctccaatcatccgacgattttgctctcatagtgaatgcaccaaaccatgagggaatacaagcaggagtggacagattctacaggacctgcaagttagtgggaatcaacatgagcaaaaagaagtcctatataaataagacagggacatttgaattcacaagctttttttatcgctatggatttgtggctaattttagcatggagctacccagctttggagtgtctggagtaaatgaatcagctgacatgagtattggagtaacagtgataaagaacaacatgataaacaatgaccttggacctgcaacggcccagatggctcttcaattgttcatcaaagactacagatacacatataggtgccataggggagacacacaaattcagacgagaagatcatttgagttaaagaagctgtgggatcaaacccaatcaaaggtagggctattagtatcagatggaggaccaaacttatacaatatacggaatcttcacattcctgaagtctgcttaaaatgggagctaatggatgatgattatcggggaagactttgtaatcccctgaatccctttgtaagtcataaagagattgattctgtaaacaatgctgtggtaatgccagcccatggtccagccaaaagcatggagtatgatgccgttgcaactacacattcctggattcccaagaggaatcgttctattctcaacacaagccaaaggggaattcttgaggatgaacagatgtaccagaagtgctgcaatctattcgagaaatttttccctagcagttcatataggagaccggttggaatttctagcatggtggaggccatggtgtctagggcccggattgatgccagggtcgactttgagtctggacggatcaagaaagaagagttctctgagatcatgaagatctgttccaccattgaagaactcagacggcaaaaataa

>H1N1_PB1_KT180374_A/Singapore/DMS1183/2011

atggatgtcaatccgactctacttttcctaaaaattccagcgcaaaatgccataagcaccacattcccttatactggagatcctccatacagccatggaacaggaacaggatacaccatggacacagtaaacagaacacaccaatactcagaaaagggaaggtggacgacaaacacagagactggtgcaccccagctcaacccgattgatggaccactacctgaggataatgaaccaagtgggtatgcacaaacagactgtgttctagaggctatggctttccttgaagaatcccacccaggaatatttgagaattcatgccttgaaacaatggaagctgttcaacaaacaagggtagataaactaactcaaggtcgccagacttatgattggacattgaacagaaatcaaccggcagcaactgcattggccaacaccatagaagtctttagatcgaatggcctaacagctaatgagtcaggaaggctaatagatttcttaaaggatgtaatgaaatcaatgaacaaagaggaaatagagataacaacccactttcaaagaaaaaggagagtaagagacaacatgaccaagaagatggtcacgcaaagaacaataggaaagaaaaagcaaagactgaataagagaggctatctaataagagcactgacattaaatacgatgaccaaagatgcagagagaggcaagttaaaaagaagggctatcgcaacacctgggatgcagattagaggtttcgtatactttgttgaaactttagctaggagcatttgcgaaaagcttgaacagtctgggctcccagtagggggcaatgaaaagaaggccaaactggcaaatgttgtgagaaagatgatgactaattcacaagacacagagatttctttcacaatcactggggacaacactaagtggaatgaaaatcaaaatcctcgaatgttcctggcgatgattacatatatcaccagaaatcaacccgagtggttcagaaacatcctgagcatggcacccataatgttctcaaacaaaatggcaagactagggaaagggtacatgttcgagagtaaaagaatgaagattcgaacacaaattccagcagaaatgctagcaagcattgacctgaagtacttcaatgaatcaacaaagaggaaaattgagaaaataaggcctcttctaatagatggcacagcatcactgagtcctgggatgatgatgggcatgttcaacatgctaagtacggtcttgggagtctcgatactgaatcttggacaaaagaaatacaccaagacaacatactggtgggatgggctccaatcatccgacgattttgctctcatagtgaatgcaccaaaccatgagggaatacaagcaggagtggacagattctacaggacctgcaagttagtgggaatcaacatgagcaaaaagaagtcatatataaataaaacagggacatttgaattcacaagctttttttatcgctatggatttgtggctaattttagcatggagctacccagctttggagtgtctggagtaaatgaatcagctgacatgagtattggagtaacagtgataaagaacaacatgataaacaatgaccttggacctgcaacggcccagatggctcttcaattgttcatcaaagactacagatacacatataggtgccataggggagacacacaaattcagacgagaagatcatttgagttaaaaaagctgtgggatcaaacccaatcaaaggtagggctattagtatcagatggaggaccaaacttatacaatatacggaatcttcacattcctgaagtctgcttaaaatgggagctaatggatgatgattatcggggaagactttgcaatcccctgaatccctttgtcagtcataaagagattgattctgtaaacaatgctgtggtaatgccagcccatggtccagccaaaagcatggaatatgatgccgttgcaactacacattcctggattcccaagaggaatcgttctattctcaacacaagccaaaggggaattcttgaggatgaacagatgtaccagaagtgctgcaatctattcgagaaatttttccctagcagttcatataggagaccggttggaatttctagcatggtggaggccatggtgtctagggcccggattgatgccagggtcgacttcgagtctggacggatcaagaaagaagagttctctgagatcatgaagatctgttctaccattgaagaactcagacggcaaaaataa

>H1N1_PB1_KX004459_A/Maryland/24/2015

atggatgtcaatccgactctacttttcctaaaaattccagcacaaaatgccataagcaccacattcccttatactggagatcctccatacagccatggaacaggaacaggatacaccatggacacagtaaacagaacacaccaatactcagaaaaggggaagtggacaacaaacacagagactggtgcaccccagctcaacccgattgacggaccactacctgaagataatgaaccaagtgggtatgcacaaacagactgtgttctagaggctatggcttttcttgaagaatcccatccaggaatatttgagaattcatgccttgaaacaatggaagttgttcaacaaacaagggtagataaactgactcaaggtcgccagacttatgattggacattaaacagaaatcaaccggcagcaactgcattggccaacaccatagaagtttttagatcgaatgacctaacagctaacgagtcaggaaggctaatagatttcttaaaggatgtgatggaatcaatgaacaaagaggaaatagagataacaacccactttcaaagaaaaaggagagtaagagacaacatgaccaagaagatggtcacgcaaagaacaatagggaagaaaaaacagagactgaataagagaggctatctaataagagcactgacattaaatacgatgaccaaagatgcagagagaggcaagttaaaaagaagggctatcgcaacacctgggatgcagattagaggtttcgtatactttgttgaaactttagctaggagcatttgcgaaaagcttgaacagtctgggctcccagtagggggcaatgaaaagaaggccaaattggcaaatgttgtgagaaagatgatgactaattcacaagatacagagatttctttcacaatcactggggacaacactaagtggaatgaaaatcaaaatcctcgaatgttcctggcgatgattacatatatcaccagaaatcaacccgagtggtttagaaacatcctgagcatggcacccataatgttctcaaataaaatggcaagactaggaaaagggtacatgttcgagagtaaaagaatgaagattcgaacacaaataccagcagaaatgctagcaagcattgatctgaagtatttcaatgaatcaacaaagaagaaaattgagaaaataaggcctcttttaatggatggcacagcatcactgagtcctgggatgatgatgggcatgttcaacatgctaagtacggtcttgggagtctcgatactgaatcttggacaaaagaaatacaccaagacaacatactggtgggatgggctccaatcatccgacgattttgctctcatagtgaatgcaccaaaccatgaaggaatacaagcaggagtggacagattctacaggacctgcaagttagtgggaatcaacatgagcaaaaagaagtcctatataaataagacagggacatttgaattcacaagctttttttatcgctatggatttgtggctaattttagcatggagctacccagctttggagtgtctggagtaaatgaatcagctgacatgagtattggagtaacagtgataaagaacaacatgataaacaatgaccttggacctgcaacggctcagatggctcttcaattgttcatcaaagactacagatacacatataggtgccataggggagacacacaaattcagacgagaagatcatttgagttgaagaagctatgggatcaaacccaatcaaaggtagggctattagtatcagatggaggaccaaacttatacaacatacggaatcttcacattcctgaagtctgcttaaaatgggagctaatggatgatgattatcggggaagactttgtaatcccctgaatccttttgtgagtcataaggagattgattctgtaaacaatgctgtggtaatgccagcccatggcccagccaaaagcatggaatatgatgccgttgctactacacattcctggattcccaagaggaatcgttctattctcaacacaagccaaaggggaattcttgaggatgaacagatgtaccagaagtgttgcaatttattcgagaaatttttccctagcagttcatataggagaccggttggaatttctagcatggtggaggccatggtgtctagggcccggattgatgccagagtcgactttgagtctggacggatcaagaaagaagagttctctgagatcatgaagatctgttccaccattgaagaactcagacggcaaaaataa

>H1N1_PB1_KX004976_A/Hawaii/01/2016

atggatgtcaatccgactctacttttcctaaaaattccagcacaaaatgccataagcaccacattcccttatactggagatcctccatacagccatggaacaggaacaggatacaccatggacacagtaaacagaacacaccaatactcagaaaaggggaagtggacaacaaacacagagactggtgcaccccagctcaacccgattgacggaccactacctgaagataatgaaccaagtgggtatgcacaaacagactgtgttctagaggctatggcttttcttgaagaatcccatccaggaatatttgagaattcatgccttgaaacaatggaagttgttcaacaaacaagggtagataaactgactcaaggtcgccagacttatgattggacattaaacagaaatcaaccggcagcaactgcattggccaacaccatagaagtttttagatcgaatgacctaacagctaacgagtcaggaaggctaatagatttcttaaaggatgtgatggaatcaatgaacaaagaggaaatagagataacaacccactttcaaagaaaaaggagagtaagagacaacatgaccaagaagatggtcacgcaaagaacaatagggaagaaaaaacagagactgaataagagaggctatctaataagagcactgacattaaatacgatgaccaaagatgcagagagaggcaagttaaaaagaagggctatcgcaacacctgggatgcagattagaggtttcgtatactttgttgaaactttagctaggagcatttgcgaaaagcttgaacagtctgggctcccagtagggggcaatgaaaagaaggccaaattggcaaatgttgtgagaaagatgatgactaattcacaagatacagagatttctttcacaatcactggggacaacactaagtggaatgaaaatcaaaatcctcgaatgttcctggcgatgattacatatatcaccagaaatcaacccgagtggtttagaaacatcctgagcatggcacccataatgttctcaaataaaatggcaagactaggaaaagggtacatgttcgagagtaaaagaatgaagattcgaacacaaataccagcagaaatgctagcaagcattgatctgaagtatttcaatgaatcaacaaagaagaaaattgagaaaataaggcctcttttaatggatggcacagcatcactgagtcctgggatgatgatgggcatgttcaacatgctaagtacggtcttgggagtctcgatactgaatcttggacaaaagaaatacaccaagacaacatactggtgggatgggctccaatcatccgacgattttgctctcatagtgaatgcaccaaaccatgaaggaatacaggcaggagtggacagattctacaggacctgcaagttagtgggaatcaacatgagcaaaaagaagtcctatataaataagacagggacatttgaattcacaagctttttttatcgctatggatttgtggctaattttagcatggagctacccagctttggagtgtctggagtaaatgaatcagctgacatgagtattggagtaacagtgataaagaacaacatgataaacaatgaccttggacctgcaacggctcagatggctcttcaattgttcatcaaagactacagatacacatataggtgccataggggagacacacaaattcagacgagaagatcatttgagttgaagaagctatgggatcaaacccaatcaaaggtagggctattagtatcagatggaggaccaaacttatacaacatacggaatcttcacattcctgaagtctgcttaaaatgggagctaatggatgatgattatcggggaagactttgtaatcccctgaatccctttgtgagtcataaggagattgattctgtaaacaatgctgtggtaatgccagcccatggcccagccaaaagcatggaatatgatgccgttgctactacacattcctggattcccaagaggaatcgttctattctcaacacaagccaaaggggaattcttgaggatgaacagatgtaccagaagtgttgcaatttattcgagaaatttttccctagcagttcatataggagaccggttggaatttctagcatggtggaggccatggtgtctagggcccggattgatgccagagtcgactttgagtctggacggatcaagaaagaagagttctctgagatcatgaagatctgttccaccattgaagaactcagacggcaaaaataa

>H1N1_PB1_KX408585_A/Georgia/11/2016

atggatgtcaatccgactctacttttcctaaaaattccagcacaaaatgccataagcaccacattcccttatactggagatcctccatacagccatggaacaggaacaggatacaccatggacacagtaaacagaacacaccaatactcagaaaaggggaagtggacaacaaacacagagactggtgcaccccagctcaacccgattgatggaccactacctgaggataatgaaccaagtgggtatgcacagacagactgtgtcctagaggctatggctttccttgaagaatcccacccaggaatatttgagaattcatgccttgaaacaatggaagttgttcaacaaacaagggtagataaactgactcaaggtcgccagacttatgattggacattaaacagaaatcaaccagcagcaactgcattggccaacaccatagaagtctttagatcgaatgacctaacagctaacgagtcaggaaggctaatagatttcttaaaggatgtaatggaatcaatgaacaaagaggaaatagagataacaacccactttcaaagaaaaaggagagtaagagacaacatgaccaagaagatggtcacgcaaagaacaatagggaagaaaaaacagagactgaataagagaggctatctaataagagcacttaccttaaatacgatgaccaaagatgcagagagaggcaagttaaaaagaagggctatcgcaacacctgggatgcagattagaggtttcgtatactttgttgaaactttagctaggagcatttgcgaaaagcttgaacagtctgggctcccagtagggggcaatgaaaagaaggccaaattggcaaatgttgtgagaaagatgatgactaattcacaagatacagagatttctttcacaatcactggggacaacactaaatggaatgaaaatcaaaatcctcgaatgttcctggcaatgattacatatatcaccagaaatcaacccgagtggtttagaaacatcctgagcatggcacccataatgttctcaaataaaatggcaagactggggaaagggtacatgttcgagagtaaaagaatgaaaattcggacacaaataccagcagaaatgctagcaagcattgatctgaagtacttcaatgaatcaacaaagaagaaaattgagaaaataaggcctcttttaatggatggcacagcatcactgagtcctgggatgatgatgggcatgttcaacatgctaagtacggtcttgggagtctcgatactgaatcttggacaaaagaaatacaccaagacaacatactggtgggatgggctccaatcatccgacgattttgctctcatagtgaatgcaccaaaccacgaaggaatacaagcaggagtggacagattctacaggacctgcaagttagtgggaatcaacatgagcaaaaagaagtcttatataaataagacagggacatttgaattcacaagctttttttatcgctatggatttgtggctaattttagcatggagctacccagctttggagtgtctggagtaaatgaatcagctgacatgagtattggagtaacagtgataaagaacaacatgataaacaatgaccttggacctgcaacggcccagatggctcttcaattgttcatcaaagactacagatacacatataggtgccacaggggagacacacaaattcagacgagaagatcatttgagttaaagaagctatgggatcaaacccaatcaaaggtagggctattagtatcagatggaggaccaaacttatacaacatacggaatcttcacattcctgaagtctgcttaaaatgggagctaatggatgatgattatcggggaagactttgtaatcccctgaatccctttgtgagtcataaggagattgattctgtaaacaatgccgtggtaatgccagcccatggtccagccaaaagcatggaatatgatgccgttgcaactacacattcctggattcccaagaggaatcgttctatcctcaacacaagccaaaggggaattcttgaggatgaacagatgtaccagaagtgttgcaatctattcgagaaatttttccctagcagttcatataggagaccggttggaatttctagcatggtggaggccatggtgtctagggcccggattgatgccagggtcgactttgagtctggacggatcaagaaagaagagttctctgagatcatgaagatctgttccaccattgaagaactcagacggcaaaaataa

>H1N1_PB1_KX408801_A/Maryland/06/2016

atggatgtcaatccgactctacttttcctgaaaattccagcacaaaatgccataagcaccacattcccttatactggagatcctccatacagccatggaacaggaacaggatacaccatggacacagtaaacagaacacaccaatactcagaaaaggggaagtggacaacaaacacagagactggtgcaccccagctcaacccgattgacggaccactacctgaagataatgaaccaagtgggtatgcacaaacagactgtgttctagaggctatggcttttcttgaagaatcccatccaggaatatttgagaattcatgccttgaaacaatggaagttgttcaacaaacaagggtagataaactgactcaaggtcgccagacttatgattggacattaaacagaaatcaaccggcagcaactgcattggccaacaccatagaagtttttagatcgaatgacctaacagctaacgagtcaggaaggctaatagatttcttaaaggatgtgatggaatcaatgaacaaagaggaaatagagataacaacccactttcaaagaaaaaggagagtaagagacaacatgaccaagaagatggtcacgcaaagaacaatagggaagaaaaaacagagactgaataagagaggctatctaataagagcactgacattaaatacgatgaccaaagatgcagagagaggcaagttaaaaagaagggctatcgcaacacctgggatgcagattagaggtttcgtatactttgttgaaactttagctaggagcatttgcgaaaagcttgaacagtctgggctcccagtagggggcaatgaaaagaaggccaaattggcaaatgttgtgagaaagatgatgactaattcacaagatacagagatttctttcacaatcactggggacaacactaagtggaatgaaaatcaaaatcctcgaatgttcctggcgatgattacatatatcaccagaaatcaacccgagtggtttagaaacatcctgagcatggcacccataatgttctcaaataaaatggcaagactaggaaaagggtacatgttcgagagtaaaagaatgaaggttcgaacacaaataccagcagaaatgctagcaagcattgatctgaagtatttcaatgaatcaacaaagaagaaaattgagaaaataaggcctcttttaatggatggcacagcatcactgagccctgggatgatgatgggcatgttcaacatgctaagtacggtcttgggagtctcgatactgaatcttggacaaaagaaatacaccaagacaacatactggtgggatgggctccaatcatccgacgattttgctctcatagtgaatgcaccaaaccatgaaggaatacaagcaggagtggacagattctacaggacctgcaagttagtgggaatcaacatgagcaaaaagaagtcctatataaataagacagggacatttgaattcacaagctttttttatcgctatggatttgtggctaattttagcatggagctacccagctttggagtgtctggagtaaatgaatcagctgacatgagtattggagtaacagtgataaagaacaacatgataaacaatgaccttggacctgcaacggctcagatggctcttcaattgttcatcaaagactacagatacacatataggtgccataggggagacacacaaattcagacgagaagatcatttgagttgaagaagctatgggatcaaacccaatcaaaggtagggctattagtatcagatggaggaccaaacttatacaacatacggaatcttcacattcctgaagtctgcttaaaatgggagctaatggatgatgattatcggggaagactttgtaatcccctgaatccctttgtgagtcataaggagattgattctgtaaacaatgctgtggtaatgccagcccatggcccagccaaaagcatggaatatgatgccgttgctactacacattcctggattcccaagaggaatcgttctattctcaacacaagccaaaggggaattcttgaggatgaacagatgtaccagaagtgttgcaatttattcgagaaatttttccctagcagttcatataggagaccggttggaatttctagcatggtggaggccatggtgtctagggcccggattgatgccagagtcgactttgagtctggacggatcaagaaagaagagttctctgagatcatgaagatctgttccaccattgaagaactcagacggcaaaaataa

>H1N1_PB1_KX409473_A/Hawaii/22/2016

atggatgtcaatccgactctacttttcctaaaaattccagcacaaaatgccataagcaccacattcccttatactggagatcctccatacagccatggaacaggaacaggatacaccatggacacagtaaacagaacacaccaatactcagaaaaggggaagtggacaacaaacacagagactggtgcaccccagctcaacccgattgacggaccactacctgaagataatgaaccaagtgggtatgcacaaacagactgtgttctagaggctatggcttttcttgaagaatcccatccaggaatatttgagaattcatgccttgaaacaatggaagttgttcaacaaacaagggtagataaactgactcaaggtcgccagacttatgattggacattaaacagaaatcaaccggcagcaactgcattggccaacaccatagaagtttttagatcgaatgacctaacagctaacgagtcaggaaggctaatagatttcttaaaggatgtgatggaatcaatgaacaaagaggaaatagagataacaacccactttcaaagaaaaaggagagtaagagacaacatgaccaagaagatggtcacgcaaagaacaatagggaagaaaaaacagagactgaataagagaggctatctaataagagcactgacattaaatacgatgaccaaagatgcagagagaggcaagttaaaaagaagggctatcgcaacacctgggatgcagattagaggtttcgtatactttgttgaaactttagctaggagcatttgcgaaaagcttgaacagtctgggctcccagtagggggcaatgaaaagaaggccaaattggcaaatgttgtgagaaagatgatgactaattcacaagatacagagatttctttcacaatcactggggacaacactaagtggaatgaaaatcaaaatcctcgaatgttcctggcgatgattacatatatcaccagaaatcaacccgagtggtttagaaacatcctgagcatggcacccataatgttctcaaataaaatggcaagactaggaaaagggtacatgttcgagagtaaaaaaatgaagattcgaacacaaataccagcagaaatgctagcaagcattgatctgaagtatttcaatgaatcaacaaagaagaaaattgagaaaataaggcctcttttaatggatggcacagcatcactgagtcctgggatgatgatgggcatgttcaacatgctaagtacggtcttgggagtctcgatactgaatcttggacaaaagaaatacaccaagacaacatactggtgggatgggctccaatcatccgacgattttgctctcatagtgaatgcaccaaaccatgaaggaatacaagcaggagtggacagattctacaggacctgcaagttagtgggaatcaacatgagcaaaaagaagtcctatataaataagacagggacatttgaattcacaagctttttttatcgctatggatttgtggctaattttagcatggagctacccagctttggagtgtctggagtaaatgaatcagctgacatgagtattggagtaacagtgataaagaacaacatgataaacaatgaccttggacctgcaacggctcagatggctcttcaattgttcatcaaagactacagatacacatataggtgccataggggagacacacaaattcaaacgagaagatcatttgagttgaagaagctatgggatcaaacccaatcaaaggtagggctattagtatcagatggaggaccaaacttatacaacatacggaatcttcacattcctgaagtctgcttaaaatgggagctaatggatgatgattatcggggaagactttgtaatcccctgaatccctttgtgagtcataaagagattgattctgtaaacaatgctgtggtaatgccagcccatggcccagccaaaagcatggaatatgatgccgttgctactacacattcctggattcccaagaggaatcgttctattctcaacacaagccaaaggggaattcttgaggatgaacagatgtaccagaagtgttgcaatttattcgagaaatttttccctagcagttcatataggagaccggttggaatttctagcatggtggaggccatggtgtctagggcccggattgatgccagagtcgactttgagtctggacggatcaagaaagaagagttctctgagatcatgaagatctgttccaccattgaagaactcagacggcaaaaataa

>H1N1_PB1_KX410497_A/Delaware/18/2016

atggatgtcaatccgactctacttttcctaaaaattccagcacaaaatgccataagcaccacattcccttatactggagatcctccatacagccatggaacaggaacaggatacaccatggacacagtaaacagaacacaccaatactcagaaaaggggaagtggacaacaaacacagagactggtgcaccccagctcaacccgattgacggaccactacctgaagataatgaaccaagtgggtatgcacaaacagactgtgttctggaggctatggcttttcttgaagaatcccatccaggaatatttgagaattcatgccttgaaacaatggaagttgttcaacaaacaagggtagataaactgactcaaggtcgccagacttatgattggacattaaacagaaatcaaccggcagcaactgcattggccaacaccatagaagtttttagatcgaatgacctaacagctaacgagtcaggaaggctaatagatttcttaaaggatgtgatggaatcaatgaacaaagaggaaatagagataacaacccactttcaaagaaaaaggagagtaagagacaacatgaccaagaagatggtcacgcaaagaacaatagggaagaaaaaacagagactgaataagagaggctatctaataagagcactgacattaaatacgatgaccaaagatgcagagagaggcaagttaaaaagaagggctatcgcaacacctgggatgcagattagaggtttcgtatactttgttgaaactttagctaggagcatttgcgaaaagcttgaacagtctgggctcccagtagggggcaatgaaaagaaggccaaattggcaaatgttgtgagaaagatgatgactaattcacaagatacagagatttctttcacaatcactggggacaacactaagtggaatgaaaatcaaaatccccgaatgttcctggcgatgattacatatatcaccagaaatcaacccgagtggtttagaaacatcctgagcatggcacccataatgttctcaaataaaatggcaagactaggaaaagggtacatgttcgagagtaaaagaatgaagattcgaacacaaataccagcagaaatgctagcaagcattgatctgaagtatttcaatgaatcaacaaagaagaaaattgagaaaataaggcctcttttaatggatggcacagcatcactgagtcctgggatgatgatgggcatgttcaacatgctaagtacggtcttgggagtctcgatactgaatcttggacaaaagaaatacaccaagacaacatactggtgggatgggctccaatcatccgacgattttgctctcatagtgaatgcaccaaaccatgaaggaatacaagcaggagtggacagattctacaggacctgcaagttagtgggaatcaacatgagcaaaaagaagtcctatataaataagacagggacatttgaattcacaagctttttttatcgctatggatttgtggctaattttagcatggagctacccagctttggagtgtctggagtaaatgaatcagctgacatgagtattggagtaacagtgataaagaacaacatgataaacaatgaccttggacctgcaacggctcagatggctcttcaattgttcatcaaagactacagatacacatataggtgccataggggagacacacaaattcagacgagaagatcatttgagttgaagaagctatgggatcaaacccaatcaaaggtagggctattagtatcagatggaggaccaaacttatacaacatacggaatcttcacattcctgaagtctgcttaaaatgggagttaatggatgatgattatcggggaagactttgtaatcccctgaatccctttgtgagtcataaggagattgattctgtaaacaatgctgtggtaatgccagcccatggcccagccaaaagcatggaatatgatgccgttgctactacacattcctggattcccaagaggaatcgttctattctcaacacaagccaaaggggaattcttgaggatgaacagatgtaccagaagtgttgcaatttattcgagaaatttttccctagcagttcatataggagaccggttggaatttctagcatggtggaggccatggtgtctagggcccggattgatgccagagtcgactttgagtctggacggatcaagaaagaagagttctctgagatcatgaagatctgttccaccattgaagaactcagacggcaaaaataa

>H1N1_PB1_KX410617_A/Maryland/10/2016

atggatgtcaatccgactctacttttcctaaaaattccagcacaaaatgccataagcaccacattcccttatactggagatcctccatacagccatggaacaggaacaggatacaccatggacacagtaaacagaacacaccaatactcagaaaaggggaagtggacaacaaacacagagactggtgcaccccagctcaacccgattgacggaccactacctgaagataatgaaccaagtgggtatgcacaaacagactgtgttctagaggctatggcttttcttgaagaatcccatccaggaatatttgagaattcatgccttgaaacaatggaagttgttcaacaaacaagggtagataaactgactcaaggtcgccagacttatgattggacattaaacagaaatcaaccggcagcaactgcattggccaacaccatagaagtttttagatcgaatgacctaacagctaacgagtcaggaaggctaatagatttcttaaaggatgtgatggaatcaacgaacaaagaggaaatagagataacaacccactttcaaagaaaaaggagagtaagagacaacatgaccaagaagatggtcacgcaaagaacaatagggaagaaaaaacagagactgaataagagaggctatctaataagagcactgacattaaatacgatgaccaaagatgcagagagaggcaagttaaaaagaagggctatcgcaacacctgggatgcagattagaggtttcgtatactttgttgaaactttagcaaggagcatttgcgaaaagcttgaacagtctgggctcccagtagggggcaatgaaaagaaggccaaattggcaaatgttgtgagaaagatgatgactaattcacaagatacagagatttctttcacaatcactggggacaacactaagtggaatgaaaatcaaaatcctcgaatgttcctggcgatgattacatatatcaccagaaatcaacccgagtggtttagaaacatcctgagcatggcacccataatgttctcaaataaaatggcaagactaggaaaagggtacatgttcgagagtaaaaaaatgaagattcgaacacaaataccagcagaaatgctagcaagcattgatctgaagtatttcaatgaatcaacaaagaagaaaattgagaaaataaggcctcttttaatggatggcacagcatcactgagtcctgggatgatgatgggcatgttcaacatgctaagtacggtcttgggagtctcgatactgaatcttggacaaaagaaatacaccaagacaacatactggtgggatgggctccaatcatccgacgattttgctctcatagtgaatgcaccaaaccatgaaggaatacaagcaggagtggacagattctacaggacctgcaagttagtgggaatcaacatgagcaaaaagaagtcctatataaataagacagggacatttgaattcacaagctttttttatcgctatggatttgtggctaattttagcatggagctacccagctttggagtgtctggagtaaatgaatcagctgacatgagtattggagtaacagtgataaagaacaacatgataaacaatgaccttggacctgcaacggctcagatggctcttcaattgttcatcaaagactacagatacacatataggtgccataggggagacacacaaattcagacgagaagatcatttgagttgaagaagctatgggatcaaacccaatcaaaggtagggctattagtatcagatggaggaccaaacttatacaacatacggaatcttcacattcctgaagtctgcttaaaatgggagctaatggatgatgattatcggggaagactttgtaatcccctgaatccctttgtgagtcataaggagattgattctgtaaacaatgctgtggtaatgccagcccatggcccagccaaaagcatggaatatgatgccgttgctactacacattcctggattcccaagaggaatcgttctattctcaacacaagccaaaggggaattcttgaggatgaacagatgtaccagaagtgttgcaatttattcgagaaatttttccctagcagttcatataggagaccggttggaatttctagcatggtggaggccatggtgtctagggcccggattgatgccagagtcgactttgagtctggacggatcaagaaagaagagttctctgagatcatgaagatctgttccaccattgaagaactcagacggcaaaaataa

>H1N1_PB1_KX918618_A/Virginia/30/2016

atggatgtcaatccgactctacttttcctaaaaattccagcacaaaatgccataagcaccacattcccttatactggagatcctccatacagccatggaacaggaacaggatacaccatggacacagtaaacagaacacaccaatactcagaaaaggggaagtggacaacaaacacagagactggtgcaccccagctcaacccgattgacggaccactacctgaagataatgaaccaagtgggtatgcacaaacagactgtgttctagaggctatggcttttcttgaagaatcccatccaggaatatttgagaattcatgccttgaaacaatggaagttgttcaacaaacaagggtagataaactgactcaaggtcgccagacttatgattggacattaaacagaaatcaaccggcagcaactgcattggccaacaccatagaagtttttagatcgaatgacctaacagctaacgagtcaggaaggctaatagatttcttaaaggatgtgatggaatcaatgaacaaagaggaaatagagataacaacccactttcaaagaaaaaggagagtaagagacaacatgaccaagaagatggtcacgcaaagaacaatagggaagaaaaaacagagactgaataagagaggctatctaataagagcactgacattaaatacgatgaccaaagatgcagagagaggcaagttaaaaagaagrgctatcgcaacacctgggatgcagattagaggtttcgtatactttgttgaaactttagctaggagcatttgcgaaaagcttgaacagtctgggctcccagtagggggcaatgaaaagaaggccaaattggcaaatgttgtgagaaagatgatgactaattcacaagatacagagatttctttcacaatcactggggacaacactaagtggaatgaaaatcaaaatcctcgaatgttcctggcgatgattacatatatcaccagaaatcaacccgagtggtttagaaacatcctgagcatggcacccataatgttctcaaataaaatggcaagactaggaaaagggtacatgttcgagagtaaaagaatgaagattcgaacacaaataccagcagaaatgctagcaagcattgatctgaagtatttcaatgaatcaacaaagaagaaaattgagaaaataaggcctcttttaatggatggcacagcatcactgagtcctgggatgatgatgggcatgttcaacatgctaagtacggtcttgggagtctcgatactgaatcttggacaaaagaaatacaccaagacaacatactggtgggatgggctccaatcatccgacgattttgctctcatagtgaatgcaccaaaccatgaaggaatacaagcaggagtggacagattctacaggacctgcaagttagtgggaatcaacatgagcaaaaagaagtcctatataaataagacagggacatttgaattcacaagctttttttatcgctatggatttgtggctaattttagcatggagctacccagctttggagtgtctggagtaaatgaatcagctgacatgagtattggagtaacagtgataaagaacaacatgataaacaatgaccttggacctgcaacggctcagatggctcttcaattgttcatcaaagactacagatacacatataggtgccataggggagacacacaaattcagacgagaagatcatttgagttgaagaagctatgggatcaaacccaatcaaaggtagggctattagtatcagatggaggaccaaacttatacaacatacggaatcttcacattcctgaagtctgcttaaaatgggagctaatggatgatgattatcggggaagactttgtaatcccctgaatccctttgtgagtcataaggagattgattctgtaaacaatgctgtggtaatgccagcccatggcccagccaaaagcatggaatatgatgccgttgctactacacattcctggattcccaagaggaatcgttctattctcaacacaagccaaaggggaattcttgaggatgaacagatgtaccagaagtgttgcaatttattcgagaaatttttccctagcagttcatataggagaccggttggaatttctagcatggtggaggccatggtgtctagggcccggattgatgccagagtcgactttgagtctggacggatcaagaaagaagagttctctgagatcatgaagatctgttccaccattgaagaactcagacggcaaaaataa

>H1N1_PB1_KY487502_A/Baltimore/0104/2016

atggatgtcaatccgactctacttttcctaaaaattccagcacaaaatgccataagcaccacattcccttatactggagatcctccatacagccatggaacaggaacaggatacaccatggacacagtaaacagaacacaccaatactcagaaaaggggaagtggacaacaaacacagagaccggtgcaccccagctcaacccgattgacggaccactacctgaagataatgaaccaagtgggtatgcacaaacagactgtgttctagaggctatggcttttcttgaagaatcccatccaggaatatttgagaattcatgccttgaaacaatggaagttgttcaacaaacaagggtagataaactgactcaaggtcgccagacttatgattggacattaaacagaaatcaaccggcagcaactgcattggccaacaccatagaagtttttagatcgaatgacctaacagctaacgagtcaggaaggctaatagatttcttaaaggatgtgatggaatcaatgaacaaagaggaaatagagataacaacccactttcaaagaaaaaggagagtaagagacaacatgaccaagaagatggtcacgcaaagaacaatagggaagaaaaaacagagactgaataagagaggctatctaataagagcactgacattaaatacgatgaccaaagatgcagagagaggcaagttaaaaagaagggctatcgcaacacctgggatgcagattagaggtttcgtatactttgttgaaactttagctaggagcatttgcgaaaagcttgaacagtctgggctcccagtagggggcaatgaaaagaaggccaaattggcaaatgttgtgagaaagatgatgactaattcacaagatacagagatttctttcacaatcactggggacaacactaagtggaatgaaaatcaaaatcctcgaatgttcctggcgatgattacatatatcaccagaaatcaacccgagtggtttagaaacatcctgagcatggcacccataatgttctcaaataaaatggcaagactaggaaaagggtacatgttcgagagtaaaagaatgaagattcgaacacaaataccagcagaaatgctagcaagcattgatctgaagtatttcaatgaatcaacaaagaagaaaattgagaaaataaggcctcttttaatggatggcacagcatcactgagtcctgggatgatgatgggcatgttcaacatgctaagtacggtcttgggagtctcgatactgaatcttggacaaaagaaatacaccaagacaacatactggtgggatgggctccaatcatccgacgattttgctctcatagtgaatgcaccaaaccatgaaggaatacaagcaggagtggacagattctacaggacctgcaagttagtgggaatcaacatgagcaaaaagaagtcctatataaataagacagggacatttgaattcacaagctttttttatcgctatggatttgtggctaattttagcatggagctacccagctttggagtgtctggagtaaatgaatcagctgacatgagtattggagtaacagtgataaagaacaacatgataaacaatgaccttggacctgcaacggctcagatggctcttcaattgttcatcaaagactacagatacacatataggtgccataggggagacacacaaattcagacgagaagatcatttgagttgaagaagctatgggatcaaacccaatcaaaggtagggctattagtatcagatggaggaccaaacttatacaacatacggaatcttcacattcctgaagtctgcttaaaatgggagctaatggatgatgattatcggggaagactttgtaatcccctgaatccctttgtgagtcataaggagattgattctgtaaacaatgctgtggtaatgccagcccatggcccagccaaaagcatggaatatgatgccgttgctactacacattcctggattcccaagaggaatcgttctattctcaacacaagccaaaggggaattcttgaggatgaacagatgtaccagaagtgttgcaatttattcgagaaatttttccctagcagttcatataggagaccggttggaatttctagcatggtggaggccatggtgtctagggcccggattgatgccagagtcgactttgagtctggacggatcaagaaagaagagttctctgagatcatgaagatctgttccaccattgaagaactcagacggcaaaaataa

>H1N1_PB1_CY220781_A/Texas/34/2017

atggatgtcaatccgactctacttttcctaaaaattccagcacaaaatgccataagcaccacattcccttatactggagatcctccatacagccatggaacaggaacgggatacaccatggacacagtaaacagaacacaccaatactcagaaaaggggaagtggacaacaaacacagagactggtgcaccccagctcaacccgattgacggaccactacctgaagataatgaaccaagtgggtatgcacaaacagactgtgttctagaggctatggcttttcttgaagaatcccatccaggaatatttgagaattcatgccttgaaacaatggaagttgttcaacaaacaagggtagataaactgactcaaggtcgccagacttatgattggacattaaacagaaatcaaccggcagcaactgcattggccaacaccatagaagtttttagatcgaatgacctaacagctaacgagtcaggaaggctgatagatttcttaaaggatgtgatggaatcaatgaacaaagaggaaatagagataacaacccactttcaaagaaaaaggagagtaagagacaacatgaccaagaagatggtcacgcaaagaacaatagggaagaaaaaacagagactgaataagagaggctatctaataagagcactgacgttaaatacgatgaccaaagatgcagagagaggcaagttaaaaagaagggctatcgcaacacctgggatgcagattagaggtttcgtatactttgttgaaactttagctaggagcatttgcgaaaagcttgaacagtctgggctcccagtagggggcaatgaaaagaaagccaaattggcaaatgttgtgagaaagatgatgactaattcacaagatacagagatttctttcacaatcactggggacaacactaagtggaatgaaaatcaaaatcctcgaatgttcctggcgatgattacatatatcaccagaaatcaacccgagtggtttagaaacatcctgagcatggcacccataatgttctcaaataaaatggcaagactaggaaaagggtacatgttcgagagtaaaagaatgaagattcgaacacaaataccagcagaaatgctggcaagcattgatctgaagtatttcaatgaatcaacaaagaagaaaattgagaaaataaggcctcttttaatggatggcacagcatcactgagtcctgggatgatgatgggcatgttcaacatgctaagtacggtcttgggagtctcgatactaaatcttggacaaaagaaatacaccaagacaacatactggtgggatgggctccaatcatccgacgattttgctctcatagtgaatgcaccaaaccatgaaggaatacaagcaggagtggacagattctacaggacctgcaagttagtgggaatcaacatgagcaaaaagaagtcctatataaataagacagggacatttgaattcacaagctttttttatcgctatggatttgtggctaattttagcatggagctacccagctttggagtgtctggagtaaatgaatcagctgacatgagtattggagtaacagtgataaagaacaacatgataaacaatgaccttggacctgcaacggctcagatggctcttcaattgttcatcaaagactacagatacacatataggtgccataggggagacacacaaattcagacgagaagatcatttgagttgaagaagctatgggatcaaacccaatcaaaggtagggctattagtatcagatggaggaccaaacttatacaacatacggaatcttcacattcctgaagtctgcttaaaatgggagctaatggatgatgattatcggggaagactttgtaatcccctgaatccctttgtgagtcataaggagattgattctgtaaacaatgctgtggtaatgccagcccatggcccagccaaaagcatggaatatgatgccgttgctactacacattcctggattcccaagaggaatcgttctattctcaacacaagccaaaggggaattcttgaggatgaacagatgtaccagaagtgttgcaatttattcgagaaatttttccctagcagttcatataggagaccggttgggatttctagcatggtggaggccatggtgtctagggcccgaattgatgccagagtcgactttgagtctggacggatcaagaaagaagagttctctgagatcatgaagatctgttccaccattgaagaactcagacggcaaaaataa

>H1N1_PB1_CY220829_A/California/17/2017

atggatgtcaatccgactctacttttcttaaaaattccagcacaaaatgccataagcaccacattcccttatactggagatcctccatatagccatggaacaggaacaggatacaccatggacacagtaaacagaacacaccaatattcagaaaaggggaagtggacaacaaacacagagactggtgcaccccagctcaacccgattgatggaccactacctgaggataatgaaccaagtgggtatgcacaaacagactgtgttctagaggctatggctttccttgaagaatcccacccaggaatatttgagaattcatgccttgaaacaatggaagttgttcaacaaacaagggtagataaactgactcaaggtcgccagacttatgattggacattaaacagaaatcaaccggcagcaactgcattggccaacaccatagaagtctttagatcgaatgrcctaacagctaacgagtcaggaaggctaatagatttcttaaaggatgtaatggaatcaatgaacaaagaggaaatagagataacaacccactttcaaagaaaaaggagagtaagagacaacatgaccaagaagatggtcacgcaaagaacaatagggaagaaaaaacagagactgaataagagaggctatctaataagagcactgacattaaatacgatgaccaaagatgcagagagaggcaagttaaaaagaagggctatcgcaacacctgggatgcagattagaggtttcgtatactttgttgaaactttagctaggagcatttgcgaaaagcttgaacagtctgggctcccagtagggggcaatgaaaagaaggccaaattggcaaatgttgtgagaaagatgatgactaattcacaagatacagagatttctttcacaatcactggggacaacactaagtggaatgaaaatcaaaatcctcgaatgttcctggcgatgattacatatatcaccagaaatcaacccgagtggtttagaaacatcctgagcatggcacccataatgttctcaaataaaatggcaagactggggaaagggtacatgttcgagagtaaaagaatgaagattcgaacacaaataccagcagaaatgctagcaagcattgatctgaagtacttcaatgaatcaacaaagaagaaaattgagaaaataaggcctcttttaatggatggcacagcatcactgagtcctgggatgatgatgggcatgttcaacatgctaagtacggtattgggagtctcgatactgaatcttggacaaaagaaatacaccaagacaacatactggtgggatgggctccaatcatccgacgattttgctctcatagtgaatgcaccaaaccatgaaggaatacaagcaggagtggacaggttctacaggacctgcaagttagtgggaatcaacatgagcaaaaagaagtcctatataaataagacagggacatttgaattcacaagctttttttatcgctatggatttgtggctaattttagcatggagctacccagctttggagtgtctggagtaaatgaatcagctgacatgagtattggagtaacagtgataaagaacaacatgataaacaatgaccttggacctgcaacggcccagatggctcttcaattgttcatcaaagactacagatacacatataggtgccataggggagacacacaaattcagacgaggagatcttttgagttaaagaagctatgggatcaaacccaatcaaaggtagggctattagtatcagatggaggaccaaacttatacaacatacggaatcttcacattcctgaagtctgcttaaaatgggagctaatggatgatgattatcggggaagactttgtaatcccctgaatccctttgtgagtcataaggagtttgattctgtaaacaatgccgtggtaatgccagcccatggtccagccaaaagcatggaatatgatgccgttgcaactacacattcctggatccccaagaggaatcgttctattctcaacacaagccaaaggggaattcttgaggatgaacagatgtaccagaagtgttgcaatctattcgagaaatttttccctagcagttcatataggagaccggttggaatttctagcatggtggaggccatggtgtctagggcccggattgatgccagagtcgactttgagtctggacggatcaagaaagaagagttctctgagatcatgaagatctgttccaccattgaagaactcagacggcaaaaataa

>H1N1_PB1_KY615599_A/Baltimore/0158/2016

atggatgtcaatccgactctacttttcctaaaaattccagcacaaaatgccataagcaccacattcccttatactggagatcctccatacagccatggaacaggaacaggatacaccatggacacagtaaacagaacacaccaatactcagaaaaggggaagtggacaacaaacacagagactggtgcaccccagctcaacccgattgacggaccactacctgaagataatgaaccaagtgggtatgcacaaacagactgtgttctagaagctatggcttttcttgaagaatcccatccaggaatatttgagaattcatgccttgaaacaatggaagttgttcaacaaacaagggtagataaactgactcaaggtcgccagacttatgattggacattaaacagaaatcaaccggcagcaactgcattggccaacaccatagaagtttttagatcgaatgacctaacagctaacgagtcaggaagactaatagatttcttaaaggatgtgatggaatcaatgaacaaagaggaaatagagataacaacccactttcaaagaaaaaggagagtaagagacaacatgaccaagaagatggtcacgcaaagaacaatagggaagaaaaaacagaggctgaataagagaggctatctaataagagcactgacattaaatacgatgaccaaagatgcagagagaggcaagttaaaaagaagggctatcgcaacacctgggatgcagattagaggtttcgtatactttgttgaaactttagctaggagcatttgcgaaaagcttgaacagtctgggctcccagtagggggcaatgaaaagaaggccaaattggcaaatgttgtgagaaagatgatgactaattcacaagatacagagatttctttcacaatcactggggacaacactaagtggaatgaaaatcaaaatcctcgaatgttcctggcgatgattacatatatcaccagaaatcaacccgagtggtttagaaacatcctgagcatggcacccataatgttctcaaataaaatggcaagactaggaaaagggtacatgttcgagagtaaaagaatgaagattcgaacacaaataccagcagaaatgctagcaagcattgatctgaagtatttcaatgaatcaacaaagaagaaaattgagaaaataaggcctcttttaatggatggcacagcatcactgagtcctgggatgatgatgggcatgttcaacatgctaagtacggtcttgggagtctcgatactgaatcttggacaaaagaagtacaccaagacaacatactggtgggatgggctccaatcatccgacgattttgctctcatagtgaatgcaccaaaccatgaaggaatacaagcaggagtggacagattctacaggacctgcaagttagtgggaatcaacatgagcaaaaagaagtcctatataaataagacagggacatttgaattcacaagctttttttatcgctatggatttgtggctaattttagcatggagctacccagctttggagtgtctggagtaaatgaatcagctgacatgagtattggagtaacagtgataaagaacaacatgataaacaatgaccttggacctgcaacggctcagatggctcttcaattgttcatcaaagactacagatacacatataggtgccataggggagacacacaaattcagacgagaagatcatttgagttgaagaagctatgggatcaaacccaatcaaaggtagggctattagtatcagatggaggaccaaacttatacaacatacggaatcttcacattcctgaagtctgcttaaaatgggagctaatggatggtgattatcggggaagactttgtaatcccctgaatccctttgtgagtcataaggagattgattctgtaaacaatgctgtggtaatgccagcccatggcccagccaaargcatggaatatgatgccgttgctactacacattcctggattcccaagaggaatcgttctattctcaacacaagccaaaggggaattcttgaggatgaacagatgtaccagaagtgttgcaatttattcgagaaatttttccctagcagttcatataggagaccggttggaatttctagcatggtggaggccatggtgtctagggcccggattgatgccagagtcgactttgagtctggacggataaagaaagaagagttctctgagatcatgaagatctgttccaccattgaagaactcagacggcaaaaataa

>H1N1_PB1_KY925416_A/Canoas/LACENRS-1023/2016

atggatgtcaatccgactctacttttcctaaaaattccagcacaaaatgccataagcaccacattcccttatactggagatcctccatacagccatggaacaggaacaggatacaccatggacacagtaaacagaacacaccaatactcagaaaaggggaagtggacaacaaacacagagactggtgcaccccagctcaacccgattgacggaccactacctgaagataatgaaccaagtgggtatgcacaaacagactgtgttctagaggctatggcttttcttgaagaatcccatccaggaatatttgagaattcatgccttgaaacaatggaagttgttcaacaaacaagggtagataagctaactcaaggtcgccagacttatgattggacattaaacagaaatcaaccggcagcaactgcattggccaacaccatagaagtttttagatcgaatgacctaacagctaacgagtcaggaaggctaatagatttcttaaaggatgtgatggaatcaatgaacaaagaggaaatagagataacaacccactttcaaagaaaaaggagagtaagagacaacatgaccaagaagatggtcacgcaaagaacaatagggaagaaaaaacagagactgaataagagaggctatctaataagagcactgacattaaatacgatgaccaaagatgcagagagaggcaagttaaaaagaagggctatcgcaacacctgggatgcagattagaggtttcgtatactttgttgaaactttagctaggagcatttgcgaaaagcttgaacagtctgggctcccagtagggggcaatgaaaagaaggccaaattggcaaatgttgtgagaaagatgatgactaattcacaagatacagagatttctttcacaatcactggggacaacactaagtggaatgaaaatcaaaatcctcgaatgttcctggcgatgattacatatatcaccagaaatcaacccgagtggtttagaaacatcctgagcatggcacccataatgttctcaaataaaatggcaagactaggaaaagggtacatgttcgagagtaaaagaatgaagattcgaacacaaataccagcagaaatgctagcaagcattgatctgaagtatttcaatgaatcaacaaagaagaaaattgagaaaataaggcctcttttaatggatggcacagcatcactgagtcctgggatgatgatgggcatgttcaacatgctaagtacggtcttgggagtctcgatactgaatcttggacaaaagaaatacaccaagacaacatactggtgggatgggctccaatcatccgacgattttgctctcatagtgaatgcaccaaaccatgaaggaatacaagcaggagttaacagattctacaggacctgcaagttagtgggaatcaacatgagcaaaaagaagtcctatataaataagacagggacatttgaattcacaagctttttttatcgctatggatttgtggctaattttagcatggagctacccagctttggagtgtctggagtaaatgaatcagctgacatgagcattggagtaacagtgataaagaacaacatgataaacaatgaccttggacctgcaacggctcagatggctcttcaattgttcatcaaagactacagatacacatataggtgccataggggagacacacaaatccagacgagaagatcatttgagttgaagaagctatgggatcaaacccaatcaaaggtagggctattagtatcagatggaggaccaaacttatacaacatacggaatcttcacattcctgaagtctgcttaaaatgggagctaatggatgatgattatcggggaagactttgtaatcccctgaatccctttgtgagtcataaggagattgattctgtaaacaatgctgtggtaatgccagcccatggcccagccaaaagcatggaatatgatgccgttgctactacacattcctggactcccaagaggaatcgttctattctcaacacaagccaaaggggaattcttgaggatgaacagatgtaccagaagtgttgcaatttattcgagaaatttttccctagcagttcatataggagaccggttggaatttctagcatggtggaggccatggtgtctagggcccggattgatgccagagtcgactttgagtctggacggatcaagaaagaagagttctctgagatcatgaagatctgttccaccattgaagaactcagacggcaaaaataa

>H1N1_PB1_KY925727_A/Porto Alegre/LACENRS-1598/2009

atggatgtcaatccgactctacttttcctaaaaattccagtgcaaaatgccataagcaccacattcccttatactggagatcctccatacagccatggaacaggaacaggatacaccatggacacagtaaacagaacacaccaatactcagaaaagggaaagtggacgacaaacacagagactggtgcaccccagctcaacccgattgatggaccactacctgaggataatgaaccaagtgggtatgcacaaacagactgtgttctagaggctatggctttccttgaagaatcccacccaggaatatttgagaattcatgccttgaaacaatggaagttgttcaacaaacaagggtagataaactaactcaaggtcgccagacttatgattggacattaaacagaaatcaaccggcagcaactgcattggccaacaccatagaagtctttagatcgaatggcctaacagctaatgagtcaggaaggctaatagatttcttaaaggatgtaatggaatcaatgaacaaagaggaaatagagataacaacccactttcaaagaaaaaggagagtaagagacaacatgaccaagaagatggtcacgcaaagaacaatagggaagaaaaaacaaagactgaataagagaggctatctaataagagcactgacattaaatacgatgaccaaagatgcagagagaggcaagttaaaaagaagggctatcgcaacacctgggatgcagattagaggtttcgtatactttgttgaaactttagctaggagcatttgcgaaaagcttgaacagtctgggctcccagtagggggcaatgaaaagaaggccaaactggcaaatgttgtgagaaagatgatgactaattcacaagacacagagatttctttcacaatcactggggacaacactaagtggaatgaaaatcaaaatcctcgaatgttcctggcgatgattacatatatcaccagaaatcaacccgagtggttcagaaacatcctgagcatggcacccataatgttctcaaacaaaatggcaagactagggaaagggtacatgttcgagagtaaaagaatgaagattcgaacacaaataccagcagaaatgctagcaagcattgacctgaagtacttcaatgaatcaacaaagaagaaaattgagaaaataaggcctcttctaatagatggcacagcatcactgagtcctgggatgatgatgggcatgttcaacatgctaagtacggtcttgggagtctcgatactgaatcttggacaaaagaaatacaccaagacaatatactggtgggatgggctccaatcatccgacgattttgctctcatagtgaatgcaccaaaccatgagggaatacaagcaggagtggacagattctacaggacctgcaagttagtgggaatcaacatgagcaaaaagaagtcctatataaataagacagggacatttgaattcacaagctttttttatcgctatggatttgtggctaattttagcatggagctacccagctttggagtgtctggagtaaatgaatcagctgacatgagtattggagtaacagtgataaagaacaacatgataaacaatgaccttggacctgcaacggcccagatggctcttcaattgttcatcaaagactacagatacacatataggtgccataggggagacacacaaattcagacgagaagatcatttgagttaaagaagctgtgggatcaaacccaatcaaaggtagggctattagtatcagatggaggaccaaacttatacaatatacggaatcttcacattcctgaagtctgcttaaaatgggagctaatggatgatgactatcggggaagactttgtaatcccctgaatccctttgtcagtcataaagagattgattctgtaaacaatgctgtggtaatgccagcccatggtccagccaaaagcatggaatatgatgccgttgcaactacacattcctggattcccaagaggaatcgttctattctcaacacaagccaaaggggaattcttgaggatgaacagatgtaccagaagtgctgcaatctattcgagaaatttttccctagcagttcatataggagaccggttggaatttctagcatggtggaggccatggtgtctagggcccggattgatgccagggtcgacttcgagtctggacggatcaagaaagaagagttctctgagatcatgaagatctgttccaccattgaagaactcagacggcaaaaataa

>H1N1_PB1_CY006201_A/New York/497/2003

atggatgtcaatccgacattacttttcttaaaagtgccagcacaaaatgctataagcacaactttcccttatactggtgaccctccttacagccatgggacaggaacagggtacaccatggatacagtcaacaggacacatcagtactcagaaagaggaagatggacaaaaaataccgaaactggagcgccgcaactcaacccaattgatgggccactaccagaagacaatgaaccaagtggctatgcccaaacagattgtgtattagaagcaatggctttccttgaagaatcccatcctggtatttttgaaaattcttgtattgaaacaatggaggttgttcagcaaacgagggtggacaaactgacacaaggcagacagacctatgactggactctaaataggaaccagcctgctgccacagcattggccaacactatagaagtattcagatcaaacggcctcatagcaaatgaatctgggaggctaatagacttccttaaagatgtaatggagtcgatggacagagacgaagtagaggtcacaactcattttcagagaaagaggagagtgagagacaatgtaactaaaaaaatggtgacccaaagaacaataggcaaaaagaaacataaattagacaaaagaagttacctaattagggcattaaccctgaacacaatgaccaaagatgctgagagggggaaactaaaacgcagagcaattgcaaccccaggaatgcaaataagggggtttgtatactttgttgagacactggcaagaagcatatgtgaaaagcttgaacaatcagggttgccagttggaggaaatgagaagaaagcaaagttagcaaatgttgtaaggaagatgatgaccaactcccaggacactgaaatttctttcaccatcactggagataacacaaaatggaacgaaaatcaaaaccctagaatgttcttggccatgatcacatatataaccaaaaatcagcctgaatggttcagaaatattctaagtattgctccaataatgttttcaaacaagatggcgagactaggtaaggggtacatgtttgaaagcaagagtatgaaactgagaactcaaatacctgcagagatgctagccgacatagatttgaaatatttcaatgattcaactaaaaagaaaattgaaaaaatccgaccattactaatagatggaactgcatcattgagtcctggaatgatgatgggcatgttcaatatgttaagcaccgtcttgggcgtctccattctgaatcttgggcaaaagagatacaccaagactacttactggtgggatggtcttcaatcgtctgatgattttgctctgattgtgaatgcacccaactatgcaggaattcaagctggagttgacaggttttatcgaacctgtaagctgctcggaattaatatgagcaaaaagaagtcttacataaacagaacaggtacctttgaattcacgagctttttctatcgttatgggtttgttgcaaatttcagcatggagcttcctagttttggggtgtctggggtcaatgaatctgcagacatgagtattggagtcactgtcatcaaaaacaatatgataaacaatgaccttggcccagcaactgctcaaatggcccttcagttatttataaaagattacaggtacacttatcgctgccacagaggtgacacacaaatacaaacccggagatcatttgagataaagaaactatgggaccaaacccgctccaaagctgggctgttggtctctgatggaggccccaatttatataacattagaaatctccatattcctgaagtctgcttgaaatgggagttgatggatgaggattaccaggggcgtttatgcaacccattgaacccgtttgtcagtcataaagagattgaatcagtgaacaatgcagtgatgatgccggcacatggtccagccaaaaatatggagtatgacgctgttgcaacaacacactcctgggttcccaaaaggaatcgatccattttgaacacgagccaaagggggatacttgaagatgagcaaatgtatcagaggtgctgcaatttatttgaaaaattcttcccaagtagctcatacagaagaccagttggaatatccagtatggtagaggctatggtttcaagagcccgaattgatgcacggattgatttcgaatctggaaggataaagaaagaggaattcgctgagatcatgaagacctgttccaccattgaagacctcagacggcaaaaatag

>H1N1_PB1_CY006921_A/New York/488/2003

atggatgtcaatccgacattacttttcttaaaagtgccagcacaaaatgctataagcacaacttttccttatactggtgaccctccttacagccatgggacaggaacagggtacaccatggatacagtcaacaggacacatcagtactcagaaagaggaagatggacaaaaaataccgaaactggagcaccgcaactcaacccaattgatgggccactaccagaagacaatgaaccaagtggctatgcccaaacagattgtgtattagaagcaatggctttccttgaagaatcccatcctggtatttttgaaaactcttgtattgaaacaatggaggttgttcagcaaacaagggtggacaaactgacacaaggcagacagacctatgactggactctaaataggaaccagcctgctgctacagcattggcaaacactatagaagtattcaggtcaaacggcctcatagcaaatgaatctgggaggctaatagacttccttaaagatgtaatggagtcaatggacagagacgaagtagaggtcacaactcattttcaaagaaagaggagagtgagagacaatgtaactaaaaaaatggtgacccaaagaacaataggcaaaaagaaacataaattagacaaaaaaagttacctaattagggcattaaccctgaacacgatgaccaaagatgctgagaggggaaaactaaaacgcagagcaattgcaaccccaggaatgcaaataagggggtttgtatactttgttgagacactggcaagaagcatatgtgaaaagcttgaacaatcagggttgccagttggaggaaatgagaagaaagcaaagttagcaaatgttgtaaggaagatgatgaccaactcccaggacactgaaatttctttcaccatcactggagataacacaaaatggaacgaaaatcaaaaccctagaatgttcttggccatgatcacatatataaccaaaaatcagcctgaatggttcagaaatattctgagtattgctccaataatgttttcaaacaagatggcgagactaggtaaggggtacatgtttgaaagcaagagtatgaaactgagaactcaaatacctgcagagatgctagccaacatagatttgaaatatttcaatgattcaactaaaaagaaaattgaaaaaatccgaccattattaatagatggaactgcatcattgagtcctggaatgatgatgggcatgttcaatatgttaagcaccgtcttgggcgtctccattctgaatcttgggcaaaagagatacaccaagactacttactggtgggatggtcttcaatcgtctgatgattttgctttgattgtgaatgcacccaactatgcaggaattcaagctggagttgacaggttctatcgaacctgtaagctgctcggaattaatatgagcaaaaagaagtcttacataaacagaacaggtacctttgaattcacgagctttttctatcgttatgggtttgttgccaatttcagcatggagcttcctagttttggggtgtctggggtcaatgaatctgcagacatgagcattggagtcactgtcatcaaaaacaatatgataaacaatgaccttggcccagcaactgctcaaatggcccttcagttatttataaaagattacaggtacacttatcgatgccacagaggtgacacacaaatacaaacccggagatcatttgagataaagaaactatgggaccaaacccgctccaaagctgggctgttggtctctgatggaggccccaatttatataacattagaaatctccatattcctgaagtctgcttgaaatgggagttgatggatgaggattaccaggggcgtttatgcaacccattgaacccgtttgtcagtcataaagagattgaatcagtgaacaatgcagtgataatgccggcacatggtccagccaaaaatatggagtatgacgctgttgcaacaacacactcctgggtccccaaaaggaatcgatccattttgaacacgagccaaagggggatacttgaagatgagcaaatgtatcagaggtgctgcaatttatttgaaaaattcttcccaagtagctcatacagaagaccagttggaatatccagtatggtagaggctatggtttcaagagcccgaattgatgcacggattgatttcgaatctggaaggataaagaaagaggaattcgctgagatcatgaagacctgttccaccattgaagacctcagacggcaaaaatag

>H1N1_PB1_DQ415296_A/Taiwan/4845/1999

atggatgtcaatccgacattacttttcttaaaagtgccagcacaaaatgctataagcacaacgtttccttatactggtgaccctccttacagccatgggacaggaacagggtacaccatggatacagtcaacaggacacatcagtactcagaaagaggaagatggacaaaaaataccgaaactggagcaccgcaactcaacccaattgatgggccactaccaaaagacaatgaaccaagtggctatgcccaaacagattgtgtattagaagcaatggctttccttgaggaatcccatcctggtatttttgaaaactcttgtattgaaacaatggaggttgttcagcaaacaagggtggacaaactgacacaaggcagacagacctatgactggactctaaataggaaccagcctgctgccacagcattggccaacactatagaagtgttcagatcaaacggcctcatagcaaatgaatctgggaggctaatagacctccttaaagatgtaatggagtcgatggacagagacgaagtagagatcacaactcattttcagagaaagaggagagtgagagacaatgtaactaaaaaaatggtgacccaaagaacaataggcaaaaagaaacataaattagacaaaaaaagttacctaattagggcattaaccctgaacacaatgaccaaagatgctgagagggggaaactaaaacgcagagcaattgcaaccccaggaatgcaaataagggggtttgtgtactttgttgagacactggcaagaagcatatgtgaaaagcttgaacaatcagggttgccagttggaggaaatgaaaagaaagcaaagttagcaaatgttgtaaggaagatgatgaccaactcccaggacactgaaatttctttcaccatcactggagataacacaaagtggaacgaaaatcaaaaccctagaatgttcttggccatgatcacatatataaccaaaaatcagcctgaatggttcagaaatattctaagtattgctccaataatgttttcaaacaaaatggcgagactaggtaaggggtacatgtttgaaagcaaaagtatgaaactgagaactcaaatacctgcagagatgctagccaacatagatttgaaatatttcaatgattcaactaaaaagaaaattgaaaaaatccggccattattaatagatggaactgcatcattgagtcctggaatgatgatgggcatgttcaaaatgttaagcaccgtcttgggcgtctccattctgaatcttgggcaaaagagatacaccaagactacttactggtgggatggtcttcaatcgtctgatgattttgctctgattgtgaatgcacccaactatgcaggaattcaagctggagttgacaggttttatcggacctgtaagctgctcggaattaatatgagcaaaaagaagtcttacataaacagaacaggtacctttgaattcacgagctttttctatcgttatgggtttgttgccaatttcagcatggagcttcctagttttggggtgtctggggtcaatgaatctgcagacatgagtattggagtcactgtcatcaaaaacaatatgataaacaatgaccttggcccagcaactgctcaaatggcccttcagttatttataaaagattacaggtacacgtatcgatgccacagaggtgacacacaaatacaaacccggagatcatttgagataaagaaactatgggaccaaacccgctccaaagctgggctgttggtctctgatggaggccccaatttatataacattagaaatctccatattcctgaagtctgcttgaaatgggagttgatggatgaggattaccaggggcgtctatgcaacccattgaacccgtttgtcagtcataaagagattgaatcagtgaacaatgcagtgatgatgccggcacatggtccagccaaaaatatggagtacgacgctgttgcaacaacacactcctgggttcccaaaaggaatcgatccattttgaatacgagccaaaggggaatacttgaggatgagcaaatgtatcagaggtgctgcaatttatttgaaaaattcttcccaagtagctcatacagaagaccagttggaatatccagtatggtagaggctatggtttccagagctcgaattgatgcacggattgatttcgaatctggaaggataaagaaagaggaattcgctgagatcatgaagacctgttccaccattgaagacctcagacggcaaaaatag

>H1N1_PB1_CY009554_A/Canterbury/65/2000

atggatgtcaatccgacattacttttcttaaaagtgccagcacaaaatgctataagcacaacttttccttatactggtgaccctccttacagccatgggacaggaacagggtacaccatggatacagtcaacaggacacatcagtactcagaaagaggaagatggacaaaaaataccgaaactggagcaccgcaactcaacccaattgatgggccactaccaaaagacaatgaaccaagtggctatgcccaaacagattgtgtattagaagcaatggctttccttgaggaatcccatcctggtatttttgaaaactcttgtattgaaacaatggaggttgttcagcaaacaagggtggacaaactgacacaaggcagacagacctatgactggactctaaataggaaccagcctgctgccacagcattggccaacactatagaagtgttcagatcaaacggcctcatagcaaatgaatctgggaggctaatagacttccttaaagatgtaatggagtcgatggacagagacgaaatagaggtcacaactcattttcagagaaagaggagagtgagagacaatgtaaccaaaaaaatggtgacccaaagaacaataggcaaaaagaaacataaattagataaaagaagttacctaattagggcattaaccctgaacacaatgaccaaagatgctgagagggggaaactaaaacgcagagcaattgcaaccccaggaatgcaaataagggggtttgtatactttgttgagacactggcaagaagcatatgtgaaaagcttgaacaatcagggttgccagttggaggaaatgaaaagaaagcaaagttagcaaatgttgtaaggaagatgatgaccaactcccaggacactgaaatttctttcaccatcactggagataacacaaaatggaacgaaaatcaaaaccctagaatgttcttggccatgatcacatatataaccaaaaatcagcctgaatggttcagaaatattctaagtattgctccaataatgttttcaaacaaaatggcgagactaggtaaggggtacatgtttgaaagcaagagtatgaaactgagaactcaaatacctgcagagatgctagccaacatagatttgaaatatttcaatgattcaactaaaaagaaaattgaaaaaatccggccattattaatagatggaactgcatcattgagtcctggaatgatgatgggcatgttcaatatgttaagcaccgtcttgggcgtctccattctgaatcttgggcaaaagagatacaccaagactacttactggtgggatggtcttcaatcgtctgatgattttgctctgattgtgaatgcacccaactatgcaggaattcaagctggagttgacaggttttatcgaacctgtaagctgctcggaattaatatgagcaaaaagaagtcttacataaacagaacaggtacctttgaattcacgagctttttctatcgttatgggtttgttgccaatttcagcatggagcttcctagttttggggtgtctggggtcaatgaatctgcagacatgagtattggagtcactgtcatcaaaaacaatatgataaacaatgaccttggcccagcaactgctcaaatggcccttcagttatttataaaagattacaggtacacgtatcgatgccacagaggtgacacacaaatacaaacccggagatcatttgagataaagaaactatgggaccaaacccgctccaaagctgggctgttggtmtctgatggaggccccaatttatataacattagaaatctccatattcctgaagtctgcttgaaatgggagttgatggatgaggattaccaagggcgtttatgcaacccattgaacccgtttgtcagtcataaagagattgaatcagtgaacaatgcagtgatgatgccggcacatggtccagccaaaaatatggagtatgacgctgttgcaacaacacactcctgggttcccaaaaggaatcgatccattttgaatacgagccaaagggggatacttgaggatgagcaaatgtatcagaggtgctgcaatttatttgaaaaattcttcccaagtagctcatacagaagaccagttggaatatccagtatggtagaggctatggtttccagagcccgaattgatgcacggattgatttcgaatctggaaggataaagaaagaggaattcgctgagatcatgaagacctgttccaccattgaagacctcagacggcaaaaatag

>H1N1_PB1_CY010162_A/Canterbury/19/2001

atggatgtcaatccgaccttacttttcttaaaagtgccagcacaaaatgctataagcacaactttcccttatactggtgatcctccttacagccatgggacaggaacagggtataccatggatacagtcaacaggacacatcagtactcagaaagaggaagatggacaaaaaataccgaaactggagcaccgcaactcaacccaattgatggaccactaccaaaagacaatgaaccaagtggctatgcccaaacagattgtgtattagaagcaatggctttccttgaggaatcccatcctggtatttttgaaaactcttgtattgaaacaatggaggttgttcagcaaacaagggtggacaaactgacacaaggcagacagacctatgactggactctaaatagaaaccagcctgctgccacagcattggccaacactatagaagtgttcagatcaaacggcctcatagcaaatgaatctgggaggctaatagacttccttaaagatgtaatggagtcgatggacagagacgaagtagagatcacaacacattttcagagaaagaggagggtgagagacaatgtaactaaaaaaatggtgacccaaagaacaataggcaaaaagaaacataaattagacaaaagaagttacctaattagggcattaaccctgaacacaatgaccaaagatgctgagagggggaaactaaaacgtagagcaattgcaaccccaggaatgcaaataagggggtttgtatactttgttgagacactggcaagaagcatatgtgaaaagcttgaacaatcagggttgccagttgggggaaatgaaaagaaagcaaagttagcaaatgttgtaaggaagatgatgaccaactcccaggacactgaaatttctttcaccatcactggagataacacaaaatggaacgaaaatcaaaaccctagaatgtttttggccatgatcacatatataaccaaaaatcagcctgaatggttcagaaatattctaagtattgctccaataatgttttcaaacaaaatggcgagactaggtaaggggtacatgtttgaaagcaagagtatgaaactgagaactcaaatacctgcagagatgctagccaacatagatttgaaatatttcaatgattcaactaaaaagaaaattgaaaaaatccggccattattaatagatggaactgcatcattgagtcctggaatgatgatgggcatgttcaatatgttaagcactgtcttgggcgtctccattctgaatcttgggcaaaagagatacaccaagactacttactggtgggatggtcttcaatcgtctgatgattttgctctgattgtgaatgcatccaactatgcaggaattcaagctggagttgacaggttttatcgaacctgtaagctgctcggaattaatatgagcaaaaagaagtcttacataaacagaacaggtacctttgaattcacgagctttttctatcgttatgggtttgttgccaatttcagcatggagcttcctagttttggggtgtctggggtcaatgagtctgcagacatgagtattggagtcactgtcatcaaaaacaatatgataaacaatgaccttggcccagcaactgctcaaatggcccttcagttatttatcaaagattacaggtacacgtatcgatgccacagaggtgacacacaaatacaaacccggagatcatttgagataaagaaactatgggaccaaacccgctccaaagctgggctgttggtctctgatggaggccccaatttatataacattagaaatctccatattcctgaagtctgcttgaaatgggagttgatggatgaggattaccaggggcgtttatgcaacccattgaacccatttgtcagtcataaagagattgagtcagtgaacaatgcagtgatgatgccggcacatggtccagccaaaaatatggagtatgacgctgttgcaacaacacactcctgggttcccaaaaggaatcgatccattttgaatacgagccaaagggggatacttgaggatgagcaaatgtatcagaggtgctgcaatttatttgaaaaattcttcccaagtagttcatacagaagaccagttggaatatccagtatggtagaggctatggtttccagagcccgaattgatgcaagaattgatttcgaatctggaaggataaagaaagaggaattcgctgagatcatgaagacctgttccaccattgaagacctcagacggcaaaaatag

>H1N1_PB1_CY010514_A/New York/615/1995

atggatgtcaatccgactttacttttcttgaaagtgccagcacaaaatgctataagcacaactttcccctatactggagaccccccttacagccatggtacagggacagggtacaccatggatacagtcaacaggacacatcagtactcagaaaggggaatatggacaaaaaataccgaaactggagcaccgcaactcaacccgattgatgggccattaccaaaagacaatgaaccaagtggctatgcccaaacagattgtgtattagaagcaatggctttccttgaggaatcccaccctggtatttttgaaaactcgtgtattgaaacgatggaggctgttcagcaaacaagagtggacaaactgacacaaggcagacagacctatgactggactctaaatagaaaccagcctgctgccacagcattggccaacactatagaagtgttcagatcaaacggcctcatagcaaatgagtctgggaggctaatagacttccttaaagatgtaatggagtcaatggacagagaagaagtagagatcacaactcattttcagagaaagagaagagtgagagacaatgtaactaaaaaaatggtgacacaaagaacaataggcaaaaagaaacataaattggacaaaagaagttatctaattagggcattaaccctgaacacaatgaccaaagatgctgagagggggaaactaaaacgtagagcaattgcaaccccaggaatgcagataagggggtttgtatactttgttgagacactggcaaggagcatatgtgagaaacttgaacaatcagggttgccagttggaggaaatgagaagaaagcaaagttagcaaatgttgtaaggaagatgatgaccaactcccaggacactgaaatttctttcaccatcactggagataacacaaaatggaacgaaaatcagaaccctagaatgtttttggctatgatcacatatataactagaaatcagcctgagtggttcagaaacattctaagtattgctccaataatgttttcaaacaaaatggcgagactaggtaaggggtacatgtttgaaagcaagagtatgaagctgagaactcaaatacctgcagaaatgctagccaacatagatttgaaatatttcaatgattcaacaaaaaagaaaattgaaaaaatccggccactattaatagatggaactgcatcactgagccctggaatgatgatgggcatgttcaatatgttaagcactgtcttgggcgtctccatcctgaatcttgggcaaaagagatacaccaagactacttactggtgggatggtcttcaatcgtctgatgattttgctctgattgtgaatgcacccaactatgcaggaattcaagctggagttgacaggttttatcgcacctgtaagctgctcggaattaatatgagcaaaaagaagtcttacataaacaggacaggtacttttgaattcacaagctttttctatcgttatgggtttgttgccaatttcagcatggaacttcctagctttggggtgtctggggtcaatgagtctgcagacatgagtattggggtcactgtcatcaaaaataatatgataaacaatgatcttggtccagcaactgctcaaatggcccttcagttattcatcaaagattacaggtacacgtatcgatgccacagaggtgacacacaaatacaaacccggagatcatttgagataaagaaactgtgggaccaaacccgctccaaagctgggctgttgatctctgatggaggccccaatttatataatattagaaatctccacattcctgaagtctgcttgaaatgggagttgatggatgaggattaccaggggcgtttatgcaatccattgaacccgtttgtcagtcataaagaaattgaatcagtgaacaatgcagtgatgatgccggcacatggtccagccaaaaatatggagtatgacgctgttgcaacaacgcactcctgggtccccaaaaggaatcgatccattttgaatacgagccaaagaggaatacttgaggatgagcaaatgtatcaaaggtgctgcaatttatttgaaaaattcttcccaagcagctcatatagaagaccagttggaatatccagtatggtagaggctatggtttccagagcccgaatcgatgcacggattgatttcgaatctggaaggataaagaaagaggagttcgctgagatcatgaagacctgttccaccattgaagacctcagacggcaaaaatag

>H1N1_PB1_CY010930_A/Memphis/4/1983

atggatgtcaatccgaccttacttttcttaaaagtgccagcacaaaatgctataagcacaactttcccttatactggagaccctccttacagccatgggacaggaacagggtacaccatggatacagtcaacaggacacatcagtactcagaaaggggaagatggacaaaaaacaccgaaactggagcaccgcaactcaacccgattgatgggccactaccaaaagacaatgaaccaagtggttatgcccaaacagattgtgtattagaagcaatggctttccttgaggaatcccatcccggtatttttgaaaactcgtgtattgaaacgatggaggttgttcagcaaacacgtgtagacaaactgacacaaggcagacagacctatgactggactctaaatagaaaccagcctgctgctacagcattggccaacaccatagaagtgttcagatcaaacggcctcatggcaaatgaatctggaaggctaatagacttccttaaagatgtaatggattcaatggatagagaagaaatggagatcacaactcattttcagagaaagagaagagtgagggacaatgtaactaagaaaatggtgacacaaagaacaataggcaaaaagaaacatagattaaacaaaaggagttatctaattagggcattaaccctgaacacaatgaccaaagatgctgagagggggaaactaaaacgtagagcaattgcaaccccagggatgcaaataagggggtttgtatactttgttgagacactggcaagaagtatatgtgaaaaacttgaacaatcagggttgccagttggaggaaatgaaaagaaagcaaagttagcaaatgttgtaaggaaaatgatgaccaattctcaggacactgaaatttctttcaccatcactggagataacacaaaatggaacgaaaatcagaaccctagaatgtttttggccatgatcacatatataaccagaaatcagcccgaatggttcagaaatattctaagtattgctccaataatgttctcaaacaaaatggcgagactaggtaaggggtacatgtttgaaagcaagagtatgaaacttagaactcaaatacctgcagaaatgctagcaaacatcgatttgaggtatttcaatgattcaacaagaaagaaaattgaaaaaatcaggccactcttaatagatggaactgcatcactgagccctggaatgatgatgggcatgttcaatatgttaagcactgtattgggcgtctccatcctgaatcttggacaaaagagatacaccaagactacttactggtgggatggtcttcaatcgtctgatgattttgctctgattgtgaatgcacccaactatgcaggaattcaagctggagttgacaggttttatcgaacctgtaagctgctcggaatcaacatgagcaagaaaaagtcctacataaacagaacaggtacttttgaattcacgagctttttttatcgttatgggtttgttgccaatttcagcatggagcttcccagttttggggtgtctgggatcaacgagtctgcagacatgagtattggagtcactgtcatcaaaaacaatatgataaacaatgatcttggtccagcaaccgctcaaatggcccttcagttattcatcaaagattacaggtacacatatcgatgccacagaggtgacacacaaatacaaacccgaagatcatttgaaataaagaaactgtgggatcaaacccgttccaaaactgggctgctggtctctgatggaggtcctaatttgtacaacattagaaatctccacattcctgaagtctgcttgaaatgggatttgatggatgaggattaccaggggcgtttatgcaacccattgaacccatttgtcagtcataaagagattgaatcagtgaacaatgcagtgatgatgccggcacatggtccagccaaaattatggagtatgatgctgttgcaacaacacactcctgggtccccaaaaggaatcgatccatcttgaatacgagccaaaggggaatacttgaggatgaacaaatgtatcagaggtgctgcaatttatttgaaaaattcttccccagcagttcatacagaagaccagtcggaatatccagtatggtagaggctatgatttccagagcccgaattgatgcacggattgatttcgaatctggaaggataaagaaagaggagttcactgagatcatgaagacctgttccaccattgaagaactcagacggcaaaaatag

>H1N1_PB1_CY012606_A/Wellington/11/2000

atggatgtcaatccgacattacttttcttaaaagtgccagcacaaaatgctataagcacaacttttccttatactggtgaccctccttacagccatgggacaggaacagggtacaccatggatacagtcaacaggacacatcagtactcagaaagaggaagatggacaaaaaataccgaaactggagcaccgcaactcaacccaattgatgggccactaccaaaagacaatgaaccaagtggctatgcccaaacagattgtgtattagaagcaatggctttccttgaggaatcccatcctggtatttttgaaaactcttgtattgaaacaatggaggttgttcagcaaacaagggtggacaaactgacacaaggcagacagacctatgactggactctaaataggaaccagcctgctgccacagcattggccaacactatagaagtgttcagatcaaacggcctcatagcaaatgaatctgggaggctaatagacttccttaaagatgtaatggagtcgatggacagagacgaaatagaggtcacaactcattttcagagaaagaggagagtgagagacaatgtaactaaaaaaatggtgacccaaagaacaataggcaaaaagaaacataaattagataaaagaagttacctaattagggcattaaccctgaacacaatgaccaaagatgctgagagggggaaactaaaacgcagagcaattgcaaccccaggaatgcaaataagggggtttgtatactttgttgagacactggcaagaagcatatgtgaaaagcttgaacaatcagggttgccagttggaggaaatgaaaagaaagcaaagttagcaaatgttgtaaggaagatgatgaccaactcccaggacactgaaatttctttcaccatcactggagataacacaaaatggaacgaaaatcaaaaccctagaatgttcttggccatgatcacatatataaccaaaaatcagcctgaatggttcagaaatattctaagtattgctccaataatgttttcaaacaaaatggcgagactaggtaaggggtacatgtttgaaagcaagagtatgaaactgagaactcaaatacctgcagagatgctagccaacatagatttgaaatatttcaatgattcaactaaaaagaaaattgaaaaaatccggccattattaatagatggaactgcatcattgagtcctggaatgatgatgggcatgttcaatatgttaagcaccgtcttgggcgtctccattctgaatcttgggcaaaagagatacaccaagactacttactggtgggatggtcttcaatcgtctgatgattttgctctgattgtgaatgcacccaactatgcaggaattcaagctggagttgacaggttttatcgaacctgtaagctgctcggaattaatatgagcaaaaagaagtcttacataaacagaacaggtacctttgaattcacgagctttttctatcgttatgggtttgttgccaatttcagcatggagcttcctagttttggggtgtctggggtcaatgaatctgcagacatgagtattggagtcactgtcatcaaaaacaatatgataaacaatgaccttggcccagcaactgctcaaatggcccttcagttatttataaaagattacaggtacacgtatcgatgccacagaggtgacacacaaatacaaacccggagatcatttgagataaagaaactatgggaccaaacccgctccaaagctgggctgttggtctctgatggaggccccaatttatataacattagaaatctccatattcctgaagtctgcttgaaatgggagttgatggatgaggattaccaagggcgtttatgcaacccattgaacccgtttgtcagtcataaagagattgaatcagtgaacaatgcagtgatgatgccggcacatggtccagccaaaaatatggagtatgacgctgttgcaacaacacactcctgggttcccaaaaggaatcgatccattttgaatacgagccaaagggggatacttgaggatgagcaaatgtatcagaggtgctgcaatttatttgaaaaattcttcccaagtagctcatacagaagaccagttggaatatccagtatggtagaggctatggtttccagagcccgaattgatgcacggattgatttcgaatctggaaggataaagaaagaggaattcgctgagatcatgaagacctgttccaccattgaagacctcagacggcaaaaatag

>H1N1_PB1_CY026841_A/Virginia/UR06-0139/2007

atggatgtcaatccgactttacttttcttaaaagtgccagcacaaaatgctataagcacaacttttccttatactggtgaccctccttacagccatgggacaggaacagggtacaccatggatacagtcaacaggacacaccagtactcagaaagaggaagatggacaaaaaataccgaaactggagcaccgcaactcaacccaattgatgggccactaccagaagacaatgaaccaagtggctatgcccaaacagattgtgtattagaagcaatggctttccttgaagaatcccatcctggtatctttgaaaactcttgtattgaaacaatggaggttgttcagcaaacaagggtggacaaactgacacaaggcagacagacctatgactggactctaaataggaaccagcctgctgcaacagcattggcaaacactatagaagtattcagatcaaacggcctcatagcaaatgaatctggaaggctaatagacttccttaaagatgtaatggagtcgatggacagagacgaagtagaggtcacaactcattttcaaagaaagaggagagtaagagacaatgtaactaaaaaaatggtgactcaaagaacaataggcaaaaagaaacataaattagacaaaagaagttacctaattagggcattaaccctgaacacaatgaccaaagatgctgagagggggaaactaaaacgcagagcaattgcaaccccaggaatgcaaataagagggtttgtatactttgttgagacactggcaagaagcatatgtgaaaagcttgaacaatcaggattgccagttggaggaaatgagaagaaagcaaagttagcaaatgttgtaaggaagatgatgaccaactcccaggacactgaaatttctttcaccatcactggagataacacaaaatggaacgaaaatcaaaaccctagaatgttcttggccatgatcacatatataaccaaaaatcagcctgaatggttcagaaatattctaagtattgctccaataatgttttcaaacaagatggcgagactaggtaaggggtacatgtttgaaagcaagagtatgaaactgagaactcaaatacctgcagagatgctagtcaacatagatttgaaatatttcaatgattcaactaaaaagaaaattgaaaaaatccgaccattattaatagatggaactgcatcattgagtcctggaatgatgatgggcatgttcaatatgttgagcaccgtcttgggcgtctccattctgaatcttgggcaaaagagatacaccaagactacttactggtgggatggtcttcaatcgtctgatgattttgctttgattgtgaatgcacccaactatgcaggaattcaagctggagttgacaggttttatcgaacctgtaagctgctcggaattaatatgagcaaaaagaagtcttacataaacagaacaggtacctttgaattcacgagctttttctatcgttatgggtttgttgccaatttcagcatggagcttcctagttttggggtgtctggggtcaatgaatctgcagacatgagtattggagtcactgtcatcaaaaacaatatgataaacaatgaccttggcccagcaactgctcaaatggcccttcagttatttataaaagattacaggtacacttatcgatgccacagaggtgacacacaaatacaaacccggagatcatttgagataaagaaactatgggaccaaacccgctccaaagctgggctgttggtctctgatggaggccccaatttatataacattagaaatctccatattcctgaagtttgcttgaaatgggagttgatggatgaggattaccaggggcgtttatgcaacccattaaacccgtttgtcagccataaagagattgaatcagtgaacaatgcagtgataatgccggcacatggcccagccaaaaatatggagtatgacgctgttgcaacaacacactcctgggtccccaaaagaaatcgatccattttgaacacgagccaaagggggatacttgaagatgagcaaatgtatcagagatgctgcaatttatttgaaaaattcttcccaagtagctcatacagaagaccagttggaatatccagtatggtagaggctatggtttcaagagcccgaattgatgcacggattgatttcgaatctggaaggataaagagggaggaatttgctgagatcatgaagatctgttccaccattgaagacctcagacggcaaaaatag

>H1N1_PB1_CY027225_A/Kentucky/UR06-0029/2007

atggatgtcaatccgacattacttttcttaaaagtgccagcacaaaatgctataagcacaacttttccttatactggtgaccctccttacagccatgggacaggaacagggtacaccatggatacagtcaacaggacacaccagtactcagaaagaggaagatggacaaaaaataccgaaactggagcaccgcaactcaacccaattgatgggccactaccagaagacaatgaaccaagtggctatgcccaaacagattgtgtattagaagcaatggctttccttgaagaatcccatcctggtatctttgaaaactcttgtattgaaacaatggaggttgttcagcaaacaagggtggacaaactgacacaaggcagacagacctatgactggactctaaataggaaccagcctgctgcaacagcattggcaaacactatagaagtattcagatcaaacggcctcatagcaaatgaatctggaaggctaatagacttccttaaagatgtaatggagtcgatggacagagacgaagtagaggtcacaactcattttcaaagaaagaggagagtaagagacaatgtaactaaaaaaatggtgactcaaagaacaataggcaaaaagaaacataaattagacaaaagaagttacctaattagggcattaaccctgaacacaatgaccaaagatgctgagagggggaaactaaaacgcagagcaattgcaaccccaggaatgcaaataagagggtttgtatactttgttgagacactggcaagaagcatatgtgaaaagcttgaacaatcaggattgccagttggaggaaatgagaagaaagcaaagttagcaaatgttgtaaggaagatgatgaccaactcccaggacactgaaatttctttcaccatcactggagataacacaaaatggaacgaaaatcaaaaccctagaatgttcttggccatgatcacatatataaccaaaaatcagcctgaatggttcagaaatattctaagtattgctccaataatgttttcaaacaagatggcgagactaggtaaggggtacatgtttgaaagcaagagtatgaaactgagaactcaaatacctgcagagatgctagccaacatagatttgaaatatttcaatgattcaactaaaaagaaaattgaaaaaatccgaccattattaatagatggaactgcatcattgagtcctggaatgatgatgggcatgttcaatatgttgagcaccgtcttgggcgtctccattctgaatcttgggcaaaagagatacaccaagactacttactggtgggatggtcttcaatcgtctgatgattttgctttgattgtgaatgcacccaactatgcaggaattcaagctggagttgacaggttttatcgaacctgtaagctgctcggaattaatatgagcaaaaagaagtcttacataaacagaacaggtacctttgaattcacgagctttttctatcgttatgggtttgttgccaatttcagcatggagcttcctagttttggggtgtctggggtcaatgaatctgcagacatgagtattggagtcacggtcatcaaaaacaatatgataaacaatgaccttggcccagcaactgctcaaatggcccttcagttatttataaaagattacaggtacacttatcgatgccacagaggtgacacacaaatacaaacccggagatcatttgagataaagaaactatgggaccaaacccgctccaaagctgggctgttggtctctgatggaggccccaatttatataacattagaaatctccatattcctgaagtttgcttgaaatgggagttgatggatgaggattaccaggggcgtttatgcaacccattaaacccgtttgtcagccataaagagattgaatcagtgaacaatgcagtgataatgccggcacatggcccagccaaaaatatggagtatgacgctgttgcaacaacacactcctgggtccccaaaagaaatcgatccattttgaacacgagccaaagggggatacttgaagatgagcaaatgtatcagaggtgctgcaatttatttgaaaaattcttcccaagtagctcatacagaagaccagttggaatatccagtatggtagaggctatggtttcaagagcccgaattgatgcacggattgatttcgaatctggaaggataaagagggaggagtttgctgagatcatgaagatctgttccaccattgaagacctcagacggcaaaagtag

>H1N1_PB1_CY027673_A/Illinois/UR06-0088/2007

atggatgtcaatccgacattacttttcttaaaagtgccagcacaaaatgctataagcacaacttttccttatactggtgaccctccttacagccatgggacaggaacagggtacaccatggatacagtcaacaggacacaccagtactcagaaagaggaagatggacaaaaaataccgaaactggagcaccgcaactcaacccaattgatgggccactaccagaagacaatgaaccaagtggctatgcccaaacagattgtgtattagaagcaatgtctttccttgaagaatcccatcctggtatctttgaaaactcttgtattgaaacaatggaggttgttcagcaaacaagggtggacaaactgacacaaggcagacagacctatgactggactctaaataggaaccagcctgctgcaacagcattggcaaacactatagaagtattcagatcaaacggcctcatagcaaatgaatctggaaggctaatagacttccttaaagatgtaatggagtcgatggacagagacgaagtagaggtcacaactcattttcaaagaaagaggagagtgagagacaatgtaactaaaaaaatggtgactcaaagaacaataggcaaaaagaaacataaattagacaaaagaagttacctaattagggcattaaccctgaacacaatgaccaaagatgctgagagggggaaactaaaacgcagagcaattgcaaccccaggaatgcaaataagagggtttgtatactttgttgagacactggcaagaagcatatgtgaaaagcttgaacaatcaggattgccagttggaggaaatgagaagaaagcaaagttagcaaatgttgtaaggaagatgatgaccaactcccaggacactgaaatttctttcaccatcactggagataacacaaaatggaacgaaaatcaaaaccctagaatgttcttggccatgatcacatatataaccaaaaatcagcctgaatggttcagaaatattctaagtattgctccaataatgttttcaaacaagatggcgagactaggtaaggggtacatgtttgaaagcaagagtatgaaactgagaactcaaatacctgcagagatgctagccaacatagatttgaaatatttcaatgattcaactaaaaagaaaattgaaaaaatccgaccattattaatagatggaactgcatcattgagtcctggaatgatgatgggcatgttcaatatgttgagcaccgtcttgggcgtctccattctgaatcttgggcaaaagagatacaccaagactacttactggtgggatggtcttcaatcgtctgatgattttgctttgattgtgaatgcacccaactatgcaggaattcaagctggagttgacaggttttatcgaacctgtaagctgctcggaattaatatgagcaaaaagaagtcttacataaacagaacaggtacctttgaattcacgagctttttctatcgttatgggtttgttgccaatttcagcatggagcttcctagttttggggtgtctggggtcaatgaatctgcagacatgagtattggagtcactgtcatcaaaaacaatatgataaacaatgaccttggcccagcaactgctcaaatggcccttcagttatttataaaagattacaggtacacttatcgatgccacagaggtgacacacaaatacaaacccggagatcatttgagataaagaaactatgggaccaaacccgctccaaagctgggctgttggtctctgatggaggccccaatttatataacattagaaatctccatattcctgaagtttgcttgaaatgggagttgatggatgaggattatcaggggcgtttatgcaacccattaaacccgtttgtcagccataaagagattgaatcagtgaacaatgcagtgataatgccggcacatggcccagccaaaaatatggagtatgacgctgttgcaacaacacactcctgggtccccaaaagaaatcgatccattttgaacacgagccaaagggggatacttgaagatgagcaaatgtatcagaggtgctgcaatttatttgagaaattcttcccaagtagctcatacagaagaccagttggaatatccagtatggtagaggctatggtttcaagagcccgaattgatgcacggattgatttcgaatctggaaggataaagagggaggaatttgctgagatcatgaagatctgttccaccattgaagacctcagacggcaaaaatag

>H1N1_PB1_CY028425_A/Illinois/UR06-0215/2007

atggatgtcaatccgacattacttttcttaaaagtgccagcacaaaatgctataagcacaacttttccttatactggtgaccctccttacagccatgggacaggaacagggtacaccatggatacagtcaacaggacacaccagtactcagaaagaggaagatggacaaaaaataccgaaactggagcaccgcaactcaacccaattgatgggccactaccagaagacaatgaaccgagtggctatgcccaaacagattgtgtattagaagcaatggctttccttgaagaatcccatcctggtatctttgaaaactcttgtattgagacaatggaggttgttcagcaaacaagggtggacaaactaacacaaggcagacagacctatgactggactctaaataggaaccagcctgctgccacagcattggcaaacactatagaagtattcagatcaaacggcctcatagcaaatgaatctgggaggctaatagacttccttaaagatgtaatggagtcgatggacatagacgaaatagaggtcacaactcattttcaaagaaagaggagagtgagagacaatgtaactaaaaaaatggtgacccaaagaacaataggcaaaaagaaacataaattagacaaaagaagttacctaattagggcattaaccctgaacacaatgaccaaagatgctgagagagggaaactaaaacgcagagcaattgcaaccccaggaatgcaaataagggggtttgtatactttgttgagacactggcaagaagcatatgtgaaaagcttgaacaatcaggattgccagttggaggaaatgagaagaaagcaaagttagcaaatgttgtaaggaagatgatgaccaactcccaggacactgaaatttctttcaccatcactggagataacacaaaatggaacgaaaatcaaaaccctagaatgttcttggccatgatcacatatataaccaaaaatcagcctgaatggttcagaaatattctaagtattgccccaataatgttttcaaacaagatggcgagactaggtaaggggtacatgtttgaaagcaagagtatgaaactgagaactcaaatacctgcagagatgctagccaacatagatttgaaatatttcaatgattcaactaaaaagaaaattgaaaaaatccgaccattattaatagatggaactgcatcattgagtcctggaatgatgatgggcatgttcaatatgttgagcaccgtcttgggcgtctccattctgaatcttgggcaaaagagatacaccaagactacttactggtgggatggtcttcaatcgtctgatgattttgctttgattgtgaatgcacccaactatgcaggaattcaagctggagttgacaggttttatcgaacctgtaagctgctcggaattaatatgagcaaaaagaagtcttacataaacagaacaggtacctttgaattcacgagctttttctatcgttatgggtttgttgccaatttcagcatggagcttcctagttttggggtgtctggggtcaatgaatctgcagacatgagtattggagtcactgtcatcaaaaacaatatgataaacaatgaccttggcccagcaactgctcaaatggcccttcagttatttataaaagattacaggtacacttatcgatgccacagaggtgacacacaaatacaaacccggagatcatttgagataaagaaactatgggaccaaacccgctccaaagctgggctgttggtctctgatggaggccccaatttatataacattagaaatctccatattcctgaagtttgcttgaaatgggagttgatggatgaggattaccaggggcgtttatgcaacccattaaacccgtttgtcagccataaagagattgaatcagtaaacaatgcagtgataatgccggcacatggtccagccaaaaatatggagtatgacgctgttgcaacaacacactcctgggtccccaaaagaaatcgatccattttgaacacgagccaaagggggatacttgaagatgagcaaatgtatcagaggtgctgcaatttatttgaaaaattcttcccaagtagctcatacagaagaccagttggaatatccagtatggtagaggctatggtttcaagagcccgaattgatgcacggattgatttcgaatctggaaggataaagaaagaggaatttgctgagatcatgaagatctgttccaccattgaagacctcagacggcaaaaatag

>H1N1_PB1_CY028473_A/California/UR06-0552/2007

atggatgtcaatccgacattacttttcttaaaagtgccagcacaaaatgctataagcacaacttttccttatactggtgaccctccttacagccatgggacaggaacagggtacaccatggatacagtcaacaggacacaccagtactcagaaagaggaagatggacaaaaaataccgaaactggagcaccgcaactcaacccaattgatgggccactaccagaagacaatgaaccaagtggctatgcccaaacagattgtgtattagaagcaatggctttccttgaagaatcccatcctggtatctttgaaaactcttgtattgaaacaatggaggttgttcagcaaacaagggtggacaaactgacacaaggcagacagacctatgactggactctaaataggaaccagcctgctgcaacagcattggcaaacactatagaagtattcagatcaaacggcctcatagcaaatgaatctggaaggctaatagacttccttaaagatgtaatggagtcgatggacagagacgaagtagaggtcacaactcattttcaaagaaagaggagagtaagagacaatgtaactaaaaaaatggtgactcaaagaacaataggcaaaaagaaacataaattagacaaaagaagttacctaattagggcattaaccctgaacacaatgaccaaagatgctgagagggggaaactaaaacgcagagcaattgcaaccccaggaatgcaaataagagggtttgtatactttgttgagacactggcaagaagcatatgtgaaaagcttgaacaatcaggattgccagttggaggaaatgagaagaaagcaaagttagcaaatgttgtaaggaagatgatgaccaactcccaggacactgaaatttctttcaccatcactggagataacacaaaatggaacgaaaatcaaaaccctagaatgttcttggccatgatcacatatataaccaaaaatcagcctgaatggttcagaaatattctaagtattgctccaataatgttttcaaacaagatggcgagactaggtaaggggtacatgtttgaaagcaagagtatgaaactgagaactcaaatacctgcagagatgctagccaacatagatttgaaatatttcaatgattcaactaaaaagaaaattgaaaaaatccgaccattattaatagatggaactgcatcattgagtcctggaatgatgatgggcatgttcaatatgttgagcaccgtcttgggcgtctccattctgaatcttgggcaaaagagatacaccaagactacttactggtgggatggtcttcaatcgtctgatgattttgctttgattgtgaatgcacccaactatgcaggaattcaagctggagttgacaggttttatcgaacctgtaagctgctcggaattaatatgagcaaaaagaagtcttacataaacagaacaggtacctttgaattcacgagctttttctatcgttatgggtttgttgccaatttcagcatggagcttcctagttttggggtgtctggggtcaatgaatctgcagacatgagtattggagtcactgtcatcaaaaacaatatgataaacaatgaccttggcccagcaactgctcaaatggcccttcagttatttataaaagattacaggtacacttatcgatgccacagaggtgacacacaaatacaaacccggagatcatttgagataaagaaactatgggaccaaacccgctccaaagctgggctgttggtctctgatgggggccccaatttatataacattagaaatctccatattcctgaagtttgcttgaaatgggagttgatggatgaggattaccaggggcgtttatgcaacccattaaacccgtttgtcagccataaagagattgaatcagtgaacaatgcagtgataatgccggcacatggcccagtcaaaaatatggagtatgacgctgttgcaacaacacactcctgggtccccaaaagaaatcgatccattttgaacacgagccaaagggggatacttgaagatgagcaaatgtatcagaggtgctgcaatttatttgaaaaattcttcccaagtagctcatacagaagaccagttggaatatccagtatggtagaggctatggtttcaagagcccgaattgatgcacggattgatttcgaatctggaaggataaagagggaggaatttgctgagatcatgaagatctgttccaccattgaagacctcagacggcaaaaatag

>H1N1_PB1_GQ117075_A/Arizona/02/2009

atggatgtcaatccgactctacttttcctaaaaactccagcgcaaaatgccataagcaccacattcccttatactggagatcctccatacagccatggaacaggaacaggatacaccatggacacagtaaacagaacacaccaatactcagaaaagggaaagtggacgacaaacacagagactggtgcaccccagctcaacccgattgatggaccactacctgaggataatgaaccaagtgggtatgcacaaacagactgtgttctagaggctatggctttccttgaagaatcccacccaggaatatttgagaattcatgccttgaaacaatggaagttgttcaacaaacaagggtagataaactaactcaaggtcgccagacttatgattggacattaaacagaaatcaaccggcagcaactgcattggccaacaccatagaagtctttagatcgaatggcctaacagctaatgagtcaggaaggctgatagatttcttaaaggatgtaatggaatcaatgaacaaagaggaaatagagataacaacccactttcaaagaaaaaggagagtaagagacaacatgaccaagaagatggtcacgcaaagaacaatagggaagaaaaaacaaagactgaataagagaggctatctaataagagcactgacattaaatacgatgaccaaagatgcagagagaggcaagttaaaaagaagggctatcgcaacacctgggatgcagattagaggtttcgtatactttgttgaaactttagctaggagcatttgcgaaaagcttgaacagtctgggctcccagtagggggcaatgaaaagaaggccaaactggcaaatgttgtgagaaagatgatgactaattcacaagacacagagatttctttcacaatcactggggacaacactaagtggaatgaaaatcaaaatcctcgaatgttcctggcgatgattacatatatcaccagaaatcaacccgagtggttcagaaacatcctgagcatggcacccataatgttctcaaacaaaatggcaagactagggaaagggtacatgttcgagagtaaaagaatgaagattcgaacacaaataccagcagaaatgctagcaagcattgacctgaagtacttcaatgaatcaacaaagaagaaaattgagaaaataaggcctcttctaatagatggcacagcatcactgagtcctgggatgatgatgggcatgttcaacatgctaagtacggtcttgggagtctcgatactgaatcttggacaaaagaaatacaccaagacaatatactggtgggatgggctccaatcatccgacgattttgctctcatagtgaatgcaccaaaccatgagggaatacaagcaggagtggacagattctacaggacctgcaagttagtgggaatcaacatgagcaaaaagaagtcctatataaataagacagggacatttgaattcacaagctttttttatcgctatggatttgtggctaattttagcatggagctacccagctttggagtgtctggagtaaatgaatcagctgacatgagtattggagtaacagtgataaagaacaacatgataaacaatgaccttggacctgcaacggcccagatggctcttcaattgttcatcaaagactacagatacacatataggtgccataggggagacacacaaattcagacgagaagatcatttgagttaaagaagctgtgggatcaaacccaatcaaaggtagggctattagtatcagatggaggaccaaacttatacaatatacggaatcttcacattcctgaagtctgcttaaaatgggagctaatggatgatgattatcggggaagactttgtaatcccctgaatccctttgtcagtcataaagagattgattctgtaaacaatgctgtggtaatgccagcccatggtccagccaaaagcatggaatatgatgccgttgcaactacacattcctggattcccaagaggaatcgttctattctcaacacaagccaaaggggaattcttgaggatgaacagatgtaccagaagtgctgcaatctattcgagaaatttttccctagcagttcatataggagaccggttggaatttctagcatggtggaggccatggtgtctagggcccggattgatgccagggtcgacttcgagtctggacgaatcaagaaagaagagttctctgagatcatgaagatctgttccaccattgaagaactcagacggcaaaaataa

>H1N1_PB1_CY040248_A/Managua/3307.01/2008

atggatgtcaatccgacattacttttcttaaaagtgccagcacaaaatgctataagcacaacttttccttatactggtgaccctccttacagccatgggacaggaacagggtacaccatggatacagtcaacaggacacaccagtactcagaaagaggaagatggacaaaaaataccgaaactggagcaccgcaacttaacccaattgatggtccactaccggaagacaatgaaccaagtggctatgcccaaacagattgtgtattagaagcaatggctttccttgaagaatcccatcccggtatctttgaaaactcttgtattgaaacaatggaggttgttcagcaaacaagggtggacaaactgacacaaggcagacagacctatgactggactctaaataggaaccagcctgctgccacagcattggcaaacactatagaagtattcagatcaaacggcctcatagcaaatgaatctgggaggctaatagacttccttaaagatgtaatggagtcgatggacagaggcgaagtagaggtcacaactcattttcaaagaaagaggagagtgagagacaatgtaactaaaaaaatggtgacccaaagaacaataggcaaaaagaaacataaattagacaaaagaagttacctaattagggcattaaccctgaacacaatgaccaaagatgctgagagggggaaactaaaacgcagagcaattgcaaccccaggaatgcaaataagggggtttgtatactttgttgagacactggcaagaagcatatgtgaaaagcttgaacaatcaggattgccagttggaggaaatgagaagaaagcaaagttagcaaatgttgtaaggaagatgatgaccaactcccaggacactgaaatttctttcaccataaccggagataacacaaaatggaacgaaaatcaaaaccctagaatgttcttggccatgatcacatatataaccaaaaatcagcctgaatggttcagaaatattctaagtattgctccaataatgttttcaaacaagatggcgagactaggtaaggggtacatgtttgaaagcaagagtatgaaactgagaactcaaatacctgcagagatgctagccaacatagatttgaaatatttcaatgattcaactaaaaagaaaattgaaaaaatccgaccattattaatagatggaactgcatcattgagtcctggaatgatgatgggcatgttcaatatgttgagcaccgtcttgggcgtctccattctgaatcttgggcaaaagagatacaccaagactacttactggtgggatggtcttcaatcgtctgatgattttgctttgattgtgaatgcacccaactatgcaggaattcaagctggagttgacaggttttatcgaacctgtaagctgctcggaattaatatgagcaaaaagaagtcttacataaacagaacaggtacctttgaattcacgagctttttctatcgttatgggtttgttgccaatttcagcatggagcttcctagttttggggtgtctggggtcaatgaatctgcagacatgagtattggagtcactgtcatcaaaaacaatatgataaacaatgaccttggcccagcaactgctcaaatggcccttcagttatttataaaagattacaggtacacttatcgatgccaccgaggtgacacacaaatacaaacccggagatcatttgagataaagaaactatgggaccaaacccgctcaaaagctgggctgttggtctctgatggaggccccaatttatataacattagaaatctccatattcctgaagtttgcttgaaatgggagttgatggatgaggattaccaggggcgtttatgcaacccattaaacccgtttgtcagccataaagagattgaatcagtgaacaatgcagtgataatgccggcacatggtccagccaaaaatatggagtatgacgctgttgcaacaacacactcctgggtccccaaaagaaatcgatccattttgaacacgagccaaagggggatacttgaagatgagcaaatgtatcagaggtgctgcaatttatttgaaaaattcttcccaagtagctcatacagaagaccagttggaatatccagtatggtagaggctatggtctcaagagcccgaattgatgcacggattgatttcgaatctggaaggataaagaaagaggaatttgctgagatcatgaagatctgttccaccattgaagacctcagacggcaaaaatga

>H1N1_PB1_CY041208_A/New York/3199/2009

atggatgtcaatccgactctacttttcctaaaaattccagcgcaaaatgccataagcaccacattcccttatactggagatcctccatacagccatggaacaggaacaggatacaccatggacacagtaaacagaacacaccaatactcagaaaagggaaagtggacgacaaacacagagactggtgcaccccagctcaacccgattgatggaccactacctgaggataatgaaccaagtgggtatgcacaaacagactgtgttctagaggctatggctttccttgaagaatcccacccaggaatatttgagaattcatgccttgaaacaatggaagttgttcaacaaacaagggtagataaactaactcaaggtcgccagacttatgattggacattaaacagaaatcaaccggcagcaactgcattggccaacaccatagaagtctttagatcgaatggcctaacagctaatgagtcaggaaggctaatagatttcttaaaggatgtaatggaatcaatgaacaaagaggaaatagagataacaacccactttcaaagaaaaaggagagtaagagacaacatgaccaagaagatggtcacgcaaagaacaatagggaagaaaaaacaaagactgaataagagaggctatctaataagagcactgacattaaatacgatgaccaaagatgcagagagaggcaagttaaaaagaagggctatcgcaacacctgggatgcagattagaggtttcgtatactttgttgaaactttagctaggagcatttgcgaaaagcttgaacagtctgggctcccagtagggggcaatgaaaagaaggccaaactggcaaatgttgtgagaaagatgatgactaattcacaagacacagagatttctttcacaatcactggggacaacactaagtggaatgaaaatcaaaatcctcgaatgttcctggcgatgattacatatatcaccagaaatcaacccgagtggttcagaaacatcctgagcatggcacccataatgttctcaaacaaaatggcaagactagggaaagggtacatgttcgagagtaaaagaatgaaaattcgaacacaaataccagcagaaatgctagcaagcattgacctgaagtacttcaatgaatcaacaaagaagaaaattgagaaaataaggcctcttctaatagatggcacagcatcactgagtcctgggatgatgatgggcatgttcaacatgctaagtacggtcttgggagtctcgatactgaatcttggacaaaagaaatacaccaagacaatatactggtgggatgggctccaatcatccgacgattttgctctcatagtgaatgcaccaaaccatgagggaatacaagcaggagtggacagattctacaggacctgcaagttagtgggaatcaacatgagcaaaaagaagtcctatataaataagacagggacatttgaattcacaagctttttttatcgctatggatttgtggctaattttagcatggagctacccagctttggagtgtctggagtaaatgaatcagctgacatgagtattggagtaacagtgataaagaacaacatgataaacaatgaccttggacctgcaacggcccagatggctcttcaattgttcatcaaagactacagatacacatataggtgccataggggagacacacaaattcagacgagaagatcatttgagttaaagaagctgtgggatcaaacccaatcaaaggtagggctattagtatcagatggaggaccaaacttatacaatatacggaatcttcacattcctgaagtctgcttaaaatgggagctaatggatgatgattatcggggaagactttgtaatcccctgaatccctttgtcagtcataaagagattgattctgtaaacaatgctgtggtaatgccagcccatggtccagccaaaagcatggaatatgatgccgttgcaactacacattcctggattcccaagaggaatcgttctattctcaacacaagccaaaggggaattcttgaggatgaacagatgtaccagaagtgctgcaatctattcgagaaatttttccctagcagttcatataggagaccggttggaatttctagcatggtggaggccatggtgtctagggcccggattgatgccagggtcgacttcgagtctggacggatcaagaaagaagagttctctgagatcatgaagatctgttccaccattgaagaactcagacggcaaaaataa

>H1N1_PB1_GQ377066_A/Arizona/01/2009

atggatgtcaatccgactctacttttcctaaaaattccagcgcaaaatgccataagcaccacattcccttatactggagatcctccatacagccatggaacaggaacaggatacaccatggacacagtaaacagaacacaccaatactcagaaaagggaaagtggacgacaaacacagagactggtgcaccccagctcaacccgattgatggaccactacctgaggataatgaaccaagtgggtatgcacaaacagactgtgttctagaggctatggctttccttgaagaatcccacccaggaatatttgagaattcatgccttgaaacaatggaagttgttcaacaaacaagggtagataaactaactcaaggtcgccagacttatgattggacattaaacagaaatcaaccggcagcaactgcattggccaacaccatagaagtctttagatcgaatggcctaacagctaatgagtcaggaaggctaatagatttcttaaaggatgtaatggaatcaatgaacaaagaggaaatagagataacaacccactttcaaagaaaaaggagagtaagagacaacatgaccaagaagatggtcacgcaaagaacaatagggaagaaaaaacaaagactgaataagagaggctatctaataagagcactgacattaaatacgatgaccaaagatgcagagagaggcaagttaaaaagaagggctatcgcaacacctgggatgcagattagaggtttcgtatactttgttgaaactttagctaggagcatttgcgaaaagcttgaacagtctgggctcccagtagggggcaatgaaaagaaggccaaactggcaaatgttgtgagaaagatgatgactaattcacaagacacagagatttctttcacaatcactggggacaacactaagtggaatgaaaatcaaaatcctcgaatgttcctggcgatgattacatatatcaccagaaatcaacccgagtggttcagaaacatcctgagcatggcacccataatgttctcaaacaaaatggcaagactagggaaagggtacatgttcgagagtaaaagaatgaagattcgaacacaaataccagcagaaatgctagcaagcattgacctgaagtacttcaatgaatcaacaaagaagaaaattgagaaaataaggcctcttctaatagatggcacagcatcactgagtcctgggatgatgatgggcatgttcaacatgctaagtacggtcttgggagtctcgatactgaatcttggacaaaagaaatacaccaagacaatatactggtgggatgggctccaatcatccgacgattttgctctcatagtgaatgcaccaaaccatgagggaatacaagcaggagtggacagattctacaggacctgcaagttagtgggaatcaacatgagcaaaaggaagtcctatataaataagacagggacatttgaattcacaagctttttttatcgctatggatttgtggctaattttagcatggagctacccagctttggagtgtctggagtaaatgaatcagctgacatgagtattggagtaacagtgataaagaacaacatgataaacaatgaccttggacctgcaacggcccagatggctcttcaattgttcatcaaagactacagatacacatataggtgccataggggagacacacaaattcagacgagaagatcatttgagttaaagaagctgtgggatcaaacccaatcaaaggtagggctattagtatcagatggaggaccaaacttatacaatatacggaatcttcacattcctgaagtctgcttaaaatgggagctaatggatgatgattatcggggaagactttgtaatcccctgaatccctttgtcagtcataaagagattgattctgtaaacaatgctgtggtaatgccagcccatggtccagccaaaagcatggaatatgatgccgttgcaactacacattcctggattcccaagaggaatcgttctattctcaacacaagccaaaggggaattcttgaggatgaacagatgtaccagaagtgctgcaatctattcgagaaatttttccctagcagttcatataggagaccggttggaatttctagcatggtggaggccatggtgtctagggcccggattgatgccagggtcgacttcgagtctggacggatcaagaaagaagagttctctgagatcatgaagatctgttccaccattgaagaattcagacggcaaaaataa

>H1N1_PB1_GQ457566_A/Saskatchewan/5131/2009

atggatgtcaatccgactctacttttcctaaaggttccagcgcaaaatgccataagcaccacattcccttatactggagatcctccatacagccatggaacaggaactggatacaccatggacacagtcaacagaacacaccaatattcagaaaaagggaaatggacgacaaacacagaaactggggcaccccagctcaacccgattgatggaccattacctgaagataatgaaccaagtggatatgcacaaacagattgtgtcctggaggccatggctttccttgaagaatcccacccagggatatttgagaattcatgccttgaaacaatggaaattgtccaacaaacaagggtggataaactaactcaaggtcgccagacttatgattggacattaaacagaaatcaaccggcagcaactgcattggccaacaccatagaagtttttaggtcaaatggtctaacagctaatgagtcaggaaggctaatagatttcctaaaggatgtaatggaatcaatggataaagaggaaatagagataacaacacactttcaaagaaaaaggagagtgagagacaacatgaccaagaagatggtcacacaaagaacaatagggaagaaaaaacaaagagtgaataagagaagttatctaataagagcactgacattgaatacgatgaccaaagatgcagagagaggcaaattaaaaaggagggctatcgcaacacctgggatgcaaattagagggttcgtgtactttgttgagactttagctagaagcatttgcgaaaagcttgaacagtccggactcccagtagggggcaatgaaaadaaagccaaattggcaaatgttgtaagaaagatgatgactaattcacaagacacagagctttctttcacaatcactggggacaacactaaatggaatgaaaaccaaaatcctcgaatgttcctggcgatgattacttacatcaccagaaatcaacccgagtggttcagaaacatactgagcatggcaccaataatgttctcaaacaaaatggcaagactaggaaaagggtacatgttcgagagtaagagaatgaagctccgaacacagataccagcagaaatgctagcaagcattgacctgaagtatttcaatgaatcaacaaggaagaaaattgagaaaataaggcctctcctaatagatggcacagcatcattgagccctgggatgatgatgggcatgttcaacatgttaagtacggttttgggagtctcaatactgaatcttggacaaaagaagtacacccggacaacatactggtgggatgggctccaatcctctgacgattttgccctcatagtaaatgcaccaaatcatgagggaatacaagcaggagtggatagattctacaggacctgcaaattagtggggatcaacatgagcaaaaagaagtcctatataaataagactgggacatttgaattcacaagctttttttatcgctatgggtttgtagctaattttagcatggagctgcccagttttggagtgtctggaataaacgaatcagctgatatgagtattggagtaacagtgataaagaacaacatgataaataatgatcttgggcctgcaacagcccagatggcccttcagttgttcatcaaagactacagatacacatatagatgccatagaggggacacagaaattcagacgagaagatcattcgagctaaagagcctgtgggatcaaacccaatcaaaggcaggattattagtatctgatggaggaccaaatttatacaatatccggaatcttcacattcctgaagtctgcttaaaatgggagctaatggatgaggattatcggggaagactttgtaatcatctgaatccctttgtcagccataaagagattgattctgtaaacagtgctgtggtgatgccagcccatggtccagccaaaagcatggaatatgatgccgttgcaactacacactcctggattcccaagaggaaccgctctattctcaacacaagccaaaggggaattcttgaggatgaacagatgtaccagaagtgctgcaacctgttcgagaaatttttccctagtagttcatacaggagaccggttggaatttctagcatggtggaggccatggtgtctagggcccggattgatgccaggattgacttcgagtctggacggattaagaaagaagagttctctgagatcatgaagatctgttccaccattgaagaactcagacggcaaaagtaa

>H1N1_PB1_CY044995_A/New York/3751/2009

atggatgtcaatccgactctacttttcctaaaaattccagcgcaaaatgccataagcaccacattcccttatactggagatcctccatacagccatggaacaggaacaggatacaccatggacacagtaaacagaacacaccaatactcagaaaagggaaagtggacgacaaacacagagactggtgcaccccagctcaacccgattgatggaccactacctgaggataatgaaccaagtgggtatgcacaaacagactgtgttctagaggctatggctttccttgaagaatcccacccaggaatatttgagaattcatgccttgaaacaatggaagttgttcaacaaacaagggtagataaactaactcaaggtcgccagacttatgattggacattaaacagaaatcaaccggcagcaactgcattggccaacaccatagaagtctttagatcgaatggcctaacagctaatgagtcaggaaggctaatagatttcttaaaggatgtaatggaatcaatgaacaaagaggaaatagagataacaacccactttcaaagaaaaaggagagtaagagacaacatgaccaagaagatggtcacacaaagaacaatagggaagaaaaaacaaagactgaataagagaggctatctaataagagcactgacattaaatacaatgaccaaagatgcagagagaggcaagttaaaaagaagggctatcgcaacacctgggatgcagattagaggtttcgtatactttgttgaaactttagctaggagcatttgcgaaaagcttgaacagtctgggctcccagtagggggcaatgaaaagaaggccaaactggcaaatgttgtgagaaagatgatgactaattcacaagacacagagatttctttcacaatcactggggacaacactaagtggaatgaaaatcaaaatcctcgaatgttcctggcgatgattacatatatcaccagaaatcaacccgagtggttcagaaacatcctaagcatggcacccataatgttctcaaacaaaatggcaagactagggaaagggtacatgttcgagagtaaaagaatgaagattcgaacacaaataccagcagaaatgctagcaagcattgacctgaagtacttcaatgaatcaacaaagaagaaaattgagaaaataaggcctcttctaatagatggcacagcatcactgagtcctgggatgatgatgggcatgttcaacatgctaagtacggtcttgggagtctcgatactgaatcttggacaaaagaaatacaccaagacaatatactggtgggatgggctccaatcatccgacgattttgctctcatagtgaatgcaccaaaccatgagggaatacaagcaggagtggacagattctacaggacctgcaagttagtgggaatcaacatgagcaaaaagaagtcctatataaataagacagggacatttgaattcacaagctttttttatcgctatggatttgtggctaattttagcatggagctacccagctttggagtgtctggagtaaatgaatcagctgacatgagtattggagtaacagtgataaagaacaacatgataaacaatgaccttggacctgcaacggcccagatggctcttcaattgttcatcaaagactacagatacacatataggtgccataggggagacacacaaattcagacgagaagatcatttgagttaaagaagctgtgggatcaaacccaatcaaaggtagggctattagtatcagatggaggtccaaacttatacaatatacggaatcttcacattcctgaagtctgcttaaaatgggagctaatggatgatgattatcggggaagactttgtaatcccctgaatccctttgtcagtcataaagagattgattctgtaaacaatgctgtggtaatgccagcccatggtccagccaaaagcatggaatatgatgccgttgcaactacacattcctggattcccaagaggaatcgttctattctcaacacaagccaaaggggaattcttgaggatgaacagatgtaccagaagtgttgcaatctattcgagaaatttttccctagcagttcatataggagaccggttggaatttctagcatggtggaggccatggtgtctagggcccggattgatgccagggtcgacttcgagtctggacggatcaagaaagaagagttctctgagatcatgaagatctgttccaccattgaagaactcagacggcaaaaataa

>H1N1_PB1_CY051669_A/New York/4735/2009

atggatgtcaatccgactctacttttcctaaaaattccagcgcaaaatgccataagcaccacattcccttatactggagatcctccatacagccatggaacaggaacaggatacaccatggacacagtaaacagaacacaccaatactcagaaaagggaaagtggacgacaaacacagagactggtgcaccccagctcaacccgatagatggaccactacctgaggataatgaaccaagtgggtatgcacaaacagactgtgttctagaggctatggctttccttgaagaatcccacccaggaatatttgagaattcatgccttgaaacaatggaagttgttcaacaaacaagggtagataaactaactcaaggtcgccagacttatgattggacattaaacagaaatcaaccggcagcaactgcattggccaacaccatagaagtctttagatcgaatggcctaacagctaatgagtcaggaaggctaatagatttcttaaaggatgtaatggaatcaatgaacaaagaggaaatagagataacaacccactttcaaagaaaaaggagagtaagagacaacatgaccaagaagatggtcacgcaaagaacaatagggaagaaaaaacaaagactgaataagagaggctatctaataagagcactgacattaaatacgatgaccaaagatgcagagagaggcaagttaaaaagaagggctatcgcaacacctgggatgcagattagaggtttcgtatactttgttgaaactttagctaggagcatttgcgaaaagcttgaacagtctgggctcccagtagggggcaatgaaaagaaggccaaactggcaaatgttgtgagaaagatgatgactaattcacaagacacagagatttctttcacaatcactggggacaacactaagtggaatgaaaatcaaaatcctcgaatgttcctggcgatgattacatatatcaccagaaatcaacccgagtggttcagaaacatcctgagcatggcacccataatgttctcaaacaaaatggcaagactagggaaagggtacatgttcgagagtaaaagaatgaagattcgaacacaaataccagcagaaatgctagcaagcattgacctgaagtacttcaatgaatcaacaaagaagaaaattgagaaaataaggcctcttctaatagatggcacagcatcactgagtcctgggatgatgatgggcatgttcaacatgctaagtacggtcttgggagtctcgatactgaatcttggacaaaagaaatacaccaagacaatatactggtgggatgggctccaatcatccgacgattttgctctcatagtgaatgcaccaaaccatgagggaatacaagcaggagtggacagattctacaggacctgcaagttagtgggaatcaacatgagcaaaaagaagtcctatataaataagactgggacatttgaattcacaagctttttttatcgctatggatttgtggctaattttagcatggagctacccagctttggagtgtctggagtaaatgaatcagctgacatgagtattggagtaacagtgataaagaacaacatgataaacaatgaccttggacctgcaacggcccagatggctcttcaattgttcatcaaagactacagatacacatataggtgccataggggagacacacaaattcagacgagaagatcatttgagttaaagaagctgtgggatcaaacccaatcaaaggtagggctattagtatcagatggaggaccaaacttatacaatatacggaatcttcacattcctgaagtctgcttaaaatgggagctaatggatgatgattatcggggaagactttgtaatcccctgaatccctttgtcagtcataaagagattgattctgtaaacaatgctgtggtaatgccagcccatggtccagccaaaagcatggaatatgatgccgttgcaactacacattcctggattcccaagaggaatcgttctattctcaacacaagccaaaggggaattcttgaggatgaacagatgtaccagaagtgctgcaatctattcgagaaatttttccctagcagttcatataggagaccggttggaatttctagcatggtggaggccatggtgtctagggcccggattgatgccagggtcgacttcgagtctggacggatcaagaaagaagagttctctgagatcatgaagatctgttccaccattgaagaactcagacggcaaaaataa

>H1N1_PB1_CY051765_A/New York/4823/2009

atggatgtcaatccgactctacttttcctaaaaattccagcgcaaaatgccataagcaccacattcccttatactggagatcctccatacagccatggaacaggaacaggatacaccatggacacagtaaacagaacacaccaatactcagaaaagggaaagtggacgacaaacacagagactggtgcaccccagctcaacccgattgatggaccactacctgaggataatgaaccaagtgggtatgcacaaacagactgtgttctagaggctatggctttccttgaagaatcccacccaggaatatttgagaactcatgccttgaaacaatggaagttgttcaacaaacaagggtagataaactaactcaaggtcgccagacttatgattggacattaaacagaaatcaaccggcagcaactgcattggccaacaccatagaagtctttagatcgaatggcctaacagctaatgagtcaggaaggctaatagatttcttaaaggatgtaatggaatcaatgaacaaagaggaaatagagataacaacccactttcaaagaaaaaggagagtaagagacaacatgaccaagaagatggtcacgcaaagaacaatagggaagaaaaaacaaagactgaataagagaggctatctaataagagcactgacattaaatacgatgaccaaagatgcagagagaggcaagttaaaaagaagggctatcgcaacacctgggatgcagattagaggtttcgtatactttgttgaaactttagctaggagcatttgcgaaaagcttgaacagtctgggctcccagtagggggcaatgaaaagaaggccaaactggcaaatgttgtgagaaagatgatgactaattcacaagacacagagatttctttcacaatcactggggacaacactaagtggaatgaaaatcaaaatcctcgaatgttcctggcgatgattacatatatcaccagaaatcaacccgagtggttcagaaacatcctgagcatggcacccataatgttctcaaacaaaatggcaagactagggaaagggtacatgttcgagagtaaaagaatgaagattcgaacacaaataccagcagaaatgctagcaagcattgacctgaagtacttcaatgaatcaacaaagaagaaaattgagaaaataaggcctcttctaatagatggcacagcatcactgagtcctgggatgatgatgggcatgttcaacatgctaagtacggtcttgggagtctcgatactgaatcttggacaaaagaaatacaccaagacaatatactggtgggatgggctccaatcatccgacgattttgctctcatagtgaatgcaccaaaccatgagggaatacaagcaggagtggacagattctacaggacctgcaagttagtgggaatcaacatgagcaaaaagaagtcctatataaataagacagggacatttgaattcacaagctttttttatcgctatggatttgtggctaattttagcatggagctacccagctttggagtgtctggagtaaatgaatcagctgacatgagtattggagtaacagtgataaagaacaacatgataaacaatgaccttggacctgcaacggcccagatggctcttcaattgttcatcaaagactacagatacacatataggtgccataggggagacacacaaattcagacgagaagatcttttgagttaaagaagctgtgggatcaaacccaatcaaaggtagggctattagtatcagatggaggaccaaacttatacaatatacggaatcttcacattcctgaagtctgcttaaaatgggagctaatggatgatgattatcggggaagactttgtaatcccctgaatccctttgtcagtcataaagagattgattctgtaaacaatgctgtggtaatgccagcccatggtccagccaaaagcatggaatatgatgccgttgcaactacacattcctggattcccaagaggaatcgttctattctcaacacaagccaaaggggaattcttgaggatgaacagatgtaccagaagtgctgcaatctattcgagaaatttttccctagcagttcatataggagaccggttggaatttctagcatggtggaggccatggtgtctagggcccggattgatgccagggtcgacttcgagtctggacggatcaagaaagaagagttctctgagatcatgaagatctgttccaccattgaagaactcagacggcaaaaataa

>H1N1_PB1_CY052168_A/New York/4788/2009

atggatgtcaatccgactctacttttcctaaaaattccagcgcaaaatgccataagcaccacattcccttatactggagatcctccatacagccatggaacaggaacaggatacaccatggacacagtaaacagaacacaccaatactcagaaaagggaaagtggacgacaaacacagagactggtgcaccccagctcaacccgattgatggaccactacctgaggataatgaaccaagtgggtatgcacaaacagactgtgttctagaggctatggctttccttgaagaatcccacccaggaatatttgagaattcatgccttgaaacaatggaagttgttcaacaaacaagggtagataaactaactcaaggtcgccagacttatgattggacattaaacagaaatcaaccggcagcaactgcattggccaacaccatagaagtctttagatcgaatggcctaacagctaatgagtcaggaaggctaatagatttcttaaaggatgtaatggaatcaatgaacaaagaggaaatagagataacaacccactttcaaagaaaaaggagagtaagagacaacatgaccaagaagatggtcacgcaaagaacaatagggaagaaaaaacaaagactgaataagagaggctatctaataagagcactgacattaaatacgatgaccaaagatgcagagagaggcaagttaaaaagaagggctatcgcaacacctgggatgcagattagaggtttcgtatactttgttgaaactttagctaggagcatttgcgaaaagcttgaacagtctgggctcccagtagggggcaatgaaaagaaggccaaactggcaaatgttgtgagaaagatgatgactaattcacaagacacagagatttctttcacaatcactggggacaacactaagtggaatgaaaatcaaaatcctcgaatgttcctggcgatgattacatatatcaccagaaatcaacccgagtggttcagaaacatcctgagcatggcacccataatgttctcaaacaaaatggcaagactagggaaagggtacatgttcgagagtaaaagaatgaagattcgaacacaaataccagcagaaatgctagcaagcattgacctgaagtacttcaatgaatcaacaaagaagaaaattgagaaaataaggcctcttctaatagatggcacagcatcactgagtcctgggatgatgatgggcatgttcaacatgctaagtacggtcttgggagtctcgatactgaatcttggacaaaagaaatacaccaagacaatatactggtgggatgggctccaatcatctgacgattttgctctcatagtgaatgcaccaaaccatgagggaatacaagcaggagtggacagattctacaggacctgcaagttagtgggaatcaacatgagcaaaaagaagtcctatataaataagacagggacttttgaattcacaagctttttttatcgctatggatttgtggctaattttagcatggagctacccagctttggagtgtctggagtaaatgaatcagctgacatgagtattggagtaacagtgataaagaacaacatgataaacaatgaccttggacctgcaacggcccagatggctcttcaattgttcatcaaagactacagatacacatataggtgccataggggagacacacaaattcagacgagaagatcatttgagttaaagaagctgtgggatcaaacccaatcaaaggtagggctattagtatcagatggaggaccaaacttatacaatatacggaatcttcacattcctgaagtctgcttaaaatgggagctaatggatgatgattatcggggaagactttgtaatcccctgaatccctttgtcagtcataaagagattgattctgtgaacaatgctgtggtaatgccagcccatggtccagccaaaagcatggaatatgatgccgttgcaactacacattcctggattcccaagaggaatcgttctattctcaacacaagccaaaggggaattcttgaggatgaacagatgtaccagaagtgctgcaatctattcgaaaaatttttccctagcagttcatataggagaccggttggaatttctagcatggtggaggccatggtgtctagggcccggattgatgccagggtcgacttcgagtctggacggatcaagaaagaagagttctctgagatcatgaagatctgttccaccattgaagaactcagacggcaaaaataa

>H1N1_PB1_GU727829_A/IIV-Anadyr/177/2009

atggatgtcaatccgactctacttttcctaaaaattccagcacaaaatgccataagcaccacattcccttatactggagatcctccatacagccatggaacaggaacaggatacaccatggacacagtaaacagaacacaccaatactcagaaaagggaaagtggacgacaaacacagagactggtgcaccccagctcaacccgattgatggaccactacctgaggataatgaaccaagtgggtatgcacaaacagactgtgttctagaggctatggctttccttgaagaatcccacccaggaatatttgagaattcatgctttgaaacaatggaagttgttcaacaaacaagggtggataaactaactcaaggtcgccagacttatgattggacattaaacagaaatcaaccggcagcaactgcattggccaacaccatagaagtctttagatcgaatggcctaacagctaatgagtcaggaaggctaatagatttcttaaaggatgtaatggaatcaatgaacaaagaggaaatagagataacaacccactttcaaagaaaaaggagagtaagagacaacatgaccaagaagatggtcacgcaaagaacaatagggaagaaaaaacaaaggctgaataagagaggctatctaataagagcactgacattaaatacgatgaccaaagatgcagagagaggcaagttaaaaagaagggctatcgcaacacctgggatgcagattagaggtttcgtatactttgttgaaactttagctaggagcatttgcgaaaagcttgaacagtctgggctcccagtagggggcaatgaaaagaaggccaaactggcaaatgttgtgagaaaaatgatgactaattcacaagacacagagatttctttcacaatcactggggacaacactaagtggaatgaaaatcaaaatcctcgaatgttcctggcgatgattacatatatcaccagaaatcaacccgagtggttcagaaacatcctgagcatggcacccataatgttctcaaacaaaatggcaagactagggaaagggtacatgttcgagagtaaaagaatgaaaattcgaacacaaataccagcagaaatgctagcaagcattgacctgaagtacttcaatgaatcaacaaagaagaaaattgagaaaataaggcctcttctaatagatggcacagcatcactgagtcctgggatgatgatgggcatgttcaacatgctaagtacggtcttgggagtctcgatactgaatcttggacaaaagaaatacaccaagacaatatactggtgggatgggctccaatcatccgacgattttgctctcatagtgaatgcaccaaaccatgagggaatacaagcaggagtggacagattctacaggacctgcaagttagtgggaatcaacatgagcaaaaagaagtcctatataaataaaacagggacatttgaattcacaagctttttttatcgctatggatttgtggctaattttagcatggagctacccagctttggagtgtctggagtaaatgaatcagctgacatgagtattggagtaacagtgataaagaacaacatgataaacaatgaccttggacctgcaacggcccagatggctcttcaattgttcatcaaagactacagatacacatataggtgccataggggagacacacaaatccagacgagaagatcatttgagttaaagaagctgtgggatcaaacccaatcaaaggtagggctattagtatcagatggaggaccaaacttatacaatatacggaatcttcacattcctgaagtctgcttaaaatgggagctaatggatgatgattatcggggaagactttgtaatcccctgaatccctttgtcagtcataaagagattgattctgtaaacaatgctgtggtaatgccagcccatggtccagccaaaagcatggaatatgatgccgttgcaactacacattcctggattcccaagaggaatcgttctattctcaacacaagccaaaggggaattcttgaggatgaacagatgtaccagaagtgctgcaatctattcgagaaatttttccctagcagttcatataggagaccggttggaatttctagcatggtggaggccatggtgtctagggcccggattgatgccagggtcgacttcgagtctggacggatcaagaaagaagagttctctgagatcatgaagatctgttccaccattgaagaactcagacggcaaaaataa

>H1N1_PB1_CY056625_A/New York/5988/2009

atggatgtcaatccgactctacttttcctaaaaattccagcgcaaaatgccataagcaccacattcccttatactggagatcctccatacagccatggaacaggaacaggatacaccatggacacagtaaacagaacacaccaatactcagaaaagggaaagtggacgacaaacacagagactggtgcaccccagctcaacccgattgatggaccactacctgaggataatgaaccaagtgggtatgcacaaacagactgtgttctagaggctatggctttccttgaagaatcccacccaggaatatttgagaattcatgccttgaaacaatggaagttgttcaacaaacaagggtagataaactaactcaaggtcgccagacttatgattggacattaaacagaaatcaaccggcagcaactgcattggccaacaccatagaagtctttagatcgaatggcctaacagctaatgagtcaggaaggctaatagatttcttaaaggatgtaatggaatcaatgaacaaagaggaaatagagataacaacccactttcaaagaaaaaggagagtaagagacaacatgaccaaaaagatggtcacgcaaagaacaatagggaagaaaaaacaaagattgaataagagaggatatctaataagagcactgacattaaatacgatgaccaaagatgcagagagaggcaagttaaaaagaagggctatcgcaacacctgggatgcagattagaggtttcgtatactttgttgaaactttagctaggagcatttgcgaaaagcttgaacagtctgggctcccagtagggggcaatgaaaagaaggccaaactggcaaatgttgtgagaaagatgatgactaattcacaagacacagagatttctttcacaatcactggggacaacactaagtggaatgaaaatcaaaatcctcgaatgttcctggcgatgattacatatatcaccagaaatcaacccgagtggttcagaaacatcctgagcatggcacccataatgttctcaaacaaaatggcaagactagggaaagggtacatgttcgagagtaaaagaatgaagattcgaacacaaataccagcagaaatgctagcaagcattgacctgaagtacttcaatgaatcaacaaagaagaaaattgagaaaataaggcctcttctaatagatggcacagcatcactgagtcctgggatgatgatgggcatgttcaacatgctaagtacggtcttgggagtctcgatactgaatcttggacaaaagaaatacaccaagacagtatactggtgggatgggctccaatcatccgacgattttgctctcatagtgaatgcaccaaaccatgagggaatacaagcaggagtggacagattctacaggacctgcaagttagtgggaatcaacatgagcaaaaagaagtcctatataaataagacagggacatttgaattcacaagctttttttatcgctatggatttgtggctaattttagcatggagctacccagctttggagtgtctggagtaaatgaatcagctgacatgagtgttggagtaacagtgataaagaacaacatgataaacaatgatcttggacctgcaacggcccagatggctcttcaattgttcatcaaagactacagatacacatataggtgccataggggagacacacaaattcagacgagaagatcatttgagttaaagaagctgtgggatcaaacccaatcaaaggtagggctattagtatcagatggaggaccaaacttatacaatatacggaatcttcacattcctgaagtctgcttaaaatgggagctaatggatgatgattatcggggaagactttgtaatcccctgaatccctttgtcagtcataaagagattgattctgtaaacaatgctgtggtaatgccagcccatggtccagccaaaagcatggaatatgatgccgttgcaactacacattcctggattcccaagaggaatcgttctattctcaacacaagccaaaggggaattcttgaggatgaacagatgtaccagaagtgctgcaatctattcgagaaatttttccctagcagttcatataggagaccggttggaatttctagcatggtggaggccatggtgtctagggcccggattgatgccagggtcgacttcgagtctggacggatcaagaaagaagagttctctgagatcatgaagatctgttccaccattgaagaactcagacggcaaaaataa

>H1N1_PB1_CY056713_A/New York/6300/2009

atggatgtcaatccgactctacttttcctaaaaattccagcgcaaaatgccataagcaccacattcccttatactggagatcctccatacagccatggaacaggaacaggatacaccatggacacagtaaacagaacacaccaatactcagaaaagggaaagtggacgacaaacacagagactggtgcaccccagctcaacccgattgatggaccactacctgaggataatgaaccaagtgggtatgcacaaacagactgtgttctagaggctatggcttttcttgaagaatcccacccaggaatatttgagaattcatgccttgaaacaatggaagttgttcaacaaacaagggtagataaactaactcaaggtcgccagacttatgattggacattaaacagaaatcaaccggcagcaactgcattggccaacaccatagaagtctttagatcgaatggcctaacagccaatgagtcaggaaggctaatagatttcttaaaggatgtaatggaatcaataaacaaagaggaaatagagataacaacccactttcaaagaaaaaggagagtaagagacaacatgaccaagaagatggtcacgcaaagaacaatagggaagaaaaaacaaagactgaataagagaggctatctaataagagcactgacattaaatacgatgaccaaagatgcagagagaggcaagttaaaaagaagggctatcgcaacacctgggatgcagattagaggtttcgtatactttgttgaaactttagctaggagcatttgcgaaaagcttgaacagtctgggctcccagtagggggcaatgaaaagaaggccaaactggcaaatgttgtgagaaagatgatgactaattcacaagacacagagatttctttcacaatcactggggacaacactaagtggaatgaaaatcaaaatcctcgaatgttcctggcgatgattacatatatcaccagaaatcaacccgagtggttcagaaacatcctgagcatggcacccataatgttctcaaacaaaatggcaagactagggaaagggtacatgttcgagagtaaaagaatgaagattcgaacacaaataccagcagaaatgctagcaagcattgacctgaagtacttcaatgaatcaacaaagaagaaaattgagaaaataaggcctcttctaatagatggcacagcatcactgagtcctgggatgatgatgggcatgttcaacatgctaagtacggtcttgggagtctcgatactgaatcttggacaaaagaaatacaccaagacaatatactggtgggatgggctccaatcatccgacgattttgctctcatagtgaatgcaccaaaccatgagggaatacaagcaggagtggacagattctacaggacctgcaagttagtgggaatcaacatgagcaaaaagaagtcctatataaataaaacagggacatttgaattcacaagctttttttatcgctatggatttgtggctaattttagcatggagctacccagctttggagtgtctggagtaaatgaatcagctgacatgagtattggagtaacagtgataaagaacaacatgataaacaatgaccttggacctgcaacggcccagatggctcttcaactgttcatcaaagactacagatacacatataggtgccataggggagacacacaaattcagacgagaagatcatttgagttaaagaagctgtgggatcaaacccaatcaaaggtagggctattagtatcagatggaggaccaaacttatacaatatacggaatcttcacattcctgaagtctgcttaaaatgggagctaatggatgatgattatcggggaagactttgtaatcccctgaatccctttgtcagtcataaagagattgattctgtaaacaatgctgtggtaatgccagcccatggtccagccaaaagcatggagtatgatgctgttgcaactacacattcctggattcccaagaggaatcgttctattctcaacacaagccaaaggggaattcttgaggatgaacagatgtaccagaagtgctgcaatctattcgagaaatttttccctagcagttcatataggagaccggttggaatttctagcatggtggaggccatggtgtctagggcccggattgatgccagggtcgacttcgagtctggacggatcgggaaggaagagttctctgagatcatgaagatctgttccaccattgaagaactcagacggcaaaaataa

>H1N1_PB1_CY060532_A/Ontario/237882/2009

atggatgtcaatccgactctacttttcctaaaaattccagcgcaaaatgccataagcaccacattcccttatactggagatcctccatacagccatggaacaggaacaggatacaccatggacacagtaaacagaacacaccaatactcagaaaagggaaagtggacgacaaacacagagactggtgcaccccagctcaacccgattgatggaccactacctgaggataatgaaccaagtgggtatgcacaaacagactgtgttctagaggctatggctttccttgaagaatcccacccaggaatatttgagaattcatgccttgaaacaatggaagttgttcaacaaacaagggtagataaactaactcaaggtcgccagacttatgattggacattaaacagaaatcaaccggcagcaactgcattggccaacaccatagaagtctttagatcgaatggcctaacagctaatgagtcaggaaggctaatagatttcttaaaggatgtaatggaatcaatgaacaaagaggaaatagaaataacaacccactttcaaagaaaaaggagagtaagagacaacatgaccaagaagatggtcacgcaaagaacaatagggaagaaaaaacaaagactgaataagagaggctatctaataagagcactgacattaaatacgatgaccaaagatgcagagagaggcaagttaaaaagaagggctatcgcaacacctgggatgcagattagaggtttcgtatactttgttgaaactttagctaggagcatttgcgaaaagcttgaacagtctgggctcccagtagggggcaatgaaaagaaggccaaactggcaaatgttgtgagaaagatgatgactaattcacaagacacagagatttctttcacaatcactggggacaacactaagtggaatgaaaatcaaaatcctcgaatgttcctggcgatgattacatatatcaccagaaatcaacccgagtggttcagaaacatcctgagcatggcacccataatgttctcaaacaaaatggcaagactagggaaagggtacatgttcgagagtaaaagaatgaagattcgaacacaaataccagcagaaatgctagcaagcattgacctgaagtacttcaatgaatcaacaaagaagaaaattgagaaaataaggcctcttctaatagacggcacagcatcactgagtcctgggatgatgatgggcatgttcaacatgctaagtacggtcttgggagtctcgatactgaatcttggacaaaagaaatacaccaagacaatatactggtgggatgggctccaatcatccgacgattttgctctcatagtgaatgcaccaaaccatgagggaatacaagcaggagtggacagattctacaggacctgcaagttagtgggaatcaacatgagcaaaaagaagtcctatataaataagacagggacatttgaattcacaagctttttttatcgctatggatttgtggctaattttagcatggagctacccagctttggagtgtctggagtaaatgaatcagctgacatgagtattggagtaacagtgataaagaacaacatgataaacaatgaccttggacctgcaacggcccagatggctcttcaattgttcatcaaagactacagatacacatataggtgccataggggagacacacaaattcagacgagaagatcatttgagttaaagaagctgtgggatcaaacccaatcaaaggtagggctattagtatcagatggaggaccaaacttatacaatatacggaatcttcacattcctgaagtctgcttaaaatgggagctaatggatgatgattatcggggaagactttgtaatcccctgaatccctttgtcagtcataaagagattgattctgtaaacaatgctgtggtaatgccagcccatggtccagccaaaagcatggaatatgatgccgttgcaactacacattcctggattcccaagaggaatcgttctattctcaacacaagccaaaggggaattcttgaggatgaacagatgtaccagaagtgctgcaatctattcgagaaatttttccctagcagttcatataggagaccggttggaatttctagcatggtggaggccatggtgtctagggcccggattgatgccagggtcgacttcgagtctggacggatcaagaaagaagagttctctgagatcatgaagatctgttccaccattgaagaactcagacggcaaaaataa

>H1N1_PB1_CY060556_A/Ontario/26184/2009

atggatgtcaatccgactctacttttcctaaaaattccagcgcaaaatgccataagcaccacattcccttatactggagatcctccatacagccatggaacaggaacaggatacaccatggacacagtaaacagaacacaccaatactcagaaaagggaaagtggacgacaaacacagagactggtgcaccccagctcaacccgattgatggaccactacctgaggataatgaaccaagtgggtatgcacaaacagactgtgttctagaggctatggctttccttgaagaatcccacccaggaatatttgagaattcatgccttgaaacaatggaagttgttcaacaaacaagggtagataaactaactcaaggtcgccagacttatgattggacattaaacagaaatcaaccggcagcaactgcattggccaacaccatagaagtctttagatcgaatggcctaacagctaatgagtcaggaaggctaatagatttcttaaaggatgtaatggaatcaatgaacaaagaggaaatagagataacaacccactttcaaagaaaaaggagagtaagagacaacatgaccaagaagatggtcacgcaaagaacaatagggaagaaaaaacaaagactgaataagagaggctatctaataagagcactgacattaaatacgatgaccaaagatgcagagagaggcaagttaaaaagaagggctatcgcaacacctgggatgcagattagaggtttcgtatactttgttgaaactttagctaggagcatttgcgaaaagcttgaacagtctgggctcccagtagggggcaatgaaaagaaggccaaactggcaaatgttgtgagaaagatgatgactaattcacaagacacagagatttctttcacaatcactggggacaacactaagtggaatgaaaatcaaaatcctcgaatgttcctggcgatgattacatatatcaccagaaatcaacccgagtggttcagaaacatcctgagcatggcacccataatgttctcaaacaaaatggcaagactagggaaagggtacatgttcgagagtaaaagaatgaagattcgaacacaaataccagcagaaatgctagcaagcattgacctgaagtacttcaatgaatcaacaaagaagaaaattgagaaaataaggcctcttctaatagatggcacagcatcactgagtcctgggatgatgatgggcatgttcaacatgctaagtacggtcttgggagtctcgatactgaatcttggacaaaagaaatacaccaagacaatatactggtgggatgggctccaatcatccgacgattttgctctcatagtgaatgcaccaaaccatgagggaatacaagcaggagtggacagattctacaggacctgcaagttagtgggaatcaacatgagcaaaaagaagtcctatataaataagacagggacatttgaattcacaagctttttttatcgctatggatttgtggctaattttagcatggagctacccagctttggagtgtctggagtaaatgaatcagctgacatgagtattggagtaacagtgataaagaacaacatgataaacaatgaccttggacctgcaacggcccagatggctcttcaattgttcatcaaagactacagatacacatataggtgccataggggagacacacaaattcagacgagaagatcatttgagttaaagaagctgtgggatcaaacccaatcaaagggagggctattagtatcagatggaggaccaaacttatacaatatacggaatcttcacattcctgaagtctgcttaaaatgggagctaatggatgatgattatcggggaagactttgtaatcccctgaatccctttgtcagtcataaagagattgattctgtaaacaatgctgtggtaatgccagcccatggtccagtcaaaagcatggaatatgatgccgttgcaactacacattcctggattcccaagaggaatcgttccattctcaacacaagccaaaggggaattcttgaggatgaacagatgtaccagaagtgctgcaatctattcgagaaatttttccctagcagttcatataggagaccggttggaatttctagcatggtggaggccatggtgtctagggcccggattgatgccagggtcgacttcgagtctggacggatcaagaaagaagagttctctgagatcatgaagatctgctccaccattgaagaactcagacggcaaaaataa

>H1N1_PB1_CY060580_A/Ontario/304434/2009

atggatgtcaatccgactctacttttcctaaaaattccagcgcaaaatgccataagcaccacattcccttatactggagatcctccatacagccatggaacaggaacaggatacaccatggacacagtaaacagaacacaccaatactcagaaaagggaaagtggacgacaaacacagagactggtgcaccccagctcaacccgattgatggaccactacctgaggataatgaaccaagtgggtatgcacaaacagactgtgttctagaggctatggctttccttgaagaatcccacccaggaatatttgagaattcatgccttgaaacaatggaagttgttcaacaaacaagggtagataaactaactcaaggtcgccagacttatgattggacattaaacagaaatcaaccggcagcaactgcattggccaacaccatagaagtctttagatcgaatggcctaacagctaatgagtcaggaaggctaatagatttcttaaaggatgtaatggaatcaatgaacaaagaggaaatagagataacaacccactttcaaagaaaaaggagagtaagagacaacatgaccaagaagatggtcacgcaaagaacaatagggaagaaaaaacaaagactgaataagagaggctatctaataagagcactgacattaaatacgatgaccaaagatgcagagagaggcaagttaaaaagaagggctatcgcaacacctgggatgcagattagaggtttcgtatactttgttgaaactttagctaggagcatttgcgaaaagcttgaacagtctgggctcccagtagggggcaatgaaaagaaggccaaactggcaaatgttgtgagaaagatgatgactaattcacaagacacagagatttctttcacaatcactggggacaacactaagtggaatgaaaatcaaaatcctcgaatgttcctggcgatgattacatatatcaccagaaatcaacccgagtggttcagaaacatcctgagcatggcacccataatgttctcaaacaaaatggcaagactagggaaagggtacatgttcgagagtaaaagaatgaagattcgaacacaaataccagcagaaatgctagcaagcattgacctgaagtacttcaatgaatcaacaaagaagaaaattgagaaaataaggcctcttctaatagatggcacagcatcactgagtcctgggatgatgatgggcatgttcaacatgctaagtacggtcttgggagtctcgatactgaatcttggacaaaagaaatacaccaagacaatatactggtgggatgggctccaatcatccgacgattttgctctcatagtgaatgcaccaaaccatgagggaatacaagcaggagtggacagattctacaggacctgcaagttagtgggaatcaacatgagcaaaaagaagtcctatataaataagacagggacgtttgaattcacaagctttttttatcgctatggatttgtggctaattttagcatggagctacccagctttggagtgtctggagtaaatgaatcagctgacatgagtattggagtaacagtgataaagaacaacatgataaacaatgaccttggacctgcaacggcccagatggctcttcaattgttcatcaaagactacagatacacatataggtgccataggggagacacacaaattcagacgagaagatcatttgagttaaagaagctgtgggatcaaacccaatcaaaggtagggctattagtatcagatggaggaccaaacttatacaatatacggaatcttcacattcctgaagtctgcttaaaatgggagctaatggatgatgattatcggggaagactttgtaatcccctgaatccctttgtcagtcataaagagattgattctgtaaacaatgctgtggtaatgccagcccatggtccagccaaaagcatggaatatgatgccgttgcaactacacattcctggattcccaagaggaatcgttctattctcaacacaagccaaaggggaattcttgaggatgaacagatgtaccagaagtgctgcaatctattcgagaaatttttccctagcagttcatataggagaccggttggaatttctagcatggtggaggccatggtgtctagggcccggattgatgccagggtcgacttcgagtctggacggatcaagaaagaagagttctctgagatcatgaagatctgttccaccattgaagaactcagacggcaaaaataa

>H1N1_PB1_CY060921_A/Texas/JMS370/2009

atggatgtcaatccgactctacttttcctaaaaattccagcgcaaaatgccataagcaccacattcccttatactggagatcctccatacagccatggaacaggaacaggatacaccatggacacagtaaacagaacacaccaatactcagaaaagggaaagtggacgacaaacacagagactggtgcaccccagctcaacccgattgatggaccactacctgaggataatgaaccaagtgggtatgcacaaacagactgcgttctagaggctatggctttccttgaagaatcccacccaggaatatttgagaattcatgccttgaaacaatggaagttgttcaacaaacaagggtagataaactaactcaaggtcgccagacttatgattggacattaaacagaaatcaaccggcagcaactgcattggccaacaccatagaagtctttagatcgaatggcctaacagctaatgagtcaggaaggctaatagatttcttaaaggatgtaatggaatcaatgaacaaagaggaaatagagataacaacccactttcaaagaaaaaggagagtaagagacaacatgaccaagaagatggtcacgcaaagaacaatagggaagaaaaaacaaagactgaataagagaggctatctaataagagcactgacattaaatacgatgaccaaagatgcagagagaggcaagttaaaaagaagggctatcgcaacacctgggatgcagattagaggtttcgtatactttgttgaaactttagctaggagcatttgcgaaaagcttgaacagtctgggctcccagtagggggcaatgaaaagaaggccaaactggcaaatgttgtgagaaagatgatgactaattcacaagacacagagatttctttcacaatcactggggacaacactaagtggaatgaaaatcaaaatcctcgaatgttcctggcgatgattacatatatcaccagaaatcaacccgagtggttcagaaacatcctgagcatggcacccataatgttctcaaacaaaatggcaagactagggaaagggtacatgttcgagagtaaaagaatgaagattcgaacacaaataccagcagaaatgctagcaagcattgacctgaagtacttcaatgaatcaacaaagaagaaaattgagaaaataaggcctcttctaatagatggcacagcatcactgagtcctgggatgatgatgggcatgttcaacatgctaagtacggtcttgggagtctcgatactgaatcttggacaaaagaaatacaccaagacaatatactggtgggatgggctccaatcatccgacgattttgctctcatagtgaatgcaccaaaccatgagggaatacaagcaggagtggacagattctacaggacctgcaagttagtgggaatcaacatgagcaaaaagaagtcctatataaataagacagggacatttgaattcacaagctttttttatcgctatggatttgtggctaattttagcatggagctacccagctttggagtgtctggagtaaatgaatcagctgacatgagtattggagtaacagtgataaagaacaacatgataaacaatgaccttggacctgcaacggcccagatggctcttcaattgttcatcaaagactacagatacacatataggtgccataggggagacacacaaattcagacgagaagatcatttgagttaaagaagctgtgggatcaaacccaatcaaaggtagggctattagtatcagatggaggaccaaacttatacaatataaggaatcttcacattcctgaagtctgcttaaaatgggagctaatggatgatgattatcggggaagactttgtaatcccctgaatccctttgtcagtcataaagagattgattctgtaaacaatgctgtggtaatgccagcccatggtccagccaaaagcatggaatatgatgccgttgcaactacacattcctggattcccaagaggaatcgttctattctcaacacaagccaaaggggaattcttgaggatgaacagatgtaccagaagtgctgcaatctattcgagaaatttttccctagcagttcatataggagaccggttggaatttctagcatggtggaggccatggtgtctagggcccggattgatgccagggtcgacttcgagtctggacggatcaagaaagaagagttctctgagatcatgaagatctgttccaccattgaagaactcagacggcaaaaataa

>H1N1_PB1_CY060961_A/Texas/JMS380/2009

atggatgtcaatccgactctacttttcctaaaaattccagcgcaaaatgccataagcaccacattcccttatactggagatcctccatacagccatggaacaggaacaggatacaccatggacacagtaaacagaacacaccaatactcagaaaagggaaagtggacgacaaacacagagactggtgcaccccagctcaacccgattgatggaccactacctgaggataatgaaccaagtgggtatgcacaaacagactgtgttctagaggctatggctttccttgaagaatcccacccaggaatatttgagaattcatgccttgaaacaatggaagttgttcaacaaacaagggtagataaactaactcaaggtcgccagacttatgattggacattaaacagaaatcaaccggcagcaactgcattggccaacaccatagaagtctttagatcaaatggcctaacagctaatgagtcaggaaggctaatagatttcttaaaggatgtaatggaatcaatgaacaaagaggaaatagagataacaacccactttcaaagaaaaaggagagtaagagacaacatgaccaagaagatggtcacgcaaagaacaatagggaagaaaaaacaaagactgaataagagaggctatctaataagagcactgacattaaatacgatgaccaaagatgcagagagaggcaagttaaaaagaagggctatcgcaacacctgggatgcagattagaggtttcgtatactttgttgaaactttagctaggagcatttgcgaaaagcttgaacagtctgggctcccagtagggggcaatgaaaagaaggccaaactggcaaatgttgtgagaaagatgatgactaattcacaagacacagagatttctttcacaatcactggggacaacactaagtggaatgaaaatcaaaatcctcgaatgttcctggcgatgattacatatatcaccagaaatcaacccgagtggttcagaaacatcctgagcatggcacccataatgttctcaaacaaaatggcaagactagggaaagggtacatgttcgagagtaaaagaatgaagattcgaacacaaataccagcagaaatgctagcaagcattgacctgaagtacttcaatgaatcaacaaagaagaaaattgagaaaataaggcctcttctaatagatggcacagcatcactgagtcctgggatgatgatgggcatgttcaacatgctaagtacggtcttgggagtttcgatactgaatcttggacaaaagaaatacaccaagacaatatactggtgggatgggctccaatcatctgacgattttgctctcatagtgaatgcaccaaaccatgagggaatacaagcaggagtggacagattctacaggacctgcaagttagtgggaatcaacatgagcaaaaagaagtcctatataaataagacagggacatttgaattcacaagctttttttatcgctatggatttgtggctaattttagcatggagctacccagctttggagtgtctggagtaaatgaatcagctgacatgagtattggagtaacagtgataaagaacaacatgataaacaatgaccttggacctgcaacggcccagatggctcttcaattgttcatcaaagactacagatacacatataggtgccataggggagacacacaaattcagacgagaagatcatttgagttaaagaagctgtgggatcaaacccaatcaaaggtagggctattagtatcagatggaggaccaaacttatacaatatacggaatcttcacattcctgaagtctgcctaaaatgggagctaatggatgatgattatcggggaagactttgtaatcccctgaatccctttgtcagtcataaagagattgattctgtaaacaatgctgtggtaatgccagcccatggtccagccaaaagcatggaatatgatgccgttgcaactacacattcctggattcccaagaggaatcgttctattctcaacacaagccaaaggggaattcttgaggatgaacagatgtaccagaagtgctgcaatctattcgagaaatttttccctagcagttcatataggagaccggttggaatttctagcatggtggaggccatggtgtctagggcccggattgatgccagggtcgacttcgagtctggacggatcaagaaagaagagttctctgagatcatgaagatctgttccaccattgaagaactcagacggcaaaaataa

>H1N1_PB1_CY062657_A/San Diego/INS74/2009

atggatgtcaatccgactctacttttcctaaaaattccagcgcaaaatgccataagcaccacattcccttatactggagatcctccatacagccatggaacaggaacaggatacaccatggacacagtaaacagaacacaccaatactcagaaaagggaaagtggacaacaaacacagagactggtgcaccccagctcaacccgattgatggaccactacctgaggataacgaaccaagtgggtatgcacaaacagactgtgttctagaggctatggctttccttgaaraatcccacccaggaatatttgagaattcatgccttgaaacaatggaagttgttcaacaaacaagggtagataaactaactcaaggtcgccagacttatgattggacattaaacagaaatcaaccggcagcaactgcattggccaacaccatagaagtctttagatcgaatggcctaacagctaatgagtcaggaaggctaatagatttcttaaaggatgtaatggaatcaatgaacaaagaggaaatagagataacaacccactttcaaagaaaaaggagagtaagagacaacatgaccaagaagatggtcacgcaaagaacaatagggaagaaaaaacaaagactgaataagagaggctatctaataagagcactgacattaaatacgatgaccaaagatgcagagagaggcaagttaaaaagaagggctatcgcaacacctgggatgcagattagaggtttcgtatactttgttgaaactttagctaggagcatttgcgaaaagcttgaacagtctgggctcccagtagggggcaatgaaaagaaggccaaactggcaaatgttgtgagaaagatgatgactaattcacaagacacagagatttctttcacaatcactggggacaacactaagtggaatgaaaatcaaaatcctcgaatgttcctggcgatgattacatatatcaccagaaatcaacccgagtggttcagaaacatcctgagcatggcacccataatgttctcaaacaaaatggcaagactagggaaagggtacatgttcgagagtaaaagaatgaagattcgaacacaaataccagcagaaatgctagcaagcattgacctgaagtacttcaatgaatcaacaaagaagaaaattgagaaaataaggcctcttctaatagatggcacagcatcactgagtcctgggatgatgatgggcatgttcaacatgctaagtacggtcttgggagtctcgatactgaatcttggacaaaagaaatacaccaagacaatatactggtgggatgggctccaatcatccgacgattttgctctcatagtgaatgcaccaaaccatgagggaatacaagcaggagtggacagattctacaggacctgcaagttagtgggaatcaacatgagcaaaaagaagtcctatataaataagacagggacatttgaattcacaagctttttttatcgctatggatttgtggctaattttagcatggagctacccagctttggagtgtctggagtaaatgaatcagctgacatgagtattggagtaacagtgataaagaacaacatgataaacaatgaccttggacctgcaacggcccagatggctcttcaaytgttcatcaaagactacagatacacatataggtgccataggggagacacacaaattcagacgagaagatcatttgagttaaagaagctgtgggatcaaacccaatcaaaggtagggctattagtatcagatggaggaccaaacttatacaatatacggaatcttcacattcctgaagtctgcttaaaatgggagctaatggatgatgattatcggggaagactttgtaatcccctgaatccctttgtcagtcataaagagattgattctgtaaacaatgctgtggtaatgccagcccatggtccagccaaaagcatggaatatgatgccgttgcaactacacattcctggattcccaagaggaatcgttctattctcaacacaagccaaaggggaattcttgaggatgaacagatgtaccagaagtgctgcaatctattcgagaaatttttccctagcagttcatataggagaccggttggaatttctagcatggtggaggccatggtgtctagggcccggattgatgccagggtcgacttcgagtctggacggatcgggaaggaagagttctctgagatcatgaagatctgttccaccattgaagaactcagacggcaaaaataa

>H1N1_PB1_CY062817_A/Odense/INS143/2009

atggatgtcaatccgactctacttttcctaaaaattccagcgcaaaatgccataagcaccacattcccctacactggagatcctccatacagccatggaacaggaacaggatacaccatggatacagtaaacagaacacaccaatactcagaaaagggaaagtggacgacaaacacagagactggtgcaccccagctcaacccgattgatggaccactacctgaggataatgaaccaagtgggtatgcacaaacagactgtgttctagaggctatggctttccttgaagaatcccacccaggaatatttgagaattcatgccttgaaacaatagaagttgttcaacaaacaagggtagataaactaactcaaggtcgccagacttatgattggactttaaacagaaaccaaccggcagcaactgcattggccaacaccatagaagtctttagatcgaatggcctaacagctaatgagtcaggaaggctaatagatttcttaaaggatgtaatggaatcaatgaacaaagaggaaatagagataacaacccactttcaaagaaaaaggagagtaagagacaacatgaccaagaagatggtcacgcaaagaacaatagggaagaaaaaacaaagactgaataagagaggctatctaataagagcactgacattaaatacgatgaccaaagatgcagagagaggcaagctaaaaagaagggctatcgcaacacctgggatgcagattagaggtttcgtatactttgttgaaactttagctaggagcatttgcgaaaagcttgaacagtctgggctcccagtagggggcaatgaaaagaaggccaaactggcaaatgttgtgagaaagatgatgactaattcacaagacacagagatttctttcacaatcactggggacaacactaagtggaatgaaaatcaaaatcctcgaatgttcctggcgatgattacatatatcaccagaaatcaacccgagtggttcagaaacatcctgagcatggcacccataatgttctcaaacaaaatggcaagactagggaaagggtacatgttcgagagtaaaagaatgaagattcgaacacaaataccagcagaaatgctagcaagcattgacctgaagtacttcaatgaatcaacaaagaagaaaattgagaaaataaggcctcttctaatagatggcacagcatcactgagtcctgggatgatgatgggcatgttcaacatgctaagtacggtcttgggagtctcgatactgaatcttggacaaaagaaatacaccaagacaatatactggtgggatgggctccaatcatccgacgattttgctctcatagtgaatgcaccaaaccatgagggaatacaagcaggagtggacagattctacaggacctgcaagttagtgggaatcaacatgagcaaaaagaagtcctatataaataagacagggacatttgaattcacaagctttttttatcgctatggatttgtggctaattttagcatggagctacccagctttggagtgtctggagtaaatgaatcagctgacatgagtattggagtaacagtgataaagaacaacatgataaacaatgaccttggacctgcaacggcccagatggctcttcaattgttcatcaaagactacagatacacatataggtgccataggggagacacacaaattcagacgagaagatcatttgagttaaagaagctgtgggatcaaacccaatcaaaggtagggctattagtatcagatggaggaccaaacttatacaatatacggaatcttcacattcctgaagtctgcttaaaatgggagctaatggatgatgattatcggggaagactttgtaatcccctgaatccctttgtcagtcataaagagattgattctgtaaacaatgctgtggtaatgccagcccatggtccagccaaaagcatggaatatgatgccgttgcaactacacattcctggattcccaagaggaatcgttctattctcaacacaagccaaaggggaattcttgaggatgaacagatgtaccagaagtgttgcaatctattcgagaaatttttccctagcagttcatataggagaccggttggaatttctagcatggtggaggccatggtgtctagggcccggattgatgccagggtcgacttcgagtctggacggatcaagaaagaagagttctctgagatcatgaagatctgttccaccattgaagaactcagacggcaaaaataa

>H1N1_PB1_CY063169_A/California/VRDL101/2009

atggatgtcaatccgactctacttttcctaaaaattccagcgcaaaatgccataagcaccacattcccttatactggagatcctccatacagccatggaacaggaacaggatacaccatggacacagtaaacagaacacaccaatactcagaaaagggaaagtggacgacaaacacagagactggtgcaccccagctcaacccgattgatggaccactacctgaggataatgaaccaagtgggtatgcacaaacagactgtgttctagaggctatggctttccttgaagaatcccacccgggaatatttgagaattcatgccttgaaacaatggaagttgttcaacaaacaagggtagataaactaactcaaggtcgccagacttatgattggacattaaacagaaatcaaccggcagcaactgcattggccaacaccatagaagtctttagatcgaatggcctaacagctaatgagtcaggaaggctaatagatttcttaaaggatgtaatggaatcaatgaacaaagaggaaatagagataacaacccactttcaaagaaaaaggagagtaagagacaacatgaccaagaagatggtcacgcaaagaacaatagggaagaaaaaacaaagactgaataagagaggctatctaataagagcactgacattaaatacgatgaccaaagatgcagagagaggcaagttaaaaagaagggctatcgcaacacctgggatgcagattagaggtttcgtatactttgttgaaactttagctaggagcatttgcgaaaagcttgaacagtctgggctcccagtagggggcaatgaaaagaaggccaaactggcaaatgttgtgagaaagatgatgactaattcacaagacacagagatttctttcacaatcactggggacaacactaagtggaatgaaaatcaaaatcctcgaatgttcctggcgatgattacatatatcaccagaaatcaacccgagtggttcagaaacatcctgagcatggcacccataatgttctcaaacaaaatggcaagactagggagagggtacatgttcgagagtaaaagaatgaagattcgaacacaaataccagcagaaatgctagcaagcattgacctgaagtacttcaatgaatcaacaaagaagaaaattgagaaaataaggcctcttctaatagatggcacagcatcactgagtcctggaatgatgatgggcatgttcaacatgctaagtacagtcttgggagtctcgatactgaaccttggacaaaagaaatacaccaagacaatatactggtgggatgggctccaatcatccgacgattttgctctcatagtgaatgcaccaaaccatgaggggatacaagcaggagtggacagattctacaggacctgcaagttagtgggaatcaacatgagcaaaaagaagtcctatataaataagacagggacatttgaattcacaagctttttttatcgctatggatttgtggctaattttagcatggagctacccagctttggagtgtctggagtaaatgaatcagctgacatgagtattggagtaacagtgataaagaacaacatgataaacaatgatcttggacctgcaacggcccagatggctcttcaattgttcatcaaagactacagatacacatacaggtgccataggggagacacacaaattcagacgagaagatcatttgagttaaagaagctgtgggatcaaacccaatcaaaggtagggctattagtatcagatggaggaccaaacttatacaatatacgaaatcttcatattcctgaagtctgcttaaaatgggagctaatggatgatgattatcggggaagactttgtaatcccctgaatccctttgtcagtcataaagagattgattctgtaaacaatgctgtggtaatgccagcccatggtccagccaaaagcatggaatatgatgccgttgcaactacacattcctggattcccaagaggaatcgttctattctcaacacaagccaaaggggaattcttgaggatgaacagatgtaccagaagtgctgcaatctattcgagaaatttttccctagcagttcatataggagaccggttggaatttctagcatggtggaggccatggtgtctagggcccggattgatgccagggtcgacttcgagtctggacggatcaagaaagaagagttctctgagatcatgaagatctgttccaccattgaagaactcagacggcaaaaataa

>H1N1_PB1_CY067077_A/Athens/INS259/2009

atggatgtcaatccgactctacttttcctaaaaattccagcgcaaaatgccataagcaccacattcccttatactggagatcctccatacagccatggaacaggaacaggatacaccatggacacagtaaacagaacacaccaatactcagaaaagggaaagtggacgacaaacacagagactggtgcaccccagctcaacccgattgatggaccactacctgaggataatgaaccaagtgggtatgcacaaacagactgtgttctagaggctatggctttccttgaagaatcccacccaggaatatttgagaattcatgccttgaaacaatggaagttgttcaacaaacaagggtagataaactaactcaaggtcgccagacttatgattggacattaaacagaaatcaaccggcagcaactgcattggccaacaccatagaagtctttagatcgaatggcctaacagctaatgagtcaggaaggctaatagatttcttaaaggatgtaatggaatcaatgaacaaagaggaaatagagataacaacccactttcaaagaaaaaggagagtaagagacaacatgaccaagaagatggtcacgcaaagaacaatagggaagaaaaaacaaagactgaataagagaggctatctaataagagcactgacattaaatacgatgaccaaagatgcagagagaggcaagttaaaaagaagggctatcgcaacacctgggatgcagattagaggtttcgtatactttgttgaaactttagctaggagcatttgcgaaaagcttgaacagtctgggctcccagtagggggcaatgaaaagaaggccaaactggcaaatgttgtgagaaagatgatgactaattcacaagacacagagatttctttcacaatcactggggacaacactaagtggaatgaaaatcaaaatcctcgaatgttcctggcgatgattacatatatcaccagaaatcaacccgagtggttcagaaacatcctgagcatggcacccataatgttctcaaacaaaatggcaagactagggaaagggtacatgttcgagagtaaaagaatgaagattcgaacacaaataccagcagaaatgctagcaagcattgacctgaagtacttcaatgaatcaacaaagaagaaaattgagaaaataaggcctcttctaatagatggcacagcatcactgagtcctggaatgatgatgggcatgttcaacatgctaagtacggtattgggagtctcgatactgaatcttggacaaaagaaatacaccaagacaatatactggtgggatgggctccaatcatccgacgattttgctctcatagtgaatgcaccaaaccatgagggaatacaagcaggagtggacagattctacaggacctgcaagttagtgggaatcaacatgagcaaaaagaagtcctatataaataagacagggacatttgaattcacaagctttttttatcgctatggatttgtggctaattttagcatggagctacccagctttggagtgtctggagtaaatgaatcagctgacatgagtattggagtaacagtgataaagaacaacatgataaacaatgaccttggacctgcaacggcccagatggctcttcaattgttcatcaaagactacagatacacatataggtgccataggggagacacacaaattcagacgagaagatcatttgagttaaagaaactgtgggatcaaacccaatcaaaggtagggctattagtatcagatggaggaccaaacttatacaatatacggaatcttcacattcctgaagtctgcttaaaatgggagctaatggatgatgattatcggggaagactttgtaatcccctgaatccctttgtcagtcataaagagattgattctgtaaacaatgctgtggtaatgccagcccatggtccagccaaaagcatggaatatgatgctgttgcaactacacattcctggattcccaagaggaatcgttctattctcaacacaagccaaaggggaattcttgaggatgaacagatgtaccagaagtgctgcaatctattcgagaaatttttccctagcagttcatataggagaccggttggaatttctagcatggtggaggccatggtgtctagggcccggattgatgccagggtcgacttcgagtctggacggatcaagaaagaagagttctctgagatcatgaagatctgttccaccattgaagaactcagacggcaaaaataa

>H1N1_PB1_HM849035_A/Kurume/K0910/2009

atggatgtcaatccgactctacttttcctaaaaattccagcgcaaaatgccataagcaccacattcccttatactggagatcctccatacagccatggaacaggaacaggatacaccatggacacagtaaacagaacacaccaatactcagaaaagggaaagtggacgacaaacacagagactggtgcaccccagctcaacccgattgatggaccactacctgaggataatgaaccaagtgggtatgcacaaacagactgtgttctagaggctatggctttccttgaagaatcccacccaggaatatttgagaattcatgccttgaaacaatggaagttgttcaacaaacaagggtagataaactaactcaaggtcgccagacttatgattggacattaaacagaaatcaaccggcagcaactgcattggccaacaccatagaagtctttagatcgaatggcctaacagctaatgagtcaggaaggctaatagatttcttaaaggatgtaatggaatcaatgaacaaagaggaaatagagataacaacccactttcaaagaaaaaggagagtaagagacaacatgaccaagaagatggtcacgcaaagaacaatagggaagaaaaaacaaagactgaataagagaggctatctaataagagcactgacattaaatacgatgaccaaagatgcagagagaggcaagttaaaaagaagggctatcgcaacacctgggatgcagattagaggtttcgtatactttgttgaaactttagctaggagcatttgcgaaaagcttgaacagtctgggctcccagtagggggcaatgaaaagaaggccaaactggcaaatgttgtgagaaagatgatgactaattcacaagacacagagatttctttcacaatcactggggacaacactaagtggaatgaaaatcaaaatcctcgaatgttcctggcgatgattacatatatcaccagaaatcaacccgagtggttcagaaacatcctgagcatggcacccataatgttctcaaacaaaatggcaagactagggaaagggtacatgttcgagagtaaaagaatgaagattcgaacacaaataccagcagaaatgctagcaagcattgacctgaagtacttcaatgaatcaacaaagaagaaaattgagaaaataaggcctcttctaatagatggcacagcatcactgagtcctgggatgatgatgggcatgttcaacatgctaagtacggtcttgggagtctcgatactgaatcttggacaaaagaaatacaccaaaacagtatactggtgggatgggctccaatcatccgacgattttgctctcatagtgaatgcaccaaaccatgagggaatacaagcaggagtggacagattctacaggacctgcaagttagtgggaatcaacatgagcaaaaagaagtcctatataaataagacagggacatttgaattcacaagctttttttatcgctatggatttgtggctaattttagcatggagctacccagctttggagtgtctggagtaaatgaatcagctgacatgagtattggagtaacagtgataaagaacaacatgataaacaatgaccttggacctgcaacggcccagatggctcttcaattgttcatcaaagactacagatacacatataggtgccataggggagacacacaaattcagacgagaagatcatttgagttaaagaagctgtgggatcaaacccaatcaaaggtagggctattagtatcagatggaggaccaaacttatacaatatacggaatcttcacattcctgaagtctgcttaaaatgggagctaatggatgatgattatcggggaagactttgtaatcccctgaatccctttgtcagtcataaagagattgattctgtaaacaatgctgtggtaatgccagcccatggtccagccaaaagcatggaatatgatgccgttgcaactacacattcctggattcccaagaggaatcgttctattctcaacacaagccaaaggggaattcttgaggatgaacagatgtaccagaagtgctgcaatctattcgagaaatttttccctagcagttcatataggagaccggttggaatttctagcatggtggaggccatggtgtctagggcccggattgatgccagggtcgacttcgagtctggacggatcaagaaagaagagttctctgagatcatgaagatctgttccaccattgaagaactcagacggcaaaaataa

>H1N1_PB1_HQ011409_A/Guangdong/01/2009

atggatgtcaatccgactctacttttcctaaaaattccagcgcaaaatgccataagcaccacattcccttatactggagatcctccatacagccatggaacaggaacaggatacaccatggacacagtaaacagaacacaccaatactcagaaaagggaaagtggacgacaaacacagagactggtgcaccccagctcaacccgattgatggaccactacctgaggataatgaaccaagtgggtatgcacaaacagactgtgttctagaggctatggctttccttgaagaatcccacccaggaatatttgagaattcatgccttgaaacaatggaagttgttcaacaaacaagggtagataaactaactcaaggtcgccagacttatgattggacattaaacagaaatcaaccggcagcaactgcattggccaacaccatagaagtctttagatcgaatggcctaacagctaatgagtcaggaaggctaatagatttcttaaaggatgtaatggaatcaatgaacaaagaggaaatagagataacaacccactttcaaagaaaaaggagagtaagagacaacatgaccaagaagatggtcacgcaaagaacaatagggaagaaaaaacaaagactgaataagagaggctatctaataagagcactgacattaaatacgatgaccaaagatgcagagagaggcaagttaaaaagaagggctatcgcaacacctgggatgcagattagaggtttcgtatactttgttgaaactttagctaggagcatttgcgaaaagcttgaacagtctgggctcccagtagggggcaatgaaaagaaggccaaactggcaaatgttgtgagaaagatgatgactaattcacaagacacagagatttctttcacaatcactggggacaacactaagtggaatgaaaatcaaaatcctcgaatgttcctggcgatgattacatatatcaccagaaatcaacccgagtggttcagaaacatcctgagcatggcacccataatgttctcaaacaaaatggcaagactagggaaagggtacatgttcgagagtaaaagaatgaaaattcgaacacaaataccagcagaaatgctagcaagcattgacctgaagtacttcaatgaatcaacaaagaagaaaattgagaaaataaggcctcttctaatagatggcacagcatcactgagtcctgggatgatgatgggcatgttcaacatgctaagtacggtcttgggagtctcgatactgaatcttggacaaaagaaatacaccaagacaatatactggtgggatgggctccaatcatccgacgattttgctctcatagtgaatgcaccaaaccatgagggaatacaagcaggagtggacagattctacaggacctgcaagttagtgggaatcaacatgagcaaaaagaagtcctatataaataagacagggacatttgaattcacaagctttttttatcgctatggatttgtggctaattttagcatggagctacccagctttggagtgtctggagtaaatgaatcagctgacatgagtattggagtaacagtgataaagaacaacatgataaacaatgaccttggacctgcaacggcccagatggctcttcaattgttcatcaaagactacagatacacatataggtgccataggggagacacacaaattcagacgagaagatcatttgagttaaagaagctgtgggatcaaacccaatcaaaggtagggctattagtatcagatggaggaccaaacttatacaatatacggaatcttcacattcctgaagtctgcttaaaatgggagctaatggatgatgattatcggggaagactttgtaatcccctgaatccctttgtcagtcataaagagattgattctgtaaacaatgctgtggtaatgccagcccatggtccagccaaaagcatggaatatgatgccgttgcaactacacattcctggattcccaagaggaatcgttctattctcaacacaagccaaaggggaattcttgaggatgaacagatgtaccagaagtgctgcaatctattcgagaaatttttccctagcagttcatataggagaccggttggaatttctagcatggtggaggccatggtgtctagggcccggattgatgccagggtcgacttcgagtctggacggatcaagaaagaagagttctctgagatcatgaagatctgttccaccattgaagaactcagacggcaaaaataa

>H1N1_PB1_CY071213_A/Frankfurt/INS402/2010

atggatgtcaatccgactctacttttcctaaaaattccagcgcaaaatgccataagcaccacattcccttatactggagatcctccatacagccatggaacaggaacaggatacaccatggacacagtaaacagaacacaccaatactcagaaaagggaaagtggacgacaaacacagagactggtgcacctcagctcaacccgattgatggaccactacctgaggataatgaaccaagtgggtatgcacaaacagactgtgttctagaggctatggctttccttgaagaatcccacccaggaatatttgagaattcatgccttgaaacaatggaagttgttcaacaaacaagggtagataaactaactcaaggtcgccagacttatgattggacattaaacagaaatcaaccggcagcaactgcattggccaacaccatagaagtctttagatcgaatggcctaacagctaatgagtcaggaaggctaatagatttcttaaaggatgtaatggaatcaatgaacaaagaggaaatagagataacaacccactttcaaagaaaaaggagagtaagagacaacatgaccaagaagatggtcacgcaaagaacaatagggaagaaaaaacaaagactgaataagagaggctatctaataagagcactgacattaaatacgatgaccaaagatgcagagagaggcaagttaaaaagaagggctatcgcaacacctgggatgcagattagaggtttcgtatactttgttgaaactttagctaggagcatttgcgaaaagcttgaacagtctgggctcccagtagggggcaatgaaaagaaggccaaactggcaaatgttgtgagaaagatgatgactaattcacaagacacagagatttctttcacaatcactggggacaacactaagtggaatgaaaatcaaaatcctcgaatgttcctggcgatgattacatatatcaccagaaatcaacccgagtggttcagaaacatcctgagcatggcacccataatgttctcaaacaaaatggcaagactagggaaagggtacatgttcgagagtaaaagaatgaagattcgaacacaaataccagcagaaatgctagcaagcattgacctgaagtacttcaatgaatcaacaaagaagaaaattgagaaaataaggcctcttctaatagatggcacagcatcactgagtcctgggatgatgatgggcatgttcaacatgctaagtacggtcttgggagtctcgatactgaatcttggacaaaagaaatacaccaagacaatatactggtgggatgggctccaatcatccgacgattttgctctcatagtgaatgcaccaaaccatgagggaatacaagcaggagtggacagattctacaggacctgcaagttagtgggaatcaacatgagcaaaaagaagtcctatataaataagacagggacatttgaattcacaagctttttttatcgctatgggtttgtggctaattttagcatggagctacccagctttggagtgtctggagtaaatgaatcagctgacatgagtattggagtaacagtgataaagaacaacatgataaacaatgaccttggacctgcaacggcccagatggctcttcaattgttcatcaaagactacagatacacatataggtgccataagggagacacacaaattcagacgagaagatcatttgagttaaagaagctgtgggatcaaacccaatcaaaggtaggactattagtatcagatggaggaccaaacttatacaatatacggaatcttcacattcctgaagtctgcttaaaatgggagctaatggatgatgattatcggggaagactttgtaatcccctgaatccctttgtcagtcataaagagattgattctgtaaacaatgctgtggtaatgccagcccatggtccagccaaaagcatggaatatgatgccgttgcaactacacattcctggattcccaagaggaaccgttctattctcaacacaagccaaaggggaattcttgaggatgaacagatgtaccagaagtgctgcaatctattcgagaaatttttccctagcagttcatataggagaccggttggaatttctagcatggtggaggccatggtgtctagggcccggattgatgccagggtcgacttcgagtctggacggatcaagaaagaagagttctctgagatcatgaagatctgttccaccattgaagaactcagacggcaaaaataa

>H1N1_PB1_CY072612_A/Managua/2155.01/2009

atggatgtcaatccgactctacttttcctaaaaattccagcgcaaaatgccataagcaccacattcccttatactggagatcctccatacagccatggaacaggaacaggatacaccatggacacagtaaacagaacacaccaatactcagaaaagggaaagtggacgacaaacacagagactggtgcaccccagctcaacccgattgatggaccactacctgaggataatgaaccaagtgggtatgcacaaacagactgtgttctagaggctatggctttccttgaagaatcccacccaggaatatttgagaattcatgccttgaaacaatggaagttgttcaacaaacaagagtagataaactaactcaaggtcgccagacttatgattggacattaaacagaaatcaaccggcagcaactgcattggccaacaccatagaagtctttagatcgaatggcctaacagctaatgagtcaggaaggctaatagatttcttaaaggatgtaatggaatcaatgaacaaagaggaaatagagataacaacccactttcaaagaaaaaggagagtaagagacaacatgaccaagaagatggtcacgcaaagaacaatagggaagaaaaaacaaagactgaataagagaggctatctaataagagcactgacattaaatacgatgaccaaagatgcagagagaggcaagttaaaaagaagggctatcgcaacacctgggatgcagattagaggtttcgtatactttgttgaaactttagctaggagcatttgcgaaaagcttgaacagtctgggctcccagtagggggcaatgaaaagaaggccaaactggcaaatgttgtgagaaagatgatgactaattcacaagacacagagatttctttcacaatcactggggacaacactaagtggaatgaaaatcaaaatcctcgaatgttcctggcgatgattacatatatcaccagaaatcaacccgagtggttcagaaacatcctgagcatggcacccataatgttctcaaacaaaatggcaagactagggaaagggtacatgttcgagagtaaaagaatgaagattcgaacacaaataccagcagaaatgctagcaagcattgacctgaagtacttcaatgaatcaacaaagaagaaaattgagaaaataaggcctcttctaatagatggcacagcatcactgagtcctgggatgatgatgggcatgttcaacatgctaagtacggtcttgggagtctcgatactgaatcttggacaaaagaaatacaccaagacaatatactggtgggatgggctccaatcatccgacgattttgctctcatagtgaatgcaccaaaccatgagggaatacaagcaggagtggacagattctacaggacctgcaagttagtgggaatcaacatgagcaaaaagaagtcctatataaataagacagggacatttgaattcacaagctttttttatcgctatggatttgtggctaattttagcatggagctacccagctttggagtgtctggagtaaatgaatcagctgacatgagtattggagtaacagtgataaagaacaacatgataaacaatgaccttggacctgcaacggcccagatggctcttcaattgttcatcaaagactacagatacacatataggtgccataggggagacacacaaattcagacgagaagatcatttgagttaaagaagctgtgggatcaaacccaatcaaaggtagggctattagtatcagatggaggaccaaacttatacaatatacggaatcttcacattcctgaagtctgcttaaaatgggagctaatggatgatgattatcggggaagactttgtaatcccctgaatccctttgtcagtcataaagagattgattctgtaaacaatgctgtggtaatgccagcccatggtccagccaaaagcatggaatatgatgccgttgcaactacacattcctggattcccaagaggaatcgttctattctcaacacaagccaaaggggaattcttgaggatgaacagatgtaccagaagtgctgcaatctattcgagaaatttttccctagcagttcatataggagaccggttggaatttctagcatggtggaggccatggtgtctagggcccggattgatgccagggtcgacttcgagtctggacgggtcaagaaagaagagttctctgagatcatgaagatctgttccaccattgaagaactcagacggcaaaaataa

>H1N1_PB1_CY072708_A/Managua/5036.01/2009

atggatgtcaatccgactctacttttcctaaaaattccagcgcaaaatgccataagcaccacattcccttatactggagatcctccatacagccatggaacaggaacaggatacaccatggacacagtaaacagaacacaccaatactcagaaaagggaaagtggacgacaaacacagagactggtgcaccccagctcaacccgattgatggaccactacctgaggataatgaaccaagtgggtatgcacaaacagactgtgttctagaggctatggctttccttgaagaatcccacccaggaatatttgagaattcatgccttgaaacaatggaagttgttcaacaaacaagagtagataaactaactcaaggtcgccagacttatgattggacattaaacagaaatcaaccggcagcaactgcattggccaacaccatagaagtctttagatcgaatggcctaacagctaatgagtcaggaaggctaatagatttcttaaaggatgtaatggaatcaatgaacaaagaggaaatagagataacaacccactttcaaagaaaaaggagagtaagagacaacatgaccaagaagatggtcacgcaaagaacaatagggaagaaaaaacaaagactgaataagagaggctatctaataagagcactgacattaaatacgatgaccaaagatgcagagagaggcaagttaaaaagaagggctatcgcaacacctgggatgcagattagaggtttcgtatactttgttgaaactttagctaggagcatttgcgaaaagcttgaacagtctgggctcccagtagggggcaatgaaaagaaggccaaactggcaaatgttgtgagaaagatgatgactaattcacaagacacagagatttctttcacaatcactggggacaacactaagtggaatgaaaatcaaaatcctcgaatgttcctggcgatgattacatatatcaccagaaatcaacccgagtggttcagaaacatcctgagcatggcacccataatgttctcaaacaaaatggcaagactagggaaagggtacatgttcgagagtaaaagaatgaagattcgaacacaaataccagcagaaatgctagcaagcattgacctgaagtacttcaatgaatcaacaaagaagaaaattgagaaaataaggcctcttctaatagatggcacagcatcactgagtcctgggatgatgatgggcatgttcaacatgctaagtacggtcttgggagtctcgatactgaatcttggacaaaagaaatacaccaagacaatatactggtgggatgggctccaatcatccgacgattttgctctcatagtgaatgcaccaaaccatgagggaatacaagcaggagtggacagattctacaggacctgcaagttagtgggaatcaacatgagcaaaaagaagtcctatataaataagacagggacatttgaattcacaagctttttttatcgctatggatttgtggctaattttagcatggagctacccagctttggagtgtctggagtaaatgaatcagctgacatgagtattggagtaacagtgataaagaacaacatgataaacaatgaccttggacctgcaacggcccagatggctcttcaattgttcatcaaagactacagatacacatataggtgccataggggagacacacaaattcagacgagaagatcatttgagttaaagaagctgtgggatcaaacccaatcaaaggtagggctattagtatcagatggaggaccaaacttatacaatatacggaatcttcacattcctgaagtctgcttaaaatgggagctaatggatgatgattatcggggaagactttgtaatcccctgaatccctttgtcagtcataaagagattgattctgtaaacaatgctgtggtaatgccagcccatggtccagccaaaagcatggaatatgatgccgttgcaactacacattcctggattcccaagaggaatcgttctattctcaacacaagccaaaggggaattcttgaggatgaacagatgtaccagaagtgctgcaatctattcgagaaatttttccctagcagttcatataggagaccggttggaatttctagcatggtggaggccatggtgtctagggcccggattgatgccagggtcgacttcgagtctggacggatcaagaaagaagagttctctgagatcatgaagatctgttccaccattgaagaactcagacggcaaaaataa

>H1N1_PB1_CY074113_A/California/VRDL192/2009

atggatgtcaatccgacattacttttcctaaaagtgccagcacaaaatgctataagcacaacttttccttatactggtgaccctccttacagccatgggacaggaacagggtacaccatggatacagtcaacaggacacaccagtactcagaaagaggaagatggacaaaaaataccgaaacgggagcaccgcaacttaacccaattgatggtcccttaccggaagacaatgaaccaagtggctatgcccaaacagattgtgtattagaagcaatggctttccttgaagaatcccatcccggtatctttgaaaactcttgtattgaaacaatggaggttgttcaacaaacaagggtggacaaactgacacaaggcagacagacctatgactggactctaaataggaaccagcctgctgccacagcattggcaaacactatagaagtattcagatcaaacggcctcatagcaaatgaatctgggaggctaatagacttccttaaagatgtaatggagtcgatggacagaggcgaagtagaggtcacaactcattttcaaagaaagaggagagtgagagacaatgtaactaaaaaaatggtgacccaaagaacaataggcaaaaagaaacataaattagacaaaagaagttacctaattagggcattaaccctgaacacaatgaccaaagatgctgagagggggaaactaaaacgcagagcaattgcaaccccaggaatgcaaataagggggtttgtatactttgttgagacactggcaagaagcatatgtgaaaagcttgaacaatcaggattgccagttggaggaaatgagaagaaagcaaagttagcaaatgttgtaaggaagatgatgaccaactcccaagacactgaaatttctttcaccataaccggagataacacaaaatggaacgaaaatcaaaaccctagaatgttcttggccatgatcacatatataaccaaaaatcagcctgaatggttcagaaatattctaagtattgctccaataatgttttcaaacaagatggcgagactaggcaaggggtacatgtttgaaagcaagagtatgaaactgagaactcaaatacctgcagagatgctagccaacatagacttgaaatatttcaatgattcaactaaaaagaaaattgaaaaaatccgaccattattaatagatggaactgcatcattgagtcctggaatgatgatgggcatgttcaatatgttgagcaccgtcttgggcgtctccattctgaatcttgggcaaaagagatacaccaagactacttactggtgggatggtcttcaatcgtctgatgattttgctttgattgtgaacgcacccaactatgcaggaattcaagctggagttgacaggttttatcgaacctgtaagctgctcggaattaatatgagcaaaaagaagtcttacataaacagaacaggtacctttgaattcacgagctttttctatcgttatgggtttgttgccaatttcagcatggagcttcctagttttggggtgtctggggtcaatgaatctgcagacatgagtattggagtcactgtcatcaaaaacaatatgataaacaatgaccttggcccagcaactgctcaaatggcccttcagttatttatcaaagattacaggtacacttatcgatgccaccgaggtgacacacaaatacaaacccggagatcatttgagataaagaaactatgggaccaaacccgttcaaaagctggactgttggtctctgatggaggccccaatttgtataacattagaaatctccatattcctgaagtttgcttgaaatgggagttgatggatgaggattaccaggggcggttatgcaacccattaaacccgtttgtcagccataaagagattgaatctgtgaacagtgcagtgataatgccggcacatggtccagccaaaaatatggagtatgacgctgttgcaacaacacactcctgggtccccaaaagaaatcgatccattttgaacacgagccaaagggggatacttgaagatgagcaaatgtatcagaggtgctgcaatttatttgaaaaattcttcccaagtagctcatacagaagaccagttggaatatccagtatggtagaggctatggtctcaagagcccgaattgatgcacggattgatttcgaatctggaaggataaagaaagaggaatttgctgagatcatgaagatctgttccaccattgaagacctcagacggcaaaaatga

>H1N1_PB1_HQ291948_A/Taiwan/10393/2005

atggatgtcaatccgacattacttttcttaaaagtgccagcacaaaatgctataagcacaacttttccttatactggtgaccctccttacagccatgggacaggaacagggtacacaatggatacagtcaacaggacacatcagtactcagaaagaggaagatggacaaagaataccgaaactggagcaccgcaactcaacccaattgatgggccactaccagaagacaatgaaccaagtggctatgcccaaacagattgtgtattagaagcaatggctttccttgaagaatcccatcctggcatttttgaaaactcttgtattgaaacaatggaggttgttcagcaaacaagggtggacaaactgacacaaggcagacagacctatgactggactctaaataggaaccagcctgctgccacagcattggccaacactatagaagtattcagatcaaacggcctcatagcaaatgaatctgggaggctaatagacttccttaaagatgtaatggagtcgatggacagagacgaagtagaggtcacaactcattttcagagaaagaggagagtgagagacaatgtaactaaaaaaatggtgacccaaagaacaataggcaaaaagaaacataaattagacaaaaggagttacctaattagagcattaaccctgaacacaatgaccaaagatgctgagagggggaaactaaaacgcagagcaattgcaaccccaggaatgcaaataagggggtttgtatactttgttgagacactggcaagaagcatatgtgaaaagcttgaacaatcagggttgcccgttggagggaacgagaagaaagcaaagttagcaaatgttgtaaggaagatgatgaccaactcccaggacactgaaatttctttcaccatcactggagataacacaaaatggaacgaaaatcaaaaccctagaatgtttttggccatgatcacatatataaccagaaatcagcctgaatggttcagaaatattctaagtattgctccaataatgttttcaaacaaaatggcgagactaggtaaggggtacatgtttgaaagcaagagtatgaaactgagaactcaaatacctgcagagatactagccgacatagatttgaaatatttcaatgagtcaactaaaaagaaaattgaaaaaatccgaccattattaatagatggaactgcatcattgagtcctggaatgatgatgggcatgttcaatatgttaagcactgtcttgggcgtctccattctgaatcttgggcaaaagagatacaccaagactacttactggtgggatggtcttcaatcgtctgatgattttgctctgatagtgaatgcgcccaactatgcaggaattcaagctggagttgacaggttttatcgaacctgtaagctgctcggaattaatatgagcaaaaagaagtcttacataaataggacaggtacctttgaattcacgagctttttctatcgttatgggtttgttgccaatttcagcatggagcttcctagttttggggtgtctggggtcaatgaatctgcagacatgagtattggagtcactgttatcaaaaacaatatgataaacaatgaccttggcccagcaactgctcaaatggcccttcagttatttataaaagattacagatacacgtatcgatgccacagaggtgacacacaaatacaaacccggagatcatttgagataaagaaattatgggaccaaacccgctccaaagctgggctgttggtctctgatggaggccccaatctatataacattagaaatctccatattcctgaagtctgcttgaaatgggagttgatggatgaggattaccaggggcgtttatgcaacccattgaacccgtttgtcagtcataaagagattgaatcagtgaacaatgcagtgatgatgccggcacatggtccagccaaaaatatggagtatgacgctgttgcaacaacacattcctgggttcccaaaaggaatcgatccattttgaacacgagccaaagggggatacttgaggatgagcaaatgtatcagaggtgctgcaatttatttgaaaaattcttcccaagtagctcatacagaagaccagttgggatatccagtatggtagaggctatgatttccagagcccgaattgatgcacggattgatttcgaatctggaaggataaagaaagaggaattcgctgagatcatgaagacctgttccaccattgaagacctcagacggcaaaaatag

>H1N1_PB1_CY077777_A/Netherlands/002Z1/1956

atggatgtcaatccgaccttacttttcttaaaagtgccagcacaaaatgctataagcacaactttcccttatactggagaccctccttacagccatgggacaggaacagggtacaccatggatactgtcaacaggacacatcagtactcagaaaggggaagatggacaaaaaacaccgaaactggagcaccacaactcaacccaattgatggaccactaccagaagacaacgaaccaagtggttatgcccaaacagattgtgtattagaagcaatggctttccttgaggaatcccatcctggtatttttgaaaactcatgtattgaaacgatggaggttgttcagcaaacacgagtggacaagctgacacaaggccgacagacctatgactggactctgaatagaaaccagcctgctgctacagcattggcaaacacaatagaagtgttcagatcaaatggcctcatggcaaatgaatccggaaggctaatagactttcttaaagatgtaatggagtcaatggacaaagaagaaatagagatcacaacccattttcagagaaagagacgagtgagggacaatgtgactaagaaaatggtgacacaaagaacaataggcaaaaagaaacagagattaaacaaaaggagttatctaattagggcattgaccctgaacacaatgaccaaagatgctgagagagggaaactaaaacgaagagcaattgcaaccccagggatgcaaataagggggtttgtatactttgttgagacactggcaagaagtatatgtgaaaaacttgaacaatcaggactgccagttggaggaaatgaaaagaaagcaaagttggcaaatgttgtaaggaagatgatgaccaattctcaggacactgaaatttctttcaccatcactggagacaacaccaaatggaacgaaaatcagaaccctagaatgtttttggccatgatcacatatataaccagaaatcagcctgaatggttcagaaatattctaagtattgctccaataatgttctcaaacaaaatggcgagactaggtaagggttacatgtttgagagcaagagtatgaaacttagaactcaaatatctgcagaaatgctagcaaacatcgatttgaagtatttcaatgattcaacaagaaagaaaattgaaaaaatccggccgctcttaatagatgggactgcatcactgagccctggaatgatgatgggcatgttcaatatgttaagcactgtattgggcgtttccatcctgaatcttgggcaaaagagatacacaaagactacttactggtgggatggtcttcaatcatctgatgattttgctctgattgtgaatgcacccaatcatgcaggaattcaggctggagttgacaggttttatcgaacttgtaagctgctcggaatcaatatgagtaagaaaaagtcttacataaacagaacaggtacatttgaattcacgagctttttctatcgttatgggtttgttgccaatttcagcatggagcttcccagttttggggtgtctgggatcaacgagtctgcagacatgagtattggggtcactgtcatcaaaaacaatatgataaacaatgatcttggtccagcaaccgctcaaatggcccttcagttattcatcaaagattacaggtacacgtatcggtgccacagaggtgacacacaaatacaaacccgaagatcatttgaaataaagaaactgtgggagcaaacccgttccaaagctgggctgctggtctccgatggaggtcctaacttatataacattagaaatctccacatccctgaagtctgtttgaaatgggaattgatggatgaggattaccaggggcgtttatgcaacccattgaacccatttgtcagtcataaagagattgaatcagtgaacaatgcagtgatgatgccggcacatggtccagccaaaaacatggagtatgatgctgtagcaacaacacactcctgggtccccaaaagaaatcgatccatcttgaatactagtcaaaggggaatacttgaagatgaacaaatgtatcaaaggtgctgcaatttatttgaaaaattcttccccagcagttcatacagaaggccagtcggaatatccagtatggtggaggctatggtttccagagcccgaattgatgcacggattgatttcgaatctggaaggataaagaaagaggatttcactgagatcatgaagatctgttccaccattgaagaactcagacggcaaaaatag

>H1N1_PB1_CY080839_A/Boston/49/2009

atggatgtcaatccgacattacttttcttaaaagtgccagcacaaaatgctataagcacaactttcccttatactggtgaccctccttacagccatgggacaggaacagggtacaccatggatacagtcaacaggacacaccagtactcagaaagaggaagatggacaaaaaataccgaaacgggagcaccgcaacttaacccaattgatggtcccttaccggaagacaatgaaccaagtggctatgcccaaacagattgtgtattagaagcaatggctttccttgaagaatcccatcccggtatctttgaaaactcttgtattgaaacaatggaggttgttcaacaaacaagggtggacaaactgacacaaggcagacagacctatgactggactctaaataggaaccagcctgctgccacagcattggcaaacactatagaagtattcagatcaaacggcctcatagcaaatgaatctgggaggctaatagacttccttaaagatgtaatggagtcgatggacagaggcgaagtagaggtcacaactcattttcaaagaaagaggagagtgagagacaatgtaactaagaaaatggtgacccaaagaacaataggcaaaaagaaacataaattagacaaaagaagttacctaattagggcattaaccctgaacacaatgaccaaagatgctgagagggggaaactaaaacgcagagcaattgcaacccctggaatgcaaataagggggtttgtatactttgttgagacactggcaagaagcatatgtgaaaagcttgaacaatcaggattgccagttggaggaaatgagaagaaagcaaagttagcaaatgttgtaaggaagatgatgaccaactcccaggacactgaaatttctttcaccataaccggagataacacaaaatggaacgaaaatcaaaaccctagaatgttcttggccatgatcacatatataaccaaaaatcagcctgaatggttcagaaatattctaagtattgctccaataatgttttcaaacaagatggcgagactaggtaaggggtacatgtttgaaagcaagagtatgaaactgagaactcaaatacctgcagaaatgctagccaacatagacttgaaatatttcaatgattcaactaaaaagaaaattgaaaaaatccgaccattattaatagatggaactgcatcattgagtcctgggatgatgatgggcatgttcaatatgttgagcaccgtcttgggcgtctccattctgaatcttgggcaaaagagatacaccaagactacttactggtgggatggtcttcaatcgtctgatgattttgctttgattgtgaacgcacccaactatgcaggaattcaagctggagttgacaggttttatcgaacctgtaagctgctcggaattaatatgagcaaaaagaagtcttacataaacagaacaggtacctttgaattcacgagctttttctatcgttatgggtttgttgccaatttcagcatggagcttcctagttttggggtgtcaggggtcaatgaatctgcagacatgagtattggagtcactgtcatcaaaaacaatatgataaacaatgaccttggcccagcaactgctcaaatggcccttcagttatttataaaagactacaggtacacttatcgatgccaccgaggtgacacacaaatacaaacccggagatcatttgagataaagaaactatgggaccaaacccgctcaaaagctggactgttggtctctgatggaggccccaatttgtataacattagaaatctccatattcctgaagtttgcttgaaatgggagttgatggatgaggattaccaggggcgtttatgcaacccattaaacccgtttgtcagccataaagagattgaatcagtgaacagtgcagtgataatgccggcacatggtccagccaaaaatatggagtatgacgctgttgcaacaacacactcctgggtccccaaaagaaatcgatccattttgaacacgagccaaagggggatacttgaagatgagcaaatgtatcagaggtgctgcaatttatttgaaaaattcttcccaagtagctcatacagaagaccagttggaatatccagtatggtagaggctatggtctcaagagcccgaattgatgcacggattgatttcgaatctggaaggataaagaaagaggaatttgctgagatcatgaagatctgttccaccattgaagacctcagacggcaaaaatga

>H1N1_PB1_CY080903_A/Boston/65/2009

atggatgtcaatccgacattacttttcttaaaagtgccagcacaaaatgctataagcacaacttttccttatactggtgaccctccttacagccatgggacaggaacagggtacaccatggatacagtcaacaggacacaccagtactcagaaagaggaagatggacaaaaaataccgaaacgggagcaccgcaacttaacccaattgatggtcccttaccggaagacaatgaaccaagtggctatgcccaaacagattgtgtattagaagcaatggctttccttgaagaatcccatcccggtatctttgaaaactcttgtattgaaacaatggaggttgttcaacaaacaagggtggacaaactgacacaaggcagacagacctatgactggactctaaataggaaccagcctgctgccacagcattggcaaacactatagaagtattcagatcaaacggcctcatagcaaatgaatctggaaggctaatagacttccttaaagatgtaatggagtcgatggacagaggcgaagtagaggtcacaactcattttcaaagaaagaggagagtgagagacaatgtaactaaaaaaatggtgacccaaagaacaataggcaaaaagaaacataaattagacaaaagaagttacctaattagggcattaaccctgaacacaatgaccaaagatgctgagagggggaaactaaaacgcagagcaattgcaaccccaggaatgcaaataagggggtttgtatactttgttgagacactggcaagaagcatatgtgaaaagcttgaacaatcaggattgccagttggaggaaatgagaagaaagcaaagttagcaaatgttgtaaggaagatgatgaccaactcccaggacactgaaatttctttcaccataaccggagacaacacaaaatggaacgaaaatcaaaaccctagaatgttcttggccatgatcacatatataaccaaaaatcagcctgaatggttcagaaatattctaagtattgctccaataatgttttcaaacaagatggcgagactaggtaaggggtacatgtttgaaagcaagagtatgaaactgagaactcaaatacctgcagagatgctagccaacatagacttgaaatatttcaatgattcaactaaaaagaaaattgaaaaaatccgaccattattaatagatggaactgcatcattgagtcctggaatgatgatgggcatgttcaatatgttgagcaccgtattgggcgtctccattctgaatcttgggcaaaagagatacaccaagactacttactggtgggatggtcttcaatcgtctgatgattttgctttaattgtgaacgcacccaactatgcaggaattcaagctggagttgacaggttttatcgaacctgtaagctgctcggaattaatatgagcaaaaagaagtcttacataaacagaacaggtacctttgaattcacgagctttttctatcgttatgggtttgttgccaatttcagcatggagcttcctagttttggggtgtctggggtcaatgaatctgcagacatgagtattggagtcactgtcatcaaaaacaatatgataaacaatgaccttggcccagcaactgctcaaatggcccttcagttatttataaaagattacaggtacacttatcgatgccaccgaggtgacacacaaatacaaacccgaagatcatttgagataaagaaactatgggaccaaacccgctcaaaagctggactgttggtctctgatggaggccccaatttgtataacattagaaatctccatattcctgaagtttgcttgaaatgggagttgatggatgaggattaccaggggcgtttatgcaacccattaaacccgtttgtcagccataaagagattgaatcagtgaacagtgcagtgataatgccggcacatggtccagccaaaaatatggagtatgacgctgttgcaacaacacactcctgggtccccaaaagaaatcgatccattttgaacacgagccaaagggggatacttgaagatgagcaaatgtatcagaggtgctgcaatttatttgaaaaattcttcccaagtagctcatacagaagaccagttggaatatccagtatggtagaggctatggtctcaagagcccgaattgatgcacggattgatttcgaatctggaaggataaagaaagaggaatttgctgagatcatgaagatctgttccaccattgaagatctcagacggcaaaaatga

>H1N1_PB1_CY084229_A/Taiwan/141/2002

atggatgtcaatccgaccttacttttcttaaaagtgccagcacaaaatgctataagcacaactttcccttatactggtgatcctccttacagccatgggacaggaacagggtacaccatggatacagtcaacaggacacatcagtactcagaaagaggaagatggacaaaaaataccgaaactggagcaccgcaactcaacccaattgatggaccactaccaaaagacaatgaaccaagtggctatgcccaaacagattgtgtattagaagcaatggctttccttgaggaatcccatcctggtatttttgaaaactcttgtattgaaacaatggaggttgttcagcaaacaagggtggacaaactgacacaaggcagacagacctatgactggactctaaatagaaaccagcctgctgccacagcattggccaacactatagaagtgttcagatcaaacggcctcatagcaaatgaatctgggaggctaatagacttccttaaagatgtaatggagtcgatggacagagacgaagtagagatcacaacacattttcagagaaagaggagggtgagagacaatgtaactaaaaaaatggtgacccaaagaacaataggcaaaaagaaacataaattagacaaaagaagttacctaattagggcattaaccctgaacacaatgaccaaagatgctgagagggggaaactaaaacgtagagcaattgcaaccccaggaatgcaaataagggggtttgtatactttgttgagacactggcaagaagcatatgtgaaaagcttgaacaatcagggttgccagtcgggggaaatgaaaagaaagcaaagttagcaaatgttgtaaggaagatgatgaccaactcccaggacactgaaatttctttcaccatcactggagataacacaaaatggaacgaaaatcaaaaccctagaatgtttttggccatgatcacatatataaccaaaaatcagcctgaatggttcagaaatattctaagtattgctccaataatgttttcaaacaaaatggcgagactaggtaaggggtacatgtttgaaagcaagagtatgaaactgagaactcaaatacctgcagagatgctagccaacatagatttgaaatatttcaatgattcaactaaaaagaaaattgaaaaaatccggccattattaatagatggaactgcatcattgagccctggaatgatgatgggcatgttcaatatgttaagcactgtcttgggcgtctccattctgaatcttgggcaaaagagatacaccaagactacttactggtgggatggtcttcaatcgtctgatgattttgctctgattgtgaatgcacccaactatgcaggaattcaagctggagttgacaggttttatcgaacctgtaagctgctcggaattaatatgagcaaaaagaagtcttacataaacagaacaggtacctttgaattcacgagctttttctatcgttatgggtttgttgccaatttcagcatggagcttcctagctttggggtgtctggggtcaatgagtctgcagacatgagtattggagtcactgtcatcaaaaacaatatgataaacaatgaccttggcccagcaactgctcaaatggcccttcagttatttatcaaagactacaggtacacgtatcgatgccacagaggtgacacacaaatacaaacccggagatcatttgagataaagaaactatgggaccaaacccgctccaaagctgggctgttggtctctgatggaggccccaatttgtataacattagaaatctccatattcctgaagtctgcttgaaatgggagttgatggatgaggattaccaggggcgtttatgcaacccattgaacccatttgtcagtcataaagagattgagtcagtgaacaatgcagtggtgatgccggcacatggtccagccaaaaatatggagtatgacgctgttgcaacaacacactcctgggttcccaaaaggaatcgatccattttgaatacgagccaaagggggatacttgaggatgagcaaatgtatcagaggtgctgcaatttatttgaaaaattcttcccaagtagttcatacagaagaccagttggaatatccagtatggtagaggctatggtttccagagcccgaattgatgcaagaattgatttcgaatctggaaggataaagaaagaggaattcgctgagatcatgaagacctgttccaccattgaagacctcagacggcaaaaatag

>H1N1_PB1_JF682627_A/Czech Republic/1/2011

atggatgtcaatccgactctacttttcctaaaaattccagcgcaaaatgccataagcaccacattcccttatactggagatcctccatacagccatggaacaggaacaggatacaccatggacacagtgaacagaacacaccaatactcagaaaagggaaagtggacgacaaacacagagactggtgcaccccagctcaacccgattgatggaccactacctgaggataatgaaccaagtgggtatgcacaaacagactgtgttctagaggctatggctttccttgaagaatcccacccagggatatttgagaattcatgccttgaaacaatggaagttgttcaacaaacaagggtagataaactaactcaaggccgccagacttatgattggacattaaacagaaatcaaccggcagcaactgcattggccaacaccatagaagtctttagatcgaatggcctaacagctaatgagtcaggcaggctaatagatttcttaaaggatgtaatggaatcaatgaacaaagaggaaatagagataacaactcactttcaaagaaaaaggagagtaagagacaacatgaccaagaagatggtcacgcaaagaacaatagggaagaaaaaacaaagactgaataagagaggctatctaataagagcactgacattaaatacgatgaccaaagatgcagagagaggcaagttaaaaagaagggctatcgcaacacctgggatgcagattagaggtttcgtatactttgttgaaactttagctaggagcatttgcgaaaagcttgaacagtctgggctcccagtagggggcaatgaaaagaaggccaaactggcaaatgttgtgagaaagatgatgactaattcacaagacacagagatttctttcacaatcactggggacaacactaagtggaatgaaaatcaaaatcctcgaatgttcctggcgatgattacatatatcaccagaaatcaacccgagtggttcagaaacatcctgagcatggcacccataatgttctcaaacaaaatggcaagactagggaaagggtacatgttcgagagtaaaagaatgaagattcgaacacaaataccagcagaaatgctagcaagcattgacctgaagtacttcaatgaatcaacaaagaagaaaattgagaaaataaggcctcttttaatagatggcacagcatcactgagtcctgggatgatgatgggcatgttcaacatgctaagtacggtcttgggagtctcgatactgaatcttggacaaaagaaatacaccaagacaatatactggtgggatgggctccaatcatccgacgattttgctctcatagtgaatgcaccaaaccatgagggaatacaagcaggagtggacagattctacaggacctgcaagttagtgggaatcaacatgagcaaaaagaagtcctatataaataagacagggacatttgaattcacaagctttttttatcgctatggatttgtggctaattttagcatggagctacccagctttggagtgtctggagtaaatgaatcagctgacatgagtattggagtaacagtgataaagaacaacatgataaacaatgaccttggacctgcaacggcccagatggctcttcaattgttcatcaaagactacagatacacatataggtgccataggggagacacacaaattcagacgagaagatcatttgagttaaagaagctgtgggatcaaacccaatcaaaggtagggctattagtatcagatggaggaccaaacttatacaatatacggaatcttcacattcctgaagtctgcttaaaatgggagctaatggatgatgattatcggggaagactttgtaatcccctgaatccctttgtaagtcataaagagattgattctgtaaacaatgctgtggtaatgccagcccatggtccagccaaaagcatggaatatgatgctgttgcaactacacattcctggattcccaagaggaatcgttctattctcaacacaagccaaaggggaattcttgaggatgaacagatgtaccagaagtgctgcaatctattcgagaaatttttccctagcagttcatataggagaccggttggaatttctagcatggtggaggccatggtgtctagggcccggattgatgccagggtcgactttgagtctggacggatcaagaaagaagagttctctgagatcatgaagatctgttccaccattgaagaactcagacggcaaaaataa

>H1N1_PB1_CY092830_A/Sydney/DD3-40/2010

atggatgtcaatccgactctacttttcctaaaaattccagcgcaaaatgccataagcaccacattcccttatactggagatcctccatacagccatggaacaggaacaggatacaccatggacacagtaaacagaacacaccaatactcagaaaagggaaagtggacgacaaacacagagactggtgcaccccagctcaacccgattgatggaccactacctgaggataatgaaccaagtgggtatgcacaaacagactgtgttctagaggctatggctttccttgaagaatcccacccaggaatatttgagaattcatgccttgaaacaatggaagttgttcaacaaacaagggtagataaactaactcaaggtcgccagacttatgattggacattaaacagaaatcaaccggcagcaactgcattggccaacaccatagaagtctttagatcgaatggcctaacagctaatgagtcaggaaggctaatagatttcttaaaggatgtaatggaatcaatgaacaaagaggaaatagagataacaacccactttcaaagaaaaaggagagtaagagacaacatgaccaagaagatggtcacgcaaagaacaatagggaagaaaaaacaaagactgaataagagaggctatctaataagagcactgacattaaatacgatgaccaaagatgcagagagaggcaagttaaaaagaagggctatcgcaacacctgggatgcagattagaggtttcgtatactttgttgaagctttagctaggagcatttgcgaaaagcttgaacagtctgggctcccagtagggggcaatgaaaagaaggccaaactggcaaatgttgtgagaaagatgatgactaattcacaagacacagagatttctttcacaatcactggggacaacactaagtggaatgaaaatcaaaatcctcgaatgttcctggcaatgattacatatatcaccagaaatcaacccgagtggttcagaaacatcctgagcatggcacccataatgttctcaaacaaaatggcaagactagggaaagggtacatgttcgagagtaaaagaatgaagattcgaacacaaataccagcagaaatgctagcaagcattgacctgaagtacttcaatgaatcaacgaagaagaaaattgagaaaataaggcctcttctaatagatggcacagcatcactgagtcctgggatgatgatgggcatgttcaacatgctaagtacggtcttgggagtctcgatactgaatcttggacaaaagaaatacaccaagacaatatactggtgggatggactccaatcatccgacgattttgctctcatagtgaatgcaccaaaccatgagggaatacaagcaggagtggacagattctacaggacctgcaaattagtgggaatcaacatgagcaaaaagaagtcctatataaataagacagggacatttgaattcacaagctttttttatcgctatggatttgtggctaattttagcatggagctacccagctttggagtgtctggagtaaatgaatcagctgacatgagtattggagtaacagtgataaagaacaacatgataaacaatgaccttggacctgcaacggcccagatggctcttcaattgttcatcaaagactacagatacacatataggtgccataggggagacacacaaattcagacgagaagatcatttgagttaaagaagctgtgggatcaaacccaatcaaaggtagggctattagtatcagatggaggaccaaacttatacaatatacggaatcttcacattcctgaagtctgcttaaaatgggagctaatggatgatgattatcggggaagactttgtaatcccctgaatccctttgtcagtcataaagagattgattctgtaaacaatgctgtggtaatgccagcccatggtccagtcaaaagcatggaatatgatgccgttgcaactacacattcctggattcccaagaggaatcgttctattctcaacacaagccaaaggggaattcttgaggatgaacagatgtaccagaagtgctgcaatctattcgagaaatttttccctagcagttcatataggagaccggttggaatttctagcatggtggaggccatggtgtctagggcccggattgatgccagggtcgacttcgagtctggacggatcaagaaagaagagttctctgagatcatgaagatctgttccaccattgaagaactcagacggcaaaaataa

>H1N1_PB1_CY088637_A/Pune/NIV10278/2009

atggatgtcaatccgactctacttttcctaaaaattccagcgcaaaatgccataagcaccacattcccttatactggagatcctccatacagccatggaacaggaacaggatacaccatggacacagtaaacagaacacaccaatactcagaaaagggaaagtggacgacaaacacagagactggtgcaccccagctcaacccgattgatggaccactacctgaggataatgaaccaagtgggtatgcacaaacagactgtgttctagaggctatggctttccttgaagaatcccacccaggaatatttgagaattcatgccttgaaacaatggaagttgttcaacaaacaagggtagataaactaactcaaggtcgccagacttatgattggacattaaacagaaatcaaccggcagcaactgcattggccaacaccatagaagtctttagatcgaatggcctaacagctaatgagtcaggaaggctaatagatttcttaaaggatgtaatggaatcaatgaacaaagaggaaatagagataacaacccactttcaaagaaaaaggagagtaagagacaacatgaccaagaagatggtcacgcaaagaacaatagggaagaaaaaacaaagactgaataagagaggctatctaataagagcactgacattaaatacgatgaccaaagatgcagagagaggcaagttaaaaagaagggctatcgcaacacctgggatgcagattagaggtttcgtatactttgttgaaactttagctaggagcatttgcgaaaagcttgaacagtctgggctcccagtagggggcaatgaaaagaaggccaaactggcaaatgttgtgagaaagatgatgactaattcacaagacacagagatttctttcacaatcactggggacaacactaagtggaatgaaaatcaaaatcctcgaatgttcctggcgatgattacatatatcaccagaaatcaacccgagtggttcagaaacatcctgagcatggcacccataatgttctcaaacaaaatggcaagactagggaaagggtacatgttcgagagtaaaagaatgaagattcgaacacaaataccagcagaaatgctagcaagcattgacctgaagtacttcaatgaatcaacaaagaagaaaattgagaaaataaggcctcttctaatagatggcacagcatcactgagtcctgggatgatgatgggcatgttcaacatgctaagtacggtcttgggagtctcgatactgaatcttggacaaaagaaatacaccaagacaatatactggtgggatgggctccaatcatccgacgattttgctctcatagtgaatgcaccaaaccatgagggaatacaagcaggagtggacagattctacaggacctgcaagttagtgggaatcaacatgagcaaaaagaagtcctatataaataagacagggacatttgaattcacaagctttttttatcgctatggatttgtggccaatttcagcatggagctggtcagcttgggagtgtctggagtaaatgaatcagctgacatgagtattggagtaacagtgataaagaacaacatgataaacaatgaccttggacctgcaacggcccagatggctcttcaattgttcatcaaagactacagatacacatataggtgccataggggagacacacaaattcagacgagaagatcatttgagttaaagaagctgtgggatcaaacccaatcaaaggtagggctattagtatcagatggaggaccaaacttatacaatatacggaatcttcacattcctgaagtctgcttaaaatgggagctaatggatgatgattatcggggaagactttgtaatcccctgaatccctttgtcagtcataaagagattgattctgtaaacaatgctgtggtaatgccagcccatggtccagccaaaagcatggaatatgatgccgttgcaactacacattcctggattcccaagaggaatcgttctattctcaacacaagccaaaggggaattcttgaggatgaacagatgtaccagaagtgctgcaatctattcgagaaatttttccctagcagttcatataggagaccggttggaatttctagcatggtggaggccatggtgtctagggcccggattgatgccagggtcgacttcgagtctggacggatcaagaaagaagagttctctgagatcatgaagatctgttccaccattgaagaactcagacggcaaaaataa

>H1N1_PB1_CY100938_A/Sullana/WRAIR1707P/2009

atggatgtcaatccgacattacttttcttaaaagtgccagcacaaaatgctataagcacaacttttccttatactggtgaccctccatacagccatggaacaggaacaggatacaccatggacacagtcaacagaacacaccagtactcagaaagaggaagatggacaaaaaataccgaaacgggagcaccgcaacttaacccaattgatggtcccttaccggaagacaatgaaccaagtggctatgcccaaacagattgtgtattagaagcaatggctttccttgaagaatcccatcccggtatctttgaaaactcttgtattgaaacaatggaggttgttcaacaaacaagggtggacaaactgacacaaggcagacagacctatgactggactctaaataggaaccagcctgcagccacagcattggcaaacactatagaagtattcagatcaaacggcctcatagcaaatgagtctgggaggctaatagacttccttaaagatgtaatggagtcgatggacagaggcgaagtagaggtcacaactcattttcaaagaaagaggagagtgagagacaatgtaactaaaaaaatggtgacccaaagaacaataggcaaaaagaaacataaattagacaaaagaagttacctaattagggcattaaccctgaacacaatgaccaaagatgctgagagggggaaactaaaacgcagagcaattgcaacccctggaatgcaaataagggggtttgtatactttgttgagacactggcaagaagcatatgtgaaaagcttgaacaatcaggattgccagttggaggaaatgagaagaaagcaaagttagcaaatgttgtaaggaagatgatgaccaactcccaggacactgaaatttctttcaccataaccggagataacacaaaatggaacgaaaatcaaaaccctagaatgttcttggccatgatcacatatataaccaaaaatcagcctgaatggttcagaaatattctaagtattgctccaataatgttttcaaacaagatggcgagactaggtaaggggtacatgtttgaaagcaagagtatgaaactgagaactcaaatacctgcagagatgctagccaacatagacttgaaatatttcaatgattcaactaaaaagaaaattgaaaaaatccgaccattattaatagatggaactgcatcattgagtcctggaatgatgatgggcatgttcaatatgttgagcacggtcttgggcgtctccattctgaatcttgggcagaagagatacaccaagactacttactggtgggatggtcttcaatcgtctgatgattttgctttgattgtgaacgcacccaactatgcaggaattcaagctggagttgacaggttttatcgaacctgtaagctgctcggaattaatatgagcaaaaagaagtcttacataaacagaacaggtacctttgaattcacgagctttttctatcgttatgggtttgttgccaatttcagcatggagcttcctagttttggggtgtcaggggtcaatgaatctgcagacatgagtattggagtcactgtcatcaaaaacaatatgataaacaatgaccttggcccagcaactgctcaaatggcccttcagttatttataaaagattacaggtacacttatcgatgccaccgaggtgacacacaaatacaaacccggagatcatttgagataaagaaactatgggaccaaacccgctcaaaagctggactgttggtctctgatggaggccccaatttgtataacattagaaatctccatattcctgaagtttgcttgaaatgggagttgatggatgaggattaccaggggcgtttatgcaacccattaaacccgtttgtcagccataaagagattgaatcagtgaacagtgcagtgataatgccggcacatggtccagccaaaaatatggagtatgacgctgttgcaacaacacactcctgggtccccaaaagaaatcgatccattttgaacacgagccaaagggggatacttgaagatgagcaaatgtatcagaggtgctgcaatttatttgaaaaattcttcccaagtagctcatacagaagaccagttggaatatccagtatggtagaggctatggtctcaagagcccgaattgatgcacggattgatttcgaatctggaaggataaagaaagaggaatttgctgagatcatgaagatctgttccaccattgaagacctcagacggcaaaaatga

>H1N1_PB1_CY095868_A/Hubei/75/2009

atggatgtcaatccgactctacttttcctaaaaattccagcgcaaaatgccataagcaccacattcccttatactggagatcctccatacagccatggaacaggaacaggatacaccatggacacagtaaacagaacacaccaatactcagaaaagggaaagtggacgacaaacacggagactggtgcaccccagctcaacccgattgatggaccactacctgaggataatgaaccaagtgggtatgcacaaacagactgtgttctagaggctatggctttccttgaagaatcccatccaggaatatttgagaattcatgccttgaaacaatggaagttgttcaacaaacaagggtagataaactaactcaaggtcgccagacttatgattggacattaaacagaaatcaaccggcagcaactgcattggccaacaccatagaagtctttagatcgaatggcctaacagctaatgagtcaggaaggctaatagatttcttaaaggatgtaatggaatcaatgaacaaagaggaaatagagataacaacccactttcaaagaaaaaggagagtaagagacaacatgaccaagaagatggtcacgcaaagaacaatagggaagaaaaaacaaagactgaataagagaggctatctaataagagcactgacattaaatacgatgaccaaagatgcagagagaggcaagttaaaaagaagggctatcgcaacacctgggatgcagattagaggtttcgtatactttgttgaaactttagctaggagcatttgcgagaagcttgaacagtctgggctcccagtagggggcaatgaaaagaaggccaaactggcaaatgttgtgagaaagatgatgactaattcacaagacacagagatttctttcacaatcactggggacaacactaagtggaatgaaaatcaaaatcctcgaatgttcctggcgatgattacatatatcaccagaaatcaacccgagtggttcagaaacatcctgagcatggcacccataatgttctcaaacaaaatggcaagactagggaaagggtacatgttcgagagtaaaagaatgaagattcgaacacaaataccagcagaaatgctagcaagcattgacctgaagtacttcaatgagtcaacaaagaagaaaattgagaaaataaggcctcttctaatagatggcacagcatcactgagtcctgggatgatgatgggcatgttcaacatgttaagtacggtcttgggagtctcgatactgaatcttggacaaaagaaatacaccaagacaatatactggtgggatgggctccaatcatccgacgattttgctctcatagtgaatgcaccaaaccatgagggaatacaagcaggagtggacagattctacaggacctgcaagttagtgggaatcaacatgagcaaaaagaagtcctatataaataagacagggacatttgaattcacaagctttttttatcgctatggatttgtggctaattttagcatggagctacccagctttggagtgtctggagtaaatgaatcagctgacatgagtattggagtaacagtgataaagaacaacatgataaacaatgaccttggacctgcaacggcccagatggctcttcaattgttcatcaaagactacagatacacatataggtgccataggggagacacacaaattcagacgagaagatcatttgagttaaagaagctgtgggatcaaacccaatcaaaggtagggctattagtatcagatggaggaccaaacttatacaatatacggaatcttcacattcctgaagtctgcttaaaatgggagctaatggatgatgattatcggggaagactttgtaatcccctgaatccctttgtcagtcataaagagattgattctgtaaacaatgctgtggtaatgccagcccatggtccagccaaaagcatggaatatgatgccgttgcaactacacattcctggattcccaagaggaatcgttctattctcaacacaagccaaaggggaattcttgaggatgaacagatgtaccagaagtgctgcaatctattcgagaaatttttccctagcagttcatataggagaccggttggaatttctagcatggtggaggccatggtgtctagggcccggattgatgccagggtcgacttcgagtctggacggatcaagaaagaagagttctctgagatcatgaagatctgttccaccattgaagaactcagacggcaaaaataa

>H1N1_PB1_CY104908_A/DaNang/DN364/2008

atggatgtcaatccgacattacttttcttaaaagtgccagcacaaaatgctataagcacaacttttccttatactggtgaccctccttacagccatgggacaggaacagggtacaccatggatacagtcaacaggacacatcagtactcagaaagaggaagatggacaaaaaataccgaaactggagcaccgcaactcaacccaattgatgggccactaccagaagacaatgaaccaagtggctatgcccaaacagattgtgtattagaagcaatggctttccttgaagaatcccatcctggtatttttgaaaactcttgtattgaaacaatggagattgttcagcaaacaagggtggacaaactgacacaaggcagacagacctatgactggactctaaataggaaccagcctgctgccacagcattggccaacactatagaagtattcagatcaaacggcctcatagcaaatgaatctgggaggctaatagacttccttaaagatgtaatggagtcgatggacagagaagaagtagaggtcacaactcattttcagagaaagaggagagtgagagacaatgtaactaaaaaaatggtgacccaaagaacaataggcaaaaagaaacataaattagacaaaaggagttacctaattagagcattaactctgaacacaatgaccaaagatgctgaaagggggaaactaaaacgcagagcaattgcaaccccaggaatgcaaataagggggtttgtatactttgttgaaacactggcaagaagcatatgtgaaaagcttgaacaatcagggttgccagttggaggaaatgagaagaaagcaaagttagcaaatgttgtaaggaagatgatgaccaactcccaggacactgaaatttctttcaccatcactggagataacacaaaatggaacgaaaatcaaaaccctagaatgtttttggccatgatcacatatataaccagaaatcagcctgaatggttcagaaatattctaagtgttgctccaataatgttttcaaacaaaatggcgagactaggtaaggggtacatgtttgaaagcaagagtatgaaactaagaactcaaatacctgcagagatactagccggcatagatttgaagtatttcaatgagtcaactaaaaagaaaattgaaaaaatccgaccattattaatagatggaactgcatcattgagtcctggaatgatgatgggcatgttcaatatgttaagcactgtcttgggcgtctccattctgaatcttgggcaaaagagatacaccaagactacttactggtgggatggtcttcaatcgtctgatgattttgctctgatagtgaatgcgcccaactatgcaggaattcaagctggagttgacaggttttatcgaacctgtaagctgctcggaattaatatgagcaaaaagaagtcttacataaacaggacgggtacctttgaattcacaagctttttctatcgttatgggtttgttgccaatttcagcatggagcttcctagttttggggtgtcaggggtcaatgaatctgcagacatgagtattggagtcactgttatcagaaacaatatgataaacaatgaccttggcccagcaactgctcaaatggcccttcagttatttataaaagattacagatatacgtatcgatgccacagaggtgacacacaaatacaaacccggagatcatttgagataaagaaattatgggaccaaacccgctccaaagctgggctgttggtttctgatggaggccccaatttatataacattagaaatctccatattcctgaagtctgtttgaaatgggagttgatggatgaagattaccaggggcgtttatgcaatccattgaacccatttgtcagtcataaagagattgaatcagtgaacaatgcagtgatgatgccggcacatggtccagccaaaaatatggagtatgacgctgttgcaacaacacattcctgggttcccaaaaggaatcgatccattttgaatacgagccaaagggggatacttgaggatgagcaaatgtatcagaggtgctgcaatttatttgaaaaattctttccaagtagctcatacagaagaccagttgggatatccagtatggtagaggctatggtttccagagcccgaattgatgcacggattgatttcgaatctggaaggataaagaaagaggaattcactgagatcatgaagacctgttccaccattgaagacctcagacggcaaaaatag

>H1N1_PB1_CY104964_A/Hue/H259/2008

atggatgtcaatccgacattacttttcttaaaagtgccagcacaaaatgctataagcacaacttttccttatactggtgaccctccttacagccatgggacaggaacagggtacaccatggatacagtcaacaggacacatcagtactcagaaagaggaagatggacaaaaaataccgaaactggagcaccgcaactcaacccaattgatgggccactaccagaagacaatgaaccaagtggctatgcccaaacagattgtgtattagaagcaatggctttccttgaagaatcccatcctggtatttttgaaaactcttgtattgaaacaatggagattgttcagcaaacaagggtggacaaactgacacaaggcagacagacctatgactggactctaaataggaaccagcctgctgccacagcattggccaacactatagaagtattcagatcaaacggcctcatagcaaatgaatctgggaggctaatagacttccttaaagatgtaatggagtcgatggacagagaagaagtagaggtcacaactcattttcagagaaagaggagagtgagagacaatgtaactaaaaaaatggtgacccaaagaacaataggcaaaaagaaacataaattagacaaaaggagttacctaattagagcattaactctgaacacaatgaccaaagatgctgaaagggggaaactaaaacgcagagcaattgcaaccccaggaatgcaaataagggggtttgtatactttgttgaaacactggcaagaagcatatgtgaaaagcttgaacaatcagggttgccagttggaggaaatgagaagaaagcaaagttagcaaatgttgtaaggaagatgatgaccaactcccaggacactgaaatttctttcaccatcactggagataacacaaaatggaacgaaaatcaaaaccctagaatgtttttggccatgatcacatatataaccagaaatcagcctgaatggttcagaaatattctaagtgttgctccaataatgttttcaaacaaaatggcgagactaggtaaggggtacatgtttgaaagcaagagtatgaaactaagaactcaaatacctgcagagatactagccggcatagatttgaagtatttcaatgagtcaactaaaaagaaaattgaaaaaatccgaccattattaatagatggaactgcatcactgagtcctggaatgatgatgggcatgttcaatatgttaagcactgtcttgggcgtctccattctgaatcttgggcaaaagagatacaccaagactacttactggtgggatggtcttcaatcgtctgatgattttgctctgatagtgaatgcgcccaactatgcaggaattcaagctggagttgacaggttttatcgaacctgtaagctgctcggaattaatatgagcaaaaagaagtcttacataaacaggacgggtacctttgaattcacaagctttttctatcgttatgggtttgttgccaatttcagcatggagcttcctagttttggggtgtcaggggtcaatgaatctgcagacatgagtattggagtcactgttatcagaaacaatatgataaacaatgaccttggcccagcaactgctcaaatggcccttcagttatttataaaagattacagatatacgtatcgatgccacagaggtgacacacaaatacaaacccggagatcatttgagataaagaaattatgggaccaaacccgctccaaagctgggctgttggtttctgatggaggccccaatttatataacattagaaatctccatattcctgaagtctgtttgaaatgggagttgatggatgaagattaccaggggcgtttatgcaatccattgaacccatttgtcagtcataaagagattgaatcagtgaacaatgcagtgatgatgccggcacatggtccagccaaaaatatggagtatgacgctgttgcaacaacacattcctgggttcccaaaaggaatcgatccattttgaatacgagccaaagggggatacttgaggatgagcaaatgtatcagaggtgctgcaatttatttgaaaaattctttccaagtagctcatacagaagaccagttgggatatccagtatggtagaggctatggtttccagagcccgaattgatgcacggattgatttcgaatctggaaggataaagaaagaggaattcgctgagatcatgaagacctgttccaccattgaagacctcagacggcaaaaatag

>H1N1_PB1_CY111705_A/Hong Kong/H090-684-V10/2009

atggatgtcaatccgactctacttttcctaaaaattccagcgcaaaatgccataagcaccacattcccttatactggagatcctccatacagccatggaacaggaacaggatacaccatggacacagtaaacagaacacaccaatactcagaaaagggaaagtggacgacaaacacagagactggtgcaccccagctcaacccgattgatggaccactacctgaggataatgaaccaagtgggtatgcacaaacagactgtgttctagaggctatggctttccttgaagaatcccacccaggaatatttgagaattcatgccttgaaacaatggaagttgttcaacaaacaagggtagataaactaactcaaggtcgccagacttatgattggacattaaacagaaatcaaccggcagcaactgcattggccaacaccatagaagtctttagatcgaatggcctaacagctaatgagtcaggaaggctaatagatttcttaaaggatgtaatggaatcaatgaacaaagaggaaatagagataacaacccactttcaaagaaaaaggagagtaagagacaacatgaccaagaagatggtcacgcaaagaacaatagggaagaaaaaacaaagactgaataagagaggctatctaataagagcactgacattaaatacgatgaccaaagatgcagagagaggcaagttaaaaagaagggctatcgcaacacctgggatgcagattagaggtttcgtatactttgttgaaactttagctaggagcatttgcgaaaagcttgaacagtctgggctcccagtagggggcaatgaaaagaaggccaaactggcaaatgttgtgagaaagatgatgactaattcacaagacacagagatttctttcacaatcactggggacaacactaagtggaatgaaaatcaaaatcctcgaatgttcctggcgatgattacatatatcaccagaaatcaacccgagtggttcagaaacatcctgagcatggcacccataatgttctcaaacaaaatggcaagactagggaaagggtacatgttcgagagtaaaagaatgaagattcgaacacaaataccagcagaaatgctagcaagcattgacctgaagtacttcaatgaatcaacaaagaagaaaattgagaaaataaggcctcttctaatagatggcacagcatcactgagtcctgggatgatgatgggcatgttcaacatgctaagtacggtcttgggagtctcgatactgaatcttggacaaaagaaatacaccaagacaatatactggtgggatgggctccaatcatccgacgattttgctctcatagtgaatgcaccaaaccatgagggaatacaagcaggagtggacagattttacaggacctgcaagttagtgggaatcaacatgagcaaaaagaagtcctatataaataagacagggacatttgaattcacaagctttttttatcgctatggatttgtggctaattttagcatggagctacccagctttggagtgtctggagtaaatgaatcagctgacatgagtattggagtaacagtgataaagaacaacatgataaacaatgaccttggacctgcaacggcccagatggctcttcaattgttcatcaaagactacagatacacatataggtgccataggggagacacacaaattcagacgagaagatcatttgagttaaagaagctgtgggatcaaacccaatcaaaggtagggctattagtatcagatggaggaccaaacttatacaatatacggaatcttcacattcctgaagtctgcttaaaatgggagctaatggatgatgattatcggggaagactttgtaatcccctgaatccctttgtcagtcataaagagattgattctgtaaacaatgctgtggtaatgccagcccatggtccagccaaaagcatggaatatgatgccgttgcaactacacattcctggattcccaagaggaatcgttctattctcaacacaagccaaaggggaattcttgaggatgaacagatgtaccagaagtgctgcaatctattcgagaaatttttccctagcagttcatataggagaccggttggaatttctagcatggtggaggccatggtgtctagggcccggattgatgccagggtcgacttcgagtctggacggatcaagaaagaagagttctctgagatcatgaagatctgttccaccattgaagaactcagacggcaaaaataa

>H1N1_PB1_CY118264_A/Malaysia/2089302/2009

atggatgtcaatccgacattacttttcttaaaagtgccagcacaaaatgctataagcacaacttttccttatactggtgaccctccttacagccatgggacaggaacagggtacaccatggatacagtcaacaggacacaccagtactcagaaagaggaagatggacaaaaaataccgaaacgggagcaccgcaacttaacccaattgatggtcccttaccggaagacaatgaaccaagtggctatgcccaaacagattgtgtattagaagcaatggctttccttgaagaatcccatcccggtatctttgaaaactcttgtattgaaacaatggaggttgttcaacaaacaagggtggacaaactgacacaaggcagacagacctatgactggactctaaataggaaccagcctgctgccacagcattggcaaacactatagaagtattcagatcaaacggcctcatagcaaatgaatctgggaggctaatagacttccttaaagatgtaatggagtcgatggacagaggcgaagtagaggtcacaactcattttcaaagaaagaggagagtgagagacaatgtaactaaaaaaatggtgacccaaagaacaataggcaaaaagaaacataaattagacaaaagaagttacctaattagggcattaaccctgaacacaatgaccaaagatgctgagagggggaaactaaaacgcagagcaattgcaaccccaggaatgcaaataagggggtttgtatactttgttgagacactggcaagaagcatatgtgaaaagcttgaacaatcaggattgccagttggaggaaatgagaagaaagcaaagttagcaaatgttgtaaggaagatgatgaccaactcccaggacactgaaatttctttcaccataaccggagataacacaaaatggaacgaaaatcaaaaccctagaatgttcttggccatgatcacatacataaccaaaaatcagcctgaatggttcagaaatattctaagtattgctccaataatgttttcaaacaagatggcgagactaggtaaggggtacatgtttgaaagcaagagtatgaaactgagaactcaaatacctgcagagatgctagccaacatagacttgaaatatttcaatgattcaactaaaaagaaaattgaaaaaatccgaccattattaatagatggaactgcatcattgagtcctggaatgatgatgggcatgttcaatatgttgagcaccgtcttgggcgtctccattctgaatcttgggcaaaagagatacaccaagactacttactggtgggatggtcttcaatcgtctgatgattttgctttgattgtgaacgcacccaactatgcaggaattcaagctggagttgacaggttttatcgaacctgtaagctgctcggaattaatatgagcaaaaagaagtcttacataaacagaacaggtacctttgaattcacgagctttttctatcgttatgggtttgttgccaatttcagcatggagcttcctagttttggggtgtctggggtcaatgaatctgcagacatgagtattggagtcactgtcatcaaaaacaatatgataaacaatgaccttggcccagcaactgctcaaatggcccttcagttatttataaaagattacaggtacacttatcgatgccaccgaggtgacacacaaatacaaacccggagatcatttgagataaagaagctatgggaccaaacccgctcaaaagctgggctgttggtctctgatggaggccccaatttgtataacattagaaatctccatattcctgaagtttgcttgaaatgggagttgatggatgaggattaccaggggcgtttatgcaacccattaaacccgtttgtcagccataaagagattgaatcagtgaacaatgcagtgataatgccggcacatggtccagccaaaaatatggagtatgacgctgttgcaacaacacactcctgggtccccaaaagaaatcgatccattttgaacacgagccaaagggggatacttgaagatgagcaaatgtatcagaggtgctgcaatttatttgaaaaattcttcccaagtagctcatacagaagaccagttggaatatccagtatggtagaggctatggtctcaagagcccgaattgatgcacggattgatttcgaatctggaaggataaagaaagaggaatttgctgagatcatgaagatctgttccaccattgaagacctcagacggcaaaaatga

>H1N1_PB1_CY119280_A/Malaysia/1842338/2007

atggatgtcaatccgacattacttttcttaaaagtgccagcacaaaatgctataagcacaacttttccttatactggtgaccctccttacagccatgggacaggaacagggtacaccatggatacagtcaacaggacacaccagtactcagaaagaggaagatggacaaaaaataccgaaactggagcaccgcaacttaacccaattgatggtccactaccggaagacaatgaaccaagtggctatgcccaaacagattgtgtattagaagcaatggctttccttgaagaatcccatcccggtatctttgaaaactcttgtattgaaacaatggaggttgttcagcaaacaagggtggacaaactgacacaaggcagacagacctatgactggactctaaataggaaccagcctgctgccacagcattagcaaacactatagaagtattcagatcaaacggcctcatagcaaatgaatctgggaggctaatagacttccttaaagatgtaatggagtcgatggacagaggcgaagtagaggtcacaactcattttcaaagaaagaggagagtgagagacaatgtaactaaaaaaatggtgacccaaagaacaataggcaaaaagaaacataaattagataaaagaagttacctaattagggcattaaccctgaacacaatgaccaaagatgctgagagggggaaactaaaacgcagagcaattgcaaccccaggaatgcaaataagggggtttgtatactttgttgagacactggcaagaagcatatgtgaaaagcttgaacaatcaggattgccagttggaggaaatgagaagaaagcaaagttagcaaatgttgtaaggaagatgatgaccaactcccaggacactgaaatttctttcaccataaccggagataacacaaaatggaacgaaaatcaaaaccctagaatgttcttggccatgatcacatatataaccaaaaatcagcctgaatggttcagaaatattctaagtattgctccaataatgttttcaaacaagatggcgagactaggtaaggggtacatgtttgaaagcaagagtatgaaactgagaacccaaatacctgcagagatgctagccaacatagatttgaaatatttcaatgattcaactaaaaagaaaattgaaaaaatccgaccattattaatagatggaactgcatcattgagtcctggaatgatgatgggcatgttcaatatgttgagcaccgtcttgggcgtctccattctgaatcttgggcaaaagagatacaccaagactacttactggtgggatggtcttcaatcgtctgatgattttgctttgattgtgaatgcacccaactatgcaggaattcaagctggagttgacaggttttatcgaacctgtaagctgctcggaattaatatgagcaaaaagaagtcttacataaacagaacaggtacctttgaattcacgagctttttctatcgttatgggtttgttgccaatttcagcatggagcttcctagttttggggtgtctggggtcaatgaatctgcagacatgagtattggagtcactgtcatcaaaaacaatatgataaacaatgaccttggcccagcaactgctcaaatggcccttcagttatttataaaagattacaggtacacttatcgatgccaccgaggtgacacacaaatacaaacccggagatcatttgagataaagaaactatgggaccaaacccgctcaaaagctgggctgttggtctctgatggaggccccaatttatataacattagaaatctccatattcctgaagtttgcttgaaatgggagttgatggatgaggattaccaggggcgtttatgcaacccattaaacccgtttgtcagccataaagagattgaatcagtgaacaatgcagtgataatgccggcacatggtccagccaaaaatatggagtatgacgctgttgcaacaacacactcctgggtccccaaaagaaatcgatccattttgaacacgagccaaagggggatacttgaagatgagcaaatgtatcagaggtgctgcaatttatttgaaaaattcttcccaagtagctcatacagaagaccagttggaatatccagtatggtagaggctatggtctcaagagcccgaattgatgcacggattgatttcgaatctggaaggataaagaaagaggaatttgctgagatcatgaagatctgttccaccattgaagacctcagacggcaaaaatga

>H1N1_PB1_CY123206_A/Singapore/GP4000/2009

atggatgtcaatccgactctacttttcctaaaacttccagcgcaaaatgccataagcaccacattcccttatactggagatcctccatacagccatggaacaggaacaggatacaccatggacacagtaaacagaacacaccaatactcagaaaagggaaagtggacgacaaacacagagactggtgcaccccagctcaacccgattgatggaccactacctgaggataatgaaccaagtgggtatgcacaaacagactgtgttctagaggctatggctttccttgaagaatcccacccaggaatatttgagaattcatgccttgaaacaatggaagttgttcaacaaacaagggtagataaactaactcaaggtcgccagacttatgattggacattaaacagaaatcaaccggcagcaactgcattggccaacaccatagaagtctttagatcgaatggcctaacagctaatgagtcaggaaggctaatagatttcttaaaggatgtaatggaatcaatgaacaaagaggaaatagagataacaacccactttcaaagaaaaaggagagtaagagacaacatgaccaagaagatggtcacgcaaagaacaatagggaagaaaaaacaaagactgaataagagaggctatctaataagagcactgacattaaatacgatgaccaaagatgcagagagaggcaagttaaaaagaagggctatcgcaacacctgggatgcagattagaggtttcgtatactttgttgaaactttagctaggagcatttgcgaaaagcttgaacagtctgggctcccagtagggggcaatgaaaagaaggccaaactggcaaatgttgtgagaaagatgatgactaattcacaagacacagagatttctttcacaatcactggggacaacactaagtggaatgaaaatcaaaatcctcgaatgttcctggcgatgattacatatatcaccagaaatcaacccgagtggttcagaaacattctgagcatggcacccataatgttctcaaacaaaatggcaagactagggaaagggtacatgttcgagagtaaaagaatgaagattcgaacacaaataccagcagaaatgctagcaagcattgacctgaagtacttcaatgaatcaacaaagaagaaaattgagaaaataaggcctcttctaatagatggcacagcatcactgagtcctgggatgatgatgggcatgttcaacatgctaagtacggtcttgggagtctcgatactgaatcttggacaaaagaaatacaccaagacaatatactggtgggatgggctccaatcatccgacgattttgctctcatagtgaatgcaccaaaccatgagggaatacaagcaggagtggacagattctacaggacctgcaagttagtgggaatcaacatgagcaaaaagaagtcctatataaataagacagggacatttgaattcacaagctttttttatcgctatggatttgtggctaattttagcatggagctacccagctttggagtgtctggagtaaatgaatcagctgacatgagtattggagtaacagtgataaagaacaacatgataaacaatgaccttggacctgcaacggcccagatggctcttcaattgttcatcaaagactacagatacacatataggtgccataggggagacacacaaattcagacgagaagatcatttgagttaaagaagctgtgggatcaaacccaatcaaaggtagggctattagtatcagatggaggaccaaacttatacaatatacggaatcttcacattcctgaagtctgcttaaaatgggagctaatggatgatgattatcggggaagactttgtaatcccctgaatccctttgtcagtcataaagagattgattctgtaaacaatgctgtggtaatgccagcccatggtccagccaaaagcatggaatatgatgccgttgcaactacacattcctggattcccaagaggaatcgttctattctcaacacaagccaaaggggaattcttgaggatgaacagatgtaccagaagtgctgcaatctattcgagaaatttttccctagcagttcatataggagaccggttggaatttctagcatggtggaggccatggtgtctagggcccggattgatgccagggtcgacttcgagtctggacggatcaagaaagaagagttctctgagatcatgaagatctgttccaccattgaagaactcagacggcaaaaataa

>H1N1_PB1_CY123228_A/Singapore/GP4148/2009

atggatgtcaatccgactctacttttcctaaaaattccagcgcaaaatgccataagcaccacattcccttatactggagatcctccatacagccatggaacaggaacaggatacaccatggacacagtaaacagaacacaccaatactcagaaaagggaaagtggacgacaaacacagagactggtgcaccccagctcaacccgattgatggaccactacctgaggataatgaaccaagtgggtatgcacaaacagactgtgttctagaggctatggctttccttgaagaatcccacccgggaatatttgagaattcatgccttgaaacaatggaagttgttcaacaaacaagggtagataaactaactcaaggtcgccagacttatgattggacattaaacagaaatcaaccggcagcaactgcattggccaacaccatagaagtctttagatcgaatggcctaacagctaatgagtcaggaaggctaatagatttcttaaaggatgtaatggaatcaatgaacaaagaggaaatagagataacaacccactttcaaagaaaaaggagagtaagagacaacatgaccaagaagatggtcacgcaaagaacaatagggaagaaaaaacaaagactgaataagagaggctatctaataagagcactgacattaaatacgatgaccaaagatgcagagagaggcaagttaaaaagaagggctatcgcaacacctgggatgcagattagaggtttcgtatactttgttgaaactttagctaggagcatttgcgaaaagcttgaacagtctgggctcccagtagggggcaatgaaaagaaggccaaactggcaaatgttgtgagaaagatgatgactaattcacaagacacagagatttctttcacaatcactggggacaacactaagtggaatgaaaatcaaaatcctcgaatgttcctggcgatgattacatatatcaccagaaatcaacccgagtggttccgaaacatcctgagcatggcacccataatgttctcaaacaaaatggcaagactagggagagggtacatgttcgagagtaaaagaatgaagattcgaacacaaataccagcagaaatgctagcaagcattgacctgaagtacttcaatgaatcaacaaagaagaaaattgagaaaataaggcctcttctaatagatggcacagcatcactgagtcctgggatgatgatgggcatgttcaacatgctaagtacagtcttgggagtctcgatactgaaccttggacaaaagaaatacaccaagacaatatactggtgggatgggctccaatcatccgacgattttgctctcatagtgaatgcaccaaaccatgaggggatacaagcaggagtggacagattctacaggacctgcaagttagtgggaatcaacatgagcaaaaagaagtcctatataaataaaacagggacatttgaattcacaagctttttttatcgctatggatttgtggctaattttagcatggagctacccagctttggagtgtctggagtaaatgaatcagctgacatgagtattggagtaacagtgataaagaacaacatgataaacaatgaccttggacctgcaacggcccagatggctcttcaattgttcatcaaagactacagatacacatacaggtgccataggggagacacacaaattcagacgagaagatcatttgagttaaagaagctgtgggatcaaacccaatcaaaggtagggctattagtatcagatggaggaccaaacttatacaatatacggaatcttcatattcctgaagtctgcttaaaatgggagctaatggatgatgattatcggggaagactttgtaatcccctgaatccctttgtcagtcataaagagattgattctgtaaacaatgctgtggtaatgccagcccatggtccagccaaaagcatggaatatgatgccgttgcaactacacattcctggattcccaagaggaatcgttctattctcaacacaagccaaaggggaattcttgaggatgaacagatgtaccagaagtgctgcaatctattcgagaaatttttccctagcagttcatataggagaccggttggaatttctagcatggtggaggccatggtgtctagggcccggattgatgccagggtcgacttcgagtctggacggatcaagaaagaagagttctctgagatcatgaagatctgttccaccattgaagaactcagacggcaaaaataa

>H1N1_PB1_CY124598_A/Singapore/GP715/2011

atggatgtcaatccgactctacttttcctaaaaattccagcgcaaaatgccataagcaccacattcccttatactggagatcctccatacagccatggaacaggaacaggatacaccatggacacagtaaacagaacacaccaatactcagaaaagggaaagtggacgacaaacacagagactggtgcaccccagctcaacccgattgatggaccactacctgaggataatgaaccaagtgggtatgcacaaacagactgtgttctagaggctatggcctttcttgaagaatcccacccaggaatatttgagaattcatgccttgaaacaatggaagttgttcaacaaacaagggtagataaactgactcaaggtcgccagacttatgattggacattaaacagaaatcaaccggcagcaactgcattggccaacaccatagaagtctttagatcgaatggcctaacagctaatgagtcaggaaggctaatagatttcttaaaggatgtaatggaatcaatgaacaaagaggaaatagagataacaacccactttcaaagaaaaaggagagtaagagacaacatgaccaagaagatggtcacgcaaagaacaatagggaagaaaaaacagagactgaataagagagactatctaataagagcactgacattaaatacgatgaccaaagatgcagagagaggcaagttaaaaagaagggctatcgcaacacctgggatgcagattagaggtttcgtatactttgttgaaactttagctaggagcatttgcgaaaagcttgaacagtctgggctcccagtagggggcaatgaaaagaaggccaaactggcaaatgttgtgagaaagatgatgactaattcacaagacacagagatttctttcacaatcactggggacaacactaagtggaatgaaaatcaaaatcctagaatgttcctggcgatgattacatatatcaccagaaatcaacccgagtggtttagaaacatcctgagcatggcacccataatgttctcaaacaaaatggcaagactagggaaagggtacatgttcgagagtaaaagaatgaagattcgaacacaaataccagcagaaatgctagcaagcattgacctgaagtacttcaatgaatcaacaaagaagaaaattgagaaaataaggcctcttttaatggatggcacagcatcactgagtcctgggatgatgatgggcatgttcaacatgctaagtacggtcttgggagtctcgatactgaatcttggacaaaagaaatacaccaagacaacatactggtgggatgggctccaatcatccgacgattttgctctcatagtgaatgcaccaaaccatgagggaatacaagcaggagtggacagattctacaggacctgcaagttagtgggaatcaacatgagcaaaaagaagtcctatataaataagacagggacatttgaattcacaagctttttttatcgctatggatttgtggctaattttagcatggagctacccagctttggagtgtctggagtaaatgaatcagctgacatgagtattggagtaacagtgataaagaacaacatgataaacaatgaccttggacctgcaacggcccagatggctcttcaattgttcatcaaagactacagatacacatatagatgccataggggagacacacaaattcagacgagaagatcatttgagttaaagaagctgtgggatcaaacccaatcaaaggcagggctattagtatcagatggaggaccaaacttatacaatatacggaatcttcacattcctgaagtctgcttaaaatgggagctaatggatgatgattatcggggaagactttgtaatcccctgaacccctttgtaagtcataaggagattgattctgtaaacaatgctgtggtaatgccagcccatggtccagccaaaagcatggaatatgatgccgttgcaactacacattcctggattcccaagaggaatcgttctattctcaacacaagccaaaggggaattcttgaggatgaacagatgtaccagaagtgctgcaatctattcgagaagtttttccctagcagttcatataggagaccggttggaatttctagcatggtggaggccatggtgtctagggcccggattgatgccagggtcgactttgagtctggacggatcaagaaagaagagttctctgagatcatgaagatctgttccaccattgaagagctcagacggcaaaaataa

>H1N1_PB1_JX309357_A/Singapore/GP1112/2010

atggatgtcaatccgactctacttttcctaaaaattccagcgcaaaatgccataagcaccacattcccttatactggagatcctccatacagccatggaacaggaacaggatacaccatggacacagtaaacagaacacaccaatactcagaaaagggaaagtggacgacaaacacagagactggtgcaccccagctcaacccgattgatggaccactacctgaggataatgaaccaagtgggtatgcacaaacagactgtgttctagaggctatggctttccttgaagaatcccacccaggaatatttgagaattcatgccttgaaacaatggaagttgttcaacaaacaagggtagataaattaactcaaggtcgccagacttatgattggacattaaacagaaatcaaccggcagcaactgcattggccaacaccatagaagtctttagatcgaatggcctaacagctaatgagtcaggaaggctaatagatttcttaaaggatgtaatggaatcaatgaacaaagaggaaatagagataacaacccactttcaaagaaaaaggagagtaagagacaacatgaccaagaagatggtcacgcaaagaacaatagggaagaaaaaacaaagactgaataagagaggctatctaataagagcactgacattaaatacgatgaccaaagatgcagagagaggcaagttaaaaagaagggctatcgcaacacctggaatgcagattagaggtttcgtatactttgttgaaactttagctaggagcatttgcgaaaagcttgaacagtctgggctcccagtagggggcaatgaaaagaaggccaaactggcaaatgttgtgagaaagatgatgactaattcacaagacacagagatttctttcacaatcactggggacaacactaagtggaatgaaaatcaaaatcctcgaatgttcctggcgatgattacatatatcaccagaaatcaacccgagtggttcagaaacatcctgagcatggcacccataatgttctcaaacaaaatggcaagactagggaaagggtacatgttcgagagtaaaagaatgaagattcgaacacaaataccagcagaaatgctagcaagcattgacctgaagtacttcaatgaatcaacaaagaagaaaattgagaaaataaggcctcttctaatagatggcacagcatcactgagtcctgggatgatgatgggcatgttcaacatgctaagtacggtcttgggagtctcaatactgaatcttggacaaaagaaatacaccaagacaatatactggtgggatgggctccaatcatccgacgattttgctctcatagtgaatgcaccaaaccatgagggaatacaagcaggagtggacagattctacaggacctgcaagttagtgggaatcaacatgagcaaaaagaagtcctatataaataagacagggacatttgaattcacaagctttttttatcgctatggatttgtggctaattttagcatggagctacccagctttggagtgtctggagtaaatgaatcagctgacatgagtattggagtaacagtgataaagaacaacatgataaacaatgaccttggacctgcaacggcccagatggctcttcaattgttcatcaaagactacagatacacatataggtgccataggggagacacacaaattcagacgagaagatcatttgagttaaagaagctgtgggatcaaacccaatcaaaggtagggctattagtatcagatggaggaccaaacttatacaatatacggaatcttcacatccctgaagtctgcttaaaatgggagctaatggatgatgattaccggggaagactttgtaatcccctgaatccctttgtcagtcataaagagattgattctgtaaacaatgctgtggtaatgccagcccatggtccagtcaaaagcatggaatatgatgccgttgcaactacacattcctggattcccaagaggaatcgttctattctcaacacaagccaaaggggaattcttgaggatgaacagatgtaccagaggtgctgcaatctattcgagaaatttttccctagcagttcatataggagaccggttggaatttctagcatggtggaggccatggtgtctagggcccggattgatgccagggtcgacttcgagtctggacggatcaagaaagaagagttctctgagatcatgaagatctgttccaccattgaagaactcagacggcaaaaataa

>H1N1_PB1_JX309474_A/Singapore/TT255/2010

atggatgtcaatccgactctacttttcctaaaaattccagcgcaaaatgccataagcaccacattcccttatactggagatcctccatacagccatggaacaggaacaggatacaccatggacacagtaaacagaacacaccaatactcagaaaagggaaagtggacgacaaacacagagactggtgcaccccagctcaacccgattgatggaccactacctgaggataatgaaccaagtgggtatgcacaaacagactgtgttctagaggctatggctttccttgaagaatcccacccaggaatatttgagaattcatgccttgaaacaatggaagttgttcaacaaacaagggtagataaactaactcaaggtcgccagacttatgattggacattaaacagaaatcaaccggcagcaactgcattggccaacaccatagaagtctttagatcgaatggcctaacagctaatgagtcaggaaggctaatagatttcttaaaggatgtaatggaatcaatgaacaaagaggaaatagagataacaacccactttcaaagaaaaaggagagtaagagacaacatgaccaagaagatggtcacgcaaagaacaatagggaagaaaaaacaaagactgaataagagaggctatctaataagagcactgacattaaatacgatgaccaaagatgcagagagaggcaagttaaaaagaagggctatcgcaacacctgggatgcagattagaggtttcgtatactttgttgaagctttagctaggagcatttgcgaaaagcttgaacagtctgggctcccagtaggaggcaatgaaaagaaggccaaactggcaaatgttgtgagaaagatgatgactaattcacaagacacagagatttctttcacaatcactggggacaacactaagtggaatgaaaatcaaaatcctcgaatgttcctggcaatgattacatatatcaccagaaatcaacccgagtggttcagaaacatcctgagcatggcacccataatgttctcaaacaaaatggcaagactagggaaagggtacatgttcgagagtaaaagaatgaagattcgaacacaaataccagcagaaatgctagcaagcattgacctgaagtacttcaatgaatcaacgaagaagaaaattgagaaaataaggcctcttctaatagatggcacagcatcactgagtcctgggatgatgatgggcatgttcaacatgctaagtacggtcttgggagtctcgatactgaatcttggacaaaagaaatacaccaagacaatatactggtgggatgggctccaatcatccgacgattttgctctcatagtgaatgcaccaaaccatgagggaatacaagcaggagtggacagattctacaggacctgcaagttagtgggaatcaacatgagcaaaaagaagtcctatataaataagacagggacatttgaattcacaagctttttttatcgctatggatttgtggctaattttagcatggagctacccagctttggagtgtctggagtaaatgaatcagctgacatgagtattggagtaacagtgataaagaacaacatgataaacaatgaccttggacctgcaacggcccagatggctcttcaattgttcatcaaagactacagatacacatataggtgccataggggagacacacaaattcagacgagaagatcatttgagttaaagaagctgtgggatcaaacccaatcaaaggtagggctattagtatcagatggaggaccaaacttatacaatatacggaatcttcacattcctgaagtctgcttaaaatgggagctaatggatgatgattatcggggaagactttgtaatcccctgaatccctttgtcagtcataaagagattgattctgtaaacaatgctgtggtaatgccagcccatggtccagtcaaaagcatggaatatgatgccgttgcaactacacattcctggattcccaagaggaatcgttctattctcaacacaagccaaaggggaattcttgaggatgaacagatgtaccagaagtgctgcaatctattcgagaaatttttccctagcagttcatataggagaccggttggaatttctagcatggtggaggccatggtgtctagggcccggattgatgccagggtcgacttcgagtctggacggatcaagaaagaagagttctctgagatcatgaagatctgctccaccattgaagaactcagacggcaaaaataa

>H1N1_PB1_JX309856_A/Singapore/GP4266/2010

atggatgtcaatccgactctacttttcctgaaaattccagcgcaaaatgccataagcaccacattcccttatactggagatcctccatacagccatggaacaggaacaggatacaccatggacacagtaaacagaacacaccaatactcagaaaagggaaagtggacgacaaacacagagactggagcaccccagctcaacccgattgatggaccactacctgaggataatgaaccaagtgggtatgcacaaacagactgtgttctagaggctatggctttccttgaagaatcccacccaggaatatttgagaattcatgccttgaaacaatggaagttgttcaacaaacaagggtagataaactaactcaaggtcgccagacttatgattggacattaaacagaaatcaaccggcagcaactgcattggccaacaccatagaagtctttagatcgaatggcctaacagctaatgagtcaggaaggctaatagatttcttaaaggatgtaatggaatcaatgaacaaagaggaaatagagataacaacccactttcaaagaaaaaggagagtaagagacaacatgaccaagaagatggtcacgcaaagaacaatagggaagaaaaaacaaaaactgaataagagaggctatctaataagagcactgacattaaatacgatgaccaaagatgcagagagaggcaagctaaaaagaagggctatcgcaacacctgggatgcagattagaggtttcgtatactttgttgaaactttagctaggagcatctgcgaaaagcttgaacagtctgggctcccagtagggggcaatgaaaagaaggccaaactggcaaatgttgtgagaaagatgatgactaattcacaagacacagagatttctttcacaatcactggggacaacactaagtggaatgaaaatcaaaatcctcgaatgtttctggcgatgattacatatatcaccagaaatcaacccgagtggttcagaaacatcctgagcatggcacccataatgttctcaaacaaaatggcaagactagggaaagggtacatgttcgaaagtaaaagaatgaagattcgaacacaaataccagcagaaatgctagcaagcattgacctgaagtacttcaatgaatcaacaaagaagaaaattgagaaaataaggcctcttctaatagatggcacagcatcactgagtcctgggatgatgatgggcatgttcaacatgctaagtacggtcttgggagtctcgatactgaatcttggacaaaagaaatacaccaagacagtatactggtgggatgggctccaatcatccgacgattttgctctcatagtgaatgcaccaaaccatgagggaatacaagcaggagtggacagattctacaggacctgcaagttagtgggaatcaacatgagcaaaaagaagtcctatataaataagacagggacatttgaattcacaagctttttttatcgctatggatttgtggctaattttagcatggagctacccagctttggagtgtctggagtaaatgaatcagctgacatgagtattggagtaacagtgataaagaataacatgataaacaatgaccttggacctgcaacggcccagatggctcttcaattgttcatcaaagactacagatacacatataggtgccataggggagacacacaaattcagacgagaagatcatttgagttaaagaagctgtgggatcaaacccaatcaaaggtagggctattagtatcagatggaggaccaaacttatacaatatacggaatcttcacattcctgaagtctgcttaaaatgggagctaatggatgatgattatcggggaagactttgtaatcccctgaatccctttgtcagtcataaagagattgattctgtaaacaatgctgtggtaatgccagcccatggtccagtcaaaagcatggaatatgatgccgttgcaactacacattcctggattcccaagaggaatcgttctattctcaacacaagccaaaggggaattcttgaggatgaacagatgtaccagaagtgctgcaatctattcgagaaatttttccctagcagttcatataggagaccggttggaatttctagcatggtggaggccatggtgtctagggcccggattgatgccagggtcgacttcgagtctggacggatcaagaaagaagagttctctgagatcatgaagatctgttccaccattgaagaactcagacggcaaaaataa

>H1N1_PB1_JX625668_A/England/139/2010

atggatgtcaatccgactctgcttttcctgaaaattccagcgcaaaatgccataagcaccacattcccttatactggagatcctccatacagccatggaacaggaacaggatacaccatggacacagtaaacagaacacaccaatactcagaaaagggaaagtggacgacaaacacagagactggtgcaccccagctcaacccgattgatggaccactacctgaggataatgaaccaagtgggtatgcacaaacagactgtgttctagaggctatggctttccttgaagaatcccacccaggaatatttgagaattcatgccttgaaacaatggaagttgttcaacaaacaagggtagataaactaactcaaggtcgccagacttatgattggacattaaacagaaatcagccggcagcaactgcattggccaacaccatagaagtctttagatcgaatggcctaacagctaatgagtcaggaaggctaatagatttcttaaaggatgtaatggaatcaatgaacaaagaggaaatagagataacaacccactttcaaagaaaaaggagagtaagagacaacatgaccaagaagatggtcacgcaaagaacaatagggaagaaaaaacaaaaactgaataagagaggctatctaataagagcactgacattaaatacgatgaccaaagatgcagagagaggcaagttaaaaagaagggctatcgcaacacctgggatgcagattagaggtttcgtatactttgttgaaactttagctaggagcatttgcgaaaagcttgaacagtctgggctcccagtagggggcaatgaaaagaaggccaaactggcaaatgttgtgagaaagatgatgactaattcacaagacacagagatttctttcacaatcactggggacaacactaagtggaatgaaaatcaaaatcctcgaatgttcctggcgatgattacatatatcaccagaaatcaacccgagtggttcagaaacatcctgagcatggcacccataatgttctcaaacaaaatggcaagactagggaaagggtacatgttcgagagtaaaagaatgaagattcgaacacaaataccagcagaaatgctagcaagcattgacctgaagtacttcaatgaatcaacaaagaagaaaattgagaaaataaggcctcttctaatagatggcacagcatcactgagtcctgggatgatgatgggcatgttcaacatgctaagtacggtcttgggagtctcgatactgaatcttggacaaaagaaatacaccaagacaatatactggtgggatgggctccaatcatccgacgattttgctctcatagtgaatgcaccaaaccatgagggaatacaagcaggagtggacagattctacaggacctgcaagttagtgggaatcaacatgagcaaaaagaagtcctatataaataagacagggacatttgaattcacaagctttttttatcgctatggatttgtggctaattttagcatggagctacccagctttggagtgtctggagtaaatgaatcagctgacatgagtattggagtaacagtgataaagaacaacatgataaacaatgaccttggacctgcaacggcccagatggctcttcaattgttcatcaaagactacagatacacatataggtgccataggggagacacacaaattcagacgagaagatcatttgagttaaagaagctgtgggatcaaacccaatcaaaggtagggctattagtatcagatggaggaccaaacttatacaatatacggaatcttcacattcctgaagtctgcttaaaatgggagctgatggatgatgattatcggggaagactttgtaatcccctgaatccctttgtcagtcataaagagattgattctgtaaacaatgctgtggtaatgccagcccatggtccagtcaaaagcatggaatatgatgccgttgcaactacacattcctggattcccaagaggaatcgttctattctcaacacaagccaaaggggaattcttgaggatgaacagatgtatcagaagtgctgcaatctattcgagaaatttttccctagcagttcatataggagaccggttggaatttctagcatggtggaggccatggtgtctagggcccggattgatgccagggtcgacttcgagtctggacggatcaagaaagaagagttctctgagatcatgaagatctgttccaccattgaagaactcagacggcaaaaataa

>H1N1_PB1_CY128209_A/Viet Nam/13032035/2009

atggatgtcaatccgactctacttttcctaaaaattccagcgcaaaatgccataagcaccacattcccttatactggagatcctccatacagccatggaacaggaacaggatacaccatggacacagtaaacagaacacaccaatactcagaaaagggaaagtggacgacaaacacagagactggtgcaccccagctcaacccgattgatggaccactacctgaggataatgaaccaagtgggtatgcacaaacagactgtgttctagaggctatggctttccttgaagaatcccacccaggaatatttgagaattcatgccttgaaacaatggaagttgttcaacaaacaagggtagataaactaactcaaggtcgccagacttatgattggacattaaacagaaatcaaccggcagcaactgcattggccaacaccatagaagtctttagatcgaatggcctaacagctaatgagtcaggaaggctaatagatttcttaaaggatgtaatggaatcaatgaacaaagaggaaatagagataacaacccactttcaaagaaaaaggagagtaagagacaacatgaccaagaagatggtcacgcaaagaacaatagggaagaaaaaacaaagactgaataagagaggctatctaataagagcactgacattaaatacgatgaccaaagatgcagagagaggcaagttaaaaagaagggctatcgcaacacctgggatgcagattagaggtttcgtatactttgttgaaactttagctaggagcatttgcgaaaagcttgaacagtctgggctcccagtagggggcaatgaaaagaaggccaaactggcaaatgttgtgagaaagatgatgactaattcacaagacacagagatttctttcacaatcactggggacaacactaagtggaatgaaaatcaaaatcctcgaatgttcctggcgatgattacatatatcaccagaaatcaacccgagtggttcagaaacatcctgagcatggcacccataatgttctcaaacaaaatggcaagactagggaaagggtacatgttcgagagtaaaagaatgaagattcgaacacaaataccagcagaaatgctagcaagcattgacctgaagtacttcaatgaatcaacaaagaagaaaattgagaaaataaggcctcttctaatagatggcacagcatcactgagtcctgggatgatgatgggcatgttcaacatgctaagtacggtcttgggagtctcgatactgaatcttggacaaaagaaatacaccaagacagtatactggtgggatgggctccaatcatccgacgattttgctctcatagtgaatgcaccaaaccatgagggaatacaagcaggagtggacagattctacaggacctgcaagttagtgggaatcaacatgagcaaaaagaagtcctatataaataaaacagggacatttgaattcacaagctttttttatcgctatggatttgtggctaattttagcatggagctacccagctttggagtgtctggagtaaatgaatcagctgacatgagtattggagtaacagtgataaagaacaacatgataaacaatgaccttggacctgcaacggcccagatggctcttcaattgttcatcaaagactacagatacacatataggtgccataggggagacacacaaattcagacgagaagatcatttgagttaaagaagctgtgggatcaaacccaatcaaaggtagggctattagtatcagatggaggaccaaacttatacaatatacggaatcttcacattcctgaagtctgcttaaaatgggagctaatggatgatgattatcggggaagactttgtaatcccctgaatccctttgtcagtcataaagagattgattctgtaaacaatgctgtggtaatgccagcccatggtccagccaaaagcatggaatatgatgccgttgcaactacacattcctggattcccaagaggaatcgttctattctcaacacaagccaaaggggaattcttgaggatgaacagatgtaccagaagtgctgcaatctattcgagaaatttttccctagcagttcatataggagaccggttggaatttctagcatggtagaggccatggtgtctagggcccggattgatgccagggtcgacttcgagtctggacggatcaagaaagaagagttctctgagatcatgaagatctgttccaccattgaagaactcagacggcaaaaataa

>H1N1_PB1_CY100536_A/Mexico City/INER11/2010

atggatgtcaatccgactctacttttcctaaaaattccagcgcaaaatgccataagcaccacattcccttatactggagatcctccatacagccatggaacaggaacaggatacaccatggacacagtaaacagaacacaccaatactcagaaaagggaaagtggacgacaaacacagagactggtgcaccccagctcaacccgattgatggaccactacctgaggataatgaaccaagtgggtatgcacaaacagactgtgttctagaggctatggctttccttgaagaatcccacccaggaatatttgagaattcatgccttgaaacaatggaagttgttcaacaaacaagggtagataaactaactcaaggtcgccagacttatgattggacattaaacagaaatcaaccggcagcaactgcattggccaacaccatagaagtctttagatcaaatggcctaacagctaatgagtcaggaaggctaatagatttcttaaaggatgtaatggaatcaatgaacaaagaggaaatagagataacaacccactttcaaagaaaaaggagagtaagagacaacatgaccaagaagatggtcacgcaaagaacaatagggaagaaaaaacaaagactgaataagagaggctatctaataagagcactgacattaaatacgatgaccaaagatgcagagagaggcaagttaaaaagaagggctatcgcaacacctgggatgcagattagaggtttcgtatactttgttgaaactttagctaggagcatttgcgaaaagcttgaacagtctgggctcccagtagggggcaatgaaaagaaggccaaactggcaaatgttgtgagaaagatgatgactaattcacaagacacagagatttctttcacaatcactggggacaacactaagtggaatgaaaatcaaaatcctcgaatgttcctggcgatgattacatacatcaccagaaatcaacccgagtggttcagaaacatcctgagcatggcacccataatgttctcaaacaaaatggcaagactagggaaagggtacatgttcgagagtaaaagaatgaagattcgaacacaaataccagcagaaatgctagcaagcattgacctgaagtacttcaatgaatcaacaaagaagaaaattgagaaaataaggcctcttctaatagatggcacagcatcactgagtcctgggatgatgatgggcatgttcaacatgctaagtactgtcttgggagtctcgatactgaatcttggacaaaagaaatacaccaagacaatatactggtgggatgggctccaatcatccgacgattttgctctcatagtgaatgcaccaaaccatgagggaatamaagcaggagtggacagattctacaggacctgcaagttagtgggaatcaacatgagcaaaaagaagtcctatataaataagacagggacatttgaattcacaagctttttttatcgctatggatttgtggctaattttagcatggagctacccagctttggagtgtctggagtaaatgaatcagctgacatgagtattggagtaacagtgataaagaacaacatgataaacaatgaccttggacctgcaacggcccagatggcacttcaattgttcatcaaagactacagatacacatataggtgccataggggagacacacaaattcagacgagaagatcatttgagttaaagaagctgtgggatcaaacccaatcaaagggagggctattagtatcagatggaggaccaaacttatacaatatacggaatcttcacattcctgaagtctgcttaaaatgggagctaatggatgatgattatcggggaagactttgtaatcccctgaatccctttgtcagtcataaagagattgattctgtaaacaatgctgtggtaatgccagcccatggtccagtcaaaagcatggaatatgatgccgttgcaactacacattcctggattcccaagaggaatcgttctattctcaacacaagccaaaggggaattcttgaggatgaacagatgtaccagaagtgctgcaatctattcgagaaatttttccctagcagttcatataggagaccggttggaatttctagcatggtggaggccatggtgtctagggcccggattgatgccagggtcgacttcgaatctggacggatcaagaaagaagagttctctgagatcatgaagatctgctccaccattgaagaactcagacggcaaaaataa

>H1N1_PB1_KC780921_A/Kansas/19/2009

atggatgtcaatccgactctacttttcctaaaaattccagcgcaaaatgccataagcaccacattcccttatactggagatcctccatacagccatggaacaggaacaggatacaccatggacacagtaaacagaacacaccaatactcagaaaagggaaagtggacgacaaacacagagactggtgcaccccagctcaacccgattgatggaccactacctgaggataatgaaccaagtgggtatgcacaaacagactgtgttctagaggctatggctttccttgaagaatcccacccaggaatatttgagaattcatgccttgaaacaatggaagttgttcaacaaacaagggtagataaactaactcaaggtcgccagacttatgattggacattaaacagaaatcaaccggcagcaactgcattggccaacaccatagaagtctttagatcgaatggcctaacagctaatgagtcaggaaggctaatagatttcttaaaggatgtaatggaatcaatgaacaaagaggaaatagagataacaacccactttcaaagaaaaaggagagtaagagacaacatgaccaagaagatggtcacgcaaagaacaatagggaagaaaaaacaaagactgaataagagaggctatctaataagagcactgacattaaatacgatgaccaaagatgcagagagaggcaagttaaaaagaagggctatcgcaacacctgggatgcagattagaggtttcgtatactttgttgaaactttagctaggagcatttgcgaaaagcttgaacagtctgggctcccagtagggggcaatgaaaagaaggccaaactggcaaatgttgtgagaaagatgatgactaattcacaagacacagagatttctttcacaatcactggggacaacactaagtggaatgaaaatcaaaatcctcgaatgttcctggcgatgattacatatatcaccagaaatcaacccgagtggttcagaaacatcctgagcatggcacccataatgttctcaaacaaaatggcaagactagggaaagggtacatgttcgagagtaaaagaatgaagattcgaacacaaataccagcagaaatgctagcaagcattgacctgaagtacttcaatgaatcaacaaagaagaaaattgagaaaataaggcctcttctaatagatggtacagcatcactgagtcctgggatgatgatgggcatgttcaacatgctaagtacggtcttgggagtctcgatactgaatcttggacaaaagaaatacaccaagacaatatattggtgggatgggctccaatcatccgacgattttgctctcatagtgaatgcaccaaaccatgagggaatacaagcaggagtggacagattctacaggacctgcaagttagtgggaatcaacatgagcaaaaagaagtcctatataaataagacagggacatttgaattcacaagctttttttatcgctatggatttgtggctaattttagcatggagctacccagctttggagtgtctggagtaaatgaatcagctgacatgagtattggagtaacagtgataaagaacaacatgataaacaatgaccttggacctgcaacggcccagatggctcttcaattgttcatcaaagactacagatacacatataggtgccataggggggacacacaaattcaaacgagaagatcatctgagttaaagaagctgtgggatcaaacccaatcaaaggtagggctattagtatcagatggaggaccaaacttatacaatatacggaatcttcacattcctgaagtctgcttaaaatgggagctaatggatgatgattatcggggaagactttgtaatcccctgaatccctttgtcagtcataaagagattgattctgtaaacaatgctgtggtaatgccagcccatggtccagccaaaagcatggaatatgatgccgttgcaactacacattcctggattcccaagaggaatcgttctattctcaacacaagccaaaggggaattcttgaggatgaacagatgtaccagaagtgctgcaatctattcgagaaatttttccctagcagttcatataggagaccggttggaatttctagcatggtggaggccatggtgtctagggcccggattgatgccagggtcgacttcgaatctggacggatcaagaaagaagagttctctgagatcatgaagatctgttccaccattgaagaactcagacggcaaaaataa

>H1N1_PB1_KC881878_A/Arizona/01/2011

atggatgtcaatccgactctacttttcctaaaaattccagcgcaaaatgccataagcaccacattcccttatactggagatcctccatacagccatggaacgggaacaggatacaccatggacacagtaaacagaacacaccaatactcagaaaagggaaagtggacgacaaacacagagactggtgcaccccagctcaacccgattgatggaccactacctgaggataatgaaccaagtgggtatgcacaaacagactgtgttctagaggctatggctttccttgaagaatcccacccaggaatatttgagaattcatgccttgaaacaatggaagttgttcaacaaacaagggtagataaactaactcaaggtcgccagacttatgattggacattaaacagaaatcaaccggcagcaactgcattggccaacaccatagaagtctttagatcgaatggcctaacagctaatgagtcaggaaggctaatagatttcttaaaggatgtaatggaatcaatgaacaaagaggaaatagagataacaacccactttcaaagaaaaaggagagtaagagacaacatgaccaagaagatggtcacgcaaagaacaatagggaagaaaaaacaaagactgaataagagaggctatctaataagagcactgacattaaatacgatgaccaaagatgcagagagaggcaagttaaaaagaagggctatcgcaacacctgggatgcagattagaggtttcgtatactttgttgaaactttagctaggagcatttgcgaaaagcttgaacagtctgggctcccagtagggggcaatgaaaagaaggccaaactggcaaatgttgtgagaaagatgatgactaattcacaagacacagagatttctttcacaatcactggagacaacactaagtggaatgaaaatcaaaatcctcgaatgttcctggcgatgattacatatatcaccagaaatcaacccgagtggttcagaaacatcctgagcatggcacccataatgttctcaaacaaaatggcaagactagggaaagggtacatgttcgagagtaaaagaatgaagattcgaacacaaataccagcagaaatgctagcaaacattgacctgaagtacttcaatgaatcaacaaagaagaaaattgagaaaataaggcctcttttaatagatggcacagcatcactgagtcctgggatgatgatgggcatgttcaacatgctaagtacggtcttgggagtctcgatactgaatcttggacaaaagaaatacaccaagacaatatactggtgggatgggctccaatcatccgacgattttgctctcatagtgaatgcaccaaaccatgagggaatacaagcaggagtggacagattctacaggacctgcaagttagtgggaatcaacatgagcaaaaagaagtcctatataaataagacagggacatttgaattcacaagctttttttatcgctatggatttgtggctaattttagcatggagctacccagctttggagtgtctggagtaaatgaatcagctgacatgagtattggagtaacagtgataaagaacaacatgataaacaatgaccttggacctgcaacggcccagatggctcttcaattgttcatcaaagactacagatacacatataggtgccataggggagacacacaaattcagacgagaagatcatttgagttaaagaagctgtgggatcaaacccaatcaaaggtagggctattagtatcagatggaggaccaaatttatacaatatacggaatcttcacattcctgaagtctgcttaaaatgggagctaatggatgatgattatcggggaagactttgtaatcccctgaatccctttgtaagtcataaagagattgattctgtaaacaatgctgtggtaatgccagcccatggtccagccaaaagcatggagtatgatgccgttgcaactacacattcctggattcccaagaggaatcgttctattctcaacacaagccaaaggggaattcttgaggatgaacagatgtaccagaagtgctgcaatctattcgagaaatttttccctagcagttcatataggagaccggttggaatttctagcatggtggaggccatggtgtctagggcccggattgatgccagagtcgactttgagtctggacggatcaagaaagaagagttctctgagatcatgaagatctgttccaccattgaagaactcagacggcaaaaataa

>H1N1_PB1_CY147761_A/Mexico/24030/2009

atggatgtcaatccgactctacttttcctaaaaattccagcgcaaaatgccataagcaccacattcccttatactggagatcctccatacagccatggaacaggaacaggatacaccatggacacagtaaacagaacacaccaatactcagaaaagggaaagtggacgacaaacacagagactggtgcaccccagctcaacccgattgatggaccactacctgaggataatgaaccaagtgggtatgcacaaacagactgtgttctagaggctatggctttccttgaagaatcccacccaggaatatttgagaattcatgccttgaaacaatggaagttgttcaacaaacaagggtagataaactaactcaaggtcgccagacttatgattggacattaaacagaaatcaaccggcagcaactgcattggccaacaccatagaagtctttagatcgaatggcctaacagctaatgagtcaggaaggctaatagatttcttaaaggatgtaatggaatcaatgaacaaagaggaaatagagataacaacccactttcaaagaaaaaggagagtaagagacaacatgaccaagaagatggtcacgcaaagaacaatagggaagaaaaaacaaagactgaataagagaggctatctaataagagcactgacattaaatacgatgaccaaagatgcagagagaggcaagttaaaaagaagggctatcgcaacacctgggatgcagattagaggtttcgtatactttgttgaaactttagctaggagcatttgcgaaaagcttgaacagtctgggctcccagtagggggcaatgaaaagaaggccaaactggcaaatgttgtgagaaagatgatgactaattcacaagacacagagatttctttcacaatcactggggacaacactaagtggaatgaaaatcaaaatcctcgaatgttcctggcgatgattacatatatcaccagaaatcaacccgagtggttcagaaacatcctgagcatggcacccataatgttctcaaacaaaatggcaagactagggaaagggtacatgttcgagagtaaaagaatgaagattcgaacacaaataccagcagaaatgctagcaagcattgacctgaagtacttcaatgaatcaacaaagaagaaaattgagaaaataaggcctcttctaatagatggcacagcatcactgagtcctgggatgatgatgggcatgttcaacatgctaagtacggtcttgggagtctcgatactgaatcttggacaaaagaaatacaccaagacaatatactggtgggatgggctccaatcatccgacgattttgctctcatagtgaatgcaccaaaccatgagggaatacaagcaggagtggacagattctacaggacctgcaagttagtgggaatcaacatgagcaaaaagaagtcctatataaataagacagggacatttgaattcacaagctttttttatcgctatggatttgtggctaattttagcatggagctacccagctttggagtgtctggagtaaatgaatcagctgacatgagtattggagtaacagtgataaagaacaacatgataaacaatgaccttggacctgcaacggcccagatggctcttcaattgttcatcaaagactacagatacacatataggtgccataggggagacacacaaattcagacgagaagatcatttgagttaaagaagctgtgggatcaaacccaatcaaaggtagggctattagtatcagatggaggaccaaacttatacaatatacggaatcttcacattcctgaagtctgcttaaaatgggagctaatggatgatgattatcggggaagactttgtaatcccctgaatccctttgtcagtcataaagagattgattctgtaaacaatgctgtggtaatgccagcccatggtccagccaaaagcatggaatatgatgccgttgcaactacacattcctggattcccaagaggaatcgttctattctcaacacaagccaaaggggaattcttgaggatgaacagatgtaccagaagtgctgcaatctattcgagaaatttttccctagcagttcatataggagaccggttggaatttctagcatggtggaggccatggtgtctagggcccggattgatgccagggtcgacttcgagtctggacggatcaagaaagaagagttctctgagatcatgaagatctgttccaccattgaagaactcagacggcaaaaataa

>H1N1_PB1_KF411318_A/Qingdao/1020/2009

atggatgtcaatccgactctacttttcctaaaaattccagcgcaaaatgccataagcaccacattcccttatactggagatcctccatacagccatggaacaggaacaggatacaccatggacacagtaaacagaacacaccaatactcagaaaagggaaagtggacgacaaacacagagactggtgcaccccagctcaacccgattgatggaccactacctgaggataatgaaccaagtgggtatgcacaaacagactgtgttctagaggctatggctttccttgaagaatcccacccaggaatatttgagaattcatgccttgaaacaatggaagttgttcaacaaacaagggtagataaactaactcaaggtcgccagacttatgattggacattaaacagaaatcaaccggcagcaactgcattggccaacaccatagaagtctttagatcgaatggcctaacagctaatgagtcaggaaggctaatagatttcttaaaagatgtaatggaatcaatgaacaaagaggaaatagagataacaacccactttcaaagaaaaaggagagtaagagacaacatgaccaagaagatggtcacgcaaagaacaatagggaagaaaaaacaaagactgaataagagaggctatctaataagagcactgacattaaatacgatgaccaaagatgcagagagaggcaagttaaaaagaagggctatcgcaacacctgggatgcagattagaggtttcgtatactttgttgaaactttagctaggagcatttgcgaaaagcttgaacagtctgggctcccagtagggggcaatgaaaagaaggccaaactggcaaatgttgtgagaaagatgatgactaattcacaagacacagagatttctttcacaatcactggggacaacactaagtggaatgaaaatcaaaatcctcgaatgttcctggcgatgattacatatatcaccagaaatcaacccgagtggttcagaaacatcctgagcatggcacccataatgttctcaaacaaaatggcaagactagggaaagggtacatgttcgagagtaaaagaatgaagattcgaacacaaataccagcagaaatgctagcaagcattgacctgaagtacttcaatgaatcaacaaagaagaaaattgagaaaataaggcctcttctaatagatggcacagcatcactgagtcctgggatgatgatgggcatgttcaacatgctaagtacggtcttgggagtctcgatactgaatcttggacaaaagaaatacaccaagacaatatactggtgggatgggctccaatcatccgacgattttgctctcatagtgaatgcaccaaaccatgagggaatacaagcaggagtggacaaattctacaggacctgcaagttagtgggaatcaacatgagcaaaaagaagtcctatataaataagacagggacatttgaattcacaagctttttttatcgctatggatttgtggctaattttagcatggagctacccagctttggagtgtctggagtaaatgaatcagctgacatgagtattggagtaacagtgataaagaacaacatgataaacaatgaccttggacctgcaacggcccagatggctcttcaattgttcatcaaagactacagatacacatataggtgccataggggagacacacaaattcagacgagaagatcatttgagttaaagaagctgtgggatcaaacccaatcaaaggtagggttattagtatcagatggaggcccaaacttatacaatatacggaatcttcacattcctgaagtctgcttaaaatgggaggtaatggatgatgattatcggggaagactttgtaatcccctgaatccctttgtcagtcataaagagattgattctgtaaacaatgctgtggtaatgccagcccatggtccagccaaaagcatggaatatgatgccgttgcatctacacattcctggattcccaagaggaatcgttctattctcaacacaagccaaaggggaattcttgaggatgaacagatgtaccagaagtgctgcaatctattcgagaaatttttccctagcagttcatataggagaccggttggaatttctagcatggtggaggccatggtgtctagggcccggattgatgccagggtcgacttcgagtctggacggatcaaaaaagaagagttctctgagatcatgaagatctgttccaccattgaagaactcagacggcaaaaataa

>H1N1_PB1_KF559468_A/Helsinki/279/2013

atggatgtcaatccgactctacttttcctaaaaattccagcacaaaatgctataagcaccacattcccttatactggagatcctccatacagccatggaacaggaacaggatacaccatggacacagtaaacagaacacaccaatactcagaaaagggaaagtggacaacaaacacagagactggtgcaccccagctcaacccgattgatggaccactacctgaggataatgaaccaagtgggtatgcacaaacagactgtgttctagaggctatggctttccttgaagaatcccacccaggaatatttgagaattcatgccttgaaacaatggaagttgttcaacaaacaagggtagataaactgactcaaggtcgccagacttatgattggacattaaacagaaatcaaccggcagcaactgcattggccaacaccatagaagtctttagatcgaatgacctaacagctaacgagtcaggaaggctaatagatttcttaaaggatgtaatggaatcaatgaacaaagaggaaatagagataacaacccactttcaaagaaaaaggagagtaagagacaacatgaccaagaagatggtcacgcaaagaacaatagggaagaaaaaacagagactgaataagagaggctatctaataagagcactgacattaaatacgatgaccaaagatgcagagagaggcaagttaaaaagaagggctatcgcaacacctgggatgcagattagaggtttcgtatactttgttgaaactttagctaggagcatttgcgaaaagcttgaacagtctggactcccagtagggggcaatgaaaagaaggccaaactggcaaatgttgtgagaaagatgatgactaattcacaagatacagagatttctttcacaatcactggggacaacactaagtggaatgaaaatcaaaatcctcgaatgttcctggcgatgattacatatatcaccagaaatcaacccgagtggtttagaaacatcctgagcatggcacccataatgttctcaaataaaatggcaagactagggaaagggtacatgttcgagagtaaaagaatgaagattcgaacacaaataccagcagaaatgctagcaagcattgatctgaagtacttcaatgaatcaacaaagaagaaaattgagaaaataaggcctcttttaatggatggcacagcatcactgagtcctgggatgatgatgggcatgttcaacatgctaagtacggtcttgggagtctcgatactgaatcttggacaaaagaaatacaccaagacaacatactggtgggatgggctccaatcatccgacgattttgctctcatagtgaatgcaccaaaccatgagggaatacaagcaggagtggacagattctacaggacctgcaagttagtgggaatcaacatgagcaaaaagaagtcctatataaataagacagggacatttgaattcacaagctttttttatcgctatggatttgtggctaattttagcatggagctacccagctttggagtgtctggagtaaatgaatcagctgacatgagtattggagtaacagtgataaagaacaacatgataaacaatgaccttggacctgcaacggcccagatggctcttcaattgttcatcaaagactacagatacacatataggtgccataggggagacacacaaattcagacgagaagatcatttgagttaaagaagctgtgggatcaaacccaatcaaaggtagggctattagtatcagatggaggaccaaacttatacaatatacggaatcttcacattcctgaagtctgcttaaaatgggagctaatggatgatgattatcggggaagactttgtaatcccctgaatccctttgtaagtcataaggagattgattctgtaaacaatgctgtggtaatgccagcccatggtccagccaaaagcatggaatatgatgccgttgcaactacacattcctggattcccaagaggaaccgttctattctcaacacaagccaaaggggaattcttgaggatgaacagatgtaccagaagtgttgcaatctattcgagaaatttttccctagcagttcatataggagaccggttggaatttctagcatggtggaggccatggtatctagggcccggattgatgccagggtcgactttgagtctgggcggatcaagaaagaagagttctctgagatcatgaagatctgttccaccattgaagaactcagacggcaaaaataa

>H1N1_PB1_KF791399_A/Czech Republic/196/2013

atggatgtcaatccgactctacttttcctaaaaattccagcacaaaatgccataagcaccacattcccttatactggagatcctccatacagccatggaacaggaacaggatacaccatggacacagtaaacagaacacaccaatactcagaaaagggaaagtggacaacaaacacagagactggtgcaccccagctcaacccgattgatggaccactacctgaggataatgaaccaagtgggtatgcacaaacagactgtgttctagaggctatggctttccttgaagaatcccacccaggaatatttgagaattcatgccttgaaacaatggaagttgttcaacaaacaagggtagataaactgactcaaggtcgccagacttatgattggacattaaacagaaatcaaccggcagcaactgcattggccaacaccatagaagtctttagatcgaatgacctaacagctaacgagtcaggaaggctaatagatttcttaaaggatgtaatggaatcaatgaacaaagaggaaatagagataacaacccactttcaaagaaaaaggagagtaagagacaacatgaccaagaagatggtcacgcaaagaacaatagggaagaaaaaacagagactgaataagagaggctatctaataagagcactgacattaaatacgatgaccaaagatgcagagagaggcaagttaaaaagaagggctatcgcaacacctgggatgcagattagaggtttcgtatactttgttgaaactttagctaggagcatttgcgaaaagcttgaacagtctggactcccagtagggggcaatgaaaagaaggccaaactggcaaatgttgtgagaaagatgatgactaattcacaagatacagagatttctttcacaatcactggggacaacactaagtggaatgaaaatcaaaatcctcgaatgttcctggcgatgattacatatatcaccagaaatcaacccgagtggtttagaaacatcctgagcatggcacccataatgttctcaaataaaatggcaagactagggaaagggtacatgttcgagagtaaaagaatgaagattcgaacacaaataccagcagaaatgctagcaagcattgatctgaagtacttcaatgaatcaacaaagaagaaaattgagaaaataaggcctcttttaatggatggcacagcatcactgagtcctgggatgatgatgggcatgttcaacatgctaagtacggtcttgggagtctcgatactgaatcttggacaaaagaaatacaccaagacaacatactggtgggatggrctccaatcmtccgacgattttgctctcatagtgaatgcaccaaaccatgagggaatacaagcaggagtggacagattctacaggacctgcaagttagtgggaatcaacatgagcaaaaagaagtcctatataaataagacagggacatttgaattcacaagctttttttatcgctatggatttgtggctaattttagcatggagctacccagctttggagtgtctggagtaaatgaatcagctgacatgagtattggagtaacagtgataaagaacaacatgataaacaatgaccttggacctgcaacggcccagatggctcttcaattgttcatcaaagactacaggtacacatataggtgccataggggagacacacaaattcagacgagaagatcatttgagttaaagaagctgtgggatcaaacccaatcaaaggtagggctattagtatcagatggaggaccaaacttatacaatatacggaatcttcacattcctgaagtctgcttaaaatgggagctaatggatgatgattatcggggaagactttgtaatcccctgaatccctttgtaagtcataaggagattgattctgtaaacaatgctgtggtaatgccagcccatggtccagccaaaagcatggaatatgatgcagttgcaactacacattcctggattcccaagaggaatcgttctattctcaacacaagccaaaggggaattcttgaggatgaacagatgtaccagaagtgttgcaatctattcgagaaatttttccctagcagttcatataggagaccggttggaatttctagcatggtggaggccatggtatctagggcccggattgatgccagggtcgactttgagtctggacggatcaagaaagaagagttctctgagatcatgaagatctgttccaccattgaagaactcagacggcaaaaataa

>H1N1_PB1_CY173429_A/New York/1175/2009

atggatgtcaatccgacattacttttcttaaaagtgccagcacaaaatgctataagcacaacttttccttatactggtgaccctccttacagccatgggacaggaacagggtacaccatggatacagtcaataggacacaccagtactcagaaagaggaagatggacaaaaaataccgaaacgggagcaccgcaacttaacccaattgatggccccttaccggaagacaatgaaccaagtggctatgcccaaacagattgtgtattagaagcaatggctttccttgaagaatcccatcccggtatctttgaaaactcttgtattgaaacaatggaggttgttcaacaaacaagggtggacaaactgacacaaggcagacagacctatgactggactctaaataggaaccagcctgctgccacagcattggcaaacactatagaagtattcagatcaaacggcctcatagcaaatgaatctggaaggctaatagacttccttaaagatgtaatggagtcgatggacagaggcgaagtagaggtcacaactcattttcaaagaaagaggagagtgagagacaatgtaactaaaaaaatggtgacccaaagaacaataggcaaaaagaaacataaattagacaaaagaagttacctaattagggcattaaccctgaacacaatgaccaaagatgctgagagggggaaactaaaacgcagagcaattgcaaccccaggaatgcaaataagggggtttgtatactttgttgagacactggcaagaagcatatgtgaaaagcttgaacaatcaggattgccagttggaggaaatgagaagaaagcaaagttagcaaatgttgtaaggaagatgatgaccaactcccaggacactgaaatttctttcaccataaccggagacaacacaaaatggaacgaaaatcaaaaccctagaatgttcttggccatgatcacatatataaccaaaaatcagcctgaatggttcagaaatattctaagtattgctccaataatgttttcaaacaagatggcgagactaggtaaggggtacatgtttgaaagcaagagtatgaaactgagaactcaaatacctgcagagatgctagccaacatagacttgaaatatttcaatgattcaactaaaaagaaaattgaaaaaatccgaccattattaatagatggaactgcatcattgagtcctggaatgatgatgggcatgttcaatatgttgagcaccgtcctgggcgtctccattctgaatcttgggcaaaagagatacaccaagactacttactggtgggatggtcttcaatcgtctgatgattttgctttgattgtgaacgcacccaactatgcaggaattcaagctggagttgacaggttctatcgaacctgtaagctactcggaattaatatgagcaaaaagaagtcttacataaacagaacaggtacctttgaattcacgagctttttctatcgttatgggtttgttgccaatttcagcatggagcttcctagttttggggtgtctggggtcaatgaatctgcagacatgagtattggagtcactgtcatcaaaaacaatatgataaacaatgaccttggcccagcaactgctcaaatggcccttcagttatttataaaagattacaggtacacttatcgatgccaccgaggtgacacacaaatacaaacccgaagatcatttgagataaagaaactatgggaccaaacccgctcaaaagctggactgttggtctctgatggaggccccaatttgtataacattagaaatctccatattcctgaagtttgcttgaaatgggagttgatggatgaggattaccaggggcgtttatgcaacccattaaacccgtttgtcagccataaagagattgaatcagtgaacagtgcagtgataatgccggcacatggtccagccaaaaatatggagtatgacgctgttgcaacaacacactcctgggtccccaaaagaaatcgatccattttgaacacgagccaaagggggatacttgaagatgagcaaatgtatcagaggtgctgcaatttatttgaaaaattcttcccaagtagctcatacagaagaccagttggaatatccagtatggtagaggctatggtctcaagagcccgaattgatgcacggattgatttcgaatctggaaggataaagaaagaggaatttgctgagatcatgaagatctgttccaccattgaagacctcagacggcaaaaatga

>H1N1_PB1_CY168541_A/Boston/YGA_01002/2012

atggatgtcaatccgactctacttttcctaaaaattccagcacaaaatgccataagcaccacattcccttatactggagatcctccatacagccatggaacaggaacaggatacaccatggacacagtaaacagaacacaccaatactcagaaaagggaaagtggacaacaaacacagagactggtgcaccccagctcaacccgattgatggaccactacctgaggataatgaaccaagtgggtatgcacaaacagattgtgttctagaggctatggctttccttgaagaatcccacccaggaatatttgagaattcatgccttgaaacaatggaagttgttcaacaaacaagggtagataaactgactcaaggtcgccagacttatgattggacattaaacagaaatcaaccggcagcaactgcattggccaacaccatagaagtctttagatcgaatgacctaacagctaacgagtcaggaaggctaatagatttcttaaaggatgtaatggaatcaatgaacaaagaggaaatagagataacaacccactttcaaagaaaaaggagagtaagagacaacatgaccaagaagatggtcacgcaaagaacaatagggaagaaaaaacagagactggataagagaggctatctaataagagcactgacattaaatacgatgaccaaagatgcagagagaggcaagttaaaaagaagggctatcgcaacacctgggatgcagattagaggtttcgtatactttgttgaaactttagctaggagcatttgcgaaaagcttgaacagtctggactcccagtagggggcaatgaaaagaaggccaaactggcaaatgttgtgagaaagatgatgactaattcacaagatacagagatttctttcacaatcactggggacaacactaagtggaatgaaaatcaaaatcctcgaatgttcctggcgatgattacatatatcaccagaaatcaacccgagtggtttagaaacatcctgagcatggcacccataatgttctcaaataaaatggcaagactagggaaagggtacatgttcgagagtaaaagaatgaagattcgaacacaaataccagcagaaatgctagcaagcattgatctgaggtacttcaatgaatcaacaaagaaaaaaattgagaaaataaggcctcttttaatggatggcacagcatcactgagtcctgggatgatgatgggcatgttcaacatgctaagtacggtcttgggagtctcgatactgaatcttggacaaaagaaatacaccaagacaacatactggtgggatgggctccaatcatccgacgattttgctctcatagtgaatgcaccaaaccatgagggaatacaagcaggagtggacagattctacaggacctgcaagttagtgggaatcaacatgagcaaaaagaagtcctatataaataagacagggacatttgaattcacaagctttttttatcgctatggatttgtggctaattttagcatggagctacccagctttggagtgtctggagtaaatgaatcagctgacatgagtattggagtaacagtgataaagaacaacatgataaacaatgaccttggacctgcaacggcccagatggctcttcaattgttcatcaaagactacagatacacatataggtgccataggggagacacacaaattcagacgagaagatcatttgagttaaagaagctgtgggatcaaacccaatcaaaggtagggctattagtatcagatggaggaccaaacttatacaatatacggaatcttcacattcctgaagtctgcttaaaatgggagctaatggatgatgattatcggggaagactttgtaatcccctgaatccctttgtaagtcataaggagattgattctgtaaacaatgctgtggtaatgccagcccatggtccagccaaaagcatggaatatgatgccgttgcaactacacattcctggattcccaagaggaatcgttctattctcaacacaagccaaaggggaattcttgaggatgaacagatgtaccagaagtgttgcaatctattcgagaaatttttccctagcagttcatataggagaccggttggaatttctagcatggtggaggccatggtatctagggcccggattgatgccagggtcgactttgagtctggacggatcaagaaagaagagttctctgagatcatgaagatctgttccaccattgaagaactcagacggcaaaaataa

>H1N1_PB1_CY178725_A/Wisconsin/301/1976

atggatgtcaatccgactttgcttttcctgaaagtgccagcacaaaatgctataagcacaacgtttccttacactggagaccccccttacagccatgggacaggaacaggatacaccatggatactgtgaacagaacacatcagtactcagaaaaaggaaaatggacaataaacaccgaaactggggcaccacaactaaatccaattgatggaccgctgcccgaggacaatgaaccaactggttatgcccaaacagactgcgtcttggaagcaatggcgttcctcgagaaatcccatcccggcatttttgaaaactcatgtcttgaaacaatggaagttattcagcaaacacgggtggacaaactaacgcaaggccggcaaactttcgattggactctgaataggaaccagcctgctgcaacagcattggccaacacaatagaagtgttcagattaaacggcctcacggccagtgaatctggaagactcatagacttcctaaaggatgttatggagtcaatggacaaagaagaaatggaaatcgtaactcatttccagagaaaaaggagggtgagggacaatatgactaagaaaatggtgacgcaaagaacaataggtaaaaagaaacagaaattaactaaaagaagttatctaattagagcattgaccttgaatacaatgacaaaagatgctgaaagaggaaaattgaaaagaagagcaattgccaccccagggatgcaaatacggggattcgtatatttcgttgagacactagcgagaagtatttgtgaaaaacttgagcaatcaggactgccagttggaggcaatgaaaagaaagccaagttggcaaacgttgtacgaaaaatgatgaccaattctcaggacactgaactctcctttaccatcactggagataatacaaaatggaatgaaaatcagaatcctcgaatgtttttagctatgattacatacataaccagaaatcagcccgaatggttcagaaatgttctaagtgttgctccaataatgttctcgaacaagatggctagattgggtaaaggatacatgtttgagagtaaaaacatgaagcttaggactcagatacctgcagagatgctatcaggcattgacctgaggtattttaatgattcaactaggaaaaaaattgaaaaaatccgaccgctcttaatagagggagctgcgtctttaagccctggaatgatgatgggtatgtttaacatgttaagcactgtgttgggtgtttccattcttaaccttggacaaaaggagtacacgaagactgcttactggtgggacggtctccaatcgtctgatgattttgctctgattgtgaacgcacccaatcatgaagggattcaagcaggggttgacaggttttatcgaacttgtaagctactcggaattaatatgagcaaaaagaaatcttacataaatagaacaggcacctttgaattcacaagctttttctatcgttatggatttgttgccaatttcagcatggagcttcctagttttggagtatctggaattaatgagtctgcagacatgagtattggagttactgtcatcaaaaacaacatgataaacaatgatcttggaccagcaacagcacaaatggctctccagttattcatcaaagattaccgatacacgtatcgctgccacaggggtgatactcaaatacaaactcgaaggtcatttgaaataaggaaattgtgggatcaaactcgttctaaggctggattgttgatttccgatggaggccccaatttatataatattaggaatctccacattcctgaagtctgcttgaagtgggaattaatggataaagattaccaggggcgattgtgcaatccgctaaacccatttgtcagccataaagagattgaatcagtgaataatgcagtagtaatgccatcacatggtccagccaaaaccatggaatacgacgctgttgccacaacgcactcctgggttcctaaaagaaatcgatccatcttgaacacaagccagagaggaatacttgaagacgagcaaatgtaccaaaagtgctgtaacctatttgaaaagttcttccctagcagctcatataggagaccagttggaatttccagtatggtggaggccatggtttccagggcccgaatcgatgcacgaattgattttgaatctggaagaataaagaaagaggatttcgctgagatcatgaagatctgttccaccattgaagatctcagacggcaaaagtag

>H1N1_PB1_KJ856229_A/Sri Lanka/24206/2009

atggatgtcaatccgactctacttttcctaaaaattccagcgcaaaatgccataagcaccacattcccttatactggagatcctccatacagccatggaacaggaacaggatacaccatggacacagtaaacagaacacaccaatactcagaaaagggaaagtggacgacaaacacagagactggtgcaccccagctcaacccgattgatggaccactacctgaggataatgaaccaagtgggtatgcacaaacagactgtgttctagaggctatggctttccttgaagaatcccacccaggaatatttgagaattcatgccttgaaacaatggaagttgttcaacaaacaagggtagataaactaactcaaggtcgccagacttatgattggacattaaacagaaatcaaccggcagcaactgcattggccaacaccatagaagtttttagatcgaatggcctaacagctaatgagtcaggaaggctaatagatttcttaaaggatgtaatggaatcaatgaacaaagaggaaatagagataacaacccactttcaaagaaaaaggagggtaagagacaacatgaccaagaagatggtcacgcaaagaacaatagggaagaaaaaacaaagactgaataagagaggctatctaataagagcactgacattaaatacgatgaccaaagatgcagagagaggcaagttaaaaagaagggctatcgcaacacctgggatgcagattagaggtttcgtatactttgttgaaactttagctaggagcatttgcgaaaagcttgaacagtctgggctcccagtagggggcaatgaaaagaaggccaaactggcaaatgttgtgagaaagatgatgactaattcacaagacacagagatttctttcacaatcactggggacaacactaagtggaatgaaaatcaaaatcctcgaatgttcctggcgatgattacatatatcaccagaaatcaacccgagtggttcagaaacatcctgagcatggcacccataatgttctcaaacaaaatggcaagactagggaaagggtacatgttcgagagtaaaagaatgaagattcgaacacaaataccagcagaaatgctagcaagcattgacctgaagtacttcaatgaatcaacaaagaagaaaattgagaaaataaggcctcttctaatagatggcacagcatcactgagtcctgggatgatgatgggcatgttcaacatgctaagtacggtcttgggagtctcgatactgaatcttggacaaaagaaatacaccaagacaatatactggtgggatgggctccaatcatccgacgattttgccctcatagtgaatgcaccaaaccatgagggaatacaagcaggagtggacagattctacaggacctgcaagttagtgggaatcaacatgagcaaaaagaagtcctatataaataagacagggacatttgaattcacaagctttttttatcgctatggatttgtggctaattttagcatggagctacccagctttggagtgtctggagtaaatgaatcagctgacatgagtattggagtaacagtgataaagaacaacatgataaacaatgaccttggacctgcaacggcccagatggctcttcaattgttcatcaaagactacagatacacatataggtgccataggggagacacacaaattcagacgagaagatcatttgagttaaagaagctgtgggatcaaacccaatcaaaggtagggctattagtatcagatggaggaccaaacttatacaatatacggaatcttcacattcctgaagtctgcttaaaatgggagctaatggatgatgattatcggggaagactttgtaatcccctgaatccctttgtcagtcataaagagattgattctgtaaacaatgctgtggtaatgccagcccatggtccagccaaaagcatggaatatgatgccgttgcaactacacattcctggattcccaagaggaatcgttctattctcaacacaagccaaaggggaattcttgaggatgaacagatgtaccagaagtgctgcaatctattcgagaaatttttccctagcagttcatataggagaccggttggaatttctagcatggtggaggccatggtgtctagggcccggattgatgccagggtcgacttcgagtctggacggatcaagaaagaagagttctctgagatcatgaagatctgttccaccattgaagaactcagacggcaaaaataa

>H1N1_PB1_KJ856158_A/Sri Lanka/21406/2010

atggatgtcaatccgactctacttttcctaaaaattccagcgcaaaatgccataagcaccacattcccttatactggagatcctccatacagccatggaacaggaacagggtacaccatggacacagtaaacagaacacaccaatactcagaaaagggaaagtggacgacaaacacagagactggtgcaccccagctcaacccgattgatggaccactacctgaggataatgaaccaagtgggtatgcacaaacagactgtgttctagaggctatggctttccttgaagaatcccacccaggaatatttgagaattcatgccttgaaacaatggaagctgttcaacaaacaagggtagataaactaactcaaggtcgccagacttatgattggacattaaacagaaatcaaccggcagcaactgcattggccaacaccatagaagtctttagatcgaatggcctaacagctaatgagtcaggaaggctaatagatttcttaaaggatgtaatggaatcactgaacaaagaggaaatagagataacaacccactttcaaagaaaaaggagagtaagagacaacatgaccaagaagatggtcacgcaaagaacaatagggaagaaaaaacaaagactgaataagagaggctatctaataagagcactgacattaaatacgatgaccaaagatgcagagagaggcaagttaaaaagaagggctatcgcaacacctgggatgcagattagaggtttcgtatactttgttgaaactttagctaggagcatttgcggaaagcttgaacagtctgggctcccagtagggggcaatgaaaagaaggccaaactggcaaatgttgtgagaaagatgatgactaattcacaagacacagagatttctttcacaatcactggggacaacactaagtggaatgaaaatcaaaatcctcgaatgttcctggcgatgattacatatatcaccagaaatcaacccgagtggttcagaaacatccttagcatggcacccataatgttctcaaacaaaatggcaagactagggaaagggtacatgttcgagagtaaaagaatgaagattcgaacacaaattccagcagacatgctagcaagcattgacctgaagtacttcaatgaatcaacaaagaaaaaaattgagaaaataaggcctcttctaatagatggcacagcatcactgagtcctgggatgatgatgggcatgttcaacatgctaagtacagtcttgggagtctcgatactgaatcttggacaaaagaaatacaccaagacaacatactggtgggatgggctccaatcatccgacgattttgctctcatagtgaatgcaccaaaccatgagggaatacaagcaggagtggacagattctacaggacctgcaagttagtgggaatcaacatgagcaaaaagaagtcatatataaataaaacagggacatttaaattcacaagctttttttatcgctatggatttgtggctaattttagcatggagctacccagctttggagtgtctggagtaaatgaatcagctgacatgagtattggagtaacagtgataaagaacaacatgataaacaatgaccttggacctgcaacggcccagatggctcttcaattgttcatcaaagactacagatacacatataggtgccataggggagacacacaaattcagacgagaagatcatttgagttaaaaaagctgtgggatcaaacccaatcaaaggtagggctattagtatcagatggaggaccaaacttatacaatatacggaatcttcacattcctgaagtctgcataaaatgggagctaatggatgatgattatcggggaagactttgtaatcccctgaatccctttgtcagtcataaagagattgattctgtaaacaatgctgtggtaatgccagcccatggcccagccaaaagcatggaatatgatgccgttgcaactacacattcctggattcccaagaggaatcgttctattctcaacacaagccaaaggggaattcttgaggatgaacagatgtaccagaagtgctgcaatctattcgagaaatttttccctagcagttcatataggagaccggttggaatttctagcatggtggaggccatggtgtctagggcccggattgatgccagggtcgacttcgagtctggacggatcaagaaagaagagttctctgagatcatgaagatctgttccaccattgaagaactcagacggcaaaaataa

>H1N1_PB1_CY188479_A/Nicaragua/6819_12/2013

atggatgtcaatccgaccctacttttcctaaaaattccagcacaaaatgccataagcaccacattcccttatactggagatcctccatacagccatggaacaggaacaggatacaccatggacacagtaaacagaacacaccaatactcagaaaggggaaagtggacaacaaacacagagactggtgcaccccagctcaacccgattgatggaccactacctgaggataatgaaccaagtgggtatgcacaaacagactgtgttctagaagctatggctttccttgaagaatcccacccaggaatatttgagaattcatgccttgaaacaatggaagttgttcaacaaacaagggtagataaactgactcaaggtcgccagacttatgattggacattaaacagaaatcaaccagcagcaactgcattagccaacaccatagaagtctttagatcgaatgacctaacagctaacgagtcaggaaggctaatagatttcttaaaagatgtaatggaatcaatgaacaaagaggaaatagagataacaacccactttcaaagaaaaaggagagtaagagacaacatgaccaagaagatggtcacgcaaagaacaatagggaagaaaaaacagagactgaataagagaggctatctaataagagcactgacattaaatacgatgaccaaagatgcagagagaggcaagttaaaaagaagggctatcgcaacacctgggatgcagattagaggtttcgtatactttgttgaaactttagctaggagcatttgcgaaaagcttgaacagtctgggctcccagtagggggcaatgaaaagaaggccaaattggcaaatgttgtgagaaagatgatgactaattcacaagatacagagatttctttcacaatcactggggacaacactaagtggaatgaaaatcaaaatcctcgaatgttcctggcgatgattacatatatcaccagaaatcaacccgagtggtttagaaacatcctgagcatggcacccataatgttctcaaataaaatggcaagactagggaaagggtacatgttcgagagtaaaagaatgaagattcgaacacaaataccagcagaaatgctagcaagcattgatctgaagtacttcaatgaatctacaaagaagaaaattgagaaaataaggcctcttttaatggatggcacagcatcactgagtcctgggatgatgatgggaatgttcaacatgctaagtacggtcttgggagtctcgatactgaatcttggacaaaagaaatacaccaagacaacatactggtgggatgggctccaatcatccgacgattttgctctcatagtgaatgcaccaaaccacgagggaatacaagcaggagtggacagattctacaggacctgcaaattagtgggaatcaacatgagcaaaaagaagtcctatataaataagacagggacatttgaattcacaagctttttttatcgctatggatttgtagctaattttagcatggagctacccagttttggagtgtctggagtaaatgaatcagctgacatgagtattggagtaacagtgataaagaacaacatgataaacaatgaccttggacctgcaacggcccagatggctcttcaattgttcatcaaagactacagatacacatataggtgccataggggagacacacaaattcagacgagaagatcatttgagttaaagaagctgtgggatcaaacccaatcaaaggtagggctattagtatcagatggaggaccaaacttatacaatatacggaatcttcacattcctgaagtctgcttaaaatgggagctaatggatgatgattatcggggaagactttgtaatcccctgaatccctttgtgagtcataaggagattgactctgtaaacaatgctgtggtaatgccagcccatggtccagccaaaagcatggaatatgatgccgttgcaactacacattcctggattcccaagaggaatcgttctattctcaacacaagccaaaggggaattcttgaggatgaacagatgtaccagaagtgttgcaatctattcgagaaatttttccctagcagttcatataggagaccggttggaatttctagcatggtggaggccatggtgtctagggcccggattgatgccagggtcgactttgaatctggacggatcaagaaagaagagttctctgagatcatgaagatctgttccaccattgaagaactcagacggcaaaaataa

>H1N1_PB1_CY189135_A/New York/WC-LVD-14-011/2014

atggatgtcaatccgactctacttttcctaaaaattccagcacaaaatgccataagcaccacattcccttatactggagatcctccatacagccatggaacaggaacaggatacaccatggacacagtaaacagaacacaccaatactcagaaaaggggaagtggacaacaaacacagagactggtgcaccccagctcaacccgattgatggaccactacctgaggataatgaaccaagtgggtatgcacaaacagactgtgttctagaggctatggctttccttgaagaatcccacccaggaatatttgagaattcatgccttgaaacaatggaaattgttcaacaaacaagggtagataaactgactcaaggtcgccagacttatgattggacattaaacagaaatcaaccggcagcaactgcattggccaacaccatagaagtctttagatcgaatgacctaacagctaacgagtcaggaaggctaatagatttcttaaaggatgtaatggaatcaatgaacaaagaggaaatagagataacaacccactttcaaagaaaaaggagagtaagagacaacatgaccaagaagatggtcactcaaagaacaatagggaagaaaaaacagagactgaataagagaggctatctaataagagcactgacattaaatacgatgaccaaagatgcagagagaggcaagttaaaaagaagggctatcgcaacacctgggatgcagattagaggtttcgtatactttgttgaaactttagctaggagcatttgcgaaaagcttgaacagtctgggctcccagtagggggcaatgaaaagaaggccaaattggcaaatgttgtgagaaagatgatgactaattcacaagatacagagatttctttcacaatcactggggacaacactaagtggaatgaaaatcaaaatcctcgaatgttcctggcgatgattacatatatcaccagaaatcaacccgagtggtttagaaacatcctgagcatggcacccataatgttctcaaataaaatggcaagactagggaaagggtacatgttcgagagtaaaagaatgaagattcgaacacaaataccagcagaaatgctagcaagcattgatctgaagtacttcaatgaatcaacaaagaagaaaattgagaaaataaggcctcttttaatggatggcacagcatcactgagtcctgggatgatgatgggcatgttcaacatgctaagtacggtcttgggagtctcgatactgaatcttggacaaaagaaatacaccaagacaacatactggtgggatgggctccaatcatccgacgattttgctctcatagtgaatgcaccaaaccatgaaggaatacaagcaggagtggacagattctacaggacctgcaagttagtgggaatcaacatgagcaaaaagaagtcctatataaataagacagggacatttgaattcacaagctttttttatcgctatggatttgtggctaattttagcatggagctacccagctttggagtgtctggagtaaatgaatcagctgacatgagtattggagtaacagtgataaagaacaacatgataaacaatgacctcggacctgcaacggcccagatggctcttcaattgttcatcaaagactacagatacacatataggtgccataggggagacacacaaattcagacgagaagatcatttgagttaaagaagctatgggatcaaacccaatcaaaggtagggctattagtatcagatggaggaccaaacttatacaacatacggaatcttcacattcctgaagtctgcttaaaatgggagctaatggatgatgattatcggggaagactttgtaatcccctaaatccctttgtgagtcataaggagattgattctgtaaacaatgctgtggtaatgccagcccatggtccagccaaaagcatggaatatgatgccgttgcaactacacattcctggattcccaagaggaatcgttctattctcaacacaagccaaaggggaattcttgaggatgaacagatgtaccagaagtgttgcaatctattcgagaaatttttccctagcagttcatataggagaccggttggaatttctagcatggtggaggccatggtgtctagggcccggattgatgccagggtcgactttgagtctggacggatcaagaaagaagagttctctgagatcatgaagatctgttccaccattgaagaactcagacggcaaaaataa

>H1N1_PB1_CY189375_A/New York/WC-LVD-14-041/2014

atggatgtcaatccgactctacttttcctaaaaattccagcacaaaatgccataagcatcacattcccttatactggagatcctccatacagccatggaacaggaacaggatacaccatggacacagtaaacagaacacaccaatactcagaaaaggggaagtggacaacaaacacagagactggtgcaccccagctcaacccgattgatggaccactacctgaggataatgaaccaagtgggtatgcacaaacagactgtgtcctagaggctatggctttccttgaagaatcccacccaggaatatttgagaattcatgccttgaaacaatggaagttgttcaacaaacaagggtagataaactgactcaaggtcgccagacttatgattggacattaaacagaaatcaaccggcagcaactgcattggccaacaccatagaagtctttagatcgaatgacctaacagctaacgagtcaggaaggctaatagatttcttaaaggatgtaatggaatcaatgaacaaagaggaaatagagataacaacccactttcaaagaaaaaggagagtaagagacaacatgaccaagaagatggtcacgcaaagaacaatagggaagaaaaaacagagactgaataagagaggctatctaataagagcactgacattaaatacgatgaccaaagatgcagagagaggcaagttaaaaagaagggctatcgcaacacctgggatgcagattagaggtttcgtatactttgttgaaactttagctaggagcatttgcgaaaagcttgaacagtctgggctcccagtagggggcaatgaaaagaaggccaaattggcaaatgttgtgagaaagatgatgactaattcacaagatacagagatttctttcacaatcactggggacaacactaaatggaatgaaaatcaaaatcctcgaatgttcctggcgatgattacatatatcaccagaaatcaacccgagtggtttagaaacatcctgagcatggcacccataatgttctcaaataaaatggcaagactggggaaagggtacatgttcgagagtaaaagaatgaagattcgaacacaaataccagcagaaatgctagcaagcattgatctgaagtacttcaatgaatcaacaaagaagaaaattgagaaaataagacctcttttaatggatggcacagcatcactgagtcctgggatgatgatgggcatgttcaacatgctaagtacggtcttgggagtctcgatactgaatcttggacaaaagaaatacaccaagacaacatactggtgggatgggctccaatcatccgacgattttgctctcatagtgaatgcaccaaaccacgaaggaatacaagcaggagtggacagattctacaggacctgcaagttagtgggaatcaacatgagcaaaaagaagtcctatataaataagacagggacatttgaattcacaagctttttttatcgctatggatttgtggctaattttagcatggagctacccagctttggagtgtctggagtaaatgaatcagctgacatgagtattggagtaacagtgataaagaacaacatgataaacaatgaccttggacctgcaacggcccagatggctcttcaattgttcatcaaagactacagatacacatataggtgccataggggagacacacaaattcagacgagaagatcatttgagttaaagaagctatgggatcaaacccaatcaaaggtagggctattagtatcagatggaggaccaaacttatacaacatacggaatcttcacattcctgaagtctgcttaaaatgggagctaatggatgatgattatcggggaagactttgtaatcccctgaatccctttgtgagtcataaggagattgattctgtaaacaatgccgtggtaatgccagcccatggtccagccaaaagcatggaatatgatgccgttgcaactacacattcctggattcccaagaggaatcgttctattctcaacacaagccaaaggggaattcttgaggatgaacagatgtaccagaagtgttgcaatctattcgagagatttttccctagcagttcatataggagaccggttggaatttctagcatggtggaggccatggtgtctagggcccggattgatgccagggtcgactttgagtctggacggatcaagaaagaagagttctctgagatcatgaagatctgttccaccattgaagaactcagacggcaaaaataa

>H1N1_PB1_KM366469_A/Helsinki/1351/2013

atggatgtcaatccgactctacttttcctaaaaattccagcacaaaatgccataagcaccacattcccttatactggagatcctccatacagccatggaacaggaacaggatacaccatggacacagtaaacagaacacaccaatactcagaaaagggaaagtggacaacaaacacagagactggtgcaccccagctcaacccgattgatggaccactacctgaggataatgaaccaagtgggtatgcacaaacagactgtgttctagaggctatggctttccttgaagaatcccacccaggaatatttgagaattcatgccttgaaacaatggaagttgttcaacaaacaagggtagataaactgactcaaggtcgccagacttatgattggacattaaacagaaatcaaccggcagcaactgcattggccaacaccatagaagtctttagatcgaatgacctaacagctaacgagtcaggaaggctaatagatttcttaaaggatgtaatggaatcaatgaacaaagaggaaatagagataacaacccactttcaaagaaaaagaagagtaagagacaacatgaccaagaagatggtcacgcaaagaacaatagggaagaaaaaacagagactgaataagagaggctatctaataagagcactgacattaaatacgatgaccaaagatgcagaaagaggcaagttaaaaagaagggctatcgcaacacctgggatgcagattagaggtttcgtatactttgttgaaactttagctaggagcatttgcgaaaagcttgaacagtctggactcccagtagggggcaatgaaaagaaggccaaattggcaaatgttgtgagaaagatgatgactaattcacaagatacagagatttctttcacaatcactggggacaacactaagtggaatgaaaatcaaaatcctcgaatgttcctggcgatgattacatatatcaccagaaatcaacccgagtggtttagaaacatcctgagcatggcacccataatgttctcaaataaaatggcaagactagggaaagggtacatgttcgagagtaaaagaatgaagattcgaacacaaataccagcagaaatgctagcaagcattgatctgaagtacttcaatgaatcaacaaagaagaaaattgagaaaataaggcctcttttaatggatggcacagcatcactgagtcctgggatgatgatgggcatgttcaacatgctaagtacggtcttgggagtctcgatactgaatcttggacaaaagaaatacaccaagacaacatactggtgggatgggctccaatcatccgacgattttgctctcatagtgaatgcaccaaaccatgagggaatacaagcaggagtggacagattctacaggacctgcaagttagtgggaatcaacatgagcaaaaagaagtcctatataaataagacagggacatttgaattcacaagctttttttatcgctatggatttgtggctaattttagcatggagctacccagctttggagtgtctggagtaaatgaatcagctgacatgagtattggagtaacagtgataaagaacaacatgataaacaatgaccttggacctgcaacggcccagatggctcttcaattgttcatcaaagactacagatacacatataggtgccataggggagacacacaaattcagacgagaagatcatttgagttaaagaagctgtgggatcaaacccaatcaaaggtagggctattagtatcagatggaggaccaaacttatacaatatacggaatcttcacattcctgaagtctgcttaaaatgggagctaatggatgatgattatcggggaagactttgtaatcccctgaatccctttgtaagtcataaggagattgattctgtaaacaatgctgtggtaatgccagctcatggtccagccaaaagcatggaatatgatgccgttgcaactacacattcctggattcccaagaggaatcgttctattctcaacacaagccaaaggggaattcttgaggatgaacagatgtaccagaagtgttgcaatctattcgagaaatttttccctagcagttcatataggagaccggttggaatttctagcatggtggaggccatggtatctagggcccggattgatgccagggtcgactttgagtctggacggatcaagaaagaagagttctcagagatcatgaagatctgttccaccattgaagaactcagacggcaaaaataa

>H1N1_PB1_KM366373_A/Helsinki/1076/2013

atggatgtcaatccgactctacttttcctaaaaattccagcacaaaatgccataagcaccacattcccttatactggagatcctccatacagccatggaacaggaacaggatacaccatggacacagtaaacagaacacaccaatactcagaaaagggaaagtggacaacaaacacagagactggtgcaccccagctcaacccgattgatggaccactacctgaggataatgaaccaagtgggtatgcacaaacagactgtgttctagaggctatggctttccttgaagaatcccacccaggaatatttgagaattcatgccttgaaacaatggaagttgttcaacaaacaagggtagataaactgactcaaggtcgccagacttatgattggacattaaacagaaatcaaccggcagcaactgcattggccaacaccatagaagtctttagatcgaatgacctaacagctaacgagtcaggaaggctaatagatttcttaaaggatgtaatggaatcaatgaacaaagaggaaatagagataacaacccactttcaaagaaaaagaagagtaagagacaacatgaccaagaagatggtcacgcaaagaacaatagggaagaaaaaacagagactgaataagagaggctatctaataagagcactgacattaaatacgatgaccaaagatgcagagagaggcaagttaaaaagaagggctatcgcaacacctgggatgcagattagaggtttcgtatactttgttgaaactttagctaggagcatttgcgaaaagcttgaacagtctggactcccagtagggggcaatgaaaagaaggccaaattggcaaatgttgtgagaaagatgatgactaattcacaagatacagagatttctttcacaatcactggggacaacactaagtggaatgaaaatcaaaatcctcgaatgttcctggcgatgattacatatatcaccagaaatcaacccgagtggtttagaaacatcctgagcatggcacccataatgttctcaaataaaatggcaagactagggaaagggtacatgttcgagagtaaaagaatgaagattcgaacacaaataccagcagaaatgctagcaagcattgatctgaagtacttcaatgaatcaacaaagaagaaaattgagaaaataaggcctcttttaatggatggcacagcatcactgagtcctgggatgatgatgggcatgttcaacatgctaagtacggtcttgggagtctcgatactgaatcttggacaaaagaaatacaccaagacaacatactggtgggatgggctccaatcatccgacgattttgctctcatagtgaatgcaccaaaccatgagggaatacaagcaggagtggacagattctacaggacctgcaagttagtgggaatcaacatgagcaaaaagaagtcatatataaataagacagggacatttgaattcacaagctttttttatcgctatggatttgtggctaattttagcatggagctacccagctttggagtgtctggagtaaatgaatcagctgacatgagtattggagtaacagtgataaagaacaacatgataaacaatgaccttggacctgcaacggcccagatggctcttcaattgttcatcaaagactacagatacacatataggtgccataggggagacacacaaattcagacgagaagatcgtttgagttaaagaagctgtgggatcaaacccaatcaaaggtagggctattagtatcagatggaggaccaaacttatacaatatacggaatcttcacattcctgaagtctgcttaaaatgggagctaatggatgatgattatcggggaagactttgtaatcccctgaatccctttgtaagtcataaggagattgattctgtaaacaatgctgtggtaatgccagcccatggtccagccaaaagcatggaatatgatgccgttgcaactacacattcctggattcccaagaggaatcgttctattctcaacacaagccaaaggggaattcttgaggatgaacagatgtaccagaagtgttgcaatctattcgagaaatttttccctagcagttcatataggagaccggttggaatttctagcatggtggaggccatggtatctagggcccggattgatgccagggtcgactttgagtctggacggatcaagaaagaagagttctcagagatcatgaagatctgttccaccattgaagaactcagacggcaaaaataa

>H1N1_PB1_KM437704_A/Finland/215/2012

atggatgtcaatccgactctacttttcctaaaaattccagcgcaaaatgccataagcatcacattcccttatactggagatcctccatacagccatggaacaggaacaggatacaccatggacacagtaaacagaacacaccaatactcagaaaagggaaagtggacgacaaacacagagactggtgcaccccagctcaacccgattgatggaccactacctgaggataacgaaccaagtgggtatgcacaaacagactgtgtcctagaggctatggctttccttgaagaatcccacccaggaatatttgagaattcatgccttgaaacaatggaagttgttcaacaaacaagagtagataaactgactcaaggtcgccagacttatgattggacattaaacagaaatcaaccggcagcaactgcattggccaacaccatagaagtctttagatcgaatggcctaacagctaatgagtcaggaaggctagtagatttcttaaaggatgtaatggaatcaatgaacaaagaggaaatagagataacaacccactttcaaagaaaaaggagagtaagagacaacatgaccaagaagatggtcacgcaaagaacaatagggaagaaaaaacagagactgaataagagaggctatctaataagagcactgacattaaatacgatgaccaaagatgcagagagaggcaagttaaaaagaagggctatcgcaacacctgggatgcagattagaggtttcgtatactttgttgaaactttagctaggagcatttgcgaaaagcttgaacaatctgggctcccagtggggggcaatgaaaagaaggccaaactggcaaatgttgtgagaaagatgatgactaattcacaagacacagagatttctttcacaatcactggggacaacactaagtggaatgaaaatcaaaatcctcgaatgttcctggcgatgattacatatatcaccaaaaatcaacccgagtggtttagaaacatcctgagcatggcacccataatgttctcaaacaaaatggcaagactagggaaagggtacatgttcgagagtaaaagaatgaagattcgaacacaaataccagcagaaatgctagcaagcattgacctgaagtacttcaatgaatcaacaaagaagaaaattgagaaaataaggcctcttttaatggatggcacagcatcactgagtcctgggatgatgatgggcatgttcaacatgctaagtacggtcttgggagtctcgatactgaatcttggacaaaagaaatacaccaagacaacatactggtgggatgggctccaatcatccgacgattttgctctcatagtgaatgcaccaaaccatgagggaatacaagcaggagtggacagattctacaggacctgcaagttagtgggaatcaacatgagcaaaaagaagtcctatataaataagacagggacatttgaattcacaagctttttttatcgctatggatttgtggctaattttagcatggagctacccagctttggagtgtctggagtaaatgaatcagctgacatgagtattggagtaacagtgataaagaacaacatgataaacaatgaccttggacctgcaacagcccagatggctcttcaattgttcatcaaagactacagatacacatatagatgccataggggagacacacaaattcagacgagaagatcatttgagttaaagaagctgtgggatcaaacccaatcaaaggtagggctattagtatcagatggaggaccaaacttatacaatatacggaatcttcacattcctgaagtctgcttaaaatgggagctaatggatgatgattatcggggaagactttgtaatcccctgaatccctttgtaagtcataaggagattgattctgtaaacaatgctgtggtaatgccagcccatggtccagccaaaagcatggaatatgatgccgttgcaactacacattcctggattcccaagaggaatcgttctattctcaacacaagccaaaggggaattcttgaggatgaacagatgtaccagaagtgctgcaatctattcgagaaatttttccctagcagttcatataggagaccggttggaatttctagcatggtggaggccatggtgtctagggcccggattgatgccagggtcgactttgagtctggacggatcaagaaagaagagttttctgagatcatgaagatctgttccaccattgaagaactcagacggcaaaaataa

>H1N1_PB1_KP222611_A/Singapore/495W/2009

atggatgtcaatccgactctacttttcctaaaaattccagcgcaaaatgccataagcaccacattcccttatactggagatcctccatacagccatggaacaggaacaggatacaccatggacacagtaaacaggacacaccaatactcagaaaggggaaagtggacgacaaacacagagactggtgcaccccagctcaacccgattgatggaccactacctgaggataatgaaccaagtgggtatgcacaaacagactgtgttctagaggctatggctttccttgaagaatcccacccaggaatatttgagaattcatgccttgaaacaatggaagttgttcaacaaacaagggtagataaactaactcaaggtcgccagacttatgattggacattaaacagaaatcaaccggcagcaactgcattggccaacaccatagaagtctttagatcgaatggcctaacagctaatgagtcaggaaggctaatagatttcttaaaggatgtaatggaatcaatgaacaaagaggaaatagagataacaacccactttcaaagaaaaaggagagtaagagacaacatgaccaagaagatggtcacgcaaagaacaatagggaagaaaaaacaaagactgaataagagaggctatctaataagagcactgacattaaatacgatgaccaaagatgcagagagaggcaagttaaaaagaagggctatcgcaacacctgggatgcagattagaggtttcgtatactttgttgaaactttagctaggagcatttgcgaaaagcttgaacagtctgggctcccagtggggggcaatgaaaagaaggccaaactggcaaatgttgtgagaaagatgatgactaattcacaagacacagagatttctttcacaatcactggggacaacactaagtggaatgaaaatcaaaatcctcgaatgttcctggcgatgattacatatatcaccagaaatcaacccgagtggttcagaaacatcctgagcatggcacccataatgttctcaaacaaaatggcaagactagggaaagggtacatgttcgagagtaaaagaatgaagattcgaacacaaataccagcagaaatgctagcaagcattgacctgaagtacttcaatgaatcaacaaagaagaaaattgagaaaataaggcctcttctaatagatggcacagcatcactgagtcctgggatgatgatgggcatgttcaacatgctaagtacggtcttgggagtctcgatactgaatcttggacaaaagaaatacaccaagacaatatactggtgggatgggctccaatcatccgacgattttgctctcatagtgaatgcaccaaaccatgagggaatacaagcaggagtggacagattctacaggacctgcaagttagtgggaatcaacatgagcaaaaagaagtcctatataaataagacagggacatttgaattcacaagctttttttatcgctatggatttgtggctaattttagcatggagctacccagctttggagtgtctggagtaaatgaatcagctgacatgagtattggagtaacagtgataaagaacaacatgataaacaatgaccttggacctgcaacggcccagatggctcttcaattgttcatcaaagactacagatacacatataggtgccataggggagacacacaaattcagacgagaagatcatttgagttaaagaagctgtgggatcaaacccaatcaaaggtagggctattagtatcagatggaggaccaaacttatacaatatacggaatcttcacattcctgaagtctgcttaaaatgggagctaatggatgatgattatcggggaagactttgtaatcccctgaatccctttgtcagtcataaagagattgattctgtaaacaatgctgtggtaatgccagcccatggtccagccaaaagcatggaatatgatgccgttgcaactacacattcctggattcccaagaggaatcgttctattctcaacacaagccaaaggggaattcttgaggatgaacagatgtaccagaagtgctgcaatctattcgagaaatttttccctagcagttcatataggagaccggttggaatttctagcatggtggaggccatggtgtctagggcccggattgatgccagggtcgacttcgagtctggacggatcaagaaagaagagttctctgagatcatgaagatctgttctaccattgaagaactcagacggcaaaaataa

>H1N1_PB1_KP637620_A/Thailand/SP07146/2008

atggatgtcaatccgacattacttttcttaaaagtgccagcacaaaatgctataagcacaacttttccttatactggtgaccctccttacagccatgggacaggaacagggtacaccatggatacagtcaacaggacacaccagtactcagaaagaggaagatggacaaaaaataccgaaacgggagcaccgcaacttaacccaattgatggtcccttaccggaagacaatgaaccaagtggctatgcccaaacagattgtgtattagaagcaatggctttccttgaagaatcccatcccggtatctttgaaaactcttgtattgaaacaatggaggttgttcaacaaacaagggtggacaaactgacacaaggcagacagacctatgactggactctaaataggaaccagcctgctgccacagcattggcaaacactatagaagtattcagatcaaacggcctcatagcaaatgaatctgggaggctaatagacttccttaaagatgtaatggagtcgatggacagaggcgaagtagaggtcacaactcattttcaaagaaagaggagagtgagagacaatgtaactaaaaaaatggtgacccaaagaacaataggcaaaaagaaacataaattagacaaaagaagttacctaattagggcattaaccctgaacacaatgaccaaagatgctgagagggggaaactaaaacgcagagcaattgcaaccccaggaatgcaaataagggggtttgtatactttgttgagacactggcaagaagcatatgtgaaaagcttgaacaatcaggattgccagttggaggaaatgagaagaaagcaaagttagcaaatgttgtaaggaagatgatgaccaactcccaggacactgaaatttctttcaccataaccggagataacacaaaatggaacgaaaatcaaaaccctagaatgttcttggccatgatcacatatataaccaaaaatcagcctgaatggttcagaaatattctaagtattgctccaataatgttttcaaacaagatggcgagactaggcaaggggtacatgtttgaaagcaagagtatgaaactgagaactcaaatacctgcagagatgctagccaacatagacttgaaatatttcaatgattcaactaaaaagaaaattgaaaaaatccgaccattattaatagatggaactgcatcattgagtcctggaatgatgatgggcatgttcaatatgttgagcaccgtcttgggcgtctccattctgaatcttgggcaaaagagatacaccaagactacttactggtgggatggtcttcaatcgtctgatgattttgctttgattgtgaacgcacccaactatgcaggaattcaagctggagttgacaggttttatcgaacctgtaagctgctcggaattaatatgagcaaaaagaagtcttacataaacagaacaggtacctttgaattcacgagctttttctatcgttatgggtttgttgccaatttcagcatggagcttcctagttttggggtgtctggggtcaatgaatctgcagacatgagtattggagtcactgtcatcaaaaacaatatgataaacaatgaccttggcccagcaactgctcaaatggcccttcagttatttatcaaagattacaggtacacttatcgatgccaccgaggtgacacacaaatacaaacccggagatcatttgagataaagaaactatgggaccaaacccgctcaaaagctggactgttggtctctgatggaggccccaatttgtataacattagaaatctccatattcctgaagtttgcttgaaatgggagttgatggatgaggattaccaggggcgtttatgcaacccattaaacccgtttgtcagccataaagagattgaatctgtgaacagtgcagtgataatgccggcacatggtccagccaaaaatatggagtatgacgctgttgcaacaacacactcctgggtccccaaaagaaatcgatccattttgaacacgagccaaagggggatacttgaagatgagcaaatgtatcagaggtgctgcaatttatttgaaaaattcttcccaagtagctcatacagaagaccagttggaatatccagtatggtagaggctatggtctcaagagcccgaattgatgcacggattgatttcgaatctggaaggataaagaaagaggaatttgctgagatcatgaagatctgttccaccattgaagacctcagacggcaaaaatga

>H1N1_PB1_KP637612_A/Thailand/SP07102/2007

atggatgtcaatccgacattacttttcttaaaagtgccagcacaaaatgctataagcacaactttcccttatactggtgaccctccttacagccatgggacaggaacagggtacaccatggatacagtcaacaggacacaccagtactcagaaagaggaagatggacaaaaaataccgaaactggagcaccgcaacttaacccaattgatggtccactaccggaagacaatgaaccaagtggctatgcccaaacagattgtgtattagaagcaatggctttccttgaagaatcccatcccggtatctttgaaaactcttgtattgaaacaatggaggttgttcagcaaacaagggtggacaaactgacacaaggcagacagacctatgactggactctaaataggaaccagcctgctgccacagcattggcaaacactatagaagtattcagatcaaacggcctcatagcaaatgaatctggaaggctaatagacttccttaaagatgtaatggagtcgatggacagaggcgaagtagaggtcacaactcattttcaaagaaagaggagagtgagagacaatgtaactaaaaaaatggtgacccaaagaacaataggcaaaaagaaacataaattagacaaaagaagttacctaattagggcattaaccctgaacacaatgaccaaagatgctgagagggggaaactaaaacgcagagcaattgcaaccccaggaatgcaaataagggggtttgtatactttgttgagacactggcaagaagcatatgtgaaaagcttgaacaatcaggattgccagttggaggaaatgagaagaaagcaaagttagcaaatgttgtaaggaagatgatgaccaactcccaggacactgaaatttctttcaccataaccggagataacacaaaatggaacgaaaatcaaaaccctagaatgttcttggccatgatcacatatataaccaaaaatcagcctgaatggttcagaaatattctaagtattgctccaataatgttttcaaacaagatggcgagactaggtaaggggtacatgtttgaaagcaagagtatgaaactgagaactcaaatacctgcagagatgctagccaacatagatttgaagtatttcaatgattcaactaaaaagaaaattgaaaaaatacggccattattaatagatggaactgcatcattgagtcctggaatgatgatgggcatgttcaatatgttgagcaccgtcttgggcgtctccattctgaatcttgggcaaaagagatacaccaagactacttactggtgggatggtcttcaatcgtctgatgattttgctttgattgtgaatgcacccaactatgcaggaattcaagctggagttgacaggttttatcgaacctgtaagctgctcggaattaatatgagcaaaaagaagtcttacataaacagaacaggtacctttgaattcacgagctttttctatcgttatgggtttgttgccaatttcagcatggagcttcctagttttggggtgtctggggtcaatgaatctgcagacatgagtattggagtcactgtcatcaaaaacaatatgataaacaatgaccttggcccagcaactgctcaaatggcccttcagttatttataaaagattacaggtacacttatcgatgccaccgaggtgacacacaaatacaaacccggagatcatttgagataaagaaactatgggaccaaacccgctcaaaagctgggctgttggtctctgatggaggccccaatttatataacattagaaatctccatattcctgaagtttgcttgaaatgggagttgatggatgaggattaccaggggcgtttatgcaacccattaaacccgtttgtcagccataaagagattgaatcagtgaacaatgcagtgataatgccggcacatggtccagccaaaaatatggagtatgacgctgttgcaacaacacactcctgggtccccaaaagaaatcgatccattttgaacacgagccaaagggggatacttgaagatgagcaaatgtatcagaggtgctgcaatttatttgaaaaattcttcccaagtagctcatacagaagaccagttggaatatccagtatggtagaggctatggtctcaagagcccgaattgatgcacggattgatttcgaatctggaaggataaagaaagaggaatttgctgagatcatgaagatctgttccaccattgaagacctcagacggcaaaaatga

>H1N1_PB1_LN867516_A/England/05140216/2010

atggatgtcaatccgactctacttttcctaaaaattccagcgcaaaatgccataagcaccacattcccttatactggagatcctccatacagccatggaacaggaacaggatacaccatggacacagtgaacagaacacaccaatactcagaaaagggaaagtggacgacaaacacagagactggtgcaccccagctcaacccgattgatggaccactacctgaggataatgaaccaagtgggtatgcacaaacagactgtgttctagaggctatggctttccttgaagaatcccacccaggaatatttgagaattcatgccttgaaacaatggaagttgttcaacaaacaagggtagataaactaactcaaggtcgccagacttatgattggacattaaacagaaatcaaccggcagcaactgcattggccaacaccatagaagtctttagatcgaatggcctaacagctaatgagtcaggcaggctaatagatttcttaaaggatgtaatggaatcaatgaacaaagaggaaatagagataacaacccactttcaaagaaaaaggagagtaagagacaacatgaccaagaagatggtcacgcaaagaacaatagggaagaaaaaacaaagactgaataagagaggctatctaataagagcactgacattaaatacgatgaccaaagatgcagagagaggcaagttaaaaagaagggctattgcaacacctgggatgcagattagaggtttcgtatactttgttgaaactttagctaggagcatttgcgaaaagcttgaacagtctggactcccagtagggggcaatgaaaagaaggccaaactggcaaatgttgtgagaaagatgatgactaattcacaagacacagagatttctttcacaatcactggggacaacactaagtggaatgaaaatcaaaatcctcgaatgttcctggcgatgattacatatatcaccagaaatcaacccgagtggttcagaaacatcctgagcatggcacccataatgttctcaaacaaaatggcaagactagggaaagggtacatgttcgagagtaaaagaatgaagattcgaacacaaataccagcagaaatgctagcaagcattgacctgaagtacttcaatgaatcaacaaagaagaaaattgagaaaataaggcctcttttaatagatggcacagcatcactgagtcctgggatgatgatgggcatgttcaacatgctaagtacggtcttgggagtctcgatactgaatcttggacaaaagaaatacaccaagacaatatactggtgggatgggctccaatcatccgacgattttgctctcatagtgaatgcaccaaaccatgagggaatacaagcaggagtggacagattctacaggacctgcaagttagtgggaatcaacatgagcaaaaagaagtcctatataaataagacagggacatttgaattcacaagctttttttatcgctatggatttgtggctaattttagcatggagctacccagctttggagtgtctggagtaaatgaatcagctgacatgagtattggagtaacagtgataaagaacaacatgataaacaatgaccttggacctgcaacggcccagatggctcttcaattgttcatcaaagactacagatacacatataggtgccataggggagacacacaaattcagacgagaagatcatttgagttaaagaagctgtgggatcaaacccaatcaaaggtagggctattagtatcagatggaggaccaaacttatacaatatacggaatcttcacattcctgaagtctgcttaaaatgggagctaatggatgatgattatcggggaagactttgtaatccccttaatccctttgtaagtcataaagagattgattctgtaaacaatgctgtggtaatgccagcccatggtccagccaaaagcatggaatatgatgccgttgcaactacacattcctggattcccaagaggaatcgttctattctcaacacaagccaaaggggaattcttgaggatgaacagatgtaccagaagtgctgcaatctattcgagaaatttttccctagcagttcatataggagaccggttggaatttctagcatggtggaggccatggtgtctagggcccggattgatgccagggtcgactttgagtctggacggatcaagaaagaagagttctctgagatcatgaagatctgttccaccattgaagaactcagacggcaaaaataa

>H1N1_PB1_LN867690_A/England/686/2010

atggatgtcaatccgactctacttttcctaaaaattccagcgcaaaatgccataagcaccacattcccttatactggagatcctccatacagccatggaacaggaacaggatacaccatggacacagtgaacagaacacaccaatactcagaaaagggaaagtggacgacaaacacagagactggtgcaccccagctcaacccgattgatggaccactacctgaggataatgaaccaagtgggtatgcacaaacagactgtgttctagaggctatggctttccttgaagaatcccacccagggatatttgagaattcatgccttgaaacaatggaagttgttcaacaaacaagggtagataaactaactcaaggtcgccagacttatgattggacattaaacagaaatcaaccggcagcaactgcattggccaacaccatagaagtctttagatcgaatggcctaacagctaatgagtcaggcaggctaatagatttcttaaaggatgtaatggaatcaatgaacaaagaggaaatagagataacaacccactttcaaagaaaaaggagagtaagagacaacatgaccaagaagatggtcacgcaaagaacaatagggaagaaaaaacaaagactgaataagagaggctatctaataagagcactgacattaaatacgatgaccaaagatgcagagagaggcaagttaaaaagaagggctatcgcaacacctgggatgcagattagaggtttcgtatactttgttgaaactttagctaggagcatttgcgaaaagcttgaacagtctgggctcccagtagggggcaatgaaaagaaggccaaactggcaaatgttgtgagaaagatgatgactaattcacaagacacagagatttctttcacaatcactggggacaacactaagtggaatgaaaatcaaaatcctcgaatgttcctggcaatgattacatatatcaccagaaatcaacccgaatggttcagaaacatcctgagcatggcacccataatgttctcaaacaaaatggcaagactagggaaagggtacatgttcgagagtaaaagaatgaagattcgaacacaaataccagcagaaatgctagcaagcattgacctgaagtacttcaatgaatcaacaaagaagaaaattgagaaaataaggcctcttttaatagatggcacagcatcactgagtcctgggatgatgatgggcatgttcaacatgctaagtacggtcttgggagtctcgatactgaatcttggacaaaagaaatacaccaagacaatatactggtgggatgggctccaatcatccgacgattttgctctcatagtgaatgcaccaaaccatgagggaatacaagcaggagtggacagattctacaggacctgcaagttagtgggaatcaacatgagcaaaaagaagtcctatataaataagacagggacatttgaattcacaagcytttnttatcgctatggatttgtggctaattttagcatggagctacccagctttggagtgtctggagtaaatgaatcagctgacatgagtattggagcaacagtgataaagaacaacatgataaacaatgaccttggacctgcaacggcccagatggctcttcaattgttcatcaaagactacagatacacatataggtgccataggggagacacacaaattcagacgagaagatcatttgagttaaagaagctgtgggatcaaacccaatcaaaggtagggctattagtatcagatggaggaccaaacttatacaatatacggaatcttcacattcctgaagtctgcttaaaatgggagctaatggatgatgattatcggggaagactttgtaatcccctgaatccctttgtaagtcataaagagattgattctgtaaacaatgctgtggtaatgccagcccatggtccagccaaaagcatggaatatgatgccgttgcaactacacattcctggattcccaagaggaatcgttctattctcaacacaagccaaaggggaattcttgaggatgaacagatgtaccagaagtgctgcaatctattcgagaaatttttccctagcagttcatataggagaccggttggaatttctagcatggtggaggccatggtgtctagggcccggattgatgccagggtcgactttgagtctggacggatcaagaaagaagagttctctgagatcatgaagatctgttccaccattgaagaactcagacggcaaaaataa

>H1N1_PB1_KT180539_A/Singapore/DMS946/2009

atggatgtcaatccgactctacttttcctaaaaattccagcgcaaaatgccataagcaccacattcccttatactggagatcctccatacagccatggaacaggaacaggatacaccatggacacagtaaacagaacacaccaatactcagaaaagggaaagtggacgacaaacacagagactggtgcaccccagctcaacccgattgatggaccactacctgaggataatgaaccaagtgggtatgcacaaacagactgtgttctagaggctatggctttccttgaagaatcccacccaggaatatttgagaattcatgccttgaaacaatggaagttgttcaacaaacaagggtagataaactaactcaaggtcgccagacttatgattggacattaaacagaaatcaaccggcagcaactgcattggccaacaccatagaagtctttagatcgaatggcctaacagctaatgagtcaggaaggctaatagatttcttaaaggatgtaatggaatcaatgaacaaagaggaaatggagataacaacccactttcaaagaaaaaggagagtaagagacagcatgaccaagaagatggtcacgcaaagaacaatagggaagaaaaaacaaagactgaataagagaggctatctaataagagcactgacattaaatacgatgaccaaagatgcagagagaggcaagttaaaaagaagggctatcgcaacacctgggatgcagattagaggtttcgtatactttgttgaaactttagctaggagcatttgcgaaaagcttgaacagtctgggctcccagtagggggcaatgaaaagaaggccaaactggcaaatgttgtgagaaagatgatgactaattcacaagacacagagatttctttcacaatcactggggacaacactaagtggaatgaaaatcaaaatcctcgaatgttcctggcgatgattacatatatcaccagaaatcaacccgagtggttcagaaacatcctgagcatggcacccataatgttctcaaacaaaatggcaaggctagggaaagggtacatgttcgagagtaaaagaatgaagattcgaacacaaataccagcagaaatgctagcaagcattgacctgaagtacttcaatgaatcaacaaagaagaaaattgagaaaataaggcctcttctaatagatggcacagcatcactgagtcctgggatgatgatgggcatgttcaacatgctaagtacggtcttgggagtctcgatactgaatcttggacaaaagaaatacaccaagacaatatactggtgggatgggctccaatcatccgacgattttgctctcatagtgaatgcaccaaaccatgagggaatacaagcaggagtggacagattctacaggacctgcaagttagtaggaatcaacatgagcaaaaagaagtcctatataaataagacagggacatttgaattcacaagctttttttatcgctatggatttgtggctaattttagcatggagctacccagctttggagtgtctggagtaaatgaatcagctgacatgagtattggagtaacagtgataaagaacaacatgataaacaatgaccttggacctgcaacggcccagatggctcttcaattgttcatcaaagactacagatacacatataggtgccataggggagacacacaaattcagacgagaagatcatttgagttaaagaagctgtgggatcaaacccaatcaaaggtagggctattagtatcagatggaggaccaaacttatacaatatacggaatcttcacattcctgaagtctgcttaaaatgggagctaatggatgatgattatcggggaagactttgtaatcccctgaatccctttgtcagtcataaagagattgattctgtaaacaatgctgtggtaatgccagcccatggtccagccaaaagcatggaatatgatgccgttgcaactacacattcctggattcccaagaggaatcgttctgttctcaacacaagccaaaggggaattcttgaggatgaacagatgtaccagaagtgctgcaatctattcgagaaatttttccctagcagttcatataggagaccggttggaatttctagcatggtggaggccatggtgtctagggccctgattgatgccagggtcgacttcgagtctggacggatcaagaaagaagagttctctgagatcatgaagatctgttccaccattgaagaactcagacggcaaaaataa

>H1N1_PB1_KT180491_A/Singapore/DMS58/2009

atggatgtcaatccgactctacttttcctaaaaattccagcgcaaaatgccataagcaccacattcccttatactggagatcctccatacagccatggaacaggaacaggatacaccatggacacagtaaacagaacacaccaatactcagaaaagggaaagtggacgacaaacacagagactggtgcaccccagctcaacccgattgatggaccactacctgaggataatgaaccaagtgggtatgcacaaacagactgtgttctagaggctatggctttccttgaagaatcccacccaggaatatttgagaattcatgccttgaaacaatggaagttgttcaacaaacaagggtagataaactaactcaaggtcgccagacttatgattggacattaaacagaaatcaaccggcagcaactgcattggccaacaccatagaagtctttagatcgaatggcctaacagctaatgagtcaggaaggctaatagatttcttaaaggatgtaatggaatcaatgaacaaagaggaaatagagataacaacccactttcaaagaaaaaggagagtaagagacaacatgaccaagaagatggtcacgcaaagaacaatagggaagaaaaaacaaagactgaataagagaggctatctaataagagcactgacattaaatacgatgaccaaagatgcagagagaggcaagttaaaaagaagggctatcgcaacacctgggatgcagattagaggtttcgtatactttgttgaagctttagctaggagcatttgcgaaaagcttgaacagtctgggctcccagtagggggcaatgaaaagaaggccaaactggcaaatgttgtgagaaagatgatgactaattcacaagacacagagatttctttcacaatcactggggacaacactaagtggaatgaaaatcaaaatcctcgaatgttcctggcaatgattacatatatcaccagaaatcaacccgagtggttcagaaacatcctgagcatggcacccataatgttctcaaacaaaatggcaagactagggaaagggtacatgttcgagagtaaaagaatgaagattcgaacacaaataccagcagaaatgctagcaagcattgacctgaagtacttcaatgaatcaacaaagaagaaaattgagaaaataaggcctcttctaatagatggcacagcatcactgagtcctgggatgatgatgggcatgttcaacatgctaagtacggtcttgggagtctcgatactgaatcttggacaaaagaaatacaccaagacaatatactggtgggatgggctccaatcatccgacgattttgctctcatagtgaatgcaccaaaccatgagggaatacaagcaggagtggacagattctacaggacctgcaagttagtgggaatcaacatgagcaaaaagaagtcctatataaataagacagggacatttgaattcacaagctttttttatcgctatggatttgtggctaattttagcatggagctacccagctttggagtgtctggagtaaatgaatcagctgacatgagtattggagtaacagtgataaagaacaacatgataaacaatgaccttggacctgcaacggcccagatggctcttcaattgttcatcaaagactacagatacacatataggtgccataggggagacacacaaattcagacgagaagatcatttgagttaaagaagctgtgggatcaaacccaatcaaaggtagggctattagtatcagatggaggaccaaacttatacaatatacggaatcttcacattcctgaagtctgcttaaaatgggagctaatggatgatgattatcggggaagactttgtaatcccctgaatccctttgtcggtcataaagagattgattctgtaaacaatgctgtggtaatgccagcccatggtccagccaaaagcatggaatatgatgccgttgcaactacacattcctggattcccaagaggaatcgttctattctcaacacaagccaaaggggaattcttgaggatgaacagatgtaccagaagtgctgcaatctattcgagaaatttttccctagcagttcatataggagaccggttggaatttctagcatggtggaggccatggtgtctagggcccagattgatgccagggtcgacttcgagtctggacggatcaagaaagaagagttctctgagatcatgaagatctgttccaccattgaagaactcagacggcaaaaataa

>H1N1_PB1_KT180420_A/Singapore/DMS1240/2012

atggatgtcaatccgactctacttttcctaaaaattccagcgcaaaatgccataagcaccacattcccttatactggagatcctccatacagccatggaacaggaacaggatacaccatggacacagtaaacagaacacaccaatactcagaaaagggaaagtggacgacaaacacagagactggtgcaccccagctcaacccgattgatggaccactacctgaggataatgaaccaagtgggtatgcacaaacagactgtgttttagaggctatggctttccttgaagaatcccacccaggaatatttgagaattcatgccttgaaacaatagaagttgttcaacaaacaagggtagataaactgactcaaggtcgccagacttatgattggacattaaacagaaatcaaccggcagcaactgcattggccaacaccatagaagtctttagatcgaatggcctaacagctaatgagtcgggaaggctaatagatttcttaaaggatgtaatggaatcaatgaacaaagaggaaatagagataacaacccactttcaaagaaaaaggagagtaagagacaacatgaccaagaagatggtcacgcaaagaacaatagggaagaaaaaacagagactgaataagagaggctatctaataagagcactaacattaaatacgatgaccaaagatgcagagagaggcaagttaaaaagaagggctatcgcaacacctgggatgcagattagaggtttcgtatactttgttgaaactttagctaggagcatttgcgaaaagcttgaacagtctgggctcccagtagggggcaatgaaaagaaggccaagctggcaaatgttgtgagaaagatgatgactaattcacaagacacagagatttctttcacaatcactggggacaacactaagtggaatgaaaatcaaaatcctcgaatgttcctggcgatgattacatatatcaccagaaatcaacccgaatggtttagaaacatcctgagcatggcacccataatgttctcaaacaaaatggcacgactagggaaagggtacatgttcgagagtaaaagaatgaagattcgaacacaaataccagcagaaatgctagcaagcattgacctgaagtacttcaatgaatcaacaaagaagaaaattgagaaaataaggcctcttttaatggatggcacagcatcactgagtcctgggatgatgatgggcatgttcaacatgctaagtacggtcttgggagtctcgatactgaatcttggacaaaagaaatacaccaagacaacatactggtgggatgggctccaatcatccgacgattttgccctcatagtgaatgcaccaaatcatgaggggatacaagcaggagtggacagattctacaggacctgcaagttagtgggaatcaacatgagcaaaaagaagtcctatataaataagacagggacatttgaattcacaagctttttttatcgctatggatttgtggctaattttagcatggagctacccagctttggagtgtctggagtaaatgaatcagctgacatgagtattggagtaacagtgataaaaaacaacatgataaacaatgaccttggacctgcaacggcccagatggctctccaattgttcatcaaagactacagatacacatataggtgccataggggagacacacaaattcagacgagaagatcatttgagttaaagaagctgtgggatcaaacccaatcaaaggtagggctattagtatcagatggaggaccaaacttatacaatatacggaatcttcacatccctgaagtctgcttaaaatgggagctaatggatgatgattatcggggaagactttgtaatcccctgaatccctttgtaagtcataaggagattgattctgtaaacaatgctgtggtaatgccagcccatggtccagccaaaagcatggaatatgatgccgttgcaactacacattcctggattcccaagaggaatcgttctattctcaacacaagccaaaggggaattcttgaggatgaacagatgtatcagaagtgctgcaatctattcgagaaatttttccctagcagttcatataggagaccggttggaatttctagcatggtggaggccatggtgtctagggcacgaattgatgccagggtcgactttgagtctggacggatcaagaaagaagagttctctgagatcatgaagatctgttccaccattgaagaactcagacggcaaaaataa

>H1N1_PB1_KT180340_A/Singapore/DMS100/2010

atggatgtcaatccgactctacttttcctaaaaattccagcgcaaaatgccataagcaccacattcccttatactggagatcctccatacagccatggaacaggaacaggatacaccatggacacagtaaccagaacacaccaatactcagaaaagggaaagtggacgacaaacacagagactggtgcaccccagctcaacccgattgatggaccactacctgaggataatgaaccaagtgggtatgcacaaacagactgtgttctagaggctatggctttccttgaggaatcccacccaggaatatttgagaattcatgccttgaaacaatggaagttgttcaacaaacaagggtagataaactaactcaaggtcgccagacttatgattggacattgaacagaaatcaaccggcagcaactgcattggccaacaccatagaagtctttagatcgaatggcctaacagctaatgagtcaggaaggctaatagatttcttaaaggatgtaatggaatcaatgaacacagaggaaatagagataacaacccactttcaaagaaaaaggagagtaagagacaacatgaccaagaagatggtcacgcaaagaacaatagggaagaaaaaacaaagactgaataagagaggctatctaataagagcactgacattaaatacgatgaccaaagatgcagagagaggcaagttaaaaagaagggctatcgcaacacctgggatgcagattagaggtttcgtatactttgttgaagctttagctaggagcatttgcgaaaagcttgaacagtctgggctcccagtagggggcaatgaaaagaaggccaaactggcaaatgttgtgagaaagatgatgactaattcacaagacacagagatttctttcacaatcactggggacaacactaagtggaatgaaaatcaaaatcctcgaatgttcctggcaatgattacatatatcaccagaaatcaacccgagtggttcagaaacatcctgagcatggcacccataatgttctcaaacaaaatggcaagactagggaaagggtacatgttcgagagtaaaagaatgaagattcgaacacaaataccagcagaaatgctagcaagcattgacctgaagtacttcaatgaatcaacaaagaagaaaattgagaaaataaggcctcttctaatagatggcacagcatcactgagtcctgggatgatgatgggcatgttcaacatgctaagtacggtcttgggagtctcgatactgaatcttggacaaaagaaatacaccaagacaatatactggtgggatgggctccaatcatccgacgattttgctctcatagtgaatgcaccaaaccatgagggaatacaagcaggagtggatagattctacaggacctgcaagttagtgggaatcaacatgagcaaaaagaagtcctatataaataagacagggacatttgaattcacaagctttttttatcgctatggatttgtggctaattttagcatggagctacccagctttggagtgtctggagtaaatgaatcagctgacatgagtattggagtaacagtgataaagaacaacatgataaacaatgaccttggacctgcaacggcccagatggctcttcaattgttcatcaaagactacagatacacatataggtgccataggggagacacacaaattcagacgagaagatcatttgagttaaagaagctgtgggatcaaacccaatcaaaggtagggctattagtatcagatggaggaccaaacttgtacaatatacggaatcttcacattcctgaagtctgcttaaaatgggagctaatggatgatgattatcggggaagactttgtaatcccctgaatccctttgtcggtcataaagagattgattctgtaaacaatgctgtggtaatgccagcccatggcccagccaaaagcatggaatatgatgccgttgcaactacacattcctggattcccaagaggaatcgttctattctcaacacaagccaaaggggaattcttgaggatgaacaggtgtaccagaagtgctgcaatctattcgagaaatttttccctagcagttcatataggagaccggttggaatttctagcatggtggaggccatggtgtctagggcccggattgatgccagggtcgacttcgagtctggacggatcaagaaagaagagttctctgagatcatgaagatctgttccaccattgaagaactcagacggcaaaaataa

>H1N1_PB1_KT836787_A/California/23/2015

atggatgtcaatccgactttacttttcctaaaaattccagcacaaaatgccataagcaccacattcccttatactggagatcctccatacagccatggaacaggaacaggatacaccatggacacagtaaacagaacacaccaatactcagaaaaggggaagtggacaacaaacacagagactggtgcaccccagctcaacccgattgatggaccactacctgaggataatgaaccaagtgggtatgcacagacagactgtgtcctagaggctatggctttccttgaagaatcccacccaggaatatttgagaattcatgccttgaaacaatggaagttgttcaacaaacaagggtagataaactgactcaaggtcgccagacttatgattggacattaaacagaaatcaaccagcagcaactgcattggccaacaccatagaagtctttagatcgaatgacctaacagctaacgagtcaggaaggctaatagatttcttaaaggatgtaatggaatcaatggacaaagaggaaatagagataacaacccactttcaaagaaaaaggagagtaagagacaacatgaccaagaagatggtcacgcaaagaacaatagggaagaaaaaacagaaactgaataagagaggctatctaataagagcactgacattaaatacgatgaccaaagatgcagagagaggcaagttaaaaagaagggctatcgcaacacctgggatgcagattagaggtttcgtatactttgttgaaactttagctaggagcatttgcgaaaagcttgaacagtctgggctcccagtagggggcaatgaaaagaaggccaaattggcaaatgttgtgagaaagatgatgactaattcacaagatacagagatttctttcacaatcactggggacaacactaaatggaatgaaaatcaaaatcctcgaatgttcctggcaatgattacatatatcaccagaaatcaacccgagtggtttagaaacatcctgagcatggcacccataatgttctcaaataaaatggcaagactggggaaagggtacatgttcgagagtaaaagaatgaaaattcgaacacaaataccagcagaaatgctagcaagcattgatctgaagtacttcaatgaatcaacaaagaagaaaattgagaaaataaggcctcttttaatggatggcacagcatcactgagtcctgggatgatgatgggcatgttcaacatgctaagtacggtcttgggagtctcgatactgaatcttggacaaaagaaatacaccaagacaacatactggtgggatgggctccaatcatccgacgattttgctctcatagtgaatgcaccaaaccacgaaggaatacaagcaggagtggacagattctacaggacctgcaagttagtgggaatcaacatgagcaaaaagaagtcttatataaataagacagggacatttgaattcacaagctttttttatcgctatggatttgtggctaattttagcatggagctacccagctttggagtgtctggagtaaatgaatcagctgacatgagtattggagtaacagtgataaagaacaacatgataaacaatgaccttggacctgcaacggcccagatggctcttcaattgttcatcaaagactacagatacacatataggtgccacaggggagacacacaaattcagacgagaagatcatttgagttaaagaagctatgggatcaaacccaatcaaaggtagggctattagtatcagatggaggaccaaacttatacaacatacggaatcttcacattcctgaagtctgcttaaaatgggagctaatggatgatgattatcggggaagactttgtaatcccctgaatccctttgtgagtcataaggagattgattctgtaaacaatgccgtggtaatgccagcccatggtccagccaaaagcatggaatatgatgccgttgcaactacacattcctggattcccaagaggaatcgttctatcctcaacacaagccaaaggggaattcttgaggatgaacagatgtaccagaagtgttgcaatctattcgagaaatttttccctagcagttcatataggagaccggttggaatttctagcatggtggaggccatggtgtctagggcccggattgatgccagggtcgactttgagtctggacggatcaagaaagaggagttctctgagatcatgaagatctgttccaccattgaagaactcagacggcaaaaataa

>H1N1_PB1_KT836904_A/California/35/2015

atggatgtcaatccgactctacttttcctaaaaattccagcacaaaatgccataagcaccacattcccttatactggagatcctccatacagccatggaacaggaacaggatacaccatggacacagtaaacagaacacaccaatactcagaaaaggggaagtggacaacaaacacagagactggtgcaccccagctcaacccgattgatggaccactacctgaggataatgaaccaagtgggtatgcacagacagactgtgtcctagaggctatggctttccttgaagaatcccacccaggaatatttgagaattcatgccttgaaacaatggaagttgttcaacaaacaagggtagataaactgactcaaggtcgccagacttatgattggacattaaacagaaatcaaccagcagcaactgcattggccaacaccatagaagtctttagatcgaatgacctaacagctaacgagtcaggaaggctaatagatttcttgaaggatgtaatggaatcaatggacaaagaggaaatagagataacaacccactttcaaagaaaaaggagagtaagagacaacatgaccaagaagatggtcacgcaaagaacaatagggaagaaaaaacagaaactgaataagagaggctatctaataagagcactgacattaaatacgatgaccaaagatgcagagagaggcaagttaaaaagaagggctatcgcaacacctgggatgcagattagaggtttcgtatactttgttgaaactttagctaggagcatttgcgaaaagcttgaacagtctgggctcccagtagggggcaatgaaaagaaggccaaattggcaaatgttgtgagaaagatgatgactaattcacaagatacagagatttctttcacaatcactggggacaacactaaatggaatgaaaatcaaaatcctcgaatgttcctggcaatgattacatatatcaccagaaatcaacccgagtggtttagaaacatcctgagcatggcacccataatgttctcaaataaaatggcaagactggggaaagggtacatgttcgagagtaaaagaatgaaaattcgaacacaaataccagcagaaatgctagcaagcattgatctgaagtacttcaatgaatcaacaaagaagaaaattgagaaaataaggcctcttttaatggatggcacagcatcactgagtcctgggatgatgatgggcatgttcaacatgctaagtacggtcttgggagtctcgatactgaatcttggacaaaagaaatacaccaagacaacatactggtgggatgggctccaatcatccgacgattttgctctcatagtgaatgcaccaaaccacgaaggaatacaagcaggagtggacagattctacaggacctgcaagttagtgggaatcaacatgagcaaaaagaagtcttatataaataagacagggacatttgaattcacaagctttttttatcgctatggatttgtggctaattttagcatggagctacccagctttggagtgtctggagtaaatgaatcagctgacatgagtattggagtaacagtgataaagaacaacatgataaacaatgaccttggacctgcaacggcccagatggctcttcaattgttcatcaaagactacagatacacatataggtgccacaggggagacacacaaattcagacgagaagatcatttgagttaaagaagctatgggatcaaacccaatcaaaggtagggctattagtatcagatggaggaccaaacttatacaacatacggaatcttcacattcctgaagtctgcttaaagtgggagctaatggatgatgattatcggggaagactttgtaatcccctgaatccctttgtgagtcataaggagattgattctgtaaacaatgccgtggtaatgccagcccatggtccagccaaaagcatggaatatgatgccgttgcaactacacattcctggattcccaagaggaatcgttctatcctcaacacaagccaaaggggaattcttgaggatgaacagatgtaccagaagtgttgcaatctattcgagaaatttttccctagcagttcatataggagaccggttggaatttctagcatggtggaggccatggtgtctagggcccggattgatgccagggtcgactttgagtctggacggatcaagaaagaggagttctctgagatcatgaagatctgttccaccattgaagaactcagacggcaaaaataa

>H1N1_PB1_KX406233_A/Delaware/04/2016

atggatgtcaatccgactctacttttcctaaaaattccagcacaaaatgccataagcaccacattcccttatactggagatcctccatacagccatggaacaggaacaggatacaccatggacacagtaaacagaacacaccaatactcagaaaaggggaagtggacaacaaacacagagactggtgcaccccagctcaacccgattgacggaccactacctgaagataatgaaccaagtgggtatgcacaaacagactgtgttctagaggctatggcttttcttgaagaatcccatccaggaatatttgagaattcatgccttgaaacaatggaagttgttcaacaaacaagggtagataaactgactcaaggtcgccagacttatgattggacattaaacagaaatcaaccggcagcaactgcattggccaacaccatagaagtttttagatcgaatgacctaacagctaacgagtcaggaaggctaatagatttcttaaaggatgtgatggaatcaatgaacaaagaggaaatagagataacaacccactttcaaagaaaaaggagagtaagagacaacatgaccaagaagatggtcacgcaaagaacaatagggaagaaaaaacagagactgaataagagaggctatctaataagagcactgacattaaatacgatgaccaaagatgcagagagaggcaagttaaaaagaagggctatcgcaacacctgggatgcagattagaggtttcgtatactttgttgaaactttagctaggagcatttgcgaaaagcttgaacagtctgggctcccagtagggggcaatgaaaagaaggccaaattggcaaatgttgtgagaaagatgatgactaattcacaagatacagagatttctttcacaatcactggggacaacactaagtggaatgaaaatcaaaatcctcgaatgttcctggcgatgattacatatatcaccagaaatcaacccgagtggtttagaaacatcctgagcatggcacccataatgttctcaaataaaatggcaagactaggaaaagggtacatgttcgagagtaaaagaatgaagattcgaacacaaataccagcagaaatgctagcaagcattgatctgaagtatttcaatgaatcaacaaagaagaaaattgagaaaataaggcctcttttaatggatggcacagcatcactgagtcctgggatgatgatgggcatgttcaacatgctaagtacggtcttgggagtctcgatactgaatcttggacaaaagaaatacaccaagacaacatactggtgggatgggctccaatcatccgacgattttgctctcatagtgaatgcaccaaaccatgaaggaatacaagcaggagtggacagattctacaggacctgcaagttagtgggaatcaacatgagcaaaaagaagtcctatataaataagacagggacatttgaattcacaagctttttttatcgctatggatttgtggctaattttagcatggagctacccagctttggagtgtctggagtaaatgaatcagctgacatgagtattggagtaacagtgataaagaacaacatgataaacaatgaccttggacctgcaacggctcagatggctcttcaattgttcatcaaagactacagatacacatataggtgccataggggagacacacaaattcagacgagaagatcatttgagttgaagaagctatgggatcaaacccaatcaaaggtagggctattagtatcagatggaggaccaaacttatacaacatacggaatcttcacattcctgaagtctgcttaaaatgggagctaatggatgatgattatcggggaagactttgtaatcccctgaatccctttgtgagtcataaggagattgattctgtaaacaatgctgtggtaatgccagcccatggcccagccaaaagcatggaatatgatgccgttgctactacacattcctggattcccaagaggaatcgttctattctcaacacaagccaaaggggaattcttgaggatgaacagatgtaccagaagtgttgcaatttattcgagaaatttttccctagcagttcatataggagaccggttggaatttctagcatggtggaggccatggtgtctagggcccggattgatgccagagtcgactttgagtctggacggatcaagaaagaagagttctctgagatcatgaagatctgttccaccattgaagaactcagacggcaaaaataa

>H1N1_PB1_KX406305_A/Massachusetts/03/2016

atggatgtcaatccgactctacttttcctaaaaattccagcacaaaatgccataagcaccacattcccttatactggagatcctccatacagccatggaacaggaacaggatacaccatggacacagtaaacagaacacaccaatactcagaaaaggggaagtggacaacaaacacagagactggtgcaccccagctcaacccgattgacggaccactacctgaagataatgaaccaagtgggtatgcacaaacagactgtgttctagaggctatggcttttcttgaagaatcccatccaggaatatttgagaattcatgccttgaaacaatggaagttgttcaacaaacaagggtagataaactgactcaaggtcgccagacttatgattggacattaaacagaaatcaaccggcagcaacagcattggccaacaccatagaagtttttagatcgaatgacctaacagctaacgagtcaggaaggctaatagatttcttaaaggatgtgatggaatcaatgaacaaagaggaaatagagataacaacccactttcaaagaaaaaggagagtaagagacaacatgaccaagaagatggtcacgcaaagaacaatagggaagaaaaaacagagactgaataagagaggctatctaataagagcactgacattaaatacgatgaccaaagatgcagagagaggcaagttaaaaagaagggctatcgcaacacctgggatgcagattagaggtttcgtatactttgttgaaactttagctaggagcatttgcgaaaagcttgaacagtctgggctcccagtagggggcaatgaaaagaaggccaaattggcaaatgttgtgagaaagatgatgactaattcacaagatacagagatttctttcacaatcactggggacaacactaagtggaatgaaaatcaaaatcctcgaatgttcctggcgatgattacatatatcaccagaaatcaacccgagtggtttagaaacatcctgagcatggcacccataatgttctcaaataaaatggcaagactaggaaaagggtacatgttcgagagtaaaagaatgaagattcgaacacaaataccagcagaaatgctagcaagcattgatctgaagtatttcaatgaatcaacaaagaagaaaattgagaaaataaggcctcttttaatggatggcacagcatcactgagtcctgggatgatgatgggcatgttcaacatgctaagtacggtcttgggagtctcgatactgaatcttggacaaaagaaatacaccaagacaacatactggtgggatgggctccaatcatccgacgattttgctctcatagtgaatgcaccaaaccatgaaggaatacaagcaggagtggacagattctacaggacctgcaagttagtgggaatcaacatgagcaaaaagaagtcctatataaataagacagggacatttgaattcacaagctttttttatcgctatggatttgtggctaattttagcatggagctacccagctttggagtgtctggagtaaatgaatcagctgacatgagtattggagtaacagtgataaagaacaacatgataaacaatgaccttggacctgcaacggctcagatggctcttcaattgttcatcaaagactacagatacacatataggtgccataggggagacacacaaattcagacgagaagatcatttgagttgaagaagctatgggatcaaacccaatcaaaggtagggctattagtatcagatggaggaccaaacttatacaacatacggaatcttcacattcctgaagtctgcttaaaatgggagctaatggatgatgattatcggggaagactttgtaatcccctgaatccctttgtgagtcataaggagattgattctgtaaacaatgctgtggtaatgccagcccatggcccagccaaaagcatggaatatgatgccgttgctactacacattcctggattcccaagaggaatcgttctattctcaacacaagccaaaggggaattcttgaggatgaacagatgtaccagaagtgttgcaatttattcgagaaatttttccctagcagttcatataggagaccggttggaatttctagcatggtggaggccatggtgtctagggcccggattgatgccagagtcgactttgagtctggacggatcaagaaagaagagttctctgagatcatgaagatctgttccaccattgaagaactcagacggcaaaaataa

>H1N1_PB1_KX406497_A/Michigan/23/2016

atggatgtcaatccgactctacttttcctaaaaattccagcacaaaatgccataagcaccacattcccttatactggagatcctccatacagccatggaacaggaacaggatacaccatggacacagtaaacagaacacaccaatactcagaaaaggggaagtggacaacaaacacagagactggtgcaccccagctcaacccgattgacggaccactacctgaagataatgaaccaagtgggtatgcacaaacagactgtgttctagaggctatggcttttcttgaagaatcccatccaggaatatttgagaattcatgccttgaaacaatggaagttgttcaacaaacaagggtagataaactgactcaaggtcgccagacttatgattggacattaaacagaaatcaaccggcagcaactgcattggccaacaccatagaagtttttagatcgaatgacctaacagccaacgagtcaggaaggctaatagatttcttaaaggatgtgatggaatcaatgaacaaagaggaaatagagataacaacccactttcaaagaaaaaggagagtaagagacaacatgaccaagaagatggtcacgcaaagaacaatagggaagaaaaaacagagactgaataagagaggctatctaataagagcactgacattaaatacgatgaccaaagatgcagagagaggcaagttaaaaagaagggctatcgcaacacctgggatgcagattagaggtttcgtatactttgttgaaactttagctaggagcatttgcgaaaagcttgaacagtctgggctcccagtagggggcaatgaaaagaaggccaaattggcaaatgttgtgagaaagatgatgactaattcacaagatacagagatttctttcacaatcactggggacaacactaagtggaatgaaaatcaaaatcctcgaatgttcctggcgatgattacatatatcaccagaaatcaacccgagtggtttagaaacatcctgagcatggcacccataatgttctcaaataaaatggcaagactaggaaaagggtacatgttcgagagtaaaagaatgaagattcgaacacaaataccagcagaaatgctagcaagcattgatctgaagtatttcaatgaatcaacaaagaagaaaattgagaaaataaggcctcttttaatggatggcacagcatcactgagtcctgggatgatgatgggcatgttcaacatgctaagtacggtcttgggagtctcgatactgaatcttggacaaaagaaatacaccaagacaacatactggtgggatgggctccaatcatccgacgattttgctctcatagtgaatgcaccaaaccatgaaggaatacaagcaggagtggacagattctacaggacctgcaagttagtgggaatcaacatgagcaaaaagaagtcctatataaataagacagggacatttgaattcacaagctttttttatcgctatggatttgtggctaattttagcatggagctacccagctttggagtgtctggagtaaatgaatcagctgacatgagtattggagtaacagtgataaagaacaacatgataaacaatgaccttggacctgcaacggctcagatggctcttcaattgttcatcaaagactacagatacacatataggtgccataggggagacacacaaattcagacgagaagatcatttgagttgaagaagctatgggatcaaacccaatcaaaggtagggctattagtatcagatggaggaccaaacttatacaacatacggaatcttcacattcctgaagtctgcttaaaatgggagctaatggatgatgattatcggggaagactttgtaatcccctgaatccctttgtgagtcataaggagattgattctgtaaacaatgctgtggtaatgccagcccatggcccagccaaaagcatggaatatgatgccgttgctactacacattcctggattcccaagaggaatcgttctattctcaacacaagccagaggggaattcttgaggatgaacagatgtaccagaagtgttgcaatttattcgagaaatttttccctagcagttcatataggagaccggttggaatttctagcatggtggaggccatggtgtctagggcccggattgatgccagagtcgactttgagtctggacggatcaagaaagaagagttctctgagatcatgaagatctgttccaccattgaagaactcagacggcaaaaataa

>H1N1_PB1_KX406841_A/Texas/25/2016

atggatgtcaatccgactctacttttcctaaaaattccagcacaaaatgccataagcaccacattcccttatactggagatcctccatacagccatggaacaggaacaggatacaccatggacacagtaaacagaacacaccaatactcagaaaaggggaagtggacaacaaacacagagactggtgcaccccagctcaacccgattgacggaccactacctgaagataatgaaccaagtgggtatgcacaaacagactgtgttctagaggctatggcttttcttgaagaatcccatccaggaatatttgagaattcatgccttgaaacaatggaagttgttcaacaaacaagggtagataaactgactcaaggtcgccagacttatgattggacattaaacagaaatcaaccggcagcaactgcattggccaacaccatagaagtttttagatcgaatgacctaacagctaacgagtcaggaaggctaatagatttcttaaaggatgtgatggaatcaatgaacaaagaggaaatagagataacaacccactttcaaagaaaaaggagagtaagagacaacatgaccaagaagatggtcacgcaaagaacaatagggaagaaaaaacagagactgaataagagaggctatctaataagagcactgacattaaatacgatgaccaaagatgcagagagaggcaagttaaaaagaagggctatcgcaacacctgggatgcagattagaggtttcgtatactttgttgaaactttagctaggagcatttgcgaaaagcttgaacagtctgggctcccagtagggggcaatgaaaagaaggccaaattggcaaatgttgtgagaaagatgatgactaattcacaagatacagagatttctttcacaatcactggggacaacactaagtggaatgaaaatcaaaatcctcgaatgttcctggcgatgattacatatatcaccagaaatcaacccgagtggtttagaaacatcctgagcatggcacccataatgttctcaaataaaatggcaagactaggaaaagggtacatgttcgagagtaaaagaatgaagattcgaacacaaataccagcagaaatgctagcaagcattgatctgaagtatttcaatgaatcaacaaagaagaaaattgagaaaataaggcctcttttaatggatggcacagcatcactgagtcctgggatgatgatgggcatgttcaacatgctaagtacggtcttgggagtctcgatactgaatcttggacaaaagaaatacaccaagacaacatactggtgggatgggctccaatcatccgacgattttgctctcatagtgaatgcaccaaaccatgaaggaatacaagcaggagtggacagattctacaggacctgcaagttagtgggaatcaacatgagcaaaaagaagtcctatataaataagacagggacatttgaattcacaagctttttttatcgctatggatttgtggctaattttagcatggagctacccagctttggagtgtctggagtaaatgaatcagctgacatgagtattggagtaacagtgataaagaacaacatgataaacaatgaccttggacctgcaacggctcagatggctcttcaattgttcatcaaagactacagatacacatataggtgccataggggagacacacaaattcagacgagaagatcatttgagttgaagaagctatgggatcaaacccaatcaaaggtagggctattagtatcagatggaggaccaaacttatacaacatacggaatcttcacattcctgaagtctgcttaaaatgggagctaatggatgatgattatcggggaagactttgtaatcccctgaatccctttgtgagtcataaggagattgattctgtaaacaatgctgtggtaatgccagcccatggcccatccaaaagcatggaatatgatgccgttgctactacacattcctggattcccaagaggaatcgttctattctcaacacaagccaaaggggaattcttgaggatgaacagatgtaccagaagtgttgcaatttattcgagaaatttttccctagcagttcatataggagaccggttggaatttctagcatggtggaggccatggtgtctagggcccggattgatgccagagtcgactttgagtctggacggatcaagaaagaagagttctctgagatcatgaagatctgttccaccattgaagaactcagacggcaaaaataa

>H1N1_PB1_KX407913_A/Michigan/48/2016

atggatgtcaatccgactctacttttcctaaaaattccagcacaaaatgccataagcaccacattcccttatactggagatcctccatacagccatggaacaggaacaggatacaccatggacacagtaaacagaacacaccaatactcagaaaaggggaagtggacaacaaacacagagactggtgcaccccagctcaacccgattgacggaccactacctgaagataatgaaccaagtgggtatgcacaaacagactgtgttctagaggctatggcttttcttgaagaatcccatccaggaatatttgagaattcatgccttgaaacaatggaagttgttcaacaaacaagggtagataaactgactcaaggtcgccagacttatgattggacattaaacagaaatcaaccggcagcaactgcattggccaacaccatagaagtttttagatcgaatgacctaacagctaacgagtcaggaaggctaatagatttcttaaaggatgtgatggaatcaatgaacaaagaggaaatagagataacaacccactttcaaagaaaaaggagagtaagagacaacatgaccaagaagatggtcacgcaaagaacaatagggaagaaaaaacagagactgaataagagaggctatctaataagagcactgacattaaatacgatgaccaaagatgcagagagaggcaagttaaaaagaagggctatcgcaacacctgggatgcagattagaggtttcgtatactttgttgaaactttagctaggagcatttgcgaaaagcttgaacagtctgggctcccagtagggggcaatgaaaagaaggccaaattggcaaatgttgtgagaaagatgatgactaattcacaagatacagagatttctttcacaatcactggggacaacactaagtggaatgaaaatcaaaatcctcgaatgttcctggcaatgattacatatatcaccagaaatcaacccgagtggtttagaaacatcctgagcatggcacccataatgttctcaaataaaatggcaagactagggaaagggtacatgttcgagagtaaaagaatgaagattcgaacacaaataccagcagaaatgctagcaagcattgatctgaagtatttcaatgaatcaacaaagaagaaaattgagaaaataaggcctcttttaatggatggcacagcatcactgagtcctgggatgatgatgggcatgttcaacatgctaagtacggtcttgggagtctcgatactgaatcttggacaaaagaaatacaccaagacaacatactggtgggatgggctccaatcatccgacgattttgctctcatagtgaatgcaccaaaccatgaaggaatacaagcaggagtggacagattctacaggacctgcaagttagtgggaatcaacatgagcaaaaagaagtcctatataaataagacagggacatttgaattcacaagctttttttatcgctatggatttgtggctaattttagcatggagctacccagctttggagtgtctggagtaaatgaatcagctgacatgagtattggagtaacagtgataaagaacaacatgataaacaatgaccttggacctgcaacggctcagatggctcttcaattgttcatcaaagactacagatacacatataggtgccataggggagacacacaaattcagacgagaagatcatttgagttgaagaagctatgggatcaaacccaatcaaaggtagggctattagtatcagatggaggaccaaacttatacaacatacggaatcttcacattcctgaagtctgcttaaaatgggagctaatggatgatgattatcggggaagactttgtaatcccctgaatccctttgtgagtcataaggagattgattctgtaaacaatgctgtggtaatgccagcccatggcccagccaaaagcatggaatatgatgccgttgctactacacattcctggattcccaagaggaatcgttctattctcaacacaagccaaaggggaattcttgaggatgaacagatgtaccagaagtgttgcaatttattcgagaaatttttccctagcagttcatataggagaccggttggaatttctagcatggtggaggccatggtgtctagggcccggattgatgccagagtcgactttgagtctggacggatcaagaaagaagagttctctgagatcatgaagatctgttccaccattgaagaactcagacggcaaaaataa

>H1N1_PB1_KX407993_A/Connecticut/16/2016

atggatgtcaatccgactctacttttcctaaaaattccagcacaaaatgccataagcaccacattcccttatactggagatcctccatacagccatggaacaggaacaggatacaccatggacacagtaaacagaacacaccaatactcagaaaaggggaagtggacaacaaacacagagactggtgcaccccagctcaacccgattgacggaccactacctgaagataatgaaccaagtgggtatgcacaaacagactgtgttctagaggctatggcttttcttgaagaatcccatccaggaatatttgagaattcatgccttgaaacaatggaagctgttcaacaaacaagggtagataaactgactcaaggtcgccagacttatgattggacattaaacagaaatcaaccggcagcaactgcattggccaacaccatagaagtttttagatcgaatgacctaacagctaacgagtcaggaaggctaatagatttcttaaaggatgtgatggaatcaatgaacaaagaggaaatagagataacaacccactttcaaagaaaaaggagagtaagagacaacatgaccaagaagatggtcacgcaaagaacaatagggaagaaaaaacagagactgaataagagaggctatctaataagagcactgacattaaatacgatgaccaaagatgcagagagaggcaagttaaaaagaagggctatcgcaacacctgggatgcagattagaggtttcgtatactttgttgaaactttagctaggagcatttgcgaaaagcttgaacagtctgggctcccagtagggggcaatgaaaagaaggccaaattggcaaatgttgtgagaaagatgatgactaattcacaagatacagagatttctttcacaatcactggggacaacactaagtggaatgaaaatcaaaatcctcgaatgttcctggcgatgattacatatatcaccagaaatcaacccgagtggtttagaaacatcctgagcatggcacccataatgttctcaaataaaatggcaagactaggaaaagggtacatgttcgagagtaaaagaatgaagattcgaacacaaataccagcagaaatgctagcaagcattgatctgaagtatttcaatgaatcaacaaagaagaaaattgagaaaataaggcctcttttaatggatggcacagcatcactgagtcctgggatgatgatgggcatgttcaacatgctaagtacagtcttgggagtctcgatactgaatcttggacaaaagaaatacaccaaaacaacatactggtgggatgggctccaatcatccgacgattttgctctcatagtgaatgcaccaaaccatgaaggaatacaagcaggagtggacagattctacaggacctgcaagttagtgggaatcaacatgagcaaaaagaagtcctatataaataagacagggacatttgaattcacaagctttttttatcgctatggatttgtggctaattttagcatggagctacccagctttggagtgtctggagtaaatgaatcagctgacatgagtattggagtaacagtgataaagaacaacatgataaacaatgaccttggacctgcaacggctcagatggctcttcaattgttcatcaaagactacagatacacatataggtgccataggggagacacacaaattcagacgagaagatcatttgagttgatgaagctatgggatcaaacccaatcaaaggtagggctattagtatcagatggaggaccaaacttatacaacatacggaatcttcacattcctgaagtctgcttaaaatgggagctaatggatgatgattatcggggaagactttgtaatcccctgaatccctttgtgagtcataaggagattgactctgtaaacaatgctgtggtaatgccagcccatggcccagccaaaagcatggaatatgatgccgttgctactacacattcctggattcccaagaggaatcgttctattctcaacacaagccaaaggggaattcttgaggatgaacagatgtaccagaagtgttgcaatttattcgagaaatttttccctagcagttcatataggagaccggttggaatttctagcatggtggaggccatggtgtctagggcccggattgatgccagagtcgactttgagtccggacggatcaagaaagaagagttctctgagatcatgaagatctgttccaccattgaagaactcagacggcaaaaataa

>H1N1_PB1_KX408753_A/Virginia/12/2016

atggatgtcaatccgactctacttttcctaaaaattccagcacaaaatgccataagcaccacattcccttatactggagatcctccatacagccatggaacaggaacaggatacaccatggacacagtaaacagaacacaccaatactcagaaaaggggaagtggacaacaaacacagagactggtgcaccccagctcaacccgattgacggaccactacctgaagataatgaaccaagtgggtatgcacaaacagactgtgttctagaggctatggcttttcttgaagaatcccatccaggaatatttgagaattcatgccttgaaacaatggaagttgttcaacaaacaagggtagataaactgactcaaggtcgccagacttatgattggacattaaacagaaatcaaccggcagcaactgcattggccaacaccatagaagtttttagatcgaatgacctaacagctaacgagtcaggaaggctaatagatttcttaaaggatgtgatggaatcaatgaacaaagaggaaatagagataacaacccactttcaaagaaaaaggagagtaagagacaacattaccaagaagatggtcacgcaaagaacaatagggaagaaaaaacagagactgaataagagaggctatctaataagagcactgacattaaatacgatgaccaaagatgcagagagaggcaaattaaaaagaagggctatcgcaacacctgggatgcagattagaggtttcgtatactttgttgaaactttagctaggagcatttgcgaaaagcttgaacagtctgggctcccagtagggggcaatgaaaagaaggccaaattggcaaatgttgtgagaaagatgatgactaattcacaagatacagagatttctttcacaatcactggggacaacactaagtggaatgaaaatcaaaatcctcgaatgttcctggcgatgattacatatatcaccagaaatcaacccgagtggtttagaaacatcctgagcatggcacccataatgttctcaaataaaatggcaagactaggaaaagggtacatgttcgagagtaaaagaatgaagattcgaacacaaataccagcagaaatgctagcaagcattgatctgaagtatttcaatgaatcaacaaagaagaaaattgagaaaataaggcctcttttaatggatggcacagcatcactgagtcctgggatgatgatgggcatgttcaacatgctaagtacggtcttgggagtctcgatactgaatcttggacaaaagaaatacaccaagacaacatactggtgggatgggctccaatcatccgacgattttgctctcatagtgaatgcaccaaaccatgaaggaatacaagcaggagtggacagattctacaggacctgcaagttagtgggaatcaacatgagcaaaaagaagtcctatataaataagacagggacatttgaattcacaagctttttttatcgctatggatttgtggctaattttagcatggagctacccagctttggagtgtctggagtaaatgaatcagctgacatgagtattggagtaacagtgataaagaacaacatgataaacaatgaccttggacctgcaacggctcagatggctcttcaattgttcatcaaagactacagatacacatataggtgccataggggagacacacaaattcagacgagaagatcatttgagttgaagaagctatgggatcaaacccaatcaaaggtagggctattagtatcagatggaggaccaaacttatacaacatacggaatcttcacattcctgaagtctgcttaaaatgggagctaatggatgatgattatcggggaagactttgtaatcccctgaatccctttgtgagtcataaggagattgattctgtaaacaatgctgtggtaatgccagcccatggcccagccaaaagcatggaatatgatgccgttgctactacacattcctggattcccaagaggaatcgttctattctcaacacaagccaaaggggaattcttgaggatgaacagatgtaccagaagtgttgcaatttattcgagaaatttttccctagcagttcatataggagaccggttggaatttctagcatggtggaggccatggtgtctagggcccggattgatgccagagtcgactttgagtctggacggatcaagaaagaagagttctctgagatcatgaagatctgttccaccattgaagaactcagacggcaaaaataa

>H1N1_PB1_KX409385_A/Texas/37/2016

atggatgtcaatccgactctacttttcctaaaaattccagcacaaaatgccataagcaccacattcccttatactggagatcctccatacagccatggaacaggaacaggatacaccatggatacagtaaacagaacacaccaatactcagaaaaggggaagtggacaacaaacacagagactggtgcaccccagctcaacccgattgacggaccactacctgaagataatgaaccaagtgggtatgcacaaacagactgtgttctagaggctatggcttttcttgaagaatcccatccaggaatatttgagaattcatgccttgaaacaatggaagttgttcaacaaacaagggtagataaactgactcaaggtcgccagacttatgattggacattaaacagaaatcaaccggcagcaactgcattggccaacaccatagaagtttttagatcgaatgacctaacagctaacgagtcaggaaggctaatagatttcttaaaggatgtgatggaatcaatgaacaaagaggaaatagagataacaacccactttcaaagaaaaaggagagtaagagacaacatgaccaagaagatggtcacgcaaagaacaatagggaagaaaaaacagagactgaataagagaggctatctaataagagcactgacattaaatacaatgaccaaagatgcagagagaggcaagttaaaaagaagggctatcgcaacacctgggatgcagattagaggtttcgtatactttgttgaaactttagctaggagcatttgcgaaaagcttgaacagtctgggctcccagtagggggcaatgaaaagaaggccaaattggcaaatgttgtgagaaagatgatgactaattcacaagatacagagatttctttcacaatcactggggacaacactaagtggaatgaaaatcaaaatcctcgaatgttcctggcgatgattacatatatcaccagaaatcaacccgagtggtttagaaacatcctgagcatggcacccataatgttctcaaataaaatggcaagactaggaaaagggtacatgttcgagagtaaaagaatgaagattcgaacacaaataccagcagaaatgctagcaagcattgatctgaagtatttcaatgaatcaacaaagaagaaaattgagaaaataaggcctcttttaatggatggcacagcatcactgagtcctgggatgatgatgggcatgttcaacatgctaagtacggtcttgggagtctcgatactgaatcttggacaaaagaaatacaccaagacaacatactggtgggatgggctccaatcatccgacgattttgctctcatagtgaatgcaccaaaccatgaaggaatacaagcaggagtggacagattctacaggacctgcaagttagtgggaatcaacatgagcaaaaagaagtcctatataaataagacagggacatttgaattcacaagctttttttatcgctatggatttgtggctaattttagcatggagctacccagctttggagtgtctggagtaaatgaatcagctgacatgagtattggagtaacagtgataaagaacaacatgataaacaatgaccttggacctgcaacggctcagatggctcttcaattgttcatcaaagactacagatacacatataggtgccataggggagacacacaaattcagacgagaagatcatttgagttgaagaagctatgggatcaaacccaatcaaaggtagggctattagtatcagatggaggaccaaacttatacaacatacggaatcttcacattcctgaagtctgcttaaaatgggagctaatggatgatgattatcggggaagactttgtaatcccctgaatccctttgtgagtcataaggagattgattctgtaaacaatgctgtggtaatgccagcccatggcccagccaaaagcatggaatatgatgccgttgctactacacattcctggattcccaagaggaatcgttctattctcaacacaagccaaaggggaattcttgaggatgaacagatgtaccagaagtgttgcaatttattcgagaaatttttccctagcagttcatataggagaccggttggaatttctagcatggtggaggccatggtgtctagggcccggattgatgccagagtcgactttgagtctggacggatcaagaaagaagagttctctgagatcatgaagatctgttccaccattgaagaactcagacggcaaaaataa

>H1N1_PB1_KX409921_A/Indiana/32/2016

atggatgtcaatccgactctacttttcctaaaaattccagcacaaaatgccataagcaccacattcccttatactggagatcctccatacagccatggaacaggaacaggatacaccatggacacagtaaacagaacacaccaatactcagaaaaggggaagtggacaacaaacacagagactggtgcaccccagctcaacccgattgacggaccactacctgaagataatgaaccaagtgggtatgcacaaacagactgtgttctagaggctatggcttttcttgaagaatcccatccaggaatatttgagaattcatgccttgaaacaatggaagttgtccaacaaacaagggtagataaactgactcaaggtcgccagacttatgattggacattaaacagaaatcaaccggcagcaactgcattggccaacaccatagaagtttttagatcgaatgacctaacagctaacgagtcaggaaggctaatagatttcttaaaggatgtgatggaatcaatgaacaaagaggaaatagagataacaacccactttcaaagaaaaaggagagtaagagacaacatgaccaagaagatggtcacgcaaagaacaatagggaagaaaaaacagagactgaataagagaggctatctaataagagcactgacattaaatacgatgaccaaagatgcagagagaggcaagttaaaaagaagggctatcgcaacacctgggatgcagattagaggtttcgtatactttgttgaaactttagctaggagcatttgcgaaaagcttgaacagtctgggctcccagtagggggcaatgaaaagaaggccaaattggcaaatgttgtgagaaagatgatgactaattcacaagatacagagatttctttcacaatcactggggacaacactaagtggaatgaaaatcaaaatcctcgaatgttcctggcgatgattacatatatcaccagaaatcaacccgagtggtttagaaacatcctgagcatggcacccataatgttctcaaataaaatggcaagactaggaaaagggtacatgttcgagagtaaaagaatgaagattcgaacacaaataccagcagaaatgctagcaagcattgatctgaagtatttcaatgaatcaacaaagaagaaaattgagaaaataaggcctcttttaatggatggcacagcatcactgagtcctgggatgatgatgggcatgttcaacatgctaagtacggtcttgggagtctcgatactgaatcttggacaaaagaaatacaccaagacaacatactggtgggatgggctccaatcatcagacgattttgctctcatagtgaatgcaccaaaccatgaaggaatacaagcaggagtggacagattctacaggacctgcaagttagtgggaatcaacatgagcaaaaagaagtcctatataaataagacagggacatttgaattcacaagctttttttatcgctatggatttgtggctaattttagcatggagctacccagctttggagtgtctggagtaaatgaatcagctgacatgagtattggagtaacagtgataaagaacaacatgataaacaatgaccttggacctgcaacggctcagatggctcttcaattgttcatcaaagactacagatacacatataggtgccataggggagacacacaaattcagacgagaagatcatttgagttgaagaagctatgggatcaaacccaatcaaaggtagggctattagtatcagatggaggaccaaacttatacaacatacggaatcttcacattcctgaagtctgcttaaaatgggagctaatggatgatgattatcggggaagactttgtaatcccctgaatccctttgtgagtcataaggagattgattctgtaaacaatgctgtggtaatgccagcccatggcccagccaaaagcatggaatatgatgccgttgctactacacattcctggattcccaagaggaatcgttctattctcaacacaagccaaaggggaattcttgaggatgaacagatgtaccagaagtgttgcaatttattcgagaaatttttccctagcagttcatataggagaccggttggaatttctagcatggtggaggccatggtgtctagggcccggattgatgccagagtcgactttgagtctggacggatcaagaaagaagagttctctgagatcatgaagatctgttccaccattgaagaactcagacggcaaaaataa

>H1N1_PB1_KX410217_A/Minnesota/38/2016

atggatgtcaatccgactctacttttcctaaaaattccagcacaaaatgccataagcaccacattcccttatactggagatcctccatacagccatggaacaggaacaggatacaccatggacacagtaaacagaacacaccaatactcagaaaaggggaagtggacaacaaacacagagactggtgcaccccagctcaacccgattgacggaccactacctgaagataatgaaccaagtgggtatgcacaaacagactgtgttctagaggctatggcttttcttgaaaaatcccatccaggaatatttgagaattcatgccttgaaacaatggaagttgttcaacaaacaagggtagataaactgactcaaggtcgccagacttatgattggacattaaacagaaatcaaccggcagcaactgcattggccaacaccatagaagtttttagatcgaatgacctaacagctaacgagtcaggaaggctaatagatttcttaaaggatgtgatggaatcaatgaacaaagaggaaatagagataacaacccactttcaaagaaaaaggagagtaagagacaacatgaccaagaagatggtcacgcaaagaacaatagggaagaaaaaacagagactgaataagagaggctatctaataagagcactgacattaaatacgatgaccaaagatgcagagagaggcaagttaaaaagaagggctatcgcaacacctgggatgcagattagaggtttcgtatactttgttgaaactttagctaggagcatttgcgaaaagcttgaacagtctgggctcccagtagggggcaatgaaaagaaggccaaattggcaaatgttgtgagaaagatgatgactaattcacaagatacagagatttctttcacaatcactggggacaacactaagtggaatgaaaatcaaaatcctcgaatgttcctggcgatgattacatatatcaccagaaatcaacccgagtggtttagaaacatcctgagcatggcacccataatgttctcaaataaaatggcaagactaggaaaagggtacatgttcgagagtaaaagaatgaagattcgaacacaaataccagcagaaatgctagcaagcattgatctgaagtatttcaatgaatcaacaaagaagaaaattgagaaaataaggcctcttttaatggatggcacagcatcactgagtcctgggatgatgatgggcatgttcaacatgctaagtacggtcttgggagtctcgatactgaatcttggacaaaagaaatacaccaagacaacatactggtgggatgggctccaatcatccgacgattttgctctcatagtgaatgcaccaaaccatgaaggaatacaagcaggagtggacagattctacaggacctgcaagttagtgggaatcaacatgagcaaaaagaagtcctatataaataagacagggacatttgaattcacaagctttttttatcgctatggatttgtggctaattttagcatggagctacccagctttggagtgtctggagtaaatgaatcagctgacatgagtattggagtaacagtgataaagaacaacatgataaacaatgaccttggacctgcaacggctcagatggctcttcaattgttcatcaaagactacagatacacatataggtgccataggggagacacacaaattcagacgagaagatcatttgagttgaagaagctatgggatcaaacccaatcaaaggtagggctattagtatcagatggaggaccaaacttatacaacatacggaatcttcacattcctgaagtctgcttaaaatgggagctaatggatgatgattatcggggaagactttgtaatcccctgaatccctttgtgagtcataaggagattgattctgtaaacaatgctgtggtaatgccagcccatggcccagccaaaagcatggaatatgatgccgttgctactacacattcctggattcccaagaggaatcgttctattctcaacacaagccaaaggggaattcttgaggatgaacagatgtaccagaagtgttgcaatttattcgagaaatttttccctagcagttcatataggagaccggttggaatttctagcatggtggaggctatggtgtctagggcccggattgatgccagagtcgactttgagtctggacggatcaagaaagaagagttctctgagatcatgaagatctgttccaccattgaagaactcagacggcaaaaataa

>H1N1_PB1_KX411241_A/North Carolina/38/2016

atggatgtcaatccgactctacttttcctaaaaattccagcacaaaatgccataagcaccacattcccttatactggagatcctccatacagccatggaacaggaacaggatacaccatggacacagtaaacagaacacaccaatactcagaaaaggggaagtggacaacaaacacagagactggtgcaccccagctcaacccgattgacggaccactacctgaagataatgaaccaagtgggtatgcacaaacagactgtgttctagaggctatggcttttcttgaagaatcccatccaggaatatttgagaattcatgccttgaaacaatggaagttgttcaacaaacaagggtagataaactgactcaaggtcgccagacttatgattggacattaaacagaaatcaaccggcagcaactgcattggccaacaccatagaagtttttagatcgaatgacctaacagctaacgagtcaggaaggctaatagatttcttaaaggatgtgatggaatcaatgaacaaagaggaaatagagataacaacccactttcaaagaaaaaggagagtaagagacaacatgaccaagaagatggtcacgcaaagaacaatagggaagaaaaaacagagactgaataagagaggctatctaataagagcactgacattaaatacgatgaccaaagatgcagagagaggcaagttaaaaagaagggctatcgcaacacctgggatgcagattagaggtttcgtatactttgttgaaactttagctaggagcatttgcgaaaagcttgaacagtctgggctcccagtagggggcaatgaaaagaaggccaaattggcaaatgttgtgagaaagatgatgactaattcacaagatacagagatttctttcacaatcactggggacaacactaagtggaatgaaaatcaaaatcctcgaatgttcctggcgatgattacatatatcaccagaaatcaacccgagtggtytagaaacatcctgagcatggcacccataatgttctcaaataaaatggcaagactaggaaaagggtacatgttcgagagtaaaagaatgaagattcgaacacaaataccagcagaaatgctagcaagcattgatctgaagtatttcaatgaatcaacaaagaagaaaattgagaaaataaggcctcttttaatggatggcacagcatcactgagtcctgggatgatgatgggcatgttcaacatgctaagtacggtcttgggagtctcgatactgaatcttggacaaaagaaatacaccaagacaacatactggtgggatgggctccaatcatccgacgattttgctctcatagtgaatgcaccaaaccatgaaggaatacaagcaggagtggacagattctacaggacctgcaagttagtgggaatcaacatgagcaaaaagaagtcctatataaataagacagggacatttgaattcacaagctttttttatcgctatggatttgtggctaattttagcatggagctacccagctttggagtgtctggagtaaatgaatcagctgacatgagtattggagtaacagtgataaagaacaacatgataaacaatgaccttggacctgcaacggctcagatggctcttcaattgttcatcaaagactacagatacacatataggtgccataggggagacacacaaattcagacgagaagatcatttgagttgaagaagctatgggatcaaacccaatcaaaggtagggctattagtatcagatggaggaccaaacttatacaacatacggaatcttcacattcctgaagtctgcttaaaatgggagctaatggatgatgattatcggggaagactttgtaatcccctgaatccctttgtgagtcataaggagattgattctgtaaacaatgctgtggtaatgccagcccatggcccagccaaaagcatggaatatgatgccgttgctactacacattcctggattcccaagaggaatcgttctattctcaacacaagccaaaggggaattcttgaggatgaacagatgtaccagaagtgttgcaatttattcgagaaatttttccctagcagttcatataggagaccggttggaatttctagcatggtggaggccatggtgtctagggcccggattgatgccagagtcgactttgagtctggacggatcaagaaagaagagttctctgagatcatgaagatctgttccaccattgaagaactcagacggcaaaaataa

>H1N1_PB1_KX411649_A/South Dakota/13/2016

atggatgtcaatccgactctacttttcctaaaaattccagcacaaaatgccataagcaccacattcccttatactggagatcctccatacagccatggaacaggaacaggatacaccatggacacagtaaacagaacacaccaatactcagaaaaggggaagtggacaacaaacacagagactggtgcaccccagctcaacccgattgacggaccactacctgaagataatgaaccaagtgggtatgcacaaacagactgtgttctagaggctatggcttttcttgaagaatcccatccaggaatatttgagaattcatgccttgaaacaatggaagttgttcaacaaacaagggtagataaactgactcaaggtcgccagacttatgattggacattaaacagaaatcaaccggcagcaactgcattggccaacaccatagaagtttttagatcgaatgacctaacagctaacgagtcaggaaggctaatagatttcttaaaggatgtgatggaatcaatgaacaaagaggaaatagagataacaacccactttcaaagaaaaaggagagtaagagacaacatgaccaagaagatggtcacgcaaagaacaatagggaagaaaaaacagagactgaataagagaggctatctaataagagcactgacattaaatacgatgaccaaagatgcagagagaggcaagttaaaaagaagggctatcgcaacacctgggatgcagattagaggtttcgtatactttgttgaaactttagctaggagcatttgcgaaaagcttgaacagtctgggctcccagtagggggcaatgaaaagaaggccaaattggcaaatgttgtgagaaagatgatgactaattcacaagatacagagatttctttcacaatcactggggacaacactaagtggaatgaaaatcaaaatcctcgaatgttcctggcgatgattacatatatcaccagaaatcaacctgagtggtttagaaacatcctgagcatggcacccataatgttctcaaataaaatggcaagactaggaaaagggtacatgttcgagagtaaaagaatgaagattcgaacacaaataccagcagaaatgctagcaagcattgatctgaagtatttcaatgaatcaacaaagaagaaaattgagaaaataaggcctcttttaatggatggcacagcatcactgagtcctgggatgatgatgggcatgttcaacatgctaagtacggtcttgggagtctcgatactgaatcttggacaaaagaaatacaccaagacaacatactggtgggatgggctccaatcatccgacgattttgctctcatagtgaatgcaccaaaccatgaaggaatacaagcaggagtggacagattctacaggacctgcaagttagtgggaatcaacatgagcaaaaagaagtcctatataaataagacagggacatttgaattcacaagctttttttatcgctatggatttgtggctaattttagcatggagctacccagctttggagtgtctggagtaaatgaatcagctgacatgagtattggagtaacagtgataaagaacaacatgataaacaatgaccttggacctgcaacggctcagatggctcttcaattattcatcaaagactacagatacacatataggtgccataggggagacacacaaattcagacgagaagatcatttgagttgaagaagctatgggatcaaacccaatcaaaggtagggctattagtatcagatggaggaccaaacttatacaacatacggaatcttcacattcctgaagtctgcttaaaatgggagctaatggatgatgattatcggggaagactttgtaatcccctgaatccctttgtgagtcataaggagattgattctgtaaacaatgctgtggtaatgccagcccatggcccagccaaaagcatggaatatgatgccgttgctactacacattcctggattcccaagaggaatcgttctattctcaacacaagccaaaggggaattcttgaggatgaacagatgtaccagaagtgttgcaatttattcgagaaatttttccctagcagttcatataggagaccggttggaatttctagcatggtggaggccatggtgtctagggcccggattgatgccagagtcgactttgagtctggacggatcaagaaagaagagttctctgagatcatgaagatctgttccaccattgaagaactcagacggcaaaaataa

>H1N1_PB1_KX411777_A/Pennsylvania/59/2016

atggatgtcaatccgactctacttttcctaaaaattccagcacaaaatgccataagcaccacattcccttatactggagatcctccatacagccatggaacaggaacaggatataccatggacacagtaaacagaacacaccaatactcagaaaaggggaagtggacaacaaacacagagactggtgcaccccagctcaacccgattgacggaccactacctgaagataatgaaccaagtgggtatgcacaaacagactgtgttctagaggctatggcttttcttgaagaatcccatccaggaatatttgagaattcatgccttgaaacaatggaagttgttcaacaaacaagggtagataaactgactcaaggtcgccagacttatgactggacagtaaacagaaatcaaccggcagcaactgcattggccaacaccatagaagtttttagatcgaatgacctaacagctaacgagtcaggaaggctaatagatttcttaaaggatgtgatggaatcaatgaacaaagaggaaatagagataacaacccactttcaaagaaaaaggagagtaagagacaacatgaccaagaagatggtcacgcaaagaacaatagggaagaaaaaacagagactgaataagagaggctatctaataagagcactgacattaaatacgatgaccaaagatgcagagagaggcaagttaaaaagaagggctatcgcaacacctgggatgcagattagaggtttcgtatactttgttgaaactttagctaggagcatttgcgaaaagcttgaacagtctgggctcccagtagggggcaatgaaaagaaggccaaattggcaaatgttgtgagaaagatgatgactaattcacaagatacagagatttctttcacaatcactggggacaacactaagtggaatgaaaatcaaaatcctcgaatgttcctggcgatgattacatatatcaccagaaatcaacccgagtggtttagaaacatcctgagcatggcacccataatgttctcaaataaaatggcaagactaggaaaagggtacatgttcgagagtaaaagaatgaagattcgaacacaaataccagcagaaatgctagcaagcattgatctgaagtatttcaatgaatcaacaaagaagaaaattgagaaaataaggcctcttttaatggatggcacagcatcactgagtcctgggatgatgatgggcatgttcaacatgctaagtacggtcttgggagtctcgatactgaatcttggacaaaagaaatacaccaagacaacatactggtgggatgggctccaatcatccgacgattttgctctcatagtgaatgcaccaaaccatgaaggaatacaagcaggagtggacagattctacaggacctgcaagttagtgggaattaacatgagcaaaaagaagtcctatataaataagacagggacatttgaattcacaagctttttttatcgctatggatttgtggctaattttagcatggagctacccagctttggagtgtctggagtaaatgaatcagctgacatgagtattggagtaacagtgataaagaacaacatgataaacaatgaccttggacctgcaacggctcagatggctcttcaattgttcatcaaagactacagatacacatataggtgccataggggagacacacaaattcagacgagaagatcatttgagttgaagaagctatgggatcaaacccaatcaaaggtagggctattagtatcagatggaggaccaaacttatacaacatacggaatcttcacattcctgaagtctgcttaaaatgggagctaatggatgatgattatcggggaagactttgtaatcccctgaatccctttgtgagtcataaggagattgattctgtaaacaatgctgtggtaatgccagcccatggcccagccaaaagcatggaatatgatgccgttgctactacacattcctggattcccaagaggaatcgttctattctcaacacaagccaaaggggaattcttgaggatgaacagatgtaccagaagtgttgcaatttattcgagaaatttttccctagcagttcatataggagaccggttggaatttctagcatggtggaggccatggtgtctagggcccggattgatgccagagtcgactttgagtctggacggatcaagaaagaagagttctctgagatcatgaagatctgttccaccattgaagaactcagacggcaaaaataa

>H1N1_PB1_KX775357_A/Arkhangelsk/CRIE-111/2016

atggatgtcaatccgactctacttttcctaaaaattccagcacaaaatgccataagcaccacattcccttatactggagatcctccatacagccatggaacaggaacaggatacaccatggacacagtaaacagaacacaccaatactcagaaaaggggaagtggacaacaaacacagagactggtgcaccccagctcaacccgattgacggaccactacctgaagataatgaaccaagtgggtatgcacaaacagactgtgttctagaggctatggcttttcttgaagaatcccatccaggaatatttgagaattcatgccttgaaacaatggaagttgttcaacaaacaagggtagataaactgactcaaggtcgccagacttatgactggacattaaacagaaatcaaccggcagcaactgcattggccaacaccatagaagtttttagatcgaatgacctaacagctaacgagtcaggaaggctaatagatttcttaaaggatgtgatggaatcaatgaacaaagaggaaatagagataacaacccactttcaaagaaaaaggagagtaagagacaacatgaccaagaagatggtcacgcaaagaacaatagggaagaaaaaacagagactgaataagagaggctatctaataagagcactgacattaaatacgatgaccaaagatgcagagagaggcaagttaaaaagaagggctatcgcaacacctgggatgcagattagaggtttcgtatactttgttgaaactttagctaggagcatttgcgaaaagcttgaacagtctgggctcccagtagggggcaatgaaaagaaggccaaattggcaaatgttgtgagaaagatgatgactaattcacaagatacagagatttctttcacaatcactggggacaacactaagtggaatgaaaatcaaaatcctcgaatgttcctggcgatgattacatatatcaccagaaatcaacccgagtggtttagaaacatcctgagcatggcacccataatgttctcaaataaaatggcaagactaggaaaagggtacatgttcgagagtaaaagaatgaagattcgaacacaaataccagcagaaatgctagcaagcattgatctgaagtatttcaatgaatcaacaaagaagaaaattgagaaaataaggcctcttttaatggatggcacagcatcactgagtcctgggatgatgatgggcatgttcaacatgctaagtacggtcttgggagtctcgatactgaatcttggacaaaagaaatacaccaagacaacatactggtgggatgggctccaatcatccgacgattttgctctcatagtgaatgcaccaaaccatgaaggaatacaagcaggagtggacagattctacaggacctgcaagttagtgggaatcaacatgagcaaaaagaagtcctatataaataagacagggacatttgaattcacaagctttttttatcgctatggatttgtggctaattttagcatggagctacccagctttggagtgtctggagtaaatgaatcagctgacatgagtattggagtaacagtgataaagaacaacatgataaacaatgaccttggacctgcaacggctcagatggctcttcaattgttcatcaaagactacagatacacatataggtgccataggggagacacacaaattcagacgagaagatcatttgagttgaagaagctatgggatcaaacccaatcaaaggtagggctattagtatcagatggaggaccaaacttatacaacatacggaatcttcacattcctgaagtctgcttaaaatgggagctaatggatgatgattatcggggaagactttgtaatcccctgaatccctttgtgagtcataaggagattgattctgtaaacaatgctgtggtaatgccagcccatggcccagccaaaagcatggaatatgatgccgttgctactacacattcctggattcccaagaggaatcgttctattctcaacacaagccaaaggggaattcttgaggatgaacagatgtaccagaagtgttgcaatttattcgagaaatttttccctagcagttcatataggagaccggttggaatttctagcatggtggaggccatggtgtctagggcccggattgatgccagagtcgactttgagtctggacggatcaagaaagaagagttctctgagatcatgaagatctgttccaccattgaagaactcagacggcaaaaataa

>H1N1_PB1_KX914970_A/Maryland/08/2016

atggatgtcaatccgactctacttttcctaaaaattccagcacaaaatgccataagcaccacattcccttatactggagatcctccatacagccatggaacaggaacaggatacaccatggacacagtaaacagaacacaccaatactcagaaaaggggaagtggacaacaaacacagagactggtgcaccccagctcaacccgattgacggaccactacctgaagataatgaaccaagtgggtatgcacaaacagactgtgttctagaggctatggcttttcttgaagaatcccatccaggaatatttgagaattcatgccttgaaacaatggaagttgttcaacaaacaagggtagataaactgactcaaggtcgccagacttatgattggacattaaacagaaatcaaccggcagcaactgcattggccaacaccatagaagtttttagatcgaatgacctaacagctaacgagtcaggaaggctaatagatttcttaaaggatgtgatggaatcaatgaacaaagaggaaatagagataacaacccactttcaaagaaaaaggagagtaagagacaacatgaccaagaagatggtcacgcaaagaacaatagggaagaaaaaacagagactgaataagagaggctatctaataagagcactgacattaaatacgatgaccaaagatgcagagagaggcaagttaaaaagaagggctatcgcaacacctgggatgcagattagaggtttcgtatactttgttgaaactttagctaggagcatttgcgaaaagcttgaacagtctgggctcccagtagggggcaatgaaaagaaggccaaattggcaaatgttgtgagaaagatgatgactaattcacaagatacagagatttctttcacaatcactggggacaacactaagtggaatgaaaatcaaaatcctcgaatgttcctggcgatgattacatatatcaccagaaatcaacccgagtggtttagaaacatcctgagcatggcacccataatgttctcaaataaaatggcaagactaggaaaagggtacatgttcgagagtaaaagaatgaagattcgaacacaaataccagcagaaatgctagcaagcattgatctgaagtatttcaatgaatcaacaaagaagaaaattgagaaaataaggcctcttttaatggatggcacagcatcactgagtcctgggatgatgatgggcatgttcaacatgctaagtacggtcttgggagtctcgatactgaatcttggacaaaagaaatacaccaagacaacatactggtgggatgggctccaatcatccgacgattttgctctcatagtgaatgcaccaaaccatgaaggaatacaagcaggagtggacagattctacaggacctgcaagttggtgggaatcaacatgagcaaaaagaagtcctatataaataagacagggacatttgaattcacaagctttttttatcgctatggatttgtggctaattttagcatggagctacccagctttggagtgtctggagtaaatgaatcagctgacatgagcattggagtaacagtgataaagaacaacatgataaacaatgaccttggacctgcaacggctcagatggctcttcaattattcatcaaagactacagatacacatataggtgccataggggagacacacaaattcagacgagaagatcatttgagttgaagaagctatgggatcaaacccaatcaaaggtagggctattagtatcagatggaggaccaaacttatacaacatacggaatcttcacattcctgaagtctgcttaaaatgggagctaatggatgatgattatcggggaagactttgtaatcccctgaatccctttgtgagtcataaggagattgattctgtaaacaatgctgtggtaatgccagcccatggcccagccaaaagcatggaatatgatgccgttgctactacacattcctggattcccaagaggaatcgttctattctcaacacaagccaaaggggaattcttgaggatgaacagatgtaccagaagtgttgcaatttattcgagaaatttttccctagcagttcatataggagaccggttggaatttctagcatggtggaggccatggtgtctagggcccggattgatgccagagtcgactttgagtctggacggatcaagaaagaagagttctctgagatcatgaagatctgttccaccattgaagaactcagacggcaaaaataa

>H1N1_PB1_KX915146_A/Indiana/39/2016

atggatgtcaatccgactctacttttcctaaaaattccagcacaaaatgccataagcaccacattcccttatactggagatcctccatacagccatggaacaggaacaggatacaccatggacacagtaaacagaacacaccaatactcagaaaaggggaagtggacaacaaacacagagactggtgcaccccagctcaacccgattgacggaccactacctgaagataatgaaccaagtgggtatgcacaaacagactgtgttctagaggctatggcttttcttgaagaatcccatccaggaatatttgagaattcatgccttgaaacaatggaagttgttcaacaaacaagggtagataaactgactcaaggtcgccagacttatgattggacattaaacagaaatcaaccggcagcaactgcattggccaacaccatagaagtttttagatcgaatgacctaacagctaacgagtcaggaaggctaatagatttcttaaaggatgtgatggaatcaatgaacaaagaggaaatagagataacaacccactttcaaagaaaaaggagagtaagagacaacatgaccaagaagatggtcacgcaaagaacaatagggaagaaaaaacagagactgaataagagaggctatctaataagagcactgacattaaatacgatgaccaaagatgcagagagaggcaaattaaaaagaagggctatcgcaacacctgggatgcagattagaggtttcgtatactttgttgaaactttagctaggagcatttgcgaaaagcttgaacagtctgggctcccagtagggggcaatgaaaagaaggccaaattggcaaatgttgtgagaaagatgatgactaattcacaagatacagagatttctttcacaatcactggggacaacactaagtggaatgaaaatcaaaatcctcgaatgttcctggcgatgattacatatatcaccagaaatcaacccgagtggtttagaaacatcctgagcatggcacccataatgttctcaaataaaatggcaagactaggaaaagggtacatgttcgagagtaaaagaatgaagattcgaacacaaataccagcagaaatgctagcaagcattgatctgaagtatttcaatgagtcaacaaagaagaaaattgagaaaataaggcctcttttaatggatggcacagcatcactgagtcctgggatgatgatgggcatgttcaacatgctaagtacggtcttgggagtctcgatactgaatcttggacaaaagaaatacaccaagacaacatactggtgggatgggctccaatcatccgacgattttgctctcatagtgaatgcaccaaaccatgaaggaatacaagcaggagtggacagattctacaggacctgcaagttagtgggaatcaacatgagcaaaaagaagtcctatataaataagacagggacatttgaattcacaagctttttttatcgctatggatttgtggctaattttagcatggagctacccagctttggagtgtctggagtaaatgaatcagctgacatgagtattggagtaacagtgataaagaacaacatgataaacaatgaccttggacctgcaacggctcagatggctcttcaattgttcatcaaagactacagatacacatataggtgccataggggagacacacaaattcagacgagaagatcatttgagttgaagaagctatgggatcaaacccaatcaaaggtagggctattagtatcagatggaggaccaaacttatacaacatacggaatcttcacattcctgaagtctgcttaaaatgggagctaatggatgatgattatcggggaagactttgtaatcccctgaatccctttgtgagtcataaggagattgattctgtaaacaatgctgtggtaatgccagcccatggcccagccaaaagcatggaatatgatgccgttgctactacacattcctggattcccaagaggaatcgttctattctcaacacaagccaaaggggaattcttgaggatgaacagatgtaccagaagtgttgcaatttattcgagaaatttttccctagcagttcatataggagaccggttggaatttctagcatggtggaggccatggtgtctagggcccggattgatgccagagtcgactttgagtctggacggatcaagaaagaagagttctctgagatcatgaagatctgttccaccattgaagaactcagacggcaaaaataa

>H1N1_PB1_KX915890_A/Connecticut/22/2016

atggatgtcaatccgaccctacttttcctaaaaattccagcacaaaatgccataagcaccacattcccttatactggagatcctccatacagccatggaacaggaacaggatacaccatggacacagtaaacagaacacaccaatactcagaaaaggggaagtggacaacaaacacagagactggtgcaccccagctcaacccgattgacggaccactacctgaagataatgaaccaagtgggtatgcacaaacagactgtgttctagaggctatggctttccttgaagaatcccatccaggaatatttgagaattcatgccttgaaacaatggaagttgttcaacaaacaagggtagataaactgactcaaggtcgccagacttatgattggacattaaacagaaatcaaccggcagcaactgcattggccaacaccatagaagtttttagatcgaatgacctaacagctaacgagtcaggaaggctaatagatttcttaaaggatgtgatggaatcaatgaacaaagaggaaatagagataacaacccactttcaaagaaaaaggagagtaagagacaacatgaccaagaagatggtcacgcaaagaacaatagggaagaaaaaacagagactgaataagagaggctatctaataagagcactgacattaaatacgatgaccaaagatgcagagagaggcaagttaaaaagaagggctatcgcaacacctgggatgcagattagaggtttcgtatactttgttgaaactttagctaggagcatttgcgaaaagcttgaacagtctgggctcccagtagggggcaatgaaaagaaggccaaattggcaaatgttgtgagaaagatgatgactaattcacaagatacagagatttctttcacaatcactggggacaacactaagtggaatgaaaatcaaaatcctcgaatgttcctggcgatgattacatatatcaccagaaatcaacccgagtggtttagaaacatcctgagcatggcacccataatgttctcaaataaaatggcaagactaggaaaagggtacatgttcgagagtaaaagaatgaagattcgaacacaaataccagcagaaatgctagcaagcattgatctgaagtatttcaatgaatcaacaaagaagaaaattgagaaaataaggcctcttttaatggatggcacagcatcactgagtcctgggatgatgatgggcatgttcaacatgctaagtacggtcttgggagtctcaatactgaatcttggacaaaagaaatacaccaagacaacatactggtgggatgggctccaatcatccgacgattttgctctcatagtgaatgcaccaaaccatgaaggaatacaagcaggagtggacagattctacaggacctgcaagttagtgggaatcaacatgagcaaaaagaagtcctatataaataagacagggacatttgaattcacaagctttttttatcgctatggatttgtggctaattttagcatggagctacccagctttggagtgtctggagtaaatgaatcagctgacatgagtattggagtgacagtgataaagaacaacatgataaacaatgaccttggacctgcaacggctcagatggctcttcaattgttcatcaaagactacagatacacatataggtgccataggggagacacacaaattcagacgagaagatcatttgagttgaagaagctatgggatcaaacccaatcaaaggtagggctattagtatcagatggaggaccaaacttatacaacatacggaatcttcacattcctgaagtctgcttaaaatgggagctaatggatgatgattatcggggaagactttgtaatcccctgaatccctttgtgagtcataaggagattgattctgtaaacaatgctgtggtaatgccagcccatggcccagccaaaagcatggaatatgatgccgttgctactacacattcctggattcccaagaggaatcgttctattctcaacacaagccaaaggggaatccttgaggatgaacagatgtaccagaagtgttgcaatttattcgagaaatttttccctagcagttcatataggagaccggttggaatttctagcatggtggaggccatggtgtctagggcccggattgatgccagagtcgactttgagtctggacggatcaagaaagaagagttctctgagatcatgaagatctgttccaccattgaagaactcagacggcaaaaataa

>H1N1_PB1_KX916018_A/North Carolina/48/2016

atggatgtcaatccgactctacttttcctaaaaattccagcacaaaatgccataagcaccacattcccttatactggagatcctccatacagccatggaacaggaacaggatacaccatggacacagtaaacagaacacaccaatactcagaaaaggggaagtggacaacaaacacagagactggtgcaccccagctcaacccgattgacggaccactacctgaagataatgaaccaagtgggtatgcacaaacagactgtgttctagaggctatggcttttcttgaagaatcccatccaggaatatttgagaattcatgccttgaaacaatggaagttgttcaacaaacaagggtagataaactgactcaaggtcgccagacttatgattggacattaaacagaaatcaaccggcagcaactgcattggccaacaccatagaagtttttagatcgaatgacctaacagctaacgagtcaggaaggctaatagatttcttaaaggatgtgatggaatcaatgaacaaagaagaaatagagataacaacccactttcaaagaaaaaggagagtaagagacaacatgaccaagaagatggtcacgcaaagaacaatagggaagaaaaaacagagactgaataagagaggctatctaataagagcactgacattaaatacgatgaccaaagatgcagagagaggcaagttaaaaagaagggctatcgcaacacctgggatgcagattagaggtttcgtatactttgttgaaactttagctaggagcatttgcgaaaagcttgaacagtctgggctcccagtagggggcaatgaaaagaaggccaaattggcaaatgttgtgagaaagatgatgactaattcacaagatacagagatttctttcacaatcactggggacaacactaagtggaatgaaaatcaaaatcctcgaatgttcctggcgatgattacatatatcaccagaaatcaacccgagtggtttagaaacatcctgagcatggcacccataatgttctcaaataaaatggcaagactaggaaaagggtacatgttcgagagtaaaagaatgaagattcgaacacaaataccagcagaaatgctagcaagcattgatctgaagtatttcaatgaatcaacaaagaagaaaattgagaaaataaggcctcttttaatggatggcacagcatcactgagtcctgggatgatgatgggcatgttcaacatgctaagtacggtcttgggagtctcgatactgaatcttggacaaaagaaatacaccaagacaacatactggtgggatgggctccaatcatccgacgattttgctctcatagtgaatgcaccaaaccatgaaggaatacaagcaggagtggacagattctacaggacttgcaagttagtggggatcaacatgagcaaaaagaagtcctatataaataagacagggacatttgaattcacaagctttttttatcgctatggatttgtggctaattttagcatggagctacctagctttggagtgtctggagtaaatgaatcagctgacatgagtattggagtaacagtgataaagaacaacatgataaacaatgaccttggacctgcaacggctcagatggctcttcaattgttcatcaaagactacagatacacatataggtgccataggggagacacacaaattcagacgagaagatcatttgagttgaagaagctatgggatcaaacccaatcaaaggtagggctattagtatcagatggaggaccaaacttatacaacatacggaatcttcacattcctgaagtctgcttaaaatgggagctaatggatgatgattatcggggaagactttgtaatcccctgaatccctttgtgagtcataaggagattgattctgtaaacaatgctgtggtaatgccagcccatggcccagccaaaagcatggaatatgatgccgttgctactacacattcctggattcccaagaggaatcgttctattctcaacacaagccaaaggggaattcttgaggatgaacagatgtaccagaagtgttgcaatttattcgagaaatttttccctagcagttcatataggagaccggttggaatttctagcatggtggaggccatggtgtctagggcccggattgatgccagagtcgactttgagtctggacggatcaagaaagaagagttctctgagatcatgaagatctgttccaccattgaagaactcagacggcaaaaataa

>H1N1_PB1_KX918450_A/Arizona/03/2016

atggatgtcaatccgactctacttttcctaaaaattccagcacaaaatgccataagcaccacattcccttatactggagatcccccatacagccatggaacaggaacaggatacaccatggacacagtaaacagaacacaccaatactcagaaaaggggaagtggacaacaaacacagagactggtgcaccccagctcaacccgattgatggaccactacctgaggataatgaaccaagtgggtatgcacagacagattgtgtcctagaggctatggctttccttgaagaatcccacccaggaatatttgagaattcatgccttgaaacaatggaagttgttcaacaaacaagagtagataaactgactcaaggtcgccagacttatgattggacattaaacagaaatcaaccagcagcaactgcattggcaaacaccatagaagtctttagatcgaatgacctaacagctaacgagtcaggaaggctaatagatttcttaaaggatgtaatggaatcaatgaataaagaggaaatagagataacaacccactttcaaagaaaaaggagagtaagagacaacatgaccaagaagatggtcacgcaaagaacaatagggaagaaaaaacagagactgaataagagaggctatctaataagagcactgacattaaatacgatgaccaaagatgcagagagaggcaagttaaaaagaagggctatcgcaacacctggaatgcagattagaggtttcgtatactttgttgaaactttagctaggagcatttgcgaaaagcttgaacagtctgggctcccagtagggggcaatgaaaagaaggccaaattggcaaatgttgtgagaaagatgatgactaattcacaagatacagagatttctttcacaatcactggagacaacactaaatggaatgaaaatcaaaatcctcgaatgttcctggcaatgattacatatatcaccagaaatcaacccgagtggtttagaaacatcctgagcatggcacccataatgttctcaaataaaatggcaagactggggaaaggttacatgttcgagagtaaaagaatgaaaattcgaacacaaataccagcagaaatgctagcaagcattgatctgaagtacttcaatgaaccaacaaagaagaaaattgagaaaataaggcctcttttaatggatggcacagcatcactgagtcctgggatgatgatgggcatgttcaacatgctaagtacggtcttgggagtctcgatactgaatcttggacaaaagaaatacaccaagacaacatactggtgggatgggctccaatcatccgacgattttgctctcatagtgaatgcaccaaaccacgaaggaatacaagcaggagtggacagattctacaggacctgcaagttagtgggaatcaacatgagcaaaaagaagtcttatataaataagacagggacatttgaattcacaagctttttttatcgctatggatttgtggctaattttagcatggagctacccagctttggagtgtctggagtaaatgaatcagctgacatgagtattggagtaacagtgataaagaacaacatgataaacaatgacattggacctgcaacggcccagatggctcttcaattgttcatcaaagactacagatacacatataggtgccacaggggagacacacaaattcagacgagaagatcatttgaattaaagaagctatgggatcaaacccaatcaaaggtagggctattagtatcagatggaggaccaaacttatacaacatacggaatcttcacattcctgaagtctgcttaaaatgggagctaatggatgatgattatcggggaagactttgtaatcccctgaatccctttgtgagtcataaggagattgattctgtaaacaatgccgtggtaatgccagcccatggtccagccaaaagcatggaatatgatgccgttgcaactacacattcctggattcccaagaggaatcgttctatcctcaacacaagccaaaggggaattcttgaggatgaacagatgtaccagaagtgttgcaatctattcgagaaatttttccctagcagttcatataggagaccggttggaatttctagcatggtggaggccatggtgtctagggcccggattgatgccagggtcgactttgagtctggacggatcaagaaagaagagttctctgagatcatgaagatctgttccaccattgaagaactcagacggcaaaaataa

>H1N1_PB1_KX918666_A/Pennsylvania/29/2016

atggatgtcaatccgactctacttttcctaaaaattccagcacaaaatgccataagcaccacattcccttatactggagatcctccatacagccatggaacaggaacaggatacaccatggacacagtaaacagaacacaccaatactcagaaaaggggaagtggacaacaaacacagagactggtgcaccccagctcaacccgattgacggaccactacctgaagataatgaaccaagtgggtatgcacaaacagactgtgttctagaggctatggcttttcttgaagaatcccatccaggaatatttgagaattcatgccttgaaacaatggaagttgttcaacaaacaagggtagataaactgactcaaggtcgccagacttatgattggacattaaacagaaatcaaccggcagcaactgcattggccaacaccatagaagtttttagatcgaatgacctaacagctaacgagtcaggaaggctaatagatttcttaaaggatgtgatggaatcaatgaacaaagaggaaatagagataacaacccactttcaaagaaaaaggagagtaagagacaacatgaccaagaagatggtcacgcaaagaacaatagggaagaaaaaacagagactgaataagagaggctatctaataagagcactgacattaaatacgatgaccaaagatgcagagagaggcaagttaaaaagaagggctatcgcaacacctgggatgcagattagaggtttcgtatactttgttgaaactttagctaggagcatttgcgaaaagcttgaacagtctgggctcccagtagggggcaatgaaaagaaggccaaattggcaaatgttgtgagaaagatgatgactaattcacaagatacagagatttctttcacaatcactggggacaacactaagtggaatgaaaatcaaaatcctcgaatgttcctggcgatgattacatatatcaccagaaatcaacccgagtggtttagaaacatcctgagcatggcacccataatgttctcaaataaaatggcaagactaggaaaagggtacatgttcgagagtaaaagaatgaagattcgaacacaaataccagcagaaatgctagcaagcattgatctgaagtatttcaatgaatcaacaaagaagaaaattgagaaaataaggcctcttttaatggatggcacagcatcactgagtcctgggatgatgatgggcatgttcaacatgctaagtacggtcttgggagtctcgatactgaatcttggacaaaagaaatacaccaagacaacatactggtgggatgggctccaatcatccgacgattttgctctcatagtgaatgcaccaaaccatgaaggaatacaagcaggagtggacagattctacaggacttgcaagttagtgggaatcaacatgagcaaaaagaagtcctatataaataagacagggacatttgaattcacaagctttttttatcgctatggatttgtggctaattttagcatggagctacccagctttggagtgtctggagtaaatgaatcagctgacatgagtattggagtaacagtgataaagaacaacatgataaacaatgaccttggacctgcaacggctcagatggctcttcaattgttcatcaaagactacagatacacatataggtgccataggggagacacacaaattcagacgagaagatcatttgagttgaagaagctatgggatcaaacccaatcaaaggtagggctattagtatcagatggaggaccaaacttatacaacatacggaatcttcacattcctgaagtctgcttaaaatgggagctaatggatgatgattatcggggaagactttgtaatcccctgaatccctttgtgagtcataaggagattgattctgtaaacaatgctgtggtaatgccagcccatggcccagccaaaagcatggaatatgatgccgttgctactacacattcctggattcccaagaggaatcgttctattctcaacacaagccaaaggggaattcttgaggatgaacagatgtaccagaagtgttgcaatttattcgagaaatttttccctagcagttcatataggagaccggttggaatttctagcatggtggaggccatggtgtctagggcccggattgatgccagagtcgactttgagtctggacggatcaagaaagaagagttctctgagatcatgaagatctgttccaccattgaagaactcagacggcaaaaataa

>H1N1_PB1_KX918706_A/Virginia/56/2016

atggatgtcaatccgactctacttttcctaaaaattccagcacaaaatgccataagcaccacattcccttatactggagatcctccatacagccatggaacaggaacaggatacaccatggacacagtaaacagaacacaccaatactcagaaaaggggaagtggacaacaaacacagagactggtgcaccccagctcaacccggttgacggaccactacctggagataatgaaccaagtgggtatgcacaaacagactgtgttctagaggctatggcttttcttgaagaatcccatccaggaatatttgagaattcatgccttgaaacaatggaagttgttcaacaaacaagggtagataaactgactcaaggtcgccagacttatgattggacattaaacagaaatcaaccggcagcaactgcattggccaacaccatagaagtttttagatcgaatgacctaacagctaacgagtcaggaaggctaatagatttcttaaaggatgtgatggaatcaatgaacaaagaggaaatagagataacaacccactttcaaagaaaaaggagagtaagagacaacatgaccaagaagatggtcacgcaaagaacaatagggaagaaaaaacagagactgaacaagagaggctatctaataagagcactgacattaaatacgatgaccaaagatgcagagagaggcaagttaaaaagaagggctatcgcaacacctgggatgcagattagaggtttcgtatactttgttgaaactttagctaggagcatttgcgaaaagcttgaacagtctgggctcccagtagggggcaatgaaaagaaggccaaattggcaaatgttgtgagaaagatgatgactaattcacaagatacagagatttctttcacaatcactggagacaacactaagtggaatgaaaatcaaaatcctcgaatgttcctggcgatgattacatatatcaccagaaatcaacccgagtggtttagaaacatcctgagcatggcacccataatgttctcaaataaaatggcaagactaggaaaagggtacatgttcgagagtaaaagaatgaagattcgaacacaaataccagcagaaatgctagcaagcattgatctgaagtatttcaatgaatcaacaaagaagaaaattgagaaaataaggcctcttttaatggatggcacagcatcactgagtcctgggatgatgatgggcatgttcaacatgctaagtacggtcttgggagtctcgatactgaatcttggacaaaagaaatacaccaagacaacatactggtgggatgggctccaatcatccgacgattttgctctcatagtgaatgcaccaaaccatgaaggaatacaagcaggagtggacagattctacaggacctgcaagttagtgggaatcaacatgagcaaaaagaagtcctatataaataagacagggacatttgaattcacaagctttttttatcgctatggatttgtggctaattttagcatggagctacccagctttggagtgtctggagtaaatgaatcagctgacatgagtattggagtaacagtgataaagaacaacatgataaacaatgaccttggacctgcaacggctcagatggctcttcaattgttcatcaaagactacagatacacatataggtgccataggggagacacacaaattcagacgagaagatcatttgagttgaagaagctatgggatcaaacccaatcaaaggtagggctattagtatcagatggaggaccaaacttatacaacatacggaatcttcacattcctgaagtctgcttaaagtgggagctaatggatgatgattatcggggaagactttgtaatcccctgaatccctttgtgagtcataaggagattgattctgtaaacaatgctgtggtaatgccagcccatggcccagccaaaagcatggaatatgatgccgttgctactacacattcctggattcccaagaggaatcgttctattctcaacacaagccaaaggggaattcttgaggatgaacagatgtaccagaagtgttgcaatttattcgagaaatttttccctagcagttcatataggagaccggttggaatttctagcatggtggaggccatggtgtctagggcccggattgatgccagagtcgactttgagtctggacggatcaagaaagaagagttctctgagatcatgaagatctgttccaccattgaagaactcagacggcaaaaataa

>H1N1_PB1_KX918842_A/Georgia/32/2016

atggatgtcaatccgactctacttttcctaaaaattccagcacaaaatgccataagcaccacattcccttatactggagatcctccatacagccatggaacaggaacaggatacaccatggacacagtaaacagaacacaccaatactcagaaaaggggaagtggacaacaaacacagagactggtgcaccccagcttaacccgattgacggaccactacctgaagataatgaaccaagtgggtatgcacaaacagactgtgttttagaggctatggcttttcttgaagaatcccatccaggaatatttgagaattcatgccttgaaacaatggaagttgttcaacaaacaagggtagataaactgactcaaggtcgccagacttatgattggacattaaacagaaatcaaccggcagcaactgcattggccaacaccatagaagtttttagatcgaatgacctaacagctaacgagtcaggaaggctaatagatttcttaaaggatgtgatggaatcaatgaacaaagaggaaatagagataacaacccactttcaaagaaaaaggagagtaagagacaacatgaccaagaagatggtcacgcaaagaacaatagggaagaaaaaacagagactgaataagagaggctatctaataagagcactgacattaaatacgatgaccaaagatgcagagagaggcaagttaaaaagaagggctatcgcaacacctgggatgcagattagaggtttcgtatactttgttgaaactttagctaggagcatttgcgaaaagcttgaacagtctgggctcccagtagggggcaatgaaaagaaggccaaattggcaaatgttgtgagaaagatgatgactaattcacaagatacagagatttctttcacaatcactggggacaacactaagtggaatgaaaatcaaaatcctcgaatgttcctggcgatgattacatatatcaccagaaatcaacccgagtggtttagaaacatcctgagcatggcacccataatgttctcaaataaaatggcaagactaggaaaagggtacatgttcgagagtaaaagaatgaagattcgaacacaaataccagcagaaatgctagcaagcattgatctgaagtatttcaatgaatcaacaaagaagaaaattgagaaaataaggcctcttttaatggatggcacagcatcactgagtcctgggatgatgatgggcatgttcaacatgctaagtacggtcttgggagtctcgatactgaatcttggacaaaagaaatacaccaagacaacatactggtgggatgggctccaatcatccgacgattttgctctcatagtgaatgcaccaaaccatgaaggaatacaagcaggagtggacagattctacaggacctgcaagttagtgggaatcaacatgagcaaaaagaagtcctatataaataagacagggacatttgaattcacaagctttttttatcgctatggatttgtggctaattttagcatggagctacccagctttggagtgtctggagtaaatgaatcagctgacatgagtattggagtaacagtgataaagaacaacatgataaacaatgaccttggacctgcaacggctcagatggctcttcaattgttcatcaaagactacagatacacatataggtgccataggggagacacacaaattcagacgagaagatcatttgagttgaagaagctatgggatcaaacccaatcaaaggtagggctattagtatcagatggaggaccaaacttatacaacatacggaatcttcacattcctgaagtctgcttaaaatgggagctaatggatgatgattatcggggaagactttgtaatcccctgaatccctttgtgagtcataaggagattgattctgtaaacaatgctgtggtaatgccagcccatggcccagccaaaagcatggaatatgatgccgttgctactacacattcctggattcccaagaggaatcgttctattctcaacacaagccaaaggggaattcttgaggatgaacagatgtaccagaagtgttgcaatttattcgagaaatttttccctagcagttcatataggagaccggttggaatttctagcatggtggaggccatggtgtctagggcccggattgatgccagagtcgactttgagtctggacggatcaagaaagaagagttctctgagatcatgaagatctgttccaccattgaagaactcagacggcaaaaataa

>H1N1_PB1_KX949112_A/Massachusetts/06/2016

atggatgtcaatccgactctacttttcctaaaaattccagcacaaaatgccataagcaccacattcccttatactggagatcctccatacagccatggaacaggaacaggatacaccatggacacagtaaacagaacacaccaatactcagaaaaggggaagtggacaacaaacacagagactggtgcaccccagctcaacccgattgacggaccactacctgaagataatgaaccaagtgggtatgcacaaacagactgtgttctagaggctatggcttttcttgaagaatcccatccaggaatatttgagaattcatgccttgaaacaatggaagttgttcaacaaacaagggtagataaactgactcaaggtcgccagacttatgattggacattaaacagaaatcaaccggcagcaactgcattggccaacaccatagaagtttttagatcgaatgacctaacagctaacgagtcaggaaggctaatagatttcttaaaggatgtgatggaatcaatgaacaaagaggaaatagagataacaacccactttcaaagaaaaaggagagtaagagacaacatgaccaagaagatggtcacgcaaagaacaatagggaagaaaaaacagagactgagtaagagaggctatctaataagagcactgacattaaatacgatgaccaaagatgcagagagaggcaagttaaaaagaagggctatcgcaacacctgggatgcagattagaggtttcgtatactttgttgaaactttagctaggagcatttgcgaaaagcttgaacagtctgggctcccagtagggggcaatgaaaagaaggccaaattggcaaatgttgtgagaaagatgatgactaattcacaagatacagagatttctttcacaatcactggggacaacactaagtggaatgaaaatcaaaatcctcgaatgttcctggcgatgattacatatatcaccagaaatcaacccgagtggtttagaaacatcctgagcatggcacccataatgttctcaaataaaatggcaagactaggaaaagggtacatgttcgagagtaaaagaatgaagattcgaacacaaataccagcagaaatgctagcaagcattgatctgaagtatttcaatgaatcaacaaagaagaaaattgagaaaataaggcctcttttaatggatggcacagcatcactgagtcctgggatgatgatgggcatgttcaacatgctaagtacggtcttgggagtctcgatactgaatcttggacaaaagaaatacaccaagacaacatactggtgggatgggctccaatcatccgacgattttgctctcatagtgaatgcaccaaaccatgaaggaatacaagcaggagtggacagattctacaggacctgcaagttagtgggaatcaacatgagcaaaaagaagtcctatataaataagacagggacatttgaattcacaagctttttttatcgctatggatttgtggctaattttagcatggagctacccagctttggagtgtctggagtaaatgaatcagctgacatgagtattggagtaacagtgataaagaacaacatgataaacaatgaccttggacctgcaacggctcagatggctcttcaattgttcatcaaagactacagatacacatataggtgccataggggagacacacaaattcagacgagaagatcatttgagttgaagaagctatgggatcaaacccaatcaaaggtagggctattagtatcagatggaggaccaaacttatacaacatacggaatcttcacattcctgaagtctgcttaaaatgggagctaatggatgatgattatcggggaagactttgtaatcccctgaatccctttgtgagtcataaggagattgattctgtaaacaatgctgtggtaatgccagcccatggcccagccaaaagcatggaatatgatgccgttgctactacacattcctggattcccaagaggaatcgttctattctcaacacaagccaaaggggaattcttgaggatgaacagatgtaccagaagtgttgcaatttattcgagaaatttttccctagcagttcatataggagaccggttggaatttctagcatggtggaggccatggtgtctagggcccggattgatgccagagtcgactttgagtctggacggatcaagaaagaagagttctctgagatcatgaagatctgttccaccattgaagaactcagacggcaaaaataa

>H1N1_PB1_KY044685_A/Alabama/01/2016

atggatgtcaatccgactctacttttcctaaaaattccagcacaaaatgccataagcaccacattcccttatactggagatcctccatacagccatggaacaggaacaggatacaccatggacacagtaaacagaacacaccaatactcagaaaaggggaagtggacaacaaacacagagactggtgcaccccagctcaacccgattgacggaccactacctgaagataatgaaccaagtgggtatgcacaaacagactgtgttctagaggctatggcttttcttgaagaatcccatccaggaatatttgagaattcatgccttgaaacaatggaagttgttcaacaaacaagggtagataaactgactcaaggtcgccagacttatgattggacattaaacagaaatcaaccggcagcaactgcattggccaacaccatagaagtttttagatcgaatgacctaacagctaacgagtcaggaaggctaatagatttcttaaaggatgtgatggaatcaatgaacaaagaggaaatagagataacaacccactttcaaagaaaaaggagagtaagagacaacatgaccaagaagatggtcacgcaaagaacaatagggaagaaaaaacagagactgaataagagaggctatctaataagagcactgacattaaatacgatgaccaaagatgcagagagaggcaagttaaaaagaagggctatcgcaacacctgggatgcagattagaggtttcgtatactttgttgaaactttagctaggagcatttgcgaaaagcttgaacagtctgggctcccagtagggggcaatgaaaagaaggccaaattggcaaatgttgtgagaaagatgatgactaattcacaagatacagagatttctttcacaatcactggggacaacactaagtggaatgaaaatcaaaatcctcgaatgttcctggcgatgattacatatatcaccagaaatcaacccgagtggtttagaaacatcctgagcatggcacccataatgttctcaaataaaatggcaagactaggaaaagggtacatgttcgagagtaaaagaatgaagattcgaacacaaataccagcagaaatgctagcaagcattgatctgaagtatttcaatgaatcaacaaaaaagaaaattgagaaaataaggcctcttttaatggatggcacagcatcactgagtcctgggatgatgatgggcatgttcaacatgctaagtacggtcttgggagtctcgatactgaatcttggacaaaagaaatacaccaagacaacatactggtgggatgggctccaatcatccgacgattttgctctcatagtgaatgcaccaaaccatgaaggaatacaagcaggagtggacagattctacaggacctgcaagttagtgggaatcaacatgagcaaaaagaagtcctatataaataagacagggacatttgaattcacaagctttttttatcgctatggatttgtggctaattttagcatggagctacccagctttggagtgtctggagtaaatgaatcagctgacatgagtattggagtaacagtgataaagaacaacatgataaacaatgaccttggacctgcaacggctcagatggctcttcaattgttcatcaaagactacagatacacatataggtgccataggggagacacacaaattcagacgagaagatcatttgagttgaagaagctatgggatcaaacccaatcaaaggtagggctattagtatcagatggaggaccaaacttatacaacatacggaatcttcacattcctgaagtctgcttaaaatgggagctaatggatgatgattatcggggaagactttgtaatcccctgaatccctttgtgagtcataaggagattgattctgtaaacaatgctgtggtaatgccagcccatggcccagccaaaagcatggaatatgatgccgttgctactacacattcctggattcccaagaggaatcgttctattctcaacacaagccaaaggggaattcttgaggatgaacagatgtaccagaagtgttgcaatttattcgagaaatttttccctagcagttcatataggagaccggttggaatttctagcatggtggaggccatggtgtctagggcccggattgatgccagagtcgactttgagtctggacggatcaagaaagaagagttctctgagatcatgaagatctgttccaccattgaagaactcagacggcaaaaataa

>H1N1_PB1_KY509799_A/Chungnam/29/2016

atggatgtcaatccgactctacttttcctaaaaattccagcacaaaatgccataagcaccacattcccttatactggagatcctccatacagccatggaacaggaacaggatacaccatggacacagtaaacagaacacaccaatactcagaaaagggaaagtggacaacaaacacagagactggtgcaccccagctcaacccgattgatggaccactacctgaggataatgaaccaagtgggtatgcacaaacagactgtgttctagaggctatggctttccttgaagaatcccacccaggaatatttgagaattcatgccttgaaacaatggaagttgttcaacaaacaagggtagataaactgactcaaggtcgccagacttatgattggacattaaacagaaatcaaccggcagcaactgcattggccaacaccatagaagtctttagatcgaatgacctaacagctaacgagtcaggaaggctaatagatttcttaaaggatgtaatggaatcaatgaacaaagaggaaatagagataacaacccactttcaaagaaaaaggagagtaagagacaacatgaccaagaagatggtcacgcaaagaacaatagggaagaaaaaacaaagactgaataagagaggctatctaataagagcactgacattaaatacgatgaccaaagatgcagagagaggcaagttaaaaagaagggctatcgcaacacctgggatgcagattagaggtttcgtatactttgttgaaactttagctaggagcatttgcgaaaagcttgaacagtctggactcccagtagggggcaatgaaaagaaggccaaattggcaaatgttgtgagaaagatgatgactaattcacaagatacagagatttctttcacaatcactggggacaacactaagtcgaatgaaaatcaaaatcttcgaatgttcctggcgatgattacatatatcaccagaaatcaacccgagtggtttagaaacatcctgagcatggcacccataatgttctcaaataaaatggcaagactagggaaagggtacatgttcgagagtaaaagaatgaagattcgaacacaaataccagcagaaatgctagcaagcattgacctgaagtacttcaatgaatcaacaaagaagaaaattgagaaaataaggcctcttttaatggatggcacagcatcactgagtcctgggatgatgatgggcatgttcaacatgctaagtacggtcttgggagtctcgatactgaatcttggacaaaagaaatacatcaagacaacatactggtgggatgggctccaatcatccgacgattttgctctcatagtgaatgcaccaaaccatgaaggaatacaagcaggagtggacagattctgcaggacctgcaagttagtgggaatcaacatgagcaaaaagaagtcctatataaataagacagggacatttgaattcacaagctttttttatcgctatggatttgtgggtaattttagcatggagctacccagctttggagtgtctggagtaaatgaatcagctgacatgagtattggagtaacagtgataaagaacaacatgataaacaatgacctcggacctgcaacggcccagatggctcttcaattgttcatcaaagactacagatacacatataggtgccataggggagacacacaaattcagacgagaagatcatttgagttaaagaagctatgggatcaaacccaatcaaaggtagggctattagtatcagatggaggaccaaacttatacaatatacggaatcttcacattcctgaagtctgcttaaaatgggagctaatggatgatgattatcggggaagactttgtaatcccctgaatccctttgtgagtcataaggagattgattctgtaaacaatgctgtggtaatgccagcccatggtccagccaaaagcatggaatatgatgccgttgcaactacacattcctggattcccaagaggaatcgttctattctcaacacaagccaaaggggaattcttgaggatgaacagatgtaccagaagtgttgcaatctattcgagaaatttttccctagcagttcatataggagaccggttggaatttctagcatggtggaggccatggtgtctagggcccggattgatgccagggtcgactttgagtctggacggatcaagaaagaagagttctctgagatcatgaagatctgttccaccattgaagaactcagacggcaaaaataa

>H1N1_PB1_KY487742_A/Keelung/0068/2016

atggatgtcaatccgactctacttttcctaaaaattccagcacaaaatgccataagcaccacattcccttatactggagatcctccatacagccatggaacaggaacaggatacaccatggacacagtaaacagaacacaccaatactcagaaaaggggaagtggacaacaaacacagagactggtgcaccccagctcaacccgattgacggaccactacctgaagataatgaaccaagtgggtatgcacaaacagactgtgttctagaggctatggcttttcttgaagaatcccatccaggaatatttgagaattcatgccttgaaacaatggaagttgttcaacaaacaagggtagataaactgactcaaggtcgccagacttatgattggacattaaacagaaatcaaccggcagcaactgcattggccaacaccatagaagtttttagatcgaatgacctaacagctaacgagtcaggaaggctaatagatttcttaaaggatgtgatggaatcaatgaacaaagaggaaatagagataacaacccactttcaaagaaaaaggagagtaagagacaacatgacaaagaagatggtcacgcaaagaacaatagggaagaaaaaacagagactgaataagagaggctatctaataagagcactgacattaaatacgatgaccaaagatgcagagagaggcaagttaaaaagaagggctatcgcaacacctgggatgcagattagaggtttcgtatactttgttgaaactttagctaggagcatttgcgaaaagcttgaacagtctgggctcccagtagggggcaatgaaaagaaggccaaattggcaaatgttgtgagaaagatgatgactaattcacaagatacagagatttctttcacaatcactggggacaacactaagtggaatgaaaatcaaaatcctcgaatgttcctggcgatgattacatatataaccagaaatcaacccgagtggtttagaaacatcctgagcatggcacccataatgttctcaaataaaatggcaagactaggaaaagggtacatgttcgagagtaaaagaatgaaaattcgaacacaaataccagcagaaatgctagcaagcattgatctgaagtatttcaatgaatcaacaaagaagaaaattgagaaaataaggcctcttttaatggatggcacagcatcactgagtcctgggatgatgatgggcatgttcaacatgctaagtacggtcttgggagtctcgatactgaatcttggacaaaagaaatacaccaagacaacatactggtgggatgggctccaatcatccgacgattttgctctcatagtgaatgcaccaaaccatgaaggaatacaagcaggagtggacagattctacaggacctgcaagttagtgggaatcaacatgagcaaaaagaagtcctatataaataagacagggacatttgaattcacaagctttttttatcgctatggatttgtggctaattttagcatggagctacccagctttggagtgtctggagtaaatgaatcagctgacatgagtattggagtaacagtgataaagaacaacatgataaacaatgaccttggacctgcaacggctcagatggctcttcaattgttcatcaaagactacagatacacatataggtgccataggggagacacacaaattcagacgagaagatcatttgagttgaagaagctatgggatcaaacccaatcaaaggtagggctattagtatcagatggaggaccaaacttatacaacatacggaatcttcacattcctgaagtctgcttaaaatgggagttaatggatgatgattatcggggaagactttgtaatcccctgaatccctttgtgagtcataaggagattgattctgtaaacaatgctgtggtaatgccagcccatggcccagccaaaagcatggaatatgatgccgttgctactacacattcctggattcccaagaggaatcgttctattctcaacacaagccaaagggggattcttgaggatgaacagatgtaccagaagtgttgcaatttattcgagaaatttttccctagcagttcatataggagaccggttggaatttctagcatggtggaggccatggtgtctagggcccggattgatgccagagtcgactttgagtctggacggatcaagaaagaagagttctctgagatcatgaagatctgttccaccattgaagaactcagacggcaaaaataa

>H1N1_PB1_KY487770_A/Baltimore/0163/2016

atggatgtcaatccgactctacttttcctaaaaattccagcacaaaatgccataagcaccacattcccttatactggagatcctccatacagccatggaacaggaacaggatacaccatggacacagtaaacagaacacaccaatactcagaaaaggggaagtggacaacaaacacagagactggtgcaccccagctcaacccgattgacggaccactacctgaagataatgaaccaagtgggtatgcacaaacagactgtgttctagaggctatggcttttcttgaagaatcccatccaggaatatttgagaattcatgccttgaaacaatggaagttgttcaacaaacaagggtagataaactgactcaaggtcgccagacttatgattggacattaaacagaaatcaaccggcagcaactgcattggccaacaccatagaagtttttagatcgaatgacctaacagctaacgagtcaggaaggctaatagatttcttaaaggatgtgatggaatcaatgaacaaagaggaaatagagataacaacccactttcaaagaaaaaggagagtaagagacaacatgaccaagaagatggtcacgcaaagaacaatagggaagaaaaaacagagactgaataagagaggctatctaataagagcactgacattgaatacgatgaccaaagatgcagagagaggcaagttaaaaagaagggctatcgcaacacctgggatgcagattagaggtttcgtatactttgttgaaactttagctaggagcatttgcgaaaagcttgaacagtctgggctcccagtagggggcaatgaaaagaaggccaaattggcaaatgttgtgagaaagatgatgactaattcacaagatacagagatttctttcacaatcactggggacaacactaagtggaatgaaaatcaaaatcctcgaatgttcctggcgatgattacatatatcaccagaaatcaacccgagtggtttagaaacatcctgagcatggcacccataatgttctcaaataaaatggcaagactaggaaaagggtacatgttcgagagtaaaagaatgaagattcgaacacaaataccagcagaaatgctagcaagcattgatctgaagtatttcaatgaatcaacaaagaagaaaattgagaaaataaggcctcttttaatggatggcacagcatcactgagtcctgggatgatgatgggcatgttcaacatgctaagtacggtcttgggagtctcgatactgaatcttggacaaaagaaatacaccaagacaacatactggtgggatgggctccaatcatccgacgattttgctctcatagtgaatgcaccaaaccatgaaggaatacaagcaggagtggacagattctacaggacctgcaagttagtgggaatcaacatgagcaaaaagaagtcctatataaataagacagggacatttgaattcacaagctttttttatcgctatggatttgtggctaattttagcatggagctacccagctttggagtgtctggagtaaatgaatcagctgacatgagtattggagtaacagtgataaagaacaacatgataaacaatgaccttggacctgcaacggctcagatggctcttcaattattcatcaaagactacagatacacatataggtgccataggggagacacacaaattcagacgagaagatcatttgagttgaagaagctatgggatcaaacccaatcaaaggtagggctattagtatcagatggaggaccaaacttatacaacatacggaatcttcacattcctgaagtctgcttaaaatgggagctaatggatgatgattatcggggaagactttgtaatcccctgaatccctttgtgagtcataaggagattgattctgtaaacaatgctgtggtaatgccagcccatggcccagccaaaagcatggaatatgatgccgttgctactacacattcctggattcccaagaggaatcgttctattctcaacacaagccaaaggggaattcttgaggatgaacagatgtaccagaagtgttgcaatttattcgagaaatttttccctagcagttcatataggagaccggttggaatttctagcatggtggaggccatggtgtctagggcccggattgatgccagagtcgactttgagtctggacggatcaagaaagaagagttctctgagatcatgaagatctgttccaccattgaagaactcagacggcaaaaataa

>H1N1_PB1_CY218855_A/Montana/04/2017

atggatgtcaatccgactctacttttcttaaaaattccagcacaaaatgccataagcaccacattcccttatactggagatcctccatatagccatggaacaggaacaggatacactatggacacagtaaacagaacacaccaatactcagaaaaggggaagtggacaacaaacacagagactggtgcaccccagctcaacccgattgatggaccactacctgaggataatgaaccaagtgggtatgcacaaacagactgtgttctagaggctatggctttccttgaagaatcccacccaggaatatttgagaattcatgccttgaaacaatggaagttgttcaacaaacaagggtagataaactgactcaaggtcgccagacttatgattggacattaaacagaaatcaaccggcagcaactgcattggccaacaccatagaagtctttagatcgaatgacctaacagctaacgagtcaggaaggctaatagatttcttaaaggatgtaatggaatcaatgaacaaagaggaaatagagataacaacccactttcaaagaaaaaggagagtaagagacaacatgaccaagaagatggtcacgcaaagaacaatagggaagaaaaaacagagactgaataagagaggctatctaataagagcactgacattaaatacgatgaccaaagatgcagagagaggcaagttaaaaagaagggctatcgcaacacctgggatgcagattagaggttttgtatactttgttgaaactttagctaggagcatttgcgaaaagcttgaacagtctgggctcccagtagggggcaatgaaaagaaggccaaattggcaaatgttgtgagaaagatgatgactaattcacaagatacagagatttctttcacaatcactggggataacactaagtggaatgaaaatcaaaatcctcgaatgttcctggcgatgattacatatatcaccagaaatcaacccgagtggtttagaaacatcctgagcatggcacccataatgttctcaaataaaatggcaagactgggaaaagggtacatgttcgagagtaaacgaatgaagattcgaacacaaataccagcagaaatgctagcaagcattgatctgaagtacttcaatgaatcaacaaagaagaaaattgagaaaatacggcctcttttaatggatggcacagcatcactgagtcctgggatgatgatgggcatgttcaacatgctaagtacggtattgggagtctcgatactgaatcttggacaaaagaaatacaccaagacaacatactggtgggatgggctccaatcatccgacgattttgctctcatagtgaatgcaccaaaccacgaaggaatacaagcaggagtggacaggttctacaggacctgcaagttagtgggaatcaacatgagcaaaaagaagtcctatataaataagacagggacatttgaattcacaagctttttttatcgctatggatttgtggctaattttagcatggagctacccagctttggagtgtctggagtaaatgaatcagctgacatgagtattggagtaacagtgataaagaacaacatgataaacaatgaccttggacctgcaacggcccagatggctcttcaattgttcatcaaagactacagatacacatataggtgccataggggggacacacaaattcagacgagaagatcttttgagttaaagaagctatgggatcaaacccaatcaaaggtagggctattagtatcagatggaggaccaaacttatacaacatacggaatcttcacattcctgaagtctgcttaaaatgggagctaatggatgatgattatcggggaagactttgtaatcccctgaatccctttgtgagtcataaggaaattgattctataaacaatgccgtggtaatgccagcccatggtccagccaaaagcatggaatatgatgccgttgcaactacacattcctggattcccaagaggaatcgttctattctcaacacaagccaaaggggaattcttgaggatgaacagatgtaccagaagtgttgcaatctattcgagaaatttttccctagcagttcatataggagaccggttggaatttctagcatggtggaggccatggtgtctagggcccggattgatgccagagtcgactttgagtctggacggatcaagaaagaagagttctctgagatcatgaagatctgttccaccattgaagaactcagacggcaaaaataa

>H1N1_PB1_CY220949_A/North Dakota/08/2017

atggatgtcaatccgactctacttttcttaaaaattccagcacaaaatgccataagcaccacattcccttatactggagatcctccatatagccatggaacaggaacaggatacaccatggacacagtaaacagaacacaccaatactcagaaaaggggaagtggacaacaaacacagagactggtgcaccccagctcaacccgattgatggaccactacctgaggataatgaaccaagtgggtatgcacaaacagactgtgttctagaggctatggctttccttgaagaatcccacccaggaatatttgagaattcatgccttgaaacaatggaagttgttcaacaaacaagggtagataaactgactcaaggtcgccagacttatgattggacattaaacagaaatcaaccggcagcaactgcattggccaacaccatagaagtctttagatcgaatgacctaacagctaacgagtcaggaaggctaatagatttcttaaaggatgtaatggaatcaatgaacaaagaggaaatagagataacaacccactttcaaagaaaaaggagagtaagagacaacatgaccaagaagatggtcacgcaaagaacaatagggaagaaaaaacagagactgaataagagaggctatctaataagagcactgacattaaatacgatgaccaaagatgcagagagaggcaagttaaaaagaagggctatcgcaacacctgggatgcagattagaggtttcgtatactttgttgaaactttagctaggagcatttgcgaaaagcttgaacagtctgggctcccagtagggggcaatgaaaagaaggccaaattggcaaatgttgtgagaaagatgatgactaattcacaagatacagagatttctttcacaatcactggggacaacactaagtggaatgaaaatcaaaatcctcgaatgttcctggcgatgatcacatatatcaccagaaatcaacccgagtggtttagaaacatcctgagcatggcacccataatgttctcaaataaaatggcaagactggggaaagggtacatgttcgagagtaaaagaatgaagattcgaacacaaataccagcagagatgctagcaagcattgatctgaagtacttcaatgaatcaacaaagaagaaaattgagaaaataaggcctcttttaatggatggcacagcatcactgagtcctgggatgatgatgggcatgttcaacatgctaagtacggtattgggagtctcgatactgaatcttggacaaaagaaatacaccaagacaacatactggtgggatgggctccaatcatccgacgattttgctctcatagtgaatgcaccaaaccacgaaggaatacaagcaggagtggacaggttctacaggacctgcaagttagtgggaatcaacatgagcaaaaagaagtcctatataaataagaccgggacatttgaattcacaagctttttttatcgctatggatttgtggctaattttagcatggagctacccagctttggagtgtctggagtaaatgaatcagctgacatgagtattggagtaacagtgataaagaacaacatgataaacaatgaccttggacctgcaacggcccagatggctcttcaattgttcatcaaagactacagatacacatataggtgccataggggagacacacaaattcagacgagaagatcttttgagttaaagaagctatgggatcaaacccaatcaaaggtagggctattagtatcagatggaggaccaaacttatacaacatacggaatcttcacattcctgaagtctgcttaaaatgggagctaatggatgatgattatcggggaagactttgtaatcccctgaatccctttgtgagtcataaggagattgattctgtaaacaatgccgtggtaatgccagcccatggtccagccaaaagcatggaatatgatgccgttgcaactacacattcctggatccccaagaggaatcgttctattctcaacacaagccaaaggggaattcttgaggatgaacagatgtaccagaagtgttgcaatctattcgagaaatttttccctagcagttcatataggagaccggttggaatttctagcatggtggaggccatggtgtctagggcccggattgatgccagagtcgactttgagtctggacggatcaagaaagaagagttctctgagatcatgaagatctgttccaccattgaagaactcagacggcaaaaataa

>H1N1_PB1_KY925409_A/Passo Fundo/LACENRS-1034/2016

atggatgtcaatccgactctacttttcctaaaaattccagcacaaaatgccataagcaccacattcccttatactggagatcctccatacagccatggaacaggaacaggatacaccatggacacagtaaacagaacacaccaatactcagaaaaggggaagtggacaacaaacacagagactggtgcaccccagctcaacccgattgacggaccactacctgaagataatgaaccaagtgggtatgcacaaacagactgtgttttagaggctatggcttttcttgaagaatcccatccaggaatatttgagaattcatgccttgaaacaatggaagttgtccaacaaacaagggtagataaactgactcaaggtcgccagacttatgattggacattaaacagaaatcaaccggcagcaactgcattggccaacaccatagaagtttttagatcgaatgacctaacagctaacgagtcaggaaggctaatagatttcttaaaggatgtgatggaatcaatgaacaaagaggaaatagagataacaacccactttcaaagaaaaaggagagtaagagacaacatgaccaagaagatggtcacgcaaagaacaatagggaagaaaaaacagagactgaataagagaggctatctaataagagcactgacattaaatacgatgaccaaagatgcagagagaggcaagttaaaaagaagggctatcgcaacacctgggatgcagattagaggtttcgtatactttgttgaaactttagctaggagcatttgcgaaaagcttgaacagtctgggctcccagtagggggcaatgaaaagaaggccaaattggcaaatgttgtgagaaagatgatgactaattcacaagatacagagatttctttcacaatcactggggacaacactaagtggaatgaaaatcaaaatcctcgaatgttcctggcgatgattacatatatcaccagaaatcaacccgagtggtttagaaacatcctgagcatggcacccataatgttctcaaataaaatggcaagactaggaaaagggtacatgttcgagagtaaaagaatgaagattcgaacacaaataccagcagaaatgctagcaagcattgatctgaagtatttcaatgaatcaacaaagaagaaaattgagaaaataaggcctcttttaatggatggcacagcatcactgagtcctgggatgatgatgggcatgttcaatatgctaagtacggtcttgggagtctcgatactgaatcttggacaaaagaaatacaccaagacaacatactggtgggatgggctccaatcatccgacgattttgctctcatagtgaatgcaccaaaccatgaaggaatacaagcaggagtggacagattctacaggacctgcaagttagtgggaatcaacatgagcaaaaagaagtcctatataaataagacagggacatttgaattcacaagctttttttatcgctatggatttgtggctaattttagcatggagctacccagctttggagtgtctggagtaaatgaatcagctgacatgagtattggagtaacagtgataaagaacaacatgataaacaatgaccttggacctgcaacggctcagatggctcttcaattgttcatcaaagactacagatacacatataggtgccataggggagacacacaaattcagacgagaagatcatttgagttgaagaagctatgggatcaaacccaatcaaaggtagggctattagtatcagatggaggaccaaacttatacaacatacggaatcttcacattcctgaagtctgcttaaaatgggagctaatggatgatgattatcggggaagactttgtaatcccctgaatccctttgtgagtcataaggagattgattctgtaaacaatgctgtggtaatgccagcccatggcccagccaaaagcatggaatatgatgccgttgctactacacattcctggagtcccaagaggaatcgttctattctcaacacaagccaaaggggaattcttgaggatgaacagatgtaccagaagtgttgcaatttattcgagaaatttttccctagcagttcatataggagaccggttggaatttctagcatggtggaggccatggtgtctagggcccggattgatgccagagtcgactttgagtctggacggatcaagaaagaagagttctctgagatcatgaagatctgttccaccattgaagaactcagacggcaaaaataa

>H1N1_PB1_KY925738_A/Porto Alegre/LACENRS-2380/2013

atggatgtcaatccgactctacttttcctaaaaattccagcacaaaatgccataagcaccacattcccttatactggagatcctccatacagccatggaacaggaacaggatacaccatggacacagtaaacagaacacaccaatactcagaaaaggggaagtggacaacaaacacagagactggtgcaccccagctcaacccgattgatggaccactacctgaggataatgaaccaagtgggtatgcacaaacagactgtgttctagaggctatggctttccttgaagaatcccacccaggaatatttgagaattcatgccttgaaacaatggaagttgttcaacaaacaagggtagataaactgactcaaggtcgccagacttatgattggacattaaacagaaatcaaccggcagcaactgcattggccaacaccatagaagtctttagatcgaatgacctaacagctaacgagtcaggaaggctaatagatttcttaaaggatgtaatggaatcaatgaacaaagaggaaatagagataacaacccactttcaaagaaaaaggagagtaagagacaacatgaccaagaagatggtcacgcaaagaacaatagggaagaaaaaacagagactgaataagagaggctatctaataagagcactgacattaaatacgatgaccaaagatgcagagagaggcaagttaaaaagaagggctatcgcaacacctgggatgcagattagaggtttcgtatactttgttgaaactttagctaggagcatttgcgaaaagcttgaacagtctgggctcccagtagggggcaatgaaaagaaggccaaattggcaaatgttgtgagaaagatgatgactaattcacaagatacagagatttctttcacaatcactggggacaacactaagtggaatgaaaatcaaaatcctcgaatgttcctagcgatgattacatatatcaccagaaatcaacccgagtggtttagaaacatcctgagcatggcacccataatgttctcaaataaaatggcaagactagggaaagggtacatgttcgagagtaaaagaatgaagattcgaacacaaataccagcagaaatgctagcaagcattgatctgaagtacttcaatgaatcaacaaagaagaaaattgagaaaataaggcctcttttaatggatggcacagcatcactgagtcctgggatgatgatgggcatgttcaacatgctaagtacggtcttgggagtctcgatactgaatcttggacaaaagaaatacaccaagacaacatactggtgggatgggctccaatcatccgacgattttgctctcatagtgaatgcaccaaaccatgaaggaatacaagcaggagtggacagattctacaggacctgcaagttagtgggaatcaacatgagcaaaaagaagtcctatataaataagacagggacatttgaattcacaagctttttttatcgctatggatttgtggctaattttagcatggagctacccagctttggagtgtctggagtaaatgaatcagctgacatgagtattggagtaacagtgataaagaacaacatgataaacaatgaccttggacctgcaacggcccagatggctcttcaattgttcatcaaagactacagatacacatataggtgccataggggagacacacaaattcagacgagaagatcatttgagttaaagaagctatgggatcaaacccaatcaaaggtagggctattagtatcagatggaggaccaaacttatacaacatacggaatcttcacattcctgaagtctgcttaaaatgggagctaatggatgatgattatcggggaagactttgtaatcccctgaatccctttgtgagtcataaggagattgattctgtaaacaatgctgtggtaatgccagcccatggtccagccaaaagcatggaatatgatgccgttgcaactacacattcctggattcccaagaggaatcgttctattctcaacacaagccaaaggggaattcttgaggatgaacagatgtaccagaagtgttgcaatctattcgagaaatttttccctagcagttcatataggagaccggttggaatttctagcatggtggaggccatggtgtctagggcccggattgatgccagggtcgactttgagtctggacggatcaagaaagaagagttctctgagatcatgaagatctgttccaccattgaagaactcagacggcaaaaataa

>H1N1_PB1_KY925324_A/Porto Alegre/LACENRS-289/2016

atggatgtcaatccgactctacttttcctaaaaattccagcacaaaatgccataagcaccacattcccttatactggagatcctccatacagccatggaacaggaacaggatacaccatggacacagtaaacagaacacaccaatactcagaaaaggggaagtggacaacaaacacagagactggtgcaccccagctcaacccgattgacggaccactacctgaagataatgaaccaagtgggtatgcacaaacagactgtgttctagaggctatggcttttcttgaagaatcccatccaggaatatttgagaattcatgccttgaaacaatggaagttgttcaacaaacaagggtagataaactaactcaaggtcgccagacttatgattggacattaaacagaaatcaaccggcagcaactgcattggccaacaccatagaagtttttagatcgaatgacctaacagctaacgagtcaggaaggctaatagatttcttaaaggatgtgatggaatcaatgaacaaagaggaaatagagataacaacccactttcaaagaaaaaggagagtaagagacaacatgaccaagaagatggtcacgcaaagaacaatagggaagaaaaaacagagactgaataagagaggctatctaataagagcactgacattaaatacgatgaccaaagatgcagagagaggcaagttaaaaagaagggctatcgcaacacctgggatgcagattagaggtttcgtatactttgttgaaactttagctaggagcatttgcgaaaagcttgaacagtctgggctcccagtagggggcaatgaaaagaaggccaaattggcaaatgttgtgagaaagatgatgactaattcacaagatacagagatttctttcacaatcactggggacaacactaagtggaatgaaaatcaaaatcctcgaatgttcctggcgatgattacatatatcaccagaaatcaacccgagtggtttagaaacatcctgagcatggcacccataatgttctcaaataaaatggcaagactaggaaaagggtacatgttcgagagtaaaagaatgaagattcgaacacaaataccagcagaaatgctagcaagcattgatctgaagtatttcaatgaatcaacaaagaagaaaattgagaaaataaggcctcttttaatggatggcacagcatcactgagtcctgggatgatgatgggcatgttcaacatgctaagtacggtcttgggagtctcgatactgaatcttggacaaaagaaatacaccaagacaacatactggtgggatgggctccaatcatccgacgattttgctctcatagtgaatgcaccaaaccatgaaggaatacaagcaggagttaacagattctacaggacctgcaagttagtgggaatcaacatgagcaaaaagaagtcctatataaataagacagggacatttgaattcacaagctttttttatcgctatggatttgtggctaattttagcatggagctacccagctttggagtgtctggagtaaatgaatcagctgacatgagcattggagtaacagtgataaagaacaacatgataaacaatgaccttggacctgcaacggctcagatggctcttcaattgttcatcaaagactacagatacacatataggtgccataggggagacacacaaatccagacgagaagatcatttgagttgaagaagctatgggatcaaacccaatcaaaggtagggctattagtatcagatggaggaccaaacttgtacaacatacggaatcttcacattcctgaagtctgcttaaaatgggagctaatggatgatgattatcggggaagactttgtaatcccctgaatccctttgtgagtcataaggagattgattctgtaaacaatgctgtggtaatgccagcccatggcccagccaaaagcatggaatatgatgccgttgctactacacattcctggattcccaagaggaatcgttctattctcaacacaagccaaaggggaattcttgaggatgaacagatgtaccagaagtgttgcaatttattcgagaaatttttccctagcagttcatataggagaccggttggaatttctagcatggtggaggccatggtgtctagggcccggattgatgccagagtcgactttgagtctggacggatcaagaaagaagagttctctgagatcatgaagatctgttccaccattgaagaactcagacggcaaaaataa

>H1N1_PB1_CY231216_A/Idaho/22/2017

atggatgtcaatccgactctacttttcctaaaaattccagcacaaaatgccataagcaccacattcccttatactggagatcctccatacagccatggaacaggaacaggatacaccatggacacagtaaacagaacacaccaatactcagaaaaggggaagtggacaacaaacacagagactggtgcaccccagctcaacccgattgacggaccactacctgaagataatgaaccaagtgggtatgcacaaacagactgtgttctagaggctatggcttttcttgaagaatcccatccaggaatatttgggaattcatgccttgaaacaatggaagttgttcaacaaacaagggtagataaactgactcaaggtcgccagacttatgattggacattaaacagaaatcaaccggcagcaactgcattggccaacaccatagaagtttttagatcgaatgacctaacagctaacgagtcaggaaggctaatagatttcttaaaggatgtgatggaatcaatgaacaaagaagaaatagagataacaacccactttcaaagaaaaaggagagtaagagacaacatgaccaagaagatggtcacgcaaagaacaatagggaagaaaaaacagagactgaataagagaggctatctaataagagcactgacattaaatacgatgaccaaagatgcagagagaggcaagttaaaaagaagggctatcgcaacacctgggatgcagattagaggtttcgtatactttgttgaaactttagctaggagcatttgcgaaaagcttgaacagtctgggctcccagtagggggcaatgaaaagaaggccaaattggcaaatgttgtgagaaagatgatgactaattcacaagatacagagatttctttcacaatcactggggacaacactaagtggaatgaaaatcaaaatcctcgaatgttcctggcgatgattacatatatcaccagaaatcaacccgagtggtttagaaacatcctgagcatggcacccataatgttctcaaataaaatggcaagactaggaaaagggtacatgttcgagagtaaaagaatgaagattcgaacacaaataccagcagaaatgctagcaagcattgatctgaagtatttcaatgaatcaacaaagaagaaaattgagaaaataaggcctcttttaatggatggcacagcatcactgagtcctgggatgatgatgggcatgttcaacatgctaagtacggtcttgggagtctcgatactgaatcttggacaaaagaaatacaccaaaacaacatactggtgggatgggctccaatcatccgacgattttgctctcatagtgaatgcaccaaaccatgaaggaatacaagcaggagtggacagattctacaggacttgcaagttagtgggaatcaacatgagcaaaaagaagtcctatataaataagacagggacatttgaattcacaagctttttttatcgctatggatttgtggctaattttagcatggagctacctagctttggagtgtctggagtaaatgaatcagctgacatgagtattggagtaacagtgataaagaacaacatgataaacaatgaccttggacctgcaacggctcaaatggctcttcaattgttcatcaaagactacagatacacatataggtgccataggggagacacacaaattcagacgagaagatcatttgagttgaagaagctatgggatcaaacccaatcaaaggtagggctattagtatcagatggaggaccaaacttatacaacatacggaatcttcacattcctgaagtctgcttaaaatgggagctaatggatgatgattatcggggaagactttgtaatcccctgaatccctttgtgagtcataaggagattgattctgtaaacaatgctgtggtaataccagcccatggcccagccaaaagcctggaatatgatgccgttgctactacacattcctggattcccaagaggaatcgttctattctcaacacaagccaaaggggaattcttgaggatgaacagatgtaccagaagtgttgcaatttattcgagaaatttttccctagcagttcatataggagaccggttggaatttctagcatggtggaggccatggtatctagggcccggattgatgccagagtcgactttgagtctggacggatcaagaaagaagagttctctgagatcatgaagatctgttccaccattgaagaactcagacggcaaaaataa

>H1N1_PB1_CY010914_A/Memphis/7/1980

atggatgtcaatccgaccttacttttcttaaaagtgccagcacaaaatgctataagcacaactttcccttatactggagaccctccttacagccatgggacaggaacagggtacaccatggatacagtcaacaggacacatcagtactcagaaaggggaagatggacaaaaaacaccgaaactggagcaccgcaactcaacccgattgatgggccactaccaaaagacaatgaaccaagtggttatgcccaaacagattgtgtattagaagcaatggctttccttgaggaatcccatcctggtatttttgaaaactcgtgtattgaaacgatggaggttgttcagcaaacacgagtggacaaactgacacaaggcagacagacctatgactggactctaaatagaaaccagcctgctgctacagcattggccaacaccatagaagtgttcagatcaaacggcctcatggcaaatgaatctggaaggctaatagacttccttaaagatgtaatggagtcaatggacagagaagaagtagagatcacaactcattttcagagaaagagaagagtgagggacaatgtaactaagaaaatggtgacacaaagaacaataggcaaaaagaaacatagattaaacaaaaggagttatctaattagggcattaaccctgaacacaatgaccaaagatgctgagagggggaaactaaaacgtagagcaattgcaaccccagggatgcaaataagggggtttgtatactttgttgagacactggcaagaagtatatgtgaaaaacttgaacaatcagggttgccagttggaggaaatgaaaagaaagcaaagttagcaaatgttgtaaggaagatgatgaccaattctcaggacactgaaatttctttcaccatcactggagataacacaaaatggaacgaaaatcagaaccctagaatgtttttggccatgatcacatatataaccagaaatcagcccgaatggttcagaaatattctaagtattgctccaataatgttctcaaacaaaatggcgagactaggtaaagggtacatgtttgaaagcaagagtatgaaacttagaactcaaatacctgcagaaatgctagcaaacatcgatttgaagtatttcaatgattcaacaagaaagaaaattgaaaaaatccggccactcttaatagatggaactgcatcactgagccctggaatgatgatgggcatgttcaatatgttaagcactgtattgggcgtctccatcctgaatcttggacaaaagagatacaccaagactacttactggtgggatggtcttcaatcgtctgatgattttgctctgattgtgaatgcacccaactatgcaggaattcaagctggagttgacaggttttatcgaacctgtaagctgctcggaatcaatatgagcaagaaaaagtcttacatgaacagaacaggtacttttgaattcacgagctttttctatcgttatgggtttgttgccaatttcagcatggagcttcccagttttggggtgtctgggatcaacgagtctgcagacatgagtattggagtcactgtcatcaaaaacaatatgataaacaatgatcttggtccagcaaccgctcaaatggcccttcagttattcatcaaagattacaggtacacgtatcgatgccacagaggtgacacacaaatacaaacccgaagatcatttgaaataaagaaactgtgggatcaaacccgttccaaagctgggctgctggtctctgatggaggtcctaatttatacaacattagaaatctccacattcctgaagtctgcttgaaatgggaattgatggatgaggattaccaggggcgtttatgcaacccattgaacccatttgtcagtcataaagagattgaatcagtgaacaatgcagtgatgatgccggcacatggtccagccaaaaatatggagtatgatgctgttgcaacaacacactcctgggtccccaaaaggaatcgatccatcttgaatacgagccaaagaggaatacttgaggatgaacaaatgtatcaaaggtgctgcaatttatttgaaaaattcttccccagcagttcatacagaagaccagtcggaatatccagtatggtagaggctatggtttccagagcccgaattgatgcacggattgatttcgaatctggaaggataaagaaagaggagttcactgagatcatgaagacctgttccaccattgaagaactcagacggcaaaaatag

>H1N1_PB1_CY011590_A/Dunedin/2/2000

atggatgtcaatccgacattacttttcttaaaagtgccagcacaaaatgctataagcacaacttttccttatactggtgaccctccttacagccatgggacaggaacagggtacaccatggatacagtcaacaggacacatcagtactcagaaagaggaagatggacaaaaaataccgaaactggagcaccgcaactcaacccaattgatgggccactaccaaaagacaatgaaccaagtggctatgcccaaacagattgtgtattagaagcaatggctttccttgaggaatcccatcctggtatttttgaaaactcttgtattgaaacaatggaggttgttcagcaaacaagggtggacaaactgacacaaggcagacagacctatgactggactctaaataggaaccagcctgctgccacagcattggccaacactatagaagtgttcagatcaaacggcctcatagcaaatgaatctgggaggctaatagacttccttaaagatgtaatggagtcgatggacagagacgaagtagaggtcacaactcattttcagagaaagaggagagtgagagacaatgtaactaaaaaaatggtgacccaaagaacaataggcaaaaagaaacataaattagacaaaaaaagttacctaattagggcattaaccctgaacacaatgaccaaagatgctgagagggggaaactaaaacgcagagcaattgcaaccccaggaatgcaaataagggggtttgtatactttgttgagacactggcaagaagcatatgtgaaaagcttgaacaatcagggttgccagttggaggaaatgaaaagaaagcaaagttagcaaatgttgtaaggaagatgatgaccaactcccaggacactgaaatttctttcaccatcactggagataacacaaaatggaacgaaaatcaaaaccctagaatgttcttggccatgatcacatatataaccaaaaatcagcctgaatggttcagaaatattctaagtattgctccaataatgttttcaaacaaaatggcgagactaggtaaggggtacatgtttgaaagcaagagtatgaaactgagaactcaaatacctgcagagatgctagccaacatagatttgaaatatttcaatgattcaactaaaaagaaaattgaaaaaatccggccattattaatagatggaactgcatcattgagtcctggaatgatgatgggcatgttcaatatgttaagcaccgtcttgggcgtctccattctgaatcttgggcaaaagagatacaccaagactacttactggtgggatggtcttcaatcgtctgatgattttgctctgattgtgaatgcacccaactatgcaggaattcaagctggagttgacaggttttatcgaacctgtaagctgctcggaattaatatgagcaaaaagaagtcttacataaacagaacaggtacctttgaattcacgagctttttctatcgttatgggtttgttgccaatttcagcatggagcttcctagttttggggtgtctggggtcaatgaatctgcagacatgagtattggagtcactgtcatcaaaaacaatatgataaacaatgaccttggcccagcaactgctcaaatggcccttcagttatttataaaagattacaggtacacgtatcgatgccacagaggtgacacacaaatacaaacccgaagatcatttgagataaagaaactatgggaccaaacccgctccaaagctgggctgttggtctctgatgggggccccaatttatataacattagaaatctccatattcctgaagtctgcttgaaatgggagttgatggatgaggattaccaggggcgtttatgcaacccattgaacccgtttgtcagtcataaagagattgaatcagtgaacaatgcagtgatgatgccggcacatggtccagccaaaaatatggagtatgacgctgttgcaacaacacactcctgggttcccaaaaggaatcgatccattttgaatacgagccaaagggggatacttgaggatgagcaaatgtatcagaggtgctgcaatttatttgaaaaattcttcccaagtagctcatacagaagaccagttggaatatccagtatggtagaggctatggtttccagagcccgaattgatgcacggattgatttcgaatctggaaggataaagaaagaggaattcgctgagatcatgaagacctgttccaccattgaagacctcagacggcaaaaatag

>H1N1_PB1_CY016649_A/New South Wales/18/1999

atggatgtcaatccgacattacttttcttaaaagtgccagcacaaaatgctataagcacaacttttccttatactggtgaccctccttacagccatgggacaggaacagggtacaccatggatacagtcaacaggacacatcagtactcagaaagaggaagatggacaaaaaataccgaaactggagcaccgcaactcaacccaattgatgggccactaccaaaagacaatgaaccaagtggctatgcccaaacagattgtgtattagaagcaatggctttccttgaggaatcccatcctggtatttttgaaaactcttgtattgaaacaatggaggttgttcagcaaacaagggtggacaaactgacacaaggcagacagacctatgactggactctaaataggaaccagcctgctgctacagcattggccaacactatagaagtgttcagatcaaacggcctcatagcaaatgaatctgggaggctaatagacttccttaaagatgtaatggagtcgatggacagagacgaagtagagatcacaactcattttcaaagaaagaggagagtgagagacaatgtaactaaaaaaatggtgacccaaagaacaataggcaaaaagaaacataaattagacaaaagaagttacctaattagggcattaaccctgaacacaatgaccaaagatgctgagagggggaaactaaaacgcagagcaattgcaaccccaggaatgcaaataagggggtttgtatactttgttgagacactggcaagaagcatatgtgaaaagcttgaacaatcagggttgccagttggaggaaatgaaaagaaagcaaagttagcaaatgttgtaaggaagatgatgaccaactcccaggacactgaaatttctttcaccatcactggagataacacaaaatggaacgaaaatcaaaaccctagaatgttcttggccatgatcacatatataaccaaaaatcagcctgaatggttcagaaatattctaagtattgctccaataatgttttcaaacaaaatggcgagactaggtaaggggtacatgtttgaaagcaagagtatgaaactgagaactcaaatacctgcagagatgctagccaacatagatttgaaatatttcaatgattcaactaaaaagaaaattgaaaaaatccggccattattaatagatggaactgcatcattgagtcctggaatgatgatgggcatgttcaatatgttaagcaccgtcttgggcgtctccattctgaatcttgggcaaaagagatacaccaagactacttactggtgggatggtcttcaatcgtctgatgattttgctctgattgtgaatgcacccaactatgcaggaattcaagctggagttgacaggttttatcgaacctgtaagctgctcggaattaatatgagcaaaaagaagtcttacataaacagaacaggtacctttgaattcacgagctttttctatcgttatgggtttgttgccaatttcagcatggagcttcctagttttggggtgtctggggtcaatgaatctgcagacatgagtattggagtcactgtcatcaaaaacaatatgataaacaatgaccttggcccagcaactgctcaaatggcccttcagttatttataaaagattacaggtacacgtatcgatgccacagaggtgacacacaaatacaaacccggagatcatttgagataaagaaactatgggaccaaacccgctccaaagctgggctgttggtctctgatggaggccccaatttatataacattagaaatctccatattcctgaagtctgcttgaaatgggagttgatggatgaggattaccaggggcgtttatgcaacccattgaacccgtttgtcagtcataaagagattgaatcagtgaacaatgcagtgatgatgccggcacatggtccagccaaaaatatggagtatgacgctgttgcaacaacacactcctgggttcccaaaaggaatcgatccattttgaatacgagccaaagggggatacttgaggatgagcaaatgtatcagaggtgctgcaatttatttgaaaaattcttcccaagtagctcatacagaagaccagttggaatatccagtatggtagaggctatggtttccagagcccgaattgatgcacggattgatttcgaatctggaaggataaaaaaagaggaattcgctgagatcatgaagacctgttccaccattgaagacctcagacggcaaaaatag

>H1N1_PB1_CY017369_A/Memphis/20/1978

atggatgtcaatccgaccttacttttcttaaaagtgccagcacaaaatgctataagcacaactttcccttatactggagaccctccttacagccatgggacaggaacagggtacaccatggatacagtcaacaggacacatcagtactcagaaaggggaagatggacaaaaaacaccgaaactggagcaccgcaactcaacccgattgatgggccactaccaaaagacaatgaaccaagtggttatgcccaaacagattgtgtattagaagcaatggctttccttgaggaatcccatcctggtatttttgaaaactcgtgtattgaaacgatggaggttgttcagcaaacacgagtggacaaactgacacaaggcagacagacctatgactggactctaaatagaaaccagcctgctgctacagcattggccaacaccatagaagtgttcagatcaaatggcctcatggcaaatgaatctggaaggctaatagacttccttaaagatgtaatggagtcaatggacagagaagaagtggagatcacaactcattttcagagaaagagaagagtgagggacaatgtgactaagaaaatgatgacacagagaacaataggcaaaaagaaacagagattaaacaaaaggagttatctaattagggcattaaccctgaacacaatgaccaaagatgctgagagagggaaactaaaacgtagagcaattgcaaccccagggatgcaaataagggggtttgtatactttgttgagacactggcaagaagtatatgtgaaaaacttgaacaatcagggttgccagttggaggaaatgaaaagaaagcaaagttggcaaatgttgtaaggaagatgatgaccaattctcaggacactgaaatttctttcaccatcactggagataacaccaaatggaacgaaaatcagaaccctagaatgtttttggccatgatcacatatataaccagaaatcagcccgaatggttcagaaatattctaagtattgctccaataatgttctcaaacaaaatggcgagactaggtaaggggtacatgtttgaaagcaagagtatgaaacttagaactcaaatacctgcagaaatgctagcaaacatcgatttgaagtatttcaatgattcaacaagaaagaaaattgaaaaaatccggccactcttaatagatgggactgcatcactgagccctggaatgatgatgggcatgttcaatatgttaagcactgtattgggcgtctccatcctgaatcttggacaaaagagatacaccaagactacttactggtgggatggtcttcaatcgtctgatgattttgctctgattgtgaatgcacccaaccatgcaggaattcaagctggagttgacaggttttatcgaacctgtaagctgctcggaatcaatatgagcaagaaaaagtcttacataaacagaacaggtacatttgaattcacgagctttttctatcgttatgggtttgttgccaatttcagcatggagcttcccagttttggggtgtctgggatcaacgagtctgcagacatgagtattggagtcactgtcatcaaaaacaatatgataaacaatgatcttggtccagcaaccgctcaaatggcccttcagttattcatcaaagattacaggtacacgtatcgatgccacagaggtgacacacaaatacaaacccgaagatcatttgaaataaagaaactgtgggagcaaacccgttccaaagctgggctgctggtctccgatggaggtcctaatttatacaacattagaaatctccacattcctgaagtctgcttgaaatgggaattgatggatgaggattaccaggggcgtttatgcaacccattgaacccatttgtcagtcataaagagattgaatcagtgaacaatgcagtgatgatgccggcacatggtccagccaaaaatatggagtatgatgctgttgcaacaacacactcctgggtccccaaaaggaatcgatccatcttgaatacgagccaaagaggaatacttgaagatgaacaaatgtatcaaaggtgctgtaatttatttgaaaaattcttccccagcagttcatacagaaggccagtcggaatatccagtatggtagaggctatggtttccagagcccgaattgatgcacggattgatttcgaatctggaaggataaagaaagaggagttcactgagatcatgaagacctgttccaccattgaagaactcagacggcaaaaatag

>H1N1_PB1_CY020163_A/Western Australia/77/2005

atggatgtcaatccgacattacttttcttaaaagtgccagcacaaaatgctataagcacaacttttccttatactggtgaccctccttacagccatgggacaggaacagggtacaccatggatacagtcaacaggacacaccagtactcagaaagaggaagatggacaaaaaataccgaaactggagcaccgcaactcaacccaattgatgggccactaccagaagacaatgaaccaagtggctatgcccaaacagattgtgtattagaagcaatggctttccttgaagaatcccatcctggtatctttgaaaactcttgtattgaaacaatggaggttgttcagcaaacaagggtggacaaactgacacaaggcagacagacctatgactggactctaaataggaaccagcctgctgccacagcattggcaaacactatagaagtattcagatcaaacggcctcatagcaaatgaatctgggaggctaatagacttccttaaagatgtaatggagtcgatggacagagacgaagtagaggtcacaactcattttcaaagaaagaggagagtgagagacaatgtaactaaaaaaatggtgacccaaagaacaataggcaaaaagaaacataaattagacaaaagaagttacctaattagggcattaaccctgaacacaatgaccaaagatgctgagagggggaaactaaaacgcagagcaattgcaaccccaggaatgcaaataagggggtttgtatactttgttgagacactggcaagaagcatatgtgaaaagcttgaacaatcaggattgccagttggaggaaatgagaagaaagcaaagttagcaaatgttgtaaggaagatgatgaccaactcccaggacactgaaatttctttcaccatcactggagataacacaaaatggaacgaaaatcaaaaccctagaatgttcttggccatgatcacatatataaccaaaaatcagcctgaatggttcaggaatattctaagtattgctccaataatgttttcaaacaagatggcgagactaggtaaggggtacatgtttgaaagcaagagtatgaaactgagaactcaaatacctgcagagatgctagccaacatagatttgaaatatttcaatgattcaactaaaaagaaaattgaaaaaatccgaccattattaatagatggaactgcatcattgagtcctggaatgatgatgggcatgttcaatatgttgagcaccgtcttgggcgtctccattttgaatcttgggcaaaagagatacaccaagactacatactggtgggatggtcttcaatcgtctgatgattttgctttgattgtgaatgcacccaactatgcaggaattcaagctggagttgacaggttttatcgaacctgtaagctgctcggaattaatatgagcaaaaagaagtcttacataaacagaacaggtaccttcgaattcacgagctttttctatcgttatgggtttgttgccaatttcagcatggagcttcctagttttggggtgtctggggtcaatgaatctgcagacatgagtattggagtcactgtcatcaaaaacaatatgataaacaatgaccttggcccagcaactgctcaaatggcccttcagttatttataaaagattacaggtacacttatcgatgccacagaggtgacacacaaatacaaacccggagatcatttgagataaagaaactatgggaccaaacccgctccaaagctgggctgttggtctctgatggaggccccaatttatataacattagaaatctccatattcctgaagtttgcttgaaatgggagttgatggatgaggattaccaggggcgtttatgcaacccattaaacccgtttgtcagccataaagagattgaatcagtgaacaatgcagtgataatgccggcacatggtccagccaaaaatatggagtatgacgctgttgcaacaacacactcctgggtccccaaaagaaatcgatccattttgaacacgagccaaagggggatacttgaagatgagcaaatgtatcagaggtgctgcaatttatttgaaaaattcttcccaagtagctcatacagaagaccagttggaatatccagtatggtagaggctatggtttcaagagcccgaattgatgcacggattgatttcgaatctggaaggataaagaaagaggaatttgctgagatcatgaagatctgttccaccattgaagacctcagacggcaaaaatag

>H1N1_PB1_CY020579_A/Tientsin/78/1977

atggatgtcaatccgaccttacttttcttaaaagtgccagcacaaaatgctataagcacaactttcccttatactggagaccctccttacagccatgggacaggaacagggtacaccatggatacagtcaacaggacacatcagtactcagaaaggggaagatggacaaaaaacaccgaaactggagcaccgcaactcaacccgattgatgggccactaccaaaagacaatgaaccaagtggttatgcccaaacagattgtgtattagaagcaatggctttccttgaggaatcccatcctggtatttttgaaaactcgtgtattgaaacgatggaggttgttcagcaaacacgagtggacaaactgacacaaggcagacagacctatgactggactctaaatagaaaccagcctgctgctacagcattggccaacaccatagaagtgttcagatcaaatggcctcatggcaaatgaatctggaaggctaatagacttccttaaagatgtaatggagtcaatggacaaagaagaagtggagatcacaactcattttcagagaaagagaagagtgagggacaatgtgactaagaaaatggtgacacagagaacaataggcaaaaagaaacagagattaaacaaaaggagttatctaattagggcattaaccctgaacacaatgaccaaagatgctgagagagggaaactaaaacgtagagcaattgcaaccccagggatgcaaataagggggtttgtatactttgttgagacactggcaagaagtatatgtgaaaaacttgaacaatcagggttgccagttggaggaaatgaaaagaaagcaaagttggcaaatgttgtaaggaagatgatgaccaattctcaggacactgaaatttctttcaccatcactggagataacaccaaatggaacgaaaatcagaaccctagaatgtttttggccatgatcacatatataaccagaaatcagcccgaatggttcagaaatattctaagtattgctccaataatgttctcaaacaaaatggcgagactaggtaagggatacatgttcgaaagcaagagtatgaaacttagaactcaaatacctgcagaaatgctagcaaacatcgatttgaagtatttcaatgattcaacaagaaagaaaattgaaaaaatccggccactcttaatagatgggactgcatcactgagccctggaatgatgatgggcatgttcaatatgttaagcactgtattgggcgtctccatcctgaatcttggacaaaagagatacaccaagactacttactggtgggatggtcttcaatcgtctgatgattttgctctgattgtgaatgcacccaaccatgcaggaattcaagctggagttgacaggttttatcgaacctgtaagctgctcggaatcaatatgagcaagaaaaagtcttacataaacagaacaggtacatttgaattcacgagctttttctatcgttatgggtttgttgccaatttcagcatggagcttcccagttttggggtgtctgggatcaacgagtctgcagacatgagtattggagtcactgtcatcaaaaacaatatgataaacaatgatcttggtccagcaaccgctcaaatggcccttcagttattcatcaaagattacaggtacacgtatcgatgccacagaggtgacacacaaatacaaacccgaagatcatttgaaataaagaaactgtgggagcaaacccgttccaaagctgggctgctggtctccgatggaggtcctaatttatacaacattagaaatctccacattcctgaagtctgcttgaaatgggaattgatggatgaggattaccaggggcgtttatgcaacccattgaacccatttgtcagtcataaagagattgaatcagtgaacaatgcagtgatgatgccggcacatggtccagccaaaaacatggagtatgatgctgttgcaacaacacactcctgggtccccaaaaggaatcgatccatcttgaatacgagccaaagaggaatacttgaagatgaacaaatgtatcaaaggtgctgcaatttatttgaaaaattcttccccagcagttcatacagaaggccagtcggaatatccagtatggtagaggctatggtttccagagcccgaattgatgcacgaattgatttcgaatctggaaggataaagaaagaggagttcactgagatcatgaagatctgttccaccattgaagaactcagacggcaaaaatag

>H1N1_PB1_CY022171_A/Auckland/581/2000

atggatgtcaatccgacattacttttcttaaaagtgccagcacaaaatgctataagcacaacttttccttatactggtgaccctccttacagccatgggacaggaacagggtacaccatggatacagtcaacaggacacatcagtactcagaaagaggaagatggacaaaaaataccgaaactggagcaccgcaactcaacccaattgatgggccactaccaaaagacaatgaaccaagtggctatgcccaaacagattgtgtattagaagcaatggctttccttgaggaatcccatcctggtatttttgaaaactcttgtattgaaacaatggaggttgttcagcaaacaagggtggacaaactgacacaaggcagacagacctatgactggactctaaataggaaccagcctgctgccacagcattggccaacactatagaagtgttcagatcaaacggcctcatagcaaatgaatctgggaggctaatagacttccttaaagatgtaatggagtcgatggacagagacgaaatagaggtcacaactcattttcagagaaagaggagagtgagagacaatgtaactaaaaaaatggtgacccaaagaacaataggcaaaaagaaacataaattagataaaagaagttacctaattagggcattaaccctgaacacaatgaccaaagatgctgagagggggaaactaaaacgcagagcaattgcaaccccaggaatgcaaataagggggtttgtatactttgttgagacactggcaagaagcatatgtgaaaagcttgaacaatcagggttgccagttggaggaaatgaaaagaaagcaaagttagcaaatgttgtaaggaagatgatgaccaactcccaggacactgaaatttctttcaccatcactggagataacacaaaatggaacgaaaatcaaaaccctagaatgttcttggccatgatcacatatataaccaaaaatcagcctgaatggttcagaaatattctaagtattgctccaataatgttttcaaacaaaatggcgagactaggtaaggggtacatgtttgaaagcaagagtatgaaactgagaactcaaatacctgcagagatgctagccaacatagatttgaaatatttcaatgattcaactaaaaagaaaattgaaaaaatccggccattattaatagatggaactgcatcattgagtcctggaatgatgatgggcatgttcaatatgttaagcaccgtcttgggcgtctccattctgaatcttgggcaaaagagatacaccaagactacttactggtgggatggtcttcaatcgtctgatgattttgctctgattgtgaatgcacccaactatgcaggaattcaagctggagttgacaggttttatcgaacctgtaagctgctcggaattaatatgagcaaaaagaagtcttacataaacagaacaggtacctttgaattcacgagctttttctatcgttatgggtttgttgccaatttcagcatggagcttcctagttttggggtgtctggggtcaatgaatctgcagacatgagtattggagtcactgtcatcaaaaacaatatgataaacaatgaccttggcccagcaactgctcaaatggcccttcagttatttataaaagattacaggtacacgtatcgatgccacagaggtgacacacaaatacaaacccggagatcatttgagataaagaaactatgggaccaaacccgctccaaagctgggctgttggtctctgatggaggccccaatttatataacattagaaatctccatattcctgaagtctgcttgaaatgggagttgatggatgaggattaccaagggcgtttatgcaacccattgaacccgtttgtcagtcataaagagattgaatcagtgaacaatgcagtgatgatgccggcacatggtccagccaaaaatatggagtatgacgctgttgcaacaacacactcctgggttcccaaaaggaatcgatccattttgaatacgagccaaagggggatacttgaggatgagcaaatgtatcagaggtgctgcaatttatttgaaaaattcttcccaagtagctcatacagaagaccagttggaatatccagtatggtagaggctatggtttccagagcccgaattgatgcacggattgatttcgaatctggaaggataaagaaagaggaattcgctgagatcatgaagacctgttccaccattgaagacctcagacggcaaaaatag

>H1N1_PB1_CY022515_A/Auckland/590/2000

atggatgtcaatccgacattacttttcttaaaagtgccagcacaaaatgctataagcacaacttttccttatactggtgaccctccttacagccatgggacaggaacagggtacaccatggatacagtcaacaggacacatcagtactcagaaagaggaagatggacaaaaaataccgaaactggagcaccgcaactcaacccaattgatgggccactaccaaaagacaatgaaccaagtggctatgcccaaacagattgtgtattagaagcaatggctttccttgaggaatcccatcctggtatttttgaaaactcttgtattgaaacaatggaggttgttcagcaaacaagggtggacaaactgacacaaggcagacagacctatgactggactctaaataggaaccagcctgctgccacagcattggccaacactatagaagtgttcagatcaaatggcctcatagcaaatgaatctgggaggctaatagacttccttaaagatgtaatggagtcgatggacagagacgaaatagaggtcacaactcattttcagagaaagaggagagtgagagacaatgtaactaaaaaaatggtgacccaaagaacaataggcaaaaagaaacataaattagataaaagaagttacctaattagggcattaaccctgaacacaatgaccaaagatgctgagagggggaaactaaaacgcagagcaattgcaaccccaggaatgcaaataagggggtttgtatactttgttgagacactggcaagaagcatatgtgaaaagcttgaacaatcagggttgccagttggaggaaatgaaaagaaagcaaagttagcaaatgttgtaaggaagatgatgaccaactcccaggacactgaaatttctttcaccatcactggagataacacaaaatggaacgaaaatcaaaaccctagaatgttcttggccatgatcacatatataaccaaaaatcagcctgaatggttcagaaatattctaagtattgctccaataatgttttcaaacaaaatggcgagactaggtaaggggtacatgtttgaaagcaagagtatgaaactgagaactcaaatacctgcagagatgctagccaacatagatttgaaatatttcaatgattcaactaaaaagaaaattgaaaaaatccggccattattaatagatggaactgcatcattgagtcctggaatgatgatgggcatgttcaatatgttaagcaccgtcttgggcgtctccattctgaatcttgggcaaaagagatacaccaagactacttactggtgggatggtcttcaatcgtctgatgattttgctctgattgtgaatgcacccaactatgcaggaattcaagctggagttgataggttttatcgaacctgtaagctgctcggaattaatatgagcaaaaagaagtcttacataaacagaacaggtacctttgaattcacgagctttttctatcgttatgggtttgttgccaatttcagcatggagcttcctagttttggggtgtctggggtcaatgaatctgcagacatgagtattggagtcactgtcatcaaaaccaatatgataaacaatgaccttggcccagcaactgctcaaatggcccttcagttatttataaaagattacaggtacacgtatcgatgccacagaggtgacacacaaatacaaacccggagatcatttgagataaagaaactatgggaccaaacccgctccaaagctgggctgttggtctctgatggaggccccaatttatataacattagaaatctccatattcctgaagtctgcttgaaatgggagttgatggatgaggattaccaagggcgtttatgcaacccattgaacccgtttgtcagtcataaagagattgaatcagtgaacaatgcagtgatgatgccggcacatggtccagccaaaaatatggagtatgacgctgttgcaacaacacactcctgggttcccaaaaggaatcgatccattttgaatacgagccaaagggggatacttgaggatgagcaaatgtatcagaggtgctgcaatttatttgaaaaattcttcccaagtagctcatacagaagaccagttggaatatccagtatggtagaggctatggtttccagagcccgaattgatgcacggattgatttcgaatctggaaggataaagaaagaggaattcgctgagatcatgaagacctgttccaccattgaagacctcagacggcaaaaatag

>H1N1_PB1_CY022539_A/Auckland/605/2001

atggatgtcaatccgaccttacttttcttaaaagtgccagcacaaaatgctataagcacaactttcccttatactggtgatcctccttacagccatgggacaggaacagggtacaccatggatacagtcaacaggacacatcagtactcagaaagaggaagatggacaaaaaataccgaaactggagcaccgcaactcaacccaattgatggaccactaccaaaagacaatgaaccaagtggctatgcccaaacagattgtgtattagaagcaatggctttccttgaggaatcccatcctggtatttttgaaaactcttgtattgaaacaatggaggttgttcagcaaacaagggtggacaaactgacacaaggcagacagacctatgactggactctaaatagaaaccagcctgctgccacagcattggccaacactatagaagtgttcagatcaaacggcctcatagcaaatgaatctgggaggctaatagacttccttaaagatgtaatggagtcgatggacagagacgaagtagagatcacaacacattttcagagaaagaggagggtgagagacaatgtaactaaaaaaatggtgacccaaagaacaataggcaaaaagaaacataaattagacaaaagaagttacctaattagggcattaaccctgaacacaatgaccaaagatgctgagagggggaaactaaaacgtagagcaattgcaaccccaggaatgcaaataagggggtttgtatactttgttgagacactggcaagaagcatatgtgaaaagcttgaacaatcagggttgccagttgggggaaatgaaaagaaagcaaagttagcaaatgttgtaaggaagatgatgaccaactcccaggacactgaaatttctttcaccatcactggagataacacaaaatggaacgaaaatcaaaaccctagaatgtttttggccatgatcacatatataaccaaaaatcagcctgaatggttcagaaatattctaagtattgctccaataatgttttcaaacaaaatggcgagactaggtaaggggtacatgtttgaaagcaagagtatgaaactgagaactcaaatacctgcagagatgctagccaacatagatttgaaatatttcaatgattcaactaaaaagaaaattgaaaaaatccggccattattaatagatggaactgcatcattgagtcctggaatgatgatgggcatgttcaatatgttaagcactgtcttgggcgtctccattctgaatcttgggcaaaagagatacaccaagactacttactggtgggatggtcttcaatcgtctgatgattttgctctgattgtgaatgcatccaactatgcaggaattcaagctggagttgacaggttttatcgaacctgtaagctgctcggaattaatatgagcaaaaagaagtcttacataaacagaacaggtacctttgaattcacgagctttttctatcgttatgggtttgttgccaatttcagcatggagcttcctagttttggggtgtctggggtcaatgagtctgcagacatgagtattggagtcactgtcatcaaaaacaatatgataaacaatgaccttggcccagcaactgctcaaatggcccttcagttatttatcaaagattacaggtacacgtatcgatgccacagaggtgacacacaaatacaaacccggagatcatttgagataaagaaactatgggaccaaacccgctccaaagctgggctgttggtctctgatggaggccccaatttatataacattagaaatctccatattcctgaagtctgcttgaaatgggagttgatggatgaggattaccaggggcgtttatgcaacccattgaacccatttgtcagtcataaagagattgagtcagtgaacaatgcagtgatgatgccggcacatggtccagccaaaaatatggagtatgacgctgttgcaacaacacactcctgggttcccaaaaggaatcgatccattttgaatacgagccaaagggggatacttgaggatgagcaaatgtatcagaggtgctgcaatttatttgaaaaattcttcccaagtagttcatacagaagaccagttggaatatccagtatggtagaggctatggtttccagagcccgaattgatgcaagaattgatttcgaatctggaaggataaagaaagaggaattcgctgagatcatgaagacctgttccaccattgaagacctcagacggcaaaaatag

>H1N1_PB1_CY025299_A/Texas/UR06-0380/2007

atggatgtcaatccgacattacttttcttaaaagtgccagcacaaaatgctataagcacaacttttccttatactggtgaccctccttacagccatgggacaggaacagggtacaccatggatacagtcaacaggacacaccagtactcagaaagaggaagatggacaaaaaataccgaaactggagcaccgcaactcaacccaattgatgggccactaccagaagacaatgaaccaagtggctatgcccaaacagattgtgtattagaagcaatggctttccttgaagaatcccatcctggtatctttgaaaactcttgtattgaaacaatggaggttgttcagcaaacaagggtggacaaactgacacaaggcagacagacctatgactggactctaaataggaaccagcctgctgcaacagcattggcaaacactatagaagtattcagatcgaacggcctcatagcaaatgaatctggaaggctaatagacttccttaaagatgtaatggagtcgatggacagagacgaagtagaggtcacaactcattttcaaagaaagaggagagtaagagacaatgtaactaaaaaaatggtgactcaaagaacaataggcaaaaagaaacataaattagacaaaagaagttacctaattagggcattaaccctgaacacaatgaccaaagatgctgagagggggaaactaaaacgcagagcaattgcaaccccaggaatgcaaataagagggtttgtatactttgttgagacactggcaagaagcatatgtgaaaagcttgaacaatcaggattgccagttggaggaaatgagaagaaagcaaagttagcaaatgttgtaaggaagatgatgaccaactcccaggacactgaaatttctttcaccatcactggagataacacaaaatggaacgaaaatcaaaaccctagaatgttcttggccatgatcacatatataaccaaaaatcagcctgaatggttcagaaatattctaagtattgctccaataatgttttcaaacaagatggcgagactaggtaaggggtacatgtttgaaagcaagagtatgaaactgagaactcaaatacctgcagagatgctagccaacatagatttgaaatatttcaatgattcaactaaaaagaaaattgaaaaaatccgaccattattaatagatggaactgcatcattgagtcctggaatgatgatgggcatgttcaatatgttgagcaccgtcttgggcgtctccattctgaatcttgggcaaaagagatacaccaagactacttactggtgggatggtcttcaatcgtctgatgattttgctttgattgtgaatgcacccaactatgcaggaattcaagctggagttgacaggttttatcgaacctgtaagctgctcggaattaatatgagcaaaaagaagtcttacataaacagaacaggtacctttgaattcacgagctttttctatcgttatgggtttgttgccaatttcagcatggagcttcctagttttggggtgtctggggtcaatgaatctgcagacatgagtattggagtcactgtcatcaaaaacaatatgataaacaatgaccttggcccagcaactgctcaaatggcccttcagttatttataaaagattacaggtacacttatcgatgccacagaggtgacacacaaatacaaacccggagatcatttgagataaagaaactatgggaccaaacccgctccaaagctgggctgttggtctctgatggaggccccaatttatataacattagaaatctccatattcctgaagtttgcttgaaatgggagttgatggatgaggattaccaggggcgtttatgcaacccattaaacccgtttgtcagccataaagagattgaatcagtgaacaatgcagtgataatgccggcacatggcccagccaaaaatatggagtatgacgctgttgcaacaacacactcctgggtccccaaaagaaatcgatccattttgaacacgagccaaagggggatacttgaagatgaacaaatgtatcagaggtgctgcaatttatttgaaaaattcttcccaagtagctcatacagaagaccagttggaatatccagtatggtagaggctatggtttcaagagcccgaattgatgcacggattgatttcgaatctggaaggataaagagggaggaatttgctgagatcatgaagatctgttccaccattgaagacctcagacggcaaaaatag

>H1N1_PB1_CY025323_A/Mississippi/UR06-0242/2007

atggatgtcaatccgactttacttttcttaaaagtgccagcacaaaatgctataagcacaacttttccttatactggtgaccctccttacagccatgggacaggaacagggtacaccatggatacagtcaacaggacacaccagtactcagaaagaggaagatggacaaaaaataccgaaactggagcaccgcaactcaacccaattgatgggccactaccagaagacaatgaaccaagtggctatgcccaaacagattgtgtattagaagcaatggctttccttgaagaatcccatcctggtatctttgaaaactcttgtattgaaacaatggaggttgttcagcaaacaagggtggacaaactgacacaaggcagacagacctatgactggactctaaataggaaccagcctgctgcaacagcattggcaaacactatagaagtattcagatcaaacggcctcatagcaaatgaatctggaaggctaatagacttccttaaagatgtaatggagtcgatggacagagacgaagtagaggtcacaactcattttcaaagaaagaggagagtaagagacaatgtaactaaaaaaatggtgactcaaagaacaataggcaaaaagaaacataaattagacaaaagaagttacctaattagggcattaaccctgaacacaatgaccaaagatgctgagagggggaaactaaaacgcagagcaattgcaaccccaggaatgcaaataagagggtttgtatactttgttgagacactggcaagaagcatatgtgaaaagcttgaacaatcaggattgccagttggaggaaatgagaagaaagcaaagttagcaaatgttgtaaggaagatgatgaccaactcccaggacactgaaatttctttcaccatcactggagataacacaaaatggaacgaaaatcaaaaccctagaatgttcttggccatgatcacatatataaccaaaaatcagcctgaatggttcagaaacattctaagtattgctccaataatgttttcaaacaagatggcgagactaggtaaggggtacatgtttgaaagcaagagtatgaaactgagaactcaaatacctgcagagatgctagtcaacatagatttgaaatatttcaatgattcaactaaaaagaaaattgaaaaaatccgaccattattaatagatggaactgcatcattgagtcctggaatgatgatgggcatgttcaatatgttgagcaccgtcttgggcgtctccattctgaatcttgggcaaaagagatacaccaagactacttactggtgggatggtcttcaatcgtctgatgattttgctttgattgtgaatgcacccaactatgcaggaattcaagctggagttgacaggttttatcgaacctgtaagctgctcggaattaatatgagcaaaaagaagtcttacataaacagaacaggtacctttgaattcacgagctttttctatcgttatgggtttgttgccaatttcagcatggagcttcctagttttggggtgtctggggtcaatgaatctgcagacatgagtattggagtcactgtcatcaaaaacaatatgataaacaatgaccttggcccagcaactgctcaaatggcccttcagttatttataaaagattacaggtacacttatcgatgccacagaggtgacacacaaatacaaacccggagatcatttgagataaagaaactatgggaccaaacccgctccaaatctgggctgttggtctctgatggaggccccaatttatataacattagaaatctccatattcctgaagtttgcttgaaatgggagttgatggatgaggattaccaggggcgtttatgcaacccattaaatccgtttgtcagccataaagagattgaatcagtgaacaatgcagtgataatgccggcacatggcccagccaaaaatatggagtatgacgctgttgcaacaacacactcctgggtccccaaaagaaatcgatccattttgaacacgagccaaagggggatacttgaagatgagcaaatgtatcagaggtgctgcaatttatttgaaaaattcttcccaagtagctcatacagaagaccagttggaatatccagtatggtagaggctatggtttcaagagcccgaattgatgcacggattgatttcgaatctggaaggataaagagggaggaatttgctgagatcatgaagatctgttccaccattgaagacctcagacggcaaaaatag

>H1N1_PB1_CY025387_A/Alabama/UR06-0455/2007

atggatgtcaatccgacattacttttcttaaaagtgccagcacaaaatgctataagcacaacttttccttatactggtgaccctccttacagccatgggacaggaacagggtacaccatggatacagtcaacaggacacaccagtactcagaaagaggaagatggacaaaaaataccgaaactggagcaccgcaactcaacccaattgatgggccactaccagaagacaatgaaccaagtggctatgcccaaacagattgtgtattagaagcaatggctttccttgaagaatcccatcctggtatctttgaaaactcttgtattgaaacaatggaggttgttcagcaaacaagggtggacaaactgacacaaggcagacagacctatgactggactctaaataggaaccagcctgctgcaacagcattggcaaacactatagaagtattcagatcaaacggcctcatagcaaatgaatctggaaggctaatagacttccttaaagatgtaatggagtcgatggacagagacgaagtagaggtcacaactcattttcaaagaaagaggagagtaagagacaatgtaactaaaaaaatggtgactcaaagaacaataggcaaaaagaaacataaattagacaaaagaagttacctaattagggcattaaccctgaacacaatgaccaaagatgctgagagggggaaactaaaacgcagagcaattgcaaccccaggaatgcaaataagagggtttgtatactttgttgagacactggcaagaagcatatgtgaaaagcttgaacaatcaggattgccagttggaggaaatgagaagaaagcaaagttagcaaatgttgtaaggaagatgatgaccaactcccaggacactgaaatttctttcaccatcactggagataacacaaaatggaacgaaaatcaaaaccctagaatgttcttggccatgatcacatatataaccaaaaatcagcctgaatggttcagaaatattctaagtattgctccaataatgttttcaaacaagatggcgagactaggtaaggggtacatgtttgaaagcaagagtatgaaactgagaactcaaatacctgcagagatgctagccaacatagatttgaaatatttcaatgattcaactaaaaagaaaattgaaaaaatccgaccattattaatagatggaactgcatcattgagtcctggaatgatgatgggcatgttcaatatgttgagcaccgtcttgggcgtctccattctgaatcttgggcaaaagagatacaccaagactacttactggtgggatggtcttcaatcgtctgatgattttgctttgattgtgaatgcacccaactatgcaggaattcaagctggagttgacaggttttatcgaacctgtaagctgctcggaattaatatgagcaaaaagaagtcttacataaacagaacaggtacctttgaattcacgagctttttctatcgttatgggtttgttgccaatttcagcatggagcttcctagttttggggtgtctggggtcaatgaatctgcagacatgagtattggagtcactgtcatcaaaaacaatatgataaacaatgaccttggcccagcaactgctcaaatggcccttcagttatttataaaagattacaggtacacttatcgatgccacagaggtgacacacaaatacaaacccggagatcatttgagataaagaaactatgggaccaaacccgctccaaagctgggctgttggtctctgatggaggccccaatttatataacattagaaatctccatattcctgaagtttgcttgaaatgggagttgatggatgaggattaccaggggcgtttatgcaacccattaaacccgtttgtcagccataaagagattgaatcagtgaataatgcagtgataatgccggcacatggcccagccaaaaatatggagtatgacgctgttgcaacaacacactcctgggtccccaaaagaaatcgatccattttgaacacgagccaaagggggatacttgaagatgagcaaatgtatcagaggtgctgcaatttatttgaaaaattcttcccaagtagctcatacagaagaccagttggaatatccagtatggtagaggctatggtttcaagagcccgaattgatgcacggattgatttcgaatctggaaggataaagagggaggaatttgctgagatcatgaagatctgttccaccattgaagacctcagacggcaaaaatag

>H1N1_PB1_CY025411_A/Colorado/UR06-0207/2007

atggatgtcaatccgacattacttttcttaaaagtgccagcacaaaatgctataagcacaacttttccttatactggtgaccctccttacagccatgggacaggaacagggtacaccatggatacagtcaacaggacacaccagtactcagaaagaggaagatggactaaaaataccgaaactggagcaccgcaactcaacccaattgatgggccactaccagaagacaatgaaccaagtggctatgcccaaacagattgtgtattagaagcaatggctttccttgaagaatcccatcctggtatctttgaaaactcttgtattgaaacaatggaggttgttcagcaaacaagggtggacaaactgacacaaggcagacagacctatgactggactctaaataggaaccagcctgctgcaacagcattggcaaacactatagaagtattcagatcaaacggcctcatagcaaatgaatctggaaggctaatagacttccttaaagatgtaatggagtcgatggacagagacgaagtagaggtcacaactcattttcaaagaaagaggagagtgagagacaatgtaactaaaaaaatggtgactcaaagaacaataggcaaaaagaaacataaattagacaaaagaagttacctaattagggcattaaccctgaacacaatgaccaaagatgctgagagggggaaactaaaacgcagagcaattgcaaccccaggaatgcaaataagagggtttgtatactttgttgagacactggcaagaagcatatgtgaaaagcttgaacaatcaggtttgccagttggaggaaatgagaagaaagcaaagttagcaaatgttgtaaggaagatgatgaccaactcccaggacactgaaatttctttcaccatcactggagataacacaaaatggaacgaaaatcaaaaccctagaatgttcttggccatgatcacatatataaccaaaaatcagcctgaatggttcagaaatattctaagtattgctccaataatgttttcaaacaagatggcgagactaggtaaggggtacatgtttgaaagcaagagtatgaaactgagaactcaaatacctgcagagatgctagccaacatagatttgaaatatttcaatgattcaactaaaaagaaaattgaaaaaatccgaccattattaatagatggaactgcatcattgagtcctggaatgatgatgggcatgttcaatatgttgagcaccgtcttgggcgtctccattctgaatcttgggcaaaagagatacaccaagactacttactggtgggatggtcttcaatcgtctgatgattttgctttgattgtgaatgcacccaactatgcaggaattcaagctggagttgacaggttttatcgaacctgtaaactgctcggaattaatatgagcaaaaagaagtcttacataaacagaacaggtacctttgaattcacgagctttttctatcgttatgggtttgttgccaatttcagcatggagcttcctagttttggggtgtctggggtcaatgaatctgcagacatgagtattggagtcactgtcatcaaaaacaatatgataaacaatgaccttggcccagcaactgctcaaatggcccttcagttatttataaaagattacaggtacacttatcgatgccacagaggtgacacacaaatacaaacccggagatcatttgagataaagaaactatgggaccaaacccgctccaaagctgggctgttggtctctgatggaggccccaatttatataacattagaaatctccatattcctgaagtttgcttgaaatgggagttgatggatgaggattaccaggggcgtttatgcaacccattaaacccgtttgtcagccataaagagattgaatcagtgaacaatgcagtgataatgccggcacatggcccagccaaaaatatggagtatgacgctgttgcaacaacacactcctgggtccccaaaagaaatcgatccattttgaacacgagccaaagggggatacttgaagatgagcaaatgtatcagaggtgctgcaatttatttgaaaaattcttcccaagtagctcatacagaagaccagttggaatatccagtatggtagaggctatggtttcaagagcccgaattgatgcacggattgatttcgaatctggaaggataaagagggaggaatttgctgagatcatgaagatctgttccaccattgaagacctcagacggcaaaaatag

>H1N1_PB1_CY025801_A/Vermont/UR06-0089/2007

atggatgtcaatccgacattacttttcttaaaagtgccagcacaaaatgctataagcacaacttttccttatactggtgaccctccttacagccatgggacaggaacagggtacaccatggatacagtcaacaggacacaccagtactcagaaagaggaagatggacaaaaaataccgaaactggagcaccgcaactcaacccaattgatgggccactaccagaagacaatgaaccaagtggctatgcccaaacagattgtgtattagaagcaatggctttccttgaagaatcccatcctggtatctttgaaaactcttgtattgaaacaatggaggttgttcagcaaacaagggtggacaaactgacacaaggcagacagacctatgactggactctaaataggaaccagcctgctgccacagcattggcaaacactatagaagtattcagatcaaacggcctcatagcaaatgaatctgggaggctaatagacttccttaaagatgtaatggagtcgatggacagagacgaagtagaggtcacaactcattttcaaagaaagaggagagtgagagacaatgtaactaaaaaaatggtgacccaaagaacaataggcaaaaagaaacataaattagacaaaagaagttacctaattagggcattaaccctgaacacaatgaccaaagatgctgaaagggggaaactaaaacgcagagcaattgcaaccccaggaatgcaaataagggggtttgtgtactttgttgagacactggcaagaagcatatgtgaaaagcttgaacaatcaggattgccagttggaggaaatgagaagaaagcaaagttagcaaatgttgtaaggaagatgatgaccaactcccaggacactgaaatttctttcaccattactggagataacacaaaatggaacgaaaatcaaaaccctagaatgttcttggccatgatcacatatataaccaaaaatcagcctgaatggttcagaaatattctaagtattgctccaataatgttttcaaacaagatggcgagactaggtaaagggtacatgtttgaaagcaagagtatgaaactgagaactcaaatacctgcagaaatgctagccaacatagatttgaaatatttcaatgattcaactaaaaagaaaattgaaaaaatccgaccattattaatagatggaactgcatcattgagtcctggaatgatgatgggcatgttcaatatgttgagcaccgtcttgggcgtctccattctgaatcttgggcaaaagagatacaccaagactacttactggtgggatggtcttcaatcgtctgatgattttgctttgattgtgaatgcacccaactatgcaggaattcaagctggagttgacaggttttatcgaacctgtaagctgctcggaattaatatgagcaaaaagaagtcttacataaacagaacaggtacctttgaattcacgagctttttctatcgttatgggtttgttgccaatttcagcatggagcttcctagttttggggtgtctggggtcaatgaatctgcagacatgagtattggagtcactgtcatcaaaaacaatatgataaacaatgaccttggcccagcaactgctcaaatggcccttcagttatttataaaagattacaggtacacttatcgatgccacagaggtgacacacaaatacaaacccggagatcatttgagataaagaaactatgggaccaaacccactccaaagctgggctgttggtctctgatggaggccccaatttatataacattagaaatctccatattcctgaagtttgcttgaaatgggagttgatggatgaggattaccaggggcgtttatgcaacccattaaacccgtttgtcagccataaagagattgaatcagtgaacaatgcagtgataatgccggcacatggtccagccaagaatatggagtatgacgctgttgcaacaacacactcctgggtccccaaaagaaatcgatccattttgaacacgagccaaagggggatacttgaagatgagcaaatgtatcagaggtgctgcaatttatttgaaagattcttcccaagtagctcatacagaagaccagttggaatatccagtatggtagaggctatggtttcaagagccagaattgatgcacggattgatttcgaatctggaaggataaagaaagaggaatttgctgagatcatgaagatctgttccaccattgaagacctcagacggcaaaaatag

>H1N1_PB1_CY026385_A/New York/UR06-0056/2007

atggatgtcaatccgacattacttttcttaaaagtgccagcacaaaatgctataagcacaacttttccttatactggtgaccctccttacagccatgggacaggaacagggtacaccatggatacagtcaacaggacacaccagtactcagaaagaggaagatggacaaaaaataccgaaactggagcaccgcaactcaacccaattgatgggccactaccagaagacaatgaaccaagtggctatgcccaaacagattgtgtattagaagcaatggctttccttgaagaatcccatcctggtatctttgaaaactcttgtattgaaacaatggaggttgttcagcaaacaagggtggacaaactgacacaaggcagacagacctatgactggactctaaataggaaccagcctgctgcaacagcattggcaaacactatagaagtattcagatcaaacggcctcatagcaaatgaatctggaaggctaatagacttccttaaagatgtaatggagtcgatggacagagacgaagtagaggtcacaactcattttcaaagaaagaggagagtaagagacaatgtaactaaaaaaatggtgactcaaagaacaataggcaaaaagaaacataaattagacaaaagaagttacctaattagggcattaaccctgaacacaatgaccaaagatgctgagagggggaaactaaaacgcagagcaattgcaaccccaggaatgcaaataagagggtttgtatactttgttgagacactggcaagaagcatatgtgaaaagcttgaacaatcaggattgccagttggaggaaatgagaagaaagcaaagttagcaaatgttgtaaggaagatggtgaccaactcccaggacactgaaatttctttcaccatcactggagataacacaaaatggaacgaaaatcaaaaccctagaatgttcttggccatgatcacatatataaccaaaaatcagcctgaatggttcagaaatattctaagtattgctccaataatgttttcaaacaagatggcgagactaggtaaggggtacatgtttgaaagcaagagtatgaaactgagaactcaaatacctgcagagatgctagccaacatagatttgaaatatttcaatgattcaactaaaaagaaaattgaaaaaatccgaccattattaatagatggaactgcatcattgagtcctggaatgatgatgggcatgttcaatatgttgagcaccgtcttgggcgtctccattctgaatcttgggcaaaagagatacaccaagactacttactggtgggatggtcttcaatcgtctgatgattttgctttgattgtgaatgcacccaactatgcaggaattcaagctggagttgacaggttttatcgaacctgtaagctgctcggaattaatatgagcaaaaagaagtcttacataaacagaacaggtacctttgaattcacgagctttttctatcgttatgggtttgttgccaatttcagcatggagcttcctagttttggggtgtctggggtcaatgaatctgcagacatgagtattggagtcactgtcatcaaaaacaatatgataaacaatgaccttggcccagcaactgctcaaatggcccttcagttatttataaaagattacaggtacacttatcgatgccacagaggtgacacacaaatacaaacccggagatcatttgagataaagaaactatgggaccaaacccgctccaaagctgggctgttggtctctgatggaggccccaatttatataacattagaaatctccatattcctgaagtttgcttgaaatgggagttgatggatgaggattaccaggggcgtttatgcaacccattaaacccgtttgtcagccataaagagattgaatcagtgaacaatgcagtgataatgccggcacatggcccagccaaaaatatggagtatgacgctgttgcaacaacacactcctgggtccccaaaagaaatcgatccattttgaacacgagccaaagggggatacttgaagatgagcaaatgtatcagaggtgctgcaatttatttgaaaaattcttcccaagtagctcatacagaagaccagttggaatatccagtatggtagaggctatggtttcaagagcccgaattgatgcacggattgatttcgaatctggaaggataaagagggaggaatttgctgagatcatgaagatctgttccaccattgaagacctcagacggcaaaaatag

>H1N1_PB1_CY026505_A/Texas/UR06-0468/2007

atggatgtcaatccgacattacttttcttaaaagtgccagcacaaaatgctataagcacaacctttccttatactggtgaccctccttacagccatggaacaggaacagggtacaccatggatacagtcaacaggacacatcagtactcagaaagaggaagatggacaaaaaataccgaaactggagcaccgcaactcaacccaattgatgggccactaccagaagacaatgaaccaagtggctatgcccaaacagattgtgtattagaagcaatggctttccttgaagaatcccatcctggtatttttgaaaactcttgtattgaaacaatggaggttgttcagcaaacaagggtggacaaactgacacaaggcagacaaacctatgactggactctaaataggaaccagcctgccgccacagcattggcaaacactatagaagtattcagatcaaatggcctcatagcaaatgaatctgggaggctaatagacttccttaaagatgtaatggagtcgatggacagagaagaagtagaggtcacaactcattttcaaagaaagaggagagtgagagacaatgtaactaaaaaaatggtgacccaaagaacaatagggaaaaagaaacataaattagacaaaagaagttacctaattagggcattaaccctgaacacaatgaccaaagatgctgagagggggaaactaaaacgcagagcaattgcaaccccaggaatgcaaataagggggtttgtatactttgttgagacactggcaagaagcatatgtgaaaagcttgaacaatcagggttgccagttggaggaaatgagaagaaagcaaagttagcaaatgttgtaaggaagatgatgaccaactcccaggacactgaaatttctttcaccatcactggagataacacaaaatggaacgagaatcaaaaccctagaatgttcttggccatgatcacatatataaccaaaaatcagcctgaatggttcagaaatattctaagtattgctccaataatgttttcaaacaaaatggcgagactaggtagggggtatatgtttgaaagcaagagtatgaaactgagaacccaaatacctgcagagatgctagccaacatagatttgaaatacttcaatgattcaactaaaaagaaaattgaaaaaattcgaccattattaatagatggaactgcatcattgagtcctggaatgatgatgggcatgttcaatatgttaagcaccgtcttgggcgtctccattctgaatcttgggcaaaagagatacaccaagactacttactggtgggatggtcttcaatcgtctgatgattttgctttgattgtgaatgcacccaattatgcaggaattcaagctggagttgacaggttttatcgaacctgtaagctgctcggaattaatatgagcaaaaagaagtcttacataaacagaacaggtacctttgaattcacgagctttttctatcgttatgggtttgttgccaatttcagcatggagcttcctagttttggggtgtctggggtcaatgaatctgcagacatgagtattggagtcactgtcatcaaaaacaatatgataaacaatgaccttggcccagcaactgctcaaatggcccttcagttatttataaaagattataggtacacttatcgatgccacagaggtgacacacaaatacaaacccgaagatcatttgaaataaagaaactatgggaccaaacccgctccaaagctgggctgttggtctctgatggaggccccaatctatataacattaggaatctacatattcctgaagtctgcttgaaatgggagttgatggatgaggattaccaggggcgtttatgcaacccattgaacccgtttgtcagccataaagagattgaatcagtgaacaatgcagtgataatgccggcacatggtccagcaaaaaatatggagtatgacgctgttgcaacaacacactcctgggtccccaaaagaaatcgatccattttgaacacgagccaaagagggatacttgaagatgagcaaatgtatcagaggtgctgcaatttatttgaaaaattcttcccaagtagctcatacagaagaccagttggaatatccagtatggtagaggctatggtttcaagagcccgaattgatgcacggattgatttcgaatctggaaggataaagaaagaggaattcgctgagatcatgaagacctgttccaccattgaagacctcagacggcaaaaatag

>H1N1_PB1_CY026529_A/Colorado/UR06-0053/2007

atggatgtcaatccgacattacttttcttaaaagtgccagcacaaaatgctataagcacaacttttccttatactggtgaccctccttacagccatggaacaggaacagggtacaccatggatacagtcaacaggacacatcagtactcagaaagaggaagatggacaaaaaataccgaaactggagcaccgcaactcaacccaattgatgggccactaccagaagacaatgaaccaagtggctatgcccaaacagattgtgtattagaagcaatggctttccttgaagaatcccatcctggtatttttgaaaactcttgtattgaaacaatggagattgttcagcaaacaagggtggacaaactgacacaaggcagacagacctatgactggactctaaataggaaccagcctgctgccacagcattggccaacactatagaagtattcagatcaaacggcctcatagcaaatgaatctgggaggctaatagacttccttaaagatgtaatggagtcgatggacagagaagaagtagaggtcacaactcattttcagagaaagaggagagtgagagacaatgtaactaaaaaaatggtgacccaaagaacaataggcaaaaagaaacataaattagacaaaaggagttacctaattagagcattaaccctgaacacaatgaccaaagatgctgaaagggggaaactaaaacgcagagcaattgcaaccccaggaatgcaaataagggggtttgtatactttgttgaaacactggcaagaagcatatgtgaaaagcttgaacaatcagggttgccagttggaggaaatgagaagaaagcaaagttggcaaatgttgtaaggaagatgatgaccaactcccaggacactgaaatttctttcaccatcactggagataacacaaaatggaacgaaaatcaaaaccctagaatgtttttggccatgatcacatatataaccagaaatcagcctgaatggttcagaaatattctaagtgttgctccaataatgttttcaaacaaaatggcgagactaggtaaggggtacatgtttgaaagcaagagtatgaaactaagaactcaaatacctgcagagatactagccgacatagatttgaagtatttcaatgagtcaactaaaaagaaaattgaaaaaatccgaccattattaatagatggaactgcatcattgagtcctggaatgatgatgggcatgttcaatatgttaagcactgtcttgggcgtctccattctgaatcttgggcaaaagagatacaccaagactacttactggtgggatggtctccaatcgtctgatgattttgctctgatagtgaatgcgcccaactatgcaggaattcaagctggagttgacaggttttatcgaacctgtaagctgctcggaattaatatgagcaaaaagaagtcttacataaacaggacaggtacctttgaattcacaagctttttctatcgttatgggtttgttgccaatttcagcatggagcttcctagttttggggtgtcaggggtcaatgaatctgcagacatgagtattggagtcactgttatcagaaacaatatgataaacaatgaccttggcccagcaactgctcaaatggcccttcagttatttataaaagattacagatacacgtatcgatgccacagaggtgacacacaaatacaaacccggagatcatttgagataaagaaattatgggaccaaacccgctccaaagctgggctgttggtttctgatggaggccccaatttatataacattagaaatctccatattcctgaagtctgtttgaaatgggagttgatggatgaggattaccaggggcgtttatgcaacccattgaacccatttgtcagtcataaagagattgaatcagtgaacaatgcagtgatgatgccggcacatggtccagccaaaaatatggagtatgacgctgttgcaacaacacattcctgggttcccaaaaggaatcgatccattttgaacacgagccaaagggggatacttgaggatgagcaaatgtatcagaggtgctgcaatttatttgaaaaattctttccaagtagctcatacagaagaccagttgggatatccagtatggtagaggctatggtttccagagcccgaattgatgcacggattgatttcgaatctggaaggataaagaaagaggaattcgctgagatcatgaagacctgttccaccattgaagacctcagacggcaaaaatag

>H1N1_PB1_CY026937_A/Virginia/UR06-0267/2007

atggatgtcaatccgacattacttttcttaaaagtgccagcacaaaatgctataagcacaacttttccttatactggtgaccctccttacagccatgggacaggaacagggtacaccatggatacagtcaacaggacacaccagtactcagaaagaggaagatggacaaaaaataccgaaactggagcaccgcaactcaacccaattgatgggccactaccagaagacaatgaaccaagtggctatgcccaaacagattgtgtattagaagcaatggctttccttgaagaatcccatcctggtatctttgaaaactcttgtattgaaacaatggaggttgttcagcaaacaagggtggacaaactgacacaaggcagacagacctatgactggactctaaataggaaccagcctgctgcaacagcattggcaaacactatagaagtattcagatcaaacggcctcatagcaaatgaatctggaaggctaatagacttccttaaagatgtaatggagtcgatggacagagacgaagtagaggtcacaactcattttcaaagaaagaggagagtgagagacaatgtaactaaaaaaatggtgactcaaagaacaataggcaaaaagaaacataaattagacaaaagaagttacctaattagggcattaaccctgaacacaatgaccaaagatgctgagagggggaaactaaaacgcagagcaattgcaaccccaggaatgcaaataagagggtttgtatactttgttgagacactggcaagaagcatatgtgaaaagcttgaacaatcaggattgccagttggaggaaatgagaagaaagcaaagttagcaaatgttgtaaggaagatgatgaccaactcccaggacactgaaatttctttcaccatcactggagataacacaaaatggaacgaaaatcaaaaccctagaatgttcttggccatgatcacatatataaccaaaaatcagcctgaatggttcagaaatattctaagtattgctccaataatgttttcaaacaagatggcgagactaggtaaggggtacatgtttgaaagcaagagtatgaaactgagaactcaaatacctgcagagatgctagccaacatagatttgaaatatttcaatgattcaactaaaaagaaaattgaaaaaatccgaccattattaatagatggaacggcatcattgagtcctggaatgatgatgggcatgttcaatatgttgagcaccgtcttgggcgtctccattctgaatcttgggcaaaagagatacaccaagactacttactggtgggatggtcttcaatcgtctgatgattttgctttgattgtgaatgcacccaactatgcaggaattcaagctggagttgacaggttttatcgaacctgtaagctgctcggaattaatatgagcaaaaagaagtcttacataaacagaacaggtacctttgaattcacgagctttttctatcgttatgggtttgttgccaatttcagcatggagcttcctagttttggggtgtctggggtcaatgaatctgcagacatgagtattggagtcactgtcatcaaaaacaatatgataaacaatgaccttggcccagcaactgctcaaatggcccttcagttatttataaaagattacaggtacacttatcgatgccacagaggtgacacacaaatacaaacccggagatcatttgagataaagaaactatgggaccaaacccgctccaaagctgggctgttggtctctgatggaggccccaatttatataacattagaaatctccatattcctgaagtttgcttgaaatgggagttgatggatgaggattaccaggggcgtttatgcaacccattaaacccgtttgtcagccataaagagattgaatcagtgaacaatgcagtgataatgccggcacatggcccagccaaaaatatggagtatgacgctgttgcaacaacacactcctgggtccccaaaagaaatcgatccattttgaacacgagccaaagggggatacttgaagatgagcaaatgtatcagaggtgctgcaatttatttgaaaaattcttcccaagtagctcatacagaagaccagttggaatatccagtatggtagaggctatggtttcaagagcccgaattgatgcacggattgatttcgaatctggaaggataaagagggaggaatttgctgagatcatgaagatctgttccaccattgaagacctcagacggcaaaaatag

>H1N1_PB1_CY026961_A/Ohio/UR06-0091/2007

atggatgtcaatccgacattacttttcttaaaagtgccagcacaaaatgctataagcacaacttttccttatactggtgaccctccttacagccatgggacaggaacagggtacaccatggatacagtcaacaggacacaccagtactcagaaagaggaagatggacaaaaaataccgaaactggagcaccgcaactcaacccaattgatgggccactaccagaagacaatgaaccaagtggctatgcccaaacagattgtgtattagaagcaatggctttccttgaagaatcccatcctggtatctttgaaaactcttgtattgaaacaatggaggttgttcagcaaacaagggtggacaaactgacacaaggcagacagacctatgactggactctaaataggaaccagcctgctgcaacagcattggcaaacactatagaagtattcagatcaaacggcctcatagcaaatgaatctggaaggctaatagacttccttaaagatgtaatggagtcgatggacagagacgaagtagaggtcacaactcattttcaaagaaagaggagagtaagagacaatgtaactaaaaaaatggtgactcaaagaacaataggcaaaaagaaacataaattagacaaaagaagttacctaattagggcattaaccctgaacacaatgaccaaagacgctgagagggggaaactaaaacgcagagcaattgcaaccccaggaatgcaaataagagggtttgtatactttgttgagacactggcaagaagcatatgtgaaaagcttgaacaatcaggattgccagttggaggaaatgagaagaaagcaaagttagcaaatgttgtaaggaagatgatgaccaactcccaggacactgaaatttctttcaccatcactggagataacacaaaatggaacgaaaatcaaaaccctagaatgttcttggccatgatcacatatataaccaaaaatcagcctgaatggttcagaaatattctaagtattgctccaataatgttttcaaacaagatggcgagactaggtaaggggtacatgtttgaaagcaagagtatgaaactgagaactcaaatacctgcagagatgctagccaacatagatttgaaatatttcaatgattcaactaaaaagaaaattgaaaaaatccgaccattattaatagatggaactgcatcattgagtcctggaatgatgatgggcatgttcaatatgttgagcaccgtcttgggcgtctccattctgaatcttgggcaaaagagatacaccaagactacttactggtgggatggtcttcaatcgtctgatgattttgctttgattgtgaatgcacccaactatgcaggaattcaagctggagttgacaggttttatcgaacctgtaagctgctcggaattaatatgagcaaaaagaagtcttacataaacagaacaggtacctttgaattcacgagctttttctatcgttatgggtttgttgccaatttcagcatggagcttcctagttttggggtgtctggggtcaatgaatctgcagacatgagtattggagtcactgtcatcaaaaacaatatgataaacaatgaccttggcccagcaactgctcaaatggcccttcagttatttataaaagattacaggtacacttatcgatgccacagaggtgacacacaaatacaaacccggagatcatttgagataaagaaactatgggaccaaacccgctccaaagctgggctgttggtctctgatggaggccccaatttatataacattagaaatctccatattcctgaagtttgcttgaaatgggagttgatggatgaggattaccaggggcgtttatgcaacccattaaacccgtttgtcagccataaagagattgaatcagtgaacaatgcagtgataatgccggcacatggcccagccaaaaatatggagtatgacgctgttgcaacaacacactcctgggtccccaaaagaaatcgatccattttgaacacgagccaaagggggatacttgaagatgagcaaatgtatcagaggtgctgcaatttatttgaaaaattcttcccaagtagctcatacagaagaccagttggaatatccagtatggtagaggctatggtttcaagagcccgaattgatgcacggattgatttcgaatctggaaggataaagagggaggaatttgctgagatcatgaagatctgttccaccattgaagacctcagacggcaaaaatag

>H1N1_PB1_CY027057_A/Kentucky/UR06-0154/2007

atggatgtcaatccgacattacttttcttaaaagtgccagcacaaaatgctataagcacaacttttccttatactggtgaccctccttacagccatgggacaggaacagggtacaccatggatacagtcaacaggacacaccagtactcagaaagaggaagatggacaaaaaataccgaaactggagcaccgcaactcaacccaattgatgggccactaccagaagacaatgaaccaagtggctatgcccaaacagattgtgtattagaagcaatggctttccttgaagaatcccatcctggtatctttgaaaactcttgtattgaaacaatggaggttgttcagcaaacaagggtggacaaactgacacaaggcagacagacctatgactggactctaaataggaaccagcctgctgcaacagcattggcaaacactatagaagtattcagatcaaacggcctcatagcaaatgaatctggaaggctaatagacttccttaaagatgtaatggagtcgatggacagagacgaagtagaggtcacaactcattttcaaagaaagaggagagtgagagacaatgtaactaaaaaaatggtgactcaaagaacaataggcaaaaagaaacataaattagacaaaagaagttacctaattagggcattaaccctgaacacaatgaccaaagatgctgagagggggaaactaaaacgcagagcaattgcaaccccaggaatgcaaataagagggtttgtatactttgttgagacactggcaagaagcatatgtgaaaagcttgaacaatcaggattgccagttggaggaaatgagaagaaagcaaagttagcaaatgttgtaaggaagatgatgaccaactcccaggacactgaaatttctttcaccatcactggagataacacaaaatggaacgaaaatcaaaaccctagaatgttcttggccatgatcacatatataaccaaaaatcagcctgaatggttcagaaatattctaagtattgctccaataatgttttcaaacaagatggcgagactaggtaaggggtacatgtttgaaagcaagagtatgaaactgagaactcaaatacctgcagagatgctagccaacatagatttgaaatatttcaatgattcaactaaaaagaaaattgaaaaaatccgaccattattaatagatggaactgcatcattgagtcctggaatgatgatgggcatgttcaatatgttgagcaccgtcttgggcgtctccattctgaatcttgggcaaaagagatacaccaagactacttactggtgggatggtcttcaatcgtctgatgattttgctttgattgtgaatgcacccaactatgcaggaattcaagctggagttgacaggttttatcgaacctgtaagctgctcggaattaatatgagcaaaaagaagtcttacataaacagaacaggtacctttgaattcacgagctttttctatcgttatgggtttgttgccaatttcagcatggagcttcctagttttggggtgtctggggtcaatgaatctgcagacatgagtattggagtcactgtcatcaaaaacaatatgataaacaatgaccttggcccagcaactgctcaaatggcccttcagttatttataaaagattacaggtacacttatcgatgccacagaggtgacacacaaatacaaacccggagatcatttgagataaagaaactatgggaccaaacccgctccaaagctgggctgttggtctctgatggaggccccaatttatataacattagaaatctccatattcctgaagtttgcttgaaatgggagttgatggatgaggattaccaggggcgtttatgcaacccattaaacccgtttgtcagccataaagagattgaatcagtgaacaatgcagtgataatgccagcacatggcccagccaaaaatatggagtatgacgctgttgcaacaacacactcctgggtccccaaaagaaatcgatccattttgaacacgagccaaagggggatacttgaagatgagcaaatgtatcagaggtgctgcaatttatttgaaaaattcttcccaagtagctcatacagaagaccagttggaatatccagtatggtagaggctatggtttcaagagcccgaattgatgcacggattgatttcgaatctggaaggataaagagggaggaatttgctgagatcatgaagatctgttccaccattgaagacctcagacggcaaaaatag

>H1N1_PB1_CY027393_A/Ohio/UR06-0233/2007

atggatgtcaatccgacattacttttcttaaaagtgccagcacaaaatgctataagcacaacttttccttatactggtgaccctccttacagccatgggacaggaacagggtacaccatggatacagtcaacaggacacaccagtactcagaaagaggaagatggacaaaaaataccgaaactggagcaccgcaactcaacccaattgatgggccactaccagaagacaatgaaccaagtggctatgcccaaacagattgtgtattagaagcaatggctttccttgaagaatcccatcctggtatctttgaaaactcttgtattgaaacaatggaggttgttcagcaaacaagggtggacaaactgacacaaggcagacagacctatgactggactctaaataggaaccagcctgctgcaacagcattggcaaacactatagaagtattcagatcaaacggcctcatagcaaatgaatctggaaggctaatagacttccttaaagatgtaatggagtcgatggacagagacgaagtagaggtcacaactcattttcaaagaaagaggagagtaagagacaatgtaactaaaaaaatggtgactcaaagaacaataggcaaaaagaaacataaattagacaaaagaagttacctaattagggcattaaccctgaacacaatgaccaaagatgctgagagggggaaactaaaacgcagagcaattgcaaccccaggaatgcaaataagagggtttgtatactttgttgagacactggcaagaagcatatgtgaaaagcttgaacaatcaggattgccagttggaggaaatgagaagaaagcaaagttagcaaatgttgtaaggaagatgatgaccaactcccaggacactgaaatttctttcaccatcactggagataacacaaaatggaacgaaaatcaaaaccctagaatgttcttggccatgatcacatatataaccaaaaatcagcctgaatggttcagaaatattctaagtattgctccaataatgttttcaaacaagatggcgagactaggtaaggggtacatgtttgaaagcaagagtatgaaactgagaactcaaatacctgcagagatgctagccaacatagatttgaaatatttcaatgattcaactaaaaagaaaattgaaaaaatccgaccattattaatagatggaactgcatcattgagtcctggaatgatgatgggcatgttcaatatgttgagcaccgtcttgggcgtctccattctgaatcttgggcaaaagagatacaccaagactacttactggtgggatggtcttcaatcgtctgatgattttgctttgattgtgaatgcacccaactatgcaggaattcaagctggagttgacaggttttatcgaacctgtaagctgctcggaattaatatgagcaaaaagaagtcttacataaacagaacaggtacctttgaattcacgagctttttctatcgttatgggtttgttgccaatttcagcatggagcttcctagttttggggtgtctggggtcaatgaatctgcagacatgagtattggagtcactgtcatcaagaacaatatgataaacaatgaccttggcccagcaactgctcaaatggcccttcagttatttataaaagattacaggtacacttatcgatgccacagaggtgacacacaaatacaaacccggagatcatttgagataaagaaactatgggaccaaacccgctccaaagctgggctgttggtctctgatggaggccccaatttatataacattagaaatctccatattcctgaagtttgcttgaaatgggagttgatggatgaggattaccaggggcgtttatgcaacccattaaacccgtttgtcagccataaagagattgaatcagtgaacaatgcagtgataatgccggcacatggcccagccaaaaatatggagtatgacgctgttgcaacaacacactcctgggtccccaaaagaaatcgatccattttgaacacgagccaaagggggatacttgaagatgagcaaatgtatcagaggtgctgcaatttatttgaaaaattcttcccaagtagctcatacagaagaccagttggaatatccagtatggtagaggctatggtttcaagagcccgaattgatgcacggattgatttcgaatctggaaggataaagagggaggaatttgctgagatcatgaagatctgttccaccattgaagacctcagacggcaaaaatag

>H1N1_PB1_CY027441_A/Illinois/UR06-0094/2007

atggatgtcaatccgacattacttttcttaaaagtgccagcacaaaatgctataagcacaacttttccttatactggtgaccctccttacagccatgggacaggaacagggtacaccatggatacagtcaacaggacacaccagtactcagaaagaggaagatggacaaaaaataccgaaactggagcaccgcaactcaacccaattgatgggccactaccagaagacaatgaaccgagtggctatgcccaaacagattgtgtattagaagcaatggctttccttgaagaatcccatcctggtatctttgaaaactcttgtattgagacaatggaggttgttcagcaaacaagggtggacaaactaacacaaggcagacagacctatgactggactctaaataggaaccagcctgctgccacagcattggcaaacactatagaagtattcagatcaaacggcctcatagcaaatgaatctgggaggctaatagacttccttaaagatgtaatggagtcgatggacatagacgaaatagaggtcacaactcattttcaaagaaagaggagagtgagagacaatgtaactaaaaaaatggtgacccaaagaacaataggcaaaaagaaacataaattagacaaaagaagttacctaattagggcattaaccctgaacacaatgaccaaagatgctgagagagggaaactaaaacgcagagcaattgcaaccccaggaatgcaaataagggggtttgtatactttgttgagacactggcaagaagcatatgtgaaaagcttgaacaatcaggattgccagttggaggaaatgagaagaaagcaaagttagcaaatgttgtaaggaagatgatgaccaactcccaggacactgaaatttctttcaccatcactggagataacacaaaatggaacgaaaatcaaaaccctagaatgttcttggccatgatcacatatataaccaaaaatcagcctgaatggttcagaaatattctaagtattgccccaataatgttttcaaacaagatggcgagactaggtaaggggtacatgtttgaaagcaagagtatgaaactgagaactcaaatacctgcagagatgctagccaacatagatttgaaatatttcaatgattcaactaaaaagaaaattgaaaaaatccgaccattattaatagatggaactgcatcattgagtcctggaatgatgatgggcatgttcaatatgttgagcaccgtcttgggcgtctccattctgaatcttgggcaaaagagatacaccaagactacttactggtgggatggtcttcaatcgtctgatgattttgctttgattgtgaatgcacccaactatgcaggaattcaagctggggttgacaggttttatcgaacctgtaagctgctcggaattaatatgagcaaaaagaagtcttacataaacagaacaggtacctttgaattcacgagctttttctatcgttatgggtttgttgccaatttcagcatggagcttcctagttttggggtgtctggggtcaatgaatctgcagacatgagtattggagtcactgtcatcaaaaacaatatgataaacaatgaccttggcccagcaactgctcaaatggcccttcagttatttataaaagattacaggtacacttatcgatgccacagaggtgacacacaaatacaaacccggagatcatttgagataaagaaactatgggaccaaacccgctccaaagctgggctgttggtctctgatggaggccccaatttatataacattagaaatctccatattcctgaagtttgcttgaaatgggagttgatggatgaggattaccaggggcgtttatgcaacccattaaacccgtttgtcagccataaagagattgaatcagtaaacaatgcagtgataatgccggcacatggtccagccaaaaatatggagtatgacgctgttgcaacaacacactcctgggtccccaaaagaaatcgatccattttgaacacgagccaaagggggatacttgaagatgagcaaatgtatcagaggtgctgcaatttatttgaaaaattcttcccaagtagctcatacagaagaccagttggaatatccagtatggtagaggctatggtttcaagagcccgaattgatgcacggattgatttcgaatctggaaggataaagaaagaggaatttgctgagatcatgaagatctgttccaccattgaagacctcagacggcaaaaatag

>H1N1_PB1_CY027665_A/Texas/UR06-0133/2007

atggatgtcaatccgacattacttttcttaaaagtgccagcacaaaatgctataagcacaacttttccttatactggtgaccctccttacagccatgggacaggaacagggtacaccatggatacagtcaacaggacacaccagtactcagaaagaggaagatggacaaaaaataccgaaactggagcaccgcaactcaacccaattgatgggccactaccagaagacaatgaaccaagtggctatgcccaaacagattgtgtattagaagcaatggctttccttgaagaatcccatcctggtatctttgaaaactcttgtattgagacaatggaggttgttcagcaaacaagggtggacaaactgacacaaggcagacagacctatgactggactctaaataggaaccagcctgctgccacagcattggcaaacactatagaagtattcagatcaaacggcctcatagcaaatgaatctgggaggctaatagacttccttaaagatgtaatggagtcgatggacagagacgaagtagaggtcacaactcattttcaaaggaagaggagagtgagagacaatgtaactaaaaaaatggtgacccaaagaacaataggcaaaaagaaacataaattagacaaaagaagttacctaattagggcattaaccctgaacacaatgaccaaagatgctgagagggggaaactaaaacgcagagcaattgcaaccccaggaatgcaaataagggggtttgtatactttgttgagacactggcaagaagcatatgtgaaaagcttgaacaatcaggattgccagttggaggaaatgagaagaaagcaaagttagcaaatgttgtaaggaagatgatgaccaactcccaggacactgaaatttctttcaccatcactggagataacacaaaatggaacgaaaatcaaaatcctagaatgttcttggccatgatcacatatataaccaaaaatcagcctgaatggttcagaaatattctaagtattgccccaataatgttttcaaacaagatggcgagactaggtaaggggtacatgtttgaaagcaagagtatgaaactgagaactcaaatacctgcagagatgctagccaacatagatttgaaatatttcaatgattcaactaaaaagaaaattgaaaaaatccgaccattattaatagatggaactgcatcattgagtcctggaatgatgatgggcatgttcaatatgttgagcaccgtcttgggcgtctccattctgaatcttgggcaaaagagatacaccaagactacttactggtgggatggtcttcaatcgtctgatgattttgctttgattgtgaatgcacccaactatgcaggaattcaagctggagttgacaggttttatcgaacctgtaagctgctcggaattaatatgagcaaaaagaagtcttacataaacagaacaggtacctttgaattcacgagctttttctatcgttatgggtttgttgccaatttcagcatggagcttcctagttttggggtgtctggggtcaatgaatctgcagacatgagtattggagtcactgtcatcaaaaacaatatgataaacaatgaccttggcccagcaacagctcaaatggcccttcagttatttataaaagattacaggtacacttatcgatgccacagaggtgacacacaaatacaaacccggagatcatttgagataaagaaactatgggaccaaacccgctccaaagctgggctgttggtctctgatggaggccccaatttatataacattagaaatctccatattcctgaagtttgcttgaaatgggagttgatggatgaggattaccaggggcgtttatgcaacccattaaacccgtttgtcagccataaagagattgagtcagtgaacaatgcagtgataatgccggcacatggtccagccaaaaatatggagtatgacgctgttgcaacaacgcactcctgggtccccaaaagaaatcgatccattttgaacacgagccaaagggggatacttgaagatgagcaaatgtatcagagatgctgtaatttatttgaaaaattcttcccaagtagctcatacagaagaccagttggaatatccagtatggtagaggctatggtttcaagagcccgaattgatgcacggattgatttcgaatctggaaggataaagaaagaggaatttgctgagatcatgaagatctgttccaccattgaagacctcagacggcaaaaatag

>H1N1_PB1_CY027737_A/Tennessee/UR06-0508/2007

atggatgtcaatccgacattacttttcttaaaagtgccagcacaaaatgctataagcacaacttttccttatactggtgaccctccttacagccatgggacaggaacagggtacaccatggatacagtcaacaggacacaccagtactcagaaagaggaagatggacaaaaaataccgaaactggagcaccgcaactcaacccaattgatgggccactaccagaagacaatgaaccaagtggctatgcccaaacagattgtgtattagaagcaatggctttccttgaagaatcccatcctggtatctttgaaaactcttgtattgaaacaatggaggttgttcagcaaacaagggtggacaaactgacacaaggcagacagacctatgactggactctaaataggaaccagcctgctgccacagcattggcaaacactatagaagtattcagatcaaacggcctcatagcaaatgaatctgggaggctaatagacttccttaaagatgtaatggagtcgatggacagagacgaagtagaggtcacaactcattttcaaagaaagaggagagtgagagacaatgtaactaaaaaaatggtgacccaaagaacaataggcaaaaagaaacataaattagacaaaagaagttacctaattagggcattaaccctgaacacaatgaccaaagatgctgaaagggggaaactaaaacgcagagcaattgcaaccccaggaatgcaaataagggggtttgtgtactttgttgagacactggcaagaagcatatgtgaaaagcttgaacaatcaggattgccagttggaggaaatgagaagaaagcaaagttagcaaatgttgtaaggaagatgatgaccaactcccaggacactgaaatttctttcaccattactggagataacacaaaatggaacgaaaatcaaaatcctagaatgttcttggccatgatcacatatataaccaaaaatcagcctgaatggttcagaaatattctaagtattgctccaataatgttttcaaacaagatggcgagactaggtaaagggtacatgtttgaaagcaagagtatgaaactgagaactcaaatacctgcagaaatgctagccaacatagatttgaaatatttcaatgattcaactaaaaagaaaattgaaaaaatccgaccattattaatagatggaactgcatcattaagtcctggaatgatgatgggcatgttcaatatgttgagcaccgtcttgggcgtctccattctgaatcttgggcaaaagggatacaccaagactacttactggtgggatggtcttcaatcgtctgatgattttgctttgattgtgaatgcacccaactatgcaggaattcaagctggagttgacaggttttatcgaacctgtaagctgctcggaattaatatgagcaaaaagaagtcttacataaacagaacaggtacctttgaattcacgagctttttctatcgttatgggtttgttgccaatttcagcatggagcttcctagttttggggtgtctggggtcaatgaatctgcagacatgagtattggagtcactgtcatcaaaaacaatatgataaacaatgaccttggcccagcaactgctcaaatggcccttcagttatttataaaagattacaggtacacttatcgatgccacagaggtgacacacaaatacaaacccggagatcatttgagataaagaaactatgggaccaaacccactccaaagctgggctgttggtctctgatggaggccccaatttatataacattagaaatctccatattcctgaagtttgcttgaaatgggagttgatggatgaggattaccaggggcgtttatgcaacccattaaacccgtttgtcagccataaagagattgaatcagtgaacaatgcagtgataatgccggcacatggtccagccaagaatatggagtatgacgctgttgcaacaacacactcctgggtccccaaaagaaatcgatccattttgaacacgagccaaagggggatacttgaagatgagcaaatgtatcagaggtgctgcaatttatttgaaaaattcttcccaagtagctcatacagaagaccagttggaatatccagcatggtagaggctatggtttcaagagccagaattgatgcacggattgatttcgaatctggaaggataaagaaagaggaatttgctgagatcatgaagatctgttccaccattgaagacctcagacggcaaaaatag

>H1N1_PB1_CY027817_A/Texas/UR06-0039/2007

atggatgtcaatccgacattacttttcttaaaagtgccagcacaaaatgctataagcacaacttttccttatactggtgaccctccttacagccatgggacaggaacagggtacaccatggatacagtcaacaggacacaccagtactcagaaagaggaagatggacaaaaaataccgaaactggagcaccgcaactcaacccaattgatgggccactaccagaagacaatgaaccaagtggctatgcccaaacagattgtgtattagaagcaatggctttccttgaagaatcccatcctggtatctttgaaaactcttgtattgaaacaatggaggttgttcagcaaacaagggtggacaaactgacacaaggcagacagacctatgactggactctaaataggaaccagcctgctgcaacagcattggcaaacactatagaagtattcagatcaaacggcctcatagcaaatgaatctggaaggctaatagacttccttaaagatgtaatggagtcgatggacagagacgaagtagaggtcacaactcattttcaaagaaagaggagagtgagagacaatgtaactaaaaaaatggtgactcaaagaacaataggcaaaaagaaacataaattagacaaaagaagttacctaattagggcattaaccctgaacacaatgaccaaagatgctgagagggggaaactaaaacgcagagcaattgcaaccccaggaatgcaaataagagggtttgtatactttgttgagacactggcaagaagcatatgtgaaaagcttgaacaatcaggattgccagttggaggaaatgagaagaaagcaaagttagcaaatgttgtaaggaagatgatgaccaactcccaggacactgaaatttctttcaccatcactggagataacacaaaatggaacgaaaatcaaaaccctagaatgttcttggccatgatcacatatataaccaaaaatcagcctgaatggttcagaaatattctaagtattgctccaataatgttttcaaacaagatggcgagactaggtaaggggtacatgtttgaaagcaagagtatgaaactgagaactcaaatacctgcagagatgctagccaacatagatttgaaatatttcaatgattcaactaaaaagaaaattgaaaaaatccgaccattattaatagatggaactgcatcattgagtcctggaatgatgatgggcatgttcaatatgttgagcaccgtcttgggcgtctccattctgaatcttgggcaaaagagatacaccaagactacttactggtgggatggtcttcaatcgtctgatgattttgctttgattgtgaatgcacccaactatgcaggaattcaagctggagttgacaggttttatcgaacctgtaagctgctcggaattaatatgagcaaaaagaagtcttacataaacagaacaggtacctttgaattcacgagctttttctatcgttatgggtttgttgccaatttcagcatggagcttcctagttttggggtgtctggggtcaatgaatctgcagacatgagtattggagtcactgtcatcaaaaacaatatgataaacaatgaccttggcccagcaactgctcaaatggcccttcagttatttataaaagattacaggtacacttatcgatgccacagaggtgacacacaaatacaaacccggagatcatttgagataaagaaactatgggaccaaacccgctccaaagctgggctgttggtctctgatggaggccccaatttatataacattagaaatctccatattcctgaagtttgcttgaaatgggagttgatggatgaggattaccaggggcgtttatgcaacccattaaacccgtttgtcagccataaagagattgaatcagtgaacaatgcagtgataatgccggcacatggcccagccaaaactatggagtatgacgctgttgcaacaacacactcctgggtccccaaaagaaatcgatccattttgaacacgagccaaagggggatacttgaagatgagcaaatgtatcagaggtgctgcaatttatttgaaaaattcttcccaagtagctcatacagaagaccagttggaatatccagtatggtagaggctatggtttcaagagcccgaattgatgcacggattgatttcgaatctggaaggataaagagggaggaatttgctgagatcatgaagatctgttccaccattgaagacctcagacggcaaaaatag

>H1N1_PB1_CY028329_A/Texas/UR06-0026/2007

atggatgtcaatccgacattacttttcttaaaagtgccagcacaaaatgctataagcacaacttttccttatactggtgaccctccttacagccatggaacaggaacagggtacaccatggatacagtcaacaggacacatcagtactcagaaagaggaagatggacaaaaaataccgaaactggagcaccgcaactcaacccaattgatgggccactaccagaagacaatgagccaagtggctatgcccaaacagattgtgtattagaagcaatggctttccttgaagaatcccatcctggtatttttgaaaactcttgtattgaaacaatggaggttgttcagcaaacaagggtggacaaactgacacaaggcagacaaacctatgactggactctaaataggaaccagcctgccgccacagcattggcaaacactatagaagtattcagatcaaatggcctcatagcaaatgaatctgggaggctaatagacttccttaaagatgtaatggagtcgatggacagagaagaagtagaggtcacaactcattttcaaagaaagaggagagtgagagacaatgtaactaaaaaaatggtgacccaaagaacaatagggaaaaagaaacataaattagacaaaagaagttacctaattagggcattaaccctgaacacaatgaccaaagatgctgagagggggaaactaaaacgcagagcaattgcaaccccaggaatgcaaataagggggtttgtatactttgttgagacactggcaagaagcatatgtgaaaagcttgaacaatcagggttgccagttggaggaaatgagaagaaagcaaagttagcaaatgttgtaaggaagatgatgaccaactcccaggacactgaaatttctttcaccatcactggagataacacaaaatggaacgagaatcaaaaccctagaatgttcttggccatgatcacatatataaccaaaaatcagcctgaatggttcagaaatattctaagtattgctccaataatgttttcaaacaaaatggcgagactaggtagggggtatatgtttgaaagcaagagtatgaaactgagaacccaaatacctgcagagatgctagccaacatagatttgaaatacttcaatgattcaactaaaaagaaaattgaaaaaattcgaccattattaatagatggaactgcatcattgagtcctggaatgatgatgggcatgttcaatatgttaagcaccgtcttgggcgtctccattctgaatcttgggcaaaagagatacaccaagactacttactggtgggatggtcttcaatcgtctgatgattttgctttgattgtgaatgcacccaattatgcaggaattcaagctggagttgacaggttttatcgaacctgtaagctgctcggaattaatatgagcaaaaagaagtcttacataaacagaacaggtacctttgaattcacgagctttttctatcgttatgggtttgttgccaatttcagcatggagcttcctagttttggggtgtctggggtcaatgaatctgcagacatgagtattggagtcactgtcatcaaaaacaatatgataaacaatgaccttggcccagcaactgctcaaatggcccttcagttatttataaaagattataggtacacttatcgatgccacagaggtgacacacaaatacaaacccgaagatcatttgaaataaagaaactatgggaccaaacccgctccaaagctgggctgttggtctctgatggaggccccaatctatataacattaggaatctacatattcctgaagtctgcttgaaatgggagttgatggatgaggattaccaggggcgtttatgcaacccattgaacccgtttgtcagccataaagagattgaatcagtgaacaatgcagtgataatgccggcacatggtccagctaaaaatatggagtatgacgctgttgcaacaacacactcctgggtccccaaaagaaatcgatccattttgaacacgagccaaagagggatacttgaagatgagcaaatgtatcagaggtgctgcaatttatttgaaaaattcttcccaagtagctcatacagaagaccagttggaatatccagtatggtagaggctatggtttcaagagcccgaattgatgcacggattgatttcgaatctggaaggataaagaaagaggaattcgctgagatcatgaagacctgttccaccattgaagacctcagacggcaaaaatag

>H1N1_PB1_FJ445043_A/England/493/2006

atggatgtcaatccgacattacttttcttaaaagtgccagcacaaaatgctataagcacaacttttccttatactggcgaccctccttacagccatgggacaggaacaggatacaccatggatacagtcaacaggacacatcagtactcagaaagaggaagatggacaaaaaataccgaaactggagcacctcaactcaacccaattgatgggccactaccagaagacaatgaaccaagtggctatgcccaaacagattgtgtattagaagcaatggctttccttgaagagtcccatcctggtatttttgaaaactcttgtattgaaacaatggaggttgttcagcaaacaagggtggacaaactgacacaaggcagacagacctatgactggactttaaataggaaccagcctgctgccacagctttggcaaacactatagaagtattcagatcaaacggcctcatagcaaatgaatctgggaggctaatagacttccttaaagatgtaatggagtcgatggacagagacgaagtagaggtcacaactcattttcaaagaaagaggagagtgagagacaatgtaactaaaaaaatggtgacccaaagaacaataggcaaaaagaaacataaattagacaaaagaagttacctaattagggcattaaccctgaacacaatgaccaaagatgctgagagggggaaactaaaacgcagagcaattgcaaccccaggaatgcaaataagggggttcgtatactttgttgagacactggcaagaagcatatgtgaaaagcttgaacaatcagggttgccagttggaggaaatgagaagaaagcaaagttagcaaatgttgtaagaaagatgatgaccaactcccaggacactgaaatttctttcaccatcactggagataacacaaaatggaacgaaaatcaaaaccctagaatgttcttggccatgatcacatatataaccaaaaatcagcctgaatggttcagaaatattctaagtattgctccaataatgttttcaaacaagatggcgagactaggtaaggggtacatgtttgaaagcaagagtatgaaactgagaactcaaatacctgcagagatgctagccaacatagatttgaaatatttcaatgattcaactaaaaagaaaattgaaaaaatccgaccattattaatagatggaactgcatcattgagtcctggaatgatgatgggcatgttcaatatgttaagcaccgtcttgggcgtctccattctgaatcttgggcaaaagagatacaccaagactacttactggtgggatggtcttcaatcgtctgatgattttgctttgattgtgaatgcacccaactatgcaggaattcaagctggagttgacaggttttatcgaacctgtaagctgctcggaattaatatgagcaaaaagaaatcttacataaacagaacaggtacctttgaattcacgagctttttctatcgttatgggtttgttgccaatttcagcatggagcttcctagttttggggtgtctggggtcaatgaatctgcagacatgagtattggagtcactgttatcaaaaacaatatgataaacaatgaccttggcccagcaactgctcaaatggcccttcagttatttataaaagattacaggtacacttatcgatgccacagaggtgacacacaaatacaaacccggagatcatttgagataaagaaactatgggaccaaacccgctccaaagctgggctgttggtctctgatggaggccccaatttatataacattagaaatctccatattcctgaagtctgcttgaagtgggagttgatggatgaggattaccaggggcgtttatgcaacccattgaacccgtttgtcagccataaagagattgaatcagtgaacaatgcagtgataatgccggcacatggtccagccaaaaatatggagtatgacgctgttgcaacaacacactcctgggtccccaaaagaaaccgatccattttgaacacgagccaaagggggatacttgaagatgagcaaatgtatcagaggtgctgcaatttatttgaaaaattcttcccaagtagctcatacagaagaccagttggaatatccagtatggtagaggctatgatttcaagagcccgaattgatgcacggattgatttcgaatctggaaggataaagaaagaggaattcgctgagatcatgaagacctgttccaccattgaagacctcagacggcaaaaatag

>H1N1_PB1_FJ445026_A/England/557/2007

atggatgtcaatccgacattacttttcttaaaagtgccagcacaaaatgctataagcacaacttttccttatactggtgaccctccttacagccatgggacaggaacagggtacaccatggatacagtcaacaggacacaccagtactcagaaagaggaagatggacaaaaaataccgaaacgggagcaccgcaacttaacccaattgatggtcccttaccggaagacaatgaaccaagtggctatgcccaaacagattgtgtattagaagcaatggctttccttgaagaatcccatcccggtatctttgaaaactcttgtattgaaacaatggaggttgttcaacaaacaagggtggacaaactgacacaaggcagacagacctatgactggactctaaataggaaccagcctgctgccacagcattggcaaacactatagaagtattcagatcaaacggcctcatagcaaatgaatctgggaggctaatagacttccttaaagatgtaatggagtcgatggacagaggcgaagtagaggtcacaactcattttcaaagaaagaggagagtgagagacaatgtaactaaaaaaatggtgacccaaagaacaataggcaaaaagaaacataaattagacaaaagaagttacctaattagggcattaaccctgaacacaatgaccaaagatgctgagagggggaaactaaaacgcagagcaattgcaaccccaggaatgcaaataagggggtttgtatactttgttgagacactggcaagaagcatatgtgaaaagcttgaacaatcaggattgccagttggaggaaatgagaagaaagcaaagttagcaaatgttgtaaggaagatgatgaccaactcccaggacactgaaatttctttcaccataaccggagataacacaaaatggaacgaaaatcaaaaccctagaatgttcttggccatgatcacatatataaccaaaaatcagcctgaatggttcagaaatattctaagtattgctccaataatgttttcaaacaagatggcgagactaggtaaggggtacatgtttgaaagcaagagtatgaaactgagaactcaaatacctgcagagatgctagccaacatagacttgaaatatttcaatgattcaactaaaaagaaaattgaaaaaatccgaccattattaatagatggaactgcatcattgagtcctggaatgatgatgggcatgttcaatatgttgagcaccgtcttgggcgtctccattctgaatcttgggcaaaagagatacaccaagactacttactggtgggatggtcttcaatcgtctgatgattttgctttgattgtgaacgcacccaactatgcaggaattcaagctggagttgacaggttttatcgaacctgtaagctgctcggaattaatatgagcaaaaagaagtcttacataaacagaacaggtacctttgaattcacgagctttttctatcgttatgggtttgttgccaatttcagcatggagcttcctagttttggggtgtctggggtcaatgaatctgcagacatgagtattggagtcactgtcatcaaaaacaatatgataaacaatgaccttggcccagcaactgctcaaatggcccttcagttatttataaaagattacaggtacacttatcgatgccaccgaggtgacacacaaatacaaacccggagatcatttgagataaagaaactatgggaccaaacccgctcaaaagctggactgttggtctctgatggaggccccaatttgtataacattagaaatctccatattcctgaagtttgcttgaaatgggagttgatggatgaggattaccaggggcgtttatgcaacccattaaacccgtttgtcagccataaagagattgaatcagtgaacagtgcagtgataatgccggcacatggtccagccaaaaatatggagtatgacgctgttgcaacaacacactcctgggtccccaaaagaaatcgatccattttgaacacgagccaaagggggatacttgaagatgagcaaatgtatcagaggtgctgcaatttatttgaaaaattcttcccaagtagctcatacagaagaccagttggaatatccagtatggtagaggctatggtctcaagagcccgaattgatgcacggattgatttcgaatctggaaggataaagaaagaggaatttgctgagatcatgaagatctgttccaccattgaagacctcagacggcaaaaatga

>H1N1_PB1_CY036861_A/Texas/UR06-0156/2007

atggatgtcaatccgacattacttttcttaaaagtgccagcacaaaatgctataagcacaacttttccttatactggtgaccctccttacagccatgggacaggaacagggtacaccatggatacagtcaacaggacacaccagtactcagaaagaggaagatggacaaaaaataccgaaactggagcaccgcaactcaacccaattgatgggccactaccagaagacaatgaaccaagtggctatgcccaaacagattgtgtattagaagcaatggctttccttgaagaatcccatcctggtatctttgaaaactcttgtattgaaacaatggaggttgttcagcaaacaagggtggacaaactgacacaaggcagacagacctatgactggactctaaataggaaccagcctgctgcaacagcattggcaaacactatagaagtattcagatcaaacggcctcatagcaaatgaatctggaaggctaatagacttccttaaagatgtaatggagtcgatggacagagacgaagtagaggtcacaactcattttcaaagaaagaggagagtgagagacaatgtaactaaaaaaatggtgactcaaagaacaataggcaaaaagaaacataaattagacaaaagaagttacctaattagggcattaaccctgaacacaatgaccaaagatgctgagagggggaaactaaaacgcagagcaattgcaaccccaggaatgcaaataagagggtttgtatactttgttgagacactggcaagaagcatatgtgaaaagcttgaacaatcaggattgccagttggaggaaatgagaagaaagcaaagttagcaaatgttgtaaggaagatgatgaccaactcccaggacactgaaatttctttcaccatcactggagataacacaaaatggaacgaaaatcaaaaccctagaatgttcttggccatgatcacatatataaccaaaaatcagcctgaatggttcagaaatattctaagtattgctccaataatgttttcaaacaagatggcgagactaggtaaggggtacatgtttgaaagcaagagtatgaaactgagaactcaaatacctgcagagatgctagccaacatagatttgaaatatttcaatgattcaactaaaaagaaaattgaaaaaatccgaccattattaatagatggaactgcatcattgagtcctggaatgatgatgggcatgttcaatatgttgagcaccgtcttgggcgtctccattctgaatcttgggcaaaagagatacaccaagactacttactggtgggatggtcttcaatcgtctgatgattttgctttgattgtgaatgcacccaactatgcaggaattcaagctggagttgacaggttttaccgaacctgtaagctgctcggaattaatatgagcaaaaagaagtcttacataaacagaacaggtacctttgaattcacgagctttttctatcgttatgggtttgttgccaatttcagcatggagcttcctagttttggggtgtctggggtcaatgaatctgcagacatgagtattggagtcactgtcatcaaaaacaatatgataaacaatgaccttggcccagcaactgctcaaatggcccttcagttatttataaaagattacaggtacacttatcgatgccacagaggtgacacacaaatacaaacccggagatcatttgagataaagaaactatgggaccaaacccgctccaaagctgggctgttggtctctgatggaggccccaatttatataacattagaaatctccatattcctgaagtttgcttgaaatgggagttgatggatgaggattatcaggggcgtttatgcaacccattaaacccgtttgtcagccataaagagattgaatcagtgaacaatgcagtgataatgccggcacatggcccagccaaaaatatggagtatgacgctgttgcaacaacacactcctgggtccccaaaagaaatcgatccattttgaacacgagccaaagggggatacttgaagatgagcaaatgtatcagaggtgctgcaatttatttgagaaattcttcccaagtagctcatacagaagaccagttggaatatccagtatggtagaggctatggtttcaagagcccgaattgatgcacggattgatttcgaatctggaaggataaagagggaggaatttgctgagatcatgaagatctgttccaccattgaagacctcagacggcaaaaatag

>H1N1_PB1_CY037781_A/Tennessee/UR06-0379/2007

atggatgtcaatccgacattacttttcttaaaagtgccagcacaaaatgctataagcacaacttttccttatactggtgaccctccttacagccatgggacaggaacagggtacaccatggatacagtcaacaggacacaccagtactcagaaagaggaagatggacaaaaaataccgaaactggagcaccgcaactcaacccaattgatgggccactaccagaagacaatgaaccaagtggctatgcccaaacagattgtgtattagaagcaatggctttccttgaagaatcccatcctggtatctttgaaaactcttgtattgaaacaatggaggttgttcagcaaacaagggtggacaaactgacacaaggcagacagacctatgactggactctaaataggaaccagcctgctgccacagcattggcaaacactatagaagtattcagatcaaacggcctcatagcaaatgaatctgggaggctaatagacttccttaaagatgtaatggagtcgatggacagagacgaagtagaggtcacaactcattttcaaagaaagaggagagtgagagacaatgtaactaaaaaaatggtgacccaaagaacaataggcaaaaagaaacataaattagacaaaagaagttatctaattagggcattaaccctgaacacaatgaccaaagatgctgaaagggggaaactaaaacgcagagcaattgcaaccccaggaatgcaaataagggggtttgtgtactttgttgagacactggcaagaagcatatgtgaaaaacttgaacaatcaggattgccagttggaggaaatgagaagaaagcaaagttagcaaatgttgtaaggaagatgatgaccaactcccaggacactgaaatttctttcaccattactggagataacacaaaatggaacgaaaatcaaaaccctagaatgttcttggccatgatcacatatataaccaaaaatcagcctgaatggttcagaaatattctaagtattgctccaataatgttttcaaacaagatggcgagactaggtaaagggtacatgtttgaaagcaagagtatgaaactgagaactcaaatacctgcagaaatgctagccaacatagatttgaaatatttcaatgattcaactaaaaagaaaattgaaaaaatccgaccattattaatagatggaactgcatcattgagtcctggaatgatgatgggcatgttcaatatgttgagcaccgtcttgggcgtctccattctgaatcttgggcaaaagagatacaccaagactacttactggtgggatggtcttcaatcgtctgatgattttgctttgattgtgaatgcacccaactatgcaggaattcaagctggagttgacaggttttatcgaacctgtaagctgctcggaattaatatgagcaaaaagaagtcttacataaacagaacaggtacctttgaattcacgagctttttctatcgttatgggtttgttgccaatttcagcatggagcttcctagttttggggtgtctggggtcaatgaatctgcagacatgagtattggagtcactgtcatcaaaaacaatatgataaacaatgaccttggcccagcaactgctcaaatggcccttcagttatttataaaagattacaggtacacttatcgatgccacagaggtgacacacaaatacaaacccggagatcatttgagataaagaaactatgggaccaaacccactccaaagctgggctgttggtctctgatggaggccccaatttatataacattagaaatctccatattcctgaagtttgcttgaaatgggagttgatggatgaggattaccaggggcgtttatgcaacccattaaacccgtttgtcagccataaagagattgaatcagtgaacaatgcagtgataatgccggcacatggtccagccaagaatatggagtatgacgctgttgcaacaacacactcctgggtccccaaaagaaatcgatccattttgaacacgagccaaagggggatacttgaagatgagcaaatgtatcagaggtgctgcaatttatttgaaaaattcttcccaagtagctcatacagaagaccagttggaatatccagtatggtagaggctatggtttcaagagccagaattgatgcacggattgatttcgaatctggaaggataaagaaagaggaatttgctgagatcatgaagatctgttccaccattgaagacctcagacggcaaaaatag

>H1N1_PB1_CY039469_A/Managua/156.01/2008

atggatgtcaatccgacattacttttcttaaaagtgccagcacaaaatgctataagcacaacttttccttatactggtgaccctccttacagccatgggacaggaacagggtacaccatggatacagtcaacaggacacaccattactcagaaagaggaagatggacaaaaaataccgaaacaggagcaccgcaacttaacccaattgatggtcccctaccggaagacaatgaaccaagtggctatgcccaaacagattgtgtattagaagcaatggctttccttgaagaatcccatcctggtatctttgaaaactcttgtattgaaacaatggaggttgttcagcaaacaagggtggacaaactgacacaaggcagacagacctatgactggactctaaataggaaccagcctgctgccacagcattggcaaacactatagaagtattcagatcaaacggcctcatagcaaatgaatctgggaggctaatagacttccttaaagatgtaatggagtcgatggacagaggcgaagtagaggtcacaactcattttcaaagaaagaggagagtgagagacaatgtaactaaaaaaatggtgacccaaagaacaataggcaaaaagaaacataaattagacaaaagaagttacctaattagggcattaaccctgaacacaatgaccaaagatgctgagagggggaaactaaaacgcagagcaattgcaaccccaggaatgcaaataagggggtttgtatactttgttgagacactggcaagaagcatatgtgaaaagcttgaacaatcaggattgccagttggaggaaatgagaagaaagcaaagttagcaaatgttgtaaggaagatgatgaccaactcccaggacactgaaatttctttcaccataaccggagataacacaaaatggaacgaaaatcaaaaccctagaatgttcttggccatgatcacatatataaccaaaaatcagcctgaatggttcagaaatattctaagtattgctccaataatgttttcaaacaagatggcgaggctaggtaaggggtacatgtttgaaagcaagagtatgaaactgagaactcaaatacctgcagagatgctagccaacatagatttgaaatatttcaatgattcaactaaaaagaaaattgaaaaaatccgaccattattaatagatggaactgcatcattgagtcctggaatgatgatgggcatgttcaatatgttgagcaccgtcttgggcgtctccattctgaatcttgggcaaaagagatacaccaagactacttactggtgggatggtcttcaatcgtctgatgattttgctttgattgtgaatgcacccaactatgcaggaattcaagctggagttgacaggttttatcgaacctgtaagctgctcggaattaatatgagcaaaaagaagtcttacataaacagaacaggtacctttgaattcacgagctttttctatcgttatgggtttgttgccaatttcagcatggagcttcctagttttggggtgtctggggtcaatgaatctgcagacatgagtattggggtcactgtcatcaaaaacaatatgataaacaatgaccttggcccagcaactgctcaaatggcccttcagttatttataaaagattacaggtacacttatcgatgccaccgaggtgacacacaaatacaaacccggagatcatttgagataaagaaactatgggaccaaacccgctcaaaagctgggctgttggtctctgatggaggccccaatttatataacattagaaatctccatattcctgaagtttgcttgaaatgggagttgatggatgaggattaccaggggcgtttatgcaacccattaaacccgtttgtcagccataaagagattgaatcagtgaacaatgcagtgataatgccggcacatggtccagccaaaaatatggagtatgacgctgttgcaacaacacactcctgggtccccaaaagaaatcgatccattttgaacacgagccaaagggggatacttgaagatgagcaaatgtatcagaggtgctgcaatttatttgaaaaattcttcccaagtagctcatacagaagaccagttggaatatccagtatggtagaggctatggtctcaagagcccgaattgatgcacggattgatttcgaatctggaaggataaagaaagaggaatttgctgagatcatgaagatctgttccaccattgaagacctcagacggcaaaaatga

>H1N1_PB1_GQ323535_A/Missouri/04/2009

atggatgtcaatccgactctacttttcctaaaaattccagcgcaaaatgccataagcaccacattcccttatactggagatcctccatacagccatggaacaggaacaggatacaccatggacacagtaaacagaacacaccaatactcagaaaagggaaagtggacgacaaacacagagactggtgcaccccagctcaacccgattgatggaccactacctgaggataatgaaccaagtgggtatgcacaaacagactgtgttctagaggctatggctttccttgaagaatcccacccaggaatatttgagaattcatgccttgaaacaatggaagttgttcaacaaacaagggtagataaactaactcaaggtcgccagacttatgattggacattaaacagaaatcaaccggcagcaactgcattggccaacaccatagaagtctttagatcgaatggcctaacagctaatgagtcaggaaggctaatagatttcttaaaggatgtaatggaatcaatgaacaaagaggaaatagagataacaacccactttcaaagaaaaaggagagtaagagacaacatgaccaagaagatggtcacgcaaagaacaatagggaagaaaaaacaaagactgaataagagaggctatctaataagagcactgacattaaatacgatgaccaaagatgcagagagaggcaagttgaaaagaagggctatcgcaacacctgggatgcagattagaggtttcgtatactttgttgaaactttagctaggagcatttgcgaaaagcttgaacagtctgggctcccagtagggggcaatgaaaagaaggccaaactggcaaatgttgtgagaaagatgatgactaattcacaagacacagagatttctttcacaatcactggggacaacactaagtggaatgaaaatcaaaatcctcgaatgttcctggcgatgattacatatatcaccagaaatcaacccgagtggttcagaaacatcctgagcatggcacccataatgttctcaaacaaaatggcaagactagggaaagggtacatgttcgagagtaaaagaatgaagattcgaacacaaataccagcagaaatgctagcaagcattgacctgaagtacttcaatgaatcaacaaagaagagaattgagaaaataaggcctctcctaatagatggcacagcatcactgagtcctgggatgatgatgggcatgttcaacatgctaagtacggtcttgggagtctcgatactgaatcttggacaaaagaaatacaccaagacaatatactggtgggatgggctccaatcatccgacgattttgctctcatagtgaatgcaccaaaccatgagggaatacaagcaggagtggacagattctacaggacctgcaagttagtgggaatcaacatgagcaaaaagaagtcctatataaataagacagggacatttgaattcacaagctttttttatcgctatggatttgtggctaattttagcatggagctacccagctttggagtgtctggagtaaatgaatcagctgacatgagtattggagtaacagtgataaagaacaacatgataaacaatgaccttggacctgcaacggcccagatggctcttcaattgttcatcaaagactacagatacacatataggtgccataggggagacacacaaattcagacgagaagatcatttgagttaaagaagctgtgggatcaaacccaatcaaaggtagggctattagtatcagatggaggaccaaacttatacaatatacggaatcttcacattcctgaagtctgcttaaaatgggagctaatggatgatgattatcggggaagactttgtaatcccctgaatccctttgtcagtcataaagagattgattctgtaaacaatgctgtggtaatgccagcccatggtccagccaaaagcatggaatatgatgccgttgcaactacacattcctggattcccaagaggaatcgttctattctcaacacaagccaaaggggaattcttgaggatgaacagatgtaccagaagtgctgcaatctattcgagaaatttttccctagcagttcatataggagaccggttggaatttctagcatggtggaggccatggtgtctagggcccggattgatgccagggtcgacttcgagtcaggacggatcaagaaagaagagttctctgagatcatgaagatctgttccaccatagaagaactcagacggcaaaaataa

>H1N1_PB1_CY044355_A/South Korea/AF10/2008

atggatgtcaatccgacattacttttcttaaaagtgccagcacaaaatgctataagcacaacttttccctatactggtgaccctccttacagccatgggacaggaacagggtacaccatggatacagtcaacaggacacatcagtactcagaaagaggaagatggacaaaaaataccgaaactggagcaccgcaactcaacccaattgatgggccactaccagaagacaatgaaccaagtggctatgcccaaacagattgtgtattagaagcaatggctttccttgaagaatcccatcctggtatttttgaaaactcttgtattgaaacaatggagattgttcagcaaacaagggtggacaaactgacacaaggcagacagacctatgactggactctaaataggaaccagcctgctgccacagcattggccaacactatagaagtattcagatcaaacggcctcatagcaaatgaatctgggaggctaatagacttccttaaagatgtaatggagtcgatggacagagaagaagtagaggtcacaactcattttcagagaaagaggagagtgagagacaatgtaactaaaaaaatggtgacccaaagaacaataggcaaaaagaaacataaattagacaaaaggagttacctaattagagcattaaccctgaacacaatgaccaaagatgctgagagggggaaactaaaacgcagagcaattgcaaccccaggaatgcaaataagggggtttgtatactttgttgaaacactggcaagaagcatatgtgaaaagcttgaacaatcagggttgccagttgggggaaatgagaagaaagcaaagttagcaaatgttgtaaggaagatgatgaccaactcccaggacactgaaatttctttcaccatcactggagataacacaaaatggaacgaaaatcaaaaccctagaatgtttttggccatgatcacatatataaccagaaatcagcctgaatggttcagaaatattctaagtgttgctccaataatgttttcaaacaaaatggcgagactaggtaaggggtacatgtttgaaagcaagagtatgaaactgagaactcaaatacctgcagagatactagccgacatagatttgaagtatttcaatgagtcaactaaaaagaaaattgaaaaaatccgaccattattaatagatggaactgcatcattgagtcctggaatgatgatgggcatgttcaatatgctaagcactgtcttgggcgtctccattctgaatcttgggcaaaagagatacaccaagactacttactggtgggatggtctccaatcgtctgatgattttgctctgatagtgaatgcgcccaactatgcaggaattcaagctggagttgacaggttttatcgaacctgtaagctgctcggaattaatatgagcaaaaagaagtcttacataaacaggacgggtacctttgaattcacaagttttttctatcgttatgggtttgttgccaatttcagcatggagcttcctagttttggagtgtcaggggtcaatgaatctgcagacatgagtattggagtcactgttatcagaaacaatatgataaacaatgaccttggcccagcaactgctcaaatggcccttcagttatttataaaagattacagatacacgtatcgatgccacagaggtgacacacaaatacaaacccggagatcatttgagataaagaaattatgggaccaaacccgctccaaagctgggctgttggtttctgatggaggccccaatttatataacattagaaatctccatattcctgaagtctgcttgaaatgggagttgatggatgaggattaccaggggcgtttatgcaacccattgaacccatttgtcagtcataaagagattgaatcagtgaacaatgcagtgatgatgccggcacatggtccagccaaaaatatggagtatgacgctgttgcaacaacacattcctgggttcccaaaaggaatcgatccattttaaacacgagccaaagggggatacttgaggatgagcaaatgtatcagaggtgctgcaatttatttgaaaaattctttccaagtagctcatacagacgaccagttgggatatccagtatggtagaggctatggtttccagagcccgaattgatgcacggattgatttcgaatctggaaggataaagaaagaggaattcgctgagatcatgaagacctgttccaccattgaagacctcagacggcaaaaatag

>H1N1_PB1_CY044379_A/Boston/26/2008

atggatgtcaatccgacattacttttcttaaaagtgccagcacaaaatgctataagcacaacttttccttatactggtgaccctccttacagccatgggacaggaacaggatacaccatggatacagtcaacaggacacaccagtactcagaaagaggaagatggacaaaaaataccgaaactggagcaccgcaacttaacccaattgatggtccactaccggaagacaatgaaccaagtggctatgcccaaacagattgtgtattagaagcaatggctttccttgaagaatcccatcccggtatctttgaaaactcttgtattgaaacaatggaggttgttcagcaaacaagggtggacaaactgacacaaggcagacagacctatgactggactctaaataggaaccagcctgctgccacagcattggcaaacactatagaagtattcagatcaaacggcctcatagcaaatgaatctgggaggctaatagacttccttaaagatgtaatggagtcgatggacagaggcgaagtagaggtcacaactcattttcaaagaaagaggagagtgagagacaatgtaactaaaaaaatggtgacccaaagaacaataggcaaaaagaaacataaattagacaaaagaagttacctaattagggcattaaccctgaacacaatgaccaaagatgctgagagggggaaactaaaacgcagagcaattgcaaccccaggaatgcaaataagggggtttgtatactttgttgagacactggcaagaagcatatgtgaaaagcttgaacaatcaggattgccagttggaggaaatgagaagaaagcaaagttagcaaatgttgtaaggaagatgatgaccaactcccaggacactgaaatttctttcaccataaccggagataacacaaaatggaacgaaaatcaaaaccctagaatgttcttggccatgatcacatatataaccaaaaatcagcctgaatggttcagaaatgttctaagtattgctccaataatgttttcaaacaagatggcgagactaggtaaggggtacatgtttgaaagcaagagtatgaaactgagaactcaaatacctgcagagatgctagctaacatagatttgaaatatttcaatgattcaactaaaaagaaaattgaaaaaatccgaccattattaatagatggaactgcatcattgagtcctggaatgatgatgggcatgttcaatatgttgagcaccgtcttgggcgtctccattctgaatcttgggcaaaagagatacaccaagactacttactggtgggatggtcttcaatcgtctgatgattttgctttgattgtgaatgcacccaactatgcaggaattcaagctggagttgacaggttttatcgaacctgtaagctgctcggaattaatatgagcaaaaagaagtcttacataaacagaacaggtacctttgaattcacgagctttttctatcgttatgggtttgttgccaatttcagcatggagcttcctagttttggggtgtctgggatcaatgaatctgcagacatgagtattggagtcactgtcatcaaaaacaatatgataaacaatgaccttggcccagcaactgctcaaatggcccttcagttatttataaaagattacaggtacacttatcgatgccaccgaggtgacacacaaatacaaacccggagatcatttgagataaagaaactatgggaccaaacccgctcaaaagctgggctgttggtctctgatggaggccccaatttatataacattagaaatctccatattcctgaagtttgcttgaaatgggagttgatggatgaggattaccaggggcgtttatgcaacccattaaacccgtttgtcagccataaagagattgaatcagtgaacaatgcagtgataatgccggcacatggtccagccaaaaatatggagtatgacgctgttgcaacaacacactcctgggtccccaaaagaaatcgatccattttgaacacgagccaaagggggatacttgaagatgagcaaatgtatcagaggtgctgcaatttatttgaaaaattcttcccaagtagctcatacagaagaccagttggaatatccagtatggcagaggctatggtctcaagagcccgaattgatgcacggattgatttcgaatctggaaggataaagaaagaggaatttgctgagatcatgaagatctgttccaccattgaagacctcagacggcaaaaatga

>H1N1_PB1_CY044570_A/Boston/35/2008

atggatgtcaatccgacattacttttcttaaaagtgccagcacaaaatgctataagcacaacttttccttatactggtgaccctccttacagccatgggacaggaacaggatacaccatggatacagtcaacaggacacaccagtactcagaaagaggaagatggacaaaaaataccgaaactggagcaccgcaacttaacccaattgatggtccactaccggaagacaatgaaccaagtggctatgcccaaacagattgtgtattagaagcaatggctttccttgaagaatcccatcccggtatctttgaaaactcttgtattgaaacaatggaggttgttcagcaaacaagggtggacaaactgacacaaggcagacagacctatgactggactctaaataggaaccagcctgctgccacagcattggcaaacactatagaagtattcagatcaaacggcctcatagcaaatgaatctgggaggctaatagacttccttaaagatgtaatggagtcgatggacagaggcgaagtagaggtcacaactcattttcaaagaaagaggagagtgagagacaatgtaactaaaaaaatggtgacccaaagaacaataggcaaaaagaaacataaattagacaaaagaagttacctaattagggcattaaccctgaacacaatgaccaaagatgctgagagggggaaactaaaacgcagagcaattgcaaccccaggaatgcaaataagggggtttgtatactttgttgagacactggcaagaagcatatgtgaaaagcttgaacaatcaggattgccagttggaggaaatgagaagaaagcaaagttagcaaatgttgtaaggaagatgatgaccaactcccatgacactgaaatttctttcaccataaccggagataacacaaaatggaacgaaaatcaaaaccctagaatgttcttggccatgatcacatatataaccaaaaatcagcctgaatggttcagaaatgttctaagtattgctccaataatgttttcaaacaagatggcgagactaggtaaggggtacatgtttgaaagcaagagtatgaaactgagaactcaaatacctgcagagatgctagctaacatagatttgaaatatttcaatgattcaactaaaaagaaaattgaaaaaatccgaccattattaatagatggaactgcatcattgagtcctggaatgatgatgggcatgttcaatatgttgagcaccgtcttgggcgtctccattctgaatcttgggcacaagagatacaccaagactacttactggtgggatggtcttcaatcgtctgatgattttgctttgattgtgaatgcacccaactatgcaggaattcaagctggagttgacaggttttatcgaatctgtaagctgctcggaattaatatgagcaaaaagaagtcttacataaacagaacaggtacctttgaattcacgagctttttctatcgttatgggtttgttgccaatttcagcatggagcttcctagttttggggtgtctgggatcaatgaatctgcagacatgagtattggagtcactgtcatcaaaaacaatatgataaacaatgaccttggcccagcaactgctcaaatggcccttcagttatttataaaagattacaggtacacttatcgatgccaccgaggtgacacacaaatacaaacccggagatcatttgagataaagaaactatgggaccaaacccgctcaaaagctgggctgttggtctctgatggaggccccaatttatataacattagaaatctccatattcctgaagtttgcttgaaatgggagttgatggatgaggattaccaggggcgtttatgcaacccattaaacccgtttgtcagccataaagagattgaatcagtgaacaatgcagtgataatgccggcacatggtccagccaaaaatatggagtatgacgctgttgcaacaacacactcctgggtccccaaaagaaatcgatccattttgaacacgagccaaagggggatacttgaagatgagcaaatgtatcagaggtgctgcaatttatttgaaaaattcttcccaagtagctcatacagaagaccagttggaatatccagtatggcagaggctatggtctcaagagcccgaattgatgcacggattgatttcgaatctggaaggataaagaaagaggaatttgctgagatcatgaagatctgttccaccattgaagacctcagacggcaaaaatga

>H1N1_PB1_GQ502904_A/Toronto/R8557/2009

atggatgtcaatccgactctacttttcctaaaaattccagcgcaaaatgccataagcaccacattcccttatactggagatcctccatacagccatggaacaggaacaggatacaccatggacacagtaaacagaacacaccaatactcagaaaagggaaagtggacgacaaacacagagactggtgcaccccagctcaacccgattgatggaccactacctgaggataatgaaccaagtgggtatgcacaaacagactgtgttctagaggctatggctttccttgaagaatcccacccaggaatatttgagaattcatgccttgaaacaatggaagttgttcaacaaacaagggtagataaactaactcaaggtcgccagacttatgattggacattaaacagaaatcaaccggcagcaactgcattggccaacaccatagaagtctttagatcgaatggcctaacagctaatgagtcaggaaggctaatagatttcttaaaggatgtaatggaatcaatgaacaaagaggaaatagagataacaacccactttcaaagaaaaaggagagtaagagacaacatgaccaagaagatggtcacgcaaagaacaatagggaagaaaaaacaaagactgaataagagaggctatctaataagagcactgacattaaatacgatgaccaaagatgcagagagaggcaagttaaaaagaagggctatcgcaacacctgggatgcagattagaggtttcgtatactttgttgaaactttagctaggagcatttgcgaaaagcttgaacagtctgggctcccagtagggggcaatgaaaagaaggccaaactggcaaatgttgtgagaaagatgatgactaattcacaagacacagagatttctttcacaatcactggggacaacactaagtggaatgaaaatcaaaatcctcgaatgttcctggcgatgattacatatatcaccagaaatcaacccgagtggttcagaaacatcctgagcatggcacccataatgttctcaaacaaaatggcaagactagggaaagggtacatgttcgagagtaaaagaatgaagattcgaacacaaataccagcagaaatgctagcaagcattgacctgaagtacttcaatgaatcaacaaagaagaaaattgagaaaataaggcctcttctaatagatggcacagcatcactgagtcctgggatgatgatgggcatgttcaacatgctaagtacggtcttgggagtctcgatactgaatcttggacaaaagaaatacaccaagacaatatactggtgggatgggctccaatcatccgacgattttgctctcatagtgaatgcaccaaaccatgagggaatacaagcaggagtggacagattctacaggacctgcaagttagtgggaatcaacatgagcaaaaagaagtcctatataaataagacagggacatttgaattcacaagctttttttatcgctatggatttgtggctaattttagcatggagctacccagctttggagtgtctggagtaaatgaatcagctgacatgagtattggagtaacagtgataaagaacaacatgataaacaatgaccttggacctgcaacggcccagatggctcttcaattgttcatcaaagactacagatacacatataggtgccataggggagacacacaaattcagacgagaagatcatttgagttaaagaagctgtgggatcaaacccaatcaaaggtggggctattagtatcagatggaggaccaaacttatacaatatacggaatcttcacattcctgaagtctgcttaaaatgggagctaatggatgatgattatcggggaagactttgtaatcccctgaatccctttgtcagtcataaagagattgattctgtaaacaatgctgtggtaatgccagcccatggtccagccaaaagcatggaatatgatgccgttgcaactacacattcctggattcccaagaggaatcgttctattctcaacacaagccaaaggggaattcttgaggatgaacagatgtaccagaagtgctgcaatctattcgagaaatttttccctagcagttcatataggagaccggttggaatttctagcatggtggaggccatggtgtctagggcccggattgatgccagggtcgacttcgagtctggacggatcaagaaagaagagttctctgagatcatgaagatctgttccaccattgaagaactcagacggcaaaaataa

>H1N1_PB1_CY044891_A/New York/3580/2009

atggatgtcaatccgactctacttttcctaaaaattccagcgcaaaatgccataagcaccacattcccttatactggagatcctccatacagccatggaacaggaacaggatacaccatggacacagtaaacagaacacaccaatactcagaaaagggaaagtggacgacaaacacagagactggtgcaccccagctcaacccgattgatggaccactacctgaggataatgaaccaagtgggtatgcacaaacagactgtgttctagaggctatggctttccttgaagaatcccacccaggaatatttgagaattcatgccttgaaacaatggaagttgttcaacaaacaagggtagataaactaactcaaggtcgccagacttatgattggacattaaacagaaatcaaccggcagcaactgcattggccaacaccatagaagtctttagatcgaatggcctaacagctaatgagtcaggaaggctaatagatttcttaaaggatgtaatggaatcaatgaacaaagaggaaatagagataacaacccactttcaaagaaaaaggagagtaagagacaacatgaccaagaagatggtcacgcaaagaacaatagggaagaaaaaacaaagactgaataagagaggctatctaataagagcactgacattaaatacgatgaccaaagatgcagagagaggcaagttaaaaagaagggctatcgcaacacctgggatgcagattagaggtttcgtatactttgttgaaactttagctaggagcatttgcgaaaagcttgaacagtctgggctcccagtagggggcaatgaaaagaaggccaaactggcaaatgttgtgagaaagatgatgactaattcgcaagacacagagatttctttcacaatcactggggacaacactaagtggaatgaaaatcaaaatcctcgaatgttcctggcgatgattacatatatcaccagaaatcaacccgagtggttcagaaacatcctgagcatggcacccataatgttctcaaacaaaatggcaagactagggaaagggtacatgttcgagagtaaaagaatgaagattcgaacacaaataccagcagaaatgctagcaagcattgacctgaagtacttcaatgaatcaacaaagaagaaaattgagaaaataaggcctcttctaatagatggcacagcatcactgagtcctgggatgatgatgggcatgttcaacatgctaagtacggtcttgggagtctcgatactgaatcttggacaaaagaaacacaccaagacaatatactggtgggatgggctccaatcatccgacgattttgctctcatagtgaatgcaccaaaccatgagggaatacaagcaggagtggacagattctacaggacctgcaagttagtgggaatcaacatgagcaaaaagaagtcctatataaataagacagggacatttgaattcacaagctttttttatcgctatggatttgtggctaattttagcatggagctacccagctttggagtgtctggagtaaatgaatcagctgacatgagtattggagtaacagtgataaagaacaacatgataaacaatgaccttggacctgcaacggcccagatggctcttcaattgttcatcaaagactacagatacacatataggtgccataggggagacacacaaattcagacgagaagatcatttgagttaaagaagctgtgggatcaaacccaatcaaaggtagggctattagtatcagatggaggaccaaacttatacaatatacggaatcttcacattcctgaagtctgcttaaaatgggagctaatggatgatgattatcggggaagactttgtaatcccctgaatccctttgtcagtcataaagagattgattctgtaaacaatgctgtggtaatgccagcccatggtccagccaaaagcatggaatatgatgccgttgcaactacacattcctggattcccaagaggaatcgttctattctcaacacaagccaaaggggaattcttgaggatgaacagatgtaccagaagtgctgcaatctattcgagaaatttttccctagcagttcatataggagaccggttggaatttctagcatggtggaggccatggtgtctagggcccggattgatgccagggtcgacttcgagtctggacggatcaagaaagaagagttctctgagatcataaagatctgttccaccattgaagaactcagacggcaaaaataa

>H1N1_PB1_CY046649_A/Wisconsin/629-D02227/2009

atggatgtcaatccgactctacttttcctaaaaattccagcgcaaaatgccataagcaccacattcccttatactggagatcctccatacagccatggaacaggaacaggatacaccatggacacagtaaacagaacacaccaatactcagaaaagggaaagtggacgacaaacacagagactggtgcaccccagctcaacccgattgatggaccactacctgaggataatgaaccaagtgggtatgcacaaacagactgtgttctagaggctatggctttccttgaagaatcccacccaggaatatttgagaattcatgccttgaaacaatggaagttgttcaacaaacaagggtagataaactaactcaaggtcgccagacttatgattggacattaaacagaaatcaaccggcagcaactgcattggccaacaccatagaagtctttagatcgaatggcctaacagctaatgagtcaggaaggctaatagatttcttaaaggatgtaatggaatcaatgaacaaagaggaaatagagataacaacccactttcaaagaaaaaggagagtaagagacaacatgaccaagaagatggtcacgcaaagaacaatagggaagaaaaaacaaagactgaataagagaggctatctaataagagcactgacattaaatacgatgaccaaagatgcagagagaggcaagttaaaaagaagggctatcgcaacacctgggatgcagattagaggtttcgtatactttgttgaaactttagctaggagcatttgcgaaaagcttgaacagtctgggctcccagtagggggcaatgaaaagaaggccaaactggcaaatgttgtgagaaagatgatgactaattcacaagacacagagatttctttcacaatcactggggacaacactaagtggaatgaaaatcaaaatcctcgaatgttcctggcgatgattacatatatcaccagaaatcaacccgagtggttcagaaacatcctgagcatggcacccataatgttctcaaacaaaatggcaagactagggaaagggtacatgttcgagagtaaaagaatgaagattcgaacacaaataccagcagaaatgctagcaagcattgacctgaagtacttcaatgaaccaacaaagaagaaaattgagaaaataaggcctcttctaatagatggcacagcatcactgagtcctgggatgatgatgggcatgttcaacatgctaagtacggtcttgggagtctcgatactgaatcttggacaaaagaaatacaccaagacaatatactggtgggatgggctccaatcatccgacgattttgctctcatagtgaatgcaccaaaccatgagggaatacaagcaggagtggacagattctacaggacctgcaagttagtgggaatcaacatgagcaaaaagaagtcctatataaataagacagggacatttgaattcacaagctttttttatcgctatggatttgtggctaattttagcatggagctacccagctttggagtgtctggagtaaatgaatcagctgacatgagtattggagtaacagtgataaagaacaacatgataaacaatgaccttggacctgcaacggcccagatggctcttcaactgttcatcaaagactacagatacacatatagatgccataggggagacacacaaattcagacgagaagatcatttgagttaaagaagctgtgggatcaaacccaatcaaaggtagggctattagtatcagatggaggaccaaacttatacaatatacggaatcttcacattcctgaagtctgcttaaaatgggagctaatggatgatgattatcggggaagactttgtaatcccctgaatccctttgtcagtcataaagagattgattccgtaaacaatgctgtggtaatgccagcccatggtccagccaaaagcatggaatatgatgccgttgcaactacacattcctggattcccaagaggaatcgttctattctcaacacaagccaaaggggaattcttgaggatgaacagatgtaccagaagtgctgcaatctattcgagaaatttttccctagcagttcatataggagaccggttggaatttctagcatggtggaggccatggtgtctagggcccggattgatgccagggtcgacttcgagtctggacggatcaagaaagaagagttctctgagatcatgaagatctgttccaccattgaagaactcagacggcaaaaataa

>H1N1_PB1_CY047356_A/New York/3365/2009

atggatgtcaatccgactctacttttcctaaaaattccagcgcaaaatgccataagcaccacattcccttatactggagatcctccatacagccatggaacaggaacaggatacaccatggacacagtaaacagaacacaccaatactcagaaaagggaaagtggacgacaaacacagagactggtgcaccccagctcaacccgattgatggaccactacctgaggataatgaaccaagtgggtatgcacaaacagactgtgttctagaggctatggctttccttgaagaatcccacccaggaatatttgagaattcatgccttgaaacaatggaagttgttcaacaaacaagggtagataaactaactcaaggtcgccagacttatgattggacattaaacagaaatcaaccggcagcaactgcattggccaacaccatagaagtctttagatcgaatggcctaacagctaatgagtcaggaaggctaatagatttcttaaaggatgtaatggaatcaatgaacaaagaggaaatagagataacaacccactttcaaagaaaaaggagagtaagagacaacatgaccaagaagatggtcacgcaaagaacaatagggaagaaaaaacaaagactgaataagagaggctatctaataagagcactgacattaaatacgatgaccaaagatgcagagagaggcaagttaaaaagaagggctatcgcaacacctgggatgcagattagaggtttcgtatactttgttgaaactttagctaggagcatttgcgaaaagcttgaacagtctgggctcccagtagggggcaatgaaaagaaggccaaactggcaaatgttgtgagaaagatgatgactaattcacaagacacagagatttctttcacaatcactggggacaacactaagtggaatgaaaatcaaaatcctcgaatgttcctggcgatgattacatatatcaccagaaatcaacccgagtggttcagaaacatcctgagcatggcacccataatgttctcaaacaaaatggcaagactagggaaagggtacatgttcgagagtaaaagaatgaagattcgaacacaaataccagcagaaatgctagcaagcattgacctgaagtacttcaatgaatcaacaaagaagaaaattgagaaaataaggcctcttctaatagatggcacagcatcactgagtcctgggatgatgatgggcatgttcaacatgctaagtacggtcttgggagtctcgatactgaatcttggacaaaagaaatacaccaagacaatatactggtgggatgggctccaatcatccgacgattttgctctcatagtgaatgcaccaaaccatgagggaatacaagcaggagtggacagattctacaggacctgcaagttagtgggaatcaacatgagcaaaaagaagtcctatataaataagacagggacatttgaattcacaagctttttttatcgctatggatttgtggctaattttagcatggagctacccagctttggagtgtctggagtaaatgaatcagctgacatgagtattggagtaacagtgataaagaacaacatgataaacaatgaccttggacctgcaacggcccagatggctcttcaattgttcatcaaagactacagatacacatataggtgccataggggagacacacaaattcagacgagaagatcatttgagttaaagaagctgtgggatcaaacccaatcaaaggtagggctattagtatcagatggaggaccaaacttatacaatatacggaatcttcacattcctgaagtctgcttaaaatgggagctaatggatgatgattatcggggaagactttgtaatcccctgaatccctttgtcagtcataaagagattgattctgtaaacaatgctgtggtaatgccagcccatggtccagccaaaagcatggaatatgatgccgttgcaactacacattcctggattcccaagaggaatcgttctattctcaacacaagccaaaggggaattcttgaggatgaacagatgtaccagaagtgctgcaatctattcgagaaatttttccctagcagttcatataggagaccggttggaatttctagcatggtggaggccatggtgtctagggcccggattgatgccagggtcgacttcgagtctggacggatcaagaaagaagagttctctgagatcatgaagatctgttccaccattgaagaactcagacggcaaaaataa

>H1N1_PB1_CY049897_A/Moscow/0253T/2009

atggatgtcaatccgactctacttttcctaaaaattccagcgcaaaatgccataagcaccacattcccttatactggagatcctccatacagccatggaacaggaacaggatacaccatggacacagtaaacagaacacaccaatactcagaaaagggaaagtggacgacaaacacagagactggtgcaccccagctcaacccgattgatggaccactacctgaggataatgaaccaagtgggtatgcacaaacagactgtgttctagaggctatggctttccttgaagaatcccacccaggaatatttgagaattcatgccttgaaacaatggaagttgttcaacaaacaagggtagataaactaactcaaggtcgccagacttatgattggacattaaacagaaatcaaccggcagcaactgcattggccaacaccatagaagtctttagatcgaatggcctaacagctaatgagtcaggaaggctaatagatttcttaaaggatgtaatggaatcaatgaacaaagaggaaatagagataacaacccactttcaaagaaaaaggagagtaagagacaacatgaccaagaagatggtcacgcaaagaacaatagggaagaaaaaacaaagactgaataagagaggctatctaataagagcactgacattaaatacgatgaccaaagatgcagagagaggcaagttaaaaagaagggctatcgcaacacctgggatgcagattagaggtttcgtatactttgttgaaactttagctaggagcatttgcgaaaagcttgaacagtctgggctcccagtagggggcaatgaaaagaaggccaaactggcaaatgttgtgagaaagatgatgactaattcacaagacacagagatttctttcacaatcactggggacaacactaagtggaatgaaaatcaaaatcctcgaatgttcctggcgatgattacatatatcaccagaaatcaacccgagtggttcagaaacatcctgagcatggcacccataatgttctcaaacaaaatggcaagactagggaaagggtacatgttcgagagtaaaagaatgaagattcgaacacaaataccagcagaaatgctagcaagcattgacctgaagtacttcaatgaatcaacaaagaagaaaattgagaaaataaggcctcttctaatagatggaacagcatcactgagtcctgggatgatgatgggcatgttcaacatgctaagtacggtcttgggagtctcgatactgaatcttggacaaaagaaatacaccaagacaatatactggtgggatgggctccaatcatccgacgattttgctctcatagtgaatgcaccaaaccatgagggaatacaagcaggagtggacagattctacaggacctgcaagttagtgggaatcaacatgagcaaaaagaagtcctatataaataagacagggacatttgaattcacaagctttttttatcgctatggatttgtggctaattttagcatggagctacccagctttggagtgtctggagtaaatgaatcagctgacatgagtattggagtaacagtgataaagaacaacatgataaacaatgaccttggacctgcaacggcccagatggctcttcaattgttcatcaaagactacagatacacatataggtgccataggggagacacacaaattcagacgagaagatcatttgagttaaagaagctgtgggatcaaacccaatcaaaggtagggctattagtatcagatggaggaccaaacttatacaatatacggaatcttcacattcctgaagtctgcttaaaatgggagctaatggatgatgattatcggggaagactttgtaatcccctgaatccctttgtcagtcataaaaagattgattctgtaaacaatgctgtggtaatgccagcccatggtccagccaaaagcatggaatatgatgccgttgcaactacacattcctggattcccaagaggaatcgttctattctcaacacaagccaaaggggaattcttgaggatgaacagatgtaccagaagtgctgcaatctattcgagaaatttttccctagcagttcatataggagaccggttggaatttctagcatggtggaggccatggtgtctagggcccggattgatgccagggtcgacttcgagtctggacggatcaagaaagaagagttctctgagatcatgaagatctgttccaccattgaagaactcagacggcaaaaataa

>H1N1_PB1_CY049921_A/New Bern/WR0670/2009

atggatgtcaatccgactctacttttcctaaaaattccagcgcaaaatgccataagcaccacattcccttatactggagatcctccatacagccatggaacaggaacaggatacaccatggacacagtaaacagaacacaccaatactcagaaaagggaaagtggacgacaaacacagagactggtgcaccccagctcaacccgattgatggaccactacctgaggataatgaaccaagtgggtatgcacaaacagactgtgttctagaggctatggctttccttgaagaatctcacccaggaatatttgagaattcatgccttgaaacaatggaagttgttcaacaaacaagggtagataaactaactcaaggtcgccagacttatgattggacattaaacagaaatcaaccggcagcaactgcattggccaacaccatagaagtctttagatcgaatggcctaacagctaatgagtcaggaaggctaatagatttcttaaaggatgtaatggaatcaatgaacaaagaggaaatagagataacaacccactttcaaagaaaaaggagagtaagagacaacatgaccaagaagatggtcacgcaaagaacaatagggaagaaaaaacaaagactgaataagagaggctatctaataagagcactgacattaaatacgatgaccaaagatgcagagagaggcaagttaaaaagaagggctatcgcaacacctgggatgcagattagaggtttcgtatactttgttgaaactttagctaggagcatttgcgaaaagcttgaacagtctgggctcccagtagggggcaatgaaaagaaggccaaactggcaaatgttgtgagaaagatgatgactaattcacaagacacagagatttctttcacaatcactggggacaacactaagtggaatgaaaatcaaaatcctcgaatgttcctggcgatgattacatatatcaccagaaatcaacccgagtggttcagaaacatcctgagcatggcacccataatgttctcaaacaaaatggcaagactagggaaagggtacatgttcgagagtaaaagaatgaagattcgaacacaaataccagcagaaatgctagcaagcattgacctgaagtacttcaatgaatcaacaaagaagaaaattgagaaaataaggcctcttctaatagatggcacagcatcactgagtcctgggatgatgatgggcatgttcaacatgctaagtacggtcttgggagtctcgatactgaatcttggacaaaagaaatacaccaagacaatatactggtgggatgggctccaatcatccgacgattttgctctcatagtgaatgcaccaaaccatgagggaatacaagcaggagtggacagattctacaggacctgcaagttagtgggaatcaacatgagcaaaaggaagtcctatataaataagacagggacatttgaattcacaagctttttttatcgctatggatttgtggctaattttagcatggagctacccagctttggagtgtctggagtaaatgaatcagctgacatgagtattggagtaacagtgataaagaacaacatgataaacaatgaccttggacctgcaacggcccagatggctcttcaattgttcatcaaagactacagatacacatataggtgccataggggagacacacaaattcagacgagaagatcatttgagttaaagaagctgtgggatcaaacccaatcaaaggtagggctattagtatcagatggaggaccaaacttatacaatatacggaatcttcacattcctgaagtctgcttaaagtgggagctaatggatgatgattatcggggaagactttgtaatcccctgaatccctttgtcagtcataaagagattgattctgtaaacaatgctgtggtaatgccagcccatggtccagccaaaagcatggaatatgatgccgttgcaactacacattcctggattcccaagaggaatcgttctattctcaacacaagccaaaggggaattcttgaggatgaacagatgtaccagaagtgctgcaatctattcgagaaatttttccctagcagttcatataggagaccggttggaatttctagcatggtggaggccatggtgtctagggcccggattgatgccagggtcgactttgagtctggacggatcaagaaagaagagttctctgagatcatgaagatctgttccaccattgaagaactcagacggcaaaaataa

>H1N1_PB1_CY050049_A/Mexico City/WR1311T/2009

atggatgtcaatccgactctacttttcctaaaaattccagcgcaaaatgccataagcaccacattcccttatactggagatcctccatacagccatggaacaggaacaggatacaccatggacacagtaaacagaacacaccaatactcagaaaagggaaagtggacgacaaacacagagactggtgcaccccagctcaacccgattgatggaccactacctgaggataatgaaccaagtgggtatgcacaaacagactgtgttctagaggctatggctttccttgaagaatcccacccaggaatatttgagaattcatgccttgaaacaatggaagttgttcaacaaacaagggtagataaactgactcaaggtcgccagacttatgattggacattaaacagaaatcaaccggcagcaactgcattggccaacaccatagaagtctttagatcgaatggcctaacagctaatgagtcaggaaggctaatagatttcttaaaggatgtaatggaatcaatgaacaaagaggaaatagagataacaacccactttcaaagaaaaaggagagtaagagacaacatgaccaagaagatggtcacgcaaagaacaatagggaagaaaaaacaaagactgaataagagaggctatctaataagagcactgacattaaatacgatgaccaaagatgcagagagaggcaagttaaaaagaagggctatcgcaacacctgggatgcagattagaggtttcgtatactttgttgaaactttagctaggagcatttgcgaaaagcttgaacagtctgggctcccagtagggggcaatgaaaagaaggccaaattggcaaatgtggtgagaaagatgatgactaattcacaagacacagagatttctttcacaatcactggggacaacactaagtggaatgaaaatcaaaatcctcgaatgttcctggcgatgattacatatatcaccagaaatcaacccgagtggttcagaaacatcctgagcatggcacccataatgttctcaaacaaaatggcaagactagggaaagggtacatgttcgagagtaaaagaatgaagattcgaacacaaataccagcagaaatgctagcaagcattgacctgaagtacttcaatgaatcaacaaagaagaaaattgagaaaataaggcctcttctaatagatggcacagcatcactgagtcctgggatgatgatgggcatgttcaacatgctaagtacggtcttgggagtctcgatactgaatcttggacaaaagaaatacaccaagacaatatactggtgggatgggctccaatcatccgacgattttgctctcatagtgaatgcaccaaaccatgagggaatacaagcaggagtggacagattctacaggacctgcaagttagtgggaatcaacatgagcaaaaagaagtcctatataaataagacagggacatttgaattcacaagctttttttatcgctatggatttgtggctaattttagcatggagctacccagctttggagtgtctggagtaaatgaatcagctgacatgagtattggagtaacagtgataaagaacaacatgataaacaatgaccttggacctgcaacggcccagatggctcttcaattgttcatcaaagactacagatacacatataggtgccataggggagacacacaaattcagacgagaagatcatttgagttaaagaagctgtgggatcaaacccaatcaaagggagggctattagtatcagatggaggaccaaacttatacaatatacggaatcttcacattcctgaagtctgcttaaaatgggagctaatggatgatgattatcggggaagactttgtaatcccctgaatccctttgtcagtcataaagagattgattctgtaaacaatgctgtggtaatgccagcccatggtccagtcaaaagcatggaatatgatgccgttgcaactacacattcctggattcccaagaggaatcgttctattctcaacacaagccaaaggggaattcttgaggatgaacagatgtaccagaagtgctgcaatctattcgagaaatttttccctagcagttcatataggagaccggttggaatttctagcatggtggaggccatggtgtctagggcccggattgatgccagggtcgacttcgagtctggacggatcaagaaagaagagttctctgagatcatgaagatctgctccaccattgaagaactcagacggcaaaaataa

>H1N1_PB1_CY051389_A/Wisconsin/629-S0254/2009

atggatgtcaatccgactctacttttcctaaaaattccagcgcaaaatgccataagcaccacattcccttatactggagatcctccatacagccatggaacaggaacaggatacaccatggacacagtaaacagaacacaccaatactcagaaaagggaaagtggacgacaaacacagagactggtgcaccccagctcaacccgattgatggaccactacctgaggataatgaaccaagtgggtatgcacaaacagactgtgttctagaggctatggctttccttgaagaatcccacccaggaatatttgagaattcatgccttgaaacaatggaagttgttcaacaaacaagggtagataaactaactcaaggtcgccagacttatgattggacattaaacagaaatcaaccggcagcaactgcattggccaacaccatagaagtctttagatcgaatggcctaacagctaatgagtcaggaaggctaatagatttcttaaaggatgtaatggaatcaatgaacaaagaggaaatagagataacaacccactttcaaagaaaaaggagagtaagagacaacatgaccaagaagatggtcacgcaaagaacaatagggaagaaaaaacaaagactgaataagagaggctatctaataagagcactgacattaaatacgatgaccaaagatgcagagagaggcaagttaaaaagaagggctatcgcaacacctgggatgcagattagaggtttcgtatactttgttgaaactttagctaggagcatttgcgaaaagcttgaacagtctgggctcccagtagggggcaatgaaaagaaggccaaactggcaaatgttgtgagaaagatgatgactaattcacaagacacagagatttctttcacaatcactggggacaacactaagtggaatgaaaatcaaaatcctcgaatgttcctggcgatgattacatatatcaccagaaatcaacccgagtggttcagaaacatcctgagcatggcacccataatgttctcaaacaaaatggcaagactagggaaagggtacatgttcgagagtaaaagaatgaagattcgaacacaaataccagcagaaatgctagcaagcattgacctgaagtacttcaatgaatcaacaaagaagaaaattgagaaaataaggcctcttctaatagatggcacagcatcactgagtcctgggatgatgatgggcatgttcaacatgctaagtacggtcttgggagtctcgatactgaatcttggacaaaagaaatacaccaagacaatatactggtgggatgggctccaatcatccgacgattttgctctcatagtgaatgcaccaaaccatgagggaatacaagcaggagtggacagattctacaggacctgcaagttagtgggaatcaacatgagcaaaaagaagtcctatataaataagacagggacatttgaattcacaagctttttttatcgctatggatttgtggctaattttagcatggagctacccagctttggagtgtctggagtaaatgaatcagctgacatgagtattggagtaacagtgataaagaacaacatgataaacaatgaccttggacctgcaacggcccagatggctcttcaattgttcatcaaagactacagatacacatatagatgccataggggagacacacaaattcagacgagaagatcatttgagttaaagaagctgtgggatcaaacccaatcaaaggtagggctattagtatcagatggaggaccaaacttatacaatatacggaatcttcacattcctgaagtctgcttaaaatgggagctaatggatgatgattatcggggaagactttgtaatcccctgaatccctttgtcagtcataaagagattgattctgtaaacaatgctgtggtaatgccagcccatggtccagccaaaagcatggaatatgatgccgttgcaactacacattcctggattcccaagaggaatcgttctattctcaacacaagccaaaggggaattcttgaggatgaacagatgtaccagaagtgctgcaatctattcgagaaatttttccctagcagttcatataggagaccggttggaatttctagcatggtggaggccatggtgtctagggcccggattgatgccagggtcgacttcgagtctggacggatcaagaaagaagagttctctgagatcatgaagatctgttccaccattgaagaactcagacggcaaaaataa

>H1N1_PB1_CY051597_A/New York/4565/2009

atggatgtcaatccgactctacttttcctaaaaattccagcgcaaaatgccataagcaccacattcccttatactggagatcctccatacagccatggaacaggaacaggatacaccatggacacagtaaacagaacacaccaatactcagaaaagggaaagtggacgacaaacacagagactggtgcaccccagctcaacccgattgatggaccactacctgaggataatgaaccaagtgggtatgcacaaacagactgcgttctagaggctatggctttccttgaagaatcccacccaggaatatttgagaattcatgccttgaaacaatggaagttgttcaacaaacaagggtagataaactaactcaaggtcgccagacttatgattggacattaaacagaaatcaaccggcagcaactgcattggccaacaccatagaagtctttagatcgaatggcctaacagctaatgagtcaggaaggctaatagatttcttaaaggatgtaatggaatcaatgaacaaagaggaaatagagataacaacccactttcaaagaaaaaggagagtaagagacaacatgaccaagaagatggtcacgcaaagaacaatagggaagaaaaaacaaagactgaataagagaggctatctaataagagcactgacattaaatacgatgaccaaagatgcagagagaggcaagttaaaaagaagggctatcgcaacacctgggatgcagattagaggtttcgtatactttgttgaaactttagctaggagcatttgcgaaaagcttgaacagtctgggctcccagtagggggcaatgaaaagaaggccaaactggcaaatgttgtgagaaagatgatgactaattcacaagacacagagatttctttcacaatcactggggacaacactaagtggaatgaaaatcaaaatcctcgaatgttcctggcgatgattacatatatcaccagaaatcaacccgagtggttcagaaacatcctgagcatggcacccataatgttctcaaacaaaatggcaagactagggaaagggtacatgttcgagagtaaaagaatgaagattcgaacacaaataccagcagaaatgctagcaagcattgacctgaagtacttcaatgaatcaacaaagaagaaaattgagaaaataaggcctcttctaatagatggcacagcatcactgagtcctgggatgatgatgggcatgttcaacatgctaagtacggtcttgggagtctcgatactaaatcttggacaaaagaaatacaccaagacaatatactggtgggatgggctccaatcatccgacgattttgctctcatagtgaatgcaccaaaccatgagggaatacaagcaggagtggacagattctacaggacctgcaagttagtgggaatcaacatgagcaaaaagaagtcctatataaataagacagggacatttgaattcacaagctttttttatcgctatggatttgtggctaattttagcatggagctacccagctttggagtgtctggagtaaatgaatcagctgacatgagtattggagtaacagtgataaagaacaacatgataaacaatgaccttggacctgcaacggcccagatggctcttcaattgttcatcaaagactacagatacacatataggtgccataggggagacacacaaattcagacgagaagatcatttgagttaaagaagctgtgggatcaaacccaatcaaaggtagggctattagtatcagatggaggaccaaacttatacaatatacggaatcttcacattcctgaagtctgcttaaaatgggagctaatggatgatgattatcggggaagactttgtaatcccctgaatccctttgtcagtcataaagagattgattctgtaaacaatgctgtggtaatgccagcccatggtccagccaaaagcatggaatatgatgccgttgcaactacacattcctggattcccaagaggaatcgttctattctcaacacaagccaaaggggaattcttgaggatgaacagatgtaccagaagtgctgcaatctatttgagaaatttttccctagcagttcatataggagaccggttggaatttctagcatggtggaggccatggtgtctagggcccggattgatgccagggtcgacttcgagtctggacggatcaagaaagaagagttctctgagatcatgaagatctgttccaccattgaagaactcagacggcaaaaataa

>H1N1_PB1_GU290053_A/Prague/196-81511/2009

atggatgtcaatccgactctacttttcctaaaaattccagcgcaaaatgccataagcaccacattcccttatactggagatcctccatacagccatggaacaggaacaggatacaccatggacacagtaaacagaacacaccaatactcagaaaagggaaagtggacgacaaacacagagactggtgcaccccagctcaacccgattgatggaccactacctgaggataataaaccaagtgggtatgcacaaacagactgtgttctagaggctatggctttccttgaagaatcccacccaggaatatttgagaattcatgccttgaaacaatggaagttgttcaacaaacaagggtagataaactaactcaaggtcgccagacttatgattggacattaaacagaaatcaaccggcagcaactgcattggccaacaccatagaagtctttagatcgaatggcctaacagctaatgagtcaggaaggctaatagatttcttaaaggatgtaatggaatcaatgaacaaagaggaaatagagataacaacccactttcaaagaaaaaggagagtaagagacaacatgaccaagaagatggtcacgcaaagaacaatagggaagaaaaaacaaagactgaataagagaggctatctaataagagcactgacattaaatacgatgaccaaagatgcagagagaggcaagttaaaaagaagggctatcgcaacacctgggatgcagattagaggtttcgtatactttgttgaaactttagctaggagcatttgcgaaaagcttgaacagtctgggctcccagtagggggcaatgaaaagaaggccaaactggcaaatgttgtgagaaagatgatgactaattcacaagacacagagatttctttcacaatcactggggacaacactaagtggaatgaaaatcaaaatcctcgaatgttcctggcgatgattacatatatcaccagaaatcaacccgagtggttcagaaacatcctgagcatggcacccataatgttctcaaacaaaatggcaagactagggaaagggtacatgttcgagagtaaaagaatgaagattcgaacacaaataccagcagaaatgctagcaagcattgacctgaagtacttcaatgaatcaacaaagaagaaaattgagaaaataaggcctcttctaatagatggcacagcatcactgagtcctgggatgatgatgggcatgttcaacatgctaagtacggtcttgggagtctcgatactgaatcttggacaaaagaaatacaccaagacaatatactggtgggatgggctccaatcatccgacgattttgctctcatagtgaatgcaccaaaccatgagggaatacaagcaggagtggacagattctacaggacctgcaagttagtgggaatcaacatgagcaaaaagaagtcctatataaataagacagggacatttgaattcacaagctttttttatcgctatggatttgtggctaattttagcatggagctacccagctttggagtgtctggagtaaatgaatcagctgacatgagtattggagtaacagtgataaagaacaacatgataaacaatgaccttggacctgcaacggcccagatggctcttcaattgttcatcaaagactacagatacacatataggtgccataggggagacacacaaattcagacgagaagatcatttgagttaaagaagctgtgggatcaaacccaatcaaaggtagggctattagtatcagatggaggaccaaacttatacaatatacggaatcttcacattcctgaagtctgcttaaaatgggagctaatggatgatgattatcggggaagactttgtaatcccctgaatccctttgtcagtcataaagagattgattctgtaaacaatgctgtggtaatgccagcccatggtccagccaaaagcatggaatatgatgccgttgcaactacacattcctggattcccaagaggaatcgttctattctcaacacaagccaaaggggaattcttgaggatgaacagatgtaccagaagtgctgcaatctattcgagaaattttttcctagcagttcatataggagaccggttggaatttctagcatggtggaggccatggtgtctagggcccggattgatgccagggtcgacttcgagtctggacggatcaagaaagaagagttctctgagatcatgaagatctgttccaccattgaagaactcagacggcaaaaataa

>H1N1_PB1_CY052445_A/Texas/45122538/2009

atggatgtcaatccgactctacttttcctaaaaattccagcgcaaaatgccataagcaccacattcccttatactggagatcctccatacagccatggaacaggaacaggatacaccatggacacagtaaacagaacacaccaatactcagaaaagggaaagtggacgacaaacacagagactggtgcaccccagctcaacccgattgatggaccactacctgaggataatgaaccaagtgggtatgcacaaacagactgtgttctagaggctatggctttccttgaagaatcccacccaggaatatttgagaattcatgccttgaaacaatggaagttgttcaacaaacaagggtagataaactaactcaaggtcgccagacttatgattggacattaaacagaaatcaaccggcagcaactgcattggccaacaccatagaagtctttagatcgaatggcctaacagctaatgagtcaggaaggctaatagatttcttaaaggatgtaatggaatcaatgaacaaagaggaaatagagataacaacccactttcaaagaaaaaggagagtaagagacaacatgaccaagaagatggtcacgcaaagaacaatagggaagaaaaaacaaagactgaataagagaggctatctaataagagcactgacattaaatacgatgaccaaagatgcagagagaggcaagttaaaaagaagggctatcgcaacacctgggatgcagattagaggtttcgtatactttgttgaaactttagctaggagcatttgcgaaaagcttgaacagtctgggctcccagtagggggcaatgaaaagaaggccaaactggcaaatgttgtgagaaagatgatgactaattcacaagacacagagatttctttcacaatcactggggacaacactaagtggaatgaaaatcaaaatcctcgaatgttcctggcgatgattacatatatcaccagaaatcaacccgagtggttcagaaacatcctgagcatggcacccataatgttctcaaacaaaatggcaagactagggaaagggtacatgttcgagagtaaaagaatgaagattcgaacacaaataccagcagaaatgctagcaagcattgacctgaagtacttcaatgaatcaacaaagaagaaaattgagaaaataaggcctcttctaatagatggcacagcatcactgagtcctgggatgatgatgggcatgttcaacatgctaagtacggtcttgggagtctcgatactgaatcttggacaaaagaaatacaccaagacaatatactggtgggatgggctccaatcatccgacgattttgctctcatagtgaatgcaccaaaccatgagggaatacaagcaggagtggacagattctacaggacctgcaagttagtgggaatcaacatgagcaaaaagaagtcctatataaataaaacagggacatttgaattcacaagctttttttatcgctatggatttgtggctaattttagcatggagctacccagctttggagtgtctggagtaaatgaatcagctgacatgagtattggagtaacagtgataaagaacaacatgataaacaatgaccttggacctgcaacggcccagatggctcttcaactgttcatcaaagactacagatacacatataggtgccataggggagacacacaaattcagacgagaagatcatttgagttaaagaagctgtgggatcaaacccaatcaaaggtagggctattagtatcagatggaggaccaaacttatacaatatacggaatcttcacattcctgaagtctgcttaaaatgggagctaatggatgatgattatcggggaagactttgtaatcccctgaatccctttgtcagtcataaagagattgattctgtaaacaatgctgtggtaatgccagcccatggtccagccaaaagcatggaatatgatgccgttgcaactacacattcctggattcccaagaggaatcgttctattctcaacacaagccaaaggggaattcttgaggatgaacagatgtaccagaagtgctgcaatctattcgagaaatttttccctagcagttcatataggagaccggttggaatttctagcatggtggaggccatggtgtctagggcccggattgatgccagggtcgacttcgagtctggacggatcaagaaagaagagttctctgagatcatgaagatctgttccaccattgaagaactcagacggcaaaaataa

>H1N1_PB1_CY052677_A/Texas/44313703/2009

atggatgtcaatccgactctacttttcctaaaaattccagcgcaaaatgccataagcaccacattcccttatactggagatcctccatacagccatggaacaggaacaggatacaccatggacacagtaaacagaacacaccaatactcagaaaagggaaagtggacgacaaacacagagactggtgcaccccagctcaacccgattgatggaccactacctgaggataatgaaccaagtgggtatgcacaaacagactgtgttctagaggctatggctttccttgaagaatcccacccaggaatatttgagaattcatgccttgaaacaatggaagttgttcaacaaacaagggtagataaactaactcaaggtcgccagacttatgattggacattaaacagaaatcaaccggcagcaactgcattggccaacaccatagaagtctttagatcgaatggcctaacagctaatgagtcaggaaggctaatagatttcttaaaggatgtaatggaatcaatgaacaaagaggaaatagagataacaacccactttcaaagaaaaaggagagtaagagacaacatgaccaagaagatggtcacgcaaagaacaatagggaagaaaaaacaaagactgaataagagaggctatctaataagagcactgacattaaatacgatgaccaaagatgcagagagaggcaagttaaaaagaagggctatcgcaacacctgggatgcagattagaggtttcgtatactttgttgaaactttagctaggagcatttgcgagaagcttgaacagtctgggctcccagtagggggcaatgaaaagaaggccaaactggcaaatgttgtgagaaagatgatgactaattcacaagacacagagatttctttcacaatcactggggacaacactaagtggaatgaaaatcaaaatcctcgaatgttcctggcgatgattacatatatcaccagaaatcaacccgagtggttcagaaacatcctgagcatggcacccataatgttctcaaacaaaatggcaagactagggaaagggtacatgttcgagagtaaaagaatgaagattcgaacacaaataccagcagaaatgctagcaagcattgacctgaagtacttcaatgaatcaacaaagaagaaaattgagaaaataaggcctcttctaatagatggcacagcatcactgagtcctgggatgatgatgggcatgttcaacatgctaagtacggtcttgggagtctcgatactgaatcttggacaaaagaaatacaccaagacaatatactggtgggatgggctccaatcatccgacgattttgctctcatagtgaatgcaccaaaccatgagggaatacaagcaggagtggacagattctacaggacctgcaagttagtgggaatcaacatgagcaaaaagaagtcctatataaataagacagggacatttgaattcacaagctttttttatcgctatggatttgtggctaattttagcatggagctacccagctttggagtgtctggagtaaatgaatcagctgacatgagtattggagtaacagtgataaagaacaacatgataaacaatgaccttggacctgcaacggcccagatggctcttcaattgttcatcaaagactacagatacacatataggtgccataggggagacacacaaattcagacgagaagatcatttgagttaaagaagctgtgggatcaaacccaatcaaaggtagggctattagtatcagatggaggaccaaacttatacaatatacggaatcttcacattcctgaagtctgcttaaaatgggagctaatggatgatgattatcggggaagactttgtaatcccctgaatccctttgtcagtcataaagagattgattctgtaaacaatgctgtggtaatgccagcccatggtccagccaaaagcatggaatatgatgccgttgcaactacacattcctggattcccaagaggaatcgttctattctcaacacaagccaaaggggaattcttgaggatgaacagatgtaccagaagtgctgcaatctattcgagaaatttttccctagcagttcatataggagaccggttggaatttctagcatggtggaggccatggtgtctagggcccggattgatgccagggtcgactccgagtctggacggatcaagaaagaagagttctctgagatcatgaagatctgttccaccattgaagaactcagacggcaaaaataa

>H1N1_PB1_CY052821_A/Texas/45072656/2009

atggatgtcaatccgactctacttttcctaaaaattccagcgcaaaatgccataagcaccacattcccttatactggagatcctccatacagccatggaacaggaacaggatacaccatggacacagtaaacagaacacaccaatactcagaaaagggaaagtggacgacaaacacagagactggtgcaccccagctcaacccgattgatggaccactacctgaggataatgagccaagtgggtatgcacaaacagactgtgttctagaggctatggctttccttgaagaatcccacccaggaatatttgagaattcatgccttgaaacaatggaagttgttcaacaaacaagggtagataaactaactcaaggtcgccagacttatgattggacattaaacagaaatcaaccggcagcaactgcattggccaacaccatagaagtctttagatcgaatggcctaacagctaatgagtcaggaaggctaatagatttcttaaaggatgtaatggaatcaatgaacaaagaggaaatagagataacaacccactttcaaagaaaaaggagagtaagagacaacatgaccaagaagatggtcacacaaagaacaatagggaagaaaaaacaaagactgaataagagaggctatctaataagagcactgacattaaatacgatgaccaaagatgcagagagaggcaagttaaaaagaagggctatcgcaacacctgggatgcagattagaggtttcgtatactttgttgaaactttagctaggagcatttgcgaaaagcttgaacagtctgggctcccagtagggggcaatgaaaagaaggccaaactggcaaatgttgtgagaaagatgatgactaattcacaagacacagagatttctttcacaatcactggggacaacactaagtggaatgaaaatcaaaatcctcgaatgttcctggcgatgattacatatatcaccagaaatcaacccgagtggttcagaaacatcctgagcatggcacccataatgttctcaaacaaaatggcaagactagggaaagggtacatgttcgagagtaaaagaatgaagattcgaacacaaataccagcagaaatgctagcaagcattgacctgaagtacttcaatgaatcaacaaagaagaaaattgaaaaaataaggcctcttctaatagatggcacagcatcactgagtcctgggatgatgatgggcatgttcaacatgctaagtacggttttgggagtctcgatactgaatcttggacaaaagaaatacaccaagacaatatactggtgggatgggctccaatcatccgacgattttgctctcatagtgaatgcaccaaaccatgagggaatacaagcaggagtggacagattctacaggacctgcaagttagtgggaatcaacatgagcaaaaagaagtcctatataaataagacagggacatttgaattcacaagctttttttatcgctatggatttgtggctaattttagcatggagctacccagctttggagtgtctggagtaaatgaatcagctgacatgagtattggagtaacagtgataaagaacaacatgataaacaatgaccttggacctgcaacggcccagatggctcttcaattgttcatcaaagactacagatacacatataggtgccataggggagacacacaaattcagacgagaagatcatttgagttaaagaagctgtgggatcaaacccaatcaaaggtagggctattagtatcagatggaggaccaaacttatacaatatacggaatcttcacattcctgaagtctgcttaaaatgggagctaatggatgatgattatcggggaagactttgtaatcccctgaatccctttgtcagtcataaagagattgattctgtaaacaatgctgtggtaatgccagcccatggtccagccaaaagcatggaatatgatgccgttgcaactacacattcctggattcccaagaggaatcgttctattctcaacacaagccaaaggggaattcttgaggatgaacagatgtaccagaagtgttgcaatctattcgagaaatttttccctagcagttcatataggagaccggttggaatttctagcatggtggaggccatggtgtctagggcccggattgatgccagggtcgacttcgagtctggacggatcaagaaagaagagttctctgagatcatgaagatctgttccaccattgaagaactcagacggcaaaaataa

>H1N1_PB1_CY053414_A/Russia/149/2009

atggatgtcaatccgactctacttttcctaaaagttccagcgcaaaatgccataagcaccacattcccttatactggagatcctccatacagccatggaacaggaacaggatacaccatggacacagtaaacagaacacaccaatactcagaaaagggaaagtggacgacaaacacagagactggtgcaccccagctcaacccgattgatggaccactacctgaggataatgaaccaagtgggtatgcacaaacagactgtgttctagaggctatggctttccttgaagaatcccacccaggaatatttgagaattcatgccttgaaacaatggaagttgttcaacaaacaagggtagataaactaactcaaggtcgccagacttatgattggacattaaacagaaatcaaccggcagcaactgcattggccaacaccatagaagtctttagatcgaatggcctaacagctaatgagtcaggaaggctaatagatttcttaaaggatgtaatggaatcaatgaacaaagaggaaatagagataacaacccactttcaaagaaaaaggagagtaagagacaacatgaccaagaagatggtcacgcaaagaacaatagggaagaaaaaacaaagactgaacaagagaggctatctaataagagcactgacattaaatacgatgaccaaagatgcagagagaggcaagttaaaaagaagggctatcgcaacacctgggatgcagattagaggtttcgtatactttgttgaaactttagctaggagcatttgcgaaaagcttgaacagtctgggctcccagtagggggcaatgaaaagaaggccaaactggcaaatgttgtgagaaagatgatgactaattcacaagacacagagatttctttcacaatcactggggacaacactaagtggaatgaaaatcaaaatcctcgaatgttcctggcgatgattacatatatcaccagaaatcaacccgagtggttcagaaacatcctgagcatggcacccataatgttctcaaacaaaatggcaagactagggaaagggtacatgttcgagagtaaaagaatgaagattcgaacacaaataccagcagaaatgctagcaagcattgacctgaagtacttcaatgaatcaacaaagaagaaaattgagaaaataaggcctcttctaatagatggcacagcatcactgagtcctgggatgatgatgggcatgttcaacatgctaagtacggtcttgggagtctcgatactgaatcttggacaaaagaaatacaccaagacaatatactggtgggatgggctccaatcatccgacgattttgctctcatagtgaatgcaccaaaccatgagggaatacaagcaggagtggacagattctacaggacctgcaagttagtgggaatcaacatgagcaaaaagaagtcctatataaataagacagggacatttgaattcacaagctttttttatcgctatggatttgtggctaattttagcatggagctacccagctttggagtgtctggagtaaatgaatcagctgacatgagtattggagtaacagtgataaagaacaacatgataaacaatgaccttggacctgcaacggcccagatggctcttcaattgttcatcaaagactacagatacacatataggtgccataggggagacacacaaattcagacgagaagatcatttgagttaaagaagctgtgggatcaaacccaatcaaaggtagggctattagtatcagatggaggaccaaacttatacaatatacggaatcttcacattcctgaagtctgcttaaaatgggagctaatggatgatgattatcggggaagactttgtaatcccctgaatccctttgtcagtcataaagagattgattctgtaaacaatgctgtggtaatgccagcccatggtccagccaaaagcatggaatatgatgccgttgcaactacacattcctggattcccaagaggaatcgttctattctcaacacaagccaaaggggaattcttgaggatgaacagatgtaccagaagtgctgcaatctattcgagaaatttttccctagcagttcatataggagaccggttggaatttctagcatggtggaggccatggtgtctagggcccggattgatgccagggtcgacttcgagtctggacggatcaagaaagaagagttctctgagatcatgaagatctgttccaccattgaagaactcagacggcaaaaataa

>H1N1_PB1_CY053625_A/Russia/165/2009

atggatgtcaatccgactctacttttcctaaaaattccagcgcaaaatgccataagcaccacattcccttatactggagatcctccatacagccatggaacaggaacaggatacaccatggacacagtaaacagaacacaccaatactcagaaaagggaaagtggacgacaaacacagagactggtgcaccccagctcaacccgattgatggaccactacctgaggataatgaaccaagtgggtatgcacaaacagactgtgttctagaggctatggctttccttgaagaatcccacccaggaatatttgagaattcatgccttgaaacaatggaagttgttcaacaaacaagggtagataaactaactcaaggtcgccagacttatgattggacattaaacagaaatcaaccggcagcaactgcattggccaacaccatagaagtctttagatcgaatggcctaacagctaatgagtcaggaaggctaatagatttcttaaaggatgtaatggaatcaatgaacaaagaggaaatagagataacaacccactttcaaagaaaaaggagagtaagagacaacatgaccaagaagatggtcacgcaaagaacaatagggaagaaaaaacaaagactgaataagagaggctatctaataagagcactgacattaaatacgatgaccaaagatgcagagagaggcaagttaaaaagaagggctatcgcaacacctgggatgcagattagaggtttcgtatactttgttgaaactttagctaggagcatttgcgaaaagcttgaacagtctgggctcccagtagggggcaatgaaaagaaggccaaactggcaaatgttgtgagaaagatgatgactaattcacaagacacagagatttctttcacaatcactggggacaacactaagtggaatgaaaatcaaaatcctcgaatgttcctggcgatgattacatatatcaccagaaatcaacccgagtggttcagaaacatcctgagcatggcacccataatgttctcaaacaaaatggcaagactagggaaagggtacatgttcgagagtaaaagaatgaagattcgaacacaaataccagcagaaatgctagcaagcattgacctgaagtacttcaatgaatcaacaaagaagaaaattgagaaaataaggcctcttctaatagatggcacagcatcactgagtcctgggatgatgatgggcatgttcaacatgctaagtacggtcttgggagtctcgatactgaatcttggacaaaagaaatacaccaagacaatatactggtgggatgggctccaatcatccgacgattttgctctcatagtgaatgcaccaaaccatgagggaatacaagcaggagtggacagattctacaggacctgcaagttagtgggaatcaacatgagcaaaaagaagtcctatataaataagacagggacatttgaattcacaagctttttttatcgctatggatttgtggctaattttagcatggagctacccagctttggagtgtctggagtaaatgaatcagctgacatgagtattggagtaacagtgataaagaacaacatgataaacaatgaccttggacctgcaacggcccagatggctcttcaattgttcatcaaagactacagatacacatataggtgccataggggagacacacaaattcagacgagaagatcatttgagttaaagaagctgtgggatcaaacccaatcaaaggtagggctattagtatcagatggaggaccaaacttatacaatatacggaatcttcacattcctgaagtctgcttaaaatgggagctaatggatgatgattatcggggaagactttgtaatcccctgaatccctttgtcagtcataaagagattgattctgtaaacaatgctgtggtaatgccagcccatggtccagccaaaagcatggaatatgatgccgttgcaactacacattcctggattcccaagaggaatcgttctattctcaacacaagccaaaggggaattcttgaggatgaacagatgtaccagaagtgctgcaatctattcgagaaatttttccctagcagttcatataggagaccggttggaatttctagcatggtggaggccatggtgtctagggcccggattgatgccagggtcgacttcgagtctggacggatcaagaaagaagagttctctgagatcatgaagatctgttccaccattgaagaactcagacggcaaaaataa

>H1N1_PB1_CY053734_A/Russia/178/2009

atggatgtcaatccgactctacttttcctaaaaattccagcgcaaaatgccataagcaccacattcccttatactggagatcctccatacagccatggaacaggaacaggatacaccatggacacagtaaacagaacacaccaatactcagaaaagggaaagtggacgacaaacacagagactggtgcaccccagctcaacccgattgatggaccactacctgaggataatgaaccaagtgggtatgcacaaacagactgtgttctagaggctatggctttccttgaagaatcccacccaggaatatttgagaattcatgccttgaaacaatggaagttgttcaacaaacaagggtagataaactaactcaaggtcgccagacttatgattggacattaaacagaaatcaaccggcagcaactgcattggccaacaccatagaagtctttagatcgaatggcctaacagctaatgagtcaggaaggctaatagatttcttaaaggatgtaatggaatcaatgaacaaagaggaaatagagataacaacccactttcaaagaaaaaggagagtaagagacaacatgaccaagaagatggtcacgcaaagaacaatagggaagaaaaaacaaagactgaataagagaggctatctaataagagcactgacattaaatacgatgaccaaagatgcagagagaggcaagttaaaaagaagggctatcgcaacacctgggatgcagattagaggtttcgtatactttgttgaaactttagctaggagcatttgcgaaaagcttgaacagtctgggctcccagtagggggcaatgaaaagaaggccaaactggcaaatgttgtgagaaagatgatgactaattcacaagacacagagatttctttcacaatcactggggacaacactaagtggaatgaaaatcaaaatcctcgaatgttcctggcgatgattacatatatcaccagaaatcaacccgagtggttcagaaacatcctgagcatggcacccataatgttctcaaacaaaatggcaagactagggaaagggtacatgttcgagagtaaaagaatgaagattcgaacacaaataccagcagaaatgctagcaagcattgacctgaagtacttcaatgaatcaacaaagaagaaaattgagaaaataaggcctcttctaatagatggcacagcatcactgagtcctgggatgatgatgggcatgttcaacatgctaagtacggtcttgggagtctcgatactgaatcttggacaaaagaaatacaccaagacaatatactggtgggatgggctccaatcatccgacgattttgctctcatagtgaatgcaccaaaccatgagggaatacaagcaggagtggacagattctacaggacctgcaagttagtgggaatcaacatgagcaaaaagaagtcctatataaataagacagggacatttgaattcacaagctttttttatcgctatggatttgtggctaattttagcatggagctacccagctttggagtgtctggagtaaatgaatcagctgacatgagcattggagtaacagtgataaagaacaacatgataaacaatgaccttggacctgcaacggcccagatggctcttcaattgttcatcaaagactacagatacacatataggtgccataggggagacacacaaattcagacgagaagatcatttgagttaaagaagctgtgggatcaaacccaatcaaaggtagggctattagtatcagatggaggaccaaacttatacaatatacggaatcttcacattcctgaagtctgcttaaaatgggagctaatggatgatgattatcggggaagactttgtaatcccctgaatccctttgtcagtcataaagagattgattctgtaaacaatgctgtggtaatgccagcccatggtccagccaaaagcatggaatatgatgccgttgcaactacacattcctggattcccaagaggaatcgttctattctcaacacaagccaaaggggaattcttgaggatgaacagatgtaccagaagtgctgcaatctattcgagaaatttttccctagcagttcatataggagaccgattggaatttctagcatggtggaggccatggtgtctagggcccggattgatgccagggtcgacttcgagtctggacggatcaagaaagaagagttctctgagatcatgaagatctgttccaccattgaagaactcagacggcaaaaataa

>H1N1_PB1_GU367323_A/Tomsk/07/2009

atggatgtcaatccgactctacttttcctaaaaattccagcgcaaaatgccataagcaccacattcccttatactggagatcctccatacagccatggaacaggaacaggatacaccatggacacagtaaacagaacacaccaatactcagaaaagggaaagtggacgacaaacacggagactggtgcaccccagctcaacccgattgatggcccactacctgaggataatgaaccaagtgggtatgcacaaacagactgtgttctagaggctatggctttccttgaagaatcccacccaggaatatttgagaattcatgccttgaaacaatggaagttgttcwacaaacaagggtagataaactaactcaaggtcgccagacttatgattggacattaaacagaaatcaaccggcagcaactgcattggccaacaccatagaagtctttagatcgaatggcctaacagctaatgagtcaggaaggctaatagatttcttaaaggatgtaatggaatcaatgaacaaagaggaaatagagataacaacccactttcaaagaaaaaggagagtaagagacaacatgaccaagaagatggtcacgcaaagaacaatagggaagaaaaaacaaagactgaataagagaggctatctaataagagcactgacattaaatacgatgaccaaagatgcagagagaggcaagttaaaaagaagggctatcgcaacacctgggatgcagattagaggtttcgtatactttgttgaaactttagctaggagcatttgcgaaaagctygaacagtctgggctcccagtagggggcaatgaaaagaaggccaaactggcaaatgttgtgagaaagatgatgactaattcacaagacacagagatttctttcacaatcactggggacaacactaagtggaatgaaaatcaaaatcctcgaatgttcctggcgatgattacatatatcaccagaaatcaacccgagtggttcagaaacatcctgagcatggcacccataatgttctcaaacaaaatggcaagactagggaaagggtacatgttcgagagtaaaagaatgaagattcgaacacaaataccagcagaaatgctagcaagcattgacctgaagtacttcaatgaatcaacaaagaagaaaattgagaaaataaggcctcttctaatagatggcacagcatcactgagtcctgggatgatgatgggcatgttcaacatgctaagtacggtcttgggagtctcgatactgaatcttggacaaaagaaatacaccaagacaatatactggtgggatgggctccaatcatccgacgattttgctctcatagtgaatgcaccaaaccatgagggaatacaagcaggagtggacagattctacaggacctgcaagttagtgggaatcaacatgagcaaaaagaagtcctatataaataagacagggacatttgaattcacaagcttttkttatcgctatggatttgtggctaattttagcatggagctacccagctttggagtgtctggagtaaatgaatcagctgacatgagtattggagtaacagtgataaagaacaatatgataaacaatgaccttggacctgcaacggcccagatggctcttcaattgttcatcaaagactacagatacacatataggtgccataggggagacacacaaattcagacgagaagatcatttgagttaaagaagctgtgggatcaaacccaatcaaaggtagggctattagtatcagatggaggaccaaacttatacaatatacggaatcttcacattcctgaagtctgcttaaaatgggagctaatggatgatgattatcggggaagactttgtaatcccctgaatccctttgtcagccataaagagattgattctgtaaacaatgctgtggtaatgccagcccatggtccagccaaaagcatggaatatgatgccgttgcaactacacattcctggattcccaagaggaatcgttctattctcaacacaagccaaaggggaattcttgaggatgaacagatgtaccagaagtgctgcaatctattcgagaaatttttccctagcagttcatataggagaccggttggaatttctagcatggtggaggccatggtgtctagggcccggattgatgccagggtcgacttcgagtctggacggatcaagaaagaagagttctctgagatcatgaagatctgttccaccattgaagaactcagacggcaaaaataa

>H1N1_PB1_CY054817_A/California/VRDL27/2009

atggatgtcaatccgactctacttttcctaaaaattccagcgcaaaatgccataagcaccacattcccttatactggagatcctccatacagccatggaacaggaacaggatacaccatggacacagtaaacagaacacaccaatactcagaaaagggaaagtggacgacaaacacagagactggtgcaccccagctcaacccgattgatggaccactacctgaggataatgaaccaagtgggtatgcacaaacagactgtgttctagaggctatggctttccttgaagaatcccacccaggaatatttgagaattcatgccttgaaacaatggaagttgttcaacaaacaagggtagataaactaactcaaggtcgccagacttatgattggacattaaacagaaatcaaccggcagcaactgcattggccaacaccatagaagtctttagatcgaatggcctaacagctaatgagtcaggaaggctaatagatttcttaaaggatgtaatggaatcaatgaacaaagaggaaatagagataacaacccactttcaaagaaaaaggagagtaagagacaacatgaccaagaagatggtcacgcaaagaacaatagggaagaaaaaacaaagactgaataagagaggatatctaataagagcactgacattaaatacgatgaccaaagatgcagagagaggcaagttaaaaagaagggctatcgcaacacctgggatgcagattagaggtttcgtatactttgttgaaactttagctaggagcatttgcgaaaagcttgaacagtctgggctcccagtagggggcaatgaaaagaaggccaaactggcaaatgttgtgagaaagatgatgactaattcacaagacacagagatttctttcacaatcactggggacaacactaagtggaatgaaaatcaaaatcctcgaatgttcctggcgatgattacatatatcaccagaaatcaacccgagtggttcagaaacatcctgagcatggcacccataatgttctcaaacaaaatggcaagactagggaaagggtacatgttcgagagtaaaagaatgaagattcgaacacaaataccagcagaaatgctagcaagcattgacctgaagtacttcaatgaatcaacaaagaagaaaattgagaaaataaggcctcttctaatagatggcgcagcatcactgagtcctgggatgatgatgggcatgttcaacatgctaagtacggtcttgggagtctcgatactgaatcttggacaaaagaaatacaccaagacagtatactggtgggatgggctccaatcatccgacgattttgctctcatagtgaatgcaccaaaccatgagggaatacaagcaggagtggacagattctacaggacctgcaagttagtgggaatcaacatgagcaaaaagaagtcctatataaataagacagggacatttgaattcacaagctttttttatcgctatggatttgtggctaattttagcatggagctacccagctttggagtgtctggagtaaatgaatcagctgacatgagtattggagtaacagtgataaagaacaacatgataaacaatgaccttggacctgcaacggcccagatggctcttcaattgttcatcaaagactacagatacacatataggtgccataggggagacacacaaattcagacgagaagatcatttgagttaaagaagctgtgggatcaaacccaatcaaaggtagggctattagtatcagatggaggaccaaacttatacaatatacggaatcttcacattcctgaagtctgcttaaaatgggagctaatggatgatgattatcggggaagactttgtaatcccctgaatccctttgtcagtcataaagagattgattctgtaaacaatgctgtggtaatgccagcccatggtccagccaaaagcatggaatatgatgccgttgcaactacacattcctggattcccaagaggaatcgttctattctcaacacaagccaaaggggaattcttgaggatgaacagatgtaccagaagtgctgcaatctattcgagaaatttttccctagcagttcatataggagaccggttggaatttctagcatggtggaggccatggtgtctagggcccggattgatgccagggtcgacttcgagtctggacggatcaagaaagaagagttctctgagatcatgaagatctgttccaccattgaagaactcagacggcaaaaataa

>H1N1_PB1_CY054857_A/California/VRDL32/2009

atggatgtcaatccgactctacttttcctaaaaattccagcgcaaaatgccataagcaccacattcccttatactggagatcctccatacagccatggaacaggaacaggatacaccatggacacagtaaacagaacacaccaatactcagaaaagggaaagtggacgacaaacacagagactggtgcaccccagctcaacccgattgatggaccactacctgaggataatgaaccaagtgggtatgcacaaacagactgtgttctagaggctatggctttccttgaagaatcccacccaggaatatttgagaattcatgccttgaaacaatggaagttgttcaacaaacaagggtagataaactaactcaaggtcgccagacttatgattggacattaaacagaaatcaaccggcagcaactgcattggccaacaccatagaagtctttagatcgaatggcctaacagctaatgagtcaggaaggctaatagatttcttaaaggatgtaatggaatcaatgaacaaagaggaaatagagataacaacccactttcaaagaaaaaggagagtaagagacaacatgaccaagaagatggtcacgcaaagaacaatagggaagaaaaaacaaagactgaataagagaggctatctaataagagcactgacattaaatacgatgaccaaagatgcagagagaggcaagttaaaaagaagggctatcgcaacacctgggatgcagattagaggtttcgtatactttgttgaaactttagctaggagcatttgcgaaaagcttgaacagtctgggctcccagtagggggcaatgaaaagaaggccaaactggcaaatgttgtgagaaagatgatgactaattcacaagacacagagatttctttcacaatcactggggacaacactaagtggaatgaaaatcaaaatcctcgaatgttcctggcgatgattacatatatcaccagaaatcaacccgagtggttcagaaacatcctgagcatggcacccataatgttctcaaacaaaatggcaagactagggaaagggtacatgttcgagagtaaaagaatgaagattcgaacacaaataccagcagaaatgctagcaagcattgacctgaagtacttcaatgaatcaacaaagaagaaaattgagaaaataaggcctcttctaatagatggcacagcatcactgagtcctgggatgatgatgggcatgttcaacatgctaagtacggtcttgggagtctcgatactgaatcttggacaaaagaaatacaccaagacaatatactggtgggatgggctccaatcatccgacgattttgctctcatagtgaatgcaccaaaccatgagggaatacaagcaggagtggacagattctacaggacctgcaagttagtgggaatcaacatgagcaaaaagaagtcctatataaataagacagggacatttgaattcacaagctttttttatcgctatggatttgtggctaattttagcatggagctacccagctttggagtgtctggagtaaatgaatcagctgacatgagtattggagtaacagtgataaagaacaacatgataaacaatgaccttggacctgcaacggcccagatggctcttcaattgttcatcaaagactacagatacacatataggtgccataggggagacacacaaattcagacgagaagatcatttgagttaaagaagctgtgggatcaaacccaatcaaaggtagggctattagtatcagatggaggaccaaacttatacaatatacggaatcttcacattcctgaagtctgcttaaaatgggagctaatggatgatgattatcggggaagactttgtaatcccctgaatccctttgtcagtcataaagagattgattctgtgaacaatgctgtggtaatgccagcccatggtccagccaaaagcatggaatatgatgccgttgcaactacacattcctggattcccaagaggaatcgttctattctcaacacaagccaaaggggaattcttgaggatgaacagatgtaccagaagtgctgcaatctattcgagaaatttttccctagcagttcatataggagaccggttggaatttctagcatggtggaggccatggtgtctagggcccggattgatgccagggtcgacttcgagtctggacggatcaagaaagaagagttctctgagatcatgaagatctgttccaccattgaagaactcagacggcaaaaataa

>H1N1_PB1_CY054937_A/California/VRDL45/2009

atggatgtcaatccgactctacttttcctaaaaattccagcgcaaaatgccataagcaccacattcccttatactggagatcctccatacagccatggaacaggaacaggatacaccatggacacagtaaacagaacacaccaatactcagaaaagggaaagtggacgacaaacacagagactggtgcaccccagctcaacccgattgatggaccactacctgaggataatgaaccaagtgggtatgcacaaacagactgtgttctagaggctatggctttccttgaagaatcccacccaggaatatttgagaattcatgccttgaaacaatggaagttgttcaacaaacaagggtagataaactaactcaaggtcgccagacttatgattggacattaaacagaaatcaaccggcagcaactgcattggccaacaccatagaagtctttagatcgaatggcctaacagctaatgagtcaggaaggctaatagatttcttaaaggatgtaatggaatcaatgaacaaagaggaaatagagataacaacccactttcaaagaaaaaggagagtaagagacaacatgaccaagaagatggtcacgcaaagaacaatagggaagaaaaaacaaagactgaataagagaggctatctaataagagcactgacattaaatacgatgaccaaagatgcagagagaggcaagttaaaaagaagggctatcgcaacacctgggatgcagattagaggtttcgtatactttgttgaaactttagctaggagcatttgcgaaaagcttgaacagtctgggctcccagtagggggcaatgaaaagaaggccaaactggcaaatgttgtgagaaagatgatgactaattcacaagacacagagatttctttcacaatcactggggacaacactaagtggaatgaaaatcaaaatcctcgaatgttcctggcgatgattacatatatcaccagaaatcaacccgagtggttcagaaacatcctgagcatggcacccataatgttctcaaacaaaatggcaagactagggaaagggtacatgttcgagagtaaaagaatgaagattcgaacacaaataccagcagaaatgctagcaagcattgacctgaagtacttcaatgaatcaacaaagaagaaaattgagaaaataaggcctcttctaatagatggcacagcatcactgagtcctgggatgatgatgggcatgttcaacatgctaagtacggtcttgggagtctcgatactgaatcttggacaaaagaaatacaccaagacaatatactggtgggatgggctccaatcatccgacgattttgctctcatagtgaatgcaccaaaccatgagggaatacaagcaggagtggacagattctacaggacctgcaagttagtgggaatcaacatgagcaaaaagaagtcctatataaataagacagggacatttgaattcacaagctttttttatcgctatggatttgtggccaattttagcatggagctacccagctttggagtgtctggagtaaatgaatcagctgacatgagtattggagtaacagtgataaagaacaacatgataaacaatgaccttggacctgcaacggcccagatggctcttcaattgttcatcaaagactacagatacacatataggtgccataggggagacacacaaattcagacgagaagatcatttgagttaaagaagctgtgggatcaaacccaatcaaaggtagggctattagtatcagatggaggaccaaacttatacaatatacggaatcttcacattcctgaagtctgcttaaaatgggagctaatggatgatgattatcggggaagactttgtaatcccctgaatccctttgccagtcataaagagattgattctgtaaacaatgctgtggtaatgccagcccatggtccagccaaaagcatggaatatgatgccgttgcaactacacattcctggattcccaagaggaatcgttctattctcaacacaagccaaaggggaattcttgaggatgaacagatgtaccagaagtgctgcaatctattcgagaaatttttccctagcagttcatataggagaccggttggaatttctagcatggtggaggccatggtgtctagggcccggattgatgccagggtcgacttcgagtctggacggatcaagaaagaagagttctctgagatcatgaagatctgttccaccattgaagaactcagacggcaaaaataa

>H1N1_PB1_CY043383_A/Niigata/07F102/2008

atggatgtcaatccgacattacttttcttaaaagtgccagcacaaaatgctataagcacaacttttccttatactggtgaccctccttacagccatgggacaggaacagggtacaccatggatacagtcaacaggacacaccagtactcagaaagaggaagatggacaaaaaataccgaaacgggagcaccgcaacttaacccaattgatggtcccttaccggaagacaatgaaccaagtggctatgcccaaacagattgtgtattagaagcaatggctttccttgaagaatcccatcccggtatctttgaaaactcttgtattgaaacaatggaggttgttcagcaaacaagggtggacaaactgacacaaggcagacagacctatgactggactctaaataggaaccagcctgctgccacagcattggcaaacactatagaagtattcagatcaaatggcctcatagcaaatgaatctgggaggctaatagacttccttaaagatgtaatggagtcgatggacagaggcgaagtagaggtcacaactcattttcaaagaaagaggagagtgagagacaatgtaactaaaaaaatggtgacccaaagaacaataggcaaaaagaaacataaattagacaaaagaagttacctaattagggcattaaccctgaacacaatgaccaaagatgctgagagggggaaactaaaacgcagagcaattgcaaccccaggaatgcaaataagggggtttgtatactttgttgagacactggcaagaagcatatgtgaaaagcttgaacaatcaggattgccagttggaggaaatgagaagaaagcaaagttagcaaatgttgtaaggaagatgatgaccaactcccaggacactgaaatttctttcaccataaccggagataacacaaaatggaacgaaaatcaaaaccctagaatgttcttggccatgatcacatatataaccaaaaatcagcctgaatggttcagaaatattctaagtattgctccaataatgttttcaaacaagatggcgagactaggtaaggggtacatgtttgaaagcaagagtatgaaactgagaactcaaatacctgcagagatgctagccaacatagatttgaaatatttcaatgattcaactaaaaagaaaattgaaaaaatccgaccattattaatagatggaactgcatcattgagtcctggaatgatgatgggcatgttcaatatgttgagcaccgtcttgggcgtctccattctgaatcttgggcaaaagagatacaccaagactacttactggtgggatggtcttcaatcgtctgatgattttgctttgattgtgaatgcacccaactatgcaggaattcaagctggagttgacaggttttatcgaacctgtaagctgctcggaattaatatgagcaaaaagaagtcttacataaacagaacaggtacctttgaattcacgagctttttctatcgttatgggtttgttgccaatttcagcatggagcttcctagttttggggtgtctggggtcaatgaatctgcagacatgagtattggagtcactgtcatcaaaaacaatatgataaacaatgaccttggcccagcaactgctcaaatggcccttcagttatttataaaagattacagatacacttatcgatgccaccgaggtgacacacaaatacaaacccggagatcatttgagataaaggaactatgggaccaaacccgctcaaaagctgggctgttggtctctgatggaggccccaatttatataacattagaaatctccatattcctgaagtttgcttgaaatgggagttgatggatgaggattaccaggggcgtttatgcaacccattaaacccgtttgtcagccataaagagattgaatcagtgaacaatgcagtgataatgccggcacatggtccagccaaaaatatggagtatgacgctgttgcaacaacacactcctgggtccccaaaagaaatcgatccattttgaacacgagccaaagggggatacttgaagatgagcaaatgtatcagaggtgctgcaatttatttgaaaaattcttcccaagtagctcatacagaagaccagttggaatatccagtatggtagaggctatggtctcaagagcccgaattgatgcacggattgatttcgaatctggaaggataaagaaagaggaatttgctgagatcatgaagatctgttccaccattgaagacctcagacggcaaaaatga

>H1N1_PB1_CY055293_A/Malaysia/5259/2009

atggatgtcaatccgactctacttttcctaaaaattccagcgcaaaatgccataagcaccacattcccttatactggagatcctccatacagccatggaacaggaacaggatacaccatggacacagtaaacagaacacaccaatactcagaaaagggaaagtggacgacaaacacagagactggtgcaccccagctcaacccgattgatggaccactacctgaggataatgaaccaagtgggtatgcacaaacagactgtgttctagaggctatggctttccttgaagaatcccacccaggaatatttgagaattcatgccttgaaacaatggaatttgttcaacaaacaagggtagataaactaactcaaggtcgccagacttatgattggacattaaacagaaatcaaccggcagcaactgcattggccaacaccatagaagtctttagatcgaatggcctaacagctaatgagtcaggaaggctaatagatttcttaaaggatgtaatggaatcaatgaacaaagaggaaatagagataacaacccactttcaaagaaaaaggagagtaagagacaacatgaccaagaagatggtcacgcaaagaacaatagggaagaaaaaacaaagactgaataagagaggctatctaataagagcactgacattaaatacgatgaccaaagatgcagagagaggcaagttaaaaagaagggctatcgcaacacctgggatgcagattagaggtttcgtatactttgttgaaactttagctaggagcatttgcgaaaagcttgaacagtctgggctcccagtagggggcaatgaaaagaaggccaaactggcaaatgttgtgagaaagatgatgactaattcacaagacacagagatttctttcacaatcactggggacaacactaagtggaatgaaaatcaaaatcctcgaatgttcctggcgatgattacatatatcaccagaaatcaacccgagtggttcagaaacatcctgagcatggcacccataatgttctcaaacaaaatggcaagactagggaaagggtacatgttcgagagtaaaagaatgaagattcgaacacaaataccagcagaaatgctagcaagcattgacctgaagtacttcaatgaatcaacaaagaagaaaattgagaaaataaggcctcttctaatagatggcacagcttcactgagtcctgggatgatgatgggcatgttcaacatgctaagtacggtcttgggagtctcgatactgaatcttggacaaaagaaatacaccaagacaatatactggtgggatgggctccaatcatccgacgattttgctctcatagtgaatgcaccaaaccatgagggaatacaagcaggagtggacagattctacaggacctgcaagttagtgggaatcaacatgagcaaaaagaagtcctatataaataagacagggacatttgaattcacaagctttttttatcgctatggatttgtggctaattttagcatggagctacccagctttggagtgtctggagtaaatgaatcagctgacatgagtattggagtaacagtgataaagaacaacatgataaacaatgaccttggacctgcaacggcccagatggctcttcaattgttcatcaaagactacagatacacatataggtgccataggggagacacacaaattcagacgagaagatcatttgagttaaagaagctgtgggatcaaacccaatcaaaggtagggctattagtatcagatggaggaccaaacttatacaatatacggaatcttcacattcctgaagtctgcttaaaatgggagctaatggatgatgattatcggggaagactttgtaatcccctgaatccctttgtcagtcataaagagattgattctgtaaacaatgctgtggtaatgccagcccatggtccagccaaaagcatggaatatgatgccgttgcaactacacattcctggattcccaagaggaatcgttctattctcaacacaagccaaaggggaattcttgaggatgaacagatgtaccagaagtgctgcaatctattcgagaaatttttccctagcagttcatataggagaccggttggaatttctagcatggtggaggccatggtgtctagggcccggattgatgccagggtcgacttcgaatctggacggatcaagaaagaagagttctctgagatcatgaagatctgttccaccattgaagaactcagacggcaaaaataa

>H1N1_PB1_CY055301_A/Singapore/GN285/2009

atggatgtcaatccgactctacttttcctaaaaattccagcgcaaaatgccataagcaccacattcccttatactggagatcctccatacagccatggaacaggaacaggatacaccatggacacagtaaacagaacacaccaatactcagaaaagggaaagtggacgacaaacacagagactggtgcaccccagctcaacccgattgatggaccactacctgaggataatgaaccaagtgggtatgcacaaacagactgtgttctagaggctatggctttccttgaagaatcccacccaggaatatttgagaattcatgccttgaaacaatggaagttgttcaacaaacaagggtagataaactaactcaaggtcgccagacttatgattggacattaaacagaaatcaaccggcagcaactgcattggccaacaccatagaagtctttagatcgaatggcctaacagctaatgagtcaggaaggctaatagatttcttaaaggatgtaatggaatcaatgaacaaagaggaaatagagataacaacccactttcaaagaaaaaggagagtaagagacaacatgaccaagaagatggtcacgcaaagaacaatagggaagaaaaaacaaagactgaataagagaggctatctaataagagcactgacattaaatacgatgaccaaagatgcagagagaggcaagttaaaaagaagggctatcgcaacacctgggatgcagattagaggtttcgtatactttgttgaaactttagctaggagcatttgcgaaaagcttgaacagtctgggctcccagtagggggcaatgaaaagaaggccaaactggcaaatgttgtgagaaagatgatgactaattcacaagacacagagatttctttcacaatcactggggacaacactaagtggaatgaaaatcaaaatcctcgaatgttcctggcgatgattacatatatcaccagaaatcaacccgagtggttcagaaacatcctgagcatggcacccataatgttctcaaacaaaatggcaagactagggaaagggtacatgttcgagagtaaaagaatgaagattcgaacacaaataccagcagaaatgctagcaagcattgacctgaagtacttcaatgaatcaacaaagaagaaaattgagaaaataaggcctcttctaatagatggcacagcatcactgagtcctgggatgatgatgggcatgttcaacatgctaagtacggtcttgggagtctcgatactgaatcttggacaaaagaaatacaccaagacagtatactggtgggatgggctccaatcatccgacgattttgctctcatagtgaatgcaccaaaccatgagggaatacaagcaggagtggacagattctacaggacctgcaagttagtgggaatcaacatgagcaaaaagaagtcctatataaataagacagggacatttgaattcacaagctttttttatcgctatggatttgtggctaattttagcatggagctacccagctttggagtgtctggagtaaatgaatcagctgacatgagtattggagtaacagtgataaagaacaacatgataaacaatgaccttggacctgcaacggcccagatggctcttcaattgttcatcaaagactacagatacacatataggtgccataggggagacacacaaattcagacgagaagatcatttgagttaaagaagctgtgggatcaaacccaatcaaaggtagggctattagtatcagatggaggaccaaacttatacaatatacggaatcttcacattcctgaagtctgcttaaaatgggagctaatggatgatgattatcggggaagactttgtaatcccctgaatccctttgtcagtcataaagagattgattctgtaaacaatgctgtggtaatgccagcccatggtccagccaaaagcatggaatatgatgccgttgcaactacacattcctggattcccaagaggaatcgttctattctcaacacaagccaaaggggaattcttgaggatgaacagatgtaccagaagtgctgcaatctattcgagaaatttttccctagcagttcatataggagaccggttggaatttctagcatggtggaggccatggtgtctagggcccggattgatgccagggtcgacttcgagtctggacggatcaagaaagaagagttctctgagatcatgaagatctgttccaccattgaagaactcagacggcaaaaataa

>H1N1_PB1_CY055477_A/California/VRDL18/2009

atggatgtcaatccgactctacttttcctaaaaattccagcgcaaaatgccataagcaccacattcccttatactggagatcctccatacagccatggaacaggaacaggatacaccatggacacagtaaacagaacacaccaatactcagaaaagggaaagtggacgacaaacacagagactggtgcaccccagctcaacccgattgatggaccactacctgaggataatgaaccaagtgggtatgcacaaacagactgtgttctagaggctatggctttccttgaagaatcccacccaggaatatttgagaattcatgccttgaaacaatggaagttgttcaacaaacaagggtagataaactaactcaaggtcgccagacttatgattggacattaaacagaaatcaaccggcagcaactgcattggccaacaccatagaagtctttagatcgaatggcctaacagctaatgagtcaggaaggctaatagatttcttaaaggatgtaatggaatcaatgaacaaagaggaaatagagataacaacccactttcaaagaaaaaggagagtaagagacaacatgaccaagaagatggtcacgcaaagaacaatagggaagaaaaaacaaagactgaataagagaggctatctaataagagcactgacattaaatacgatgaccaaagatgcagagagaggcaagttaaaaagaagggctatcgcaacacctgggatgcagattagaggtttcgtatactttgttgaaactttagctaggagcatttgcgaaaagcttgaacagtctgggctcccagtagggggcaatgaaaagaaggccaaactggcaaatgttgtgagaaagatgatgactaattcacaagacacagagatttctttcacaatcactggggacaacactaagtggaatgaaaatcaaaatcctcgaatgttcctggcgatgattacatatatcaccagaaatcaacccgagtggttcagaaacatcctgagcatggcacccataatgttctcaaacaaaatggcaagactagggaaagggtacatgttcgagagtaaaagaatgaagattcgaacacaaataccagcagaaatgctagcaagcattgacctgaagtacttcaatgaatcaacaaagaagaaaattgagaaaataaggcctcttctaatagatggcacagcatcactgagtcctgggatgatgatgggcatgttcaacatgctaagtacggtcttgggagtctcgatactgaatcttggacaaaagaaatacaccaagacaatatactggtgggatgggctccaatcatccgacgattttgctctcatagtgaatgcaccaaaccatgagggaatacaagcaggagtggacagattctacaggacctgcaagttagtgggaatcaacatgagcaaaaagaagtcctatataaataagacagggacatttgaattcacaagctttttttatcgctatggatttgtggctaattttagcatggagctacccagctttggagtgtctggagtaaatgaatcagctgacatgagtattggagtaacagtgataaagaacaacatgataaacaatgaccttggacctgcaacggcccagatggctcttcaactgttcatcaaagactacagatacacatataggtgccataggggagacacacaaattcagacgagaagatcatttgagttaaagaagctgtgggatcaaacccaatcaaaggtagggctattagtatcagatggaggaccaaacttatacaatatacggaatcttcacattcctgaagtctgcttaaaatgggagctaatggatgatgattatcggggaagactttgtaatcccctgaatccctttgtcagtcataaagagattgattctgtaaacaatgctgtggtaatgccagcccatggtccagccaaaagcatggaatatgatgccgttgcaactacacattcctggattcccaagaggaatcgttctattctcaacacaagccaaaggggaattcttgaggatgaacagatgtaccagaagtgctgcaatctattcgagaaatttttccctagcagttcatataggagaccggttggaatttctagcatggtggaggccatggtgtctagggcccggattgatgccagggtcgacttcgagtctggacggatcaagaaagaagagttctctgagatcatgaagatctgttccaccattgaagaactcagacggcaaaaataa

>H1N1_PB1_CY055674_A/Australia/29/2009

atggatgtcaatccgactctacttttcctaaaaattccagcgcaaaaagccataagcaccacattcccttatactggagatcctccatacagccatggaacaggaacaggatacaccatggacacagtaaacagaacacaccaatactcagaaaagggaaagtggacgacaaacacagagactggtgcaccccagctcaacccgattgatggaccactacctgaggataatgaaccaagtgggtatgcacaaacagactgtgttctagaggctatggctttccttgaagaatcccacccaggaatatttgagaattcatgccttgaaacaatggaagttgttcaacaaacaagggtagataaactaactcaaggtcgccagacttatgattggacattaaacagaaatcaaccggcagcaactgcattggccaacaccatagaagtttttagatcgaatggcctaacagctaatgagtcaggaaggctaatagatttcttaaaggatgtaatggaatcaatgaacaaagaggaaatagagataacaacccactttcaaagaaaaaggagagtaagagacaacatgaccaagaagatggtcacgcaaagaacaatagggaagaaaaaacaaagactgaataagagaggctatctaataagagcactgacattaaatacgatgaccaaagatgcagagagaggcaagttaaaaagaagggctatcgcaacacctgggatgcagattagaggtttcgtatactttgttgaaactttagctaggagcatttgcgaaaagcttgaacagtctgggctcccagtagggggcaatgaaaagaaggccaaactggcaaatgttgtgagaaagatgatgactaattcacaagacacagagatttctttcacaatcactggggacaacactaagtggaatgaaaatcaaaatcctcgaatgttcctggcgatgattacatatatcaccagaaatcaacccgagtggttcagaaacatcctgagcatggcacccataatgttctcaaacaaaatggcaagactagggaaagggtacatgttcgagagtaaaagaatgaagattcgaacacaaataccagcagaaatgctagcaagcattgacctgaagtacttcaatgaatcaacaaagaagaaaattgagaaaataaggcctcttctaatagatggcacagcatcactgagtcctgggatgatgatgggcatgttcaacatgctaagtacggtcttgggagtctcgatactgaatcttggacaaaagaaatacaccaagacaatatactggtgggatgggctccaatcatccgacgattttgctctcatagtgaatgcaccaaaccatgagggaatacaagcaggagtggacagattctacaggacctgcaagttagtgggaatcaacatgagcaaaaagaagtcctatataaataagacagggacatttgaattcacaagctttttttatcgctatggatttgtggctaattttagcatggagctacccagctttggagtgtctggagtaaatgaatcagctgacatgagtattggagtaacagtgataaagaacaacatgataaacaatgaccttggacctgcaacggcccagatggctcttcaattgttcatcaaagactacagatacacatataggtgccataggggagacacacaaattcagacgagaagatcatttgagttaaagaagctgtgggatcaaacccaatcaaaggtagggctattagtatcagatggaggaccaaacttatacaatatacggaatcttcacattcctgaagtctgcttaaaatgggagctaatggatgatgattatcggggaagactttgtaatcccctgaatccctttgtcagccataaagagattgattctgtaaacaatgctgtggtaatgccagcccatggtccagccaaaagcatggaatatgatgccgttgcaactacacattcctggattcccaagaggaatcgttctattctcaacacaagccaaaggggaattcttgaggatgaacagatgtaccagaagtgctgcaatctattcgagaaatttttccctagcagttcatataggagaccggttggaatttctagcatggtggaggccatggtgtctagggcccggattgatgccagggtcgacttcgagtctggacggatcaagaaagaagagttctctgagatcatgaagatctgttccaccattgaagaactcagacggcaaaaataa

>H1N1_PB1_CY055890_A/Australia/69/2009

atggatgtcaatccgactctacttttcctaaaaattccagcgcaaaatgccataagcaccacattcccttatactggagatcctccatacagccatggaacaggaacaggatacaccatggacacagtaaacagaacacaccaatactcagaaaagggaaagtggacgacaaacacagagactggtgcaccccagctcaacccgattgacggaccactacctgaggataatgaaccaagtgggtatgcacaaacagactgtgttctagaggctatggctttccttgaagaatcccacccaggaatatttgagaattcatgccttgaaacaatggaagttgttcaacaaacaagggtagataaactaactcaaggtcgccagacttatgattggacattaaacagaaatcaaccggcagcaactgcattggccaacaccatagaagtctttagatcgaatggcctaacagctaatgagtcaggaaggctaatagatttcttaaaggatgtaatggaatcaatgaacaaagaggaaatagagataacaacccactttcaaagaaaaaggagagtaagagacaacatgaccaagaagatggtcacgcaaagaacaatagggaagaaaaaacaaagactgaataagagaggctatctaataagagcactgacattaaatacgatgaccaaagatgcagagagaggtaagttaaaaagaagggctatcgcaacacctgggatgcagattagaggtttcgtatactttgttgaaactttagctaggagcatttgcgaaaagcttgaacagtctgggctcccagtagggggcaatgaaaagaaggccaaactggcaaatgttgtgagaaagatgatgactaattcacaagacacagagatttctttcacaatcactggggacaacactaagtggaatgaaaatcaaaatcctcgaatgttcctggcgatgattacatatatcaccagaaatcaacccgagtggttcagaaacatcctgagcatggcacccataatgttctcaaacaaaatggcaagactagggaaagggtacatgttcgagagtaaaagaatgaagattcgaacacaaataccagcagaaatgctagcaagcattgacctgaagtacttcaatgaatcaacaaagaaaaaaattgagaaaataaggcctcttctaatagatggcacagcgtcactgagtcctgggatgatgatgggcatgttcaacatgctaagtacggtcttgggagtctcgatactgaatcttggacaaaagaaatacaccaagacaatatactggtgggatgggctccaatcatccgacgattttgctctcatagtgaatgcaccaaaccatgagggaatacaagcaggagtggacagattctacaggacctgcaagttagtgggaatcaacatgagcaaaaagaagtcctatataaataagacagggacatttgaattcacaagctttttttatcgctatggatttgtggctaattttagcatggagctacccagctttggagtgtctggagtaaatgaatcagctgacatgagtattggagtaacagtgataaagaacaacatgataaacaatgaccttggacctgcaacggcccagatggctcttcaattgttcatcaaagactacagatacacatataggtgccataggggagacacacaaattcagacgagaagatcatttgagttaaagaagctgtgggatcaaacccaatcaaaggtagggctattagtatcagatggaggaccaaacttatacaatatacggaatcttcacattcctgaagtctgcttaaaatgggagctaatggatgatgattatcggggaagactttgtaatcccctgaatccctttgtcagtcataaagagattgattctgtaaacaatgctgtggtaatgccagcccatggtccagccaaaagcatggaatatgatgccgttgcaactacacattcctggattcccaagaggaatcgttctattctcaacacaagccaaaggggaattcttgaggatgaacagatgtaccagaagtgctgcaatctattcgagaaatttttccctagcagttcatataggagaccggttggaatttctagcatggtggaggccatggtgtctagggcccggattgatgccagggtcgacttcgagtctggacggatcaagaaagaagagttctctgagatcatgaagatctgttccaccattgaagaactcagacggcaaaaataa

>H1N1_PB1_CY056529_A/New York/5130/2009

atggatgtcaatccgactctacttttcctaaaaattccagcgcaaaatgccataagcaccacattcccttatactggagatcctccatacagccatggaacaggaacaggatacaccatggacacagtaaacagaacacaccaatactcagaaaagggaaagtggacgacaaacacagagactggtgcaccccagctcaacccgattgatggaccactacctgaggataatgaaccaagtgggtatgcacaaacagactgtgttctagaggctatggctttccttgaagaatcccacccaggaatatttgagaattcatgccttgaaacaatggaagttgttcaacaaacaagggtagataaactaactcaaggtcgccagacttatgattggacattaaacagaaatcaaccggcagcaactgcattggccaacaccatagaagtctttagatcgaatggcctaacagctaatgagtcaggaaggctaatagatttcttaaaggatgtaatggaatcaatgaacaaagaggaaatagagataacaacccactttcaaagaaaaaggagagtaagagacaacatgaccaagaagatggtcacgcaaagaacaatagggaagaaaaaacaaagactgaataagagaggctatctaataagagcactgacattaaatacgatgaccaaagatgcagagagaggcaagttaaaaagaagggctatcgcaacacctgggatgcagattagaggtttcgtatactttgttgaaactttagctaggagcatttgcgaaaagcttgaacagtctgggctcccagtagggggcaatgaaaagaaggccaaactggcaaatgttgtgagaaagatgatgactaattcacaagacacagagatttctttcacaatcactggggacaacactaagtggaatgaaaatcaaaatcctcgaatgttcctggcgatgattacatatatcaccagaaatcaacccgagtggttcagaaacatcctgagcatggcacccataatgttctcaaacaaaatggcaagactagggaaagggtacatgttcgagagtaaaagaatgaagattcgaacacaaataccagcagaaatgctagcaagcattgacctgaagtacttcaatgaatcaacaaagaagaaaattgagaaaataaggcctcttctaatagatggcacagcatcactgagtcctgggatgatgatgggcatgttcaacatgctaagtacggtcttgggagtctcgatactgaatcttggacaaaagaaatacaccaagacaatatactggtgggatgggctccaatcatccgacgattttgctctcatagtgaatgcaccaaaccatgagggaatacaagcaggagtggacagattctacaggacctgcaagttagtgggaatcaacatgagcaaaaagaagtcctatataaataagacagggacatttgaattcacaagctttttttatcgctatgggtttgtggctaattttagcatggagctacctagctttggagtgtctggagtaaatgaatcagctgacatgagtattggagtaacagtgataaagaacaacatgataaacaatgaccttggacctgcaacggcccagatggctcttcaattgttcatcaaagactacagatacacatataggtgccataagggagacacacaaattcagacgagaagatcatttgagttaaagaagctgtgggatcaaacccaatcaaaggtagggctattagtatcagatggaggaccaaacttatacaatatacggaatcttcacattcctgaagtctgcttaaaatgggagctaatggatgatgattatcggggaagactttgtaatcccctgaatccctttgtcagtcataaagagattgattctgtaaacaatgctgtggtaatgccagcccatggtccagccaaaagtatggaatatgatgccgttgcaactacacattcctggattcccaagaggaatcgttctattctcaacacaagccaaaggggaattcttgaggatgaacagatgtaccagaagtgctgcaatctattcgagaaatttttccctagcagttcatataggagaccggttggaatttctagcatggtggaggccatggtgtctagggcccggattgatgccagggtcgacttcgagtctggacggatcaagaaagaagagttctctgagatcatgaagatctgttccaccattgaagaactcagacggcaaaaataa

>H1N1_PB1_CY056561_A/New York/5199/2009

atggatgtcaatccgactctacttttcctaaaaattccagcgcaaaatgccataagcaccacattcccttatactggagatcctccatacagccatggaacaggaacaggatacaccatggacacagtaaacagaacacaccaatactcagaaaagggaaagtggacgacaaacacagagactggtgcaccccagctcaacccgattgatggaccactacctgaggataatgaaccaagtgggtatgcacaaacagactgtgttctagaggctatggctttccttgaagaatcccacccaggaatatttgagaattcatgccttgaaacaatggaagttgttcaacaaacaagggtagataaactaactcaaggtcgccagacttatgattggacattaaacagaaatcaaccggcagcaactgcattggccaacaccatagaagtctttagatcgaatggcctaacagctaatgagtcaggaaggctaatagatttcttaaaggatgtaatggaatcaatgaacaaagaggaaatagagataacaacccactttcaaagaaaaaggagagtaagagacaacatgaccaagaagatggtcacgcaaagaacaatagggaagaaaaaacaaagactgaataagagaggctatctaataagagcactgacattaaatacgatgaccaaagatgcagagagaggcaagttaaaaagaagggctatcgcaacacctgggatgcagattagaggtttcgtatactttgttgaaactttagctaggagcatttgcgaaaagcttgaacagtctgggctcccagtagggggcaatgaaaagaaggccaaactggcaaatgttgtgagaaagatgatgactaattcacaagacacagagatttctttcacaatcactggggacaacactaagtggaatgaaaatcaaaatcctcgaatgttcctggcgatgattacatatattaccagaaatcaacccgagtggttcagaaacatcctgagcatggcacccataatgttctcaaacaaaatggcaagactagggaaagggtacatgttcgagagtaaaagaatgaagattcgaacacaaataccagcagaaatgctagcaagcattgacctgaagtacttcaatgaatcaacaaagaagaaaattgagaaaataaggcctcttctaatagatggcacagcatcactgagtcctgggatgatgatgggcatgttcaacatgctaagtacggtcttgggagtctcgatactgaatcttggacaaaagaaatacaccaagacaatatactggtgggatgggctccaatcatccgacgattttgctctcatagtgaatgcaccaaaccatgagggaatacaagcaggagtggacagattctacaggacctgcaagttagtgggaatcaacatgagcaaaaagaagtcctatataaataagacagggacatttgaattcacaagctttttttatcgctatgggtttgtggctaattttagcatggagctacccagctttggagtgtctggagtaaatgaatcagctgacatgagtattggagtaacagtgataaagaacaacatgataaacaatgaccttggacctgcaacggcccagatggctcttcaattgttcatcaaagactacagatacacatataggtgccataagggagacacacaaattcagacgagaagatcatttgagttaaagaagctgtgggatcaaacccaatcaaaggtagggctattagtatcagatggaggaccaaacttatacaatatacggaatcttcacattcctgaagtctgcttaaaatgggagctaatggatgatgattatcggggaagactttgtaatcccctgaatccctttgtcagtcataaagagattgattctgtaaacaatgctgtggtaatgccagcccatggtccagccaaaagcatggaatatgatgccgttgcaactacacattcctggattcccaagaggaatcgttctattctcaacacaagccaaaggggaattcttgaggatgaacagatgtaccagaagtgctgcaatctattcgagaaatttttccctagcagttcatataggagaccggttggaatttctagcatggtggaggccatggtgtctagggcccggattgatgccagggtcgacttcgagtctggacggatcaagaaagaagagttctctgagatcatgaagatctgttccaccattgaagaactcagacggcaaaaataa

>H1N1_PB1_CY057404_A/Wisconsin/629-D01469/2009

atggatgtcaatccgactctacttttcctaaaaattccagcgcaaaatgccataagcaccacattcccttatactggagatcctccatacagccatggaacaggaacaggatacaccatggacacagtaaacagaacacaccaatactcagaaaagggaaagtggacgacaaacacagagactggtgcaccccagctcaacccgattgatggaccactacctgaggataatgaaccaagtgggtatgcacaaacagactgtgttctagaggctatggctttccttgaagaatcccacccaggaatatttgagaattcatgccttgaaacaatggaagttgttcaacaaacaagggtagataaactaactcaaggtcgccagacttatgattggacattaaacagaaatcaaccggcagcaactgcattggccaacaccatagaagtctttagatcgaatggcctaacagccaatgagtcaggaaggctaatagatttcttaaaggatgtaatggaatcaatgaacaaagaggaaatagagataacaacccactttcaaagaaaaaggagagtaagagacaacatgaccaagaagatggtcacgcaaagaacaatagggaagaaaaaacaaagactgaataagagaggctatctaataagagcactgacattaaatacgatgaccaaagatgcagagagaggcaagttaaaaagaagggctatcgcaacacctgggatgcagattagaggtttcgtatactttgttgaaactttagctaggagcatttgcgaaaagcttgaacagtctgggctcccagtagggggcaatgaaaagaaggccaaactggcaaatgttgtgagaaagatgatgactaattcacaagacacagagatttctttcacaatcactggggacaacactaagtggaatgaaaatcaaaatcctcgaatgttcctggcgatgattacatatatcaccagaaatcaacccgagtggttcagaaacatcctgagcatggcacccataatgttctcaaacaaaatggcaagactagggaaagggtacatgttcgagagtaaaagaatgaagattcgaacacaaataccagcagaaatgctagcaagcattgacctgaagtacttcaatgaatcaacaaagaagaaaattgagaaaataaggcctcttctaatagatggcacagcatcactgagtcctgggatgatgatgggcatgttcaacatgctaagtacggtattgggagtctcgatactgaatcttggacaaaagaaatacaccaagacaatatactggtgggatgggctccaatcatccgacgattttgctctcatagtgaatgcaccaaaccatgagggaatacaagcaggagtggacagattctacaggacctgcaagttagtgggaatcaacatgagcaaaaagaagtcctatataaataagacagggacatttgaattcacaagctttttttatcgctatggatttgtggctaattttagcatggagctacccagctttggagtgtctggagtaaatgaatcagctgacatgagtattggagtaacagtgataaagaacaacatgataaacaatgaccttggacctgcaacggcccagatggctcttcaattgttcatcaaagactacagatacacatataggtgccataggggagacacacaaattcagacgagaagatcatttgagttaaagaagctgtgggatcaaacccaatcaaaggtagggctattagtatcagatggaggaccaaacttatacaatatacggaatcttcacattcctgaagtctgcttaaaatgggagctaatggatgatgattatcggggaagactttgtaatcccctgaatccctttgtcagtcataaagagattgattctgtaaacaatgctgtggtaatgccagcccatggtccagccaaaagcatggaatatgatgccgttgcaactacacattcctggattcccaagaggaatcgttctattctcaacacaagccaaaggggaattcttgaggatgaacagatgtaccagaagtgctgcaatctattcgagaaatttttccctagcagttcatataggagaccggttggaatttctagcatggtggaggccatggtgtctagggcccggattgatgccagggtcgacttcgagtctggacggatcaagaaagaagagttctctgagatcatgaagatctgttccaccattgaagaactcagacggcaaaaataa

>H1N1_PB1_CY057580_A/Wisconsin/629-S1252/2009

atggatgtcaatccgactctactcttcctaaaaattccagcgcaaaatgccataagcaccacattcccttatactggagatcctccatacagccatggaacaggaacaggatacaccatggacacagtaaacagaacacaccaatactcagaaaagggaaagtggacgacaaacacagagactggtgcaccccagctcaacccgattgatggaccactacctgaggataatgaaccaagtgggtatgcacaaacagactgtgttctagaggctatggctttccttgaagaatcccacccaggaatatttgagaattcatgccttgaaacaatggaagttgttcaacaaacaagggtagataaactaactcaaggtcgccagacttatgattggacattaaacagaaatcaaccggcagcaactgcattggccaacaccatagaagtctttagatcgaatggcctaacagctaatgagtcaggaaggctaatagatttcttaaaggatgtaatggaatcaatgaacaaagaggaaatagagataacaacccactttcaaagaaaaaggagagtaagagacaacatgaccaagaagatggtcacgcaaagaacaatagggaagaaaaaacaaagactgaataagagaggctatctaataagagcactgacattaaatacgatgaccaaagatgcagagagaggcaagttaaaaagaagggctatcgcaacacctgggatgcagattagaggtttcgtatactttgttgaaactttagctaggagcatttgcgaaaagcttgaacagtctgggctcccagtagggggcaatgaaaagaaggccaaactggcaaatgttgtgagaaagatgatgactaattcacaagacacagagatttctttcacaatcactggggacaacactaagtggaatgaaaatcaaaatcctcgaatgttcctggcgatgattacatatatcaccagaaatcaacccgagtggttcagaaacatcctgagcatggcacccataatgttctcaaacaaaatggcaagactagggaaagggtacatgttcgagagtaaaagaatgaagattcgaacacaaataccagcagaaatgctagcaagcattgacctgaagtacttcaatgaatcaacaaagaagaaaattgagaaaataaggcctcttctaatagatggcacagcatcactgagtcctgggatgatgatgggcatgttcaacatgctaagtacggtcttgggagtctcgatactgaatcttggacaaaagaaatacaccaagacaatatactggtgggatgggctccaatcatccgacgattttgctctcatagtgaatgcaccaaaccatgagggaatacaagcaggagtggacagattctacaggacctgcaagttagtgggaatcaacatgagcaaaaagaagtcctatataaataagacagggacatttgaattcacaagctttttttatcgctatggatttgtggctaattttagcatggagctacccagctttggagtgtctggagtaaatgaatcagctgacatgagtattggagtaacagtgataaagaacaacatgataaacaatgaccttggacctgcaacggcccagatggctcttcaattgttcatcaaagactacagatacacatataggtgccataggggagacacacaaattcagacgagaagatcatttgagttaaagaagctgtgggatcaaacccaatcaaaggtagggctattagtatcagatggaggaccaaacttatacaatatacggaatcttcacattcctgaagtctgcttaaaatgggagctaatggatgatgattatcggggaagactttgtaatcccctgaatccctttgtcagtcataaagagattgattctgtaaacaatgctgtggtaatgccagcccatggtccagccaaaagcatggaatatgatgccgttgcaactacacattcctggattcccaagaggaatcgttctattctcaacacaagccaaaggggaattcttgaggatgaacagatgtaccagaagtgctgcaatctattcgagaaatttttccctagcagttcatataggagaccggttggaatttctagcatggtggaggccatggtgtctagggcccggattgatgccagggtcgacttcgagtctggacggatcaagaaagaagagttctctgagatcatgaagatctgttccaccattgaagaactcagacggcaaaaataa

>H1N1_PB1_CY057996_A/Wisconsin/629-D00935/2009

atggatgtcaatccgactctacttttcctaaaaattccagcgcaaaatgccataagcaccacattcccttatactggagatcctccatacagccatggaacaggaacaggatacaccatggacacagtaaacagaacacaccaatactcagaaaagggaaagtggacgacaaacacagagactggtgcaccccagctcaacccgattgatggaccactacctgaggataatgaaccaagtgggtatgcacaaacagactgtgttctagaggctatggctttccttgaagaatcccacccaggaatatttgagaattcatgccttgaaacaatggaagttgttcaacaaacaagggtagataaactaactcaaggtcgccagacttatgattggacattaaacagaaatcaaccggcagcaactgcattggccaacaccatagaagtctttagatcgaatggcctaacagctaatgagtcaggaaggctaatagatttcttaaaggatgtaatggaatcaatgaacaaagaggaaatagagataacaacccactttcaaagaaaaaggagagtaagagacaacatgaccaagaagatggtcacgcaaagaacaatagggaagaaaaaacaaagactgaataagagaggctatctaataagagcactgacattaaatacgatgaccaaagatgcagagagaggcaagttaaaaagaagggctatcgcaacacctgggatgcagattagaggtttcgtatactttgttgaaactttagctaggagcatttgcgaaaagcttgaacagtctgggctcccagtagggggcaatgaaaagaaggccaaactggcaaatgttgtgagaaagatgatgactaattcacaagacacagagatttctttcacaatcactggggacaacactaagtggaatgaaaatcaaaatcctcgaatgttcctggcgatgattacatatatcaccagaaatcaacccgagtggttcagaaacattctgagcatggcacccataatgttctcaaacaaaatggcaagactagggaaagggtacatgttcgagagtaaaagaatgaagattcgaacacaaataccagcagaaatgctagcaagcattgacctgaagtacttcaatgaatcaacaaagaagaaaattgagaaaataaggcctcttctaatagatggcacagcatcactgagtcctgggatgatgatgggcatgttcaacatgctaagtacggtcttgggagtctcgatactgaatcttggacaaaagaaatacaccaagacaatatactggtgggatgggctccaatcatccgacgattttgctctcatagtgaatgcaccaaaccatgagggaatacaagcaggagtggacagattctacaggacctgcaagttagtgggaatcaacatgagcaaaaagaagtcctatataaataagacagggacatttgaattcacaagctttttttatcgctatgggtttgtggctaattttagcatggagctacccagctttggagtgtctggagtaaatgaatcagctgacatgagtattggagtaacagtgataaagaacaacatgataaacaatgaccttggacctgcaacggcccagatggctcttcaattgttcatcaaagactacagatacacatataggtgccataagggagacacacaaattcagacgagaagatcatttgagttaaagaagctgtgggatcaaacccaatcaaaggtagggctattagtatcagatggaggaccaaacttatacaatatacggaatcttcacattcctgaagtctgcttaaaatgggagctaatggatgatgattatcggggaagactttgtaatcccctgaatccctttgtcagtcataaagagattgattctgtaaacaatgctgtggtaatgccagcccatggtccagccaaaagcatggaatatgatgccgttgcaactacacattcctggattcccaagaggaatcgttctattctcaacacaagccaaaggggaattcttgaggatgaacagatgtaccagaagtgctgcaatctattcgagaaatttttccctagcagttcatataggagaccggttggaatttctagcatggtggaggccatggtgtctagggcccggattgatgccagggtcgacttcgagtctggacggatcaagaaagaagagttctctgagatcatgaagatctgttccaccattgaagaactcagacggcaaaaataa

>H1N1_PB1_CY058170_A/Managua/4016.02/2009

atggatgtcaatccgactctacttttcctaaaaattccagcgcaaaatgccataagcaccacattcccttatactggagatcctccatacagccatggaacaggaacaggatacaccatggacacagtaaacagaacacaccaatactcagaaaagggaaagtggacgacaaacacagagactggtgcaccccagctcaacccggttgatggaccactacctgaggataatgaaccaagtgggtatgcacaaacagactgtgttctagaggctatggctttccttgaagaatcccacccgggaatatttgagaattcatgccttgaaacaatggaagttgttcaacaaacaagggtagataaactaactcaaggtcgccagacttatgattggacattaaacagaaatcaaccggcagcaactgcattggccaacaccatagaagtctttagatcgaatggcctaacagctaatgagtcaggaaggctaatagatttcttaaaggatgtaatggaatcaatgaacaaagaggaaatagagataacaacccactttcaaagaaaaaggagagtaagagacaacatgaccaagaagatggtcacgcaaagaacaatagggaagaaaaaacaaagactgaataagagaggctatctaataagagcactgacattaaatacgatgaccaaagatgcagagagaggcaagttaaaaagaagggctatcgcaacacctgggatgcagattagaggtttcgtatactttgttgaaactttagctaggagcatttgcgaaaagcttgaacagtctgggctcccagtagggggcaatgaaaagaaggccaaactggcaaatgttgtgagaaagatgatgactaattcacaagacacagagatttctttcacaatcactggggacaacactaagtggaatgaaaatcaaaatcctcgaatgttcctggcgatgattacatatatcaccagaaatcaacccgagtggttcagaaacatcctgagcatggcacccataatgttctcaaacaaaatggcaagactagggagagggtacatgttcgagagtaaaagaatgaagattcgaacacaaataccagcagaaatgctagcaagcattgacctgaagtacttcaatgaatcaacaaagaagaaaattgagaaaataaggcctcttctaatagatggcacagcatcactgagtcctgggatgatgatgggcatgttcaacatgctaagtacggtcttgggagtctcgatactgaatctcggacaaaagaaatacaccaagacaatatactggtgggatgggctccaatcatccgacgattttgctctcatagtgaatgcaccaaaccatgagggaatacaagcaggagtggacagattctacaggacctgcaagttagtgggaatcaacatgagcaaaaagaagtcctatataaataagacagggacatttgaattcacaagctttttttatcgctatggatttgtggctaattttagcatggagctacccagctttggagtgtctggagtaaatgaatcagctgacatgagtattggagtaacagtgataaagaacaacatgataaacaatgaccttggacctgcaacggcccagatggctcttcaattgttcatcaaagactacagatacacatataggtgccataggggagacacacaaattcagacgagaagatcatttgagttaaagaagctgtgggatcaaacccaatcaaaggtagggctattagtatcagatggaggaccaaacttatacaatatacggaatcttcacattcctgaagtctgcttaaaatgggagctaatggatgatgattatcggggaagactttgtaatcccctgaatccctttgtcagtcataaagagattgattctgtaaacaatgctgtggtaatgccagcccatggtccagccaaaagcatggaatatgatgccgttgcaactacacattcctggattcccaagaggaatcgttctattctcaacacaagccaaaggggaattcttgaggatgaacagatgtaccagaagtgctgcaatctattcgagaaatttttccctagcagttcatataggagaccggttggaatttctagcatggtggaggccatggtgtctagggcccggattgatgccagggtcgacttcgagtctggacggatcaagaaagaagagttctctgagatcatgaagatctgttccaccattgaagaaatcagacggcaaaaataa

>H1N1_PB1_CY058690_A/Texas/46182018/2009

atggatgtcaatccgactctacttttcctaaaaattccagcgcaaaatgccataagcaccacattcccttatactggagatcctccatacagccatggaacaggaacaggatacaccatggacacagtaaacagaacacaccaatactcagaaaagggaaagtggacgacaaacacagagactggtgcaccccagctcaacccgattgatggaccactacctgaggataatgaaccaagtgggtatgcacaaacagactgtgttctagaggctatggctttccttgaagaatcccacccaggaatatttgagaattcatgccttgaaacaatggaagttgttcaacaaacaagggtagataaactaactcaaggtcgccagacttatgattggacattaaacagaaatcaaccggcagcaactgcattggccaacaccatagaagtctttagatcgaatggcctaacagctaatgagtcaggaaggctaatagatttcttaaaggatgtaatggaatcaatgaacaaagaggaaatagagataacaacccactttcaaagaaaaaggagagtaagagacaacatgaccaagaagatggtcacgcaaagaacaatagggaagaaaaaacaaagactgaataagagaggctatctaataagagcactgacattaaatacgatgaccaaagatgcagagagaggcaagttaaaaagaagggctatcgcaacacctgggatgcagattagaggtttcgtatactttgttgaaactttagctaggagcatttgcgaaaagcttgaacagtctgggctcccagtagggggcaatgaaaagaaggccaaactggcaaatgttgtgagaaagatgatgactaattcacaagacacagagatttctttcacaatcactggggacaacactaagtggaatgaaaatcaaaatcctcgaatgttcctggcgatgattacatatatcaccagaaatcaacccgagtggttcagaaacatcctgagcatggcacccataatgttctcaaacaaaatggcaagactagggaaagggtacatgttcgagagtaaaagaatgaagattcgaacacaaataccagcagaaatgctagcaagcattgacctgaagtacttcaatgaatcaacaaagaagaaaattgagaaaataaggcctcttctaatagatggcacagcatcactgagtcctgggatgatgatgggcatgttcaacatgctaagtacggtcttgggagtctcgatactgaatcttggacaaaagaaatacaccaagacaatatactggtgggatgggctccaatcatccgacgattttgctctcatagtgaatgcaccaaaccatgagggaatacaagcaggagtggacagattctacaggacctgcaagttagtgggaatcaacatgagcaaaaagaagtcctatataaataagacagggacatttgaattcacaagctttttttatcgctatggatttgtggctaattttagcatggagctacccagctttggagtgtctggagtaaatgaatcagctgacatgagtattggagtaacagtgataaagaacaacatgataaacaatgaccttggacctgcaacggcccagatggctcttcaattgttcatcaaagactacagatacacatataggtgccataggggagacacacaaattcagacgagaagatcatttgagttaaagaagctgtgggatcaaacccaatcaaagggagggctattagtatcagacggaggaccaaacttatacaatgtacggaatcttcacattcctgaagtctgcttaaaatgggagctaatggatgatgattatcggggaagactttgtaatcccctgaatccctttgtcagtcataaagagattgattctgtaaacaatgctgtggtaatgccagcccatggtccagtcaaaagcatggaatatgatgccgttgcaactacacattcctggattcccaagaggaatcgttctattctcaacacaagccaaaggggaattcttgaggatgaacagatgtatcagaagtgctgcaatctattcgagaaatttttccctagcagttcatataggagaccggttggaatttctagcatggtggaggccatggtgtctagggcccggattgatgccagggtcgacttcgagtctggacggatcaagaaagaagagttctctgagatcatgaagatctgctccaccattgaagaactcagacggcaaaaataa

>H1N1_PB1_GU931815_A/Hong Kong/415742/2009

atggatgtcaatccgactctacttttcctaaaaattccagcgcaaaatgccataagcaccacattcccttatactggagatcctccatacagccatggaacaggaacaggatacaccatggacacagtaaacagaacacaccaatactcagaaaagggaaagtggacgaccaacacagagactggtgcaccccagctcaacccgattgatggaccactacctgaggataatgaaccaagtgggtatgcacaaacagactgtgttctagaggctatggctttccttgaagaatcccacccaggaatatttgagaattcatgccttgaaacaatggaagttgttcaacaaacaagggtagataaactaactcaaggtcgccagacttatgattggacattaaacagaaatcaaccggcagcaactgcattggccaacaccatagaagtctttagatcgaatggcctaacagcaaatgagtcaggaaggctaatagatttcttaaaggatgtaatggaatcaatgaacaaagaggaaatagagataacaacccactttcaaagaaaaaggagagtaagagacaacatgaccaagaagatggtcacgcaaagaacaatagggaagaaaaaacaaagactgaataagagaggctatctaataagagcactgacattaaatacgatgaccaaagatgcagagagaggcaagttaaaaagaagggctatcgcaacacctgggatgcagattagaggtttcgtatactttgttgaaactttagctaggagcatttgcgaaaagcttgaacagtctgggctcccagtagggggcaatgaaaagaaggccaaactggcaaatgttgtgagaaagatgatgactaattcacaagacacagagatttctttcacaatcactggggacaacactaagtggaatgaaaatcaaaatccccgaatgttcctggcgatgattacatatatcaccagaaatcaacccgagtggttcagaaacatcctgagcatggcacccataatgttctcaaacaaaatggcaagactagggaaagggtacatgttcgagagtaaaagaatgaaaattcgaacwcaaataccagcagaaatgctagcaagcattgacctgaagtacttcaatgaatcaacaaagaagaaaattgagaaaataaggcctcttctaatagatggcacagcatcactgagtcctgggatgatgatgggcatgttcaacatgctaagtacggtcttgggagtctcgatactgaatcttggacaaaagaaatacaccaagacaatatactggtgggatgggctccaatcatccgacgattttgctctcatagtgaatgcaccaaaccatgagggaatacaagcaggagtggacagattctacaggacctgcaagttagtgggaatcaacatgagcaaaaagaagtcctatataaataagacagggacatttgaattcacaagctttttttatcgctatggatttgtggctaattttagcatggagctacccagctttggagtgtctggagtaaatgaatcagctgacatgagtattggagtaacagtgataaagaacaacatgataaacaatgaccttggacctgcaacggcccagatggctcttcaattgttcatcaaagactacagatacacatataggtgccataggggagacacacaaattcagacgagaagatcatttgagttaaagaagctgtgggatcaaacccaatcaaaggtagggctattagtatcagatggaggaccaaacttatacaatatacggaatcttcacattcctgaagtctgcttaaaatgggagctaatggatgatgattatcggggaagactttgtaatcccctgaatccctttgtcagtcataaagagattgattctgtaaacaatgctgtggtaatgccagcccatggtccagccaaaagcatggaatatgatgccgttgcaactacacattcctggattcccaagaggaatcgttctattctcaacmcaagccaaaggggaattyttgaggatgaacagatgtaccagaagtgctgcaatctattcgagaaatttttccctagcagttcatataggagaccggttggaatttctagcatggtggaggccatggtgtctagggcccggattgatgccagggtcgacttcgagtctggacggatcaagaaagaagagttctctgagatcatgaagatctgttccaccattgaagaactcagacggcaaaaataa

>H1N1_PB1_CY060724_A/Ontario/328474/2009

atggatgtcaatccgactctacttttcctaaaaattccagcgcaaaatgccataagcaccacattcccttatactggagatcctccatacagccatggaacaggaacaggatacaccatggacacagtaaacagaacacaccaatactcagaaaagggaaagtggacgacaaacacagagactggtgcaccccagctcaacccgattgatggaccactacctgaggataatgaaccaagtgggtatgcacaaacagactgtgttctagaggctatggctttccttgaagaatcccacccaggaatatttgagaattcatgccttgaaacaatggaagttgttcaacaaacaagggtagataaactaactcaaggtcgccagacttatgattggacattaaacagaaatcaaccggcagcaactgcattggccaacaccatagaagtctttagatcgaatggcctaacagctaatgagtcaggaaggctaatagatttcttaaaggatgtaatggaatcaatgaacaaagaggaaatagagataacaacccactttcaaagaaaaaggagagtaagagacaacatgaccaagaagatggtcacgcaaagaacaatagggaagaaaaaacaaagactgaataagagaggctatctaataagagcactgacattaaatacgatgaccaaagatgcagagagaggcaagttaaaaagaagggctatcgcaacacctgggatgcagattagaggtttcgtatactttgttgaaactttagctaggagcatttgcgaaaagcttgaacaggctgggctcccagtagggggcaatgaaaagaaggccaaactggcaaatgttgtgagaaagatgatgactaattcacaagacacagagatttctttcacaatcactggggacaacactaagtggaatgaaaatcaaaatcctcgaatgttcctggcgatgattacatatatcaccagaaatcaacccgagtggttcagaaacatcctgagcatggcacccataatgttctcaaacaaaatggcaagactagggaaagggtacatgttcgagagtaaaagaatgaagattcgaacacaaataccagcagaaatgctagcaagcattgacctgaagtacttcaatgaatcaacaaagaagaaaattgagaaaataaggcctcttctaatagatggcacagcatcactgagtcctgggatgatgatgggcatgttcaacatgctaagtacggtcttgggagtctcgatactgaatcttggacaaaagaaatacaccaagacaatatactggtgggatgggctccaatcatccgacgattttgctctcatagtgaatgcaccaaaccatgagggaatacaagcaggagtggacagattctacaggacctgcaagttagtgggaatcaacatgagcaaaaagaagtcctatataaataagacagggacatttgaattcacaagctttttttatcgctatggatttgtggctaattttagcatggagctacccagctttggagtgtctggagtaaatgaatcagctgacatgagtattggagtaacagtgataaagaacaacatgataaacaatgaccttggacctgcaacggcccagatggctcttcaactgttcatcaaagactacagatacacatataggtgccataggggagacacacaaattcagacgagaagatcatttgagttaaagaagctgtgggatcaaacccaatcaaaggtagggctattagtatcagatggaggaccaaacttatacaatatacggaatcttcacattcctgaagtctgcttaaaatgggagctaatggatgatgattatcggggaagactttgtaatcccctgaatccctttgtcagtcataaagagattgattctgtaaacaatgctgtggtaatgccagcccatggtccagccaaaagcatggaatatgatgccgttgcaactacacattcctggattcccaagaggaatcgttctattctcaacacaagccaaaggggaattcttgaggatgaacagatgtaccagaagtgctgcaatctattcgagaaatttttccctagcagttcatataggagaccggttggaatttctagcatggtggaggccatggtgtctagggcccggattgatgccagggtcgacttcgagtctggacggatcgggaaggaagagttctctgagatcatgaagatctgttccaccattgaagaactcagacggcaaaaataa

>H1N1_PB1_CY060748_A/Ontario/9739/2009

atggatgtcaatccgactctacttttcctaaaaattccagcgcaaaatgccataagcaccacattcccttatactggagatcctccatacagccatggaacaggaacaggatacaccatggacacagtaaacagaacacaccaatactcagaaaagggaaagtggacgacaaacacagagactggtgcaccccagctcaacccgattgatggaccactacctgaggataatgaaccaagtgggtatgcacaaacagactgtgttctagaggctatggctttccttgaagaatcccacccaggaatatttgagaattcatgccttgaaacaatggaagttgttcaacaaacaagggtagataaactaactcaaggtcgccagacttatgattggacattaaacagaaatcaaccggcagcaactgcattggccaacaccatagaagtctttagatcgaatggtctaacagctaatgagtcaggaaggctaatagatttcttaaaggatgtaatggaatcaatgaacaaagaggaaatagagataacaacccactttcaaagaaaaaggagagtaagagacaacatgaccaagaagatggtcacacaaagaacaatagggaagaaaaaacaaagactgaataagagaggctatctaataagagcactgacattaaatacgatgaccaaagatgcagagagaggcaagttaaaaagaagggctatcgcaacacctgggatgcagattagaggtttcgtatactttgttgaaactttagctaggagcatttgcgaaaagcttgaacagtctgggctcccagtagggggcaatgaaaagaaggccaaactggcaaatgttgtgagaaagatgatgactaattcacaagacacagagatttctttcacaatcactggggacaacactaagtggaatgaaaatcaaaatcctcgaatgttcctggcgatgattacatatatcaccagaaatcaacccgagtggttcagaaacatcctgagcatggcacccataatgttctcaaacaaaatggcaagactagggaaagggtacatgttcgagagtaaaagaatgaagattcgaacacaaataccagcagaaatgctagcaagcattgacctgaagtacttcaatgaatcaacaaagaagaaaattgagaaaataaggcctcttctaatagatggcacagcatcactgagtcctgggatgatgatgggcatgttcaacatgctaagtacggtcttgggagtctcgatactgaatcttggacaaaagaaatacaccaagacaatatactggtgggatgggctccaatcatccgacgattttgctctcatagtgaatgcaccaaaccatgagggaatacaagcaggagtggacagattctacaggacctgcaagttagtgggaatcaacatgagcaaaaagaagtcctatataaataagacagggacatttgaattcacaagctttttttatcgctatggatttgtggctaattttagcatggagctacccagctttggagtgtctggagtaaatgaatcagctgacatgagtattggagtaacagtgataaagaacaacatgataaacaatgaccttggacctgcaacggcccagatggctcttcaattgttcatcaaagactacagatacacatataggtgccataggggagacacacaaattcagacgagaagatcatttgagttaaagaagctgtgggatcaaacccaatcaaaggtagggctattagtatcagatggaggaccaaacttatacaatatacggaatcttcacattcctgaagtctgcttaaaatgggagctaatggatgatgattatcggggaagactttgtaatcccctgaatccctttgtcagtcataaagagattgattctgtaaacaatgctgtggtaatgccagcccatggtccagccaaaagcatggaatatgatgccgttgcaactacacattcctggattcccaagaggaatcgttctattctcaacacaagccaaaggggaattcttgaggatgaacagatgtaccagaagtgttgcaatctattcgagaaatttttccctagcagttcatataggagaccggttggaatttctagcatggtggaggccatggtgtctagggcccggattgatgccagggtcgacttcgagtctggacggatcaagaaagaagagttctctgagatcatgaagatctgttccaccattgaagaactcagacggcaaaaataa

>H1N1_PB1_CY061912_A/New York/6903/2009

atggatgtcaatccgactctacttttcctaaaaattccagcgcaaaatgctataagcaccacattcccttatactggagatcctccatacagccatggaacaggaacaggatacaccatggacacagtaaacagaacacaccaatactcagaaaagggaaagtggacgacaaacacagagactggtgcaccccagctcaacccgattgatggaccactacctgaggataatgaaccaagtgggtatgcacaaacagactgtgttctagaggctatggctttccttgaagaatcccacccaggaatatttgagaattcatgccttgaaacaatggaaattgttcaacaaacaagggtagataaactaactcaaggtcgccagacttatgattggacattaaacagaaatcaaccggcagcaactgcattggccaacaccatagaagtctttagatcgaatggcctaacagctaatgagtcaggaaggctaatagatttcttaaaggatgtaatggaatcaatgaacaaagaggaaatagagataacaacccactttcaaagaaaaaggagagtaagagacaacatgaccaagaagatggtcacgcaaagaacaatagggaagaaaaaacaaagactgaataagagaggctatctaataagagcactgacattaaatacgatgaccaaagatgcagagagaggcaagttaaaaagaagggctatcgcaacaccagggatgcagattagaggtttcgtatactttgttgaaactttagctaggagcatttgcgaaaagcttgaacagtctgggctcccagtagggggcaatgaaaagaaggccaaactggcaaatgttgtgagaaagatgatgactaattcacaagacacagagatttctttcacaatcactggggacaacactaagtggaatgaaaatcaaaatcctcgaatgttcctggcgatgattacatatatcaccagaaatcaacccgagtggttcagaaacatcctgagcatggcacccataatgttctcaaacaaaatggcaagactagggaaagggtacatgttcgagagtaaaagaatgaagattcgaacacaaataccagcagaaatgctagcaagcattgacctgaagtacttcaatgaatcaacaaagaagaaaattgagaaaataaggcctcttctaatagatggcacagcatcactgagtcctgggatgatgatgggcatgttcaacatgctaagtacagtcttgggagtctcgatactgaatcttggacaaaagaaatacaccaagacaatatactggtgggatgggctccaatcatccgacgattttgctctcatagtgaatgcaccaaaccatgagggaatacaagcaggagtggacagattctacaggacctgcaagttagtgggaatcaacatgagcaaaaagaagtcctatataaataagacagggacatttgaattcacaagctttttttatcgctatggatttgtggctaattttagcatggagctacccagctttggagtgtctggagtaaatgaatcagctgacatgagtattggagtaacagtgataaagaacaacatgataaacaatgaccttggacctgcaacggcccagatggctcttcaattgttcatcaaagactacagatacacatataggtgccataagggagacacacaaattcagacgagaagatcatttgagttaaagaagctgtgggatcaaacccaatcaaaggtagggctattagtatcagatggaggaccaaacttatacaatatacggaatcttcacattcctgaagtctgcttaaaatgggagctaatggatgatgattatcggggaagactttgtaatcccctgaatccctttgtcagtcataaagagattgattctgtaaacaatgctgtggtaatgccagcccatggtccagctaaaagcatggaatatgatgccgttgcaactacacattcctggattcccaagaggaatcgttctattctcaacacaagccaaaggggaattcttgaggatgaacagatgtaccagaagtgctgcaatctattcgagaaatttttccctagcagttcatataggagaccggttggaatttctagcatggtggaggccatggtgtctagggcccggattgatgccagggtcgacttcgagtctggacggatcaagaaagaagagttctctgagatcatgaagatctgttccaccattgaagaactcagacggcaaaaataa

>H1N1_PB1_CY062769_A/Madrid/INS134/2009

atggatgtcaatccgactctacttttcctaaaaattccagcgcaaaatgccataagcaccacattcccttatactggagatcctccatacagccatggaacaggaacaggatacaccatggacacagtaaacagaacacaccaatactcagaaaagggaaagtggacgacaaacacagagactggtgcaccccagctcaacccgattgatggaccactacctgaggataatgaaccaagtgggtatgcacaaacagactgtgttctagaggctatggctttccttgaagaatcccacccaggaatatttgagaattcatgccttgaaacaatggaagttgttcaacaaacaagggtagataaactaactcaaggtcgccagacttatgattggacattaaacagaaatcaaccggcagcaactgcattggccaacaccatagaagtctttagatcgaatggcctaacagctaatgagtcaggaaggctaatagatttcttaaaggatgtaatggaatcaatgaacaaagaggaaatagagataacaacccactttcaaagaaaaaggagagtaagagacaacatgaccaagaagatggtcacgcaaagaacaatagggaagaaaaaacaaagactgaataagagaggctatctaataagagcactgacattaaatacgatgaccaaagatgcagagagaggcaagttaaaaagaagggctatcgcaacacctgggatgcagattagaggtttcgtatactttgttgaaactttagctaggagcatttgcgaaaagcttgaacagtctgggctcccagtagggggcaatgaaaagaaggccaaactggcaaatgttgtgagaaagatgatgactaattcacaagacacagagatttctttcacaatcactggggacaacactaagtggaatgaaaatcaaaatcctcgaatgttcctggcgatgattacatatatcaccagaaatcaacccgagtggttcagaaacatcctgagcatggcacccataatgttctcaaacaaaatggcaagactagggaaagggtacatgttcgagagtaaaagaatgaagattcgaacacaaataccagcagaaatgctagcaagcattgacctgaagtacttcaatgaatcaacaaagaagaaaattgagaaaataaggcctcttctaatagatggcacagcatcactgagtcctgggatgatgatgggcatgttcaacatgctaagtactgtcttgggagtctcgatactgaatcttggacaaaagaaatacaccaagacaatatactggtgggatgggctccaatcatccgacgattttgctctcatagtgaatgcaccaaaccatgagggaatacaagcaggagtggacagattctacaggacctgcaagttagtgggaatcaacatgagcaaaaagaagtcctatataaataagacagggacatttgaattcacaagctttttttatcgctatggatttgtggctaattttagcatggagctacccagctttggagtgtctggagtaaatgaatcagctgacatgagtattggagtaacagtgataaagaacaacatgataaacaatgaccttggacctgcaacggcccagatggctcttcaattgttcatcaaagactacagatacacatataggtgccataggggagacacacaaattcagacgagaagatcatttgagttaaagaagctgtgggatcaaacccaatcaaaggtagggctattagtatcagatggaggaccaaacttatacaatatacggaatcttcacattcctgaagtctgcttaaaatgggagctaatggatgatgattatcggggaagactttgtaatcccctgaatccctttgtcagtcataaagagattgattctgtaaacaatgctgtggtaatgccagcccatggtccagccaaaagcatggaatatgatgccgttgcaactacacattcctggattcccaagaggaatcgttctattctcaacacaagccaaaggggaattcttgaggatgaacagatgtaccagaagtgctgcaatctattcgagaaatttttccctagcagttcatataggagaccggttggaatttctagcatggtggaggccatggtgtctagggcccggattgatgccagggtcgacttcgagtctggacggatcaagaaagaagagttctctgagatcatgaagatctgttccaccattgaagaactcagacggcaaaaataa

>H1N1_PB1_CY063017_A/Tallinn/INS183/2010

atggatgtcaatccgactctacttttcctaaaaattccagcgcaaaatgccataagcaccacattcccttatactggagatcctccatacagccatggaacaggaacaggatacaccatggacacagtaaacagaacacaccaatactcagaaaagggaaagtggacgacaaacacagagactggtgcaccccagctcaacccgattgatggaccactacctgaggataatgaaccaagtgggtatgcacaaacagactgtgttctagaggctatggctttccttgaagaatcccacccaggaatatttgagaattcatgccttgaaacaatggaagttgttcaacaaacaagggtagataaactaactcaaggtcgccagacttatgattggacattaaacagaaatcaaccggcagcaactgcattggccaacaccatagaagtctttagatcgaatggcctaacagctaatgagtcaggaaggctaatagatttcttaaaggatgtaatggaatcaatgaacaaagaggaaatagagataacaacccactttcaaagaaaaaggagagtaagagacaacatgaccaagaagatggtcacgcaaagaacaatagggaagaaaaaacaaagactgaataagagaggctatctaataagagcactgacattaaatacgatgaccaaagatgcagagagaggcaagttaaaaagaagggctatcgcaacacctgggatgcagattagaggtttcgtatactttgttgaaactttagctaggagcatttgcgaaaagcttgaacagtctgggctcccagtagggggcaatgaaaagaaggccaaactggcaaatgttgtgagaaagatgatgactaattcacaagacacagagatttctttcacaatcactggggataacactaagtggaatgaaaatcaaaatcctcgaatgttcctggcgatgattacatatatcaccagaaatcaacccgagtggttcagaaacatcctgagcatggcacccataatgttctcaaacaaaatggcaagactagggaaagggtacatgttcgagagtaaaagaatgaagattcgaacacaaataccagcagaaatgctagcaagcattgacctgaagtacttcaatgaatcaacaaagaagaaaattgagaaaataaggcctcttctaatagatggcacagcatcactgagtcctgggatgatgatgggcatgttcaacatgctaagtacggtcttgggagtctcgatactgaatcttggacaaaagaaatacaccaagacaatatattggtgggatgggctccaatcatccgacgattttgctctcatagtgaatgcaccaaaccatgagggaatacaagcaggagtggacagattctacaggacctgcaagttagtgggaatcaacatgagcaaaaagaagtcctatataaataagacagggacatttgaattcacaagctttttttatcgctatggatttgtggctaattttagcatggagctacccagctttggagtatctggagtaaatgaatcagctgacatgagtattggagtaacagtgataaagaacaacatgataaacaatgaccttggacctgcaacggcccagatggctcttcaattgttcatcaaagactacagatacacatataggtgccataggggggacacacaaattcagacgagaagatcatttgagttaaagaagctgtgggatcaaacccaatcaaaggtagggctattagtatcagatggaggaccaaacttatacaatatacggaatcttcacattcctgaagtttgcttaaaatgggagctaatggatgatgattatcggggaagactttgtaatcccctgaatccctttgtcagtcataaagagattgattctgtaaacaatgctgtggtaatgccagcccatggtccagccaaaagcatggaatatgatgccgtcgcaactacacattcctggattcccaagaggaatcgttctattctcaacacaagccaaaggggaattcttgaggatgaacagatgtaccagaagtgctgcaatctattcgagaaatttttccctagcagttcatataggagaccggttggaatttctagcatggtggaggccatggtgtctagggcccggattgatgccagggtcgacttcgagtctggacggatcaagaaagaagagttctctgagatcatgaagatctgttccaccattgaagaactcagacggcaaaaataa

>H1N1_PB1_CY063233_A/Wisconsin/629-D00675/2009

atggatgtcaatccgactctacttttcctaaaaattccagcgcaaaatgccataagcaccacattcccttatactggagatcctccatacagccatggaacaggaacaggatacaccatggatacagtaaacagaacacaccaatactcagaaaagggaaagtggacgacaaacacagagactggtgcaccccagctcaacccgattgatggaccactacctgaggataatgaaccaagtgggtatgcacaaacagactgtgttctagaggctatggctttccttgaagaatcccacccaggaatatttgagaattcatgccttgaaacaatggaagttgttcaacaaacaagggtagataaactaactcaaggtcgccagacttatgattggacattaaacagaaatcaaccggcagcaactgcattggccaacaccatagaagtctttagatcgaatggcctaacagctaatgagtcaggaaggctaatagatttcttaaaggatgtaatggaatcaatgaacaaagaggaaatagagataacaacccactttcaaagaaaaaggagagtaagagacaacatgaccaagaagatggtcacgcaaagaacaatagggaagaaaaaacaaagactgaataagagaggctatctaataagagcactgacattaaatacgatgaccaaagatgcagagagaggcaagttaaaaagaagggctatcgcaacacctgggatgcagattagaggtttcgtatactttgttgaaactttagctaggagcatttgcgaaaagcttgaacagtctgggctcccagtagggggcaatgaaaagaaggccaaactggcaaatgttgtgagaaagatgatgactaattcacaagacacagagatttctttcacaatcactggggacaacactaagtggaatgaaaatcaaaatcctcgaatgttcctggcgatgattacatatatcaccagaaatcaacccgagtggttcagaaacatcctgagcatggcacccataatgttctcaaacaaaatggcaagactagggaaagggtacatgttcgagagtaaaagaatgaagattcgaacacaaataccagcagaaatgctagcaagcattgacctgaagtacttcaatgaatcaacaaagaagaaaattgagaaaataaggcctcttctaatagatggcacagcatcactgagtcctgggatgatgatgggcatgttcaacatgctaagtacggtcttgggagtctcgatactgaatcttggacaaaagaaatacaccaagacaatatactggtgggatgggctccaatcatccgacgattttgctctcatagtgaatgcaccaaaccatgagggaatacaagcaggagtggacagattctacaggacctgcaagttagtgggaatcaacatgagcaaaaagaagtcctatataaataagacagggacatttgaattcacaagctttttttatcgctatggatttgtggctaattttagcatggagctacccagctttggagtgtctggagtaaatgaatcagctgacatgagtattggagtaacagtgataaagaacaacatgataaacaatgaccttggacctgcaacggcccagatggctcttcaattgttcatcaaagactacagatacacatataggtgccataggggagacacacaaattcagacgagaagatcatttgagttaaagaagctgtgggatcaaacccaatcaaaggtagggctattagtatcagatggaggaccaaacttatacaatatacggaatcttcacattcctgaagtctgcttaaaatgggagctaatggatgatgattatcggggaagactttgtaatcccctgaatccctttgtcagtcataaagagattgattctgtaaacaatgctgtggtaatgccagcccatggtccagccaaaagcatggaatatgatgccgttgcaaccacacattcctggattcccaagaggaatcgttctattctcaacacaagccaaaggggaattcttgaggatgaacagatgtaccagaagtgctgcaatctattcgagaaatttttccctagcagttcatataggagaccggttggaatttctagcatggtggaggccatggtgtctagggcccggattgatgccagggtcgacttcgagtctggacggatcaagaaagaagagttctctgagatcatgaagatctgttccaccattgaagaactcagacggcaaaaataa

>H1N1_PB1_CY063604_A/Bonn/INS128/2009

atggatgtcaatccgactctacttttcctaaaaattccagcgcaaaatgccataagcaccacattcccttatactggagatcctccatacagccatggaacaggaacaggatacaccatggacacagtaaacagaacacaccaatactcagaaaagggaaagtggacgacaaacacagagactggtgcaccccagctcaacccgattgatggaccactacctgaggataatgaaccaagtgggtatgcacaaacagactgtgttctagaggctatggctttccttgaagaatcccacccaggaatatttgagaattcatgccttgaaacaatggaagttgttcaacaaacaagggtagataaactaactcaaggtcgccagacttatgattggacattaaacagaaatcaaccggcagcaactgcattggccaacaccatagaagtctttagatcgaatggcctaacagctaatgagtcaggaaggctaatagatttcttaaaggatgtaatggaatcaatgaacaaagaggaaatagagataacaacccactttcaaagaaaaaggagagtaagagacaacatgaccaagaagatggtcacgcaaagaacaatagggaagaaaaaacaaagactgaataagagaggctatctaataagagcactgacattaaatacgatgaccaaagatgcagagagaggcaagttaaaaagaagggctatcgcaacacctgggatgcagattagaggtttcgtatactttgttgaaactttagctaggagcatttgcgaaaagcttgaacagtctgggctcccagtagggggcaatgaaaagaaggccaaactggcaaatgttgtgagaaagatgatgactaattcacaagacacagagatttctttcacaatcactggggacaacactaagtggaatgaaaatcaaaatcctcgaatgttcctggcgatgattacatatatcaccagaaaycaacccgagtggttcagaaacatcctgagcatggcacccataatgttctcaaacaaaatggcaagactagggaaagggtacatgttcgagagtaaaagaatgaagattcgaacacaaataccagcagaaatgctagcaagcattgacctgaagtacttcaatgaatcaacaaagaagaaaattgagaaaataaggcctcttctaatagatggcacagcatcactgagtcctgggatgatgatgggcatgttcaacatgctaagtacggtcttgggagtctcgatactgaatcttggacaaaagaaatacaccaagacaatatactggtgggatgggctccaatcatccgacgattttgctctcatagtgaatgcaccaaaccatgagggaatacaagcaggagtggacagattctacaggacctgcaagttagtgggaatcaacatgagcaaaaagaagtcctatataaataagacagggacatttgaattcacaagctttttttatcgctatggatttgtggctaattttagcatggagctacccagctttggagtgtctggagtaaatgaatcagctgacatgagtattggagtaacagtgataaagaacaacatgataaacaatgaccttggacctgcaacggcccagatggctcttcaattgttcatcaaagactacagatacacatataggtgccataggggagacacacaaattcagacgagaagatcatttgagttaaagaagctgtgggatcaaacccaatcaaaggtagggctattagtatcagatggaggaccaaacttatacaatatacggaatcttcacattcctgaagtctgcttaaaatgggagctaatggatgatgattatcggggaagactttgtaatcccctgaatccctttgtcagtcataaagagattgattctgtaaacaatgctgtggtaatgccagcccatggtccagccaaaagcatggaatatgatgccgttgcaactacacattcctggattcccaagaggaatcgttctattctcaacacaagccaaaggggaattcttgaggatgaacagatgtaccagaagtgctgcaatctattcgagaaatttttccctagcagttcatataggagaccggttggaatttctagcatggtggaggccatggtgtctagggcccggattgatgccagggtcgacttcgagtctggacggatcaagaaagaagagttctctgagatcatgaagatctgttccaccattgaagaactcagacggcaaaaataa

>H1N1_PB1_CY064993_A/California/VRDL6/2010

atggatgtcaatccgactctacttttcctaaaaattccagcgcaaaatgctataagcaccacattcccttatactggagatcctccatacagccatggaacaggaacaggatacaccatggacacagtaaacagaacacaccaatactcagaaaagggaaagtggacgacaaacacagagactggtgcaccccagctcaacccgattgatggaccactacctgaggataatgaaccaagtgggtatgcacaaacagactgtgttctagaggctatggctttccttgaagaatcccacccgggaatatttgagaattcatgccttgaaacaatggaagttgttcaacaaacaagggtagataaactaactcaaggtcgccagacttatgattggacattaaacagaaatcaaccggcagcaactgcattggccaacaccatagaggtcttcagatcaaatggcctaacagctaatgagtcaggaaggctaatagatttcttaaaggatgtaatggaatcaatgaacaaagaggaaatagagataacaacccactttcaaagaaaaaggagagtaagagacaacatgaccaagaagatggtcacgcaaagaacaatagggaagaaaaaacaaagactgaataaaagaggctatctaataagagcactgacattaaatacaatgaccaaagatgcagagagaggcaagttaaaaagaagggctatcgcaacacctgggatgcagattagaggtttcgtatactttgttgaaactttagctaggagcatttgcgaaaagcttgaacagtctgggctcccagtagggggcaatgaaaagaaggccaaactggcaaatgttgtgagaaagatgatgactaattcacaagacacagagatttctttcacaatcactggggacaacactaagtggaatgaaaatcaaaatcctcgaatgttcctggcgatgattacatatatcaccagaaatcaacccgagtggttcagaaacatcctgagcatggcacccataatgttctcaaacaaaatggcaagactagggaaagggtacatgttcgagagtaaaagaatgaagattcgaacacaaataccagcagaaatgttagcaagcattgacctgaagtacttcaatgaatcaacaaagaagaaaattgagaaaataaggcctcttctaatagatggcacagcatcactgagtcctgggatgatgatgggcatgttcaacatgctaagtacggtcttgggagtctcgatactgaatcttggacaaaagaaatacaccaagacaatatactggtgggatgggctccaatcatccgacgattttgctctcatagtgaatgcaccaaaccatgagggaatacaagcaggagtggacagattctacaggacctgcaagttagtgggaatcaacatgagcaaaaagaagtcctatataaataagacagggacatttgaattcacaagctttttttatcgctatggatttgtggctaattttagcatggagctacccagctttggagtgtctggagtaaatgaatcagctgacatgagtattggagtaacagtgataaagaacaacatgataaacaatgaccttggacctgcaacggcccagatggctcttcaattgttcatcaaagactacagatacacatataggtgccataggggagacacacaaattcagacgagaagatcatttgagttaaagaagctgtgggatcaaacccaatcaaagggagggctattagtatcagatggaggaccaaacttatacaatatacggaatcttcacattcctgaagtctgcttaaaatgggagctaatggatgatgattatcggggaagactttgtaatcccctgaatccctttgtcagtcataaagagattgattctgtaaacaatgctgtggtaatgccagcccatggtccagtcaaaagcatggaatatgatgccgttgcaactacacattcctggattcccaagaggaatcgttctattctcaacacaagccaaaggggaattcttgaggatgaacagatgtaccagaagtgctgcaatctattcgagaaatttttccctagcagttcatataggagaccggttggaatttctagcatggtggaggccatggtgtctagggcccggattgatgccagggtcgacttcgagtctggacggatcaagaaagaagagttctctgagatcatgaagatctgctccaccattgaagaactcagacggcaaaaataa

>H1N1_PB1_HM567606_A/England/328/2009

atggatgtcaatccgactctacttttcctaaaaattccagcgcaaaatgccataagcaccacattcccttatactggagatcctccatacagccatggaacaggaacaggatacaccatggacacagtaaacagaacacaccaatactcagaaaagggaaagtggacgacaaacacagagactgatgcaccccagctcaacccgattgatggaccactacctgaggataatgaaccaagtgggtatgcacaaacagactgtgttctagaggctatggctttccttgaagaatcccacccaggaatatttgagaattcatgccttgaaacaatggaagttgttcaacaaacaagggtagataaactaactcaaggtcgccagacttatgattggacattaaacagaaatcaaccggcagcaactgcattggccaacaccatagaagtctttagatcgaatggcctaacagctaatgagtcaggaaggctaatagatttcttaaaggatgtaatggaatcaatgaacaaagaggaaatagagataacaacccactttcaaagaaaaaggagagtaagagacaacatgaccaagaagatggtcacgcaaagaacaatagggaagaaaaaacaaagactgaataagagaggctatctaataagagcactgacattaaatacgatgaccaaagatgcagagagaggcaagttaaaaagaagggctatcgcaacacctgggatgcagattagaggtttcgtatactttgttgaaactttagctaggagcatttgcgaaaagcttgaacagtctgggctcccagtagggggcaatgaaaagaaggccaaactggcaaatgttgtgagaaagatgatgactaattcacaagacacagagatttctttcacaatcactggggacaacactaagtggaatgaaaatcaaaatcctcgaatgttcctggcgatgattacatatatcaccagaaatcaacccgagtggttcagaaacatcctgagcatggcacccataatgttctcaaacaaaatggcaagactagggaaagggtacatgttcgagagtaaaagaatgaagattcgaacacaaataccagcagaaatgctagcaagcattgacctgaagtacttcaatgaatcaacaaagaagaaaattgagaaaataaggcctcttctaatagatggcacagcatcactgagtcctgggatgatgatgggcatgttcaacatgctaagtacggtcttgggagtctcgatactgaatcttggacaaaagaaatacaccaagacaatatactggtgggatgggctccaatcatccgacgattttgctctcatagtgaatgcaccaaaccatgagggaatacaagcaggagtggacagattctacaggacctgcaagttagtgggaatcaacatgagcaaaaagaagtcctatataaataagacagggacatttgaattcacaagctttttttatcgctatggatttgtggctaattttagcatggagctacccagctttggagtgtctggagtaaatgaatcagctgacatgagtattggagtaacagtgataaagaacaacatgataaacaatgaccttggacctgcaacggcccagatggctcttcaattgttcatcaaagactacagatacacatataggtgccataggggagacacacaaattcagacgagaagatcatttgagttaaagaagctgtgggatcaaacccaatcaaaggcagggctattagtatcagatggaggaccaaacttatacaatatacggaatcttcacattcctgaagtctgcttaaaatgggagctaatggatgatgattatcggggaagactttgtaatcccctgaatccctttgtcagtcataaagagattgattctgtaaacaatgctgtggtaatgccagcccatggtccagccaaaagcatggaatatgatgccgttgcaactacacattcctggattcccaagaggaatcgttctattctcaacacaagccaaaggggaattcttgaggatgaacagatgtaccagaagtgctgcaatctattcgagaaatttttccctagcagttcatataggagaccggttggaatttctagcatggtggaggccatggtgtctagggcccggattgatgccagggtcgacttcgagtctggacggatcaagaaagaagagttctctgagatcatgaagatctgttccaccattgaagaactcagacggcaaaaataa

>H1N1_PB1_HM567974_A/England/1111/2009

atggatgtcaatccgactctacttttcctaaaaattccagcgcaaaatgccataagcaccacattcccttatactggagatcctccatacagccatggaacaggaacaggatacaccatggacacagtaaacagaacacaccaatactcagaaaagggaaagtggacgacaaatacagagactggtgcaccccagctcaacccgattgatggaccactacctgaggataatgaaccaagtgggtatgcacaaacagactgtgttctagaggctatggctttccttgaagaatcccacccaggaatatttgagaattcatgccttgaaacaatggaagttgttcaacaaacaagggtagataaactaactcaaggtcgccagacttatgattggacattaaacagaaatcaaccggcagcaactgcgttggccaacaccatagaagtctttagatcgaatggcctaacagctaatgagtcaggaaggctaatagatttcttaaaggatgtaatggaatcaatgaacaaagaggaaatagagataacaacccactttcaaagaaaaaggagagtaagagacaacatgaccaagaagatggtcacgcaaagaacaatagggaagaaaaaacaaagactgaataagagaggctatctaataagagcactgacattaaatacgatgaccaaagatgcagagagaggcaagttaaaaagaagggctatcgcaacacctgggatgcagattagaggtttcgtatactttgttgaaactttagctaggagcatttgcgaaaagcttgaacagtctgggctcccagtagggggcaatgaaaagaaggccaaactggcaaatgttgtgagaaagatgatgactaattcacaagacacagagatttctttcacaatcactggggacaacactaagtggaatgaaaatcaaaatcctcgaatgttcctggcgatgattacatatatcaccagaaatcaacccgagtggttcagaaacatcctgagcatggcacccataatgttctcaaacaaaatggcaagactagggaaagggtacatgttcgagagtaaaagaatgaagattcgaacacaaataccagcagaaatgctagcaagcattgacctgaagtacttcaatgaatcaacaaagaagaaaattgagaaaataaggcctcttctaatagatggcacagcatcactgagtcctgggatgatgatgggcatgttcaacatgctaagtacggtcttgggagtctcgatactgaatcttggacaaaagaaatacaccaagacaatatactggtgggatgggctccaatcatccgacgattttgctctcatagtgaatgcaccaaaccatgagggaatacaagcaggagtggacagattctacaggacctgcaagttagtgggaatcaacatgagcaaaaagaagtcctatataaataagacagggacatttgaattcacaagctttttttatcgctatggatttgtggctaattttagcatggagctacccagctttggagtgtctggagtaaatgaatcagctgacatgagtattggagtaacagtgataaagaacaacatgataaacaatgaccttggacctgcaacggcccagatggctcttcaactgttcatcaaagactacagatacacatataggtgccataggggagacacacaaattcagacgagaagatcatttgagttaaagaagctgtgggatcaaacccaatcaaaggtagggctattagtatcagatggaggaccaaacttatacaatatacggaatcttcacattcctgaagtctgcttaaaatgggagctaatggatgatgattatcggggaagactttgtaatcccctgaatccctttgtcagtcataaagagattgattctgtaaacaatgctgtggtaatgccagcccatggtccagccaaaagcatggaatatgatgccgttgcaactacacattcctggattcccaagaggaatcgttccattctcaacacaagccaaaggggaattcttgaggatgaacagatgtaccagaagtgctgcaatctattcgagaaatttttccctagcagttcatataggagaccggttggaatttctagcatggtggaggccatggtgtctagggcccggattgatgccagggtcgacttcgagtctggacggatcgggaaggaagagttctctgagatcatgaagatctgttccaccattgaagaactcagacggcaaaaataa

>H1N1_PB1_HM568142_A/England/H093780034/2009

atggatgtcaatccgactctacttttcctaaaaattccagcgcaaaatgccataagcaccacattcccttatactggagatcctccatacagccatggaacaggaacaggatacaccatggacacagtaaacagaacacaccaatactcagaaaagggaaagtggacgacaaacacagagactggtgcaccccagctcaacccgattgatggaccactacctgaggataatgaaccaagtgggtatgcacaaacagactgtgttctagaggctatggctttccttgaagaatcccacccaggaatatttgagaattcatgccttgaaacaatggaagttgttcaacaaacaagggtagataaactaactcaaggtcgccagacttatgattggacattaaacagaaatcaaccggcagcaactgcattggccaacaccatagaagtctttagatcgaatggcctaacagctaatgagtcaggaaggctaatagatttcttaaaggatgtaatggaatcaatgaacaaagaggaaatagagataacaacccactttcaaagaaaaaggagagtaagagacaacatgaccaagaagatggtcacgcaaagaacaatagggaagaaaaaacaaagactgaataagagaggctatctaataagagcactgacattaaatacgatgaccaaagatgcagagagaggcaagttaaaaagaagggctatcgcaacacctgggatgcagattagaggtttcgtatactttgttgaaactttagctaggagcatttgcgaaaagcttgaacagtctgggctcccagtagggggcaatgaaaagaaggccaaactggcaaatgttgtgagaaagatgatgactaattcacaagacacagagatttctttcacaatcactggggacaacactaagtggaatgaaaatcaaaatcctcgaatgttcctggcgatgattacatatatcaccagaaatcaacccgagtggttcagaaacatcctgagcatggcacccataatgttctcaaacaaaatggcaagactagggaaagggtacatgttcgagagtaaaagaatgaagattcgaacacaaataccagcagaaatgctagcaagcattgacctgaagtacttcaatgaatcaacaaagaagaaaattgagaaaataaggcctcttctaatagatggcacagcatcactgagtcctgggatgatgatgggcatgttcaacatgctaagtacggtcttgggagtctcgatactgaatcttggacaaaagaaatacaccaagacaatatactggtgggatgggctccaatcatccgacgattttgctctcatagtgaatgcaccaaaccatgagggaatacaagcaggagtggacagattctacaggacctgcaagttagtgggaatcaacatgagcaaaaagaagtcctatataaataagacagggacatttgaattcacaagctttttttatcgctatggatttgtggctaattttagcatggagctacccagctttggagtgtctggagtaaatgaatcagctgacatgagtattggagtaacagtgataaagaacaacatgataaacaatgaccttggacctgcaacggcccagatggctcttcaattgttcatcaaagactacagatacacatataggtgccataggggagacacacaaattcagacgagaagatcatttgagttaaagaagctgtgggatcaaacccaatcaaaggtagggctattagtatcagatggaggaccaaacttatacaatatacgaaatcttcacattcctgaagtctgcttaaaatgggagctaatggatgatgattatcggggaagactttgtaatcccctgaatccctttgtcagtcataaagagattgattctgtaaacaatgctgtggtaatgccagcccatggtccagccaaaagcatggaatatgatgccgttgcaactacacattcctggattcccaagaggaatcgttctattctcaacacaagccaaaggggaattcttgaggatgaacagatgtaccagaagtgctgcaatctattcgagaaatttttccctagcagttcatataggagaccggttggaatttctagcatggtggaggccatggtgtctagggcccggattgatgccagggtcgacttcgagtctggacggatcaagaaagaagagttctctgagatcatgaagatctgttccaccattgaaaaactcagacggcaaaaataa

>H1N1_PB1_CY065910_A/Netherlands/1083/2009

atggatgtcaatccgactctacttttcctaaaaattccagcgcaaaatgccataagcaccacattcccttatactggagatcctccatacagccatggaacaggaacaggatacaccatggacacagtaaacagaacacaccaatactcagaaaagggaaagtggacgacaaacacagagactggtgcaccccagctcaacccgattgatggaccactacctgaggataatgaaccaagtgggtatgcacaaacagactgtgttctagaggctatggctttccttgaagaatcccacccaggaatatttgagaattcatgccttgaaacaatggaagttgttcaacaaacaagggtagataaactaactcaaggtcgccagacttatgattggacattaaacagaaatcaaccggcagcaactgcattggccaacaccatagaagtctttagatcgaatggcctaacagctaatgagtcaggaaggctaatagatttcttaaaggatgtaatggaatcaatgaacaaagaggaaatagagataacaacccactttcaaagaaaaaggagagtaagagacaacatgaccaagaagatggtcacgcaaagaacaatagggaagaaaaaacaaagactgaataagagaggctatctaataagagcactgacattaaatacgatgaccaaagatgcagagagaggcaagttaaaaagaagggctatcgcaacacctgggatgcagattagaggtttcgtatactttgttgaaactttagctaggagcatttgcgaaaagcttgaacagtctgggctcccagtagggggcaatgaaaagaaggccaaactggcaaatgttgtgagaaagatgatgactaattcacaagacacagagatttctttcacaatcactggggacaacactaagtggaatgaaaatcaaaatcctcgaatgttcctggcgatgattacatatatcaccagaaatcaacccgagtggttcagaaacatcctgagcatggcacccataatgttctcaaacaaaatggcaagactagggaaagggtacatgttcgagagtaaaagaatgaagattcgaacacaaataccagcagaaatgctagcaagcattgacctgaagtacttcaatgaatcaacaaagaagaaaattgagaaaataaggcctcttctaatagatggcacagcatcactgagtcctgggatgatgatgggcatgttcaacatgctaagtacggtcttgggagtctcgatactgaatcttggacaaaagaaatacaccaagacaatatactggtgggatgggctccaatcatccgacgattttgctctcatagtgaatgcaccaaaccatgagggaatacaagcaggagtggacagattctacaggacctgcaagttagtgggaatcaacatgagcaaaaagaagtcctatataaataagacagggacatttgaattcacaagctttttttatcgctatggatttgtggctaattttagcatggagctacccagctttggagtgtctggagtaaatgaatcagctgacatgagtattggagtaacagtgataaagaacaatatgataaacaatgaccttggacctgcaacggcccagatggctcttcaattgttcatcaaagactacagatacacatataggtgccataggggagacacacaaattcagacgagaagatcatttgagttaaagaagctgtgggatcaaacccaatcaaaggtagggctattagtatcagatggaggaccaaacttatacaatatacggaatcttcacattcctgaagtctgcttaaaatgggagctaatggatgatgattatcggggaagactttgtaatcccctgaatccctttgtcagtcataaagagattgattctgtaaacaatgctgtggtaatgccagcccatggtccagccaaaagcatggaatatgatgccgttgcaactacacattcctggattcccaaaaggaatcgttctattctcaacacaagccaaaggggaattcttgaggatgaacagatgtaccagaagtgctgcaatctattcgagaaatttttccctagcagttcatataggagaccggttggaatttctagcatggtggaggccatggtgtctagggcccggattgatgccagggtcgacttcgagtctggacggatcaagaaagaagagttctctgagatcatgaagatctgttccaccattgaagaactcagacggcaaaaataa

>H1N1_PB1_CY066373_A/California/VRDL124/2009

atggatgtcaatccgactctacttttcctaaaaattccagcgcaaaatgccataagcaccacattcccttatactggagatcctccatacagccatggaacaggaacaggatacaccatggacacagtaaacagaacacaccaatactcagaaaagggaaagtggacgacaaacacagagactggtgcaccccagctcaacccgattgatggaccactacctgaggataatgaaccaagtgggtatgcacaaacagactgtgttctagaggctatggctttccttgaagaatcccacccaggaatatttgagaattcatgccttgaaacaatggaagttgttcaacaaacaagggtagataaactaactcaaggtcgccagacttatgattggacattaaacagaaatcaaccggcagcaactgcattggccaacaccatagaagtctttagatcgaatggcctaacagctaatgagtcaggaaggctaatagatttcttaaaggatgtaatggaatcaatgaacaaagaggaaatagagataacaacccactttcaaagaaaaaggagagtaagagacaacatgaccaagaagatggtcacgcaaagaacaatagggaagaaaaaacaaagactgaataagagaggctatctaataagagcactgacattaaatacgatgaccaaagatgcagagagaggcaagttaaaaagaagggctatcgcaacacctgggatgcagattagaggtttcgtatactttgttgaaactttagctaggagcatttgcgaaaagcttgaacagtctgggctcccagtagggggcaatgaaaagaaggccaaactggcaaatgttgtgagaaagatgatgactaattcacaagacacagagatttctttcacaatcactggggacaacactaagtggaatgaaaatcaaaatcctcgaatgttcctggcgatgattacatatatcaccagaaatcaacccgagtggttcagaaacatcttgagcatggcacccataatgttctcaaacaaaatggcaagactagggaaagggtacatgttcgagagtaaaagaatgaagattcggacacaaataccagcagaaatgctagcaagcattgacctgaagtacttcaatgaatcaacaaagaagaaaattgagaaaataaggcctcttctaatagatggcacagcatcactgagtcctgggatgatgatgggcatgttcaacatgctaagtacggtcttgggagtctcgatactgaatcttggacaaaagaaatacaccaagacaatatactggtgggatgggctccaatcatccgacgattttgctctcatagtgaatgcaccaaaccatgagggaatacaagcaggagtggacagattctacaggacctgcaagttagtgggaatcaacatgagcaaaaagaagtcctatataaataagacagggacatttgaattcacaagctttttttatcgctatggatttgtggctaattttagcatggagctacccagctttggagtgtctggagtaaatgaatcagctgacatgagtattggagtaacagtgataaagaacaacatgataaacaatgaccttggacctgcaacggcccagatggctcttcaactgttcatcaaagactacagatacacatataggtgccataggggagacacacaaattcagacgagaagatcatttgagttaaagaagctgtgggatcaaacccaatcaaagatagggctattagtatcagatggaggaccaaacttatacaatatacggaatcttcacattcctgaagtctgcttaaaatgggagctaatggatgatgattatcggggaagactttgtaatcccctgaatccctttgtcagtcataaagagattgattctgtaaacaatgctgtggtaatgccagcccatggtccagccaaaagcatggaatatgatgccgttgcaaccacacattcctggattcccaagaggaatcgttctattctcaacacaagccaaaggggaattcttgaggatgaacagatgtaccagaagtgctgcaatctattcgagaaatttttccctagcagttcatataggagaccggttggaatttctagcatggtggaggccatggtgtctagggcccggattgatgccagggtcgacttcgagtctggacggatcgggaaggaagagttctctgagatcatgaagatctgctccaccattgaagaactcagacggcaaaaataa

>H1N1_PB1_CY066981_A/Aarhus/INS242/2009

atggatgtcaatccgactctacttttcctaaaaattccagcgcaaaatgccataagcaccacattcccttatactggagatcctccatacagccatggaacaggaacaggatacaccatggacacagtaaacagaacacaccaatactcagaaaagggaaagtggacgacaaacacagagactggtgcaccccagctcaacccgattgatggaccactacctgaggataatgaaccaagtgggtatgcacaaacagactgtgttctagaggctatggctttccttgaagaaacccacccaggaatatttgagaattcatgccttgaaacaatggaagttgttcaacaaacaagggtagataaactaactcaaggtcgccagacttatgattggacattaaacagaaatcaaccggcagcaactgcattggccaacaccatagaagtctttagatcgaatggcctaacagctaatgagtcaggaaggctaatagatttcttaaaggatgtaatggaatcaatgaacaaagaggaaatagagataacaacccactttcaaagaaaaaggagagtaagagacaacatgaccaagaagatggtcacgcaaagaacaatagggaagaaaaaacaaagactgaataagagaggctatctaataagagcactgacattaaatacgatgaccaaagatgcagagagaggcaagttaaaaagaagggctatcgcaacacctgggatgcagattagaggtttcgtatactttgttgaaactttagctaggagcatttgcgaaaagcttgaacagtctgggctcccagtagggggcaatgaaaagaaggccaaactggcaaatgttgtgagaaagatgatgactaattcacaagacacagagatttctttcacaatcactggggacaacactaagtggaatgaaaatcaaaatcctcgaatgttcctggcgatgattacatatatcaccagaaatcaacccgagtggttcagaaacatcctgagcatggcacccataatgttctcaaacaaaatggcaagactagggaaagggtacatgttcgagagtaaaagaatgaagattcgaacacaaataccagcagaaatgctagcaagcattgacctgaagtacttcaatgaatcaacaaagaagaaaattgagaaaataaggcctcttctaatagatggcacagcatcactgagtcctgggatgatgatgggcatgttcaacatgctaagtacggtattgggagtctcgatactgaatcttggacaaaagaaatacaccaagacaatatactggtgggatgggctccaatcatccgacgattttgctctcatagtgaatgcaccaaaccatgagggtatacaagcaggagtggacagattctacaggacctgcaagttagtgggaatcaacatgagcaaaaagaagtcctatataaataagacagggacatttgaattcacaagctttttttatcgctatggatttgtggctaattttagcatggagctacccagctttggagtgtctggagtaaatgaatcagctgacatgagtattggagtaacagtgataaagaacaacatgataaacaatgaccttggacctgcaacggcccagatggctcttcaattgttcatcaaagactacagatacacatataggtgccataggggagacacacaaattcagacgagaagatcatttgagttaaagaagctgtgggatcaaacccaatcaaaggtagggctattagtatcagatggaggaccaaacttatacaatatacggaatcttcacattcctgaagtctgcttaaaatgggagctaatggatgatgattatcggggaagactttgtaatcccctgaatccctttgtcagtcataaagagattgattctgtaaacaatgctgtggtaatgccagcccatggtccagccaaaagcatggaatatgatgccgttgcaactacacattcctggattcccaagaggaatcgttctattctcaacacaagccaaaggggaattcttgaggatgaacagatgtaccagaagtgctgcaatctattcgagaaatttttccctagcagttcatataggagaccggttggaatttctagcatggtggaggccatggtgtctagggcccggattgatgccagggtcgacttcgagtctggacggatcaagaaagaagagttctctgagatcatgaagatctgttccaccattgaagaactcagacggcaaaaataa

>H1N1_PB1_CY067037_A/Aarhus/INS251/2009

atggatgtcaatccgactctacttttcctaaaaattccagcgcaaaatgccataagcaccacattcccttatactggagatcctccatacagccatggaacaggaacaggatacaccatggacacagtaaacagaacacaccaatactcagaaaagggaaagtggacgacaaacacagagactggtgcaccccagctcaacccgattgatggaccactacctgaggataatgaaccaagtgggtatgcacaaacagactgtgttctagaggctatggctttccttgaagaatcccacccaggaatatttgagaattcatgccttgaaacaatggaagttgttcaacaaacaagggtagataaactaactcaaggtcgccagacttatgattggacattaaacagaaatcaaccggcagcaactgcattggccaacaccatagaagtctttagatcgaatggcctaacagctaatgagtcaggaaggctaatagatttcttaaaggatgtaatggaatcaatgaacaaagaggaaatagagataacaacccactttcaaagaaaaaggagagtaagagacaacatgaccaagaagatggtcacgcaaagaacaatagggaagaaaaaacaaagactgaacaagagaggctatctaataagagcactgacattaaatacgatgaccaaagatgcagagagaggcaagttaaaaagaagggctatcgcaacacctgggatgcagattagaggtttcgtatactttgttgaaactttagctaggagcatttgcgaaaagcttgaacagtctgggctcccagtagggggcaatgaaaagaaggccaaactggcaaatgttgtgagaaagatgatgactaattcacaagacacagagatttctttcacaatcactggggacaacactaagtggaatgaaaatcaaaatcctcgaatgttcctggcgatgattacatatatcaccagaaatcaacccgagtggttcagaaacatcctgagcatggcacccataatgttctcaaacaaaatggcaagactagggaaagggtacatgttcgagagtaaaagaatgaagattcgaacacaaataccagcagaaatgctagcaagcattgacctgaagtacttcaatgaatcaacaaagaagaaaattgagaaaataaggcctcttctaatagatggcacagcatcactgagtcctgggatgatgatgggcatgttcaacatgctaagtacggtcttgggagtctcgatactgaatcttggacaaaagaaatacaccaagacaatatactggtgggatgggctccaatcatccgacgattttgctctcatagtgaatgcaccaaaccatgagggaatacaagcaggagtggacagattctacaggacctgcaagttagtgggaatcaacatgagcaaaaagaagtcctatataaataagacagggacatttgaattcacaagctttttttatcgctatggatttgtggctaattttagcatggagctacccagctttggagtgtctggagtaaatgaatcagctgacatgagtattggagtaacagtgataaagaacaacatgataaacaatgaccttggacctgcaacggcccagatggctcttcaattgttcatcaaagactacagatacacatataggtgccataggggagacacacaaattcagacgagaagatcatttgagttaaagaagctgtgggatcaaacccaatcaaaggtagggctattagtatcagatggaggaccaaacttatacaatatacggaatcttcacattcctgaagtctgcttaaaatgggagctaatggatgatgattatcggggaagactttgtaatcccctgaatccctttgtcagtcataaagagattgattctgtaaacaatgctgtggtaatgccagcccatggtccagccaaragcatggaatatgatgccgttgcaactacacattcctggattcccaagaggaatcgttctattctcaacacaagccaaaggggaattcttgaggatgaacagatgtaccagaagtgctgcaatctattcgagaaatttttccctagcagttcatataggagaccggttggaatttctagcatggtggaggccatggtgtctagggcccggattgatgccagggtcgacttcgagtctggacggatcaagaaagaagagttctctgagatcatgaagatctgttccaccattgaagaactcagacggcaaaaataa

>H1N1_PB1_HQ011416_A/Guangdong/45/2009

atggatgtcaatccgactctacttttcctaaaaattccagcgcaaaatgccataagcaccacattcccttatactggagatcctccatacagccatggaacaggaacaggatacaccatggacacagtaaacagaacacaccaatactcagaaaagggaaagtggacgacaaacacagagactggtgcaccccagctcaacccgattgatggaccactacctgaggataatgaaccaagtgggtatgcacaaacagactgtgttctagaggctatggctttccttgaagaatcccacccaggaatatttgagaattcatgccttgaaacaatggaagttgttcaacaaacaagggtagataaactaactcaaggtcgccagacttatgattggacattaaacagaaatcaaccggcagcaactgcattggccaacaccatagaagtctttagatcgaatggcctaacagctaatgagtcaggaaggctaatagatttcttaaaggatgtaatggaatcaatgaacaaagaggaaatagagataacaacccactttcaaagaaaaaggagagtaagagacaacatgaccaagaagatggtcacgcaaagaacaatagggaagaaaaaacaaagactgaataagagaggctatctaataagagcactgacattaaatacgatgaccaaagatgcagagagaggcaagttaaaaagaagggctatcgcaacacctgggatgcagattagaggtttcgtatactttgttgaaactttagctaggagcatttgcgaaaagcttgaacagtctgggctcccagtagggggcaatgaaaagaaggccaaactggcaaatgttgtgagaaagatgatgactaattcacaagacacagagatttctttcacaatcactggggacaacactaagtggaatgaaaatcaaaatcctcgaatgttcctggcgatgattacatatatcaccagaaatcaacccgagtggttcagaaacatcctgagcatggcacccataatgttctcaaacaaaatggcaagactagggaaagggtacatgttcgagagtaaaagaatgaagattcgaacacaaataccagcagaaatgctagcaagcattgacctgaagtacttcaatgaatcaacaaagaagaaaattgagaaaataaggcctcttctaatagatggcacagcatcactgagtcctgggatgatgatgggcatgttcaacatgctaagtacggtcttgggagtctcgatactgaatcttggacaaaagaaatacaccaagacaatatactggtgggatgggctccaatcatccgacgattttgctctcatagtgaatgcaccaaaccatgagggaatacaagcaggagtggacagattctacaggacctgcaagttagtgggaatcaacatgagcaaaaagaagtcctatataaataagacagggacatttgaattcacaagctttttttatcgctatggatttgtggctaattttagcatggagctacccagctttggagtgtctggagtaaatgaatcagctgacatgagtattggagtaacagtgataaagaacaacatgataaacaatgaccttggacctgcaacggcccagatggctcttcaattgttcatcaaagactacagatacacatataggtgccataggggagacacacaaattcagacgagaagatcatttgagttaaagaagctgtgggatcaaacccaatcaaaggtagggctattagtatcagatggaggaccaaacttatacaatatacggaatcttcacattcctgaagtctgcttaaaatgggagctaatggatgatgattatcggggaagactttgtaatcccctgaatccctttgtcagtcataaagagattgattctgtaaacaatgctgtggtaatgccagcccatggtccagccaaaagcatggaatatgatgccgttgcaactacacattcctggattcccaagaggaatcgttctattctcaacacaagccaaaggggaattcttgaggatgaacagatgtaccagaagtgctgcaatctattcgagaaatttttccctagcagttcatataggagaccggttggaatttctagcatggtggaggccatggtgtctagggcccggattgatgccagggtcgacttcgagtctggacggatcaagaaagaagagttctctgagatcatgaagatctgttccaccattgaagaactcagacggcaaaaataa

>H1N1_PB1_CY069379_A/Alaska/WRAIR1114P/2009

atggatgtcaatccgacattacttttcttaaaagtgccagcacaaaatgctataagcacaacttttccttatactggtgaccctccttacagccatgggacaggaacagggtacaccatggatacagtcaataggacacaccagtactcagaaagaggaagatggacaaaaaataccgaaacgggagcaccgcaacttaacccaattgatggtcccttaccggaagacaatgaaccaagtggctatgcccaaacagattgtgtattagaggcaatggctttccttgaagaatcccatcccggtatctttgaaaactcttgtattgaaacaatggaggttgttcaacaaacaagggtggacaaactgacacaaggcagacagacctatgactggactctaaataggaaccagcctgctgccacagcattggcaaacactatagaagtattcagatcaaacggcctcatagcaaatgaatctggaaggctaatagacttccttaaagatgtaatggagtcgatggacagaggcgaagtagaggtcacaactcattttcaaagaaagaggagagtgagagacaatgtaactaaaaaaatggtgacccaaagaacaataggcaaaaagaaacataaattagacaaaagaagttacctaattagggcattaaccctgaacacaatgaccaaagatgctgagagggggaaactaaaacgcagagcaattgcaaccccaggaatgcaaataagggggtttgtatactttgttgagacactggcaagaagcatatgtgaaaagcttgaacaatcaggattgccagttggaggaaatgagaagaaagcaaagttagcaaatgttgtaaggaagatgatgaccaactcccaggacactgaaatttctttcaccataaccggagacaacacaaaatggaacgaaaatcaaaaccctagaatgttcttggccatgatcacatatataaccaaaaatcagcctgaatggttcagaaatattctaagtattgctccaataatgttttcaaacaagatggcgagactaggtaaggggtacatgtttgaaagcaagagtatgaaactgagaactcaaatacctgcagagatgctagccaacatagacttgaaatatttcaatgattcaactaaaaagaaaattgaaaaaatccgaccattattaatagatggaactgcatcattgagtcctggaatgatgatgggcatgttcaatatgttgagcaccgtcttgggcgtctccattctgaatcttgggcaaaagagatacaccaagactacttactggtgggatggtcttcaatcgtctgatgattttgctttgattgtgaacgcacccaactatgcaggaattcaagctggagttgacaggttctatcgaacctgtaagctgctcggaattaatatgagcaaaaagaagtcttacataaacagaacaggtacctttgaattcacgagctttttctatcgttatgggtttgttgccaatttcagcatggagcttcctagttttggggtgtctggggtcaatgaatctgcagacatgagtattggagtcactgtcatcaaaaacaatatgataaacaatgaccttggcccagcaactgctcaaatggcccttcagttatttataaaagattacaggtacacttatcgatgccaccgaggtgacacacaaatacaaacccgaagatcatttgagataaagaaactatgggaccaaacccgctcaaaagctggactgttggtctctgatggaggccccaatttgtataacattagaaatctccatattcctgaagtttgcttgaaatgggagttgatggatgaggattaccaggggcgtttatgcaacccattaaacccgtttgtcagccataaagagattgaatcagtgaacagtgcagtgataatgccggcacatggtccagccaaaaatatggagtatgacgctgttgcaacaacacactcctgggtccccaaaagaaatcgatccattttgaacacgagccaaagggggatacttgaagatgagcaaatgtatcagaggtgctgcaatttatttgaaaaattcttcccaagtagctcatacagaagaccagttggaatatccagtatggtagaggctatggtctcaagagcccgaattgatgcacggattgatttcgaatctggaaggataaagaaagaggaatttgctgagatcatgaagatctgttccaccattgaagacctcagacggcaaaaatga

>H1N1_PB1_CY071141_A/Athens/INS389/2010

atggatgtcaatccgactctacttttcctaaaaattccagcgcaaaatgccataagcaccacattcccttatactggagatcctccatacagccatggaacaggaacaggatacaccatggacacagtaaacagaacacaccaatactcagaaaagggaaagtggacgacaaacacagagactggtgcaccccagctcaacccgattgatggaccactacctgaggataatgaaccaagtgggtatgcacaaacagactgtgttctagaggctatggctttccttgaagaatcccacccaggaatatttgagaattcatgccttgaaacaatggaagttgttcaacaaacaagggtagataaactaactcaaggtcgccagacttatgattggacattaaacagaaatcaaccggcagcaactgcattggccaacaccatagaagtctttagatcgaatggcctaacagctaatgagtcaggaaggctaatagatttcttaaaggatgtaatggaatcaatgaacaaagaggaaatagagataacaacccactttcaaagaaaaaggagagtaagagacaacatgaccaagaagatggtcacgcaaagaacaatagggaagaaaaaacaaagactgaataagagaggctatctaataagagcactgacattaaatacgatgaccaaagatgcagagagaggcaagttaaaaagaagggctatcgcaacacctgggatgcagattagaggtttcgtatactttgttgaaactttagctaggagcatttgcgaaaagcttgaacaatctgggctcccagtagggggcaatgaaaagaaggccaaactggcaaatgttgtgagaaagatgatgactaattcacaagacacagagatttctttcacaatcactggggacaacactaagtggaatgaaaatcaaaatcctcgaatgttcctggcgatgattacatatatcaccagaaatcaacccgagtggttcagaaacatcctgagcatggcacccataatgttctcaaacaaaatggcaagactagggaaagggtacatgttcgagagtaaaagaatgaagattcgaacacaaataccagcagaaatgctagcaagcattgacctgaagtacttcaatgaatcaacaaagaagaaaattgagaaagtaaggcctcttctaatagatggcacagcatcactgagtcctgggatgatgatgggcatgttcaacatgctaagtacggtcttgggagtctcgatactgaatcttggacaaaagaaatacaccaagacaatatactggtgggatgggctccaatcatccgacgattttgctctcatagtgaatgcaccaaaccatgagggaatacaagcaggagtggacagattctacaggacctgcaagttagtgggaatcaacatgagcaaaaagaagtcctatataaataagacagggacatttgaattcacaagctttttttatcgctatggatttgtggctaattttagcatggagctacccagctttggagtgtctggagtaaatgaatcagctgacatgagtattggagtaacagtgataaagaacaacatgataaacaatgaccttggacctgcaacggcccagatggctcttcaattgttcatcaaagactacagatacacatataggtgccataggggagacacacaaattcagacgagaagatcatttgagttaaagaagctgtgggatcaaacccaatcaaaggtagggctattagtatcagatggaggaccaaacttatacaatatacggaatcttcacattcctgaagtctgcttaaaatgggagctaatggatgatgattatcggggaagactttgtaatcccctgaatccctttgtcagtcataaagagattgattctgtaaacaatgctgtggcaatgccagcccatggtccagccaaaagcatggaatatgatgccgttgcaactacacattcctggattcccaagaggaatcgttctattctcaacacaagccaaaggggaattcttgaggatgaacagatgtaccagaagtgctgcaatctattcgagaaatttttccctagcagttcatataggagaccggttggaatttctagcatggtggaggccatggtgtctagggcccggattgatgccagggtcgacttcgagtctggacggatcaagaaagaagagttctctgagatcatgaagatctgttccaccattgaagaactcagacggcaaaaataa

>H1N1_PB1_CY071149_A/Athens/INS390/2010

atggatgtcaatccgactctacttttcctaaaaattccagcgcaaaatgccataagcaccacattcccttatactggagatcctccatacagccatggaacaggaacaggatacaccatggacacagtaaacagaacacaccaatactcagaaaagggaaagtggacgacaaacacagagactggtgcaccccagctcaacccgattgatggaccactacctgaggataatgaaccaagtgggtatgcacaaacagactgtgttctagaggctatggctttccttgaagaatcccacccaggaatatttgagaattcatgccttgaaacaatggaagttgttcaacaaacaagggtagataaactaactcaaggtcgccagacttatgattggacattaaacagaaatcaaccggcagcaactgcattggccaacaccatagaagtctttagatcgaatggcctaacagctaatgagtcaggaaggctaatagatttcttaaaggatgtaatggaatcaatgaacaaagaggaaatagagataacaacccactttcaaagaaaaaggagagtaagagacaacatgaccaagaagatggtcacgcaaagaacaatagggaagaaaaaacaaagactgaataagagaggctatctaataagagcactgacattaaatacgatgaccaaagatgctgagagaggcaagttaaaaagaagggctatcgcaacacctgggatgcagattagaggtttcgtatactttgtcgaaactttagctaggagcatttgcgaaaagcttgaacaatctgggctcccagtagggggcaatgaaaagaaggccaaactggcaaatgttgtgagaaagatgatgactaattcacaagacacagagatttctttcacaatcactggggacaacactaagtggaatgaaaatcaaaatcctcgaatgttcctggcgatgattacatatatcaccagaaatcaacccgagtggttcagaaacatcctgagcatggcacccataatgttctcaaacaaaatggcaagactagggaaagggtacatgttcgagagtaaaagaatgaagattcgaacacaaataccagcagaaatgctagcaagcattgacctgaagtacttcaatgaatcaacaaagaagaaaattgagaaaataaggcctcttctaatagatggcacagcatcactgagtcctgggatgatgatgggcatgttcaacatgctaagtacggtcttgggagtctcgatactgaatcttggacaaaagaaatacaccaagacaatatactggtgggatgggctccaatcatccgacgattttgctctcatagtgaatgcaccaaaccatgagggaatacaagcaggagtggacagattctacaggacctgcaagttagtgggaatcaacatgagcaaaaagaagtcctatataaataagacagggacatttgaattcacaagctttttttatcgctatggatttgtggctaattttagcatggagctacccagctttggagtgtctggagtaaatgaatcagctgacatgagtattggagtaacagtgataaagaacaacatgataaacaatgaccttggacctgcaacggcccagatggctcttcaattgttcatcaaagactacagatacacatataggtgccataggggagacacacaaattcagacgagaagatcatttgagttaaagaagctgtgggatcaaacccaatcaaaggtagggctattagtatcagatggaggaccaaacttatacaatatacggaatcttcacattcctgaagtctgcttaaaatgggagctaatggatgatgattatcggggaagactttgtaatcccctgaatccctttgtcagtcataaagagattgattccgtaaacaatgctgtggtaatgccagcccatggtccagccaaaagcatggaatatgatgccgttgcaactacacattcctggattcccaagaggaatcgttctattctcaacacaagccaaaggggaattcttgaggatgaacagatgtaccagaagtgctgcaatctattcgagaaatttttccctagcagttcatataggagaccggttggaatttctagcatggtggaggccatggtgtctagggcccggattgatgccagggtcgacttcgagtctggacggatcaagaaagaagagttctctgagatcatgaagatctgttccaccattgaagaactcagacggcaaaaataa

>H1N1_PB1_CY071349_A/Bangkok/INS426/2010

atggatgtcaatccgactctacttttcctaaaaattccagcgcaaaatgccataagcaccacattcccttatactggggatcctccatacagccatggaacaggaacaggatacaccatggacacagtaaacagaacacaccaatactcagaaaagggaaagtggacgacaaacacagagactggtgcaccccagctcaacccgattgatggaccactacctgaggataatgaaccaagtgggtatgcacaaacagactgtgttctagaggctatggctttccttgaagaatcccacccaggaatatttgagaattcatgccttgaaacaatggaagttgttcaacaaacaagggtagataaactaactcaaggtcgccagacttatgattggaccttaaacagaaatcaaccggcagcaactgcattggccaacaccatagaagtctttagatcgaatggcctaacagctaatgagtcaggaaggctaatagatttcttaaaggatgtaatggaatcaatgaacaaagaggaaatagagataacaacccactttcaaagaaaaaggagagtaagagacaacatgaccaagaagatggtcacgcaaagaacaatagggaagaaaaaacaaagactgaataagagaggctatctaataagagcactgacattaaatacgatgaccaaggatgcagagagaggcaagttaaaaagaagggctatcgcaacacccgggatgcagattagaggtttcgtatactttgttgaaactttagctaggagcatttgcgaaaagcttgaacagtctgggctcccagtagggggcaatgaaaagaaggccaaactggcaaatgttgtgagaaagatgatgactaattcacaagacacagagatttctttcacaatcactggggacaacactaagtggaatgaaaatcaaaatcctcgaatgttcctggcgatgattacatatatcaccagaaatcaacccgagtggttcagaaacatcctgagcatggcacccataatgttctcaaacaaaatggcaagactagggaaagggtacatgttcgagagtaaaagaatgaagattcgaacacaaataccagcagaaatgctagcaagcattgacctgaagtacttcaatgaatcaacaaaggggaaaattgagaaaataaggcctcttctaatagatggcacagcatcactgagtcctgggatgatgatgggcatgttcaacatgctaagtacggtcttgggagtctcgatactgaatcttggacaaaagaaatacaccaagacagtatactggtgggatgggctccaatcatccgatgattttgctctcatagtgaatgcaccaaaccatgagggaatacaagcaggagtggacagattctacaggacctgcaagttagtgggaatcaacatgagcaaaaagaagtcctatataaataagacagggacatttgaattcacaagctttttttatcgctatggatttgtggctaattttagcatggagctacccagctttggagtgtctggagtaaatgaatcagctgacatgagtattggagtaacagtgataaagaacaacatgataaacaatgaccttggacctgcaacggcccagatggctcttcaattgttcatcaaagactacagatacacatataggtgccataggggagacacacaaattcagacgagaagatcatttgagttaaagaagctgtgggatcaaacccaatcaaaggtagggctattagtatcagatggaggaccaaacttatacaatatacggaatcttcacattcctgaagcctgcttaaaatgggagctaatggatgatgattatcggggaagactttgtaatcccctgaatccctttgtcagtcataaagagattgattctgtaaacaatgctgtggtaatgccagcccatggtccagccaaaagcatggaatatgatgccgttgcaactacacattcctggattcccaagaggaatcgttctattctcaacacaagccaaaggggaattcttgaggatgaacagatgtaccagaagtgctgcaatctattcgagaaatttttccctagcagttcatataggagaccggttggaatttctagcatggtggaggccatggtgtctagggcccggattgatgccagggtcgacttcgagtctggacggatcaagaaagaagagttctctgagatcatgaagatctgttccaccattgaagaactcagacggcaaaaataa

>H1N1_PB1_CY071608_A/Ft Carson/WR1448P/2009

atggatgtcaatccgactctacttttcctaaaaattccagcgcaaaatgccataagcaccacattcccttatactggagatcctccatacagccatggaacaggaacaggatacaccatggacacagtaaacagaacacaccaatactcagaaaagggaaagtggacgacaaacacagagactggtgcaccccagctcaacccgattgatggaccactacctgaggataatgaaccaagtgggtatgcacaaacagactgtgttctagaggctatggctttccttgaagaatcccacccaggaatatttgagaattcatgccttgaaacaatggaagttgttcaacaaacaagggtagataaactaactcaaggtcgccagacttatgattggacattaaacagaaatcaaccggcagcaactgcattggccaacaccatagaagtctttagatcgaatggcctaacagctaatgagtcaggaaggctaatagatttcttaaaggatgtaatggaatcaatgaacaaagaggaaatagagataacaacccactttcaaagaaaaaggagagtaagagacaacatgaccaagaagatggtcacgcaaagaacaatagggaagaaaaaacaaagactgaataagagaggctatctaataagagcactgacattaaatacgatgaccaaagatgcagagagaggcaagttaaaaagaagggctatcgcaacacctgggatgcagattagaggtttcgtatactttgttgaaactttagctaggagcatttgcgaaaagcttgaacagtctgggctcccagtagggggcaatgaaaagaaagccaaattggcaaatgtggtgagaaagatgatgactaattcacaagacacagagatttctttcacaatcactggggacaacactaagtggaatgaaaatcaaaatcctcgaatgttcctggcgatgattacatatatcaccagaaatcaacccgagtggttcagaaacatcctgagcatggcacccataatgttctcaaacaaaatggcaagactagggaaagggtacatgttcgagagtaaaagaatgaagattcgaacacaaataccagcagaaatgctagcaagcattgacctgaagtacttcaatgaatcaacaaagaagaaaattgagaaaataaggcctcttctaatagatggcacagcatcactgagtcctgggatgatgatgggcatgttcaacatgctaagtacggtcttgggagtctcgatactgaatcttggacaaaagaaatacaccaagacaatatactggtgggatgggctccaatcatccgacgattttgctctcatagtgaatgcaccaaaccatgagggaatacaagcaggagtggacagattctacaggacctgcaagttagtgggaatcaacatgagcaaaaagaagtcctatataaataagacagggacatttgaattcacaagctttttttatcgctatggatttgtggctaattttagcatggagctacccagctttggagtgtctggagtaaatgaatcagctgacatgagtattggagtaacagtgataaagaacaacatgataaacaatgaccttggacctgcaacggcccaaatggctcttcaattgttcatcaaagactacagatacacatataggtgccataggggggacacacaaattcagacgagaagatcatttgagttaaaggagctgtgggatcaaacccaatcaaaggtagggctattagtatcagatggaggaccaaacttatacaatatacggaatcttcacattcctgaagtctgcttaaaatgggagctaatggatgatgattatcggggaagactttgtaatcccctgaatccctttgtcagtcataaagagattgattctgtaaacaatgctgtggtaatgccagcccatggtccagccaaaagcatggaatatgatgccgttgcaactacacattcctggattcccaagaggaatcgttctattctcaacacaagccaaaggggaattcttgaggatgaacagatgtaccagaagtgctgcaatctattcgagaaatttttccctagcagttcatataggagaccggttggaatttctagcatggtggaggccatggtgtctagggcccggattgatgccagggtcgacttcgagtctggacggatcaagaaagaagagttctctgagatcatgaagatctgttccaccattgaagaactcagacggcaaaaataa

>H1N1_PB1_CY072284_A/Warsaw/INS312/2009

atggatgtcaatccgactctacttttcctaaaaattccagcgcaaaatgccataagcaccacattcccttatactggagatcctccatacagccatggaacaggaacaggatacaccatggacacagtaaacagaacacaccaatactcagaaaagggaaagtggacgacaaacacagagactggtgcaccccagctcaacccgattgatggaccactacctgaggataatgaaccaagtgggtatgcacaaacagactgtgttctagaggctatggctttccttgaggaatcccacccaggaatatttgagaattcatgccttgaaacaatggaagttgttcaacaaacaagggtagataaactaactcaaggtcgccagacttatgattggacattaaacagaaatcaaccggcagcaactgcattggccaacaccatagaagtatttagatcgaatggcctaacagctaatgagtcaggaaggctaatagatttcttaaaggatgtaatggaatcaatgaacaaagaggaaatagagataacaacccactttcaaagaaaaaggagagtaagagacaacatgaccaagaagatggtcacgcaaagaacaatagggaagaaaaaacaaagactgaataagagaggctatctaataagagcactgacattaaatacgatgaccaaagatgcagagagaggcaagttaaaaagaagggctatcgcaacacctgggatgcagattagaggtttcgtatactttgttgaaactttagctaggagcatttgcgaaaagcttgaacagtctgggctcccagtagggggcaatgaaaagaaggccaaactggcaaatgttgtgagaaagatgatgactaattcacaagacacagagatttctttcacaatcactggggacaacactaagtggaatgaaaatcaaaatcctcgaatgttcctggcgatgattacatatatcaccagaaatcaacccgagtggttcagaaacatcctgagcatggcacccataatgttctcaaacaaaatggcaagactagggaaagggtacatgttcgagagtaaaagaatgaagattcgaacacaaataccagcagaaatgctagcaagcattgacctgaagtacttcaatgaatcaacaaagaagaaaattgagaaaataaggcctcttctaatagatggcacagcatcactgagtcctgggatgatgatgggcatgttcaacatgctaagtacggtcttgggagtctcgatactgaatcttggacaaaagaaatacaccaagacaatatactggtgggatgggctccaatcatccgacgattttgctctcatagtgaatgcaccaaaccatgagggaatacaagcaggagtggacagattctacaggacctgcaagttagtgggaatcaacatgagcaaaaagaagtcctatataaataagacagggacatttgaattcacaagctttttttatcgctatggatttgtggctaattttagcatggagctacccagctttggagtgtctggagtaaatgaatcagctgacatgagtattggagtaacagtgataaagaacaacatgataaacaatgaccttggacctgcaacggcccagatggctctccaattgttcatcaaagactacagatacacatataggtgccataggggagacacacaaattcagacgagaagatcatttgagttaaagaagctgtgggatcaaacccaatcaaaggtagggctattagtatcagatggaggaccaaacttatacaatatacggaatcttcacattcctgaagtctgcttaaaatgggagctaatggatgatgattatcggggaagactttgtaatcccctgaatccctttgtcagtcataaagagattgattctgtaaacaatgctgtggtaatgccagcccatggtccagtcaaaagcatggaatatgatgccgttgcaactacacattcctggattcccaagaggaatcgttctattctcaacacaagccaaaggggaattcttgaggatgaacagatgtaccagaagtgctgcaatctatttgagaaatttttccctagcagttcatataggagaccggttggaatttctagcatggtggaggccatggtgtctagggcccggattgatgccagggtcgacttcgagtctggacggatcaagaaagaagagttctctgagatcatgaagatctgttccaccattgaagaactcagacggcaaaaataa

>H1N1_PB1_CY072316_A/Warsaw/INS316/2009

atggatgtcaatccgactctacttttcctaaaaattccagcgcaaaatgccataagcaccacattcccttatactggagatcctccatacagccatggaacaggaacaggatacaccatggacacagtaaacagaacacaccaatactcagaaaagggaaagtggacgacaaacacagagactggtgcaccccagctcaacccgattgatggaccactacctgaggataatgaaccaagtgggtatgcacaaacagactgtgttctagaggctatggctttccttgaagaatcccacccaggaatatttgagaattcatgccttgaaacaatggaagttgttcaacaaacaagggtagataaactaactcaaggtcgccagacttatgattggacattaaacagaaatcaaccggcagcaactgcattggccaacaccatagaagtctttagatcgaatggcctaacagctaatgagtcaggaaggctaatagatttcttaaaggatgtaatggaatcaatgaacaaagaggaaatagagataacaacccactttcaaagaaaaaggagagtaagagacaacatgaccaagaagatggtcacgcaaagaacaatagggaagaaaaaacaaagactgaataagagaggctatctaataagagcactgacattaaatacgatgaccaaagatgcagagagaggcaagttaaaaagaagggctatcgcaacacctgggatgcagattagaggtttcgtatactttgttgaaactttagctaggagcatttgcgaaaagcttgaacagtctgggctcccagtagggggcaatgaaaagaaggccaaactggcaaatgttgtgagaaagatgatgactaattcacaagacacagagatttctttcacaatcactggggacaacactaagtggaatgaaaatcaaaatcctcgaatgttcctggcgatgattacatatatcaccagaaatcaacccgagtggttcagaaacatcctgagcatggcacccataatgttctcaaacaaaatggcaagactagggaaagggtacatgttcgagagtaaaagaatgaagattcgaacacaaataccagcagaaatgctagcaagcattgacctgaagtacttcaatgaatcaacaaagaagaaaattgagaaaataaggcctcttctaatagatggcacagcatcactgagtcctgggatgatgatgggcatgttcaacatgctaagtacggtcttgggagtctcgatactgaatcttggacaaaagaaatacaccaaaacaacatattggtgggatgggctccaatcatccgacgattttgctctcatagtgaatgcaccaaaccatgagggaatacaagcaggagtggacagattctacaggacctgcaagttagtgggaatcaacatgagcaaaaagaagtcctatataaataagacagggacatttgaattcacaagctttttttatcgctatggatttgtggctaattttagcatggagctacccagctttggagtgtctggagtgaatgaatcagctgacatgagtattggagtaacagtgataaagaacaacatgataaacaatgaccttggacctgcaacggcccagatggctcttcaattgttcatcaaagactacagatacacatataggtgccataggggggacacacaaattcagacgagaagatcatttgagttaaagaagctgtgggatcaaacccaatcaaaggtagggctattagtatcagatggaggaccaaacttatacaatatacggaatcttcacattcctgaagtctgcttaaaatgggagctaatggatgatgattatcggggaagactttgtaatcccctgaatccctttgtcagtcataaagagattgattctgtaaacaatgctgtggtaatgccagcccatggtccagccaaaagcatggaatatgatgccgttgcaactacacattcctggattcccaagaggaatcgttctattctcaacacaagccaaaggggaattcttgaggatgaacatatgtaccagaagtgctgcaatctattcgagaaatttttccctagcagttcatataggagaccggttggaatttctagcatggtggaggccatggtgtctagggcccggattgatgccagggtcgacttcgagtctggacggatcaagaaagaagagttctctgagatcatgaagatctgttccaccattgaagaactcagacggcaaaaataa

>H1N1_PB1_CY072828_A/Managua/5115.01/2009

atggatgtcaatccgactctacttttcctaaaaattccagcgcaaaatgccataagcaccacattcccttatactggagatcctccatacagccatggaacaggaacaggatacaccatggacacagtaaacagaacacaccaatactcagaaaagggaaagtggacgacaaacacagagactggtgcaccccagctcaacccgattgatggaccactacctgaggataatgaaccaagtgggtatgcacaaacagactgtgttctagaggctatggctttccttgaagaatcccacccaggaatatttgagaattcatgccttgaaacaatggaagttgttcaacaaacaagggtagataaactaactcaaggtcgccagacttatgattggacattaaacagaaatcaaccggcagcaactgcattggccaacaccatagaagtctttagatcgaatggcctaacagctaatgagtcaggaaggctaatagatttcttaaaggatgtaatggaatcaatgaacaaagaggaaatagagataacaacccactttcaaagaaaaaggagagtaagagacaacatgaccaagaagatggtcacgcaaagaacaatagggaagaaaaaacaaagactgaataagagaggctatctaataagagcactgacattaaatacaatgaccaaagatgcagagagaggcaagttaaaaagaagggctatcgcaacacctgggatgcagattagaggtttcgtatactttgttgaaactttagctaggagcatttgcgaaaagcttgaacagtctgggctcccagtagggggcaatgaaaagaaggccaaactggcaaatgttgtgagaaagatgatgactaattcacaagacacagagatttctttcacaatcactggggacaacactaagtggaatgaaaatcaaaatcctcgaatgttcctggcgatgattacatatatcaccagaaatcaacccgagtggttcagaaacatcctgagcatggcacccataatgttctcaaacaaaatggcaagactagggaaagggtacatgttcgagagtaaaagaatgaagattcgaacacaaataccagcagaaatgctagcaagcattgacctgaagtacttcaatgaatcaacaaagaagaaaattgagaaaataaggcctcttctaatagatggcacagcatcactgagtcctgggatgatgatgggcatgttcaacatgctaagtacggtcttgggagtctcgatactgaatcttggacaaaagaaatacaccaagacaatatactggtgggatgggctccaatcatccgacgattttgctctcatagtgaatgcaccaaaccatgagggaatacaagcaggagtggacagattctacaggacctgcaagttagtgggaatcaatatgagcaaaaagaagtcctatataaataagacagggacatttgaattcacaagctttttttatcgctatggatttgtggctaattttagcatggagctacccagctttggagtgtctggagtaaatgaatcagctgacatgagtattggagtaacagtgataaagaacaacatgataaacaatgaccttggacctgcaacggcccagatggctcttcaattgttcatcaaagactacagatacacatataggtgccataggggagacacacaaattcagacgagaagatcatttgagttaaagaagctgtgggatcaaacccaatcaaaggtagggctattagtatcagatggaggaccaaacttatacaatatacggaatcttcacattcctgaagtctgcttaaaatgggagctaatggatgatgattatcggggaagactttgtaatcccctgaatccctttgtcagtcataaagagattgattctgtaaacaatgctgtggtaatgccagcccatggtccagccaaaagcatggaatatgatgccgttgcaactacacattcctggattcccaagaggaatcgttctattctcaacacaagccaaaggggaattcttgaggatgaacagatgtaccagaagtgctgcaatctattcgagaaatttttccctagcagttcatataggagaccggttggaatttctagcatggtggaggccatggtgtctagggcccggattgatgccagggtcgacttcgagtctggacggatcaagaaagaagagttctctgagatcatgaagatctgttccaccattgaagaactcagacggcaaaaataa

>H1N1_PB1_CY073559_A/Managua/2978.04/2009

atggatgtcaatccgactctacttttcctaaaaattccagcgcaaaatgccataagcaccacattcccttatactggagatcctccatacagccatggaacaggaacaggatacaccatggacacagtaaacagaacacaccaatactcagaaaagggaaagtggacgacaaacacagagactggtgcaccccagctcaacccgattgatggaccactacctgaggataatgaaccaagtgggtatgcacaaacagactgtgttctagaggctatggctttccttgaagaatcccacccgggaatatttgagaattcatgccttgaaacaatggaagttgttcaacaaacaagggtagataaactaactcaaggtcgccagacttatgattggacattaaacagaaatcaaccggcagcaactgcattggccaacaccatagaagtctttagatcgaatggcctaacagctaatgagtcaggaaggctaatagatttcttaaaggatgtaatggaatcaatgaacaaagaggaaatagagataacaacccactttcaaagaaaaaggagagtaagagacaacatgaccaagaagatggtcacgcaaagaacaatagggaagaaaaaacaaagactgaataagaaaggctatctaataagagcactgacattaaatacgatgaccaaagatgcagagagaggcaagttaaaaagaagggctatcgcaacacctgggatgcagattagaggtttcgtatactttgttgaaactttagctaggagcatttgcgaaaagcttgaacagtctgggctcccagtagggggcaatgaaaagaaggccaaactggcaaatgttgtgagaaagatgatgactaattcacaagacacagagatttctttcacaatcactggggacaacactaagtggaatgaaaatcaaaatcctcgaatgttcctggcgatgattacatatatcaccagaaatcaacccgagtggttcagaaacatcctgagcatggcacccataatgttctcaaacaaaatggcaagactagggagagggtacatgttcgagagtaaaagaatgaagattcgaacacaaataccagcagaaatgctagcaagcattgacctgaagtacttcaatgaatcaacaaagaagaaaattgagaaaataaggcctcttctaatagatggcacagcatcactgagtcctgggatgatgatgggcatgttcaacatgctaagtacggtcttgggagtctcgatactgaatctcggacaaaagaaatacaccaagacaatatactggtgggatgggctccaatcatccgacgattttgctctcatagtgaatgcaccaaaccatgagggaatacaagcaggagtggacagattctacaggacctgcaagttagtgggaatcaacatgagcaaaaagaagtcctatataaataagacagggacatttgaattcacaagctttttttatcgctatggatttgtggctaattttagcatggagctacccagctttggagtgtctggagtaaatgaatcagctgacatgagtattggagtaacagtgataaagaacaacatgataaacaatgaccttggacctgcaacggcccagatggctcttcaattgttcatcaaagactacagatacacatataggtgccataggggagacacacaaattcagacgagaagatcatttgagttaaagaagctgtggaatcaaacccaatcaaaggtagggctattagtatcagatggaggaccaaacttatacaatatacggaatcttcacattcctgaagtctgcttaaaatgggagctaatggatgatgattatcggggaagactttgtaatcccctgaatccctttgtcagtcataaagagattgattctgtaaacaatgctgtggtaatgccagcccatggtccagccaaaagcatggaatatgatgccgttgcaactacacattcctggattcccaagaggaatcgttctattctcaacacaagccaaaggggaattcttgaggatgaacagatgtaccagaagtgctgcaatctattcgagaaatttttccctagcagttcatataggagaccggttggaatttctagcatggtggaggccatggtgtctagggcccggattgatgccagggtcgactttgagtctggacggatcaagaaagaagagttctctgagatcatgaagatctgttccaccattgaagaactcagacggcaaaaataa

>H1N1_PB1_CY073599_A/Managua/5295.02/2009

atggatgtcaatccgactctacttttcctaaaaattccagcgcaaaatgccataagcaccacattcccttatactggagatcctccatacagccatggaacaggaacaggatacaccatggacacagtaaacagaacacaccaatactcagaaaagggaaagtggacgacaaacacagagactggtgcaccccagctcaacccgattgatggaccactacctgaggataatgaaccaagtgggtatgcacaaacagactgtgttctagaggctatggctttccttgaagaatcccacccaggaatatttgagaattcatgccttgaaacaatggaagttgttcaacaaacaagggtagataaactaactcaaggtcgccagacttatgattggacattaaacagaaatcaaccggcagcaactgcattggccaacaccatagaagtctttagatcgaatggcctaacagctaatgagtcaggaaggctaatagatttcttaaaggatgtaatggaatcaatgaacaaagaggaaatagagataacaacccactttcaaagaaaaaggagagtaagagacaacatgaccaagaagatggtcacgcaaagaacaatagggaagaaaaaacaaagactgaataagagaggctatctaataagagcactgacattaaatacgatgaccaaagatgcagagagaggcaagttaaaaagaagggctatcgcaacacctgggatgcagattagaggtttcgtatactttgttgaaactttagctaggagcatttgcgaaaagcttgaacagtctgggctcccagtagggggcaatgaaaagaaggccaaactggcaaatgttgtgagaaagatgatgactaattcacaagacacagagatttctttcacaatcactggggacaacactaagtggaatgaaaatcaaaatcctcgaatgttcctggcgatgattacatatatcaccagaaatcaacccgagtggttcagaaacatcctgagcatggcacccataatgttctcaaacaaaatggcaagactagggaaagggtacatgttcgagagtaaaagaatgaagattcgaacacaaataccagcagaaatgctagcaagcattgacctgaagtacttcaatgaatcaacaaagaagaaaattgagaaaataaggcctcttctaatagatggcacagcatcactgagtcctgggatgatgatgggcatgttcaacatgctaagtacggtcttgggagtctcgatactgaatcttggacaaaagaaatacaccaagacaatatactggtgggatgggctccaatcatccgacgattttgctctcatagtgaatgcaccaaaccatgagggaatacaagcaggagtggacagattctacaggacctgcaagttagtgggaatcaacatgagcaaaaagaagtcctatataaataagacagggacatttgaattcacaagctttttttatcgctatggatttgtggctaattttagcatggagctacccagctttggagtgtctggagtaaatgaatcagctgacatgagtattggagtaacagtgataaagaacaacatgataaacaatgaccttggacctgcaacggcccagatggctcttcaattgttcatcaaagactacagatacacatataggtgccataggggagacacacaaattcagacgagaagatcatttgagttaaagaagctgtgggatcaaacccaatcaaaggtagggctattagtatcagatggaggaccaaacttatacaatatacggaatcttcacattcctgaagtctgcttaaaatgggaactaatggatgatgattatcggggaagactttgtaatcccctgaatccctttgtcagtcataaagagattgattctgtaaacaatgctgtggtaatgccagcccatggtccagccaaaagcatggaatatgatgccgttgcaactacacattcctggattcccaagaggaatcgttctattctcaacacaagccaaaggggaattcttgaggatgaacagatgtaccagaagtgctgcaatctattcgagaaatttttccctagcagttcatataggagaccggttggaatttctagcatggtggaggccatggtgtctagggcccggattgatgccagggtcgacttcgagtctggacggatcaagaaagaagagttctctgagatcatgaagatctgttccaccattgaagaactcagacggcaaaaataa

>H1N1_PB1_CY073755_A/Copenhagen/INS434/2009

atggatgtcaatccgactctacttttcctaaaaattccagcacaaaatgccataagcaccacattcccttatactggagatcctccatacagccatggaacaggaacaggatacaccatggacacagtaaacagaacacaccaatactcagaaaagggaaagtggacgacaaacacagagactggtgcaccccagctcaacccgattgatggaccactacctgaggataatgaaccaagtgggtatgcacaaacagactgtgttctagaggctatggctttccttgaagaatcccacccaggaatatttgagaattcatgccttgaaacaatggaagttgttcaacaaacaagggtggataaactaactcaaggtcgccagacttatgattggacattaaacagaaatcaaccggcagcaactgcattggccaacaccatagaagtctttagatcgaatggcctaacagctaatgagtcaggaaggctaatagatttcttaaaggatgtaatggaatcaatgaacaaagaggaaatagagataacaacccactttcaaagaaaaaggagagtaagagacaacatgaccaagaagatggtcacgcaaagaacaatagggaagaaaaaacaaaggctgaataagagaggctatctaataagagcactgacattaaatacgatgaccaaagatgcagagagaggcaagttaaaaagaagggctatcgcaacacctgggatgcagattagaggtttcgtatactttgttgaaactttagctaggagcatttgcgaaaagcttgaacagtctgggctcccagtagggggcaatgaaaagaaggccaaactggcaaatgttgtgagaaaaatgatgactaattcacaagacacagagatttctttcacaatcactggggacaacactaagtggaatgaaaatcaaaatcctcgaatgttcctggcgatgattacatatatcaccagaaatcaacccgagtggttcagaaacatcctgagcatggcacccataatgttctcaaacaaaatggcaagactagggaaagggtacatgttcgagagtaaaagaatgaagattcgaacacaaataccagcagaaatgctagcaagcattgacctgaagtacttcaatgaatcaacaaagaagaaaattgagaaaataaggcctcttctaatagatggcacagcatcactgagtcctgggatgatgatgggcatgttcaacatgctaagtacggtcttgggagtctcgatactgaatcttggacaaaagaaatacaccaagacaatatactggtgggatgggctccaatcatccgacgattttgctctcatagtgaatgcaccaaaccatgagggaatacaagcaggagtggacagattctataggacctgcaagttagtgggaatcaacatgagcaaaaagaagtcctatataaataagacagggacatttgaattcacaagctttttttatcgctatggatttgtggctaattttagcatggagctacccagctttggagtgtctggagtaaatgaatcagctgacatgagtattggagtaacagtgataaagaacaacatgataaacaatgaccttggacctgcaacggcccagatggctcttcaattgttcatcaaagactacagatacacatataggtgccataggggagacacacaaatccagacgagaagatcatttgagttaaagaagctgtgggatcaaacccaatcaaaggtagggctattagtatcagatggaggaccaaacttatacaatatacggaatcttcacattcctgaagtctgcttaaaatgggagctaatggatgatgattatcggggaagactttgtaaccccctgaatccctttgtcagtcataaagagattgattctgtaaacaatgctgtggtaatgccagcccatggtccagccaaaagcatggaatatgatgccgttgcaactacacattcctggattcccaagaggaatcgttctattctcaacacaagccaaaggggaattcttgaggatgaacagatgtaccagaagtgctgcaatctattcgagaaatttttccctagcagttcatataggagaccggttggaatttctagcatggtggaggccatggtgtctagggcccggattgatgccagggtcgacttcgagtctggacggatcaagaaagaagagttctctgagatcatgaagatctgttccaccattgaagaactcagacggcaaaaataa

>H1N1_PB1_CY073859_A/California/VRDL175/2009

atggatgtcaatccgacattacttttcttaaaagtgccagcacaaaatgctataagcacaacttttccttatactggtgaccctccttacagccatgggacaggaacagggtacaccatggatacagtcaacaggacacaccagtactcagaaagaggaagatggacagaaaataccgaaacgggagcaccgcaacttaacccaattgatggtcccttaccggaagacaatgaaccaagtggctatgcccaaacagattgtgtattagaagcaatggctttccttgaagaatcccatcccggtatctttgaaaactcttgtattgaaacaatggaggttgttcaacaaacaagggtggacaaactgacacaaggcagacagacctatgactggactctaaataggaaccagcctgctgccacagcattggcaaacactatagaagtattcagatcaaacggcctcatagcaaatgaatctgggaggctaatagacttccttaaagatgtaatggagtcgatggacagaggcgaagtagaggtcacaacacattttcaaagaaagaggagagtgagagacaatgtaactaaaaaaatggtgacccaaagaacaataggcaaaaagaaacataaattagacaaaagaagttacctgattagggcattaaccctgaacacaatgaccaaagatgctgagagggggaaactaaaacgcagagcaattgcaaccccaggaatgcaaataagggggtttgtatactttgttgagacactggcaagaagcatatgtgaaaagcttgaacaatcaggattgccagttggaggaaatgagaagaaagcaaagttagcaaatgttgtaaggaagatgatgaccaactcccaggacactgaaatttctttcaccataaccggagataacacaaaatggaacgaaaatcaaaaccctagaatgttcttggccatgatcacatatataaccaaaaatcagcctgaatggttcagaaatattctaagtattgctccaataatgttttcaaacaagatggcgagactaggtaaggggtacatgtttgaaagcaagagtatgaaactgagaactcaaatacctgcagagatgctagccaacatagacttgaaatatttcaatgattcaactaaaaagaaaattgaaaaaatccgaccattattaatagatggaactgcatcattgagtcctggaatgatgatgggcatgttcaatatgttgagcaccgtcttgggcgtctccatcctgaatcttgggcaaaagagatacaccaagactacttactggtgggatggtcttcaatcgtctgatgattttgctttgattgtgaatgcacccaactatgcaggaattcaagctggagttgacaggttttatcgaacctgtaagctgctcggaattaatatgagcaaaaagaagtcttacataaacagaacaggtacctttgaattcacgagctttttctatcgttatgggtttgttgctaatttcagcatggagcttcctagttttggggtgtctggggtcaatgaatctgcagacatgagtattggagtcactgtcatcaaaaacaatatgataaacaatgaccttggcccagcaactgctcaaatggcccttcagttatttataaaagattacaggtacacttatcgatgccaccgaggtgacacacaaatacaaacccggagatcatttgagataaagaaactatgggaccaaacccgctcaaaagctgggctgttggtctctgatggaggccccaatttgtataacattagaaatctccatatccctgaagtttgtttgaaatgggagttgatggatgaggattaccaggggcgtttatgcaacccattaaacccgtttgtcagccataaagagattgaatcagtgaacaatacagtgataatgccggcacatggtccagccaaaaatatggagtatgacgctgttgcaacaacacactcctgggtccccaaaagaaatcgatccattttgaacacgagccaaagggggatacttgaagatgagcaaatgtatcagaggtgctgcaatttatttgaaaaattcttcccaagtagctcatacagaagaccagttggaatatccagtatggtagaggctatggtctcaagagcccgaattgatgcacggattgatttcgaatctggaaggataaagaaagaggaatttgctgagatcatgaagatctgttccaccattgaagacctcagacggcaaaaatga

>H1N1_PB1_CY074361_A/California/VRDL252/2009

atggatgtcaatccgacattacttttcttaaaagtgccagcacaaaatgctataagcacaactttcccttatactggtgaccctccttacagccatgggacaggaacagggtacaccatggacacagtcaataggacacaccagtactcagaaagaggaagatggacaaaaaataccgaaacgggagcaccgcaacttaacccaattgatggtcccttaccggaagacaatgaaccaagtggctatgcccaaacagattgtgtattagaagcaatggctttccttgaagaatcccatcccggtatctttgaaaactcttgtattgaaacaatggaggttgttcaacaaacaagggtggacaaactgacacaaggcagacagacctatgactggactctaaataggaaccagcctgctgccacagcattggcaaacactatagaagtattcagagcaaacggcctcatagcaaatgaatctggaaggctaatagacttccttaaagatgtaatggagtcgatggacagaggcgaagtagaggtcacaactcattttcaaagaaagaggagagtgagagacaatgtaactaaaaaaatggtgacccaaagaacaataggcaaaaagaaacataaattagacaaaagaagttacctaattagggcattaaccctgaacacaatgaccaaagatgctgagagggggaaactaaaacgcagagcaattgcaaccccaggaatgcaaataagggggtttgtatactttgttgagacactggcaagaagcatatgtgaaaagcttgaacaatcaggattgccagttggaggaaatgagaagaaagcgaagttagcaaatgttgtaaggaagatgatgaccaactcccaggacactgaaatttctttcaccataaccggagacaacacaaaatggaacgaaaatcaaaaccctagaatgttcttggccatgatcacatatataaccaaaaatcagcctgaatggttcagaaatattctaagtattgctccaataatgttttcaaataagatggcgagactaggtaaggggtacatgtttgaaagcaagagtatgaaactgagaactcaaatacctgcagagatgctagccaacatagacttgaaatatttcaatgattcaactaaaaagaaaattgaaaaaatccgaccattattaatagatggaacagcatcattgagtcctggaatgatgatgggcatgttcaatatgttgagcaccgtcttgggcgtctccattctgaatcttgggcaaaagagatacaccaagactacttactggtgggatggtcttcaatcgtctgacgattttgctttgattgtgaacgcacccaactatgcaggaattcaagctggagttgacaggttttatcgaacctgtaagctgctcggaattaatatgagcaaaaagaagtcttatataaacagaacaggtacctttgaattcacgagctttttctatcgttatgggtttgttgccaatttcagcatggagcttcctagttttggggtgtctggggtcaatgaatctgcagacatgagtattggagtcactgtcatcaaaaacaatatgataaacaatgaccttggcccagcaactgctcaaatggcccttcagttatttataaaagattacaggtacacttatcgatgccaccgaggtgacacacaaatacaaacccgaagatcatttgagataaagaaactatgggaccaaacccgctcaaaagctggactgttggtctctgatggaggccccaatttgtataacattagaaatctccatattcctgaagtttgcttgaaatgggagttgatggatgaggattaccaggggcgtttatgcaacccattaaacccgtttgtcagccataaagagattgaatcagtgaacagtgcaatgataatgccggcacatggtccagccaaaaatatggagtatgacgctgttgcaacaacacactcctgggtccccaaaagaaatcgatccattttgaacacgagccaaagggggatacttgaagatgagcaaatgtatcagaggtgctgcaatttatttgaaaaattcttcccaagtagctcatacagaagaccagttggaatatccagtatggtagaggctatggtctcaagagcccgaattgatgcacggattgatttcgaatctggaaggataaagaaagaggaatttgctgagatcatgaagatctgttccaccattgaagacctcagacggcaaaaatga

>H1N1_PB1_CY074393_A/California/VRDL264/2009

atggatgtcaatccgacattacttttcttaaaagtgccagcacaaaatgctataagcacaacttttccttatactggtgaccctccttacagccatgggacaggaacagggtacaccatggatacagtcaacaggacacaccagtactcagaaagaggaagatggacaaaaaacaccgaaacgggagcaccgcaacttaacccaattgatggtcccttaccggaagacaatgaaccaagtggctatgcccaaacagattgtgtattagaagcaatggctttccttgaagaatcccatcccggtatctttgaaaactcttgtattgaaacaatggaggttgttcaacaaacaagggtggacaaactgacacaaggcagacagacctatgactggactctaaataggaaccagcctgctgccacagcattggcaaacactatagaagtattcagatcaaacggcctcatagcaaatgaatctgggaggctaatagacttccttaaagatgtaatggagtcaatggacagaggcgaagtagaggtcacaactcattttcaaagaaagaggagagtgagagacaatgtaactaaaaaaatggtgacccaaagaacaataggcaaaaagaaacataaattagacaaaagaagttacctgattagggcattaaccctgaacacaatgaccaaagatgctgagagggggaaactaaaacgcagagcaattgcaaccccaggaatgcaaataagggggtttgtatactttgttgagacactggcaagaaacatatgtgaaaagcttgaacaatcaggattgccagttggaggaaatgagaagaaagcaaagttagcaaatgttgtaaggaagatgatgaccaactcccaggacactgaaatttctttcaccataaccggagataacacaaaatggaacgaaaatcaaaaccctagaatgttcttggccatgatcacatatataaccaaaaatcagcctgaatggttcagaaatattctaagtattgctccaataatgttttcaaacaagatggcgagactaggtaaggggtacatgtttgaaagcaagagtatgaaactgagaactcaaatacctgcagagatgctagccaacatagacttgaaatatttcaatgattcaactaaaaagaaaattgaaaaaatccgaccattattaatagatggaactgcatcattgagtcctggaatgatgatgggcatgttcaatatgttgagcaccgtcttgggcgtctccattctgaatcttgggcaaaagagatacaccaagactacttactggtgggatggtcttcaatcgtctgatgattttgctttgattgtgaacgcacccaactatgcaggaattcaagctggagttgataggttttatcgaacctgtaagctgctcggaattaatatgagcaaaaagaagtcttacataaacagaacaggtacctttgaattcacgagctttttctatcgttatgggtttgttgccaatttcagcatggagcttcctagttttggggtgtctggggtcaatgaatctgcagacatgagtattggagtcactgtcatcaaaaacaacatgataaacaatgaccttggcccagcaactgctcaaatggcccttcagttatttataaaagattacaggtacacttatcgatgccaccgaggtgacacacaaatacaaacccggagatcatttgagataaagaaactatgggaccaaacccgctcaaaagctgggctgttggtctctgatggaggccccaatttgtataacattagaaatctccatattcctgaagtttgcttgaaatgggagttgatggatgaggattaccaggggcgtttatgcaacccattaaacccgtttgtcagccataaagagattgaatcagtgaacaatgcagtgataatgccggcacatggtccagccaaaaatatggagtatgacgctgttgcaacaacacactcctgggtccccaaaagaaatcgatccattttgaacacgagccaaagggggatacttgaagatgagcaaatgtatcagaggtgctgcaatttatttgaaaaattcttcccaagtagctcatacagaagaccagttggaatatccagtatggtagaggctatggtctcaagagcccgaattgatgcacggattgatttcgaatctggaaggataaagaaagaggaatttgctgagatcatgaagatctgttccaccattgaagacctcagacggcagaaatga

>H1N1_PB1_CY074425_A/California/VRDL275/2009

atggatgtcaatccgacattacttttcctaaaagtgccagcacaaaatgctataagcacaacttttccttatactggtgaccctccttacagccatgggacaggaacagggtacaccatggatacagtcaacaggacacaccagtactcagaaagaggaagatggacaaaaaataccgaaacgggagcaccgcaacttaacccaattgatggtcccttaccggaagacaatgaaccaagtggctatgcccaaacagattgtgtattagaagcaatggctttccttgaagaatcccatcccggtatctttgaaaactcttgtattgaaacaatggaggttgttcaacaaacaagggtggacaaactgacacaaggcagacagacctatgactggactctagataggaaccagcctgctgccacagcattggcaaacactatagaagtattcagatcaaacggcctcatagcaaatgaatctgggaggctaatagacttccttaaagatgtaatggagtcgatggacagaggcgaagtagaggtcacaactcattttcaaagaaagaggagagtgagagacaatgtaactaaaaaaatggtgacccaaagaacaataggcaaaaagaaacataaattagacaaaagaagttacctaattagggcattaaccctgaacacaatgaccaaagatgctgagagggggaaactaaaacgcagagcaatcgcaaccccaggaatgcaaataagggggtttgtatactttgttgagacactggcaagaagcatatgtgaaaagcttgaacaatcaggattgccagttggaggaaatgagaagaaagcaaagttagcaaatgttgtaaggaagatgatgaccaactcccaagacactgaaatttctttcaccataaccggagataacacaaaatggaacgaaaatcaaaaccctagaatgttcttggccatgatcacatatataaccaaaaatcagcctgaatggttcagaaatattctaagtattgctccaataatgttttcaaacaagatggcgagactaggcaaggggtacatgtttgaaagcaagagtatgaaactgagaactcaaatacctgcagagatgctagccaacatagacttgaaatatttcaatgattcaactaaaaagaaaattgaaaaaatccgaccattattaatagatggaactgcatcattgagtcctggaatgatgatgggcatgttcaatatgttgagcaccgtcttgggcgtctccattctgaatcttgggcaaaagagatacaccaagactacttactggtgggatggtcttcaatcgtctgatgattttgctttgattgtgaacgcacccaactatgcaggaattcaagctggagttgacaggttttatcgaacctgtaagctgctcggaattaatatgagcaaaaagaagtcttacataaacagaacaggtacctttgaattcacgagctttttctatcgttatgggtttgttgccaatttcagcatggagcttcctagttttggggtgtctggggtcaatgaatctgcagacatgagtattggagtcactgtcatcaaaaacaatatgataaacaatgaccttggcccagcaactgctcaaatggcccttcagttatttatcaaagattacaggtacacttatcgatgccaccgaggtgacacacaaatacaaacccggagatcatttgagataaagaaactatgggaccaaacccgctcaaaagctggactgttggtctctgatggaggccccaatttgtataacattagaaatctccatattcctgaagtttgcttgaaatgggagttgatggatgaggattaccaggggcggttatgcaacccattaaacccgtttgtcagccataaagagattgaatctgtgaacagtgcagtgataatgccggcacatggtccagccaaaaatatggagtatgacgctgttgcaacaacacactcctgggtccccaaaagaaatcgatccattttgaacacgagccaaagggggatacttgaagatgagcaaatgtatcagaggtgctgcaatttatttgaaaaattcttcccaagtagctcatacagaagaccagttggaatatccagtatggtagaggctatggtctcaagagcccgaattgatgcacggattgatttcgaatctggaaggataaagaaagaggaatttgctgagatcatgaagatctgttccaccattgaagacctcagacggcaaaaatga

>H1N1_PB1_CY074505_A/California/VRDL297/2009

atggatgtcaatccgacattacttttcttaaaagtgccagcacaaaatgctataagcacaacttttccttatactggtgaccctccttacagccatgggacaggaacagggtacaccatggatacagtcaacaggacacaccagtactcagaaagaggaagatggacaaaaaataccgaaacgggagcaccgcaacttaacccaattgatggtcccttaccggaagacaatgaaccaagtggctatgcccaaacagattgtgtattagaagcaatggctttccttgaagaatcccatcccggtatctttgaaaactcttgtattgaaacaatggaggttgttcaacaaacaagggtggacaaactgacacaaggcagacagacctatgactggactctaaataggaaccagcctgctgccacagcattggcaaacactatagaagtattcagatcaaacggcctcatagcaaatgaatctgggaggctaatagacttccttaaagatgtaatggaatcgatggacagaggcgaagtagaggtcacaactcattttcaaagaaagaggagagtgagagacaatgtaactaaaaaaatggtgacccaaagaacaataggcaaaaagaaacataaattagacaaaagaagttacctaattagggcattaaccctgaacacaatgaccaaagatgctgagagggggaaactaaaacgcagagcaattgcaaccccaggaatgcaaataagggggtttgtatactttgttgagacactggcaagaagcatatgtgaaaagcttgaacaatcaggattgccagttggaggaaatgagaagaaagcaaagttagcaaatgttgtaaggaagatgatgaccaactcccaggacactgaaatttctttcaccataaccggagataacacaaaatggaacgaaaatcaaaaccctagaatgttcttggccatgatcacatatataaccaaaaatcagcctgaatggttcagaaatattctaagtattgctccaataatgttttcaaacaagatggcgagactaggcaaggggtacatgtttgaaagcaagagtatgaaactgagaactcaaatacctgcagagatgctagccaacatagacttgaaatatttcaatgattcaactaaaaagaaaattgaaaaaatccgaccattattaatagatggaactgcatcattgagtcctggaatgatgatgggcatgttcaatatgttgagcaccgtcttgggcgtctccattctgaatcttgggcaaaagagatacaccaagactacttactggtgggatggtcttcaatcgtctgatgattttgctttgattgtgaacgcacccaactatgcaggaattcaagctggagttgacaggttttatcgaacctgtaagctgctcggaattaatatgagcaaaaagaagtcttacataaacagaacaggtacctttgaattcacgagctttttctatcgttatgggtttgttgccaatttcagcatggagcttcctagttttggggtgtctggggtcaatgaatctgcagacatgagtattggagtcactgtcatcaaaaacaatatgataaacaatgaccttggcccagcaactgctcaaatggcccttcagttatttatcaaagattacaggtacacttatcgatgccaccgaggtgacacacaaatacaaacccggagatcatttgagataaagaaactatgggaccaaacccgctcaaaagctggactgttggtctctgatggaggccccaatttgtacaacattagaaatctccatattcctgaagtttgcttgaaatgggagttgatggatgaggattaccaggggcgtttatgcaacccattaaacccgtttgtcagccataaagagattgaatctgtgaacagtgcagtgataatgccggcacatggtccagccaaaaatatggagtatgacgctgttgcaacaacacactcctgggtccccaaaagaaatcgatccattttgaacacgagccaaagggggatacttgaagatgagcaaatgtatcagaggtgctgcaatttatttgaaaaattcttcccaagtagctcatacagaagaccagttggaatatccagtatggtagaggctatggtctcaagagcccgaattgatgcacggattgatttcgaatctggaaggataaagaaagaggaatttgctgagatcatgaagatctgttccaccattgaagacctcagacggcaaaaatga

>H1N1_PB1_HQ291945_A/Taiwan/6566/2006

atggatgtcaatccgacattacttttcttaaaagtgccagcacaaaatgctataagcacaacttttccttatactggtgaccctccttacagccatgggacaggaacagggtacaccatggatacagtcaacaggacacatcagtactcagaaagaggaagatggacaaaaaataccgaaactggagcaccgcaactcaacccaattgatgggccactaccagaagacaatgaaccaagtggctatgcccaaacagattgtgtattagaagcaatggctttccttgaagaatcccatcctggtatttttgaaaactcttgtattgaaacaatggagattgttcagcaaacaagggtggacaaactgacacaaggcagacagacctatgactggactctaaataggaaccagcctgctgccacagcattggccaacactatagaagtattcagatcaaatggcctcatagcaaatgaatctgggaggctaatagacttccttaaagatgtaatggagtcgatggacagagaagaagtagaggtcacaactcattttcagagaaagaggagagtgagagacaatgtaactaaaaaaatggtgacccaaagaacaataggcaaaaagaaacataaattagacaaaaggagttacctaattagagcattaaccctgaacacaatgaccaaagatgctgagagggggaaactaaaacgcagagcaattgcaaccccaggaatgcaaataagggggtttgtatactttgttgaaacactggcaagaagcatatgtgaaaagcttgaacaatcagggttgccagttggaggaaatgagaagaaagcaaagttagcaaatgttgtaaggaagatgatgaccaactcccaggacactgaaatttctttcaccatcactggagataacacaaaatggaacgaaaatcaaaaccctagaatgtttttggccatgatcacatatataaccagaaaccagcctgaatggttcagaaatattctaagtgttgctccaataatgttttcaaacaaaatggcgagactaggtaaggggtacatgtttgaaagcaagagtatgaaactgagaactcaaatacctgcagagatactagccgacatagatttgaagtatttcaatgagtcaactaaaaagaaaattgaaaaaatccgaccattattaatagatggaactgcatcattgagtcctggaatgatgatgggcatgttcaatatgttaagcactgtcttgggcgtctccattctgaatcttgggcaaaagagatacaccaagactacttactggtgggatggccttcaatcgtctgatgattttgctctgatagtgaatgcgcccaactatgcaggaattcaagctggagttgacaggttttatcgaacctgtaagctgctcggaattaatatgagcaaaaagaagtcttacataaacaggacaggtacctttgaattcacaagctttttctatcgttatgggtttgttgccaatttcagcatggagcttcctagttttggggtgtctggggtcaatgaatctgcagacatgagtattggagtcactgttatcagaaacaatatgataaacaatgaccttggcccagcaactgctcaaatggcccttcagttatttataaaagattacagatacacgtatcgatgccacagaggtgacacacaaatacaaacccggagatcatttgagataaagaaattatgggaccaaacccgctccaaagctgggctgttggtttctgatggaggccccaatttatataacattagaaatctccatattcctgaagtctgcttgaaatgggagttgatggatgaggattaccaggggcgtttatgcaacccattgaacccatttgtcagtcataaagagattgaatcagtgaacaatgcagtgatgatgccggcacatggtccagccaaaaatatggagtatgacgctgttgcaacaacacattcctgggttcccaaaaggaatcgatccattttgaacacgagccaaagggggatacttgaggatgagcaaatgtatcagaggtgctgcaatttatttgaaaaattctttccaagtagctcatacagaagaccagttgggatatccagtatggtagaggctatggtttccagagcccgaattgatgcacggattgatttcgaatctggaaggataaagaaagaggaattcgctgagatcatgaagacctgttccaccattgaagacctcagacggcaaaaatag

>H1N1_PB1_CY075361_A/Chile/3375/2009

atggatgtcaatccgactctacttttcctaaaaattccagcgcaaaatgccataagcaccacattcccttatactggagatcctccatacagccatggaacaggaacaggatacaccatggacacagtaaacagaacacaccaatactcagaaaagggaaagtggacgacaaacacagagactggtgcaccccagctcaacccgattgatggaccactacctgaggataatgaaccaagtgggtatgcacaaacagactgtgttctagaggctatggctttccttgaagaatcccacccaggaatatttgagaattcatgccttgaaacaatggaagttgttcaacaaacaagggtagataaactaactcaaggtcgccagacttatgattggacattaaacagaaatcaaccggcagcaactgcattggccaacaccatagaagtctttagatcgaatggcctaacagctaatgagtcaggaaggctaatagatttcttaaaggatgtaatggaatcaatgaacaaagaggaaatagagataacaacccactttcaaagaaaaaggagagtaagagacaacatgaccaagaagatggtcactcaaagaacaatagggaagaaaaaacaaagactgaataagagaggctatctaataagagcactgacattaaatacgatgaccaaagatgcagagagaggcaagttaaaaagaagggctatcgcaacacctgggatgcagattagaggtttcgtatactttgttgaaactttagctaggagcatttgcgaaaagcttgaacagtctgggctcccagtagggggcaatgaaaagaaggccaaactggcaaatgttgtgagaaagatgatgactaattcacaagacacagagatttctttcacaatcactggggacaacactaagtggaatgaaaatcaaaatcctcgaatgttcctggcgatgattacatatatcaccagaaatcaacccgagtggttcagaaacatcctgagcatggcacccataatgttctcaaacaaaatggcaagactagggaaagggtacatgttcgagagtaaaagaatgaagattcgaacacaaataccagcagaaatgctagcaagcattgacctgaagtacttcaatgaatcaacaaagaagaaaattgagaaaataaggcctcttctaatagatggcacagcatcactgagtcctgggatgatgatgggcatgttcaacatgctaagtacggtcttgggagtctcgatactgaatcttggacaaaagaaatacaccaagacaatatactggtgggatgggctccaatcatccgacgattttgctctcatagtgaatgcaccaaaccatgagggaatacaagcaggagtggacagattctacaggacctgcaagttagtgggaatcaacatgagcaaaaagaagtcctatataaataagacagggacatttgaattcacaagctttttttatcgctatggatttgtggctaattttagcatggagctacccagctttggagtgtctggagtaaatgaatcagctgacatgagtattggagtaacagtgataaagaacaacatgataaacaatgaccttggacctgcaacggcccagatggctcttcaattgttcatcaaagactacagatacacatataggtgccataggggagacacacaaattcagacgagaagatcatttgagttaaagaagctgtgggatcaaacccaatcaaaggtagggctattagtatcagatggaggaccaaacttatacaatatacggaatcttcacattcctgaagtctgcttaaaatgggagctaatggatgatgattatcggggaagactttgtaatcccctgaatccctttgtcagtcataaagagattgattctgtaaacaatgctgtggtaatgccagcccatggtccagccaaaagcatggaatatgatgccgttgcaactacacattcctggattcccaagaggaatcgttctattctcaacacaagccaaaggggaattcttgaggatgaacagatgtaccagaagtgctgcaatctattcgagaaatttttccctagcagttcatataggagaccggttggaatttctagcatggtggaggccatggtgtctagggcccggattgatgccagggtcgacttcgagtctggacggatcaagaaagaagagttctctgagatcatgaagatctgttccaccattgaagaactcagacggcaaaaataa

>H1N1_PB1_CY075530_A/Boston/606/2009

atggatgtcaatccgactctacttttcctaaaaattccagcgcaaaatgccataagcaccacattcccttatactggagatcctccatacagccatggaacaggaacaggatacaccatggacacagtaaacagaacacaccaatactcagaaaagggaaagtggacgacaaacacagagactggtgcaccccagctcaacccgattgatggaccactacctgaggataatgaaccaagtgggtatgcacaaacagactgtgttctagaggctatggctttccttgaagaatcccacccaggaatatttgagaattcatgccttgaaacaatggaagttgttcaacaaacaagggtagataaactaactcaaggtcgccagacttatgattggacattaaacagaaatcaaccggcagcaactgcattggccaacaccatagaagtctttagatcgaatggcctaacagctaatgagtcaggaaggctaatagatttcttaaaggatgtaatggaatcaatgaacaaagaggaaatagagataacaacccactttcaaagaaaaaggagagtaagagacaacatgaccaagaagatggtcacgcaaagaacaatagggaagaaaaaacaaagactgaataagagaggctatctaataagagcactgacattaaatacgatgaccaaagatgcagagagaggcaagttaaaaagaagggctatcgcaacacctgggatgcagattagaggtttcgtatactttgttgaaactttagctaggagcatttgcggaaagcttgaacagtctgggctcccagtagggggcaatgaaaagaaggccaaactggcaaatgttgtgagaaagatgatgactaattcacaagacacagagatttctttcacaatcactggggacaacactaagtggaatgaaaatcaaaatcctcgaatgttcttggcgatgattacatatatcaccagaaatcaacccgagtggttcagaaacatcctgagcatggcacccataatgttctcaaacaaaatggcaagactagggaaagggtacatgttcgagagtaaaagaatgaagattcgaacacaaataccagcagaaatgctagcaagcattgacctgaagtacttcaatgaatcaacaaagaagaaaattgagaaaataaggcctcttctaatagatggcacagcatcactgagtcctgggatgatgatgggcatgttcaacatgctaagtacggtcttgggagtctcgatactgaatcttggacaaaagaaatacaccaagacaatatactggtgggatgggctccaatcatccgacgattttgctctcatagtgaatgcaccaaaccatgagggaatacaagcaggagtggacagattctacaggacctgcaagttagtgggaatcaacatgagcaaaaagaagtcctatataaataagacagggacatttgaattcacaagctttttttatcgctatggatttgtggctaattttagcatggagctacccagctttggagtgtctggagtaaatgaatcagctgacatgagtattggagtaacagtgataaagaacaacatgataaacaatgaccttggacctgcaacggcccagatggctcttcaattgttcatcaaagactacagatacacatataggtgccataggggagacacacaaattcagacgagaagatcatttgagttaaagaagctgtgggatcaaacccaatcaaaggtagggctattagtatcagatggaggaccaaacttatacaatatacggaatcttcacattcctgaagtctgcttaaaatgggagctaatggatgatgattatcggggaagactttgtaatcccctgaatccctttgtcagtcataaagagattgattctgtaaacaatgctgtggtaatgccagcccatggtccagccaaaagcatggaatatgatgccgttgcaactacacattcctggattcccaagaggaatcgttctattctcaacacaagccaaaggggaattcttgaggatgaacagatgtaccagaagtgctgcaatctattcgagaaatttttccctagcagttcatataggagaccggttggaatttctagcatggtggaggccatggtgtctagggcccggattgatgccagggtcgacttcgagtctggacggatcaagaaagaagagttctctgagatcatgaagatctgttccaccattgaagaactcagacggcaaaaataa

>H1N1_PB1_CY077887_A/Liverpool/1951

atggatgtcaatccgaccttacttttcttaaaagtgccagcacaaaatgctataagcacaactttcccttatactggagaccctccttacagccatgggacaggaacagggtacaccatggatactgtcaacaggacacatcagtactcagaaaggggaagatggacaaaaaacaccgaaactggagcaccacaactcaacccaattgatgggccactaccagaagacaatgaaccaagtggttatgcccaaacagattgtgtattagaagcaatggctttccttgaggaatcccatcctggtatttttgaaaactcatgtattgaaacgatggaggttgttcagcaaacacgagtggacaagctgacacaaggccgacagacctatgactggactctgaatagaaaccagcctgctgctacagcattggccaacacaatagaagtgttcagatcaaatggcctcatggcaaatgaatctggaaggctaatagactttcttaaggatgtaatggagtcaatggacaaagaagaaatggaggtcacaactcattttcagagaaagagacgagtgagggacaatgtgactaagaaaatggtgacacaaagaacaataggcaaaaagaaacagagattaaacaaaaggagttatctaattagggcattgaccctgaacacaatgaccaaagatgctgagagagggaaactaaaacgaagagcaattgcaaccccagggatgcaaataagggggtttgtatactttgttgagacactggcaagaagtatatgtgaaaaacttgaacaatcaggattgccagttggaggaaatgaaaagaaagcaaagttggcaaatgttgtaaggaagatgatgaccaattctcaggacactgaaatttctttcaccatcactggagataacaccaaatggaacgaaaatcagaaccctagaatgtttttggccatgatcacatatataaccagaaatcagcccgaatggttcagaaatattctaagtattgctccaataatgttctcaaacaaaatggcgagactaggtaagggttacatgtttgagagcaagagtatgaaacttagaactcaaatacctgcagaaatgatagcaaacattgatttgaagtatttcaatgattcaacaagaaagaaaattgaaaaaatccggccgctcttaatagatgggactgcatcactgagccctggaatgatgatgggcatgttcaatatgttaagcactgtattgggcgtctccatcctgaatcttggacaaaagagatacaccaagactacttactggtgggatggtcttcaatcatctgatgattttgctctgattgtgaatgcacccaatcatgcaggaattcaagctggagttgacaggttttatcgaacctgtaagctgctcggaatcaatatgagcaagaaaaagtcttacataaacagaacaggtacatttgaattcacgagctttttctatcgttatgggtttgttgccaatttcagcatggagcttcccagttttggggtgtctgggatcaacgagtctgcagacatgagtattggggtcactgtcatcaaaaacaatatgataaacaatgatcttggtccagcaaccgctcaaatggcccttcagttattcatcaaagattacaggtacacgtatcgatgccacagaggtgacacacaaatacaaacccgaagatcatttgaaataaagaaactatgggagcaaacccgttccaaagctgggctgctggtctccgatggaggtcctaatttatataacattagaaatctccacattcctgaagtctgcttgaaatgggaattgatggatgaggattaccaggggcgtttatgcaacccattgaacccatttgtcagtcataaagagattgaatcagtgaacaatgcagtgatgatgccggcacatggtccagccaaaaacatggagtatgatgctgtagcaacaacacactcctgggtccccaaaagaaatcgatccatcttgaatacgagccaaaggggaatacttgaagatgaacaaatgtatcaaaggtgctgcaatttatttgaaaaattcttccccagcagttcatacagaaggccagtcggaatatccagtatggtggaggctatggtttccagagcccgaattgatgcacggattgatttcgaatctggaaggataaagaaagaggatttcactgagatcatgaagatctgttccaccattgaagaactcagacggcaaaaatag

>H1N1_PB1_CY074031_A/Argentina/HNRG36/2009

atggatgtcaatccgactctacttttcctaaaaattccagcgcaaaatgccataagcaccacattcccttatactggagatcctccatacagccatggaacaggaacaggatacaccatggacacagtaaacagaacacaccaatactcagaaaagggaaagtggacgacaaacacagagactggtgcaccccagctcaacccgattgatggaccactacctgaggataatgaaccaagtgggtatgcacaaacagactgtgttctagaggctatggctttccttgaagaatcccacccaggaatatttgagaattcatgccttgaaacaatggaagttgttcaacaaacaagggtagataaactaactcaaggtcgccagacttatgattggacattaaacagaaatcaaccggcagcaactgcattggccaacaccatagaagtctttagatcgaatggcctaacagctaatgagtcaggaaggctaatagatttcttaaaggatgtaatggaatcaatgaacaaagaggaaatagagataacaacccactttcaaagaaaaaggagagtaagagacaacatgaccaagaagatggtcacgcaaagaacaatagggaagaaaaaacaaagactgaataagagaggctatctaataagagcactgacattaaatacgatgaccaaagatgcagagagaggcaagttaaaaagaagggctatcgcaacacctgggatgcagattagaggtttcgtatactttgttgaaactttagctaggagcatttgcgaaaagcttgaacagtctgggctcccagtagggggcaatgaaaagaaggccaaactggcaaatgttgtgagaaagatgatgactaattcacaagacacagagatttctttcacaatcactggggacaacactaagtggaatgaaaatcaaaatcctcgaatgttcctggcgatgattacatatatcaccagaaatcaacccgagtggttcagaaacatcctgagcatggcacccataatgttctcaaacaaaatggcaagactagggaaagggtacatgttcgagagtaaaagaatgaagattcgaacacaaataccagcagaaatgctagcaagcattgacctaaagtacttcaatgaatcaacaaagaagaaaattgagaaaataaggcctcttctaatagatggcacagcatcactgagtcctgggatgatgatgggcatgttcaacatgctaagtacggtcttgggagtctcgatactgaatcttggacaaaagaaatacaccaagacaatatactggtgggatgggctccaatcatccgacgattttgctctcatagtgaatgcaccaaaccatgagggaatacaagcaggagtggacagattctacaggacctgcaagttagtgggaatcaacatgagcaaaaagaagtcctatataaataagacagggacatttgaattcacaagctttttttatcgctatggatttgtggctaattttagcatggagctacccagctttggagtgtctggagtaaatgaatcagctgacatgagtattggagtaacagtgataaagaacaacatgataaacaatgaccttggacctgcaacggcccagatggctcttcaattgttcatcaaagactacagatacacatataggtgccataggggagacacacaaattcagacgagaagatcatttgagttaaagaagctgtgggatcaaacccaatcaaaggtagggctattagtatcagatggaggaccaaacttatacaatatacggaatcttcacattcctgaagtctgcttaaaatgggagctaatggatgatgactatcggggaagactttgtaatcccctgaatccctttgtcagtcataaagagattgattctgtaaacaatgctgtggtaatgccagcccatggtccagccaaaaacatggaatatgatgccgttgcaactacacattcctggattcccaagaggaatcgttctattctcaacacaagccaaaggggaattcttgaggatgaacagatgtaccagaagtgctgcaatctattcgagaaatttttccctagcagttcatataggagaccggttggaatttctagcatggtggaggccatggtgtctagggcccggattgatgccagggtcgacttcgagtctggacggatcaagaaagaagagttctctgagatcatgaagatctgttccaccattgaagaactcagacggcaaaaataa

>H1N1_PB1_CY080664_A/Boston/14/2009

atggatgtcaatccgacattacttttcttaaaagtgccagcacaaaatgctataagcacaacttttccttatactggtgaccctccttacagccatgggacaggaacagggtacaccatggatacagtcaacaggacacaccagtactcagaaagaggaagatggacaaaaaataccgaaacgggagcaccgcaacttaacccaattgatggtcccttaccggaagacaatgaaccaagtggctatgcccaaacagattgtgtattagaagcaatggctttccttgaagaatcccatcccggtatctttgaaaactcttgtattgaaacaatggaggttgttcaacaaacaagggtggacaaactgacacaaggcagacagacctatgactggactctaaataggaaccagcctgctgccacagcattggcaaacactatagaagtattcagatcaaacggcctcatagcaaatgaatctggaaggctaatagacttccttaaagatgtaatggagtcgatggacagaggcgaagtagaggtcacaactcattttcaaagaaagaggagagtgagagacaatgtaactaaaaaaatggtgacccaaagaacaataggcaaaaagaaacataaattagacaaaagaagttacctaattagggcattaaccctgaacacaatgaccaaagatgctgagagggggaaactaaaacgcagagcaattgcaaccccaggaatgcaaataagggggtttgtatactttgttgagacactggcaagaagcatatgtgaaaagcttgaacaatcaggattgccagttggaggaaatgagaagaaagcaaagttagcaaatgttgtaaggaagatgatgaccagctcccaggacactgaaatttctttcaccataaccggagacaacacaaaatggaacgaaaatcaaaaccctagaatgttcttggccatgatcacatatataaccaaaaatcagcctgaatggttcagaaatattctaagtattgctccaataatgttttcaaacaagatggcgagactaggtaaggggtacatgtttgaaagcaagagtatgaaactgagaactcaaatacctgcagagatgctagccaacatagacttgaaatatttcaatgattcaactaaaaagaaaattgaaaaaatccgaccattattaatagatggaactgcatcattgagtcctggaatgatgatgggcatgttcaatatgttgagcaccgtcttgggcgtctccattctgaatcttgggcaaaagagatacaccaagactacttactggtgggatggtcttcaatcgtctgatgattttgctttaattgtgaacgcacccaactatgcaggaattcaagctggagttgacaggttttatcgaacctgtaagctgctcggaattaatatgagcaaaaagaagtcttacataaacagaacaggtacctttgaattcacgagctttttctatcgttatgggtttgttgccaatttcagcatggagcttcctagttttggggtgtctggggtcaatgaatctgcagacatgagtattggagtcactgtcatcaaaaacaatatgataaacaatgaccttggcccagcaactgctcaaatggcccttcagttatttataaaagattacaggtacacttatcgatgccaccgaggtgacacacaaatacaaacccgaagatcatttgagataaagaaactatgggatcaaacccgctcaaaagctggactgttggtctctgatggaggccccaatttgtataacattagaaatctccatattcctgaagtttgcttgaaatgggagttgatggatgaggattaccaggggcgtttatgcaacccattaaacccgtttgtcagccataaagagattgaatcagtgaacagtgcagtgataatgccggcacatggtccagccaaaaatatggagtatgacgctgttgcaacaacacactcctgggtccccaaaagaaatcgatccattttgaacacgagccaaagggggatacttgaagatgagcaaatgtatcagaggtgctgcaatttatttgaaaaattcttcccaagtagctcatacagaagaccagttggaatatccagtatggtagaggctatggtctcaagagcccgaattgatgcacggattgatttcgaatctggaaggataaagaaagaggaatttgctgagatcatgaagatctgttccaccattgaagacctcagacggcaaaaatga

>H1N1_PB1_CY081099_A/Ontario/804635/2010

atggatgtcaacccgactctacttttcctgaaaattccagcgcaaaatgccataagcaccacattcccttatactggagatcctccatacagccatggaacaggaacaggatacaccatggacacagtaaacagaacacaccaatactcagaaaagggaaaatggacgacaaacacagagactggtgcaccccagctcaacccgattgatggaccactacctgaggataatgaaccaagtgggtatgcacaaacagactgtgttctagaggctatggctttccttgaagaatcccacccaggaatatttgagaattcatgccttgaaacaatggaagttgttcaacaaacaagggtagataaactaactaaaggtcgccagacttatgattggacattaaacagaaatcagccggcagcaactgcattggccaacaccatagaagtctttagatcgaatggcctaacagctaatgagtcaggaaggctaatagatttcttaaaggatgtaatggaatcaatgaacaaagaggaaatagagataacaacccactttcaaagaaaaaggagagtaagagacaacatgaccaagaagatggtcacgcaaagaacaatagggaagaaaaaacaaaaactaaataagagaggctatctaataagggcactgacattaaatacgatgaccaaagatgcagagagaggcaagttaaaaagaagggctatcgcaacacctgggatgcagattagaggtttcgtatactttgttgaaactttagctaggagcatttgcgaaaagcttgaacagtctgggctcccagtagggggcaatgaaaagaaggccaaactggcaaatgttgtgagaaagatgatgactaattcacaagacacagagatttctttcacaatcactggggacaacactaagtggaatgaaaatcaaaatcctcgaatgttcctggcgatgattacatatatcaccagaaatcaacccgaatggttcagaaacatcctgagcatggcacccataatgttctcaaacaaaatggcaagactagggaaagggtacatgttcgagagtaaaaaaatgaagattcgaacacaaataccagcagaaatgctagcaagcattgacctgaagtacttcaatgaatcaacaaagaagaaaattgagaaaataaggcctcttctaatagatggcacagcatcactgagtcctgggatgatgatgggcatgttcaacatgctaagtacggtcttgggagtctcgatactgaatcttggacaaaagaaatacaccaagacagtatactggtgggatgggctccaatcatccgacgattttgctctcatagtgaatgcaccaaaccatgagggaatacaagcaggagtggacagattctacaggacctgcaagttagtgggaattaacatgagcaaaaagaagtcctatataaataagacagggacatttgaattcacaagctttttttatcgctatggatttgtggctaattttagcatggagctacccagctttggagtgtctggagtaaatgaatcagctgacatgagtattggagtaacagtgataaagaacaacatgataaacaatgaccttggacctgcaacggcccagatggctcttcaattgttcatcaaagactacagatacacatataggtgccataggggagacacacaaattcagacgagaagatcatttgagttaaagaagctgtgggatcaaacccaatcaaaggtagggctattagtatcagatggaggaccaaacttatacaatatacggaatcttcacattcctgaagtctgcttaaaatgggagctaatggatgatgattatcggggaagactttgtaatcccctgaatccctttgtcagtcataaagagattgattctgtaaacaatgctgtggtaatgccagcccatggtccagttaaaagcatggaatatgatgccgttgcaactacacattcctggattcccaagaggaatcgttctattctcaacacaagccaaaggggaattcttgaggatgaacagatgtaccagaagtgctgcaatctattcgagaaatttttccctagcagttcatataggagaccggttggaatttctagcatggtggaggccatggtgtctagggcccggattgatgccagggttgacttcgagtctggacggatcaagaaagaagagttctctgagatcatgaagatctgttccaccattgaagaactcagacggcaaaaataa

>H1N1_PB1_CY083751_A/Barcelona/INS189/2009

atggatgtcaatccgactctacttttcctaaaaattccagcgcaaaatgccataagcaccacattcccttatactggagatcctccatacagccatggaacaggaacaggatacaccatggacacagtaaacagaacacaccaatactcagaaaagggaaagtggacgacaaacacagagactggtgcaccccaactcaacccgattgatggaccactacctgaggataatgaaccaagtgggtatgcacaaacagactgtgttctagaggctatggctttccttgaagaatcccacccaggaatatttgagaattcatgccttgaaacaatggaagttgttcaacaaacaagggtagataaactaactcaaggtcgccagacttatgattggacattaaacagaaatcaaccggcagcaactgcattggccaacaccatagaagtctttagatcgaatggcctaacagctaatgagtcaggaaggctaatagatttcttaaaggatgtaatggaatcaatgaacaaagaggaaatagagataacaacccactttcaaagaaaaaggagagtaagagacaacatgaccaagaagatggtcacgcaaagaacaatagggaagaaaaaacaaagactgaataagagaggctatctaataagagcactgacattaaatacgatgaccaaagatgcagagagaggcaagttaaaaagaagggctatcgcaacacctgggatgcagattagaggtttcgtatactttgttgaaactttagctaggagcatttgcgaaaagcttgaacagtctgggctcccagtagggggcaatgaaaagaaggccaaactggcaaatgttgtgagaaagatgatgactaattcacaagacacagagatttctttcacaatcactggggacaacactaagtggaatgaaaatcaaaatcctcgaatgttcctggcgatgattacatatatcaccagaaatcaacccgagtggttcagaaacatcctgagcatggcaccaataatgttctcaaacaaaatggcaagactagggaaagggtacatgttcgagagtaaaagaatgaagattcgaacacaaataccagcagaaatgctagcaagcattgacctgaagtacttcaatgaatcaacaaagaagaaaattgagaaaataaggcctcttctaatagatggcacagcatcactgagtcctgggatgatgatgggcatgttcaacatgctaagtacggtcttgggagtctcgatactgaatcttggacaaaagaaatacaccaagacaatatactggtgggatgggctccaatcatccgacgattttgctctcatagtgaatgcaccaaaccatgagggaatacaagcaggagtggacagattctacaggacctgcaagttagtgggaatcaacatgagcaaaaagaagtcctatataaataagacagggacatttgaattcacaagctttttttatcgctatggatttgtggctaattttagcatggagctacccagctttggagtgtctggagtaaatgaatcagctgacatgagtattggagtaacagtgataaagaacaacatgataaacaatgaccttggacctgcaacggcccagatggctcttcaattgttcatcaaagactacagatacacatataggtgccataggggagacacacaaattcagacgagaagatcatttgagttaaagaagctgtgggatcaaacccaatcaaaggtagggctattagtatcagatggaggaccaaacttatacaatatacggaatcttcacattcctgaagtctgcttaaaatgggagctaatggatgatgattatcggggaagactttgtaatcccctgaacccctttgtcagtcataaagagattgattctgtaaacaatgctgtggtaatgccagcccatggtccagccaaaagcatggaatatgatgccgttgcaactacacattcctggattcccaagaggaatcgttctattctcaacacaagccaaaggggaattcttgaggatgaacagatgtaccagaagtgctgcaatctattcgagaaatttttccctagcagttcatataggagaccggttggaatttctagcatggtggaggccatggtgtctagggcccggattgatgccagggtcgacttcgagtctggacggatcaagaaagaagagttctctgagatcatgaagatctgttccaccattgaagaactcagacggcaaaaataa

>H1N1_PB1_CY089145_A/Boston/77/2009

atggatgtcaatccgacattacttttcttaaaagtgccagcacaaaatgctataagcacaacttttccttatactggtgaccctccttacagccatgggacaggaacagggtacaccatggatacagtcaacaggacacaccagtactcagaaagaggaagatggacaaaaaataccgaaacgggagcaccgcaacttaacccaattgatggtcccttaccggaagacaatgaaccaagtggctatgcccaaacagattgtgtattagaagcaatggctttccttgaagaatcccatcccggtatctttgaaaactcttgtattgaaacaatggaggttgttcaacaaacaagggtggacaaactgacacaaggcagacagacctatgactggactctaaataggaaccagcctgctgccacagcattggcaaacactatagaagtattcagatcaaacggcctcatagcaaatgaatctggaaggctaatagacttccttaaagatgtaatggagtcgatggacagaggcgaagtagaggtcacaactcattttcaaagaaagaggagagtgagagacaatgtaactaaaaaaatggtgacccaaagaacaataggcaaaaagaaacataaattagacaaaagaagttacctaattagggcattaaccctgaacacaatgaccaaagatgctgagagggggaaactaaaacgcagagcaattgcaaccccaggaatgcaaataagggggtttgtatactttgttgagacactggcaagaagcatatgtgaaaagcttgaacaatcaggattgccagttggaggaaatgagaagaaagcaaagttagcaaatgttgtaaggaagatgatgaccagctcccaggacactgaaatttctttcaccataaccggagacaacacaaaatggaacgaaaatcaaaaccctagaatgttcttggccatgatcacatatataaccaaaaatcagcctgaatggttcagaaatattctaagtattgctccaataatgttttcaaacaagatggcgagactaggtaaggggtacatgtttgaaagcaagagtatgaaactgagaactcaaatacctgcagagatgctagccaacatagacttgaaatatttcaatgattcaactaaaaagaaaattgaaaaaatccgaccattattaatagatggaactgcatcattgagtcctggaatgatgatgggcatgttcaatatgttgagcaccgtcttgggcgtctccattctgaatcttgggcaaaagagatacaccaagactacttactggtgggatggtcttcaatcgtctgatgattttgctttaattgtgaacgcacccaactatgcaggaattcaagctggagttgacaggttttatcgaacctgtaagctgctcggaattaatatgagcaaaaagaagtcttacataaacagaacaggtacctttgaattcacgagctttttctatcgttatgggtttgttgccaatttcagcatggagcttcctagttttggggtgtctggggtcaatgaatctgcagacatgagtattggagtcactgtcatcaaaaacaatatgataaacaatgaccttggcccagcaactgctcaaatggcccttcagttatttataaaagattacaggtacacttatcgatgccaccgaggtgacacacaaatacaaacccgaagatcatttgagataaagaaactatgggatcaaacccgctcaaaagctggactgttggtctctgatggaggccccaatttgtataacattagaaatctccatattcctgaagtttgcttgaaatgggagttgatggatgaggattaccaggggcgtttatgcaacccattaaacccgtttgtcagccataaagagattgaatcagtgaacagtgcagtgataatgccggcacatggtccagccaaaaatatggagtatgacgctgttgcaacaacacactcctgggtccccaaaagaaatcgatccattttgaacacgagccaaagggggatacttgaagatgagcaaatgtatcagaggtgctgcaatttatttgaaaaattcttcccaagtagctcatacagaagaccagttggaatatccagtatggtagaggctatggtctcaagagcccgaattgatgcacggattgatttcgaatctggaaggataaagaaagaggaatttgctgagatcatgaagatctgttccaccattgaagacctcagacggcaaaaatga

>H1N1_PB1_HQ154076_A/KOL/46/2009

atggatgtcaatccgacattacttttcttaaaagtgccagcacaaaatgctataagcacaacttttccttatactggtgaccctccttacagccatgggacaggaacagggtacaccatggatacagtcaacaggacacaccagtactcagaaagaggaagatggacaaaaaataccgaaacgggagcaccgcaactcaacccaattgatggtcccttaccggaagacaatgaaccaagtggctatgcccaaacagattgtgtattagaagcaatggctttccttgaagaatcccatcccggtatctttgaaaattcttgtattgaaacaatggaggttgttcaacaaacaagggtggacaaactgacacaaggcagacagacctatgactggactctaaataggaaccagcctgctgccacagcattggcaaacactatagaagtattcagatcaaacggcctcatagcaaatgaatctgggaggctaatagacttccttaaagatgtaatggagtcgatggacagaggcgaagtagaggtcacaactcattttcaaagaaagaggagagtgagagacaatgtaactaaaaagatggtgacccaaagaacaataggcaaaaagaaacataaattagacaaaagaagttacctaattagggcattaaccctgaacacaatgaccaaagatgctgagagggggaaactaaaacgcagagcaattgcaaccccaggaatgcaaataagggggtttgtatactttgttgagacactggcaagaagcatatgtgaaaagcttgaacaatcaggattgccagttggaggaaatgagaagaaagcaaagttagcaaatgttgtaaggaagatgatgaccaactcccaggacactgaaatttctttcaccataaccggggataacacaaaatggaacgaaaatcaaaaccctagaatgttcttggccatgatcacatatataaccaaaaatcagcctgaatggttcagaaatattctaagtattgctccaataatgttttcaaacaagatggcgagactaggtaaggggtacatgtttgaaagcaagagtatgaaactgagaactcaaatacctgcagagatgctagccaacatagacttgaaatatttcaatgattcaactaaaaagaaaattgaaaaaatccgaccattattaatagatggaactgcatcattgagtcctggaatgatgatgggcatgttcaatatgttgagcaccgtcttgggcgtctccattctgaatcttgggcaaaagagatacaccaagactacttactggtgggatggtcttcaatcgtctgatgattttgctttgattgtgaacgcacccaactatgcaggaattcaagctggagttgacaggttttatcgaacctgtaagctgctcggaattaatatgagcaaaaagaagtcttacataaacagaacaggtacctttgaattcacgagctttttctatcgttatgggtttgttgccaatttcagcatggagcttcctagttttggggtgtctggggtcaatgaatctgcagacatgagtattggagtcactgtcatcaaaaacaatatgataaacaatgaccttggcccagcaactgctcaaatggcccttcagttatttataaaagattacagatacacttatcgatgccaccggggtgacacacaaatacaaacccggagatcatttgagataaagaaactatgggaccaaacccgctcaaaagctggactgttggtctctgatggaggccccaatttgtataacattagaaatctccatattcctgaagtttgcttgaaatgggagttgatggatgaggattaccaggggcgtttatgcaacccattaaacccgtttgtcagccataaagagattgaatctgtgaacagtgcagtgataatgccggcacatggtccagccaaaaatatggagtatgacgctgttgcaacaacacactcctgggtccccaaaagaaatcgatccattttgaacacgagccaaagggggatacttgaagatgagcaaatgtatcagaggtgctgcaatttatttgaaaaattcttcccaagtagctcatacagaagaccagttggaatatccagtatggtagaggctatggtctcaagagcccgaattgatgcacggattgatttcgaatctggaaggataaagaaagaggaatttgctgagatcatgaagatctgttccaccattgaagacctcagacggcaaaaatga

>H1N1_PB1_CY090843_A/Managua/0305_10/2010

atggatgtcaatccgactctacttttcctaaaaattccagcgcaaaatgccataagcaccacattcccttatactggagatcctccatacagccatggaacaggaacaggatacaccatggacacagtaaacagaacacaccaatactcagaaaagggaaagtggacgacaaacacagagactggtgcaccccagctcaacccgattgatggaccactacctgaggataatgaaccaagtgggtatgcacaaacagactgtgttctagaggctatggctttccttgaagaatcccacccaggaatatttgagaattcatgccttgaaacaatggaagttgttcaacaaacaagggtagataaactaactcaaggtcgccagacttatgattggacattaaacagaaatcaaccggcagcaactgcattggccaacaccatagaagtctttagatcgaatggcctaacagctaatgagtcaggaaggctaatagatttcttaaaggatgtaatggagtcaatgaacaaagaggaaatagagataacaacccactttcaaagaaaaaggagagtaagagacaacatgaccaagaagatggtcacgcaaagaacaatagggaagaaaaaacaaagactgaataagagaggctatctaataagagcactgacattaaatacgatgaccaaagatgcagagagaggcaagttaaaaagaagggctatcgcaacacctgggatgcagattagaggtttcgtatactttgttgaaactttagctaggagcatttgcgaaaagcttgaacagtctgggctcccagtagggggcaatgaaaagaaggccaaactggcaaatgttgtgagaaagatgatgactaattcacaagacacagagatttctttcacaatcactggggacaacactaagtggaatgaaaatcaaaatcctcgaatgttcctggcgatgattacatatatcaccagaaatcaacccgagtggttcagaaacatcctgagcatggcacccataatgttctcaaacaaaatggcaagactagggaaagggtacatgttcgagagtaaaagaatgaagattcgaacacaaataccagcagaaatgctagcaagcattgacctgaaatacttcaatgaatcaacaaagaagaaaattgagaaaataaggcctcttctaatagatggcacagcatcactgagtcctgggatgatgatgggcatgttcaacatgctaagtacggtcttgggagtctcgatattgaatcttggacaaaagaaatacaccaagacaatatactggtgggatgggctccaatcatccgacgattttgctctcatagtgaatgcaccaaaccatgagggaatacaagcaggagtggacagattctacaggacctgcaagttagtgggaatcaacatgagcaaaaagaagtcctatataaataagacagggacatttgaattcacaagctttttttatcgctatggatttgtggctaattttagcatggagctacccagctttggagtgtctggagtaaatgaatcagctgacatgagtattggagtaacagtgataaagaacaacatgataaacaatgaccttggacctgcaacggcccagatggctcttcaattgttcatcaaagactacagatacacatataggtgccataggggagacacacaaattcagacgagaagatcatttgagttaaaaaagctgtgggatcaaacccaatcaaaggcagggctattagtatcagatggaggaccaaacgtatacaatatacggaatcttcacattcctgaagtctgcttaaaatgggagctaatggatgatgattatcggggaagactttgtaatcccctgaacccctttgtcagtcataaagagattgattctgtaaacaatgctgtggtaatgccagcccatggtccagtcaaaagcatggaatatgatgccgttgcaactacacattcctggattcccaagaggaatcgttctattctcaacacaagccaaaggggaattcttgaggatgaacagatgtaccagaagtgctgcaatctattcgagaaatttttccctagcagttcatataggagaccggttggaatttctagcatggtggaggccatggtgtctagggcccggattgatgccagggtcgacttcgagtctggacggatcaagaaagaagagttctctgagatcatgaagatctgttccaccattgaagaactcagacggcaaaaataa

>H1N1_PB1_CY090867_A/Netherlands/2631c/2010

atggatgtcaatccgactctacttttcctaaaaattccagcgcaaaatgccataagcaccacattcccttatactggagatcctccatacagccatggaacaggaacaggatacaccatggacacagtaaacagaacacaccaatactcagaaaagggaaagtggacgacaaacacagagactggtgcaccccagctcaacccgattgatggaccactacctgaggataatgaaccaagtgggtatgcacaaacagactgtgttctagaggctatggctttccttgaagaatcccacccaggaatatttgagaattcatgccttgaaacaatggaagttgttcaacaaacaagggtagataaactaactcaaggtcgccagacttatgattggacattaaacagaaatcaaccggcagcaactgcattggccaacaccatagaagtctttagatcgaatggcctgacagctaatgagtcaggaaggctaatagatttcttaaaggatgtaatggaatcaatgaacaaagaggaaatagagataacaacccactttcaaagaaaaaggagagtaagagacaacatgaccaagaagatgatcacgcaaagaacaatagggaagaaaaaacaaagactgaataagagaggctatctaataagagcactgacattaaatacgatgaccaaagatgcagagagaggcaagttaaaaagaagggctatcgcaacacctgggatgcagattagaggtttcgtatactttgttgaaactttagctaggagcatttgcgaaaagcttgaacagtctgggctcccagtagggggcaatgaaaagaaggccaaactggcaaatgttgtgagaaagatgatgactaattcacaagacacagagatttctttcacaatcactggggacaacactaagtggaatgaaaatcaaaatcctcgaatgttcctggcgatgattacatatatcaccagaaatcaacccgagtggttcagaaacatcctgagcatggcacccataatgttctcaaacaaaatggcaagactagggaaagggtacatgttcgagagtaaaagaatgaagattcgaacacaaataccagcagaaatgctagcaagcattgacctgaagtacttcaatgaatcaacaaagaagaaaattgagaaaataaggcctcttctaatagatggcacagcatcactgagtcctgggatgatgatgggcatgttcaacatgctaagcacggtcttgggagtctcgatactgaatcttggacaaaagaaatacaccaagacaatatactggtgggatgggctccaatcatccgacgattttgctctcatagtgaatgcaccaaaccatgagggaatacaagcaggagtggacagattctacaggacctgcaagttagtgggaatcaacatgagcaaaaagaagtcctatataaataagacagggacatttgaattcacaagctttttttatcgctatggatttgtggctaattttagcatggagctacccagctttggagtgtctggagtaaatgaatcagctgacatgagtattggagtaacagtgataaagaacaacatgataaacaatgaccttggacctgcaacggcccagatggctcttcaattgttcatcaaagactacagatacacatataggtgccataggggagacacacaaattcagacgagaagatcatttgagttaaagaagctgtgggatcaaacccaatcaaaggtagggctattagtatcagatggaggaccaaacttatacaatatacggaatcttcacattcctgaagtctgcttaaaatgggagctaatggatgatgattatcggggaagactttgtaatcccctgaatccctttgtcagtcataaagagattgattctgtaaacaatgctgtggtaatgccagcccatggtccagccaaaagcatggaatatgatgccgttgcaactacacattcctggattcccaagaggaatcgttctattctcaacacaagccaaaggggaattcttgaggatgaacagatgtaccagaagtgctgcaatctattcgagaaatttttccctagcagttcatataggagaccggttggaatttctagcatggtggaggccatggtgtctagggcccggattgatgccagggtcgacttcgagtctggacggatcaagaaagaagagttctctgagatcatgaagatctgttccaccattgaagaactcagacggcaaaaataa

>H1N1_PB1_JN187220_A/Taiwan/6200/2009

atggatgtcaatccgactctacttttcctaaaaattccagcgcaaaatgccataagcaccacattcccttatactggagatcctccatacagccatggaacaggaacaggatacaccatggacacagtaaacagaacacaccaatactcagaaaagggaaagtggacgacaaacacagagactggtgcaccccagctcaacccgattgatggaccactacctgaggataatgaaccaagtgggtatgcacaaacagactgtgttctagaggctatggctttccttgaagaatcccacccaggaatatttgagaattcatgccttgaaacaatggaagttgttcaacaaacaagggtagataaactaactcaaggtcgccagacttatgattggacattaaacagaaatcaaccggcagcaactgcattggccaacaccatagaagtctttagatcgaatggcctaacagctaatgagtcaggaaggctaatagatttcttaaaggatgtaatggaatcaatgaacaaagaggaaatagagataacaacccactttcaaagaaaaaggagagtaagagacaacatgaccaagaagatggtcacgcaaagaacaatagggaagaaaaaacaaagactgaataagagaggctatctaataagagcactgacattaaatacgatgaccaaagatgcagagagaggcaagttaaaaagaagggctatcgcaacacctgggatgcagattagaggtttcgtatactttgttgaagctttagctaggagcatttgcgaaaagcttgaacagtctgggctcccagtagggggcaatgaaaagaaggccaaactggcaaatgttgtgagaaagatgatgactaattcacaagacacagagatttctttcacaatcactggggacaacactaagtggaatgaaaatcaaaatcctcgaatgttcctggcaatgattacatatatcaccagaaatcaacccgagtggttcagaaacatcctgagcatggcacccataatgttctcaaacaaaatggcaagactagggaaagggtacatgttcgagagtaaaagaatgaagattcgaacacaaataccagcagaaatgctagcaagcattgacctgaagtacttcaatgaatcaacaaagaagaaaattgagaaaataaggcctcttctaatagatggcacagcatcactgagtcctgggatgatgatgggcatgttcaacatgctaagtacggtcttgggagtctcgatactgaatcttggacaaaagaaatacaccaagacaatatactggtgggatgggctccaatcatccgacgattttgctctcatagtgaatgcaccaaaccatgaggaaatacaagcaggagtggacagattctacaggacctgcaagttagtgggaatcaacatgagcaaaaagaagtcctatataaataagacagggacatttgaattcacaagctttttttatcgctatggatttgtggctaattttagcatggagctacccagctttggagtgtctggagtaaatgaatcagctgacatgagtattggagtaacagtgataaagaacaacatgataaacaatgaccttggacctgcaacggcccagatggctcttcaattgttcatcaaagactacagatacacatataggtgccataggggagacacacaaattcagacgagaagatcatttgagttaaagaagctgtgggatcaaacccaatcaaaggtagggctattagtatcagatggaggaccaaacttatacaatatacggaatcttcacattcctgaagtctgcttaaaatgggagctaatggatgatgattatcggggaagactttgtaatcccctgaatccctttgtcagtcataaagagattgattctgtaaacaatgctgtggtaatgccagcccatggtccagccaaaagcatggaatatgatgccgttgcaactacacattcctggattcccaagaggaatcgttctattctcaacacaagccaaaggggaattcttgaggatgaacagatgtaccagaagtgctgcaatctattcgagaaatttttccctagcagttcatataggagaccggttggaatttctagcatggtggaggccatggtgtctagggcccggattgatgccagggtcgacttcgagtctggacggatcaagaaagaagagttctctgagatcatgaagatctgttccaccattgaagaactcagacggcaaaaataa

>H1N1_PB1_CY093139_A/Sydney/DD3-11/2010

atggatgtcaatccgactctacttttcctaaaaattccagcgcaaaatgccataagcaccacattcccttatactggagatcctccatacagccatggaacaggaacaggatacaccatggacacagtaaacagaacacaccaatactcagaaaagggaaagtggacgacaaacacagagactggtgcaccccagctcaacccgattgatggaccactacctgaggataatgaaccaagtgggtatgcacaaacagactgtgttctagaggctatggctttccttgaagaatcccacccaggaatatttgagaattcatgccttgaaacaatggaagttgttcaacaaacaagggtagataaactaactcaaggtcgccagacttatgattggacattaaacagaaatcaaccggcagcaactgcattggccaacaccatagaggtctttagatcgaatggcctaaaagctaatgattcaggaaggctaatagatttcttaaaggatgtaatggaatcaatgaacaaagaggaaatagagataacaacccactttcaaagaaaaaggagagtaagagacaatatgaccaagaagatggtcacgcaaagaacaatagggaagaaaaaacaaagactgaataagagaggctatctaataagagcactgacattaaacacgatgaccaaagatgcagagagaggcaagttaaaaagaagggctatcgcaacacctgggatgcagattagaggtttcgtatactttgttgaaactttagctaggagcatttgcgaaaagcttgaacagtctgggctcccagtagggggcaatgaaaagaaggccaaactggcaaatgttgtgagaaagatgatgactaattcacaagacacggagatttctttcacaatcactggggacaacactaagtggaatgaaaatcaaaatcctcgaatgttcctggcgatgattacatatatcaccagaaatcaacccgagtggttcagaaacatcctgagcatggcacccataatgttctcaaacaaaatggcaagactagggaaagggtacatgttcgagagtaaaagaatgaagattcgaacacaaataccagcagaaatgctagcaagcattgacctgaagtacttcaatgaatcaacaaagaagaaaattgagaaaataaggcctcttctaatagatggcacagcatcactgagtcctgggatgatgatgggcatgttcaacatgctaagtacggtcttgggagtctcgatactgaatcttggacaaaagaaatacaccaagacaatatactggtgggatgggctccaatcatccgacgattttgctctcatagtgaatgcaccaaaccatgagggaatacaagcaggagtggacagattctacaggacctgcaagttagtgggaatcaacatgagcaaaaagaagtcctatataaataagacagggacatttgaattcacaagctttttttatcgctatggatttgtggctaattttagcatggagctacccagctttggagtgtctggagtaaatgaatcagctgacatgagtattggagtaacagtgataaagaacaacatgataaacaatgaccttggacctgcaacggcccagatggctcttcaattgttcatcaaagactacagatacacatataggtgccataggggagacacacaaattcagacgagaagatcatttgagttaaagaagctgtgggatcaaacccaatcaaaggtagggctattagtatcagatggaggaccaaacttatacaatatacggaatcttcacatccctgaagtttgcttaaaatgggagctgatggatgatgattatcggggaagactttgtaatcccctgaatccctttgtcagtcataaagagattgattctgtaaacaatgctgtggtaatgccagcccatggtccagtcaaaagcatggaatatgatgccgttgcaactacacattcctggattcccaagaggaatcgttctattctcaacacaagccaaaggggaattcttgaggatgaacagatgtaccagaagtgctgcaatctattcgagaaatttttccctagcagttcatataggagaccggttggaatttctagcatggtggaggccatggtgtctagggcccggattgatgccagggtcgacttcgagtctggacggatcaagaaagaagagttctctgagatcatgaagatctgttccaccattgaagaactcagacggcaaaaataa

>H1N1_PB1_CY096080_A/Khon Kaen/INS443/2010

atggatgtcaatccgactctacttttcctaaaaattccagcgcaaaatgccataagcaccacattcccttatactggagatcctccatacagccatggaacaggaacaggatacaccatggacacagtaaacagaacacaccaatactcagaaaagggaaagtggacgacaaacacagagactggtgcaccccagctcaacccgattgatggaccactacctgaggataatgaaccaagtgggtatgcacaaacagactgtgttctagaggctatggctttccttgaagaatcccacccaggaatatttgagaattcatgccttgaaacaatggaagttgttcaacaaacaagggtagataaactaactcaaggtcgccagacttatgattggacattaaacagaaatcaaccggcagcaactgcattggccaacaccatagaagtctttagatcgaatggcctaacagctaatgagtcaggaaggctaatagatttcttaaaggatgtaatggaatcaatgaacaaagaggaaatagagataacaacccactttcaaagaaaaaggagagtaagagacaacatgaccaagaagatggtcacgcaaagaacaatagggaagaaaaaacaaagactgaataagagaggctatctaataagagcactgacattaaatacgatgaccaaagatgcagagagaggcaagttaaaaagaagggctatcgcaacacctgggatgcagattagaggtttcgtatactttgttgaaactttagctaggagcatttgcgaaaagcttgaacagtctgggctcccagtagggggcaatgaaaagaaggccaaactggcaaatgttgtgagaaagatgatgactaattcacaagacacagagatttctttcacaatcactggggacaacactaagtggaatgaaaatcaaaatcctcgaatgttcctggcgatgattacatatatcaccagaaatcaacccgagtggttcagaaacatcttgagcatggcacccataatgttctcaaacaaaatggcaagactagggaaagggtacatgttcgagagtaaaagaatgaagattcgaacacaaataccagcagaaatgctagcaagcattgacctgaagtacttcaatgaatcaacaaagaagaaaattgagaaaataaggcctcttctaatagatggcacagcatcactgagtcctgggatgatgatgggcatgttcaacatgctaagtacggtcttgggagtctcgatactgaatcttggacaaaagaaatacaccaagacaatatactggtgggatgggctccaatcatccgacgattttgctctcatagtgaatgcaccaaaccatgagggaatacaagcaggagtggacagattctacaggacctgcaagttagtgggaatcaacatgagcaaaaagaagtcctatataaataagacagggacatttgaattcacaagctttttttatcgctatggatttgtggctaattttagcatggagctacccagctttggagtgtctggagtaaatgaatcagctgacatgagtattggagtaacagtgataaagaacaacatgataaacaatgaccttggacctgcaacagcccagatggctcttcaactgttcatcaaagactacagatacacatataggtgccataggggagacacacaaattcagacgagaagatcatttgagttaaagaagctgtgggatcaaacccaatcaaaggtagggctattagtatcagatggaggaccaaacttatacaatatacggaatcttcacattcctgaagtctgcttaaaatgggagctaatggatgatgattatcggggaagactttgtaatcccctgaatccctttgtcagtcataaagagattgattctgtaaacaatgctgtggtaatgccagcccatggtccagccaaaagcatggaatatgatgccgttgcaactacacattcctggattcccaagaggaatcgttctattctcaacacaagccaaaggggaattcttgaggatgaacagatgtaccagaagtgctgcaatctattcgagaaatttttccctagcagttcatataggagaccggttggaatttctagcatggtggaggccatggtgtctagggcccggattgatgccagggtcgacttcgagtctggacggatcgggaaggaagagttctctgagatcatgaagatctgttccaccattgaagaactcagacggcaaaaataa

>H1N1_PB1_CY097874_A/Mexico/47N/2009

atggatgtcaatccgactctacttttcctaaaaattccagcgcaaaatgccataagcaccacattcccttatactggagatcctccatacagccatggaacaggaacaggatacaccatggacacagtaaacagaacacaccaatactcagaaaagggaaagtggacgacaaacacagagactggtgcaccccagctcaacccgattgatggaccactacctgaggataatgaaccaagtgggtatgcacaaacagactgtgttctagaggctatggctttccttgaagaatcccacccaggaatatttgagaattcatgccttgaaacaatggaagttgttcaacaaacaagggtagataaactaactcaaggtcgccagacttatgattggacattaaacagaaatcaaccggcagcaactgcattggccaacaccatagaagtctttagatcgaatggcctaacagctaatgagtcaggaaggctaatagatttcttaaaggatgtaatggaatcaatgaacaaagaggaaatagagataacaacccactttcaaagaaaaaggagaataagagacaacatgaccaagaagatggtcacgcaaagaacaatagggaagaaaaaacaaagactgaataagagaggctatctaataagagcactgacattaaatacgatgaccaaagatgcagagagaggcaagttaaaaagaagggctatcgcaacacctgggatgcagattagaggtttcgtatactttgttgaaactttagctaggagcatttgcgaaaagcttgaacagtctgggctcccagtagggggcaatgaaaagaaggccaagttggcaaatgttgtgagaaagatgatgactaattcacaagacacagagatttctttcacaatcactggggacaacactaagtggaatgaaaatcaaaatcctcgaatgttcctggcgatgattacatatatcaccagaaatcaacccgagtggttcagaaacatcctgagcatggcacccataatgttctcaaacaaaatggcaagactagggaaagggtacatgttcgagagtaaaagaatgaagattcgaacacaaataccagcagaaatgctagcaagcattgacctgaagtacttcaatgaatcaacaaagaagaaaattgagaaaataaggcctcttctaatagatggcacagcatcactgagtcctgggatgatgatgggcatgttcaacatgctaagtacggtcttgggagtctcgatactgaatcttggacaaaagaaatacaccaagacaatatactggtgggatgggctccaatcatccgacgattttgctctcatagtgaatgcaccaaaccatgagggaatacaagcaggagtggacagattctacaggacctgcaagttagtgggaatcaacatgagcaaaaagaagtcctatataaataagacagggacatttgaattcacaagctttttttatcgctatggatttgtggctaattttagcatggagctacccagctttggagtgtctggagtaaatgaatcagctgacatgagtattggagtaacagtgataaagaacaacatgataaacaatgaccttggacctgcaacggcccagatggctcttcaattgttcatcaaagactacagatacacatataggtgccataggggagacacacaaattcagacgagaagatcatttgagttaaagaagctgtgggatcaaacccaatcaaaggtagggctattagtatcagatggaggaccaaacttatacaatatacggaatcttcacattcctgaagtctgcttaaaatgggagctaatggatgatgattatcggggaagactttgtaatcccctgaatccctttgtcagtcataaagagattgattctgtaaacaatgctgtggtaatgccagcccatggtccagccgaaagcatggaatatgatgccgttgcaactacacattcctggattcccaagaggaatcgttctattctcaacacaagccaaaggggaattcttgaggatgaacagatgtaccagaagtgctgcaatctattcgagaaatttttccctagcagttcatataggagaccggttggaatttctagcatggtggaggccatggtgtctagggcccggattgatgccagggtcgacttcgagtctggacggatcaagaaagaagagttctctgagatcatgaagatctgttccaccattgaagaactcagacggcaaaaataa

>H1N1_PB1_CY083453_A/Lima/WRAIR1687P/2009

atggatgtcaatccgactctacttttcctaaaaattccagcgcaaaatgccataagcaccacattcccttatactggagatcctccatacagccatggaacaggaacaggatacaccatggacacagtaaacagaacacaccaatactcagaaaagggaaagtggacgacaaacacagagactggtgcaccccagctcaacccgattgatggaccactacctgaggataatgaaccaagtgggtatgcacaaacagactgtgttctagaggctatggctttccttgaagaatcccacccgggaatatttgagaattcatgccttgaaacaatggaagttgttcaacaaacaagggtagataaactaactcaaggtcgccagacttatgattggacattaaacagaaatcaaccggcagcaactgcattggccaacaccatagaagtctttagatcgaatggcctaacagctaatgagtcaggaaggctaatagatttcttaaaggatgtaatggaatcaatgaacaaagaggaaatagagataataacccactttcaaagaaaaaggagagtaagagacaacatgaccaagaagatggtcacgcaaagaacaatagggaagaaaaaacaaagactgaataagagaggctatctaataagagcactgacattaaatacgatgaccaaagatgcagagagaggcaagttaaaaagaagggctatcgcaacacctgggatgcagattagaggtttcgtatactttgttgaaactttagctaggagcatttgcgaaaagcttgaacagtctgggctcccagtagggggcaatgaaaagaaagccaaattggcaaatgttgtgagaaagatgatgactaattcacaagacacagagatttctttcacaatcactggggacaacactaagtggaatgaaaatcaaaatcctcgaatgttcctggcgatgattacatatatcaccagaaatcaacccgagtggttcagaaacatcctgagcatggcacccataatgttctcaaacaaaatggcaagactagggaaagggtacatgttcgagagtaaaagaatgaagattcgaacacaaataccagcagaaatgctagcaagcattgacctgaagtacttcaatgaatcaacaaagaagaaaattgagaaaataaggcctcttctaatagatggcacagcatcactgagtcctgggatgatgatgggcatgttcaacatgctaagtacggtcttgggagtctcgatactgaaccttggacaaaagaaatacaccaagacaatatactggtgggatgggctccaatcatccgacgattttgctctcatagtgaatgcaccaaatcatgagggaatacaagcaggagtggacagattctacaggacctgcaagttagtgggaatcaacatgagcaaaaagaagtcctatataaataagacagggacatttgaattcacaagctttttttatcgctatggatttgtggctaattttagcatggagctacccagctttggagtgtctggagtaaatgaatcagctgacatgagtattggagtaacagtgataaagaacaacatgataaacaatgaccttggacctgcaacggcccagatggctcttcaattgttcatcaaagactacagatacacatataggtgccataggggagacacacaaattcagacgagaagatcatttgagttaaagaagctgtgggatcaaacccaatcaaaggtagggctattagtatcagatggaggaccaaacttatacaatatacggaatcttcacattcctgaagtctgcttaaaatgggagctaatggatgatgattatcggggaagactttgtaatcccctgaatccctttgtcagtcataaagagattgattctgtaaacaatgctgtggtaatgccagcccatggtccagccaaaagcatggaatatgatgccgttgcaactacacattcctggattcccaagaggaatcgttctattctcaacacaagccaaaggggaattcttgaggatgaacagatgtaccagaagtgctgcaatctattcgagaaatttttccctagcagttcatataggagaccggttggaatttctagcatggtggaggccatggtgtctagggcccggattgatgccagggtcgacttcgagtctggacggatcaagaaagaagagttctctgagatcatgaagatctgttccaccattgaagaactcagacggcaaaaataa

>H1N1_PB1_CY097986_A/Budapest/WRAIR3794N/2011

atggatgtcaatccgactctacttttcctaaaaactccagcgcaaaatgccataagcaccacattcccttatactggagatcctccatacagccatggaacaggaacaggatacaccatggacacagtgaacagaacacaccaatactcagaaaagggaaagtggacgacaaacacagagactggtgcaccccagctcaacccgattgatggaccactacctgaggataatgaaccaagtgggtatgcacaaacagactgtgttctagaggctatggctttccttgaagaatcccacccagggatatttgagaattcatgccttgaaacaatggaagttgttcaacaaacaagggtagataaactaactcaaggtcgccagacttatgattggacattaaacagaaatcaaccggcagcaactgcattggccaacaccatagaagtctttagatcgaatggcctaacagctaatgagtcaggcaggctaatagatttcttaaaggatgtaatggaatcaatgaacaaagaggaaatagagataacaacccactttcaaagaaaaaggagagtaagagacaacatgaccaagaagatggtcacgcaaagaacaatagggaagaaaaaacaaagactgaataagagaggctatctaataagagcactgacattaaatacgatgaccaaagatgcagagagaggcaagttaaaaagaagggctatcgcaacacctgggatgcagattagaggtttcgtatactttgttgaaactttagctaggagcatttgcgaaaagcttgaacagtctgggctcccagtagggggcaatgaaaagaaaggcaaactggcaaatgttgtgagaaagatgatgactaattcacaagacacagagatttctttcacaatcactggggacaacactaagtggaatgaaaatcaaaatcctcgaatgttcctggcgatgattacatatatcaccagaaatcaacccgagtggttcagaaacatcctgagcatggcacccataatgttctcaaacaaaatggcaagactagggaaagggtacatgttcgagagtaaaagaatgaagattcgaacacaaataccagcagaaatgctagcaagcattgacctgaagtacttcaatgaatcaacaaagaagaaaattgagaaaataaggcctcttttaatagatggcacagcatcactgagtcctgggatgatgatgggcatgttcaacatgctaagtacggtcttgggagtctcgatactgaatcttggacaaaagaaatacaccaagacaatatactggtgggatgggctccaatcatccgacgattttgctctcatagtgaatgcaccaaaccatgagggaatacaagcaggagtggacagattctacaggacctgcaagttagtgggaatcaacatgagcaaaaagaagtcctatataaataagacagggacatttgaattcacaagctttttttatcgctatggatttgtggctaattttagcatggagctacccagctttggagtgtctggagtaaatgaatcagctgacatgagtattggagtaacagtgataaagaacaacatgataaacaatgaccttggacctgcaacggcccagatggctcttcaattgttcatcaaagactacagatacacatataggtgccataggggagacacacaaattcagacgagaagatcatttgagttaaagaagctgtgggatcaaacccaatcaaaggtagggctattagtatcagatggaggaccaaacttatacaatatacggaatcttcacattcctgaagtctgcttaaaatgggagctaatggatgatgattatcggggaagactttgtaatcccctgaatccctttgtaagtcataaagagattgattctgtaaacaatgctgtggtaatgccagcccatggtccagccaaaagcatggaatatgatgccgttgcaactacacattcctggattcccaagaggaatcgttctattctcaacacaagccaaaggggaattcttgaggatgaacagatgtaccagaagtgctgcaatctattcgagaaatttttccctagcagttcatataggagaccggttggaatttctagcatggtggaggccatggtgtctagggcccggattgatgccagggtcgactttgagtctggacgggtcaagaaagaagagttctctgagatcatgaagatctgttccaccattgaagaactcagacggcaaaaataa

>H1N1_PB1_CY098569_A/Bangkok/INS520/2010

atggatgtcaatccgactctacttttcctaaaaattccagcgcaaaatgccataagcaccacattcccttatactggagatcctccatacagccatggaacaggaacaggatacaccatggacacagtaaacagaacacaccaatactcagaaaagggaaagtggacgacaaacacagagactggtgcaccccagctcaacccgattgatggaccactacctgaggataatgaaccaagtgggtatgcacaaacagactgtgttctagaggctatggctttccttgaagaatcccacccaggaatatttgagaattcatgccttgaaacaatggaagttgttcaacaaacaagggtagataaactgactcaaggtcgccagacttatgattggacattaaacagaaatcaaccggcagcaactgcattggccaacaccatagaagtctttagatcgaatggcctaacagctaatgagtcaggaaggctaatagatttcttaaaggatgtaatggaatcaatgaacaaagaggaaatagagataacaacccactttcaaagaaaaaggagagtaagagacaacatgaccaagaagatggtcacgcaaagaacaatagggaagaaaaaacagagactgaataagagaggctatctaataagagcactgacattaaatacgatgaccaaagatgcagagagaggcaagttaaaaagaagggctatcgcaacacctgggatgcagattagaggtttcgtatactttgttgaaactttagctaggagcatttgcgaaaagcttgaacagtctgggctcccagtagggggcaatgaaaagaaggccaaactggcaaatgttgtgagaaagatgatgactaattcacaagacacagagatttctttcacaatcactggggacaacactaagtggaatgaaaatcaaaatcctcgaatgttcctggcgatgattacatatatcaccagaaatcaacccgagtggtttagaaacatcctgagcatggcacccataatgttctcaaacaaaatggcaagactagggaaagggtacatgttcgagagtaaaagaatgaaaattcgaacacaaataccagcagaaatgctagcaagcattgacctgaagtacttcaatgaatcaacaaagaagaaaattgagaaaataaggcctcttttaatggatggcacagcatcactgagtcctgggatgatgatgggcatgttcaacatgctaagtacggtcttgggagtctcgatactgaatcttggacaaaagaaatacaccaagacaatatactggtgggatgggctccaatcatccgacgattttgctctcatagtgaatgcaccaaaccatgagggaatacaagcaggagtggacagattctacaggacctgcaagttagtgggaatcaacatgagcaaaaagaagtcctatataaataagacagggacatttgaattcacaagctttttttatcgctatggatttgtggctaattttagcatggagctacccagctttggagtgtctggagtaaatgaatcagctgacatgagtattggagtaacagtgataaagaacaacatgataaacaatgaccttggacctgcaacggcccagatggctcttcaattgttcatcaaagactacagatacacatataggtgccataggggagacacacaaattcagacgagaagatcatttgagttaaagaagctgtgggatcaaacccaatcaaaggtagggctattagtatcagatggaggaccaaacttatacaatatacggaatcttcacatccctgaagtctgcttaaaatgggagctaatggatgatgattatcggggaagactttgtaatcccctgaatccctttgtaagtcataaggagattgattctgtaaacaatgctgtggtaatgccagcccatggtccagccaaaagcatggaatatgatgccgttgcaactacacattcctggattcccaagaggaatcgttctattctcaacacaagccaaaggggaattcttgaggatgaacagatgtaccagaagtgctgcaatctattcgagaaatttttccctagcagttcatataggagaccggttggaatttctagcatggtggaggccatggtgtctagggcccggattgatgccagggtcgactttgagtctggacggatcaagaaagaagagttctctgagatcatgaagatctgttccaccattgaagaactcagacggcaaaaataa

>H1N1_PB1_CY065204_A/England/204/2009

atggatgtcaatccgactctacttttcctaaaaattccagcgcaaaatgccataagcaccacattcccttatactggagatcctccatacagccatggaacaggaacaggatacaccatggacacagtaaacagaacacaccaatactcagaaaagggaaagtggacgacaaacacagagactggtgcaccccagctcaacccgattgatggaccactacctgaggataatgaaccaagtgggtatgcacaaacagactgtgttctagaggctatggctttccttgaagaatcccacccaggaatatttgagaattcatgccttgaaacaatggaagttgttcaacaaacaagggtagataaactaactcaaggtcgccagacttatgattggacattaaacagaaatcaaccggcagcaactgcattggccaacaccatagaagtctttagatcgaatggcctaacagctaatgagtcaggaaggctaatagatttcttaaaggatgtaatggaatcaatgaacaaagaggaaatagagataacaacccactttcaaagaaaaaggagagtaagagacaacatgaccaagaagatggtcacgcaaagaacaatagggaagaaaaaacaaagactgaataagagaggctatctaataagagcactgacattaaatacgatgaccaaagatgcagagagaggcaagttaaaaagaagggctatcgcaacacctgggatgcagattagaggtttcgtatactttgttgaaactttagctaggagcatttgcgaaaagcttgaacagtctgggctcccagtagggggcaatgaaaagaaggccaaactggcaaatgttgtgagaaagatgatgactaattcacaagacacagagatttctttcacaatcactggggacaacactaagtggaatgaaaatcaaaatcctcgaatgttcctggcgatgattacatatatcaccagaaatcaacccgagtggttcagaaacatcctgagcatggcacccataatgttctcaaacaaaatggcaagactagggaaagggtacatgttcgagagtaaaagaatgaagattcgaacacaaataccagcagaaatgctagcaagcattgacctgaagtacttcaatgaatcaacaaagaagaaaattgagaaaataaggcctcttctaatagatggcacagcatcactgagtcctgggatgatgatgggcatgttcaacatgctaagtacggtcttgggagtctcgatactgaatcttggacaaaagaaatacaccaagacagtatactggtgggatgggctccaatcatccgacgattttgctctcatagtgaatgcaccaaaccatgagggaatacaagcaggagtggacagattctacaggacctgcaagttagtgggaatcaacatgagcaaaaagaagtcctatataaataagacagggacatttgaattcacaagctttttttatcgctatggatttgtggctaattttagcatggagctacccagctttggagtgtctggagtaaatgaatcagctgacatgagtattggagtaacagtgataaagaacaacatgataaacaatgaccttggacctgcaacggcccagatggctcttcaattgttcatcaaagactacagatacacatataggtgccataggggagacacacaaattcagacgagaagatcatttgagttaaagaagctgtgggatcaaacccaatcaaaggtagggctattagtatcagatggaggaccaaacttatacaatatacggaatcttcacattcctgaagtctgcttaaaatgggagctaatggatgatgattatcggggaagactttgtaatcccctgaatccctttgtcagtcataaagagattgattctgtaaacaatgctgtggtaatgccagcccatggtccagccaaaagcatggaatatgatgccgttgcaactacacattcctggattcccaagaggaatcgttctattctcaacacaagccaaaggggaattcttgaggatgaacagatgtaccagaagtgctgcaatctattcgagaaatttttccctagcagttcatataggagaccggttggaatttctagcatggtggaggccatggtgtctagggcccggattgatgccagggtcgacttcgagtctggacggatcaagaaagaagagttctctgagatcatgaagatctgttccaccattgaagaactcagacggcaaaaataa

>H1N1_PB1_CY095998_A/Zhejiang/92/2009

atggatgtcaatccgactctacttttcctaaaaattccagcgcaaaatgccataagcaccacattcccttatactggagatcctccatacagccatggaacaggaacaggatacaccatggacacagtaaacagaacacaccaatactcagaaaagggaaagtggacgacaaacacagagactggtgcaccccagctcaacccgattgatggaccactacctgaggataatgaaccaagtgggtatgcacaaacagactgtgttctagaggctatggctttccttgaagaatcccacccaggaatatttgagaattcatgccttgaaacaatggaagttgttcaacaaacaagggtagataaactaactcaaggtcgccagacttatgattggacattaaacagaaatcaaccggcagcaactgcattggccaacaccatagaagtctttagatcgaatggcctaacagctaatgagtcaggaaggctaatagatttcttaaaggatgtaatggaatcaatgaacaaagaggaaatagagataacaacccactttcaaagaaaaaggagagtaagagacaacatgaccaagaagatggtcacgcaaagaacaatagggaagaaaaaacaaagactgaataagagaggctatctaataagagcactgacattaaatacgatgaccaaagatgcagagagaggcaagttaaaaagaagggctatcgcaacacctgggatgcagattagaggtttcgtatactttgttgaaactttagctaggagcatttgcgaaaagcttgaacagtctgggctcccagtagggggcaatgaaaagaaggccaaactggcaaatgttgtgagaaagatgatgactaattcacaagacacagagatttctttcacaatcactggggacaacactaagtggaatgaaaatcaaaatcctcgaatgttcctggcgatgattacatatatcaccagaaatcaacccgaatggttcagaaacatcctgagcatggcacccataatgttctcaaacaaaatggcaagactagggaaagggtacatgttcgagagtaaaagaatgaagattcgaacacaaataccagcagaaatgctagcaagcattgacctgaagtacttcaatgaatcaacaaagaagaaaattgagaaaataaggcctcttctaatagatggcacagcatcactgagtcctgggatgatgatgggcatgttcaacatgctaagtacggtcttgggagtctcgatactgaatcttggacaaaagaaatacaccaagacaatatactggtgggatgggctccaatcatccgacgattttgctctcatagtgaatgcaccaaaccatgagggaatacaagcaggagtggacagattctacaggacctgcaagttagtgggaatcaacatgagcaaaaagaagtcctatataaataagacagggacatttgaattcacaagctttttttatcgctatggatttgtggctaattttagcatggagctacccagctttggagtgtctggagtaaatgaatcagctgacatgagtattggagtaacagtgataaagaacaacatgataaacaatgaccttggacctgcaacggcccagatggctcttcaattgttcatcaaagactacagatacacatataggtgccataggggagatacacaaattcagacgagaaggtcatttgagttaaagaagctgtgggatcaaacccaatcaaaggtagggctattagtatcagatggaggaccaaacttatacaatatacggaatcttcacattcctgaagtctgcttaaaatgggagctaatggatgatgactatcggggaagactttgtaatcccctgaatccctttgtcagtcataaagagattgattctgtaaacaatgctgtggtaatgccagcccatggtccagccaaaagcatggaatatgatgccgttgcaactacacattcctggattcccaagaggaatcgttctattctcaacacaagccaaaggggaattcttgaggatgaacagatgtaccagaagtgctgcaatctattcgagaaatttttccctagcagttcatataggagaccggttggaatttctagcatggtggaggccatggtgtctagggcccggattgatgccagggtcgacttcgagtctggacggatcaagaaagaagagttctctgagatcatgaagatctgttccaccattgaagaactcagacggcaaaaataa

>H1N1_PB1_CY104892_A/DaNang/DN329/2008

atggatgtcaatccgacattacttttcttaaaagtgccagcacaaaatgctataagcacaacttttccttatactggtgaccctccttacagccatgggacaggaacagggtacaccatggatacagtcaacaggacacatcagtactcagaaagaggaagatggacaaaaaataccgaaactggagcaccgcaactcaacccagttgatgggccactaccagaagacaatgaaccaagtggctatgcccaaacagattgtgtattagaagcaatggctttccttgaagaatcccatcctggtatttttgaaaactcttgtattgaaacaatggagattgttcagcaaacaagggtggacaaactgacacaaggcagacagacctatgactggactctaaataggaaccagcctgctgccacagcattggccaacactatagaagtattcagatcaaacggcctcatagcaaatgaatctgggaggctaatagacttccttaaagatgtaatggagtcgatggacagagaagaagtagaggtcacaactcattttcagagaaagaggagagtgagagacaatgtaactaaaaaaatggtgacccaaagaacaataggcaaaaagaaacataaattagacaaaaggagttacctaattagagcattaactctgaacacaatgaccaaagatgctgaaagggggaaactaaaacgcagagcaattgcaaccccaggaatgcaaataagggggtttgtctactttgttgaaacactggcaagaagcatatgtgaaaagcttgaacaatcagggttgccagttggaggaaatgagaagaaagcaaagttagcaaatgttgtaaggaagatgatgaccaactcccaggacactgaaatttctttcaccatcactggagataacacaaagtggaacgaaaatcaaaaccctagaatgtttttggccatgatcacatatataaccagaaatcagcctgaatggttcagaaatattctaagtgttgctccaataatgttttcaaacaaaatggcgagactaggtaaggggtacatgtttgaaagcaagagtatgaaactaagaactcaaatacctgcagagatactagccggcatagatttgaagtatttcaatgagtcaactaaaaagaaaattgaaaaaatccgaccattattaatagatggaactgcatcattgagtcctggaatgatgatgggcatgttcaatatgttaagcactgtcttgggcgtctccattctgaatcttgggcaaaagagatacaccaagactacttactggtgggatggtcttcaatcgtctgatgattttgctctgatagtgaatgcgcccaactatgcaggaattcaagctggagttgacaggttttatcgaacctgtaagctgctcggaattaatatgagcaaaaagaagtcttacataaacaggacgggtacctttgaattcacaagctttttctatcgttatgggtttgttgccaatttcagcatggagcttcctagttttggggtgtcaggggtcaatgaatctgcagacatgagtattggagtcactgttatcagaaacaatatgataaacaatgaccttggcccagcaactgctcaaatggcccttcagttatttataaaagattacagatatacgtatcgatgccacagaggtgacacacaaatacaaacccggagatcatttgagataaagaaattatgggaccaaacccgctccaaagctgggctgttggtttctgatggaggccccaatttatataacattagaaatctccatattcctgaagtctgtttgaaatgggagttgatggatgaagattaccaggggcgtttatgcaatccattgaacccatttgtcagtcataaagagattgaatcagtgaacaatgcagtgatgatgccggcacatggtccagccaaaaatatggagtatgacgctgttgcaacaacacattcctgggttcccaaaaggaatcgatccattttgaatacaagccaaagggggatacttgaggatgagcaaatgtatcagaggtgctgcaatttatttgaaaaattctttccaagtagctcatacagaagaccagttgggatatccagtatggtagaggctatggtttccagagcccgaattgatgcacggattgatttcgaatctggaaggataaagaaagaggaattcgctgagatcatgaagacctgttccaccattgaagacctcagacggcaaaaatag

>H1N1_PB1_CY105108_A/KhanhHoa/KH38/2008

atggatgtcaatccgacattacttttcttaaaagtgccagcacaaaatgctataagcacaacttttccttatactggtgaccctccttacagccatgggacaggaacagggtacaccatggatacagtcaacaggacacaccagtactcagaaagaggaagatggacaaaaaataccgaaactggagcaccgcaacttaacccaattgatggtccactaccggaagacaatgaaccaagtggctatgcccaaacagattgtgtattagaagcaatggctttccttgaagaatcccatcccggtatctttgaaaactcttgtattgaaacaatggaggttgttcagcaaacaagggtggacaaactgacacaaggcagacagacctatgactggactctaaataggaaccagcctgctgccacagcattggcaaacactatagaagtattcagatcaaacggcctcatagcaaatgaatctgggaggctaatagacttccttaaagatgtaatggagtcgatggacagaggcgaagtagaggtcacaactcattttcaaagaaagaggagagtgagagacaatgtaactaaaaaaatggtgacccaaagaacaataggcaaaaagaaacataaattagacaaaagaagttacctaattagggcattaaccctgaacacaatgaccaaagatgctgagagagggaaactaaaacgcagagcaattgcaaccccaggaatgcaaataagggggtttgtatactttgttgagacactggcaagaagcatatgtgaaaagcttgaacaatcaggattgccagttggaggaaatgagaagaaagcaaagttagcaaatgttgtaagaaagatgatgaccaactcccaggacactgaaatttctttcaccataaccggagataacacaaaatggaacgaaaatcaaaaccctagaatgttcttggccatgatcacatatataaccaaaaatcagcctgaatggttcagaaatattctaagtattgctccaataatgttttcaaacaagatggcgagactaggtaaggggtacatgtttgaaagcaagagtatgaaactgagaactcaaatacctgcagagatgctagccaacatagatttgaaatatttcaatgattcaactaaaaagaaaattgaaaaaatccgaccattattaatagatggaactgcatcattgagtcctggaatgatgatgggcatgttcaatatgttgagcaccgtcttgggcgtctccattctgaatcttgggcaaaagagatacaccaagactacttactggtgggatggtcttcaatcgtctgatgattttgctttgattgtgaatgcacccaactatgcaggaattcaagctggagttgacaggttttatcgaacctgtaagctgctcggaattaatatgagcaaaaagaagtcttacataaacagaacaggtacctttgaattcacaagctttttctatcgttatgggtttgttgccaatttcagcatggagcttcctagttttggggtgtctggggtcaatgaatctgcagacatgagtattggagtcactgtcatcaaaaacaatatgataaacaatgaccttggcccagcaactgctcaaatggcccttcagttatttataaaagattacaggtacacttatcgatgccaccgaggtgacacacaaatacaaacccggagatcatttgagataaagaaactatgggaccaaacccgctcaaaagctgggctgttggtctctgatggaggccccaatttatataacattagaaatctccatattcctgaagtttgcttgaaatgggagttgatggatgaggattaccaggggcgtttatgcaacccattaaacccgtttgtcagccataaagagattgaatcagtgaacaatgcagtgataatgccggcacatggtccagccaaaaatatggagtatgacgctgttgcaacaacacactcctgggtccccaaaagaaatcgatccattttgaacacgagccaaagagggatacttgaagatgagcaaatgtatcagaggtgctgcaatttatttgaaaaattcttcccaagtagctcatacagaagaccagttggaatatccagtatggtagaggctatggtctcaagagcccgaattgatgcacggattgatttcgaatctggaaggataaagaaagaggaatttgctgagatcatgaagatctgttccaccattgaagacctcagacggcaaaaatga

>H1N1_PB1_CY047825_A/Argentina/8019/2009

atggatgtcaatccgactctacttttcctaaaaattccagcgcaaaatgccataagcaccacattcccttatactggagatcctccatacagccatggaacaggaacaggatacaccatggacacagtaaacagaacacaccaatactcagaaaagggaaagtggacgacaaacacagagactggtgcaccccagctcaacccgattgatggaccactacctgaggataatgaaccaagtgggtatgcacaaacagactgtgttctagaggctatggctttccttgaagaatcccacccaggaatatttgagaattcatgccttgaaacaatggaagttgttcaacaaacaagggtagataaactaactcaaggtcgccagacttatgattggacattaaacagaaatcaaccggcagcaactgcattggccaacaccatagaagtctttagatcgaatggcctaacagctaatgagtcaggaaggctaatagatttcttaaaggatgtaatggaatcaatgaacaaagaggaaatagagataacaacccactttcaaagaaaaaggagagtaagagacaacatgaccaagaagatggtcacgcaaagaacaatagggaagaaaaaacaaagactgaataagagaggctatctaataagagcactgacattaaatacgatgaccaaagatgcagagagaggcaagttaaaaagaagggctatcgcaacacctgggatgcagattagaggtttcgtatactttgttgaaactttagctaggagcatttgcgaaaagcttgaacagtctgggctcccagtagggggcaatgaaaagaaggccaaactggcaaatgttgtgagaaagatgatgactaattcacaagacacagagatttctttcacaatcactggggacaacactaagtggaatgaaaatcaaaatcctcgaatgttcctggcgatgattacatatatcaccagaaatcaacccgagtggttcagaaacatcctgagcatggcacccataatgttctcaaacaaaatggcaagactagggaaagggtacatgttcgagagtaaaagaatgaagattcgaacacaaataccagcagaaatgctagcaagcattgacctgaagtacttcaatgaatcaacaaagaagaaaattgagaaaataaggcctcttctaatagatggcacagcatcactgagtcctgggatgatgatgggcatgttcaacatgctaagtacggtcttgggagtctcgatactgaatcttggacaaaagaaatacaccaagacaatatactggtgggatgggctccaatcatccgacgattttgctctcatagtgaatgcaccaaaccatgagggaatacaagcaggagtggacagattctacaggacctgcaagttagtgggaatcaacatgagcaaaaagaagtcctatataaataagacagggacatttgaattcacaagctttttttatcgctatggatttgtggctaattttagcatggagctacccagctttggagtgtctggagtaaatgaatcagctgacatgagtattggagtaacagtgataaagaacaacatgataaacaatgaccttggacctgcaacggcccagatggctcttcaattgttcatcaaagactacagatacacatataggtgccataggggagacacacaaattcagacgagaagatcatttgagttaaagaagctgtgggatcaaacccaatcaaaggtagggctattagtatcagatggaggaccaaacttatacaatatacggaatcttcacattcctgaagtctgcttaaaatgggagctaatggatgatgactatcggggaagactttgtaatcccctgaatccctttgtcagtcataaagagattgattctgtaaacaatgctgtggtaatgccagcccatggtccagccaaaagcatggaatatgatgccgttgcaactacacattcctggattcccaagaggaatcgttctattctcaacacaagccaaaggggaattcttgaggatgaacagatgtaccagaagtgctgcaatctattcgagaaatttttccctagcagttcatataggagaccggttggaatttctagcatggtggaggccatggtgtctagggcccggattgatgccagggtcgatttcgagtctggacggatcaagaaagaagagttctctgagatcatgaagatctgttccaccattgaagaactcagacggcaaaaataa

>H1N1_PB1_CY047889_A/Argentina/9333/2009

atggatgtcaatccgactctacttttcctaaaaattccagcgcaaaatgccataagcaccacattcccttatactggagatcctccatacagccatggaacaggaacaggatacaccatggacacagtaaacagaacacaccaatactcagaaaagggaaagtggacgacaaacacagagactggtgcaccccagctcaacccgattgatggaccactacctgaggataatgaaccaagtgggtatgcacaaacagactgtgttctagaggctatggctttccttgaagaatcccacccaggaatatttgagaattcatgccttgaaacaatggaagttgttcaacaaacaagggtagataaactaactcaaggtcgccagacttatgattggacattaaacagaaatcaaccggcagcaactgcattggccaacaccatagaagtctttagatcgaatggcctaacagctaatgagtcaggaaggctaatagatttcttaaaggatgtaatggaatcaatgaacaaagaggaaatagagataacaacccactttcaaagaaaaaggagagtaagagacaacatgaccaagaaaatggtcacgcaaagaacaatagggaagaaaaaacaaagactgaataagagaggctatctaataagagcactgacattaaatacgatgaccaaagatgcagagagaggcaagttaaaaagaagggctatcgcaacacctgggatgcagattagaggtttcgtatactttgttgaaactttagctaggagcatttgcgaaaagcttgaacagtctgggctcccagtagggggcaatgaaaagaaggccaaactggcaaatgttgtgagaaagatgatgactaattcacaagacacagagatttctttcacaatcactggggacaacactaagtggaatgaaaatcaaaatcctcgaatgttcctggcgatgattacatatatcaccagaaatcaacccgagtggttcagaaacatcctgagcatggcacccataatgttctcaaacaaaatggcaagactagggaaagggtacatgttcgagagtaaaagaatgaagattcgaacacaaataccagcagaaatgctagcaagcattgacctgaagtacttcaatgaatcaacaaagaagaaaattgagaaaataaggcctcttctaatagatggcacagcatcactgagtcctgggatgatgatgggcatgttcaacatgctaagtacggtcttgggagtctcgatactgaatcttggacaaaagaaatacaccaagacaatatactggtgggatgggctccaatcatccgacgattttgctctcatagtgaatgcaccaaaccatgagggaatacaagcaggagtggacagattctacaggacctgcaagttagtgggaatcaacatgagcaaaaagaagtcctatataaataagacagggacatttgaattcacaagctttttttatcgctatggatttgtggctaattttagcatggagctacccagctttggagtgtctggagtaaatgaatcagctgacatgagtattggagtaacagtgataaagaacaacatgataaacaatgaccttggacctgcaacggcccagatggctcttcaattgttcatcaaagactacagatacacatataggtgccataggggagacacacaaattcagacgagaagatcatttgagttaaagaagctgtgggatcaaacccaatcaaaggtagggctattagtatcagatggaggaccaaacttatacaatatacggaatcttcacattcctgaagtctgcttaaaatgggagctaatggatgatgattatcggggaaggctttgtaatcccctgaatccctttgtcagtcataaagagattgattctgtaaacaatgctgtggtaatgccagcccatggtccagccaaaagcatggaatatgatgccgttgcaactacacattcctggattcccaagaggaatcgttctattctcaacacaagccaaaggggaattcttgaggatgaacagatgtaccagaagtgctgcaatctattcgagaaatttttccctagcagttcatataggagaccggttggaatttctagcatggtggaggccatggtgtctagggcccggattgatgccagggtcgacttcgagtctggacggatcaagaaagaagagttctctgagatcatgaagatctgttccaccattgaagaactcagacggcaaaaataa

>H1N1_PB1_JQ431373_A/Reunion/2923-3-M1E/2009

atggatgtcaatccgactctacttttcctaaaaattccagcgcaaaatgccataagcaccacattcccttatactggagatcctccatacagccatggaacaggaacaggatacaccatggacacagtaaacagaacacaccaatactcagaaaagggaaagtggacgacaaacacagagactggtgcaccccagctcaacccgattgatggaccactacctgaggataatgaaccaagtgggtatgcacaaacagactgtgttctagaggctatggctttccttgaagaatcccacccaggaatatttgagaattcatgccttgaaacaatggaagttgttcaacaaacaagggtagataaactaactcaaggtcgccagacttatgattggacattaaacagaaatcaaccggcagcaactgcattggccaacaccatagaagtctttagatcgaatggcctaacagataatgagtcaggaaggctaatagatttcttaaaggatgtaatggaatcaatgaacaaagaggaaatagagataacaacccactttcaaagaaaaaggagagtaagagacaacataaccaagaagatggtcacgcaaagaacaatagggaagaaaaaacaaagactgaataagagaggctatctaataagagcactgacattaaatacgatgaccaaagatgcagagagaggcaagttaaaaagaagggctatcgcaacacctgggatgcagattagaggtttcgtatactttgttgaaactttagctaggagcatttgcgaaaagcttgaacagtctgggctcccagtagggggcaatgaaaagaaggccaaactggcaaatgttgtgagaaagatgatgactaattcacaagacacagagatttctttcacaatcactggggacaacactaagtggaatgaaaatcaaaatcctcgaatgttcctggcgatgattacatatatcaccagaaatcaacccgagtggttcagaaacatcctgagcatggcacccataatgttctcaaacaaaatggcaagactagggaaagggtacatgttcgagagtaaaagaatgaagattcgaacacaaataccagcagaaatgctagcaagcattgacctgaagtacttcaatgaatcaacaaagaagaaaattgagaaaataaggcctcttctaatagatggcacagcatcactgagtcctgggatgatgatgggcatgttcaacatgctaagtacggtcttgggagtctcgatactgaatcttggacaaaagaaatacaccaagacaatatactggtgggatgggctccaatcatccgacgattttgctctcatagtgaatgcaccaaaccatgagggaatacaagcaggagtggacagattctacaggacctgcaagttagtgggaatcaacatgagcaaaaagaagtcctatataaataagacagggacatttgaattcacaagctttttttatcgctatggatttgtggctaattttagcatggagctacccagctttggagtgtctggagtaaatgaatcagctgacatgagtattggagtaacagtgataaagaacaacatgataaacaatgaccttggacctgcaacggcccagatggctcttcaattgttcatcaaagactacagatacacatataggtgccataggggagacacacaaattcagacgagaagatcatttgagttaaagaagctgtgggatcaaacccaatcaaaggtagggctattagtatcagatggaggaccaaacttatacaatatacggaatcttcacattcctgaagtctgcttaaaatgggagctaatggatgatgattatcggggaagactttgtaatcccctgaatccctttgtcagtcataaagagattgattctgtaaacaatgctgtggtaatgccagcccatggtccagccaaaagcatggaatatgatgccgttgcaactacacattcctggattcccaagaggaatcgttctattctcaacacaagccaaaggggaattcttgaggatgaacagatgtaccagaagtgctgcaatctattcgagaaatttttccctagcagttcatataggagaccggttggaatttctagcatggtggaggccatggtgtctagggcccggattgatgccagggtcgacttcgagtctggacggatcaagaaagaagagttctctgagatcatgaagatctgttccaccattgaagaactcagacggcaaaaataa

>H1N1_PB1_CY111260_A/Boston/DOA21/2011

atggatgtcaatccgactctacttttcctaaaaattccagcgcaaaatgccataagcaccacattcccttatactggagaccctccatacagccatggaacaggaacaggatacaccatggacacagtaaacagaacacaccaatactcagaaaagggaaagtggacgacaaacacagagactggtgcaccccagctcaacccgattgatggaccactacctgaggataatgaaccaagtgggtatgcacaaacagactgtgttctagaggctatggctttccttgaagaatcccacccaggaatatttgagaattcatgccttgaaacaatggaagttgttcaacaaacaagggtagataaactaactcaaggtcgccagacttatgattggacattaaacagaaatcaaccggcagcaactgcattggccaacaccatagaagtctttagatcgaatggcctaacagctaatgagtcaggaaggctaatagatttcttaaaggatgtaatggaatcaatgaacaaagaggaaatagagataacaacccactttcaaagaaaaaggagagtaagagacaacatgaccaagaagatggtcacgcaaagaacaatagggaagaaaaaacaaagactgaataagagaggctatctaataagagcactgacattaaatacgatgaccaaagatgcagagagaggcaagttaaaaagaagggctatcgcaacacctgggatgcagattagaggtttcgtatactttgttgaaactttagctaggagcatttgcgaaaagcttgaacagtctgggctcccagtagggggcaatgaaaagaaggccaaactggcaaatgttgtgagaaagatgatgactaattcacaagacacagagatttctttcacaatcactggggacaacactaagtggaatgaaaaccaaaatcctcgaatgttcctggcgatgattacatatatcaccagaaatcaacccgagtggttcagaaacatcctgagcatggcacccataatgttctcaaacaaaatggcaagactagggaaagggtacatgttcgagagtaaaagaatgaagattcgaacgcaaataccagcagaaatgctagcaagcattgacctgaagtacttcaatgaatcaacaaagaagaaaattgagaaaataaggcctcttttaatagatggcacagcatcactgagtcctgggatgatgatgggcatgttcaacatgctaagtacggtcttgggagtctcgatactgaatcttggacaaaagaaatacaccaagacaatatactggtgggatgggctccaatcatccgacgattttgctctcatagtgaatgcaccaaaccatgagggaatacaagcaggagtggacagattttacaggacctgcaagttagtgggaatcaacatgagcaaaaagaagtcctatataaataagacagggacatttgaattcacaagctttttttatcgctatggatttgtggctaattttagcatggagctacccagctttggagtgtctggagtaaatgaatcagctgacatgagtattggagtaacagtgataaagaacaacatgataaacaatgaccttggacctgcaacggcccagatggctcttcaattgttcatcaaagactacagatacacatataggtgccataggggagacacacaaattcagacgagaagatcatttgagttaaagaagctgtgggatcaaacccaatcaaaggtagggctattagtatcagatggaggaccaaacttatacaatatacggaatcttcacattcctgaagtctgcttaaaatgggagctaatggatgatgattatcggggaagactttgtaatcccctgaatccctttgtaagtcataaagagattgattctgtaaacaatgctgtgataatgccagctcatggtccagccaaaagcatggaatatgatgccgttgcaactacacattcctggattcccaagaggaatcgttctattctcaacacaagccaaaggggaattcttgaggatgaacagatgtaccagaagtgctgcaatctattcgagaaatttttccctagcagttcatataggagaccggttggaatttctagcatggtggaggccatggtgtctagggcccggattgatgccagggtcgactttgagtctggacggatcaagaaagaagagttctctgagatcatgaagatctgttccaccattgaagaactcagacggcaaaaataa

>H1N1_PB1_CY111404_A/Boston/DOA73/2011

atggatgtcaatccgactctacttttcctaaaaattccagcgcaaaatgccataagcaccacattcccttatactggagatcctccatacagccatggaacaggaacaggatacaccatggacacagtgaacagaacacaccaatactcagaaaagggaaagtggacgacaaacacagagactggtgcaccccagctcaacccgattgatggaccactacctgaggataatgaaccaagtgggtatgcacaaacagactgtgttctagaggctatggctttccttgaagaatcccacccaggaatatttgataattcatgccttgaaacaatggaagttgttcaacaaacaagggtagataaactaactcaaggtcgccagacttatgattggacattaaacagaaatcaaccggcagcaactgcattggccaacaccatagaagtctttagatcgaatggcctaacagctaatgagtcaggcaggctaatagatttcttaaaggatgtaatggaatcaatgaacaaagaggaaatagagataacaacccactttcaaagaaaaaggagagtaagagacaacatgaccaagaagatggtcacgcaaagaacaatagggaagaaaaaacaaagactgaataagagaggctatctaataagagcactgacattaaatacgatgaccaaagatgcagagagaggcaagttaaaaagaagggctatcgcaacacctgggatgcagattagaggtttcgtgtactttgttgaaactttagctaggagcatttgcgaaaagcttgaacagtctgggctcccagtagggggcaatgaaaagaaggccaaactggcaaatgttgtgagaaagatgatgactaattcacaagacacagagatttctttcacaatcactggggacaacactaagtggaatgaaaatcaaaatcctcgaatgttcctggcgatgattacatatatcaccagaaatcaacccgagtggttcagaaacatcctgagcatggcacccataatgttctcaaacaaaatggcaagactagggaaagggtacatgttcgagagtaaaagaatgaagattcggacacaaataccagcagaaatgctagcaagcattgacctgaagtacttcaatgaatcaacaaagaagaaaattgagaaaataaggcctcttttaatagatggcacagcatcactgagtcctgggatgatgatgggcatgttcaacatgctaagtacggtcttgggagtctcgatactgaatcttggacaaaagaaatacaccaagacaatatactggtgggatgggctccaatcatccgacgattttgctctcatagtgaatgcaccaaaccatgagggaatacaagcaggagtggacagattctacaggacctgcaagttagtgggaatcaacatgagcaaaaagaagtcctatataaataagacagggacatttgaattcacaagctttttttatcgctatggatttgtggctaattttagcatggagctacccagctttggagtgtctggagtaaatgaatcagctgacatgagtattggagtaacagtgataaagaacaacatgataaacaatgaccttggacctgcaacggcccagatggctcttcaattgttcatcaaagactacagatacacatataggtgccataggggagacacacaaattcagacgagaagatcatttgagttaaagaagctgtgggatcaaacccaatcaaaggtagggctattagtatcagatggaggaccaaacttatacaatatacggaatcttcacattcctgaagtctgcttaaaatgggagctaatggatgatgattatcggggaagactttgtaatcccctgaatccctttgtaagtcataaagagattgattctgtaaacaatgctgtggtaatgccagcccatggtccagccaaaagcatggaatatgatgccgttgcaactacacattcctggattcccaagaggaatcgttctattctcaacacaagccaaaggggaattcttgaggatgaacagatgtaccagaagtgctgcaatctattcgagaaatttttccctagcagttcatataggagaccggttggaatttctagcatggtggaggccatggtgtctagggcccggattgatgccagggtcgactttgagtctggacggatcaagaaagaagagttctctgagatcatgaagatctgttccaccattgaagaactcagacggcaaaaataa

>H1N1_PB1_CY118173_A/Malaysia/1999752/2008

atggatgtcaatccgacattacttttcttaaaagtgccagcacaaaatgctataagcacaacttttccttatactggtgaccctccttacagccatgggacaggaacagggtacaccatggatacagtcaacaggacacaccagtactcagaaagaggaagatggacaaaaaataccgaaacgggagcaccgcaacttaacccaattgatggtcccttaccggaagacaatgaaccaagtggctatgcccaaacagattgtgtattagaagcaatggctttccttgaagaatcccatcccggtatctttgaaaactcttgtattgaaacaatggaggttgttcaacaaacaagggtggacaaactgacacaaggcagacagacctatgactggactctaaatagaaaccagcctgctgccacagcattggcaaacactatagaagtattcagatcaaacggcctcatagcaaatgaatctgggaggctaatagacttccttaaagatgtaatggagtcgatggacagaggcgaagtagaggtcacaactcattttcaaagaaagaggagagtgagagacaatgtaactaaaaaaatggtgacccaaagaacaataggcaaaaagaaacataaattagacaaaagaagttacctaattagggcattaaccctgaacacaatgaccaaagatgctgagagggggaaactaaaacgcagagcaattgcaaccccaggaatgcaaataagggggtttgtatactttgttgagacactggcaagaagcatatgtgaaaagcttgaacaatcaggattgccagttggaggaaatgagaagaaagcaaagttagcaaatgttgtaaggaagatgatgaccaactcccaggacactgaaatttctttcaccataaccggagataacacaaaatggaacgaaaatcaaaaccctagaatgttcttggccatgatcacatacataaccaaaaatcagcctgaatggttcagaaatattctaagtattgctccaataatgttttcaaacaagatggcgagactaggtaaggggtacatgtttgaaagcaagagtatgaaactgagaactcaaatacctgcagagatgctagccaacatagacttgaaatatttcaatgattcaactaaaaagaaaattgaaaaaatccgaccattattaatagatggaactgcatcattgagtcctggaatgatgatgggcatgttcaatatgttgagcaccgtcttgggcgtctccattctgaatcttgggcaaaagagatacaccaagactacttactggtgggatggtcttcaatcgtctgatgattttgctttgattgtgaacgcacccaactatgcaggaattcaagctggagttgacaggttttatcgaacctgtaagctgctcggaattaatatgagcaaaaagaagtcttacataaacagaacaggtacctttgaattcacgagctttttctatcgttatgggtttgttgccaatttcagcatggagcttcctagktttggggtgtctggggtcaatgaatctgcagacatgagtattggagtcactgtcatcaaaaacaatatgataaacaatgaccttggcccagcaactgctcaaatggcccttcagttatttataaaagattacaggtacacttatcgatgccaccgaggtgacacacaaatacaaacccggagatcatttgagataaagaagctatgggaccaaacccgctcaaaagctgggctgttggtctctgatggaggccccaatttgtataacattagaaatctccatattcctgaagtttgcttgaaatgggagttgatggatgaggattaccaggggcgtttatgcaacccattaaacccgtttgtcagccataaagagattgaatcagtgaacaatgcagtgataatgccggcacatggtccagccaaaaatatggagtatgacgctgttgcaacaacacactcctgggtccccaaaagaaatcgatccattttgaacacgagccaaagggggatacttgaagatgagcaaatgtatcagaggtgctgcaatttatttgaaaaattcttcccaagtagctcatacagaagaccagttggaatatccagtatggtagaggctatggtctcaagagcccgaattgatgcacggattgatttcgaatctggaaggataaagaaagaggaatttgctgagatcatgaagatctgttccaccattgaagacctcagacggcaaaaatga

>H1N1_PB1_CY123927_A/Singapore/ON355/2009

atggatgtcaatccgactctacttttcctaaaaattccagcgcaaaatgccataagcaccacattcccttatactggagatcctccatacagccatggaacaggaacaggatacaccatggacacagtaaacagaacacaccaatactcagaaaagggaaagtggacgacaaacacagagactggtgcaccccagctcaacccgattgatggaccactacctgaggataatgaaccaagtgggtatgcacaaacagactgtgttctagaggctatggctttccttgaagaatcccacccaggaatatttgagaattcatgccttgaaacaatggaagttgttcaacaaacaagggtagataaactaactcaaggtcgccagacttatgattggacattaaacagaaatcaaccggcagcaactgcattggccaacaccatagaagtctttagatcgaatggcctaacagctaatgagtcaggaaggctaatagatttcttaaaggatgtaatggaatcaatgaacaaagaggaaatagagataacaacccactttcaaagaaaaaggagagtaagagacaacatgaccaagaagatggtcacgcaaagaacaatagggaagaaaaaacaaagactgaataagagaggctatctaataagagcactgacattaaatacgatgaccaaagatgcagagagaggcaagttaaaaagaagggctatcgcaacacctgggatgcagattagaggtttcgtatactttgttgaaactttagctaggagcatttgcgaaaagcttgaacagtctgggctcccagtagggggcaatgaaaagaaggccaaactggcaaatgttgtgagaaagatgatgactaattcacaagacacagagatttctttcacaatcactggggacaacactaagtggaatgaaaatcaaaatcctcgaatgttcctggcgatgattacatatatcaccagaaatcaacccgagtggttcagaaacatcctgagcatggcacccataatgttctcaaacaaaatggcaagactagggaaagggtacatgttcgagagtaaaagaatgaagattcgaacacaaataccagcagaaatgctagcaagcattgacctgaagtacttcaatgaatcaacaaagaagaaaattgagaaaataaggcctcttctaatagatggcacagcatcactgagtcctgggatgatgatgggcatgttcaacatgctaagtacggtcttaggagtctcgatactgaatcttggacaaaagaaatacaccaagacaatatactggtgggatgggctccaatcatccgacgattttgctctcatagtgaatgcaccaaaccatgagggaatacaagcaggagtggacagattctacaggacctgcaagttagtgggaatcaacatgagcaaaaagaagtcctatataaataagacagggacatttgaattcacaagctttttttatcgctatggatttgtggctaattttagcatggagctacccagctttggagtgtctggagtaaatgaatcagctgacatgagtattggagtaacagtgataaagaacaacatgataaacaatgaccttggacctgcaacggcccagatggctcttcaattgttcatcaaagactacagatacacatataggtgccataggggagacacacaaattcagacgagaagatcatttgagttaaagaagctgtgggatcaaacccaatcaaaggtagggctattagtatcagatggaggaccaaacttatacaatatacggaatcttcacattcctgaagtctgcttaaaatgggagctaatggatgatgattatcggggaagactttgtaatcccctgaatccctttgtcagtcataaagagattgattctgtaaacaatgctgtggtaatgccagcccatggtccagccaaaagcatggaatatgatgccgttgcaactacacattcctggattcccaagaggaatcgttctattctcaacacaagccaaaggggaattcttgaggatgaacagatgtaccagaagtgctgcaatctattcgagaaatttttccctagcagttcatataggagaccggttggaatttctagcatggtggaggccatggtgtctagggcccggattgatgccagggtcgacttcgagtctggacggatcaagaaagaagagttctctgagatcatgaagatctgttccaccattgaagaactcagacggcaaaaataa

>H1N1_PB1_CY124716_A/Singapore/KK160/2011

atggatgtcaatccgactctacttttcctaaaaattccagcgcaaaatgccataagcaccacattcccttatactggagatcctccatacagccatggaacaggaacaggatacaccatggacacagtaaacagaacacaccaatactcagaaaagggaaagtggacgacaaacacagagactggtgcaccccagctcaacccgattgatggaccactacctgaggataatgaaccaagtgggtatgcacaaacagactgtgttctagaggctatggctttccttgaagaatcccacccaggaatatttgagaattcatgccttgaaacaatggaagttgttcaacaaacaagggtagataaactgactcaaggtcgccagacttatgattggacattaaacagaaatcaaccggcagcaactgcattggccaacaccatagaagtctttagatcgaatggcctaacagctaatgagtcaggaaggctaatagatttcttaaaggatgtaatggaatcaatgaacaaagaggaaatagagataacaacccactttcaaagaaaaaggagagtaagagacaacatgaccaagaagatggtcacgcaaagaacaatagggaagaaaaaacagagactgaataagagagactatctaataagagcactgacattaaatacgatgaccaaagatgcagagagaggcaagttaaaaagaagggctatcgcaacacctgggatgcagattagaggtttcgtatactttgttgaaactttagctaggagcatttgcgaaaagcttgaacagtctgggctcccagtagggggcaatgaaaagaaggccaaactggcaaatgttgtgagaaagatgatgactaattcacaagacacagagatttctttcacaatcactggggacaacactaagtggaatgaaaatcaaaatcctagaatgttcctggcgatgattacatatatcaccagaaatcaacccgagtggtttagaaacatcctgagcatggcacccataatgttctcaaacaaaatggcaagactagggaaagggtacatgttcgagagtaaaagaatgaagattcgaacacaaataccagcagaaatgctagcaagcattgacctgaagtacttcaatgaatcaacaaagaagaaaattgagaaaataaggcctcttttaatggatggcacagcatcactgagtcctgggatgatgatgggcatgttcaacatgctaagtacggtcttgggagtatcgatactgaatcttggacaaaagaaatacaccaagacaacatactggtgggatgggctccaatcatccgacgattttgctctcatagtgaatgcaccaaaccatgagggaatacaagcaggagtggacagattctacaggacctgcaagttagtgggaatcaacatgagcaaaaagaagtcctatataaataagacagggacatttgaattcacaagctttttttatcgctatggatttgtggctaattttagcatggagctacccagctttggagtgtctggagtaaatgaatcagctgacatgagtattggagtaacagtgataaagaacaacatgataaacaatgaccttggacctgcaacggcccagatggctcttcaattgttcatcaaagactacagatacacatataggtgccataggggagacacacaaattcagacgagaagatcatttgagttaaagaagctgtgggatcaaacccaatcaaaggtggggctattagtatcagatggaggaccaaacttatacaatatacggaatcttcacattcctgaagtctgcttaaaatgggagctaatggatgatgattatcggggaagactttgtaatcccctgaatccctttgtaagtcataaggagattgattctgtaaacaatgctgtggtaatgccagcccatggtccagccaaaagcatggaatatgatgccgttgcaactacacattcctggattcccaagaggaatcgttctattctcaacacaagccaaaggggaattcttgaggatgaacagatgtaccagaagtgctgcaatctattcgagaaatttttccctagcagttcatataggagaccggttggaatttctagcatggtggaggccatggtgtctagggcccggattgatgccagggtcgactttgagtctggacggatcaagaaagaagagttctctgagatcatgaagatctgttccaccattgaagagctcagacggcaaaaataa

>H1N1_PB1_AB704516_A/Yamaguchi/247/2009

atggatgtcaatccgactctacttttcctaaaaattccagcgcaaaatgccataagcaccacattcccttatactggagatcctccatacagccatggaacaggaacaggatacaccatggacacagtaaacagaacacaccaatattcagaaaagggaaagtggacgacaaacacagagactggtgcaccccagctcaacccgattgatggaccactacctgaggataatgaaccaagtgggtatgcacaaacagactgtgttctagaggctatggctttccttgaagaatcccacccaggaatatttgagaattcatgccttgaaacaatggaagttgttcaacaaacaagggtagataaactaactcaaggtcgccagacttatgattggacattaaacagaaatcaaccggcagcaactgcattggccaacaccatagaagtctttagatcgaatggcctaacagctaatgagtcaggaaggctaatagatttcttaaaggatgtaatggaatcaatgaacaaagaggaaatagagataacaacccactttcaaagaaaaaggagagtaagagacaacatgaccaagaagatggtcacgcaaagaacaatagggaagaaaaaacaaagactgaataagagaggctatctaataagagcactgacattaaatacgatgaccaaagatgcagagagaggcaagttaaaaagaagggctatcgcaacacctgggatgcagattagaggtttcgtatactttgttgaaactttagctaggagcatttgcgaaaagcttgaacagtctgggctcccagtagggggcaatgaaaagaaggccaaactggcaaatgttgtgagaaagatgatgactaattcacaagacacagagatttctttcacaatcactggggacaacactaagtggaatgaaaatcaaaatcctcgaatgttcctggcgatgattacatatatcaccagaaatcaacccgagtggttcagaaacatcctgagcatggcacccataatgttctcaaacaaaatggcaagactagggaaagggtacatgttcgagagtaaaagaatgaagattcgaacacaaataccagcagaaatgctagcaagcattgacctgaagtacttcaatgactcaacaaagaagaaaattgagaaaataaggcctcttctaatagatggcacagcatcactgagtcctgggatgatgatgggaatgttcaacatgctaagtacggtcttgggagtctcgatactgaatcttggacaaaagaaatacaccaagacaatatactggtgggatgggctccaatcatccgacgattttgctctcatagtgaatgcaccaaaccatgagggaatacaagcaggagtggacagattctacaggacctgcaagttagtgggaatcaacatgagcaaaaagaagtcctatataaataagacagggacatttgaattcacaagctttttttatcgctatggatttgtggctaattttagcatggagctacccagctttggagtgtctggagtaaatgaatcagctgacatgagtattggagtaacagtgataaagaacaacatgataaacaatgaccttggacctgcaacggcccagatggctcttcaattgttcatcaaagactacagatacacatataggtgccataggggagacacacaaattcagacgagaagatcatttgagttaaagaagctgtgggatcaaacccaatcaaaggtagggctattagtatcagatggaggaccaaacttatacaatatacggaatcttcacattcctgaagtctgcttaaaatgggagctaatggatgatgattatcggggaagactttgtaatcccctgaatccctttgtcagtcataaagagattgattctgtaaacaatgctgtggtaatgccagcccatggtccagtcaaaagcatggaatatgatgccgttgcaactacacattcctggattcccaagaggaatcgttctattctcaacacaagccaaaggggaattcttgaggatgaacagatgtaccagaagtgctgcaatctattcgagaaatttttccctagcagttcatataggagaccggttggaatttctagcatggtggaggccatggtgtctagggcccggattgatgccagggtcgacttcgagtctggacggatcaagaaagaagagttctctgagatcatgaagatctgctccaccattgaagaactcagacggcaaaaataa

>H1N1_PB1_CY125018_A/Nanchang/16A/1999

atggatgtcaatccgaccttacttttcttaaaagtgccagcacaaaatgctataagcacaacttttccttacactggagaccctccttacagccatgggacaggaacagggtacaccatggatacagtcaaccggacacatcagtactcagaaagaggaagatggacaaaaaataccgaaactggagcaccgcaactcaacccaattgatgggccactaccaaaagacaatgaaccaagtggctatgcccaaacagattgtgtattagaagcaatggctttccttgaggaatcccatcctggtatttttgaaaactcttgtattgaaacaatggaggttgttcagcaaacaagggtggacaaactgacacaaggcagacagacctatgactggactctaaatagaaaccagcctgctgccacagcattggccaacactatagaagtattcagatcaaacggcctcatagcaaatgaatctgggaggctaatagacttccttaaagatgtaatggagtcgatggacagagacgaagtagagatcacaactcattttcagagaaagaggagagtgagagacaatgtaactaaaaaaatggtgacccaaagaacaataggcaaaaagaaacataaattagacaaaagaagttacctaattagggcattaaccctgaacacaatgaccaaagatgctgagagggggaaactaaaacgtagagcaattgcaaccccaggaatgcaaataagggggtttgtatactttgttgagacactggcaaggagcatatgtgaaaagcttgaacaatcagggttgccagttggaggaaatgaaaagaaagcaaagttagcaaatgttgtaaggaagatgatgaccaactcccaggacactgaaatttctttcaccatcactggagataacacaaaatggaacgaaaatcaaaaccctagaatgttcttggccatgatcacatatataaccaaaaatcagcctgaatggttcaggaatattctaagtattgctccaataatgttttcaaacaaaatggcgagactaggtaaggggtacatgtttgaaagcaagagtatgaaattgagaactcaaatacctgcagagatgctagccaacatagatttgaaatatttcaatgattcaacaaaaaagaaaattgaaaaaatccggccattattaatagatggaactgcatcattgagtcctggaatgatgatgggcatgttcaatatgttaagcactgtcttgggcgtctccattctgaatcttgggcaaaagagatacaccaagactacttactggtgggatggtcttcaatcgtctgatgattttgctctgattgtgaatgcacccaactatgcaggaatccaagctggagttgacaggttttatcgaacctgtaagctgctcggaattaatatgagcaaaaagaagtcttacataaacagaacaggtacctttgaattcacgagctttttctatcgttatgggtttgttgccaatttcagcatggagcttcctagttttggggtgtctggggtcaatgagtctgcagacatgagtattggagtcactgtcatcaaaaacaatatgataaacaatgaccttggcccagcaactgctcaaatggcccttcagttatttatcaaagattacaggtacacgtatcgatgccacagaggtgacacacaaatacaaacccggagatcatttgagataaagaaactatgggaccaaacccgctccaaagctgggctgttggtctctgatggaggccccaatttatataacattagaaatctccacattcctgaagtctgcttgaaatgggagttgatggatgaggattaccaggggcgtttgtgcaacccattgaacccgtttgtcagtcataaagagattgaatcagtgaacaatgcagtgatgatgccggcacatggtccagccaaaaatatggagtatgacgctgttgcaacaacacactcctgggttcccaaaaggaatcgatccattttgaatacgagccaaagggggatacttgaggatgagcaaatgtatcagaggtgctgcaatttatttgaaaaattcttcccaagtagctcatacagaagaccagttggaatatccagtatggtagaggctatggtttccagagcccgaattgatgcacggattgatttcgaatctggaaggataaagaaagaggagttcgctgagatcatgaagacctgttccaccattgaagacctcagacggcaaaaatag

>H1N1_PB1_CY125042_A/Wellington/47/1992

atggatgtcaatccgaccttacttttcttaaaagtgccagcacaaaatgctataagcacaactttcccctatactggagaccccccttacagccatgggacagggacagggtacaccatggatacagtcaacaggacacatcagtactcagaaagaggaagatggacaaaaaataccgaaactggagcaccgcaactcaacccgattgatgggccactaccaaaagacaatgaaccaagtggctatgcccaaacagattgtgtattagaagcaatggctttccttgaggaatcccaccctggtatttttgaaaactcgtgtattgaaacaatggaggttgttcagcaaacaagagtggacaaactgacacaaggcagacagacctatgactggactctaaatagaaaccagcctgctgccacagcattggccaacactatagaagtgttcagatcaaacggcctcatagcaaatgaatctgggaggctaatagacttccttaaagatgtaatggagtcaatggacagagaagaagtagagatcacaactcattttcagagaaagagaagagtgagagacaatgtaactaaaaaaatggtgacacaaagaacaataggcaaaaagaaacataaattggacaaaagaagttatctaattagggcattaaccctgaacacaatgaccaaagatgctgagagggggaaactaaaacgtagagcaattgcaaccccaggaatgcaaataagggggtttgtatactttgttgagacactggcaaggagcatatgtgaaaagcttgaacaatcagggttgccagttggaggaaatgagaagaaagcaaagttagcaaatgttgtaaggaagatgatgaccaactcccaggacactgaaatttctttcaccatcactggagataacacaaaatggaacgaaaatcagaaccctagaatgtttttggctatgatcacatatataaccagaaatcagcctgaatggttcagaaacattctaagtattgctccaataatgttttcaaacaaaatggcgagactaggtaaggggtacatgtttgaaagcaagagtatgaaactgagaactcaaatacctgcagaaatgctagccaacatagatttgaaatatttcaatgattcaacaaaaaagaaaattgaaaaaatccggccactattaatagatggaactgcatcactgagccctggaatgatgatgggcatgttcaatatgttaagcactgtcttgggcgtctccatcctgaatcttgggcaaaagagatacaccaagactacttactggtgggatggtcttcaatcgtctgatgattttgctctgattgtgaatgcacccaactatgcaggaattcaagctggagttgacaggttttatcgaacctgtaagctgctcggaattaatatgagcaaaaagaagtcttacataaacaggacaggtacttttgaattcacgagctttttctatcgttatgggtttgttgccaatttcagcatggaacttcctagttttggggtgtctggggtcaatgagtctgcagacatgagtattggagtcactgtcatcaaaaacaatatgataaacaatgatcttggtccagcaactgctcaaatggcccttcagttattcatcaaagattacaggtacacgtatcgatgccacagaggtgacacacaaatacaaacccggagatcatttgagataaagaaactgtgggaccaaacccgctccaaagctgggctgttggtctctgatggaggccccaatttatataatattagaaatctccacattcctgaagtctgcttgaaatgggagttgatggatgaggattaccaggggcgtttatgcaatccattgaacccgtttgtcagtcataaagaaattgaatcagtgaacaatgcagtgatgatgccggcacatggtccagccaaaaatatggagtatgacgctgttgcaacaacacactcctgggtccccaaaaggaatcgatccattttgaatacgagccaaagaggaatacttgaggatgagcaaatgtatcaaaggtgctgcaatttatttgaaaaattcttcccaagcagctcatacagaagaccagttggaatatccagtatggtagaggccatggtttccagagcccgaattgatgcacggattgatttcgaatctggaaggataaagaaagaggagttcgctgagatcatgaagacctgttccaccattgaagacctcagacggcaaaaatag

>H1N1_PB1_JX309063_A/Singapore/GP489/2010

atggatgtcaatccgactctacttttcctaaaaattccagcgcaaaatgccataagcaccacattcccttatactggagatcctccatacagccatggaacaggaacaggatacaccatggacacagtaaacagaacacaccaatactcagaaaagggaaagtggacgacaaacacagagactggtgcaccccagctcaacccgattgatggaccactacctgaggataatgaaccaagtgggtatgcacaaacagactgtgttctagaggctatggctttccttgaagaatcccacccaggaatatttgagaattcatgccttgaaacaatggaagttgttcaacaaacaagggtagataaactaactcaaggtcgccagacttatgattggacattaaacagaaatcaaccggcagcaactgcattggccaacaccatagaagtctttagatcgaatggcctaacagctaatgagtcaggaaggctaatagatttcttaaaggatgtaatggaatcaatgaacaaagaggaaatagagataacaacccactttcaaagaaaaaggagagtaagagacaacatgaccaagaagatggtcacgcaaagaacaatagggaagaaaaaacaaagactgaataagagaggctatctaataagagcactgacattaaatacgatgaccaaagatgcagagagaggcaagttaaaaagaagggctatcgcaacacctgggatgcagattagaggtttcgtatactttgttgaaactttagctaggagcatttgcgaaaagcttgaacagtctgggctcccagtagggggcaatgaaaagaaggccaaactggcaaatgttgtgagaaagatgatgactaattcacaagacacagagatttctttcacaatcactggggacaacactaagtggaatgaaaatcaaaatcctcgaatgttcctggcgatgattacatatatcaccagaaatcaacccgagtggttcagaaacatcctgagcatggcacccataatgttctcaaacaaaatggcaagactagggaaagggtacatgttcgagagtaaaagaatgaagattcgaacacaaatacctgcagaaatgctagcaagcattgacctgaagtacttcaatgaatcaacaaagaagaaaattgagaaaataaggcctcttctaatagatggcacagcatcactgagtcctgggatgatgatgggcatgttcaacatgctaagtacggtcttgggagtctcgatactgaatcttggacaaaagaaatacaccaagacaatatactggtgggatgggctccaatcatccgacgattttgctctcatagtgaatgcaccaaaccatgagggaatacaagcaggagtggacagattctacaggacctgcaagttagtgggaatcaacatgagcaaaaagaagtcctatataaataagacagggacatttgaattcacaagctttttttatcgctatggatttgtggctaattttagcatggagctacccagctttggagtgtctggagtaaatgaatcagctgacatgagtattggagtaacagtgataaagaacaacatgataaacaatgaccttggacctgcaacggcccagatggctcttcaattgttcatcaaagactacagatacacatataggtgccataggggagacacacaaattcagacgagaagatcatttgagttaaagaagctgtgggatcaaacccaatcaaaggcagggctattagtatcagatggaggaccaaacttatacaatatacggaatcttcacattcctgaagtctgcttaaaatgggagctaatggatgatgattatcggggaagactttgtaatcccctgaatccctttgtcagtcataaagagattgattctgtaaacaatgctgtggtaatgccagcccatggtccagccaaaagcatggaatatgatgccgttgcaactacacattcctggattcccaagaggaatcgttctattctcaacacaagccaaaggggaattcttgaggatgaacagatgtaccagaagtgctgcaatctattcgagagatttttccctagcagttcatataggagaccggttggaatttctagcatggtggaggccatggtgtctagggcccggattgatgccagggtcgacttcgagtctggacggatcaagaaagaagagttctctgagatcatgaagatctgttccaccattgaagaactcagacggcaaaaataa

>H1N1_PB1_CY069747_A/England/527/2009

atggatgtcaatccgactctacttttcctaaaaattccagcgcaaaatgccataagcaccacattcccttatactggagatcctccatacagccatggaacaggaacaggatacaccatggacacagtaaacagaacacaccaatactcagaaaagggaaagtggacgacaaacacagagactggtgcaccccagctcaacccgattgatggaccactacctgaggataatgaaccaagtgggtatgcacaaacagactgtgttctagaggctatggctttccttgaagaatcccacccaggaatatttgagaattcatgccttgaaacaatggaagttgttcaacaaacaagggtagataaactaactcaaggtcgccagacttatgattggacattaaacagaaatcaaccggcagcaactgcattggccaacaccatagaagtctttagatcgaatggcctaacagctaatgagtcaggaaggctaatagatttcttaaaggatgtaatagaatcaatgaacaaagaggaaatagagataacaacccactttcaaagaaaaaggagagtaagagacaacatgaccaagaagatggtcacgcaaagaacaatagggaagaaaaaacaaagactgaataagagaggctatctaataagagcactgacattaaatacgatgaccaaagatgcagagagaggcaagttaaaaagaagggctatcgcaacacctgggatgcagattagaggtttcgtatactttgttgaaactttagctaggagcatttgcgaaaagcttgaacagtctgggctcccagtagggggcaatgaaaagaaggccaaactggcaaatgttgtgagaaagatgatgactaattcacaagacacagagatttctttcacaatcactggggacaacactaagtggaatgaaaatcaaaatcctcgaatgttcctggcgatgattacatatatcaccagaaatcaacccgagtggttcagaaacatcctgagcatggcacccataatgttctcaaacaaaatggcaagattagggaaagggtacatgttcgagagtaaaagaatgaagattcgaacacaaataccagcagaaatgctagcaagcattgacctgaagtacttcaatgaatcaacaaagaagaaaattgagaaaataaggcctcttctaatagatggcacagcatcactgagtcctgggatgatgatgggcatgttcaacatgctaagtacggtcttgggagtctcgatactgaatcttggacaaaagaaatacaccaagacaatatactggtgggatgggctccaatcatccgacgattttgctctcatagtgaatgcaccaaaccatgagggaatacaagcaggagtggacagattctacaggacctgcaagttagtgggaatcaacatgagcaaaaagaagtcctatataaataagacagggacatttgaattcacaagctttttttatcgctatggatttgtggctaattttagcatggagctacccagcttcggagtgtctggagtaaatgaatcagctgacatgagcattggagtaacagtgataaagaacaacatgataaacaatgaccttggacctgcaacggcccagatggctcttcaattgttcatcaaagactacagatacacatataggtgccataggggagacacacaaattcagacgagaagatcatttgagttaaagaagctgtgggatcaaacccaatcaaaggtagggctattagtatcagatggaggaccaaacttatacaatatacggaatcttcacattcctgaagtctgcttaaaatgggagctaatggatgatgattatcggggaagactttgtaatcccctgaatccctttgtcagtcataaagagattgattctgtaaacaatgctgtggtaatgccagcccatggtccagccaaaagcatggaatatgatgccgttgcaactacacattcctggattcccaagaggaatcgttctattctcaacacaagccaaaggggaattcttgaggatgaacagatgtaccagaagtgctgcaatctattcgagaaatttttccctagcagttcatataggagaccggttggaatttctagcatggtggaggccatggtgtctagggcccggattgatgccagggtcgacttcgagtctggacggatcaagaaagaagagttctctgagatcatgaagatctgttccaccattgaagaactcagacggcaaaaataa

>H1N1_PB1_JX625589_A/Northern Ireland/94620068/2009

atggatgtcaatccgactctacttttcctaaaaattccagcgcaaaatgccataagcaccacattcccttatactggagatcctccatacagccatggaacaggaacaggatacaccatggacacagtaaacagaacacaccaatactcagaaaagggaaagtggacgacaaacacagagactggtgcaccccagctcaacccgattgatggaccactacctgaggataatgaaccaagtgggtatgcacaaacagactgtgttctagaggctatggctttccttgaagaatcccacccaggaatatttgagaattcatgccttgaagcaatggaagttgttcaacaaacaagggtagataaactaactcaaggtcgccagacttatgattggacattaaacagaaatcaaccggcagcaactgcattggccaacaccatagaagtctttagatcgaatggcctaacagctaatgagtcaggaaggctaatagatttcttaaaggatgtaatggaatcaatgaacaaagaggaaatagagataacaacccactttcaaagaaaaaggagagtaagagacaacatgaccaagaagatggtcacgcaaagaacaatagggaagaaaaaacaaagactgaataagagaggctatctaataagagcactgacattaaatacgatgaccaaagatgcagagagaggcaagttaaaaagaagggctatcgcaacacctgggatgcagattagaggtttcgtatactttgttgaaactttagctaggagcatttgcgaaaagcttgaacagtctgggctcccagtagggggcaatgaaaagaaggccaaactggcaaatgttgtgagaaagatgatgactaattcacaagacacagagatttctttcacaatcactggggacaacactaagtggaatgaaaatcaaaatcctcgaatgttcctggcgatgattacatatatcaccagaaatcaacccgagtggttcagaaacatcctgagcatggcacccataatgttctcaaacaaaatggcaagactagggaaagggtacatgttcgagagtaaaagaatgaagattcgaacacaaataccagcagaaatgctagcaagcattgacctgaagtacttcaatgaatcaacaaagaagaaaattgagaaaataaggcctcttctaatagatggcacagcatcactgagtcctgggatgatgatgggcatgttcaacatgctaagtacggtcttgggagtctcgatactgaatcttggacaaaagaaatacaccaagacaatatactggtgggatgggctccaatcatccgacgattttgctctcatagtgaatgcaccaaaccatgagggaatacaagcaggagtggacagattctacaggacctgcaagttagtgggaatcaacatgagcaaaaagaagtcctatataaataagacagggacatttgaattcacaagctttttttatcgctatggatttgtggctaattttagcatggagctacccagctttggagtgtctggagtaaatgaatcagctgacatgagtattggagtaacagtgataaagaacaacatgataaacaatgatcttggacctgcaacggcccagatggctcttcaattgttcatcaaagactacagatacacatataggtgccataggggagacacacaaattcagacgagaagatcatttgagttaaagaagctgtgggatcaaacccaatcaaaggtagggctattagtatcagatggaggaccaaacttatacaatatacggaatcttcacattcctgaagtctgcttaaaatgggagctaatggatgatgattatcggggaagactttgtaatcccctgaatccctttgtcagtcataaagagattgattctgtaaacaatgctgtggtaatgccagcccatggtccagccaaaagcatggaatatgatgccgttgcaactacacattcctggattcccaagaggaatcgttctattctcaacacaagccaaaggggaattcttgaggatgaacagatgtaccagaagtgctgcaatctattcgagaaatttttccctagcagttcatataggagaccggttggaatttctagcatggtggaggccatggtgtctagggcccggattgatgccagggtcgacttcgagtctggacggatcaagaaagaagagttctctgagatcatgaagatctgttccaccattgaagaactcagacggcaaaaataa

>H1N1_PB1_CY128329_A/Viet Nam/13032093/2009

atggatgtcaatccgactctacttttcctaaaaattccagcgcaaaatgccataagcaccacattcccttatactggagatcctccatacagccatggaacaggaacaggatacaccatggacacagtaaacagaacacaccaatactcagaaaagggaaagtggacgacaaacacagagactggtgcaccccagctcaacccgattgatggaccactacctgaggataatgaaccaagtgggtatgcacaaacagactgtgttctagaggctatggctttccttgaagaatcccacccaggaatatttgagaattcatgccttgaaacaatggaagttgttcaacaaacaagggtagataaactaactcagggtcgccagacttatgattggacattaaacagaaatcaaccggcagcaactgcattggccaacaccatagaagtctttagatcgaatggcctaacagctaatgagtcaggaaggctaatagatttcttaaaggatgtaatggaatcaatgaacaaagaggaaatagagataacaacccactttcaaagaaaaaggagagtaagagacaacatgaccaagaagatggtcacgcaaagaacaatagggaagaaaaaacaaagactgaataagagaggctatctaataagagcactgacattaaatacgatgaccaaagatgcagagagaggcaagttaaaaagaagggctatcgcaacacctgggatgcagattagaggtttcgtatactttgttgaaactttagctaggagcatttgcgaaaagcttgaacagtctgggctcccagtagggggcaatgaaaagaaggccaaactggcaaatgttgtgagaaagatgatgactaattcacaagacacagagatttctttcacaatcactggggacaacactaagtggaatgaaaatcaaaatcctcgaatgttcctggcgatgattacatatatcaccagaaatcaacccgagtggttcagaaacatcctgagcatggcacccataatgttctcaaacaaaatggcaagactagggaaagggtacatgttcgagagtaaaagaatgaagattcgaacacaaataccagcagaaatgctagcaagcattgacctgaagtacttcaatgaatcaacaaagaaaaaaattgagaaaataaggcctcttctaatagatggcacagcatcactgagtcctgggatgatgatgggcatgttcaacatgctaagtacggtcttgggagtctcgatactgaatcttggacaaaagaaatacaccaagacaatatactggtgggatgggctccaatcatccgacgattttgctctcatagtgaatgcaccaaaccatgagggaatacaagcaggagtagacagattctacaggacctgcaagttagtgggaatcaacatgagcaaaaagaagtcctatataaataagacagggacatttgaattcacaagctttttttatcgctatggatttgtggctaattttagcatggagctacccagctttggagtgtctggagtaaatgaatcagctgacatgagtattggagtaacagtgataaagaacaacatgataaacaatgaccttggacctgcaacggcccagatggctcttcaattgttcatcaaagactacagatacacatataggtgccataggggagacacacaaattcagacgagaagatcatttgagttaaagaagctgtgggatcaaacccaatcaaaggtagggctattagtatcagatggaggaccaaacttatacaatatacggaatcttcacattcctgaagtctgcttaaaatgggagctaatggatgatgattatcggggaagactttgtaatcccctgaatccctttgtcagtcataaagagattgattctgtaaataatgctgtggtaatgccagcccatggtccagccaaaagcatggaatatgatgccgttgcaactacacattcctggattcccaagaggaatcgttctattctcaacacaagccaaaggggaattcttgaggatgaacagatgtaccagaagtgctgcaatctattcgagaaatttttccctagcagttcatataggagaccggttggaatttctagcatggtggaggccatggtgtctagggcccggattgatgccagggtcgacttcgagtctggacggatcaagaaagaagagttctctgagatcatgaagatctgttccaccattgaagaactcagacggcaaaaataa

>H1N1_PB1_CY129652_A/Sheffield/INS545/2011

atggatgtcaatccgactctacttttcctaaaaattccagcgcaaaatgccataagcaccacattcccttatactggagatcctccatacagccatggaacaggaacaggacacaccatggacacagtaaacagaacacaccaatactcagaaaagggaaagtggacgacaaacacagagactggtgcaccccagctcaacccgattgatggaccactacctgaggataatgaaccaagtgggtatgcacaaacagactgtgttctagaggctatggctttccttgaagaatcccacccaggaatatttgagaattcatgccttgaaacaatggaagttgttcaacaaacaagggtagataaactaactcaaggtcgccagacttatgattggacattaaacagaaatcaaccggcagcaactgcattggccaacaccatagaagtctttagatcgaatggcctaacagctaatgagtcaggaaggctaatagatttcttaaaggatgtaatggaatcaatgaacaaagaggaaatagagataacaacccactttcaaagaaaaaggagagtaagagacaacatgaccaagaagatggtcacgcaaagaacaatagggaagaaaaaacaaagactgaataagagaggctatctaataagagcactgacattaaatacgatgaccaaagatgcagagagaggcaagttaaaaagaagggctatcgcaacacctgggatgcagattagaggtttcgtatactttgttgaaactttagctaggagcatttgcgaaaagcttgaacagtctgggctcccagtagggggcaatgaaaagaaggccaaactggcaaatgttgtgagaaagatgatgactaattcacaagacacagagatttctttcacaatcactggggacaacactaagtggaatgaaaaccaaaatcctcgaatgttcctggcgatgattacatatatcaccagaaatcaacccgagtggttcagaaacatcctgagcatggcacccataatgttctcaaacaaaatggcaagactagggaaagggtacatgttcgagagtaaaagaatgaagattcgaacgcaaataccagcagaaatgctagcaagcattgacctgaagtacttcaatgaatcaacaaagaagaaaattgagaaaataaggcctcttttaatagatggcacagcatcactgagccctgggatgatgatgggcatgttcaacatgctaagtacggtcttgggagtctcgatactgaatcttggacaaaagaaatacaccaagacaatatactggtgggatgggctccaatcatccgacgattttgctctcatagtgaatgcaccaaaccatgagggaatacaagcaggagtggacagattttacaggacctgcaagttagtgggaatcaacatgagcaaaaagaagtcctatataaataagacagggacatttgaattcacaagctttttttatcgctatggatttgtggctaattttagcatggagctacccagctttggagtgtctggagtaaatgaatcagctgacatgagtattggagtaacagtgataaagaacaacatgataaacaatgaccttggacctgcaacggcccagatggctcttcaattgttcatcaaagactacagatacacatataggtgccataggggagacacacaaattcagacgagaagatcatttgagttaaagaagctgtgggatcaaacccaatcaaaggtagggctattagtatcagatggaggaccaaacttatacaatatacggaatcttcacattcctgaagtctgcttaaaatgggagctaatggatgatgactatcggggaagactttgtaatcccctgaatccctttgtaagtcataaagagattgattctgtaaacaatgctgtgataatgccagctcatggtccagccaaaagcatggaatatgatgccgttgcaactacacattcctggattcccaagaggaatcgttctattctcaacacaagccaaaggggaattcttgaggatgaacagatgtaccagaagtgctgcaatctattcgagaaatttttccctagcagttcatataggagaccggttggaatttctagcatggtggaggccatggtgtctagggcccggattgatgccagggtcgactttgagtctggacggatcaagaaagaagagttctctgagatcatgaagatctgttccaccattgaagaactcagacggcaaaaataa

>H1N1_PB1_CY129676_A/Athens/INS552/2011

atggatgtcaatccgactctacttttcctaaaaattccagcgcaaaatgccataagcaccacattcccttatactggagatcctccatacagccatggaacaggaacaggatacaccatggacacagtaaacagaacacaccaatactcagaaaagggaaagtggacgacaaacacagagactggtgcaccccagctcaacccgattgatggaccactacctgaggataatgaaccaagtgggtatgcacagacagactgtgttctagaggctatggctttccttgaagaatcccacccaggaatatttgagaattcatgccttgaaacaatggaagctgttcaacaaacaagggtagataaactaactcaaggtcgccagacttatgattggacattaaacagaaatcaaccggcagcaactgcattggccaacaccatagaagtctttagatcgaatggcctaacagctaatgagtcaggaaggctaatagatttcttaaaggatgtaatggaatcaatgaacaaagaggaaatagagataacaacccactttcaaagaaaaaggagagtaagagacaacatgaccaagaagatggtcacgcaaagaacaatagggaagaaaaaacaaagactgaataagagaggctatctaataagagcactgacattaaatacgatgaccaaagatgcagagagaggcaagttaaaaagaagggctattgcaacacctgggatgcagattagaggtttcgtatactttgttgaaactttagctaggagcatttgcgaaaagcttgaacagtctgggctcccagtagggggcaatgaaaagaaggccaaactggcaaatgttgtgagaaagatgatgactaattcacaagacacagagatttctttcacaatcactggggacaacactaagtggaatgaaaatcaaaatcctcgaatgttcctggcgatgattacatatatcaccagaaatcaacccgagtggttcagaaacatcctgagcatggcacccataatgttctcaaacaaaatggcaagactagggaaagggtacatgttcgagagtaaaagaatgaggattcgaacacaaataccagcagaaatgctagcaagcattgacctgaagtacttcaatgaatcaacaaagaagaaaattgagaaaataaggcctcttctaatagatggcacagcatcactgagtcctgggatgatgatgggcatgttcaacatgctaagtacggtcttgggagtctcgatactgaatcttggacaaaagaaatacaccaagacaacatactggtgggatgggctccaatcatccgacgattttgctctcatagtgaatgcaccaaaccatgagggaatacaagcaggagtggacagattctacaggacctgcaagttagtgggaatcaacatgagcaaaaagaagtcatatataaataaaacagggacatttgaattcacaagctttttttatcgctatggatttgtggctaattttagcatggagctacccagctttggagtgtctggagtaaatgaatcagctgacatgagtattggagtaacagtgataaagaacaacatgataaacaatgaccttggacctgcaacagcccagatggctcttcaattgttcatcaaagactacagatacacatataggtgccataggggagacacacaaattcagacgagaagatcatttgagttaaaaaagctgtgggatcaaacccaatcaaaggtagggttattagtatcagatggaggaccaaacttatacaatatacggaatcttcacattcctgaagtctgcttaaaatgggagctaatggatgatgattatcggggaagactttgtaatcccctgaatccctttgtcagtcataaagagattgattctgtaaacaatgctgtggtaatgccagcccatggtccagccaaaagcatggaatatgatgccgttgcaactacacattcctggattcccaagaggaatcgttctattctcaacacaagccaaaggggaattcttgaggatgaacagatgtaccagaagtgctgcaatctattcgagaaatttttccctagcagttcatataggagaccggttggaatttctagcatggtggaggccatggtgtctagggcccggattgatgccagggtcgacttcgagtctggacggatcaagaaagaagagttctctgagatcatgaagatctgttccaccattgaagaactcagacggcaaaaataa

>H1N1_PB1_CY129772_A/Bangkok/INS581/2010

atggatgtcaatccgactctacttttcctgaaaattccagcgcaaaatgccataagcaccacattcccttatactggagatcctccatacagccatggaacaggaacaggatacaccatggacacagtaaacagaacacaccaatactcagaaaagggaaagtggacgacaaacacagagactggagcaccccagctcaacccgattgatggaccactacctgaggataatgaaccaagtgggtatgcacaaacagactgtgttctagaggctatggctttccttgaagaatcccacccaggaatatttgagaattcatgccttgaaacaatggaagttgttcaacaaacaagggtagataagctaactcaaggtcgccaaacttatgattggacattaaacagaaatcaaccggcagcaactgcattggccaacaccatagaagtctttagatcgaatggcctaacagctaatgagtcaggaaggctaatagatttcttaaaggatgtaatggaatcaatgaacaaagaggaaatagagataacaacccactttcaaagaaaaaggagagtaagagacaacatgaccaagaagatggtcacgcaaagaacaatagggaagaaaaaacaaaaactgaataagagaggctatctaataagagcactgacattaaatacgatgaccaaagatgcagagagaggcaagctaaaaagaagggctatcgcaacacctgggatgcagattagaggtttcgtatactttgttgaaactttagctaggagcatctgcgaaaagcttgaacagtctgggctcccagtagggggcaatgaaaagaaggccaaactggcaaatgttgtgagaaagatgatgactaattcacaagacacagagatttctttcacaatcactggggacaacactaagtggaatgaaaatcaaaatcctcgaatgtttctggcgatgattacatatatcaccagaaatcaacccgagtggttcagaaacatcctgagcatggcacccataatgttctcaaacaaaatggcaagactagggaaagggtacatgttcgagagtaaaagaatgaagattcgaacacaaataccagcagaaatgctagcaagcattgacctgaagtacttcaatgaatcaacaaagaagaaaattgagaaaataaggcctcttctaatagatggcacagcatcactgagtcctgggatgatgatgggcatgttcaacatgctaagtacggtcttgggagtctcgatactgaatcttggacaaaagaaatacaccaagacagtatactggtgggatgggctccaatcatccgacgattttgctctcatagtgaatgcaccaaaccatgagggaatacaagcaggagtggacagattctacaggacctgcaagttagtgggaatcaacatgagcaaaaagaagtcctatataaataagacagggacatttgaattcacaagctttttttatcgctatggatttgtggctaattttagcatggagctacccagctttggagtgtctggagtaaatgaatcagctgacatgagtattggagtaacagtgataaagaataacatgataaacaatgaccttggacctgcaacggcccagatggctcttcaattgttcatcaaagactacagatacacatataggtgccataggggagacacacaaattcagacgagaagatcatttgagttaaagaagctgtgggatcaaacccaatcaaaggtagggctattagtatcagatggaggaccaaacttatacaatatacgaaatcttcacattcctgaagtctgcttaaaatgggagctaatggatgatgattatcggggaagactttgtaatcccctgaatccctttgtcagtcataaagagattgattctgtaaacaatgctgtggtaatgccagcccatggtccagtcaaaagcatggaatatgatgccgttgcaactacacattcctggattcccaagaggaatcgttctattctcaacacaagccaaaggggaattcttgaggatgaacagatgtaccagaagtgctgcaatctattcgagaaatttttccctagcagttcatataggagaccggttggaatttctagcatggtggaggccatggtgtctagggcccggattgatgccagggtcgacttcgagtctggacggatcaagaaagaagagttctctgagatcatgaagatctgttccaccattgaagaactcagacggcaaaaataa

>H1N1_PB1_KC473860_A/Guangzhou/GIRD74/2010

atggatgtcaatccgactctacttttcctaaaaattccagcgcaaaatgccataagcaccacattcccttatactggagatcctccatacagccatggaacaggaacaggatacaccatggacacagtaaacagaacacaccaatactcagaaaagggaaagtggacgacaaacacggagactggtgcaccccagctcaacccgattgatggaccactacctgaggataatgaaccaagtgggtatgcacaaacagactgtgttctagaggctatggctttccttgaagaatcccacccaggaatatttgagaattcatgccttgaaacaatggaagttgttcaacaaacaagggtagataaactaactcaaggtcgccagacttatgattggacattaaacagaaatcaaccggcagcaactgcattggccaacaccatagaagtctttagatcgaatggtctaacagctaatgagtcaggaaggctaatagatttcttaaaggatgtaatggaatcaatgaacaaagaggaaatagagataacaacccactttcaaagaaaaaggagagtaagagacaacatgaccaagaaaatggtcacgcaaagaacaatagggaagaaaaaacaaagactgaataagagaggctatctaataagagcactgacattaaatacgatgaccaaagatgcagagagaggcaagttaaaaagaagggctatcgcaacacctgggatgcagattagaggtttcgtatactttgttgaaactttagctaggagcatttgcgaaaagcttgaacagtctgggctcccagtagggggcaatgaaaagaaggccaaactggcaaatgttgtgagaaagatgatgactaattcacaagacacagagatttctttcacaatcactggggacaacactaagtggaatgaaaatcaaaatcctcgaatgttcctagcgatgattacatatatcaccagaaatcaacccgagtggttcagaaacatcctgagcatggcacccataatgttctcaaacaaaatggcaagactagggaaagggtacatgttcgagagtaaaagaatgaagattcgaacacaaataccagcagaaatgctagcaagcattgacctgaagtacttcaatgaatcaacaaagaagaaaattgagaaaataaggcctcttctaatagatggcacagcatcactgagtcctgggatgatgatgggcatgttcaacatgctaagtacggtcttgggagtctcgatactgaatcttggacaaaagaaatacaccaagacaatatactggtgggatgggctccaatcatccgacgattttgctctcatagtgaatgcaccaaaccatgagggaatacaagcaggagtggacagattctacaggacctgcaagttagtgggaatcaacatgagcaaaaagaagtcctatataaataagacagggacatttgaattcacaagctttttttatcgctatggatttgtggctaattttagcatggagctacccagctttggagtgtctggagtgaatgaatcagctgacatgagtattggagtaacagtgataaagaacaacatgataaacaatgaccttggacctgcaacggcccagatggctcttcaattgttcatcaaagactacagatacacatataggtgccataggggagacacacaaattcagacgagaagatcatttgagttaaagaagctgtgggatcaaacccaatcaaagatagggctattagtatcagatggaggaccaaacttatacaatatacggaatcttcacattcctgaagtctgcttaaaatgggagctaatggatgatgattatcggggaagactttgtaatcccctgaatccctttgtcagtcataaagagattgattctgtaaacaatgctgtggtaatgccagcccatggtccagccaaaagcatggaatatgatgccgttgcaactacacattcctggattcccaagaggaatcgttctattctcaacacaagccaaaggggaattcttgaggatgaacagatgtaccaaaagtgctgcaatctattcgagaaatttttccctagcagttcatataggagaccggttggaatttctagcatggtggaggccatggtgtctagggcccggattgatgccagggtcgacttcgagtctggacggatcaagaaagaagagttctctgagatcatgaagatctgttccaccattgaagaactcagacggcaaaaataa

>H1N1_PB1_KC780135_A/New Mexico/08/2009

atggatgtcaatccgactctacttttcctaaaaattccagcgcaaaatgccataagcaccacattcccttatactggagatcctccatacagccatggaacaggaacaggatacaccatggacacagtaaacagaacacaccaatactcagaaaagggaaagtggacgacaaacacagagactggtgcaccccagctcaacccggttgatggaccactacctgaggataatgaaccaagtgggtatgcacaaacagactgtgttctagaggctatggctttccttgaagaatcccacccaggaatatttgagaattcatgccttgaaacaatggaagttgttcaacaaacaagggtagataaactaactcaaggtcgccagacttatgattggacattaaacagaaatcaaccggcagcaactgcattggccaacaccatagaagtctttagatcgaatggcctaacagctaatgagtcaggaaggctaatagatttcttaaaggatgtaatggaatcaatgaacaaagaggaaatagagataacaacccactttcaaagaaaaaggagagtaagagacaacatgaccaagaagatggtcacgcaaagaacaatagggaagaaaaaacaaagactgaataagagaggctatctaataagagcactgacattaaatacgatgaccaaagatgcagagagaggcaagttaaaaagaagggctatcgcaacacctgggatgcagattagaggtttcgtatactttgttgaaactttagctaggagcatttgcgaaaagcttgaacagtctgggctcccagtagggggcaatgaaaagaaggccaaactggcaaatgttgtgagaaagatgatgactaattcacaagacacagagatttctttcacaatcactggggacaacactaagtggaatgaaaatcaaaatcctcgaatgttcctggcgatgattacatatatcaccagaaatcaacccgagtggttcagaaacatcctgagcatggcacccataatgttctcaaacaaaatggcaagactagggaaagggtacatgttcgagagtaaaagaatgaagattcgaacacaaataccagcagaaatgctagcaagcattgacctgaagtacttcaatgaatcaacaaagaagaaaattgagaaaataaggcctcttctaatagatggcacagcatcactgagtcctgggatgatgatgggcatgttcaacatgctaagtacggtcttgggagtctcgatactgaatcttggacaaaagaaatacaccaagacaatatactggtgggatgggctccaatcatccgacgattttgctctcatagtgaatgcaccaaaccatgagggaatacaagcaggagtggacagattctacaggacctgcaagttagtgggaatcaacatgagcaaaaagaagtcctatataaataagacagggacatttgaattcacaagctttttttatcgctatggatttgtggctaattttagcatggagctacccagctttggagtgtctggagtaaatgaatcagctgacatgagtattggagtaacagtgataaagaacaacatgataaacaatgaccttggacctgcaacggcccagatggctcttcaattgttcatcaaagactacagatacacatataggtgccataggggagacacacaaattcagacgagaagatcatttgagttaaagaagctgtgggatcaaacccaatcaaaggtagggctattagtatcagatggaggaccaaacttatacaatatacggaatcttcacattcctgaagtctgcttaaaatgggagctaatggatgatgattatcggggaagactttgtaatcccctgaatccctttgtcagtcataaagagattgattctgtaaacaatgctgtggtaatgccagcccatggtccagccaaaagcatggaatatgatgccgttgcaactacacattcctggattcccaagaggaatcgttctattctcaacacaagccaaaggggaattcttgaggatgaacagatgtaccagaagtgctgcaatctattcgagaaatttttccctagcagttcatataggagaccggttggaatttctagcatggtggaggccatggtgtctagggcccggattgatgccagggtcgacttcgagtctggacggatcaagaaagaagagttctctgagatcatgaagatctgttccaccattgaagaactcagacggcaaaaataa

>H1N1_PB1_KC780542_A/New Jersey/01/2010

atggatgtcaatccgactctacttttcctaaaaattccagcgcaaaatgccataagcaccacattcccttatactggagatcctccatacagccatggaacaggaacaggatacaccatggacacagtaaacagaacacaccaatactcagaaaagggaaagtggacgacaaacacagagactggtgcaccccagctcaacccgattgatggaccactacctgaggataatgaaccaagtgggtatgcacaaacagactgtgttctagaggctatggctttccttgaagaatcccacccaggaatatttgagaattcatgccttgaaacaatggaagttgttcaacaaacaagggtagataaactaactcaaggtcgccagacttatgattggacattaaacagaaatcaaccggcagcaactgcattggccaacaccatagaagtctttagatcgaatggcctaacagctaatgaatcaggaaggctaatagatttcttaaaggatgtaatggaatcaatgaacaaagaggaaatagagataacaacccactttcaaagaaaaaggagagtaagagacaacatgaccaagaagatggtcacgcaaagaacaatagggaagaaaaaacaaagactgaataagagaggctatctaataagagcactgacattaaatacgatgaccaaagatgcagagagaggcaagttaaaaagaagggctatcgcaacacctgggatgcagattagaggtttcgtatactttgttgaaactttagctaggagcatttgcgaaaagcttgaacagtctgggctcccagtagggggcaatgaaaagaaggccaaactggcaaatgttgtgagaaagatgatgactaattcacaagacacagagatttctttcacaatcactggggacaacactaagtggaatgaaaatcaaaatcctcgaatgttcctggcgatgattacatatatcaccagaaatcaacccgagtggttcagaaacatcctgagcatggcacccataatgttctcaaacaaaatggcaagactagggaaagggtacatgtttgagagtaaaagaatgaagattcgaacacaaataccagcagaaatgctagcaagcattgacctgaagtacttcaatgaatcaacaaagaagaaaattgagaaaataaggcctcttctaatagatggcacagcatcactgagtcctgggatgatgatgggcatgttcaacatgctaagtacggtcttgggagtctcgatactgaatcttggacaaaagaaatacaccaagacaatatactggtgggatgggctccaatcatccgacgattttgctctcatagtgaatgcaccaaaccatgagggaatacaagcaggagtggacagattctacaggacctgcaagttagtgggaatcaacatgagcaaaaagaagtcctatataaataagacagggacatttgaattcacaagctttttttatcgctatggatttgtggctaattttagcatggagctgcccagctttggagtgtctggagtaaatgaatcagctgacatgagtattggagtaacagtgataaagaacaacatgataaacaatgaccttggacctgcaacggcccagatggctcttcaactgttcatcaaagactacagatacacatataggtgccataggggggacacacaaattcagacgagaagatcatttgagttaaagaagctgtgggatcaaacccaatcaaaggtagggctattagtatcagatggaggaccaaacttatacaatatacggaatcttcacattcctgaagtctgcttaaaatgggagctaatggatgatgattatcggggaagactttgtaatcccctgaatccctttgtcagtcataaagagattgattctgtaaacaatgctgtggtaatgccagcccatggtccagccaaaagcatggaatatgatgccgttgcaactacacattcctggattcccaagaggaatcgttctattctcaacacaagccaaaggggaattcttgaggatgaacagatgtaccagaagtgctgcaatctattcgagaaatttttccctagcagttcatataggagaccggttggaatttctagcatggtggaggccatggtgtctagggcccggattgatgccagggtcgacttcgagtctggacggatcgggaaggaagagttctctgagatcatgaagatctgttccaccattgaagagctcagacggcaaaaataa

>H1N1_PB1_KC780577_A/Virginia/01/2010

atggatgtcaatccgactctacttttcctaaaaattccagcgcaaaatgccataagcaccacattcccttatactggagatcctccatacagccatggaacaggaacaggatacaccatggacacagtaaacagaacacaccaatactcagaaaaaggaaagtggacgacaaacacagagactggtgcaccccagctcaacccgattgatggaccactacctgaggataatgaaccaagtgggtatgcacaaacagactgtgttctagaggctatggctttccttgaagaatcccacccaggaatatttgagaattcatgccttgaaacaatggaagttgttcaacaaacaagggtagataaactaactcaaggtcgccagacttatgattggacattaaacagaaatcaaccggcagcaactgcattggccaacaccatagaagtctttagatcgaatggcctaacagctaatgagtcaggaaggctaatagatttcttaaaggatgtaatggaatcaatgaacaaagaggaaatagagataacaacccactttcaaagaaaaaggagagtaagagacaacatgaccaagaagatggtcacgcaaagaacaatagggaagaaaaaacaaagactgaataagagaggctatctaataagagcactgacattaaatacgatgaccaaagatgcagagagaggcaagttaaaaagaagggctatcgcaacacctgggatgcagattagaggtttcgtatactttgttgaaactttagctaggagcatttgcgaaaagcttgaacagtctgggctcccagtagggggcaatgaaaagaaggccaaactggcaaatgttgtgagaaagatgatgactaattcacaagacacagagatttctttcacaatcactggggacaacactaagtggaatgaaaatcaaaatcctcgaatgttcctggcgatgattacatatatcaccagaaatcaacccgagtggttcagaaacatcctgagcatggcacccataatgttctcaaacaaaatggcaagactagggaaagggtacatgttcgagagtaaaagaatgaagattcgaacacaaataccagcagaaatgctagcaagcattgacctgaagtacttcaatgaatcaacaaagaagaaaattgagaaaataaggcctcttctaatagatggcacagcatcactgagtcctgggatgatgatgggcatgttcaacatgctaagtacggtcttaggagtctcgatactaaatcttggacaaaagaaatacaccaagacaatatactggtgggatgggctccaatcatccgacgattttgctctcatagtgaatgcaccaaaccatgagggaatacaagcaggggtggacagattctacaggacctgcaagttagtgggaatcaacatgagcaaaaagaagtcctatataaataagacagggacatttgaattcacaagctttttttatcgctatggatttgtggctaattttagcatggagctacccagctttggagtgtctggagtaaatgaatcagctgacatgagtattggagtaacagtgataaagaacaacatgataaacaatgaccttggacctgcaacggcccagatggctcttcaattgttcatcaaagactacagatacacatataggtgccataggggagacacacaaattcagacgagaagatcatttgagttaaagaagctgtgggatcaaacccaatcaaaggtagggctattagtatcagatggaggaccaaacttatacaatatacggaatcttcacattcctgaagtctgcttaaaatgggagctaatggatgatgattatcggggaagactttgtaatcccctgaatccctttgtcagtcataaagagattgattctgtaaacaatgctgtggtaatgccagcccatggtccagccaaaagcatggaatatgatgccgttgcaactacacattcctggattcccaagaggaatcgttctattctcaacacaagccaaaggggaattcttgaggatgaacagatgtaccagaagtgctgcaatctattcgagaaatttttccctagcagttcatataggagaccggttggaatttctagcatggtggaggccatggtgtctagggcccggattgatgccagggtcgacttcgagtctggacggatcaagaaagaagagttctctgagatcatgaagatctgttccaccattgaagaactcagacggcaaaaataa

>H1N1_PB1_KC780831_A/Kansas/20/2009

atggatgtcaatccgactctacttttcctaaaaattccagcgcaaaatgccataagcaccacattcccttatactggagatcctccatacagccatggaacaggaacaggatacaccatggacacagtaaacagaacacaccaatactcagaaaagggaaagtggacracaaacacagagactggtgcaccccagctcaacccgattgatggaccactacctgaggataatgaaccaagtgggtatgcacaaacagactgtgttctagaggctatggctttccttgaagaatcccacccaggaatatttgagaattcatgccttgaaacaatggaagttgttcaacaaacaagggtagataaactaactcaaggtcgccagacttatgattggacattaaacagaaatcaaccggcagcaactgcattggccaacaccatagaagtctttagatcgaatggcctaacagctaatgagtcaggaaggctaatagatttcttaaaggatgtaatggaatcaatgaacaaagaggaaatagagataacaacccactttcaaagaaaaaggagagtaagagacaacatgaccaagaagatggtcacgcaaagaacaatagggaagaaaaaacaaagactgaataagagaggctatctaataagagcactgacattaaatacgatgaccaaagatgcagagagaggcaagttaaaaagaagggctatcgcaacacctgggatgcagattagaggtttcgtatactttgttgaaactttagctaggagcatttgcgaaaagcttgaacagtctgggctcccagtagggggcaatgaaaagaaggccaaactggcaaatgttgtgagaaagatgatgactaattcacaagacacagagatttctttcacaatcactggggacaacactaagtggaatgaaaatcaaaatcctcgaatgttcctggcgatgattacatatatcaccagaaatcaacccgagtggttcagaaacatcctgagcatggcacccataatgttctcaaacaaaatggcaagactagggaaagggtacatgttcgagagtaaaagaatgaagattcgaacacaaataccagcagaaatgctagcaagcattgacctgaagtacttcaatgaatcaacaaagaagaaaattgagaaaataaggcctcttctaatagatggcacagcatcactgagtcctgggatgatgatgggcatgttcaacatgctaagtacggtcttgggagtctcgatactgaatcttggacaaaagaaatacaccaagacaatatactggtgggatgggctccaatcatccgacgattttgctctcatagtgaatgcaccaaaccatgagggaatacaagcaggagtggacagattctacaggacctgcaagttagtgggaatcaacatgagcaaaaagaagtcctatataaataagacagggacatttgaattcacaagctttttttatcgctatggatttgtggctaattttagcatggagctacccagctttggagtgtctggagtaaatgaatcagctgacatgagtattggagtaacagtgataaagaacaacatgataaacaatgaccttggacctgcaacggcccagatggctcttcaattgttcatcaaagactacagatacacatataggtgccataggggagacacacaaattcagacgagaagatcatttgagttaaagaagctgtgggatcaaacccaatcaaaggtagggctattagtatcagatggaggaccaaacttatacaatatacggaatcttcacattcctgaagtctgcttaaaatgggagctaatggatgatgattatcggggaagactttgtaatcccctgaatccctttgtcagtcataaagagattgattctgtaaacaatgctgtggtaatgccagcccatggtccagccaaaagcatggaatatgatgccgttgcaactacacattcctggattcccaagaggaatcgttctattctcaacacaagccaaaggggaattcttgaggatgaacagatgtaccagaagtgctgcaatctattcgagaaatttttccctagcagttcatataggagaccggttggaatttctagcatggtggaggccatggtgtctagggcccggattgatgccagggtcgacttcgagtctggacggatcaagaaagaagagttctctgagatcatgaagatctgttccaccattgaagaactcagacggcaaaaataa

>H1N1_PB1_KC781184_A/Wyoming/01/2010

atggatgtcaatccgactctacttttcctaaaaattccagcgcaaaatgccataagcaccacattcccttatactggagatcctccatacagccatggaacaggaacaggatacaccatggacacagtaaacagaacacaccaatactcagaaaagggaaagtggacgacaaacacagagactggtgcaccccagctcaacccgattgatggaccactacctgaggataatgaaccaagtgggtatgcacaaacagactgtgttctagaggctatggctttccttgaagaatcccacccaggaatatttgagaattcatgccttgaaacaatggaagttgttcaacaaacaagggtagataaactaactcaaggtcgccagacttatgattggacattaaacagaaatcaaccggcagcaactgcattggccaacaccatagaagtctttagatcgaatggcctaacagctaatgagtcaggaaggctaatagatttcttaaaggatgtaatggaatcaatgaacaaagaggaaatagagataacaacccactttcaaagaaaaaggagagtaagagacaacatgaccaagaagatggtcacgcaaagaacaatagggaagaaaaaacaaagactgaataagagaggctatctaataagagcactgacattaaatacgatgaccaaagatgcagagagaggcaagttaaaaagaagggctatcgcaacacctgggatgcagattagagggttcgtatattttgttgaaactttagctaggagcatttgcgaaaagcttgaacagtctgggctcccagtagggggcaatgaaaagaaggccaaactggcaaatgttgtgagaaagatgatgactaattcacaagacacagagatttctttcacaatcactggggacaacactaagtggaatgaaaatcaaaatcctcgaatgttcctggcgatgattacatatatcaccagaaatcaacccgagtggttcagaaacatcctgagcatggcacccataatgttctcaaacaaaatggcaagactagggaaagggtacatgttcgagagtaaaagaatgaagattcgaacacaaataccagcagaaatgctagcaagcattgacctgaagtacttcaatgaatcaacaaagaagaaaattgagaaaataaggcctcttctaatagatggcacagcatcactgagtcctgggatgatgatgggcatgttcaacatgctaagtacggtcttgggagtctcgatactgaatcttggacaaaagaaatacaccaagacaatatactggtgggatgggctccaatcatccgacgattttgctctcatagtgaatgcaccaaaccatgagggaatacaagcaggagtggacagattctacaggacctgcaagttagtgggaatcaacatgagcaaaaagaagtcctatataaataagacagggacatttgaattcacaagctttttttatcgctatggatttgtggctaattttagcatggagctacccagctttggagtgtctggagtaaatgaatcagctgacatgagtattggagtaacagtgataaagaacaacatgataaacaatgaccttggacctgcaacggcccagatggctcttcaattgttcatcaaagactacagatacacatataggtgccataggggagacacacaaattcagacgagaagatcatttgagttaaagaagctgtgggatcaaacccaatcaaagggagggctattagtatcagatggaggaccaaacttatacaatatacggaatcttcacattcctgaagtctgcttaaaatgggagctaatggatgatgattatcggggaagactttgtaatcccctgaatccctttgtcagtcataaagagattgattctgtaaacaatgctgtggtaatgccagcccatggtccagtcaaaagcatggaatatgatgccgttgcaactacacattcctggattcccaagaggaatcgttctattctcaacacaagccaaaggggaattcttgaggatgaacagatgtaccagaagtgctgcaatctattcgagaaatttttccctagcagttcatataggagaccggttggaatttctagcatggtggaggccatggtgtctagggcccggattgatgccagggtcgacttcgagtctggacggatcaagaaagaagagttctctgagatcatgaagatctgctccaccattgaagaactcagacggcaaaaataa

>H1N1_PB1_KC781727_A/Oregon/30/2009

atggatgtcaatccgactctacttttcctaaaaattccagcgcaaaatgccataagcaccacattcccttatactggagatcctccatacagccatggaacaggaacaggatacaccatggacacagtaaacagaacacaccaatactcagaaaagggaaagtggacgacaaacacagagactggtgcaccccagctcaacccgattgatggaccactacctgaggataatgaaccaagtgggtatgcacaaacagactgtgttctagaggctatggctttccttgaagaatcccacccaggaatatttgagaattcatgccttgaaacaatggaagttgttcaacaaacaagggtagataaactaactcaaggtcgccagacttatgattggacattaaacagaaatcaaccggcagcaactgcattggccaacaccatagaagtctttagatcgaatggcctaacagctaatgagtcaggaaggctaatagatttcttaaaggatgtaatggaatcaatgaacaaagaggaaatagagataacaacccattttcaaagaaaaaggagagtaagagacaacatgaccaagaagatggtcacgcaaagaacaatagggaagaaaaaacaaagactgaataagagaggctatctaataagagcactgacattaaatacgatgaccaaagatgcagagagaggcaagttaaaaagaagggctatcgcaacacctgggatgcagattagaggtttcgtatactttgttgaaactttagctaggagcatttgcgaaaagcttgaacagtctgggctcccagtagggggcaatgaaaagaaggccaaactggcaaatgttgtgagaaagatgatgactaattcacaagacacagagatttctttcacaatcactggggacaacactaagtggaatgaaaatcaaaatcctcgaatgttcctggcgatgattacatatatcaccagaaatcaacccgagtggttcagaaacatcctgagcatggcacccataatgttctcaaacaaaatggcaagactagggaaagggtacatgttcgagagtaaaagaatgaagattcgaacacaaataccagcagaaatgctagcaagcattgacctgaagtacttcaatgaatcaacaaagaagaaaattgagaaaataaggcctcttctaatagatggcacagcatcactgagtcctgggatgatgatgggcatgttcaacatgctaagtacggtcttgggagtctcgatactgaatcttggacaaaagaaatacaccaagacaatatactggtgggatgggctccaatcatccgacgattttgctctcatagtgaatgcaccaaaccatgagggaatacaagcaggagtggacagattctacaggacctgcaagttagtgggaatcaacatgagcaaaaagaagtcctatataaataagacagggacatttgaattcacaagctttttttatcgctatggatttgtggctaattttagcatggagctacccagctttggagtgtctggagtaaatgaatcagctgacatgagtattggagtaacagtgataaagaacaacatgataaacaatgaccttggacctgcaacggcccagatggctcttcaattgttcatcaaagactacagatacacatataggtgccataggggagacacacaaattcagacgagaagatcatttgagttaaagaagctgtgggatcaaacccaatcaaaggtagggctattagtatcagatggaggaccaaacttatacaatatacggaatcttcacattcctgaagtctgcttaaaatgggagctaatggatgatgattatcggggaagactttgtaatcccctgaatccctttgtcagtcataaagagattgattctgtaaacaatgctgtggtaatgccagcccatggtccagccaaaagcatggaatatgatgccgttgcaactacacattcctggattcccaagaggaatcgttctattctcaacacaagccaaaggggaattcttgaggatgaacagatgtaccagaagtgctgcaatctattcgagaaatttttccctagcagttcatataggagaccggttggaatttctagcatggtggaggccatggtgtctagggcccggattgatgccagggtcgacttcgagtctggacggatcaagaaagaagagttctctgagatcatgaagatctgttccaccattgaagaactcagacggcaaaaataa

>H1N1_PB1_KC781790_A/Wisconsin/26/2009

atggatgtcaatccgactctacttttcctaaaaattccagcgcaaaatgccataagcaccacattcccttatactggagatcctccatacagccatggaacaggaacaggatacaccatggacacagtaaacagaacacaccaatactcagaaaagggaaagtggacgacaaacacagagactggtgcaccccagctcaacccgattgatggaccactacctgaggataatgaaccaagtgggtatgcacaaacagactgtgttctagaggctatggctttccttgaagaatcccacccaggaatatttgagaattcatgccttgaaacaatggaagttgttcaacaaacaagggtagataaactaactcaaggtcgccagacttatgattggacattaaacagaaatcaaccggcagcaactgcattggccaacaccatagaagtctttagatcgaatggcctaacagctaatgagtcaggaaggctaatagatttcttaaaggatgtaatggaatcaatgaacaaagaggaaatagagataacaacccactttcaaagaaaaaggagagtaagagacaacatgaccaagaagatggtcacgcaaagaacaatagggaagaaaaaacaaagactgaataagagaggctatctaataagagcactgacattaaatacgatgaccaaagatgcagagagaggcaagttaaaaagaagggctatcgcaacacctgggatgcagattagaggtttcgtatactttgttgaaactttagctaggagcatttgcgaaaagcttgaacagtctgggctcccagtagggggcaatgaaaagaaggccaaactggcaaatgttgtgagaaagatgatgactaattcacaagacacagagatttctttcacaatcactggggacaacactaagtggaatgaaaatcaaaatcctcgaatgttcctggcgatgattacatatatcaccagaaatcaacccgagtggttcagaaacatcctgagcatggcacccataatgttctcaaacaaaatggcaagactagggaaagggtacatgttcgagagtaaaagaatgaagattcgaacacaaataccagcagaaatgctagcaagcattgacctgaagtacttcaatgaatcaacaaagaagaaaattgagaaaataaggcctcttctaatagatggcacagcatcactgagtcctgggatgatgatgggcatgttcaacatgctaagtacggtcttgggagtctcgatactgaatcttggacaaaagaaatacaccaagacaatatactggtgggatgggctccaatcatccgacgattttgctctcatagtgaatgcaccaaaccatgagggaatacaagcaggagtggacagattctacaggacctgcaagttagtgggaatcaacatgagcaaaaagaagtcctatataaataagacagggacatttgaattcacaagctttttttatcgctatggatttgtggctaattttagcatggagctacccagctttggagtgtctggagtaaatgaatcagctgacatgagtattggagtaacagtgataaagaacaacatgataaacaatgaccttggacctgcaacggcccagatggctcttcaattgttcatcaaagactacagatacacatataggtgccataggggagacacacaaattcagacgagaagatcatttgagttaaagaagctgtgggatcaaacccaatcaaaggtagggctattagtatcagatggaggaccaaacttatacaatatacggaatcttcacattcctgaagtctgcttaaaatgggagctaatggatgatgattatcggggaagactttgtaatcccctgaatccctttgtcagtcataaagagattgattctgtaaacaatgctgtggtaatgccaacccatggtccagccaaaagcatggaatatgatgccgttgcaactacacattcctggattcccaagaggaatcgttctattctcaacacaagccaaaggggaattcttgaggatgaacagatgtaccagaagtgctgcaatctattcgagaaatttttccctagcagttcatataggagaccggttggaatttctagcatggtggaggccatggtgtctagggcccggattgatgccagggtcgacttcgagtctggacggatcaagaaagaagagttctctgagatcatgaagatctgttccacctttgaagaactcagacggcaaaaataa

>H1N1_PB1_KC881818_A/California/17/2011

atggatgtcaatccgactctacttttcctgaaaattccagcgcaaaatgccataagcactacattcccttatactggagatcctccatacagccatggaacaggaacaggatacaccatggacacagtaaacagaacacaccaatactcagaaaagggaaagtggacgacaaacacagagactggagcaccccagctcaacccgattgatggaccactacctgaggataatgaaccaagtgggtatgcacaaacagactgtgttctagaggctatggctttccttgaagaatcccacccaggaatatttgagaattcatgccttgaaacaatggaagttgttcaacaaacaagggtagataaactaactcaaggtcgccagacttatgattggacattaaacagaaatcaaccggcagcaactgcattggccaacaccatagaagtctttagatcgaatggcctaacagctaatgagtcaggaaggctaatagatttcttaaaggatgtaatggaatcaatgaacaaagaggaaatagagataacaacccactttcaaagaaaaaggagagtaagagacaacatgaccaagaagatggtcacgcaaagaacaatagggaagaaaaaacaaaaactgaataagagaggctatctaataagagcactgacattaaatacgatgaccaaagatgcagagagaggcaagttaaaaagaagggctatcgcaacacctgggatgcagattagaggtttcgtatactttgttgaaactttagctaggagcatctgcgaaaagcttgaacagtctgggctcccagtagggggcaatgaaaagaaggccaaactggcaaatgttgtgagaaagatgatgactaattcacaagacacagagatttctttcacaatcactggggacaacactaagtggaatgaaaatcaaaatcctcgaatgtttctggcgatgattacatatatcaccagaaatcaacccgagtggttcagaaacatcctgagcatggcacccataatgttctcaaacaaaatggcaagactagggaaagggtacatgttcgagagtaaaagaatgaagattcgaacacaaataccagcagaaatgctagcaagcattgacctgaagtacttcaatgaatcaacaaagaagaaaattgagaaaataaggcctcttctaatagatggcacagcatcactgagtcctgggatgatgatgggcatgttcaacatgctaagtacggtcttgggagtctcgatactgaatcttggacaaaagaaatacaccaagacagtatactggtgggatgggctccaatcatccgacgattttgctctcatagtgaatgcaccaaaccatgagggaatacaagcaggagtggacagattctacaggacctgcaagttagtgggaatcaacatgagcaaaaagaagtcctatataaataagacagggacatttgaattcacaagctttttttatcgctatggatttgtggctaattttagcatggagctacccagctttggagtgtctggagtaaatgaatcagctgacatgagtattggagtaacagtgataaagaataacatgataaacaatgaccttggacctgcgacggcccagatggctcttcaattgttcatcaaagactacagatacacatataggtgccataggggagacacacaaattcagacgagaagatcatttgagttaaagaagctgtgggatcaaacccaatcaaaggtagggctattagtatcagatggaggaccaaacttatacaatatacggaatcttcacattcctgaagtctgcttaaaatgggagctaatggatgatgattatcggggaagactttgtaatcccctgaatccctttgtcagtcataaagagattgattctgtaaacaatgctgtggtaatgccagcccatggtccagtcaaaagcatggaatatgatgccgttgcaactacacattcctggattcccaagaggaatcgttctattctcaacacaagccaaaggggaattcttgaggatgaacagatgtaccagaagtgctgcaatctattcgagaaatttttccctagcagttcatataggagaccggttggaatttctagcatggtggaggccatggtgtctagggcccggattgatgccagggtcgacttcgagtctggacggatcaagaaagaagagttctctgagatcatgaagatctgttccaccattgaagaactcagacggcaaaaataa

>H1N1_PB1_KC881845_A/Kentucky/09/2010

atggatgtcaatccgactctacttttcctaaaaattccagcgcaaaatgccataagcaccacattcccttatactggagatcctccatacagccatggaacaggaacaggatacaccatggacacagtaaacagaacacaccaatactcagaaaagggaaagtggacgacaaacacagagactggtgcaccccagctcaacccgattgatggaccactacctgaggataatgaaccaagtgggtatgcacaaacagactgtgttctagaggctatggctttccttgaagaatcccacccaggaatatttgagaattcatgccttgaaacaatggaagttgttcaacaaacaagggtagataaactaactcaaggtcgccagacttatgattggacattaaacagaaatcaaccggcagcaactgcattggccaacaccatagaagtctttagatcgaatggcctaacagctaatgagtcaggaaggctaatagatttcttaaaggatgtaatggaatcaatgaacaaagaggaaatagagataacaacccactttcaaagaaaaaggagagtaagagacaacatgaccaagaagatggtcacgcaaagaacaatagggaagaaaaaacaaagactgaataagagaggctatctaataagagcactgacattaaatacgatgaccaaagatgcagagagaggcaagttaaaaagaagggctatcgcaacacctgggatgcagattagaggtttcgtatactttgttgaaactttagctaggagcatttgcgaaaagcttgaacagtctggactcccagtagggggcaatgaaaagaaggccaaactggcaaatgttgtaagaaagatgatgactaattcacaagacacagagatttctttcacaatcactggggacaacactaagtggaatgaaaaccaaaatcctcgaatgttcctggcgatgattacatatatcaccagaaatcaacccgagtggttcagaaacatcctgagcatggcacccataatgttctcaaacaaaatggcaagactagggaaagggtacatgttcgagagtaaaagaatgaagattcgaacgcaaataccagcagaaatgctagcaagcattgacctgaagtacttcaatgaatcaacaaagaagaaaattgagaaaataaggcctcttttaatagatggcacagcatcactgagtcctgggatgatgatgggcatgttcaacatgctaagtacggtcttgggagtctcgatactgaatcttggacaaaagaaatacaccaagacaatatactggtgggatgggctccaatcatccgacgattttgctctcatagtgaatgcaccaaaccatgagggaatacaagcaggagtggacagattttacaggacctgcaagttagtgggaatcaacatgagcaaaaagaagtcctatataaataagacagggacatttgaattcacaagctttttttatcgctatggatttgtggctaattttagcatggagctacccagctttggagtgtctggagtaaatgaatcagctgacatgagtattggagtaacagtgataaagaacaacatgataaacaatgaccttggacctgcaacggcccagatggctcttcaattgttcatcaaagactacagatacacatataggtgccataggggagacacacaaattcagacgagaagatcatttgagttaaagaagctgtgggatcaaacccaatcaaaggtagggctattagtatcagatggaggaccaaacttatacaatatacggaatcttcacattcctgaagtctgcttaaaatgggagctaatggatgatgattatcggggaagactttgtaatcccctgaatccctttgtaagtcataaagagattgattctgtaaacaatgctgtggtaatgccagcccatggtccagccaaaagcatggaatatgatgccgttgcaactacacattcctggattcccaagaggaatcgttctattctcaacacaagccaaaggggaattcttgaggatgaacagatgtaccagaagtgctgcaatctattcgagaaatttttccctagcagttcatataggagaccggttggaatttctagcatggtggaggccatggtgtctagggcccggattgatgccagggtcgactttgagtctggacggatcaagaaagaagagttctctgagatcatgaagatctgttccaccattgaagaactcagacggcaaaaataa

>H1N1_PB1_KF057098_A/Jiangsu/1/2011

atggatgtcaacccgactctacttttcttgaaagtgccagcgcaaaatgctataagcactacattcccttacactggagatcctccatacagccatggaacagggacaggatacaccatggacacggtgaacaggacacaccaatattcagaaaggggaagatggacaacaaacacagagactggagcacctcagcttaaccctattgatggaccactacctgaggacaatgagccaagcgggtatgctcaaacagactgcgtcctggaggcaatggctttccttgaggaatcccacccagggatatttgaaaactcgtgtcttgaaacaatggagattgtccaacaaacaagagtggacaaactgacccaaggccgtcagacctatgactggacattaaatagaaaccagccggctgcaactgccttggccaacacaatagaggtcttcagattgaacagtctaacagccaatgaatcaggaagattgatagatttccttaaggatgtgatggaatcaatggataaagaagagatggaaataacaacacatttccaaaggaaaagaagaataagggacaacataaccaaaaaaatggtaacacaaaggacaataggaaagaaaaagcagaaactaaacaaaaggagttatctaataagagcattgacattaaacacaatgacaaaggacgcagaaagaggcaaattgaaaaggcgtgcaattgcaacaccagggatgcagatcagagggtttgtgtacttcgtagaaacactggcgaggagcatctgtgagaaacttgagcaatctgggctcccagttggaggaaatgaaaagaaagccaaattggcaaatgttgtgagaaaaatgatgaccaactcacaggatacagagctctccttcacaattactggagacaacaccaaatggaatgagaatcaaaatcccagggtgtttctggcgatgataacgtacatcacaagaaatcagcctgaatggttcagaaatgttttaagcattgcccctataatgttctcaaacaaagtggcaaggctagggaaaggatacatgttcgaaagtaagagcatgaaactacggacacaaataccagcagaaatgcttgcagatattgacttgaaatacttcaatgaatcaacaaggaaaaaaattgagaagataaggccactcctaatagatggcacagcctcattaagtcctggaatgatgatgggcatgtttaacatgcttagcactgtcctaggagtttcaatcctgaatcttgggcaaaagagatacactaaaactacatactggtgggacggactccaatcctccgatgacttcgccctcatagtgaatgcacctaaccatgagggaatacaagcgggagtgaacagattctataggacctgcaaactggttgggatcaatatgagtaaaaagaaatcttacataaataggacaggaacatttgaattcacaagctttttctaccgctacgggtttgtagccaattttagtatggagctacccagctttggggtgtctggggtcaacgaatcggctgatatgagcattggagtgacagtgataaagaacaacatgataaacaatgatctcgggccagcaactgcccaaatggctcttcagctattcgttaaggactacagatatacataccggtgccacagaggtgacacgcaaatccaaacgaaaagatcatttgagctaaaaaggctatgggagcagacccattcaaaaacaggactactaatttcagatggaggaccaaatctatataatatccggaatctccacattccggaagtttgcctgaagtgggagctaatggatatagactatcgggggagattgtgtaatcctctgaatccatttgttaaccacaagggaattgaatccataaacagtgccgtggtaatgccagctcatggtccagctaagagtatggaatatgatgctgttgcaactacacattcttggattcccaaaaggaaccgttccattctcaataccagccagaggggaattcttgaggatgaacaaatgtaccagaagtgctgtaatctatttgaaaaattcttccctagcagttcatacaggaggccagttggaatttccagcatggtggaggccatggtatctagggccagaattgatgcacggattgatttcgagtctggaaggattaagaaagaagagtttgctgagatcatgaagacctgttccaccattgaagagctcagacggcaaaagtag

>H1N1_PB1_CY147324_A/New Caledonia/20-JY2/1999

atggatgtcaatccgacattacttttcttaaaagtgccagcacaaaatgctataagcacaacttttccttatactggtgaccctccttacagccatgggacaggaacagggtacaccatggatacagtcaacaggacacatcagtactcagaaagaggaagatggacaaaaaataccgaaactggagcaccgcaactcaacccaattgatgggccactaccaaaagacaatgaaccaagtggctatgcccaaacagattgtgtattagaagcaatggctttccttgaggaatcccatcctggtatttttgaaaactcttgtattgaaacaatggaggttgttcagcaaacaagggtggacaaactgacacaaggcagacagacctatgactggactctaaataggaaccagcctgctgccacagcattggccaacactatagaagtgttcagatcaaacggcctcatagcaaatgaatctgggaggctaatagacttccttaaagatgtaatggagtcgatggacagagacgaagtagagatcacaactcattttcaaagaaagaggagagtgagagacaatgtaactaaaaaaatggtgacccaaagaacaataggcaaaaagaaacataaattagacaaaagaagttacctaattagggcattaaccctgaacacaatgaccaaagatgctgagagggggaaactaaaacgcagagcaattgcaaccccaggaatgcaaataagggggtttgtatactttgttgagacactggcaagaagcatatgtgaaaagcttgaacaatcagggttgccagttggaggaaatgaaaagaaagcaaagttagcaaatgttgtaaggaagatgatgaccaactcccaggacactgaaatttctttcaccatcactggagataacacaaaatggaacgaaaatcaaaaccctagaatgttcttggccatgatcacatatataaccaaaaatcagcctgaatggttcagaaatattctaagtattgctccaataatgttttcaaacaaaatggcgagactaggtaaggggtacatgtttgaaagcaagagtatgaaactgagaactcaaatacctgcagagatgctagccaacatagatttgaaatatttcaatgattcaactaaaaagaaaattgaaaaaatccggccattattaatagatggaactgcatcattgagtcctggaatgatgatgggcatgttcaatatgttaagcaccgtcttgggcgtctccattctgaatcttgggcaaaagagatacaccaagactacttactggtgggatggtcttcaatcgtctgatgattttgctctgattgtgaatgcacccaactatgcaggaattcaagctggagttgacaggttttatcgaacctgtaagctgctcggaattaatatgagcaaaaagaagtcttacataaacagaacaggtacctttgagttcacgagctttttctatcgttatgggtttgttgccaatttcagcatggagcttcctagttttggggtgtctggggtcaatgaatctgcagacatgagtattggagtcactgtcatcaaaaacaatatgataaacaatgaccttggcccagcaactgctcaaatggcccttcagttatttataaaagattacaggtacacgtatcgatgccacagaggtgacacacaaatacaaacccggagatcatttgagataaagaaactatgggaccaaacccgctccaaagctgggctgttggtctctgatggaggccccaatttatataacattagaaatctccatattcctgaagtctgcttgaaatgggagttgatggatgaggattaccaggggcgtttatgcaacccattgaacccgtttgtcagtcataaagagattgaatcagtgaacaatgcagtgatgatgccggcacatggtccagccaaaaatatggagtatgacgctgttgcaacaacacactcctgggttcccaaaaggaatcgatccattttgaatacgagccaaagggggatacttgaggatgagcaaatgtatcagaggtgctgcaatttatttgaaaaattcttcccaagtagctcatacagaagaccagttggaatatccagtatggtagaggctatggtttccagagcccgaattgatgcacggattgatttcgaatctggaaggataaaaaaagaggaattcgctgagatcatgaagacctgttccaccattgaagacctcagacggcaaaaatag

>H1N1_PB1_CY147348_A/Fort Monmouth/1-JY2/1947

atggatgtcaatccgaccttacttttcttaaaagtgccagcacaaaatgctataagcacaactttcccttatactggagaccctccttacagccatggaacagggacaggatacaccatggatactgtcaacaggacacatcagtactcagaaaggggaaggtggacaacaaacaccgaaactggagcaccacaactcaacccgattgatggaccactgccagaagacaatgaaccaagtggttatgcccaaacagattgtgtattggaagcaatggctttccttgaggaatcccatcctggtatttttgaaaactcgtgtattgagacaatggaggttgttcagcaaacacgagtggacaaactgacacaaggccgacagacctatgactggaccctaaatagaaaccagcctgctgcaacagcattggccaacacaatagaagtgttcagatcaaatggtctcacggccaatgaatctggaaggctaatagacttccttaaggatgtaatgaaatcaatggacaaagaagaaatggagatcacaactcattttcagagaaagagacgagtgagagacaatgtgactaagaaaatggtgacacagagaacaataggtaaaaggaagcagagattgaacaaaagaagttatctaattagagcattgaccctgaacacaatgaccaaagatgctgagagagggaagctaaaacgaagagcaattgcaaccccagggatgcaaataagagggtttgtatattttgttgagacactggcaagaagtatatgtgaaaaacttgaacaatcaggattaccagttggaggcaatgagaagaaagcaaagttggcaaatgttgtaaggaagatgatgaccaattctcaggacactgaaatttctttcaccatcactggagataacaccaaatggaacgaaaatcagaaccctaggatgtttttggccatgatcacatatatgaccagaaatcagccagaatggttcagaaatgttttaagtattgctccaataatgttctcaaacaaaatggcgagactaggaaagggatacatgtttgaaagcaagagtatgaaacttagaactcaaatacctgcagaaatgctagcaaacatcgatttgaagtatttcaatgattcaacaagaaagaagattgaaaaaatccgaccactcttaatagatgggactgcatcactgagccctggtatgatgatgggcatgttcaatatgttaagcactgtattgggcgtctccatcctgaatcttggacaaaagagatacaccaagactacttactggtgggatggtcttcaatcttctgatgattttgctctgattgtgaatgcacccaatcatgaaggaattcaagccggagttgacaggttttatcgaacctgtaagctactcggaatcaatatgagcaagaaaaagtcttacataaacagaacaggtacatttgaattcacgagctttttctatcgttatgggtttgttgccaatttcagcatggagcttcccagttttggggtttctggaatcaacgagtccgcagacatgagtattggagttactgtcatcaaaaacaatatgataaacaatgatcttggtccagcaacagctcaaatggcccttcagttgttcatcaaagattacaggtacacatatcgatgccatagaggtgacacacaaatacaaacccgaagatcatttgaaataaagaaactgtgggagcaaacccgttccaaagctggactgctagtctccgacggaggctcaaatttatacaacattagaaatctccacattcctgaagtctgcttgaaatgggaattgatggatgaagattaccgggggcgtttatgcaacccactgaacccatttgtcagtcataaagagattgaatcagtgaacaatgcagtgatgatgccggcacatggtccagccaaaaacatggaatatgatgctgttgcaacaacacactcctggatccccaaaagaaatcggtccatcttgaatacgagccagagaggaatacttgaagatgaacaaatgtatcaaaggtgctgcaatttatttgaaaaattcttccccagcagttcatacagaagaccagtcggaatatccagtatggtggaggctatggtttcaagagcccgaattgatgcacggattgacttcgaatcaggaaggataaagaaagaggggttcgctgagatcatggagatctgttccaccattgaagagctcggacggcaaaggtgg

>H1N1_PB1_CY147689_A/Mexico/24019/2009

atggatgtcaatccgacattacttttcttaaaagtgccagcacaaaatgctataagcacaacttttccttatactggtgaccctccttacagccatgggacaggaacagggtacaccatggatacagtcaataggacacaccagtactcagaaagaggaagatggacaaaaaataccgaaacgggagcaccgcaacttaacccaattgatggtcccttaccggaagacaatgaaccaagtggctatgcccaaacagattgtgtattagaagcaatggctttccttgaagaatcccatcccggtatctttgaaaactcttgtattgaaacaatggaggttgttcaacaaacaagggtggacaaactgacacaaggcagacagacctatgactggactctaaataggaaccagcctgctgccacagcattggcaaacactatagaagtattcagatcaaacggcctcatagcaaatgaatctggaaggctaatagacttccttaaagatgtaatggagtcgatggacagaggcgaagtagaggtcacaactcattttcaaagaaagaggagagtgagagacaatgtaactaaaaaaatggtgacccaaagaacaataggcaaaaagaaacataaattagacaaaagaagttacctaattagggcattaaccctgaacacaatgaccaaagatgctgagagggggaaactaaaacgcagagcaattgcaaccccagggatgcaaataagggggtttgtatactttgttgagacactggcaagaagcatatgtgaaaagcttgaacaatcaggattgccagttggaggaaatgagaagaaagcaaagttagcaaatgttgtaaggaagatgatgaccaactcccaggacactgaaatttctttcaccataaccggagacaacacaaaatggaacgaaaatcaaaaccctagaatgttcttggccatgatcacatatataaccaaaaatcagcctgaatggttcagaaatattctaagtattgctccaataatgttttcaaacaagatggcgagactaggtaaggggtacatgtttgaaagcaagagtatgaaactgagaactcaaatacctgcagagatgctagccaacatagacttgaaatatttcaatgattcaactaaaaagaaaattgaaaaaatccgaccattgttaatagatggaactgcatcattgagtcctggaatgatgatgggcatgttcaatatgttgagcaccgtcttgggcgtctccattctgaatcttgggcaaaagagatacaccaagactacttactggtgggatggtcttcaatcgtctgatgattttgctttgattgtgaacgcacccaactatgcaggaattcaagctggagttgacaggttctatcgaacctgtaagctgctcggaattaatatgagcaaaaagaagtcttacataaacagaacaggtacctttgaattcacgagctttttctatcgttatgggtttgttgccaatttcagcatggagcttcctagttttggggtgtctggggtcaatgaatctgcagacatgagtattggagtcactgtcatcaaaaacaatatgataaacaatgaccttggcccagcaactgctcaaatggcccttcagttatttataaaagattacaggtacacttatcgatgccaccgaggtgacacacaaatacaaacccgaagatcatttgagataaagaaactatgggaccaaacccgctcaaaagctggactgttggtctctgatggaggccccaatttgtataacattagaaatctccatattcctgaagtttgcttgaaatgggagttgatggatgaggattaccaggggcgtttatgcaacccattaaacccgtttgtcagccataaagagattgaatcagtgaacagtgcagtgataatgccggcacatggtccagccaaaaatatggagtatgacgctgttgcaacaacacactcctgggtccccaaaagaaatcgatccattttgaacacgagccaaagggggatacttgaagatgagcaaatgtatcagaggtgctgcaatttatttgaaaaattcttcccaagtagctcatacagaagaccagttggaatatccagtatggtagaggctatggtctcaagagcccgaattgatgcacggattgatttcgaatctggaaggataaagaaagaggaatttgctgagatcatgaagatctgttccaccattgaagacctcagacggcaaaaatga

>H1N1_PB1_CY147833_A/Mexico/24044/2009

atggatgtcaatccgactctacttttcctaaaaattccagcgcaaaatgccataagcaccacattcccttatactggagatcctccatacagccatggaacaggaacaggatacaccatggacacagtaaacagaacacaccaatactcagaaaagggaaagtggacgacaaacacagagactggtgcaccccagctcaacccgattgatggaccactacctgaggataatgaaccaagtgggtatgcacaaacagactgtgttctagaggctatggctttccttgaagaatcccacccaggaatatttgagaattcatgccttgaaacaatggaagttgttcaacaaacaagggtagataaactaactcaaggtcgccagacttatgattggacattaaacagaaatcaaccggcagcaactgcattggccaacaccatagaagtctttagatcgaatggcctaacagctaatgagtcaggaaggctaatagatttcttaaaggatgtaatggaatcaatgaacaaagaggaaatagagataacaacccactttcaaagaaaaaggagagtaagagacaacatgaccaagaagatggtcacgcaaagaacaatagggaagaaaaaacaaagactgaataagagaggctatctaataagagcactgacattaaatacgatgaccaaagatgcagagagaggcaagttaaaaagaagggctatcgcaacacctgggatgcagattagaggtttcgtatactttgttgaaactttagctaggagcatttgcgaaaagcttgaacagtctgggctcccagtagggggcaatgaaaagaaggccaaactggcaaatgttgtgagaaagatgatgactaattcacaagacacagagatttctttcacaatcactggggacaacactaagtggaatgaaaatcaaaatcctcgaatgttcctggcgatgattacatatatcaccagaaatcaacccgagtggttcagaaacatcctgagcatggcacccataatgttctcaaacaaaatggcaagactagggaaagggtacatgttcgagagtaaaagaatgaagattcgaacacaaataccagcagaaatgctagcaagcattgacctgaagtacttcaatgaatcaacaaagaagaaaattgagaaaataaggcctcttctaatagatggcacagcatcactgagtcctgggatgatgatgggcatgttcaacatgctaagtacggtcttgggagtctcgatactgaatcttggacaaaagaaatacaccaagacaatatactggtgggatgggctccaatcatccgacgattttgctctcatagtgaatgcaccaaaccatgagggaatacaagcaggagtggacagattctacaggacctgcaagttagtgggaatcaacatgagcaaaaagaagtcctatataaataagacagggacatttgaattcacaagctttttttatcgctatggatttgtggctaattttagcatggagctacccagctttggagtgtctggagtaaatgaatcagctgacatgagtattggagtaacagtgataaagaacaacatgataaacaatgaccttggacctgcaacggcccagatggctcttcaattgttcatcaaagactacagatacacatataggtgccataggggagacacacaaattcagacgagaagatcatttgagttaaagaagctgtgggatcaaacccaatcaaaggtagggctattagtatcagatggaggaccaaacttatacaatatacggaatcttcacattcctgaagtctgcttaaaatgggagctaatggatgatgattatcggggaagactttgtaatcccctgaatccctttgtcagtcataaagagattgattctgtaaacaatgctgtggtaatgccagcccatggtccagccaaaagcatggaatatgatgccgttgcaactacacattcttggattcccaagaggaatcgttctattctcaacacaagccaaaggggaattcttgaggatgaacagatgtaccagaagtgctgcaatctattcgagaaatttttccctagcagttcatataggagaccggttggaatttctagcatggtggaggccatggtgtctagggcccggattgatgccagggtcgacttcgagtctggacggatcaagaaagaagagttctctgagatcatgaagatctgttccaccattgaagaactcagacggcaaaaataa

>H1N1_PB1_KF559924_A/Helsinki/668/2013

atggatgtcaatccgactctacttttcctaaaaattccagcacaaaatgccataagcaccacattcccttatactggagatcctccatacagccatggaacaggaacaggatacaccatggacacagtaaacagaacacatcaatactcagaaaagggaaagtggacaacaaacacagagactggtgcaccccagctcaacccgattgatggaccactacctgaggataatgaaccaagtgggtatgcacaaacagactgtgttctagaggctatggctttccttgaagaatcccacccaggaatatttgagaattcatgtcttgaaacaatggaagttgttcaacaaacaagggtagacaaactgactcaaggtcgccagacttatgattggacattaaacagaaatcaaccggcagcaactgcattggccaacaccatagaagtatttagatcgaatgacctaacagctaatgagtcaggaaggctaatagatttcttaaaggatgtaatggaatcaatgaacaaagaggaaatagagataacaacccactttcaaagaaaaaggagagtaagagacaacatgaccaagaagatggtcacgcaaagaacaatagggaagaaaaaacagagactgaataagagaggctatctaataagagcactgacattaaatacgatgaccaaagatgcagagagaggcaagttaaaaagaagggctatcgcaacacctgggatgcagattagaggtttcgtatactttgttgaaactttagctaggagcatttgcgaaaagcttgaacagtctgggctcccagtagggggcaatgaaaagaaggccaaactggcaaatgttgtgagaaagatgatgactaattcacaagacacagagatttctttcacaatcactggggacaacactaagtggaatgaaaatcaaaatcctcgaatgttcctggcgatgattacatatatcaccagaaatcaacccgagtggtttagaaacatcctgagcatggcacccataatgttctcaaacaaaatggcaagactagggaaagggtacatgttcgagagtaaaagaatgaagatccgaacacaaataccagcagaaatgctagcaagcattgacctgaagtacttcaatgaatcaacaaagaagaaaattgagaaaataaggcctcttttaatggatggcacagcatcactgagtcctgggatgatgatgggcatgttcaacatgctaagtacggtcttgggagtctcgatactgaatcttggacaaaagaagtacaccaagacaacatactggtgggatgggctccaatcatccgacgattttgctctcatagtgaatgcaccaaactatgagggaatacaagcaggagtggacagattctacaggacctgcaagttagtgggaatcaacatgagcaaaaagaagtcctatataaataagacagggacatttgaattcacaagctttttttatcgctatggatttgtggctaattttagcatggagctacccagctttggagtgtctggggtaaatgaatcagctgacatgagtattggagtaacagtgataaagaacaacatgataaacaatgaccttggacctgcaacggcccagatggctcttcaattgttcatcaaagactacagatacacatataggtgccataggggagacacacaaattcagacgagaagatcatttgagttaaagaagctgtgggatcaaacccaatcaaaggtagggctattagtatcagatggaggaccaaacttatacaatatacggaatcttcacattcctgaagtctgcttaaaatgggagctaatggatgatgattatcggggaagactttgtaatcccctgaatccctttgtaagtcataaggagattgattctgtaaacaatgctgtggtaatgccagcccatggtccagccaaaagcatggaatatgatgccgttgcaactacacattcctggattcccaagaggaatcgttctattctcaacacaagccaaaggggaattcttgaggatgaacagatgtaccagaagtgctgcaatctattcgagaaatttttccctagcagttcatataggagaccggttggaatttctagcatggtggaggccatggtgtctagggcccggattgatgccagggtcgactttgagtctggacggatcaagaaagaagagttctctgagatcatgaagatctgttccaccattgaagaactcagacggcaaaaataa

>H1N1_PB1_KF559916_A/Helsinki/664/2013

atggatgtcaatccgactctacttttcctaaaaattccagcgcaaaatgccataagcaccacattcccttatactggagatcctccatacagccatggaacaggaacaggatacaccatggacacagtaaacagaacacaccaatactcagaaaagggaaagtggacaacaaacacagagactggtgcaccccagctcaacccgattgatggaccactacctgaggataatgaaccaagtgggtatgcacaaacagactgtgttctagaggctatggctttccttgaagaatcccacccaggaatatttgagaattcatgccttgaaacaatggaagttgttcaacaaacaagggtagataaactaactcaaggtcgccagacttatgattggacattaaacagaaatcaaccggcagcaactgcattggccaacaccatagaagtctttagatcgaatggcctaacagctaatgagtcaggaaggctaatagatttcttaaaggatgtaatggaatcaatgaacaaagaggaaatagagataacaacccactttcaaagaaaaaggagagtaagagacaacatgaccaagaagatggtcacgcaaagaacaatagggaagaaaaaacaaagactgaataagagaggctatctaataagagcactgacattaaatacgatgaccaaagatgcagagagaggcaagttaaaaagaagggctatcgcaacacctgggatgcagattagaggtttcgtatactttgttgaaactttagctaggagcatttgcgaaaagcttgaacagtctggactcccagtagggggcaatgaaaagaaggccaaactggcaaatgttgtgagaaagatgatgactaattcacaagatacagagatttctttcacaatcactggggacaacactaagtggaatgaaaatcaaaatcctcgaatgttcctggcgatgattacatatatcaccagaaatcaacccgagtggtttagaaacatcctgagcatggcacccataatgttctcaaataaaatggcaagactagggaaagggtacatgttcgagagtaaaagaatgaagattcgaacacaaataccagcagaaatgctagcaagcattgatctgaagtacttcaatgaatcaacaaagaagaaaattgagaaaataaggcctcttttaatggatggcacagcatcactgagtcctgggatgatgatgggcatgttcaacatgctaagtacggtcttgggagtctcgatactgaatcttggacaaaagaaatacaccaagacaatatactggtgggatgggctccaatcatccgacgattttgctctcatagtgaatgcaccaaaccatgagggaatacaagcaggagtggacagattctacaggacctgcaagttagtgggaatcaacatgagcaaaaagaagtcctatataaataagacagggacatttgaattcacaagctttttttatcgctatggatttgtggctaattttagcatggagctacccagctttggagtgtctggagtaaatgaatcagctgacatgagtattggagtaacagtgataaagaacaacatgataaacaatgaccttggacctgcaacggcccagatggctcttcaattgttcatcaaagactacagatacacatataggtgccataggggagacacacaaattcagacgagaagatcatttgagttaaagaagctgtgggatcaaacccaatcaaaggtagggctattagtatcagatggaggaccaaacttatacaatatacggaatcttcacattcctgaagtctgcttaaaatgggagctaatggatgatgattatcggggaagactttgtaatcccctgaatccctttgtaagtcataaggagattgattctgtaaacaatgctgtggtaatgccagcccatggtccagccaaaagcatggaatatgatgccgttgcaactacacattcctggattcccaagaggaatcgttctattctcaacacaagccaaaggggaattcttgaggatgaacagatgtaccagaagtgttgcaatctattcgagaaatttttccctagcagttcatataggagaccggttggaatttctagcatggtggaggccatggtatctagggcccggattgatgccagggtcgactttgagtctggacggatcaagaaagaagagttctctgagatcatgaagatctgttccaccattgaagaactcagacggcaaaaataa

>H1N1_PB1_KF559892_A/Helsinki/629/2013

atggatgtcaatccgactctacttttcctaaaaattccagcacaaaatgccataagcaccacattcccttatactggagatcctccatacagccatggaacaggaacaggatacaccatggacacagtaaacagaacacaccaatactcagaaaagggaaagtggacaacaaacacagagactggtgcaccccagctcaacccgattgatggaccactacctgaggataatgaaccaagcgggtatgcacaaacagactgtgttctagaggctatggctttccttgaagaatcccacccaggaatatttgagaattcatgccttgaaacaatggaagttgttcaacaaacaagagtagataaactgactcaaggtcgccagacttatgattggacattaaacagaaatcaaccggcagcaactgcattggccaacaccatagaagtctttagatcgaatgacctaacagctaacgagtcaggaaggctaatagatttcttaaaggatgtaatggaatcaatgaacaaagaggaaatagagataacaacccactttcaaagaaaaaggagaataagagacaacatgaccaagaagatggtcacgcaaagaacaatagggaagaaaaaacagagactgaataagagaggctatctaataagagcactgacattaaatacgatgaccaaagatgcagagagaggcaagttaaaaagaagggctatcgcaacacctgggatgcagattagaggtttcgtatactttgttgaaactttagctaggagcatttgcgaaaagcttgaacagtctggactcccagtagggggcaatgaaaagaaggccaaactggcaaatgttgtgagaaagatgatgactaattcacaagatacagagatttctttcacaatcactggggacaacactaagtggaatgaaaatcaaaatcctcgaatgttcctggcgatgattacatatatcaccagaaatcaacccgagtggtttagaaacatcctgagcatggcacccataatgttctcaaataaaatggcaagactagggaaagggtacatgttcgagagtaaaagaatgaagattcgaacacaaataccagcagaaatgctagcaagcattgatctgaagtacttcaatgaatcaacaaagaagaaaattgagaaaataaggcctcttttaatggatggcacagcatcactgagtcctgggatgatgatgggcatgttcaacatgctaagtacggtcttgggagtctcgatactgaatcttggacaaaagaaatacaccaagacaacatactggtgggatgggctccaatcatccgacgattttgctctcatagtgaatgcaccaaaccatgagggaatacaagcaggagtggacagattctacaggacctgcaagttagtgggaatcaacatgagcaaaaagaagtcctatataaataagacagggacatttgaattcacaagctttttttatcgctatggatttgtggctaattttagcatggagctacccagctttggagtgtctggagtaaatgaatcagctgacatgagtattggagtaacagtgataaagaacaacatgataaacaatgaccttggacctgcaacagcccagatggctcttcaattgttcatcaaagactacagatacacatataggtgccataggggagacacacaaattcagacgagaagatcatttgagttaaagaagctgtgggatcaaacccaatcaaaggtagggctattagtatcagatggaggaccaaacttatacaatatacggaatcttcacattcctgaagtctgcttaaaatgggagctaatggatgatgattatcggggaagactttgtaatcccctgaatccctttgtaagtcataaggagattgattctgtaaacaatgctgtggtaatgccagcccatggtccagccaaaagcatggaatatgatgccgttgcaactacacattcctggattcccaagaggaatcgttctattctcaacacaagccaaaggggaattcttgaggatgaacagatgtaccagaagtgttgcaatctattcgagaaatttttccctagcagttcatataggagaccggttggaatttctagcatggtggaggccatgatatctagggcccggattgatgccagggtcgactttgagtctggacggatcaagaaagaagagttctcagagatcatgaagatctgttccaccattgaagaactcagacggcaaaaataa

>H1N1_PB1_KF559804_A/Helsinki/568/2013

atggatgtcaatccgactctacttttcctaaaaattccagcacaaaatgccataagcaccacattcccttatactggagatcctccatacagccatggaacaggaacaggatacaccatggacacagtaaacagaacacaccaatactcagaaaagggaaagtggacaacaaacacagagactggtgcaccccagctcaacccgattgatggaccactacctgaggataatgaaccaagtgggtatgcacaaacagactgtgttctagaggctatggctttccttgaagaatcccacccaggaatatttgagaattcatgccttgaaacaatggaagttgttcaacaaacaagggtagataaactgactcaaggtcgccagacttatgattggacattaaacagaaatcaaccggcagcaactgcattggccaacaccatagaagtctttagatcgaatgacctaacagctaacgagtcaggaaggctaatagatttcttaaaggatgtaatggaatcaatgaacaaagaggaaatagagataacaacccactttcaaagaaaaagaagagtaagagacaacatgaccaagaagatggtcacgcaaagaacaatagggaagaaaaaacagagactgaataagagaggctatctaataagagcactgacattaaatacgatgaccaaagatgcagagagaggcaagttaaaaagaagggctatcgcaacacctgggatgcagattagaggtttcgtatactttgttgaaactttagctaggagcatttgcgaaaagcttgaacagtctggactcccagtagggggcaatgaaaagaaggccaaattggcaaatgttgtgagaaagatgatgactaattcacaagatacagagatttctttcacaatcactggggacaacactaagtggaatgaaaatcaaaatcctcgaatgttcctggcgatgattacatatatcaccagaaatcaacccgagtggtttagaaacatactgagcatggcacccataatgttctcaaataaaatggcaagactagggaaagggtacatgttcgagagtaaaagaatgaagattcgaacacaaataccagcagaaatgctagcaagcattgatctgaagtacttcaatgaatcaacaaagaagaaaattgagaaaataaggcctcttttaatggatggcacagcatcattgagtcctgggatgatgatgggcatgttcaacatgctaagtacggtcttgggagtctcgatactgaatcttggacaaaagaaatacaccaagacaacatactggtgggatgggctccaatcatccgacgattttgctctcatagtgaatgcaccaaaccatgagggaatacaagcaggagtggacagattctacaggacctgcaagttagtgggaatcaacatgagcaaaaagaagtcctatataaataagacagggacatttgaattcacaagctttttttatcgctatggatttgtggctaattttagcatggagctacccagctttggagtgtctggagtaaatgaatcagctgacatgagtattggagtaacagtgataaagaacaacatgataaacaatgaccttggacctgcaacggcccagatggctcttcaattgttcatcaaagactacagatacacatataggtgccataggggagacacacaaattcagacgagaagatcatttgagttaaagaagctgtgggatcaaacccaatcaaaggtagggctattagtatcagatggaggaccaaacttatacaatatacggaatcttcacattcctgaagtctgcttaaaatgggagctaatggatgatgattatcggggaagactttgtaatcccctgaatccctttgtaagtcataaggagattgattctgtaaacaatgctgtggtaatgccagcccatggtccagccaaaagcatggaatatgatgccgttgcaactacacattcctggattcccaagaggaatcgttctattctcaacacaagccaaaggggaattcttgaggatgaacagatgtaccagaagtgttgcaatctattcgagaaatttttccctagcagttcatataggagaccggttggaatttctagcatggtggaggccatggtatctagggcccggattgatgccagggtcgactttgagtctggacggatcaagaaagaagagttctcagagatcatgaagatctgttccaccattgaagaactcagacggcaaaaataa

>H1N1_PB1_KF559788_A/Helsinki/551/2013

atggatgtcaatccgactctacttttcctaaaaattccagcacaaaatgccataagcaccacattcccttatactggagatcctccatacagccatggaacaggaacaggatacaccatggacacagtaaacagaacacaccaatactcagaaaagggaaagtggacaacaaacacagagactggtgcaccccagctcaacccgattgatggaccactacctgaggataatgaaccaagtgggtatgcacaaacagactgtgttctagaggctatggctttccttgaagaatcccacccaggaatatttgagaattcatgccttgaaacaatggaagttgttcaacaaacaagggtagataaactgactcaaggtcgccagacttatgattggacattaaacagaaatcaaccggcagcaactgcattggccaacaccatagaagtctttagatcgaatgacctaacagctaacgagtcaggaaggctaatagatttcttaaaggatgtaatggaatcaatgaacaaagaggaaatagagataacaacccactttcaaagaaaaagaagagtaagagacaacatgaccaagaagatggtcacgcaaagaacaatagggaagaaaaaacagagactgaataagagaggctatctaataagagcactgacattaaatacgatgaccaaagatgcagagagaggcaagttaaaaagaagggctatcgcaacacctgggatgcagattagaggtttcgtatactttgttgaaactttagctaggagcatttgcgaaaagcttgaacagtctggactcccagtagggggcaatgaaaagaaggccaaattggcaaatgttgtgagaaagatgatgactaattcacaagatacagagatttctttcacaatcactggggacaacactaagtggaatgaaaatcaaaatcctcgaatgttcctggcgatgattacatatatcaccagaaatcaacccgagtggtttagaaacatcctgagcatggcacccataatgttctcaaataaaatggcaagactagggaaagggtacatgttcgagagtaaaagaatgaagattcgaacacaaataccagcagaaatgctagcaagcattgatctgaagtacttcaatgaatcaacaaagaagaaaattgagaaaataaggcctcttttaatggatggcacagcatcactgagtcctgggatgatgatgggcatgttcaacatgctaagtacggtcttgggagtctcgatactgaatcttggacaaaagaaatacaccaagacaacatactggtgggatgggctccaatcatccgacgattttgctctcatagtgaatgcaccaaaccatgagggaatacaagcaggagtggacagattctacaggacctgcaagttagtgggaatcaacatgagcaaaaagaagtcctacataaataagacagggacatttgaattcacaagctttttttatcgctatggatttgtggctaattttagcatggagctacccagctttggagtgtctggagtaaatgaatcagctgacatgagtattggagtaacagtgataaagaacaacatgataaacaatgaccttggacctgcaacggcccagatggctcttcaattgttcatcaaagactacagatacacatataggtgccataggggagacacacaaattcagacgagaagatcatttgagttaaagaagctgtgggatcaaacccaatcaaaggtagggctattagtatcagatggaggaccaaacttatacaatatacggaatcttcacattcctgaagtctgcttaaaatgggagctaatggatgatgattatcggggaagactttgtaatcccctgaatccctttgtaagtcataaggagattgattctgtaaacaatgctgtggtaatgccagcccatggtccagccaaaagcatggaatatgatgccgttgcaactacacattcctggattcccaagaggaatcgttctattctcaacacaagccaaaggggaattcttgaggatgaacagatgtaccagaagtgttgcaatctattcgagaaatttttccctagcagttcatataggagaccggttggaatttctagcatggtggaggccatggtatctagggcccggattgatgccagggtcgactttgagtctggacggatcaagaaagaagagttctcagagatcatgaagatctgttccaccattgaagaactcagacggcaaaaataa

>H1N1_PB1_KF559644_A/Helsinki/440/2013

atggatgtcaatccgactctacttttcctaaaaattccagcacaaaatgccataagcaccacattcccttatactggagatcctccatacagccatggaacaggaacaggatacaccatggacacagtaaacagaacacaccaatactcagaaaagggaaagtggacaacaaacacagagactggtgcaccccagctcaacccgattgatggaccactacctgaggataatgaaccaagtgggtatgcacaaacagactgtgttctagaggctatggctttccttgaagaatcccacccaggaatatttgagaattcatgccttgaaacaatggaagttgttcaacaaacaagggtagataaactgactcaaggtcgccagacttatgattggacattaaacagaaatcaaccggcagcaactgcattggccaacaccatagaagtctttagatcgaatgacctaacagctaacgagtcaggaaggctaatagatttcttaaaggatgtaatggaatcaatgaacaaagaggaaatagagataacaacccactttcaaagaaaaaggagagtaagagacaacatgaccaagaagatggtcacgcaaagaacaatagggaagaaaaaacagagactgaataagagaggctatctaataagagcactgacattaaatacgatgaccaaagatgcagagagaggcaagttaaaaagaagggctatcgcaacacctgggatgcagattagaggtttcgtatactttgttgaaactttagctaggagcatttgcgaaaagcttgaacagtctgggctcccagtagggggcaatgaaaagaaggccaaactggcaaatgttgtgagaaagatgatgactaattcacaagatacagagatttctttcacaatcactggggacaacactaagtggaatgaaaatcaaaatcctcgaatgttcctggcgatgattacatatatcaccagaaatcaacccgagtggtttagaaacatcctgagcatggcacccataatgttctcaaataaaatggcaagactagggaaagggtacatgttcgagagtaaaagaatgaagattcgaacacaaataccagcagaaatgctagcaagcattgatctgaagtacttcaatgaatcaacaaagaagaaaattgagaaaataaggcctcttttaatggatggcacagcatcactgagtcctgggatgatgatgggcatgttcaacatgctaagtacggtcttgggagtctcgatactgaatcttggacaaaagaaatacaccaagacaacatactggtgggatgggctccaatcatccgacgattttgctctcatagtgaatgcaccaaaccatgagggaatacaagcaggagtggacagattctacaggacctgcaagttagtgggaatcaacatgagcaaaaagaagtcctatataaataagacagggacatttgaattcacaagctttttttatcgctatggatttgtggctaattttagcatggagctacccagctttggagtgtctggagtaaatgaatcagctgacatgagtattggagtaacagtgataaagaacaacatgataaacaatgaccttggacctgcaacggcccagatggctcttcaattgttcatcaaagactacagatacacatataggtgccataggggagacacacaaattcagacgagaagatcatttgagttaaagaagctgtgggatcaaacccaatcaaaggtagggctattagtatcagatggaggaccaaacttatacaatatacggaatcttcacattcctgaagtctgcttaaaatgggagctaatggatgatgattatcggggaagactttgtaatcccctgaatccctttgtaagtcataaggagattgattctgtaaacaatgctgtggtaatgccagcccatggtccagccaaaagcatggaatatgatgccgttgcaactacacattcctggattcccaagaggaatcgttctattctcaacacaagccaaaggggaattcttgaggatgaacagatgtaccagaagtgttgcaatctattcgagaaatttttccctagcagttcatataggagaccggttggaatttctagcatggtggaggccatggtatctagggcccggattgatgccagggtcgactttgagtctggacggatcaagaaagaagagttctcagagatcatgaagatctgttccaccattgaagaactcagacggcaaaaataa

>H1N1_PB1_KF791455_A/Czech Republic/187/2013

atggatgtcaatccgactctacttttcctaaaaattccagcacaaaatgccataagcaccacattcccttatactggagatcctccatacagccatggaacaggaacaggatacaccatggacacagtaaacagaacacaccaatactcagaaaagggaaagtggacaacaaacacagagactggtgcaccccagctcaacccgattgatggaccactacctgaggataatgaaccaagtgggtatgcacaaacagactgtgttctagaggctatggctttccttgaagaatcccacccaggaatatttgagaattcatgccttgaaacaatggaagttgttcaacaaacaagggtagataaactgactcaaggtcgccagacttatgattggacattaaacagaaatcaaccggcagcaactgcattggccaacaccatagaagtctttagatcgaatgacctaacagctaacgagtcaggaaggctaatagatttcttaaaggatgtaatggaatcaatgaacaaagaggaaatagagataacaacccactttcaaagaaaaaggagagtaagagacaacatgaccaagaagatggtcacgcaaagaacaatagggaagaaaaaacagagactgaataagagaggctatctaataagagccctgacattaaatacgatgaccaaagatgcagagagaggcaagttaaaaagaagggctatcgcaacacctgggatgcaaattagaggtttcgtatactttgttgaaactttagctaggagcatttgcgaaaagcttgaacagtctggactcccagtagggggcaatgaaaagaaggccaaactggcaaatgttgtgagaaagatgatgactaattcacaagatacagagatttctttcacaatcactggggacaacactaagtggaatgaaaatcaaaatcctcgaatgttcctggcgatgattacatatatcaccagaaatcaacccgagtggtttagaaacatcctgagcatggcacccataatgttctcaaataaaatggcaagactagggaaagggtacatgttcgagagtaaaagaatgaagattcgaacacaaataccagcagaaatgctagcaagcattgatctgaagtacttcaatgaatcaacaaagaagaaaattgagaaaataaggcctcttttaatggatggcacagcatcactgagtcctgggatgatgatgggcatgttcaacatgctaagtacggtcttgggagtctcgatactgaatcttggacaaaagaaatacaccaagacaacatactggtgggatgggctccaatcatccgacgattttgctctcatagtgaatgcaccaaaccatgagggaatacaagcaggagtggacagattctacaggacctgcaagttagtgggaatcaacatgagcaaaaagaagtcctatataaataagacagggacatttgaattcacaagctttttttatcgctatggatttgtggctaattttagcatggagctacccagctttggagtgtctggagtaaatgaatcagctgacatgagtattggagtaacagtgataaagaacaacatgataaacaatgaccttggacctgcaacggcccagatggctcttcaattgttcatcaaagactacaggtacacatataggtgccataggggagacacacaaattcagacgagaagatcatttgagttaaagaagctgtgggatcaaacccaatcaaaggtagggctattagtatcagatggaggaccaaacttatacaatatacggaatcttcacattcctgaagtctgcttaaaatgggagctaatggatgatgattatcggggaagactttgtaatcccctgaatccctttgtaagtcataaggagattgattctgtaaacaatgctgtggtaatgccagcccatggtccagccaaaagcatggaatatgatgcagttgcaactacacattcctggattcccaagaggaatcgttctattctcaacacaagccaaaggggaattcttgaggatgaacagatgtaccagaagtgttgcaatctattcgagaaatttttccctagcagttcatataggagaccggttggaatttctagcatggtggaggccatggtatctagggcccggattgatgccagggtcgactttgagtctggacggatcaagaaagaagagttctctgagatcatgaagatctgttccaccattgaagaactcagacggcaaaaataa

>H1N1_PB1_CY167434_A/Tennessee/F1076A/2010

atggatgtcaatccgactctacttttcctaaaaattccagcgcaaaatgccataagcaccacattcccttatactggagatcctccatacagccatggaacaggaacaggatacaccatggacacagtaaacagaacacaccaatactcagaaaagggaaagtggacgacaaacacagagactggtgcaccccagctcaacccgattgatggaccactacctgaggataatgaaccaagtgggtatgcacaaacagactgtgttctagaggctatggctttccttgaagaatcccacccaggaatatttgagaattcatgccttgaaacaatggaagttgttcaacaaacaagggtagataaactaactcaaggtcgccagacttatgattggacattaaacagaaatcaaccggcagcaactgcattggccaacaccatagaagtctttagatcgaatggcctaacagctaatgagtcaggaaggctaatagatttcttaaaggatgtaatggaatcaatgaacaaagaggaaatagagataacaacccactttcaaagaaaaaggagagtaagagacaacatgaccaagaagatggtcacgcaaagaacaatagggaagaaaaaacaaagactgaataagagaggctatctaataagagcactgacattaaatacgatgaccaaagatgcagagagaggcaagttaaaaagaagggctatcgcaacacctgggatgcagattagaggtttcgtatactttgttgaaactttagctaggagcatttgcgaaaagcttgaacagtctgggctcccagtagggggcaatgaaaagaaggccaaactggcaaatgttgtgagaaagatgatgactaattcacaagacacagaaatttctttcacaatcactggggacaacactaagtggaatgaaaatcaaaatcctcgaatgttcctggcgatgattacatatatcaccagaaatcaacccgagtggttcagaaacatcttgagcatggcacccataatgttctcaaacaaaatggcaagactagggaaagggtacatgttcgagagtaaaagaatgaagattcgaacacaaataccagcagaaatgctagcaagcattgacctgaagtacttcaatgaatcaacaaagaagaaaattgagaaaataaggcctcttctaatagatggcacagcatcactgagtcctgggatgatgatgggcatgttcaacatgctaagtacggtcttgggagtctcgatactgaatcttggacaaaagaaatacaccaagacaatatactggtgggatgggctccaatcatccgacgattttgctctcatagtgaatgcaccaaaccatgagggaatacaagcaggagtggacagattctacaggacctgcaagttagtgggaatcaacatgagcaaaaagaagtcctatataaataagacagggacatttgaattcacaagctttttttatcgctatggatttgtggctaattttagcatggagctacccagctttggagtgtctggagtaaatgaatcagctgacatgagtattggagtaacagtgataaagaacaacatgataaacaatgaccttggacctgcaacggcccagatggctcttcaactgttcatcaaagactacagatacacatatagatgccataggggagacacacaaattcagacgagaagatcatttgagttaaagaagctgtgggatcaaacccaatcaaaggtagggctattagtatcagatggaggaccaaacttatacaatatacggaatcttcacattcctgaagtctgcttaaaatgggagctaatggatgatgattatcggggaagactttgtaatcccctgaatccctttgtcagtcataaagagattgattctgtaaacaattctgtggtaatgccagcccatggtccagccaaaagcatggaatatgatgccgttgcaactacacattcctggattcccaagaggaatcgttctattctcaacacaagccaaaggggaattcttgaggatgaacagatgtaccagaagtgctgcaatctattcgagaaatttttccctagcagttcatataggagaccggttggaatttctagcatggtggaggccatggtgtctagggcccggattgatgccagggtcgacttcgagtctggacgaatcgggaaggaagagttctctgagatcatgaagatctgttccaccattgaagaactcagacggcaaaaataa

>H1N1_PB1_CY167778_A/Tennessee/F1035/2010

atggatgtcaatccgactctacttttcctaaaaattccagcgcaaaatgccataagcaccacattcccttatactggagatcctccatacagccatggaacaggaacaggatacaccatggacacagtaaacagaacacaccaatactcagaaaagggaaagtggacgacaaacacagagactggtgcaccccagctcaacccgattgatggaccactacctgaggataatgaaccaagtgggtatgcacaaacagactgtgttctagaggctatggctttccttgaagaatcccacccaggaatatttgagaattcatgccttgaaacaatggaagttgttcaacaaacaagggtagataaactaactcaaggtcgccagacttatgattggacattaaacagaaatcaaccggcagcaactgcattggccaacaccatagaagtctttagatcgaatggcctaacagctaatgagtcaggaaggctaatagatttcttaaaggatgtaatggaatcaatgaacaaagaggaaatagagataacaacccactttcaaagaaaaaggagagtaagagacaacatgaccaagaagatggtcacgcaaagaacaatagggaagaaaaaacaaagactgaataagagaggctatctaataagagcactgacattaaatacgatgaccaaagatgcagagagaggcaagttaaaaagaagggctatcgcaacacctgggatgcagattagaggtttcgtatactttgttgaaactttagctaggagcatttgcgaaaagcttgaacagtctgggctcccagtagggggcaatgaaaagaaggccaaactggcaaatgttgtgagaaagatgatgactaattcacaagacacagagatttctttcacaatcactggggacaacactaagtggaatgaaaatcaaaatcctcgaatgttcctggcgatgattacatatatcaccagaaatcaacccgagtggttcagaaacatcctgagcatggcacccataatgttctcaaacaaaatggcaagactagggaaagggtacatgttcgagagtaaaagaatgaagattcgaacacaaataccagcagaaatgctagcaagcattgacctgaagtacttcaatgaatcaacaaagaagaaaattgagaaaataaggcctcttctaatagatggcacagcatcactgagtcctgggatgatgatgggcatgttcaacatgctaagtacggtcttgggagtctcgatactgaatcttggacaaaagaaatacaccaagacaatatactggtgggatgggctccaatcatccgacgattttgctctcatagtgaatgcaccaaaccatgagggaatacaagcaggagtggacagattctacaggacctgcaagttagtgggaatcaacatgagcaaaaagaagtcctatataaataagacagggacatttgaattcacaagctttttttatcgctatggatttgtggctaattttagcatggagctacccagctttggagtgtctggagtaaatgaatcagctgacatgagtattggagtaacagtgataaagaacaacatgataaacaatgaccttggacctgcaacggcccagatggctcttcaactgttcatcaaagactacagatacacatataggtgccataggggagacacacaaattcagacgagaagatcatttgagttaaagaagctgtgggatcaaacccaatcaaaggtagggctattagtatcagatggaggaccaaacttatacaatatacggaatcttcacattcctgaagtctgcttaaaatgggagctaatggatgatgattatcggggaagactttgtaatcccctgaatccctttgtcagtcataaagagattgattctgtaaacaatgctgtggtaatgccagcccatggtccagccaaaagcatggaatatgatgccgttgcaactacacattcctggattcccaagaggaatcgttctattctcaacacaagccaaaggggaattcttgaggatgaacagatgtaccagaagtgctgcaatctattcgagaaatttttccctagcagttcatataggagaccggttggaatttctagcatggtggaggccatggtgtctagggcccggattgatgccagggtcgacttcgagtctggacggatcgggaaggaagagttctctgagatcatgaagatctgttccaccattgaagagctcagacggcaaaaataa

>H1N1_PB1_CY172549_A/New York/1050/2006

atggatgtcaatccgacattacttttcttaaaagtgccagcacaaaatgctataagcacaacttttccttataccggtgaccctccttacagccatggaacaggaacagggtacaccatggatacagtcaacaggacacaccagtactcagaaagaggaagatggacaaaaaataccgaaactggagcaccgcaactcaacccaattgatgggccactaccagaagacaatgaaccaagtggctatgcccaaacagattgtgtattagaagcaatggctttccttgaagaatcccatcctggtatctttgaaaactcttgtattgaaacaatggaggttgttcagcaaacaagggtggacaaactgacacaaggcagacagacctatgactggactctaaataggaaccagcctgctgcaacagcattggcaaacactatagaagtattcagatcaaacggcctcatagcaaatgaatctggaaggctaatagacttccttaaagatgtaatggagtcgatggacagagacgaagtagaggtcacaactcattttcaaagaaagaggagagtgagagacaatgtaactaaaaaaatggtgactcaaagaacaataggcaaaaagaaacataaattagacaaaagaagttacctaattagggcattaaccctgaacacaatgaccaaagatgctgagagggggaaactaaaacgcagagcaattgcaaccccaggaatgcaaataagagggtttgtatactttgttgagacactagcaagaagcatatgtgaaaagcttgaacaatcaggattgccagttggaggaaatgagaagaaagcaaagttagcaaatgttgtaaggaagatgatgaccaactcccaggacactgaaatttctttcaccatcactggagataacacaaaatggaacgaaaatcaaaaccctagaatgttcttggccatgatcacatatataaccaaaaatcagcctgaatggttcagaaatattctaagtattgctccaataatgttttcaaacaagatggcgagactaggtaaggggtacatgtttgaaagcaagagtatgaaactgagaactcaaatacctgcagagatgctagccaacatagatttgaaatatttcaatgattcaactaaaaagaaaattgaaaaaatccgaccattattaatagatggaactgcatcattaagtcctggaatgatgatgggcatgttcaatatgttgagcaccgtcttgggcgtctccattctgaatcttgggcaaaagagatacaccaagactacatactggtgggatggtcttcaatcgtctgatgattttgctttgattgtgaatgcacccaactatgcaggaattcaagctggagttgacaggttttatcgaacctgtaagctgctcggaattaatatgagcaaaaagaagtcttacataaacagaacaggtacctttgaattcacgagctttttctatcgttatgggtttgttgccaatttcagcatggagcttcctagttttggggtgtctggggtcaatgaatctgcagacatgagtattggagtcactgtcatcaaaaacaatatgataaacaatgaccttggcccagcaactgctcaaatggcccttcagttatttataaaagattacaggtacacttatcgatgccacagaggtgacacacaaatacaaacccggagatcatttgagataaagaaactatgggaccaaacccgctccaaagctgggctgttggtctctgatggaggccccaatttatataacattagaaatctccatattcctgaagtttgcttgaaatgggagttgatggatgaggattaccaggggcgtttatgcaacccattaaacccgtttgtcagccataaagagattgaatcagtgaacaatgcagtgataatgccggcacatggcccagccaaaaatatggagtatgacgctgttgcaacaacacactcctgggtccccaaaagaaatcgatccattttgaacacgagccaaagggggatacttgaagatgagcaaatgtatcagaggtgctgcaatttatttgaaaaattcttcccaagtagctcatacagaagaccagttggaatatccagtatggtagaggctatggtttcaagagcccgaattgatgcacggattgatttcgaatctggaaggataaagagggaggaatttgctgagatcatgaagatctgttccaccattgaagacctcagacggcaaaaatag

>H1N1_PB1_CY172973_A/New York/1112/2008

atggatgtcaatccgacattacttttcttaaaagtgccagcacaaaatgctataagcacaacttttccttatactggtgaccctccttacagccatgggacaggaacaggatacaccatggatacagtcaacaggacacaccagtactcagaaagaggaagatggacaaaaaataccgaaactggagcaccgcaacttaacccaattgatggtccactaccggaagacaatgaaccaagtggctatgcccaaacagattgtgtattagaagcaatggctttccttgaagaatcccatcccggtatctttgaaaactcctgtattgaaacaatggaggttgttcagcaaacaagggtggacaaactgacacaaggcagacagacctatgactggactctaaataggaaccagcctgctgccacagcattggcaaacactatagaagtattcagatcaaacggcctcatagcaaatgaatctgggaggctaatagacttccttaaagatgtaatggagtcgatggacagaggcgaagtagaggtcacaactcattttcaaagaaagaggagagtgagagacaatgtaactaaaaaaatggtgacccaaagaacaataggcaaaaagaaacataaattagacaaaagaagttacctaattagggcattaaccctgaacacaatgaccaaagatgctgagagggggaaactaaaacgcagagcaattgcaaccccaggaatgcaaataagggggtttgtatactttgttgagacactggcaagaagcatatgtgaaaagcttgaacaatcaggattgccagttggaggaaatgagaagaaagcaaagttagcaaatgttgtaaggaagatgatgaccaactcccaggacactgaaatttctttcaccataaccggagataacacaaaatggaacgaaaatcaaaaccctagaatgttcttggccatgatcacatatataaccaaaaatcagcctgaatggttcagaaatattctaagtattgctccaataatgttttcaaacaagatggcgagactaggtaaggggtacatgtttgaaagcaagagtatgaaactgagaactcaaatacctgcagagatgctagccaacatagatttgaaatatttcaatgattcaactaaaaagaaaattgaaaaaatccgaccattattaatagatggaactgcatcattgagtcctggaatgatgatgggcatgttcaatatgttgagcaccgtcttgggcgtctccattctgaatcttgggcaaaagagatacaccaagactacttactggtgggatggtcttcaatcgtctgatgattttgctttgattgtgaatgcacccaactatgcaggaattcaagctggagttgacaggttttatcgaacctgtaagctgctcggaattaatatgagcaaaaagaagtcttacataaacaaaacaggtacctttgaattcacgagctttttctatcgttatgggtttgttgccaatttcagcatggagcttcctagtttcggggtgtctggggtcaatgaatctgcagacatgagtattggagtcactgtcatcaaaaacaatatgataaacaatgaccttggcccagcaactgctcaaatggcccttcagttatttataaaagattacaggtacacttatcgatgccaccgaggtgacacacaaatacaaacccggagatcatttgagataaagaaactatgggaccaaacccgctcaaaggctgggctgttggtctctgatggaggccccaatttatataacattagaaatctccatattcctgaagtttgcttgaaatgggagttgatggatgaggattaccaggggcgtttatgcaacccattaaacccgtttgtcagccataaagagattgaatcagtgaacaatgcagtgataatgccggcacatggtccagccaaaaatatggagtatgacgctgttgcaacaacacactcctgggttcccaaaagaaatcgatccattttgaacacgagccaaagggggatacttgaagatgagcaaatgtatcagaggtgctgcaatttatttgaaaaattcttcccaagtagctcatacagaagaccagttggaatatccagtatggtagaggctatggtctcaagagcccgaattgatgcacggattgatttcgaatctggaaggataaagaaagaggaatttgctgagatcatgaagatctgttccaccattgaagacctcagacggcaaaaatga

>H1N1_PB1_CY173333_A/New York/1161/2009

atggatgtcaatccgacattacttttcttaaaagtgccagcacaaaatgctataagcacaacttttccttatactggtgaccctccttacagccatgggacaggaacagggtacaccatggatacagtcaacaggacacaccagtactcagaaagaggaagatggacaaaaaataccgaaacgggagcaccgcaacttaacccaattgatggtcccttaccggaagacaatgaaccaagtggctatgcccaaacagattgtgtattagaagcaatggctttccttgaagaatcccatcccggtatctttgaaaactcttgtattgaaacaatggaggttgttcaacaaacaagggtggacaaactgacacaaggcagacagacctatgactggactctaaataggaaccagcctgctgccacagcattggcaaacactatagaagtattcagatcaaacggcctcatagcaaatgaatctggaaggctaatagacttccttaaagatgtaatggagtcgatggacagaggcgaagtagaggtcacaactcattttcaaagaaagaggagagtgagagacaatgtaactaaaaaaatggtgacccaaagaacaataggcaaaaagaaacataaattagacaaaagaagttacctaattagggcattaaccctgaacacaatgaccaaagatgctgagagggggaaactaaaacgcagagcaattgcaaccccaggaatgcaaataagggggtttgtatactttgttgagacactggcaagaagcatatgtgaaaagcttgaacaatcaggattgccagttggaggaaatgagaagaaagcaaagttagcaaatgttgtaagaaagatgatgaccaactcccaggacactgaaatttctttcaccataaccggagacaacacaaaatggaacgaaaatcaaaaccctagaatgttcttggccatgatcacatatataaccaaaaatcagcctgaatggttcagaaatattctaagtattgctccaataatgttttcaaacaagatggcgagactaggtaaggggtacatgtttgaaagcaagagtatgaaactgagaactcaaatacctgcagagatgctagccaacatagacttgaaatatttcaatgattcaactaaaaagaaaattgaaaaaatccgaccattattaatagatggaactgcatcattgagtcctggaatgatgatgggcatgttcaatatgttgagcaccgtcttgggcgtctccattctgaatcttgggcaaaagagatacaccaagactacttactggtgggatggtcttcaatcgtctgatgattttgctttgattgtgaacgcacccaactatgcaggaattcaagctggagttgacaggttttatcgaacctgtaagctgctcggaattaatatgagcaaaaagaagtcttacataaacagaacaggtacctttgaattcacgagctttttttatcgttatgggtttgttgccaatttcagcatggagcttcctagttttggggtgtctggggtcaatgaatctgcagacatgagtattggagtcactgtcatcaaaaacaatatgataaacaatgaccttggcccagcaactgctcaaatggcccttcagttatttataaaagattacaggtacacttatcgatgccaccgaggtgacacacaaatacaaacccgaagatcatttgagataaagaaactatgggaccaaacccgctcaaaagctggactgttggtctctgatggaggccccaatttgtataacattagaaatctccatattcctgaagtttgcttgaaatgggagttgatggatgaggattaccaggggcgtttatgcaacccattaaacccgtttgtcagccataaagagattgaatcagtgaacagtgcagtgataatgccggcacatggtccagccaaaaatatggagtatgacgctgttgcaacaacacactcctgggtccccaaaagaaatcgatccattttgaacacgagccaaagggggatacttgaagatgagcaaatgtatcagaggtgctgcaatttatttgaaaaattcttcccaagtagctcatacagaagaccagttggaatatccagtatggtagaggctatggtctcaagagcccgaattgatgcacggattgatttcgaatctggaaggataaagaaagaggaatttgctgagatcatgaagatctgttccaccattgaagacctcagacggcaaaaatga

>H1N1_PB1_CY173397_A/New York/1171/2009

atggatgtcaatccgacattacttttcttaaaagtgccagcacaaaatgctataagcacaacttttccttatactggtgaccctccttacagccatgggacaggaacagggtacaccatggatacagtcaataggacacaccagtactcagaaagaggaagatggacaaaaaataccgaaacgggagcaccgcaacttaacccaattgatggccccttaccggaagacaatgaaccaagtggctatgcccaaacagattgtgtattagaagcaatggctttccttgaagaatcccatcccggtatctttgaaaactcttgtattgaaacaatggaggttgttcaacaaacaagggtggacaaactgacacaaggcagacagacctatgactggactctaaataggaaccagcctgctgccacagcattggcaaacactatagaagtattcagatcaaacggcctcatagcaaatgaatctggaaggctaatagacttccttaaagatgtaatggagtcgatggacagaggcgaagtagaggtcacaactcattttcaaagaaagaggagagtgagagacaatgtaactaaaaaaatggtgacccaaagaacaataggcaaaaagaaacataaattagacaaaagaagttacctaattagggcattaaccctgaacacaatgaccaaagatgctgagagggggaaactaaaacgcagagcaattgcaaccccaggaatgcaaataagggggtttgtatactttgttgagacactggcaagaagcatatgtgaaaagcttgaacaatcaggattgccagttggaggaaatgagaagaaagcaaagttagcaaatgttgtaaggaagatgatgaccaactcccaggacactgaaatttctttcaccataaccggagacaacacaaaatggaacgaaaatcaaaaccctagaatgttcttggccatgatcacatatataaccaaaaatcagcctgaatggttcagaaatattctaagtattgctccaataatgttttcaaacaagatggcgagactaggtaaggggtacatgtttgaaagcaagagtatgaaactgagaactcaaatacctgcagagatgctagccaacatagacttgaaatatttcaatgattcaactaaaaagaaaattgaaaaaatccgaccattattaatagatggaactgcatcattgagtcctggaatgatgatgggcatgttcaatatgttgagcaccgtcctgggcgtctccattctgaatcttgggcaaaagagatacaccaagactacttactggtgggatggtcttcaatcgtctgatgattttgctttgattgtgaacgcacccaactatgcaggaattcaagctggagttgacaggttctatcgaacctgtaagctactcggaattaatatgagcaaaaagaagtcttacataaacagaacaggtacctttgaattcacgagctttttctatcgttatgggtttgttgccaatttcagcatggagcttcctagttttggggtgtctggggtcaatgaatctgcagacatgagtattggagtcactgtcatcaaaaacaatatgataaacaatgaccttggcccagcaactgctcaaatggcccttcagttatttataaaagattacaggtacacttatcgatgccaccgaggtgacacacaaatacaaacccgaagatcatttgagataaagaaactatgggaccaaacccgctcaaaagctggactgttggtctctgatggaggccccaatttgtataacattagaaatctccatattcctgaagtttgcttgaaatgggagttgatggatgaggattaccaggggcgtttatgcaacccattaaacccgtttgtcagccataaagagattgaatcagtgaacagtgcagtgataatgccggcacatggtccagccaaaaatatggagtatgacgctgttgcaacaacacactcctgggtccccaaaagaaatcgatccattttgaacacgagccaaagggggatacttgaagatgagcaaatgtatcagaggtgctgcaatttatttgaaaaattcttcccaagtagctcatacagaagaccagttggaatatccagtatggtagaggctatggtctcaagagcccgaattgatgcacggattgatttcgaatctggaaggataaagaaagaggaatttgctgagatcatgaagatctgttccaccattgaagacctcagacggcaaaaatga

>H1N1_PB1_CY176456_A/Kowloon/INS3_638/2011

atggatgtcaatccgactctacttttcctaaaaattccagcgcaaaatgccataagcaccacattcccttatactggagatcctccatacagccatggaacaggaacaggatacaccatggacacagtaaacagaacacaccaatactcagaaaagggaaagtggacgacgaacacagagactggtgcaccccagctcaacccgattgatggaccactacctgaggataatgaaccaagtgggtatgcacaaacagactgtgttctagaggctatggctttccttgaagaatcccacccaggaatatttgagaattcatgccttgaaacaatggaagttgttcaacaaacaagggtagataaactaactcaaggtcgccagacttatgattggacattaaacagaaatcaaccggcagcaactgcattggccaacaccatagaagtctttagatcgaatggcctaacagctaatgagtcaggaaggctaatagatttcttaaaggatgtaatggaatcaatgaacaaagaggaaatagagataacaacccactttcaaagaaaaaggagagtaagagacaacatgaccaagaagatggtcacgcaaagaacaatagggaagaaaaaacaaagactgaataagagaggctatctaataagagcactgacattaaatacgatgaccaaagatgcagagagaggcaagttaaaaagaagggctatcgcaacacctgggatgcagattagaggtttcgtatactttgttgaaactttagctaggagcatttgcgaaaagcttgaacagtctgggctcccagtagggggcaatgaaaagaaggccaaactggcaaatgttgtgagaaagatgatgactaattcacaagacacagagatttctttcacaatcactggggacaacactaagtggaatgaaaatcaaaatcctcgaatgttcctggcgatgattacatatatcaccagaaatcaacccgagtggttcagaaacatcctgagcatggcacccataatgttctcaaacaaaatggcaagactagggaaagggtacatgttcgagagtaaaagaatgaagattcgaacacaaataccagcagaaatgctagcaagcattgacctgaagtacttcaatgaatcaacaaagaagaaaattgagaaaataaggcctcttttaatagatggcacagcatcactgagtcctgggatgatgatgggcatgttcaacatgctaagtacggtcttgggagtctcgatactgaatcttggacaaaagaaatacaccaagacaatatactggtgggatgggctccaatcatccgacgattttgctctcatagtgaatgcaccaaaccatgagggaatacaagcaggagtggacagattctacaggacctgcaagttagtgggaatcaacatgagtaaaaagaagtcctatataaataagacagggacatttgaattcacaagctttttttatcgctatggatttgtggctaattttagcatggagctacccagctttggagtgtctggagtaaatgaatcagctgacatgagtattggagtaacagtgataaagaacaacatgataaacaatgaccttggacctgcaacggcccagatggctcttcaattgttcatcaaagactacagatacacatataggtgccataggggagacacacaaattcagacgagaagatcatttgagttaaagaagctgtgggatcaaacccaatcaaaggtagggctattagtatcagatggaggaccaaacttatacaatatacggaatcttcacattcctgaagtctgcttaaaatgggagctaatggatgatgattatcggggaagactttgtaatcccctgaatccctttgtaagtcataaagagattgattctgtaaacaatgctgtggtaatgccagcccatggtccagccaaaagcatggagtatgatgccgttgcaactacacattcctggattcccaagaggaatcgttctattctcaacacaagccaaaggggaattcttgaggatgaacagatgtaccagaagtgctgcaatctattcgagaaatttttccctagcagttcatataggagaccggttggaatttctagcatggtggaggccatggtgtctagggcccggattgatgccagggtcgactttgagtctggacggatcaagaaagaagagttctctgagatcatgaagatctgttccaccattgaagaactcagacggcaaaaataa

>H1N1_PB1_CY176552_A/Headington/INS3_650/2011

atggatgtcaatccgactctacttttcctaaaaattccagcgcaaaatgccataagcaccacattcccttatactggagatcctccatacagccatggaacaggaacaggatacaccatggacacagtgaacagaacacaccaatactcagaaaagggaaagtggacgacaaacacagagactggtgcaccccagctcaacccgattgatggaccactacctgaggataatgaaccaagtgggtatgcacaaacagactgtgttctagaggctatggctttccttgaagaatcccacccagggatatttgagaattcatgccttgaaacaatggaagttgttcaacaaacaagggtagataaactaactcaaggtcgccagacttatgattggacattaaacagaaatcaaccggcagcaactgcattggccaacaccatagaagtctttagatcgaatggcctaacagctaatgagtcaggcaggctaatagatttcttaaaggatgtaatggaatcaatgaacaaagaggaaatagagataacaacccactttcaaagaaaaaggagagtaagagacaacatgaccaagaagatggtcacgcaaagaacaatagggaagaaaaaacaaagactgaataagagaggctatctaataagagcactgacattaaatacgatgaccaaagatgcagagagaggcaagttaaaaagaagggctatcgcaacacctgggatgcagattagaggtttcgtatactttgttgaaactttagctaggagcatttgcgaaaagcttgaacagtctgggctcccagtagggggcaatgaaaagaaggccaaactggcaaatgttgtgagaaagatgatgactaattcacaagacacagagatttctttcacaatcactggggacaacactaagtggaatgaaaatcaaaatcctcgaatgttcctggcgatgattacatatatcaccagaaatcaacccgagtggttcagaaacatcctgagcatggcacccataatgttctcaaacaaaatggcaagactagggaaagggtacatgttcgagagtaaaagaatgaagattcgaacacaaataccagcagaaatgctagcaagcattgacctgaagtacttcaatgaatcaacaaagaagaaaattgagaaaataaggcctcttttaatagatggcacagcatcactgagtcctgggatgatgatgggcatgttcaacatgctaagtacggtcttgggagtctcgatactgaatcttggacaaaagaaatacaccaagacaatatactggtgggatgggctccaatcatccgacgattttgctctcatagtgaatgcaccaaaccatgagggaatacaagcaggagtggacagattctacaggacctgcaagttagtggggatcaacatgagcaaaaagaagtcctatataaataagacagggacatttgaattcacaagctttttttatcgctatggatttgtggctaattttagcatggagctacccagctttggagtgtctggagtaaatgaatcagctgacatgagtattggagtaacagtgataaagaacaacatgataaacaatgaccttggacctgcaacggcccagatggctcttcaattgttcatcaaagactacagatacacatataggtgccataggggagacacacaaattcagacgagaagatcatttgagttaaagaagctgtgggatcaaacccaatcaaaggtagggctattagtatcagatggaggaccaaacttatacaatatacggaatcttcacattcctgaagtctgcttaaaatgggagctaatggatgatgattatcggggaagactttgtaatcccctgaatccctttgtaagtcataaagagattgattctgtaaacaatgctgtggtaatgccagcccatggtccagccaaaagcatggaatatgatgccgttgcaactacacattcctggattcccaagaggaatcgttctattctcaacacaagccaaaggggaattcttgaggatgaacagatgtaccagaagtgctgcaatctattcgagaaatttttccctagcagttcatataggagaccggttggaatttctagcatggtggaggccatggtgtctagggcccggattgatgccagggtcgactttgagtctggacggatcaagaaagaagagttctctgagatcatgaagatctgttccaccattgaagaactcagacggcaaaaataa

>H1N1_PB1_KJ561728_A/Czech Republic/190/2013

atggatgtcaatccgactctacttttcctaaaaattccagcacaaaatgccataagcaccacattcccttatactggagatcctccatacagccatggaacaggaacaggatacaccatggacacagtaaacagaacacaccaatactcagaaaagggaaagtggacaacaaacacagagactggtgcaccccagctcaacccgattgatggaccactacctgaggataatgaaccaagtgggtatgcacaaacagactgtgttctagaggctatggctttccttgaagaatcccacccaggaatatttgagaattcatgccttgaaacaatggaagttgttcaacaaacaagggtagataaactgactcaaggtcgccagacttatgattggacattaaacagaaatcaaccggcagcaactgcattggccaacaccatagaagtctttagatcgaatgacctaacagctaacgagtcaggaaggctaatagatttcttaaaggatgtaatggaatcaatgaacaaagaggaaatagagataacaacccactttcaaagaaaaaggagagtaagagacaacatgaccaagaagatggtcacgcaaagaacaatagggaagaaaaaacagagactgaataagagaggctatctaataagagcactgacattaaatacgatgaccaaagatgcagagagaggcaagttaaaaagaagggctatcgcaacacctgggatgcagattagaggtttcgtatactttgttgaaactttagctaggagcatttgcgaaaagcttgaacagtctggactcccagtagggggcaatgaaaagaaggccaaactggcaaatgttgtgagaaagatgatgactaattcacaagatacagagatttctttcacaatcactggggacaacactaagtggaatgaaaatcaaaatcctcgaatgttcctggcgatgattacatatatcaccagaaatcaacccgagtggtttagaaacatcctgagcatggcacccataatgttctcaaataaaatggcaagactagggaaagggtacatgttcgagagtaaaagaatgaagattcgaacacaaataccagcagaaatgctagcaagcattgatctgaagtacttcaatgaatcaacaaagaagaaaattgagaaaataaggcctcttttaatggatggcacagcatcactgagtcctgggatgatgatgggcatgttcaacatgctaagtacggtcttgggagtctcgatactgaatcttggacaaaagaaatacaccaagacaacatactggtgggatgggctccaatcatccgacgattttgctctcatagtgaatgcaccaaaccatgagggaatacaagcaggagtggacagattctacaggacctgcaagttagtgggaatcaacatgagcaaaaagaagtcctatataaataagacagggacatttgaattcacaagctttttttatcgctatggatttgtggctaattttagcatggagctacccagctttggagtgtctggagtaaatgaatcagctgacatgagtattggagtaacagtgataaagaacaacatgataaacaatgaccttggacctgcaacggcccagatggctcttcaattgttcatcaaagactacagatacacatataggtgccataggggagacacacaaattcagacgagaagatcatttgagttaaagaagctgtgggatcaaacccaatcaaaggtagggctattagtatcagatggaggaccaaacttatacaatatacggaatcttcacattcctgaagtctgcttaaaatgggagctaatggatgatgattatcggggaagactttgtaatcccctgaatccctttgtaagtcataaggagattgattctgtaaacaatgctgtggtaatgccagcccatggtccagccaaaagcatggaatatgatgccgttgcaactacacattcctggattcccaagaggaatcgttctattctcaacacaagccaaaggggaattcttgaggatgaacagatgtaccagaagtgttgcaatctattcgagaaatttttccctagcagttcatataggaggccggttggaatttctagcatggtggaggccatggtatctagggcccggattgatgccagggtcgactttgagtctggacggatcaagaaagaagagttctcagagatcatgaagatctgttccaccattgaagaactcagacggcaaaaataa

>H1N1_PB1_CY073779_A/Vienna/INS369/2010

atggatgtcaatccgactctacttttcctaaaaattccagcgcaaaatgccataagcaccacattcccttatactggagatcctccatacagccatggaacaggaacaggatacaccatggacacagtaaacagaacacaccaatactcagaaaagggaaagtggacgacaaacacagagactggtgcaccccagctcaacccgattgatggaccactacctgaggataatgaaccaagtgggtatgcacaaacagactgtgttctagaggctatggctttccttgaagaatcccacccaggaatatttgagaattcatgccttgaaacaatggaagttgttcaacaaacaagggtagataaactagctcaaggtcgccagacttatgattggacattaaacagaaatcaaccggcagcaactgcattggccaacaccatagaagtctttagatcaaatggcctaacagctaatgagtcaggaaggctaatagatttcttaaaggatgtaatggaatcaatgaacaaagaggaaatagagataacaacccactttcaaagaaaaaggagagtaagagacaacatgaccaagaagatggtcacgcaaagaacaatagggaagaaaaaacaaagactgaataagagaggctatctaataagagcactgacattaaatacgatgaccaaagatgcagagagaggcaagttaaaaagaagggctatcgcaacacctgggatgcagattagaggtttcgtatactttgttgaaactttagctaggagcatttgcgaaaagcttgaacagtctgggctcccagtagggggcaatgaaaagaaggccaaactggcaaatgttgtgagaaagatgatgactaattcacaagacacagagatttctttcacaatcactggggacaacactaagtggaatgaaaatcaaaatcctcgaatgttcctggcgatgattacatatatcaccagaaatcaacccgagtggttcagaaacatcctgagcatggcacccataatgttctcaaacaaaatggcaagactagggaaagggtacatgttcgagagtaaaagaatgaagattcgaacacaaataccagcagaaatgctagcaagcattgacctgaagtacttcaatgaatcaacaaagaagaaaattgagaaaataaggcctcttctaatagatggcacagcatcactgagtcctgggatgatgatgggcatgttcaacatgctaagtacggtcttgggagtctcgatactgaatcttggacaaaagaaatacaccaagacaatatactggtgggatgggctccaatcatccgacgattttgctctcatagtgaatgcaccaaaccatgagggaatacaagcaggagtggacagattctacaggacctgcaagttagtgggaatcaacatgagcaaaaagaagtcctatataaataagacagggacatttgaattcacaagctttttttatcgctatggatttgtggctaattttagcatggagctacccagctttggagtgtctggagtaaatgaatcagctgacatgagtattggagtaacagtgataaagaacaacatgataaacaatgaccttggacctgcaacggcccagatggctcttcaattgttcatcaaagactacagatacacatataggtgccataggggagacacacaaattcagacgagaagatcatttgagttaaagaagctgtgggatcaaacccaatcaaaggtagggctattagtatcagatggaggaccaaacttatacaatatacggaatcttcacattcctgaagtctgcttaaaatgggagctaatggatgatgattatcggggaagactttgtaatcccctgaatccctttgtcagtcataaagagattgattctgtaaacaatgctgtggtaatgccagcccatggtccagccaaaagcatggaatatgatgccgttgcaactacacattcctggattcccaagaggaatcgttctattctcaacacaagccaaaggggaattcttgaggatgaacagatgtaccagaagtgctgcaatctattcgagaaatttttccctagcagttcatataggagaccggttggaatttctagcatggtggaggccatggtgtctagggcccggattgatgccagggtcgacttcgagtctggacggatcaagaaagaagagttctctgagatcatgaagatctgttccaccattgaagaactcagacggcaaaaataa

>H1N1_PB1_KJ856112_A/Sri Lanka/20887/2010

atggatgtcaatccgactctacttttcctaaaaattccagcgcaaaatgccataagcaccacattcccttacactggagatcctccatacagccatggaacaggaacaggatacaccatggacacagtaaacagaacacaccaatactcagaaaagggaaagtggacgacaaacacagagactggtgcaccccagctcaacccgattgatggaccactacctgaggataatgaaccaagtgggtatgcacaaacagactgtgttctagaggctatggctttccttgaagaatcccacccaggaatatttgagaattcatgccttgaaacaatggaagctgttcaacaaacaagggtagataaactaactcaaggtcgccagacttatgattggacattaaacagaaatcaaccggcagcaactgcattggccaacaccatagaagtctttagatcgaatggcctaacagctaatgagtcaggaaggctaatagatttcttaaaggatgtaatggaatcaatgaacaaagaggaaatagagataacaacccactttcaaagaaaaaggagagtaagagacaacatgaccaagaagatggtcacgcaaagaacaatagggaagaaaaaacaaagactgaataagagaggctatctaataagagcactgacattaaatacgatgaccaaagatgcagagagaggcaagttaaaaagaagggctatcgcaacacctgggatgcagattagaggtttcgtatactttgttgaaactttagctaggagcatttgcgaaaagcttgaacagtctgggctcccagtagggggcaatgaaaagaaggccaaactggcaaatgttgtgagaaagatgatgactaattcacaagacacagagatttctttcacaatcactggggacaacactaagtggaatgaaaatcaaaatcctcgaatgttcctggcgatgattacatatatcaccagaaatcaacccgagtggttcagaaacatccttagcatggcacccataatgttctcaaacaaaatggcaagactagggaaagggtacatgttcgagagtaaaagaatgaagattcgaacacaaattccagcagaaatgctagcaagcattgacctgaagtacttcaatgaatcaacaaagaagaaaattgagaaaataaggcctcttctaatagatggcacagcatcactgagtcctgggatgatgatgggcatgttcaacatgctaagtacggtcttgggagtctcgatactgaatcttggacaaaagaaatacaccaagacaacatactggtgggatgggctccaatcatccgacgattttgctctcatagtgaatgcaccaaaccatgagggaatacaagcaggagtggacagattctacaggacctgcaagttagtgggaatcaacatgagcaaaaagaagtcatatataaataaaacagggacatttgaattcacaagctttttttatcgctatggatttgtggctaattttagcatggagctacccagctttggagtgtctggagtaaatgaatcagctgacatgagtattggagtaacagtgataaagaacaacatgataaacaatgaccttggacctgcaacggcccagatggctcttcaattgttcatcaaagactacagatacacatataggtgccataggggagacacacaaattcagacgagaagatcatttgagttaaaaaagctgtgggatcaaacccaatcaaaggtagggctattagtatcagatggaggaccaaacttatacaatatacggaatcttcacattcctgaagtctgcttaaaatgggagctaatggatgatgattatcggggaagactttgtaatcccctgaatccctttgtcagtcataaagagattgattctgtaaacaatgctgtggtaatgccagcccatggtccagccaaaagcatggaatatgatgccgttgcaactacacattcctggattcccaagaggaatcgttctattctcaacacaagccaaaggggaattcttgaggatgaacagatgtaccagaagtgctgcaatctattcgagaaatttttccctagcagttcatataggagaccggttggaatttctagcatggtggaggccatggtgtctagggcccggattgatgccagggtcgacttcgagtctggacggatcaagaaagaagagttctctgagatcatgaagatctgttccaccattgaagaactcagacggcaaaaataa

>H1N1_PB1_KJ943176_A/Santiago/p21d2/2011

atggatgtcaatccgactctacttttcctaaaaattccagcgcaaaatgccataagcaccacattcccttatactggagatcctccatacagccatggaacaggaacaggatacaccatggacacagtaaacagaacacaccaatactcagaaaagggaaggtggacgacaaacacagagactggtgcaccccagctcaacccgattgatggaccactacctgaggataatgaaccaagtggatatgcacaaacagactgtgttctagaggctatggctttccttgaagaatcccacccaggaatatttgagaattcatgccttgaaacaatggaagctgttcaacaaacaagggtagataaactaactcaaggtcgccagacttatgattggacattgaacagaaatcaaccggcagcaactgcattggccaacaccatagaagtctttagatcgaatggcctaacagctaatgagtcaggaaggctaatagatttcttaaaggatgtgatggaatcaatgaacaaagaggaaatagagataacaacccactttcaaagaaaaaggagagtaagagacaacatgaccaagaagatggtcacgcaaagaacaataggaaagaaaaagcaaagactgaataagagaggctatctaataagagcactgacattaaatacgatgaccaaagatgcagagagaggcaagttaaaaagaagggctatcgcaacacctgggatgcagattagaggtttcgtatactttgttgaaactttagctaggagcatttgcgaaaagcttgaacagtctgggctcccagtagggggcaatgaaaagaaggccaaactggcaaatgttgtgagaaagatgatgactaattcacaagacacagagatttctttcacaatcactggggacaacactaagtggaatgaaaatcaaaatcctcgaatgttcctggcgatgattacatatataaccagaaatcaacccgagtggttcagaaacatcctgagcatggcacccataatgttctcaaacaaaatggcaagactagggaaagggtacatgttcgagagtaaaagaatgaagattcgaacacaaatcccagcagaaatgctagcaagcattgacctgaagtacttcaatgaatcaacaaagaggaaaattgagaaaataaggcctcttctaatagatggcacagcatcactgagtcctgggatgatgatgggcatgttcaacatgctaagtacggtcttgggagtctcgatactgaatcttggacaaaagaaatacaccaagacaacatactggtgggatgggctccaatcatccgacgattttgctctcatagtgaatgcaccaaaccatgagggaatacaagcaggagtggacagattctacaggacctgcaagttagtgggaatcaacatgagcaaaaagaagtcatatataaataaaacagggacatttgaattcacaagctttttttatcgctatggatttgtggctaattttagcatggagctacccagctttggagtgtctggagtaaatgaatcagctgacatgagtattggagtaacagtgataaagaacaacatgataaacaatgaccttggacctgcaacggcccagatggctcttcaattgttcatcaaagactacagatacacatataggtgccataggggagacacacaaattcagacgagaagatcatttgagttaaaaaagctgtgggatcaaacccaatcaaaggtagggctattagtatcagatggaggaccaaacttatacaatatacggaatcttcacattcctgaagtctgcttaaaatgggagctaatggatgatgattatcggggaagactttgcaatcccctgaatccctttgtcagtcataaagagattgattctgtaaacaatgctgtggtaatgccagcccatggtccagccaaaagcatggaatatgatgccgttgcaactacacattcctggattcccaagaggaatcgttctattctcaacacaagccaaaggggaattcttgaggatgaacagatgtaccagaagtgctgcaatctattcgagaaatttttccctagcagttcatataggagaccggttggaatttctagcatggtggaggccatggtgtctagggcccggattgatgccagggtcgacttcgagtctggacggatcaagaaagaagagttctctgagatcatgaagatctgttccaccattgaagaactcagacggcaaaaataa

>H1N1_PB1_KM219157_A/Helsinki/220N/2014

atggatgtcaatccgactctacttttcctaaaaattccagcacaaaatgccataagcaccacattcccttatactggagatcctccatacagccatggaacaggaacaggatacaccatggacacagtaaacagaacacaccaatactcagaaaagggaaagtggacaacaaacacagagactggtgcaccccagctcaacccgattgatggaccactacctgaggataatgaaccaagtgggtatgcacaaacagactgtgttctagaggctatggctttccttgaagaatcccacccaggaatatttgagaattcatgccttgaaacaatggaagttgttcaacaaacaagggtagataaactgactcaaggtcgccagacttatgattggacattaaacagaaatcaaccggcagcaactgcattggccaacaccatagaagtctttagatcgaatgacctaacagctaacgagtcaggaaggctaatagatttcttaaaggatgtaatggaatcaatgaacaaagaggaaatagagataacaacccactttcaaagaaaaaggagagtaagagacaacatgaccaagaagatggtcacgcaaagaacaataggtaagaaaaaacagagactgaataagagaggctatctaataagagcactgacattaaatacgatgaccaaagatgcagagagaggcaagttaaaaagaagggctatcgcaacacctgggatgcagattagaggtttcgtatactttgttgaaactttagctaggagcatttgcgaaaagcttgaacagtctgggctcccagtagggggcaatgaaaagaaggccaaattggcaaatgttgtgagaaagatgatgactaattcacaagatacagagatttctttcacaatcactggggacaacactaagtggaatgaaaatcaaaatcctcgaatgttcctggcgatgattacatatatcaccagaaatcaacccgagtggtttagaaacatcctgagcatggcacccataatgttctcaaataaaatggcaagactagggaaagggtacatgttcgagagtaaaagaatgaagattcgaacacaaataccagcagaaatgctagcaaacattgatctgaagtacttcaatgaatcaacaaagaagaaaattgagaaaataaggcctcttttaatggatggcacagcatcactgagtcctgggatgatgatgggcatgttcaacatgctaagtacggtcttgggagtctcgatactgaatcttggacaaaagaaatacaccaagacaacatactggtgggatgggctccaatcatccgacgattttgctctcatagtgaatgcaccaaaccatgaaggaatacaagcaggagtggacagattctacaggacctgcaagttagtgggaatcaacatgagcaaaaagaagtcctatataaataagacagggacatttgaattcacaagctttttttatcgctatggatttgtggctaattttagcatggagctacccagctttggagtgtctggagtaaatgaatcagctgacatgagtattggagtaacagtgataaagaacaacatgataaacaatgaccttggacctgcaacggcccagatggctcttcaattgttcatcaaagactacagatacacatataggtgccataggggagacacacaaattcagacgagaagatcatttgagttaaagaagctatgggatcaaacccaatcaaaggtagggctattagtatcagatggaggaccaaacttatacaacatacggaatcttcacattcctgaagtctgcttaaaatgggagctaatggatgatgattatcggggaagactttgtaatcccctgaatccctttgtgagtcataaggagattgattctgtaaacaatgctgtggtaatcccagcccatggtccagccaaaagcatggaatatgatgccgttgcaactacacattcctggattcccaagaggaatcgttctattctcaacacaagccaaagggggattcttgaggatgaacagatgtaccagaagtgttgcaatctattcgagaaatttttccctagcagttcatataggagaccggttggaatttctagcatggtggaggccatggtgtctagggcccggattgatgccagggtcgactttgagtctggacggatcaagaaagaagagttctctgagatcatgaagatctgttccaccattgaagaactcagacggcaaaaataa

>H1N1_PB1_KM409067_A/Rhode Island/09/2013

atggatgtcaatccgactctacttttcctaaagattccagcacaaaatgccataagcaccacattcccttatactggagatcctccatacagccatggaacaggaacaggatacaccatggacacagtaaacagaacacaccaatactcagaaaaggggaagtggacaacaaacacagagactggtgcaccccagctcaacccgattgatggaccactacctgaggataatgaaccaagtgggtatgcacaaacagactgtgttctagaggctatggctttccttgaagaatcccacccaggaatatttgagaattcatgccttgaaacaatggaaattgttcaacaaacaagggtagataaactgactcaaggtcgccagacttatgattggacattaaacagaaatcaaccggcagcaactgcattggccaacaccatagaagtctttagatcgaatgacctaacagctaacgagtcaggaaggctaatagatttcttaaaggatgtaatggaatcaatgaacaaagaggaaatagagataacaacccactttcaaagaaaaaggagagtaagagacaacatgaccaagaagatggtcactcaaagaacaatagggaagaaaaaacagagactgaataagagaggctatctaataagagcactgacattaaatacgatgaccaaagatgcagagagaggcaagttaaaaagaagggctatcgcaacacctgggatgcaaattagaggtttcgtatactttgttgaaactttagctaggagcatttgcgaaaagcttgaacagtctgggctcccagtagggggcaatgaaaagaaggccaaattggcaaatgttgtgagaaagatgatgactaattcacaagatacagagatttctttcacaatcactggggacaacactaagtggaatgaaaatcaaaatcctcgaatgttcctggcgatgattacatatatcaccagaaatcaacccgagtggtttagaaacatcctgagcatggcacccataatgttctcaaataaaatggcaagactagggaaagggtacatgttcgagagtaaaagaatgaagattcgaacacaaataccagcagaaatgctagcaagcattgatctgaagtacttcaatgaatcaacaaagaagaaaattgagaaaataaggcctcttttaatggatggcacagcatcactgagtcctgggatgatgatgggcatgttcaacatgctaagtacggtcttgggagtctcgatactgaatcttggacaaaagaaatacaccaagacaacatactggtgggatgggctccaatcatccgacgattttgctctcatagtgaatgcaccaaaccatgaaggaatacaagcaggagtggacagattctacaggacctgcaagttagtgggaatcaacatgagcaaaaagaagtcctatataaataagacagggacatttgaattcacaagctttttttatcgctatggatttgtggctaattttagcatggagctacccagctttggagtgtctggggtaaatgaatcagctgacatgagtattggagtaacagtgataaagaacaacatgataaacaatgacctcggacctgcaacggcccagatggctcttcaattgttcatcaaagactacagatacacatataggtgccataggggagacacacaaattcagacgagaagatcatttgagttaaagaagctatgggatcaaacccaatcaaaggtagggctattagtatcagatggaggaccaaacttatacaacatacggaatcttcacattcctgaagtctgcttaaaatgggagctaatggatgatgattatcggggaagactttgtaatcccctgaatccctttgtgagtcataaggagattgattctgtaaacaatgctgtggtaatgccagcccatggtccagccaaaagcatggaatatgatgccgttgcaactacacattcctggattcccaagaggaatcgttctattctcaacacaagtcaaaggggaattcttgaggatgaacagatgtaccagaagtgttgcaatctattcgagaaatttttccctagcagttcatataggagaccggttggaatttctagcatggtggaggccatggtgtctagggccaggattgatgccagggtcgactttgagtctggacggatcaagaaagaagagttctctgagatcatgaagatctgttccaccattgaagaactcagacggcaaaaataa

>H1N1_PB1_CY188815_A/New York/WC-LVD-14-060/2014

atggatgtcaatccgactctacttttcctaaaaattccagcacaaaatgccataagcaccacattcccttatactggagatcctccatacagccatggaacaggaacaggatacaccatggacacagtaaacagaacacaccaatactcagaaaaggggaaatggacgacaaacacagagactggtgcaccccagctcaacccgattgatggaccactacctgaggataatgaaccaagtgggtatgcacaaacagactgtgtcctagaggctatggctttccttgaagaatcccacccaggaatatttgagaattcatgccttgaaacaatggaagttgttcaacaaacaagggtagataaactgactcaaggtcgccagacttatgattggacattaaacagaaatcaaccggcagcaactgcattggccaacaccatagaagtctttagatcgaatgacctaacagctaacgagtcaggaaggctaatagatttcttaaaggatgtaatggaatcaatgaacaaagaggaaatagagataacaacccactttcaaagaaaaaggagagtaagagacaacatgaccaagaagatggtcacgcaaagaacaatagggaagaaaaaacagagactgaataagagaggctatctaataagagcactgacattaaatacgatgaccaaagatgcagagagaggcaagttaaaaagaagggctatcgcaacacctgggatgcagattagaggtttcgtatactttgttgaaactttagctaggagcatttgcgaaaagcttgaacagtctgggctcccagtagggggcaatgaaaagaaggccaaattggcaaatgttgtgagaaagatgatgactaattcacaagatacagagatttctttcacaatcactggggacaacactaaatggaatgaaaatcaaaatcctcgaatgttcctggcgatgattacatatatcaccagaaatcaacccgagtggtttagaaacatcctgagcatggcacccataatgttctcaaataaaatggcaagactggggaaagggtacatgttcgagagtaaaagaatgaagattcgaacacaaataccagcagaaatgctagcaagcattgatctgaagtacttcaatgaatcaacaaagaagaaaattgagaaaataagacctcttttaatggatggcacagcatcactgagtcctgggatgatgatgggcatgttcaacatgctaagtacggtcttgggagtctcgatactgaatcttggacaaaagaaatacaccaagacaacatactggtgggatgggctccaatcatccgacgattttgctctcatagtgaatgcaccaaaccacgaaggaatacaagcaggagtggacagattctacaggacctgcaagttagtgggaatcaacatgagcaaaaagaagtcctatataaataagacagggacatttgaattcacaagctttttttatcgctatggatttgtggctaattttagcatggagctacccagctttggagtgtctggagtaaatgaatcagctgacatgagtattggagtaacagtgataaagaacaacatgataaacaatgaccttggacctgcaacggcccagatggctcttcaattgttcatcaaagactacagatacacatataggtgccataggggagacacacaaattcaaacgagaagatcatttgagttaaagaagctatgggatcaaacccaatcaaaggtagggctattagtatcagatggaggaccaaacttatacaacatacggaatcttcacattcctgaagtctgcttaaaatgggagctaatggatgatgattatcggggaagactttgtaatcccctgaatccctttgtgagtcataaggagattgattctgtaaacaatgccgtgataatgccagcccatggtccagccaaaagcatggaatatgatgccgttgcaactacacattcctggattcccaagaggaatcgttctattctcaacacaagccaaaggggaattcttgaggatgaacagatgtaccagaagtgttgcaatctattcgagaaatttttccctagcagttcatataggagaccggttggaatttctagcatggtggaggccatggtgtctagggcccggattgatgccagggtcgactttgagtctggacgggtcaagaaagaagagttctctgagatcatgaagatctgttccaccattgaagaactcagacggcaaaaataa

>H1N1_PB1_CY188895_A/New York/WC-LVD-13-010/2013

atggatgtcaatccgactctacttttcctaaaaattccagcacaaaatgccataagcaccacattcccttatactggagatcctccatacagccatggaacaggaacaggatacaccatggacacagtaaacagaacacaccaatactcagaaaaggggaagtggacaacaaacacagagactggtgcaccccagctcaacccgattgatggaccactacctgaggataatgaaccaagtgggtatgcacaaacagactgtgttctagaggctatggctttccttgaagaatcccacccaggaatatttgagaattcatgccttgaaacaatggaaattgttcaacaaacaagggtagataaactgactcaaggtcgccagacttatgattggacattaaacagaaatcaaccggcagcaactgcattggccaacaccatagaagtctttagatcgaatgacctaacagctaacgagtcaggaaggctaatagatttcttaaaggatgtaatggaatcaatgaacaaagaggaaatagagataacaacccactttcaaagaaaaaggagagtaagagacaacatgaccaagaagatggtcactcaaagaacaatagggaagaaaaaacagagactgaataagagaggctatctaataagagcactgacattaaatacgatgaccaaagatgcagagagaggcaagttaaaaagaagggctatcgcaacacctgggatgcagattagaggtttcgtatactttgttgaaactttagctaggagcatctgcgaaaagcttgaacagtctgggctcccagtagggggcaatgaaaagaaagccaaattggcaaatgttgtgagaaagatgatgactaattcacaagatacagagatttctttcacaatcactggggacaacactaagtggaatgaaaatcaaaatcctcgaatgttcctggcgatgattacatatatcaccagaaatcaacccgagtggtttagaaacatcctgagcatggcacccataatgttctcaaataaaatggcaagactagggaaagggtacatgttcgagagtaaaagaatgaagattcgaacacaaataccagcagaaatgctagcaagcattgatctgaagtacttcaatgaatcaacaaagaagaaaattgagaaaataaggcctcttttaatggatggcacagcatcactgagtcctgggatgatgatgggcatgttcaacatgctaagtacggtcttgggagtctcgatactgaatcttggacaaaagaaatacaccaagacaacatactggtgggatgggctccaatcatccgacgattttgctctcatagtgaatgcaccaaaccatgaaggaatacaagcaggagtggacagattctacaggacctgcaagttagtgggaatcaacatgagcaaaaagaagtcctatataaataagacagggacatttgaattcacaagctttttttatcgctatggatttgtggctaattttagcatggagctacccagctttggagtatctggagtaaatgaatcagctgacatgagtattggagtaacagtgataaagaacaacatgataaacaatgacctcggacctgcaacggcccagatggctcttcaattgttcatcaaagactacagatacacatataggtgccataggggagacacacaaattcagacgagaagatcatttgagttaaagaagctatgggatcaaacccaatcaaaggtagggctattagtatcagatggaggaccaaacttatacaacatacggaatcttcacattcctgaagtctgcttaaaatgggagctaatggatgatgattatcggggaagactttgtaatcccctgaatccctttgtgagtcataaggagattgattctgtaaacaatgctgtggtaatgccagcccatggtccagccaaaagcatggaatatgatgccgttgcaactacacattcctggattcccaagaggaatcgttctattctcaacacaagccaaaggggaattcttgaggatgaacagatgtaccagaagtgttgcaatctattcgagaaatttttccctagcagttcatataggagaccggttggaatttctagcatggtggaggccatggtgtctagggcccggattgatgccagggtcgactttgagtctggacggatcaagaaagaagagttctctgagatcatgaagatctgttccaccattgaagaactcagacggcaaaaataa

>H1N1_PB1_CY189463_A/New York/WC-LVD-14-053/2014

atggatgtcaatccgactctacttttcctaaaaattccagcacaaaatgccataagcaccacattcccttatactggagatcctccatacagccatggaacaggaacaggatacaccatggacacagtaaacagaacacaccaatactcagaaaaggggaagtggacaacaaacacagagactggtgcaccccagctcaacccgattgatggaccactacctgaggataatgaaccaagtgggtatgcacaaacagactgtgtcctagaggctatggctttccttgaagaatcccacccaggaatatttgagaattcatgccttgaaacaatggaagttgttcaacaaacaagggtagataaactgactcaaggtcgccagacttatgattggacattaaacagaaatcaaccggcagcaactgcattggccaacaccatagaagtctttagatcgaatgacctaacagctaacgagtcaggaaggctaatagatttcttaaaggatgtaatggaatcaatgaacaaagaggaaatagagataacaacccactttcaaagaaaaaggagagtaagagacaacatgaccaagaagatggtcacgcaaagaacaatagggaagaaaaaacagagactgaataagagaggctacctaataagagcactgacattaaatacgatgaccaaagatgcagagagaggcaagttaaaaagaagggctatcgcaacacctgggatgcagattagaggtttcgtatactttgttgaaactttagctaggagcatttgcgaaaagcttgaacagtctgggctcccagtagggggcaatgaaaagaaggccaaattggcaaatgttgtgagaaagatgatgactaattcacaagatacagagatttctttcacaatcactggggacaacactaaatggaatgaaaatcaaaatcctcgaatgttcctggcgatgattacatatatcaccagaaatcaacccgagtggtttagaaacatcctgagcatggcacccataatgttctcaaataaaatggcaagactggggaaagggtacatgttcgagagtaaaagaatgaagattcgaacacaaataccagcagaaatgctagcaagcattgatctgaagtacttcaatgaatcaacaaagaagaaaattgagaaaataagacctcttttaatggatggcacagcatcactgagtcctgggatgatgatgggcatgttcaacatgctaagtacagtcttgggagtctcgatactgaatcttggacaaaagaaatacaccaagacaacatactggtgggatgggctccaatcatccgacgattttgctctcatagtgaatgcaccaaaccacgaaggaatacaagcaggagtggacagattctacaggacctgcaagttagtgggaatcaacatgagcaaaaagaagtcctatataaataagacagggacatttgaattcacaagctttttttatcgctatggatttgtggctaattttagcatggagctacccagctttggagtgtctggagtaaatgaatcagctgacatgagtattggagtaacagtgataaagaacaacatgatgaacaatgaccttggacctgcaacggcccagatggctcttcaattgttcatcaaagactacagatacacatataggtgccataggggagacacacaaattcagacgagaagatcatttgagttaaagaagctatgggatcaaacccaatcaaaggtagggctattagtatcagatggaggaccaaacttatacaacatacggaatcttcacattcctgaagtctgcttaaaatgggagctaatggatgatgattatcggggaagactttgtaatcccctgaatccctttgtgagtcataaggagattgattctgtaaacaatgccgtggtaatgccagcccatggtccagccaaaagcatggaatatgatgccgttgcaactacacattcctggattcccaagaggaatcgttctattctcaacacaagccaaaggggaattcttgaggatgaacagatgtaccagaagtgttgcaatctattcgagaaatttttccctagcagttcatataggagaccggttggaatttctagcatggtggaggccatggtgtctagggcccggattgatgccagggtcgactttgagtctggacggatcaagaaagaagagttctctgagatcatgaagatctgttccaccattgaagaactcagacggcaaaaataa

>H1N1_PB1_CY189495_A/New York/WC-LVD-14-057/2014

atggatgtcaatccgactctacttttcctaaaaattccagcacaaaatgccataagcaccacattcccttatactggagatcctccatacagccatggaacaggaacaggatacaccatggacacagtaaacagaacacaccaatactcagaaaaggggaagtggacaacaaacacagagactggtgcaccccagctcaacccgattgatggaccactacctgaggataatgaaccaagtgggtatgcacaaacagactgtgtcctagaggctatggctttccttgaagaatcccacccaggaatatttgagaattcatgccttgaaacaatggaagttgttcaacaaacaagggtagataaactgactcaaggtcgccagacttatgattggacattgaacagaaatcaaccggcagcaactgcattggccaacaccatagaagtctttagatcgaatgacctaacagctaacgagtcaggaaggctaatagatttcttaaaggatgtaatggaatcaatgaacaaagaggaaatagagataacaacccactttcaaagaaaaaggagagtaagagacaacatgaccaagaagatggtcacgcaaagaacaatagggaagaaaaaacagagactgaataagagaggctatctaataagagcactgacattaaatacgatgaccaaagatgcagagagaggcaagttaaaaagaagggctatcgcaacacctgggatgcagattagaggtttcgtatactttgttgaaactttagctaggagcatttgcgaaaagcttgaacagtctgggctcccagtagggggcaatgaaaagaaggccaaattggcaaatgttgtgagaaagatgatgactaattcacaagatacagagatttctttcacaatcactggggacaacactaaatggaatgaaaatcaaaatcctcgaatgttcttggcgatgattacatatatcaccagaaatcaacccgagtggtttagaaacatcctgagcatggcacccataatgttctcaaataaaatggcaagactggggaaagggtacatgttcgagagtaaaagaatgaagattcgaacacaaataccagcagaaatgctagcaagcattgatctgaagtacttcaatgaatcaacaaagaagaaaattgagaaaataagacctcttttaatggatggcacagcatcactgagtcctgggatgatgatgggcatgttcaacatgctaagtacggtcttgggagtctcgatactgaatcttggacaaaagaaatacaccaagacaacatactggtgggatgggctccaatcatccgacgattttgctctcatagtgaatgcaccaaaccacgaaggaatacaagcaggagtggacagattctacaggacctgcaagttagtgggaatcaacatgagcaaaaagaagtcctatataaataagacagggacatttgaattcacaagctttttttatcgctatggatttgtggctaattttagcatggagctacccagctttggagtgtctggagtaaatgaatcagctgacatgagtattggagtaacagtgataaagaacaacatgataaacaatgaccttggacctgcaacggcccagatggctcttcaattgttcatcaaagactacagatacacatataggtgccataggggagacacacaaattcagacgagaagatcatttgagttaaagaagctatgggatcaaacccaatcaaaggtagggctattagtatcagatggaggaccaaacttatacaacatacggaatcttcacattcctgaagtctgcttaaaatgggagctaatggatgatgattatcggggaagactttgtaatcccctgaatccctttgtgagtcataaggagattgattctgtaaacaatgccgtggtaatgccagcccatggtccagccaaaagcatggaatatgatgccgttgcaactacacattcctggattcccaagaggaatcgttctattctcaacacaagccaaaggggaattcttgaggatgaacagatgtaccagaagtgttgcaatctattcgagaaatttttccctagcagttcatataggagaccggttggaatttctagcatggtggaggccatggtgtctagggcccggattgatgccagggtcgactttgagtctgggcggatcaagaaagaagagttctctgagatcatgaagatctgttccaccattgaagaactcagacggcaaaaataa

>H1N1_PB1_KM437728_A/Finland/31/2012

atggatgtcaatccgactctacttttcctaaaaattccagcacaaaatgccataagcaccacattcccttatactggagatcctccatacagccatggaacaggaacaggatacaccatggacacagtaaacagaacacaccaatactcagaaaagggaaagtggacaacaaacacagagactggtgcaccccagctcaacccgattgatggaccactacctgaggataatgaaccaagtgggtatgcacaaacagactgtgttctagaggctatggctttccttgaagaatcccacccaggaatatttgagaattcatgccttgaaacaatggaagttgttcaacaaacaagggtagataaactgactcaaggtcgccagacttatgattggacattaaacagaaatcaaccggcagcaactgcattggccaacaccatagaagtctttagatcgaatgacctaacagctaacgagtcaggaaggctaatagatttcttaaaggatgtaatggaatcaatgaacaaagaggaaatagagataacaacccattttcaaagaaaaagaagagtaagagacaacatgaccaagaagatggtcacgcaaagaacaataggtaagaaaaaacagagactgaataagagaggctatctaataagagcactgacattaaatacgatgaccaaagatgcagagagaggcaagttaaaaagaagggctatcgcaacacctgggatgcagattagaggtttcgtatactttgttgaaactttagctaggagcatttgcgaaaagcttgaacagtctgggctcccagtagggggcaatgaaaagaaggccaaattggcaaatgttgtgagaaagatgatgactaattcacaagatacagagatttctttcacaatcactggggacaacactaagtggaatgaaaatcaaaatcctcgaatgttcctggcgatgattacatatatcaccagaaatcaacccgagtggtttagaaacatcctgagcatggcacccataatgttctcaaataaaatggcaagactagggaaagggtacatgttcgagagtaaaagaatgaagattcgaacacaaataccagcagaaatgctagcaaacattgatctgaagtacttcaatgaatcaacaaagaagaaaattgagaaaataaggcctcttttaatggatggcacagcatcactgagtcctgggatgatgatgggcatgttcaacatgctaagtacggtcttgggagtctcgatactgaatcttggacaaaagaaatacaccaagacaacatactggtgggatgggctccaatcatccgacgattttgctctcatagtgaatgcaccaaaccatgaaggaatacaagcaggagtggacagattctacaggacctgcaagttagtgggaatcaacatgagcaaaaagaaatcctatataaataagacagggacatttgaattcacaagctttttttatcgctatggatttgtggctaattttagcatggagctacccagctttggagtgtctggagtaaatgaatcagctgacatgagtattggagtaacagtgataaagaacaacatgataaacaatgaccttggacctgcaacggcccagatggctcttcaattgttcatcaaagactacagatacacatataggtgccataggggagacacacaaattcagacaagaagatcatttgagttaaagaagctatgggatcaaacccaatcaaaggtagggctattagtatcagatggaggaccaaacttatacaacatacggaatcttcacattcctgaagtctgcttaaaatgggagctaatggatgatgattatcggggaaggctttgtaatcccctgaatccctttgtgagtcataaggagattgattctgtaaacaatgctgtggtaatcccagcccatggtccagccaaaagcatggaatatgatgccgttgcaactacacattcctggattcccaagaggaatcgttctattctcaacacaagccaaaggggaattcttgaggatgaacagatgtaccagaagtgttgcaatctattcgagaaatttttccctagcagttcatataggagaccggttggaatttctagcatggtggaggccatggtgtctagggcccggattgatgccagggtcgactttgagtctggacggatcaagaaagaagagttctctgagatcatgaagatctgttccaccattgaagaactcagacggcaaaaataa

>H1N1_PB1_KM437648_A/Finland/109/2014

atggatgtcaatccgactctacttttcctaaaaattccagcacaaaatgccataagcaccacattcccttatactggagatcctccatacagccatggaacaggaacaggatacaccatggacacagtaaacagaacacaccaatactcagaaaagggaaagtggacaacaaacacagagactggtgcaccccagctcaacccgattgatggaccactacctgaggataatgaaccaagtgggtatgcacaaacagactgtgttctagaggctatggctttccttgaagaatcccacccaggaatatttgagaattcatgccttgaaacaatggaagttgttcaacaaacaagggtagataaactgactcaaggtcgccagacttatgattggacattaaacagaaatcaaccggcagcaactgcattggccaacaccatagaagtctttagatcgaatgacctaacagctaacgagtcaggaaggctaatagatttcttaaaggatgtaatggaatcaatgaacaaagaggaaatagagataacaacccactttcaaagaaaaagaagagtaagagacaacatgaccaagaagatggtcacgcaaagaacaatagggaagaaaaaacagagactgaataagagaggctatctaataagagcactgacattaaatacgatgaccaaagatgcagagagaggcaagttaaaaagaagggctatcgcaacacctgggatgcagattagaggtttcgtatactttgttgaaactttagctaggagcatttgcgaaaagcttgaacagtctgggctcccagtagggggcaatgaaaagaaggccaaattggcaaatgttgtgagaaagatgatgactaattcacaagatacagagatttctttcacaatcactggggacaacactaagtggaatgaaaatcaaaatcctcgaatgttcctggcgatgattacatatatcaccagaaatcaacccgagtggtttagaaacatcctgagcatggcacccataatgttctcaaataaaatggcaagactagggaaagggtacatgttcgagagtaaaagaatgaagattcgaacacaaataccagcagaaatgctagcaaacattgatctgaagtacttcaatgaatcaacaaagaagaaaattgagaaaataaggcctcttttaatggatggcacagcatcactgagtcctgggatgatgatgggcatgttcaacatgctaagtacggtcttgggagtctcgatactgaatcttggacaaaagaaatacaccaagacaacatactggtgggatgggctccaatcatccgacgattttgctctcatagtgaatgcaccaaaccatgaaggaatacaagcaggagtggacagattctacaggacctgcaagttagtgggaatcaacatgagcaaaaagaaatcctatataaataagacagggacatttgaattcacaagctttttttatcgctatggatttgtggctaattttagcatggagctacccagctttggagtgtctggagtaaatgaatcagctgacatgagtattggagtaacagtgataaagaacaacatgataaacaatgaccttggacctgcaacggcccagatggctcttcaattgttcatcaaagactacagatacacatataggtgccataggggagacacacaaattcagacaagaagatcatttgagttaaagaagctatgggatcaaacccaatcaaaggtagggctattagtatcagatggaggaccaaacttatacaacatacggaatcttcacattcctgaagtctgcttaaaatgggagctaatggatgatgattatcggggaagactttgtaatcccctgaatccctttgtgagtcataaggagattgattctgtaaacaatgctgtggtaatcccagcccatggtccagccaaaagcatggaatatgatgccgttgcaactacacattcctggattcccaagaggaatcgttctattctcaacacaagccaaaggggaattcttgaggatgaacagatgtaccagaagtgttgcaatctattcgagaaatttttccctagcagttcatataggagaccggttggaatttctagcatggtggaggccatggtgtctagggcccggattgatgccagggtcgactttgagtctggacggatcaagaaagaagagttctctgagatcatgaagatctgttccaccattgaagaactcagacggcaaaaataa

>H1N1_PB1_KM654740_A/Connecticut/Flu182/2011

atggatgtcaatccgactctacttttcctaaaaattccagcgcaaaatgccataagcaccacattcccttatactggagatcctccatacagccatggaacaggaacaggatacaccatggacacagtaaacagaacacaccaatactcagaaaagggaaagtggacgacaaacacagagactggtgcaccccagctcaacccgattgatggaccactacctgaggataatgaaccaagtgggtatgcacaaacagactgtgttctagaggctatggctttccttgaagaatcccacccaggaatatttgagaattcatgccttgaaacaatggaagttgttcaacaaacaagggtagataaactaactcaaggtcgccagacttatgattggacattaaacagaaatcaaccggcagcaactgcattggccaacaccatagaagtctttagatcaaatgacctaacagctaatgagtcaggaaggctaatagatttcttaaaggatgtaatggaatcaatgaacaaagaggaaatagagataacaacccactttcaaagaaaaaggagagtaagagacaacatgaccaagaagatggtcacgcaaagaacaatagggaagaaaaaacaaagactgaataagagaggctatctaataagagcactgacattaaatacgatgaccaaagatgcagagagaggcaaattaaaaagaagggctatcgcaacacctgggatgcagattagaggtttcgtatactttgttgaaactttagctaggagcatttgcgaaaagcttgaacagtctgggctcccagtagggggcaatgaaaagaaggccaaactggcaaatgttgtgagaaagatgatgactaattcacaagacacagagatttctttcacaatcactggggacaacactaagtggaatgaaaatcaaaatcctcgaatgttcctggcgatgattacatacatcaccagaaatcaacccgagtggttcagaaacatcctgagcatggcacccataatgttctcaaacaaaatggcaagactagggaaagggtacatgttcgagagtaaaagaatgaagattcgaacacaaataccagcagaaatgctagcaagcattgacctgaagtacttcaatgaatcaacaaagaagaaaattgagaaaataaggcctcttctaatagatggcacagcatcactgagtcctgggatgatgatgggcatgttcaacatgctaagtacggtcttgggagtctcgatactgaatcttggacaaaagaaatacaccaagacaatatactggtgggatgggctccaatcatccgatgattttgctctcatagtgaatgcaccaaaccatgagggaatacaagcaggagtggacagattctacaggacctgcaagttagtgggaatcaacatgagcaaaaagaagtcctatataaataagacagggacatttgaattcacaagctttttttatcgctatggatttgtggctaattttagcatggagctacccagctttggagtgtctggagtaaatgaatcagctgacatgagtattggagtaacagtgataaagaacaacatgataaacaatgaccttggacctgcaacggcccagatggcacttcaattgttcatcaaagactacagatacacatataggtgccataggggagacacacaaatccagacgagaagatcatttgagttaaagaagctgtgggatcaaacccaatcaaagggagggctattagtatcagatggaggaccaaacttatacaatatacggaatcttcacattcctgaagtctgcttaaaatgggagctaatggatgatgattatcggggaagactttgtaatcccctgaatccctttgtcagtcataaagagattgattctgtaaacaatgctgtggtaatgccagcccatggtccagtcaaaagcatggaatatgatgccgttgcaactacacattcctggattcccaagaggaatcgttctattctcaacacaagccaaaggggaattcttgaggatgaacagatgtaccagaagtgctgcaatctattcgagaaatttttccctagcagttcatataggagaccggttggaatttctagcatggtggaggccatggtgtctagggcccggattgatgccaggatcgacttcgaatctggacggatcaagaaagaagagttctctgagatcatgaagatctgctccaccattgaagaactcagacggcaaaaataa

>H1N1_PB1_CY189685_A/Nicaragua/30238_01_TR2/2013

atggatgtcaatccgactctacttttcctaaaaattccagcacaaaatgccataagcaccacattcccttatactggagatcctccatacagccatggaacaggaacaggatacaccatggacacagtaaacagaacacaccaatactcagaaaagggaaagtggacaacaaacacagagactggtgcaccccagctcaacccgattgatggaccactacctgaggataatgaaccaagtgggtatgcacaaacagactgtgttctagaggctatggctttccttgaagaaacccacccaggaatatttgagaattcatgccttgaaacaatggaagttgttcaacaaacaagggtagataaactgactcaaggtcgccagacttatgattggacattaaacagaaatcaaccggcagcaactgcattggccaacacaatagaagtctttagatcgaatgacctaacagctaacgagtcaggaaggctaatagatttcttaaaggatgtaatggaatcaatgaacaaagaggaaatagagataataacccactttcaaagaaaaaggagagtaagagacaacatgaccaagaagatggtcacgcaaaggacaatagggaagaaaaaacagagactgaataagagaggctatctaataagagcactgacattaaatacgatgaccaaagatgcagagagaggcaagttaaaaagaagggctattgcaacacctgggatgcagattagaggtttcgtatactttgttgaaactttagctaggagcatttgcgaaaagcttgaacagtctggactcccagtagggggcaatgaaaagaaggccaaactggcaaatgttgtgagaaagatgatgactaattcacaagatacagagatttctttcacaattactggggacaacactaagtggaatgaaaatcaaaatcctcgaatgttcctggcgatgattacatatatcaccagaaatcaacccgagtggtttagaaacatcctgagcatggcacccataatgttctcaaataaaatggcaagactagggaaagggtacatgttcgagagtaaaagaatgaagattcgaacacaaataccagcagaaatgctagcaagcattgatctgaagtacttcaatgaatcaacaaagaagaaaattgagaaaataaggcctcttttaatggatggcacagcatcactgagtcctgggatgatgatgggcatgttcaacatgctaagtacggtcttgggagtctcgatactgaatcttggacaaaagaaatacaccaagacaacatactggtgggatgggctccaatcatccgacgattttgctctcatagtgaatgcaccaaaccatgagggaatacaagcaggagtggacagattctacaggacctgcaagttagtgggaatcaacatgagcaaaaagaagtcctatataaataagacagggacatttgaattcacaagctttttttatcgctatggatttgtggctaattttagcatggagctacccagctttggagtgtctggagtaaatgaatcagctgacatgagtattggagtaacagtgataaagaacaacatgataaacaatgaccttggacctgcaacggcccagatggctcttcaattgttcatcaaagactacagatacacatataggtgccataggggagacacacaaattcagacgagaagatcatttgagttaaagaagctgtgggatcaaacccaatcaaaggtagggctattagtatcagatggagggccaaacttgtacaatatacggaatcttcacattcctgaagtctgcttgaaatgggagctaatggatgatgattatcggggaagactttgcaatcccctgaatccctttgtaagtcataaggagattgattctgtaaacaatgctgtggtaatgccagcccatggtccagccaaaagcatggaatatgatgccgttgcaactacacattcctggattcccaagaggaatcgttctattctcaacacaagccaaaggggaattcttgaggatgaacagatgtaccagaagtgttgcaatctattcgagaaatttttccctagcagttcatataggagaccggttggaatttctagcatggtggaggccatggtatctagggcccggattgatgccagggtcgactttgagtctggacggatcaagaaagaagagttctctgagatcatgaagatctgttccaccattgaagaactcagacggcaaaaataa

>H1N1_PB1_KP637644_A/Thailand/SP08207/2009

atggatgtcaatccgacattactcttcttaaaagtgccagcacaaaatgctataagcacaacttttccttatactggtgaccctccttacagccatgggacaggaacagggtacaccatggatacagtcaacaggacacaccagtactcagaaagaggaagatggacaaaaaataccgaaacgggagcaccgcaacttaacccaattgatggtcccttaccggaagacaatgaaccaagtggctatgcccaaacagattgtgtattagaagcaatggctttccttgaagaatcccatcccggtatctttgaaaactcttgtattgaaacaatggaggttgttcaacaaacaagggtggacaaactgacacaaggcagacagacctatgactggactctaaataggaaccagcctgctgccacagcattggcaaacactatagaagtatttagatcaaacggcctcatagcaaatgaatctgggaggttaatagacttccttaaagatgtaatggagtcgatggacagaggcgaagtagaggtcacaactcattttcaaagaaagaggagagtgagagacaatgtaactaaaaaaatggtgacccaaagaacaataggaaaaaagaaacataaattagacaaaaggagttacctaattagggcattaaccctgaacacaatgaccaaagatgctgagagggggaaactaaaacgcagagcaattgcaaccccaggaatgcaaataagggggtttgtatactttgttgagacactggcaagaagcatatgtgaaaagcttgaacaatcaggattgccagttggaggaaatgagaagaaagcaaagttagcaaatgttgtaaggaagatgatgaccaactcccaggacactgaaatttctttcaccataaccggagataacacaaaatggaacgaaaatcaaaaccctagaatgttcttggccatgatcacatatataaccaaaaatcagcctgaatggttcagaaatattctaagtattgctccaataatgttttcaaacaagatggcgagactaggcaaggggtacatgtttgaaagcaagagtatgaaactgagaactcaaatacctgcagagatgctagccaacatagacttgaaatatttcaatgattcaactaaaaggaaaattgaaaaaatccgaccattattaatagatggaactgcatcattgagtcctggaatgatgatgggcatgttcaatatgttgagcacagtcttgggcgtctccattctgaatcttgggcaaaagagatacaccaagactacttactggtgggatggtcttcaatcgtctgatgattttgctttgattgtgaacgcacccaattatgcaggaattcaagctggagttgacaggttttatcgaacctgtaagctgctcggaattaatatgagcaaaaagaagtcttacataaacagaacaggtacctttgaattcacgagctttttctatcgttatgggtttgttgccaatttcagcatggagcttcctagttttggggtgtctggggtcaatgaatctgcagacatgagtattggagtcactgtcatcaaaaacaatatgataaacaatgaccttggcccagcaactgctcaaatggcccttcagttatttatcaaagattacaggtacacttatcgatgccaccgaggtgacacacaaatacaaacccggagatcatttgagataaagaaactatgggaccaaacccgctcaaaagctggactgttggtctctgatggaggccccaatttgtataacattagaaatctccatattcctgaagtttgcttgaaatgggagttgatggatgaggattaccaggggcgtttatgcaacccattaaacccgtttgtcagccataaagagattgaatctgtgaacagtgcagtgataatgccggcacatggtccagccaaaaatatggagtatgacgctgttgcaacaacacactcctgggtccccaaaagaaatcgatccattttgaacacgagccaaagggggatacttgaagatgagcaaatgtatcagaggtgctgcaatttatttgaaaaattcttcccaagtagctcatacagaagaccagttggaatatccagtatggtagaggctatggtctcaagagcccgaattgatgcacggattgatttcgaatctggaaggataaagaaagaggaatttgctgagatcatgaagatctgttccaccattgaagacctcagacggcaaaaatga

>H1N1_PB1_LN846540_A/England/331/2010

atggatgtcaatccgactctgcttttcctgaaaattccagcgcaaaatgccataagcaccacattcccttatactggagatcctccatacagccatggaacaggaacaggatacaccatggacacagtaaacagaacacaccaatactcagaaaagggaaagtggacgacaaacacagagactggtgcaccccagctcaacccgattgatggaccactacctgaggataatgaaccaagtgggtatgcacaaacagactgtgttctagaggctatggctttccttgaagaatcccacccaggaatatttgagaattcatgccttgaaacaatggaagttgttcaacaaacaagggtagataaactaactcaaggtcgccagacttatgattggacattaaacagaaatcaaccggcagcaactgcattggccaacaccatagaagtctttagatcgaatggcctaacagctaatgagtcaggaaggctaatagatttcttaaaggatgtaatggaatcaatgaacaaagaggaaatagagataacaacccactttcaaagaaaaaggagagtaagagacaacatgaccaagaagatggtcacgcaaagaacaatagggaagaaaaaacaaaaactgaataagagaggctatctaataagagcactgacgttaaatacgatgaccaaagatgcagagagaggcaagttaaaaagaagggctatcgcaacacctgggatgcagattagaggtttcgtatactttgttgaagctttagctaggagcatttgcgaaangcttgaacagtctgggctcccagtagggggcaatgaaaagaaggccaaactggcaaatgttgtgagaaagatgatgactaattcacaagacacagagatttctttcacaatcactggggacaacactaagtggaatgaaaatcaaaatcctcgaatgttcctggcgatgattacatatatcaccagaaatcaacccgagtggttcagaaacatcctgagcatggcacccataatgttctcaaacaaaatggcaagactagggaaagggtacatgttcgagagtaaaagaatgaagattcgaacacaaataccagcagaaatgctagcaagcattgacctgaagtacttcaacgaaccaacaaagaagaaaattgagaaaataaggcctcttctaatagatggcacagcatcactgagtcctgggatgatgatgggcatgttcaacatgctaagtacggtcttgggagtctcgatactgaatcttggacaaaagaaatacaccaagacaatatactggtgggatgggctccaatcatccgacgattttgctctcatagtgaatgcaccaaaccatgagggaatacaagcaggagtggacagattctacaggacctgcaagttagtgggaatcaacatgagcaaaaagaagtcctatataaataagacagggacatttgaattcacaagctttttttatcgctatggatttgtggctaattttagcatggagctacccagctttggagtgtctggagtaaatgaatcagctgacatgagtattggagtaacagtgataaagaacaacatgataaacaatgaccttggacctgcaacggcccagatggctcttcaattgttcatcaaagactacagatacacatataggtgccataggggagacacacaaattcagacgagaagatcatttgagttaaagaagctgtgggatcaaacccaatcaaaggtagggctattagtatcagatggaggaccaaacttatacaatatacggaatcttcacattcctgaagtctgcttaaaatgggagctgatggatgatgattatcggggaagactttgtaatcccctgaatccctttgtcagtcataaagagattgattctgtaaacaatgctgtggtaatgccagcccatggtccagtcaaaagcatggaatatgatgccgttgcaactacacattcctggatccccaagaggaatcgttctattctcaacacaagccaaaggggaattcttgaggatgaacagatgtatcagaagtgctgcaatctattcgagaaatttttccctagcagttcatataggagaccggttggaatttctagcatggtggaggccatggtgtctagggcccggattgatgccagggtcgacttcgagtctggacggatcaagaaagaagagttctctgagatcatgaagatctgttccaccattgaagaactcagacggcaaaaataa

>H1N1_PB1_LN846572_A/England/10740571/2010

atggatgtcaatccgactctacttttcctaaaaattccagcgcaaaatgccataagcaccacattcccttatactggagatcctccatacagccatggaacaggaacaggatacaccatggacacagtaaacagaacacaccaatactcagaaaagggaaagtggacgacaaacacagagactggtgcaccccagctcaacccgattgatggaccactacctgaggataatgaaccaagtgggtatgcacaaacagactgtgttctagaggctatggctttccttgaagaatcccacccaggaatatttgagaattcatgccttgaaacaatggaagttgttcaacaaacaagggtagataaactaactcaaggtcgccagacttacgattggacattaaacagaaatcaaccggcagcaactgcattggccaacaccatagaagtctttagatcgaatggcctaacagctaatgagtcaggaaggctaatagatttcttaaaggatgtaatggaatcaatgaacaaagaggaaatagagataacaacccactttcaaagaaaaaggagaataagagacaacatgaccaagaagatggtcacgcaaagaacaatagggaagnaaaaacaaagactgaataagagaggctatctaataagagcactgacattaaatacgatgaccaaagatgcagagagaggcaagttaaaaagaagggctatcgcaacacctgggatgcagattagaggtttcgtatactttgttgaaactttagctaggagcatttgcgaaaagcttgaacagtctgggctcccagtagggggcaatgaaaagaaggccaaactggcaaatgttgtgagaaagatgatgactaattcacaagacacagagatttctttcacaatcactggggacaacactaagtggaatgaaaatcaaaatcctcgaatgttcctggcgatgattacatatatcaccagaaatcaacccgagtggttcagaaacatcctgagcatggcacccataatgttctcaaacaaaatggcaagactagggaaagggtacatgttcgagagtaaaagaatgaagattcgaacacaaataccagcagaaatgctagcaagcattgacctgaagtacttcaatgaatcaacaaagaagaaaattgagaaaataaggcctcttttaatagatggcacagcatcactgagtcctgggatgatgatgggcatgttcaacatgctaagtacggtcttgggagtctcgatactgaatcttggacaaaagaaatacaccaagacaatatactggtgggatgggctccaatcatccgacgattttgctctcatagtgaatgcaccaaaccatgagggaatacaagcaggagtggacagattctacaggacctgcaagttagtgggaatcaacatgagcaaaaagaagtcctatataaataagacagggacatttgaattcacaagcttttnttatcgctatggatttgtggctaattttagcatggagctacccagctttggagtgtctggagtaaatgaatcagctgacatgagtattggagtaacagtgataaagaacaacatgataaacaatgaccttggacctgcaacggcccagatggctcttcaattgttcatcaaagactacagatacacatataggtgccataggggagacacacaaattcagacgagaagatcatttgagttaaagaagctgtgggatcaaacccaatcaaaggtagggctattagtatcagatggaggaccaaacttatacaatatacggaatcttcacattcctgaagtctgcttaaaatgggagctaatggatgatgattatcggggaagactttgtaatcccctgaatccctttgtaagtcataaagagattgattctgtaaacaatgctgtggtaatgccagcccatggtccagccaaaagcatggagtatgatgccgttgcaactacacattcctggactcccaagaggaatcgttctattctcaacacaagccaaaggggaattcttgaggatgaacagatgtaccagaagtgctgcaatctattcgagaaatttttccctagcagttcatataggagaccggttggaatttctagcatggtggaggccatggtgtctagggcccggattgatgccagggtcgactttgagtctggacggatcaagaaagaagagttctctgagatcatgaagatctgttccaccattgaagaactcagacggcataaataa

>H1N1_PB1_LN846588_A/England/00380718/2010

atggatgtcaatccgactctacttttcctaaaaattccagcgcaaaatgccataagcaccacattcccttatactggagatcctccatacagccatggaacaggaacaggatacaccatggacacagtaaacagaacacaccaatactcagaaaagggaaagtggacgacaaacacagagactggtgcaccccagctcaacccgattgatggaccactacctgaggataatgaaccaagtgggtatgcacaaacagactgtgttctagaggctatggctttccttgaagaatcccacccaggaatatttgagaattcatgccttgaaacaatggaagttgttcaacaaacaagggtagataaactaactcaaggtcgccagacttatgattggacattaaacagaaatcaaccggcagcaactgcattggccaacaccatagaagtctttagatcgaatggcctaacagctaatgagtcaggaaggctaatagatttcttaaaagatgtaatggaatcaatgaacaaagaggaaatagagataacaacccactttcaaagaaaaaggagagtaagagacaacatgaccaagaagatggtcacgcaaagaacaatagggaagaaaaaacaaagactgaataagagaggctatctaataagagcactgacattaaatacgatgaccaaagatgcagagagaggcaagttaaaaagaagggctatcgcaacacctgggatgcagattagaggtttcgtatactttgttgaaactttagctaggagcatttgcgaaaagcttgaacagtctgggctcccagtaggaggcaatgaaaagaaggccaaactggcaaatgttgtgagaaagatgatgactaattcacaagacacagagatttctttcacaatcactggggacaacactaagtggaatgaaaatcaaaatcctcgaatgttcctggcgatgattacatatatcaccagaaatcaacccgagtggttcagaaacatcctgagcatggcacccataatgttctcaaacaaaatggcaagactagggaaagggtacatgttcgagagtaaaagaatgaagattcgaacacaaataccagcagaaatgctagcaagcattgacctgaagtacttcaatgaatcaacaaagaagaaaattgagaaaataaggcctcttctaatagatggcacagcatcactgagtcctgggatgatgatgggcatgttcaacatgctaagtacggtcttgggagtctcgatactgaatcttggacaaaagaaatacaccaagacaatatactggtgggatgggctccaatcatccgacgattttgctctcatagtgaatgcaccaaaccatgagggaatacaagcaggagtggacagattctacagaacctgcaagttagtgggaatcaacatgagcaaaaagaagtcctatataaataagacagggacatttgaattcacaagctttttttatcgctatggatttgtggctaattttagcatggagctacccagctttggtgtgtctggagtaaatgaatcagctgacatgagtattggagtaacagtgataaagaacaacatgataaacaatgaccttggacctgcaacggcccagatggctcttcaattgttcatcaaagactacagatacacatataggtgccataggggagacacacaaattcagacgagaagatcatttgagttaaagaagctgtgggatcaaacccaatcaaaggtagggctattagtatcagatggaggaccaaacttatacaatatacggaatcttcacattcctgaagtctgcttaaaatgggagctaatggatgatgattatcggggaagactttgtaatcccctgaatccctttgtcagtcataaagagattgattctgtaaacaatgctgtggtaatgccagcccatggtccagccaaaagcatggaatatgatgccgttgcaactacacattcctggattcccaagaggaatcgctctattctcaacacaagccaaaggggaattcttgaggatgaacagatgtaccagaagtgctgcaatctattcgagaaatttttccctagcagttcatataggagaccggttggaatttctagcatggtggaggccatggtgtctagggcccggattgatgccagggtcgacttcgagtctggacggatcaagaaagaagagttctctgagatcatgaagatctgttccaccattgaagaactcagacggcaaaaacaa

>H1N1_PB1_LN846621_A/England/343/2010

atggatgtcaatccgactctacttttcctaaaaattccagcgcaaaatgccataagcaccacattcccttatactggagatcctccatacagccatggaacaggaacaggatacaccatggacacagtaaacagaacacaccaatactcagaaaagggaaagtggacgacaaacacagagactggtgcaccccagctcaacccgattgatggaccactacctgaggataatgaaccaagtgggtatgcacaaacagactgtgttctagaggctatggctttccttgaagaatcccacccaggaatatttgagaattcatgccttgaaacaatggaagttgttcaacaaacaagggtagataaactaactcaaggtcgccagacttatgattggacattaaacagaaatcaaccggcagcaactgcattggccaacaccatagaagtctttagatcgaatggcctaacagctaatgagtcaggaaggctaatagatttcttaaaggatgtaatggaatcaatgaacaaagaggaaatagagataacaacccactttcaaagaaaaaggagagtaagagacaacatgaccaagaagatggtcacgcaaagaacaatagggaagaaaaaacaaagactgaataagagaggctatctaataagagcactgacattaaatacgatgaccaaagatgcagagagaggcaagttaaaaagaagggctatcgcaacacctgggatgcagattagaggtttcgtatactttgttgaaactttagctaggagcatttgcgaaaagcttgaacagtctgggctcccagtagggggcaatgaaaagaaggccaaactggcaaatgttgtgagaaagatgatgactaattcacaagacacagagatttctttcacaatcactggggacaacactaagtggaatgaaaaccaaaatcctcgaatgttcctggcgatgattacatatatcaccagaaatcaacccgagtggttcagaaacatcctgagcatggctcccataatgttctcaaacaaaatggcaagactagggaaagggtacatgttcgagagtaaaagaatgaagattcgaacgcaaataccagcagaaatgctagcaagcattggcctgaagtacttcaatgaatcaacaaagaagaaaattgagaaaataaggcctcttttaatagatggcacagcatcactgagtcctgggatgatgatgggcatgttcaacatgctaagtacggtcttgggagtctcgatactgaatcttggacaaaagaaatacaccaagacaatatactggtgggatgggctccaatcatccgacgattttgctctcatagtgaatgcaccaaaccatgagggaatacaagcaggagtggacagattttacaggacctgcaagttagtgggaatcaacatgagcaaaaagaagtcctatataaataagacagggacatttgaattcacaagctttttttatcgctatggatttgtggctaattttagcatggagctacccagctttggagtgtctggagtaaatgaatcagctgacatgagtattggagtaacagtgataaagaacaacatgataaacaatgaccttggacctgcaacggcccagatggctcttcaattgttcatcaaagactacagatacacatataggtgccataggggagacacacaaattcagacgagaagatcatttgagttaaagaagctgtgggatcaaacccaatcaaaggtagggctattagtatcagatggaggaccaaacttatacaatatacggaatcttcacattcctgaagtctgcttaaaatgggagctaatggatgatgactatcggggaagactttgtaatcccctgaatccctttgtaagtcataaagagattgattctgtaaacaatgctgtgataatgccagctcatggtccaaccaaaagcatggaatatgatgccgttgcaactacacattcctggattcccaagaggaatcgttctattctcaacacaagccaaaggggaattcttgaggatgaacagatgtaccagaagtgctgcaatctattcgagaaatttttccctagcagttcatataggagaccggttggaatttctagcatggtggaggccatggtgtctagggcccggattgatgccagggtcgactttgagtctggacggatcaagaaagaagagttctctgagatcatgaagatctgttccaccattgaagaactcagacggcaaaaataa

>H1N1_PB1_LN867500_A/England/04980055/2010

atggatgtcaatccgactctacttttcctaaaaattccagcgcaaaatgccataagcaccacattcccttatactggagatcctccatacagccatggaacaggaacaggatacaccatggacacagtaaacagaacacaccaatactcagaaaagggaaagtggacgacaaacacagagactggtgcaccccagctcaacccgattgatggaccactacctgaggataatgaaccaagtgggtatgcacaaacagactgtgttctagaggctatggctttccttgaagaatcccacccaggaatatttgagaattcatgccttgaaacaatggaagttgttcaacaaacaagggtagataaactaactcaaggtcgccagacttatgattggacattaaacagaaatcaaccggcagcaactgcattggccaacaccatagaagtctttagatcgaatggcctaacagctaatgagtcaggaaggctaatagatttcttaaaggatgtaatggaatcaatgaacaaagaggaaatagagataacaacccactttcaaagaaaaaggagagtaagagacaacatgaccaagaagatggtcacgcaaagaacaatagggaagaaaaaacaaagactgaataagagaggctatctaataagagcactgacattaaatacgatgaccaaagatgcagagagaggcaagttaaaaagaagggctatcgcaacacctgggatgcagattagaggtttcgtatactttgttgaaactttagctaggagcatttgcgaaaagcttgaacagtctgggctcccagtagggggcaatgaaaagaaggccaaactggcaaatgttgtgagaaagatgatgactaattcacaagacacagagatttctttcacaatcactggggacaacactaagtggaatgaaaaccaaaatcctcgaatgttcctggcgatgattacatatatcaccagaaatcaacccgagtggttcagaaacatcctgagcatggcacccataatgttctcaaacaaaatggcaagactagggaaagggtacatgttcgagagtaaaagaatgaagattcgaacgcaaataccagcagaaatgctagcaagcattgacctgaagtacttcaatgaatcaacaaagaagaaaattgagaaaataaggcctcttttaatagatggcacagcatcactgagtcctgggatgatgatgggcatgttcaacatgctaagtacggtcttgggagtctcgatactgaatcttggacaaaagaaatacaccaagacaatatactggtgggatgggctccaatcatccgacgattttgctctcatagtgaatgcaccaaaccatgagggaatacaagcaggagtggacagattttacaggacctgcaagttagtgggaatcaacatgagcaaaaagaagtcctatataaataagacagggacatttgaattcacaagcttttnttatcgctatggatttgtggctaattttagcatggagctacccagctttggagtgtctggagtaaatgaatcagctgacatgagtattggagtaacagtgataaagaacaacatgataaacaatgaccttggacctgcaacggcccagatggctcttcaattgttcatcaaagactacagatacacatataggtgccataggggagacacacaaattcagacgagaagatcatttgagttaaagaagctgtgggatcaaacccaatcaaaggtagggctattagtatcagatggaggaccaaacttatacaatatacggaatcttcacattcctgaagtctgcttaaaatgggagctaatggatgatgactatcggggaagactttgtaatcccctgaatccctttgtaagtcataaagagattgattctgtaaacaatgctgtgataatgccagctcatggtccagccaaaagcatggaatatgatgccgttgcaactacacattcctggattcccaagaggaatcgttctattctcaacacaagccaaaggggaattcttgaggatgaacagatgtaccagaagtgctgcaatctattcgagaaatttttccctagcagttcatataggagaccggttggaatttctagcatggtggaggccatggtgtctagggcccggattgatgccagggtcgactttgagtctggacggatcaagaaagaagagttctctgagatcatgaagatctgttccaccattgaagaactcagacggcaaaaataa

>H1N1_PB1_LN867698_A/England/05100301/2010

atggatgtcaatccgactctacttttcctaaaaattccagcgcaaaatgccataagcaccacattcccttatactggagatcctccatacagccatggaacaggaacaggatacaccatggacacagtaaacagaacacaccaatactcagaaaagggaaagtggacgacaaacacagagactggtgcaccccagctcaacccgattgatggaccactacctgaggataatgaaccaagtgggtatgcacaaacagactgtgttctagaggctatggctttccttgaagaatcccacccaggaatatttgagaattcatgccttgaaacaatggaagttgttcaacaaacaagggtagataaactaactcaaggtcgccagacttatgattggacattaaacagaaatcaaccggcagcaactgcattggccaacaccatagaagtctttagatcgaatggcctaacagctaatgagtcaggaaggctaatagatttcttaaaggatgtaatggaatcaatgaacaaagaggaaatagagataacaacccactttcaaagaaaaaggagagtaagagacaacatgaccaagaagatggtcacgcaaagaacaatagggaagaaaaaacaaagactgaataagagaggctatctaataagagcactgacattaaatacgatgaccaaagatgcagagagaggcaagttaaaaagaagggctatcgcaacacctgggatgcagattagaggtttcgtatactttgttgaaactttagctaggagcatttgcgaaaagcttgaacagtctgggctcccagtagggggcaatgaaaagaaggccaaactggcaaatgttgtgagaaagatgatgactaattcacaagacacagagatttctttcacaatcactggggacaacactaagtggaatgaaaaccaaaatcctcgaatgttcctggcgatgattacatatatcaccagaaatcaacccgagtggttcagaaacatcctgagcatggcacccataatgttctcaaacaaaatggcaagactagggaaagggtacatgttcgagagtaaaagaatgaagattcgaacgcaaataccagcagaaatgctagcaagcattgacctgaagtacttcaatgaatcaacaaagaagaaaattgagaaaataaggcctcttttaatagatggcacagcatcactgagtcctgggatgatgatgggcatgttcaacatgctaagtacggtcttgggagtctcgatactgaatcttggacaaaagaaatacaccaagacaatatactggtgggatgggctccaatcatccgacgattttgctctcatagtgaatgcaccaaaccatgagggaatacaagcaggagtggacagattttacaggacctgcaagttagtgggaatcaacatgagcaaaaagaagtcctatataaataagacagggacatttgaattcacaagctttttttatcgctatggatttgtggctaattttagcatggagctacccagctttggagtgtctggagtaaatgaatcagctgacatgagtattggagtaacagtgataaagaacaacatgataaacaatgaccttggacctgcaacggcccagatggctcttcaattgttcatcaaagactacagatacacatataggtgccataggggagacacacaaattcagacgagaagatcatttgagttaaagaagctgtgggatcaaacccaatcaaaggtagggctattagtatcagatggaggaccaaacttatacaatatacggaatcttcacattcctgaagtctgcttaaaatgggagctaatggatgatgattatcggggaagactttgtaatcccctgaatccctttgtaagtcataaagagattgattctgtaaacaatgctgtgataatgccagctcatggtccagccaaaagcatggaatatgatgccgttgcaactacacattcctggattcccaagaggaatcgttctattctcaacacaagccaaaggggaattcttgaggatgaacagatgtaccagaagtgctgcaatctattcgagaaatttttccctagcagttcatataggagaccggttggaatttctagcannntggaggccatggtgtctagggcccggattgatgccagggtcgactttgagtctggacggatcaagaaagaagagttctctgagatcatnaagatctgttccaccattgaagaactcagacggcaaaaataa

>H1N1_PB1_KT180511_A/Singapore/DMS71/2010

atggatgtcaatccgactctacttttcctaaaaattccagcgcaaaatgccataagcaccacattcccttatactggagatcctccatacagccatggaacaggaacaggatacaccatggacacagtaaacagaacacaccaatactcagaaaagggaaagtggacgacaaacacagagactggtgcaccccagctcaacccgattgatggaccactacctgaggataatgaaccaagtgggtatgcacaaacagactgtgttctagaggctatggctttccttgaagaatcccacccaggaatatttgagaattcatgccttgaaacaatggaagttgttcaacaaacaagggtagataaactaactcaaggtcgccagacttatgattggacattaaacagaaatcaaccggcagcaactgcattggccaacaccatagaagtctttagatcgaatggcctaacagctaatgagtcaggaaggctaatagatttcttaaaggatgtaatggaatcaatgaacaaagaggaaatagagataacaacccactttcaaagaaaaaggagagtaagagacaacatgaccaagaagatggtcacgcaaagaacaatagggaagaaaaaacaaagactgaataagagaggctatctaataagagcactgacattaaatacgatgaccaaagatgcagagagaggcaagttaaaaagaagggctatcgcaacacctgggatgcagattagaggtttcgtatactttgttgaagctttagctaggagcatttgcgaaaagcttgaacagtctgggctcccagtagggggcaatgaaaagaaggccaaactggcaaatgttgtgagaaagatgatgactaattcacaagacacagagatttctttcacaatcactggggacaacactaagtggaatgaaaatcaaaatcctcgaatgttcctggcaatgattacatatatcaccagaaatcaacccgagtggttcagaaacatcctgagcatggcacccataatgttctcaaacaaaatggcaagactagggaaagggtacatgttcgagagtaaaagaatgaagattcgaacacaaataccagcagaaatgctagcaagcattgacctgaagtacttcaatgaatcaacaaagaagaaaattgagaaaataaggcctcttctaatagatggcacagcatcactgagtcctgggatgatgatgggcatgttcaacatgctaagtacggtcttgggagtctcgatactgaatcttggacaaaagaaatacaccaagacaatatactggtgggatgggctccaatcatccgacgattttgctctcatagtgaatgcaccaaaccatgagggaatacaagcaggagtggacagattctacaggacctgcaaattagtgggaatcaacatgagcaaaaagaagtcctatataaataagacagggacatttgaattcacaagctttttttatcgctatggatttgtggctaattttagcatggagctacccagctttggagtgtctggagtaaatgaatcagctgacatgagtattggagtaacagtgataaagaacaacatgataaacaatgaccttggacctgcaacggcccagatggctcttcaattgttcatcaaagactacagatacacatataggtgccataggggagacacacaaattcagacgagaagatcatttgagttaaagaagctgtgggatcaaacccaatcaaaggtagggctattagtatcagatgggggaccaaacttatacaatatacgaaatcttcacattcctgaagtctgcttaaaatgggagctaatggatgatgattatcggggaagactttgtaatcccctgaatccctttgtcagtcataaagagattgattctgtaaacaatgctgtggtaatgccagcccatggtccagccaaaagcatggaatatgatgccgttgcaactacacattcctggattcccaagaggaatcgttctattctcaacacaagccaaaggggaattcttgaggatgaacagatgtaccagaagtgctgcaatctattcgagaaatttttccctagcagttcatataggagaccggttggaatttctagtatggtagaggccatggtgtctagggcccggattgatgccagggtcgacttcgagtctggacggatcaagaaagaagagttctctgagatcatgaagatctgttccaccattgaagaactcagacggcaaaaataa

>H1N1_PB1_KT180488_A/Singapore/DMS56/2009

atggatgtcaatccgactctacttttcctaaaaattccagcgcaaaatgccataagcaccacattcccttatactggagatcctccatacagccatggaacaggaacaggatacaccatggacacagtaaacagaacacaccaatactcagaaaagggaaagtggacgacaaacacggagactggtgcaccccagctcaacccgattgatggaccactacctgaggataatgaaccaagtgggtatgcacaaacagactgtgttctagaggctatggctttccttgaagaatcccacccaggaatatttgagaattcatgccttgaaacaatggaagttgttcaacaaacaagggtagataaactaactcaaggtcgccagacttatgattggacattaaacagaaatcaaccggcagcaactgcattggccaacaccatagaagtctttagatcgaatggcctaacagctaatgagtcaggaaggctaatagatttcttaaaggatgtaatggaatcaatgaacaaagaggaaatagagataacaacccactttcaaagaaaaaggagagtaagagacaacatgaccaagaagatggtcacgcaaagaacaatagggaagaaaaaacaaagactgaataagagaggctatctaataagagcactgacattaaatacgatgaccaaagatgcagagagaggcaagttaaaaagaagggctatcgcaacacctgggatgcagattagaggtttcgtatactttgttgaaactttagctaggagcatttgcgaaaagcttgaacagtctgggctcccagtagggggcaatgaaaagaaggctaaactggcaaatgttgtgagaaagatgatgactaattcacaagacacagagatttctttcacaatcactggggacaacactaagtggaatgaaaatcaaaatcctcgaatgttcctggcgatgattacatatatcaccagaaatcaacccgagtggttcagaaacatcctgagcatggcacccataatgttctcaaacaaaatggcaagactagggaaagggtacatgttcgagagtaaaagaatgaagattcgaacacaaataccagcagaaatgctagcaagcattgacctgaagtacttcaatgaatcaacaaagaagaaaattgagaaaataaggcctcttctaatagatggcacagcatcactgagtcctgggatgatgatgggcatgttcaacatgctaagtacggtcttgggagtctcgatactaaatcttggacaaaagaaatacaccaagacaatatactggtgggatgggctccaatcatccgacgattttgctctcatagtgaatgcaccaaaccatgagggaatacaagcaggagtggacagattctacaggacctgcaagttagtgggaatcaacatgagcaaaaagaagtcctatataaataagacagggacatttgaattcacaagctttttttatcgctatggatttgtggctaattttagcatggagctacccagctttggagtgtctggagtaaatgaatcagctgacatgagtattggagtaacagtgataaagaacaacatgataaacaatgaccttggacctgcaacggcccagatggctcttcaattgttcatcaaagactacagatacacatataggtgccataggggagacacacaaattcagacgagaagatcatttgagttaaagaagctgtgggatcaaacccaatcaaaggtagggctattagtatcagatggaggaccaaacttatacaatatacggaatcttcacattcctgaagtctgcttaaaatgggagctaatggatgatgattatcggggaagactttgtaatcccctgaatccctttgtcagtcataaagagattgattctgtaaacaatgctgtggtaatgccagcccatggtccagccaaaagcatggaatatgatgccgttgcaactacacattcctggattcccaagaggaatcgttctattctcaacacaagccaaaggggaattcttgaggatgaacagatgtaccagaagtgctgcaatctattcgagaaatttttccctagcagttcatataggagaccggttggaatttctagcatggtggaggccatggtgtctagggcccggattgatgccagggtcgacttcgagtctggacggatcaagaaagaagagttctctgagatcatgaagatctgttccaccattgaagaactcagacggcaaaaataa

>H1N1_PB1_KT180475_A/Singapore/DMS43/2009

atggatgtcaatccgactctacttttcctaaaaattccagcgcaaaatgccataagcaccacattcccttatactggagatcctccatacagccatggaacaggaacaggatacaccatggacacagtaaacagaacacaccaatactcagaaaagggaaagtggacgacaaacacagagactggtgcaccccagctcaacccgattgatggaccactacctgaggataatgaaccaagtgggtatgcacaaacagactgtgttctagaggctatggctttccttgaagaatcccacccaggaatatttgagaattcatgccttgaaacaatggaagttgttcaacaaacaagggtagataaactaactcaaggtcgccagacttatgattggacattaaacagaaatcaaccggcagcaactgcattggccaacaccatagaagtctttagatcgaatggcctaacagctaatgagtcaggaaggctaatagatttcttaaaggatgtaatggaatcaatgaacaaagaggaaatagagataacaacccactttcaaagaaaaaggagagtaagagacaacatgaccaagaagatggtcacacaaagaacaatagggaagaaaaaacaaagactgaataagagaggctatctaataagagcactgacattaaatacgatgaccaaagatgcagagagaggcaagttaaaaagaagggctatcgcaacacctgggatgcagattagaggtttcgtatactttgttgaaactttagctaggagcatttgcgaaaagcttgaacagtctgggctcccagtagggggcaatgaaaagaaggccaaactggcaaatgttgtgagaaagatgatgactaattcacaagacacagagatttctttcacaatcactggggacaacactaagtggaatgaaaatcaaaatcctcgaatgttcctggcgatgattacatatatcaccagaaatcaacccgagtggttcagaaacatcctgagcatggcacccataatgttctcaaacaaaatggcaagactagggaaagggtacatgttcgagagtaaaagaatgaagattcgaacacaaataccagcagaaatgctagcaagcattgacctgaagtacttcaatgaatcaacaaagaagaaaattgagaaaataaggcctcttctaatagatggcacagcatcactgagtcctgggatgatgatgggcatgttcaacatgctaagtacggtcttgggagtctcgatactgaatcttggacaaaagaaatacaccaagacaatatactggtgggatgggctccaatcatccgacgattttgctctcatagtgaatgcaccaaaccatgagggaatacaagcaggagtggacagattctacaggacctgcaagttagtgggaatcaacatgagcaaaaagaagtcctatataaataagacagggacatttgaattcacaagctttttttatcgctatggatttgtggctaattttagcatggagctacccagctttggagtgtctggagtaaatgaatcagctgacatgagtattggagtaacagtgataaagaacaacatgataaacaatgaccttggacctgcaacggcccagatggctcttcaattgttcatcaaagactacagatacacatataggtgccataggggagacacacaaattcagacgagaagatcatttgagttaaagaagctgtgggatcaaacccaatcaaaggtagggctattagtatcagatggaggaccaaacttatacaatatacggaatcttcacattcctgaagtctgcttaaaatgggagctaatggatgatgattatcggggaagactttgtaatcccctgaatccctttgtcagtcataaagagattgattctgtaaacaatgctgtggtaatgccagcccatggtccagccaaaagcatggaatatgatgccgttgcaactacacattcctggattcccaagaggaatcgttctattctcaacacaagccaaaggggaattcttgaggatgaacagatgtaccagaagtgctgcaatctattcgagaaatttttccctagcagttcatataggagaccggttggaatttctagcatggtggaggccatggtgtctagggcccggattgatgccagggtcgacttcgagtctggacggatcaagaaagaagagttctctgagatcatgaagatctgttccaccattgaagaactcagacggcaaaaataa

>H1N1_PB1_KT180453_A/Singapore/DMS19/2009

atggatgtcaatccgactctacttttcctaaaaattccagcgcaaaatgccataagcaccacattcccttatactggagatcctccatacagccatggaacaggaacaggatacaccatggacacagtaaacagaacacaccaatactcagaaaagggaaagtggacgacaaacacagagactggtgcaccccagctcaacccgattgatggaccactacctgaggataatgaaccaagtgggtatgcacaaacagactgtgttctagaggctatggctttccttgaagaatcccacccaggaatatttgagaattcatgccttgaaacaatggaagttgttcaacaaacaagggtagataaactaactcaaggtcgccagacttatgattggacattaaacagaaatcaaccggcagcaactgcattggccaacaccatagaagtctttagatcgaatggcctaacagctaatgagtcaggaaggctaatagatttcttaaaggatgtaatggaatcaatgaacaaagaggaaatagagataacaacccactttcaaagaaaaaggagagtaagagacaacatgaccaagaagatggtcacgcaaagaacaatagggaagaaaaaacaaagactgaataagagaggctatctaataagagcactgacattaaatacgatgaccaaagatgcagagagaggcaagttaaaaagaagggctatcgcaacacctgggatgcagattagaggtttcgtatactttgttgaagctttagctaggagcatttgcgaaaagcttgaacagtctgggctcccagtagggggcaatgaaaagaaggccaaactggcaaatgttgtgagaaagatgatgactaattcacaagacacagagatttctttcacaatcactggggacaacactaagtggaatgaaaatcaaaatcctcgaatgttcctggcaatgattacatatatcaccagaaatcaacccgagtggttcagaaacatcctgagcatggcacccataatgttctcaaacaaaatggcaagactagggaaagggtacatgttcgagagtaaaagaatgaagattcgaacacaaataccagcagaaatgctagcaagcattgacctgaagtacttcaatgaatcaacaaagaagaaaattgagaaaataaggcctcttctaatagatggcacagcatcactgagtcctgggatgatgatgggcatgttcaacatgctaagtacggtcttgggagtctcgatactgaatcttggacaaaagaaatacaccaagacaatatactggtgggatgggctccaatcatccgacgattttgctctcatagtgaatgcaccaaaccatgagggaatacaagcaggagtggacagattctacaggacctgcaagttagtgggaatcaacatgagcaaaaagaagtcctatataaataagacagggacatttgaattcacaagctttttttatcgctatggatttgtggctaattttagcatggagctacccagctttggagtgtctggagtaaatgaatcagctgacatgagtattggagtaacagtgataaagaacaacatgataaacaatgaccttggacctgcaacggcccagatggctcttcaattgttcatcaaagactacagatacacatataggtgccataggggagacacacaaattcagacgagaagatcatttgagttaaagaagctgtgggatcaaacccaatcaaaggtagggctattagtatcagatggaggaccaaacttatacaatatacggaatcttcacattcctgaagtctgcttaaaatgggagctaatggatgatgattatcggggaagactttgtaatcccctgaatccctttgtcagtcataaagagattgattctgtaaacaatgctgtggtaatgccagcccatggtccagccaaaagcatggaatatgatgccgttgcaactacacattcctggattcccaagaggaatcgttctattctcaacacaagccaaaggggaattcttgaggatgaacagatgtaccagaagtgctgcaatctattcgagaaatttttccctagcagttcatataggagaccggttggaatttctagcatggtggaggccatggtgtctagggcccggattgatgccagggtcgacttcgagtctggacggatcaagaaagaagagttctctgagatcatgaagatctgttccaccattgaagaactcagacggcaaaaataa

>H1N1_PB1_KT180411_A/Singapore/DMS1230/2011

atggatgtcaatccgactctacttttcctaaaaattccagcgcaaaatgccataagcaccacattcccttatactggagatcctccatacagccatggaacaggaacaggatacaccatggacacagtaaacagaacacaccaatactcagaaaagggaaagtggacgacaaacacagagactggtgcaccccagctcaacccgattgatggaccactacctgaggataatgaaccaagtgggtatgcacaaacagactgtgttctagaggctatggctttccttgaagaatcccacccaggaatatttgagaattcatgccttgaaacaatggaagttgttcaacaaacaagggtagacaaactaactcaaggtcgccagacttatgattggacattaaacagaaatcaaccggcagcaactgcattggccaacaccatagaagtctttagatcgaatggcctaacagctaatgagtcaggaaggctaatagatttcttaaaggatgtaatggaatcaatgaacaaagaggaaatagagataacaacccactttcaaagaaaaaggagagtaagagacaacatgaccaagaagatggtcacgcaaagaacaatagggaagaaaaaacaaagactgaataagagaggctatctaataagagcactgacattaaatacgatgaccaaagatgcagagagaggcaagttaaaaagaagggctatcgcaacacctggaatgcagattagaggtttcgtatactttgttgaaactttagctaggagcatttgcgaaaagcttgaacagtctgggctcccagtagggggcaatgaaaagaaggccaaactggcaaatgttgtgagaaagatgatgactaattcacaagacacagagatctctttcacaatcactggggacaacactaagtggaatgaaaaccaaaatcctcgaatgttcctggcgatgattacatatatcaccagaaatcaacccgagtggttcagaaacatcctgagcatggcacccataatgttctcaaacaaaatggcaagactagggaaagggtacatgttcgagagtaaaagaatgaagattcgaacgcaaataccagcagaaatgctagcaagcattgacctgaagtacttcaatgaatcaacaaagaagaaaattgagaaaataaggcctcttttaatagatggcacagcatcactgagtcctgggatgatgatgggcatgttcaacatgctaagtacggtcttgggagtctcgatactgaatcttggacaaaagaaatacaccaagacaatatactggtgggatgggctccaatcatccgacgattttgctctcatagtgaatgcaccaaaccatgagggaatacaagcaggagtggacagattttacaggacctgcaagttagtgggaatcaacatgagcaaaaagaagtcctatataaataagacagggacatttgaattcacaagctttttttatcgctatggatttgtggctaattttagcatggagctacccagctttggagtgtctggagtaaatgaatcagctgacatgagtattggagtaacagtgataaagaacaacatgataaacaatgaccttggacctgcaacggcccagatggctcttcaattgttcatcaaagactacagatacacatataggtgccataggggagacacacaaattcagacgagaagatcatttgagttaaagaagctgtgggatcaaacccaatcaaaggtagggctattagtatcagatggaggaccaaacttatacaatatacggaatcttcacattcctgaagtctgcttaaaatgggagctaatggatgatgattatcggggaagactttgtaatcccctgaatccctttgtaagtcataaagagattgattctgtaaacaatgctgtggtaatgccagcccatggtccagccaaaagcatggaatatgatgccgttgcaactacacattcctggattcccaagaggaatcgttctattctcaacacaagccaaaggggaattcttgaggatgaacagatgtaccagaagtgctgcaatctattcgagaaatttttccctagcagttcatataggagaccggttggaatttctagcatggtggaggccatggtgtctagggcccggattgatgccagggtcgactttgagtctggacggatcaagaaagaagagttctctgagatcatgaagatctgttccaccattgaagaactcagacggcaaaaataa

>H1N1_PB1_KT180408_A/Singapore/DMS1227/2011

atggatgtcaatccgactctacttttcctaaaaattccagcgcaaaatgccataagcaccacattcccttatactggagatcctccatacagccatggaacaggaacaggatacaccatggacacagtaaacagaacacaccaatactcagaaaagggaaagtggacgacaaacacagagactggtgcaccccagctcaacccgattgatggaccactacctgaggataatgaaccaagtgggtatgcacagacagactgtgttctagaggctatggctttccttgaagaatcccacccaggaatatttgagaattcatgccttgaaacaatggaagctgttcaacaaacaagggtagataaactaactcaaggtcgccagacttatgattggacattaaacagaaatcaaccggcagcaactgcattggccaacaccatagaagtctttagatcgaatggcctaacagctaatgagtcaggaaggctaatagatttcttaaaggatgtaatggaatcaatgaacaaagaggaaatagagataacaacccactttcaaagaaaaaggagagtaagagacaacatgaccaagaagatggtcacgcaaagaacaatagggaagaaaaaacaaagactgaataagagaggctatctaataagagcactgacattaaatacgatgaccaaagatgcagagagaggcaagttaaaaagaagggctattgcaacacctgggatgcagattagaggtttcgtatactttgttgaaactttagctaggagcatttgcgaaaagcttgaacagtctgggctcccagtagggggcaatgaaaagaaggccaaactggcaaatgttgtgagaaagatgatgactaattcacaagacacagagatttctttcacaatcactggggacaacactaagtggaatgaaaatcaaaatcctcgaatgttcctggcgatgattacatatatcaccagaaatcaacccgagtggttcagaaacatcctgagcatggcacccataatgttctcaaacaaaatggcaagactagggaaagggtacatgttcgagagtaaaagaatgaggattcgaacacaaataccagcagaaatgctagcaagcattgacctgaagtacttcaatgaatcaacaaagaagaaaattgagaaaataaggcctcttctaatagatggcacagcatcactgagtcctgggatgatgatgggcatgttcaacatgctaagtacggtcttgggagtctcgatactgaatcttggacaaaagaaatacaccaagacaacatactggtgggatgggctccaatcatccgacgattttgctctcatagtgaatgcaccaaaccatgagggaatacaagcaggagtggacagattctacaggacctgcaagttagtgggaatcaacatgagcaaaaagaagtcatatataaataaaacagggacatttgaattcacaagctttttttatcgctatggatttgtggctaattttagcatggagctacccagctttggagtgtctggagtaaatgaatcagctgacatgagtattggagtaacagtgataaagaacaacatgataaacaatgaccttggacctgcaacggcccagatggctcttcaattgttcatcaaagactacagatacacatataggtgccataggggagacacacaaattcaaacgagaagatcatttgagttaaaaaagctgtgggatcaaacccaatcaaaggtagggctattagtatcagatggaggaccaaacttatacaatatacggaatcttcacattcctgaagtctgcttaaaatgggagctaatggatgatgattatcggggaagactttgtaatcccctgaatccctttgtcagtcataaagagattgattctgtaaacaatgctgtggtaatgccagcccatggtccagccaaaagcatggaatatgatgccgttgcaactacacattcctggattcccaagaggaatcgttctattctcaacacaagccaaaggggaattcttgaggatgaacagatgtaccagaagtgctgcaatctattcgagaaatttttccctagcagttcatataggagaccggttggaatttctagcatggtggaggccatggtgtctagggcccggattgatgccagggtcgacttcgagtctggacggatcaagaaagaagagttctctgagatcatgaagatctgttccaccattgaagaactcagacggcaaaaataa

>H1N1_PB1_KT180386_A/Singapore/DMS120/2010

atggatgtcaatccgactctacttttcctaaaaattccagcgcaaaatgccataagcaccacattcccttatactggagatcctccatacagccatggaacaggaacaggatacaccatggacacagtaaacagaacacaccaatactcagaaaagggaaagtggacgacaaacacagagactggtgcaccccagctcaacccgattgatggaccactacctgaggataatgaaccaagtgggtatgcacaaacagactgtgttctggaggctatggctttccttgaagaatcccacccaggaatatttgagaattcatgccttgaaacaatggaagttgttcaacaaacaagggtagataaactaactcaaggtcgccagacttatgattggacattaaatagaaatcaaccggcagcaactgcattggccaacaccatagaagtctttagatcgaatggtctaacagctaatgagtcaggaaggctaatagatttcttaaaggatgtaatggaatcaatgaacaaagaggaaatagagataacaacccactttcaaagaaaaaggagagtaagagacaacatgaccaagaagatggtcacgcaaagaacaatagggaagaaaaaacaaagactgaagaagagaggctatctaataagagcactgacattaaatacgatgaccaaagatgcagagagaggcaagttaaaaagaagggctatcgcaacacctgggatgcagattagaggtttcgtatactttgttgaaactttagctaggagcatttgcgaaaagcttgaacagtctgggctcccagtagggggcaatgaaaagaaagccaaactggcaaatgttgtgagaaagatgatgactaattcacaagacacagagatttctttcacaatcactggggacaacactaagtggaatgaaaatcaaaatcctcgaatgttcctggcaatgattacatatatcaccagaaatcaacccgagtggttcagaaacatcctgagcatggcacccataatgttctcaaacaaaatggcaagactagggaaagggtacatgttcgagagtaaaagaatgaagattcgaacacaaataccagcagaaatgctagcaagcattgacctgaagtacttcaatgaatcaacaaagaaaaaaattgagaaaataaggcctcttctaatagatggcacagcatcactgagtcctgggatgatgatgggcatgttcaacatgctaagtacggtcttgggagtctcgatactgaatcttggacaaaagaaatacaccaagacaacatactggtgggatgggctccaatcatccgacgattttgctctcatagtgaatgcaccaaaccatgagggaatacaagcaggagtggacagattctacaggacctgcaagttagtgggaatcaacatgagcaaaaagaagtcctatataaataagacagggacatttgaattcacaagctttttttatcgctatggatttgtggctaattttagcatggagctacccagctttggagtgtctggagtaaatgaatcagctgacatgagtattggagtaacagtgataaagaacaacatgataaacaatgaccttggacctgcaacggcccagatggctcttcaattgttcatcaaagactacagatacacatataggtgccataggggagacacacaaattcagacgagaagatcatttgagttaaagaagctgtgggatcaaacccaatcaaaggtagggctattagtatcagatggaggaccaaacttatacaatatacggaatcttcacattcctgaagtctgcttaaaatgggagctaatggatgatgattatcggggaagactttgtaatcccctgaatccctttgtcagtcataaagagattgattctgtaaacaatgctgtggtaatgccagcccatggtccagccaaaagcatggaatatgatgcagttgcaactacacattcctggattcccaagaggaatcgttctattctcaacacaagccaaaggggaattcttgaggatgaacagatgtaccagaagtgctgcaatctattcgagaaatttttccctagcagttcatataggagaccggttggaatttctagcatggtggaggccatggtgtctagggcccggattgatgccagggtcgacttcgagtctggacggatcaagaaagaagagttctctgagatcatgaagatctgttccaccattgaagaactcagacggcaaaaataa

>H1N1_PB1_KT180345_A/Singapore/DMS106/2010

atggatgtcaatccgactctacttttcctaaaaattccagcgcaaaatgccataagcaccacattcccttatactggagatcctccatacagccatggaacaggaacaggatacaccatggacacagtaaacagaacacaccaatactcagaaaagggaaagtggacgacaaacacagagactggtgcaccccagctcaacccgattgatggaccactacctgaggataatgaaccaagtgggtatgcacaaacagactgtgttctagaggctatggctttccttgaagaatcccacccaggaatatttgagaattcatgccttgaaacaatggaagttgttcaacaaacaagggtagacaaactaactcaaggtcgccagacttatgattggacattaaacagaaatcaaccggcagcaactgcattggccaacaccatagaagtctttagatcgaatggcctaacagctaatgagtcaggaaggctaatagatttcttaaaggatgtaatggaatcaatgaacaaagaggaaatagagataacaacccactttcaaagaaaaaggagagtaagagacaacatgaccaagaagatggtcacgcaaagaacaatagggaagaaaaaacaaagactgaataagagaggctatctaataagagcactgacattaaatacgatgaccaaagatgcagagagaggcaagttaaaaagaagggctatcgcaacacctgggatgcagattagaggtttcgtatactttgttgaagctttagctaggagcatttgcgaaaagcttgaacagtctgggctcccagtagggggcaatgaaaagaaggccaaactggcaaatgttgtgagaaagatgatgactaattcacaagacacagagatttctttcacaatcactggggacaacactaagtggaatgaaaatcaaaatcctcgaatgttcctggcaatgattacatatatcaccagaaatcaacccgagtggttcagaaacatcctgagcatggcacccataatgttctcaaacaaaatggcaagactagggaaagggtacatgttcgagagtaaaagaatgaagattcgaacacaaataccagcagaaatgctagcaagcattgacctgaagtacttcaatgaatcaacgaagaagaaaattgagaaaataaggcctcttctaatagatggcacagcatcactgagtcctgggatgatgatgggcatgttcaacatgctaagtacggtcttgggagtctcgatactgaatcttggacaaaagaaatacaccaagacaatatactggtgggatgggctccaatcatccgacgattttgctctcatagtgaatgcaccaaaccatgagggaatacaagcaggagtggacagattctacaggacctgcaagttagtgggaatcaacatgagcaaaaagaagtcctatataaataagacagggacatttgaattcacaagctttttttatcgctatggatttgtggctaattttagcatggagctacccagctttggagtgtctggagtaaatgaatcagctgacatgagtattggagtaacagtgataaagaacaacatgataaacaatgaccttggacctgcaacggcccagatggctcttcaattgttcatcaaagactacagatacacatataggtgccataggggagacacacaaattcagacgagaagatcatttgagttaaagaagctgtgggatcaaacccaatcaaaggtagggctattagtatcagatggaggaccaaacttatacaatatacggaaccttcacattcctgaagtctgcttaaaatgggagctaatggatgatgattatcggggaagactttgtaatcccctgaatccctttgtcagtcataaagagattgattctgtaaacaatgctgtggtaatgccagcccatggtccagtcaaaagcatggaatatgatgccgttgcaactacacattcctggattcccaagaggaatcgttctattctcaacacaagccaaaggggaattcttgaggatgaacagatgtaccagaagtgctgcaatctattcgagaaatttttccctagcagttcatataggagaccggttggaatttctagcatggtggaggccatggtgtctagggcccggattgatgccagggtcgacttcgagtctggacggatcaagaaagaagagttctctgagatcatgaagatctgttccaccattgaagaactcagacggcaaaaataa

>H1N1_PB1_KT274654_A/Louisiana/34/2013

atgggtgtcaatccgactctacttttcctaaaaattccagcacaaaatgccataagcaccacattcccttatactggagatcctccatacagccatggaacaggaacaggatacaccatggacacagtaaacagaacacaccaatactcagaaaaggggaagtggacaacaaacacagagactggtgcaccccagctcaacccgattgatggaccactacctgaggataatgaaccaagtgggtatgcacaaacagactgtgttctagaggctatggctttccttgaagaatcccacccaggaatatttgagaattcatgccttgaaacaatggaaattgttcaacaaacaagggtagataaactgactcaaggtcgccagacttatgattggacattaaacagaaatcaaccggcagcaactgcattggccaacaccatagaagtctttagatcgaatgacctaacagctaacgagtcaggaaggctaatagatttcttaaaggatgtaatggaatcaatgaacaaagaggaaatagagataacaacccactttcaaagaaaaaggagagtaagagacaacatgaccaagaagatggtcactcaaagaacaatagggaagaaaaaacagagactgaataagagaggctatctaataagagcactgacattaaatacgatgaccaaagatgcagagagaggcaagttaaaaagaagggctatcgcaacacctgggatgcagattagaggtttcgtatactttgttgaaactttagctaggagcatttgcgaaaagcttgaacagtctgggctcccagtagggggcaatgaaaagaaggccaaattggcaaatgttgtgagaaagatgatgactaattcacaagatacagagatttctttcacaatcactggggacaacactaagtggaatgaaaatcaaaatcctcgaatgttcctggcgatgattacatatatcaccagaaatcaacccgagtggtttagaaacatcctgagcatggcacccataatgttctcaaataaaatggcaagactagggaaagggtacatgttcgagagtaaaagaatgaagattcgaacacaaataccagcagaaatgctagcaagcattgatctgaagtacttcaatgaatcaacaaagaagaaaattgagaaaataaggcctcttttaatggatggcacagcatcactgagtcctgggatgatgatgggcatgttcaacatgctaagtacggtcttgggagtctcgatactgaatcttggacaaaagaaatacaccaagacaacatactggtgggatgggctccaatcatccgacgattttgctctcatagtgaatgcaccaaaccatgaaggaatacaagcaggagtggacagattctacaggacctgcaagttagtgggaatcaacatgagcaaaaagaagtcctatataaataagacagggacatttgaattcacaagctttttttatcgctatggatttgtggctaattttagcatggagctacccagctttggagtgtctggagtaaatgaatcagctgacatgagtattggagtaacagtgataaagaacaacatgataaacaatgacctcggacctgcaacggcccagatggctcttcaattgttcatcaaagactacagatacacatataggtgccataggggagacacacaaattcagacgagaagatcatttgagttaaagaagctatgggatcaaacccaatcaaaggtagggctattagtatcagatggaggaccaaacttatacaacatacggaatcttcacattcctgaagtctgcttaaaatgggagctaatggatgatgattatcggggaagactttgtaatcccctgaatccctttgtgagtcataaggagattgattctgtaaacaatgctgtggtaatgccagcccatggtccagccaaaagcatggaatatgatgccgttgcaactacacattcctggattcccaagaggaatcgttctattctcaacacaagccaaaggggaattcttgaggatgaacagatgtaccagaagtgttgcaatctattcgagaaatttttccctagcagttcatataggagaccggttggaatttctagcatggtggaggccatggtgtctagggcccggattgatgccagggtcgactttgagtctggacggatcaagaaagaagagttctctgagatcatgaagatctgttccaccattgaagaactcagacggcaaaaataa

>H1N1_PB1_KT836613_A/Hawaii/64/2014

atggatgtcaatccgactctacttttcctaaaaattccagcacaaaatgccataagcaccacattcccttatactggagatcctccatacagccatggaacaggaacaggatacaccatggacacagtaaacagaacacaccaatactcagaaaaggggaagtggacaacaaacacagagactggtgcaccccagctcaacccgattgatggaccactacctgaggataatgaaccaagtgggtatgcacagacagactgtgtcctagaggctatggctttccttgaagaatcccacccaggaatatttgagaattcatgccttgaaacaatggaagttgttcaacaaacaagggtagataaactgactcaaggtcgccagacttatgattggacattaaacagaaatcaaccagcagcaactgcattggccaacaccatagaagtctttagatcgaatgacctaacagctaacgagtcaggaaggctaatagatttcttaaaggatgtaatggaatcaatggacaaagaggaaatagagataacaacccactttcaaagaaaaaggagagtaagagacaacatgaccaagaagatggtcacgcaaagaacaatagggaagaaaaaacagaaactgaataagagaggctatctaataagagcactgacattaaatacgatgaccaaagatgcagagagaggcaagttaaaaagaagggctatcgcaacacctgggatgcagattagaggtttcgtatactttgttgaaactttagctaggggcatttgcgaaaagcttgaacagtctgggctcccagtagggggcaatgaaaagaaggccaaattggcaaatgttgtgagaaagatgatgactaattcacaagatacagagatttctttcacaatcactggggacaacactaaatggaatgaaaatcaaaatcctcgaatgttcctggcaatgattacatatatcaccagaaatcaacccgagtggtttagaaacatcctgagcatggcacccataatgttctcaaataaaatggcaagactggggaaagggtacatgttcgagagtaaaagaatgaaaattcgaacacaaataccagcagaaatgctagcaagcattgatctgaagtacttcaatgaatcaacaaagaagaaaattgagaaaataaggcctcttttaatggatggcacagcatcactgagtcctgggatgatgatgggcatgttcaacatgctaagtacggtcttgggagtctcgatactgaatcttggacaaaagaaatacaccaagacaacatactggtgggatgggctccaatcatccgacgattttgctctcatagtgaatgcaccaaaccacgaaggaatacaagcaggagtggacagattctacaggacctgcaagttagtgggaatcaacatgagcaaaaagaagtcttatataaataagacagggacatttgaattcacaagctttttttatcgctatggatttgtggctaattttagcatggagctacccagctttggagtgtctggagtaaatgaatcagctgacatgagtattggagtaacagtgataaagaacaacatgataaacaatgaccttggacctgcaacggcccagatggctcttcaattgttcatcaaagactacagatacacatataggtgccacaggggagacacacaaattcagacgagaagatcatttgagttaaagaagctatgggatcaaacccaatcaaaggtagggctattagtatcagatggaggaccaaacttatacaacatacggaatcttcacattcctgaagtctgcttaaaatgggagctaatggatgatgattatcggggaagactttgtaatcccctgaatccctttgtgagtcataaggagattgattctgtaaacaatgccgtggtaatgccagcccatggtccagccaaaagcatggaatatgatgccgttgcaactacacattcctggattcccaagaggaatcgttctatcctcaacacaagccaaaggggaattcttgaggatgaacagatgtaccagaagtgttgcaatctattcgagaaatttttccctagcagttcatataggagaccggttggaatttctagcatggtggaggccatggtgtctagggcccggattgatgccagggtcgactttgagtctggacggatcaagaaagaggagttctctgagatcatgaagatctgttccaccattgaagaactcagacggcaaaaataa

>H1N1_PB1_KT836791_A/Washington/23/2015

atggatgtcaatccgactctacttttcctaaaaattccagcacaaaatgccataagcaccacattcccttatactggagatcctccatacagccatggaacaggaacaggatacaccatggacacagtaaacagaacacaccaatactcagaaaaaggaaagtggacaacaaacacagagactggtgcaccccagctcaacccgattgacggaccactacctgaagataatgaaccaagtgggtatgcacaaacagactgtgttctagaggctatggctttccttgaagaatcccatccaggaatatttgagaattcatgccttgaaacaatggaagttgttcaacaaacaagagtagataaactgactcaaggtcgccagacttatgattggacattaaacagaaatcaaccggcagcaactgcattggccaacaccatagaagtctttagatcgaatgacctaacagctaacgagtcaggaaggctaatagatttcttaaaggatgtgatggaatcaatgaacaaagaggaaatagagataacaacccactttcaaagaaaaaggagagtaagagacaacatgaccaagaagatggtcacgcaaagaacaatagggaagaaaaaacagagactgaataagagaggctatctaataagagcactgacattaaatacgatgaccaaagatgcagagagaggcaagttaaaaagaagggctatcgcaacacctgggatgcagattagaggtttcgtatactttgttgaaactttagctaggagcatttgcgaaaagcttgaacagtctgggctcccagtagggggcaatgaaaagaaggccaaattggcaaatgttgtgagaaagatgatgactaattcacaagatacagagatttctttcacaatcactggggacaacactaagtggaatgaaaatcaaaatcctcgaatgttcctggcgatgattacatatatcaccagaaatcaacccgagtggtttagaaacatcctgagcatggcacccataatgttctcaaataaaatggcaagactaggaaaagggtacatgttcgagagtaaaagaatgaagattcgaacacaaataccggcagaaatgctagcaagcattgatctgaagtatttcaatgaatcaacaaagaagaaaattgagaaaataaggcctcttttaatggatggcacagcatcactgagtcctgggatgatgatgggcatgttcaacatgctaagtacggtcttgggagtctcgatactgaatcttggacaaaagaaatacaccaagacaacatactggtgggatgggctccaatcatccgacgattttgctctcatagtgaatgcaycaaaccatgaaggaatacaagcaggagtggacagattctacaggacctgcaagttagtgggaatcaacatgagcaaaaagaagtcctatataaataagacagggacatttgaattcacaagctttttttatcgctatggatttgtggctaattttagcatggagctacccagctttggagtgtctggagtaaatgaatcagctgacatgagtattggagtaacagtgataaagaacaacatgataaacaatgaccttggacctgcaacggctcagatggctcttcaattgttcatcaaagactacagatacacatataggtgccataggggagacacacaaattcaaacgagaagatcatttgagttgaagaagctatgggatcaaacccaatcaaaggtagggctattagtatcagatggaggaccaaacttatacaacatacggaatcttcacattcctgaagtctgcttaaaatgggagctaatggatgatgattatcggggaagactttgtaatcccctgaatccctttgtgagtcataaggagattgattctgtaaacaatgctgtggtaatgccagcccatggtccagccaaaagcatggaatatgatgccgttgcaactacacattcctggattcccaagaggaatcgttctattctcaacacaagccaaaggggaattcttgaggatgaacagatgtaccagaagtgttgcaatttattcgagaaatttttccctagcagttcatataggagaccggttggaatttctagcatggtggaggccatggtgtctagggcccggattgatgccagagtcgactttgagtctggacggatcaagaaagaagagttctctgagatcatgaagatctgttccaccattgaagaactcagacggcaaaaataa

>H1N1_PB1_KT836825_A/California/92/2014

atggatgtcaatccgactctacttttcctaaaaattccagcacaaaatgccataagcaccacattcccttatactggagatcctccatacagccatggaacaggaacaggatacaccatggacacagtaaacagaacacaccagtactcagaaaaggggaagtggacaacaaacacagagactggtgcaccccagctcaacccgattgatggaccactacctgaggataatgaaccaagtgggtatgcacagacagactgtgtcctagaggctatggctttccttgaagaatcccacccaggaatatttgagaattcatgccttgaaacaatggaagttgttcaacaaacaagggtagataaactgactcaaggtcgccagacttatgattggacattaaacagaaatcaaccagcagcaactgcattggccaacaccatagaagtctttagatcgaatgacctaacagctaacgagtcagggaggctaatagatttcttaaaggatgtaatggaatcaatgaacaaagaggaaatagagataacaacccactttcaaagaaaaaggagagtaagagacaacatgaccaagaagatggtcacgcaaagaacaatagggaagaaaaaacagagactgaataagagaggctatctaataagagcactgacattaaatacgatgaccaaagatgcagagagaggcaagttaaaaagaagggctatcgcaacacctgggatgcagattagaggtttcgtatactttgttgaaactttagctaggagcatttgcgaaaagcttgaacagtctgggctcccagtagggggcaatgaaaagaaggccaaattggcaaatgttgtgagaaagatgatgactaattcacaagatacagagatttctttcacaatcactggggacaacactaaatggaatgaaaatcaaaatcctcgaatgttcctggcaatgattacatatatcaccagaaatcaacccgagtggtttagaaacatcctgagcatggcacccataatgttctcaaataaaatggcaagactggggaaagggtacatgttcgagagtaaaagaatgaaaattcgaacacaaataccagcagaaatgctagcaaacattgatctgaagtacttcaatgaatcaacaaagaagaaaattgagaaaataaggcctcttttaatggatggcacagcatcactgagtcctgggatgatgatgggcatgttcaacatgctaagtacggtcttgggagtctcgatactgaatcttggacaaaagaaatacaccaagacaacatactggtgggatgggctccaatcatccgacgattttgctctcatagtgaatgcaccaaaccatgaaggaatacaagcaggagtggacagattctacaggacctgcaagttagtgggaatcaacatgagcaaaaagaagtcttatataaataagacagggacatttgaattcacaagctttttttatcgctatggatttgtggctaattttagcatggagctacccagctttggagtgtctggagtaaatgaatcagctgacatgagtattggagtaacagtgataaagaacaacatgataaacaatgaccttggacctgcaacggcccagatggctcttcaattgttcatcaaagactacagatacacatataggtgccacaagggagacacacaaattcagacgagaagatcatttgagttaaagaagctatgggatcaaacccaatcaaaggtagggctattagtatcagatggaggaccaaacttatacaacatacggaatcttcacattcctgaagtctgcttaaaatgggagctaatggatgatgattatcggggaagactttgtaatcccctgaatccctttgtgaatcataaggagattgattctgtaaacaatgccgtggtaatgccagcccatggtccagccaaaaacatggaatatgatgccgttgcaactacacattcctggattcccaagaggaatcgttctatcctcaacacaagccaaaggggaattcttgaggatgaacagatgtaccagaagtgttgcaatctattcgagaaatttttccctagcagttcatataggagaccggttggaatttctagcatggtggaggccatggtgtctagggcccggattgatgccagggtcgactttgagtctggacggatcaagaaagaagagttctctgagatcatgaagatctgttccaccattgaagaactcagacggcaaaaataa

>H1N1_PB1_KU589298_A/Indiana/30/2015

atggatgtcaatccgactctacttttcctaaaaattccagcacaaaatgccataagcaccacattcccttatactggagatcctccatacagccatggaacaggaacaggatacaccatggacacagtaaacagaacacaccaatactcagaaaaggggaagtggacaacaaacacagagactggtgcaccccagctcaacccgattgacggaccactacctgaagataatgaaccaagtgggtatgcacaaacagactgtgttctagaggctatggcttttcttgaagaatcccatccaggaatatttgagaattcatgccttgaaacaatggaagttgttcaacaaacaagggtagataaactgactcaaggtcgccagacttatgattggacattaaacagaaatcaaccggcagcaactgcattggccaacaccatagaagtttttagatcgaatgacctaacagctaacgagtcaggaaggctaatagatttcttaaaggatgtgatggaatcaatgaacaaagaggaaatagagataacaacccactttcaaagaaaaaggaaagtaagagacaacatgaccaagaagatggtcacgcaaagaacaatagggaagaaaaaacagagactgaataagagaggctatctaataagagcactgacattaaatacgatgaccaaagatgcagagagaggcaagttaaaaagaagggctatcgcaacacctgggatgcagattagaggtttcgtatactttgttgaaactttagctaggagcatttgcgaaaagcttgaacagtctgggctcccagtagggggcaatgaaaagaaggccaaattggcaaatgttgtaagaaagatgatgactaattcacaagatacagagatttctttcacaatcactggggacaacactaagtggaatgaaaatcaaaatcctcgaatgttcctggcgatgattacatatatcaccagaaatcaacccgagtggtttagaaacatcctgagcatggcacccataatgttctcaaataaaatggcaagactaggaaaagggtacatgttcgagagtaaaagaatgaagattcgaacacaaataccagcagaaatgctagcaagcattgatctgaagtatttcaatgaatcaacaaagaagaaaattgagaaaataaggcctcttttaatggatggcacagcatcactgagtcctgggatgatgatgggcatgttcaacatgctaagtacggtcttgggagtctcgatactgaatcttggacaaaagaaatacaccaagacaacatactggtgggatgggctccaatcatccgacgattttgctctcatagtgaatgcaccaaaccatgaaggaatacaagcaggagtggacagattctacaggacctgcaagttagtgggaatcaacatgagcaaaaagaagtcctatataaataagacagggacatttgaattcacaagctttttttatcgctatggatttgtggctaattttagcatggagctacccagctttggagtgtctggagtaaatgaatcagctgacatgagtattggagtaacagtgataaagaacaacatgataaacaatgaccttggacctgcaacggctcagatggctcttcaattgttcatcaaagactacagatacacatataggtgccataggggagacacacaaattcagacgagaagatcatttgagttgaagaagctatgggatcaaacccaatcaaaggtagggctattagtatcagatggaggaccaaacttatacaacatacggaatcttcacattcctgaagtctgcttaaaatgggagctaatggatgatgattatcggggaagactttgtaatcccctgaatccctttgtgagtcataaggagattgattctgtaaacaatgctatggtaatgccagcccatggcccagccaaaagcatggaatatgatgccgttgctactacacattcctggatccccaagaggaatcgttctattctcaacacaagccaaaggggaattcttgaggatgaacagatgtaccagaagtgttgcaatttattcgagaaatttttccctagcagttcatataggagaccggttggaatttctagcatggtggaggccatggtgtctagggcccggattgatgccagagtcgactttgagtctggacggatcaagaaagaagagttctctgagatcatgaagatctgttccaccattgaagaactcagacggcaaaaataa

>H1N1_PB1_KX028877_A/DC/4/2016

atggatgtcaatccgactctacttttcctaaaaattccagcacaaaatgccataagcaccacattcccttatactggagatcctccatacagccatggaacaggaacaggatacaccatggacacagtaaacagaacacaccaatactcagaaaaggggaagtggacaacaaacacagagactggtgcaccccagctcaacccgattgacggaccactacctgaagataatgaaccaagtgggtatgcacaaacagactgtgttctagaggctatggcttttcttgaagaatcccatccaggaatatttgagaattcatgccttgaaacaatggaagttgttcaacaaacaagggtagataaactgactcaaggtcgccagacttatgattggacattaaacagaaatcaaccggcagcaactgcattggccaacaccatagaagtttttagatcgaatgacctaacagctaacgagtcaggaaggctaatagatttcttaaaggatgtgatggaatcaatgaacaaagaggaaatagagataacaacccactttcaaagaaaaaggagagtaagagacaacatgaccaagaagatggtcacgcaaagaacaatagggaagaaaaaacagagactgaataagagaggctatctaataagagcactgacattaaatacgatgaccaaagatgcagagagaggcaagttaaaaagaagggctatcgcaacacctgggatgcagattagaggtttcgtatactttgttgaaactttagctaggagcatttgcgaaaagcttgaacagtctgggctcccagtagggggcaatgaaaaaaaggccaaattggcaaatgttgtgagaaagatgatgactaattcacaagatacagagatttctttcacaatcactggggacaacactaagtggaatgaaaatcaaaatcctcgaatgttcctggcgatgattacatatatcaccagaaatcaacccgagtggtttagaaacatcctgagcatggcacccataatgttctcaaataaaatggcaagactaggaaaagggtacatgttcgagagtaaaagaatgaagattcgaacacaaataccagcagaaatgctagcaagcattgatctgaagtatttcaatgaatcaacaaagaagaaaattgagaaaataaggcctcttttaatggatggcacagcatcactgagtcctgggatgatgatgggcatgttcaacatgctaagtacggtcttgggagtctcgatactgaatcttggacaaaagaaatacaccaagacaacatactggtgggatgggctccaatcatccgacgattttgctctcatagtgaatgcaccaaaccatgaaggaatacaagcaggagtggacagattctacaggacctgcaagttagtgggaatcaacatgagcaaaaagaagtcctatataaataagacagggacatttgaattcacaagctttttttatcgctatggatttgtggctaattttagcatggagctacccagctttggagtgtctggagtaaatgaatcagctgacatgagtattggagtaacagtgataaagaacaacatgataaacaatgaccttggacctgcaacggctcagatggctcttcaattgttcatcaaagactacagatacacatataggtgccataggggagacacacaaattcagacgagaagatcatttgagttgaagaagctatgggatcaaacccaatcaaaggtagggctattagtatcagatggaggaccaaacttatacaacatacggaatcttcacattcctgaagtctgcttaaaatgggagctaatggatgatgattatcggggaagactttgtaatcccctgaatccctttgtgagtcataaggagattgattctgtaaacaatgctgtggtaatgccagcccatggcccagccaaaagcatggaatatgatgccgttgctactacacattcctggattcccaagaggaatcgttctattctcaacacaagccaaaggggaattcttgaggatgaacagatgtaccagaagtgttgcaatttattcgagaaatttttccctagcagttcatataggagaccggttggaatttctagcatggtggaggccatggtgtctagggcccggattgatgccagagtcgactttgagtctggacggatcaagaaagaagagttctctgagatcatgaagatctgttccaccattgaagaactcagacggcaaaaataa

>H1N1_PB1_KX004120_A/Pennsylvania/41/2015

atggatgtcaatccgactctactttttctaaaaattccagcacaaaatgccataagcaccacattcccttatactggagatcctccatacagccatggaacaggaacaggatacaccatggacacagtaaacagaacacaccaatactcagaaaaggggaagtggacaacaaacacagagactggtgcaccccagctcaacccgattgacggaccactacctgaagataatgaaccaagtgggtatgcacaaacagactgtgttctagaggctatggctttccttgaagaatcccatccaggaatatttgagaattcatgccttgaaacaatggaagttgttcaacaaacaagggtagataaactgactcaaggtcgccagacttatgattggacattaaacagaaatcaaccggcagcaactgcattggccaacaccatagaagtctttagatcgaatgacctaacagctaacgagtcaggaaggctaatagatttcttaaaggatgtgatggaatcaatgaacaaagaggaaatagagataacaacccactttcaaagaaaaaggagagtaagagacaacatgaccaagaagatggtcacgcagagaactatagggaagaaaaaacagagactgaataagagaggctatctaataagagcactgacattaaatacgatgaccaaagatgcagagagaggcaagttaaaaagaagggctatcgcaacacctgggatgcagattagaggtttcgtatactttgttgaaactttagctaggagcatttgcgaaaagcttgaacagtctggactcccagtagggggcaatgaaaagaaggccaaattggcaaatgttgtgagaaagatgatgactaattcacaagatacagagatttctttcacaatcactggggacaacactaagtggaatgaaaatcaaaatcctcgaatgttcctggcgatgattacatatatcaccagaaatcaacccgagtggtttagaaacatcctgagcatggcacccataatgttctcaaataaaatggcaagactaggaaaagggtacatgttcgagagtaaaagaatgaagattcgaacacaaataccagcagaaatgctagcaagcattgatctgaagtatttcaatgaatcaacaaagaagaaaattgagaaaataaggcctcttttaatggatggcacagcatcactgagtcctgggatgatgatgggcatgttcaacatgctaagtacggtcttgggagtttcgatactgaatcttggacaaaagaaatacaccaagacaacatactggtgggatgggctccaatcatccgacgattttgctctcatagtgaatgcaccaaaccatgaaggaatacaagcaggagtggacagattctacaggacctgcaagttagtgggaatcaacatgagcaaaaagaagtcctatataaataagacagggacatttgaattcacaagctttttttatcgctatggatttgtggctaattttagcatggagctacccagctttggagtgtctggagtaaatgaatcagctgacatgagtattggagtaacagtgataaagaacaacatgataaacaatgaccttggacctgcaacggctcagatggctcttcaattgttcatcaaagactacagatacacatataggtgccataggggagacacacaaattcagacgagaagatcatttgagttgaagaagctatgggatcaaacccaatcaaaggtagggctattagtatcagatggaggaccaaacttatacaacatacggaatcttcacattcctgaagtctgcttaaaatgggagctaatggatgatgattatcggggaagactttgtaatcccctgaatccctttgtgagtcataaggagattgattctgtaaacaatgctgtggtaatgccagcccatggtccagccaaaagcatggaatatgatgccgttgcaactacacattcctggattcccaagaggaatcgttctattctcaacacaagccaaaggggaatccttgaggatgaacagatgtaccagaagtgttgcaatttattcgagaaatttttccctagcagttcatataggagaccggttggaatttctagcatggtggaggccatggtgtctagggcccggattgatgccagagtcgactttgagtctggacggatcaagaaagaagagttctctgagatcatgaagatctgttccaccattgaagaactcagacggcaaaaataa

>H1N1_PB1_KX004152_A/Florida/88/2015

atggatgtcaatccgactctacttttcctaaaaattccagcacaaaatgccataagcaccacattcccttatactggagatcctccatatagccatggaacaggaacaggatacaccatggacacagtaaacagaacacaccaatactcagaaaaggggaagtggacaacaaacacagagactggtgcaccccagctcaacccgattgatggaccactacctgaggataatgaaccaagtgggtatgcacaaacagactgtgttctagaggctatggctttccttgaagaatcccacccaggaatatttgagaattcatgccttgaaacaatggaagttgttcaacaaacaagggtagataaactgactcaaggtcgccagacttatgattggacattaaacagaaatcaaccggcagcaactgcattggccaacaccatagaagtctttagatcgaatgacctaacagctaacgagtcaggaaggctaatagatttcttaaaggatgtaatggaatcaatgaacaaagaggaaatagagataacaacccactttcaaagaaaaaggagagtaagagacaacatgaccaagaagatggtcacgcaaagaacaatagggaagaaaaaacagagactgaataagagaggctatctaataagagcactgacattaaatacgatgaccaaagatgcagagagaggcaagttaaaaagaagggctatcgcaacacctgggatgcagattagaggtttcgtatactttgttgaaactttagctaggagcatttgcgaaaagcttgaacagtctgggctcccagtagggggcaatgaaaagaaggccaaattggcaaatgttgtgagaaagatgatgactaattcacaagatacagagatttctttcacaatcactggggacaacactaagtggaatgaaaatcaaaatcctcgaatgttcctggcgatgattacatatatcaccagaaatcaacccgagtggtttagaaacatcctgagcatggcacccataatgttctcaaataaaatggcaagactggggaaagggtacatgttcgagagtaaaagaatgaagattcgaacacaaataccagcagaaatgctagcaagcattgatctgaagtacttcaatgaatcaacaaagaagaaaattgagaaaataaggcctcttttaatggatggcacagcatcactgagtcctgggatgatgatgggcatgttcaacatgctaagtacggtattgggagtcgcgatactgaatcttgggcaaaagaaatacaccaagacaacatactggtgggatgggctccaatcatccgacgattttgctctcatagtgaatgcaccaaaccacgaaggaatacaagcaggagtggacaggttctacaggacctgcaagttagtgggaatcaacatgagcaaaaagaagtcctatataaataagacagggacatttgaattcacaagctttttttatcgctatggatttgtggctaattttagcatggagctacccagctttggagtgtctggagtaaatgaatcagctgacatgagtattggagtaacagtgataaagaacaacatgataaacaatgaccttggacctgcaacggcccagatggctcttcaattgttcatcaaagactacagatacacatataggtgccataggggagacacacaaattcagacgagaagatcttttgagttaaagaagctatgggatcaaacccaatcaaaggtagggctattagtatcagatggagggccaaacttatacaacatacggaatcttcacattcctgaagtctgcttaaaatgggagctaatggatgatgattatcggggaagactttgtaatcccctgaatccctttgtgagtcataaggagattgattctgtaaacaatgccgtggtaatgccagcccatggtccagccaaaagcatggaatatgatgccgttgcaactacacattcctggattcccaagaggaatcgttctattctcaacacaagccaaaggggaattcttgaggatgaacagatgtaccagaagtgttgcaatctattcgagaaatttttccctagcagttcatataggagaccgattggaatttctagcatggtggaggccatggtgtctagggcccggattgatgccagagtcgactttgagtctggacggatcaagaaagaagagttctctgagatcatgaagatctgttccaccattgaagaactcagacggcaaaaataa

>H1N1_PB1_KX005080_A/Pennsylvania/06/2016

atggatgtcaatccgactctacttttcctaaaaattccagcacaaaatgccataagcaccacattcccttatactggagatcctccatacagccatggaacaggaacaggatacaccatggacacagtaaacagaacacaccaatactcagaaaaggggaagtggacaacaaacacagagactggtgcaccccagctcaacccgattgacggaccactacctgaagataatgaaccaagtgggtatgcacaaacagactgtgttctagaggctatggcttttcttgaagaatcccatccaggaatatttgagaattcatgccttgaaacaatggaagttgttcaacaaacaagggtagataaactgactcaaggtcgccagacttatgattggacattaaacagaaatcaaccggcagcaactgcattggccaacaccatagaagtttttagatcgaatgacctaacagctaacgagtcaggaaggctaatagatttcttaaaggatgtgatggaatcaatgaacaaagaggaaataaagataacaacccactttcaaagaaaaaggagagtaagagacaacatgaccaagaagatggtaacgcaaagaacaatagggaagaaaaaacagagactgaacaagagaggctatctaataagagcactgacattaaatacgatgaccaaagatgcagagagaggcaagttaaaaagaagggctatcgcaacacctgggatgcagattagaggtttcgtatactttgttgaaactttagctaggagcatttgcgaaaagcttgaacagtctgggctcccagtagggggcaatgaaaagaaggccaaattggcaaatgttgtgagaaagatgatgactaattcacaagatacagagatttctttcacaatcactggggacaacactaagtggaatgaaaatcaaaatcctcgaatgttcctggcgatgattacatatatcaccagaaatcaacccgagtggtttagaaacatcctgagcatggcacccataatgttctcaaataaaatggcaagactaggaaaagggtacatgttcgagagtaaaagaatgaagattcgaacacaaataccagcagaaatgctagcaagcattgatctgaagtatttcaatgaatcaacaaagaagaaaattgagaaaataaggcctcttttaatggatggcacagcatcactgagtcctgggatgatgatgggcatgttcaacatgctaagtacggtcttgggagtctcgatactgaatcttggacaaaagaaatacaccaagacaacatactggtgggatgggctccaatcatccgacgattttgctctcatagtgaatgcaccaaaccatgaaggaatacaagcaggagtggacagattctacaggacctgcaagttagtgggaatcaacatgagcaaaaagaagtcctatataaataagacagggacatttgaattcacaagctttttttatcgctatggatttgtggctaattttagcatggagctacccagctttggagtgtctggagtaaatgaatcagctgacatgagtattggagtaacagtgataaagaacaacatgataaacaatgaccttggacctgcaacggctcagatggctcttcaattgttcatcaaagactacagatacacatataggtgccataggggagacacacaaattcagacgagaagatcatttgagttgaagaagctatgggatcaaacccaatcaaaggtagggctattagtatcagatggaggaccaaacttatacaacatacggaatctccacattcctgaagtctgcttaaaatgggagctaatggatgatgattatcggggaagactttgtaatcccctgaatccctttgtgagtcataaggagattgactctgtaaacaatgctgtggtaatgccagcccatggcccagccaaaagcatggaatatgatgccgttgctactacacattcctggattcccaagaggaatcgttctattctcaacacaagccaaaggggaattcttgaggatgaacagatgtaccagaagtgttgcaatttattcgagaaatttttccctagcagttcatataggagaccggttggaatttctagcatggtggaggccatggtgtctagggcccggattgatgccagagtcgactttgagtctggacggataaagaaagaagagttctctgagatcatgaagatctgttccaccattgaagaactcagacggcaaaaataa

>H1N1_PB1_KX005488_A/Rhode Island/02/2016

atggatgtcaatccgactctacttttcctaaaaattccagcacaaaatgccataagcaccacattcccttatactggagatcctccatacagccatggaacaggaacaggatacaccatggacacagtaaacagaacacaccaatactcagaaaaggggaagtggacaacaaacacagagactggtgcaccccagctcaacccgattgacggaccactacctgaagataatgaaccaagtgggtatgcacaaacagactgtgttctagaggctatggcttttcttgaagaatcccatccaggaatatttgagaattcatgccttgaaacaatggaagttgttcaacaaacaagggtagataaactgactcaaggtcgccagacttatgactggacattaaacagaaatcaaccggcagcaactgcattggccaacaccatagaagtttttagatcgaatgacctaacagctaacgagtcaggaaggctaatagatttcttaaaggatgtgatggaatcaatgaacaaagaggaaatagagataacaacccactttcaaagaaaaaggagagtaagagacaacatgaccaagaagatggtcacgcaaagaacaatagggaagaaaaaacagagactgaataagagaggctatctaataagagcactgacattaaatacgatgaccaaagatgcagagagaggcaagttaaaaagaagggctatcgcaacacctgggatgcagattagaggtttcgtatactttgttgaaactttagctaggagcatttgcgaaaagcttgaacagtctgggctcccagtagggggcaatgaaaagaaggccaaattggcaaatgttgtgagaaagatgatgactaattcacaagatacagagatttctttcacaatcactggggacaacactaagtggaatgaaaatcaaaatcctcgaatgttcctggcgatgattacatatatcaccagaaatcaacccgagtggtttagaaacatcctgagcatggcacccataatgttctcaaataaaatggcaagactaggaaaagggtacatgttcgagagtaaaagaatgaagattcgaacacaaataccagcagaaatgctagcaagcattgatctgaagtatttcaatgaatcaacaaagaagaaaattgagaaaataaggcctcttttaatggatggcacagcatcactgagtcctgggatgatgatgggcatgttcaacatgctaagtacggtcttgggagtctcgatactgaatcttggacaaaagaaatacaccaagacaacatactggtgggatgggctccaatcatccgacgattttgctctcatagtgaatgcaccaaaccatgaaggaatacaagcaggagtggacagattctacaggacctgcaagttagtgggaatcaacatgagcaaaaagaagtcctatataaataagacagggacatttgaattcacaagctttttttatcgctatggatttgtggctaattttagcatggagctacccagctttggagtgtctggagtaaatgaatcagctgacatgagtattggagtaacagtgataaagaacaacatgataaacaatgaccttggacctgcaacggctcagatggctcttcaattgttcatcaaagactacagatacacatataggtgccataggggagacacacaaattcagacgagaagatcatttgagttgaagaagctatgggatcaaacccaatcaaaggtagggctattagtatcagatggaggaccaaacttatacaacatacggaatcttcacattcctgaagtctgcttaaaatgggagctaatggatgatgattatcggggaagactttgtaatcccctgaatccctttgtgagtcataaggagattgattctgtaaacaatgctgtggtaatgccagcccatggcccagccaaaagcatggaatatgatgccgttgctactacacattcctggattcccaagaggaatcgttctattctcaacacaagccaaaggggaattcttgaggatgaacagatgtaccagaagtgttgcaatttattcgagaaatttttccctagcagttcatataggagaccggttggaatttctagcatggtggaggccatggtgtctagggcccggattgatgccagagtcgactttgagtctggacggatcaagaaagaggagttctctgagatcatgaagatctgttccaccattgaagaactcagacggcaaaaataa

>H1N1_PB1_KX006016_A/Colorado/02/2016

atggatgtcaatccgactctacttttcctaaaaattccagcacaaaatgccataagcaccacattcccttatactggagatcctccatacagccatggaacaggaacaggatacaccatggacacagtaaacagaacacaccaatactcagaaaaggggaagtggacaacaaacacagagactggtgcaccccagctcaacccgattgacggaccactacctgaagataatgaaccaagtgggtatgcacaaacagactgtgttctagaggctatggcttttcttgaagaatcccatccaggaatatttgagaattcatgccttgaaacaatggaagttgttcaacaaacaagggtagataaactgactcaaggtcgccagacttatgattggacattaaacagaaatcaaccggcagcaactgcattggccaacaccatagaagtttttagatcgaatgacctaacagctaacgagtcaggaaggctaatagatttcttaaaggatgtgatggaatcaatgaacaaagaggaaatagagataacaacccactttcaaagaaaaaggagagtaagagacaacatgaccaagaagatggtcacgcaaagaacaatagggaagaaaaaacagagactgaacaagagaggctatctaataagagcactgacattaaatacgatgaccaaagatgcagagagaggcaagttaaaaagaagggctatcgcaacacctgggatgcagattagaggtttcgtatactttgttgaaactttagctaggagcatttgcgaaaagcttgaacagtctgggctcccagtagggggcaatgaaaagaaggccaaattggcaaatgttgtgagaaagatgatgactaattcacaagatacagagatttctttcacaatcactggggacaacactaagtggaatgaaaatcaaaatcctcgaatgttcctggcgatgattacatatatcaccagaaatcaacccgagtggtttagaaacatcctgagcatggcacccataatgttctcaaataaaatggcaagactaggaaaagggtacatgttcgagagtaaaagaatgaaaattcgaacacaaataccagcagaaatgctagcaagcattgatctgaagtatttcaatgaatcaacaaagaagaaaattgagaaaataaggcctcttttaatggatggcacagcatcactgagtcctgggatgatgatgggcatgttcaacatgctaagtacggtcttgggagtctcgatactgaatcttggacaaaagaaatacaccaagacaacatactggtgggatgggctccaatcatccgacgattttgctctcatagtgaatgcaccaaaccatgaaggaatacaagcaggagtggacagattctacaggacctgcaagttagtgggaatcaacatgagcaaaaagaagtcctatataaataagacagggacatttgaattcacaagctttttttatcgctatggatttgtggctaattttagcatggagctacccagctttggagtgtctggagtaaatgaatcagctgacatgagtattggagtaacagtgataaagaacaacatgataaacaatgaccttggacctgcaacggctcagatggctcttcaattgttcatcaaagactacagatacacatataggtgccataggggagacacacaaattcagacgagaagatcatttgagttgaagaagctatgggatcaaacccaatcaaaggtagggctattagtatcagatggaggaccaaacttatacaacatacggaatcttcacattcctgaagtctgcttaaaatgggagctaatggatgatgattatcggggaagactttgtaatcccctgaatccctttgtgagtcataaggagattgattctgtaaacaatgctgtggtaatgccagcccatggcccagccaaaagcatggaatatgatgccgttgctactacacattcctggattcccaagaggaatcgttctattctcaacacaagccaaaggggaattcttgaggatgaacagatgtaccagaagtgttgcaatttattcgagaaatttttccctagcagttcatataggagaccggttggaatttctagcatggtggaggccatggtgtctagggcccggattgatgccagagtcgactttgagtctggacggatcaataaagaagagttctctgagatcatgaagatctgttccaccattgaagaactcagacggcaaaaataa

>H1N1_PB1_KX006328_A/Minnesota/10/2016

atggatgtcaatccgactctacttttcctaaaaattccagcacaaaatgccataagcaccacattcccttatactggagatcctccatacagccatggaacaggaacaggatacaccatggacacagtaaacagaacacaccaatactcagaaaaggggaagtggacaacaaacacagagactggtgcaccccagctcaacccgattgacggaccactacctgaagataatgaaccaagtgggtatgcacaaacagactgtgttctagaggctatggcttttcttgaagaatcccatccaggaatatttgagaattcatgccttgaaacaatggaagttgttcaacaaacaagggtagataaactgactcaaggtcgccagacttatgattggacattaaacagaaatcaaccggcagcaactgcattggccaacaccatagaagtttttagatcgaatgacctaacagctaacgagtcaggaaggctaatagatttcttaaaggatgtgatggaatcaatgaacaaagaggaaatagagataacaacccactttcaaagaaaaaggagagtaagagacaacatgaccaagaagatggtcacgcaaagaacaatagggaagaaaaaacagagactgaataagagaggctatctaataagagcactgacattaaatacgatgaccaaagatgcagagagaggcaagttaaaaagaagggctatcgcaacacctgggatgcagattagaggtttcgtatactttgttgaaactttagctaggagcatttgcgaaaagcttgaacagtctgggctcccagtagggggcaatgaaaagaaggccaaattggcaaatgttgtgagaaagatgatgactaattcacaagatacagagatttctttcacaatcactggggacaacactaagtggaatgaaaatcaaaatcctcgaatgttcctggcgatgattacatatatcaccagaaatcaacccgagtggtttagaaacatcctgagcatggcacccataatgttctcaaataaaatggcaagactaggaaaagggtacatgttcgagagtaaaagaatgaagattcgaacacaaataccagcagaaatgctagcaagcattgatctgaagtatttcaatgaatcaacaaagaagaaaattgagaaaataaggcctcttttaatggatggcacagcatcactgagtcctgggatgatgatgggcatgttcaacatgctaagtacggtcttgggagtctcgatactgaatcttggacaaaagaaatacaccaagacaacatactggtgggatgggctccaatcatccgacgattttgctctcatagtgaatgcaccaaaccatgaaggaatacaagcaggagtggacagattctacaggacctgcaagttagtgggaatcaacatgagcaaaaagaagtcctatataaataagacagggacatttgaattcacaagctttttttatcgctatggatttgtggctaattttagcatggagctacccagctttggagtgtctggagtaaatgaatcagctgacatgagtattggagtaacagtgataaagaacaacatgataaacaatgaccttggacctgcaacggctcagatggctcttcaattgttcatcaaagactacagatacacatataggtgccataggggagacacacaaattcagacgagaagatcatttgagttgaagaagctatgggatcaaacccaatcaaaggtagggctattagtatcagatggaggaccaaacttatacaacatacggaatcttcacattcctgaagtctgcttaaaatgggagctaatggatgatgattatcggggaagactttgtaatcccctgaatccctttgtgagtcataaggagattgattctgtaaacaatgctgtggtaatgccagcccatggcccagccaaaagcatggaatatgatgccgttgctactacacattcctggattcccaagaggaatcgttctattctcaacacaagccaaaggggaattcttgaggatgaacagatgtaccagaagtgttgcaatttattcgagaaatttttccctagcagttcatataggagaccggttggaatttctagcatggtggaggccatggtgtctagggcccggattgatgccagagtcgactttgagtctggacggatcaagaaagaagagttctctgagatcatgaagatctgtttcaccattgaagaactcagacggcaaaaataa

>H1N1_PB1_KX406153_A/California/06/2016

atggatgtcaatccgactctacttttcctaaaaattccagcacaaaatgccataagcaccacattcccttatactggagatcctccatacagccatggaacaggaacaggatacaccatggacacagtaaacagaacacaccaatactcagaaaaggggaaatggacaacaaacacagagactggtgcaccccagctcaacccgattgacggaccactacctgaagataatgaaccaagtgggtatgcccaaacagactgtgttctagaggctatggcttttcttgaagaatcccatccaggaatatttgagaattcatgccttgaaacaatggaagttgttcaacaaacaagggtagataaactgactcaaggtcgccagacttatgattggacattaaacagaaatcaaccggcagcaactgcattggccaacaccatagaagtttttagatcgaatgacctaacagctaacgagtcaggaaggctaatagatttcttaaaggatgtgatggaatcaatgaacaaagaggaaatagagataacaacccactttcaaagaaaaaggagagtaagagacaacatgaccaagaagatggtcacgcaaagaacaatagggaagaaaaaacagagactgaataagagaggctatctaataagagcactgacattaaatacgatgaccaaagatgcagagagaggcaagttaaaaagaagggctatcgcaacacctgggatgcagattagaggtttcgtatactttgttgaaactttagctaggagcatttgcgaaaagcttgaacagtctgggctcccagtagggggcaatgaaaagaaggccaaattggcaaatgttgtgagaaagatgatgactaattcacaagatacagagatttctttcacaatcactggggacaacactaagtggaatgaaaatcaaaatcctcgaatgttcctggcgatgattacatatatcaccagaaatcaacccgagtggtttagaaacatcctgagcatggcacccataatgttctcaaataaaatggcaagactaggaaaagggtacatgttcgagagtaaaagaatgaagattcgaacacaaataccagcagaaatgctagcaagcattgatctgaagtatttcaatgaatcaacaaagaagaaaattgagaaaataaggcctcttttaatggatggcacagcatcactgagtcctgggatgatgatgggcatgttcaacatgctaagtacggtcttgggagtctcgatactgaatcttggacaaaagaaatacaccaagacaacatactggtgggatgggctccaatcatccgacgattttgctctcatagtgaatgcaccaaaccatgaaggaatacaagcaggagtggacagattctacaggacctgcaagttagtgggaatcaacatgagcaaaaagaagtcctatataaataagacagggacatttgaattcacaagctttttttatcgctatggatttgtggctaattttagcatggagctacccagctttggagtgtctggagtaaatgaatcagctgacatgagtattggagtaacagtgataaagaacaacatgataaacaatgaccttggacctgcaacggctcagatggctcttcaattgttcatcaaagactacagatacacatataggtgccataggggagacacacaaattcagacgagaagatcatttgagttgaagaagctatgggatcaaacccaatcaaaggtagggctattagtatcagatggaggaccaaacttatacaacatacggaatcttcacattcctgaagtctgcttaaaatgggagctaatggatgatgattatcggggaagactttgtaatcccctgaatccctttgtgagtcataaggagattgattctgtaaacaatgctgtggtaatgccagcccatggcccagccaaaagcatggaatatgatgccgttgctactacacattcctggattcccaagaggaatcgttctattctcaacacaagccaaaggggaattcttgaggatgaacagatgtaccagaaatgttgcaatttattcgagaaatttttccctagcagttcatataggagaccggttggaatttctagcatggtggaggccatggtgtctagggcccggattgatgccagagtcgactttgagtctggacggatcaagaaagaagagttctctgagatcatgaagatctgttccaccattgaagaactcagacggcaaaaataa

>H1N1_PB1_KX406193_A/North Carolina/07/2016

atggatgtcaatccgactctacttttcctaaaaattccagcacaaaatgccataagcaccacattcccttatactggagatcctccatacagccatggaacaggaacaggatacaccatggacacagtaaacagaacacaccaatactcagaaaaggggaagtggacaacaaacacagagactggtgcaccccagctcaacccgattgacggaccactacctgaagataatgaaccaagtgggtatgcacaaacagactgtgttctagaggctatggcttttcttgaagaatcccatccaggaatatttgagaattcatgccttgaaacaatggaagttgttcaacaaacaagggtagataaactgactcaaggtcgccagacttatgattggacattaaacagaaatcaaccggcagcaactgcattggccaacaccatagaagtttttagatcgaatgacctaacagctaacgagtcaggaaggttaatagatttcttaaaggatgtgatggaatcaatgaacaaagaggaaatagagataacaacccactttcaaagaaaaaggagagtaagagacaacatgaccaagaagatggtcacgcaaagaacaatagggaagaaaaaacagagactgaataagagaggctatctaataagagcactgacattaaatacgatgaccaaagatgcagagagaggcaagttaaaaagaagggctatcgcaacacctgggatgcagattagaggtttcgtatactttgttgaaactttagctaggagcatttgcgaaaagcttgaacagtctgggctcccagtagggggcaatgaaaagaaggccaaattggcaaatgttgtgagaaagatgatgactaattcacaagatacagagatttctttcacaatcactggggacaacaccaagtggaatgaaaatcaaaatcctcgaatgttcctggcgatgattacatatatcaccagaaatcaacccgagtggtttagaaacatcctgagcatggcacccataatgttctcaaataaaatggcaagactaggaaaagggtacatgttcgagagtaaaagaatgaagattcgaacacaaataccagcagaaatgctagcaagcattgatctgaagtatttcaatgaatcaacaaagaagaaaattgagaaaataaggcctcttttaatggatggcacagcatcactgagtcctgggatgatgatgggcatgttcaacatgctaagtacggtcttgggagtctcgatactgaatcttggacaaaagaaatacaccaagacaacatactggtgggatgggctccaatcatccgacgattttgctctcatagtgaatgcaccaaaccatgaaggaatacaagcaggagtggacagattctacaggacctgcaagttagtgggaatcaacatgagcaaaaagaagtcctatataaataagacagggacatttgaattcacaagctttttttatcgctatggatttgtggctaattttagcatggagctacccagctttggagtgtctggagtaaatgaatcagctgacatgagtattggagtaacagtgataaagaacaacatgataaacaatgaccttggacctgcaacggctcagatggctcttcaattattcatcaaagactacagatacacatataggtgccataggggagacacacaaattcagacgagaagatcatttgagttgaagaagctatgggatcaaacccaatcaaaggtagggctattagtatcagatggaggaccaaacttatacaacatacggaatcttcacattcctgaagtctgcttaaaatgggagctaatggatgatgattatcggggaagactttgtaatcccctgaatccctttgtgagtcataaggagattgattctgtaaacaatgctgtggtaatgccagcccatggcccagccaaaagcatggaatatgatgccgttgctactacacattcctggattcccaagaggaatcgttctattctcaacacaagccaaaggggaattcttgaggatgaacagatgtaccagaagtgttgcaatttattcgagaaatttttccctagcagttcatataggagaccggttggaatttctagcatggtggaggccatggtgtctagggcccggattgatgccagagtcgactttgagtctggacggatcaagaaagaagagttctctgagatcatgaagatctgttccaccattgaagaactcagacggcaaaaataa

>H1N1_PB1_KX407257_A/North Dakota/17/2016

atggatgtcaatccgactctacttttcctaaaaattccagcacaaaatgccataagcaccacattcccttatactggagatcctccatacagccatggaacaggaacaggatacaccatggacacagtaaacagaacacaccaatactcagaaaaggggaagtggacaacaaacacagagactggtgcaccccagctcaacccgattgacggaccactacctgaagataatgaaccaagtgggtatgcacaaacagactgtgttctagaggctatggcttttcttgaagaatcccatccaggaatatttgagaattcatgccttgaaacaatggaagttgttcaacaaacaagggtagataaactgactcaaggtcgccagacttatgattggacattaaacagaaatcaaccggcagcaactgcattggccaacaccatagaagtttttagatcgaatgacctaacagctaacgagtcaggaaggctaatagatttcttaaaggatgtgatggaatcaatgaacaaagaggaaatagagataacaacccactttcaaagaaaaaggagagtaagagacaacatgaccaagaagatggtcacgcaaagaacaatagggaagaaaaaacagagactgaataagagaggctatctaataagagcactgacattaaatacgatgaccaaagatgcagagagaggcaagttaaaaagaagggctatcgcaacacctgggatgcagattagaggtttcgtatactttgttgaaactttagctaggagcatttgcgaaaagcttgaacagtctgggctcccagtagggggcaatgaaaagaaggccaaattggcaaatgttgtgagaaagatgatgactaattcacaagatacagagatttctttcacaatcactggggacaacactaagtggaatgaaaatcaaaatcctcgaatgttcctggcgatgattacatatatcaccagaaatcaacccgagtggtttagaaacatcctgagcatggcacccataatgttctcaaataaaatggcaagactaggaaaagggtacatgttcgagagtaaaagaatgaagattcgaacacaaataccagcagaaatgctagcaagcattgatctgaagtatttcaatgaatcaacaaagaagaaaattgagaaaataaggcctcttttaatggatggcacagcatcactgagtcctgggatgatgatgggcatgttcaacatgctaagtacggtcttgggagtctcgatactgaatcttggacaaaagaaatacaccaagacaacatactggtgggatgggctccaatcatccgacgattttgctctcatagtgaatgcaccaaaccatgaaggaatacaagcaggagtggacagattctacaggacctgcaagttagtgggaatcaacatgagcaaaaagaagtcctatataaataagacagggacatttgaattcacaagctttttttatcgctatggatttgtggctaattttagcatggagctacccagctttggagtgtctggagtaaatgaatcagctgacatgagtattggagtaacagtgataaagaacaacatgataaacaatgaccttggacctgcaacggctcagatggctcttcaattgttcatcaaagactacagatacacatataggtgccataggggagacacacaaattcagacgagaagatcatttgagttgaagaagctatgggatcaaacccaatcaaaggtagggctattagtatcagatggaggaccaaacttatacaacatacggaatcttcacattcctgaagtctgcttaaaatgggagctaatggatgatgattatcggggaagactttgtaatcccctgaatccctttgtgagtcataaggagattgattctgtaaacaatgctgtggtaatgccagcccatggcccagccaaaagcatggaatatgatgccgttgctactacacattcctggattcccaagaggaatcgttctattctcaacacaagccaaaggggaattcttgaggatgaacagatgtaccagaagtgttgcaatttattcgagaaatttttccctagcagttcatataggagaccggttggaatttctagcatggtggaggccatggtgtctagggcccggattgatgccagagtcgactttgagtctggacggatcaagaaagaagagttctctgagatcatgaagatctgttccaccattgaagaactcagacggcaaagataa

>H1N1_PB1_KX407297_A/Ohio/09/2016

atggatgtcaatccgactctacttttcctaaaaattccagcacaaaatgccataagcaccacattcccttatactggagatcctccatacagccatggaacaggaacaggatacaccatggacacagtaaacagaacacaccaatactcagaaaaggggaagtggacaacaaacacagagactggtgcaccccagctcaacccgattgacggaccactacctgaagataatgaaccaagtgggtatgcacaaacagactgtgttctagaggctatggcttttcttgaagaatcccatccaggaatatttgagaattcatgccttgaaacaatggaagttgttcaacaaacaagggtagataaactgactcaaggtcgccagacttatgattggacattaaacagaaatcaaccggcagcaactgcattggccaacaccatagaagtttttagatcgaatgacctaacagctaacgagtcaggaaggctaatagatttcttaaaggatgtgatggaatcaatgaacaaagaggaaatagagataacaacccactttcaaagaaaaaggagagtaagagacaacatgaccaagaagatggtcacgcaaagaacaatagggaagaaaaaacagagactgaataagagaggctatctaataagagcactgacattaaatacgatgaccaaagatgcagagagaggcaagttaaaaagaagggctatcgcaacacctgggatgcagattagaggtttcgtatactttgttgaaactttagctaggagcatttgcgaaaagcttgaacagtctgggctcccagtagggggcaatgaaaagaaggccaaattggcaaatgttgtgagaaagatgatgactaattcacaagatacagagatttctttcacaatcactggggacaacaccaagtggaatgaaaatcaaaatcctcgaatgttcctggcgatgattacatatatcaccagaaatcaacccgagtggtttagaaacatcctgagcatggcacccataatgttctcaaataaaatggcaagactaggaaaagggtacatgttcgagagtaaaagaatgaagattcgaacacaaataccagcagaaatgctagcaagcattgatctgaagtatttcaatgaatcaacaaagaagaaaattgagaaaataaggcctcttttaatggatggcacagcatcactgagtcctgggatgatgatgggcatgttcaacatgctaagtacggtcttgggagtctcgatactgaatcttggacaaaagaaatacaccaagacaacatactggtgggatgggctccaatcatccgacgattttgctctcatagtgaatgcaccaaaccatgaaggaatacaagcaggagtggacagattctacaggacctgcaagttagtgggaatcaacatgagcaaaaagaagtcctatataaataagacagggacatttgaattcacaagctttttttatcgctatggatttgtggctaattttagcatggagctacccagctttggagtgtctggagtaaatgaatcagctgacatgagtattggagtaacagtgataaagaacaacatgataaacaatgaccttggacctgcaacggctcagatggctcttcaattgttcatcaaagactacagatacacatataggtgccataggggagacacacaaattcagacgagaagatcatttgagttgaagaagctatgggatcaaacccaatcaaaggtagggctattagtatcagatggaggaccaaacttatacaacatacggaatcttcacattcctgaagtctgcttaaaatgggagctaatggatgatgattatcggggaagactttgtaatcccctgaatccctttgtgagtcataaggagattgattctgtaaacaatgctgtggtaatgccagcccatggcccagccaaaagcatggaatatgatgccgttgctactacacattcctggattcccaagaggaatcgttctattctcaacacaagccaaaggggaattcttgaggatgaacagatgtaccagaagtgttgcaatttattcgagaaatttttccctagcagttcatatagaagaccggttggaatttctagcatggtggaggccatggtgtctagggcccggattgatgccagagtcgactttgagtctggacggatcaagaaagaagagttctctgagatcatgaagatctgttccaccattgaagaactcagacggcaaaaataa

>H1N1_PB1_KX407545_A/Hawaii/09/2016

atggatgtcaatccgactctacttttcctaaagattccagcacaaaatgccataagcaccacattcccttatactggagatcctccatacagccatggaacaggaacaggatacaccatggacacagtaaacagaacacaccaatactcagaaaaggggaagtggacaacaaacacagagactggtgcaccccagctcaacccgattgacggaccactacctgaagataatgaaccaagtgggtatgcacaaacagactgtgttctagaggctatggcttttcttgaagaatcccatccaggaatatttgagaattcatgccttgaaacaatggaagttgttcaacaaacaagggtagataaactgactcaaggtcgccagacttatgattggacattaaacagaaatcaaccggcagcaactgcattggccaacaccatagaagtttttagatcgaatgacctaacagctaacgagtcaggaaggctaatagatttcttaaaggatgtgatggaatcaatgaacaaagaggaaatagagataacaacccactttcaaagaaaaaggagagtaagagacaacatgaccaagaagatggtcacgcaaagaacaatagggaagaaaaaacagagactgaataagagaggctatctaataagagcactgacattaaatacgatgaccaaagatgcagagagaggcaagttaaaaagaagggctatcgcaacacctgggatgcagattagaggtttcgtatactttgttgaaactttagctaggagcatttgcgaaaagcttgaacagtctgggctcccagtagggggcaatgaaaagaaggccaaattggcaaatgttgtgagaaagatgatgactaattcacaagatacagagatttctttcacaatcactggggacaacactaagtggaatgaaaatcaaaatcctcgaatgttcctggcgatgattacatatatcaccagaaatcaacccgagtggtttagaaacatcctgagcatggcacccataatgttctcaaataaaatggcaagactaggaaaagggtacatgttcgagagtaaaagaatgaagattcgaacacaaataccagcagaaatgctagcaagcattgatctgaagtatttcaatgaatcaacaaagaagaaaattgagaaaataaggcctcttttaatggatggcacagcatcactgagtcctgggatgatgatgggcatgttcaacatgctaagtacggtcttgggagtctcgatactgaatcttggacaaaagaaatacaccaagacaacatactggtgggatgggctccaatcatccgacgattttgctctcatagtgaatgcaccaaaccatgaaggaatacaagcaggagtggacagattctacaggacctgcaagttagtgggaatcaacatgagcaaaaagaagtcctatataaataagacagggacatttgaattcacaagctttttttatcgctatggatttgtggctaattttagcatggagctacccagctttggagtgtctggagtaaatgaatcagctgacatgagtattggagtaacagtgataaagaacaacatgataaacaatgaccttggacctgcaacggctcagatggctcttcaattgttcatcaaagactacagatacacatataggtgtcataggggagacacacaaattcagacgagaagatcatttgagttgaagaagctatgggatcaaacccaatcaaaggtagggctattagtatcagatggaggaccaaacttatacaacatacggaatcttcacattcctgaagtctgcttaaaatgggagctaatggatgatgattatcggggaagactttgtaatcccctgaatccctttgtgagtcataaggagattgattctgtaaacaatgctgtggtaatgccagcccatggcccagccaaaagcatggaatatgatgccgttgctactacacattcctggattcccaagaggaatcgttctattctcaacacaagccaaaggggaattcttgaggatgaacagatgtaccagaagtgttgcaatttattcgagaaatttttccctagcagttcatataggagaccggttggaatttctagcatggtggaggccatggtgtctagggcccggattgatgccagagtcgactttgagtctggacggatcaagaaagaagagttctctgagatcatgaagatctgttccaccattgaagaactcagacggcaaaaataa

>H1N1_PB1_KX407769_A/Pennsylvania/16/2016

atggatgtcaatccgactctacttttcctaaaaattccagcacaaaatgccataagcaccacattcccttatactggagatcctccatacagccatggaacaggaacaggatacaccatggacacagtaaacagaacacaccaatactcagaaaaggggaagtggacaacaaacacagagactggtgcaccccagctcaacccgattgacggaccactacctgaagataatgaaccaagtgggtatgcacaaacagactgtgttctagaggctatggcttttcttgaagaatcccatccaggaatatttgagaattcatgccttgaaacaatggaagttgttcaacaaacaagggtagataaactgactcaaggtcgccagacttatgattggacattaaacagaaatcaaccggcagcaactgcattggccaacaccatagaagtttttagatcgaatgacctaacagctaacgagtcaggaaggctaatagatttcttaaaggatgtgatggaatcaatgaacaaagaggaaatagagataacaacccactttcaaagaaaaaggagagtaagagacaacatgaccaagaagatggtcacgcaaagaacaatagggaagaaaaaacagagactgaataagagaggctatctaataagagcactgacattaaatacgatgaccaaagatgcagagagaggcaagttaaaaagaagggctatcgcaacacctgggatgcagattagaggtttcgtatactttgttgaaactttrgctaggagcatttgcgaaaagcttgaacagtctgggctcccagtagggggcaatgaaaagaaggccaaattggcaaatgttgtgagaaagatgatgactaattcacaagatacagagatttctttcacaatcactggggacaacactaagtggaatgaaaatcaaaatcctcgaatgttcctggcgatgattacatatatcaccagaaatcaacccgagtggtttagaaacatcctgagcatggcacccataatgttctcaaataaaatggcaagactaggaaaagggtacatgttcgagagtaaaagaatgaagattcgaacacaaataccagcagaaatgctagcaagcattgatctgaagtatttcaatgaatcaacaaagaagaaaattgagaaaataaggcctcttttaatggatggcacagcatcactgagtcctgggatgatgatgggcatgttcaacatgctaagtacggtcttgggagtctcgatactgaatcttggacaaaagaaatacaccaagacaacatactggtgggatgggctccaatcatccgacgattttgctctcatagtgaatgcaccaaaccatgaaggaatacaagcaggagtggacagattctacaggacctgcaagttagtgggaatcaacatgagcaaaaagaagtcctatataaataagacagggacatttgaattcacaagctttttttatcgctatggatttgtggctaattttagcatggagctacccagctttggagtgtctggagtaaatgaatcagctgacatgagtattggagtaacagtgataaagaacaacatgataaacaatgaccttggacctgcaacggctcagatggctcttcaattattcatcaaagactacagatacacatataggtgccataggggagacacacaaattcagacgagaagatcatttgagttgaagaagctatgggatcaaacccaatcaaaggtagggctattggtatcagatggaggaccaaacttatacaacatacggaatcttcacattcctgaagtctgcttaaaatgggagctaatggatgatgattatcggggaagactttgtaatcccctgaatccctttgtgagtcataaggagattgattctgtaaacaatgctgtggtaatgccagcccatggcccagccaaaagcatggaatatgatgccgttgctactacacattcctggattcccaagaggaatcgttctattctcaacacaagccaaaggggaattcttgaggatgaacagatgtaccagaagtgttgcaatttattcgagaaatttttccctagcagttcatataggagaccggttggaatttctagcatggtggaggccatggtgtctagggcccggattgatgccagagtcgactttgagtctggacggatcaagaaagaagagttctctgagatcatgaagatctgttccaccattgaagaactcagacggcaaaaataa

>H1N1_PB1_KX407817_A/Louisiana/05/2016

atggatgtcaatccgactctacttttcctaaaaattccagcacaaaatgccataagcaccacattcccttatactggagatcctccatacagccatggaacaggaacaggatacaccatggacacagtaaacagaacacaccaatactcagaaaaggggaagtggacaacaaacacagagactggtgcaccccagctcaacccgattgacggaccactacctgaagataatgaaccaagtgggtatgcacaaacagactgtgttctagaggctatggcttttcttgaagaatcccatccaggaatatttgagaattcatgccttgaaacaatggaagttgttcaacaaacaagggtagataaactgactcaaggtcgccagacttatgattggacattaaacagaaatcaaccggcagcaactgcattggccaacaccatagaagtttttagatcgaatgacctaacagctaacgagtcaggaaggctaatagatttcttaaaggatgtgatggaatcaatgaacaaagaggaaatagagataacaacccactttcaaagaaaaaggagagtaagagacaacatgaccaagaagatggtcacgcaaagaacaatagggaagaaaaaacagagactgaataagagaggctatctaataagagcactgacattaaatacgatgaccaaagatgcagagagaggcaagttaaaaagaagggctatcgcaacacctgggatgcagattagaggtttcgtatactttgttgaaactttagctaggagcatttgcgaaaagcttgaacagtctgggctcccagtagggggcaatgaaaagaaggccaaattggcaaatgttgtgagaaagatgatgactaattcacaagatacagagatttctttcacaatcactggggacaacactaagtggaatgaaaatcaaaatcctcgaatgttcctggcgatgattacatatatcaccagaaatcaacccgagtggtttagaaacatcctgagcatggcacccataatgttctcaaataaaatggcaagactaggaaaagggtacatgttcgagagtaaaagaatgaagattcgaacacaaataccagcagaaatgctagcaagcattgatctgaagtatttcaatgaatcaacaaagaagaaaattgagaaaataaggcctcttttaatggatggcacagcatcactgagtcctgggatgatgatgggcatgttcaacatgctaagtacggtcttgggagtctcgatactgaatcttggacaaaagaaatacaccaagacaacatactggtgggatgggctccaatcatccgacgattttgctctcatagtgaatgcaccaaaccatgaaggaatacaagcaggagtggacagattctacaggacctgcaagttagtgggaatcaacatgagcaaaaagaagtcctatataaataagacagggacatttgaattcacaagctttttttatcgctatggatttgtggctaattttagcatggagctacccagctttggagtgtctggagtaaatgaatcagctgacatgagtattggagtaacagtgataaagaacaacatgataaacaatgaccttggacctgcaacggctcagatggctcttcaattgttcatcaaagactacagatacacatataggtgccataggggagacacacaaattcagacgagaagatcatttgagttgaagaagctatgggatcaaacccaatcaaaggtagggctattagtatcagatggaggaccaaacttatacaacatacggaatcttcacattcctgaagtctgcttaaaatgggagctaatggatgatgattatcggggaagactttgtaaccccctgaatccctttgtgagtcataaggagattgattctgtaaacaatgctgtggtaatgccagcccatggcccagccaaaagcatggaatatgatgccgttgctactacacattcctggattcccaagaggaatcgttctattctcaacacaagccaaaggggaattcttgaggatgaacagatgtaccagaagtgttgcaatttattcgagaaatttttccctagcagttcatataggagaccggttggaatttctagcatggtggaggccatggtgtctagggcccggattgatgccagagtcgactttgagtctggacggatcaagaaagaagagttctctgagatcatgaagatctgttccaccattgaagaactcagacggcaaaaataa

>H1N1_PB1_KX408225_A/New Jersey/01/2016

atggatgtcaatccgactctacttttcctaaaaattccagcacaaaatgccataagcaccacattcccttatactggagatcctccatacagccatggaacaggaacaggatacaccatggacacagtaaacagaacacaccaatactcagaaaaggggaagtggacaacaaacacagagactggtgcaccccagctcaacccgattgacggaccactacctgaagataatgaaccaagtgggtatgcacaaacagactgtgttctagaggctatggctttccttgaagaatcccatccaggaatatttgagaattcatgccttgaaacaatggaagttgttcaacaaacaagggtagataaactgactcaaggccgccagacttatgattggacattaaacagaaatcaaccggcagcaactgcattggccaacaccatagaagtctttagatcgaatgacctaacagctaacgagtcaggaaggctaatagatttcttaaaggatgtgatggaatcaatgaacaaagaggaaatagagataacaacccactttcaaagaaaaaggagagtaagagacaacatgaccaagaagatggtcacgcaaagaacaatagggaagaaaaaacagagactgaataagagaggctatctaataagagcactgacattaaatacgatgaccaaagatgcagagagaggcaagttaaaaagaagggctatcgcaacacctgggatgcagattagaggtttcgtatactttgttgaaactttagctaggagcatttgcgaaaagcttgaacagtctgggctcccagtagggggcaatgaaaagaaggccaaattggcaaatgttgtgagaaagatgatgactaattcacaagatacagagatttctttcacaatcactggggacaacactaagtggaatgaaaatcaaaatcctcgaatgttcctggcgatgattacatatatcaccagaaatcaacccgagtggtttagaaacatcctgagcatggcacccataatgttctcaaataaaatggcaagactaggaaaagggtacatgttcgagagtaaaagaatgaagattcgaacacaaataccagcagaaatgctagcaagcattgatctgaagtatttcaatgaatcaacaaagaagaaaattgaaaaaataaggcctcttttaatggatggcacagcatcactgagtcctgggatgatgatgggcatgttcaacatgctaagtacggtcttgggagtctcgatactgaatcttggacaaaagaaatacaccaagacaacatactggtgggatgggctccaatcatccgacgattttgctctcatagtgaatgcaccaaatcatgaaggaatacaagcaggagtggacagattctacaggacctgcaagttagtgggaatcaacatgagcaaaaagaagtcctatataaataagacagggacatttgaattcacaagctttttttatcgctatggatttgtggctaattttagcatggagctacccagctttggagtgtctggagtaaatgaatcagctgacatgagtattggagtaacagtgataaagaacaacatgataaacaatgaccttggacctgcaacggctcagatggctcttcaattgttcatcaaagactacagatacacatataggtgccataggggagacacacaaattcagacgagaagatcatttgagttgaagaagctatgggatcaaacccaatcaaaggtagggctattagtatcagatggaggaccaaacttatacaacatacggaatcttcacattcctgaagtctgcttaaaatgggagctaatggatgatgattatcggggaagactttgtaatcccctgaatccctttgtgagtcataaggagattgattctgtaaacaatgctgtggtaatgccagcccatggtccagccaaaagcatggaatatgatgccgttgcaactacacattcctggattcccaagaggaatcgttctattctcaacacaagccaaaggggaattcttgaggatgaacagatgtaccagaagtgttgcaatttattcgagaaatttttccctagcagttcatataggagaccggttggaatttctagcatggtggaggccatggtgtctagggcccggattgatgccagagtcgactttgagtctggacggatcaagaaagaagagttctctgagatcatgaagatctgttccaccattgaagaactcagacggcaaaaataa

>H1N1_PB1_KX408529_A/Georgia/06/2016

atggatgtcaatccgactctacttttcctaaaaattccagcacaaaatgccataagcaccacattcccttatactggagatcctccatacagccatggaacaggaacaggatacaccatggacacagtaaacagaacacaccaatactcagaaaaggggaagtggacaacaaacacagagactggtgcaccccagctcaacccgattgacggaccactacctgaagataatgaaccaagtgggtatgcacaaacagactgtgttctagaggctatggcttttcttgaagaatcccatccaggaatatttgagaattcatgccttgaaacaatggaagttgttcaacaaacaagggtagataaactgactcaaggtcgccagacttatgattggacattaaacagaaatcaaccggcagcaactgcattggccaacaccatagaagtttttagatcgaatgacctaacagctaacgagtcaggaaggctaatagatttcttaaaggatgtgatggaatcaatgaacaaagaggaaatagagataacaacccactttcaaagaaaaaggagagtaagagacaacatgaccaagaagatggtcacgcaaagaacaatagggaagaaaaaacagagactgaataagagaggctatctaataagagcactgacattaaatacgatgaccaaagatgcagagagaggcaagttaaaaagaagggctatcgcaacacctgggatgcagattagaggtttcgtatactttgttgaaactttagctaggagcatttgcgaaaagcttgaacagtctgggctcccagtagggggcaatgaaaagaaggccaaattggcaaatgttgtgagaaagatgatgactaattcacaagatacagagatttctttcacaatcactggggacaacactaagtggaatgaaaatcaaaatcctcgaatgttcctggcgatgattacatatatcaccagaaatcaacccgagtggtttagaaacatcctgagcatggcacccataatgttctcaaataaaatggcaagactaggaaaagggtacatgttcgagagtaaaaaaatgaagattcgaacacaaataccagcagaaatgctagcaagcattgatctgaagtatttcaatgaatcaacaaagaagaaaattgagaaaataaggcctcttttaatggatggcacagcatcactgagtcctgggatgatgatgggcatgttcaacatgctaagtacggtcttgggagtctcgatactgaatcttggacaaaagaaatacaccaagacaacatactggtgggatgggctccaatcatccgacgattttgctctcatagtgaatgcaccaaaccatgaaggaatacaagcaggagtggacagattctacaggacctgcaagttagtgggaatcaacatgagcaaaaagaagtcctatataaataagacagggacatttgaattcacaagctttttttatcgctatggatttgtggctaattttagcatggagctacccagctttggagtgtctggagtaaatgaatcagctgacatgagtattggagtaacagtgataaagaacaacatgataaacaatgaccttggacctgcaacggctcagatggctcttcaattgttcatcaaagactacagatacacatataggtgccataggggagacacacaaattcaaacgagaagatcatttgagttgaagaagctatgggatcaaacccaatcaaaggtagggctattagtatcagatggaggaccaaacttatacaacatacggaatcttcacattcctgaagtctgcttaaaatgggagctaatggatgatgattatcggggaagactttgtaatcccctgaatccctttgtgagtcataaggagattgattctgtaaacaatgctgtggtaatgccagcccatggcccagccaaaaacatggaatatgatgccgttgctactacacattcctggattcccaagaggaatcgttctattctcaacacaagccaaaggggaattcttgaggatgaacagatgtaccagaagtgttgcaatttattcgagaaatttttccctagcagttcatataggagaccggttggaatttctagcatggtggaggccatggtgtctagggcccggattgatgccagagtcgactttgagtctggacggatcaagaaagaagagttctctgagatcatgaagatctgttccaccattgaagaactcagacggcaaaaataa

>H1N1_PB1_KX408617_A/Georgia/15/2016

atggatgtcaatccgactctacttttcctaaaaattccagcacaaaatgccataagcaccacattcccttatactggagatcctccatacagccatggaacaggaacaggatacaccatggacacagtgaacagaacacaccaatactcagaaaaggggaagtggacaacaaacacagagactggtgcaccccagctcaacccgattgacggaccactacctgaagataatgaaccaagtgggtatgcacaaacagactgtgttctagaggctatggcttttcttgaagaatcccatccaggaatatttgagaattcatgccttgaaacaatggaagttgttcaacaaacaagggtagataaactgactcaaggtcgccagacttatgattggacattaaacagaaatcaaccggcagcaactgcattggccaacaccatagaagtttttagatcgaatgacctaacagctaacgagtcaggaaggctaatagatttcttaaaggatgtgatggaatcaatgaacaaagaggaaatagagataacaacccactttcaaagaaaaaggagagtaagagacaacatgaccaagaagatggtcacgcaaagaacaatagggaagaaaaaacagagactgaataagagaggctatctaataagagcactgacattaaatacgatgaccaaagatgcagagagaggcaagttaaaaagaagggctatcgcaacacctgggatgcagattagaggtttcgtatactttgttgaaactttagctaggagcatttgcgaaaagcttgaacagtctgggctcccagtagggggcaatgaaaagaaggccaaattggcaaatgttgtgagaaagatgatgactaattcacaagatacagagatttctttcacaatcactggggacaacactaagtggaatgaaaatcaaaatcctcgaatgttcctggcgatgattacatatatcaccagaaatcaacccgagtggtttagaaacatcctgagcatggcacccataatgttctcaaataaaatggcaagactaggaaaagggtacatgttcgagagtaaaagaatgaagattcgaacacaaataccagcagaaatgctagcaagcattgatctgaagtatttcaatgaatcaacaaagaagaaaattgagaaaataaggcctcttttaatggatggcacagcatcactgagtcctgggatgatgatgggcatgttcaacatgctaagtacggtcttgggagtctcgatactgaatcttggacaaaagaaatacaccaagacaacatactggtgggatgggcttcaatcatccgacgattttgctctcatagtgaatgcaccaaaccatgaaggaatacaagcaggagtggacagattctacaggacctgcaagttagtgggaatcaacatgagcaaaaagaagtcctatataaataagacagggacatttgaattcacaagctttttttatcgctatggatttgtggctaattttagcatggagctacccagctttggagtgtctggagtaaatgaatcagctgacatgagtattggagtaacagtgataaagaacaacatgataaacaatgaccttggacctgcaacggctcagatggctcttcaattgttcatcaaagactacagatacacatataggtgccataggggagacacacaaattcagacgagaagatcatttgagttgaagaagctatgggatcaaacccaatcaaaggtagggctattagtatcagatggaggaccaaacttatacaacatacggaatcttcacattcctgaagtctgcttaaaatgggagctaatggatgatgattatcggggaagactttgtaatcccctgaatccctttgtgagtcataaggagattgattctgtaaacaatgctgtggtaatgccagcccatggcccagccaaaagcatggaatatgatgccgttgctactacacattcctggattcccaagaggaatcgttctattctcaacacaagccaaaggggaattcttgaggatgaacagatgtaccagaagtgttgcaatttattcgagaaatttttccctagcagttcatataggagaccggttggaatttctagcatggtggaggccatggtgtctagggcccggattgatgccagagtcgactttgagtctggacggatcaagaaagaagagttctctgagatcatgaagatctgttccaccattgaagaactcagacggcaaaaataa

>H1N1_PB1_KX408761_A/Virginia/13/2016

atggatgtcaatccgactctacttttcctaaaaattccagcacaaaatgccataagcaccacattcccttatactggagatcctccatacagccatggaacaggaacaggatacaccatggacacagtaaacagaacacaccaatactcagaaaaggggaagtggacaacaaacacagagactggtgcaccccagctcaacccgattgacggaccactacctgaarataatgaaccaagtgggtatgcacaaacagactgtgttctagaggctatggcttttcttgaagaatcccatccaggaatatttgagaattcatgccttgaaacaatggaagttgttcaacaaacaagggtagataaactgactcaaggtcgccagacttatgattggacattaaacagaaatcaaccggcagcaactgcattggccaacaccatagaagtttttagatcgaatgacctaacagctaacgagtcaggaaggctaatagatttcttaaaggatgtgatggaatcaatgaacaaagaggaaatagagataacaacccactttcaaagaaaaaggagagtaagagacaacatgaccaagaagatggtcacgcaaagaacaatagggaagaaaaaacagagactgaataagagaggctatctaataagagcactgacattaaatacgatgaccaaagatgcagagagaggcaagttaaaaagaagggctatcgcaacacctgggatgcagattagaggtttcgtatactttgttgaaactttagctaggagcatttgcgaaaagcttgaacagtctgggctcccagtagggggcaatgaaaagaaggccaaattggcaaatgttgtgagaaagatgatgactaattcacaagatacagagatttctttcacaatcactggggacaacactaagtggaatgaaaatcaaaatcctcgaatgttcctggcgatgattacatatatcaccagaaatcaacccgagtggtttagaaacatcctgagcatggcacccataatgttctcaaataaaatggcaagactaggaaaagggtacatgttcgagagtaaaagaatgaagattcgaacacaaataccagcagaaatgctagcaagcattgatctgaagtatttcaatgaatcaacaaagaagaaaattgagaaaataaggcctcttttaatggatggcacagcatcactgagtcctgggatgatgatgggcatgttcaacatgctaagtacggtcttgggagtctcgatactgaatcttggacaaaagaaatacaccaagacaacatactggtgggatgggctccaatcatccgacgattttgctctcatagtsaatgcaccaaaccatgaaggaatacaagcaggggtggacagattctacaggacctgcaagttagtgggaatcaacatgagcaaaaagaagtcctatataaataagacagggacatttgaattcacaagctttttttatcgctatggatttgtggctaattttagcatggagctacccagctttggagtgtctggagtaaatgaatcagctgacatgagtattggagtaacagtgataaagaacaacatgataaacaatgaccttggacctgcaacggctcagatggctcttcaattgttcatcaaagactacagatacacatataggtgccataggggagacacacaaattcagacgagaagatcatttgagttgaagaagctatgggatcaaacccaatcaaaggtagggctattagtatcagatggaggaccaaacttatacaacatacggaatcttcacattcctgaagtctgcttaaaatgggagctaatggatgatgattatcggggaagactttgtaatcccctgaatccctttgtgagtcataaggagattgattctgtaaacaatgctgtggtaatgccagcccatggcccagccaaaagcatggaatatgatgccgttgctactacacattcctggattcccaagaggaatcgttctattctcaacacaagccaaaggggaattcttgaggatgaacagatgtaccagaagtgttgcaatttattcgagaaatttttccctagcagttcatataggagaccggttggaatttctagcatggtggaggccatggtgtctagggcccggattgatgccagagtcgactttgagtctggacggatcaagaaagaagagttctctgagatcatgaagatctgttccaccattgaagaactcagacggcaaaaataa

>H1N1_PB1_KX409153_A/Missouri/04/2016

atggatgtcaatccgactctacttttcctaaaaattccagcacaaaatgccataagcaccacattcccttatactggagatcctccatacagccatggaacaggaacaggatacaccatggacacagtaaacagaacacaccaatactcagaaaaggggaagtggacaacaaacacagagactggtgcaccccagctcaacccgattgacggaccactacctgaagataatgaaccaagtgggtatgcacaaacagactgtgttctagaggctatggcttttcttgaagaatcccatccaggaatatttgagaattcatgccttgaaacaatggaagttgttcaacaaacaagggtagataaactgactcaaggtcgccagacttatgattggacattaaacagaaatcaaccggcagcaactgcattggccaacaccatagaagtttttagatcgaatgacctaacagctaacgagtcaggaaggctaatagatttcttaaaggatgtgatggaatcaatgaacaaagaggaaatagagataacaacccactttcaaagaaaaaggagagtaagagacaacatgaccaagaagatggtcacgcaaagaacaatagggaagaaaaaacagagactgaataagagaggctatctaataagagcactgacattaaatacgatgaccaaagatgcagagagaggcaagttaaaaagaagggctatcgcaacacctgggatgcagattagaggtttcgtatactttgttgaaactttagctaggagcatttgcgaaaagcttgaacagtctgggctcccagtagggggcaatgaaaagaaggccaaattggcaaatgttgtgagaaagatgatgactaattcacaagatacagagatttctttcacaatcactggggacaacactaagtggaatgaaaatcaaaatcctcgaatgttcctggcgatgattacatatatcaccagaaatcaacccgagtggtttagaaacatcctgagcatggcacccataatgttctcaaataaaatggcaagactaggaaaagggtacatgttcgagagtaaaagaatgaagattcgaacacaaataccagcagaaatgctagcaagcattgatctgaagtatttcaatgaatcaacaaagaagaaaattgagaaaataaggcctcttttaatggatggcacagcatcactgagtcctgggatgatgatgggcatgttcaacatgctaagtacggtcttgggagtctcgatactgaatcttggacaaaagaaatacaccaagacaacatactggtgggatgggctccaatcatccgacgattttgctctcatagtgaatgcaccaaaccatgaaggaatacaagcaggagtggacagattctacaggacctgcaagttagtgggaatcaacatgagcaaaaagaagtcctatataaataagacagggacatttgaattcacaagctttttttatcgctatggatttgtggctaattttagcatggagctacccagctttggagtgtctggagtaaatgaatcagctgacatgagtattggagtaacagtgataaagaacaacatgataaacaatgaccttggacctgcaacggctcagatggctcttcaattgttcatcaaagactacagatacacatataggtgccataggggagacacacaaattcagacgagaagatcatttgagttgaagaagctatgggatcaaacccaatcaaaggtagggctattagtatcagatggaggaccaaacttatacaacatacggaatcttcacattcctgaagtctgcttaaaatgggagctaatggataatgattatcggggaagactttgtaatcccctgaatccctttgtgagtcataaggagattgattctgtaaacaatgctgtggtaatgccagcccatggcccagccaaaagcatggaatatgatgccgttgctactacacattcctggattcccaagaggaatcgttctattctcaacacaagccaaaggggaattcttgaggatgaacagatgtaccagaagtgttgcaatttattcgagaaatttttccctagcagttcatataggagaccggttggaatttctagcatggtggaggccatggtgtctagggcccggattgatgccagagtcgactttgagtctggacggatcaagaaagaagagttctctgagatcatgaagatctgttccaccattgaagaactcagacggcaaaaataa

>H1N1_PB1_KX409793_A/Wisconsin/39/2016

atggatgtcaatccgactctacttttcctaaaaattccagcacaaaatgccataagcaccacattcccttatactggagatcctccatacagccatggaacaggaacaggatacaccatggacacagtaaacagaacacaccaatactcagaaaaggggaagtggacaacaaacacagagactggtgcaccccagctcaacccgattgacggaccactacctgaagataatgaaccaagtgggtatgcacaaacagactgtgttctagaggctatggcttttcttgaagaatcccatccaggaatatttgagaattcatgccttgaaacaatggaagttgttcaacaaacaagggtagataaactgactcaaggtcgccagacttatgattggacattaaacagaaatcaaccggcagcaactgcattggccaacaccatagaagtttttagatcgaatgacctaacagctaacgagtcaggaaggctaatagatttcttaaaggatgtgatggaatcaatgaacaaagaggaaatagagataacaacccattttcaaagaaaaaggagagtaagagacaacatgaccaagaagatggtcacgcaaagaacaatagggaagaaaaaacagagactgaataagagaggctatctaataagagcactgacattaaatacgatgaccaaagatgcagagagaggcaagttaaaaagaagggctatcgcaacacctgggatgcagattagaggtttcgtatactttgttgaaactttagctaggagcatttgcgaaaagcttgaacagtctgggctcccagtagggggcaatgaaaagaaggccaaattggcaaatgttgtgagaaagatgatgactaattcacaagatacagagatttctttcacaatcactggggacaacactaagtggaatgaaaatcaaaatcctcgaatgttcctggcgatgattacatatatcaccagaaatcaacccgagtggtttagaaacatcctgagcatggcacccataatgttctcaaataaaatggcaagactaggaaaagggtacatgttcgagagtaaaagaatgaagattcgaacacaaataccggcagaaatgctagcaagcattgatctgaagtatttcaatgaatcaacaaagaagaaaattgagaaaataaggcctcttttaatggatggcacagcatcactgagtcctgggatgatgatgggaatgttcaacatgctaagtacggtcttgggagtctcgatactgaatcttggacaaaagaaatacaccaagacaacatactggtgggatgggctccaatcatccgacgattttgctctcatagtgaatgcaccaaaccatgaaggaatacaagcaggagtggacagattctacaggacctgcaagttagtgggaatcaacatgagcaaaaagaagtcctatataaataagacagggacatttgaattcacaagctttttttatcgctatggatttgtggctaattttagcatggagctacccagctttggagtgtctggagtaaatgaatcagctgacatgagtattggagtaacagtgataaagaacaacatgataaacaatgaccttggacctgcaacggctcagatggctcttcaattgttcatcaaagactacagatacacatataggtgccataggggagacacacaaattcagacgagaagatcatttgagttgaagaagctatgggatcaaacccaatcaaaggtagggctattagtatcagatggaggaccaaacttatacaacatacggaatcttcacattcctgaagtctgcttaaaatgggagttaatggatgatgattatcggggaagactttgtaatcccctgaatccctttgtgagtcataaggagattgattctgtaaacaatgctgtggtaatgccagcccatggcccagccaaaagcatggaatatgatgccgttgctactacacattcctggattcccaagaggaatcgttctattctcaacacaagccaaaggggaattcttgaggatgaacagatgtaccagaagtgttgcaatttattcgagaaatttttccctagcagttcatataggagaccggttggaatttctagcatggtggaggccatggtgtctagggcccggattgatgccagagtcgactttgagtctggacggatcaagaaagaagagttctctgagatcatgaagatctgttccaccattgaagaactcagacggcaaaaataa

>H1N1_PB1_KX410137_A/Wyoming/06/2016

atggatgtcaatccgactctacttttcctaaaaattccagcacaaaatgccataagcaccacattcccttatactggagatcctccatacagccatggaacaggaacaggatacaccatggacacagtaaacagaacacaccaatactcagaaaaggggaagtggacaacaaacacagagactggtgcaccccagctcaacccgattgacggaccactacctgaagataatgaaccaagtgggtatgcacaaacagactgtgttctagaggctatggcttttcttgaagaatcccatccaggaatatttgagaattcatgccttgaaacaatggaagttgttcaacaaacaagggtagataaactgactcaaggtcgccagacttatgattggacattaaacagaaatcaaccggcagcaactgcattggccaacaccatagaagtttttagatcgaatgacctaacagctaacgagtcaggaaggctaatagatttcttaaaggatgtgatggaatcaatgaacaaagaggaaatagagataacaacccactttcaaagaaaaaggagagtaagagacaacatgaccaagaagatggtcacgcaaagaacaatagggaagaaaaaacagagactgaataagagaggctatctaataagagcactgacattaaatacgatgaccaaagatgcagagagaggcaagttaaaaagaagggctatcgcaacacctgggatgcagattagaggtttcgtatactttgttgaaactttagctaggagcatttgcgaaaagcttgaacagtctgggctcccagtagggggcaatgaaaagaaggccaaattggcaaatgttgtgagaaagatgatgactaattcacaagatacagagatttctttcacaatcactggggacaacactaagtggaatgaaaatcaaaatcctcgaatgttcctggcgatgattacatatatcaccagaaatcaacccgagtggtttagaaacatcctgagcatggcacccataatgttctcaaataaaatggcaagactaggaaaagggtacatgttcgagagtaaaagaatgaagattcgaacacaaataccagcagaaatgctagcaagcattgatctgaagtatttcaatgaatcaacaaagaagaaaattgagaaaataaggcctcttttaatggatggcacagcatcactgagtcctgggatgatgatgggcatgttcaacatgctaagtacggtcttgggagtctcgatactgaatcttggacaaaagaaatacaccaagacaacatactggtgggatgggctccaatcatccgacgattttgctctcatagtgaatgcaccaaaccatgaaggaatacaagcaggagtggacagattctacaggacctgcaagttagtgggaatcaacatgagcaaaaagaagtcctatataaataagacagggacatttgaattcacaagctttttttatcgctatggatttgtggctaattttagcatggagctacccagctttggagtgtctggagyaaatgaatcagctgacatgagtattggagtaacagtgataaagaacaacatgataaacaatgaccttggacctgcaacggctcagatggctcttcaattgttcatcaaagactacagatacacatataggtgccataggggagacacacaaattcagacgagaagatcatttgagttgaagaagctatgggatcaaacccaatcaaaggtagggctattagtatcagatggaggaccaaacttatacaacatacggaatcttcacattcctgaagtctgcttaaaatgggagctaatggatgatgattatcggggaagactttgtaatcccctgaatccctttgtgagtcataaggagattgattctgtaaacaatgctgtggtaatgccagcccatggcccagccaaaagcatggaatatgatgccgttgctactacacattcctggattcccaagaggaatcgttctattctcaacacaagccaaaggggaattcttgaggatgaacagatgtaccagaagtgttgcaatttattcgagaaatttttccctagcagttcatataggagaccggttggaatttctagcatggtggaggccatggtgtctagggcccggattgatgccagagtcgactttgagtctggacggatcaagaaagaagagttctctgagatcatgaagatctgttccaccattgaagaactcagacggcaaaaataa

>H1N1_PB1_KX410665_A/Ohio/17/2016

atggatgtcaatccgactctacttttcctaaaaattccagcacaaaatgccataagcaccacattcccttatactggagatcctccatacagccatggaacaggaacaggatacaccatggacacagtaaacagaacacaccaatactcagaaaaggggaagtggacaacaaacacagagactggtgcaccccagctcaacccgattgacggaccactacctgaagataatgaaccaagtgggtatgcacaaacagactgtgttctagaggctatggcttttcttgaagaatcccatccaggaatatttgagaattcatgccttgaaacaatggaagttgttcaacaaacaagggtagataaactgactcaaggtcgccagacttatgattggacattaaacagaaatcaaccggcagcaactgcattggccaacaccatagaagtttttagatcgaatgacctaacagctaacgagtcaggaaggctaatagatttcttaaaggatgtgatggaatcaatgaacaaagaggaaatagagataacaacccactttcaaagaaaaaggagagtaagagacaacatgaccaagaagatggtcacgcaaagaacaatagggaagaaaaaacagagactgaataagagaggctatctaataagagcactgacattaaatacgatgaccaaagatgcagagagaggcaagttaaaaagaagggctatcgcaacacctgggatgcagattagaggtttcgtatactttgttgaaactttagctaggagcatttgcgaaaagcttgaacagtctgggctcccagtagggggcaatgaaaagaaggccaaattggcaaatgttgtgagaaagatgatgactaattcacaagatacagagatttctttcacaatcactggggacaacactaagtggaatgaaaatcaaaatcctcgaatgttcctggcgatgattacatatatcaccagaaatcaacccgagtggtttagaaacatcctgagcatggcacccataatgttctcaaataaaatggcaagactaggaaaagggtacatgttcgagagtaaaagaatgaagattcgaacacaaataccagcagaaatgctagcaagcattgatctgaagtatttcaatgaatcaacaaagaagaaaattgagaaaataaggcctcttttaatggatggcacagcatcactgagtcctgggatgatgatgggcatgttcaacatgctaagtacggtcttgggagtctcgatactgaatcttggacaaaagaaatacaccaagacaacatactggtgggatgggctccaatcatccgacgattttgctctcatagtgaatgcaccaaaccatgaaggaatacaagcaggagtggacagattctacaggacctgcaagttagtgggaatcaacatgagcaaaaagaagtcctatataaataagacagggacatttgaattcacaagctttttttaccgctatggatttgtggctaattttagcatggagctacccagctttggagtgtctggagtaaatgaatcagctgacatgagtattggagtaacagtgataaagaacaacatgataaacaatgaccttggacctgcaacggctcagatggctcttcaattgttcatcaaagactacagatacacatataggtgccataggggagacacacaaattcagacgagaagatcatttgagttgaagaagctatgggatcaaacccaatcaaaggtagggctattagtatcagatggaggaccaaacttatacaacatacggaatcttcacattcctgaagtctgcttaaaatgggagctaatggatgatgattatcggggaagactttgtaatcccctgaatccctttgtgagtcataaggagattgattctgtaaacaatgctgtggtaatgccagcccatggcccagccaaaagcatggaatatgatgccgttgctactacacattcctggattcccaagaggaatcgttctattctcaacacaagccaaaggggaattcttgaggatgaacagatgtaccagaagtgttgcaatttattcgagaaatttttccctagcagttcatataggagaccggttggaatttctagcatggtggaggccatggtgtctagggcccggattgatgccagagtcgactttgagtctggacggatcaagaaagaagagttctctgagatcatgaagatctgttccaccattgaagaactcagacggcaaaaataa

>H1N1_PB1_KX411129_A/Illinois/25/2016

atggatgtcaatccgactctacttttcctaaaaattccagcacaaaatgccataagcaccacattcccttatactggagatcctccatacagccatggaacaggaacaggatacaccatggacacagtaaacagaacacaccaatactcagaaaaggggaagtggacaacaaacacagagactggtgcaccccagctcaacccgattgacggaccactacctgaagataatgaaccaagtgggtatgcacaaacagactgtgttctagaggctatggcttttcttgaagaatcccatccaggaatatttgagaattcatgccttgaaacaatggaagttgttcaacaaacaagggtagataaactgactcaaggtcgccagacttatgattggacattaaacagaaatcaaccggcagcaactgcattggccaacaccatagaagtttttagatcgaatgacctaacagctaacgagtcaggaaggctaatagatttcttaaaggatgtgatggaatcaatgaacaaagaggaaatagagataacaacccactttcaaagaaaaaggagagtaagagacaacatgaccaagaagatggtcacgcaaagaacaatagggaagaaaaaacagagactgaataagagaggctatctaataagagcactgacattaaatacgatgaccaaagatgcagagagaggcaagttaaaaagaagggctatcgcaacacctgggatgcagattagaggtttcgtatactttgttgaaactttagctaggagcatttgcgaaaagcttgaacagtctgggctcccagtagggggcaatgaaaagaaggccaaattggcaaatgttgtaagaaagatgatgactaattcacaagatacagagatttctttcacaatcactggggacaacactaagtggaatgaaaatcaaaatcctcgaatgttcctggcgatgattacatatatcaccagaaatcaacccgagtggtttagaaacatcctgagcatggcacccataatgttctcaaataaaatggcaagactaggaaaagggtacatgttcgagagtaaaagaatgaagattcgaacacaaataccagcagaaatgctagcaagcattgatctgaagtatttcaatgaatcaacaaagaagaaaattgagaaaataaggcctcttttaatggatggcacagcatcactgagtcctgggatgatgatgggcatgttcaacatgctaagtacggtcttgggagtctcgatactgaatcttggacaaaagaaatacaccaagacaacatactggtgggatgggctccaatcatccgacgattttgctctcatagtgaatgcaccaaaccatgaaggaatacaagcaggagtggacaggttctacaggacctgcaagttagtgggaatcaacatgagcaaaaagaagtcctatataaataagacagggacatttgaattcacaagctttttttatcgctatggatttgtggctaattttagcatggagctacccagctttggagtgtctggagtaaatgaatcagctgacatgagtattggagtaacagtgataaagaacaacatgataaacaatgaccttggacctgcaacggctcagatggctcttcaattgttcatcaaagactacagatacacatataggtgccataggggagacacacaaattcagacgagaagatcatttgagttgaagaagctatgggatcaaacccaatcaaaggtagggctattagtatcagatggaggaccaaacttatacaacatacggaatcttcacattcctgaagtctgcttaaaatgggagctaatggatgatgattatcggggaagactttgtaatcccctgaatccctttgtgagtcataaggagattgattctgtaaacaatgctgtggtaatgccagcccatggcccagccaaaagcatggaatatgatgccgttgctactacacattcctggattcccaagaggaatcgttctattctcaacacaagccaaaggggaattcttgaggatgaacagatgtaccagaagtgttgcaatttattcgagaaatttttccctagcagttcatataggagaccggttggaatttctagcatggtggaggctatggtgtctagggcccggattgatgccagagtcgactttgagtctggacggatcaagaaagaagagttctctgagatcatgaagatctgttccaccattgaagaactcagacggcaaaaataa

>H1N1_PB1_KX411393_A/Michigan/67/2016

atggatgtcaatccgactctacttttcctaaaaattccagcacaaaatgccataagcaccacattcccttatactggagatcctccatacagccatggaacaggaacaggatacaccatggacacagtaaacagaacacaccaatactcagaaaaggggaagtggacaacaaacacagagactggtgcaccccagctcaacccgattgacggaccactacctgaagataatgaaccaagtgggtatgcacaaacagactgtgttctagaggctatggcttttcttgaagaatcccatccaggaatatttgagaattcatgccttgaaacaatggaagttgttcaacaaacaagggtagataaactgactcaaggtcgccagacttatgattggacattaaacagaaatcaaccggcagcaactgcattggccaacaccatagaagtttttagatcgaatgacctaacagctaacgagtcaggaaggctaatagatttcttaaaggatgtgatggaatcaatgaacaaagaggaaatagagataacaacccactttcaaagaaaaaggagagtaagagacaacatgaccaagaagatggtcacgcaaagaacaatagggaagaaaaaacagagactgaataagagaggctatctaataagagcactgacattaaatacgatgaccaaagatgcagagagaggcaagttaaaaagaagggctatcgcaacacctgggatgcagattagaggtttcgtatactttgttgaaactttagctaggagcatttgcgaaaagcttgaacagtctgggctcccagtagggggcaatgaaaagaaggccaaattggcaaatgttgtgagaaagatgatgactaattcacaagatacagagatttctttcacaatcactggggacaacactaagtggaatgaaaatcaaaatcctcgaatgttcctggcgatgattacatatatcaccagaaatcaacccgagtggtttagaaacatcctgagcatggcacccataatgttctcaaataaaatggcaagactaggaaaagggtacatgttcgagagtaaaagaatgaagattcgaacacaaataccagcagaaatgctagcaagcattgatctgaagtatttcaatgaatcaacaaagaagaaaattgagaaaataaggcctcttttaatggatggcacagcatcactgagtcctgggatgatgatgggcatgttcaacatgctaagtacggtcttgggagtctcgatactgaatcttggacaaaagaaatacaccaagacaacatactggtgggatgggctccaatcatccgacgattttgctctcatagtgaatgcaccaaaccatgaaggaatacaagcaggagtggacagattctacaggacctgcaagttagtgggaatcaacatgagcaaaaagaagtcctatataaataagacagggacatttgaattcacaagctttttttatcgctatggatttgtggctaattttagcatggagctacccagctttggagtgtctggagtaaatgaatcagctgatatgagtattggagtaacagtgataaagaacaacatgataaacaatgaccttggacctgcaacggctcagatggctcttcaattgttcatcaaagactacagatacacatataggtgccataggggagacacacaaattcagacgagaagatcatttgagttgaagaagctatgggatcaaacccaatcaaaggtagggctattagtatcagatggaggaccaaacttatacaacatacggaatcttcacattcctgaagtctgcttaaaatgggagctaatggatgatgattatcggggaagactttgtaatcccctgaatccctttgtgagtcataaggagattgattctgtaaacaatgctgtggtaatgccagcccatggcccagccaaaagcatggagtatgatgccgttgctactacacattcctggattcccaagaggaatcgttctattctcaacacaagccaaaggggaattcttgaggatgaacagatgtaccagaagtgttgcaatttattcgagaaatttttccctagcagttcatataggagaccggttggaatttctagcatggtggaggccatggtgtctagggcccggattgatgccagagtcgactttgagtctggacggatcaagaaagaagagttctctgagatcatgaagatctgttccaccattgaagaactcagacggcaaaaataa

>H1N1_PB1_KX411465_A/Wisconsin/58/2016

atggatgtcaatccgactctacttttcctaaaaattccagcacaaaatgccataagcaccacattcccttatactggagatcctccatacagccatggaacaggaacaggatacaccatggacacagtaaacagaacacaccaatactcagaaaaggggaagtggacaacaaacacagagactggtgcaccccagctcaacccgattgacggaccactacctgaagataatgaaccaagtgggtatgcacaaacagactgtgttctagaggctatggcttttcttgaagaatcccatccaggaatatttgagaattcatgccttgaaacaatggaagttgttcaacaaacaagggtagataaactgactcaaggtcgccagacttatgattggacattaaacagaaatcaaccggcagcaactgcattggccaacaccatagaagtttttagatcgaatgacctaacagctaacgagtcaggaaggctaatagatttcttaaaggatgtgatggaatcaatgaacaaagaggaaatagagataacaacccactttcaaagaaaaaggagagtaagagacaacatgaccaagaagatggtcacgcaaagaacaatagggaagaaaaaacagagactgaataagagaggctatctaataagagcactgacattaaatacgatgaccaaagatgcagagagaggcaagttaaaaagaagggctatcgcaacacctgggatgcagattagaggtttcgtatactttgttgaaactttagctaggagcatttgcgaaaagcttgaacagtctgggctcccagtagggggcaatgaaaagaaggccaaattggcaaatgttgtgagaaagatgatgactaattcacaagatacagagatttctttcacaatcactggggacaacactaagtggaatgaaaatcaaaatcctcgaatgttcctggcgatgattacatatatcaccagaaatcaacccgagtggtttagaaacatcctgagcatggcacccataatgttctcaaataaaatggcaagactaggaaaagggtacatgttcgagagtaaaagaatgaagattcgaacacaaataccagcagaaatgctagcaagcattgatctgaagtatttcaatgaatcaacaaagaagaaaattgagaaaataaggcctcttttaatggatggtacagcatcactgagtcctgggatgatgatgggcatgttcaacatgctaagtacggtcttgggagtctcgatactgaatcttggacaaaagaaatacaccaagacaacatactggtgggatgggctccaatcatccgacgattttgctctcatagtgaatgcaccaaaccatgaaggaatacaagcaggagtggacagattctacaggacctgcaagttagtgggaatcaacatgagcaaaaagaagtcctatataaataagacagggacatttgaattcacaagctttttttaccgctatggatttgtggctaattttagcatggagctacccagctttggagtgtctggagtaaatgaatcagctgacatgagtattggagtaacagtgataaagaacaacatgataaacaatgaccttggacctgcaacggctcagatggctcttcaattgttcatcaaagactacagatacacatataggtgccataggggagacacacaaattcagacgagaagatcatttgagttgaggaagctatgggatcaaacccaatcaaaggtagggctattagtatcagatggaggaccaaacttatacaacatacggaatcttcacattcctgaagtctgcttaaaatgggagctaatggatgatgattatcggggaagactttgtaatcccctgaatccctttgtgagtcataaggagattgattctgtaaacaatgctgtggtaatgccagcccatggcccagccaaaagcatggaatatgatgccgttgctactacacattcctggattcccaagaggaatcgttctattctcaacacaagccaaaggggaattcttgaggatgaacagatgtaccagaagtgttgcaatttattcgagaaatttttccctagcagttcatataggagaccggttggaatttctagcatggtggaggccatggtgtctagggcccggattgatgccagagtcgactttgagtctggacggatcaagaaagaagagttctctgagatcatgaagatctgttccaccattgaagaactcagacggcaaaaataa

>H1N1_PB1_KX915378_A/New Mexico/42/2016

atggatgtcaatccgactctacttttcctaaaaattccagcacaaaatgccataagcaccacattcccttatactggagatcctccatacagccatggaacaggaacaggatacaccatggacacagtaaacagaacacaccaatactcagaaaaggggaagtggacaacaaacacagagactggtgcaccccagctcaacccgattgacggaccactacctgaagataatgaaccaagtgggtatgcacaaacagactgtgttctagaggctatggcttttcttgaagaatcccatccaggaatatttgagaattcatgccttgaaacaatggaagttgttcaacaaacaagggtagataaactgactcaaggtcgccagacttatgattggacattaaacagaaatcaaccggcagcaactgcattggccaacaccatagaagtttttagatcgaatgacctaacagctaacgaatcaggaaggctaatagatttcttaaaggatgtgatggaatcaatgaacaaagaggaaatagagataacaacccactttcaaagaaaaaggagagtaagagacaacatgaccaagaagatggtcacgcaaagaacaatagggaagaaaaaacagagactgaataagagaggctatctaataagagcactgacattaaatacgatgaccaaagatgcagagagaggcaagttaaaaagaagggctatcgcaacacctgggatgcagattagaggtttcgtatactttgttgaaactttagctaggagcatttgcgaaaagcttgaacagtctgggctcccagtagggggcaatgaaaagaaggccaaattggcaaatgttgtgagaaagatgatgactaattcacaagatacagagatttctttcacaatcactggggacaacactaagtggaatgaaaatcaaaatcctcgaatgttcctggcgatgattacatatatcaccagaaatcaacccgagtggtttagaaacatcctgagcatggcacccataatgttctcaaataaaatggcaagactaggaaaagggtacatgttcgagagtaaaagaatgaagattcgaacacaaataccagcagaaatgctagcaagcattgatctgaagtatttcaatgaatcaacaaagaagaaaattgagaaaataaggcctcttttaatggatggcacagcatcactgagtcctgggatgatgatgggcatgttcaacatgctaagtacggtcttgggagtctcgatactgaatcttggacaaaagaaatacaccaagacaacatactggtgggatgggctccaatcatccgacgattttgctctcatagtgaatgcaccaaaccatgaaggaatacaagcaggagtggacagattctacaggacctgcaagttagtgggaatcaacatgagcaaaaagaagtcctatataaataagacagggacatttgaattcacaagctttttttatcgctatggatttgtggctaattttagcatggagctacccagctttggagtgtctggagtaaatgaatcagctgacatgagtattggagtaacagtgataaagaacaacatgataaacaatgaccttggacctgcaacggctcagatggctcttcaattgttcatcaaagactacagatacacatataggtgccataggggagacacacaaattcagacgagaagatcatttgagttgaagaagctatgggatcaaacccaatcaaaggkagggctattagtatcagatggaggaccaaacttatacaacatacggaatcttcacattcctgaagtctgcttaaaatgggagctaatggatgatgattatcggggaagactttgtaatcccctgaatccctttgtgagtcataaggagattgattctgtaaacaatgctgtggtaatgccagcccatggcccagccaaaagcatggaatatgatgccgttgctactacacattcctggattcccaagaggaatcgttctattctcaacacaagccaaaggggaattcttgaggatgaacagatgtaccagaagtgttgcaatttattcgagaaatttttccctagcagttcatataggagaccggttggaatttctagcatggtggaggccatggtgtctagggcccggattgatgccagagtcgactttgagtctggacggatcaagaaagaagagttctctgagatcatgaagatctgttccaccattgaagaactcagacggcaaaaataa

>H1N1_PB1_KX915562_A/Rhode Island/23/2016

atggatgtcaatccgactctacttttcctaaaaattccagcacaaaatgccataagcaccacattcccttacactggagatcctccatacagccatggaacaggaacaggatacaccatggacacagtaaacagaacacaccaatactcagaraaggggaagtggacaacaaacacagagactggtgcaccccagctcaacccgattgacggaccactacctgaagataatgaaccaagtgggtatgcacaaacagactgtgttctagaggctatggcttttcttgaagaatcccatccaggaatatttgagaattcatgccttgaaacaatggaagttgttcaacaaacaagggtagataaactgactcaaggtcgccagacttatgattggacattaaacagaaatcaaccggcagcaactgcattggccaacaccatagaagtttttagatcgaatgacctaacagctaacgagtcaggaaggctaatagatttcttaaaggatgtgatggaatcaatgaacaaagaggaaatagagataacaacccactttcaaagaaaaaggagagtaagagacaacatgaccaagaagatggtcacgcaaagaacaatagggaagaaaaaacagagactgaataagagaggctatctaataagagcactgacattaaatacgatgaccaaagatgcagagagaggcaagttaaaaagaagggctatcgcaacacctgggatgcagattagaggttttgtatactttgttgaaactttagctaggagcatttgcgaaaagcttgaacagtctggactcccagtagggggcaatgaaaagaaggccaaattggcaaatgttgtgagaaagatgatgactaattcacaagatacagagatttctttcacaatcactggggacaacactaagtggaatgaaaatcagaatcctcgaatgttcctggcgatgattacatatatcaccagaaatcaacccgagtggtttagaaacatcctgagcatggcacccataatgttctcaaataaaatggcaagactaggaaaagggtacatgttcgagagtaaaagaatgaagattcgaacacaaataccagcagaaatgctggcaagcattgatctgaagtatttcaatgaatcaacaaagaagaaaattgagaaaataaggcctcttttaatggatggcacagcatcactgagtcctgggatgatgatgggcatgttcaacatgctaagtacggtcttgggagtctcgatactgaatcttggacaaaagaaatacaccaagacaacatactggtgggatgggctccaatcatccgacgattttgctctcatagtgaatgcaccaaaccatgaaggaatacaagcaggagtggacagattctacaggacctgcaagttagtgggaatcaacatgagcaaaaagaagtcctatataaataagacagggacatttgaattcacaagctttttttatcgctatggatttgtggctaattttagcatggagctacccagcttcggagtgtctggagtaaatgaatcagctgacatgagtattggagtaacagtgataaagaacaacatgataaacaatgaccttggacctgcaacggctcagatggcccttcaattgttcatcaaagactacagatacacatataggtgccataggggagacacacaaattcagacgagaagatcatttgagttgaagaagctatgggatcaaacccaatcaaaggtagggctattagtatcagatggaggaccaaacttatacaacatacggaatcttcacattcctgaagtctgcttaaaatgggagctaatggatgatgattatcggggaagactttgtaatcccctgaatccctttgtgagtcataaggagattgattctgtaaacaatgctgtggtaatgccagcccatggcccagccaaaagcatggaatatgatgccgttgctactacacattcctggattcccaagaggaatcgttctattctcaacacaagccaaaggggaattcttgaggatgaacagatgtaccagaagtgttgcaatttattcgagaaatttttccctagcagttcatataggagaccggttggaatttccagcatggtggaggccatggtgtctagggcccggattgatgccagagtcgactttgagtctggacggatcaagaaagaagagttctctgagatcatgaagatctgttccaccattgaagaactcagacggcaaaaataa

>H1N1_PB1_KX915898_A/Connecticut/24/2016

atggatgtcaatccgactctacttttcctaaaaattccagcacaaaatgccataagcaccacattcccttatactggagatcctccatacagccatggaacaggaacaggatacaccatggacacagtaaacagaacacaccaatactcagaaaaggggaagtggacaacaaacacagagactggtgcaccccagctcaacccgattgacggaccactacctgaagataatgaaccaagtgggtatgcacaaacagactgtgttctagaggctatggcttttcttgaagaatcccatccaggaatatttgagaattcatgccttgaaacaatggaagttgttcaacaaacaagggtagataaactgactcaaggtcgccagacttatgattggacattaaacagaaatcaaccggcagcaactgcattggccaacaccatagaagtttttagatcgaatgacctaacagctaacgagtcaggaaggctaatagatttcttaaaggatgtgatggaatcaatgaacaaagaggaaatagagataacaacccactttcaaagaaaaaggagagtaagagacaacatgaccaagaagatggtcacgcaaagaacaatagggaagaaaaaacagagactgaataagagaggctatctaataagagcactgacattaaatacgatgaccaaagatgcagagagaggcaagttaaaaagaagggctatcgcaacacctgggatgcagattagaggtttcgtatactttgttgaaactttagctaggagcatttgcgaaaagcttgaacagtctgggctcccagtagggggcaatgaaaagaaggccaaattggcaaatgttgtgagaaagatgatgactaattcacaagatacagagatttctttcacaatcactggggacaacactaagtggaatgaaaatcaaaatcctcgaatgttcctggcgatgattacatatatcaccagaaatcaacccgagtggtttagaaacatcctgagcatggcacccataatgttctcaaataaaatggcaagactaggaaaagggtacatgttcgagagtaaaagaatgaagattcgaacacaaataccagcagaaatgctagcaagcattgatctgaagtatttcaatgaatcaacaaagaagaaaattgagaaaataaggcctcttttaatggatggcacagcatcactgagtcctgggatgatgatgggcatgttcaacatgctaagtacggtcttgggagtctcgatactgaatcttggacaaaagaaatacaccaagacaacatactggtgggatgggctccaatcatccgacgattttgctctcatagtgaatgcaccaaaccatgaaggaatacaagcaggagtggacagattctacaggacctgcaagttagtgggaatcaacatgagcaaaaagaagtcctatataaataagacagggacatttgaattcacaagctttttttatcgctatggatttgtggctaattttagcatggagctacccagctttggagtgtctggagtaaatgaatcagctgacatgagtattggagtaacagtgataaagaacaacatgataaacaatgaccttggacctgcaacggctcagatggctcttcaattattcatcaaagactacagatacacatataggtgccataggggagacacacaaattcagacgagaagatcatttgagttgaagaaactatgggatcaaacccaatcaaaggtagggctattagtatcagatggaggaccaaacttatacaacatacggaatcttcacattcctgaagtctgcttaaaatgggagctaatggatgatgattatcggggaagactttgtaatcccctgaatccctttgtgagtcataaggagattgattctgtaaacaatgctgtggtaatgccagcccatggcccagccaaaagcatggaatatgatgccgttgctactacacattcctggattcccaagaggaatcgttctattctcaacacaagccaaaggggaattcttgaggatgaacagatgtaccagaagtgttgcaatttattcgagaaatttttccctagcagttcatataggagaccggttggaatttctagcatggtggaggccatggtgtctagggcccggattgatgccagagtcgactttgagtctggacggatcaagaaagaagagttctctgagatcatgaagatctgttccaccattgaagaactcagacggcaaaaataa

>H1N1_PB1_KX919178_A/Georgia/44/2016

atggatgtcaatccgactctacttttcctaaaaattccagcacaaaatgccataagcaccacattcccttatactggagatcctccatacagccatggaacaggaacaggatacaccatggacacagtaaacagaacacaccaatactcagaaaaggggaagtggacaacaaacacagagactggtgcaccccagctcaacccgattgacggaccactacctgaagataatgaaccaagtgggtatgcacaaacagactgtgttctagaggctatggcttttcttgaagaatcccatccaggaatatttgagaattcatgccttgaaacaatggaagttgttcaacaaacaagggtagataaactgactcaaggtcgccagacttatgattggacattaaacagaaatcaaccggcagcaactgcattggccaacaccatagaagtttttagatcgaatgacctaacagctaacgagtcaggaaggctaatagatttcttaaaggatgtgatggaatcaatgaacaaagaggaaatagagataacaacccactttcaaagaaaaaggagagtaagagacaacatgaccaagaagatggtcacgcaaagaacaatagggaagaaaaaacagagactgaataagagaggctatctaataagagcactgacattaaatacgatgaccaaagatgcagagagaggcaagttaaaaagaagggctatcgcaacacctgggatgcagattagaggtttcgtatactttgttgaaactttagctaggagcatttgcgaaaagcttgaacagtctgggctcccagtagggggcaatgaaaagaaggccaaattggcaaatgttgtgagaaagatgatgactaattcacaagatacagagatttctttcacaatcactggggacaacactaagtggaatgaaaatcaaaatcctcgaatgttcctggcgatgattacatatatcaccagaaatcaacccgagtggtttagaaacatcctgagcatggcacccataatgttctcaaataaaatggcaagactaggaaaagggtacatgttcgagagtaaaaaaatgaagattcgaacacaaataccagcagaaatgctagcaagcattgatctgaagtatttcaatgaatcaacaaagaagaaaattgagaaaataaggcctcttttaatggatggcacagcatcactgagtcctgggatgatgatgggcatgttcaacatgctaagtacggtcttgggagtctcgatactgaaccttggacaaaagaaatacaccaagacaacatactggtgggatgggctccaatcatccgacgattttgctctcatagtgaatgcaccaaaccatgaaggaatacaagcaggagtggacagattctacaggacctgcaagttagtgggaatcaacatgagcaaaaagaagtcctatataaataagacagggacatttgaattcacaagctttttttatcgctatggatttgtggctaattttagcatggagctacccagctttggagtgtctggagtaaatgaatcagctgacatgagtattggagtaacagtgataaagaacaacatgataaacaatgaccttggacctgcaacggctcagatggctcttcaattgttcatcaaagactacagatacacatataggtgccatagaggagacacacaaattcaaacgagaagatcatttgagttgaagaagctatgggatcaaacccaatcaaaggtagggctattagtatcagatggaggaccaaacttatacaacatacggaatcttcacattcctgaagtctgcttaaaatgggagctaatggatgatgattatcggggaagactttgtaatcccctgaatccctttgtgagtcataaggagattgattctgtaaacaatgctgtggtaatgccagcccatggcccagccaaaagcatggaatatgatgccgttgctactacacattcctggattcccaagaggaatcgttctattctcaacacaagccaaaggggaattcttgaggatgaacagatgtaccagaagtgttgcaatttattcgagaaatttttccctagcagttcatataggagaccggttggaatttctagcatggtggaggccatggtgtctagggcccggattgatgccagagtcgactttgagtctggacggatcaagaaagaagagttctctgagatcatgaagatctgttccaccattgaagaactcagacggcaaaaataa

>H1N1_PB1_KX919250_A/California/112/2016

atggatgtcaatccgactctacttttcctaaaaattccagcacaaaatgccataagcaccacattcccttatactggagatcctccatacagccatggaacaggaacaggatacaccatggacacagtaaacagaacacaccaatactcagaaaaggggaagtggacaacaaacacagagactggtgcaccccagcttaacccgattgacggaccactacctgaagataatgaaccaagtgggtatgcacaaacagactgtgttctagaggctatggcttttcttgaagaatcccatccaggaatatttgagaattcatgccttgaaacaatggaagttgttcaacaaacaagggtagataaactgactcaaggtcgccagacttatgattggacattaaacagaaatcaaccggcagcaactgcattggccaacaccatagaagtttttagatcgaatgacctaacagctaacgagtcaggaaggctaatagatttcttaaaggatgtgatggaatcaatgaacaaagaggaaatagagataacaacccactttcaaagaaaaaggagagtaagagacaacatgaccaagaagatggtcacgcaaagaacaatagggaagaaaaaacagagactgaataagagaggctatctaataagagcactgacattaaatacgatgaccaaagatgcagagagaggcaagttaaaaagaagggctatcgcaacacctgggatgcagattagaggtttcgtatactttgttgaaactttagctaggagcatttgcgaaaagcttgaacagtctgggctcccagtagggggcaatgaaaagaaggccaaattggcaaatgttgtgagaaagatgatgactaattcacaagatacagagatttctttcacaatcactggggacaacactaagtggaatgaaaatcaaaatcctcgaatgttcctggcgatgattacatatatcaccagaaatcaacccgagtggtttagaaacatcctgagcatggcacccataatgttctcaaataaaatggcaagactaggaaaagggtacatgttcgagagtaaaagaatgaagattcgaacacaaataccagcagaaatgctagcaagcattgatctgaagtatttcaatgaatcaacaaagaagaaaattgagaaaataaggcctcttttaatggatggcacagcatcactgagtcctgggatgatgatgggcatgttcaacatgctaagtacggtcttgggagtctcgatactgaatcttggacaaaagaaatacaccaagacaacatactggtgggatgggctccaatcatccgacgattttgctctcatagtgaatgcaccaaaccatgaaggaatacaagcaggagtggacagattctacaggacctgcaagttagtgggaatcaacatgagcaaaaagaagtcctatataaataagacagggacatttgaattcacaagctttttttatcgctatggatttgtggctaattttagcatggagctacccagctttggagtgtctggagtaaatgaatcagctgacatgagtattggagtaacagtgataaagaacaacatgataaacaatgaccttggacctgcaacggctcagatggctcttcaattgttcatcaaagactacagatacacatataggtgccataggggagacacacaaattcagacgagaagatcatttgagttgaagaagctatgggatcaaacccaatcaaaggtagggctattagtatcagatggaggaccaaacttatacaacatacggaatcttcacattcctgaagtctgcttaaaatgggagctaatggatgatgattatcggggaagactttgtaatcccctgaatccctttgtgagtcataaggagattgattctgtaaacaatgctgtggtaatgccagcccatggcccagccaaaagcatggaatatgatgccgttgctactacacattcctggattcccaagaggaatcgttctattctcaacacaagccaaaggggaattcttgaggatgaacagatgtaccagaagtgttgcaatttattcgagaaatttttccctagcagttcatataggagaccggttggaatttctagcatggtggaggccatggtgtctagggcccggattgatgccagagtcgactttgagtctggacggatcaagaaagaagagttctctgagatcatgaagatctgttccaccattgaagaactcagacggcaaaaataa

>H1N1_PB1_KX919258_A/California/113/2016

atggatgtcaatccgactctacttttcctaaaaattccagcacaaaatgccataagcaccacattcccttatactggagatcctccatacagccatggaacaggaacaggatacaccatggacacagtaaacagaacacaccaatactcagaaaaggggaagtggacaacaaacacagagactggtgcaccccagctcaacccgattgacggaccactacctgaagataatgaaccaagtgggtatgcacaaacagactgtgttctagaggctatggcttttcttgaagaatcccatccaggaatatttgagaattcatgccttgaaacaatggaagttgttcaacaaacaagggtagataaactgactcaaggtcgccagacttatgattggacattaaacagaaatcaaccggcagcaactgcattggccaacaccatagaagtttttagatcgaatggcctaacagctaacgagtcaggaaggctaatagatttcttaaaggatgtgatggaatcaatgaacaaagaggaaatagagataacaacccactttcaaagaaaaaggagagtaagagacaacatgaccaagaagatggtcacgcaaagaacaatagggaagaaaaaacagagactgaataagagaggctatctaataagagcactgacattaaatacgatgaccaaagatgcagagagaggcaagttaaaaagaagggctatcgcaacacctgggatgcagattagaggtttcgtatactttgttgaaactttagctaggagcatttgcgaaaagcttgaacagtctgggctcccagtagggggcaatgaaaagaaggccaaactggcaaatgttgtgagaaagatgatgactaattcacaagatacagagatttctttcacaatcactggggacaacactaagtggaatgaaaatcaaaatcctcgaatgttcctggcgatgattacatatatcaccagaaatcaacccgagtggtttagaaacatcctgagcatggcacccataatgttctcaaataaaatggcaagactaggaaaagggtacatgttcgagagtaaaagaatgaagattcgaacacaaataccagcagaaatgctagcaagcattgatctgaagtatttcaatgaatcaacaaagaagaaaattgagaaaataaggcctcttttaatggatggcacagcatcactgagtcctgggatgatgatgggcatgttcaacatgctaagtacggtcttgggagtctcgatactgaatcttggacaaaagaaatacaccaagacaacatactggtgggatgggctccaatcatccgacgattttgctctcatagtgaatgcaccaaaccatgaaggaatacaagcaggagtggacagattctacaggacttgcaagttagtgggaatcaacatgagcaaaaagaagtcctatataaataagacagggacatttgaattcacaagctttttttatcgctatggatttgtggctaattttagcatggagctacccagctttggagtgtctggagtaaatgaatcagctgacatgagtattggagtaacagtgataaagaacaacatgataaacaatgaccttggacctgcaacggctcagatggctcttcaattgttcatcaaagactacagatacacatataggtgccataggggagacacacaaattcagacgagaagatcatttgagttgaagaagctatgggatcaaacccaatcaaaggtagggctattagtatcagatggaggaccaaacttatacaacatacggaatcttcacattcctgaagtctgcttaaaatgggagctaatggatgatgattatcggggaagactttgtaatcccctgaatccctttgtgagtcataaggagattgattctgtaaacaatgctgtggtaatgccagcccatggcccagccaaaagcatggaatatgatgccgttgctactacacattcctggattcccaagaggaatcgttctattctcaacacaagccaaaggggaattcttgaggatgaacagatgtaccagaagtgttgcaatttattcgagaaatttttccctagcagttcatataggagaccggttggaatttctagcatggtggaggccatggtgtctagggcccggattgatgccagagtcgactttgagtctggacggatcaagaaagaggagttctctgagatcatgaagatctgttccaccattgaagaactcagacggcaaaaataa

>H1N1_PB1_KX919298_A/Hawaii/53/2016

atggatgtcaatccgactctacttttcctaaaaattccagcacaaaatgccataagcaccacattcccttatactggagatcctccatacagccatggaacaggaacaggatacaccatggacacagtaaacagaacacaccaatactcagaaaaggggaagtggactacaaacacagagactggtgcaccccagcttaacccgattgacggaccactacctgaagataatgaaccaagtgggtatgcacaaacagactgtgttctagaggctatggcttttcttgaagaatcccatccaggaatatttgagaattcatgccttgaaacaatggaagttgttcaacaaacaagggtagataaactgactcaaggtcgccagacttatgattggacattaaacagaaatcaaccggcagcaactgcattggccaacaccatagaagtttttagatcgaatgacctaacagctaacgagtcaggaaggctaatagatttcttaaaggatgtgatggaatcaatgaacaaagaggaaatagagataacaacccactttcaaagaaaaaggagagtaagagacaacatgaccaagaagatggtcacgcaaagaacaatagggaagaaaaaacagagactgaataagagaggctatctaataagagcactgacattaaatacgatgaccaaagatgcagagagaggcaagttaaaaagaagggctatcgcaacacctgggatgcagattagaggtttcgtatactttgttgaaactttagctaggagcatttgcgaaaagcttgaacagtctgggctcccagtagggggcaatgaaaagaaggccaaattggcaaatgttgtgagaaagatgatgactaattcacaagatacagagatttctttcacaatcactggggacaacactaaatggaatgaaaatcaaaatcctcgaatgttcctggcgatgattacatatatcaccagaaatcaacccgagtggtttagaaacatcctgagcatggcacccataatgttctcaaataaaatggcaagactaggaaaagggtacatgttcgagagtaaaagaatgaagattcgaacacaaataccagcagaaatgctagcaagcattgatctgaagtatttcaatgaatcaacaaagaagaaaattgagaaaataaggcctcttttaatggatggcacagcatcactgagtcctgggatgatgatgggcatgttcaacatgctaagtacggtcttgggagtctcgatactgaatcttggacaaaagaaatacaccaagacaacatactggtgggatgggctccaatcatccgacgattttgctctcatagtgaatgcaccaaaccatgaaggaatacaagcaggagtggacagattctacaggacctgcaagttagtgggaatcaacatgagcaaaaagaagtcctatataaataagacagggacatttgaattcacaagctttttttatcgctatggatttgtggctaattttagcatggagctacccagctttggagtgtctggagtaaatgaatcagctgacatgagtattggagtaacagtgataaagaacaacatgataaacaatgaccttggacctgcaacggctcagatggctcttcaattgttcatcaaagactacagatacacatataggtgccataggggagacacacaaattcagacgagaagatcatttgagttgaagaagctatgggatcaaacccaatcaaaggtagggctattagtatcagatggaggaccaaacttatacaacatacggaatcttcacattcctgaagtctgcttaaaatgggagctaatggatgatgattatcggggaagactttgtaatcccctgaatccctttgtgagtcataaggagattgattctgtaaacaatgctgtggtaatgccagcccatggcccagccaaaagcatggaatatgatgccgttgctactacacattcctggattcccaagaggaatcgttctattctcaacacaagccaaaggggaattcttgaggatgaacagatgtaccagaagtgttgcaatttattcgagaaatttttccctagcagttcatataggagaccggttggaatttctagcatggtggaggccatggtgtctagggcccggattgatgccagagtcgactttgagtctggacggatcaagaaagaagagttctctgagatcatgaagatctgttccaccattgaagaactcagacggcaaaaataa

>H1N1_PB1_KX919378_A/Texas/144/2016

atggatgtcaatccgactctacttttcctaaaaattccagcacaaaatgccataagcaccacattcccttatactggagatcctccatacagccatggaacaggaacaggatacaccatggacacagtaaacagaacacaccaatactcagaaaaggggaagtggacaacaaacacagagactggtgcaccccagctcaacccgattgacggaccactacctgaagataatgaaccaagtgggtatgcacaaacagactgtgttctagaggctatggcttttcttgaagaatcccatccaggaatatttgagaattcatgccttgaaacaatggaagttgtccaacaaacaagggtagataaactgactcaaggtcgccagacttatgattggacattaaacagaaatcaaccggcagcaactgcattggccaacaccatagaagtttttagatcgaatgacctaacagctaacgagtcaggaaggctaatagatttcttaaaggatgtgatggaatcaatgaacaaagaggaaatagagataacaacccactttcaaagaaaaaggagagtaagagacaacatgaccaagaagatggtcacgcaaagaacaatagggaagaaaaaacagagactgaataagagaggctatctaataagagcactgacattaaatacgatgaccaaagatgcagagagaggcaagttaaaaagaagggctatcgcaacacctgggatgcagattagaggtttcgtatactttgttgaaactttagctaggagcatttgcgaaaagcttgaacagtctgggctcccagtagggggcaatgaaaagaaggccaaattggcaaatgttgtgagaaagatgatgactaattcacaagatacagagatttctttcacaatcactggggacaacactaagtggaatgaaaatcaaaatcctcgaatgttcctggcgatgattacatatatcaccagaaatcaacccgagtggtttagaaacatcctgagcatggcacccataatgttctcaaataaaatggcaagactaggaaaagggtacatgttcgagagtaaaagaatgaagattcgaacacaaataccagcagaaatgctaacaagcattgatttgaagtatttcaatgaatcaacaaagaagaaaattgagaaaataaggcctcttttaatggatggcacagcatcactgagtcctgggatgatgatgggcatgttcaacatgctaagtacggtcttgggagtctcgatactgaatcttggacaaaagaaatacaccaagacaacatactggtgggatgggctccaatcatccgacgattttgctctcatagtgaatgcaccaaaccatgaaggaatacaagcaggagtggacagattctacaggacctgcaagttagtgggaatcaacatgagcaaaaagaagtcctatataaataagacagggacatttgaattcacaagctttttttatcgctatggatttgtggctaattttagcatggagctacccagctttggagtgtctggagtaaatgaatcagctgacatgagtattggagtaacagtgataaagaacaacatgataaacaatgaccttggacctgcaacggctcagatggctcttcaattgttcatcaaagactacagatacacatataggtgccataggggagacacacaaattcagacgagaagatcatttgagttgaagaagctatgggatcaaacccaatcaaaggtagggctattagtatcagatggaggaccaaacttatacaacatacggaatcttcacattcctgaagtctgcttaaaatgggagctaatggatgatgattatcggggaagactttgtaatcccctgaatccctttgtgagtcataaggagattgattctgtaaacaatgctgtggtaatgccagcccatggcccagccaaaagcatggaatatgatgccgttgctactacacattcctggattcccaagaggaatcgttctattctcaacacaagccaaaggggaattcttgaggatgaacagatgtaccagaagtgttgcaatttattcgagaaatttttccctagcagttcatataggagaccggttggaatttctagcatggtggaggctatggtgtctagggcccggattgatgccagagtcgactttgagtctggacggatcaagaaagaagagttctctgagatcatgaagatctgttccaccattgaagaactcagacggcaaaaataa

>H1N1_PB1_KX919426_A/Idaho/30/2016

atggatgtcaatccgactctacttttcctaaaaattccagcacaaaatgccataagcaccacattcccttatactggagatcctccatacagccatggaacaggaacaggatacaccatggacacagtaaacagaacacaccaatactcagaaaaagggaagtggacaacaaacacagagactggtgcaccccagctcaacccgattgacggaccactacctgaagataatgaaccaagtgggtatgcacaaacagactgtgttctagaggctatggcttttcttgaagaatcccatccaggaatatttgagaattcatgccttgaaacaatggaagttgttcaacaaacaagggtagataaactgactcaaggtcgccaaacttatgattggacattaaacagaaatcaaccggcagcaactgcattggccaacaccatagaagtttttagatcgaatgacctaacagctaacgagtcaggaaggctaatagatttcttaaaggatgtgatggaatcaatgaacaaagaggaaatagagataacaacccactttcaaagaaaaaggagagtaagagacaacatgaccaagaagatggtcacgcaaagaacaatagggaagaaaaaacagagactgaataagagaggctatctaataagagcactgacattaaatacgatgaccaaagatgcagagagaggcaagttaaaaagaagggctatcgcaacacctgggatgcagattagaggtttcgtatactttgttgaaactttagctaggagcatttgcgagaagcttgaacagtctgggctcccagtagggggcaatgaaaagaaggccaaattggcaaatgttgtgagaaagatgatgactaattcacaagatacagagatttctttcacaatcactggggacaacactaagtggaatgaaaatcaaaatcctcgaatgttcctggcgatgattacatatatcaccagaaatcaacccgagtggtttagaaacatcctgagcatggcacccataatgttctcaaataaaatggcaagactaggaaaagggtacatgttcgagagtaaaagaatgaagattcgaacacaaataccagcagaaatgctagcaagcattgatctgaagtatttcaatgaatcaacaaagaagaaaattgagaaaataaggcctcttttaatggatggcacagcatcactgagtcctgggatgatgatgggcatgttcaacatgctaagtacggtcttgggagtctcgatattgaatcttggacaaaagaaatacaccaagacaacatactggtgggatgggctccaatcatccgacgattttgctctcatagtgaatgcaccaaaccatgaaggaatacaagcaggagtggacagattctacaggacctgcaagttagtgggaatcaacatgagcaaaaagaagtcctatataaataagacagggacatttgaattcacaagctttttttatcgctatggatttgtggctaattttagcatggagctacccagctttggagtgtctggagtaaatgaatcagctgacatgagtattggagtaacagtgataaagaacaacatgataaacaatgaccttggacctgcaacggctcagatggctcttcaattgttcatcaaagactacagatacacatataggtgccataggggagacacacaaattcagacgagaagatcatttgagttgaagaagctatgggatcaaacccaatcaaaggtagggctattagtatcagatggaggaccaaacttatacaacatacggaatcttcacattcctgaagtctgcttaaaatgggagctaatggatgatgattatcggggaagactttgtaatcccctgaatccctttgtgagtcataaggagattgattctgtaaacaatgctgtggtaatgccagcccatggcccagccaaaagcatggaatatgatgccgttgctactacacattcctggattcccaagaggaatcgttctattctcaacacaagccaaaggggaattcttgaggatgaacagatgtaccagaagtgttgcaatttattcgagaaatttttccctagcagttcatataggagaccggttggaatttctagcatggtggaggccatggtgtctagggcccggattgatgccagagtcgactttgagtctggacggatcaagaaagaagagttctctgagatcatgaagatctgttccaccattgaagaactcagacggcaaaaataa

>H1N1_PB1_KY003252_A/California/104/2016

atggatgtcaatccgactctacttttcctaaaaattccagcacaaaatgccataagcaccacattcccttatactggagatcctccatacagccatggaacaggaacaggatacaccatggacacagtaaacagaacacaccaatactcagaaaaggggaagtggacaacaaacacagagactggtgcaccccagctcaacccgattgacggaccactacctgaagataatgaaccaagtgggtatgcacaaacagactgtgttctagaggctatggcttttcttgaagaatcccatccaggaatatttgagaattcatgccttgaaacaatggaagttgttcaacaaacaagggtagataaactgactcaaggtcgccagacttatgattggacattaaacagaaatcaaccggcagcaactgcattggccaacaccatagaagtttttagatcgaatgacctaacagctaacgagtcaggaaggctaatagatttcttaaaggatgtgatggaatcaatgaacaaagaggaaatagagataacaacccactttcaaagaaaaaggagagtaagagacaacatgaccaagaagatggtcacgcaaagaacaatagggaagaaaaaacagagactgaataagagaggctatctaataagagcactgacattaaatacgatgaccaaagatgcagagagaggcaagttaaaaagaagggctatcgcaacacctgggatgcagattaggggtttcgtatactttgttgaaactttagctaggagcatttgcgaaaagcttgaacagtctgggctcccagtagggggcaatgaaaagaaggccaaattggcaaatgttgtgagaaagatgatgactaattcacaagatacagagatttctttcacaatcactggggacaacactaagtggaatgaaaatcaaaatcctcgaatgttcctggcgatgattacatatatcaccagaaatcaacccgagtggtttagaaacatcctgagcatggcacccataatgttctcaaataaaatggcaagactaggaaaagggtacatgttcgagagtaaaagaatgaagattcgaacacaaataccagcagaaatgctagcaagcattgatctgaagtatttcaatgaatcaacaaagaagaaaattgagaaaataaggcctcttttaatggatggcacagcatcactgagtcctgggatgatgatgggcatgttcaacatgctaagtacggtcttgggagtctcgatactgaatcttggacaaaagaaatacaccaagacaacatactggtgggatgggctccaatcatccgacgattttgctctcatagtgaatgcaccaaaccatgaaggaatacaagcaggagtggacagattctacaggacctgcaagttagtgggaatcaacatgagcaaaaagaagtcctatataaataagacagggacatttgaattcacaagctttttttatcgctatggatttgtggctaattttagcatggagctacccagctttggagtgtctggagtaaatgaatcagctgacatgagtattggagtaacagtgataaagaacaacatgataaacaatgaccttggacctgcaacggctcagatggctcttcaattgttcatcaaagactacagatacacatataggtgccataggggagacacacaaattcagacgagaagatcatttgagttgaagaagctatgggatcaaacccaatcaaaggtagggctattagtatcagatggaggaccaaacttatacaacatacggaatcttcacattcctgaagtctgcttaaaatgggagctaatggatgatgattatcggggaagactttgtaatcccctgaatccctttgtgagtcataaggagattgattctgtaaacaatgctgtggtaatgccagcccatggcccagccaaaagcatggaatatgatgccgttgctactacacattcctggattcccaagaggaatcgttctattctcaacacaagccaaaggggaattcttgaggatgaacagatgtaccagaagtgttgcaatttattcgagaaatttttccctagcagttcatataggagaccggttggaatttctagcatggtggaggccatggtgtctagggcccggattgatgccagagtcgactttgagtctggacggatcaagaaagaagagttctctgagatcatgaagatctgttccaccattgaagaactcagacggcaaaaataa

>H1N1_PB1_KY003313_A/Hawaii/66/2016

atggatgtcaatccgactctacttttcctaaaaattccagcacaaaatgccataagcaccacattcccttatactggagatcctccatacagccatggaacaggaacaggatacaccatggacacagtaaacagaacacaccaatactcagaaaaggggaagtggacaacaaacacagagactggtgcaccccagctcaacccgattgacggaccactacctgaagataatgaaccaagtgggtacgcacaaacagactgtgttctagaggctatggcttttcttgaagaatcccatccaggaatatttgagaattcatgccttgaaacaatggaagttgttcaacaaacaagggtagataaactgactcaaggtcgccagacttatgattggacattaaacagaaatcaaccggcagcaactgcattggccaacaccatagaagtttttagatcgaatgacctaacagctaacgagtcaggaaggctaatagatttcttaaaggatgtgatggaatcaatgaacaaagaggaaatagagataacaacccactttcaaagaaaaaggagagtaagagacaacatgaccaagaagatggtcacgcaaagaacaatagggaagaaaaaacagagactgaataagagaggctatctaataagagcactgacattaaatacaatgaccaaagatgcagagagaggcaagttaaaaagaagggctattgcaacacctgggatgcagattagaggttttgtatactttgttgaaactttagctaggagcatttgcgaaaagcttgaacagtctgggctcccagtagggggcaatgaaaagaaggccaaattggcaaatgttgtgagaaagatgatgactaattcacaagatacagagatttctttcacaatcactggggacaacactaagtggaatgaaaatcaaaatcctcgaatgttcctggcgatgattacatatatcaccagaaatcaacccgagtggtttagaaacatcctgagcatggcacccataatgttctcaaataaaatggcaagactaggaaaagggtacatgttcgagagtaaaagaatgaagattcgaacacaaataccagcagaaatgctagcaagcattgatctgaagtatttcaatgaatcaacaaagaagaaaattgagaaaataaggcctcttttaatggatggcacagcatcactgagtcctgggatgatgatgggcatgttcaacatgctaagtacggtcttgggagtctcgatactgaatcttggacaaaagaaatacaccaagacaacatactggtgggatgggctccaatcatccgacgattttgctctcatagtgaatgcaccaaaccatgaaggaatacaagcaggagtggacagattctacaggacctgcaagttagtgggaatcaacatgagcaaaaagaagtcctatataaataagacagggacatttgaattcacaagctttttttatcgctatggatttgtggctaattttagcatggagctacccagctttggagtgtctggagtaaatgaatcagctgacatgagtattggagtaacagtgataaagaacaacatgataaacaatgaccttggacctgcaacggctcagatggctcttcaattgttcatcaaagactacagatacacatataggtgccataggggagacacacaaattcagacgagaagatcatttgagttgaagaagctatgggatcaaacccaatcaaaggtagggctattagtatcagatggaggaccaaacttatacaacatacggaatcttcacattcctgaagtctgcttaaaatgggagctaatggatgatgattatcggggaagactttgtaatcccctgaatccctttgtgagtcataaggagattgattctgtaaacaatgctgtggtaatgccagcccatggcccagccaaaagcatggaatatgatgccgttgctactacacattcctggattcccaagaggaatcgttctattctcaacacaagccaaaggggaattcttgaggatgaacagatgtaccagaagtgttgcaatttattcgagaaatttttccctagcagttcatataggagaccggttggaatttctagcatggtggaggccatggtgtctagggcccggattgatgccagagtcgactttgagtctggacggatcaagaaagaagagttctctgagatcatgaagatctgttccaccattgaagaactcagacggcaaaaataa

>H1N1_PB1_KY045185_A/Colorado/17/2016

atggatgtcaatccgactctacttttcctaaaaattccagcacaaaatgccataagcaccacattcccttatactggagatcctccatacagccatggaacaggaacaggatacaccatggacacagtaaacagaacacaccaatactcagaaaaggggaagtggacaacaaacacagagactggtgcaccccagctcaacccgattgatggaccactacctgaggataatgaaccaagtgggtatgcacagacagactgtgtcctagaggctatggctttccttgaagaatcccatccaggaatatttgagaattcatgccttgaaacaatggaagttgttcaacaaacaagggtagataaactgactcaaggtcgccagacttatgattggacattaaacagaaatcaaccagcagcaactgcattggccaacaccatagaagtctttagatcgaatgacctaacagctaacgagtcaggaaggctaatagatttcttaaaggatgtaatggaatcaatgaacaaagaggaaatagagataacaacccacttccaaagaaaaaggagagtaagagacaacatgaccaagaagatggtcacgcaaagaacaatagggaagaaaaaacagagactgaataagagaggctatctaataagagcactgacattaaatacgatgaccaaagatgcagagagaggcaagttaaaaagaagggctatcgcaacacctgggatgcagattagaggtttcgtatactttgttgaaactttagctaggagcatttgcgaaaagcttgaacagtctgggctcccagtagggggcaatgaaaagaaggccaaattggcaaatgttgtgagaaagatgatgactaattcacaagatacagagatttctttcacaatcactggggacaacactaaatggaatgaaaatcaaaatcctcgaatgttcctggcaatgattacatatatcaccagaaatcaacccgagtggtttagaaacatcctgagcatggcacccataatgttctcaaataaaatggcaagactggggaaagggtacatgttcgagagtaaaagaatgaaaattcgaacacaaataccagcagaaatgctagcaagcattgatctgaagtacttcaatgaatcaacaaagaagaaaattgagaaaataaggcctcttttaatggatggcacagcatcactgagtcctgggatgatgatgggcatgttcaacatgctaagtacggtcttgggagtctcgatactgaatcttggacaaaagaaatacaccaagacaacatactggtgggatgggctccaatcatccgacgattttgctctcatagtgaatgcaccaaaccacgaaggaatacaagcaggagtggacagattctacaggacctgcaagttagtgggaatcaacatgagcaaaaagaagtcttatataaataagacagggacatttgaattcacaagctttttttatcgctatggatttgtggctaattttagcatggagctacccagctttggagtgtctggagtaaatgaatcagctgacatgagtattggagtaacagtgataaagaacaacatgataaacaatgaccttggacctgcaacggcccagatggctcttcaattgttcatcaaagactacagatacacatataggtgccacaggggagacacacaaattcagacgagaagatcatttgagttaaagaagctatgggatcaaacccaatcaaaggtagggctattagtatcagatggaggaccaaacttatacaacatacggaatcttcacattcctgaagtctgcttaaaatgggagctaatggatgatgattatcggggaagactttgtaatcccctgaatccctttgtgagtcataaggagattgattctgtaaacaatgccgtggtaatgccagcccatggtccagccaaaagcatggaatatgatgccgttgcaactacacattcctggattcccaagaggaatcgttctatcctcaacacaagccaaaggggaattcttgaggatgaacagatgtaccagaagtgttgcaatctattcgagaaatttttccctagcagttcatataggagaccggttggaatttctagcatggtggaggccatggtgtctagggcccggattgatgccagggtcgactttgagtctggacggatcaagaaagaagagttctctgagatcatgaagatctgttccaccattgaagaactcagacggcaaaaataa

>H1N1_PB1_KY289714_A/Montana/51/2016

atggatgtcaatccgactctacttttcttaaaaattccagcacaaaatgccataagcaccacattcccttatactggagatcctccatatagccatggaacaggaacaggatacactatggacacagtaaacagaacacaccaatactcagaaaaggggaagtggacaacaaacacagagactggtgcaccccagctcaacccgattgatggaccactacctgaggataatgaaccaagtgggtatgcacaaacagactgtgttctagaggctatggctttccttgaagaatcccacccaggaatatttgagaattcatgccttgaaacaatggaagttgttcaacaaacaagggtagataaactgactcaaggtcgccagacttatgattggacattaaacagaaatcaaccggcagcaactgcattggccaacaccatagaagtctttagatcgaatgacctaacagctaacgagtcaggaaggctaatagatttcttaaaggatgtaatggaatcaatgaacaaagaggaaatagagataacaacccactttcaaagaaaaaggagagtaagagacaacatgaccaagaagatggtcacgcaaagaacaatagggaagaaaaaacagagactgaataagagaggctatctaataagagcactgacattaaatacgatgaccaaagatgcagagagaggcaagttaaaaagaagggctatcgcaacacctgggatgcagattagaggtttcgtatactttgttgaaactttagctaggagcatttgcgaaaagcttgaacagtctgggctcccagtagggggcaatgaaaagaaggccaaattggcaaatgttgtgagaaagatgatgactaattcacaagatacagagatttctttcacaatcactggggacaacactaagtggaatgaaaatcaaaatcctcgaatgttcctggcgatgattacatatatcaccagaaatcaacccgagtggtttagaaacatcctgagcatggcacccataatgttctcaaataaaatggcaagactgggaaaagggtacatgttcgagagtaaacgaatgaagattcgaacacaaataccagcagaaatgctagcaagcattgatctgaagtacttcaatgaatcaacaaagaagaaaattgagaaaataaggcctcttttaatggatggcacagcatcactgagtcctgggatgatgatgggcatgttcaacatgctaagtacggtattgggagtctcgatactgaatcttggacaaaagaaatacaccaagacaacatactggtgggatgggctccaatcatccgacgattttgctctcatagtgaatgcaccaaaccacgaaggaatacaagcaggagtggacaggttctacaggacctgcaagttagtgggaatcaacatgagcaaaaagaagtcctatataaataagacagggacatttgaattcacaagctttttttatcgctatggatttgtggctaattttagcatggagctacccagctttggagtgtctggagtaaatgaatcagctgacatgagtattggagtaacagtgataaagaacaacatgataaacaatgaccttggacctgcaacggcccagatggctcttcaattgttcatcaaagactacagatacacatataggtgccataggggggacacacaaattcagacgagaagatcttttgagttaaagaagctatgggatcaaacccaatcaaaggtagggctattagtatcagatggaggaccaaacttatacaacatacggaatcttcacattcctgaagtctgcttaaaatgggagctaatggatgatgattatcggggaagactttgtaatcccctgaatccctttgtgagtcataaggaaattgattctgtaaacaatgccgtggtaatgccagcccatggtccagccaaaagcatggaatatgatgccgttgcaactacacattcctggattcccaagaggaatcgttctattctcaacacaagccaaaggggaattcttgaggatgaacagatgtaccagaagtgttgcaatctattcgagaaatttttccctagcagttcatataggagaccggttggaatttctagcatggtggaggccatggtgtctagggcccggattgatgccagagtcgactttgagtctggacggatcaagaaagaagagttctctgagatcatgaagatctgttccaccattgaagaactcagacggcaaaaataa

>H1N1_PB1_KY487194_A/Baltimore/0092/2016

atggatgtcaatccgactctacttttcctaaaaattccagcacaaaatgccataagcaccacattcccttatactggagatcctccatacagccatggaacaggaacaggatacaccatggacacagtaaacagaacacaccaatactcagaaaaagggaagtggacaacaaacacagagactggtgcaccccagctcaacccgattgacggaccactacctgaagataatgaaccaagtgggtatgcacaaacagactgtgttctagaggctatggcttttcttgaagaatcccatccaggaatatttgagaattcatgccttgaaacaatggaagttgttcaacaaacaagggtagataaactgactcaaggtcgccagacttatgattggacattaaacagaaatcaaccggcagcaactgcattggccaacaccatagaagtttttagatcgaatgacctaacagctaacgagtcaggaaggctaatagatttcttaaaggatgtgatggaatcaatgaacaaagaggaaatagagataacaacccactttcaaagaaaaaggagagtaagagacaacatgaccaagaagatggtcacgcaaagaacaatagggaagaaaaaacagagactgaataagagaggctatctaataagagcactgacattaaatacgatgaccaaagatgcagagagaggcaagttaaaaagaagggctatcgcaacacctgggatgcagattagaggtttcgtatactttgttgaaactttagctaggagcatttgcgaaaagcttgaacagtctgggctcccagtagggggcaatgaaaagaaggccaaattggcaaatgttgtgagaaagatgatgactaattcacaagatacagagatttctttcacaatcactggggacaacactaagtggaatgaaaatcaaaatcctcgaatgttcctggcgatgattacatatatcaccagaaatcaacccgagtggtttagaaacatcctgagcatggcacccataatgttctcaaataaaatggcaagactaggaaaagggtacatgttcgagagtaaaagaatgaagattcgaacacaaataccagcagaaatgctagcaagcattgatctgaagtatttcaatgaatcaacaaagaagaaaattgagaaaataaggcctcttttaatggatggcacagcatcactgagtcctgggatgatgatgggcatgttcaacatgctaagtacggtcttgggagtctcgatattgaatcttggacaaaagaaatacaccaagacaacatactggtgggatgggctccaatcatccgacgattttgctctcatagtgaatgcaccaaaccatgaaggaatacaagcaggagtggacagattctacaggacctgcaagttagtgggaatcaacatgagcaaaaagaagtcctatataaataagacagggacatttgaattcacaagctttttttatcgctatggatttgtggctaattttagcatggagctacccagctttggagtgtctggagtaaatgaatcagctgacatgagtattggagtaacagtgataaagaacaacatgataaacaatgaccttggacctgcaacggctcagatggctcttcaattgttcatcaaagactacagatacacatataggtgccataggggagacacacaaattcagacgagaagatcatttgagttgaagaagctatgggatcaaacccaatcaaaggtagggctattagtatcagatggaggaccaaacttatacaacatacggaatcttcacattcctgaagtctgcttaaaatgggagctaatggatgatgattatcggggaagactttgtaatcccctgaatccctttgtgagtcataaggagattgattctgtaaacaatgctgtggtaatgccagcccatggcccagccaaaagcatggaatatgatgccgttgctactacacattcctggattcccaagaggaatcgttctattctcaacacaagccaaaggggaattcttgaggatgaacagatgtaccagaagtgttgcaatttattcgagaaatttttccctagcagttcatataggagaccggttggaatttctagcatggtggaggccatggtgtctagggcccggattgatgccagagtcgactttgagtctggacggatcaagaaagaagagttctctgagatcatgaagatctgttccaccattgaagaactcagacggcaaaaataa

>H1N1_PB1_KY487538_A/Taipei/0040/2016

atggatgtcaatccgactctacttttcctaaaaattccagcacaaaatgccataagcaccacattcccttatactggagatcctccatacagccatggaacaggaacaggatacaccatggacacagtaaacagaacacaccaatactcagaaaaggggaagtggacaacaaacacagagactggtgcaccccagctcaacccgattgacggaccactacctgaagataatgaaccaagtgggtatgcacaaacagactgtgttctagaggctatggcttttcttgaagaatcccatccaggaatatttgagaattcatgccttgaaacaatggaagttgttcaacaaacaagggtagataaactgactcaaggtcgccagacttatgattggacattaaacagaaatcaaccggcagcaactgcattggccaacaccatagaagtttttagatcgaatgacctaacagctaacgagtcaggaaggctaatagatttcttaaaggatgtgatggaatcaatgaacaaagaggaaatagagataacaacccactttcaaagaaaaaggagagtaagagacaacatgaccaagaagatggtcacgcaaagaacaatagggaagaaaaaacagagactgaataagagaggctatctaataagagcactgacattaaatacgatgaccaaagatgcagagagaggcaagttaaaaagaagggctatcgcaacacctgggatgcagattagaggtttcgtatactttgttgaaactttagctaggagcatttgcgaaaagcttgaacagtctgggctcccagtagggggcaatgaaaagaaggccaaattggcaaatgttgtgagaaagatgatgactaattcacaagatacagagatttctttcacaatcactggggacaacactaagtggaatgaaaatcaaaatcctcgaatgttcctggcgatgattacatatatcaccagaaatcaacccgagtggtttagaaacatcctgagcatggcacccataatgttctcaaataaaatggcaaggctaggaaaagggtacatgttcgagagtaaaagaatgaagattcgaacacaaataccagcagaaatgctagcaagcattgatctgaagtatttcaatgaatcaacaaagaagaaaattgagaaaataaggcctcttttaatggatggcacagcatcactgagtcctgggatgatgatgggcatgttcaacatgctaagtacggtcttgggagtctcgatactgaatcttggacaaaagaaatacaccaagacaacatactggtgggatgggctccaatcatccgacgattttgctctcatagtgaatgcaccaaaccatgaaggaatacaagcaggagtggacagattctacaggacctgcaagttagtgggaatcaacatgagcaaaaagaagtcctatataaataagacagggacatttgaattcacaagctttttttatcgctatggatttgtggctaattttagcatggagctacccagctttggagtgtctggagtaaatgaatcagctgacatgagtattggagtaacagtgataaagaacaacatgataaacaatgaccttggacctgcaacggctcagatggctcttcaattgttcatcaaagactacagatacacatataggtgccataggggagacacacaaattcagacgagaagatcatttgagttgaagaagctatgggatcaaacccaatcaaaggtagggctattagtatcagatggaggaccaaacttatacaacatacggaatcttcacattcctgaagtctgcttaaaatgggagctaatggatgatgattatcggggaagactttgtaatcccctgaatccctttgtgagtcataaggagattgattctgtaaacaatgctgtggtaatgccagcccatggcccagccaaaagcatggaatatgatgccgttgctactacacattcctggattcccaagaggaatcgttctattctcaacacaagccaaaggggaattcttgaggatgaacagatgtaccagaagtgttgcaatttattcgagaaatttttccctagcagttcatataggagaccggttggaatttctagcatggtggaggccatggtgtctagggcccggattgatgccagagtcgactttgagtctggacggatcaagaaagaagagttctctgagatcatgaagatctgttccaccattgaagaactcagacggcaaaaataa

>H1N1_PB1_CY217095_A/Maryland/06/2017

atggatgtcaatccgactctacttttcctaaaaattccagcacaaaatgccataagcaccacattcccttatactggagatcctccatacagccatggaacaggaacaggatacaccatggacacagtaaacagaacacaccaatactcagaaaaggggaagtggacaacaaacacagagactggtgcaccccagctcaacccgattgacggaccactacctgaagataatgaaccaagtgggtatgcacaaacagactgtgttctagaggctatggcttttcttgaagaatctcatccaggaatatttgagaattcatgccttgaaacaatggaagttgttcaacaaacaagggtagataaactgactcaaggtcgccagacttatgattggacattaaacagaaatcaaccggcagcaactgcattggccaacaccatagaagtttttagatcgaatgacctaacagctaacgagtcaggaaggctaatagatttcttaaaggatgtgatggaatcaatgaacaaagaagaaatagagataacaacccactttcaaagaaaaaggagagtaagagacaacatgaccaagaagatggtcacgcaaagaacaatagggaagaaaaaacagagactgaataagagaggctatctaataagagcactgacattaaatacgatgaccaaagatgcagagagaggcaagttaaaaagaagggctatcgcaacacctgggatgcagattagaggtttcgtatactttgttgaaactttagctaggagcatttgcgaaaagcttgaacagtctgggctcccagtagggggcaatgaaaagaaggccaaattggcaaatgttgtgagaaagatgatgactaattcacaagatacagagatttctttcacaatcactggggacaacactaagtggaatgaaaatcaaaatcctcgaatgttcctggcgatgattacatatatcaccagaaatcaacccgagtggtttagaaacatcctgagcatggcacccataatgttctcaaataaaatggcaagactaggaaaagggtacatgttcgagagtaaaagaatgaagattcgaacacaaataccagcagaaatgctagcaagcattgatctgaagtatttcaatgaatcaacaaagaagaaaattgagaaaataaggcctcttttaatggatggcacagcatcactgagtcctgggatgatgatgggcatgttcaacatgctaagtacagtcttgggagtctcgatactgaatcttggacaaaagaaatacaccaagacaacatactggtgggatgggctccaatcatccgacgattttgctctcatagtgaatgcaccaaaccatgaaggaatacaagcaggagtggacagattctacaggacttgcaagttagtgggaatcaacatgagcaaaaagaagtcctatataaataagacagggacatttgaattcacaagctttttttatcgctatggatttgtggctaattttagcatggagctacctagctttggagtgtctggagtaaatgaatcagctgacatgagtattggagtaacagtgataaagaacaacatgataaacaatgaccttggacctgcaacggctcaaatggctcttcaattgttcatcaaagactacagatacacatataggtgccataggggagacacacaaattcagacgagaagatcatttgagttgaagaagctatgggatcaaacccaatcaaaggtagggctattagtatcagatggaggaccaaacttatacaacatacggaatcttcacattcctgaagtctgcttaaaatgggagctaatggatgatgattatcggggaagactctgtaatcccctgaatccctttgtgagtcataaggagattgattctgtaaacaatgctgtggtaatgccagcccatggcccagccaaaagcctggaatatgatgccgttgctactacacattcctggattcccaagaggaatcgttctattctcaacacaagccaaaggggaattcttgaggatgaacagatgtaccagaagtgttgcaatttattcgagaaatttttccctagcagttcatataggagaccggttggaatttctagcatggtggaggccatggtatctagggcccggattgatgccagagtcgactttgagtctggacggatcaagaaagaagagttctctgagatcatgaagatctgttccaccattgaagaactcagacggcaaaaataa

>H1N1_PB1_CY218879_A/Texas/22/2017

atggatgtcaatccgactctacttttcctaaaaattccagcacaaaatgccataagcaccacattcccttatactggagatcctccatacagccatggaacaggaacaggatacaccatggacacagtaaacagaacacaccaatactcagaaaaggggaagtggacaacaaacacagagactggtgcaccccagctcaacccaattgacggaccactacctgaagataatgaaccaagtgggtatgcacaaacagactgtgttctagaggctatggcttttcttgaagaatcccatccaggaatatttgagaattcatgccttgaaacaatggaagttgttcaacaaacaagggtagataaactgactcaaggtcgccagacttatgattggacattaaacagaaatcaaccggcagcaactgcattggccaacaccatagaagtttttagatcgaatgacctaacagctaacgagtcaggaaggctaatagatttcttaaaggatgtgatggaatcaatgaacaaagaggaaatagagataacaacccactttcaaagaaaaaggagagtaagagacaacatgaccaagaagatggtcacgcaaagaacaatagggaagaaaaaacagagactgaataagagaggctatctaataagagcactgacattaaatacgatgaccaaagatgcagagagaggcaagttaaaaagaagggctatcgcaacacctgggatgcagattagaggtttcgtatactttgttgaaactttagctaagagcatttgcgaaaagcttgaacagtctgggcttccagtagggggcaatgaaaagaaggccaaattggcaaatgttgtgagaaagatgatgactaattcacaagatacagagatttctttcacaatcactggggacaacactaagtggaatgaaaatcaaaatcctcgaatgttcctggcgatgattacatatatcaccagaaatcaacccgagtggtttagaaacatcctgagcatggcacccataatgttctcaaataaaatggcaagactaggaaaagggtacatgttcgagagtaaaagaatgaagattcgaacacaaataccagcagaaatgctagcaagcattgatctgaagtatttcaatgaatcaacaaagaagaaaattgagaaaataaggcctcttttaatggatggcacagcatcactgagtcctgggatgatgatgggcatgttcaacatgctaagtacggtcttgggagtctcgatactgaatcttggacaaaagaaatacaccaagacaacatactggtgggatgggctccaatcatccgacgattttgctctcatagtgaatgcaccaaaccatgaaggaatacaagcaggagtggacagattctacaggacctgcaagttagtgggaatcaacatgagcaaaaagaagtcctatataaataagacagggacatttgaattcacaagctttttttatcgctatggatttgtggctaattttagcatggagctacccagctttggagtgtctggagtgaatgaatcagctgacatgagtattggagtaacagtgataaagaacaacatgataaacaatgaccttggacctgcaacggctcagatggctcttcaattgttcatcaaagactacagatacacatataggtgccataggggagacacacaaattcagacgagaagatcatttgagttgaagaagctatgggaccaaacccaatcaaaggtagggctattagtatcagatggaggaccaaacttatacaacatacggaatcttcacattcctgaagtctgcttaaaatgggagctaatggatgatgattatcggggaagactttgtaatcccctgaatccctttgtgagtcataaggagattgattctgtaaacaatgctgtggtaatgccagcccatggcccagccaaaagcatggaatatgatgccgttgctactacacattcctggattcccaagaggaatcgttctattctcaacacaagccaaaggggaattcttgaggatgaacagatgtaccagaagtgttgcaatttattcgagaaatttttccctagcagttcatataggagaccagttggaatttctagcatggtggaggccatggtgtctagggcccggattgatgccagagtcgactttgagtctggacggatcaagaaagaagagttctccgagatcatgaagatctgttccaccattgaagaactcagacggcaaaaataa

>H1N1_PB1_CY220821_A/California/19/2017

atggatgtcaatccgactctacttttcttaaaaattccagcacaaaatgccataagcaccacattcccttatactggagatcctccatatagccatggaacaggaacaggatacaccatggacacagtaaacagaacacaccaatactcagaaaaggggaagtggacaacaaacacagagactggtgcaccccagctcaacccgattgatggaccactacctgaggataatgaaccaagtgggtatgcacaaacagactgtgttctagaggctatggctttccttgaagaatcccacccaggaatatttgagaattcatgccttgaaacaatggaagttgttcaacaaacaagggtagataaactgactcaaggtcgccagacttatgattggacattaaacagaaatcaaccggcagcaactgcattggccaacaccatagaagtctttagatcgaatgacctaacagctaacgagtcaggaaggctaatagatttcttaaaggatgtaatggaatcaatgaacaaagaggaaatagagataacaacccactttcaaagaaaaaggagagtaagagacaacatgaccaagaagatggtcacgcaaagaacaatagggaagaaaaaacagagactgaataagagaggctatctaataagagcactgacattaaatacgatgaccaaagatgcagagagaggcaagttaaaaagaagggctatcgcaacacctgggatgcagattagaggtttcgtatactttgttgaaactttagctaggagcatttgcgaaaagcttgaacagtctgggctcccagtagggggcaatgaaaagaaggccaaattggcaaatgttgtgagaaagatgatgactaattcacaagatacagagatttctttcacaatcactggggacaacactaagtggaatgaaaatcaaaatcctcgaatgttcctggcgatgattacatatatcaccagaaatcaacccgagtggtttagaaacatcctgagcatggcacccataatgttctcaaataaaatggcaagactggggaaagggtacatgttcgagagtaaaagaatgaagattcgaacacaaataccagcagaaatgctagcaagcattgatctgaagtacttcaatgaatcaacaaagaagaaaattgagaaaataaggcctcttttaatggatggcacagcatcactgagccctgggatgatgatgggcatgttcaacatgctaagtacggtattgggagtctcgatactgaatcttggacaaaagaaatacaccaagacaacatactggtgggatgggctccaatcatccgacgattttgctctcatagtgaatgcaccaaaccatgaaggaatacaagcaggagtggacaggttctacaggacctgcaagttagtgggaatcaacatgagcaaaaagaagtcctatataaataagacagggacatttgaattcacaagctttttttatcgctatggatttgtggctaattttagcatggagctacccagctttggagtgtctggagtaaatgaatcagctgacatgagtattggagtaacagtgataaagaacaacatgataaacaatgaccttggacctgcaacggcccagatggctcttcaattgttcatcaaagactacagatacacatataggtgccataggggagacacacaaattcagacgaggagatcttttgagttaaagaagctatgggatcaaacccaatcaaaggtagggctattagtatcagatggaggaccaaacttatacaacatacggaatcttcacattcctgaagtctgcttaaaatgggagctaatggatggtgattatcggggaagactttgtaatcccctgaatccctttgtgagtcataaggagattgattctgtaaacaatgccgtggtaatgccagcccatggtccagccaaaagcatggaatatgatgccgttgcaactacacattcctggatccccaagaggaatcgttctattctaaacacaagccaaaggggaattcttgaggatgaacagatgtaccagaagtgttgcaatctattcgagaaatttttccctagcagttcatataggagaccggttggaatttctagcatggtggaggccatggtgtctagggcccagattgatgccagagtcgactttgagtctggacggatcaagaaagaagagttctctgagatcatgaagatctgttccaccattgaagaactcagacggcaaaaataa

>H1N1_PB1_CY225428_A/Maryland/08/2017

atggatgtcaatccgactctacttttcctaaaaattccagcacaaaatgccataagcaccacattcccttatactggagatcctccatacagccatggaacaggaacaggatacaccatggacacagtaaacagaacacaccaatactcagaaaaggggaagtggacaacaaacacagagactggtgcaccccagctcaacccgattgacggaccactacctgaagataatgaaccaagtgggtatgcacaaacagactgtgttctagaggctatggcttttcttgaagaatctcatccaggaatatttgagaattcatgccttgaaacaatggaagttgttcaacaaacaagggtagataaactgactcaaggtcgccagacttatgattggacattaaacagaaatcaaccggcagcaactgcattggccaacaccatagaagtttttagatcgaatgacctaacagctaacgagtcaggaaggctaatagatttcttaaaggatgtgatggaatcaatgaacaaagaagaaatagagataacaacccactttcaaagaaaaaggagagtaagagacaacatgaccaagaagatggtcacgcaaagaacaatagggaagaaaaaacagagactgaataagagaggctatctaataagagcactgacattaaatacgatgaccaaagatgcagagagaggcaagttaaaaagaagggctatcgcaacacctgggatgcagattagaggtttcgtatactttgttgaaactttagctaggagcatttgcgaaaagcttgaacagtctgggctcccagtagggggcaatgaaaagaaggccaaattggcaaatgttgtgagaaagatgatgactaattcacaagatacagagatttctttcacaatcactggggacaacactaagtggaatgaaaatcaaaatcctcgaatgttcctggcgatgattacatatatcaccagagatcaacccgagtggtttagaaacatcctgagcatggcacccataatgttctcaaataaaatggcaagactaggaaaagggtacatgttcgagagtaaaagaatgaagattcgaacacaaataccagcagaaatgctagcaagcattgatctgaagtatttcaatgaatcaacaaagaagaaaattgagaaaataaggcctcttttaatggatggcacagcatcactgagtcctgggatgatgatgggcatgttcaacatgctaagtacggtcttgggagtctcgatactgaatcttggacaaaagaaatacaccaagacaacatactggtgggatgggctccaatcatccgacgattttgctctcatagtgaatgcaccaaaccatgaaggaatacaagcaggagtggacagattctacaggacttgcaagttagtgggaatcaacatgagcaaaaagaagtcctatataaataagacagggacatttgaattcacaagctttttttatcgctatggatttgtggctaattttagcatggagctacctagctttggagtgtctggagtaaatgaatcagctgacatgagtattggagtaacagtgataaagaacaacatgataaacaatgaccttggacctgcaacggctcaaatggctcttcaattgttcatcaaagactacagatacacatataggtgccataggggagacacacaaattcagacgagaagatcatttgagttgaagaagctatgggatcaaacccaatcaaaggtagggctattagtatcagatggaggaccaaacttatacaacatacggaatcttcacattcctgaagtctgcttaaaatgggagctaatggatgatgattatcggggaagactttgtaatcccctgaatccctttgtgagtcataaggagattgattctgtaaacaatgctgtggtaatgccagcccatggcccagccaaaagcctggaatatgatgccgttgctactacacattcctggattcccaagaggaatcgttctattctcaacacaagccaaaggggaattcttgaggatgaacagatgtaccagaagtgttgcaatttattcgagaaatttttccctagcagttcatataggagaccggttggaatttctagcatggtggaggccatggtatctagggcccggattgatgccagagtcgactttgagtctggacggatcaagaaagaagagttctctgagatcatgaagatctgttccaccattgaagaactcagacggcaaaaataa

>H1N1_PB1_KY925997_A/Campina das Missoes/LACENRS-2882/2009

atggatgtcaatccgactctacttttcctaaaaattccagcgcaaaatgccataagcaccacattcccttatactggagatcctccatacagccatggaacaggaacaggatacaccatggacacagtaaacagaacacaccaatactcagaaaagggaaagtggacgacaaacacagagactggtgcaccccagctcaacccgattgatggaccactacctgaggataatgaaccaagtgggtatgcacaaacagactgtgttctagaggctatggctttccttgaagaatcccacccaggaatatttgagaattcatgccttgaaacaatggaagttgttcaacaaacaagggtagataaactaactcaaggtcgccagacttatgattggacattaaacagaaatcaaccggcagcaactgcattggccaacaccatagaagtctttagatcgaatggcctaacagctaatgagtcaggaaggctaatagatttcttaaaggatgtaatggaatcaatgaacaaagaggaaatagagataacaacccactttcaaagaaaaaggagagtaagagacaacatgaccaagaagatggtcacgcaaagaacaatagggaagaaaaaacaaagactgaataagagaggctatctaataagagcactgacattaaatacgatgaccaaagatgcagagagaggcaagttaaaaagaagggctatcgcaacacctgggatgcagattagaggtttcgtatactttgttgaaactttagctaggagcatttgcgaaaagcttgaacagtctgggctcccagtagggggcaatgaaaagaaggccaaactggcaaatgttgtgagaaagatgatgactaattcacaagacacagagatttctttcacaatcactggggacaacactaagtggaatgaaaatcaaaaccctcgaatgttcctggcgatggttacatatatcaccagaaatcaacccgagtggttcagaaacatcctgagcatggcacccataatgttctcaaacaaaatggcaagactagggaaagggtacatgttcgagagtaaaagaatgaagattcgaacacaaataccagcagaaatgctagcaagcattgacctgaagtacttcaatgaatcaacaaagaagaaaattgagaaaataaggcctcttctaatagatggcacagcatcactgagtcctgggatgatgatgggcatgttcaacatgctaagtacggtcttgggagtctcgatactgaatcttggacaaaagaaatacaccaagacaatatactggtgggatgggctccaatcatccgacgattttgctctcatagtgaatgcaccaaaccatgagggaatacaagcaggagtggacagattctacaggacctgcaagttagtgggaatcaacatgagcaaaaagaagtcctatataaataagacagggacatttgaattcacaagctttttttatcgctatggatttgtggctaattttagcatggagctacccagctttggagtgtctggagtaaatgaatcagctgacatgagtattggagtaacagtgataaagaacaacatgataaacaatgaccttggacctgcaacggcccagatggctcttcaattgttcatcaaagactacagatacacatataggtgccataggggagacacacaaattcagacgagaagatcatttgagttaaagaagctgtgggatcaaacccaatcaaaggtagggctattagtatcagatggaggaccaaacttatacaatatacggaatcttcacattcctgaagtctgcttaaaatgggagctaatggatgatgattatcggggaagactttgtaatcccctgaatccctttgtcagtcataaagagattgattctgtaaacaatgctgtggtaatgccagcccatggtccagccaaaagcatggaatatgatgccgttgcaactacacattcctggattcccaagaggaatcgttctattctcaacacaagccaaaggggaattcttgaggatgaacagatgtaccagaagtgctgcaatctattcgagaaatttttccctagcagttcatataggagaccggttggaatttctagcatggtggaggccatggtgtctagggcccggattgatgccagggtcgacttcgagtctggacggatcaagaaagaagagttctctgagatcatgaagatctgttccaccattgaagaactcagacggcaaaaataa

>H1N1_PB1_KY925412_A/Porto Alegre/LACENRS-2121/2009

atggatgtcaatccgactctacttttcctaaaaattccagcgcaaaatgccataagcaccacattcccttatactggagatcctccatacagccatggaacaggaacaggatacaccatggacacagtaaacagaacacaccaatactcagaaaagggaaagtggacgacaaacacagagactggtgcaccccagctcaacccgattgatggaccactacctgaggataatgaaccaattgggtatgcacaaacagactgtgttctagaggctatggctttccttgaagaatcccacccaggaatatttgagaattcatgccttgaaacaatggaagttgttcaacaaacaagggtagataaactaactcaaggtcgccagacttatgattggacattaaacagaaatcaaccggcagcaactgcattggccaacaccatagaagtctttagatcgaatggcctaacagctaatgagtcaggaaggctaatagatttcttaaaggatgtaatggaatcaatgaacaaagaggaaatagagataacaacccactttcaaagaaaaaggagagtaagagacaacatgaccaagaagatggtcacgcaaagaacaatagggaagaaaaaacaaagactgaataagagaggctatctaataagagcactgacattaaatacgatgaccaaagatgcagagagaggcaagttaaaaagaagggctatcgcaacacctgggatgcagattagaggtttcgtatactttgttgaaactttagctaggagcatttgcgaaaagcttgaacagtctgggctcccagtagggggcaatgaaaagaaggccaaactggcaaatgttgtgagaaagatgatgactaattcacaagacacagagatttctttcacaatcactggggacaacactaagtggaatgaaaatcaaaatcctcgaatgttcctggcgatgattacatatatcaccagaaatcaacccgagtggttcagaaacatcctgagcatggcacccataatgttctcaaacaaaatggcaagactagggaaagggtacatgttcgagagtaaaagaatgaagattcgaacacaaataccagcagaaatgctagcaagcattgacctgaagtacttcaatgaatcaacaaagaagaaaattgagaaaataaggcctcttctaatagatggcacagcatcactgagtcctgggatgatgatgggcatgttcaacatgctaagtacggtcttgggagtttcgatactgaatcttggacaaaagaaatacaccaagacaatatactggtgggatgggctccaatcatccgacgattttgctctcatagtgaatgcaccaaaccatgagggaatacaagcaggagtggacagattctacaggacctgcaagttagtgggaatcaacatgagcaaaaagaagtcctatataaataagacagggacatttgaattcacaagctttttttatcgctatggatttgtggctaattttagcatggagctacccagctttggggtgtctggagtaaatgaatcagctgacatgagtattggagtaacagtgataaagaacaacatgataaacaatgaccttggacctgcaacggcccagatggctcttcaattgttcatcaaagactacagatacacatataggtgccataggggagacacacaaattcagacgagaagatcatttgagttaaagaagctgtgggatcaaacccaatcaaaggtagggctattagtatcagatggaggaccaaacttatacaatatacggaatcttcacattcctgaagtctgcttaaaatgggagctaatggatgatgattatcggggaagactttgtaatcccctgaatccctttgtcagtcataaagagattgattctgtaaacaatgctgtggtaatgccagcccatggtccagccaaaagcatggaatatgatgccgttgcaactacacattcctggattcccaagaggaatcgttctattctcaacacaagccaaaggggaattcttgaggatgaacagatgtaccagaagtgctgcaatctattcgagaaatttttccctagcagttcatataggagaccggttggaatttctagcatggtggaggccatggtgtctagggcccggattgatgccagggtcgacttcgagtctggacggatcaagaaagaagagttctctgagatcatgaagatctgttccaccattgaagaactcagacggcaaaaataa

>H1N1_PB1_KY925461_A/Porto Alegre/LACENRS-2314/2014

atggatgtcaatccgactctacttttcctaaaaattccagcacaaaatgccataagcaccacattcccttatactggagatcctccatacagccatggaacaggaacaggatacaccatggacacagtaaacagaacacaccaatactcagaaaaggggaagtggacaacaaacacagagactggtgcaccccagctcaacccgattgatggaccactacctgaggataatgaaccaagtgggtatgcacaaacagactgtgtcctagaggctatggctttccttgaagaatcccacccaggaatatttgagaattcatgccttgaaacaatggaagttgttcaacaaacaagggtagataaactgactcaaggtcgccagacttatgattggacattaaacagaaatcaaccggcagcaactgcattggccaacaccatagaagtctttagatcgaatgacctgacagctaacgagtcaggaaggctaatagatttcttaaaggatgtaatggaatcaatgaacaaagaggaaatagagataacaacccactttcaaagaaaaaggagagtaagagacaacatgaccaagaagatggtcacgcaaagaacaatagggaagaaaaaacagagactgaataagagaggctatctaataagagcactgacattaaatacgatgaccaaagatgcagagagaggcaagttaaaaagaagggctatcgcaacacctgggatgcagattagaggtttcgtatactttgttgaaactttagctaggagcatttgcgaaaagcttgaacagtctgggctcccagtagggggcaatgaaaagaaggccaaattggcaaatgttgtgagaaagatgatgactaattcacaagatacagagatttctttcacaatcactggggacaacactaaatggaatgaaaatcaaaatccccgaatgttcctggctatgattacatatatcaccagaaatcaacccgagtggtttagaaacatcctgagcatggcacccataatgttctcaaataaaatggcaagactggggaaagggtacatgttcgagagtaaaagaatgaagattcgaacacaaataccagcagaaatgctagcaagcattgatctgaagtacttcaatgaatcaacaaagaagaaaattgagaaaataaggcctcttttaatggatggcacagcatcactgagtcctgggatgatgatgggcatgttcaacatgctaagtacggtcttgggagtctcgatactgaatcttggacaaaagaaatacaccaagacaacatactggtgggatgggctccaatcatccgacgattttgctctcatagtgaatgcaccaaaccacgaaggaatacaagcaggagtggacagattctacaggacctgcaagttagtgggaatcaacatgagcaaaaagaagtcctatataaataagacagggacatttgaattcacaagctttttttatcgctatggatttgtggctaattttagcatggagctacccagctttggagtgtctggagtaaatgaatcagctgacatgagtattggagtaacagtgataaagaacaacatgataaacaatgaccttggacctgcaacggcccagatggctcttcaattgttcatcaaagactacagatacacatataggtgccataggggagacacacaaattcagacgagaagatcatttgagttaaagaagctatgggatcaaacccaatcaaaggtagggctattagtatcagatggaggaccaaacttatacaacatacggaatcttcacattcctgaagtctgcttaaaatgggagctaatggatgatgattatcggggaagactttgtaatcccctgaatccctttgtgagtcataaggagattgattctgtaaacaatgccgtggtaatgccagcccatggtccagccaaaagcatggaatatgatgccgttgcaactacacattcctggattcccaagaggaatcgttctattctcaacacaagccaaaggggaattcttgaggatgaacagatgtaccagaagtgttgtaatctattcgagaaatttttccctagcagttcatataggagaccagttggaatttctagcatggtggaggccatggtgtctagggcccggattgatgccagggtcgactttgagtctggacggatcaagaaagaagagttctctgagaccatgaagatctgttccaccattgaagaactcagacggcaaaaataa

>H1N1_PB1_KY926289_A/Porto Alegre/LACENRS-908/2012

atggatgtcaatccgactctacttttcctaaaaattccagcgcaaaatgccataagcaccacattcccttatactggagatcctccatacagccatggaacaggaacaggatacaccatggacacagtaaacagaacacaccaatactcagaaaagggaaagtggacgacaaacacagagactggtgcaccccagctcaacccgattgatggaccactacctgaggataatgaaccaagtgggtatgcacaaacagactgtgttctagaggctatggctttccttgaagaatcccacccaggaatatttgagaattcatgccttgaaacaatggaagttgttcaacaaacaagggtagataaactaactcaaggtcgccagacttatgattggacactaaacagaaatcaaccggcagcaactgcattggccaacaccatagaagtctttagatcgaatggcctaacagctaatgagtcaggaaggttaatagatttcttaaaggacgtaatggaatcaatgaacaaagaggaaatagagataacaacccactttcaaagaaaaaggagagtgagagacaacatgaccaagaaaatggtcacgcaaagaacaatagggaagaaaaaacaaagactgaataagagaggctatctaataagagcactgacattaaatacgatgaccaaagatgcagagagaggcaagttaaaaagaagggctatcgcaacacctgggatgcagattagaggtttcgtatattttgttgaaactttagctaggagcatttgcgaaaagcttgaacagtctgggctcccagtagggggcaatgaaaagaaggccaaactggcaaatgttgtgaggaagatgatgactaattcacaagacacagagatttctttcacaatcactggggacaacactaagtggaatgaaaaccaaaatcctcgaatgttcctggcgatgattacatatatcaccagaaatcaacccgagtggttcagaaacatcctgagcatggcacccataatgttctcaaacaaaatggcaagactagggaaagggtacatgttcgagagtaaaagaatgaagattcgaacgcaaataccagcagaaatgctagcaagcattgacctgaagtacttcaatgaatcaacaaagaagaaaattgagaaaataagacctcttttaatagatggcacagcatcactgagtcctgggatgatgatgggcatgttcaacatgctaagtacggtcttgggagtctcgatactgaatcttggacaaaagaaatacaccaagacaatatactggtgggatgggctccaatcatccgacgattttgctctcatagtgaatgcaccaaaccatgagggaatacaagcaggagtggacagattttacaggacctgcaagttagtaggaatcaacatgagcaaaaagaagtcctatataaataagacagggacatttgaattcacaagctttttttatcgctatggatttgtggctaattttagcatggagctacccagctttggagtgtctggagtaaatgaatcagctgacatgagtattggagtaacagtgataaagaacaacatgataaacaatgaccttggacctgcaacggcccagatggctcttcaattgttcatcaaagactacagatacacatataggtgccataggggagacacacaaattcagacgagaagatcatttgagttaaagaagctgtgggatcaaacccaatcaaaggtagggctattagtatcagatggaggaccaaacttatacaatatacggaatcttcacattcctgaagtctgcttaaaatgggagctaatggatgatgattatcggggaagactttgtaatcccctgaatccctttgtaagtcataaagagattgattctgtaaacaatgctgtggtaatgccagctcatggtccagccaaaagcatggaatatgatgccgttgcaactacacattcctggattcccaagaggaatcgctctattctcaacacaagccaaaggggaattcttgaggatgaacagatgtaccagaagtgctgcaatctattcgagaaatttttccctagcagttcatataggagaccggttggaatttctagcatggtggaggccatggtgtctagggcccggattgatgccagggtcgactttgagtctggacggatcaagaaagaagagttctctgagatcatgaagatctgttccaccattgaagaactcagacggcaaaaataa

>H1N1_PB1_M25932_A/Beijing/11/1956

atggatgtcaatccgaccttacttttcttaaaagtgccagcacaaaatgctataagcacaactttcccttatactggagaccctccttacagccatgggacaggaacagggtacaccatggatactgtcaacaggacacatcagtactcagaaaggggaagatggacaaaaaacaccgaaactggagcaccacagctcaacccaattgatgggccactaccagaagacaatgaaccaagtggttatgcccaaacagattgtgtattagaagcaatggctttccttgaggaatcccatcctggtatttttgaaaactcatgtattgaaacgatggaggttgttcagcaaacacgagtggacaagctgacacaaggccgacagacctatgactggactctgaatagaaaccagcctgctgctacagcattggcaaacacaatagaagtgttcagatcaaatggcctcatggcaaatgaatccggaaggctaatagactttcttaaagatgtaatggagtcaatggacaaagaagaaatagagatcacaacccattttcagagaaagagacgggtgagggacaatgtgactaagaaaatggtgacacaaagaacaataggcaaaaagaaacagagattaaacaaaaggagttatctaattagggcattgaccctgaacacaatgaccaaagatgctgagagagggaaactaaaacgaagagcaattgcaaccccagggatgcaaataagggggtttgtatactttgttgagacactggcaagaagtatatgtgaaaaacttgaacaatcaggactgccagttggaggaaatgaaaagaaagcaaagttggcaaatgttgtaaggaagatgatgaccaattctcaggacactgaaatttctttcaccatcactggagacaacaccaaatggaacgaaaatcaaaaccctagaatgtttttggccgtgatcacatatataaccagaaatcagcccgaatggttcagaaatattctaagtattgctccaataatgttctcaaacaaaatggcgagactaggtaagggttacatgtttgagagcaagagtatgaaacttagaactcaaatatctgcagaaatgctagcaaacatcgatttgaagtatttcaatgattcaacaagaaagaaaattgaaaaaatccggccgctcttaatagatgggactgcatcactgagccctggaatgatgatgggcatgttcaatatgttaagcactgtattgggcgtttccatcctgaatcttggacaaaagagatacacaaagactacttactggtgggatggtcttcaatcatctgatgattttgctctgattgtgaatgcacccaatcatgcaggaattcaggctggagttgacaggttttatcgaacttgtaagctgctcggaatcaatatgagtaagaaaaagtcttacataaacagaacaggtacatttgaattcactagctttttctatcgttatgggtttgttgccaatttcagcatggagcttcccagttttggggtgtctgggatcaacgagtctgcagatatgagtattggggtcactgtcatcaaaaacaatatgataaacaatgatcttggtccagcaaccgctcaaatggcccttcagttattcatcaaagattacaggtacacgtatcggtgccacagaggtgacacacaaatacaaacccgaagatcatttgaaataaagaaactgtgggagcaaacccgttccaaagctgggctgctggtctccgatggaggtcctaacttatataacattagaaatctccacacccctgaagtctgtttgaaatgggaattgatggatgaggattaccaggggcgtttatgcaacccattgaacccatttgtcagtcataaagagattgaatcagtgaacaatgcagtgatgatgccggcacatggtccagccaaaaacatggagtatgatgctgtagcaacaacacactcctgggtccccaaaagaaatcgatccatcttgaatactagtcaaaggggaatacttgaagatgaacaaatgtatcaaaggtgctgcaatttatttgaaaggttcttccccagcagttcatacagaaggccagtcggaatatccagtatggtggaggctatggtttccagagcccgaattgatgcacggattgatttcgaatctggaaggataaagaaagaggatttcactgagatcatgaagatctgttccaccattgaagaactcagacggcaaaaatag

>H1N1_PB1_CY002678_A/New York/310/2001

atggatgtcaatccaacattacttttcttaaaagtgccagcacaaaatgctataagcacaacttttccttatactggtgaccctccttacagccatgggacaggaacagggtacaccatggatacagtcaacaggacacatcagtactcagaaagaggaaggtggacaaaaaataccgaaactggagcaccgcaactcaacccaattgatgggccactaccaaaagacaatgaaccaagtggctatgcccaaacagattgtgtattagaagcaatggctttccttgaggaatcccatcctggtatttttgaaaactcttgtattgaaacaatggaggttgttcagcaaacaagggtggacaaactgacacaaggcagacagacctatgactggactctaaataggaaccagcctgctgccacagcattggccaacactatagaagtgttcagatcaaatggcctcatagcaaatgaatctgggaggctaatagacttccttaaagatgtaatggagtcgatggacagagacgaagtagagatcacaactcattttcagagaaagaggagagtaagagacaatgtaactaaaaaaatggtgacccaaagaacaataggcaaaaagaaacataaattagacaaaagaagttacctaattagggcattaaccctgaacacaatgaccaaagatgctgagagggggaaactaaaacgcagagcaattgcaaccccaggaatgcaaataagggggtttgtatactttgttgagacactggcaagaagcatatgtgaaaagcttgaacaatcagggttgccagttggaggaaatgaaaagaaagcaaagttagcaaatgttgtaaggaagatgatgaccaactcccaggacactgaaatttctttcacaatcactggagataacacaaaatggaacgaaaatcaaaaccctagaatgttcttggccatgatcacatatataaccaaaaatcagcctgaatggttcagaaatattttaagtattgctccaataatgttttcaaacaaaatggcgagactaggtaaggggtacatgtttgaaagcaagagtatgaaactgagaactcaaatacctgcagagatgctagccaacatagatttgaaatatttcaatgattcaactaaaaagaaaattgaaaaaatccggccattattaatagatggaactgcatcattgagtcctggaatgatgatgggcatgttcaatatgttaagcaccgtcttgggcgtctccattctgaatcttgggcaaaagagatacaccaagactacttactggtgggatggtcttcaatcgtctgatgattttgctctgattgtgaatgcacccaaccatgcaggcattcaagctggagttgacaggttttatcgaacctgtaagctgctcggaattaatatgagcaaaaagaagtcttacataaacagaacaggtacctttgaattcacgagcttcttctatcgttatgggtttgttgccaatttcagcatggagcttcctagttttggggtgtctggggtcaatgaatctgcagacatgagtattggagtcactgtcatcaaaaacaatatgataaacaatgaccttggcccagcaactgctcaaatggcccttcagttatttataaaagattacaggtacacgtatcgatgccacagaggtgacacacaaatacaaacccggagatcatttgaaataaagaaactatgggaccaaacccgctccaaagctgggctgttggtctctgatggaggccccaatttatataacattagaaatctccatattcctgaagtctgcttgaaatgggagttgatggatgaagattaccaggggcgtttatgcaacccattgaacccgtttgtcagtcataaagagattgaatcagtgaacaatgcagtgatgatgccggcacatggtccagccaaaaatatggagtatgacgctgttgcaacaacacactcctgggtccccaaaaggaatcgatccattttgaatacgagccaaagggggatacttgaggatgagcaaatgtatcagaggtgctgcaatttatttgaaaaattcttcccaagtagctcatacagaagaccagttggaatatccagtatggtagaggctatggtttccagagcccgaattgatgcacggattgatttcgaatctggaaggataaagaaagaggaattcgctgagatcatgaagacctgttccaccattgaagacctcagacggcaaaaatag

>H1N1_PB1_CY003030_A/New York/341/2001

atggatgtcaatccgaccttacttttcttaaaagtgccagcacaaaatgctataagcacaactttcccttatactggtgatcctccttacagccatgggacaggaacagggtataccatggatacagtcaacaggacacatcagtactcagaaagaggaagatggacaaaaaataccgaaactggagcaccgcaactcaacccaattgatggaccactaccaaaagacaatgaaccaagtggctatgcccaaacagattgtgtattagaagcaatggctttccttgaggaatcccatcctggtatttttgaaaactcttgtattgaaacaatggaggttgttcagcaaacaagggtggacaaactgacacaaggcagacagacctatgactggactctaaatagaaaccagcctgctgccacagcattggccaacactatagaagtgttcagatcaaacggcctcatagcaaatgaatctgggaggctaatagacttccttaaagatgtaatggagtcgatggacagagacgaagtagagatcacaacacattttcagagaaagaggagggtgagagacaatgtaactaaaaaaatggtgacccaaagaacaataggcaaaaagaaacataaattagacaaaagaagttacctaattagggcattaaccctgaacacaatgaccaaagatgctgagagggggaaactaaaacgtagagcaattgcaaccccaggaatgcaaataagggggtttgtatactttgttgagacactggcaagaagtatatgtgaaaagcttgaacaatcagggttgccagttgggggaaatgaaaagaaagcaaagttagcaaatgttgtaaggaagatgatgaccaactcccaggacactgaaatttctttcaccatcactggagataacacaaaatggaacgaaaatcaaaaccctagaatgtttttggccatgatcacatatataaccaaaaatcagcctgaatggttcagaaatattctaagtattgctccaataatgttttcaaacaaaatggcgagactaggtaaggggtacatgtttgaaagcaagagtatgaaactgagaactcaaatacctgcagagatgctagccaacatagatttgaaatatttcaatgattcaactaaaaagaaaattgaaaaaatccggccattattaatagatggaactgcatcattgagtcctggaatgatgatgggcatgttcaatatgttaagcaccgtcttgggcgtctccattctgaatcttgggcaaaagagatacaccaagactacttactggtgggatggtcttcaatcgtctgatgattttgctctgattgtgaatgcacccaactatgcaggaattcaagctggagttgacaggttttatcgaacctgtaagctgctcggaattaatatgagcaaaaagaagtcttacataaacagaacaggtacctttgaattcacgagctttttctatcgttatgggtttgttgccaatttcagcatggagcttcctagttttggggtgtctggggtcaatgagtctgcagacatgagtattggagtcactgtcatcaaaaacaatatgataaacaatgaccttggcccagcaactgctcaaatggcccttcagttatttatcaaagattacaggtacacgtatcgatgccacagaggtgacacacaaatacaaacccggagatcatttgagataaagaaactatgggaccaaacccgctccaaagctgggctgttggtctctgatggaggccccaatttatataacattagaaatctccatattcctgaagtctgcttgaaatgggagttgatggatgaggattaccaggggcgtttatgcaacccattgaacccatttgtcagtcataaagagattgagtcagtgaacaatgcagtgatgatgccggcacatggtccagccaaaaatatggagtatgacgctgttgcaacaacacactcctgggttcccaaaaggaatcgatccattttgaatacgagccaaagggggatacttgaggatgagcaaatgtatcagaggtgctgcaatttatttgaaaaattcttcccaagtagctcatacagaagaccagttggaatatccagtatggtagaggctatggtttccagagcccgaattgatgcaagaattgatttcgaatctggaaggataaagaaagaggaattcgctgagatcatgaagacctgttccaccattgaagacctcagacggcaaaaatag

>H1N1_PB1_CY006369_A/New York/303/2001

atggatgtcaatccgactttacttttcttaaaagtgccagcacaaaatgctataagcacaacttttccttatactggtgatcctccttacagccatgggacaggaacagggtacaccatggatacagtcaacaggacacatcagtactcagaaagaggaagatggacaaaaaataccgaaactggagcaccgcaactcaacccaattgatggaccactaccaaaagacaatgaaccaagtggctatgcccaaacagattgtgtattagaagcaatggctttccttgaggaatcccatcctggtatttttgaaaactcttgtattgaaacaatggaggttgttcagcaaacaagggtggacaaactgacacaaggcagacagacctatgactggactctaaatagaaaccagcctgctgccacagcattggccaacactatagaagtgttcagatcaaacggcctcatagcaaatgaatctgggaggctaatagacttccttaaagatgtaatggagtcgatggacagagacgaagtagagatcacaacacattttcagagaaagaggagggtgagagacaatgtaactaaaaaaatggtgacccaaagaacaataggcaaaaagaaacataaattagacaaaagaagttacctaattagggcattaaccctgaacacaatgaccaaagatgctgagagggggaaactaaaacgtagagcaattgcaaccccaggaatgcaaataagggggtttgtatactttgttgagacactggcaagaagcatatgtgaaaagcttgaacaatcagggttgccagttgggggaaatgaaaagaaagcaaagttagcaaatgttgtaaggaagatgatgaccaactcccaggacactgaaatttctttcaccataactggagataacacaaaatggaacgaaaatcaaaaccctagaatgtttttggccatgatcacatatataaccaaaaatcagcctgaatggttcagaaatattctaagtattgctccaataatgttttcaaacaaaatggcgagactaggtaaggggtacatgtttgaaagcaagagtatgaaactgagaactcaaatacctgcagagatgctagccaacatagatttgaaatatttcaatgattcaactaaaaagaaaattgaaaaaatccggccattattaatagatggaactgcatcattgagtcctggaatgatgatgggcatgttcaatatgttaagcaccgtcttgggcgtctccattctgaatcttgggcaaaagagatacaccaagactacttactggtgggatggtcttcaatcgtctgatgattttgctctgattgtgaatgcacccaactatgcaggaattcaagctggagttgacaggttttatcgaacctgtaagctgctcggaattaatatgagcaaaaagaagtcttacataaacagaacaggtacctttgaattcacgagctttttctatcgttatgggtttgttgccaatttcagcatggagcttcctagttttggggtgtctggggtcaatgagtctgcagacatgagtattggagtcactgtcatcaaaaacaatatgataaacaatgaccttggcccagcaactgctcaaatggcccttcagttatttatcaaagattacaggtacacgtatcgatgccacagaggtgacacacaaatacaaacccggagatcatttgagataaagaaactatgggaccaaacccgctccaaagctgggctgttggtctctgatggaggccccaatttatataacattagaaatctccatattcctgaagtctgcttgaaatgggagttgatggatgaggattaccaggggcgtttatgcaacccattgaacccatttgtcagtcataaagagattgagtcagtgaacaatgcagtgatgatgccggcacatggtccagccaaaaatatggagtatgacgctgttgcaacaacacactcctgggttcccaaaaggaatcgatccattttgaatacgagccaaagggggatacttgaggatgagcaaatgtatcagaggtgctgcaatttatttgaaaaattcttcccaagtagctcatacagaagaccagttggaatatccagtatggtagaggctatggtttccagagcccgaattgatgcaagaattgatttcgaatctggaaggataaagaaagaggaattcgctgagatcatgaagacctgttccaccattgaagacctcagacggcaaaaatag

>H1N1_PB1_CY007473_A/Canterbury/106/2004

atggatgtcaatccgacattacttttcttaaaagtgccagcacaaaatgctataagcacaacttttccttatactggtgaccctccttacagccatgggacaggaacagggtacaccatggatacagtcaacaggacacatcagtactcagaaagaggaagatggacaaaaaataccgaaactggagcaccgcaactcaacccaattgatgggccactaccagaagacaatgaaccaagtggctatgcccaaacagattgtgtattagaagcaatggctttccttgaagaatcccatcctggtatttttgaaaactcttgtattgaaacaatggaggttgttcagcaaacaagggtggacaaactgacacaaggcagacagacctatgactggactctaaataggaaccagcctgctgccacagcattggccaacactatagaagtattcagatcaaacggcctcatagcaaatgaatctgggaggctaatagacttccttaaagatgtaatggagtcgatggacagagaagaagtagaggtcacaactcattttcagagaaagaggagagtgagagacaatgtaactaaaaaaatggtgacccaaagaacaataggcaaaaagaaacataaattagacaaaaggagttacctaattagagcattaaccctgaacacaatgaccaaagatgctgagagggggaaactaaaacgcagagcaattgcaaccccaggaatgcaaataagggggtttgtatactttgttgaaacactggcaagaagcatatgtgaaaagcttgaacaatcagggttgccagttggaggaaatgagaaaaaagcaaagttagcaaatgttgtaaggaagatgatgaccaactcccaggacactgaaatttctttcaccatcactggagataacacaaaatggaacgaaaatcaaaaccctagaatgtttttggccatgatcacatatataaccagaaatcagcctgaatggttcagaaatattctaagtattgctccaataatgttttcaaacaaaatggcgagactaggtaaggggtacatgtttgaaagcaagagtatgaaactgagaactcaaatacctgcagagatactagccgacatagatttgaagtatttcaatgagtcaactaaaaagaaaattgaaaaaatccgaccattattaatagatggaactgcatcattgagtcctggaatgatgatgggcatgttcaatatgttaagcactgtcttgggcgtctccattctgaatcttgggcaaaagagatacaccaagactacttactggtgggatggtcttcaatcgtctgatgattttgctctgatagtgaatgcgcccaactatgcaggaattcaagctggagttgacaggttttatcgaacctgtaagctgctcggaattaatatgagcaaaaagaagtcttacataaacaggacaggtacctttgaattcacgagctttttctatcgttatgggtttgttgccaatttcagcatggagcttcctagttttggggtgtctggggtcaatgaatctgcagacatgagtattggagtcactgttatcaaaaacaatatgataaacaatgaccttggcccagcaactgctcaaatggcccttcagttatttataaaagattacagatacacgtatcgatgccacagaggtgacacacaaatacaaacccggagatcatttgagataaagaaattatgggaccaaacccgctccaaagctgggctgttggtttctgatggaggccccaatttatataacattagaaatctccatattcctgaagtctgcttgaaatgggagttgatggatgaggattaccaggggcgtttatgcaacccattgaacccgtttgtcagtcataaagagattgaatcagtgaacaatgcagtgatgatgccggcacatggtccagccaaaaatatggagtatgacgctgttgcaacaacacattcctgggttcccaaaaggaatcgatccattttgaacacgagccaaagggggatacttgaggatgagcaaatgtatcagaggtgctgcaatttatttgaaaaattcttcccaagtagctcatacagaagaccagttgggatatccagtatggtagaggctatggtttccagagcccgaattgatgcacggattgatttcgaatctggaaggataaagaaagaggaattcgctgagatcatgaagacctgttccaccattgaagacctcagacggcaaaaatag

>H1N1_PB1_CY008154_A/New York/307/2001

atggatgtcaatccgactttacttttcttaaaagtgccagcacaaagtgctataagcacaactttcccctatactggagaccccccttacagccatgggacagggacagggtacaccatggatacagtcaacaggacacatcagtactcagaaagaggaagatggacaaaaaataccgaaactggagcaccgcaactcaacccgattgatgggccattaccaaaagacaatgaaccaagtggctatgcccaaacagattgtgtattagaagcaatggctttccttgaggaatcccatcctggtatttttgaaaactcatgtattgaaacgatggaggctgttcagcaaacaagagtggacaaactgacacaaggaagacagacctatgactggactctaaatagaaaccagcctgctgccacagcattggccaacactatagaagtgttcagatcaaacggcctcatatcaaatgagtctgggaggctaatagacttccttaaagatgtaatggagtcaatggacagagaagaagtagagatcacaactcactttcagagaaagagaagagtgagagacaatgtaactaaaaaaatggtgacacaaagaacaataggcaaaaagaaacataaattggacaaaagaagttatctaattagggcattaaccctgaacacaatgaccaaagatgctgagagggggaaactaaaacgtagagctattgcaaccccaggaatgcagataagggggtttgtatactttgttgagacactggcaaggagcatatgtgagaaacttgagcaatcagggttaccagttggaggaaatgagaagaaagcaaagttagcaaatgttgtaaggaagatgatgaccaactcccaggacactgaaatttctttcaccatcactggagataacacaaaatggaacgaaaatcagaaccctagaatgtttttggctatgatcacatatataaccagaaatcagcctgaatggttcagaaacattctaagtattgctccaataatgttttcaaacaaaatggcgagattaggtaaggggtacatgtttgaaagcaagagtatgaagctgagaactcaaatacctgcagaaatgctggccaacatagatttgaaatatttcaatgattcaacaaaaaagaaaattgaaaaaatccggccactattaatagatggaactgcatcactgagtcctggaatgatgatgggcatgttcaatatgttgagcactgtcttgggcgtctccatcctgaatcttggacaaaagagatacaccaagaccacttactggtgggatggtcttcaatcgtctgatgattttgctctgattgtgaatgcacccaactatgcaggaattcaagctggagttgacaggttttatcgaacctgtaagctgctcggaattaatatgagcaaaaagaagtcttacataaacaggacaggtacttttgaatttacaagctttttctatcgttatggatttgttgccaattttagcatggaacttcctagttttggggtgtctggggttaatgagtctgcagacatgagtattggagtcactgtcatcaaaaataatatgataaacaatgatcttggtccagcaactgcccaaatggcccttcagttattcatcaaagattacaggtatacgtatcgatgccacagaggtgacacacaaatacaaacccggagatcatttgagataaaaaaactgtgggaccaaacccgttccaaagctgggctgttggtctctgatggaggccccaatttatataatattagaaatctccacattcctgaagtctgcttgaaatgggagttgatggatgaggattaccaagggcgtttatgcaatccattgaacccttttgtcagtcataaagaaattgaatcagtgaacaatgcagtgatgatgccggcacatggtccagccaaaaatgtggaatatgacgctgttgcaacaacgcactcctgggtccccaaaaggaatcgatccattttgaatacgagccaaagaggaatacttgaggatgagcaaatgtatcaaaggtgctgcaatttgtttgaaaagttcttcccaagcagctcatatagaagaccagttggaatatccagtatggtagaggccatggtttccagagcccgaatcgatgcacggattgatttcgaatctggaaggataaagaaagaagagttcgctgagatcatgaagacctgttccaccattgaagacctcagacggcaaaaatag

>H1N1_PB1_CY009210_A/South Canterbury/35/2000

atggatgtcaatccgacattacttttcttaaaagtgccagcacaaaatgctataagcacaacttttccttatactggtgaccctccttacagccatgggacaggaacagggtacaccatggatacagtcaacaggacacatcagtactcagaaagaggaagatggacaaaaaataccgaaactggagcaccgcaactcaacccaattgatgggccactaccaaaagacaatgaaccaagtggctatgcccaaacagattgtgtattagaagcaatggctttccttgaggaatcccatcctggtatttttgaaaactcttgtattgaaacaatggaggttgttcagcaaacaagggtggacaaactgacacaaggcagacagacctatgactggactctaaataggaaccagcctgctgccacagcattggccaacactatagaagtgttcagatcaaacggcctcatagcaaatgaatctgggaggctaatagacttccttaaagatgtaatggagtcgatggacagagacgaaatagaggtcacaactcattttcagagaaagaggagagtgagagacaatgtaactaaaaaaatggtgacccaaagaacaataggcaaaaagaaacataaattagataaaagaagttacctaattagggcattaaccctgaacacaatgaccaaagatgctgagagggggaaactaaaacgcagagcaattgcaaccccaggaatgcaaataagggggtttgtatactttgttgagacactggcaagaagcatatgtgaaaagcttgaacaatcagggttgccagttggaggaaatgaaaagaaagcaaagttagcaaatgttgtaaggaagatgatgaccaactcccaggacactgaaatttctttcaccatcactggagataacacaaaatggaacgaaaatcaaaaccctagaatgttcttggccatgatcacatatataaccaaaaatcagcctgaatggttcagaaatattctaagtattgctccaataatgttttcaaacaaaatggcgagactaggtaaagggtacatgtttgaaagcaagagtatgaaactgagaactcaaatacctgcagagatgctagccaacatagatttgaaatacttcaatgattcaactaaaaagaaaattgaaaaaatccggccattattaatagatggaactgcatcattgagtcctggaatgatgatgggcatgttcaatatgttaagcaccgtcttgggcgtctccattctgaatcttgggcaaaagagatacaccaagactacttactggtgggatggtcttcaatcgtctgatgattttgctctgattgtgaatgcacccaactatgcaggaattcaagctggagttgacaggttttatcgaacctgtaagctgctcggaattaatatgagcaaaaagaagtcttacataaacaaaacaggtacctttgaattcacgagctttttctatcgttatgggtttgttgccaatttcagcatggagcttcctagttttggggtgtctggggtcaatgaatctgcagacatgagtattggagtcactgtcatcaaaaacaatatgataaacaatgaccttggcccagcaactgctcaaatggcccttcagttatttataaaagattacaggtacacgtatcgatgccacagaggtgacacacaaatacaaacccggagatcatttgagataaagaaactatgggaccaaacccgctccaaagctgggctgttggtctctgatggaggccccaatttatataacattagaaatctccatattcctgaagtctgcttgaaatgggagttgatggatgaggattaccaagggcgtttatgcaacccattgaacccgtttgtcagtcataaagagattgaatcagtgaacaatgcagtgatgatgccggcacatggtccagccaaaaatatggagtatgacgctgttgcaacaacacactcctgggttcccaaaaggaatcgatccattttgaatacgagccaaagggggatacttgaggatgagcaaatgtatcagaggtgctgcaatttatttgaaaaattcttcccaagtagctcatacagaagaccagttggaatatccagtatggtagaggctatggtttccagagcccgaattgatgcacggattgatttcgaatctggaaggacaaagaaagaggaattcgctgagatcatgaagacctgttccaccattgaagacctcagacggcaaaaatag

>H1N1_PB1_CY009874_A/South Canterbury/15/2001

atggatgtcaatccgaccttacttttcttaaaagtgccagcacaaaatgctataagcacaactttcccttatactggtgatcctccttacagccatgggacaggaacagggtacaccatggatacagtcaacaggacacatcagtactcagaaagaggaagatggacaaaaaataccgaaactggagcaccgcaactcaacccaattgatggaccactaccaaaagacaatgaaccaagtggctatgcccaaacagattgtgtattagaagcaatggctttccttgaggaatcccatcctggtatttttgaaaactcttgtattgaaacaatggagattgttcagcaaacaagggtggacaaactgacacaaggcagacagacctatgactggactctaaatagaaaccagcctgctgccacagcattggccaacactatagaagtgttcagatcaaacggcctcatagcaaatgaatctgggaggctaatagacttccttaaagatgtaatggagtcgatggacagagacgaagtagagatcacaacacattttcagagaaagaggagggtgagagacaatgtaactaaaaaaatggtgacccaaagaacaataggcaaaaagaaacataaattagacaaaagaagttacctaattagggcattaaccctgaacacaatgaccaaagatgctgagagggggaaactaaaacgtagagcaattgcaaccccaggaatgcaaataagggggtttgtatactttgttgagacactggcaagaagcatatgtgaaaagcttgaacaatcagggttgccagttgggggaaatgaaaagaaagcaaagttagcaaatgttgtaaggaagatgatgaccaactcccaggacactgaaatttctttcaccatcactggagataacacaaaatggaacgaaaatcaaaaccctagaatgtttttggccatgatcacatatataaccaaaaatcagcctgaatggttcagaaatattctaagtattgctccaataatgttttcaaacaaaatggcgagactaggtaaggggtacatgtttgaaagcaagagtatgaaactgagaactcaaatacctgcagagatgctagccaacatagatttgaaatatttcaatgattcaactaaaaagaaaattgaaaaaatccggccattattaatagatggaactgcatcattgagtcctggaatgatgatgggcatgttcaatatgttaagcactgtcttgggcgtctccattctgaatcttgggcaaaagagatacaccaagactacttactggtgggatggtcttcaatcgtctgatgattttgctctgattgtgaatgcatccaactatgcaggaattcaagctggagttgacaggttttatcgaacctgtaagctgctcggaattaatatgagcaaaaagaagtcttacataaacagaacaggtacctttgaattcacgagctttttctatcgctatgggtttgtcgccaatttcagcatggagcttcctagttttggggtgtctggggtcaatgagtctgcagacatgagtattggagtcactgtcatcaaaaacaatatgataaacaatgaccttggcccagcaactgctcaaatggcccttcagttatttatcaaagattacaggtacacgtatcgatgccacagaggtgacacacaaatacaaacccggagatcatttgagataaagaaactatgggaccaaacccgctccaaagctgggctgttggtctctgatggaggccccaatttatataacattagaaatctccatattcctgaagtctgcttgaaatgggagttgatggatgaggattaccaggggcgtttatgcaacccattgaacccatttgtcagtcataaagagattgagtcagtgaacaatgcagtgatgatgccggcacatggtccagccaaaaatatggagtatgacgctgttgcaacaacacactcctgggttcccaaaaggaatcgatccattttgaatacgagccaaagggggatacttgaggatgagcaaatgtatcagaggtgctgcaatttatttgaaaaattcttcccaagtagttcatacagaagaccagttggaatatccagtatggtagaggctatggtttccagagcccgaattgatgcaagaattgatttcgaatctggaaggataaagaaagaggaattcgctgagatcatgaagacctgttccaccattgaagacctcagacggcaaaaatag

>H1N1_PB1_CY010106_A/Canterbury/27/2000

atggatgtcaatccgaccttacttttcttaaaagtgccagcacaaaatgctataagcacaacttttccttatactggtgaccctccttacagccatgggacaggaacagggtacaccatggatacagtcaacaggacacatcagtactcagaaagaggaagatggacaaaaaataccgaaactggagcaccgcaactcaacccaattgatgggccactaccaaaagacaatgaaccaagtggctatgcccaaacagattgtgtattagaagcaatggctttccttgaggaatcccatcctggtatttttgaaaactcttgtattgaaacaatggaggttgttcagcaaacaagggtggacaaactgacacaaggcagacagacctatgactggactctaaatagaaaccagcctgctgccacagcattggccaacactatagaagtgttcagatcaaacggcctcatagcaaatgaatctgggaggctaatagacttccttaaagatgtaatggagtcgatggacaaagacgaagtagagatcacaactcattttcagagaaagaggagagtgagagacaatgtaactaaaaaaatggtgacccaaagaacaataggcaaaaagaaacataaattagacaaaagaagttacctaattagggcattaaccctgaacacaatgaccaaagatgctgagagggggaaactaaaacgtagagcaattgcaaccccaggaatgcaaataagggggtttgtatactttgttgagacactggcaagaagcatatgtgaaaagcttgaacaatcagggttgccagttggaggaaatgaaaagaaagcaaagttagcaaatgttgtaaggaagatgatgaccaactcccaggacactgaaatttctttcaccatcactggagataacacaaaatggaacgaaaatcaaaaccctagaatgttcttggccatgatcacatatataaccaaaaatcagcctgaatggttcagaaatattctaagtattgctccaataatgttttcaaacaaaatggcgagactaggtaaggggtacatgtttgaaagcaagagtatgaaactgagaactcaaatacctgcagagatgctagccaacatagatctgaaatatttcaatgattcaactaaaaagaaaattgaaaaaatccggccattattaatagatggaactgcatcattgagtcctggaatgatgatgggcatgttcaatatgttaagcaccgtcttgggcgtctccattctgaatcttgggcaaaagagatacaccaagactacttactggtgggatggtcttcaatcgtctgatgattttgctctgattgtgaatgcacccaactatgcaggaattcaagctggagttgacaggttttatcgaacctgtaagctgctcggaattaatatgagcaaaaagaagtcttacataaacagaacaggtacctttgaattcacgagctttttctatcgttatgggtttgttgccaatttcagcatggagcttcctagttttggggtgtctggggtcaatgagtctgcagacatgagtattggagtcactgtcatcaaaaacaatatgataaacaatgaccttggcccagcaactgctcaaatggcccttcagttatttatcaaagattacaggtacacgtatcgatgccacagaggtgacacacaaatacaaacccggagatcatttgagataaagaaactatgggaccaaacccgctccaaagctgggctgttggtctctgatggaggccccaatttatataacattagaaatctccatattcctgaagtctgcttgaaatgggagttgatggatgaggattaccaggggcgtttatgcaacccattgaatccgtttgtcagtcataaagagattgaatcagtgaacaatgcagtgatgatgccggcacatggtccagccaaaaatatggagtatgacgctgttgcaacaacacactcctgggttcccaaaaggaatcgatccattttgaatacgagccaaagggggatacttgaggatgagcaaatgtatcagaggtgctgcaatttatttgaaaaattcttcccaagtagctcatacagaagaccagttggaatatccagtatggtagaggctatggtttccagagcccgaattgatgcacggattgatttcgaatctggaaggataaagaaagaggaattcgctgagatcatgaagacctgttccaccattgaagacctcagacggcaaaaatag

>H1N1_PB1_CY010218_A/Canterbury/30/2001

atggatgtcaatccgaccttacttttcttaaaagtgccagcacaaaatgctataagcacaactttcccttatactggtgatcctccttacagccatgggacaggaacagggtacaccatggatacagtcaacaggacacatcagtactcagaaagaggaagatggacaaaaaataccgaaactggagcaccgcaactcaacccaattgatggaccactaccaaaagacaatgaaccaagtggctatgcccaaacagattgtgtattagaagcaatggctttccttgaggaatcccatcctggtatttttgaaaactcttgtattgaaacaatggaggttgttcagcaaacaagggtggacaaactgacacaaggcagacagacctatgactggactctaaatagaaaccagcctgctgccacagcattggccaacactatagaagtgttcagatcaaacggcctcatagcaaatgaatctgggaggctaatagacttccttaaagatgtaatggagtcgatggacagagacgaagtagagatcacaacacattttcagagaaagaggagggtgagagacaatgtaactaaaaaaatggtgacccaaagaacaataggcaaaaagaaacataaattagacaaaagaagttacctaattagggcattaaccctgaacacaatgaccaaagatgctgagagggggaaactaaaacgtagagcaattgcaaccccaggaatgcaaataagggggtttgtatactttgttgagacactggcaagaagcatatgtgaaaagcttgaacaatcagggttgccagttgggggaaatgaaaagaaagcaaagttagcaaatgttgtaaggaagatgatgaccaactcccaggacactgaaatttctttcaccatcactggagataacacaaaatggaacgaaaatcaaaaccctagaatgtttttggccatgatcacatatataaccaaaaatcagcctgaatggttcagaaatattctaagtattgctccaataatgttttcaaacaaaatggcgagactaggtaaggggtacatgtttgaaagcaagagtatgaaactgagaactcaaatacctgcagagatgctagccaacatagatttgaaatatttcaatgattcaactaaaaagaaaattgaaaaaatccggccattattaatagatggaactgcatcattgagtcctggaatgatgatgggcatgttcaatatgttaagcactgtcttgggcgtctccattctgaatcttgggcaaaagagatacaccaagactacttactggtgggatggtcttcaatcgtctgatgattttgctctgattgtgaatgcatccaactatgcaggaattcaagctggagttgacaggttctatcgaacctgtaagctgctcggaattaatatgagcaaaaagaagtcttacataaacagaacaggtacctttgaattcacgagctttttctatcgttatgggtttgttgccaatttcagcatggagcttcctagttttggggtgtctggggtcaatgagtctgcagacatgagtattggagtcactgtcatcaaaaacaatatgataaacaatgaccttggcccagcaactgctcaaatggcccttcagttatttatcaaagattacaggtacacgtatcgatgtcacagaggtgacacacaaatacaaacccggagatcatttgagataaagaaactatgggaccaaacccgctccaaagctgggctgttggtctctgatggaggccccaatttatataacattagaaatctccatattcctgaagtctgcttgaaatgggagttgatggatgaggattaccaggggcgtttatgcaacccattgaacccatttgtcagtcataaagagattgagtcagtgaacaatgcagtgatgatgccggcacatggtccagccaaaaatatggagtatgacgctgttgcaacaacacactcctgggttcccaaaaggaatcgatccattttgaatacgagccaaagggggatacttgaggatgagcaaatgtatcagaggtgctgcaatttatttgaaaaattcttcccaagtagttcatacagaagaccagttggaatatccagtatggtagaggctatggtttccagagcccgaattgatgcaagaattgatttcgaatctggaaggataaagaaagaggaattcgctgagatcatgaagacctgttccaccattgaagacctcagacggcaaaaatag

>H1N1_PB1_CY010282_A/Canterbury/58/2001

atggatgtcaatccgaccttacttttcttaaaagtgccagcacaaaatgctataagcacaactttcccttatactggtgatcctccttacagccatgggacaggaacagggtacaccatggatacagtcaacaggacacatcagtactcagaaagaggaagatggacaaaaaataccgaaactggagcaccgcaactcaacccaattgatggaccactaccaaaagacaatgaaccaagtggctatgcccaaacagattgtgtattagaagcaatggctttccttgaggaatcccatcctggtatttttgaaaactcttgtattgaaacaatggaggttgttcagcaaacaagggtggacaaactgacacaaggcagacagacctatgactggactctaaatagaaaccagcctgctgccacagcattggccaacactatagaagtgttcagatcaaacggcctcatagcaaatgaatctgggaggctaatagacttccttaaagatgtaatggagtcgatggacagagacgaagtagagatcacaacacattttcagagaaagaggagggtgagagacaatgtaactaaaaaaatggtgacccaaagaacaataggcaaaaagaaacataaattagacaaaagaagttacctaattagggcattaaccctgaacacaatgaccaaagatgctgagagggggaaactaaaacgtagagcaattgcaaccccaggaatgcaaataagggggtttgtatactttgttgagacactggcaagaagcatatgtgaaaagcttgaacaatcagggttgccagttgggggaaatgaaaagaaagcaaagttagcaaatgttgtaaggaagatgatgaccaactcccaggacactgaaatttctttcaccatcactggagataacacaaaatggaacgaaaatcaaaaccctagaatgtttttggccatgatcacatatataaccaaaaatcagcctgaatggttcagaaatattctaagtattgctccaataatgttttcaaacaaaatggcgagactaggtaaggggtacatgtttgaaagcaagagtatgaaactgagaactcaaatacctgcagagatgctagccaacatagatttgaaatatttcaatgattcaactaaaaagaaaattgaaaaaatccggccattattaatagatggaactgcatcattgagtcctggaatgatgatgggcatgttcaatatgttaagcactgtcttgggcgtctccattctgaatcttgggcaaaagagatacaccaagactacttactggtgggatggtcttcaatcgtctgatgattttgctctgattgtgaatgcatccaactatgcaggaattcaagctggagttgacaggttttatcgaacctgtaagctgctcggaattaatatgagcaaaaagaagtcttacataaacagaacaggtacctttgaattcacgagctttttctatcgttatgggtttgttgccaatttcagcatggagcttcctagttttggggtgtctggggtcaatgagtctgcagacatgagtattggagtcactgtcatcaaaaacaatatgataaacaatgaccttggcccagcaactgctcaaatggcccttcagttatttatcaaagattacaggtacacgtatcgatgccacagaggtgacacacaaatacaaacccgaagatcatttgagataaagaaactatgggaccaaacccgctccaaagctgggctgttggtctctgatggaggccccaatttatataacattagaaatctccatattcctgaagtctgcttgaaatgggagttgatggatgaggattaccaggggcgtttatgcaacccattgaacccatttgtcagtcataaagagattgagtcagtgaacaatgcagtgatgatgccggcacatggtccagccaaaaatatggagtatgacgctgttgcaacaacacactcctgggttcccaaaaggaatcgatccattttgaatacgagccaaagggggatacttgaggatgagcaaatgtatcagaggtgctgcaatttatttgaaaaattcttcccaagtagttcatacagaagaccagttggaatatccagtatggtagaggctatggtttccagagcccgaattgatgcaagaattgatttcgaatctggaaggataaagaaagaggaattcgctgagatcatgaagacctgttccaccattgaagacctcagacggcaaaaatag

>H1N1_PB1_CY010370_A/Baylor/11515/1982

atggatgtcaatccgaccttacttttcttaaaagtgccagcacaaaatgctataagcacgactttcccttatactggagaccctccttacagccatgggacaggaacagggtacaccatggatacagtcaacaggacacatcagtactcagaaaggggaagatggacaaaaaacaccgaaactggagcaccgcaactcaacccgattgatgggccactaccaaaagacaatgaaccaagtggttatgcccaaacagattgtgtattagaagcaatggctttccttgaggaatcccatcctggtatttttgaaaactcgtgtattgaaacgatggaggttgttcagcaaacacgagtggacaaactgacacaaggcagacagacctatgactggactctaaatagaaaccagcctgctgctacagcattggccaacaccatagaagtgttcagatcaaacggcctcatggcaaatgaatctggaaggctaatagacttccttaaagatgtaatggagtcaatggacagggaagaagtagagatcacaactcattttcagagaaagagaagagtgagggacaatgtgactaagaaaatggtgacacagagaacaataggcaaaaagaaacataaattaaacaaaaggagttatctaattagggcattgactctgaacacaatgaccaaagacgctgagagggggaaactaaaacgtagagcaattgcaaccccagggatgcaaataagggggtttgtatactttgttgagacactggcaagaagtatatgtgaaaaacttgaacaatccgggttgccagttggaggaaatgaaaagaaagcaaagttagcaaatgttgtaaggaagatgatgaccaattctcaggacactgaaatttctttcaccatcactggagataacacaaaatggaacgaaaatcagaaccctagaatgtttttggccatgatcacatatataaccagaaatcagcccgaatggttcagaaatattctaagtattgctccaataatgttctcaaacaaaatggcgagactaggtaaggggtacatgtttgaaagcaagagtatgaaacttagaactcaaatacctgcagaaatgctagcaaacatcgatctgaagtatttcaatgattcaacaagaaagaaaattgaaaaaatccggccactcctaatagatggaactgcatcactgagccctggaatgatgatgggcatgttcaatatgttaagcactgtattgggcgtctccatcctgaatcttggacaaaagagatacaccaagactacttactggtgggatggtcttcaatcgtctgatgattttgctctgattgtgaatgcacccaatcatgcaggaattcaagctggagttgacaggttttatagaacctgtaagctgctcggaatcaatatgagcaagaaaaagtcttacatgaacagaacaggtacttttgaattcacgagctttttctatcgttatgggtttgttgccaatttcagcatggagcttcccagttttggggtgtctgggatcaacgagtctgcagacatgagtattggagtcactgtcatcaaaaacaatatgataaacaatgatcttggtccagcaaccgctcaaatggcccttcagttattcatcaaagattacaggtacacgtatcgatgccatagaggtgacacacaaatacaaactcgaagatcatttgaaataaagaaactatgggatcaaacccgttccaaagctgggctgttggtctctgatggaggtcctaatttatacaacattagaaatctccacattcctgaagtctgcttgaaatgggaattaatggatgaggattaccgggggcgtttatgcaacccattgaacccatttgtcagtcataaagaaattgaatcagtgaacaatgcagtggtgatgccggcacatggtccagccaaaaatatggagtatgatgctgttgcaacaacacactcctgggtccccaaaaggaatcgatccatcttgaatacgagccaaagaggaatacttgaggatgaacaaatgtatcaaaggtgctgcaatttatttgaaaaattcttccccagcagttcatacagaagaccagtcggaatatccagtatggtagaggctatggtttccagagcccgaattgatgcacggattgatttcgaatctggaaggataaagaaagaggagttcactgagatcatgaagacctgttccaccattgaagaactcagacggcaaaaatag

>H1N1_PB1_DQ508895_A/USSR/90/1977

atggatgtcaatccgaccttacttttcttaaaagtgccagcacaaaatgctataagcacaactttcccttatactggagaccctccttacagccatgggacaggaacagggtacaccatggatacagtcaacaggacacatcagtactcagaaaggggaagatggacaaaaaacaccgaaactggagcaccgcaactcaacccgattgatgggccactaccaaaagacaatgaaccaagtggttatgcccaaacagattgtgtattagaagcaatggctttccttgaggaatcccatcctggtatttttgaaaactcgtgtattgaaacgatggaggttgttcagcaaacacgagtggacaaactgacacaaggcagacagacctatgactggactctaaatagaaaccagcctgctgctacagcattggccaacaccatagaagtgttcagatcaaatggcctcatggcaaatgaatctggaaggctaatagacttccttaaagatgtaatggagtcaatggacagagaagaagtggagatcacaactcattttcagagaaagagaagagtgagggacaatgtgactaagaaaatggtgacacagagaacaataggcaaaaagaaacagagattaaacaaaaggagttatctaattagggcattaaccctgaacacaatgaccaaagatgctgagagagggaaactaaaacgtagagcaattgcaaccccagggatgcaaataagggggtttgtatactttgttgagacactggcaagaagtatatgtgaaaaacttgaacaatcagggttgccagttggaggaaatgaaaagaaagcaaagttggcaaatgttgtaaggaagatgatgaccaattctcaggacactgaaatttctttcaccatcactggagataacaccaaatggaacgaaaatcagaaccctagaatgtttttggccatgatcacatatataaccagaaatcagcccgaatggttcagaaatattctaagtattgctccaataatgttctcaaacaaaatggcgagactaggtaaggggtacatgtttgaaagcaagagtatgaaacttagaactcaaatacctgcagaaatgctagcaaacatcgatttgaagtatttcaatgattcaacaagaaagaaaattgaaaaaatccggccactcttaatagatgggactgcatcactgagccctggaatgatgatgggcatgttcaatatgttaagcactgtattgggcgtctccatcctgaatcttggacaaaagagatacaccaagactacttactggtgggatggtcttcaatcgtctgatgattttgctctgattgtgaatgcacccaaccatgcaggaattcaagctggagttgacaggttttatcgaacctgtaagctgctcggaatcaatatgagcaagaaaaagtcttacataaacagaacaggtacatttgaattcacgagctttttctatcgttatgggtttgttgccaatttcagcatggagcttcccagttttggggtgtctgggatcaacgagtctgcagacatgagtattggagtcactgtcatcaaaaacaatatgataaacaatgatcttggtccagcaaccgctcaaatggcccttcagttattcatcaaagattacaggtacacgtatcgatgccacagaggtgacacacaaatacaaacccgaagatcatttgaaataaagaaactgtgggagcaaacccgttccaaagctgggctgctggtctccgatggaggtcctaatttatacaacattagaaatctccacattcctgaagtctgcttgaaatgggaattgatggatgaggattaccaggggcgtttatgcaacccattgaacccatttgtcagtcataaagagattgaatcagtgaacaatgcagtgatgatgccggcacatggtccagccaaaaatatggagtatgatgctgttgcaacaacacactcctgggtccccaaaaggaatcgatccatcttgaatacgagccaaagaggaatacttgaagatgaacaaatgtatcaaaggtgctgcaatttatttgaaaaattcttccccagcagttcatacagaaggccagtcggaatatccagtatggtagaggctatggtttccagagcccgaattgatgcacggattgatttcgaatctggaaggataaagaaagaggagttcactgagatcatgaagacctgttccaccattgaagaactcagacggcaaaaatag

>H1N1_PB1_CY010770_A/Canterbury/20/2001

atggatgtcaatccgaccttacttttcttaaaagtgccagcacaaaatgctataagcacaactttcccttatactggtgatcctccttacagccatgggacaggaacagggtacaccatggatacagtcaacaggacacatcagtactcagaaagaggaagatggacaaaaaataccgaaactggagcaccgcaactcaacccaattgatggaccactaccaaaagacaatgaaccaagtggctatgcccaaacagattgtgtattagaagcaatggctttccttgaggaatcccatcctggtatttttgaaaactcttgtattgaaacaatggaggttgttcagcaaacaagggtggacaaactgacacaaggcagacagacctatgactggactctaaatagaaaccagcctgctgccacagcattggccaacactatagaagtgttcagatcaaacggcctcatagcaaatgaatctgggaggctaatagacttccttaaagatgtaatggagtcgatggacagagacgaagtagagatcacaacacattttcagagaaagaggagggtgagagacaatgtaactaaaaaaatggtgacccaaagaacaataggcaaaaagaaacataaattagacaaaagaagttacctaattagggcattaaccctgaacacaatgaccaaagatgctgagagggggaaactaaaacgtagagcaattgcaaccccaggaatgcaaataagggggtttgtatactttgttgagacactggcaagaagcatatgtgaaaagcttgaacaatcagggttgccagttgggggaaatgaaaagaaagcaaagttagcaaatgttgtaaggaagatgatgaccaactcccaggacactgaaatttctttcaccatcactggagataacacaaaatggaacgaaaatcaaaaccctagaatgtttttggccatgatcacatatataaccaaaaatcagcctgaatggttcagaaatattctaagtattgctccaataatgttttcaaacaaaatggcgagactaggtaaggggtacatgtttgaaagcaagagtatgaaactgagaactcaaatacctgcagagatgctagccaacatagatttgaaatatttcaatgattcaactaaaaagaaaattgaaaaaatccggccattattaatagatggaactgcatcattgagtcctggaatgatgatgggcatgttcaatatgttaagcactgtcttgggcgtctccattctgaatcttgggcaaaagagatacaccaagactacttactggtgggatggtcttcaatcgtctgatgattttgctctgattgtgaatgcatccaactatgcaggaattcaagctggagttgacaggttttatcgaacctgtaagctgctcggaattaatatgagcaaaaagaagtcttacataaacagaacaggtacctttgaattcacgagctttttctatcgttatgggtttgttgccaatttcagcatggagcttcctagttttggggtgtctggggtcaatgagtctgcagacatgagtattggagtcactgtcatcaaaaacaatatgataaacaatgaccttggcccagcaactgctcaaatggcccttcagttatttatcaaagattacaggtacacgtatcgatgtcacagaggtgacacacaaatacaaacccggagatcatttgagataaagaaactatgggaccaaacccgctccaaagctgggctgttggtctctgatggaggccccaatttatataacattagaaatctccatattcctgaagtctgcttgaaatgggagttgatggatgaggattaccaggggcgtttatgcaacccattgaacccatttgtcagtcataaagagattgagtcagtgaacaatgcagtgatgatgccggcacatggtccagccaaaaatatggagtatgacgctgttgcaacaacacactcctgggttcccaaaaggaatcgatccattttgaatacgagccaaagggggatacttgaggatgagcaaatgtatcagaggtgctgcaatttatttgaaaaattcttcccaagtagttcatacagaagaccagttggaatatccagtatggtagaggctatggtttccagagcccgaattgatgcaagaattgatttcgaatctggaaggataaagaaagaggaattcgctgagatcatgaagacctgttccaccattgaagacctcagacggcaaaaatag

>H1N1_PB1_CY010874_A/Memphis/10/1978

atggatgtcaatccgaccttacttttcttaaaagtgccagcacaaaatgctataagcacaactttcccttatactggagaccctccttacagccatgggacaggaacagggtacaccatggatacagtcaacaggacacatcagtactcagaaaggggaagatggacaaaaaacaccgaaactggagcaccgcaactcaacccgattgatgggccactaccaaaagacaatgaaccaagtggttatgcccaaacagattgtgtattagaagcaatggctttccttgaggaatcccatcctggtatttttgaaaactcgtgtattgaaacgatggaggttgttcagcaaacacgagtggacaaactgacacaaggcagacagacctatgactggactctaaatagaaaccagcctgctgctacagcattggccaacaccatagaagtgttcagatcaaatggcctcatggcaaatgaatctggaaggctaatagacttccttaaagatgtaatggagtcaatggacagagaagaagtggagatcacaactcattttcagagaaagagaagagtgagggacaatgtgactaagaaaatggtgacacagagaacaataggcaaaaagaaacagagattaaacaaaaggagttatctaattagggcattaaccctgaacacaatgaccaaagatgctgagagagggaaactaaaacgtagagcaattgcaaccccagggatgcaaataagggggtttgtatactttgttgagacactggcaagaagtatatgtgaaaaacttgaacaatcagggttgccagttggaggaaatgaaaagaaagcaaagttggcaaatgttgtaaggaagatgatgaccaattctcaggacactgaaatttctttcaccatcactggagataacaccaaatggaacgaaaatcagaaccctagaatgtttttggccatgatcacatatataaccagaaatcagcccgaatggttcagaaatattctaagtattgctccaataatgttctcaaacaaaatggcgagactaggtaaggggtacatgtttgaaagcaagagtatgaaacttagaactcaaatacctgcagaaatgctagcaaacatcgatttgaagtatttcaatgattcaacaagaaagaaaattgaaaaaatccggccactcttaatagatgggactgcatcactgagccctggaatgatgatgggcatgttcaatatgttaagcactgtattgggcgtctccatcctgaatcttggacaaaagagatacaccaagactacttactggtgggatggtcttcaatcgtctgatgattttgctctgattgtgaatgcacccaaccatgcaggaattcaagctggagttgacaggttttatcgaacctgtaagctgctcggaatcaatatgagcaagaaaaagtcttacataaacagaacaggtacatttgaattcacgagctttttctatcgttatgggtttgttgccaatttcagcatggagcttcccagttttggggtgtctgggatcaacgagtctgcagacatgagtattggagtcactgtcatcaaaaacaatatgataaacaatgatcttggtccagcaaccgctcaaatggcccttcagttattcatcaaagattacaggtacacgtatcgatgccacagaggtgacacacaaatacaaacccgaagatcatttgaaataaagaaactgtgggagcaaacccgttccaaagctgggctgctggtctccgatggaggtcctaatttatacaacattagaaatctccacattcctgaagtctgcttgaaatgggaattgatggatgaggattaccaggggcgtttatgcaacccattgaacccatttgtcagtcataaagagattgaatcagtgaacaatgcagtgatgatgccggcacatggtccagccaaaaatatggagtatgatgctgttgcaacaacacactcctgggtccccaaaaggaatcgatccatcttgaatacgagccaaagaggaatacttgaagatgaacaaatgtatcaaaggtgctgtaatttatttgaaaaattcttccccagcagttcatacagaaggccagtcggaatatccagtatggtagaggctatggtttccagagcccgaattgatgcacggattgatttcgaatctggaaggataaagaaagaggagttcactgagatcatgaagacctgttccaccattgaagaactcagacggcaaaaatag

>H1N1_PB1_CY014013_A/Wellington/11/2005

atggatgtcaatccgacattacttttcttaaaagtgccagcacaaaatgctataagcacaacttttccttatactggtgaccctccttacagccatgggacaggaacagggtacaccatggatacagtcaacaggacacaccagtactcagaaagaggaagatggacaaaaaataccgaaactggagcaccgcaactcaacccaattgatgggccactaccagaagacaatgaaccaagtggctatgcccaaacagattgtgtattagaagcaatggctttccttgaagaatcccatcctggtatctttgaaaactcttgtattgaaacaatggaggttgttcagcaaacaagggtggacaaactgacacaaggcagacagacctatgactggactctaaataggaaccagcctgctgccacagcattggcaaacactatagaagtattcagatcaaacggcctcatagcaaatgaatctgggaggctaatagacttccttaaagatgtaatggagtcgatggacagagacgaagtagaggtcacaactcattttcaaagaaagaggagagtgagagacaatgtaactaaaaaaatggtgacccaaagaacaataggcaaaaagaaacataaattagacaaaagaagttacctaattagggcattaaccctgaacacaatgaccaaagatgctgagagggggaaactaaaacgcagagcaattgcaaccccaggaatgcaaataagggggtttgtatactttgttgagacactggcaagaagcatatgtgaaaagcttgaacaatcaggattgccagttggagggaatgagaagaaagcaaagttagcaaatgttgtaaggaagatgatgaccaactcccaggacactgaaatttctttcaccatcactggagataacacaaaatggaacgaaaatcaaaaccctagaatgttcttggccatgatcacatatataaccaaaaatcagcctgaatggttcagaaatattctaagtattgctccaataatgttttcaaacaagatggcgagactaggtaaggggtacatgtttgaaagcaagagtatgaaactgagaactcaaatacctgcagagatgctagccaacatagatttgaaatatttcaatgattcaactaaaaagaaaattgaaaaaatccgaccattattaatagatggaactgcatcattgagtcctggaatgatgatgggcatgttcaatatgttgagcaccgtcttgggcgtctccattctgaatcttgggcaaaagagatacaccaagactacatactggtgggatggtcttcaatcgtctgatgattttgctttgattgtgaatgcacccaactatgcaggaattcaagctggagttgacaggttttatcgaacctgtaagctgctcggaattaatatgagcaaaaagaagtcttacataaacagaacaggtaccttcgaattcacgagctttttctatcgttatgggtttgttgccaatttcagcatggagcttcctagttttggggtgtctggggtcaatgaatctgcagacatgagtattggagtcactgtcatcaaaaacaatatgataaacaatgaccttggcccagcaactgctcaaatggcccttcagttatttataaaagattacaggtacacttatcgatgccacagaggtgacacacaaatacaaacccggagatcatttgagataaagaaactatgggaccaaacccgctccaaagctgggctgttggtctctgatggaggccccaatttatataacattagaaatctccatattcctgaagtttgcttgaaatgggagttgatggatgaggattaccaggggcgtttatgcaacccattaaacccgtttgtcagccataaagagattgaatcagtgaacaatgcagtgataatgccggcacatggtccagccaaaaatatggagtatgacgctgttgcaacaacacactcctgggtccccaaaagaaatcgatccattttgaacacgagccaaagggggatacttgaagatgagcaaatgtatcagaggtgctgcaatttatttgaaaaattcttcccaagtagctcatacagaagaccagttggaatatccagtatggtagaggctatggtttcaagagcccgaattgatgcacggattgatttcgaatctggaaggataaagaaagaggaatttgctgagatcatgaagatctgttccaccattgaagacctcagacggcaaaaatag

>H1N1_PB1_CY016258_A/Nanchang/25/1996

atggatgtcaatccgaccttacttttcttaaaagtgccagcacaaaatgctataagcacaacttttccttatactggagaccctccttacagccatgggacaggaacagggtacaccatggatacagtcaacaggacacatcagtactcagaaagaggaagatggacaaaaaataccgaaactggagcaccgcaactcaacccaattgatgggccactaccaaaagacaatgaaccaagtggctatgcccaaacagattgtgtattagaagcaatggctttccttgaggaatcccatcctggtatttttgaaaactcttgtattgaaacaatggaggttgttcagcaaacaagggtggacaaactgacacaaggcagacagacctatgactggactctaaatagaaaccagcctgctgccacagcattggccaacactatagaagtgttcagatcaaacggcctcatagcaaatgaatctgggaggctaatagacttccttaaagatgtaatggagtcgatggacagagacgaagtagagatcacaactcattttcagagaaagaggagagtgagagacaatgtaactaaaaaaatggtgacccaaagaacaataggcaaaaagaaacataaattagacaaaagaagttacctaattagggcattaaccctgaacacaatgaccaaagatgctgagagggggaaactaaaacgtagagcaattgcaaccccaggaatgcaaataagggggtttgtatactttgttgagacactggcaaggagcatatgtgaaaagcttgaacaatcagggttgccagttggaggaaatgaaaagaaagcaaagttagcaaatgttgtaaggaagatgatgaccaactcccaggacactgaaatttctttcaccatcactggagataacacaaaatggaacgaaaatcaaaaccctagaatgttcttggccatgatcacatatataaccaaaaatcagcctgaatggttcagaaatattctaagtattgctccaataatgttttcaaacaaaatggcgagactaggtaaggggtacatgtttgaaagcaagagtatgaaactgagaactcaaatacctgcagagatgctagccaacatagatttgaaatatttcaatgattcaacaaaaaagaaaattgaaaaaatccggccattattaatagatggaactgcatcattgagtcctggaatgatgatgggcatgttcaatatgttaagcactgtcttgggcgtctccattctgaatcttgggcaaaagagatacaccaagactacttactggtgggatggtcttcaatcgtctgatgattttgctctgattgtgaatgcacccaactatgcaggaattcaagctggagttgacaggttttatcgaacctgtaagctgctcggaattaatatgagcaaaaagaagtcttacataaacagaacaggtacctttgaattcacgagctttttctatcgttatgggtttgttgccaatttcagcatggagcttcctagttttggggtgtctggggtcaatgagtctgcagacatgagtattggagtcactgtcatcaaaaacaatatgataaacaatgatcttggcccagcaactgctcaaatggcccttcagttatttatcaaagattacaggtacacgtatcgatgccacagaggtgacacacaaatacaaacccggagatcatttgagataaagaaactatgggaccaaacccgctccaaagctgggctgttggtctctgatggaggccccaatttatataacattagaaatctccacattcctgaagtctgcttgaaatgggagttgatggatgaggattaccaggggcgtttatgcaacccattgaacccgtttgtcagtcataaagagattgaatcagtgaacaatgcagtgatgatgccggcacatggtccagccaaaaatatggagtatgacgctgttgcaacaacacactcctgggttcccaaaaggaatcgatccattttgaatacgagccaaagggggatacttgaggatgagcaaatgtatcagaggtgctgcaatttatttgaaaaattcttcccaagtagctcatacagaagaccagttggaatatccagtatggtagaggctatggtttccagagcccgaattgatgcacggattgatttcgaatctggaaggataaagaaagaggagttcgctgagatcatgaagacctgttccaccattgaagacctcagacggcaaaaatag

>H1N1_PB1_CY016737_A/South Australia/64/2000

atggatgtcaatccgactttacttttcttaaaagtgccagcacaaaatgctataagcacaactttcccctatactggagaccccccttacagccatgggacagggacagggtacaccatggatacagttaacaggacacatcagtactcagaaagaggaagatggacaaaaaataccgaaactggagcaccgcaactcaacccgattgatgggccattaccaaaagacaatgagccaagtggctatgcccaaacagattgtgtattagaagcaatggctttccttgaggaatcccatcctggtatttttgaaaactcatgtattgaaacgatggaggctgttcagcaaacaagagtggacaaactgacacaaggaagacagacctatgactggactctaaatagaaaccagcctgctgccacagcattggccaacactatagaagtgttcagatcaaacggcctcatagcaaatgagtctgggaggctaatagacttccttaaagatgtaatggagtcaatggacagagaagaagtagagatcacaactcactttcagagaaagagaagagtgagagacaatgtaactaaaaaaatggtgacacaaagaacaataggcaaaaagaaacataaattggacaaaagaagttatctaattagggcattaaccctgaacacaatgaccaaagatgctgagagggggaaactaaaacgtagagctattgcaaccccaggaatgcagataagggggtttgtatactttgttgagacactggcaaggagcatatgtgagaaacttgagcaatcagggttaccagttggaggaaatgagaagaaagcaaagttagcaaatgttgtaaggaagatgatgaccaactcccaggacactgaaatttctttcaccatcactggagataacacaaaatggaacgaaaatcagaaccctagaatgtttttggctatgatcacatatataaccagaaatcagcctgaatggttcagaaacattctaagtattgctccaataatgttttcaaacaaaatggcgagactaggtaaggggtacatgtttgaaagcaagagtatgaagctgagaactcaaatacctgcagaaatactggccaacatagatttgaaatatttcaatgattcaacaaaaaagaaaattgaaaaaatccggccattattaatagatggaactgcatcactgagtcctggaatgatgatgggcatgttcaatatgttgagcactgtcttgggcgtctccatcctgaatcttggacaaaagagatacaccaagaccacttactggtgggatggtcttcaatcgtctgatgattttgctctgattgtgaatgcgcccaactatgcaggaattcaagctggagttgacaggttttatcgaacctgtaagctgctcggaattaatatgagcaaaaagaagtcttacataaacaggacaggtacttttgaatttacaagctttttctatcgttatgggtttgttgccaattttagcatggaacttcctagttttggggtgtctggggttaatgagtctgcagacatgagtattggaatcactgtcatcaaaaataatatgataaacaatgatcttggtccagcaactgcccaaatggcccttcagttattcatcaaagattacaggtatacgtatcgatgccacagaggtgacacacaaatacaaacccggagatcatttgagataaaaaaactgtgggaccaaacccgttccaaagctgggctgttggtctctgatggaggccccaatttatataatattagaaatctccacattcctgaagtctgcttgaaatgggagttgatggatgaggattaccaagggcgtttatgcaatccattgaacccttttgtcagtcataaagaaattgaatcagtgaacaatgcagtgatgatgccggcacatggtccagccaaaaatgtggaatatgacgctgttgcaacaacgcactcctgggtccccaaaaggaatcgatccattttgaatacgagccaaagaggaatacttgaggatgagcaaatgtatcaaaggtgctgcaatttgtttgaaaagttcttcccaagcagctcatatagaagaccagttggaatatccagtatggtagaggccatggtttccagagcccgaatcgatgcacggattgatttcgaatctggaaggataaagaaagaagagttcgctgagatcatgaagacctgttccaccattgaagacctcagacggcaaaaatag

>H1N1_PB1_CY017225_A/Memphis/27/1983

atggatgtcaatccgaccttacttttcttaaaagtgccagcacaaaatgctataagcacaactttcccttatactggagaccctccttacagccatgggacaggaacagggtacaccatggatacagtcaacaggacacatcagtactcagaaaggggaagatggacaaaaaacaccgaaactggagcaccgcaactcaacccgattgatgggccactaccaaaagacaatgaaccaagtggttatgcccaaacagattgtgtattagaagcaatggctttccttgaggaatcccatcccggtatttttgaaaactcgtgtattgaaacgatggaggttgttcagcaaacacgtgtagacaaactgacacaaggcagacagacctatgactggactctaaatagaaaccagcctgctgctacagcattggccaacaccatagaagtgttcagatcaaacggcctcatggcaaatgaatctggaaggctaatagacttccttaaagatgtaatggattcaatggatagagaagaaatggagatcacaactcattttcagagaaagagaagagtgagggacaatgtaactaagaaaatggtgacacaaagaacaataggcaaaaagaaacatagattaaacaaaaggagttatctaattagggcattaaccctgaacacaatgaccaaagatgctgagagggggaaactaaaacgtagagcaattgcaaccccagggatgcaaataagggggtttgtatactttgttgagacactggcaagaagtatatgtgaaaaacttgaacaatcagggttgccagttggaggaaatgaaaagaaagcaaagttagcaaatgttgtaaggaaaatgatgaccaattctcaggacactgaaatttctttcaccatcactggagataacacaaaatggaacgaaaatcagaaccctagaatgtttttggccatgatcacatatataaccagaaatcagcccgaatggttcagaaatattctaagtattgctccaataatgttctcaaacaaaatggcgagactaggtaaggggtacatgtttgaaagcaagagtatgaaacttagaactcaaatacctgcagaaatgctagcaaacatcgatttgaggtatttcaatgattcaacaagaaagaaaattgaaaaaatcaggccactcttaatagatggaactgcatcactgagccctggaatgatgatgggcatgttcaatatgttaagcactgtattgggcgtctccatcctgaatcttggacaaaagagatacaccaagactacttactggtgggatggtcttcaatcgtctgatgattttgctctgattgtgaatgcacccaactatgcaggaattcaagctggagttgacaggttttatcgaacctgtaagctgctcggaatcaacatgagcaagaaaaagtcctacataaacagaacaggtacttttgaattcacgagctttttttatcgttatgggtttgttgccaatttcagcatggagcttcccagttttggggtgtctgggatcaacgagtctgcagacatgagtattggagtcactgtcatcaaaaacaatatgataaacaatgatcttggtccagcaaccgctcaaatggcccttcagttattcatcaaagattacaggtacacatatcgatgccacagaggtgacacacaaatacaaacccgaagatcatttgaaataaagaaactgtgggatcaaacccgttccaaaactgggctgctggtctctgatggaggtcctaatttgtacaacattagaaatctccacattcctgaagtctgcttgaaatgggatttgatggatgaggattaccaggggcgtttatgcaacccattgaacccatttgtcagtcataaagagattgaatcagtgaacaatgcagtgatgatgccggcacatggtccagccaaaattatggagtatgatgctgttgcaacaacacactcctgggtccccaaaaggaatcgatccatcttgaatacgagccaaaggggaatacttgaggatgaacaaatgtatcagaggtgctgcaatttatttgaaaaattcttccccagcagttcatacagaagaccagtcggaatatccagtatggtagaggctatgatttccagagcccgaattgatgcacggattgatttcgaatctggaaggataaagaaagaggagttcactgagatcatgaagacctgttccaccattgaagaactcagacggcaaaaatag

>H1N1_PB1_CY017385_A/South Australia/26/2000

atggatgtcaatccgactttacttttcttaaaagtgccagcacaaaatgctataagcacaactttcccctatactggagaccccccttacagccatgggacagggacagggtacaccatggatacagttaacaggacacatcagtactcagaaagaggaagatggacaaaaaataccgaaactggagcaccgcaactcaacccgattgatgggccattaccaaaagacaatgagccaagtggctatgcccaaacagattgtgtattagaagcaatggctttccttgaggaatcccatcctggtatttttgaaaactcatgtattgaaacgatggaggctgttcagcaaacaagagtggacaaactgacacaaggaagacagacctatgactggactctaaatagaaaccagcctgctgccacagcattggccaacactatagaagtgttcagatcaaacggcctcatagcaaatgagtctgggaggctaatagacttccttaaagatgtaatggagtcaatggacagagaagaagtagagatcacaactcactttcagagaaagagaagagtgagagacaatgtaactaaaaaaatggtgacacaaagaacaataggcaaaaagaaacataaattggacaaaagaagttatctaattagggcattaaccctgaacacaatgaccaaagatgctgagagggggaaactaaaacgtagagctattgcaaccccaggaatgcagataagggggtttgtatactttgttgagacactggcaaggagcatatgtgagaaacttgagcaatcagggttaccagttggaggaaatgagaagaaagcaaagttagcaaatgttgtaaggaagatgatgaccaactcccaggacactgaaatttctttcaccatcactggagataacacaaaatggaacgaaaatcagaaccctagaatgtttttggctatgatcacatatataaccagaaatcagcctgaatggttcagaaacattctaagtattgctccaataatgttttcaaacaaaatggcgagactaggtaaggggtacatgtttgaaagcaagagtatgaagctgagaactcaaatacctgcagaaatactggccaacatagatttgaaatatttcaatgattcaacaaaaaagaaaattgaaaaaatccggccattattaatagatggaactgcatcactgagtcctggaatgatgatgggcatgttcaatatgttgagcactgtcttgggcgtctccatcctgaatcttggacaaaagagatacaccaagaccacttactggtgggatggtcttcaatcgtctgatgattttgctctgattgtgaatgcgcccaactatgcaggaattcaagctggagttgacaggttttatcgaacctgtaagctgctcggaattaatatgagcaaaaagaagtcttacataaacaggacaggtacttttgaatttacaagctttttctatcgttatgggtttgttgccaattttagcatggaacttcctagttttggggtgtctggggttaatgagtctgcagacatgagtattggaatcactgtcatcaaaaataatatgataaacaatgatcttggtccagcaactgcccaaatggcccttcagttattcatcaaagattacaggtatacgtatcgatgccacagaggtgacacacaaatacaaacccggagatcatttgagataaaaaaactgtgggaccaaacccgttccaaagctgggctgttggtctctgatggaggccccaatttatataatattagaaatctccacattcctgaagtctgcttgaaatgggagttgatggatgaggattaccaagggcgtttatgcaatccattgaacccttttgtcagtcataaagaaattgaatcagtgaacaatgcagtgatgatgccggcacatggtccagccaaaaatgtggaatatgacgctgttgcaacaacgcactcctgggtccccaaaaggaatcgatccattttgaatacgagccaaagaggaatacttgaggatgagcaaatgtatcaaaggtgctgcaatttgtttgaaaagttcttcccaagcagctcatatagaagaccagttggaatatccagtatggtagaggccatggtttccagagcccgaatcgatgcacggattgatttcgaatctggaaggataaagaaagaagagttcgctgagatcatgaagacctgttccaccattgaagacctcagacggcaaaaatag

>H1N1_PB1_CY019059_A/Memphis/41/1983

atggatgtcaatccgaccttacttttcttaaaagtgccagcacaaaatgctataagcacaactttcccttatactggagaccctccttacagccatgggacaggaacagggtacaccatggatacagtcaacaggacacatcagtactcagaaaggggaagatggacaaaaaacaccgaaactggagcaccgcaactcaacccgattgatgggccactaccaaaagacaatgaaccaagtggttatgcccaaacagattgtgtattagaagcaatggctttccttgaggaatcccatcccggtatttttgaaaactcgtgtattgaaacgatggaggttgttcagcaaacacgtgtagacaaactgacacaaggcagacagacctatgactggactctaaatagaaaccagcctgctgctacagcattggccaacaccatagaagtgttcagatcaaacggcctcatggcaaatgaatctggaaggctaatagacttccttaaagatgtaatggattcaatggatagagaagaaatggagatcacaactcattttcagagaaagagaagagtgagggacaatgtaactaagaaaatggtgacacaaagaacaataggtaaaaagaaacatagattaaacaaaaggagttatctaattagggcattaaccctgaacacaatgaccaaagatgctgagagggggaaactaaaacgtagagcaattgcaaccccagggatgcaaataagggggtttgtatactttgttgagacactggcaagaagtatatgtgaaaaacttgaacaatcagggttgccagttggaggaaatgaaaagaaagcaaagttagcaaatgttgtaaggaaaatgatgaccaattctcaggacactgaaatttctttcaccatcactggagataacacaaaatggaacgaaaatcagaaccctagaatgtttttggccatgatcacatatataaccagaaatcagcccgaatggttcagaaatattctaagtattgctccaataatgttctcaaacaaaatggcgagactaggtaaggggtacatgtttgaaagcaagagtatgaaacttagaactcaaatacctgcagaaatgctagcaaacatcgatttgaggtatttcaatgattcaacaagaaagaaaattgaaaaaatcaggccactcttaatagatggaactgcatcactgagccctggaatgatgatgggcatgttcaatatgttaagcactgtattgggcgtctccatcctgaatcttggacaaaagagatacaccaagactacttactggtgggatggtcttcaatcgtctgatgattttgctctgattgtgaatgcacccaactatgcaggaattcaagctggagttgacaggttttatcgaacctgtaagctgctcggaatcaacatgagcaagaaaaagtcctacataaacagaacaggtacttttgaattcacaagctttttttatcgttatgggtttgttgccaatttcagcatggagcttcccagttttggggtgtctgggatcaacgagtctgcagacatgagtattggagtcactgtcatcaaaaacaatatgataaacaatgatcttggtccagcaaccgctcaaatggcccttcagttattcatcaaagattacaggtacacatatcgatgccacagaggtgacacacaaatacaaacccgaagatcatttgaaataaagaaactgtgggatcaaacccgttccaaaactgggctgctggtctctgatggaggtcctaatttgtacaacattagaaatctccacattcctgaagtctgcttgaaatgggatttgatggatgaggattaccaggggcgtttatgcaacccattgaacccatttgtcagtcataaagagattgaatcagtgaacaatgcagtgatgatgccggcacatggtccagccaaaattatggagtatgatgctgttgcaacaacacactcctgggtccccaaaaggaatcgatccatcttgaatacgagccaaaggggaatacttgaggatgaacaaatgtatcagaggtgctgcaatttatttgaaaaattcttccccagcagttcatacagaagaccagtcggaatatccagtatggtagaggctatgatttccagagcccgaattgatgcacggattgatttcgaatctggaaggataaagaaagaggaattcactgagatcatgaagacctgttccaccattgaagaactcagacggcaaaaatag

>H1N1_PB1_CY019227_A/Memphis/31/1983

atggatgtcaatccgaccttacttttcttaaaagtgccagcacaaaatgctataagcacaactttcccttatactggagaccctccttacagccatgggacaggaacagggtacaccatggatacagtcaacaggacacatcagtactcagaaaggggaagatggacaaaaaacaccgaaactggagcaccgcaactcaacccgattgatgggccactaccaaaagacaatgaaccaagtggttatgcccaaacagattgtgtattagaagcaatggctttccttgaggaatcccatcccggtatttttgaaaactcgtgtattgaaacgatggaggttgttcagcaaacacgtgtagacaaactgacacaaggcagacagacctatgactggactctaaatagaaaccagcctgctgctacagcattggccaacaccatagaagtgttcagatcaaacggcctcatggcaaatgaatctggaaggctaatagacttccttaaagatgtaatggattcaatggatagagaagaaatggagatcacaactcattttcagagaaagagaagagtgagggacaatgtaactaagaaaatggtgacacaaagaacaataggcaaaaagaaacatagattaaacaaaaggagttatctaattagggcattaaccctgaacacaatgaccaaagatgctgagagggggaaactaaaacgtagagcaattgcaaccccagggatgcaaataagggggtttgtatactttgttgagacactggcaagaagtatatgtgaaaaacttgaacaatcagggttgccagttggaggaaatgaaaagaaagcaaagttagcaaatgttgtaaggaaaatgatgaccaattctcaggacactgaaatttctttcaccatcactggagataacacaaaatggaacgaaaatcagaaccctagaatgtttttggccatgatcacatatataaccagaaatcagcccgaatggttcagaaatattctaagtattgctccaataatgttctcaaacaaaatggcgagactaggtaaggggtacatgtttgaaagcaagagtatgaaacttagaactcaaatacctgcagaaatgctagcaaacatcgatttgaggtatttcaatgattcaacaagaaagaaaattgaaaaaatcaggccactcttaatagatggaactgcatcactgagccctggaatgatgatgggcatgttcaatatgttaagcactgtattgggcgtctccatcctgaatcttggacaaaagagatacaccaagactacttactggtgggatggtcttcaatcgtctgatgattttgctctgattgtgaatgcacccaactatgcaggaattcaagctggagttgacaggttttatcgaacctgtaagctgctcggaatcaacatgagcaagaaaaagtcctacataaacagaacaggtacttttgaattcacgagctttttttatcgttatgggtttgttgccaatttcagcatggagcttcccagttttggggtgtctgggatcaacgagtctgcagacatgagtattggagtcactgtcatcaaaaacaatatgataaacaatgatcttggtccagcaaccgctcaaatggcccttcagttattcatcaaagattacaggtacacatatcgatgccacagaggtgacacacaaatacaaacccgaagatcatttgaaataaagaaactgtgggatcaaacccgttccaaaactgggctgctggtctctgatggaggtcctaatttgtacaacattagaaatctccacattcctgaagtctgcttgaaatgggatttgatggatgaggattaccaggggcgtttatgcaacccattgaacccatttgtcagtcataaagagattgaatcagtgaacaatgcagtggtgatgccggcacatggtccagccaaaattatggagtatgatgctgttgcaacaacacactcctgggtccccaaaaggaatcgatccatcttgaatacgagccaaaggggaatacttgaggatgaacaaatgtatcagaggtgctgcaatttatttgaaaaattcttccccagcagttcatacagaagaccagtcggaatatccagtatggtagaggctatgatttccagagcccgaattgatgcacggattgatttcgaatctggaaggataaagaaagaggagttcactgagatcatgaagacctgttccaccattgaagaactcagacggcaaaaatag

>H1N1_PB1_CY019745_A/Memphis/1/1979

atggatgtcaatccgactctacttttcttaaaagttccagcgcaaaatgccataagcaccacattcccttatactggagatcctccatacagccatggaacaggaacaggatacaccatggacacagtcaacagaacacatcaatattcagaaaaagggaagtggacaacaaacacagagactggggcgccccaacttaacccgattgatggaccactacctgaggataatgagccaagtggatatgcacaaacagactgtgtcctggaagctatggctttccttgaggaatcccacccagggatctttgagaactcgtgccttgaaacaatggaagtcgttcaacaaacaagggtggacagactgacccaaggtcgtcagacctatgattggacattaaatagaaatcaaccggccgcaactgcattagccaacactatagaagttttcagatcgaatggtctaacagctaatgagtcgggaaggctaatagatttcctcaaggatgtgatggaatcaatggataaagaggaaatagagataacaacacacttccaaagaaaaagaagagtaagagacaacatgaccaagaaaatggtcacacaaagaacaataggaaagaaaaagcagagagtgaacaagagaagctatctaataagagcattaacattgaacacaatgaccaaagatgcagaaagaggtaaattaaagagaagggctattgcaacacccgggatgcaaatcagagggttcgtgtactttgttgaaactctagctaggagcatttgtgagaagcttgaacagtctggacttccagttggaggtaatgaaaagaaggccaaactggcaaatgttgtgagaaagatgatgactaattcacaagacacagagctttctttcacaattactggagacaatactaagtggaatgaaaatcaaaatcctcgaatgttcctggcgatgattacatatatcacaaaaaatcaacctgaatggttcagaaacatcctgagcatcgcacccataatgttctcaaacaaaatggcgagactagggaaaggatacatgttcgaaagtaagagaatgaagctccgaacacaaataccagcagaaatgctagcaagcattgacctaaagtatttcaatgaatcaacaagaaagaaaattgagaaaataaggcctcttctaatagatggcacagcgtcattgagccctggaatgatgatgggcatgttcaacatgctaagtacggttttaggagtctcaatactgaatcttggacaaaagaaatacaccaaaacaacatactggtgggatgggctccaatcctctgatgattttgctctcatagtgaatgcaccaaatcatgagggaatacaagcaggagtggatagattctacagaacctgcaagttagtcggaatcaacatgagcaagaagaagtcctatataaataggacaggaacgtttgaattcacaagctttttttatcgctatggatttgtggccaattttagcatggagctgcccagttttggagtgtctgggattaatgaatcagctgatatgagcattggagtaacagtgataaagaacaacatgataaacaatgaccttggaccagcaacagcccagatggctcttcaactgttcatcaaggactacagatatacatatcggtgccacagaggagacacacaaattcagacgaggagatcattcgagctaaagaagctgtgggagcaaacccgatcaaaggcaggactattggtttcagatggaggaccaaacttatacaatatccggaatcttcacatccctgaagtctgcttaaagtgggagttaatggatgaggactatcagggaagactttgtaatcccctgaatccatttgtcagccataaagagattgagtctgtaaacaatgctgtggtaatgccagctcatggtccagccaagagcatggaatatgacgctgttgcaactacacactcctggattcccaagaggaaccgctctattctcaacacaagccaaaggggaattcttgaggatgaacagatgtatcagaagtgctgcaacctgttcgagaaatttttccccagtagttcatacaggagaccggttggaatttccagcatggtggaggccatggtgtctagggcccggattgatgccagaattgacttcgagtctggacgaattaagaaagaagagttctccgagatcatgaagatctgttccaccattgaagagctcagacggcaaaaataa

>H1N1_PB1_CY019785_A/Memphis/4/1987

atggatgtcaatccgactttacttttcttaaaagtgccagcacaaaatgctataagcacaactttcccttatactggagaccctccttacagccatgggacagggacagggtacaccatggatacagtcaacaggacacatcagtactcagaaagaggaagatggacaaaaaataccgaaactggagcaccgcaactcaacccgattgatgggccactaccaaaggacaatgaaccaagtggctatgcccaaacagattgtgtattagaagcaatggctttccttgaggaatcccatcctggtatttttgaaaactcgtgtattgaaacaatggaggttgttcagcaaacaagagtggacaaactgacacaaggcagacagacctatgactggactctaaatagaaaccagcctgctgctacagcattggccaacactatagaagtgttcagatcaaacggcctcatagcaaatgaatctgggaggttaatagacttccttaaagatgtaatggagtcaatggacagagaagaagtagagatcacaactcattttcagagaaagagaagagtgagagacaatgtaactaaaaaaatggtgacacaaagaacaataggcaaaaagaaacatagattaaacaaaagaagttatctaattagggcattaaccctgaacacaatgaccaaagatgctgagagggggaaactaaaacgtagagcaattgcaaccccaggaatgcaaataagggggtttgtatactttgttgagacactggcaagaagtatatgtgaaaaacttgaacaatcagggttgccagttggaggaaatgagaagaaagcaaagttagcaaatgttgtaaggaagatgatgaccaattcccaggacactgaaatttctttcaccatcactggagataacacaaaatggaacgaaaatcagaaccctagaatgtttttggccatgatcacatatataaccagaaatcagcctgaatggttcagaaatattctaagtattgctccaataatgttctcaaacaaaatggcgagactaggtaaggggtacatgtttgaaagcaagagtatgaaacttagaactcaaatacctgcagaaatgctagcaaacatcgatttgaagtatttcaatgattcaacaagaaagaaaattgaaaaaatccggccactattaatagatggaactgcatcactgagccctggaatgatgatgggcatgttcaatatgttaagcactgtattgggcgtctccatcctgaatctcggacaaaagagatacaccaagactacttactggtgggatggtcttcaatcgtctgatgattttgctctgattgtgaatgcacccaactatgcaggaattcaagctggagttgacaggttttatcgaacctgtaagctgctcggaattaatatgagcaaaaaaaagtcttacataaacagaacaggtacttttgaattcacgagctttttctatcgttatgggtttgttgccaatttcagcatggagcttcctagttttggggtgtctggggtcaatgagtctgcagacatgagtattggagtcactgtcatcaaaaacaatatgataaacaatgatcttggcccagcaactgctcaaatggcccttcagttattcatcaaagattacaggtacacgtatcgatgccacagaggtgacacacaaatacaaacccggagatcatttgaaataaagaaactgtgggatcaaacccgctccaaagctgggctgctggtctctgatggaggtcctaatttatacaacattagaaatctccacattcctgaagtctgcttgaaatgggaattgatggatgaggattaccagggacgtttatgcaacccattgaacccatttgtcagccataaagagattgaatcagtgaacaatgcagtgatgatgccggcacatggtccagccaaaaatatggagtatgacgctgttgcaacaacacactcctgggtccccaaaaggaatcgatccatcttaaatacgagccaaagaggaatacttgaggatgagcaaatgtatcaaaggtgctgcaatttatttgaaaaattcttccccagcagctcatacagaagaccagttggaatatccagtatggtagaggccatggtttccagagcccgaattgatgcacggattgatttcgaatctggaaggataaagaaagaggagttcactgagatcatgaagacctgttccaccattgaagaactcagacggcaaaaatag

>H1N1_PB1_CY019889_A/Memphis/5/2003

atggatgtcaatccgacattacttttcttaaaagtgccagcacaaaatgctataagcacaacttttccttatactggtgaccctccttacagccatgggacaggaacagggtacaccatggatacagtcaacaggacacatcagtactcagaaagaggaagatggacaaaaaataccgaaactggagcaccgcaactcaacccaattgatgggccactaccagaagacaatgaaccaagtggctatgcccaaacagattgtgtattagaagcaatggctttccttgaagaatcccatcctggtatttttgaaaactcttgtattgaaacaatggaggttgttcagcaaacaagggtggacaaactgacacaaggcagacagacctatgactggactctaaataggaaccagcctgctgccacagcattggcaaacactatagaagtattcagatcaaacggcctcatagcaaatgaatctgggaggctaatagacttccttaaagatgtaatggagtcgatggacagagacgaagtagaggtcacaactcattttcaaagaaagaggagagtgagagacaatgtaactaaaaaaatggtgacccaaagaacaataggcaaaaagaaacataaattagacaaaagaagttacctaattagggcattaaccctgaacacaatgaccaaagatgctgagagggggaaactaaaacgcagagcaattgcaaccccaggaatgcaaataagggggtttgtatactttgttgagacactggcaagaagcatatgtgaaaagcttgaacaatcagggttgccagttggaggaaatgagaagaaagcaaagttagcaaatgttgtaaggaagatgatgaccaactcccaggacactgaaatttctttcaccatcactggagataacacaaaatggaacgaaaatcaaaaccctagaatgttcttggccatgatcacatatataaccaaaaatcagcctgaatggttcagaaatattctaagtattgctccaataatgttttcaaacaagatggcgagactaggtaaggggtacatgtttgaaagcaagagtatgaaactgagaactcaaatacctgcagagatgctagccaacatagatttgaaatatttcaatgattcaactaaaaagaaaattgaaaaaatccgaccattattaatagatggaactgcatcattgagtcctggaatgatgatgggcatgttcaatatgttaagcaccgtcttgggcgtctccattctgaatcttgggcaaaagagatacaccaagactacttactggtgggatggtcttcaatcgtctgatgattttgctttgattgtgaatgcacccaactatgcaggaattcaagctggagttgacaggttttatcgaacctgtaagctgctcggaattaatatgagcaaaaagaagtcttacatcaacagaacaggtacctttgaattcacgagctttttctatcgttatgggtttgttgccaatttcagcatggagcttcctagttttggggtgtctggggtcaatgaatctgcagacatgagtattggagtcactgtcatcaaaaacaatatgataaacaatgaccttggcccagcaactgctcaaatggcccttcagttatttataaaagattacaggtacacttatcgatgccacagaggtgacacacaaatacaaacccggagatcatttgagataaagaaactatgggaccaaacccgctccaaagctgggctgttggtctctgatggaggccccaatttatataacattagaaatctccatattcctgaagtctgcttgaaatgggagttgatggatgaggattaccaggggcgtttatgcaacccattgaacccgtttgtcagccataaagagattgaatcagtgaacaatgcagtgataatgccggcacatggtccagccaaaaatatggagtatgacgctgttgcaacaacacactcctgggtccccaaaagaaatcgatccattttgaacacgagccaaagggggatacttgaagatgagcaaatgtatcagaggtgctgcaatttatttgaaaaattcttcccaagtagctcatacagaagaccagttggaatatccagtatggtagaggctatggtttcaagagcccgaattgatgcacggattgatttcgaatctggaaggataaagaaagaggaattcgctgagatcatgaagacctgttccaccattgaagacctcagacggcaaaaatag

>H1N1_PB1_CY019977_A/Roma/1949

atggatgtcaatccgaccttacttttcttaaaagtgccagcacaaaatgctataagcacaactttcccttatactggagaccctccttacagccatgggacaggaacagggtacaccatggatacagtcaacaggacacatcagtactcagaaaggggaagatggacaaaaaacaccgaaactggagcaccacaactcaacccgattgatgggccactaccagaagacaatgaaccaagtggttatgcccaaacagattgtgtattagaagcaatggctttccttgaggaatcccatcctggtatttttgaaaactcgtgtattgaaacgatggaggttgttcagcaaacacgagtggacaaactgacacaaggccgacagacctatgactggactctaaatagaaaccagcctgctgctacagcattggccaacacaatagaagtgttcagatcaaatggcctcatggcaaatgaatctggaaggctaatagacttccttaaggatgtaatggagtcaatggacaaagaagaagtggagatcacaactcattttcagagaaagagacgagtgagggacaatgtgactaagaaaatggtgacacagagaacaataggcaaaaagaaacagagattaaacaaaaggagttatctaattagggcattgaccctgaacacaatgaccaaagatgctgagagagggaaactaaaacgaagagcaattgcaaccccagggatgcaaataagggggtttgtatactttgttgagacactggcaagaagtatatgtgaaaaacttgaacaatcaggattgccagttggaggaaatgaaaagaaagcaaagttggcaaatgttgtaaggaagatgatgaccaattctcaggacactgaaatttctttcaccatcactggagataacaccaaatggaacgaaaatcagaaccctagaatgtttttggccatgatcacatatataaccagaaatcagcccgaatggttcagaaatattctaagtattgctccaataatgttctcaaacaaaatggcgagactaggtaaggggtacatgtttgagagcaagagtatgaaacttagaactcaaatacctgcagaaatgctagcaaacatcgatttgaagtatttcaatgattcaacaagaaagaaaattgaaaaaatccggccactcttaatagatgggactgcatcactgagccctggaatgatgatgggcatgttcaatatgttaagcactgtattgggcgtctccatcctgaatcttggacaaaagagatacaccaagactacttactggtgggatggtcttcaatcatctgatgattttgctctgattgtgaatgcacccaaccatgcaggaattcaagctggagttgacaggttttatcgaacctgtaagctgctcggaatcaatatgagcaagaaaaagtcttacataaacagaacaggcacatttgaattcacgagctttttctatcgttatgggtttgttgccaatttcagcatggagcttcccagttttggggtgtctgggatcaacgagtctgcagacatgagtattggagtcactgtcatcaaaaacaatatgataaacaatgatcttggtccagcaaccgctcaaatggcccttcagttattcatcaaagattacaggtacacgtatcgatgccacagaggtgacacacaaatacaaacccgaagatcatttgaaataaagaaactgtgggagcaaacccgttccaaagctgggctgctggtctccgatggaggtcctaatttatacaacattagaaatctccacattcctgaagtctgcttgaaatgggaattgatggatgaggattaccaggggcgtttatgcaacccattgaacccatttgtcagtcataaagagattgaatcagtgaacaatgcagtgatgatgccggcacatggtccagccaaaaacatggagtatgatgctgttgcaacaacacactcctgggtccccaaaagaaatcgatccatcttgaatacgagccaaagaggaatacttgaagatgaacaaatgtatcaaaggtgctgcaatttatttgaaaaattcttccccagcagttcatacagaaggccagtcggaatatccagtatggtggaggctatggtttccagagcccgaattgatgcacggattgatttcgaatctggaaggataaagaaagaggagttcactgagatcatgaagatctgttccaccattgaagaactcagacggcaaaaatag

>H1N1_PB1_EF467819_A/Puerto Rico/8/1934

atggatgtcaatccgaccttacttttcttaaaagtgccagcacaaaatgctataagcacaactttcccttatactggagaccctccttacagccatgggacaggaacaggatacaccatggatactgtcaacaggacacatcagtactcagaaaagggaagatggacaacaaacaccgaaactggagcaccgcaactcaacccgattgatgggccactgccagaagacaatgaaccaagtggttatgcccaaacagattgtgtattggaggcgatggctttccttgaggaatcccatcctggtatttttgaaaactcgtgtattgaaacgatggaggttgttcagcaaacacgagtagacaagctgacacaaggccgacagacctatgactggactctaaatagaaaccaacctgctgcaacagcattggccaacacaatagaagtgttcagatcaaatggcctcacggccaatgagtctggaaggctcatagacttccttaaggatgtaatggagtcaatgaacaaagaagaaatggggatcacaactcattttcagagaaagagacgggtgagagacaatatgactaagaaaatgataacacagagaacaatgggtaaaaagaagcagagattgaacaaaaggagttatctaattagagcattgaccctgaacacaatgaccaaagatgctgagagagggaagctaaaacggagagcaattgcaaccccagggatgcaaataagggggtttgtatactttgttgagacactggcaaggagtatatgtgagaaacttgaacaatcagggttgccagttggaggcaatgagaagaaagcaaagttggcaaatgttgtaaggaagatgatgaccaattctcaggacaccgaactttctttcaccatcactggagataacaccaaatggaacgaaaatcagaatcctcggatgtttttggccatgatcacatatatgaccagaaatcagcccgaatggttcagaaatgttctaagtattgctccaataatgttctcaaacaaaatggcgagactgggaaaagggtatatgtttgagagcaagagtatgaaacttagaactcaaatacctgcagaaatgctagcaagcatcgatttgaaatatttcaatgattcaacaagaaagaagattgaaaaaatccgatcgctcttaatagaggggactgcatcattgagccctggaatgatgatgggcatgttcaatatgttaagcactgtattaggcgtctccatcctgaatcttggacaaaagagatacaccaagactacttactggtgggatggtcttcaatcctctgacgattttgctctgattgtgaatgcacccaatcatgaagggattcaagccggagtcgacaggttttatcgaacctgtaagctacttggaatcaatatgagcaagaaaaagtcttacataaacagaacaggtacatttgaattcacaagttttttctatcgttatgggtttgttgccaatttcagcatggagcttcccagttttggggtgtctgggatcaacgagtcagcggacatgagtattggagttactgtcatcaaaaacaatatgataaacaatgatcttggtccagcaacagctcaaatggcccttcagttgttcatcaaagattacaggtacacgtaccgatgccatagaggtgacacacaaatacaaacccgaagatcatttgaaataaagaaactgtgggagcaaacccgttccaaagctggactgctggtctccgacggaggcccaaatttatacaacattagaaatctccacattcctgaagtctgcctaaaatgggaattgatggatgaggattaccaggggcgtttatgcaacccactgaacccatttgtcagccataaagaaattgaatcaatgaacaatgcagtgatgatgccagcacatggtccagccaaaaacatggagtatgatgctgttgcaacaacacactcctggatccccaaaagaaatcgatccatcttgaatacaagtcaaagaggagtacttgaggatgaacaaatgtaccaaaggtgctgcaatttatttgaaaaattcttccccagcagttcatacagaagaccagtcgggatatccagtatggtggaggctatggtttccagagcccgaattgatgcacggattgatttcgaatctggaaggataaagaaagaagagttcactgagatcatgaagatctgttccaccattgaagagctcagacggcaaaaatag

>H1N1_PB1_CY020267_A/Memphis/1/2001

atggatgtcaatccgacattacttttcttaaaagtgccagcacaaaatgctataagcacaacttttccttatactggtgaccctccttacagccatgggacaggaacagggtacaccatggacacagtcaacaggacacatcagtactcagaaagaggaagatggacaaaaaataccgaaactggagcaccgcaactcaacccaattgatgggccactaccaaaagacaatgaaccaagtggctatgcccaaacagattgtgtattagaagcaatggctttccttgaggaatcccatcctggtatttttgaaaactcttgtattgaaacaatggaggttgttcagcaaacaagggtggacaaactgacacaaggcagacagacctatgactggacgctaaataggaaccagcctgctgccacagcattggccaacactatagaagtgttcagatcaaatggcctcatagcaaatgaatctgggaggctaatagacttccttaaagatgtaatggagtcgatggacagagacgaagtagagatcacaactcattttcagagaaagaggagagtaagagacaatgtaactaaaaaaatggtgacccaaagaacaataggcaagaagaaacataaattagacaaaagaagttacctaattagggcattaaccctgaacacaatgaccaaagatgctgagagggggaaactaaaacgcagagcaattgcaaccccaggaatgcaaataagggggtttgtatactttgttgagacactggcaagaagcatatgtgaaaagcttgaacaatcagggttgccagttggaggaaatgaaaagaaagcaaagttagcaaatgttgtaaggaagatgatgaccaattcccaggacactgaaatttctttcaccatcactggagataacacaaaatggaacgaaaatcaaaaccctagaatgttcttggccatgatcacctatataaccaaaaatcagcctgaatggttcagaaatattttaagtattgctccaataatgttttcaaacaaaatggcgagactaggtaaggggtacatgtttgaaagcaagagtatgaaactgagaactcaaatacctgcagagatgctagccaacatagatttgaaatatttcaatgattcaactaaaaagaaaattgaaaaaatccggccattattaatagatggaactgcatcattgagtcctggaatgatgatgggcatgttcaatatgttaagcaccgtcttgggcgtctccattctgaatcttgggcaaaagagatacaccaagactacttactggtgggatggtcttcaatcgtctgatgattttgctctgattgtgaatgcacccaactatgcaggaattcaagctggagttgacaggttttatcgaacctgtaagctgctcggaattaatatgagcaaaaagaagtcttacataaacagaacaggtacctttgaattcacgagctttttctatcgttatgggtttgttgccaatttcagcatggagcttcctagttttggggtgtctggggtcaatgaatctgcggacatgagtattggagtcactgtcatcaaaaacaatatgataaacaatgaccttggcccagcaactgctcaaatggcccttcagttatttataaaagattacaggtacacgtatcgatgccacagaggtgacacacaaatacaaacccggagatcatttgagataaagaaactatgggaccaaacccgctccaaagctgggctgttggtctctgatggaggccccaatttatataacattagaaatctccatattcctgaagtctgcttgaaatgggagttgatggatgaggattaccaggggcgtttatgcaacccattgaacccgtttgtcagtcataaagagattgaatcagtgaacaatgcagtgatgatgccggcacatggtccagccaaaaatatggagtatgacgctgttgcaacaacacactcctgggtccccaaaaggaatcgatccattttgaatacgagccaaagggggatacttgaggatgagcaaatgtatcagaggtgctgcaatttatttgaaaaattcttcccaagtagctcatacagaagaccagttggaatatccagtatggtagaggctatggtttccagagcccgaattgatgcacggattgatttcgaatctggaaggataaagaaagaggaattcgctgagatcatgaagacctgttccaccattgaagacctcagacggcaaaaataa

>H1N1_PB1_CY020491_A/Tonga/14/1984

atggatgtcaatccgaccttacttttcttaaaagtgccagcacaaaatgctataagcacaactttcccttatactggagaccctccttacagccatgggacaggaacagggtacaccatggatacagtcaacaggacacatcagtactcagaaaggggaagatggacaaaaaacaccgaaactggagcaccgcaactcaacccgattgatgggccattaccaaaagacaatgaaccaagtggttatgcccaaacagattgtgtattagaagcaatggctttccttgaggaatcccatcccggtatttttgaaaactcatgtattgaaacgatggaggttgttcagcaaacacgtgtagacaaactgacacagggcagacagacctatgactggactctaaatagaaaccagcctgctgctacagcattggccaacaccatagaagtgttcagatcaaacggcctcatggcaaatgaatctggaaggctaatagacttccttaaagatgtaatggattcaatggatagagaagaagtggagatcacaactcattttcagagaaagagaagagtgagggacaatgtaactaagaaaatggtgacacaaagaacaataggcaaaaagaaacatagattaaacaaaaggagttatctaattagggcattaaccctgaacacaatgaccaaagatgctgagagggggaaactaaaacgtagagcaattgcaaccccagggatgcaaataagggggtttgtatactttgttgagacactggcaagaagtatatgtgaaaaacttgaacaatcagggttgccagttggaggaaatgaaaagaaagcaaagttagcaaatgttgtaaggaaaatgatgaccaattctcaggacactgaaatttctttcaccatcactggagataacacaaaatggaacgaaaatcagaaccctagaatgtttttggccatgatcacttatataaccagaaatcagcccgaatggttcagaaatattctaagtattgctccaataatgttctcaaacaaaatggcgagactaggtaaggggtacatgtttgaaagcaagagtatgaaacttagaactcaaatacctgcagaaatgctagcaaacatcgatttgagatatttcaatgattcaacgagaaagaaaattgaaaaaatccggccactcttaatagatggaactgcatcactgagccctggaatgatgatgggcatgttcaatatgttaagcactgtattgggcgtctccatcctgaatcttggacaaaagagatacaccaagaccacttactggtgggatggtcttcaatcgtctgatgattttgctctgattgtgaatgcacccaactatgcaggaattcaagctggagttgacaggttttatcgaacctgtaagctgctcggaatcaacatgagcaagaaaaagtcctacataaacagaacaggtacttttgaattcacgagctttttctatcgttatgggtttgttgccaatttcagcatggagctccccagttttggggtgtctgggatcaacgagtctgcagacatgagtattggagtcactgtcatcaaaaacaatatgataaacaatgatcttggtccagcaaccgctcaaatggcccttcagttattcatcaaagattacaggtacacatatcgatgccacagaggtgacacacaaatacaaacccgaagatcatttgaaataaagaaactgtgggatcaaacccgttccaaagctgggctgctggtctctgatggaggtcctaatttatacaacattagaaatctccacattcctgaagtctgcttgaaatgggatttgatggatgaggattaccagggtcgtttatgcaacccattgaacccatttgtcagtcataaagagattgaatcagtgaacaatgcagtgatgatgccggcacatggtccagccaaaattatggagtatgatgctgttgcaacaacacactcctgggtccccaaaaggaatcgatccatcttgaatacgagccaaaggggaatacttgaggatgaacaaatgtatcagaggtgctgcaacttatttgaaaaattcttccccagcagttcatatagaagaccagtcggaatatccagtatggtagaggctatgatttccagagcccgaattgatgcacggattgatttcgaatctggaagaataaagaaagaggagttcactgagatcatgaagacctgttccaccattgaagaactcagacggcaaaaatag

>H1N1_PB1_CY021715_A/AA/Huston/1945

atggatgtcaatccgatcttacttttcttaaaagttccagcacaaaatgctataagcacaactttcccttatactggagaccctccttacagccatgggacaggaacaggatataccatggatactgtcaacaggacacatcagtactcagaaaggggaaaatggacaacaaacaccgaaactggagcaccacaactcaacccgattgatgggccactgccagaagacaatgaaccgagtggttatgcccaaacagattgtgtattggaagcaatggctttccttgaggaatcccaccctggtatttttgaaaactcatgcattgaaacgatggaggttgttcagcaaacacgagtggacaagctgacacaaggccgacagacctatgactggactctaaatagaaaccagcctgccgcaacagcattggccaacacaatagaagtgttcagatcaaatggcctcacggccaatgaatctggaaggctaatagacttccttaaggatgtaatggagtcaatggacaaagaagaaatagagatcacaactcattttcagagaaagagacgagtgagagacaatgtgactaagaagatggtgacacagagaacaataggcaaaaggaagcaaagattgaacaaaaggagttatctaattagagcattgaccctgaacacaatgaccaaagatgctgagagagggaaactaaaacggagagcaattgcaaccccagggatgcaaataagggggtttgtatactttgttgagacactggcaagaagtatatgtgaaaaacttgaacaatcaggattgccagttggaggcaatgagaagaaagcaaagttggcaaatgttgtaaggaagatgatgaccaattctcaggacactgaaatttctttcaccatcactggagataacaccaaatggaacgaaaatcagaaccctaggatgtttttggccatgatcacatatattaccagaactcagcccgaatggttcagaaatgttctaagtattgctccaataatgttctcaaacaaaatggcgagactaggaaaggggtacatgtttgagagcaagagtatgaaacttagaactcaaatacctgcagaaatgctagcaaacatcgatttgaagtatttcaatgattcaacaagaaagaagattgaaaaaatccggccgctcttaatagatgggactgcatcactgagccctggaatgatgatgggcatgttcaatatgttaagcactgtattgggcgtctccattctgaatcttggacaaaagagatacaccaagaccacttactggtgggatggtcttcaatcttctgatgattttgctctgattgtgaatgcacccaaccatgaaggaattcaagcaggagttgacaggttttatcgaacctgtaagctactcggaatcaatatgagcaagaaaaagtcttacataaacagaacaggtacatttgaattcacgagctttttctatcgttatgggtttgttgcaaatttcagcatggagcttcccagttttggggtgtctgggatcaacgagtctgcagacatgagtattggagtcactgtcatcaaaaacaatatgataaacaatgatcttggtccagcaacagctcaaatggcccttcagttgttcatcaaagattacaggtacacatatcgatgccatagaggtgacacacaaatacaaacccgaagatcatttgaaataaagaaactgtgggagcaaacccgttctaaagctggactgctggtctccgacggaggcccaaatttatacaacattagaaatctccacattcctgaagtctgtttaaaatgggaattgatggatgaggattaccaggggcgtttatgcaacccactgaacccatttgtcagtcataaagagattgaatcagtgaacaatgcagtaatgatgccggcacatggtccagccaaaaacatggagtatgatgctgttgcaacaacacactcctggatccccaaaagaaatcggtccatcttgaatacaagccaaagaggaatacttgaagatgaacaaatgtatcaaaggtgctgcaatttatttgaaaaattcttccccagcagttcatacagaagaccagtcggaatatccagtatggtggaggctatggtttccagagcccgaattgatgcacggattgatttcgaatctggaaggataaagaaggaggagttcactgagatcatgaagatctgttccaccattgaagaactcagacggcaaaaatag

>H1N1_PB1_CY021963_A/New Jersey/1976

atggacgtcaatccgactttgcttttcttgaaagtgccagcacaaaatgctataagcacaacgtttccttacactggagaccccccttacagccatgggacaggaacaggatacaccatggatactgtgaacagaacacatcagtactcagaaagaggaaaatggacaataaacaccgaaactggggcaccacaactaaatccaattgatggaccgctgccagaggacaatgaaccaactggttatgctcaaacagactgcgtattggaagcaatggcgttcctcgagaaatcccatcccggcatttttgagaactcatgtcttgaaacgatggaagttgttcagcaaacacgggtggacaaactaacgcaaggtcggcaaactttcgattggactctgaataggaaccagcctgctgcaacagcattggccaacacaatagaagtgttcagattgaacggcctcacggccagtgaatctggaagactcatagacttcctaaaggatgttatggaatcaatggacaaagaagaaatggaaatcgtaactcatttccagagaaaaaggagagtgagggacaatatgactaagaaaatggtgacgcaaagaacaataggtaaaaagaaacagaagttaaataaaagaagctatctaattagagcattgaccttgaacacaatgacaaaagatgctgaaagaggaaaattgaaaagaagagcaattgccaccccagggatgcaaatacggggattcgtatatttcgttgagacactagcgagaagtatttgtgaaaaacttgaacaatcaggactgccagttggaggcaatgaaaagaaagccaaattggcaaatgttgtacgaaaaatgatgaccaattctcaggacactgaactctcctttactatcactggagataataccaaatggaatgaaaatcagaatcctcgaatgtttttagctatgattacatacataaccagaaatcagcccgaatggttcagaaatgttctaagtgttgctccaataatgttctcgaacaagatggctagattgggtaaaggatacatgtttgagagtaaaaacatgaagcttaggactcagatacctgcagagatgctatcaggcattgacctgaggtattttaatgattcaactaggaaaaaaattgaaaaaatccgaccgctcttaatagagggagctgcttctttaagccctggaatgatgatgggtatgtttaacatgttaagcactgtgttgggtgtttccattcttaaccttggacaaaaggagtacacgaagactgcttactggtgggacggtctccaatcgtctgatgattttgctctgattgtgaacgcacccaattatgaagggattcaagcaggagttgacaggttttatcgaacctgtaagctactcggaattaatatgagcaaaaagaaatcttacataaatagaacaggcacttttgaattcacaagctttttctatcgttatggatttgttgccaatttcagcatggagcttcctagttttggagtgtctggaattaatgagtctgcagacatgagtattggagttactgtcatcaaaaacaacatgataaacaatgatcttggaccagcaacagcacaaatggctctccagttgttcatcaaagattaccgatacacgtatcgctgccacaggggtgatactcaaatacaaactcgaaggtcatttgaaataaagaaattgtgggatcaaacccgttctaaggctggattgttggtttccgatggaggccccaatttatataatatcaggaatctccacattcctgaagtctgcttgaagtgggaattaatggataaagattaccaggggcgattgtgcaatccgctaaacccatttgtcagccataaagagattgaatcagtgaataatgcagtagttatgccatcacatggtccagccaaagccatggaatacgacgctgttgccacaacgcactcctgggttcctaaaagaaatcgatccatcttgaacacaagccagagaggaatacttgaagacgagcaaatgtaccaaaaatgctgtaacctatttgaaaagttcttccctagcagctcatataggagaccagttgggatttccagtatggtggaggccatggtttccagggcccgaatcgatgcacgaattgattttgaatctggaaggataaagaaagaggatttcgctgagatcatgaagatctgttccaccattgaagatctcagacggcaaaagtag

>H1N1_PB1_CY021979_A/Memphis/1/1987

atggatgtcaatccgactttacttttcttaaaagtgccagcacaaaatgctataagcacaactttcccttatactggagaccctccttacagccatgggacagggacagggtacaccatggatacagtcaacagaacacatcagtactcagaaagaggaagatggacaaaaaataccgaaactggagcaccgcaactcaacccgattgatgggccactaccaaaagacaatgaaccaagtggctatgcccaaacagattgtgtattagaagcaatggctttccttgaggaatcccatcctggtatttttgaaaactcgtgtattgaaacaatggaggttgttcagcaaacaagagtggacaaactgacacaaggcagacagacctatgactggactctaaatagaaaccagcctgctgctacagcattggccaacactatagaagtgttcagatcaaacggcctcatagcaaatgaatctgggaggttaatagacttccttaaagatgtaatggagtcaatggacagagaagaagtagagatcacaactcattttcagagaaagagaagagtgagagacaatgtaactaaaaaaatggtgacacaaagaacaataggcaaaaagaaacatagattaaacaaaagaagttatctaattagggcattaaccctgaacacaatgaccaaagatgctgagagggggaaactaaaacgtagagcaattgcaaccccaggaatgcaaataagggggtttgtatactttgttgagacactggcaagaagtatatgtgaaaaacttgaacaatcagggttgccagttggaggaaatgagaagaaagcaaagttagcaaatgttgtaaggaagatgatgaccaattcccaggacactgaaatttctttcacaatcactggagataacacaaaatggaacgaaaatcagaaccctagaatgtttttggccatgatcacatatataaccagaaatcaacctgaatggttcagaaatattctaagtattgctccaataatgttctcaaacaaaatggcgagactaggtaaggggtacatgtttgaaagcaagagtatgaaacttagaactcaaatacctgcagaaatgctagcaaacatcgatttgaagtatttcaatgattcaacaagaaagaaaattgaaaaaatccggccactattaatagatggaactgcatcactgagccctggaatgatgatgggcatgttcaatatgttaagcactgtattgggcgtctccatcctgaatctcggacaaaagagatacaccaagactacttactggtgggatggtcttcaatcgtctgatgattttgctctgattgtgaatgcacccaactatgcaggaattcaagctggagttgacaggttttatcgaacctgtaagctgctcggaattaatatgagcaaaaaaaagtcttacataaacagaacaggtacttttgaattcacgagctttttctatcgttatgggtttgttgccaatttcagcatggagcttcctagttttggggtgtctggggtcaatgagtctgcagacatgagtattggagtcactgtcatcaaaaacaatatgataaacaatgatcttggcccagcaactgctcaaatggcccttcagttattcatcaaagattacaggtacacgtatcgatgccacagaggtgacacacaaatacaaacccggagatcatttgaaataaagaaactgtgggatcaaacccgctccaaagctgggctgctggtctctgatggaggtcctaatttatacaacattagaaatctccacattcctgaagtctgcttgaaatgggaattgatggatgaggattaccagggacgtttatgcaacccattgaacccatttgtcagccataaagagattgaatcagtgaacaatgcagtgatgatgccggcacatggtccagccaaaaatatggagtatgacgctgttgcaacaacacactcctgggtccccaaaaggaatcgatccatcttaaatacgagccaaagaggaatacttgaggatgagcaaatgtatcaaaggtgctgcaatttatttgaaaaattcttccccagcagctcatacagaagaccagttggaatatccagtatggtagaggccatggtttccagagcccgaattgatgcacggattgatttcgaatctggaaggataaagaaagaggagttcactgagatcatgaagacctgttccaccattgaagaactcagacggcaaaaatag

>H1N1_PB1_CY022547_A/Auckland/606/2001

atggatgtcaatccgaccttacttttcttaaaagtgccagcacaaaatgctataagcacaactttcccttatactggtgatcctccttacagccatgggacaggaacagggtataccatggatacagtcaacaggacacatcagtactcagaaagaggaagatggacaaaaaataccgaaactggagcaccgcaactcaacccaattgatggaccactaccaaaagacaatgaaccaagtggctatgcccaaacagattgtgtattagaagcaatggctttccttgaggaatcccatcctggtatttttgaaaactcttgtattgaaacaatggaggttgttcagcaaacaagggtggacaaactgacacaaggcagacagacctatgactggactctaaatagaaaccagcctgctgccacagcattggccaacactatagaagtgttcagatcaaacggcctcatagcaaatgaatctgggaggctaatagacttccttaaagatgtaatggagtcgatggacagagacgaagtagagatcacaacacattttcagagaaagaggagggtgagagacaatgtaactaaaaaaatggtgacccaaagaacaataggcaaaaagaaacataaattagacaaaagaagttacctaattagggcattaaccctgaacacaatgaccaaagatgctgagagggggaaactaaaacgtagagcaattgcaaccccaggaatgcaaataagggggtttgtatactttgttgagacactggcaagaagcatatgtgaaaagcttgaacaatcagggttgccagttgggggaaatgaaaagaaagcaaagttagcaaatgttgtaaggaagatgatgaccaactcccaggacactgaaatttctttcaccatcactggagataacacaaaatggaacgaaaatcaaaaccctagaatgtttttggccatgatcacatatataaccaaaaatcagcctgaatggttcagaaatattctaagtattgctccaataatgttttcaaacaaaatggcgagactaggtaaggggtacatgtttgaaagcaagagtatgaaactgagaactcaaatacctgcagagatgctagccaacatagatttgaaatatttcaatgattcaactaaaaagaaaattgaaaaaatccggccattattaatagatggaactgcatcattgagtcctggaatgatgatgggcatgttcaatatgttaagcactgtcttgggcgtctccattctgaatcttgggcaaaagagatacaccaagactacttactggtgggatggtcttcaatcgtctgatgattttgctctgattgtgaatgcatccaactatgcaggaattcaagctggagttgacaggttttatcgaacctgtaagctgctcggaattaatatgagcaaaaagaagtcttacataaacagaacaggtacctttgaattcacgagctttttctatcgttatgggtttgttgccaatttcagcatggagcttcctagttttggggtgtctggggtcaatgagtctgcagacatgagtattggagtcactgtcatcaaaaacaatatgataaacaatgaccttggcccagcaactgctcaaatggcccttcagttatttatcaaagattacaggtacacgtatcgatgccacagaggtgacacacaaatacaaacccggagatcatttgagataaagaaactatgggaccaaacccgctccaaagctgggctgttggtctctgatggaggccccaatttatataacattagaaatctccatattcctgaagtctgcttgaaatgggagttgatggatgaggattaccaggggcgtttatgcaacccattgaacccatttgtcagtcataaagagattgagtcagtgaacaatgcagtgatgatgccggcacatggtccagccaaaaatatggagtatgacgctgttgcaacaacacactcctgggttcccaaaaggaatcgatccattttgaatacgagccaaagggggatacttgaggatgagcaaatgtatcagaggtgctgcaatttatttgaaaaattcttcccaagtagttcatacagaagaccagttggaatatccagtatggtagaggctatggtttccagagcccgaattgatgcaagaattgatttcgaatctggaaggataaagaaagaggaattcgctgagatcatgaagacctgttccaccattgaagacctcagacggcaaaaatag

>H1N1_PB1_CY025825_A/Vermont/UR06-0574/2007

atggatgtcaatccgacattacttttcttaaaagtgccagcacaaaatgctataagcacaacttttccttatactggtgaccctccttacagccatgggacaggaacagggtacaccatggatacagtcaacaggacacaccagtactcagaaagaggaagatggacaaaaaatacagaaactggagcaccgcaactcaacccaattgatgggccactaccagaagacaatgaaccaagtggctatgcccaaacagattgtgtattagaagcaatggctttccttgaagaatcccatcctggtatctttgaaaactcttgtattgaaacaatggaggttgttcagcaaacaagggtggacaaactgacacaaggcagacagacctatgactggactctaaataggaaccagcctgctgcaacagcattggcaaacactatagaagtattcagatcaaacggcctcatagcaaatgaatctggaaggctaatagacttccttaaagatgtaatggagtcgatggacagagacgaagtagaggtcacaacccattttcaaagaaagagaagagtgagagacaatgtgactaaaaaaatggtgactcaaagaacaataggcaaaaagaaacataaattagacaaaagaagttacctaattagggcattaaccctgaacacaatgaccaaagatgctgagagggggaaactaaaacgcagagcaattgcaaccccaggaatgcaaataagagggtttgtatactttgttgagacactggcaagaagcatatgtgaaaagcttgaacaatcaggattgccagttggaggaaatgagaagaaagcaaagttagcaaatgttgtaaggaagatgatgaccaactcccaggacactgaaatttctttcaccatcactggagataacacaaaatggaacgaaaatcaaaaccctagaatgttcttggccatgatcacatatataaccaaaaatcagcctgaatggttcagaaatattctaagtattgctccaataatgttttcaaacaagatggcgagactaggtaaggggtacatgtttgaaagcaagagtatgaaactgagaactcaaatacctgcagagatgctagccaacatagatttgaaatatttcaatgattcaactaaaaagaaaattgaaaaaatccgaccattattaatagatggaactgcatcattgagtcctggaatgatgatgggcatgttcaatatgttgagcaccgtcttgggcgtctccattctgaatcttgggcaaaagagatacaccaagactacttactggtgggatggtcttcaatcgtctgatgattttgctttgattgtgaatgcacccaactatgcaggaattcaagctggagttgacaggttttatcgaacctgtaagctgctcggaattaatatgagcaaaaagaagtcttacataaacagaacaggtacctttgaattcacgagctttttctatcgttatgggtttgttgccaatttcagcatggagcttcctagttttggggtgtctggggtcaatgaatctgcagacatgagtattggagtcactgtcatcaaaaacaatatgataaacaatgaccttggcccagcaactgctcaaatggcccttcagttatttataaaagattacaggtacacttatcgatgccacagaggtgacacacaaatacaaacccggagatcatttgagataaagaaactatgggaccaaacccgctccaaagctggactgttggtctctgatggaggccccaatttatataacattagaaatctccatattcctgaagtttgcttgaaatgggagttgatggatgaggattaccaggggcgtttatgcaacccattaaacccgtttgtcagccataaagagattgaatcagtgaacaatgcagtgataatgccggcacatggcccagccaaaaatatggagtatgacgctgttgcaacaacacactcctgggtccccaaaagaaatcgatccattttgaacacgagccaaagggggatacttgaagatgagcaaatgtatcagaggtgctgcaatttatttgaaaaattcttcccaagtagctcatacagaagaccagttggaatatccagtatggtagaggctatggtttcaagagcccgaattgatgcacggattgatttcgaatctggaaggataaagagggaggaatttgctgagatcatgaagatctgttccaccattgaagacctcagacggcaaaaatag

>H1N1_PB1_CY025953_A/Kentucky/UR06-0257/2007

atggatgtcaatccgacattacttttcttaaaagtgccagcacaaaatgctataagcacaacttttccttatactggtgaccctccttacagccatgggacaggaacagggtacaccatggatacagtcaacaggacacaccagtactcagaaagaggaagatggacaaaaaataccgaaactggagcaccgcaactcaacccaattgatgggccactaccagaagacaatgaaccaagtggctatgcccaaacagattgtgtattagaagcaatggctttccttgaagaatcccatcctggtatctttgaaaactcttgtattgaaacaatggaggttgttcagcaaacaagggtggacaaactgacacaaggcagacagacctatgactggactctaaataggaaccagcctgctgcaacagcattggcaaacactatagaagtattcagatcaaacggcctcatagcaaatgaatctggaaggctaatagacttccttaaagatgtaatggagtcgatggacagagacgaagtagaggtcacaactcattttcaaagaaagaggagagtgagagacaatgtaactaaaaaaatggtgactcaaagaacaataggcaaaaagaaacataaattagacaaaagaagttacctaattagggcattaaccctgaacacaatgaccaaagatgctgagagggggaaactaaaacgcagagcaattgcaaccccaggaatgcaaataagagggtttgtatactttgttgagacactggcaagaagcatatgtgaaaagcttgaacaatcaggattgccagttggaggaaatgagaagaaagcaaagttagcaaatgtagtaaggaagatgatgaccaactcccaggacactgaaatttctttcaccatcactggagataacacaaaatggaacgaaaatcaaaaccctagaatgttcttggccatgatcacatatataaccaaaaatcagcctgaatggttcagaaatattctaagtattgctccaataatgttttcaaacaagatggcgagactaggtaaggggtacatgtttgaaagcaagagtatgaaactgagaactcaaatacctgcagagatgctagccaacatagatttgaaatatttcaatgattcaactaaaaagaaaattgaaaaaatccgaccattattaatagatggaactgcatcattgagtcctggaatgatgatgggcatgttcaatatgttgagcaccgtcttgggcgtctccattctgaatcttgggcaaaagagatacaccaagactacttactggtgggatggtcttcaatcgtctgatgattttgctttgattgtaaatgcacccaactatgcaggaattcaagctggagttgacaggttttatcgaacctgtaagctgctcggaattaatatgagcaaaaagaagtcttacataaacagaacaggtacctttgaattcacgagctttttctatcgttatgggtttgttgccaatttcagcatggagcttcctagttttggggtgtctggggtcaatgaatctgcagacatgagtattggagtcactgtcatcaaaaacaatatgataaacaatgaccttggcccagcaactgctcaaatggcccttcagttatttataaaagattacaggtacacttatcgatgccacagaggtgacacacaaatacaaacccggagatcatttgagataaagaaactatgggaccaaacccgctccaaagctgggctgttggtctttgatggaggccccaatttatataacattagaaatctccatattcctgaagtttgcttgaaatgggagttgatggatgaggattatcaggggcgtttatgcaacccattaaacccgtttgtcagccataaagagattgaatcagtgaacaatgcagtgataatgccggcacatggcccagccaaaaatatggagtatgacgctgttgcaacaacacactcctgggtccccaaaagaaatcgatccattttgaacacgagccaaagggggatacttgaagatgagcaaatgtatcagaggtgctgcaatttatttgagaaattcttcccaagtagctcatacagaagaccagttggaatatccagtatggtagaggctatggtttcaagagcccgaattgatgcacggattgatttcgaatctggaaggataaagagggaggaatttgctgagatcatgaagatctgttccaccattgaagacctcagacggcaaaaatag

>H1N1_PB1_CY026209_A/Kentucky/UR06-0187/2007

atggatgtcaatccgacattacttttcttaaaagtgccagcacaaaatgctataagcacaacttttccttatactggtgaccctccttacagccatgggacaggaacagggtacaccatggatacagtcaacaggacacaccagtactcagaaagaggaagatggacaaaaaataccgaaactggagcaccgcaactcaacccaattgatgggccactaccagaagacaatgaaccaagtggctatgcccaaacagattgtgtattagaagcaatggctttccttgaagaatcccatcctggtatctttgaaaactcttgtattgaaacaatggaggttgttcagcaaacaagggtggacaaactgacacaaggcagacagacctatgactggactctaaataggaaccagcctgctgcaacagcattggcaaacactatagaagtattcagatcaaacggcctcatagcaaatgaatctggaaggctaatagacttccttaaagatgtaatggagtcgatggacagagacgaagtagaggtcacaactcattttcaaagaaagaggagagtaagagacaatgtaactaaaaaaatggtgactcaaagaacaataggcaaaaagaaacataaattagacaaaagaagttacctaattagggcattaaccctgaacacaatgaccaaagatgctgagagggggaaactaaaacgcagagcaattgcaaccccaggaatgcaaataagagggtttgtatactttgttgagacactggcaagaagcatatgtgaaaagcttgaacaatcaggattgccagttggaggaaatgagaagaaagcaaagttagcaaatgttgtaaggaagatgatgaccaactcccaggacactgaaatttctttcaccatcactggagataacacaaaatggaacgaaaatcaaaaccctagaatgttcttggccatgatcacatatataaccaaaaatcagcctgaatggttcagaaatattctaagtattgctccaataatgttttcaaacaagatggcgagactaggtaaggggtacatgtttgaaagcaagagtatgaaactgagaactcaaatacctgcagagatgctagccaacatagatttgaaatatttcaatgattcaactaaaaagaaaattgaaaaaatccgaccattattaatagatggaactgcatcattgagtcctggaatgatgatgggcatgttcaatatgttgagcaccgtcttgggcgtctccattctgaatcttgggcaaaagagatacaccaagactacttactggtgggatggtcttcaatcgtctgatgattttgctttgattgtgaatgcacccaactatgcaggaattcaagctggagttgacaggttttatcgaacctgtaagctgctcggaattaatatgagcaaaaagaagtcttacataaacagaacaggtacctttgaattcacgagctttttctatcgttatgggtttgttgccaatttcagcatggagcttcctagttttggggtgtctggggtcaatgaatctgcagacatgagtattggagtcactgtcatcaagaacaatatgataaacaatgaccttggcccagcaactgctcaaatggcccttcagttatttataaaagattacaggtacacttatcgatgccacagaggtgatacacaaatacaaacccggagatcatttgagataaagaaactatgggaccaaacccgctccaaagctgggctgttggtctctgatggaggccccaatttatataacattagaaatctccatattcctgaagtttgcttgaaatgggagttgatggatgaggattaccaggggcgtttatgcaacccattaaacccgtttgtcagccataaagagattgaatcagtgaacaatgcagtgataatgccggcacatggcccagccaaaaatatggagtatgacgctgttgcaacaacacactcctgggtccccaaaagaaatcgatccattttgaacacgagccaaagggggatacttgaagatgagcaaatgtatcagaggtgctgcaatttatttgaaaaattcttcccaagtagctcatacagaagaccagttggaatatccagtatggtagaggctatggtttcaagagcccgaattgatgcacggattgatttcgaatctggaaggataaagagggaggaatttgctgagatcatgaagatctgttccaccattgaagacctcagacggcaaaaatag

>H1N1_PB1_CY026345_A/Texas/UR06-0542/2007

atggatgtcaatccgacattacttttcttaaaagtgccagcacaaaatgctataagcacaacttttccttatactggtgaccctccttacagccatggaacaggaacagggtacaccatggatacagtcaacaggacacatcagtactcagaaagaggaagatggacaaaaaataccgaaactggagcaccgcaactcaacccaattgatgggccactaccagaagacaatgaaccaagtggctatgcccaaacagattgtgtattagaagcaatggctttccttgaagaatcccatcctggtatttttgaaaactcttgtattgaaacaatggaggttgttcagcaaacaagggtggacaaactgacacaaggcagacaaacctatgactggactctaaataggaaccagcctgccgccacagcattggcaaacactatagaagtattcagatcaaatggcctcatagcaaatgaatctgggaggctaatagacttccttaaagatgtaatggagtcgatggacagagaagaagtagaggtcacaactcattttcaaagaaagaggagagtgagagacaatgtaactaaaaaaatggtgacccaaagaacaatagggaaaaagaaacataaattagacaaaagaagttacctaattagggcattaaccctgaacacaatgaccaaagatgctgagagggggaaactaaaacgcagagcaattgcaaccccaggaatgcaaataagggggtttgtatactttgttgagacactggcaagaagcatatgtgaaaagcttgaacaatcagggttgccagttggaggaaatgagaagaaagcaaagttagcaaatgttgtaaggaagatgatgaccaactcccaggacactgaaatttctttcaccatcactggagataacacaaaatggaacgagaatcaaaaccctagaatgttcttggccatgatcacatatataaccaaaaatcagcctgaatggttcagaaatattctaagtattgctccaataatgttttcaaacaaaatggcgagactaggtagggggtatatgtttgaaagcaagagtatgaaactgagaacccaaatacctgcagagatgctagccaacatagatttgaaatacttcaatgattcaactaaaaagaaaattgaaaaaattcgaccattattaatagatggaactgcatcattgagtcctggaatgatgatgggcatgttcaatatgttaagcaccgtcttgggcgtctccattctgaatcttgggcaaaagagatacaccaagactacttactggtgggatggtcttcaatcgtctgatgattttgctttgattgtgaatgcacccaattatgcaggaattcaagctggagttaacaggttttatcgaacctgtaagctgctcggaattaatatgagcaaaaagaagtcttacataaacagaacaggtacctttgaattcacgagctttttctatcgttatgggtttgttgccaatttcagcatggagcttcctagttttggggtgtctggggtcaatgaatctgcagacatgagtattggagtcactgtcatcaaaaacaatatgataaacaatgaccttggcccagcaactgctcaaatggcccttcagttatttataaaagattataggtacacttatcgatgccacagaggtgacacacaaatacaaacccgaagatcatttgaaataaagaaactatgggaccaaacccgctccaaagctgggctgttggtctctgatggaggccccaatctatataacattaggaatctacatattcctgaagtctgcttgaaatgggagttgatggatgaggattaccaggggcgtttatgcaacccattgaacccgtttgtcagccataaagagattgaatcagtgaacaatgcagtgataatgccggcacatggtccagcaaaaaatatggagtatgacgctgttgcaacaacacactcctgggtccccaaaagaaatcgatccattttgaacacgagccaaagagggatacttgaagatgagcaaatgtatcagaggtgctgcaatttatttgaaaaattcttcccaagtagctcatacagaagaccagttggaatatccagtatggtagaggctatggtttcaagagcccgaattgatgcacggattgatttcgaatctggaaggataaagaaagaggaattcgctgagatcatgaagacctgttccaccattgaagacctcagacggcaaaaatag

>H1N1_PB1_CY026361_A/California/UR06-0232/2007

atggatgtcaatccgacattacttttcttaaaagtgccagcacaaaatgctataagcacaacttttccttatactggtgaccctccttacagccatgggacaggaacagggtacaccatggatacagtcaacaggacacaccagtactcagaaagaggaagatggacaaaaaataccgaaactggagcaccgcaactcaacccaattgatgggccactaccagaagacaatgaaccgagtggctatgcccaaacagattgtgtattagaagcaatggctttccttgaagaatcccatcctggtatctttgaaaactcttgtattgagacaatggaggttgttcagcaaacaagggtggacaaactgacacaaggcagacagacctatgactggactctaaataggaaccagcctgctgccacagcattggcaaacactatagaagtattcagatcaaacggcctcatagcaaatgaatctgggaggctaatagacttccttaaagatgtaatggagtcgatggacatagacgaagtagaggtcacaactcattttcaaagaaagaggagagtgagagacaatgtaactaaaaaaatggtgacccaaagaacaataggcaaaaagaaacataaattagacaaaagaagttacctaattagggcattaaccctgaacacaatgaccaaagatgctgagagagggaaactaaaacgcagagcaattgcaaccccaggaatgcaaataagggggtttgtatactttgttgagacactggcaagaagcatatgtgaaaagcttgaacaatcaggattgccagttggaggaaatgagaagaaagcaaagttagcaaatgttgtaaggaagatgatgaccaactcccaggacactgaaatttctttcaccatcactggagataacacaaaatggaacgaaaatcaaaaccctagaatgttcttggccatgatcacatatataaccaaaaatcagcctgaatggttcagaaatattctaagtattgccccaataatgttttcaaacaagatggcgagactaggtaaggggtacatgtttgaaagcaagagtatgaaactgagaactcaaatacctgcagagatgctagccaacatagatttgaaatatttcaatgattcaactaaaaagaaaattgaaaaaatccgaccattattaatagatggaactgcatcattgagtcctggaatgatgatgggcatgttcaatatgttgagcaccgtcttgggcgtctccattctgaatcttgggcaaaagagatacaccaagactacttactggtgggatggtcttcaatcgtctgatgatttcgctttgattgtgaatgcacccaactatgcaggaattcaagctggagttgacaggttttatcgaacctgtaagctgctcggaattaatatgagcaaaaagaagtcttacataaacagaacaggtacctttgaattcacgagctttttctatcgttatgggtttgttgccaatttcagcatggagcttcctagttttggggtgtctggggtcaatgaatctgcagacatgagtattggagtcactgtcatcaaaaacaatatgataaacaatgaccttggcccagcaactgctcaaatggcccttcagttatttataaaagattacaggtacacttatcgatgccacagaggtgacacacaaatacaaacccggagatcatttgagataaagaaactatgggaccaaacccgctctaaagctgggctgttggtctctgatggaggccccaatttatataacattagaaatctccatattcctgaagtttgcttgaaatgggagttgatggatgaggattaccaggggcgtttatgcaacccattaaacccgtttgtcagccataaagagattgaatcagtaaacaatgcagtgataatgccggcacatggtccagccaaaaatatggagtatgacgctgttgcaacaacacactcctgggtccccaaaagaaatcgatccattttgaacacgagccaacgggggatacttgaagatgagcaaatgtatcagaggtgctgcaatttatttgaaaaattcttcccaagtagctcatacagaagaccagttggaatatccagtatggtagaggctatggtttcaagagcccgaattgatgcacggattgatttcgaatctggaaggataaagaaagaggaatttgctgagatcatgaagatctgttccaccattgaagacctcagacggcaaaaatag

>H1N1_PB1_CY026369_A/Texas/UR06-0175/2007

atggatgtcaatccgacattacttttcttaaaagtgccagcacaaaatgctataagcacaacttttccttatactggtgaccctccttacagccatgggacaggaacagggtacaccatggatacagtcaacaggacacaccagtactcagaaagaggaagatggacaaaaaataccgaaactggagcaccgcaactcaacccaattgatgggccactaccagaagacaatgaaccaagtggctatgcccaaacagattgtgtattagaagcaatggctttccttgaagaatcccatcctggtatctttgaaaactcttgtattgaaacaatggaggttgttcagcaaacaagggtggacaaactgacacaaggcagacagacctatgactggactctaaataggaaccagcctgctgcaacagcattggcaaacactatagaagtattcagatcgaacggcctcatagcaaatgaatctggaaggctaatagacttccttaaagatgtaatggagtcgatggacagagacgaagtagaggtcacaactcattttcaaagaaagaggagagtaagagacaatgtaactaaaaaaatggtgactcaaagaacaataggcaaaaagaaacataaattagacaaaagaagttacctaattagggcattaaccctgaacacaatgaccaaagatgctgagagggggaaactaaaacgcagagcaattgcaaccccaggaatgcaaataagagggtttgtatactttgttgagacactggcaagaagcatatgtgaaaagcttgaacaatcaggattgccagttggaggaaatgagaagaaagcaaagttagcaaatgttgtaaggaagatgatgaccaactcccaggacactgaaatttctttcaccatcactggagataacacaaaatggaacgaaaatcaaaaccctagaatgttcttggccatgatcacatatataaccaaaaatcagcctgaatggttcagaaatattctaagtattgctccaataatgttttcaaacaagatggcgagactaggtaaggggtacatgtttgaaagcaagagtatgaaactgagaactcaaatacctgcagagatgctagccaacatagatttgaaatatttcaatgattcaactaaaaagaaaattgaaaaaatccgaccattattaatagatggaactgcatcattgagtcctggaatgatgatgggcatgttcaatatgttgagcaccgtcttgggcgtctccattctgaatcttgggcaaaagagatacaccaagactacttactggtgggatggtcttcaatcgtctgatgattttgctttgattgtgaatgcacccaactatgcaggaattcaagctggagttgacaggttttatcgaacctgtaagctgctcggaattaatatgagcaaaaagaagtcttacataaacagaacaggtacctttgaattcacgagctttttctatcgttatgggtttgttgccaatttcagcatggagcttcctagttttggggtgtctggggtcaatgaatctgcagacatgagtattggagtcactgtcatcaaaaacaatatgataaacaatgaccttggcccagcaactgctcaaatggcccttcagttatttataaaagattacaggtacacttatcgatgccacagaggtgacacacaaatacaaacccggagatcatttgagataaagaaactatgggaccaaacccgctccaaagctgggctgttggtctctgatggaggccccaatttatataacattagaaatctccatattcctgaagtttgcttgaaatgggagttgatggatgaggattaccaggggcgtttatgcaacccattaaacccgtttgtcagccataaagagattgaatcagtgaacaatgcagtgataatgccggcacatggcccagccaaaaatatggagtatgacgctgttgcaacaacacactcctgggtccccaaaagaaatcgatccattttgaacacgagccaaagggggatacttgaagatgaacaaatgtatcagaggtgctgcaatttatttgaaaaattcttcccaagtagctcatacagaagaccagttggaatatccagtatggtagaggctatggtttcaagagcccgaattgatgcacggattgatttcgaatctggaaggataaagagggaggaatttgctgagatcatgaagatctgttccaccattgaagacctcagacggcaaaaatag

>H1N1_PB1_CY026633_A/New York/UR06-0199/2007

atggatgtcaatccgacattacttttcttaaaagtgccagcacaaaatgctataagcacaacttttccttatactggtgaccctccttacagccatgggacaggaacagggtacacaatggatacagtcaacaggacacatcagtactcagaaagaggaagatggacaaataataccgaaactggagcaccgcaactcaacccaattgatgggccactaccagaagacaatgaaccaagtggctatgcccaaacagattgtgtattagaagcaatggctttccttgaagaatcccatcctggcatttttgaaaactcttgtattgaaacaatggaggttgttcagcaaacaagggtggacaaactgacacaaggcagacagacctatgactggactctaaataggaaccagcctgctgccacagcattggccaacactatagaagtattcagatcaaacggcctcatagcaaatgaatctgggaggctaatagacttccttaaagatgtaatggagtcgatggacagagacgaagtagaggtcacaactcattttcagagaaagaggagagtgagagacaatgtaactaaaaaaatggtgacccaaagaacaataggcaaaaagaaacataaattagacaaaaggagttacctaattagagcattaaccctgaacacaatgaccaaagatgctgagagggggaaactaaaacgcagagcaattgcaaccccaggaatgcaaataagggggtttgtatactttgttgagacactggcaagaagcatatgtgaaaagcttgaacaatcagggttgcccgttggggggaacgagaagaaagcaaagttagcaaatgttgtaaggaagatgatgaccaactcccaggacactgaaatttctttcaccatcactggagataacacaaaatggaacgaaaatcaaaaccctagaatgtttttggccatgatcacatatataaccagaaatcagcctgaatggttcagaaatattctaagtattgctccaataatgttttcaaacaaaatggcgagactaggtaaggggtacatgtttgaaagcaagagtatgaaactgagaactcaaatacctgcagagatactagccgacatagatttgaaatatttcaatgagtcaactaaaaagaaaattgaaaaaatccgaccattattaatagatggaactgcatcattgagtcctggaatgatgatgggaatgttcaatatgttaagcactgtcttgggcgtctccattctgaaccttgggcaaaagagatacaccaagactacttactggtgggatggtcttcaatcgtctgatgattttgctctgatagtgaatgcgcccaactatgcaggaattcaagctggagttgacaggttttatcgaacctgtaagctgctcggaattaatatgagcaaaaagaagtcttacataaataggacaggtacctttgaattcacgagctttttctatcgttatgggtttgttgccaatttcagcatggagcttcctagttttggggtgtctggggtcaatgaatctgcagacatgagtattggagtcactgttatcaaaaacaatatgataaacaatgaccttggcccagcaactgctcaaatggcccttcagttatttataaaagattacagatacacgtatcgatgccacagaggtgacacacaaatacaaacccggagatcatttgagataaagaaattatgggaccaaacccgctccaaagctgggctgttggtctctgatggaggccccaatctatataacattagaaatctccatattcctgaagtctgcttgaaatgggagttgatggatgaggattaccaggggcgtttatgcaacccattgaacccttttgtcagtcataaagagattgaatcagtgaacaatgcagtgatgatgccggcacatggtccagccaaaaatatggagtatgacgctgttgcaacaacacattcctgggttcccaaaaggaatcgatccattttgaacacgagccaaagggggatacttgaggatgagcaaatgtatcagaggtgctgcaatttatttgaaaaattcttcccaagtagctcatacagaagaccagttgggatatccagtatggtagaggctatgatttccagagcccgaattgatgcacggattgatttcgaatctggaaggataaagaaagaggaattcgctgagatcatgaagacctgttccaccattgaagacctcagacggcaaaaatag

>H1N1_PB1_CY026697_A/Illinois/UR06-0224/2007

atggatgtcaatccgacattacttttcttaaaagtgccagcacaaaatgctataagcacaacttttccttatactggtgaccctccttacagccatgggacaggaacagggtacaccatggatacagtcaacaggacacaccagtactcagaaagaggaagatggacaaaaaataccgaaactggagcaccgcaactcaacccaattgatgggccactaccagaagacaatgaaccaagtggctatgcccaaacagattgtgtattagaagcaatggctttccttgaagaatcccatcctggtatctttgaaaactcttgtattgaaacaatggaggttgttcagcaaacaagggtggacaaactgacacaaggcagacagacctatgactggactctaaataggaaccagcctgctgcaacagcattggcaaacactatagaagtattcagatcaaacggcctcatagcaaatgaatctggaaggctaatagacttccttaaagatgtaatggagtcgatggacagagacgaagtagaggtcacaactcattttcaaagaaagaggagagtgagagacaatgtaactaaaaaaatggtgactcaaagaacaataggcaaaaagaaacataaattagacaaaagaagttacctaattagggcattaaccctgaacacaatgaccaaagatgctgagagggggaaactaaaacgcagagcaattgcaaccccaggaatgcaaataagagggtttgtatactttgttgagacactggcaagaagcatatgtgaaaagcttgaacaatcaggattgccagttggaggaaatgagaagaaagcaaagttagcaaatgttgtaaggaagatgatgaccaactcccaggacactgaaatttctttcaccatcactggagataacacaaaatggaacgaaaatcaaaaccctagaatgttcttggccatgatcacatatataaccaaaaatcagcctgaatggttcagaaatattctaagtattgctccaataatgttttcaaacaagatggcgagactaggtaaggggtacatgtttgaaagcaagagtatgaaactgagaactcaaatacctgcagagatgctagccaacatagatttgaaatatttcaatgattcaactaaaaagaaaattgaaaaaatccgaccattattaatagatggaactgcatcattgagtcctggaatgatgatgggcatgttcaatatgttgagcaccgtcttgggcgtctccattctgaatcttggacaaaagagatacaccaagactacttactggtgggatggtcttcaatcgtctgatgattttgctttgattgtgaatgcacccaactatgcaggaattcaagctggagttgacaggttttatcgaacctgtaagctgctcggaattaatatgagcaaaaagaagtcttacataaacagaacaggtacctttgaattcacgagctttttctatcgttatgggtttgttgccaatttcagcatggagcttcctagttttggggtgtctggggtcaatgaatctgcagacatgagtattggagtcactgtcatcaaaaacaatatgataaacaatgaccttggcccagcaactgctcaaatggcccttcagttatttataaaagattacaggtacacttatcgatgccacagaggtgacacacaaatacaaacccggagatcatttgagataaagaaactatgggaccaaacccgctccaaagctgggctgttggtctctgatggaggccccaatttatataacattagaaatctccatattcctgaagtttgcttgaaatgggagttgatggatgaggattatcaggggcgtttatgcaacccattaaacccgtttgtcagccataaagagattgaatcagtgaacaatgcagtgataatgccggcacatggcccagccaaaaatatggagtatgacgctgttgcaacaacacactcctgggtccccaaaagaaatcgatccattttgaacacgagccaaagggggatacttgaagatgagcaaatgtatcagaggtgctgcaatttatttgagaaattcttcccaagtagctcatacagaagaccagttggaatatccagtatggtagaggctatggtttcaagagcccgaattgatgcacggattgatttcgaatctggaaggataaagagggaggaatttgctgagatcatgaagatctgttccaccattgaagacctcagacggcaaaaatag

>H1N1_PB1_CY026737_A/New York/UR06-0134/2007

atggatgtcaatccgactttacttttcttaaaagtgccagcacaaaatgctataagcacaacttttccttatactggtgaccctccttacagccatgggacaggaacagggtacaccatggatacagtcaacaggacacaccagtactcagaaagaggaagatggacaaaaaataccgaaactggagcaccgcaactcaacccaattgatgggccactaccagaagacaatgaaccaagtggctatgcccaaacagattgtgtattagaagcaatggctttccttgaagaatcccatcctggtatctttgaaaactcttgtattgaaacaatggaggttgttcagcaaacaagggtggacaaactgacacaaggcagacagacctatgactggactctaaataggaaccagcctgctgcaacagcattggcaaacactatagaagtattcagatcaaacggcctcatagcaaatgaatctggaaggctaatagacttccttaaagatgtaatggagtcgatggacagagacgaaatagaggtcacaactcattttcaaagaaagaggagagtaagagacaatgtaactaaaaaaatggtgactcaaagaacaataggcaaaaagaaacataaattagacaaaagaagttacctaattagggcattaaccctgaacacaatgaccaaagatgctgagagggggaaactaaaacgcagagcaattgcaaccccaggaatgcaaataagagggtttgtatactttgttgagacactggcaagaagcatatgtgaaaagcttgaacaatcaggattgccagttggaggaaatgagaagaaagcaaagttagcaaatgttgtaaggaagatgatgaccaactcccaggacactgaaatttctttcaccatcactggagataacacaaaatggaacgaaaatcaaaaccctagaatgttcttggccatgatcacatatataaccaaaaatcagcctgaatggttcagaaatattctaagtattgctccaataatgttttcaaacaagatggcgagactaggtaaggggtacatgtttgaaagcaagagtatgaaactgagaactcaaatacctgcagagatgctagtcaacatagatttgaaatatttcaatgattcaactaaaaagaaaattgaaaaaattcgaccattattaatagatggaactgcatcattgagtcctggaatgatgatgggcatgttcaatatgttgagcaccgtcttgggcgtctccattctgaatcttgggcaaaagagatacaccaagactacttactggtgggatggtcttcaatcgtctgatgattttgctttgattgtgaatgcacccaactatgcaggaattcaagctggagttgacaggttttatcgaacctgtaagctgctcggaattaatatgagcaaaaagaagtcttacataaacagaacaggtacctttgaattcacgagctttttctatcgttatgggtttgttgccaatttcagcatggagcttcctagttttggggtgtctggggtcaatgaatctgcagacatgagtattggagtcactgtcatcaaaaacaatatgataaacaatgaccttggcccagcaactgctcaaatggcccttcagttatttataaaagattacaggtacacttatcgatgccacagaggtgacacacaaatacaaacccggagatcatttgagataaagaaactatgggaccaaacccgctccaaagctgggctgttggtctctgatggaggccccaatttatataacattagaaatctccatattcctgaagtttgcttgaaatgggagttgatggatgaggattaccaggggcgtttatgcaacccattaaacccgtttgtcagccataaagagattgaatcagtgaacaatgcagtgataatgccggcacatggcccagccaagaatatggagtatgacgctgttgcaacaacacactcctgggtccccaaaagaaatcgatccattttgaacacgagccaaagggggatacttgaagatgagcaaatgtatcagaggtgctgcaatttatttgaaaaattcttcccaagtagctcatacagaagaccagttggaatatccagtatggtagaggctatggtttcaagagcccgaattgatgcacggattgatttcgaatctggaaggataaagagggaggaatttgctgagatcatgaagatctgttccaccattgaagacctcagacggcaaaaatag

>H1N1_PB1_CY026809_A/Kentucky/UR06-0058/2007

atggatgtcaatccgacattacttttcttaaaagtgccagcacaaaatgctataagcacaacttttccttatactggtgaccctccttacagccatgggacaggaacagggtacaccatggatacagtcaacaggacacaccagtactcagaaagaggaagatggacaaaaaataccgaaactggagcaccgcaactcaacccaattgatgggccactaccagaagacaatgaaccaagtggctatgcccaaacagattgtgtattagaagcaatggctttccttgaagaatcccatcctggtatctttgaaaactcttgtattgaaacaatggaggttgttcagcaaacaagggtggacaaactgacacaaggcagacagacctatgactggactctaaataggaaccagcctgctgcaacagcattggcaaacactatagaagtattcagatcaaacggcctcatagcaaatgaatctggaaggctaatagacttccttaaagatgtaatggagtcgatggacagagacgaagtagaggtcacaactcattttcaaagaaagaggagagtaagagacaatgtaactaaaaaaatggtgactcaaagaacaataggcaaaaagaaacataaattagacaaaagaagttacctaattagggcattaaccctgaacacaatgaccaaagacgctgagagggggaaactaaaacgcagagcaattgcaaccccaggaatgcaaataagagggtttgtatactttgttgagacactggcaagaagcatatgtgaaaagcttgaacaatcaggattgccagttggaggaaatgagaagaaagcaaagttagcaaatgttgtaaggaagatgatgaccaactcccaggacactgaaatttctttcaccatcactggagataacacaaaatggaacgaaaatcaaaaccctagaatgttcttggccatgatcacatatataaccaaaaatcagcctgaatggttcagaaatattctaagtattgctccaataatgttttcaaacaagatggcgagactaggtaaggggtacatgtttgaaagcaagagtatgaaactgagaactcaaatacctgcagagatgctagccaacatagatttgaaatatttcaatgattcaactaaaaagaaaattgaaaaaatccgaccattattaatagatggaactgcatcattgagtcctggaatgatgatgggcatgttcaatatgttgagcaccgtcttgggcgtctccattctgaatcttgggcaaaagagatacaccaagactacttactggtgggatggtcttcaatcgtctgatgattttgctttgattgtgaatgcacccaactatgcaggaattcaagctggagttgacaggttttatcgaacctgtaagctgctcggaattaatatgagcaaaaagaagtcttacataaacagaacaggtacctttgaattcacgagctttttctatcgttatgggtttgttgccaatttcagcatggagcttcctagttttggggtgtctggggtcaatgaatctgcagacatgagtattggagtcactgtcatcaaaaacaatatgataaacaatgaccttggcccagcaactgctcaaatggcccttcagttatttataaaagattacaggtacacttatcgatgccacagaggtgacacacaaatacaaacccggagatcatttgagataaagaaactatgggaccaaacccgctccaaagctgggctgttggtctctgatggaggccccaatttatataacattagaaatctccatattcctgaagtttgcttgaaatgggagttgatggatgaggattaccaggggcgtttatgcaacccattaaacccgtttgtcagccataaagagattgaatcagtgaacaatgcagtgataatgccggcacatggcccagccaaaaatatggagtatgacgctgttgcaacaacacactcctgggtccccaaaagaaatcgatccattttgaacacgagccaaagggggatacttgaagatgagcaaatgtatcagaggtgctgcaatttatttgaaaaattcttcccaagtagctcatacagaagaccagttggaatatccagtatggtagaggctatggtttcaagagcccgaattgatgcacggattgatttcgaatctggaaggataaagagggaggaatttgctgagatcatgaagatctgttccaccattgaagacctcagacggcaaaaatag

>H1N1_PB1_CY026873_A/Kentucky/UR06-0127/2007

atggatgtcaatccgacattacttttcttaaaagtgccagcacaaaatgctataagcacaacttttccttatactggtgaccctccttacagccatgggacaggaacagggtacaccatggatacagtcaacaggacacaccagtactcagaaagaggaagatggacaaaaaataccgaaactggagcaccgcaactcaacccaattgatgggccactaccagaagacaatgaaccaagtggctatgcccaaacagattgtgtattagaagcaatggctttccttgaagaatcccatcctggtatctttgaaaactcttgtattgaaacaatggaggttgttcagcaaacaagggtggacaaactgacacaaggcagacagacctatgactggactctaaataggaaccagcctgctgcaacagcattggcaaacactatagaagtattcagatcaaacggcctcatagcaaatgaatctggaaggctaatagacttccttaaagatgtaatggagtcgatggacagagacgaagtagaggtcacaactcattttcaaagaaagaggagagtgagagacaatgtaactaaaaaaatggtgactcaaagaacaataggcaaaaagaaacataaattagacaaaagaagttacctaattagggcattaaccctgaacacaatgaccaaagatgctgagagggggaaactaaaacgcagagcaattgcaaccccaggaatgcaaataagagggtttgtatactttgttgagacactggcaagaagcatatgtgaaaagcttgaacaatcaggattgccagttggaggaaatgagaagaaagcaaagttagcaaatgttgtaaggaagatgatgaccaactcccaggacactgaaatttctttcaccatcactggagataacacaaaatggaacgaaaatcaaaaccctagaatgttcttggccatgatcacatatataaccaaaaatcagcctgaatggttcagaaatattctaagtattgctccaataatgttttcaaacaagatggcgagactaggtaaggggtacatgtttgaaagcaagagtatgaaactgagaactcaaatacctgcagagatgctagccaacatagatttgaaatatttcaatgattcaactaaaaagaaaattgaaaaaatccgaccattattaatagatggaactgcatcattgagtcctggaatgatgatgggcatgttcaatatgttgagcaccgtcttgggcgtctccattctgaatcttgggcaaaagagatacaccaagactacttactggtgggatggtcttcaatcgtctgatgattttgctttgattgtgaatgcacccaactatgcaggaattcaagctggagttgacaggttttatcgaacctgtaagctgctcggaattaatatgagcaaaaagaagtcttacataaacagaacaggtacctttgaattcacgagctttttctatcgttatgggtttgttgccaatttcagcatggagcttcctagttttggggtgtctggggtcaatgaatctgcagacatgagtattggagtcactgtcatcaaaaacaatatgataaacaatgaccttggcccagcaactgctcaaatggcccttcagttatttataaaagattacaggtacacttatcgatgccacagaggtgacacacaaatacaaacccggagatcatttgagataaagaaactatgggaccaaacccgctccaaagctgggctgttggtctctgatggaggccccaatttatataacattagaaatctccatattcctgaagtttgcttgaaatgggagttgatggatgaggattaccaggggcgtttatgcaacccattaaacccgtttgtcagccataaagagattgaatcagtgaacaatgcagtgataatgccagcacatggcccagccaaaaatatggagtatgacgctgttgcaacaacacactcctgggtccccaaaagaaatcgatccattttgaacacgagccaaagggggatacttgaagatgagcaaatgtatcagaggtgctgcaatttatttgaaaaattcttcccaagtagctcatacagaagaccagttggaatatccagtatggtagaggctatggtttcaagagcccgaattgatgcacggattgatttcgaatctggaaggataaagagggaggaatttgctgagatcatgaagatctgttccaccattgaagacctcagacggcaaaaatag

>H1N1_PB1_CY027009_A/Ohio/UR06-0465/2007

atggatgtcaatccgacattacttttcttaaaagtgccagcacaacatgctataagcacaacttttccttatactggtgaccctccttacagccatgggacaggaacagggtacaccatggatacagtcaacaggacacaccagtactcagaaagaggaagatggacaaaaaataccgaaactggagcaccgcaactcaacccaattgatgggccactaccagaagacaatgaaccaagtggctatgcccaaacagattgtgtattagaagcaatggctttccttgaagaatcccatcctggtatctttgaaaactcttgtattgaaacaatggaggttgttcagcaaacaagggtggacaaactgacacaaggcagacagacctatgactggactctaaataggaaccagcctgctgcaacagcattggcaaacactatagaagtattcagatcaaacggcctcatagcaaatgaatctggaaggctaatagacttccttaaagatgtaatggagtcgatggacagagacgaagtagaggtcacaactcattttcaaagaaagaggagagtaagagacaatgtaactaaaaaaatggtgactcaaagaacaataggcaaaaagaaacataaattagacaaaagaagttacctaattagggcattaaccctgaacacaatgaccaaagacgctgagagggggaaactaaaacgcagagcaattgcaaccccaggaatgcaaataagagggtttgtatactttgttgagacactggcaagaagcatatgtgaaaagcttgaacaatcaggattgccagttggaggaaatgagaagaaagcaaagttagcaaatgttgtaaggaagatgatgaccaactcccaggacactgaaatttctttcaccatcactggagataacacaaaatggaacgaaaatcaaaaccctagaatgttcttggccatgatcacatatataaccaaaaatcagcctgaatggttcagaaatattctaagtattgctccaataatgttttcaaacaagatggcgagactaggtaaggggtacatgtttgaaagcaagagtatgaaactgagaactcaaatacctgcagagatgctagccaacatagatttgaaatatttcaatgattcaactaaaaagaaaattgaaaaaatccgaccattattaatagatggaactgcatcattgagtcctggaatgatgatgggcatgttcaatatgttgagcaccgtcttgggcgtctccattctgaatcttgggcaaaagagatacaccaagactacttactggtgggatggtctccaatcgtctgatgattttgctttgattgtgaatgcacccaactatgcaggaattcaagctggagttgacaggttttatcgaacctgtaagctgctcggaattaatatgagcaaaaagaagtcttacataaacagaacaggtacctttgaattcacgagctttttctatcgttatgggtttgttgccaatttcagcatggagcttcctagttttggggtgtctggggtcaatgaatctgcagacatgagtattggagtcactgtcatcaaaaacaatatgataaacaatgaccttggcccagcaactgctcaaatggcccttcagttatttataaaagattacaggtacacttatcgatgccacagaggtgacacacaaatacaaacccggagatcatttgagataaagaaactatgggaccaaacccgctccaaagctgggctgttggtctctgatggaggccccaatttatataacattagaaatctccatattcctgaagtttgcttgaaatgggagttgatggatgaggattaccaggggcgtttatgcaacccattaaacccgtttgtcagccataaagagattgaatcagtgaacaatgcagtgataatgccggcacatggcccagccaaaaatatggagtatgacgctgttgcaacaacacactcctgggtccccaaaagaaatcgatccattttgaacacgagccaaagggggatacttgaagatgagcaaatgtatcagaggtgctgcaatttatttgaaaaattcttcccaagtagctcatacagaagaccagttggaatatccagtatggtagaggctatggtttcaagagcccgaattgatgcacggattgatttcgaatctggaaggataaagagggaggaatttgctgagatcatgaagatctgttccaccattgaagacctcagacggcaaaaatag

>H1N1_PB1_CY027449_A/Ohio/UR06-0121/2007

atggatgtcaatccgacattacttttcttaaaagtgccagcacaaaatgctataagcacaacttttccttatactggtgaccctccttacagccatgggacaggaacagggtacaccatggatacagtcaacaggacacaccagtactcagaaagaggaagatggacaaaaaataccgaaactggagcaccgcaactcaacccaattgatgggccactaccagaagacaatgaaccaagtggctatgcccaaacagattgtgtattagaagcaatggctttccttgaagaatcccatcctggtatctttgaaaactcttgtattgaaacaatggaggttgttcagcaaacaagggtggacaaactgacacaaggcagacagacctatgactggactctaaataggaaccagcctgctgcaacagcattggcaaacactatagaagtattcagatcaaacggcctcatagcaaatgaatctggaaggctaatagacttccttaaagatgtaatggagtcgatggacagagacgaagtagaggtcacaactcattttcaaagaaagaggagagtaagagacaatgtaactaaaaaaatggtgactcaaagaacaataggcaaaaagaaacataaattagacaaaagaagttacctaattagggcattaaccctgaacacaatgaccaaagacgctgagagggggaaactaaaacgcagagcaattgcaaccccaggaatgcaaataagagggtttgtatactttgttgagacactggcaagaagcatatgtgaaaagcttgaacaatcaggattgccagttggaggaaatgagaagaaagcaaagttagcaaatgttgtaaggaagatgatgaccaactcccaggacactgaaatttctttcaccatcactggagataacacaaaatggaacgaaaatcaaaaccctagaatgttcttggccatgatcacatatataaccaaaaatcagcctgaatggttcagaaatattctaagtattgctccaataatgttttcaaacaagatggcgagactaggtaaggggtacatgtttgaaagcaagagtatgaaactgagaactcaaatacctgcagagatgctagccaacatagatttgaaatatttcaatgattcaactaaaaagaaaattgaaaaaatccgaccattattaatagatggaactgcatcattgagtcctggaatgatgatgggcatgttcaatatgttgagcaccgtcttgggcgtctccattctgaatcttgggcaaaagagatacaccaagactacttactggtgggatggtcttcaatcgtctgatgattttgctttgattgtgaatgcacccaactatgcaggaattcaagctggagttgacaggttttatcgaacctgtaagctgctcggaattaatatgagcaaaaagaagtcttacataaacagaacaggtacctttgaattcacgagctttttctatcgttatgggtttgttgccaatttcagcatggagcttcctagttttggggtgtctggggtcaatgaatctgcagacatgagtattggagtcactgtcatcaaaaacaatatgataaacaatgaccttggcccagcaactgctcaaatggcccttcagttatttataaaagattacaggtacacttatcgatgccacagaggtgacacacaaatacaaacccggagatcatttgagataaagaaactatgggaccaaacccgctccaaagctgggctgttggtctctgatggaggccccaatttatataacattagaaatctccatattcctgaagtttgcttgaaatgggagttgatggatgaggattaccaggggcgtttatgcaacccattaaacccgtttgtcagccataaagagattgaatcagtgaacaatgcagtgataatgccggcacatggcccagccaaaaatatggagtatgacgctgttgcaacaacacactcctgggtccccaaaagaaatcgatccattttgaacacgagccaaagggggatacttgaagatgagcaaatgtatcagaggtgctgcaatttatttgaaaaattcttcccaagtagctcatacagaagaccagttggaatatccagtatggtagaggctatggtttcaagagcccgaattgatgcacggattgatttcgaatctggaaggataaagagggaggaatttgctgagatcatgaagatctgttccaccattgaagacctcagacggcaaaaatag

>H1N1_PB1_CY027489_A/Illinois/UR06-0115/2007

atggatgtcaatccgacattacttttcttaaaagtgccagcacaaaatgctataagcacaacttttccttatactggtgaccctccttacagccatgggacaggaacagggtacaccatggatacagtcaacaggacacaccagtactcagaaagaggaagatggacaaaaaataccgaaactggagcaccgcaactcaacccaattgatgggccactaccagaagacaatgaaccgagtggctatgcccaaacagattgtgtattagaagcaatggctttccttgaagaatcccatcctggtatctttgaaaactcttgtattgagacaatggaggttgttcagcaaacaagggtggacaaactaacacaaggcagacagacctatgactggactctaaataggaaccagcctgctgccacagcattggcaaacactatagaagtattcagatcaaacggcctcatagcaaatgaatctgggaggctaatagacttccttaaagatgtaatggagtcgatggacatagacgaaatagaggtcacaactcattttcaaagaaagaggagagtgagagacaatgtaactaaaaaaatggtgacccaaagaacaataggcaaaaagaaacataaattagacaaaagaagttacctaattagggcattaaccctgaacacaatgaccaaagatgctgagagagggaaactaaaacgcagagcaattgcaaccccaggaatgcaaataagggggtttgtatactttgttgagacactggcaagaagcatatgtgaaaagcttgaacaatcaggattgccagttggaggaaatgagaagaaagcaaagttagcaaatgttgtaaggaagatgatgaccaactcccaggacactgaaatttctttcaccatcactggagataacacaaaatggaacgaaaatcaaaaccctagaatgttcttggccatgatcacatatataaccaaaaatcagcctgaatggttcagaaatattctaagtattgccccaataatgttttcaaacaagatggcgagactaggtaaggggtacatgtttgaaagcaagagtatgaaactgagaactcaaatacctgcagagatgctagccaacatagatttgaaatatttcaatgattcaactaaaaagaaaattgaaaaaatccgaccattattaatagatggaactgcatcattgagtcctggaatgatgatgggcatgttcaatatgttgagcaccgtcttgggcgtctccattctgaatcttgggcaaaagagatacaccaagactacttactggtgggatggtcttcaatcgtctgatgattttgctttgattgtgaatgcacccaactatgcaggaattcaagctggagttgacaggttttatcgaacctgtaagctgctcggaattaatatgagcaaaaagaagtcttacataaacagaacaggtacctttgaattcacgagctttttctatcgttatgggtttgttgccaatttcagcatggagcttcctagttttggggtgtctggggtcaatgaatctgcagacatgagtattggagtcactgtcatcaaaaacaatatgataaacaatgaccttggcccagcaactgctcaaatggcccttcagttatttataaaagattacaggtacacttatcgatgccacagaggtgacacacaaatacaaacccggagatcatttgagataaagaaactatgggaccaaacccgctccaaagctgggctgttggtctctgatggaggccccaatttatataacattagaaatctccatattcctgaagtttgcttgaaatgggagttgatggatgaggattaccaggggcgtttatgcaacccattaaacccgtttgtcagccataaagagattgaatcagtaaacaatgcagtgataatgccggcacatggtccagccaaaaatatggagtatgacgctgttgcaacaacacactcctgggtccccaaaagaaatcgatccattttgaacacgagccaaagggggatacttgaagatgagcaaatgtatcagaggtgttgcaatttatttgaaaaattcttcccaagtagctcatacagaagaccagttggaatatccagtatggtagaggctatggtttcaagagcccgaattgatgcacggattgatttcgaatctggaaggataaagaaagaggaatttgctgagatcatgaagatctgttccaccattgaagacctcagacggcaaaaatag

>H1N1_PB1_CY027497_A/Tennessee/UR06-0473/2007

atggatgtcaatccgactttacttttcttaaaagtgccagcacaaaatgctataagcacaacttttccttatactggtgaccctccttacagccatgggacaggaacagggtacaccatggatacagtcaacaggacacaccagtactcagaaagaggaagatggacaaaaaataccgaaactggagcaccgcaactcaacccaattgatgggccactaccagaagacaatgaaccaagtggctatgcccaaacagattgtgtattagaagcaatggctttccttgaagaatcccatcctggtatctttgaaaactcttgtattgaaacaatggaggttgttcagcaaacaagggtggacaaactgacacaaggcagacagacctatgactggactctaaataggaaccagcctgctgcaacagcattggcaaacactatagaagtattcagatcaaacggcctcatagcaaatgaatctggaaggctaatagacttccttaaagatgtaatggagtcgatggacagagacgaagtagaggtcacaactcattttcaaagaaagaggagagtaagagacaatgtaactaaaaaaatggtgactcaaagaacaataggcaaaaagaaacataaattagacaaaagaagttacctaattagggcattaaccctgaacacaatgaccaaagatgctgagagggggaaactaaaacgcagagcaattgcaaccccaggaatgcaaataagagggtttgtatactttgttgagacactggcaagaagcatatgtgaaaaacttgaacaatcaggattgccagttggaggaaatgagaagaaagcaaagttagcaaatgttgtaaggaagatgatgaccaactcccaggacactgaaatttctttcaccatcactggagataacacaaaatggaacgaaaatcaaaaccctagaatgttcttggccatgatcacatatataaccaaaaatcagcctgaatggttcagaaatattctaagtattgctccaataatgttttcaaacaagatggcgagactaggtaaggggtacatgtttgaaagcaagagtatgaaactgagaactcaaatacctgcagagatgctagtcaacatagatttgaaatatttcaatgattcaactaaaaagaaaattgaaaaaatccgaccattattaatagatggaactgcatcattgagtcctggaatgatgatgggcatgttcaatatgttgagcaccgtcttgggcgtctccattctgaatcttgggcaaaagagatacaccaagactacttactggtgggatggtcttcaatcgtctgatgattttgctttgattgtgaatgcacccaactatgcaggaattcaagctggagttgacaggttttatcgaacctgtaagctgctcggaattaatatgagcaaaaagaagtcttacataaacagaacaggtacctttgaattcacgagctttttctatcgttatgggtttgttgccaatttcagcatggagcttcctagttttggggtgtctggggtcaatgaatctgcagacatgagtattggagtcactgtcatcaaaaacaatatgataaacaatgacctcggcccagcaactgctcaaatggcccttcagttatttataaaagattacaggtacacttatcgatgccacagaggtgacacacaaatacaaacccggagatcatttgaaataaagaaactatgggaccaaacccgctccaaagctgggctgttggtctctgatggaggccccaatttatataacattagaaatctccatattcctgaagtttgcttgaaatgggagttgatggatgaggattaccaggggcgtttatgcaacccattaaacccgtttgtcagccataaagagattgaatcagtgaacaatgcagtgataatgccggcacatggcccagccaaaaatatggagtatgacgctgttgcaacaacacactcctgggtccccaaaagaaatcgatccattttgaacacgagccaaagggggatacttgaagatgagcaaatgtatcagaggtgctgcaatttatttgaaaaattcttcccaagtagctcatacagaagaccagttggaatatccagtatggtagaggctatggtttcaagagcccgaattgatgcacggattgatttcgaatctggaaggataaagagggaggaatttgctgagatcatgaagatctgttccaccattgaagacctcagacggcaaaaatag

>H1N1_PB1_CY027617_A/Florida/UR06-0447/2007

atggatgtcaatccgacattacttttcttaaaagtgccagcacaaaatgctataagcacaacttttccttatactggtgaccctccttacagccatgggacaggaacagggtacaccatggatacagtcaacaggacacaccagtactcagaaagaggaagatggacaaaaaatacagaaactggagcaccgcaactcaacccaattgatgggccactaccagaagacaatgaaccaagtggctatgcccaaacagattgtgtattagaagcaatggctttccttgaagaatcccatcctggtatctttgaaaactcttgtattgaaacaatggaggttgttcagcaaacaagggtggacaaactgacacaaggcagacagacctatgactggactctaaataggaaccagcctgctgcaacagcattggcaaacactatagaagtattcagatcaaacggcctcatagcaaatgaatctggaaggctaatagacttccttaaagatgtaatggagtcgatggacagagacgaagtagaggtcacaacccattttcaaagaaagagaagagtgagagacaatgtgactaaaaaaatggtgactcaaagaacaataggcaaaaagaaacataaattagacaaaagaagttacctaattagggcattaaccctgaacacaatgaccaaagatgctgagagggggaaactaaaacgcagagcaattgcaaccccaggaatgcaaataagagggtttgtatactttgttgagacactggcaagaagcatatgtgaaaagcttgaacaatcaggattgccagttggaggaaatgagaagaaagcaaagttagcaaatgttgtaaggaagatgatgaccaactcccaggacactgaaatttctttcaccatcactggagataacacaaaatggaacgaaaatcaaaaccctagaatgttcttggccatgatcacatatataaccaaaaatcagcctgaatggttcagaaatattctaagtattgctccaataatgttttcaaacaagatggcgagactaggtaaggggtacatgtttgaaagcaagagtatgaaactgagaactcaaatacctgcagagatgctagccaacatagatttgaaatatttcaatgattcaactaaaaagaaaattgaaaaaatccgaccattattaatagatggaactgcatcattgagtcctggaatgatgatgggcatgttcaatatgttgagcaccgtcttgggcgtctccattctgaatcttgggcaaaagagatacaccaagactacttactggtgggatggtcttcaatcgtctgatgattttgctttgattgtgaatgcacccaactatgcaggaattcaagctggagttgacaggttttatcgaacctgtaagctgctcggaattaatatgagcaaaaagaagtcttacataaacagaacaggtacctttgaattcacgagctttttctatcgttatgggtttgttgccaatttcagcatggagcttcctagttttggggtgtctggggtcaatgaatctgcagacatgagtattggagtcactgtcatcaaaaacaatatgataaacaatgaccttggcccagcaactgctcaaatggcccttcagttatttataaaagattacaggtacacttatcgatgccacagaggtgacacacaaatacaaacccggagatcatttgagataaagaaactatgggaccaaacccgctccaaagctgggctgttggtctctgatggaggccccaatttatataacattagaaatctccatattcctgaagtttgcttgaaatgggagttgatggatgaggattaccaggggcgtttatgcaacccattaaacccgtttgtcagccataaagagattgaatcagtgaacaatgcagtgataatgccggcacatggcccagccaaaaatatggagtatgacgctgttgcaacaacacactcctgggtccccaaaagaaatcgatccattttgaacacgagccaaagggggatacttgaagatgagcaaatgtatcagaggtgctgcaatttatttgaaaaattcttcccaagtagctcatacagaagaccagttggaatatccagtatggtagaggctatggtttcaagagcccgaattgatgcacggattgatttcgaatctggaaggataaagagggaggaatttgctgagatcatgaagatctgttccaccattgaagacctcagacggcaaaaatag

>H1N1_PB1_CY027705_A/Illinois/UR06-0131/2007

atggatgtcaatccgacattacttttcttaaaagtgccagcacaaaatgctataagcacaacttttccttatactggtgaccctccttacagccatgggacaggaacagggtacaccatggatacagtcaacaggacacaccagtactcagaaagaggaagatggacaaaaaataccgaaactggagcaccgcaactcaacccaattgatgggccactaccagaagacaatgaaccgagtggctatgcccaaacagattgtgtattagaagcaatggctttccttgaagaatcccatcctggtatctttgaaaactcttgtattgagacaatggaggttgttcagcaaacaagggtggacaaactaacacaaggcagacagacctatgactggactctaaataggaaccagcctgctgccacagcattggcaaacactatagaagtattcagatcaaacggcctcatagcaaatgaatctgggaggctaatagacttccttaaagatgtaatggagtcgatggacatagacgaaatagaggtcacaactcattttcaaagaaagaggagagtgagagacaatgtaactaaaaaaatggtgacccaaagaacaataggcaaaaagaaacataaattagacaaaagaagttacctaattagggcattaaccctgaacacaatgaccaaagatgctgagagagggaaactaaaacgcagagcaattgcaaccccaggaatgcaaataagggggtttgtatactttgttgagacactggcaagaagcatatgtgaaaagcttgaacaatcaggattgccagttggaggaaatgagaagaaagcaaagttagcaaatgttgtaaggaagatgatgaccaactcccaggacactgaaatttctttcaccatcactggagataacacaaaatggaacgaaaatcaaaaccctagaatgttcttggccatgatcacatatataaccaaaaatcagcctgaatggttcagaaatattctaagtattgccccaataatgttttcaaacaagatggcgagactaggtaaggggtacatgtttgaaagcaagagtatgaaactgagaactcaaatacctgcagagatgctagccaacatagatttgaaatatttcaatgattcaactaaaaagaaaattgaaaaaatccgaccattattaatagatggaactgcatcattgagtcctggaatgatgatgggcatgttcaatatgttgagcaccgtcttgggcgtctccattctgaatcttgggcaaaagagatacaccaagactacttactggtgggatggtcttcaatcgtctgatgattttgctttgattgtgaatgcacccaactatgcaggaattcaagctggagttgacaggttttatcgaacctgtaagctgctcgggattaatatgagcaaaaagaagtcttacataaacagaacaggtacctttgaattcacgagctttttctatcgttatgggtttgttgccaatttcagcatggagcttcctagttttggggtgtctggggtcaatgaatctgcagacatgagtattggagtcactgtcatcaaaaacaatatgataaacaatgaccttggcccagcaactgctcaaatggcccttcagttatttataaaagattacaggtacacttatcgatgccacagaggtgacacacaaatacaaacccggagatcatttgagataaagaaactatgggaccaaacccgctccaaagctgggctgttggtctctgatggaggccccaatttatataacattagaaatctccatattcctgaagtttgcttgaaatgggagttgatggatgaggattaccaggggcgtttatgcaacccattaaacccgtttgtcagccataaagagattgaatcagtaaacaatgcagtgataatgccggcacatggtccagccaaaaatatggagtatgacgctgttgcaacaacacactcctgggtccccaaaagaaatcgatccattttgaacacgagccaaagggggatacttgaagatgagcaaatgtatcagaggtgctgcaatttatttgaaaaattcttcccaagtagctcatacagaagaccagttggaatatccagtatggtagaggctatggtttcaagagcccgaattgatgcacggattgatttcgaatctggaaggataaagaaagaggaatttgctgagatcatgaagatctgttccaccattgaagacctcagacggcaaaaatag

>H1N1_PB1_CY027921_A/Kentucky/UR06-0259/2007

atggatgtcaatccgacattacttttcttaaaagtgccagcacaaaatgctataagcacaacttttccttatactggtgaccctccttacagccatgggacaggaacagggtacaccatggatacagtcaacaggacacaccagtactcagaaagaggaagatggacaaaaaataccgaaactggagcaccgcaactcaacccaattgatgggccactaccagaagacaatgaaccaagtggctatgcccaaacagattgtgtattagaagcaatggctttccttgaagaatcccatcctggtatctttgaaaactcttgtattgaaacaatggaggttgttcagcaaacaagggtggacaaactgacacaaggcagacagacctatgactggactctaaataggaaccagcctgctgcaacagcattggcaaacactatagaagtattcagatcaaacggcctcatagcaaatgaatctggaaggctaatagacttccttaaagatgtaatggagtcgatggacagagacgaagtagaggtcacaactcattttcaaagaaagaggagagtgagagacaatgtaactaaaaaaatggtgactcaaagaacaataggcaaaaagaaacataaattagacaaaagaagttacctaattagggcattaaccctgaacacaatgaccaaagatgctgagagggggaaactaaaacgcagagcaattgcaaccccaggaatgcaaataagagggtttgtatactttgttgagacactggcaagaagcatatgtgaaaagcttgaacaatcaggattgccagttggaggaaatgagaagaaagcaaagttagcaaatgttgtaaggaagatgatgaccaactcccaggacactgaaatttctttcaccatcactggagataacacaaaatggaacgaaaatcaaaaccctagaatgttcttggccatgatcacatatataaccaaaaatcagcctgaatggttcagaaatattctaagtattgctccaataatgttttcaaacaagatggcgagactaggtaaggggtacatgtttgaaagcaagagtatgaaactgagaactcaaatacctgcagagatgctagccaacatagatttgaaatatttcaatgattcaactaaaaagaaaattgaaaaaatccgaccattattaatagatggaactgcatcattgagtcctggaatgatgatgggcatgttcaatatgttgagcaccgtcttgggcgtctccattctgaatcttgggcaaaagagatacaccaagactacttactggtgggatggtcttcaatcgtctgatgattttgctttgattgtgaatgcacccaactatgcaggaattcaagctggagttgacaggttttatcgaacctgtaagctgctcggaattaatatgagcaaaaagaagtcttacataaacagaacaggtacctttgaattcacgagctttttctatcgttatgggtttgttgccaatttcagcatggagcttcctagttttggggtgtctggggtcaatgaatctgcagacatgagtattggagtcactgtcatcaaaaacaatatgataaacaatgaccttggcccagcaactgctcaaatggccctccagttatttataaaagattacaggtacacttatcgatgccacagaggtgacacacaaatacaaacccggagatcatttgagataaagaaactatgggaccaaacccgctccaaagctgggctgttggtctctgatggaggccccaatttatataacattagaaatctccatattcctgaagtttgcttgaaatgggagttgatggatgaggattaccaggggcgtttatgcaacccattaaacccgtttgtcagccataaagagattgaatcagtgaacaatgcagtgataatgccggcacatggcccagccaaaaatatggagtatgacgctgttgcaacaacacactcctgggtccccaaaagaaatcgatccattttgaacacgagccaaagggggatacttgaagatgagcaaatgtatcagaggtgctgcaatttatttgaaaaattcttcccaagtagctcatacagaagaccagttggaatatccagtatggtagaggctatggtttcaagagcccgaattgatgcacggattgatttcgaatctggaaggataaagagggaggaatttgctgagatcatgaagatctgttccaccattgaagacctcagacggcaaaaatag

>H1N1_PB1_CY028017_A/Kansas/UR06-0084/2007

atggatgtcaatccgacattacttttcttaaaagtgccagcacaaaatgctataagcacaacttttccttatactggtgaccctccttacagccatgggacaggaacagggtacaccatggatacagtcaacaggacacaccagtactcagaaagaggaagatggacaaaaaataccgaaactggagcaccgcaactcaacccaattgatgggccactaccagaagacaatgaaccaagtggctatgcccaaacagattgtgtattagaagcaatggctttccttgaagaatcccatcctggtatctttgaaaactcttgtattgaaacaatggaggttgttcagcaaacaagggtggacaaactgacacaaggcagacagacctatgactggactctaaataggaaccagcctgctgcaacagcattggcaaacactatagaagtattcagatcaaacggcctcatagcaaatgaatctggaaggctaatagacttccttaaagatgtaatggagtcgatggacagagacgaagtagaggtcacaactcattttcaaagaaagaggagagtgagagacaatgtaactaaaaaaatggtgactcaaagaacaataggcaaaaagaaacataaattagacaaaagaagttacctaattagggcattaaccctgaacacaatgaccaaagatgctgagagggggaaactaaaacgcagagcaattgcaaccccaggaatgcaaataagagggtttgtatactttgttgagacactggcaagaagcatatgtgaaaagcttgaacaatcaggattgccagttggaggaaatgagaagaaagcaaagttagcaaatgttgtaaggaagatgatgaccaactcccaggacactgaaatttctttcaccatcactggagataacacaaaatggaacgaaaatcaaaaccctagaatgttcttggccatgatcacatatataaccaaaaatcagcctgaatggttcagaaatattctaagtattgctccaataatgttttcaaacaagatggcgagactaggtaaggggtacatgtttgaaagcaagagtatgaaactgagaactcaaatacctgcagagatgctagccaacatagatttgaaatatttcaatgattcaactaaaaagaaaattgaaaaaatccgaccattattaatagatggaactgcatcattgagtcctggaatgatgatgggcatgttcaatatgttgagcaccgtcttgggcgtctccattctgaatcttgggcaaaagagatacaccaagactacttactggtgggatggtcttcaatcgtctgatgattttgctttgattgtgaatgcacccaactatgcaggaattcaagctggagttgacaggttttatcgaacctgtaagctgctcggaattaatatgagcaaaaagaagtcttacataaacagaacaggtacctttgaattcacgagctttttctatcgttatgggtttgttgccaatttcagcatggagcttcctagttttggggtgtctggggtcaatgaatctgcagacatgagtattggagtcactgtcatcaaaaacaatatgataaacaatgaccttggcccagcaactgctcaaatggcccttcagttatttataaaagattacaggtacacttatcgatgccacagaggtgacacacaaatacaaacccggagatcatttgagataaagaaactatgggaccaaacccgctccaaagctgggctgttggtctctgatggaggccccaatttatataacattagaaatctccatattcctgaagtttgcttgaaatgggagttgatggatgaggattatcaggggcgtttatgcaacccattaaacccgtttgtcagccataaagagattgaatcagtgaacaatgcagtgataatgccggcacatggcccagccaaaaatatggagtatgacgctgttgcaacaacacactcctgggtccccaaaagaaatcgatccattttgaacacgagccaaagggggatacttgaagatgagcaaatgtatcagaggtgctgcaatttatttgagaaattcttcccaagtagctcatacagaagaccagttggaatatccagtatggtagaggctatggtttcaagagcccgaattgatgcacggattgatttcgaatctggaaggataaagagggaggaatttgctgagatcatgaagatctgttccaccattgaagacctcagacggcaaaaatag

>H1N1_PB1_CY028041_A/Kentucky/UR06-0046/2007

atggatgtcaatccgacattacttttcttaaaagtgccagcacaaaatgctataagcacaacttttccttatactggtgaccctccttacagccatgggacaggaacagggtacaccatggatacagtcaacaggacacaccagtactcagaaagaggaagatggacaaaaaataccgaaactggagcaccgcaactcaacccaattgatgggccactaccagaagacaatgaaccaagtggctatgcccaaacagattgtgtattagaagcaatggctttccttgaagaatcccatcctggtatctttgaaaactcttgtattgaaacaatggaggttgttcagcaaacaagggtggacaaactgacacaaggcagacagacctatgactggactctaaataggaaccagcctgctgccacagcattggcaaacactatagaagtattcagatcaaacggcctcatagcaaatgaatctgggaggctaatagacttccttaaagatgtaatggagtcgatggacagagacgaagtagaggtcacaactcattttcaaagaaagaggagagtgagagacaatgtaactaaaaaaatggtgacccaaagaacaataggcaaaaagaaacataaattagacaaaagaagttacctaattagggcattaaccctaaacacaatgaccaaagatgctgaaagggggaaactaaaacgcagagcaattgcaaccccaggaatgcaaataagggggtttgtgtactttgttgagacactggcaagaagcatatgtgaaaagcttgaacaatcaggattgccagttggaggaaatgagaagaaagcaaagttagcaaatgttgtaaggaagatgatgaccaactcccaggacactgaaatttctttcaccattactggagataacacaaaatggaacgaaaatcaaaaccctagaatgttcttggccatgatcacatatataaccaaaaatcagcctgaatggttcagaaatattctaagtattgctccaataatgttttcaaacaagatggcgagactaggtaaagggtacatgtttgaaagcaagagtatgaaactgagaactcaaatacctgcagaaatgctagccaatatagatttgaaatatttcaatgattcaactaaaaagaaaattgaaaaaatccgaccattattaatagatggaactgcatcattgagtcctggaatgatgatgggcatgttcaatatgttgagcaccgtcttgggcgtctccattctgaatcttgggcaaaagagatacaccaagactacttactggtgggatggtcttcaatcgtctgatgattttgctttgattgtgaatgcacccaactatgcaggaattcaagctggagttgacaggttttatcgaacctgtaagctgctcggaattaatatgagcaaaaagaagtcttacataaacagaacaggtacctttgaattcacgagctttttctatcgttatgggtttgttgccaatttcagcatggagcttcctagttttggggtgtctggggtcaatgaatctgcagacatgagtattggagtcactgtcatcaaaaacaatatgataaacaatgaccttggcccagcaactgctcaaatggcccttcagttatttataaaagattacaggtacacttatcgatgccacagaggtgacacacaaatacaaacccggagatcatttgagataaagaaactatgggaccaaacccactccaaagctgggctgttggtctctgatggaggccccaatttatataacattagaaatctccatattcctgaagtttgcttgaaatgggagttgatggatgaggattaccaggggcgtttatgcaacccattaaacccgtttgtcagccataaagagattgaatcagtgaacaatgcagtgataatgccggcacatggtccagccaagaatatggagtatgacgctgttgcaacaacacactcctgggtccccaaaagaaatcgatccattttgaacacgagccaaagggggatacttgaagatgagcaaatgtatcagaggtgctgcaatttatttgaaaaattcttcccaagtagctcatacagaagaccagttggaatatccagtatggtagaggctatggtttcaagagccagaattgatgcacggattgatttcgaatctggaaggataaagaaagaggaatttgctgagatcatgaagatctgttccaccattgaagacctcagacggcaaaaatag

>H1N1_PB1_CY028049_A/Kentucky/UR06-0401/2007

atggatgtcaatccgacattacttttcttaaaagtgccagcacaaaatgctataagcacaacttttccttatactggtgaccctccttacagccatgggacaggaacagggtacaccatggatacagtcaacaggacacaccagtactcagaaagaggaagatggacaaaaaataccgaaactggagcaccgcaactcaacccaattgatgggccactaccagaagacaatgaaccaagtggctatgcccaaacagattgtgtattagaagcaatggctttccttgaagaatcccatcctggtatctttgaaaactcttgtattgaaacaatggaggttgttcagcaaacaagggtggacaaactgacacaaggcagacagacctatgactggactctaaataggaaccagcctgctgcaacagcattggcaaacactatagaagtattcagatcaaacggcctcatagcaaatgaatctggaaggctaatagacttccttaaagatgtaatggagtcgatggacagagacgaagtagaggtcacaactcattttcaaagaaagaggagagtgagagacaatgtaactaaaaaaatggtgactcaaagaacaataggcaaaaagaaacataaattagacaaaagaagttacctaattagggcattaaccctgaacacaatgaccaaagatgctgagagggggaaactaaaacgcagagcaattgcaaccccaggaatgcaaataagagggtttgtatactttgttgagacactggcaagaagcatatgtgaaaagcttgaacaatcaggattgccagttggaggaaatgagaagaaagcaaagttagcaaatgttgtaaggaagatgatgaccaactcccaggacactgaaatttctttcaccatcactggagataacacaaaatggaacgaaaatcaaaaccctagaatgttcttggccatgatcacatatataaccaaaaatcagcctgaatggttcagaaatattctaagtattgctccaataatgttttcaaacaagatggcgagactaggtaaggggtacatgtttgaaagcaagagtatgaaactgagaactcaaatacctgcagagatgctagccaacatagatttgaaatatttcaatgattcaactaaaaagaaaattgaaaaaatccgaccattattaatagatggaactgcatcattgagtcctggaatgatgatgggcatgttcaatatgttgagcaccgtcttgggcgtctccattctgaatcttgggcaaaagagatacaccaagactacttactggtgggatggtcttcaatcgtctgatgattttgctttgattgtgaatgcacccaactatgcaggaattcaagctggagttgacaggttttatcgaacctgtaagctgctcggaattaatatgagcaaaaagaagtcttacataaacagaacaggtacctttgaattcacgagctttttctatcgttatgggtttgttgccaatttcagcatggagcttcctagttttggggtgtctggggtcaatgaatctgcagacatgagtattggagtcactgtcatcaaaaacaatatgataaacaatgaccttggcccagcaactgctcaaatggcccttcagttatttataaaagattacaggtacacttatcgatgccacagaggtgacacacaaatacaaacccggagatcatttgagataaagaaactatgggaccaaacccgctccaaagctgggctgttggtctctgatggaggccccaatttatataacattagaaatctccatattcctgaagtttgcttgaaatgggagttgatggatgaggattaccaggggcgtttatgcaacccattaaacccgtttgtcagccataaagagattgaatcagtgaacaatgcagtgataatgccagcacatggcccagccaaaaatatggagtatgacgctgttgcaacaacacactcctgggtccccaaaagaaatcgatccattttgaacacgagccaaagggggatacttgaagatgagcaaatgtatcagaggtgctgcaatttatttgaaaaattcttcccaagtagctcatacagaagaccagttggaatatccagtatggtagaggctatggtttcaagagcccgaattgatgcacggattgatttcgaatctggaaggataaagagggaggaatttgctgagatcatgaagatctgttccaccattgaagacctcagacggcaaaaatag

>H1N1_PB1_CY028169_A/Kentucky/UR06-0258/2007

atggatgtcaatccgacattacttttcttaaaagtgccagcacaaaatgctataagcacaacttttccttatactggtgaccctccttacagccatgggacaggaacagggtacaccatggatacagtcaacaggacacaccagtactcagaaagaggaagatggacaaaaaataccgaaactggagcaccgcaactcaacccaattgatgggccactaccagaagacaatgaaccaagtggctatgcccaaacagattgtgtattagaagcaatggctttccttgaagaatcccatcctggtatctttgaaaactcttgtattgaaacaatggaggttgttcagcaaacaagggtggacaaactgacacaaggcagacagacctatgactggactctaaataggaaccagcctgctgcaacagcattggcaaacactatagaagtattcagatcaaacggcctcatagcaaatgaatctggaaggctaatagacttccttaaagatgtaatggagtcgatggacagagacgaagtagaggtcacaactcattttcaaagaaagaggagagtgagagacaatgtaactaaaaaaatggtgactcaaagaacaataggcaaaaagaaacataaattagacaaaagaagttacctaattagggcattaaccctgaacacaatgaccaaagatgctgagagggggaaactaaaacgcagagcaattgcaaccccaggaatgcaaataagagggtttgtatactttgttgagacactggcaagaagcatatgtgaaaagcttgaacaatcaggattgccagttggaggaaatgagaagaaagcaaagttagcaaatgttgtaaggaagatgatgaccaactcccaggacactgaaatttctttcaccatcactggagataacacaaaatggaacgaaaatcaaaaccctagaatgttcttggccatgatcacatatataaccaaaaatcagcctgaatggttcagaaatattctaagtattgctccaataatgttttcaaacaagatggcgagactaggtaaggggtacatgtttgaaagcaagagtatgaaactgagaactcaaatacctgcagagatgctagccaacatagatttgaaatatttcaatgattcaactaaaaagaaaattgaaaaaatccgaccattattaatagatggaactgcatcattgagtcctggaatgatgatgggcatgttcaatatgttgagcaccgtcttgggcgtctccattctgaatcttgggcaaaagagatacaccaagactacttactggtgggatggtcttcaatcgtctgatgattttgctttgattgtgaatgcacccaactatgcaggaattcaagctggagttgacaggttttatcgaacctgtaagctgctcggaattaatatgagcaaaaagaagtcttacataaacagaacaggtacctttgaattcacgagctttttctatcgttatgggtttgttgccaatttcagcatggagcttcctagttttggggtgtctggggtcaatgaatctgcagacatgagtattggagtcactgtcatcaaaaacaatatgataaacaatgaccttggcccagcaactgctcaaatggcccttcagttatttataaaagattacaggtacacttatcgatgccacagaggtgacacacaaatacaaacccggagatcatttgagataaagaaactatgggaccaaacccgctccaaagctgggctgttggtctctgatggaggccccaatttatataacattagaaatctccatattcctgaagtttgcttgaaatgggagttgatggatgaggattaccaggggcgtttatgcaacccattaaacccgtttgtcagccataaagagattgaatcagtgaacaatgcagtgataatgccagcacatggcccagccaaaaatatggagtatgacgctgttgcaacaacacactcctgggtccccaaaagaaatcgatccattttgaacacgagccaaagggggatacttgaagatgagcaaatgtatcagaggtgctgcaatttatttgaaaaattcttcccaagtagctcatacagaagaccagttggaatatccagtatggtagaggctatggtttcaagagcccgaattgatgcacggattgatttcgaatctggaaggataaagagggaggaatttgctgagatcatgaagatctgttccaccattgaagacctcagacggcaaaaatag

>H1N1_PB1_CY028337_A/Texas/UR06-0250/2007

atggatgtcaatccgacattacttttcttaaaagtgccagcacaaaatgctataagcacaacttttccttatactggtgaccctccttacagccatgggacaggaacagggtacaccatggatacagtcaacaggacacaccagtactcagaaagaggaagatggacaaaaaataccgaaactggagcactgcaactcaacccaattgatgggccactaccagaagacaatgaaccaagtggctatgcccaaacagattgtgtattagaagcaatggctttccttgaagagtcccatcctggtatctttgaaaactcttgtattgaaacaatggaggttgttcagcaaacaagggtggacaaactgacacaaggcagacagacctatgactggactctaaataggaaccagcctgctgcaacagcattggcaaacactatagaagtattcagatcaaacggcctcatagcaaatgaatctggaaggctaatagacttccttaaagatgtaatggagtcgatggacagagacgaagtagaggtcacaactcattttcaaagaaagaggagagtaagagacaatgtaactaaaaaaatggtgactcaaagaacaataggcaaaaagaaacataaattagacaaaagaagttacctaattagggcattaaccctgaacacaatgaccaaagatgctgagagggggaaactaaaacgcagagcaattgcaaccccaggaatgcaaataagagggtttgtatactttgttgagacactggcaagaagcatatgtgaaaagcttgaacaatcaggattgccagttggaggaaatgagaagaaagcaaagttagcaaatgttgtaaggaagatgatgaccaactcccaggacactgaaatttctttcaccatcactggagataacacaaaatggaacgaaaatcaaaaccctagaatgttcttggccatgatcacatatataaccaaaaatcagcctgaatggttcagaaatattctaagtattgctccaataatgttttcaaacaagatggcgagactaggtaaggggtacatgtttgaaagcaagagtatgaaactgagaactcaaatacctgcagagatgctagccaacatagatttgaaatatttcaatgattcaactaaaaagaaaattgaaaaaatccgaccattattaatagatggaactgcatcattgagtcctggaatgatgatgggcatgttcaatatgttgagcaccgtcttgggcgtctccattctgaatcttgggcaaaagagatacaccaagactacttactggtgggatggtcttcaatcgtctgatgattttgctttgattgtgaatgcacccaactatgcaggaattcaagctggagttgacaggttttatcgaacctgtaagctgctcggaattaatatgagcaaaaagaagtcttacataaacagaacaggtacctttgaattcacgagctttttctaccgttatgggtttgttgccaatttcagcatggagcttcctagttttggggtgtctggggtcaatgaatctgcagacatgagtattggagtcactgtcatcaaaaacaatatgataaacaatgaccttggcccagcaactgctcaaatggcccttcagttatttataaaagattacaggtacacttatcgatgccacagaggtgacacacaaatacaaacccggagatcatttgagataaagaaactatgggaccaaacccgctccaaagctgggctgttggtctctgatggaggccccaatttatataacattagaaatctccatattcctgaagtttgcttgaaatgggagttgatggatgaggattaccaggggcgtttatgcaacccattaaacccgtttgtcagccataaagagattgaatcagtgaacaatgcagtgataatgccggcacatggcccagccaaaaatatggagtatgacgctgttgcaacaacacactcctgggtccccaaaagaaatcgatccattttgaacacgagccaaagggggatacttgaagatgagcaaatgtatcagaggtgctgcaatttatttgaaaaattcttcccaagtagctcatacagaagaccagttggaatatccagtatggtagaggctatggtttcaagagcccgaattgatgcacggattgatttcgaatctggaaggataaagagggaggaatttgctgagatcatgaagatctgttccaccattgaagacctcagacggcaaaaatag

>H1N1_PB1_CY028401_A/Tennessee/UR06-0277/2007

atggatgtcaatccgacattacttttcttaaaagtgccagcacaaaatgctataagcacaacttttccttatactggtgaccctccttacagccatgggacaggaacagggtacaccatggatacagtcaacaggacacaccagtactcagaaagaggaagatggacaaaaaataccgaaactggagcaccgcaactcaacccaattgatgggccactaccagaagacaatgaaccaagtggctatgcccaaacagattgtgtattagaagcaatggctttccttgaagaatcccatcctggtatctttgaaaactcttgtattgaaacaatggaggttgttcagcaaacaagggtggacaaactgacacaaggcagacagacctatgactggactctaaataggaaccagcctgctgccacagcattggcaaacactatagaagtattcagatcaaacggcctcatagcaaatgaatctgggaggctaatagacttccttaaagatgtaatggagtcgatggacagagacgaagtagaggtcacaactcattttcaaagaaagaggagagtgagagacaatgtaactaaaaaaatggtgacccaaagaacaataggcaaaaagaaacataaattagacaaaagaagttacctaattagggcattaaccctgaacacaatgaccaaagatgctgaaagggggaaactaaaacgcagagcaattgcaaccccaggaatgcaaataagggggtttgtatactttgttgagacactggcaagaagcatatgtgaaaagcttgaacaatcaggattgccagttggaggaaatgagaagaaagcaaagttagcaaatgttgtaaggaagatgatgaccaactcccaggacactgaaatttctttcaccattactggagataacacaaaatggaacgaaaatcaaaaccctagaatgttcttggccatgatcacatatataaccaaaaatcagcctgaatggttcagaaatattctaagtattgctccaataatgttttcaaacaagatggcgagactaggtaaagggtacatgtttgaaagcaagagtatgaaactgagaactcaaatacctgcagaaatgctagccaacatagatttgaaatatttcaatgattcaactaaaaagaaaattgaaaaaatccgaccattattaatagatggaactgcatcattgagtcctggaatgatgatgggcatgttcaatatgttgagcaccgtcttgggcgtctccattctgaatcttgggcaaaagagatacaccaagactacttactggtgggatggtcttcaatcgtctgatgattttgctttgattgtgaatgcacccaactatgcaggaattcaagctggagttgacaggttttatcgaacctgtaagctgctcggaattaatatgagcaaaaagaagtcttacataaacagaacaggtacctttgaattcacgagctttttctatcgttatgggtttgttgctaacttcagcatggagcttcctagttttggggtgtctggggtcaatgaatctgcagacatgagtattggagtcactgtcatcaaaaacaatatgataaacaatgaccttggcccagcaactgctcaaatggcccttcagttatttataaaagattacaggtacacttatcggtgccacagaggtgacacacaaatacaaacccggagatcatttgagataaagaaactatgggaccaaacccgctccaaagctgggctgttggtctctgatggaggccccaatttatataacattagaaatctccatattcctgaagtttgcttaaaatgggagttgatggatgaggaataccaggggcgtttatgcaacccattaaacccgtttgtcagccataaagagattgaatcagtgaacaatgcagtgataatgccggcacatggtccagccaaaaatatggagtatgacgctgttgcaacaacacactcctgggtccccaaaagaaatcgatccattttgaacacgagccaaagggggatacttgaagatgagcaaatgtatcagaggtgctgcaatttatttgaaaaattcttcccaagtagctcatacagaagaccagttggaatatccagtatggtagaggctatggtttcaagagcccgaattgatgcacggattgatttcgaatctggaaggataaagaaagaggaatttgctgagatcatgaagatctgttccaccattgaagacctcagacggcaaaaatag

>H1N1_PB1_CY028465_A/California/UR06-0442/2007

atggatgtcaatccgacattacttttcttaaaagtgccagcacaaaatgctataagcacaacttttccttatactggtgaccctccttacagccatgggacaggaacagggtacaccatggatacagtcaacaggacacaccagtactcagaaagaggaagatggacaaaaaataccgaaactggagcaccgcaactcaacccaattgatgggccactaccagaagacaatgaaccgagtggctatgcccaaacagattgtgtattagaagcaatggctttccttgaagaatcccatcctggtatctttgaaaactcttgtattgagacaatggaggttgttcagcaaacaagggtggacaaactgacacagggcagacagacctatgactggactctaaataggaaccagcctgctgccacagcattggcaaacactatagaagtattcagatcaaacggcctcatagcaaatgaatctgggaggctaatagacttccttaaagatgtaatggagtcgatggacatagacgaagtagaggtcacaactcattttcaaaggaagaggagagtgagagacagtgtaactaaaaaaatggtgacccaaagaacaataggcaaaaagaaacataaattagacaaaagaagttacctaattagggcattaaccctgaacacaatgaccaaagatgctgagagagggaaactaaaacgcagagcaattgcaaccccaggaatgcaaataagggggtttgtatactttgttgagacactggcaagaagcatatgtgaaaagcttgaacaatcaggattgccagttggaggaaatgagaagaaagcaaagttagcaaatgttgtaaggaagatgatgaccaactcccaggacactgaaatttctttcaccatcactggagataacacaaaatggaacgaaaatcaaaaccctagaatgttcttggccatgatcacatatataaccaaaaatcagcctgaatggttcagaaatattctaagtattgccccaataatgttttcaaacaagatggcgagactaggtaaggggtacatgtttgaaagcaagagtatgaaactgagaactcaaatacctgcagagatgctagccaacatagatttgaaatatttcaatgattcaactaaaaagaaaattgaaaaaatccgaccattattaatagatggaactgcatcattgagtcctggaatgatgatgggcatgttcaatatgttgagcaccgtcttgggcgtctccattctgaatcttgggcaaaagagatacaccaagactacttactggtgggatggtcttcaatcgtctgatgattttgctttgattgtgaatgcacccaactatgcaggaattcaagctggagttgacaggttttatcgaacctgtaagctgctcggaattaatatgagcaaaaagaagtcttacataaacagaacaggtacctttgaattcacgagctttttctatcgttatgggtttgttgccaatttcagcatggagcttcctagttttggggtgtctggggtcaatgaatctgcagacatgagtattggagtcactgtcatcaaaaacaatatgataaacaatgaccttggcccagcaactgctcaaatggcccttcagttatttataaaagattacaggtacacttatcgatgccacagaggtgacacacaaatacaaacccggagatcatttgagataaagaaactatgggaccaaacccgctctaaagctgggctgttggtctctgatggaggccccaatttatataacattagaaatctccatattcctgaagtttgcttgaaatgggagttgatggatgaggattatcaggggcgtttatgcaacccattaaacccgtttgtcagccataaagagattgaatcagtaaacaatgcagtgataatgccggcacatggtccagccaaaaatatggagtatgacgctgttgcaacaacacactcctgggtccccaaaagaaatcgatccattttgaacacgagccaacgggggatacttgaagatgagcaaatgtatcagaggtgctgcaatttatttgaaaaattcttcccaagtagctcatacagaagaccagttggaatatccagtatggtagaggctatggtttcaagagcccgaattgatgcacggattgatttcgaatctggaaggataaagaaagaggaatttgctgagatcatgaagatctgttccaccattgaagacctcagacggcaaaaatag

>H1N1_PB1_CY031138_A/Virginia/UR06-0228/2007

atggatgtcaatccgacattacttttcttaaaagtgccagcacaaaatgctataagcacaacttttccttatactggtgaccctccttacagccatgggacaggaacagggtacaccatggatacagtcaacaggacacaccagtactcagaaagaggaagatggacaaaaaataccgaaactggagcaccgcaactcaacccaattgatgggccactaccagaagacaatgaaccaagtggctatgcccaaacagattgtgtattagaagcaatggctttccttgaagaatcccatcctggtatctttgaaaactcttgtattgaaacaatggaggttgttcagcaaacaagggtggacaaactgacacaaggcagacagacctatgactggactctaaataggaaccagcctgctgcaacagcattggcaaacactatagaagtattcagatcaaacggcctcatagcaaatgaatctggaaggctaatagacttccttaaagatgtaatggagtcgatggacagagacgaagtagaggtcacaactcattttcaaagaaagaggagagtgagagacaatgtaactaaaaaaatggtgactcaaagaacaataggcaaaaagaaacataaattagacaaaagaagttacctaattagggcattaaccctgaacacaatgaccaaagatgctgagagggggaaactaaaacgcagagcaattgcaaccccaggaatgcaaataagagggtttgtatactttgttgagacactggcaagaagcatatgtgaaaagcttgaacaatcaggattgccagttggaggaaatgagaagaaagcaaagttagcaaatgttgtaaggaagatgatgaccaactcccaggacactgaaatttctttcaccatcactggagataacacaaaatggaacgaaaatcaaaaccctagaatgttcttggccatgatcacatatataaccaaaaatcagcctgaatggttcagaaatattctaagtattgctccaataatgttttcaaacaagatggcgagactaggtaaggggtacatgtttgaaagcaagagtatgaaactgagaactcaaatacctgcagagatgctagccaacatagatttgaaatatttcaatgattcaactaaaaagaaaattgaaaaaatccgaccattattaatagatggaacggcatcattgagtcctggaatgatgatgggcatgttcaatatgttgagcaccgtcttgggcgtctccattctgaatcttgggcaaaagagatacaccaagactacttactggtgggatggtcttcaatcgtctgatgattttgctttgattgtgaatgcacccaactatgcaggaattcaagctggagttgacaggttttatcgaacctgtaagctgctcggaattaatatgagcaaaaagaagtcttacataaacagaacaggtacctttgaattcacgagctttttctatcgttatgggtttgttgccaatttcagcatggagcttcctagttttggggtgtctggggtcaatgaatctgcagacatgagtattggagtcactgtcatcaaaaacaatatgataaacaatgaccttggcccagcaactgctcaaatggcccttcagttatttataaaagattacaggtacacttatcgatgccacagaggtgacacacaaatacaaacccggagatcatttgagataaagaaactatgggaccaaacccgctccaaagctgggctgttggtctctgatggaggccccaatttatataacattagaaatctccatattcctgaagtttgcttgaaatgggagttgatggatgaggattaccaggggcgtttatgcaacccattaaacccgtttgtcagccataaagagattgaatcagtgaacaatgcagtgataatgccggcacatggcccagccaaaaatatggagtatgacgctgttgcaacaacacactcctgggtccccaaaagaaatcgatccattttgaacacgagccaaagggggatacttgaagatgagcaaatgtatcagaggtgctgcaatttatttgaaaaattcttcccaagtagctcatacagaagaccagttggaatatccagtatggtagaggctatggtttcaagagcccgaattgatgcacggattgatttcgaatctggaaggataaagagggaggaatttgctgagatcatgaagatctgttccaccattgaagacctcagacggcaaaaatag

>H1N1_PB1_CY038925_A/Managua/107.01/2008

atggatgtcaatccgacattacttttcttaaaagtgccagcacaaaatgctataagcacaacttttccttatactggtgaccctccttacagccatgggacaggaacagggtacaccatggatacagtcaacaggacacaccagtactcagaaagaggaagatggacaaaaaataccgaaactggagcaccgcaacttaacccaattgatggtccactaccggaagacaatgaaccaagtggctatgcccaaacagattgtgtattagaagcaatggctttccttgaagaatcccatcccggtatctttgaaaactcttgtattgaaacaatggaggttgttcagcaaacaagggtggacaaactgacacaaggcagacagacctatgactggactctaaataggaaccagcctgctgccacagcattggcaaacactatagaagtattcagatcaaacggcctcatagcaaatgaatctgggaggctaatagacttccttaaagatgtaatggagtcgatggacagaggcgaagtagaggtcacaactcattttcaaagaaagaggagagtgagagacaatgtaactaaaaaaatggtgacccaaagaacaataggcaaaaagaaacataaattagacaaaagaagttacctaattagggcattaaccctgaacacaatgaccaaagatgctgagagggggaaactaaaacgcagagcaattgcaaccccaggaatgcaaataagggggtttgtatactttgttgagacactggcaagaagcatatgtgaaaagcttgaacaatcaggattgccagttggaggaaatgagaagaaagcaaagttagcaaatgttgtaaggaagatgatgaccaactcccaggacactgaaatttctttcaccataaccggagataacacaaaatggaacgaaaatcaaaaccctagaatgttcttggccatgatcacatatataaccaaaaatcagcctgaatggttcagaaatattctaagtattgctccaataatgttttcaaacaaaatggcgagactaggtaaggggtacatgtttgaaagcaagagtatgaaactgagaactcaaatacctgcagagatgctagccaacatagatttgaaatatttcaatgattcaactaaaaagaaaattgaaaaaatccgaccactattaatagatggaactgcatcattgagtcctggaatgatgatgggcatgttcaatatgttgagcaccgtcttgggcgtctccattctgaatcttgggcaaaagagatacaccaagactacttactggtgggatggtcttcaatcgtctgatgattttgctttgattgtgaatgcacccaactatgcaggaattcaagctggagttgacaggttttatcgaacctgtaagctgctcggaattaatatgagcaaaaagaagtcttacataaacagaacaggtacctttgaattcacgagctttttctatcgttatgggtttgttgccaatttcagcatggagcttcctagttttggggtgtctggggtcaatgaatctgcagacatgagtattggggtcactgtcatcaaaaacaatatgataaacaatgaccttggcccagcaactgctcaaatggcccttcagttatttataaaagattacaggtacacttatcgatgccaccgaggtgacacacaaatacaaacccggagatcatttgagataaagaaactatgggaccaaacccgctcaaaagctgggctgttggtctctgatggaggccccaatttatataacattagaaatctccatattcctgaagtttgcttgaaatgggagttgatggatgaggattaccaggggcgtttatgcaacccattaaacccgtttgtcagccataaagagattgaatcagtgaacaatgcagtgataatgccggcacatggtccagccaaaaatatggagtatgacgctgttgcaacaacacactcctgggtccccaaaagaaatcgatccattttgaacacgagccaaagggggatacttgaagatgagcaaatgtatcagaggtgctgcaatttatttgaaaaattcttcccaagtagctcatacagaagaccagttggaatatccagtatggtagaggctatggtctcaagagcccgaattgatgcacggattgatttcgaatctggaaggataaagaaagaggaatttgctgagatcatgaagatctgttccaccattgaagacctcagacggcaaaaatga

>H1N1_PB1_CY039133_A/Managua/521.01/2008

atggatgtcaatccgacattacttttcttaaaagtgccagcacaaaatgctataagcacaacttttccttatactggtgaccctccttacagccatgggacaggaacagggtacaccatggatacagtcaacaggacacaccattactcagaaagaggaagatggacaaaaaataccgaaacaggagcaccgcaacttaacccaattgatggtcccctaccggaagacaatgaaccaagtggctatgcccaaacagattgtgtattagaagcaatggctttccttgaagaatcccatcctggtatctttgaaaactcttgtattgaaacaatggaggttgttcagcaaacaagggtggacaaactgacacaaggcagacagacctatgactggactctaaataggaaccagcctgctgccacagcattggcaaacactatagaagtattcagatcaaacggcctcatagcaaatgaatctgggaggctaatagacttccttaaagatgtaatggagtcgatggacagaggcgaagtagaggtcacaactcattttcaaagaaagaggagagtgagagacaatgtaactaaaaaaatggtgacccaaagaacaataggcaaaaagaaacataaattagacaaaagaagttacctaattagggcattaaccctgaacacaatgaccaaagatgctgagagggggaaactaaaacgcagagcaattgcaaccccaggaatgcaaataagggggtttgtatactttgttgagacactggcaagaagcatatgtgaaaagcttgaacaatcaggattgccagttggaggaaatgagaagaaagcaaagttagcaaatgttgtaaggaagatgatgaccaactcccaggacactgaaatttctttcaccataaccggagataacacaaaatggaacgaaaatcaaaaccctagaatgttcttggccatgatcacatatataaccaaaaatcagcctgaatggttcagaaatattctaagtattgctccaataatgttttcaaacaagatggcgaggctaggtaaggggtacatgtttgaaagcaagagtatgaaactgagaactcaaatacctgcagagatgctagccaacatagatttgaaatatttcaatgattcaactaaaaagaaaattgaaaaaatccgaccattattaatagatggaactgcatcattgagtcctggaatgatgatgggcatgttcaatatgttgagcaccgtcttgggcgtctccattctgaatcttgggcaaaagagatacaccaagactacttactggtgggatggtcttcaatcgtctgatgattttgctttgattgtgaatgcacccaactatgcaggaattcaagctggagttgacaggttttatcgaacctgtaagctgctcggaattaatatgagcaaaaagaagtcttacataaacagaacaggtacctttgaattcacgagctttttctatcgttatgggtttgttgccaatttcagcatggagcttcctagttttggggtgtctggggtcaatgaatctgcagacatgagtattggggtcactgtcatcaaaaacaatatgataaacaatgaccttggcccagcaactgctcaaatggcccttcagttatttataaaagattacaggtacacttatcgatgccaccgaggtgacacacaaatacaaacccggagatcatttgagataaagaaactatgggaccaaacccgctcaaaagctgggctgttggtctctgatggaggccccaatttatataacattagaaatctccatattcctgaagtttgcttgaaatgggagttgatggatgaggattaccaggggcgtttatgcaacccattaaacccgtttgtcagccataaagagattgaatcagtgaacaatgcagtgataatgccggcacatggtccagccaaaaatatggagtatgacgctgttgcaacaacacactcctgggtccccaaaagaaatcgatccattttgaacacgagccaaagggggatacttgaagatgagcaaatgtatcagaggtgctgcaatttatttgaaaaattcttcccaagtagctcatacagaagaccagttggaatatccagtatggtagaggctatggtctcaagagcccgaattgatgcacggattgatttcgaatctggaaggataaagaaagaggaatttgctgagatcatgaagatctgttccaccattgaagacctcagacggcaaaaatga

>H1N1_PB1_FJ966080_A/California/04/2009

atggatgtcaatccgactctacttttcctaaaaattccagcgcaaaatgccataagcaccacattcccttatactggagatcctccatacagccatggaacaggaacaggatacaccatggacacagtaaacagaacacaccaatactcagaaaagggaaagtggacgacaaacacagagactggtgcaccccagctcaacccgattgatggaccactacctgaggataatgaaccaagtgggtatgcacaaacagactgtgttctagaggctatggctttccttgaagaatcccacccaggaatatttgagaattcatgccttgaaacaatggaagttgttcaacaaacaagggtagataaactaactcaaggtcgccagacttatgattggacattaaacagaaatcaaccggcagcaactgcattggccaacaccatagaagtctttagatcgaatggcctaacagctaatgagtcaggaaggctaatagatttcttaaaggatgtaatggaatcaatgaacaaagaggaaatagagataacaacccactttcaaagaaaaaggagagtaagagacaacatgaccaagaagatggtcacgcaaagaacaatagggaagaaaaaacaaagactgaataagagaggctatctaataagagcactgacattaaatacgatgaccaaagatgcagagagaggcaagttaaaaagaagggctatcgcaacacctgggatgcagattagaggtttcgtatactttgttgaaactttagctaggagcatttgcgaaaagcttgaacagtctgggctcccagtagggggcaatgaaaagaaggccaaactggcaaatgttgtgagaaagatgatgactaattcacaagacacagagatttctttcacaatcactggggacaacactaagtggaatgaaaatcaaaatcctcgaatgttcctggcgatgattacatatatcaccagaaatcaacccgagtggttcagaaacatcctgagcatggcacccataatgttctcaaacaaaatggcaagactagggaaagggtacatgttcgagagtaaaagaatgaagattcgaacacaaataccagcagaaatgctagcaagcattgacctgaagtacttcaatgaatcaacaaagaagaaaattgagaaaataaggcctcttctaatagatggcacagcatcactgagtcctgggatgatgatgggcatgttcaacatgctaagtacggtcttgggagtctcgatactgaatcttggacaaaagaaatacaccaagacaatatactggtgggatgggctccaatcatccgacgattttgctctcatagtgaatgcaccaaaccatgagggaatacaagcaggagtggacagattctacaggacctgcaagttagtgggaatcaacatgagcaaaaagaagtcctatataaataagacagggacatttgaattcacaagctttttttatcgctatggatttgtggctaattttagcatggagctacccagctttggagtgtctggagtaaatgaatcagctgacatgagtattggagtaacagtgataaagaacaacatgataaacaatgaccttggacctgcaacggcccagatggctcttcaattgttcatcaaagactacagatacacatataggtgccataggggagacacacaaattcagacgagaagatcatttgagttaaagaagctgtgggatcaaacccaatcaaaagtagggctattagtatcagatggaggaccaaacttatacaatatacggaatcttcacattcctgaagtctgcttaaaatgggagctaatggatgatgattatcggggaagactttgtaatcccctgaatccctttgtcagtcataaagagattgattctgtaaacaatgctgtggtaatgccagcccatggtccagccaaaagcatggaatatgatgccgttgcaactacacattcctggattcccaagaggaatcgttctattctcaacacaagccaaaggggaattcttgaggatgaacagatgtaccagaagtgctgcaatctattcgagaaatttttccctagcagttcatataggagaccggttggaatttctagcatggtggaggccatggtgtctagggcccggattgatgccagggtcgacttcgagtctggacggatcaagaaagaagagttctctgagatcatgaagatctgttccaccattgaagaactcagacggcaaaaataa

>H1N1_PB1_FJ998226_A/Mexico/InDRE4487/2009

atggatgtcaatccgactctacttttcctaaaaattccagcgcaaaatgccataagcaccacattcccttatactggagatcctccatacagccatggaacaggaacaggatacaccatggacacagtaaacagaacacaccaatactcagaaaagggaaagtggacgacaaacacagagactggtgcaccccagctcaacccgattgatggaccactacctgaggataatgaaccaagtgggtatgcacaaacagactgtgttctagaggctatggctttccttgaagaatcccacccaggaatatttgagaattcatgccttgaaacaatggaagttgttcaacaaacaagggtagataaactaactcaaggtcgccagacttatgattggacattaaacagaaatcaaccggcagcaactgcattggccaacaccatagaagtctttagatcgaatggcctaacagctaatgagtcaggaaggctaatagatttcttaaaggatgtaatggaatcaatgaacaaagaggaaatagagataacaacccactttcaaagaaaaaggagagtaagagacaacatgaccaagaagatggtcacgcaaagaacaatagggaagaaaaaacaaagactgaataagagaggctatctaataagagcactgacattaaatacgatgaccaaagatgcagagagaggcaagttaaaaagaagggctatcgcaacacctgggatgcagattagaggtttcgtatactttgttgaaactttagctaggagcatttgcgaaaagcttgaacagtctgggctcccagtagggggcaatgaaaagaaggccaaactggcaaatgttgtgagaaagatgatgactaattcacaagacacagagatttctttcacaatcactggggacaacactaagtggaatgaaaatcaaaatcctcgaatgttcctggcgatgattacatatatcaccagaaatcaacccgagtggttcagaaacatcctgagcatggcacccataatgttctcaaacaaaatggcaagactagggaaagggtacatgttcgagagtaaaagaatgaagattcgaacacaaataccagcagaaatgctagcaagcattgacctgaagtacttcaatgaatcaacaaagaagaaaattgagaaaataaggcctcttctaatagatggcacagcatcactgagtcctgggatgatgatgggcatgttcaacatgctaagtacggtcttgggagtctcgatactgaatcttggacaaaagaaatacaccaagacaatatactggtgggatgggctccaatcatccgacgattttgctctcatagtgaatgcaccaaaccatgagggaatacaagcaggagtggacagattctacaggacctgcaagttagtgggaatcaacatgagcaaaaagaagtcctatataaataagacagggacatttgaattcacaagctttttttatcgctatggatttgtggctaattttagcatggagctacccagctttggagtgtctggagtaaatgaatcagctgacatgagtattggagtaacagtgataaagaacaacatgataaacaatgaccttggacctgcaacggcccagatggctcttcaattgttcatcaaagactacagatacacatataggtgccataggggagacacacaaattcagacgagaagatcatttgagttaaagaagctgtgggatcaaacccaatcaaaggtagggctattagtatcagatggaggaccaaacttatacaatatacggaatcttcacattcctgaagtctgcttaaaatgggagctaatggatgatgattatcggggaagactttgtaatcccctgaatccctttgtcagtcataaagagattgattctgtaaacaatgctgtggtaatgccagcccatggtccagccaaaagcatggaatatgatgccgttgcaactacacattcctggattcccaagaggaatcgttctattctcaacacaagccaaaggggaattcttgaggatgaacagatgtaccagaagtgctgcaatctattcgagaaatttttccctagcagttcatataggagaccggttggaatttctagcatggtggaggccatggtgtctagggcccggattgatgccagggtcgacttcgagtctggacggatcaagaaagaagagttctctgagatcatgaagatctgttccaccattgaagaactcagacggcaaaaataa

>H1N1_PB1_GQ117069_A/Minnesota/02/2009

atggatgtcaatccgactctacttttcctaaaaattccagcgcaaaatgccataagcaccacattcccttatactggagatcctccatacagccatggaacaggaacaggatacaccatggacacagtaaacagaacacaccaatactcagaaaagggaaagtggacgacaaacacagagactggtgcaccccagctcaacccgattgatggaccactacctgaggataatgaaccaagtgggtatgcacaaacagactgtgttctagaggctatggctttccttgaagaatcccacccaggaatatttgagaattcatgccttgaaacaatggaagttgttcaacaaacaagggtagataaactaactcaaggtcgccagacttatgattggacattaaacagaaatcaaccggcagcaactgcattggccaacaccatagaagtctttagatcgaatggcctaacagctaatgagtcaggaaggctaatagatttcttaaaggatgtaatggaatcaatgaacaaagaggaaatagagataacaacccactttcaaagaaaaaggagagtaagagacaacatgaccaagaagatggtcacgcaaagaacaatagggaagaaaaaacaaagactgaataagagaggctatctaataagagcactgacattaaatacgatgaccaaagatgcagagagaggcaagttaaaaagaagggctatcgcaacacctgggatgcagattagaggtttcgtatactttgttgaaactttagctaggagcatttgcgaaaagcttgaacagtctgggctcccagtagggggcaatgaaaagaaggccaaactggcaaatgttgtgagaaagatgatgactaattcacaagacacagagatttctttcacaatcactggggacaacactaagtggaatgaaaatcaaaatcctcgaatgttcctggcgatgattacatatatcaccagaaatcaacccgagtggttcagaaacatcctgagcatggcacccataatgttctcaaacaaaatggcaagactagggaaagggtacatgttcgagagtaaaagaatgaagattcgaacacaaataccagcagaaatgctagcaagcattgacctgaagtacttcaatgaatcaacaaagaagaaaattgagaaaataargcctcttctaatagatggcacagcatcactgagtcctgggatgatgatgggcatgttcaacatgctaagtacggtcttgggagtctcgatactgaatcttggacaaaagaaatacaccaagacaatatactggtgggatgggctccaatcatccgacgattttgctctcatagtgaatgcaccaaaccatgagggaatacaagcaggagtggacagattctacaggacctgcaagttagtgggaatcaacatgagcaaaaagaagtcctatataaataagacagggacatttgaattcacaagctttttttatcgctatggatttgtggctaattttagcatggagctacccagctttggagtgtctggagtaaatgaatcagctgacatgagtattggagtaacagtgataaagaacaacatgataaacaatgaccttggacctgcaacggcccagatggctcttcaattgttcatcaaagactacagatacacatataggtgccataggggagacacacaaattcagacgagaagatcatttgagttaaagaagctgtgggatcaaacccaatcaaaggtagggctattagtatcagatggaggaccaaacttatacaatatacggaatcttcacattcctgaagtctgcttaaaatgggagctaatggatgatgattatcggggaagactttgtaatcccctgaatccctttgtcagtcataaagagattgattctgtaaacaatgctgtggtaatgccagcccatggtccagccaaaagcatggaatatgatgccgttgcaactacacattcttggattcccaagaggaatcgttctattctcaacacaagccaaaggggaattcttgaggatgaacagatgtaccagaagtgctgcaatctattcgagaaatttttccctagcagttcatataggagaccggttggaatttctagcatggtggaggccatggtgtctagggcccggattgatgccagggtcgacttcgagtctggacggatcaagaaagaagagttctctgagatcatgaagatctgttccaccattgaagaactcagacggcaaaaataa

>H1N1_PB1_GQ117093_A/Indiana/09/2009

atggatgtcaatccgactctacttttcctaaaaattccagcgcaaaatgccataagcaccacattcccttatactggagatcctccatacagccatggaacaggaacaggatacaccatggacacagtaaacagaacacaccaatactcagaaaagggaaagtggacgacaaacacagagactggtgcaccccagctcaacccgattgatggaccactacctgaggataatgaaccaagtgggtatgcacaaacagactgtgttctagaggctatggctttccttgaagaatcccacccaggaatatttgagaattcatgccttgaaacaatggaagttgttcaacaaacaagggtagataaactaactcaaggtcgccagacttatgattggacattaaacagaaatcaaccggcagcaactgcattggccaacaccatagaagtctttagatcgaatggcctaacagctaatgagtcaggaaggctaatagatttcttaaaggatgtaatggaatcaatgaacaaagaggaaatagagataacaacccactttcaaagaaaaaggagagtaagagacaacatgaccaagaagatggtcacacaaagaacaatagggaagaaaaaacaaagactgaataagagaggctatctaataagagcactgacattaaatacgatgaccaaagatgcagagagaggcaagttaaaaagaagggctatcgcaacacctgggatgcagattagaggtttcgtatactttgttgaaactttagctaggagcatttgcgaaaagcttgaacagtctgggctcccagtagggggcaatgaaaagaaggccaaactggcaaatgttgtgagaaagatgatgactaattcacaagacacagagatttctttcacaatcactggggacaacactaagtggaatgaaaatcaaaatcctcgaatgttcctggcgatgattacatatatcaccagaaatcaacccgagtggttcagaaacatcctgagcatggcacccataatgttctcaaacaaaatggcaagactagggaaagggtacatgttcgagagtaaaagaatgaagattcgaacacaaataccagcagaaatgctagcaagcattgacctgaagtacttcaatgaatcaacaaagaagaaaattgagaaaataaggcctcttctaatagatggcacagcatcactgagtcctgggatgatgatgggcatgttcaacatgctaagtacggtcttgggagtctcgatactgaatcttggacaaaagaaatacaccaagacaatatactggtgggatgggctccaatcatccgacgattttgctctcatagtgaatgcaccaaaccatgagggaatacaagcaggagtagacagattctacaggacctgcaagttagtgggaatcaacatgagcaaaaagaagtcctatataaataagacagggacatttgaattcacaagctttttttatcgctatggatttgtggctaattttagcatggagctacccagctttggagtgtctggagtaaatgaatcagctgacatgagtattggagtaacagtgataaagaacaacatgataaacaatgaccttggacctgcaacggcccagatggctcttcagttgttcatcaaagactacagatacacatataggtgccataggggagacacacaaattcagacgagaagatcatttgagttaaagaagctgtgggatcaaacccaatcaaaggtagggctattagtatcagatggaggaccaaacttatacaatatacggaatcttcacattcctgaagtctgcttaaaatgggagctaatggatgatgattatcggggaagactttgtaatcccctgaatccctttgtcagtcataaagagattgattctgtaaacaatgctgtggtaatgccagcccatggtccagccaaaagcatggaatatgatgccgttgcaactacacattcttggattcccaagaggaatcgttctattctcaacacaagccaaaggggaattcttgaggatgaacagatgtaccagaagtgctgcaatctattcgagaaatttttccctagcagttcatataggagaccggttggaatttctagcatggtggaggccatggtgtctagggcccggattgatgccagggtcgacttcgagtctggacggatcaagaaagaagagttctctgagatcatgaagatctgttccaccattgaagaactcagacggcaaaaataa

>H1N1_PB1_CY039997_A/New Jersey/8/1976

atggacgtcaatccgactttgcttttcttgaaagtgccagcacaaaatgctataagcacaacgtttccttacactggagaccccccttacagccatgggacaggaacaggatacaccatggatactgtgaacagaacacatcagtactcagaaagaggaaaatggacaataaacaccgaaactggggcaccacaactaaatccaattgatggaccgctgccagaggacaatgaaccaactggttatgctcaaacagactgcgtattggaagcaatggcgttcctcgagaaatcccatcccggcatttttgagaactcatgtcttgaaacgatggaagttgttcagcaaacacgggtggacaaactaacgcaaggtcggcaaactttcgattggactctgaataggaaccagcctgctgcaacagcattggccaacacaatagaagtgttcagattgaacggcctcacggccagtgaatctggaagactcatagacttcctaaaggatgttatggaatcaatggacaaagaagaaatggaaatcgtaactcatttccagagaaaaaggagagtgagggacaatatgactaagaaaatggtgacgcaaagaacaataggtaaaaagaaacagaagttaaataaaagaagctatctaattagagcattgaccttgaacacaatgacaaaagatgctgaaagaggaaaattgaaaagaagagcaattgccaccccagggatgcaaatacggggattcgtatatttcgttgagacactagcgagaagtatttgtgaaaaacttgaacaatcaggactgccagttggaggcaatgaaaagaaagccaaattggcaaatgttgtacgaaaaatgatgaccaattctcaggacactgaactctcctttactatcactggagataataccaaatggaatgaaaatcagaatcctcgaatgtttttagctatgattacatacataaccagaaatcagcccgaatggttcagaaatgttctaagtgttgctccaataatgttctcgaacaagatggctagattgggtaaaggatacatgtttgagagtaaaaacatgaagcttaggactcagatacctgcagagatgctatcaggcattgacctgaggtattttaatgattcaactaggaaaaaaattgaaaaaatccgaccgctcttaatagagggagctgcttctttaagccctggaatgatgatgggtatgtttaacatgttaagcactgtgttgggtgtttccattcttaaccttggacaaaaggagtacacgaagactgcttactggtgggacggtctccaatcgtctgatgattttgctctgattgtgaacgcacccaattatgaagggattcaagcaggagttgacaggttttatcgaacctgtaagctactcggaattaatatgagcaaaaagaaatcttacataaatagaacaggcacttttgaattcacaagctttttctatcgttatggatttgttgccaatttcagcatggagcttcctagttttggagtgtctggaattaatgagtctgcagacatgagtattggagttactgtcatcaaaaacaacatgataaacaatgatcttggaccagcaacagcacaaatggctctccagttgttcatcaaagattaccgatacacgtatcgctgccacaggggtgatactcaaatacaaactcgaaggtcatttgaaataaagaaattgtgggatcaaacccgttctaaggctggattgttggtttccgatggaggccccaatttatataatatcaggaatctccacattcctgaagtctgcttgaagtgggaattaatggataaagattaccaggggcgattgtgcaatccgctaaacccatttgtcagccataaagagattgaatcagtgaataatgcagtagttatgccatcacatggtccagccaaagccatggaatacgacgctgttgccacaacgcactcctgggttcctaaaagaaatcgatccatcttgaacacaagccagagaggaatacttgaagacgagcaaatgtaccaaaaatgctgtaacctatttgaaaagttcttccctagcagctcatataggagaccagttgggatttccagtatggtggaggccatggtttccagggcccgaatcgatgcacgaattgattttgaatctggaaggataaagaaagaggatttcgctgagatcatgaagatctgttccaccattgaagatctcagacggcaaaagtag

>H1N1_PB1_GQ160544_A/Ohio/07/2009

atggatgtcaatccgactctacttttcctaaaaattccagcgcaaaatgccataagcaccacattcccttatactggagatcctccatacagccatggaacaggaacaggatacaccatggacacagtaaacagaacacaccaatactcagaaaagggaaagtggacgacaaacacagagactggtgcaccccagctcaacccgattgatggaccactacctgaggataatgaaccaagtgggtatgcacaaacagactgtgttctagaggctatggctttccttgaagaatcccacccaggaatatttgagaattcatgccttgaaacaatggaagttgttcaacaaacaagggtagataaactaactcaaggtcgccagacttatgattggacattaaacagaaatcaaccggcagcaactgcattggccaacaccatagaagtctttagatcgaatggcctaacagctaatgagtcaggaaggctaatagatttcttaaaggatgtaatggaatcaatgaacaaagaggaaatagagataacaacccactttcaaagaaaaaggagagtaagagacaacatgaccaagaagatggtcacgcaaagaacaatagggaagaaaaaacaaagactgaataagagaggctatctaataagagcactgacattaaatacgatgaccaaagatgcagagagaggcaagttaaaaagaagggctatcgcaacacctgggatgcagattagaggtttcgtatactttgttgaaactttagctaggagcatttgcgaaaagcttgaacagtctgggctcccagtagggggcaatgaaaagaaggccaaactggcaaatgttgtgagaaagatgatgactaattcacaagacacagagatttctttcacaatcactggggacaacactaagtggaatgaaaatcaaaatcctcgaatgttcctggcgatgattacatatatcaccagaaatcaacccgagtggttcagaaacatcctgagcatggcacccataatgttctcaaacaaaatggcaagactagggaaagggtacatgttcgagagtaaaagaatgaagattcgaacacaaataccagcagaaatgctagcaagcattgacctgaagtacttcaatgaatcaacaaagaaaaaaattgagaaaataaggcctcttctaatagatggcacagcatcactgagtcctgggatgatgatgggcatgttcaacatgctaagtacggtcttgggagtctcgatactgaatcttggacaaaagaaatacaccaagacaatatactggtgggatgggctccaatcatccgacgattttgctctcatagtgaatgcaccaaaccatgagggaatacaagcaggagtggacagattctacaggacctgcaagttagtgggaatcaacatgagcaaaaagaagtcctatataaataagacagggacatttgaattcacaagctttttttatcgctatggatttgtggctaattttagcatggagctacccagctttggagtgtctggagtaaatgaatcagctgacatgagtattggagtaacagtgataaagaacaacatgataaacaatgaccttggacctgcaacggcccagatggctcttcaattgttcatcaaagactacagatacacatataggtgccataggggagacacacaaattcagacgagaagatcatttgagttaaagaagctgtgggatcaaacccaatcaaaggtagggctattagtatcagatggaggaccaaacttatacaatatacggaatcttcacattcctgaagtctgcttaaaatgggagctaatggatgatgattatcggggaagactttgtaatcccctgaatccctttgtcagtcataaagagattgattctgtaaacaatgctgtggtaatgccagcccatggtccagccaaaagcatggaatatgatgccgttgcaactacacattcctggattcccaagaggaatcgttctattctcaacacaagccaaaggggaattcttgaggatgaacagatgtaccagaagtgctgcaatctattcgagaaatttttccctagcagttcatataggagaccggttggaatttctagcatggtggaggccatggtgtctagggcccggattgatgccagggtcgacttcgagtctggacggatcaagaaagaagagttctctgagatcatgaagatctgttccaccattgaagaactcagacggcaaaaataa

>H1N1_PB1_CY040168_A/Managua/3180.01/2008

atggatgtcaatccgacattacttttcttaaaagtgccagcacaaaatgctataagcacaacttttccttatactggtgaccctccttacagccatgggacaggaacagggtacaccatggatacagtcaacaggacacaccagtactcagaaagaggaagatggacaaaaaataccgaaactggagcaccgcaacttaacccaattgatggtccactaccggaagacaatgaaccaagtggctatgcccaaacagattgtgtattagaagcaatggctttccttgaagaatcccatcccggtatctttgaaaactcttgtattgaaacaatggaggttgttcagcaaacaagggtggacaaactgacacaaggcagacagacctatgactggactctaaataggaaccagcctgctgccacagcattggcaaacactatagaagtattcagatcaaacggcctcatagcaaatgaatctgggaggctaatagacttccttaaagatgtaatggagtcgatggacagaggcgaagtagagatcacaactcattttcaaagaaagaggagagtgagagacaatgtaactaaaaaaatggtgacccaaagaacaataggcaaaaagaaacataaattagacaaaagaagttacctaattagggcattaaccctgaacacaatgaccaaagatgctgagagggggaaactaaaacgcagagcaattgcaaccccaggaatgcaaataagggggtttgtatactttgttgagacactggcaagaagcatatgtgaaaagcttgaacaatcaggattgccagttggaggaaatgagaagaaagcaaagttagcaaatgttgtaaggaagatgatgaccaactcccaggacactgaaatttctttcaccataaccggagataacacaaaatggaacgaaaatcaaaaccctagaatgttcttggccatgatcacatatataaccaaaaatcagcctgaatggttcagaaatattctaagtattgctccaataatgttttcaaacaagatggcgagactaggtaaggggtacatgtttgaaagcaagagtatgaaactgagaactcaaatacctgcagagatgctagccaacatagatttgaaatatttcaatgattcaactaaaaagaaaattgaaaaaatccgaccattattaatagatggaactgcatcattgagtcctggaatgatgatgggcatgttcaatatgttgagcaccgtcttgggcgtctccattctgaatcttgggcaaaagagatacaccaagactacttactggtgggatggtcttcaatcgtctgatgattttgctttgattgtgaatgcacccaactatgcaggaattcaagctggagttgacaggttttatcgaacctgtaagctgctcggaattaatatgagcaaaaagaagtcttacataaacaggacaggtacctttgaattcacgagctttttctatcgttatgggtttgttgccaattttagcatggagcttcctagttttggggtgtctggggtcaatgaatctgcagacatgagtattggagtcactgtcatcaaaaacaatatgataaacaatgaccttggcccagcaactgctcaaatggcccttcagttatttataaaagattacaggtacacttatcgatgccaccgaggtgacacacaaatacaaacccggagatcttttgagataaagaaactatgggaccaaacccgctcaaaagctgggctgttggtctctgacggaggccccaatttatataacattagaaatctccatattcctgaagtttgcttgaaatgggagttgatggatgaggattaccaggggcgtttatgcaacccattaaacccgtttgtcagccataaagagattgaatcagtgaacaatgcagtgataatgccggcacatggtccagccaaaaatatggagtatgatgctgttgcaacaacacactcctgggtccccaaaagaaatcgatccattttgaacacgagccaaaggggggtacttgaagatgagcaaatgtatcagaggtgctgcaatttatttgaaaaattcttcccaagtagctcatacagaagaccagttggaatatccagtatggtagaggctatggtctcaagagcccgaattgatgcacggattgatttcgaatctggaaggataaagaaagaggaatttgctgagatcatgaagatctgttccaccattgaagacctcagacggcaaaaatga

>H1N1_PB1_CY040240_A/Managua/2860.01/2008

atggatgtcaatccgacattacttttcttaaaagtgccagcacaaaatgctataagcacaacttttccttatactggtgaccctccttacagccatgggacaggaacagggtacaccatggatacagtcaacaggacacaccagtactcagaaagaggaagatggacaaaaaataccgaaactggagcaccgcaacttaacccaattgatggtccactaccggaagacaatgaaccaagtggctatgcccaaacagattgtgtattagaagcaatggctttccttgaagaatcccatcccggtatctttgaaaactcttgtattgaaacaatggaggttgttcagcaaacaagggtggacaaactgacacaaggcagacagacctatgactggactctaaataggaaccagcctgctgccacagcattggcaaacactatagaagtattcagatcaaacggcctcatagccaatgaatctggaaggctaatagacttccttaaagatgtaatggagtcgatggacagaggcgaagtagaggtcacaactcattttcaaagaaagaggagagtgagagacaatgtaactaaaaaaatggtgacccaaagaacaataggcaaaaagaaacataaattagacaaaagaagttacctaattagggcattaaccctgaacacaatgaccaaagatgctgagagggggaaactaaaacgcagagcaattgcaaccccaggaatgcaaataagggggtttgtatactttgttgagacactggcaagaagcatatgtgaaaagcttgaacaatcaggattgccagttggagggaatgagaagaaagcaaagttagcaaatgttgtaaggaagatgatgaccaactcccaggacactgaaatttctttcaccataaccggagataacacaaaatggaacgaaaatcaaaaccctagaatgttcttggccatgatcacatatataaccaaaaatcagcctgaatggttcagaaatattctaagtattgctccaataatgttttcaaacaagatggcgagactaggtaaggggtacatgtttgaaagcaagagtatgaaactgagaactcaaatacctgcagagatgctagccaacatagatttgaaatatttcaatgattcaactaaaaagaaaattgaaaaaatccgaccattattaatagatggaactgcatcattgagtcctggaatgatgatgggcatgttcaatatgttgagcaccgtcttgggcgtctccattctgaatcttgggcaaaagagatacaccaagactacttactggtgggatggtcttcaatcgtctgatgattttgctttgattgtgaatgcacccaactatgcaggaattcaagctggagttgacaggttttatcgaacctgtaagctgctcggaattaatatgagcaaaaagaagtcttacataaacagaacaggtacctttgaattcacgagctttttctatcgttatgggtttgttgccaatttcagcatggagcttcctagttttggggtgtctggggtcaatgaatctgcagacatgagtattggagtcactgtcatcaaaaacaatatgataaacaatgaccttggcccagcaactgctcaaatggcccttcagttatttataaaagattacaggtacacttatcgatgccaccggggtgacacacaaatacaaacccggagatcgtttgagataaagaaactatgggaccaaacccgctcaaaagctgggctgttggtctctgatggaggccccaatttatataacattagaaatctccatattcctgaagtttgcctgaaatgggagttgatggatgaggattaccaggggcgtttatgcaacccattaaacccgtttgtcagccataaagagattgaatcagtgaacaatgcagtgataatgccggcacatggtccagccaaaaatatggagtatgacgctgttgcaacaacacactcctgggtccccaaaagaaatcgatccattttgaacacgagccaaagggggatacttgaagatgagcaaatgtatctgaggtgctgcaatttatttgaaaaattcttcccaagtagctcatacagaagaccagttggaatatccagtatggtagaggctatggtctcaagagcccgaattgatgcacggattgatttcgaatctggaaggataaagaaagaggaatttgctgagatcatgaagatctgttccaccattgaagacctcagacggcaaaaatga

>H1N1_PB1_GQ221692_A/GuangzhouSB/01/2009

atggatgtcaatccgactctacttttcctaaaaattccagcgcaaaatgccataagcaccacattcccttatactggagatcctccatacagccatggaacaggaacaggatacaccatggacacagtaaacagaacacaccaatactcagaaaagggaaagtggacgacaaacacagagactggtgcaccccagctcaacccgattgatggaccactacctgaggataatgaaccaagtgggtatgcacaaacagactgtgttctagaggctatggctttccttgaagaatcccacccaggaatatttgagaattcatgccttgaaacaatggaagttgttcaacaaacaagggtagataaactaactcaaggtcgccagacttatgattggacattaaacagaaatcaaccggcagcaactgcattggccaacaccatagaagtctttagatcgaatggcctaacagctaatgagtcaggaaggctaatagatttcttaaaggatgtaatggaatcaatgaacaaagaggaaatagagataacaatccactttcaaagaaaaaggagagtaagagacaacatgaccaagaagatggtcacgcaaagaacaatagggaagaaaaaacaaagactgaataagagaggctatctaataagagcactgacattaaatacgatgaccaaagatgcagagagaggcaagttaaaaagaagggctatcgcaacacctgggatgcagattagaggtttcgtatactttgttgaaactttagctaggagcatttgcgaaaagcttgaacagtctgggctcccagtagggggcaatgaaaagaaggccaaactggcaaatgttgtgagaaagatgatgactaattcacaagacacagagatttctttcacaatcactggggacaacactaagtggaatgaaaatcaaaatcctcgaatgttcctggcgatgattacatatatcaccagaaatcaacccgagtggttcagaaacatcctgagcatggcacccataatgttctcaaacaaaatggcaagactagggaaagggtacatgttcgagagtaaaagaatgaaaattcgaacacaaataccagcagaaatgctagcaagcattgacctgaagtacttcaatgaatcaacaaagaagaaaattgagaaaataaggcctcttctaatagatggcacagcatcactgagtcctgggatgatgatgggcatgttcaacatgctaagtacggtcttgggagtctcgatactgaatcttggacaaaagaaatacaccaagacaatatactggtgggatgggctccaatcatccgacgattttgctctcatagtgaatgcaccaaaccatgagggaatacaagcaggagtggacagattctacaggacctgcaagttagtgggaatcaacatgagcaaaaagaagtcctatataaataagacagggacatttgaattcacaagctttttttatcgctatggatttgtggctaattttagcatggagctacccagctttggagtgtctggagtaaatgaatcagctgacatgagtattggagtaacagtgataaagaacaacatgataaacaatgaccttggacctgcaacggcccagatggctcttcaattgttcatcaaagactacagatacacatataggtgccataggggagacacacaaattcagacgagaagatcatttgagttaaagaagctgtgggatcaaacccaatcaaaggtagggctattagtatcagatggaggaccaaacttatacaatatacggaatcttcacattcctgaagtctgcttaaaatgggagctaatggatgatgattatcggggaagactttgtaatcccctgaatccctttgtcagtcataaagagattgattctgtaaacaatgctgtggtaatgccagcccatggtccagccaaaagcatggaatatgatgccgttgcaactacacattcctggattcccaagaggaatcgttctattctcaacacaagccaaaggggaattcttgaggatgaacagatgtaccagaagtgctgcaatctattcgagaaatttttccctagcagttcatataggagaccggttggaatttctagcatggtggaggccatggtgtctagggcccggattgatgccagggtcgacttcgagtctggacggatcaagaaagaagagttctctgagatcatgaagatctgttccaccattgaagaactcagacggcaaaaataa

>H1N1_PB1_GQ222047_A/Osaka/2/2009

atggatgtcaatccgactctacttttcctaaaaattccagcgcaaaatgccataagcaccacattcccttatactggagatcctccatacagccatggaacaggaacaggatacaccatggacacagtaaacagaacacaccaatactcagaaaagggaaagtggacgacaaacacagagactggtgcaccccagctcaacccgattgatggaccactacctgaggataatgaaccaagtgggtatgcacaaacagactgtgttctagaggctatggctttccttgaagaatcccacccaggaatatttgagaattcatgccttgaaacaatggaagttgttcaacaaacaagggtagataaactaactcaaggtcgccagacttatgattggacattaaacagaaatcaaccggcagcaactgcattggccaacaccatagaagtctttagatcgaatggcctaacagctaatgagtcaggaaggctaatagatttcttaaaggatgtaatggaatcaatgaacaaagaggaaatagagataacaacccactttcaaagaaaaaggagagtaagagacaacatgaccaagaagatggtcacgcaaagaacaatagggaagaaaaaacaaagactaaataagagaggctatctaataagagcactgacattaaatacgatgaccaaagatgcagagagaggcaagttaaaaagaagggctatcgcaacacctgggatgcagattagaggtttcgtatactttgttgaaactttagctaggagcatttgcgaaaagcttgaacagtctgggctcccagtagggggcaatgaaaagaaggccaaactggcaaatgttgtgagaaagatgatgactaattcacaagacacagagatttctttcacaatcactggggacaacactaagtggaatgaaaatcaaaatcctcgaatgttcctggcgatgattacatatatcaccagaaatcaacccgagtggttcagaaacatcctgagcatggcacccataatgttctcaaacaaaatggcaagactagggaaagggtacatgttcgagagtaaaagaatgaagattcgaacacaaataccagcagaaatgctagcaagcattgacctgaagtacttcaatgaatcaacaaagaagaaaattgagaaaataaggcctcttctaatagatggcacagcatcactgagtcctgggatgatgatgggcatgttcaacatgctaagtacggtcttgggagtctcgatactgaatcttggacaaaagaaatacaccaagacaatatactggtgggatgggctccaatcatccgacgattttgctctcatagtgaatgcaccaaaccatgagggaatacaagcaggagtggacagattctacaggacctgcaagttagtgggaatcaacatgagcaaaaagaagtcctatataaataagacagggacatttgaattcacaagctttttttatcgctatggatttgtggctaattttagcatggagctacccagctttggagtgtctggagtaaatgaatcagctgacatgagtattggagtaacagtgataaagaacaacatgataaacaatgaccttggacctgcaacggcccagatggctcttcaattgttcatcaaagactacagatacacatataggtgccataggggagacacacaaattcagacgagaagatcatttgagttaaagaagctgtgggatcaaacccaatcaaaggtagggctattagtatcagatggaggaccaaacttatacaatatacggaatcttcacattcctgaagtctgcttaaaatgggagctaatggatgatgattatcggggaagactttgtaatcccctgaatccctttgtcagtcataaagagattgattctgtaaacaatgctgtggtaatgccagcccatggtccagccaaaagcatggaatatgatgccgttgcaactacacattcctggactcccaagaggaatcgttctattctcaacacaagccaaaggggaattcttgaggatgaacagatgtaccagaagtgctgcaatctattcgagaaatttttccctagcagttcatataggagaccggttggaatttctagcatggtggaggccatggtgtctagggcccggattgatgccagggtcgacttcgagtctggacggatcaagaaagaagagttctctgagatcatgaagatctgttccaccattgaagaactcagacggcaaaaataa

>H1N1_PB1_CY041200_A/New York/3190/2009

atggatgtcaatccgactctacttttcctaaaaattccagcgcaaaatgccataagcaccacattcccttatactggagatcctccatacagccatggaacaggaacaggatacaccatggacacagtaaacagaacacaccaatactcagaaaagggaaagtggacgacaaacacagagactggtgcaccccagctcaacccgattgatggaccactacctgaggataatgaaccaagtgggtatgcacaaacagactgtgttctagaggctatggctttccttgaagaatcccacccaggaatatttgagaattcatgccttgaaacaatggaagttgttcaacaaacaagggtagataaactaactcaaggtcgccagacttatgattggacattaaacagaaatcaaccggcagcaactgcattggccaacaccatagaagtctttagatcgaatggcctaacagctaatgagtcaggaaggctaatagatttcttaaaggatgtaatggaatcaatgaacaaagaggaaatagagataacaacccactttcaaagaaaaaggagagtaagagacaacatgaccaagaagatggtcacgcaaagaacaatagggaagaaaaaacaaagactgaataagagaggctatctaataagagcactgacattaaatacgatgaccaaagatgcagagagaggcaagttaaaaagaagggctatcgcaacacctgggatgcagattagaggtttcgtatactttgttgaaactttagctaggagcatttgcgaaaagcttgaacagtctgggctcccagtagggggcaatgaaaagaaggccaaactggcaaatgttgtgagaaagatgatgactaattcacaagacacagagatttctttcacaatcactggggacaacactaagtggaatgaaaatcaaaatcctcgaatgttcctggcgatgattacatatatcaccagaaatcaacccgagtggttcagaaacatcctgagcatggcacccataatgttctcaaacaaaatggcaagactagggaaagggtacatgttcgagagtaaaagaatgaagattcgaacacaaataccagcagaaatgctagcaagcattgacctgaagtacttcaatgaatcaacaaagaagaaaattgagaaaataaggcctcttctaatagatggcacagcatcactgagtcctgggatgatgatgggcatgttcaacatgctaagtacggtcttgggagtctcgatactgaatcttggacaaaagaaatacaccaagacaatatactggtgggatgggctccaatcatccgacgattttgctctcatagtgaatgcaccaaaccatgagggaatacaagcaggagtggacagattctacaggacctgcaagttagtgggaatcaacatgagcaaaaagaagtcctatataaataagacagggacatttgaattcacaagctttttttatcgctatggatttgtggctaattttagcatggagctacccagctttggagtgtctggagtaaatgaatcagctgacatgagtattggagtaacagtgataaagaacaacatgacaaacaatgaccttggacctgcaacggcccagatggctcttcaattgttcatcaaagactacagatacacatataggtgccataggggagacacacaaattcagacgagaagatcatttgagttaaagaagctgtgggatcaaacccaatcaaaggtagggctattagtatcagatggaggaccaaacttatacaatatacggaatcttcacattcctgaagtctgcttaaaatgggagctaatggatgatgattatcggggaagactttgtaatcccctgaatccctttgtcagtcataaagagattgattctgtaaacaatgctgtggtaatgccagcccatggtccagccaaaagcatggaatatgatgccgttgcaactacacattcctggattcccaagaggaatcgttctattctcaacacaagccaaaggggaattcttgaggatgaacagatgtaccagaagtgctgcaatctattcgagaaatttttccctagcagttcatataggagaccggttggaatttctagcatggtggaggccatggtgtctagggcccggattgatgccagggtcgacttcgagtctggacggatcaagaaagaagagttctctgagatcatgaagatctgttccaccattgaagaactcagacggcaaaaataa

>H1N1_PB1_GQ232026_A/Illinois/01/2009

atggatgtcaatccgactctacttttcctaaaaattccagcgcaaaatgccataagcaccacattcccttatactggagatcctccatacagccatggaacaggaacaggatacaccatggacacagtaaacagaacacaccgatactcagaaaagggaaagtggacgacaaacacagagactggtgcaccccagctcaacccgattgatggaccactacctgaggataatgaaccaagtgggtatgcacaaacagactgtgttctagaggctatggctttccttgaagaatcccacccaggaatatttgagaattcatgccttgaaacaatggaagttgttcaacaaacaagggtagataaactaactcaaggtcgccagacttatgattggacattaaacagaaatcaaccggcagcaactgcattggccaacaccatagaagtctttagatcgaatggcctaacagctaatgagtcaggaaggctaatagatttcttaaaggatgtaatggaatcaatgaacaaagaggaaatagagataacaacccactttcaaagaaaaaggagagtaagagacaacatgaccaagaagatggtcacgcaaagaacaatagggaagaaaaaacaaagactgaataagagaggctatctaataagagcactgacattaaatacgatgaccaaagatgcagagagaggcaagttaaaaagaagggctatcgcaacacctgggatgcagattagaggtttcgtatactttgttgaaactttagctaggagcatttgcgaaaagcttgaacagtctgggctcccagtagggggcaatgaaaagaaggccaaactggcaaatgttgtgagaaagatgatgactaattcacaagacacagagatttctttcacaatcactggggacaacactaagtggaatgaaaatcaaaatcctcgaatgttcctggcgatgattacatatatcaccagaaatcaacccgagtggttcagaaacatcctgagcatggcacccataatgttctcaaacaaaatggcaagactagggaaagggtacatgttcgagagtaaaagaatgaagattcgaacacaaataccagcagaaatgctagcaagcattgacctgaagtacttcaatgaatcaacaaagaagaaaattgagaaaataaggcctcttctaatagatggcacagcatcactgagtcctgggatgatgatgggcatgttcaacatgctaagtacggtcttgggagtctcgatactgaatcttggacaaaagaaatacaccaagacaatatactggtgggatgggctccaatcatccgacgattttgctctcatagtgaatgcaccaaaccatgagggaatacaagcaggagtggacagattctacaggacctgcaagttagtgggaatcaacatgagcaaaaagaagtcctatataaataagacagggacatttgaattcacaagctttttttatcgctatggatttgtggctaattttagcatggagctacccagctttggagtgtctggagtaaatgaatcagctgacatgagtattggagtaacagtgataaagaacaacatgataaacaatgaccttggacctgcaacggcccagatggctcttcaattgttcatcaaagactacagatacacatataggtgccataggggagacacacaaattcagacgagaagatcatttgagttaaagaagctgtgggatcaaacccaatcaaaggtagggctattagtatcagatggaggaccaaacttatacaatatacggaatcttcacattcctgaagtctgcttaaaatgggagctaatggatgatgattatcggggaagactttgtaatcccctgaatccctttgtcagtcataaagagattgattctgtaaacaatgctgtggtaatgccagcccatggtccagccaaaagcatggaatatgatgccgttgcaactacacattcctggattcccaagaggaatcgttctattctcaacacaagccaaaggggaattcttgaggatgaacagatgtaccagaagtgctgcaatctattcgagaaatttttccctagcagttcatataggagaccggttggaatttctagcatggtggaggccatggtgtctagggcccggattgatgccagggtcgacttcgagtctggacggatcaagaaagaagagttctctgagatcatgaagatctgttccaccattgaagaactcagacggcaaaaataa

>H1N1_PB1_CY041651_A/New York/3313/2009

atggatgtcaatccgactctacttttcctaaaaattccagcgcaaaatgccataagcaccacattcccttatactggagatcctccatacagccatggaacaggaacaggatacaccatggacacagtaaacagaacacaccaatactcagaaaagggaaagtggacgacaaacacagagactggtgcaccccagctcaacccgattgatggaccactacctgaggataatgaaccaagtgggtatgcacaaacagactgtgttctagaggctatggctttccttgaagaatcccacccaggaatatttgagaattcatgccttgaaacaatggaagttgttcaacaaacaagggtagataaactaactcaaggtcgccagacttatgattggacattaaacagaaatcaaccggcagcaactgcattggccaacaccatagaagtctttagatcgaatggcctaacagctaatgagtcaggaaggctaatagatttcttaaaggatgtaatggaatcaatgaacaaagaggaaatagagataacaacccactttcaaagaaaaaggagagtaagagacaacatgaccaagaagatggtcacgcaaagaacaatagggaagaaaaaacaaagactgaataagagaggctatctaataagagcactgacattaaatacgatgaccaaagatgcagagagaggcaagttaaaaagaagggctatcgcaacacctgggatgcagattagaggtttcgtatactttgttgaaactttagctaggagcatttgcgaaaagcttgaacagtctgggctcccagtagggggcaatgaaaagaaggccaaactggcaaatgttgtgagaaagatgatgactaattcacaagacacagagatttctttcacaatcactggggacaacactaagtggaatgaaaatcaaaatcctcgaatgttcctggcgatgattacatatatcaccagaaatcaacccgagtggttcagaaacatcctgagcatggcacccataatgttctcaaacaaaatggcaagactagggaaagggtacatgttcgagagtaaaagaatgaagattcgaacacaaataccagcagaaatgctagcaagcattgacctgaagtacttcaatgaatcaacaaagaagaaaattgagaaaataaggcctcttctaatagatggcacagcatcactgagtcctgggatgatgatgggcatgttcaacatgctaagtacggtcttgggagtctcgatactgaatcttggacaaaagaaatacaccaagacaatatactggtgggatgggctccaatcatccgacgattttgctctcatagtgaatgcaccaaaccatgagggaatacaagcaggagtggacagattctacaggacctgcaagttagtgggaatcaacatgagcaaaaagaagtcctatataaataagacagggacatttgaattcacaagctttttttatcgctatggatttgtggctaattttagcatggagctacccagctttggagtgtctggagtaaatgaatcagctgacatgagtattggagtaacagtgataaagaacaacatgataaacaatgaccttggacctgcaacggcccagatggctcttcaattgttcatcaaagactacagatacacatataggtgccataggggagacacacaaattcagacgagaagatcatttgagttaaagaagctgtgggatcaaacccaatcaaaggtagggctattagtatcagatggaggaccaaacttatacaatatacggaatcttcacattcctgaagtctgcttaaaatgggagctaatggatgatgattatcggggaagactttgtaatcccctgaatccctttgtcagtcataaagagattgattctgtaaacaatgctgtggtaatgccagcccatggtccagccaaaagcatggaatatgatgccgttgcaactacacattcctggattcccaagaggaatcgttctattctcaacacaagccaaaggggaattcttgaggatgaacagatgtaccagaagtgctgcaatctattcgagaaatttttccctagcagttcatataggagaccggttggaatttctagcatggtggaggccatggtgtctagggcccggattgatgccagggtcgacttcgagtctggacggatcaagaaagaagagttctctgagatcatgaagatctgttccaccattgaagaactcagacggcaaaaataa

>H1N1_PB1_GQ288370_A/Fuzhou/01/2009

atggatgtcaatccgactctacttttcctaaaaattccagcgcaaaatgccataagcaccacattcccttatactggagatcctccatacagccatggaacaggaacaggatacaccatggacacagtaaacagaacacaccaatactcagaaaagggaaagtggacgacaaacacagagactggtgcaccccagctcaacccgattgatggaccactacctgaggataatgaaccaagtgggtatgcacaaacagactgtgttctagaggctatggctttccttgaagaatcccacccaggaatatttgagaattcatgccttgaaacaatggaagttgttcaacaaacaagggtagataaactaactcaaggtcgccagacttatgattggacattaaacagaaatcaaccggcagcaactgcattggccaacaccatagaagtctttagatcgaatggcctaacagctaatgagtcaggaaggctaatagatttcttaaaggatgtaatggaatcaatgaacaaagaggaaatagagataacaacccactttcaaagaaaaaggagagtaagagacaacatgaccaagaagatggtcacgcaaagaacaatagggaagaaaaaacaaagactgaataagagaggctatctaataagagcactgacattaaatacgatgaccaaagatgcagagagaggcaagttaaaaagaagggctatcgcaacacctgggatgcagattagaggtttcgtatactttgttgaaactttagctaggagcatttgcgaaaagcttgaacagtctgggctcccagtagggggcaatgaaaagaaggccaaactggcaaatgttgtgagaaagatgatgactaattcacaagacacagagatttctttcacaatcactggggacaacactaagtggaatgaaaatcaaaatcctcgaatgttcctggcgatgattacatatatcaccagaaatcaacccgagtggttcagaaacatcctgagcatggcacccataatgttctcaaacaaaatggcaagactagggaaagggtacatgttcgagagtaaaagaatgaagattcgaacacaaataccagcagaaatgctagcaagcattgacctgaagtacttcaatgaatcaacaaagaagaaaattgagaaaataaggcctcttctaatagatggcacagcatcactgagtcctgggatgatgatgggcatgttcaacatgctaagtacggtcttgggagtctcgatactgaatcttggacaaaagaaatacaccaagacaatatactggtgggatgggctccaatcatccgacgattttgctctcatagtgaatgcaccaaaccatgagggaatacaagcaggagtggacagattctacaggacctgcaagttagtgggaatcaacatgagcaaaaagaagtcctatataaataagacagggacatttgaattcacaagctttttttatcgctatggatttgtggctaattttagcatggagctacccagctttggagtgtctggagtaaatgaatcagctgacatgagtattggagtaacagtgataaagaacaacatgataaacaatgaccttggacctgcaacggcccagatggctcttcaattgttcatcaaagactacagatacacatataggtgccataggggagacacacaaattcagacgagaagatcatttgagttaaagaagctgtgggatcaaacccaatcaaaggtagggctattagtatcagatggaggaccaaacttatacaatatacggaatcttcacattcctgaagtctgcttaaaatgggagctaatggatgatgattatcggggaagactttgtaatcccctgaatccctttgtcagtcataaagagattgattctgtaaacaatgctgtggtaatgccagcccatggtccagccaaaagcatggaatatgatgccgttgcaactacacattcctggattcccaagaggaatcgttctattctcaacacaagccaaagaggaattcttgaggatgaacagatgtaccagaagtgctgcaatctattcgagaaatttttccctagcagttcatataggagaccggttggaatttctagcatggtggaggccatggtgtctagggcccggattgatgccagggtcgacttcgagtctggacggatcaagaaagaagagttctctgagatcatgaagatctgttccaccattgaagaactcagacggcaaaaataa

>H1N1_PB1_GQ340062_A/Shanghai/143T/2009

atggatgtcaatccgactctacttttcctaaaaattccagcgcaaaatgccataagcaccacattcccttatactggagatcctccatacagccatggaacaggaacaggatacaccatggacacagtaaacagaacacaccaatactcagaaaagggaaagtggacgacaaacacagagactggtgcaccccagctcaacccgattgatggaccactacctgaggataatgaaccaagtgggtatgcacaaacagactgtgttctagaggctatggctttccttgaagaatcccacccaggaatatttgagaattcatgccttgaaacaatggaagttgttcaacaaacaagggtagataaactaactcaaggtcgccagacttatgattggacattaaacagaaatcaaccggcagcaactgcattggccaacaccatagaagtctttagatcgaatggcctaacagctaatgagtcaggaaggctaatagatttcttaaaggatgtaatggaatcaatgaacaaagaggaaatagagataacaacccactttcaaagaaaaaggagagtaagagacaacatgaccaagaagatggtcacacaaagaacaatagggaagaaaaaacaaagactgaataagagaggctatctaataagagcactgacattaaatacgatgaccaaagatgcagagagaggcaagttaaaaagaagggctatcgcaacacctgggatgcagattagaggtttcgtatactttgttgaaactttagctaggagcatttgcgaaaagcttgaacagtctgggctcccagtagggggcaatgaaaagaaggccaaactggcaaatgttgtgagaaagatgatgactaattcacaagacacagagatttctttcacaatcactggggacaacactaagtggaatgaaaatcaaaatcctcgaatgttcctggcgatgattacatatatcaccagaaatcaacccgagtggttcagaaacatcctgagcatggcacccataatgttctcaaacaaaatggcaagactagggaaagggtacatgttcgagagtaaaagaatgaagattcgaacacaaataccagcagaaatgctagcaagcattgacctgaagtacttcaatgaatcaacaaagaagaaaattgagaaaataaggcctcttctaatagatggcacagcatcactgagtcctgggatgatgatgggcatgttcaacatgctaagtacggtcttgggagtctcgatactgaatcttggacaaaagaaatacaccaagacaatatactggtgggatgggctccaatcatccgacgattttgctctcatagtgaatgcaccaaaccatgagggaatacaagcaggagtggacagattctacaggacctgcaagttagtgggaatcaacatgagcaaaaagaagtcctatataaataagacagggacatttgaattcacaagctttttttatcgctatggatttgtggctaattttagcatggagctacccagctttggagtgtctggagtaaatgaatcagctgacatgagtattggagtaacagtgataaagaacaacatgataaacaatgaccttggacctgcaacggcccagatggctcttcaattgttcatcaaagactacagatacacatataggtgccataggggagacacacaaattcagacgagaagatcatttgagttaaagaagctgtgggatcaaacccaatcaaaggtagggctattagtatcagatggaggaccaaacttatacaatatacggaatcttcacattcctgaagtctgcttaaaatgggagctaatggatgatgattatcggggaagactttgtaatcccctgaatccctttgtcagtcataaagagatcgattctgtaaacaatgctgtggtaatgccagcccatggtccagccaaaagcatggaatatgatgccgttgcaactacacattcctggattcccaagaggaatcgttctattctcaacacaagccaaaggggaattcttgaggatgaacagatgtaccagaagtgttgcaatctattcgagaaatttttccctagcagttcatataggagaccggttggaatttctagcatggtggaggccatggtgtctagggcccggattgatgccagggtcgacttcgagtctggacggatcaagaaagaagagttctctgagatcatgaagatctgttccaccattgaagaactcagacggcaaaaataa

>H1N1_PB1_GQ402310_A/Mexico/InDRE13495/2009

atggatgtcaatccgactctacttttcctaaaaattccagcgcaaaatgccataagcaccacattcccttatactggagatcctccatacagccatggaacaggaacaggatacaccatggacacagtaaacagaacacaccaatactcagaaaagggaaagtggacgacaaacacagagactggtgcaccccagctcaacccgattgatggaccactacctgaggataatgaaccaagtgggtatgcacaaacagactgtgttctagaggctatggctttccttgaagaatcccacccaggaatatttgagaattcatgccttgaaacaatggaagttgttcaacaaacaagggtagataaactaactcaaggtcgccagacttatgattggacattaaacagaaatcaaccggcagcaactgcattggccaacaccatagaagtctttagatcgaatggcctaacagctaatgagtcaggaaggctaatagatttcttaaaggatgtaatggaatcaatgaacaaagaggaaatagagataacaacccactttcaaagaaaaaggagagtaagagacaacatgaccaagaagatggtcacgcaaagaacaatagggaagaaaaaacaaagactgaataagagaggctatctaataagagcactgacattaaatacgatgaccaaagatgcagagagaggcaagttaaaaagaagggctatcgcaacacctgggatgcagattagaggtttcgtatactttgttgaaactttagctaggagcatttgcgaaaagcttgaacagtctgggctcccagtagggggcaatgaaaagaaggccaaactggcaaatgttgtgagaaagatgatgactagttcacaagacacagagatttctttcacaatcactggggacaacactaagtggaatgaaaatcaaaatcctcgaatgttcctggcgatgattacatatatcaccagaaatcaacccgagtggttcagaaacatcctgagcatggcacccataatgttctcaaacaaaatggcaagactagggaaagggtacatgttcgagagtaaaagaatgaagattcgaacacaaataccagcagaaatgctagcaagcattgacctgaagtacttcaatgaatcaacaaagaagaaaattgagaaaataaggcctcttctaatagatggcacagcatcactgagtcctgggatgatgatgggcatgttcaacatgctaagtacagtcttgggagtctcgatactgaatctyggacaaaagaaatacaccaagacaatatactggtgggatgggctccaatcatccgacgattttgctctcatagtgaatgcaccaaaccatgagggaatacaagcaggagtggacagattctacaggacctgcaagttagtgggaatcaacatgagcaaaaagaagtcctatataaataagacagggacatttgaattcacaagctttttttatcgctatggatttgtggctaattttagcatggagctacccagctttggagtgtctggagtaaatgaatcagctgacatgagtattggagtaacagtgataaagaacaacatgataaacaatgaccttggacctgcaacggcccagatggctcttcaattgttcatcaaagactacagatacacatataggtgccataggggagacacacaaattcagacgagaagatcatttgagttgaagaagctgtgggatcaaacccaatcaaaggtagggctattagtatcagatggaggaccaaacttatacaatatacggaatcttcacattcctgaagtctgcttaaaatgggagctaatggatgatgattatcggggaagactttgtaatcccctgaatccctttgtcagtcataaagagattgattctgtaaacaatgctgtggtaatgccagcccatggtccagccaaaagcatggaatatgatgccgttgcaactacacattcctggattcccaagaggaatcgttctattctcaacacaagccaaaggggaattcttgaggatgaacagatgtaccagaagtgctgcaatctattcgagaaatttttccctagcagttcatataggagaccggttggaatttctagcatggtggaggccatggtgtctagggcccggattgatgccagggtcgacttcgagtctggacggatcaagaaagaagagttctctgagatcatgaagatctgttccaccattgaagaactcagacggcaaaaataa

>H1N1_PB1_GQ402323_A/Canada-MB/RV1982/2009

atggatgtcaatccgactctacttttcctaaaaattccagcgcaaaatgccataagcaccacattcccttatactggagatcctccatacagccatggaacaggaacaggatacaccatggacacagtaaacagaacacaccaatactcagaaaagggaaagtggacgacaaacacagagactggtgcaccccagctcaacccgattgatggaccactacctgaggataatgaaccaagtgggtatgcacaaacagactgtgttctagaggctatggctttccttgaagaatcccacccaggaatatttgagaattcatgccttgaaacaatggaagttgttcaacaaacaagggtagataaactaactcaaggtcgccagacttatgattggacattaaacagaaatcaaccggcagcaactgcattggccaacaccatagaagtctttagatcgaatggtctaacagctaatgagtcaggaaggctaatagatttcttaaaggatgtaatggaatcaatgaacaaagaggaaatagagataacaacccactttcaaagaaaaaggagagtaagagacaacatgaccaagaagatggtcacacaaagaacaatagggaagaaaaaacaaagactgaataagagaggctatctaataagagcactgacattaaatacgatgaccaaagatgcagagagaggcaagttaaaaagaagggctatcgcaacacctgggatgcagattagaggtttcgtatactttgttgaaactttagctaggagcatttgcgaaaagcttgaacagtctgggctcccagtagggggcaatgaaaagaaggccaaactggcaaatgttgtgagaaagatgatgactaattcacaagacacagagatttctttcacaatcactggggacaacactaagtggaatgaaaatcaaaatcctcgaatgttcctggcgatgattacatatatcaccagaaatcaacccgagtggttcagaaacatcctgagcatggcacccataatgttctcaaacaaaatggcaagactagggaaagggtacatgttcgagagtaaaagaatgaagattcgaacacaaataccagcagaaatgctagcaagcattgacctgaagtacttcaatgaatcaacaaagaagaaaattgagaaaataaggcctcttctaatagatggcacagcatcactgagtcctgggatgatgatgggcatgttcaacatgctaagtacggtcttgggagtctcgatactgaatcttggacaaaagaaatacaccaagacaatatactggtgggatgggctccaatcatccgacgattttgctctcatagtgaatgcaccaaaccatgagggaatacaagcaggagtggacagattctacaggacctgcaagttagtgggaatcaacatgagcaaaaagaagtcctatataaataagacagggacatttgaattcacaagctttttttatcgctatggatttgtggctaattttagcatggagctacccagctttggagtgtctggagtaaatgaatcagctgacatgagtattggagtaacagtgataaagaacaacatgataaacaatgaccttggacctgcaacggcccagatggctcttcaattgttcatcaaagactacagatacacatataggtgccataggggagacacacaaattcagacgagaagatcatttgagttaaagaagctgtgggatcaaacccaatcaaaggtagggctattagtatcagatggaggaccaaacttatacaatatacggaatcttcacattcctgaagtctgcttaaaatgggagctaatggatgatgattatcggggaagactttgtaatcccctgaatccctttgtcagtcataaagagattgattctgtaaacaatgctgtggtaatgccagcccatggtccagccaaaagcatggaatatgatgctgttgcaactacacattcctggattcccaagaggaatcgttctattctcaacacaagccaaaggggaattcttgaggatgaacagatgtaccagaagtgttgcaatctattcgagaaatttttccctagcagttcatataggagaccggttggaatttctagcatggtggaggccatggtgtctagggcccggattgatgccagggtcgacttcgagtctggacgggtcaagaaagaagagttctctgagatcatgaagatctgttccaccattgaagaactcagacggcaaaaataa

>H1N1_PB1_GQ411903_A/Toronto/T5308/2009

atggatgtcaatccgactctacttttcctaaaaattccagcgcaaaatgccataagcaccacattcccttatactggagatcctccatacagccatggaacaggaacaggatacaccatggacacagtgaacagaacacaccaatactcagaaaagggaaagtggacgacaaacacagagactggtgcaccccagctcaacccgattgatggaccactacctgaggataatgaaccaagtgggtatgcacaaacagactgtgttctagaggctatggctttccttgaagaatcccacccaggaatatttgagaattcatgccttgaaacaatggaagttgttcaacaaacaagggtagataaactaactcaaggtcgccagacttatgattggacattaaacagaaatcaaccggcagcaactgcattggccaacaccatagaagtctttagatcgaatggtctaacagctaatgagtcaggaaggctaatagatttcttaaaggatgtaatggaatcaatgaacaaagaggaaatagagataacaacccactttcaaagaaaaaggagagtaagagacaacatgaccaagaagatggtcacgcaaagaacaatagggaagaaaaaacaaagactgaataagagaggctatctaataagagcactgacattaaatacgatgaccaaagatgcagagagaggcaagttaaaaagaagggctatcgcaacacctgggatgcagattagaggtttcgtatactttgttgaaactttagctaggagcatttgcgaaaagcttgaacagtctgggctcccagtagggggcaatgaaaagaaggccaaactggcaaatgttgtgagaaagatgatgactaattcacaagacacagagatttctttcacaatcactggggacaacactaagtggaatgaaaatcaaaatcctcgaatgttcctggcgatgattacatatatcaccagaaatcaacccgagtggttcagaaacatcctgagcatggcacccataatgttctcaaacaaaatggcaagactagggaaagggtacatgttcgagagtaaaagaatgaagattcgaacacaaataccagcagaaatgctagcaagcattgacctgaagtacttcaatgaatcaacaaagaagaaaattgagaaaataaggcctcttctaatagatggcacagcatcactgagtcctgggatgatgatgggcatgttcaacatgctaagtacggtcttgggagtctcgatactgaatcttggacaaaagaaatacaccaagacaatatactggtgggatgggctccaatcatccgacgattttgctctcatagtgaatgcaccaaaccatgagggaatacaagcaggagtggacagattctacaggacctgcaagttagtgggaatcaacatgagcaaaaggaagtcctatataaataagacagggacatttgaattcacaagctttttttatcgctatggatttgtggctaattttagcatggagctacccagctttggagtgtctggagtaaatgaatcagctgacatgagtattggagtaacagtgataaagaacaacatgataaacaatgaccttggacctgcaacggcccagatggctcttcaattgttcatcaaagactacagatacacatataggtgccataggggagacacacaaattcagacgagaagatcatttgagttaaagaagctgtgggatcaaacccaatcaaaggtagggctattagtatcagatggaggaccaaacttatacaatatacggaatcttcacattcctgaagtctgcttaaaatgggagctaatggatgatgattatcggggaagactttgtaatcccctgaatccctttgtcagtcataaagagattgattctgtaaacaatgctgtggtaatgccagcccatggtccagccaaaagcatggaatatgatgccgttgcaactacacattcctggattcccaagaggaatcgttctattctcaacacaagccaaaggggaattcttgaggatgaacagatgtaccagaagtgctgcaatctattcgagaaatttttccctagcagttcatataggagaccggttggaatttctagcatggtggaggccatggtgtctagggcccggattgatgccagggtcgacttcgagtctggacggatcaagaaagaagagttctctgagatcatgaagatctgttccaccattgaagaactcagacggcaaaaataa

>H1N1_PB1_CY043348_A/Denmark/528/2009

atggatgtcaatccgactctacttttcctaaaaattccagcgcaaaatgccataagcaccacattcccttatactggagatcctccatacagccatggaacaggaacaggatacaccatggacacagtaaacagaacacaccaatactcagaaaagggaaagtggacgacaaacacagagactggtgcaccccagctcaacccgattgatggaccactacctgaggataatgaaccaagtgggtatgcacaaacagactgtgttctagaggctatggctttccttgaagaatcccacccaggaatatttgagaattcatgccttgaaacaatggaagttgttcaacaaacaagggtagataaactaactcaaggtcgccagacttatgattggacattaaacagaaatcaaccggcagcaactgcattggccaacaccatagaagtctttagatcgaatggcctaacagctaatgagtcaggaaggctaatagatttcttaaaggatgtaatggaatcaatgaacaaagaggaaatagagataacaacccactttcaaagaaaaaggagagtaagagacaacatgaccaagaagatggtcacgcaaagaacaatagggaagaaaaaacaaagactgaataagaggggctatctaataagagcactgacattaaatacgatgaccaaagatgcagagagaggcaagttaaaaagaagggctatcgcaacacctgggatgcagattagaggtttcgtatactttgttgaaactttagctaggagcatttgcgaaaagcttgaacagtctgggctcccagtagggggcaatgaaaagaaggccaaactggcaaatgttgtgagaaagatgatgactaattcacaagacacagagatttctttcacaatcactggggacaacactaagtggaatgaaaatcaaaatcctcgaatgttcctggcgatgattacatatatcaccagaaatcaacccgagtggttcagaaacatcctgagcatggcacccataatgttctcaaacaaaatggcaagactagggaaagggtacatgttcgagagtaaaagaatgaagattcgaacacaaataccagcagaaatgctagcaagcattgacctgaagtacttcaatgaatcaacaaagaagaaaattgagaaaataaggcctcttctaatagatggcacagcatcactgagtcctgggatgatgatgggcatgttcaacatgctaagtacggtcttgggagtctcgatactgaatcttggacaaaagaaatacaccaagacaatatactggtgggatgggctccaatcatccgacgattttgctctcatagtgaatgcaccaaaccatgagggaatacaagcaggagtggacagattctacaggacctgcaagttagtgggaatcaacatgagcaaaaagaagtcctatataaataagacagggacatttgaattcacaagctttttttatcgctatggatttgtggctaattttagcatggagctacccagctttggagtgtctggagtaaatgaatcagctgacatgagtattggagtaacagtgataaagaacaacatgataaacaatgaccttggacctgcaacggcccagatggctcttcaattgttcatcaaagactacagatacacatataggtgccataggggagacacacaaattcagacgagaagatcatttgagttaaagaagctgtgggatcaaacccaatcaaaggtagggctattagtatcagatggaggaccaaacttatacaatatacggaatcttcacattcctgaagtctgcttaaaatgggagctaatggatgatgattatcggggaagactttgtaatcccctgaatccctttgtcagtcataaagagattgattctgtaaacaatgctgtggtaatgccagcccatggtccagccaaaagcatggaatatgatgccgttgcaactacacattcctggattcccaagaggaatcgttctattctcaacacaagccaaaggggaattcttgaggatgaacagatgtaccagaagtgctgcaatctattcgagaaatttttccctagcagttcatataggagaccggttggaatttctagcatggtggaggccatggtgtctagggcccggattgatgccagggtcgactccgagtctggacggatcaagaaagaagagttctctgagatcatgaagatctgttccaccattgaagaactcagacggcaaaaataa

>H1N1_PB1_CY043790_A/Managua/954.02/2008

atggatgtcaatccgacattacttttcttaaaagtgccagcacaaaatgctataagcacaacttttccttatactggtgaccctccttacagccatgggacaggaacagggtacaccatggatacagtcaacaggacacaccagtactcagaaagaggaagatggacaaaaaataccgaaactggagcaccgcaacttaacccaattgatggtccactaccggaagacaatgaaccaagtggctatgcccaaacagattgtgtattagaagcaatggctttccttgaagaatcccatcccggtatctttgaaaactcttgtattgaaacaatggagattgttcagcaaacaagggtggacaaactgacacaaggcagacagacctatgactggactctaaataggaaccagcctgctgccacagcattggcaaacactatagaagtattcagatcaaacggcctcatagcaaatgaatctgggaggctaatagacttccttaaagatgtaatggagtcgatggacagaggcgaagtagaggtcacaactcattttcaaagaaagaggagagtgagagacaatgtaactaaaaaaatggtgacccaaagaacaataggcaaaaagaaacataaattagacaaaagaagttacctaattagggcattaaccctgaacacaatgaccaaagatgctgagagggggaaactaaaacgcagagcaattgcaaccccaggaatgcaaataagggggtttgtatactttgttgagacactggcaagaagcatatgtgaaaagcttgaacaatcaggattgccagttggaggaaatgagaagaaagcaaagttagcaaatgttgtaaggaagatgatgaccaactcccaggacactgaaatttctttcaccataaccggagataacacaaaatggaacgaaaatcaaaaccctagaatgttcttggccatgatcacatatataaccaaaaatcagcctgaatggttcagaaatattctaagtattgctccaataatgttttcaaacaaaatggcgagactaggtaaggggtacatgtttgaaagcaagagtatgaaactgagaactcaaatacctgcagagatgctagccaacatagatttgaaatatttcaatgattcaactaaaaagaaaattgaaaaaatccgaccactattaatagatggaactgcatcattgagtcctggaatgatgatgggcatgttcaatatgttgagcaccgtcttgggcgtctccattctgaatcttgggcaaaagagatacaccaagactacttactggtgggatggtcttcaatcgtctgatgattttgctttgattgtgaatgcacccaactatgcaggaattcaagctggagttgacaggttttatcgaacctgtaagctgctcggaattaatatgagcaaaaagaagtcttacataaacagaacaggtacctttgaattcacgagctttttctatcgttatgggtttgttgccaatttcagcatggagcttcctagttttggggtgtctggggtcaatgaatctgcagacatgagtattggagtcactgtcatcaaaaacaatatgataaacaatgaccttggcccagcaactgctcaaatggcccttcagttatttataaaagattacaggtacacttatcgatgccaccgaggtgacacacaaatacaaacccggagatcatttgagataaagaaactatgggaccaaacccgctcaaaagctgggctgttggtctctgatggaggccccaatttatataacattagaaatctccatattcctgaagtttgcttgaaatgggagttgatggatgaggattaccaggggcgtttatgcaacccattaaacccgtttgtcagccataaagagattgaatcagtgaacaatgcagtgataatgccggcacatggtccagccaaaaatatggagtatgacgctgttgcaacaacacactcctgggtccccaaaagaaatcgatccattttgaacacgagccaaaggggggtacttgaagatgagcaaatgtatcagaggtgctgcaatttatttgaaaaattcttcccaagtagctcatacagaagaccagttggaatatccagtatggtagaggctatggtctcaagagcccgaattgatgcacggattgatttcgaatctggaaggataaagaaagaggaatttgctgagatcatgaagatctgttccaccattgaagacctcagacggcaaaaatga

>H1N1_PB1_GQ457468_A/Utah/11/2009

atggatgtcaatccgactctacttttcctaaaaattccagcgcaaaatgccataagcaccacattcccttatactggagatcctccatacagccatggaacaggaacaggatacaccatggacacagtaaacagaacacaccaatactcagaaaagggaaagtggacgacaaacacagagactggtgcaccccagctcaacccgattgatggaccactacctgaggataatgaaccaagtgggtatgcacaaacagactgtgttctagaggctatggctttccttgaagaatcccacccaggaatatttgagaattcatgccttgaaacaatggaagttgttcaacaaacaagggtagataaactaactcaaggtcgccagacttatgattggacattaaacagaaatcaaccggcagcaactgcattggccaacaccatagaagtctttagatcgaatggcctaacagctaatgagtcaggaaggctaatagatttcttaaaggatgtaatggaatcaatgaacaaagaggaaatagagataacaacccactttcaaagaaaaaggagagtaagagacaacatgaccaagaagatggtcacgcaaagaacaatagggaagaaaaaacaaagactgaataagagaggctatctaataagagcactgacattaaatacgatgaccaaagatgcagagagaggcaagttaaaaagaagggctatcgcaacacctgggatgcagattagaggtttcgtatactttgttgaaactttagctaggagcatttgcgaaaagcttgaacagtctgggctcccagtagggggcaatgaaaagaaggccaaactggcaaatgttgtgagaaagatgatgactaattcacaagacacagagatttctttcacaatcactggggacaacactaagtggaatgaaaatcaaaatcctcgaatgttcctggcgatgattacatatatcaccagaaatcaacccgagtggttcagaaacatcctgagcatggcacccataatgttctcaaacaaaatggcaagactagggaaagggtacatgttcgagagtaaaagaatgaagattcgaacacaaataccagcagaaatgctagcaagcattgacctgaagtacttcaatgaatcaacaaagaagaaaattgagaaaataaggcctcttctaatagatggcacagcatcactgagtcctgggatgatgatgggcatgttcaacatgctaagtacggtcttgggagtctcgatactgaatcttggacaaaagaaatacaccaagacaatatattggtgggatgggctccaatcatccgacgattttgctctcatagtgaatgcaccaaaccatgagggaatacaagcaggagtggacagattctacaggacctgcaagttagtgggaatcaacatgagcaaaaagaagtcctatataaataagacagggacatttgaattcacaagctttttttatcgctatggatttgtggctaattttagcatggagctacccagctttggagtgtctggagtaaatgaatcagctgacatgagtattggagtaacagtgataaagaacaacatgataaacaatgaccttggacctgcaacggcccagatggctcttcaattgttcatcaaagactacagatacacatataggtgccataggggggacacacaaattcagacgagaagatcatttgagttaaagaagctgtgggatcaaacccaatcaaaggtagggctattagtatcagatggaggaccaaacttatacaatatacggaatcttcacattcctgaagtctgcttaaaatgggagctaatggatgatgattatcggggaagactttgtaatcccctgaatccctttgtcagtcataaagagattgattctgtaaacaatgctgtggtaatgccagcccatggtccagccaaaagcatggaatatgatgccgttgcaactacacattcctggattcccaagaggaatcgttctattctcaacacaagccaaaggggaattcttgaggatgaacagatgtaccagaagtgctgcaatctattcgagaaatttttccctagcagttcatataggagaccggttggaatttctagcatggtggaggccatggtgtctagggcccggattgatgccagggtcgacttcgagtctggacggatcaagaaagaagagttctctgagatcatgaagatctgttccaccattgaagaactcagacggcaaaaataa

>H1N1_PB1_GQ465752_A/Canada-PQ/RV1758/2009

atggatgtcaatccgactctacttttcctaaaaattccagcgcaaaatgccataagcaccacattcccttatactggagatcctccatacagccatggaacaggaacaggatacaccatggacacagtaaacagaacacaccaatactcagaaaagggaaagtggacgacaaacacagagactggtgcaccccagctcaacccgattgatggaccactacctgaggataatgaaccaagtgggtatgcacaaacagactgtgttctagaggctatggctttccttgaagaatcccacccaggaatatttgagaattcatgccttgaaacaatggaagttgttcaacaaacaagggtagataaactaactcaaggtcgccagacttatgattggacattaaacagaaatcaaccggcagcaactgcattggccaacaccatagaagtctttagatcgaatggcctaacagctaatgagtcaggaaggctaatagatttcttaaaggatgtaatggaatcaatgaacaaagaggaaatagagataacaacccactttcaaagaaaaaggagagtaagagacaacatgaccaagaagatggtcacgcaaagaacaatagggaagaaaaaacaaagactgaataagagaggctatctaataagagcactgacattaaatacgatgaccaaagatgcagagagaggcaagttaaaaagaagggctatcgcaacacctgggatgcagattagaggtttcgtatactttgttgaaactttagctaggagcatttgcgaaaagcttgaacagtctgggctcccagtagggggcaatgaaaagaaggccaaactggcaaatgttgtgagaaagatgatgactaattcacaagacacagagatttctttcacaatcactggggacaacactaagtggaatgaaaatcaaaatcctcgaatgttcctggcgatgattacatatatcaccagaaatcaacccgagtggttcagaaacatcctgagcatggcacccataatgttctcaaacaaaatggcaagactagggaaagggtacatgttcgagagtaaaagaatgaagattcgaacacaaataccagcagaaatgctagcaagcattgacctgaagtacttcaatgaatcaacaaagaagaaaattgagaaaataaggcctcttttaatagatggcacagcatcactgagtcctgggatgatgatgggcatgttcaacatgctaagtacggtcttgggagtctcgatactgaatcttggacaaaagaaatacaccaagacaatatactggtgggatgggctccaatcatccgacgattttgctctcatagtgaatgcaccaaaccatgagggaatacaagcaggagtggacagattctacaggacctgcaagttagtgggaatcaacatgagcaaaaagaagtcctatataaataagacagggacatttgaattcacaagctttttttatcgctatggatttgtggctaattttagcatggagctacccagctttggagtgtctggagtaaatgaatcagctgacatgagtattggagtaacagtgataaagaacaacatgataaacaatgaccttggacctgcaacggcccagatggctcttcaattgttcatcaaagactacagatacacatataggtgccataggggagacacacaaattcagacgagaagatcatttgagttaaagaagctgtgggatcaaacccaatcaaaggtagggctattagtatcagatggaggaccaaacttatacaatatacggaatcttcacattcctgaagtctgcttaaaatgggagctaatggatgatgattatcggggaagactttgtaatcccctgaatccctttgtcagtcataaagagattgattctgtaaacaatgctgtggtaatgccagcccatggtccagccaaaagcatggaatatgatgccgttgcaactacacattcctggattcccaagaggaatcgttctattctcaacacaagccaaaggggaattcttgaggatgaacagatgtaccagaagtgctgcaatctattcgagaaatttttccctagcagttcatataggagaccggttggaatttctagcatggtggaggccatggtgtctagggcccggattgatgccagggtcgacttcgagtctggacggatcaagaaagaagagttctctgagatcatgaagatctgttccaccattgaagaactcagacggcaaaaataa

>H1N1_PB1_CY046617_A/Wisconsin/629-D01055/2009

atggatgtcaatccgactctacttttcctaaaaattccagcgcaaaatgccataagcaccacattcccttatactggagatcctccatacagccatggaacaggaacaggatacaccatggacacagtaaacagaacacaccaatactcagaaaagggaaagtggacgacaaacacagagactggtgcaccccagctcaacccgattgatggaccactacctgaggataatgaaccaagtgggtatgcacaaacagactgtgttctagaggctatggctttccttgaagaatcccacccaggaatatttgagaattcatgccttgaaacaatggaagttgttcaacaaacaagggtagataaactaactcaaggtcgccagacttatgattggacattaaacagaaatcaaccggcagcaactgcattggccaacaccatagaagtctttagatcgaatggcctaacagctaatgagtcaggaaggctaatagatttcttaaaggatgtaatggaatcaatgaacaaagaggaaatagagataacaacccactttcaaagaaaaaggagagtaagagacaacatgaccaagaagatggtcacgcaaagaacaatagggaagaaaaaacaaagactgaataagagaggctatctaataagagcactgacattaaatacgatgaccaaagatgcagagagaggcaagttaaaaagaagggctatcgcaacacctgggatgcagattagaggtttcgtatactttgttgaaactttagctaggagcatttgcgaaaagcttgaacagtctgggctcccagtagggggcaatgaaaagaaggccaaactggcaaatgttgtgagaaagatgatgactaattcacaagacacagagatttctttcacaatcactggggacaacactaagtggaatgaaaatcaaaatcctcgaatgttcctggcgatgattacatatatcaccagaaatcaacccgagtggttcagaaacatcctgagcatggcacccataatgttctcaaacaaaatggcaagactagggaaagggtacatgttcgagagtaaaagaatgaagattcgaacacaaataccagcagaaatgctagcaagcattgacctgaagtacttcaatgaaccaacaaagaagaaaattgagaaaataaggcctcttctaatagatggcacagcatcactgagtcctgggatgatgatgggcatgttcaacatgctaagtacggtcttgggagtctcgatactgaatcttggacaaaagaaatacaccaagacaatatactggtgggatgggctccaatcatccgacgattttgctctcatagtgaatgcaccaaaccatgagggaatacaagcaggagtggacagattctacaggacctgcaagttagtgggaatcaacatgagcaaaaagaagtcctatataaataagacagggacatttgaattcacaagctttttttatcgctatggatttgtggctaattttagcatggagctacccagctttggagtgtctggagtaaatgaatcagctgacatgagtattggagtaacagtgataaagaacaacatgataaacaatgaccttggacctgcaacggcccagatggctcttcaactgttcatcaaagactacagatacacatatagatgccataggggagacacacaaattcagacgagaagatcatttgagttaaagaagctgtgggatcaaacccaatcaaaggtagggctattagtatcagatggaggaccaaacttatacaatatacggaatcttcacattcctgaagtctgcttaaaatgggagctaatggatgatgattatcggggaagactttgtaatcccctgaatccctttgtcagtcataaagagattgattctgtaaacaatgctgtggtaatgccagcccatggtccagccaaaagcatggaatatgatgccgttgcaactacacattcctggattcccaagaggaatcgttctattctcaacacaagccaaaggggaattcttgaggatgaacagatgtaccagaagtgctgcaatctattcgagaaatttttccctagcagttcatataggagaccggttggaatttctagcatggtggaggccatggtgtctagggcccggattgatgccagggtcgacttcgagtctggacggatcaagaaagaagagttctctgagatcatgaagatctgttccaccattgaagaactcagacggcaaaaataa

>H1N1_PB1_CY046825_A/Wisconsin/629-D02013/2009

atggatgtcaatccgactctacttttcctaaaaattccagcgcaaaatgccataagcaccacattcccttatactggagatcctccatacagccatggaacaggaacaggatacaccatggacacagtaaacagaacacaccaatactcagaaaagggaaagtggacgacaaacacagagactggtgcaccccagctcaacccgattgatggaccactacctgaggataatgaaccaagtgggtatgcacaaacagactgtgttctagaggctatggctttccttgaagaatcccacccgggaatatttgagaattcatgccttgaaacaatggaagttgttcaacaaacaagggtagataaactaactcaaggtcgccagacttatgattggacattaaacagaaatcaaccggcagcaactgcattggccaacaccatagaagtctttagatcgaatggcctaacagctaatgagtcaggaaggctaatagatttcttaaaggatgtaatggaatcaatgaacaaagaggaaatagagataacaacccactttcaaagaaaaaggagagtaagagacaacatgaccaagaagatggtcacgcaaagaacaatagggaagaaaaaacaaagactgaataagagaggctatctaataagagcactgacattaaatacgatgaccaaagatgcagagagaggcaagttaaaaagaagggctatcgcaacacctgggatgcagattagaggtttcgtatactttgttgaaactttagctaggagcatttgcgaaaagcttgaacagtctgggctcccagtagggggcaatgaaaagaaggccaaactggcaaatgttgtgagaaagatgatgactaattcacaagacacagagatttctttcacaatcactggggacaacactaagtggaatgaaaatcaaaatcctcgaatgttcctggcgatgattacatatatcaccagaaatcaacccgagtggttcagaaacatcctgagcatggcacccataatgttctcaaacaaaatggcaagactagggagagggtacatgttcgagagtaaaagaatgaagattcgaacacaaataccagcagaaatgctagcaagcattgacctgaagtacttcaatgaatcaacaaagaagaaaattgagaaaataaggcctcttctaatagatggcacagcatcactgagtcctgggatgatgatgggcatgttcaacatgctaagtacggtcttgggagtctcgatactgaatcttggacaaaagaaatacaccaagacaatatactggtgggatgggctccaatcatccgacgattttgctctcatagtgaatgcaccaaaccatgagggaatacaagcaggagtggacagattctacaggacctgcaagttagtgggaatcaacatgagcaaaaagaagtcctatataaataagacagggacatttgaattcacaagctttttttatcgctatggatttgtggctaattttagcatggagctacccagctttggagtgtctggagtaaatgaatcagctgacatgagtattggagtaacagtgataaagaacaacatgataaacaatgaccttggacctgcaacggcccagatggctcttcaattgttcatcaaagactacagatacacatataggtgccataggggagacgcacaaattcagacgagaagatcatttgagttaaagaagctgtgggatcaaacccaatcaaaggtagggctattagtatcagatggaggaccaaacttatacaatatacggaatcttcacattcctgaagtctgcttaaaatgggagctaatggatgatgattatcggggaagactttgtaatcccctgaatccctttgtcagtcataaagagattgattctgtaaacaatgctgtggtaatgccagcccatggtccagccaaaagcatggaatatgatgccgttgcaactacacattcctggattcccaagaggaatcgttctattctcaacacaagccaaaggggaattcttgaggatgaacagatgtaccagaagtgctgcaatctattcgagaaatttttccctagcagttcatataggagaccggttggaatttctagcatggtagaggccatggtgtctagggcccggattgatgccagggtcgacttcgagtctggacggatcaagaaagaagagttctctgagatcatgaagatctgttccaccattgaagaactcagacggcaaaaataa

>H1N1_PB1_GU112096_A/Zhejiang/DTID-ZJU02/2009

atggatgtcaatccgactctacttttcctaaaaattccagcgcaaaatgccataagcaccacattcccttatactggagatcctccatacagccatggaacaggaacaggatacaccatggacacagtaaacagaacacaccaatactcagaaaagggaaagtggacgacaaacacagagactggtgcaccccagctcaacccgattgatggaccactacctgaggataatgaaccaagtgggtatgcacaaacagactgtgttctagaggctatggctttccttgaagaatcccacccaggaatatttgagaattcatgccttgaaacaatggaagttgttcaacaaacaagggtagataaactaactcaaggtcgccagacttatgattggacattaaacagaaatcaaccggcagcaactgcattggccaacaccatagaagtctttagatcgaatggcctaacagctaatgagtcaggaaggctaatagatttcttaaaggatgtaatggaatcaatgaacaaagaggaaatagagataacaacccactttcaaagaaaaaggagagtaagagacaacatgaccaagaagatggtcacgcaaagaacaatagggaagaaaaaacaaagactgaataagagaggctatctaataagagcactgacattaaatacgatgaccaaagatgcagagagaggcaaattaaaaagaagggctatcgcaacacctgggatgcagattagaggtttcgtatactttgttgaaactttagctaggagcatttgcgaaaagcttgaacagtctgggctcccagtagggggcaatgaaaagaaggccaaactggcaaatgttgtgagaaagatgatgactaattcacaagacacagagatttctttcacaatcactggggacaacactaagtggaatgaaaatcaaaatcctcgaatgttcctggcgatgattacatatatcaccagaaatcaacccgagtggttcagaaacatcctgagcatggcacccataatgttctcaaacaaaatggcaagactagggaaagggtacatgttcgagagtaaaagaatgaagattcgaacacaaataccagcagaaatgctagcaagcattgacctgaagtacttcaatgaatcaacaaagaagaaaattgagaaaataaggcctcttctaatagatggcacagcatcactgagtcctgggatgatgatgggcatgttcaacatgctaagtacggtcttgggagtctcgatattgaatcttggacaaaagaaatacaccaagacaatatactggtgggatgggctccaatcatccgacgattttgctctcatagtgaatgcaccaaaccatgagggaatacaagcaggagtggacagattctacaggacttgcaagttagtgggaatcaacatgagcaaaaagaagtcctatataaataagacagggacatttgaattcacaagctttttttatcgctatggatttgtggctaattttagcatggagctacccagctttggagtgtctggagtaaatgaatcagctgacatgagtattggagtaacagtgataaagaacaacatgataaacaatgaccttggacctgcaacggcccagatggctcttcaattgttcatcaaagactacagatacacatataggtgccataggggagacacacaaattcagacgagaagatcatttgagttaaagaagctgtgggatcaaacccaatcaaaggtagggctattagtatcagatggaggaccaaacttatacaatatacggaatcttcacattcctgaagtctgcttaaaatgggagctaatggatgatgactatcggggaagactttgtaatcccctgaatccctttgtcagtcataaagagattgattctgtaaacaatgctgtggtaatgccagcccatggtccagccaaaagcatggaatatgatgccgttgcaactacacattcctggattcccaagaggaatcgttctattctcaacacaagccaaaggggaattcttgaggatgaacagatgtaccagaagtgctgcaatctattcgagaaatttttccctagcagttcatataggagaccggttggaatttctagcatggtggaggccatggtgtctagggcccggattgatgccagggtcgacttcgagtctggacggatcaagaaagaagagttctctgagatcatgaagatctgttccaccattgaagaactcagacggcaaaaataa

>H1N1_PB1_GU136025_A/Aichi/198/2009

atggatgtcaatccgactctacttttcctaaaaattccagcgcaaaatgccataagcaccacattcccttatactggagatcctccatacagccatggaacaggaacaggatacaccatggacacagtaaacagaacacaccaatactcagaaaagggaaagtggacgacaaacacagagactggtgcaccccagctcaacccgattgatggaccactacctgaggataatgaaccaagtgggtatgcacaaacagactgtgttctagaggctatggctttccttgaagaatcccacccaggaatatttgagaattcatgccttgaaacaatggaagttgttcaacaaacaagggtagataaactaactcaaggtcgccagacttatgattggacattaaacagaaatcaaccggcagcaactgcattggccaacaccatagaagtctttagatcgaatggcctaacagctaatgagtcaggaaggctaatagatttcttaaaggatgtaatggaatcaatgaacaaagaggaaatagagataacaacccactttcaaagaaaaaggagagtaagagacaacatgaccaagaagatggtcacgcaaagaacaatagggaagaaaaaacaaagactgaataagagaggctatctaataagagcactgacattaaatacgatgaccaaagatgcagagagaggcaagttaaaaagaagggctatcgcaacacctgggatgcagattagaggtttcgtatactttgttgaaactttagctaggagcatttgcgaaaagcttgaacagtctgggctcccagtagggggcaatgaaaagaaggccaaactggcaaatgttgtgagaaagatgatgactaactcacaagacacagagatttctttcacaatcactggggacaacactaagtggaatgaaaatcaaaatcctcgaatgttcctggcgatgattacatatatcaccagaaatcaacccgagtggttcagaaacatcctgagcatggcacccataatgttctcaaacaaaatggcaaggctagggaaagggtacatgttcgagagtaaaagaatgaagattcgaacacaaataccagcagaaatgctagcaagcattgacctgaagtacttcaatgaatcaacaaagaagaaaattgagaaaataaggcctcttctaatagatggcacagcatcactgagtcctgggatgatgatgggcatgttcaacatgctaagtacggtcttgggagtctcgatactgaatcttggacaaaagaaatacaccaagacaatatactggtgggatgggctccaatcatccgacgattttgctctcatagtgaatgcaccaaaccatgagggaatacaagcaggagtggacagattctacaggacctgcaagttagtgggaatcaacatgagcaaaaagaagtcctatataaataagacagggacatttgaattcacaagctttttttatcgctatggatttgtggctaattttagcatggagctacccagctttggagtgtctggagtaaatgaatcagctgacatgagtattggagtaacagtgataaagaacaacatgataaacaatgaccttggacctgcaacggcccagatggctcttcaattgttcatcaaagactacagatacacatataggtgccataggggagacacacaaattcagacgagaagatcatttgagttaaagaagctgtgggatcaaacccaatcaaaggtagggctattagtatcagatggaggaccaaacttatacaatatacggaatcttcacatccctgaagtctgcttaaaatgggagctaatggatgatgattatcggggaagactttgtaatcccctgaatccctttgtcagtcataaagagattgattctgtaaacaatgctgtggtaatgccagcccatggtccagccaaaagcatggaatatgatgccgttgcaactacacattcctggattcccaagaggaatcgttctattctcaacacaagccaaaggggaattcttgaggatgaacagatgtaccagaagtgctgcaatctattcgagaaatttttccctagcagttcatataggagaccggttggaatttctagcatggtggaggccatggtgtctagggcccggattgatgccagggtcgacttcgagtctggacggatcaagaaagaagagttctctgagatcatgaagatctgttccaccattgaagaactcagacggcaaaaataa

>H1N1_PB1_GU136042_A/Myanmar/JP90/2009

atggatgtcaatccgactctacttttcctaaaaattccagcgcaaaatgccataagcaccacattcccttatactggagatcctccatacagccatggaacaggaacaggatacaccatggacacagtaaacagaacacaccaatactcagaaaagggaaagtggacgacaaacacagagactggtgcaccccagctcaacccgattgatggatcactacctgaggataatgaaccaagtgggtatgcacaaacagactgtgttctagaggctatggctttccttgaagaatcccacccaggaatatttgagaattcatgccttgaaacaatggaagttgttcaacaaacaagggtagataaactaactcaaggtcgccagacttatgattggacattaaacagaaatcaaccggcagcaactgcattggccaacaccatagaagtttttagatcgaatggcctaacagctaatgagtcaggaaggctaatagatttcttaaaggatgtaatggaatcaatgaacaaagaggaaatagagataacaacccactttcaaagaaaaaggagagtaagagacaacatgaccaagaagatggtcacgcaaagaacaatagggaagaaaaaacaaagactgaataagagaggctatctaataagagcactgacattaaatacgatgaccaaagatgcagagagaggcaagttaaaaagaagggctatcgcaacacctgggatgcagattagaggtttcgtatactttgttgaaactttagctaggagcatttgcgaaaagcttgaacagtctgggctcccagtagggggcaatgaaaagaaggccaaactggcaaatgttgtgagaaagatgatgactaattcacaagacacagagatttctttcacaatcactggggacaacactaagtggaatgaaaatcaaaatcctcgaatgttcctggcgatgattacatatatcaccagaaatcaacccgagtggttcagaaacatcctgagcatggcacccataatgttctcaaacaaaatggcaagactagggaaagggtacatgttcgagagtaaaagaatgaagattcgaacacaaataccagcagaaatgctagcaagcattgacctgaagtacttcaatgaatcaacaaagaagaaaattgagaaaataaggcctcttctaatagatggcacagcatcactgagtcctgggatgatgatgggcatgttcaacatgctaagtacggtcttgggagtctcgatactgaatcttggacaaaagaaatacaccaagacaatatactggtgggatgggctccaatcatccgacgattttgctctcatagtgaatgcaccaaaccatgagggaatacaagcaggagtggacagattctacaggacctgcaagttagtgggaatcaacatgagcaaaaagaagtcctatataaataagacagggacatttgaattcacaagctttttttatcgctatggatttgtggctaattttagcatggagctacccagctttggagtgtctggagtaaatgaatcagctgacatgagtattggagtaacagtgataaagaacaacatgataaacaatgaccttggacctgcaacggcccagatggctcttcaattgttcatcaaagactacagatacacatataggtgccataggggagacacacaaattcagacgagaagatcatttgagttaaagaagctgtgggatcaaacccaatcaaaggtagggctattagtatcagatggaggaccaaacttatacaatatacggaatcttcacattcctgaagtctgcttaaaatgggagctaatggatgatgattatcggggaagactttgtaatcccctgaatccctttgtcagtcataaagagattgattctgtaaacaatgctgtggtaatgccagcccatggtccagccaaaagcatggaatatgatgccgttgcaactacacattcctggattcccaagaggaatcgttctattctcaacacaagccaaaggggaattcttgaggatgaacagatgtaccagaagtgctgcaatctattcgagaaatttttccctagcagttcatataggagaccggttggaatttctagcatggtggaggccatggtgtctagggcccggattgatgccagggtcgacttcgagtctggacggatcaagaaagaagagttctctgagatcatgaagatctgttccaccattgaagaactcagacggcaaaaataa

>H1N1_PB1_CY050180_A/Mexico City/015/2009

atggatgtcaatccgactctacttttcctaaaaattccagcgcaaaatgccataagcaccacattcccttatactggagatcctccatacagccatggaacaggaacaggatacaccatggacacagtaaacagaacacaccaatactcagaaaagggaaagtggacgacaaacacagagactggtgcaccccagctcaacccgattgatggaccactacctgaggataatgaaccaagtgggtatgcacaaacagactgtgttctagaggctatggctttccttgaagaatcccacccaggaatatttgagaattcatgccttgaaacaatggaagttgttcaacaaacaagggtagataaactaactcaaggtcgccagacttatgattggacattaaacagaaatcaaccggcagcaactgcattggccaacaccatagaagtctttagatcgaatggcctaacagctaatgagtcaggaaggctaatagatttcttaaaggatgtaatggaatcaatgaacaaagaggaaatagagataacaacccactttcaaagaaaaaggagaataagagacaacatgaccaagaagatggtcacgcaaagaacaatagggaagaaaaaacaaagactgaataagagaggctatctaataagagcactgacattaaatacgatgaccaaagatgcagagagaggcaagttaaaaagaagggctatcgcaacacctgggatgcagattagaggtttcgtatactttgttgaaactttagctaggagcatttgcgaaaagcttgaacagtctgggctcccagtagggggcaatgaaaagaaggccaaactggcaaatgttgtgagaaagatgatgactaattcacaagacacagagatttctttcacaatcactggggacaacactaagtggaatgaaaatcaaaatcctcgaatgttcctggcgatgattacatatatcaccagaaatcaacccgagtggttcagaaacatcctgagcatggcacccataatgttctcaaacaaaatggcaagactagggaaagggtacatgttcgagagtaaaagaatgaagattcgaacacaaataccagcagaaatgctagcaagcattgacctgaagtacttcaatgaatcaacaaagaagaaaattgagaaaataaggcctcttctaatagatggcacagcatcactgagtcctgggatgatgatgggcatgttcaacatgctaagtacggtcttgggagtctcgatactgaatcttggacaaaagaaatacaccaagacaatatactggtgggatgggctccaatcatccgacgattttgctctcatagtgaatgcaccaaaccatgagggaatacaagcaggagtggacagattctacaggacctgcaagttagtgggaatcaacatgagcaaaaagaagtcctatataaataagacagggacatttgaattcacaagctttttttatcgctatggatttgtggctaattttagcatggagctacccagctttggagtgtctggagtaaatgaatcagctgacatgagtattggagtaacagtgataaagaacaacatgataaacaatgaccttggacctgcaacggcccagatggctcttcaattgttcatcaaagactacagatacacatataggtgccataggggagacacacaaattcagacgagaagatcatttgagttaaagaagctgtgggatcaaacccaatcaaaggtagggctattagtatcagatggaggaccaaacttatacaatatacggaatcttcacattcctgaagtctgcttaaaatgggagctaatggatgatgattatcggggaagactttgtaatcctctgaatccctttgtcagtcataaagagattgattctgtaaacaatgctgtggtaatgccagcccatggtccagccaaaagcatggaatatgatgccgttgcaactacacattcctggattcccaagaggaatcgttctattctcaacacaagccaaaggggaattcttgaggatgaacagatgtaccagaagtgctgcaatctattcgagaaatttttccctagcagttcatataggagaccggttggaatttctagcatggtggaggccatggtgtctagggcccggattgatgccagggtcgacttcgagtctggacggatcaagaaagaagagttctctgagatcatgaagatctgttccaccattgaagaactcagacggcaaaaataa

>H1N1_PB1_CY050434_A/Wisconsin/629-D02324/2009

atggatgtcaatccgactctacttttcctaaaaattccagcgcaaaatgccataagcaccacattcccttatactggagatcctccatacagccatggaacaggaacaggatacaccatggacacagtaaacagaacacaccaatactcagaaaagggaaagtggacgacaaacacagagactggtgcaccccagctcaacccgattgatggaccactacctgaggataatgaaccaagtgggtatgcacaaacagactgtgttctagaggctatggctttccttgaagaatcccacccaggaatatttgagaattcatgccttgaaacaatggaagttgttcaacaaacaagggtagataaactaactcaaggtcgccagacttatgattggacattaaacagaaatcaaccggcagcaactgcattggccaacaccatagaagtctttagatcgaatggcctaacagctaatgagtcaggaaggctaatagatttcttaaaggatgtaatggaatcaatgaacaaagaggaaatagagataacaacccactttcaaagaaaaaggagagtaagagacaacatgaccaagaagatggtcacgcaaagaacaatagggaagaaaaaacaaagactgaataagagaggctatctaataagagcactgacattaaatacgatgaccaaagatgcagagagaggcaagttaaaaagaagggctatcgcaacacctgggatgcagattagaggtttcgtatactttgttgaaactttagctaggagcatttgcgaaaagcttgaacagtctgggctcccagtagggggcaatgaaaagaaggccaaactggcaaatgttgtgagaaagatgatgactaattcacaagacacagagatttctttcacaatcactggggacaacactaagtggaatgaaaatcaaaatcctcgaatgttcctggcgatgattacatatatcaccagaaatcaacccgagtggttcagaaacatcctgagcatggcacccataatgttctcaaacaaaatggcaagactagggaaagggtacatgttcgagagtaaaagaatgaagattcgaacacaaataccagcagaaatgctagcaagcattgacctgaagtacttcaatgaatcaacaaagaagaaaattgagaaaataaggcctcttctaatagatggcacagcatcactgagtcctgggatgatgatgggcatgttcaacatgctaagtacggtcttgggagtctcgatactgaatcttggacaaaagaaatacaccaagacaatatactggtgggatgggctccaatcatccgacgattttgctctcatagtgaatgcaccaaaccatgagggaatacaagcaggagtggacagattctacaggacctgcaagttagtgggaatcaacatgagcaaaaagaagtcctatataaataagactgggacatttgaattcacaagctttttttatcgctatggatttgtggctaattttagcatggagctacccagctttggagtgtctggagtaaatgaatcagctgacatgagtattggagtaacagtgataaagaacaacatgataaacaatgaccttggacctgcaacggcccagatggctcttcaattgttcatcaaagactacagatacacatataggtgccataggggagacacacaaattcagacgagaagatcatttgagttaaagaagctgtgggatcaaacccaatcaaaggtagggctattagtatcagatggaggaccaaacttatacaatatacggaatcttcacattcctgaagtctgcttaaaatgggagctaatggatgatgattatcggggaagactttgtaatcccctgaatccctttgtcagtcataaagagattgattctgtaaacaatgctgtggtaatgccagcccatggtccagccaaaagcatggaatatgatgccgttgcaactacacattcctggattcccaagaggaatcgttctattctcaacacaagccaaaggggaattcttgaggatgaacagatgtaccagaagtgctgcaatctattcgagaaatttttccctagcagttcatataggagaccggttggaatttctagcatggtggaggccatggtgtctagggcccggattgatgccagggtcgacttcgagtctggacggatcaagaaagaagagttctctgagatcatgaagatctgttccaccattgaagaactcagacggcaaaaataa

>H1N1_PB1_CY050893_A/Mexico City/018/2009

atggatgtcaatccgactctacttttcctaaaaattccagcgcaaaatgccataagcaccacattcccttatactggagatcctccatacagccatggaacaggaacaggatacaccatggacacagtaaacagaacacaccaatactcagaaaagggaaagtggacgacaaacacagagactggtgcaccccagctcaacccgattgatggaccactacctgaggataatgaaccaagtgggtatgcacaaacagactgtgttctagaggctatggctttccttgaagaatcccacccaggaatatttgagaattcatgccttgaaacaatggaagttgttcaacaaacaagggtagataaactaactcaaggtcgccagacttatgattggacattaaacagaaatcaaccggcagcaactgcattggccaacaccatagaagtctttagatcgaatggcctaacagctaatgagtcaggaaggctaatagatttcttaaaggatgtaatggaatcaataaacaaagaggaaatagagataacaacccactttcaaagaaaaaggagagtaagagacaacatgaccaagaagatggtcacgcaaagaacaatagggaagaaaaaacaaagactgaataagagaggctatctaataagagcactgacattaaatacgatgaccaaagatgcagagagaggcaagttaaaaagaagggctatcgcaacacctgggatgcagattagaggtttcgtatactttgttgaaactttagctaggagcatttgcgaaaagcttgaacagtctgggctcccagtagggggcaatgaaaagaaggccaaactggcaaatgttgtgagaaagatgatgactaattcacaagacacagagatttctttcacaatcactggggacaacactaagtggaatgaaaatcaaaatcctcgaatgttcctggcgatgattacatatatcaccagaaatcaacccgagtggttcagaaacatcctgagcatggcacccataatgttctcaaacaaaatggcaagactagggaaagggtacatgttcgagagtaaaagaatgaagattcgaacacaaataccagcagaaatgctagcaagcattgacctgaagtacttcaatgaatcaacaaagaagaaaattgagaaaataaggcctcttctaatagatggcacagcatcactgagtcctgggatgatgatgggcatgttcaacatgctaagtacggtcttgggagtctcgatactgaatcttggacaaaagaaatacaccaagacaatatactggtgggatgggctccaatcatccgacgattttgctctcatagtgaatgcaccaaaccatgagggaatacaagcaggagtggacagattctacaggacctgcaagttagtgggaatcaacatgagcaaaaagaagtcctatataaataagacagggacatttgaattcacaagctttttttatcgctatggatttgtggctaattttagcatggagctacccagctttggagtgtctggagtaaatgaatcagctgacatgagtattggagtaacagtgataaagaacaacatgataaacaatgaccttggacctgcaacggcccagatggctcttcaattgttcatcaaagactacagatacacatataggtgccataggggagacacacaaattcagacgagaagatcatttgagttaaagaagctgtgggatcaaacccaatcaaaggtagggctattagtatcagatggaggaccaaacttatacaatatacggaatcttcacattcctgaagtctgcttaaaatgggagctaatggatgatgattatcggggaagactttgtaatcccctgaatccctttgtcagtcataaagagattgattctgtaaacaatgctgtggtaatgccagcccatggtccagccaaaagcatggaatatgatgccgttgcaactacacattcctggattcccaagaggaatcgttctattctcaacacaagccaaaggggaattcttgaggatgaacagatgtaccagaagtgctgcaatctattcgagaaatttttccctagcagttcatataggagaccggttggaatttctagcatggtggaggccatggtgtctagggcccggattgatgccagggtcgacttcgaatctggacggatcaagaaagaagagttctctgagatcatgaagatctgttccaccattgaagaactcagacggcaaaaataa

>H1N1_PB1_CY050925_A/Wisconsin/629-D02213/2009

atggatgtcaatccgactctacttttcctaaaaattccagcgcaaaatgccataagcaccacattcccttatactggagatcctccatacagccatggaacaggaacaggatacaccatggacacagtaaacagaacacaccaatactcagaaaagggaaagtggacgacaaacacagagactggtgcaccccagctcaacccgattgatggaccactacctgaggataatgaaccaagtgggtatgcacaaacagactgtgttctagaggctatggctttccttgaagaatcccacccaggaatatttgagaattcatgccttgaaacaatggaagttgttcaacaaacaagggtagataaactaactcaaggtcgccagacttatgattggacattaaacagaaatcaaccggcagcaactgcattggccaacaccatagaagtctttagatcgaatggcctaacagctaatgagtcaggaaggctaatagatttcttaaaggatgtaatggaatcaatgaacaaagaggaaatagagataacaacccactttcaaagaaaaaggagagtaagagacaacatgaccaagaagatggtcacgcaaagaacaatagggaagaaaaaacaaagactgaataagagaggctatctaataagagcactgacattaaatacgatgaccaaagatgcagagagaggcaagttaaaaagaagggctatcgcaacacctgggatgcagattagaggtttcgtatactttgttgaaactttagctaggagcatttgcgaaaagcttgaacagtctgggctcccagtagggggcaatgaaaagaaggccaaactggcaaatgttgtgagaaagatgatgactaattcacaagacacagagatttctttcacaatcactggggacaacactaagtggaatgaaaatcaaaatcctcgaatgttcctggcgatgattacatatatcaccagaaatcaacccgagtggttcagaaacatcctgagcatggcacccataatgttctcaaacaaaatggcaagactagggaaagggtacatgttcgagagtaaaagaatgaagattcgaacacaaataccagcagaaatgctagcaagcattgacctgaagtacttcaatgaatcaacaaagaagaaaattgagaaaataaggcctcttctaatagatggcacagcatcactgagtcctgggatgatgatgggcatgttcaacatgctaagtacggtcttgggagtctcgatactgaatcttggacaaaagaaatacaccaagacaatatactggtgggatgggctccaatcatctgacgattttgctctcatagtgaatgcaccaaaccatgagggaatacaagcaggagtggacagattctacaggacctgcaagttagtgggaatcaacatgagcaaaaagaagtcctatataaataagacaggracatttgaattcacaagctttttttatcgctatggatttgtggctaattttagcatggagctacccagctttggagtgtctggagtaaatgaatcagctgacatgagtattggagtaacagtgataaagaacaacatgataaacaatgaccttggacctgcaacggcccagatggctcttcaattgttcatcaaagactacagatacacatataggtgccataggggagacacacaaattcagacgagaagatcatttgagttaaagaagctgtgggatcaaacccaatcaaaggtagggctattagtatcagatggaggaccaaacttatacaatatacggaatcttcacattcctgaagtctgcttaaaatgggagctaatggatgatgattatcggggaagactttgtaatcccctgaatccctttgtcagtcataaagagattgattctgtaaacaatgctgtggtaatgccagcccatggtccagccaaaagcatggaatatgatgccgttgcaactacacattcctggattcccaagaggaatcgttctattctcaacacaagccaaaggggaattcttgaggatgaacagatgtaccagaagtgctgcaatctattcgagaaatttttccctagcagttcatataggagaccggttggaatttctagcatggtggaggccatggtgtctagggcccggattgatgccagggtcgacttcgagtctggacggatcaagaaagaagagttctctgagatcatgaagatctgttccaccattgaagaactcagacggcaaaaataa

>H1N1_PB1_CY051149_A/Wisconsin/629-D01734/2009

atggatgtcaatccgactctacttttcctaaaaattccagcgcaaaatgccataagcaccacattcccttatactggagatcctccatacagccatggaacaggaacaggatacaccatggacacagtaaacagaacacaccaatactcagaaaagggaaagtggacgacaaacacagagactggtgcaccccagctcaacccgattgatggaccactacctgaggataatgaaccaagtgggtatgcacaaacagactgtgttctagaggctatggctttccttgaagaatcccacccaggaatatttgagaattcatgccttgaaacaatggaagttgttcaacaaacaagggtagataaactaactcaaggtcgccagacttatgattggacattaaacagaaatcaaccggcagcaactgcattggccaacaccatagaagtctttagatcgaatggcctaacagctaatgagtcaggaaggctaatagatttcttaaaggatgtaatggaatcaatgaacaaagaggaaatagagataacaacccactttcaaagaaaaaggagagtaagagacaacatgaccaagaagatggtcacgcaaagaacaatagggaagaaaaaacaaagactgaataagagaggctatctaataagagcactgacattaaatacgatgaccaaagatgcagagagaggcaagttaaaaagaagggctatcgcaacacctgggatgcagattagaggtttcgtatactttgttgaaactttagctaggagcatttgcgaaaagcttgaacagtctgggctcccagtagggggcaatgaaaagaaggccaaactggcaaatgttgtgagaaagatgatgactaattcacaagacacagagatttctttcacaatcactggggacaacactaagtggaatgaaaatcaaaatcctcgaatgttcctggcgatgattacatatatcaccagaaatcaacccgagtggttcagaaacatcctgagcatggcacccataatgttctcaaacaaaatggcaagactagggaaagggtacatgttcgagagtaaaagaatgaagattcgaacacaaataccagcagaaatgctagcaagcattgacctgaagtacttcaatgaatcaacaaagaagaaaattgagaaaataaggcctcttctaatagatggcacagcatcactgagtcctgggatgatgatgggcatgttcaacatgctaagtacggtcttgggagtctcgatactgaatcttggacaaaagaaatacaccaagacaatatactggtgggatgggctccaatcatccgacgattttgctctcatagtgaatgcaccaaaccatgagggaatacaagcaggagtggacagattctacaggacctgcaaattagtgggaatcaacatgagcaaaaagaagtcctatataaataagacagggacatttgaattcacaagctttttttatcgctatggatttgtggctaattttagcatggagctacccagctttggagtgtctggagtaaatgaatcagctgacatgagtattggagtaacagtgataaagaacaacatgataaacaatgaccttggacctgcaacggcccagatggctcttcaattgttcatcaaagactacagatacacatataggtgccataggggagacacacaaattcagacgagaagatcatttgagttaaagaagctgtgggatcaaacccaatcaaaggtagggctattagtatcagatggaggaccaaacttatacaatatacggaatcttcacattcctgaagtctgcttaaaatgggagctaatggatgatgattatcggggaagactttgtaatcccctgaatccctttgtcagtcataaagagattgattctgtaaacaatgctgtggtaatgccagcccatggtccagccaaaagcatggaatatgatgccgttgcaactacacattcctggattcccaagaggaatcgttctattctcaacacaagccaaaggggaattcttgaggatgaacagatgtaccagaagtgctgcaatctattcgagaaatttttccctagcagttcatataggagaccggttggaatttctagcatggtggaggccatggtgtctagggcccggattgatgccagggtcgacttcgagtctggacggatcaagaaagaagagttctctgagatcatgaagatctgttccaccattgaagaactcagacggcaaaaataa

>H1N1_PB1_CY051205_A/Wisconsin/629-D02024/2009

atggatgtcaatccgactctacttttcctaaaaattccagcgcaaaatgccataagcaccacattcccttatactggagatcctccatacagccatggaacaggaacaggatacaccatggacacagtaaacagaacacaccaatactcagaaaagggaaagtggacgacaaacacagagactggtgcaccccagctcaacccgattgatggaccactacctgaggataatgaaccaagtgggtatgcacaaacagactgtgttctagaggctatggctttccttgaagaatcccacccaggaatatttgagaattcatgccttgaaacaatggaagttgttcaacaaacaagggtagataaactaactcaaggtcgccagacttatgattggacattaaacagaaatcaaccggcagcaactgcattggccaacaccatagaagtctttagatcgaatggcctaacagctaatgagtcaggaaggctaatagatttcttaaaggatgtaatggaatcaatgaacaaagaggaaatagagataacaacccactttcaaagaaaaaggagagtaagagacaacatgaccaagaagatggtcacgcaaagaacaatagggaagaaaaaacaaagactgaataagagaggctatctaataagagcactgacattaaatacgatgaccaaagatgcagagagaggcaagttaaaaagaagggctatcgcaacacctgggatgcagattagaggtttcgtatactttgttgaaactttagctaggagcatttgcgaaaagcttgaacagtctgggctcccagtagggggcaatgaaaagaaggccaaactggcaaatgttgtgagaaagatgatgactaattcacaagacacagagatttctttcacaatcactggggacaacactaagtggaatgaaaatcaaaatcctcgaatgttcctggcgatgattacatatatcaccagaaatcaacccgagtggttcagaaacatcctgagcatggcacccataatgttctcaaacaaaatggcaagactagggaaagggtacatgttcgagagtaaaagaatgaagattcgaacacaaataccagcagaaatgctagcaagcattgacctgaagtacttcaatgaatcaacaaagaagaaaattgagaaaataaggcctcttctaatagatggcacagcatcactgagtcctgggatgatgatgggcatgttcaacatgctaagtacggtcttgggagtctcgatactgaatcttggacaaaagaaatacaccaagacaatatactggtgggatgggctccaatcatctgacgattttgctctcatagtgaatgcaccaaaccatgagggaatacaagcaggagtggacagattctacaggacctgcaagttagtgggaatcaacatgagcaaaaagaagtcctatataaataagacagggacatttgaattcacaagctttttttatcgctatggatttgtggctaattttagcatggagctacccagctttggagtgtctggagtaaatgaatcagctgacatgagtattggagtaacagtgataaagaacaacatgataaacaatgaccttggacctgcaacggcccagatggctcttcaattgttcatcaaagactacagatacacatataggtgccataggggagacacacaaattcagacgagaagatcatttgagttaaagaagctgtgggatcaaacccaatcaaaggtagggctattagtgtcagatggaggaccaaacttatacaatatacggaatcttcacattcctgaagtctgcttaaaatgggagctaatggatgatgattatcggggaagactttgtaatcccctgaatccctttgtcagtcataaagagattgattctgtaaacaatgctgtggtaatgccagcccatggtccagccaaaagcatggaatatgatgccgttgcaactacacattcctggattcccaagaggaatcgttctattctcaacacaagccaaaggggaattcttgaggatgaacaggtgtaccagaagtgctgcaatctattcgagaaatttttccctagcagttcatataggagaccggttggaatttctagcatggtggaggccatggtgtctagggcccggattgatgccagggtcgacttcgagtctggacggatcaagaaagaagagttctctgagatcatgaagatctgttccaccattgaagaactcagacggcaaaaataa

>H1N1_PB1_CY051996_A/Norway/3364-2/2009

atggatgtcaatccgactctacttttcctaaaaattccagcgcaaaatgccataagcaccacattcccttatactggagatcctccatacagccatggaacaggaacaggatacaccatggacacagtaaacagaacacaccaatactcagaaaagggaaagtggacgacaaacacagagactggtgcaccccagctcaacccgattgatggaccactacctgaggataatgaaccaagtgggtatgcacaaacagactgtgttctagaggctatggctttccttgaagaatcccacccaggaatatttgagaattcatgccttgaaacaatggaagttgttcaacaaacaagggtagataaactaactcaaggtcgccagacttatgattggacattaaacagaaatcaaccggcagcaactgcattggccaacaccatagaagtctttagatcgaatggcctaacagctaatgagtcaggaaggctaatagatttcttaaaggatgtaatggaatcaatgaacaaagaggaaatagagataacaacccactttcaaagaaaaaggagagtaagagacaacatgaccaagaagatggtcacgcaaagaacaatagggaagaaaaaacaaagactgaacaagagaggctatctaataagagcactgacattaaatacgatgaccaaagatgcagagagaggcaagttaaaaagaagggctatcgcaacacctgggatgcagattagaggtttcgtatactttgttgaaactttagctaggagcatttgcgaaaagcttgaacagtctgggctcccagtagggggcaatgaaaagaaggccaaactggcaaatgttgtgagaaagatgatgactaattcacaagacacagagatttctttcacaatcactggggacaacactaagtggaatgaaaatcaaaatcctcgaatgttcctggcgatgattacatatatcaccagaaatcaacccgagtggttcagaaacatcctgagcatggcacccataatgttctcaaacaaaatggcaagactagggaaagggtacatgttcgagagtaaaagaatgaagattcgaacacaaataccagcagaaatgctagcaagcattgacctgaagtacttcaatgaatcaacaaagaagaaaattgagaaaataaggcctcttctaatagatggcacagcatcactgagtcctggaatgatgatgggcatgttcaacatgctaagtacggtcttgggagtctcgatactgaatcttggacaaaagaaatacaccaagacaatatactggtgggatgggctccaatcatccgacgattttgctctcatagtgaatgcaccaaaccatgagggaatacaagcaggagtggacagattctacaggacctgcaagttagtgggaatcaacatgagcaaaaagaagtcctatataaataagacagggacatttgaattcacaagctttttttatcgctatggatttgtggctaattttagcatggagctacccagctttggagtgtctggagtaaatgaatcagctgacatgagtattggagtaacagtgataaagaacaacatgataaacaatgaccttggacctgcaacggcccagatggctcttcaattgttcatcaaagactacagatacacatataggtgccataggggagacacacaaattcagacgagaagatcatttgagttaaagaagctgtgggatcaaacccaatcaaaggtagggctattagtatcagatggaggaccaaacttatacaatatacggaatcttcacattcctgaagtctgcttaaaatgggagctaatggatgatgattatcggggaagactttgtaatcccctgaatccctttgtcagtcataaagagattgattctgtaaacaatgctgtggtaatgccagcccatggtccagccaaaagcatggaatatgatgccgttgcaactacacattcctggattcccaagaggaatcgttctattctcaacacaagccaaaggggaattcttgaggatgaacagatgtaccagaagtgctgcaatctattcgagaaatttttccctagcagttcatataggagaccggttggaatttctagcatggtggaggccatggtgtctagggcccggattgatgccagggtcgacttcgagtctggacggatcaagaaagaagagttctctgagatcatgaagatctgttccaccattgaagaactcagacggcaaaaataa

>H1N1_PB1_CY052413_A/Texas/43132503/2009

atggatgtcaatccgactctacttttcctaaaaattccagcgcaaaatgccataagcaccacattcccttatactggagatcctccatacagccatggaacaggaacaggatacaccatggacacagtaaacagaacacaccaatactcagaaaagggaaagtggacgacaaacacagagactggtgcaccccagctcaacccgattgatggaccactacctgaggataatgaaccaagtgggtatgcacaaacagactgtgttctagaggctatggctttccttgaagaatcccacccaggaatatttgagaattcatgccttgaaacaatggaagttgttcaacaaacaagggtagataaactaactcaaggtcgccagacttatgattggacattaaacagaaatcaaccggcagcaactgcattggccaacaccatagaagtctttagatcgaatggcctaacagctaatgagtcaggaaggctaatagatttcttaaaggatgtaatggaatcaatgaacaaagaggaaatagagataacaacccactttcaaagaaaaaggagagtaagagacaacatgaccaagaagatggtcacacaaagaacaatagggaagaaaaaacaaagactgaataagagaggctatctaataagagcactgacattaaatacgatgaccaaagatgcagagagaggcaagttaaaaagaagggctatcgcaacacctgggatgcagattagaggtttcgtatactttgttgaaactttagctaggagcatttgcgaaaagcttgaacagtctgggctcccagtagggggcaatgaaaagaaggccaaactggcaaatgttgtgagaaagatgatgactaattcacaagacacagagatttctttcacaatcactggggacaacactaagtggaatgaaaatcaaaatcctcgaatgttcctggcgatgattacatatatcaccagaaatcaacccgagtggttcagaaacatcctgagcatggcacccataatgttctcaaacaaaatggcaagactagggaaagggtacatgttcgagagtaaaagaatgaagattcgaacacaaataccagcagaaatgctagcaagcattgacctgaagtacttcaatgaatcaacaaagaagaaaattgagaaaataaggcctcttctaatagatggcacagcatcactgagtcctgggatgatgatgggcatgttcaacatgctaagtacggtcttgggagtctcgatactgaatcttggacaaaagaaatacaccaagacaatatactggtgggatgggctccaatcatccgacgattttgctctcatagtgaatgcaccaaaccatgagggaatacaagcaggagtggacagattctacagaacctgcaagttagtgggaatcaacatgagcaaaaagaagtcctatataaataagacagggacatttgaattcacaagctttttttatcgctatggatttgtggctaattttagcatggagctacccagctttggagtgtctggagtaaatgaatcagctgacatgagtattggagtaacagtgataaagaacaacatgataaacaatgaccttggacctgcaacggcccagatggctcttcaattgttcatcaaagactacagatacacatataggtgccataggggagacacacaaattcagacgagaagatcatttgagttaaagaagctgtgggatcaaacccaatcaaaggtagggctattagtatcagatggaggaccaaacttatacaatatacggaatcttcacattcctgaagtctgcttaaaatgggagctaatggatgatgattatcggggaagactttgtaatcccctgaatccctttgtcagtcataaagagattgattctgtaaacaatgctgtggtaatgccagcccacggtccagccaaaagcatggaatatgatgccgttgcaactacacattcctggattcccaagaggaatcgttctattctcaacacaagccaaaggggaattcttgaggatgaacagatgtaccagaagtgttgcaatctattcgagaaatttttccctagcagttcatataggagaccggttggaatttctagcatggtggaggccatggtgtctagggcccggattgatgccagggtcgacttcgagtctggacggatcaagaaagaagagttctctgagatcatgaagatctgttccaccattgaagaactcagacggcaaaaataa

>H1N1_PB1_CY052557_A/Texas/45122774/2009

atggatgtcaatccgactctacttttcctaaaaattccagcgcaaaatgccataagcaccacattcccttatactggagatcctccatacagccatggaacaggaacaggatacaccatggacacagtaaacagaacacaccaatactcagaaaagggaaagtggacgacaaacacagagactggtgcaccccagctcaacccgattgatggaccactacctgaggataatgaaccaagtgggtatgcacaaacagactgtgttctagaggctatggctttccttgaagaatcccacccaggaatatttgagaattcatgccttgaaacaatggaagttgttcaacaaacaagggtagataaactaactcaaggtcgccagacttatgattggacattaaacagaaatcaaccggcagcaactgcattggccaacaccatagaagtctttagatcgaatggcctaacagctaatgagtcaggaaggctaatagatttcttaaaggatgtaatggaatcaatgaacaaagaggaaatagagataacaacccactttcaaagaaaaaggagagtaagagacaacatgaccaagaagatggtcacgcaaagaacaatagggaagaaaaaacaaagactgaataagagaggctatctaataagagcactgacattaaatacgatgaccaaagatgcagagagaggcaagttaaaaagaagggctatcgcaacacctgggatgcagattagaggtttcgtatactttgttgaaactttagctaggagcatttgcgaaaagcttgaacagtctgggctcccagtagggggcaatgaaaagaaggccaaactggcaaatgttgtgagaaagatgatgactaattcacaagacacagagatttctttcacaatcactggggacaacactaagtggaatgaaaatcaaaatcctcgaatgttcctggcgatgattacatatatcaccagaaatcaacccgagtggttcagaaacatcctgagcatggcacccataatgttctcaaacaaaatggcaagactagggaaagggtacatgttcgagagtaaaagaatgaagattcgaacacaaataccagcagaaatgctagcaagcattgacctgaagtacttcaatgaatcaacaaagaagaaaattgagaaaataaggcctcttctaatagatggcacagcatcactgagccctgggatgatgatgggcatgttcaacatgctaagtacggtcttgggagtctcgatactgaatcttggacaaaagaaatacaccaagacagtatactggtgggatgggctccaatcatccgacgattttgctctcatagtgaatgcaccaaaccatgagggaatacaagcaggagtggacagattctacaggacctgcaagttagtgggaatcaacatgagcaaaaagaagtcctatataaataagacagggacatttgaattcacaagctttttttatcgctatggatttgtggctaattttagcatggagctacccagctttggagtgtctggagtaaatgaatcagctgacatgagtattggagtaacagtgataaagaacaacatgataaacaatgaccttggacctgcaacggcccagatggctcttcaattgttcatcaaagactacagatacacatataggtgccataggggagacacacaaattcagacgagaagatcatttgagttaaagaagctgtgggaccaaacccaatcaaaggtagggctattagtatcagatggaggaccaaacttatacaatatacggaatcttcacattcctgaagtctgcttaaaatgggagctaatggatgatgattatcggggaagactttgtaatcccctgaatccctttgtcagtcataaagagattgattctgtaaacaatgctgtggtaatgccagcccatggtccagccaaaagcatggaatatgatgccgttgcaactacacattcctggattcccaagaggaatcgttctattctcaacacaagccaaaggggaattcttgaggatgaacagatgtaccagaagtgctgcaatctattcgaggaatttttccctagcagttcatataggagaccggttggaatttctagcatggtggaggccatggtgtctagggcccggattgatgccagggtcgacttcgagtctggacggatcaagaaagaagagttctctgagatcatgaagatctgttccaccattgaagaactcagacggcaaaaataa

>H1N1_PB1_CY052629_A/Texas/44302167/2009

atggatgtcaatccgactctacttttcctaaaaattccagcgcaaaatgccataagcaccacattcccttatactggagatcctccatacagccatggaacaggaacaggatacaccatggacacagtaaacagaacacaccaatactcagaaaagggaaagtggacgacaaacacagagactggtgcaccccagctcaacccgattgatggaccactacctgaggataatgaaccaagtgggtatgcacaaacagactgtgttctagaagctatggctttccttgaagaatcccacccaggaatatttgagaattcatgccttgaaacaatggaagttgttcaacaaacaagggtagataaactaactcaaggtcgccagacttatgattggacattaaacagaaatcaaccggcagcaactgcattggccaacaccatagaagtctttagatcgaatggcctaacagctaatgagtcaggaaggctaatagatttcttaaaggatgtaatggaatcaatgaacaaagaggaaatagagataacaacccactttcaaagaaaaaggagagtaagagacaacatgaccaagaagatggtcacgcaaagaacaatagggaagaaaaaacaaagactgaataagagaggctatctaataagagcactgacattaaatacgatgaccaaagatgcagagagaggcaagttaaaaagaagggctatcgcaacacctgggatgcagattagaggtttcgtatactttgttgaaactttagctaggagcatttgcgaaaagcttgaacagtctgggctcccagtagggggcaatgaaaagaaggccaaactggcaaatgttgtgagaaagatgatgactaattcacaagacacagagatttctttcacaatcactggggacaacactaagtggaatgaaaatcaaaatcctcgaatgttcctggcgatgattacatatatcaccagaaatcaacccgagtggttcagaaacatcctgagcatggcacccataatgttctcaaacaaaatggcaagactagggaaagggtacatgttcgagagtaaaagaatgaagattcgaacacaaataccagcagaaatgctagcaagcattgacctgaagtacttcaatgaatcaacaaagaagaaaattgagaaaataaggcctcttctaatagatggcacagcatcactgagtcctgggatgatgatgggcatgttcaacatgctaagtacggtcttgggagtctcgatactgaatcttggacaaaagaaatacaccaagacaatatattggtgggatgggctccaatcatccgacgattttgctctcatagtgaatgcaccaaaccatgagggaatacaagcaggagtggacagattctacaggacctgcaagttagtgggaatcaacatgagcaaaaagaagtcctatataaataagacagggacatttgaattcacaagctttttttatcgctatggatttgtggctaattttagcatggagctacccagctttggagtgtctggagtaaatgaatcagctgacatgagtattggagtaacagtgataaagaacaacatgataaacaatgaccttggacctgcaacggcccagatggctcttcaattgttcatcaaagactacagatacacatataggtgccataggggggacacacaaattcagacgagaagatcatttgagttaaagaagctgtgggatcaaacccaatcaaaggtagggctattagtatcagatggaggaccaaacttatacaatatacggaatcttcacattcctgaagtctgcttaaaatgggagctaatggatgatgattatcggggaagactttgtaatcccctgaatccctttgtcagtcataaagagattgattctgtaaacaatgctgtggtaatgccagcccatggtccagccaaaagcatggaatatgatgccgttgcaactacacattcctggattcccaagaggaatcgttctattctcaacacaagccaaaggggaattcttgaggatgaacagatgtaccagaagtgctgcaatctattcgagaaatttttccctagcagttcatataggagaccggttggaatttctagcatggtggaggccatggtgtctagggcccggattgatgccagggtcgacttcgagtctggacggatcaagaaagaagagttctctgagatcatgaagatctgttccaccattgaagaactcagacggcaaaaataa

>H1N1_PB1_CY052701_A/Texas/45083819/2009

atggatgtcaatccgactctacttttcctaaaaattccagcgcaaaatgccataagcaccacattcccttatactggagatcctccatacagccatggaacaggaacaggatacaccatggacacagtaaacagaacacaccaatactcagaaaagggaaagtggacgacaaacacagagactggtgcaccccagctcaacccgattgatggaccactacctgaggataatgaaccaagtgggtatgcacaaacagactgtgttctagaggctatggctttccttgaagaatcccacccaggaatatttgagaattcatgccttgaaacaatggaagttgttcaacaaacaagggtagataaactaactcaaggtcgccagacttatgattggacattaaacagaaatcaaccggcagcaactgcattggccaacaccatagaagtctttagatcgaatggcctaacagctaatgagtcaggaaggctaatagatttcttaaaggatgtaatggaatcaatgaacaaagaggaaatagagataacaacccactttcaaagaaaaaggagagtaagagacaacatgaccaagaagatggtcacgcaaagaacaatagggaagaaaaaacaaagactgaataagagaggctatctaataagagcactgacattaaatacgatgaccaaagatgcagagagaggcaagttaaaaagaagggctatcgcaacacctgggatgcagattagaggtttcgtatactttgttgaaactttagctaggagcatttgcgaaaagcttgaacagtctgggctcccagtagggggcaatgaaaagaaggccaaactggcaaatgttgtgagaaagatgatgactaattcacaagacacagagatttctttcacaatcactggggacaacactaagtggaatgaaaatcaaaatcctcgaatgttcctggcgatgattacatatatcaccagaaatcaacccgagtggttcagaaacatcctgagcatggcacccataatgttctcaaacaaaatggcaagactagggaaagggtacatgttcgagagtaaaagaatgaagattcgaacacaaataccagcagaaatgctagcaagcattgacctgaagtacttcaatgaatcaacaaagaagaaaattgagaaaataaggcctcttctaatagatggcacagcatcactgagtcctgggatgatgatgggcatgttcaacatgctaagtacggtcttgggagtctcgatactgaatcttggacaaaagaaatacaccaagacagtatactggtgggatgggctccaatcatccgacgattttgctctcatagtgaatgcaccaaaccatgagggaatacaagcaggagtggacagattctacaggacctgcaagttagtgggaatcaacatgagcaaaaagaagtcctatataaataagacagggacatttgaattcacaagctttttttatcgctatggatttgtggctaattttagcatggagctacccagctttggagtgtctggagtaaatgaatcagctgacatgagtattggagtaacagtgataaagaacaacatgataaacaatgaccttggacctgcaacggcccagatggctcttcaattgttcatcaaagactacagatacacatataggtgccataggggagacacacaaattcagacgagaagatcatttgagttaaagaagctgtgggaccaaacccaatcaaaggtagggctattagtatcagatggaggaccaaacttatacaatatacggaatcttcacattcctgaagtctgcttaaaatgggagctaatggatgatgattatcggggaagactttgtaatcccctgaatccctttgtcagtcataaagagattgattctgtaaacaatgctgtggtaatgccagcccatggtccagccaaaagcatggaatatgatgccgttgcaactacacattcctggattcccaagaggaatcgttctattctcaacacaagccaaaggggaattcttgaggatgaacagatgtaccagaagtgctgcaatctattcgagaaatttttccctagcagttcatataggagaccggttggaatttctagcatggtggaggccatggtgtctagggcccggattgatgccagggtcgacttcgagtctggacggatcaagaaagaagagttctctgagatcatgaagatctgttccaccattgaagaactcagacggcaaaaataa

>H1N1_PB1_CY052909_A/Texas/45032708/2009

atggatgtcaatccgactctacttttcctaaaaattccagcgcaaaatgccataagcaccacattcccttatactggagatcctccatacagccatggaacaggaacaggatacaccatggacacagtaaacagaacacaccaatactcagaaaagggaaagtggacgacaaacacagagactggtgcaccccagctcaacccgattgatggaccactacctgaggataatgaaccaagtgggtatgcacaaacagactgtgttctagaggctatggctttccttgaagaatcccacccgggaatatttgagaattcatgccttgaaacaatggaagttgttcaacaaacaagggtagataaactaactcaaggtcgccagacttatgattggacattaaacagaaatcaaccggcagcaactgcattggccaacaccatagaagtctttagatcgaatggcctaacagctaatgagtcaggaaggctaatagatttcttaaaggatgtaatggaatcaatgaacaaagaggaaatagagataacaacccactttcaaagaaaaaggagagtaagagacaacatgaccaagaagatggtcacgcaaagaacaatagggaagaaaaaacaaagactgaataagagaggctatctaataagagcactgacattaaatacgatgaccaaagatgcagagagaggcaagttaaaaagaagggctatcgcaacacctgggatgcagattagaggtttcgtatactttgttgaaactttagctaggagcatttgcgaaaagcttgaacagtctgggctcccagtagggggcaatgaaaagaaggccaaactggcaaatgttgtgagaaagatgatgactaattcacaagacacagagatttctttcacaatcactggggacaacactaagtggaatgaaaatcaaaatcctcgaatgttcctggcgatgattacatatatcaccagaaatcaacccgagtggttcagaaacatcctgagcatggcacccataatgttctcaaacaaaatggcaagactagggagagggtacatgttcgagagtaaaagaatgaagattcgaacacaaataccagcagaaatgctagcaagcattgacctgaagtacttcaatgaatcaacaaagaagaaaattgagaaaataaggcctcttctaatagatggcacagcatcactgagtcctgggatgatgatgggcatgttcaacatgctaagtacggtcttgggagtctcgatactgaatcttggacaaaagaaatacaccaagacaatatactggtgggatgggctccaatcatccgacgattttgctctcatagtgaatgcaccaaaccatgagggaatacaagcaggagtggacagattctacaggacctgcaagttagtgggaatcaacatgagcaaaaagaagtcctatataaataagacagggacatttgaattcacaagctttttttatcgctatggatttgtggctaattttagcatggagctacccagctttggagtgtctggagtaaatgaatcagctgacatgagtattggagtaacagtgataaagaacaacatgataaacaatgaccttggacctgcaacggcccagatggctcttcaattgttcatcaaagactacagatacacatataggtgccataggggagacacacaaattcagacgagaagatcatttgagttaaagaagctgtgggatcaaacccaatcaaaggtagggctattagtatcagatggaggaccaaacttatacaatatacggaatcttcacattcctgaagtctgcttaaaatgggagctaatggatgatgattatcggggaagactttgtaatcccctgaatccctttgtcagtcataaagagattgattctgtaaacaatgctgtggtaatgccagcccatggtccagccaaaagcatggaatatgatgccgttgcaactacacattcctggattcccaagaggaatcgttctattctcaacacaagccaaaggggaattcttgaggatgaacagatgtaccagaagtgctgcaatctattcgagaaatttttccctagcagttcatataggagaccggttggaatttccagcatggtggaggccatggtgtctagggcccggattgatgccagggtcgacttcgagtctggacggatcaagaaagaagagttctctgagatcatgaagatctgttccaccattgaagaactcagacggcaaaaataa

>H1N1_PB1_CY053133_A/Houston/20OS/2009

atggatgtcaatccgactctacttttcctaaaaattccagcgcaaaatgccataagcaccacattcccttatactggagatcctccatacagccatggaacaggaacaggatacaccatggacacagtaaacagaacacaccaatactcagaaaagggaaagtggacgacaaacacagagactggtgcaccccagctcaacccgattgatggaccactacctgaggataatgaaccaagtgggtatgcacaaacagactgtgttctagaggctatggctttccttgaagaatcccacccaggaatatttgagaattcatgccttgaaacaatggaagtcgttcaacaaacaagggtagataaactaactcaaggtcgccagacttatgattggacattaaacagaaatcaaccggcagcaactgcattggccaacaccatagaagtctttagatcgaatggcctaacagctaatgagtcaggaaggctaatagatttcttaaaggatgtaatggaatcaatgaacaaagaggaaatagagataacaacccactttcaaagaaaaaggagagtaagagacaacatgaccaagaagatggtcacgcaaagaacaatagggaagaaaaaacaaagactgaataagagaggctatctaataagagcactgacattaaatacgatgaccaaagatgcagagagaggcaaattaaaaagaagggctatcgcaacacctgggatgcagattagaggtttcgtatactttgttgaaactttagctaggagcatttgcgaaaagcttgaacagtctgggctcccagtagggggcaatgaaaagaaggccaaactggcaaatgttgtgagaaagatgatgactaattcacaagacacagagatttctttcacaatcactggggacaacactaagtggaatgaaaatcaaaatcctcgaatgttcctggcgatgattacatatatcaccagaaatcaacccgagtggttcagaaacatcctgagcatggcacccataatgttctcaaacaaaatggcaagactagggaaagggtacatgttcgagagtaaaagaatgaagattcgaacacaaataccagcagaaatgctagcaagcattgacctgaagtacttcaatgaatcaacaaagaagaaaattgagaaaataaggcctcttctaatagatggcacagcatcactgagtcctgggatgatgatgggcatgttcaacatgctaagtacggtcttgggagtctcgatactgaatcttggacaaaagaaatacaccaagacaatatactggtgggatgggctccaatcgtccgacgattttgctctcatagtgaatgcaccaaaccatgagggaatacaagcaggagtggacagattctacaggacctgcaagttagtgggaatcaacatgagcaaaaagaagtcctatataaataagacagggacatttgaattcacaagctttttttatcgctatggatttgtggctaattttagcatggagctacccagctttggagtgtctggagtaaatgaatcagctgacatgagtattggagtaacagtgataaagaacaacatgataaacaatgaccttggacctgcaacggcccagatggctcttcaattgttcatcaaagactacagatacacatataggtgccataggggagacacacaaattcagacgagaagatcatttgagttaaagaagctgtgggatcaaacccaatcaaaggtagggctattagtatcagatggaggaccaaacttatacaatatacggaatcttcacattcctgaagtctgcttaaaatgggagctaatggatgatgattatcggggaagactttgtaatcccctgaatccctttgtcagtcataaagagattgattctgtaaacaatgctgtggtaatgccagcccatggtccagccaaaagcatggaatatgatgccgttgcaactacacattcttggattcccaagaggaatcgttctattctcaacacaagccaaaggggaattcttgaggatgaacagatgtaccagaagtgctgcaatctattcgagaaatttttccctagcagttcatataggagaccggttggaatttctagcatggtggaggccatggtgtctagggcccggattgatgccagggtcgacttcgagtctggacggatcaagaaagaagagttctctgagatcatgaagatctgttccaccattgaagaactcagacggcaaaaataa

>H1N1_PB1_CY052184_A/New York/4881/2009

atggatgtcaatccgactctacttttcctaaaaattccagcgcaaaatgccataagcaccacattcccttatactggagatcctccatacagccatggaacaggaacaggatacaccatggacacagtaaacagaacacaccaatactcagaaaagggaaagtggacgacaaacacagagactggtgcaccccagctcaacccgattgatggaccactacctgaggataatgaaccaagtgggtatgcacaaacagactgtgttctagaggctatggctttccttgaagaatcccacccaggaatatttgagaactcatgccttgaaacaatggaagttgttcaacaaacaagggtagataaactaactcaaggtcgccagacttatgattggacattaaacagaaatcaaccggcagcaactgcattggccaacaccatagaagtctttagatcgaatggcctaacagctaatgagtcaggaaggctaatagatttcttaaaggatgtaatggaatcaatgaacaaagaggaaatagagataacaacccactttcaaagaaaaaggagagtaagagacaacatgaccaagaagatggtcacgcaaagaacaatagggaagaaaaaacaaagactgaataagagaggctatctaataagagcactgacattaaatacgatgaccaaagatgcagagagaggcaagttaaaaagaagggctatcgcaacacctgggatgcagattagaggtttcgtatactttgttgaaactttagctaggagcatttgcgaaaagcttgaacagtctgggctcccagtagggggcaatgaaaagaaggccaaactggcaaatgttgtgagaaagatgatgactaattcacaagacacagagatttctttcacaatcactggggacaacactaagtggaatgaaaatcaaaatcctcgaatgttcctggcgatgattacatatatcaccagaaatcaacccgagtggttcagaaacatcctgagcatggcacccataatgttctcaaacaaaatggcaagactagggaaagggtacatgttcgagagtaaaagaatgaagattcgaacacaaataccagcagaaatgctagcaagcattgacctgaagtacttcaatgaatcaacaaagaagaaaattgagaaaataaggcctcttctaatagatggcacagcatcactgagtcctgggatgatgatgggcatgttcaacatgctaagtacggtcttgggagtctcgatactgaatcttggacaaaagaaatacaccaagacaatatactggtgggatgggctccaatcatccgacgattttgctctcatagtgaatgcaccaaaccatgagggaatacaagcaggagtggacagattctacaggacctgcaagttagtgggaatcaacatgagcaaaaagaagtcctatataaataagacagggacatttgaattcacaagctttttttatcgctatggatttgtggctaattttagcatggagctacccagctttggagtgtctggagtaaatgaatcagctgacatgagtattggagtaacagtgataaagaacaacatgataaacaatgaccttggacctgcaacggcccagatggctcttcaattgttcatcaaagactacagatacacatataggtgccataggggagacacacaaattcagacgagaagatcttttgagttaaagaagctgtgggatcaaacccaatcaaaggtagggctattagtatcagatggaggaccaaacttatacaatatacggaatcttcacattcctgaagtctgcttaaaatgggagctaatggatgatgattatcggggaagactttgtaatcccctgaatccctttgtcagtcataaagagattgattctgtaaacaatgctgtggtaatgccagcccatggtccagccaaaagcatggaatatgatgccgttgcaactacacattcctggattcccaagaggaatcgttctattctcaacacaagccaaaggggaattcttgaggatgaacagatgtaccagaagtgctgcaatctattcgagaaatttttccctagcagttcatataggagaccggttggaatttctagcatggtggaggccatggtgtctagggcccggattgatgccagggtcgacttcgagtctggacggatcaagaaagaagagttctctgagatcatgaagatctgttccaccattgaagaactcagacggcaaaaataa

>H1N1_PB1_CY052280_A/Texas/42151049/2009

atggatgtcaatccgactctacttttcctaaaaattccagcgcaaaatgccataagcaccacattcccttatactggagatcctccatacagccatggaacaggaacaggatacaccatggacacagtaaacagaacacaccaatactcagaaaagggaaagtggacgacaaacacagagactggtgcaccccagctcaacccgattgatggaccactacctgaggataatgaaccaagtgggtatgcacaaacagactgtgttctagaggctatggctttccttgaagaatcccacccaggaatatttgagaattcatgccttgaaacaatggaagttgttcaacaaacaagggtagataagctaactcaaggtcgccagacttatgattggacattaaacagaaatcaaccggcagcaactgcattggccaacaccatagaagtctttagatcgaatggcctaacagctaatgagtcaggaaggctaatagatttcttaaaggatgtaatggaatcaatgaacaaagaggaaatagagataacaacccactttcaaagaaaaaggagagtaagagacaacatgaccaagaagatggtcacacaaagaacaatagggaagaaaaaacaaagactgaataagagaggctatctaataagagcactgacattaaatacgatgaccaaagatgcagagagaggcaagttaaaaagaagggctatcgcaacacctgggatgcagattagaggtttcgtatactttgttgaaactttagctaggagcatttgcgaaaagcttgaacagtctgggctcccagtagggggcaatgaaaagaaggccaaactggcaaatgttgtgagaaagatgatgactaattcacaagacacagagatttctttcacaatcactggggacaacactaagtggaatgaaaatcaaaatcctcgaatgttcctggcgatgattacatatatcaccagaaatcaacccgagtggttcagaaacatcctgagcatggcacccataatgttctcaaacaaaatggcaagactagggaaagggtacatgttcgagagtaaaagaatgaagattcgaacacaaataccagcagaaatgctagcaagcattgacctgaagtacttcaatgaatcaacaaagaagaaaattgagaaaataaggcctcttctaatagatggcacagcatcactgagtcctgggatgatgatgggcatgttcaacatgctaagtacggtcttgggagtctcgatactgaatcttggacaaaagaaatacaccaagacaatatactggtgggatgggctccaatcatccgacgattttgctctcatagtgaatgcaccaaaccatgagggaatacaagcaggagtggacagattctacaggacctgcaagttagtgggaatcaacatgagcaaaaagaagtcctatataaataagacagggacatttgaattcacaagctttttttatcgctatggatttgtggctaattttagcatggagctacccagctttggagtgtctggagtaaatgaatcagctgacatgagtattggagtaacagtgataaagaacaacatgataaacaatgaccttggacctgcaacggcccagatggctcttcaattgttcatcaaagactacagatacacatataggtgccataggggagacacacaaattcagacgagaagatcatttgagttaaagaagctgtgggatcaaacccaatcaaaggtagggctattagtatcagatggaggaccaaacttatacaatatacggaatcttcacattcctgaagtctgcttaaaatgggagctaatggatgatgattatcggggaagactttgtaatcccctgaatccctttgtcagtcataaagagattgattctgtaaacaatgctgtggtaatgccagcccatggtccagccaaaagcatggaatatgatgccgttgcaactacacattcctggattcccaagaggaatcgttctattctcaacacaagccaaaggggaattcttgaggatgaacagatgtaccagaagtgttgcaatctattcgagaaatttttccctagcagttcatataggagaccggttggaatttctagcatggtggaggccatggtgtctagggcccggattgatgccagggtcgacttcgagtctggacggatcaagaaagaagagttctctgagatcatgaagatctgttccaccattgaagaactcagacggcaaaaataa

>H1N1_PB1_CY054636_A/Russia/12/2009

atggatgtcaatccgactctacttttcctaaaaattccagcgcaaaatgccataagcaccacattcccttatactggagatcctccatacagccatggaacaggaacaggatacaccatggacacagtaaacagaacacaccaatactcagaaaagggaaagtggacgacaaacacagagactggtgcaccccagctcaacccgattgatggaccactacctgaggataatgaaccaagtgggtatgcacaaacagactgtgttctagaggctatggctttccttgaagaatcccacccaggaatatttgagaattcatgccttgaaacaatggaagttgttcaacaaacaagggtagataaactaactcaaggtcgccagacttatgattggacattaaacagaaatcaaccggcagcaactgcattggccaacaccatagaagtctttagatcgaatggcctaacagctaatgagtcaggaaggctaatagatttcttaaaggatgtaatggaatcaatgaacaaagaggaaatagagataacaacccactttcaaagaaaaaggagagtaagagacaacatgaccaagaagatggtcacgcaaagaacaatagggaagaaaaaacaaagactgaataagagaggctatctaataagagcactgacattaaatacgatgaccaaagatgcagagagaggcaagttaaaaagaagggctatcgcaacacctgggatgcagattagaggtttcgtatactttgttgaaactttagctaggagcatttgcgaaaagcttgaacagtctgggctcccagtagggggcaatgaaaagaaggccaaactggcaaatgttgtgagaaagatgatgactaattcacaagacacagagatttctttcacaatcactggggacaacactaagtggaatgaaaatcaaaatcctcgaatgttcctggcgatgattacatatatcaccagaaatcaacccgagtggttcagaaacatcctgagcatggcacccataatgttctcaaacaaaatggcaagactagggaaagggtacatgttcgagagtaaaagaatgaagattcgaacacaaataccagcagaaatgctagcaagcattgacctgaagtacttcaatgaatcaacaaagaagaaaattgagaaaataaggcctcttctaatagatggcacagcatcactgagtcctgggatgatgatgggcatgttcaacatgctaagtacggtcttgggagtctcgatactgaatcttggacaaaagaaatacaccaagacaatatactggtgggatgggctccaatcatccgacgattttgctctcatagtgaatgcaccaaaccatgagggaatacaagcaggagtggacagattctacaggacctgcaagttagtgggaatcaacatgagcaaaaagaagtcctatataaataagacagggacatttgaattcacaagctttttttatcgctatggatttgtggctaattttagcatggagctacccagctttggagtgtctggagtaaatgaatcatctgacatgagtattggagtaacagtgataaagaacaacatgataaacaatgaccttggacctgcaacggcccagatggctcttcaattgttcatcaaagactacagatacacatataggtgccataggggagacacacaaattcagacgagaagatcatttgagttaaagaagctgtgggatcaaacccaatcaaaggtagggctattagtatcagatggaggaccaaacttatacaatatacggaatcttcacattcctgaagtctgcttaaaatgggagctaatggatgatgattatcggggaagactttgtaatcccctgaatccctttgtcagtcataaagagattgattctgtaaacaatgctgtggtaatgccagcccatggtccagccaaaagcatggaatatgatgccgttgcaactacacattcctggattcccaagaggaatcgttctattctcaacacaagccaaaggggaattcttgaggatgaacagatgtaccagaagtgctgcaatctattcgagaaatttttccctagcagttcatataggagaccggttggaatttctagcatggtggaggccatggtgtctagggcccggattgatgccagggtcgacttcgagtctggacggatcaagaaagaagagttctctgagatcatgaagatctgttccaccattgaagaactcagacggcaaaaataa

>H1N1_PB1_CY054660_A/Russia/100/2009

atggatgtcaatccgactctacttttcctaaaaattccagcgcaaaatgccataagcaccacattcccttatactggagatcctccatacagccatggaacaggaacaggatacaccatggacacagtaaacagaacacaccaatactcagaaaagggaaagtggacgacaaacacggagactggtgcaccccagctcaacccgattgatggaccactacctgaggataatgaaccaagtgggtatgcacaaacagactgtgttctagaggctatggctttccttgaagaatcccacccaggaatatttgagaattcatgccttgaaacaatggaagttgttcaacaaacaagggtagataaactaactcaaggtcgccagacttatgattggacattaaacagaaatcaaccggcagcaactgcattggccaacaccatagaagtctttagatcgaatggcctaacagctaatgagtcaggaaggctaatagatttcttaaaggatgtaatggaatcaatgaacaaagaggaaatagagataacaacccactttcaaagaaaaaggagagtaagagacaacatgaccaagaagatggtcacgcaaagaacaatagggaagaaaaaacaaagactgaataagagaggctatctaataagagcactgacattaaatacgatgaccaaagatgcagagagaggcaagttaaaaagaagggctatcgcaacacctgggatgcagattagaggtttcgtatactttgttgaaactttagctaggagcatttgcgaaaagcttgaacagtctgggctcccagtagggggcaatgaaaagaaggccaaactggcaaatgttgtgagaaagatgatgactaattcacaagacacagagatttctttcacaatcactggggacaacactaagtggaatgaaaatcaaaatcctcgaatgttcctggcgatgattacatatatcaccagaaatcaacccgagtggttcagaaacatcctgagcatggcacccataatgttctcaaacaaaatggcaagactagggaaagggtacatgttcgagagtaaaagaatgaagattcgaacacaaataccagcagaaatgctagcaagcattgacctgaagtacttcaatgaatcaacaaagaagaaaattgagaaaataaggcctcttctaatagatggcacagcatcactgagtcctgggatgatgatgggcatgttcaacatgctaagtacggtcttgggggtctcgatactgaatcttggacaaaagaaatacaccaagacaatatactggtgggatgggctccaatcatccgacgattttgctctcatagtgaatgcaccaaaccatgagggaatacaagcaggagtggacagattctacaggacctgcaagttagtgggaatcaacatgagcaaaaagaagtcctatataaataagacagggacatttgaattcacaagctttttttatcgctatggatttgtggctaattttagcatggagctacccagctttggagtgtctggagtaaatgaatcagctgacatgagtattggagtaacagtgataaagaacaacatgataaacaatgaccttggacctgcaacggcccagatggctcttcaattgttcatcaaagactacagatacacatataggtgccataggggagacacacaaattcagacgagaagatcatttgagttaaagaagctgtgggatcaaacccaatcaaaggtagggctattagtatcagatggaggaccaaacttatacaatatacggaatcttcacattcctgaagtctgcttaaaatgggagctaatggatgatgattatcggggaagactttgtaatcccctgaatccctttgtcagtcataaagagattgattctgtaaacaatgctgtggtaatgccagcccatggtccagccaaaagcatggaatatgatgccgttgcaactacacattcctggattcccaagaggaatcgttctattctcaacacaagccaaaggggaattcttgaggatgaacagatgtaccagaagtgctgcaatctattcgagaaatttttccccagcagttcatataggagaccggttggaatttctagcatggtggaggccatggtgtctagggcccggattgatgccagggtcgacttcgagtctggacggatcaagaaagaagagttctctgagatcatgaagatctgttccaccattgaagaactcagacggcaaaaataa

>H1N1_PB1_GU480934_A/Irkutsk/02/2009

atggatgtcaatccgactctacttttcctaaaaattccagcgcaaaatgccataagcaccacattcccttatactggagatcctccatacagccatggaacaggaacaggatacaccatggacacagtaaacagaacacaccaatactcagaaaagggaaagtggacgacaaacacagagactggtgcaccccagctcaacccgattgatggaccactacctgaggataatgaaccaagtgggtatgcacaaacagactgtgttctagaggctatggctttccttgaagaatcccacccaggaatatttgagaattcatgccttgaaacaatggaagttgttcaacaaacaagggtagataaactaactcaaggtcgccagacttatgattggacattaaacagaaatcaaccggcagcaactgcattggccaacaccatagaagtctttagatcgaatggcctaacagctaatgagtcaggaaggctaatagatttcttaaaggatgtaatggaatcaatgaacaaagaggaaatagagataacaacccactttcaaagaaaaaggagagtaagagacaacatgaccaagaagatggtcacgcaaagaacaatagggaagaaaaaacaaagactgaataagagaggctatctaataagagcactgacattaaatacgatgaccaaagatgcagagagaggcaagttaaaaagaagggctatcgcaacacctgggatgcagattagaggtttcgtatactttgttgaaactttagctaggagcatttgcgaaaagcttgaacagtctgggctcccagtagggggcaatgaaaagaaggccaaactggcaaatgttgtgagaaagatgatgactaattcacaagacacagagatttctttcacaatcactggggacaacactaagtggaatgaaaatcaaaatcctcgaatgttcctggcgatgattacatatatcaccagaaatcaacccgagtggttcagaaacatcctgagcatggcacccataatgttctcaaacaaaatggcaagactagggaaagggtacatgttcgagagtaaaagaatgaagattcgaacacaaataccagcagaaatgctagcaagcattgacctgaagtacttcaatgaatcaacaaagaagaaaattgagaaaataaggcctcttctaatagatggcacagcatcactgagtcctgggatgatgatgggcatgttcaacatgctaagtacggtcttgggagtctcgatactgaatcttggacaaaagaaatacaccaagacaatatactggtgggatgggctccaatcatccgacgattttgctctcatagtgaatgcaccaaaccatgagggaatacaagcaggagtggacagattctacaggacctgcaagttagtgggaatcaacatgagcaaaaagaagtcctatataaataagacagggacatttgaattcacaagctttttttatcgctatggatttgtggctaattttagcatggagctacccagctttggagtgtctggagtaaatgaatcagctgacatgagtattggagtaacagtgataaagaacaatatgataaacaatgaccttggacctgcaacggcccagatggctcttcaattgttcatcaaagactacagatacacatataggtgccataggggagacacacaaattcagacgagaagatcatttgagttaaagaagctgtgggatcaaacccaatcaaaggtagggctattagtatcagatggaggaccaaacttatacaatatacggaatcttcacattcctgaagtctgcttaaaatgggagctaatggatgatgattatcggggaagactttgtaatcccctgaatccctttgtcagccataaagagattgattctgtaaacaatgctgtggtaatgccagcccatggtccagccaaaagcatggaatatgatgccgttgcaactacacattcctggattcccaagaggaatcgttctattctcaacacaagccaaaggggaattcttgaggatgaacagatgtaccagaagtgctgcaatctattcgagaaatttttccctagcagttcatataggagaccggttggaatttctagcatggtggaggccatggtgtctagggcccggattgatgccagggtcgacttcgagtctggacggatcaagaaagaagagttctctgagatcatgaagatctgttccaccattgaagaactcagacggcaaaaataa

>H1N1_PB1_CY054833_A/California/VRDL29/2009

atggatgtcaatccgactctacttttcctaaaaattccagcgcaaaatgccataagcaccacattcccttatactggagatcctccatacagccatggaacaggaacaggatacaccatggacacagtaaacagaacacaccaatactcagaaaagggaaagtggacgacaaacacagagactggtgcaccccagctcaacccgattgatggaccattacctgaggataatgaaccaagtgggtatgcacaaacagactgtgttctagaggctatggctttccttgaagaatcccacccaggaatatttgagaattcatgccttgaaacaatggaagttgttcaacaaacaagggtagataaactaactcaaggtcgccagacttatgattggacattaaacagaaatcaaccggcagcaactgcattggccaacaccatagaagtctttagatcgaatggcctaacagctaatgagtcaggaaggctaatagatttcttaaaggatgtaatggaatcaatgaacaaagaggaaatagagataacaacccactttcaaagaaaaaggagagtaagagacaacatgaccaagaagatggtcacgcaaagaacaatagggaagaaaaaacaaagactgaataagagaggctatctaataagagcactgacattaaatacgatgaccaaagatgcagagagaggcaagttaaaaagaagggctatcgcaacacctgggatgcagattagaggtttcgtatactttgttgaaactttagctaggagcatttgcgaaaagcttgaacagtctgggctcccagtagggggcaatgaaaagaaggccaaactggcaaatgttgtgagaaagatgatgactaattcacaagacacagagatttctttcacaatcactggggacaacactaagtggaatgaaaatcaaaatcctcgaatgttcctggcgatgattacatatatcaccagaaatcaacccgagtggttcagaaacatcctgagcatggcacccataatgttctcaaacaaaatggcaagactagggaaagggtacatgttcgagagtaaaagaatgaagattcgaacacaaataccagcagaaatgctagcaagcattgacctgaagtacttcaatgaatcaacaaagaagaaaattgagaaaataaggcctcttctaatagatggcacagcatcactgagtcctgggatgatgatgggcatgttcaacatgctaagtacggtcttgggagtctcgatactgaatcttggacaaaagaaatacaccaagacaatatactggtgggatgggctccaatcatccgacgattttgctctcatagtgaatgcaccaaaccatgagggaatacaagcaggagtggacagattctacaggacctgcaagttagtgggaatcaacatgagcaaaaagaagtcctatataaataagacagggacatttgaattcacaagctttttttatcgctatggatttgtggctaattttagcatggagctacccagctttggagtgtctggagtaaatgaatcagctgacatgagtattggagtaacagtgataaagaacaacatgataaacaatgaccttggacctgcaacggcccagatggctcttcaattgttcatcaaagactacagatacacatataggtgccataggggagacacacaaattcagacgagaagatcatttgagttaaagaagctgtgggatcaaacccaatcaaaggtagggctattagtatcagatggaggaccaaacttatacaatatacggaatcttcacattcctgaagtctgcttaaaatgggagctaatggatgatgattatcggggaagactttgtaatcccctgaatccctttgtcagtcataaagagattgattctgtaaacaatgctgtggtaatgccagcccatggtccagccaaaagcatggaatatgatgccgttgcaactacacattcctggattcccaagaggaatcgttctattctcaacacaagccaaaggggaattcttgaggatgaacagatgtaccagaagtgctgcaatctattcgagaaatttttccctagcagttcatataggagaccggttggaatttctagcatggtggaggccatggtgtctagggcccggattgatgccagggtcgacttcgagtctggacggatcaagaaagaagagttctctgagatcatgaagatctgttccaccattgaagaactcagacggcaaaaataa

>H1N1_PB1_CY054945_A/California/VRDL48/2009

atggatgtcaatccgactctacttttcctaaaaattccagcgcaaaatgccataagcaccacattcccttatactggagatcctccatacagccatggaacaggaacaggatacaccatggacacagtaaacagaacacaccaatactcagaaaagggaaagtggacgacaaacacagagactggtgcaccccagctcaacccgattgatggaccactacctgaggataatgaaccaagtgggtatgcacaaacagactgtgttctagaggctatggctttccttgaagaatcccacccaggaatatttgagaattcatgccttgaaacaatagaagttgttcaacaaacaagggtagataaactaactcaaggtcgccagacttatgattggacattaaacagaaatcaaccggcagcaactgcattggccaacaccatagaagtctttagatcgaatggcctaacagctaatgagtcaggaaggctaatagatttcttaaaggatgtaatggaatcaatgaacaaagaggaaatagagataacaacccactttcaaagaaaaaggagagtaagagacaacatgaccaagaagatggtcacgcaaagaacaatagggaagaaaaaacaaagactgaataagagaggctatctaataagagcactgacattaaatacgatgaccaaagatgcagagagaggcaagttaaaaagaagggctatcgcaacacctgggatgcagattagaggtttcgtatactttgttgaaactttagctaggagcatttgcgaaaagcttgaacagtctgggctcccagtagggggcaatgaaaagaaggccaaactggcaaatgttgtgagaaagatgatgactaattcacaagacacagagatttctttcacaatcactggggacaacactaagtggaatgaaaatcaaaatcctcgaatgttcctggcgatgattacatatatcaccagaaatcaacccgagtggttcagaaacatcctgagcatggcacccataatgttctcaaacaaaatggcaagactagggaaagggtacatgttcgagagtaaaagaatgaagattcgaacacaaataccagcagaaatgctagcaagcattgacctgaagtacttcaatgaatcaacaaagaagaaaattgagaaaataaggcctcttctaatagatggcacagcatcactgagtcctgggatgatgatgggcatgttcaacatgctaagtacggtcttgggagtctcgatactgaatcttggacaaaagaaatacaccaagacaatatactggtgggatgggctccaatcatccgacgattttgctctcatagtgaatgcaccaaaccatgagggaatacaagcaggagtggacagattctacaggacctgcaagttagtgggaatcaacatgagcaaaaagaagtcctatataaataagacagggacatttgaattcacaagctttttttatcgctatggatttgtggctaattttagcatggagctacccagctttggagtgtctggagtaaatgaatcagctgacatgagtattggagtaacagtgataaagaacaacatgataaacaatgaccttggacctgcaacggcccagatggctcttcaattgttcatcaaagactacagatacacatataggtgccataggggagacacacaaattcagacgagaagatcatttgagttaaagaagctgtgggatcaaacccaatcaaaggtagggctattagtatcagatggaggaccaaacttatacaatatacggaatcttcacattcctgaagtctgcttaaaatgggagctaatggatgatgattatcggggaagactttgtaatcccctgaatccctttgtcagtcataaagagattgattctgtaaacaatgctgtggtaatgccagcccatggtccagccaaaagcatggaatatgatgccgttgcaactacacattcctggattcccaagaggaatcgttctattctcaacacaagccaaaggggaattcttgaggatgaacagatgtaccagaagtgctgcaatctattcgagaaatttttccctagcagttcatataggagaccggttggaatttctagcatggtggaggccatggtgtctagggcccggattgatgccagggtcgacttcgagtctggacggatcaagaaagaagagttctctgagatcatgaagatctgttccaccattgaagaactcagacggcaaaaataa

>H1N1_PB1_CY043407_A/Gunma/07G002/2008

atggatgtcaatccgacattacttttcttaaaagtgccagcacaaaatgctataagcacaacttttccttatactggtgaccctccttacagccatgggacaggaactgggtacaccatggatacagtcaacaggacacatcagtactcagaaagaggaagatggacaaaaaataccgaaactggggcaccgcaactcaacccaattgatgggccactaccagaagacaatgaaccaagtggctatgcccaaacagattgtgtattagaagcaatggctttccttgaagaatcccatcctggtatttttgaaaactcttgtattgaaacaatggagattgttcagcaaacaagggtggacaaactgacacaaggcagacagacctatgactggactctaaataggaaccagcctgctgccacagcattggccaacactatagaagtattcagatcaaacggcctcatagcaaatgaatctgggaggctaatagacttccttaaagatgtaatggagtcgatggacagagaagaagtagaggtcacaactcattttcagagaaagaggagagtaagagacaatgtaactaaaaaaatggtgacccaaagaacaataggcaaaaagaaacataaattagacaaaaggagttacctaattagagcattaactctgaacacaatgaccaaagatgctgaaagagggaaactaaaacgcagagcaattgcaaccccaggaatgcaaataagggggtttgtatactttgttgaaacactggcaagaagcatatgtgaaaagcttgaacaatcagggttgccagttggaggaaatgagaagaaagcaaagttagcaaatgttgtaaggaagatgatgaccaactcccaggacactgaaatttctttcaccatcactggagataacacaaaatggaacgaaaatcaaaaccctagaatgtttttggccatgatcacatatataaccagaaatcagcctgaatggttcagaaatattctaagtgttgctccaataatgttttcaaacaaaatggcgagactaggtaaggggtacatgtttgaaagcaagagtatgaaactaagaactcaaatacctgcagagatactagccgacatagatttgaagtatttcaatgagtcaactaaaaagaaaattgaaaaaatccgaccattattaatagatggaactgcatcattgagtcctggaatgatgatgggcatgttcaatatgttaagcactgtcttgggcgtatccattctgaatcttgggcaaaagagatacaccaagactacttactggtgggatggtcttcaatcgtctgatgattttgctctgatagtgaatgcgcccaactatgcaggaattcaagctggagttgacaggttttatcgaacctgtaagctgctcggaattaatatgagcaaaaagaagtcttacataaacaggacgggtacctttgaattcacaagctttttctatcgttatgggtttgttgccaatttcagcatggagcttcctagttttggggtgtcaggggtcaatgaatctgcagacatgagtattggagtcactgttatcagaaacaatatgataaacaatgaccttggcccagcaactgctcaaatggcccttcagttatttataaaagattacagatacacgtatcgatgccacagaggtgacacacaaatacaaacccggagatcatttgagataaagaaattatgggaccaaacccgctccaaagctgggctgttggttgctgatggaggccccaatttatataacattagaaatctccatattcctgaagtctgtttgaaatgggagttgatggatgaggattaccaggggcgtttatgcaatccattgaacccatttgtcagtcataaagagattgaatcagtgaacaatgcagtgatgatgccggcacatggtccagccaaaaatatggagtatgacgctgttgcaacaacacattcctgggttcccaaaaggaatcgatccattttgaatacgagccaaagggggatacttgaggatgagcaaatgtatcagaggtgctgcaatttatttgaaaaattctttccaagtagctcatacagaagaccagttgggatatccagtatggtagaggctatggtttccagagcccgaattgatgcacggattgatttcgaatctggaaggataaagaaagaggaattcgctgagatcatgaagacctgttccaccattgaagacctcagacggcaaaaatag

>H1N1_PB1_CY043447_A/Kyoto/07K316/2008

atggatgtcaatccgacattacttttcttaaaagtgccagcacaaaatgctataagcacaacttttccttatactggtgaccctccttacagccatgggacaggaacaggatacaccatggatacagtcaacaggacacaccagtactcagaaagaggaagatggacaaaaaataccgaaactggagcaccgcaacttaacccaattgatggtccactaccggaagacaatgaaccaagtggctatgcccaaacagattgtgtattagaagcaatggctttccttgaagaatcccatcccggtatctttgaaaactcttgtattgaaacaatggaggttgttcagcaaacaagggtggacaaactgacacaaggcagacagacctatgactggactctaaataggaaccagcctgctgccacagcattggcaaacactatagaagtattcagatcaaacggcctcatagcaaatgaatctgggaggctaatagacttccttaaagatgtaatggagtcgatggacagaggcgaagtagaggtcacaactcattttcaaagaaagaggagagtgagagacaatgtaactaaaaaaatggtgacccaaagaacaataggcaaaaagaaacataaattagacaaaagaagttacctaattagggcattaaccctgaacacaatgaccaaagatgctgagagggggaaactaaaacgcagagcaattgcaaccccaggaatgcaaataagggggtttgtatactttgttgagacactggcaagaagcatatgtgaaaagcttgaacaatcaggattgccagttggaggaaatgagaagaaagcaaagttagcaaatgttgtaaggaagatgatgaccaactcccaggacactgaaatttctttcaccataaccggagataacacaaaatggaacgaaaatcaaaaccctagaatgttcttggccatgatcacatatataaccaaaaatcagcctgaatggttcagaaatgttctaagtattgctccaataatgttttcaaacaagatggcgagactaggtaaggggtacatgtttgaaagcaagagtatgaaactgagaactcaaatacctgcagagatgctagctaacatagatttgaaatatttcaatgattcaactaaaaggaaaattgaaaaaatccgaccattattaatagatggaactgcatcattgagtcctggaatgatgatgggcatgttcaatatgttgagcaccgtcttgggcgtctccattctgaatcttgggcaaaagagatacaccaagactacttactggtgggatggtcttcaatcgtctgatgattttgctttgattgtgaatgcacccaactatgcaggaattcaagctggagttgacaggttttatcgaacctgtaagctgctcggaattaatatgagcaaaaagaagtcttatataaacagaacaggtacctttgaattcacgagctttttctatcgttatgggtttgttgccaatttcagcatggagcttcctagttttggggtgtctggggtcaatgaatctgcagacatgagtattggagtcactgtcatcaaaaacaatatgataaacaatgaccttggcccagcaactgctcaaatggcccttcagttatttataaaagattacaggtacacttatcggtgccaccgaggtgacacacaaatacaaacccggagatcatttgagataaagaaactatgggaccaaacccgctcaaaagctgggctgttggtctctgatggaggccccaatttatataacattagaaatctccatattcctgaagtttgcttgaaatgggagttgatggatgaggattaccaggggcgtttatgcaacccattaaacccgtttgtcagccataaagagattgaatcagtgaacaatgcagtgataatgccggcacatggtccagccaaaaatatggagtatgacgctgttgcaacaacacactcctgggtccccaaaagaaatcgatccattttgaacacgagccaaagggggatacttgaagatgagcaaatgtatcagaggtgctgcaatttatttgaaaaattcttcccaagtagctcatacagaagaccagttggaatatccagtatggtagaggctatggtctcaagagcccgaattgatgcacggattgatttcgaatctggaaggataaagaaagaggaatttgctgagatcatgaagatctgttccaccattgaagacctcagacggcaaaaatga

>H1N1_PB1_CY043495_A/Niigata/08F031/2009

atggatgtcaatccgacattacttttcttaaaagtgccagcacaaaatgctataagcacaacttttccttatactggtgaccctccttacagccatgggacaggaacagggtacaccatggatacagtcaacaggacacaccagtactcagaaagaggaagatggacaaaaaataccgaaacgggagcaccgcaacttaacccaattgatggtcccttaccggaagacaatgaaccaagtggctatgcccaaacagattgtgtattagaagcaatggctttccttgaagaatcccatcccggtatctttgaaaactcttgtattgaaacaatggaggttgttcaacaaacaagggtggacaaactgacacaaggcagacagacctatgactggactctaaataggaaccagcctgctgccacagcattggcaaacactatagaagtattcagatcaaacggcctcatagcaaatgaatctgggaggctaatagacttccttaaagatgtaatggagtcgatggacagaggcgaagtagaggtcacaactcattttcaaagaaagaggagagtgagagacaatgtaactaaaaaaatggtgacccaaagaacaataggcaaaaagaaacataaattagacaaaagaagttacctaattagggcattaaccctgaacacaatgaccaaagatgctgagagggggaaactaaaacgcagagcaattgcaaccccaggaatgcaaataagagggtttgtatactttgttgagacactggcaagaagcatatgtgaaaagcttgaacaatcaggattgccagttggaggaaatgagaagaaagcaaagttagcaaatgttgtaaggaagatgatgaccaactcccaggacactgaaatttctttcaccataaccggagataacacaaaatggaacgaaaatcaaaaccctagaatgttcttggccatgatcacatatataaccaaaaatcagcctgaatggttcagaaatattctaagtattgctccaataatgttttcaaataagatggcgagactaggcaaggggtacatgtttgaaagcaagagtatgaaactgagaactcaaatacctgcagagatgctagccaacatagacttgaaatatttcaatgattcaacaaaaaagaaaattgaaaaaatccgaccattattaatagatggaactgcatcattgagtcctggaatgatgatgggcatgttcaatatgttgagcaccgtcttgggcgtctccattctgaatcttgggcaaaagagatacaccaagactacttactggtgggatggtcttcaatcgtctgatgattttgctttgattgtgaacgcacccaactatgcaggaattcaagctggagttgacaggttttatcgaacctgtaagctgctcggaattaatatgagcaaaaagaagtcttacataaacagaacaggtacctttgaattcacgagctttttctatcgttatgggtttgttgccaattttagcatggagcttcctagttttggggtgtctggggtcaatgaatctgcagacatgagtattggagtcactgtcatcaaaaacaatatgataaacaatgaccttggcccagcaactgctcaaatggcccttcagttatttatcaaagattacaggtacacttatcgatgccaccgaggtgacacacaaatacaaacccggagatcatttgagataaagaaactatgggaccaaacccgctcaaaagctggactgttggtctctgatggaggccccaatttgtataacattagaaatctccatattcctgaagtttgcttgaaatgggagttgatggatgaggattaccaggggcgtttatgcaacccattaaacccgtttgtcagccataaagagattgaatctgtgaacagtgcagtgataatgccggcacatggtccagccaaaaatatggagtatgacgctgttgcaacaacacactcctgggtccccaaaagaaatcgatccattttgaacacgagccaaagggggatacttgaagatgagcaaatgtatcagaggtgctgcaatttatttgaaaaattcttcccaagtagctcatacagaagaccagttggaatatccagtatggtagaggctatggtctcaagagcccgaattgatgcacggattgatttcgaatctggaaggataaagaaagaggaatttgctgagatcatgaagatctgttccaccattgaagacctcagacggcaaaaatga

>H1N1_PB1_CY043535_A/Kyoto/08K056/2009

atggatgtcaatccgacattacttttcttaaaagtgccagcacaaaatgctataagcacaacttttccttatactggtgaccctccttacagccatgggacaggaacagggtacaccatggatacagtcaacaggacacaccagtactcagaaagaggaagatggacaaaaaataccgaaacgggagcaccgcaacttaacccaattgatggtcccttaccggaagacaatgaaccaagtggctatgcccaaacagattgtgtattagaagcaatggctttccttgaagaatcccatcccggtatctttgaaaactcttgtattgaaacaatggaggttgttcaacaaacaagggtggacaaactgacacaaggcagacagacctatgactggactctaaataggaaccagcctgctgccacagcattggcaaacactatagaagtattcagatcaaacggcctcatagcaaatgaatctgggaggctaatagacttccttaaagatgtaatggagtcgatggacagaggcgaagtagaggtcacaactcattttcaaagaaagaggagagtgagagacaatgtaactaaaaaaatggtgacccaaagaacaataggcaaaaagaaacataaattagacaaaagaagttacctaattagggcattaaccctgaacacaatgaccaaagatgctgagagggggaaactaaaacgcagagcaattgcaaccccaggaatgcaaataagggggtttgtatactttgttgagacactggcaagaagcatatgtgaaaagcttgaacaatcaggattgccagttggaggaaatgagaagaaagcaaagttagcaaatgttgtaaggaagatgatgaccaactcccaggacactgaaatttctttcaccataaccggagataacacaaaatggaacgaaaatcaaaaccctagaatgttcttggccatgatcacatatataaccaaaaatcagcctgaatggttcagaaatattctaagtattgctccaataatgttttcaaacaagatggcgagactaggtaaggggtacatgtttgaaagcaagagtatgaaactgagaactcaaatacctgcagagatgctagccaacatagacttgaaatatttcaatgattcaactaaaaagaaaattgaaaaaatccgaccattattaatagatggaactgcatcattgagtcctggaatgatgatgggcatgttcaatatgttgagcaccgtcttgggcgtctccattctgaatcttgggcaaaagagatacaccaagactacttactggtgggatggtcttcaatcgtctgatgattttgctttgattgtgaacgcacccaactatgcaggaattcaagctggagttgacaggttttatcgaacctgtaagctgctcggaattaatatgagcaaaaagaagtcttacataaacagaacaggtacctttgaattcacgagctttttctatcgttatgggtttgttgccaatttcagcatggagcttcctagttttggggtgtctggggtcaatgaatctgcagacatgagtattggagtcactgtcatcaaaaacaatatgataaacaatgaccttggcccagcaactgctcaaatggcccttcagttatttataaaagattacaggtacacttatcgatgccaccgaggtgacacacaaatacaaacccggagatcatttgagataaaaaaactatgggaccaaacccgctcaaaagctgggctgttggtctctgatggaggccccaatttgtataacattagaaatctccatattcctgaagtttgcttgaaatgggaattgatggatgaggactaccaggggcgtttatgcaacccattaaacccgtttgtcagccataaagagattgaatcagtgaacaatgcagtgataatgccggcacatggtccagccaaaaatatggagtatgacgctgttgcaacaacacactcctgggtccccaaaagaaatcgatccattttgaacacgagccaaagggggatacttgaagatgagcaaatgtatcagaggtgctgcaatttatttgaaaaattcttcccaagtagctcatacagaagaccagttggaatatccagtatggtagaggctatggtctcaagagcccgaattgatgcacggattgatttcgaatctggaaggataaagaaagaggaatttgctgagatcatgaagatctgttccaccattgaagacctcagacggcaaaaatga

>H1N1_PB1_CY055492_A/California/VRDL73/2009

atggatgtcaatccgactctacttttcctaaaaattccagcgcaaaatgccataagcaccacattcccttatactggagatcctccatacagccatggaacaggaacaggatacaccatggacacagtaaacagaacacaccaatactcagaaaagggaaagtggacgacaaacacagagactggtgcaccccagctcaacccgattgatggaccactacccgaggataatgaaccaagtgggtatgcacaaacagactgtgttctagaggctatggctttccttgaagaatcccacccaggaatatttgagaattcatgccttgaaacaatggaagttgttcaacaaacaagggtagataaactaactcaaggtcgccagacttatgattggacattaaacagaaatcaaccggcagcaactgcattggccaacaccatagaagtctttagatcgaatggcctaacagctaatgagtcaggaaggctaatagatttcttaaaggatgtaatggaatcaatgaacaaagaggaaatagagataacaacccactttcaaagaaaaaggagagtaagagacaacatgaccaagaagatggtcacgcaaagaacaatagggaagaaaaaacaaagactgaataagagaggctatctaataagagcactgacattaaatacgatgaccaaagatgcagagagaggcaagttaaaaagaagggctatcgcaacacctgggatgcagattagaggtttcgtatactttgttgaaactttagctaggagcatttgcgaaaagcttgaacagtctgggctcccagtagggggcaatgaaaagaaggccaaactggcaaatgttgtgagaaagatgatgactaattcacaagacacagagatttctttcacaatcactggggacaacactaagtggaatgaaaatcaaaatcctcgaatgttcctggcgatgattacatatatcaccagaaatcaacccgagtggttcagaaacatcctgagcatggcacccataatgttctcaaacaaaatggcaagactagggaaagggtacatgttcgagagtaaaagaatgaagattcgaacacaaataccagcagaaatgctagcaagcattgacctgaagtacttcaatgaatcaacaaagaagaaaattgagaaaataaggcctcttctaatagatggcacagcatcactgagtcctgggatgatgatgggcatgttcaacatgctaagtacggtcttgggagtctcgatactgaatcttggacaaaagaaatacaccaagacaatatactggtgggatgggctccaatcatccgacgattttgctctcatagtgaatgcaccaaaccatgagggaatacaagcaggagtggacagattctacaggacctgcaagttagtgggaatcaacatgagcaaaaagaagtcctatataaataaaacagggacatttgaattcacaagctttttttatcgctatggatttgtggctaattttagcatggagctacccagctttggagtgtctggagtaaatgaatcagctgacatgagtattggagtaacagtgataaagaacaacatgataaacaatgaccttggacctgcaacggcccagatggctcttcaactgttcatcaaagactacagatacacatataggtgccataggggagacacacaaattcagacgagaagatcatttgagttaaagaagctgtgggatcaaacccaatcaaaggtagggctattagtatcagatggaggaccaaatttatacaatatacggaatcttcacattcctgaagtctgcttaaaatgggagctaatggatgatgattatcggggaagactttgtaatcccctgaatccctttgtcagtcataaagagattgattctgtaaacaatgctgtggtaatgccagcccatggtccagccaaaagcatggaatatgatgccgttgcaactacacattcctggattcccaagaggaatcgttctattctcaacacaagccaaaggggaattcttgaggatgaacagatgtaccagaagtgctgcaatctattcgagaaatttttccctagcagttcatataggagaccggttggaatttctagcatggtggaggccatggtgtctagggcccggattgatgccagggtcgacttcgagtctggacggatcaagaaagaagagttctctgagatcatgaagatctgttccaccattgaagaactcagacggcaaaaataa

>H1N1_PB1_CY055532_A/Australia/1/2009

atggatgtcaatccgactctacttttcctaaaaattccagcgcaaaatgccataagcaccacattcccttatactggagatcctccatacagccatggaacaggaacaggatacaccatggacacagtaaacagaacacaccaatactcagaaaagggaaagtggacgacaaacacagagactggtgcaccccagctcaacccgattgatggaccactacctgaggataatgaaccaagtgggtatgcacaaacagactgtgttctagaggctatggctttccttgaagaatcccacccaggaatatttgagaattcatgccttgaaacaatggaagttgttcaacaaacaagggtagataaactaactcaaggtcgccagacttatgattggacattaaacagaaatcaaccggcagcaactgcattggccaacaccatagaagtttttagatcgaatggcctaacagctaatgagtcaggaaggctaatagatttcttaaaggatgtaatgaaatcaatgaacaaagaggaaatagagataacaacccactttcaaagaaaaaggagagtaagagacaacatgaccaagaagatggtcacgcaaagaacaatagggaagaaaaaacaaagactgaataagagaggctatctaataagagcactgacattaaatacgatgaccaaagatgcagagagaggcaagttaaaaagaagggctatcgcaacacctgggatgcagattagaggtttcgtatactttgttgaaactttagctaggagcatttgcgaaaagcttgaacagtctgggctcccagtagggggcaatgaaaagaaggccaaactggcaaatgttgtgagaaagatgatgactaattcacaagacacagagatttctttcacaatcactggggacaacactaagtggaatgaaaatcaaaatcctcgaatgttcctggcgatgattacatatatcaccagaaatcaacccgagtggttcagaaacatcctgagcatggcacccataatgttctcaaacaaaatggcaagactagggaaagggtacatgttcgagagtaaaagaatgaagattcgaacacaaataccagctgaaatgctagcaagcattgacctgaagtacttcaatgaatcaacaaagaagaaaattgagaaaataaggcctcttctaatagatggcacagcatcactgagtcctgggatgatgatgggcatgttcaacatgctaagtacggtcttgggagtctcgatactgaatcttggacaaaagaaatacaccaagacaatatactggtgggatgggctccaatcatccgacgattttgctctcatagtgaatgcaccaaaccatgagggaatacaagcaggagtggacagattctacaggacctgcaagttagtgggaatcaacatgagcaaaaagaagtcctatataaataagacagggacatttgaattcacaagctttttttatcgctatggatttgtggctaattttagcatggagctacccagctttggagtgtctggagtaaatgaatcagctgacatgagtattggagtaacagtgataaagaacaacatgataaacaatgaccttggacctgcaacggcccagatggctcttcaattgttcatcaaagactacagatacacatataggtgccataggggagacacacaaattcagacgagaagatcatttgagttaaagaagctgtgggatcaaacccaatcaaaggtagggctattagtatcagatggaggaccaaacttatacaatatacggaatcttcacattcctgaagtctgcttaaaatgggagctaatggatgatgattatcggggaagactttgtaatcccctgaatccctttgtcagtcataaagagattgattctgtaaacaatgctgtggtaatgccagcccatggtccagccaaaagcatggaatatgatgccgttgcaactacacattcctggattcccaagaggaatcgttctattctcaacacaagccaaaggggaattcttgaggatgaacagatgtaccagaagtgctgcaatctattcgagaaatttttccctagcagttcatataggagaccggttggaatttctagcatggtggaggccatggtgtctagggcccggattgatgccagggtcgacttcgagtctggacggatcaagaaagaagagttctctgagatcatgaagatctgttccaccattgaagaactcagacggcaaaaataa

>H1N1_PB1_CY056122_A/District of Columbia/INS31/2009

atggatgtcaatccgactctacttttcctaaaaattccagcgcaaaatgccataagcaccacattcccttatactggagatcctccatacagccatggaacaggaacaggatacaccatggacacagtaaacagaacacaccaatactcagaaaagggaaagtggacgacaaacacagagactggtgcaccccagctcaacccgattgatggaccactacctgaggataatgaaccaagtgggtatgcacaaacagactgtgttctagaggctatggctttccttgaagaatcccacccaggaatatttgagaattcatgccttgaaacaatggaagttgttcaacaaacaagggtagataaactgactcaaggtcgccagacttatgattggacattaaacagaaatcaaccggcagcaactgcattggccaacaccatagaagtctttagatcgaatggcctaacagctaatgagtcaggaaggctaatagatttcttaaaggatgtaatggaatcaatgaacaaagaggaaatagagataacaacccactttcaaagaaaaaggagagtaagagacaacatgaccaagaagatggtcacgcaaagaacaatagggaagaaaaaacaaagactgaataagagaggctatctaataagagcactgacattaaatacgatgaccaaagatgcagagagaggcaagttaaaaagaagggctatcgcaacacctgggatgcagattagaggtttcgtatactttgttgaaactttagctaggagcatttgcgaaaagcttgaacagtctgggctcccagtagggggcaatgaaaagaaggccaaactggcaaatgttgtgagaaagatgatgactaattcacaagacacagagatttctttcacaatcactggggacaacactaagtggaatgaaaatcaaaatcctcgaatgttcctggcgatgattacatatatcaccagaaatcaacccgagtggttcagaaacatcctgagcatggcacccataatgttctcaaacaaaatggcaagactagggaaagggtacatgttcgagagtaaaagaatgaagattcgaacacaaataccagcagaaatgctagcaagcattgacctgaagtacttcaatgaatcaacaaagaagaaaattgagaaaataaggcctcttctaatagatggcacagcatcactgagtcctgggatgatgatgggcatgttcaacatgctaagtacggtcttgggagtctcgatactgaatcttggacaaaagaaatacaccaagacaatatactggtgggatgggctccaatcatccgacgattttgctctcatagtgaatgcaccaaaccatgagggaatacaagcaggagtggacagattctacaggacctgcaagttagtgggaatcaacatgagcaaaaagaagtcctatataaataagacagggacatttgaattcacaagctttttttatcgctatggatttgtggctaattttagcatggagctacccagctttggagtgtctggagtaaatgaatcagctgacatgagtattggagtaacagtgataaagaacaacatgataaacaatgaccttggacctgcaacggcccagatggctcttcaattgttcatcaaagactacagatacacatataggtgccataggggagacacacaaattcagacgagaagatcatttgagttaaagaagctgtgggatcaaacccaatcaaaggtagggctattagtatcagatggaggaccaaacttatacaatatacggaatcttcacattcctgaagtctgcttaaaatgggagctaatggatgatgattatcggggaagactttgtaatcccctgaatccctttgtcagtcataaagagattgattctgtaaacaatgctgtggtaatgccagcccatggtccagccaaaagcatggaatatgatgccgttgcaactacacattcctggattcccaagaggaatcgttctattctcaacacaagccaaaggggaattcttgaggatgaacagatgtaccagaagtgctgcaatctattcgagaaatttttccctagcagttcatataggagaccggttggaatttctagcatggtggaggccatggtgtctagggcccggattgatgccagggtcgacttcgagtctggacggatcaagaaagaagagttctctgagatcatgaagatctgttccaccattgaagaactcagacggcaaaaataa

>H1N1_PB1_CY056146_A/San Diego/INS35/2009

atggatgtcaatccgactctacttttcctaaaaattccagcgcaaaatgccataagcaccacattcccttatactggagatcctccatacagccatggaacaggaacaggatacaccatggacacagtaaacagaacacaccaatactcagaaaagggaaagtggacgacaaacacagagactggtgcaccccagctcaacccgattgatggaccactacctgaggataatgaaccaagtgggtatgcacaaacagactgtgttctagaggctatggctttccttgaagaatcccacccgggaatatttgagaattcatgccttgaaacaatggaagttgttcaacaaacaagggtagataaactaactcaaggtcgccagacttatgattggacattaaacagaaatcaaccggcagcaactgcattggccaacaccatagaagtctttagatcgaatggcctaacagctaatgagtcaggaaggctaatagatttcttaaaggatgtaatggaatcaatgaacaaagaggaaatagagataacaacccactttcaaagaaaaaggagagtaagagacaacatgaccaagaagatggtcacgcaaagaacaatagggaagaaaaaacaaagactgaataagagaggctatctaataagagcactgacattaaatacgatgaccaaagatgcagagagaggcaagttaaaaagaagggctatcgcaacacctgggatgcagattagaggtttcgtatactttgttgaaactttagctaggagcatttgcgaaaagcttgaacagtctgggctcccagtagggggcaatgaaaagaaggccaaactggcaaatgttgtgagaaagatgatgactaattcacaagacacagagatttctttcacaatcactggggacaacactaagtggaatgaaaatcaaaatcctcgaatgttcctggcgatgattacatatatcaccagaaatcaacccgagtggttcagaaacatcctgagcatggcacccataatgttctcaaacaaaatggcaagactagggagagggtacatgttcgagagtaaaagaatgaagattcgaacacaaataccagcagaaatgctagcaagcattgacctgaagtacttcaatgaatcaacaaagaagaaaattgagaaaataaggcctcttctaatagatggcacagcatcactgagtcctggaatgatgatgggcatgttcaacatgctaagtacagtcttgggagtctcgatactgaaccttggacaaaagaaatacaccaagacaatatactggtgggatgggctccaatcatccgacgattttgctctcatagtgaatgcaccaaaccatgaggggatacaagcaggagtggacagattctacaggacctgcaagttagtgggaatcaacatgagcaaaaagaagtcctatataaataagacagggacatttgaattcacaagctttttttatcgctatggatttgtggctaattttagcatggagctacccagctttggagtgtctggagtaaatgaatcagctgacatgagtattggagtaacagtgataaagaacaacatgataaacaatgaccttggacctgcaacggcccagatggctcttcaattgttcatcaaagactacagatacacatacaggtgccataggggagacacacaaattcagacgagaagatcatttgagttaaagaagctgtgggatcaaacccaatcaaaggtagggctattagtatcagatggaggaccaaacttatacaatatacggaatcttcatattcctgaagtctgcttaaaatgggagctaatggatgatgattatcggggaagactttgtaatcccctgaatccctttgtcagtcataaagagattgattctgtaaacaatgctgtggtaatgccagcccatggtccagccaaaagcatggaatatgatgccgttgcaactacacattcctggattcccaagaggaatcgttctattctcaacacaagccaaaggggaattcttgaggatgaacagatgtaccagaagtgctgcaatctattcgagaaatttttccctagcagttcatataggagaccggttggaatttctagcatggtggaggccatggtgtctagggcccggattgatgccagggtcgacttcgagtctggacggatcaagaaagaagagttctctgagatcatgaagatctgttccaccattgaagaactcagacggcaaaaacaa

>H1N1_PB1_CY056202_A/San Diego/INS48/2009

atggatgtcaatccgactctacttttcctaaaaattccagcgcaaaatgccataagcaccacattcccttatactggagatcctccatacagccatggaacaggaacaggatacaccatggacacagtaaacagaacacaccaatactcagaaaagggaaagtggacgacaaacacagagactggtgcaccccagctcaacccgattgatggaccactacctgaggataatgaaccaagtgggtatgcacaaacagactgtgttctagaggctatggctttccttgaagaatcccacccaggaatatttgagaattcatgccttgaaacaatggaagttgttcaacaaacaagggtagataaactaactcaaggtcgccagacttatgattggacattaaacagaaatcaaccggcagcaactgcattggccaacaccatagaagtctttagatcgaatggcctaacagctaatgagtcaggaaggctaatagatttcttaaaggatgtaatggaatcaatgaacaaagaggaaatagagataacaacccactttcaaagaaaaaggagagtaagagacaacatgaccaagaagatggtcacgcaaagaacaatagggaagaaaaaacaaagactgaataagagaggctatctaataagagcactgacattaaatacgatgaccaaagatgcagagagaggcaagttaaaaagaagggctatcgcaacacctgggatgcagattagaggtttcgtatactttgttgaaactttagctaggagcatttgcgaaaagcttgaacagtctgggctcccagtagggggcaatgaaaagaaggccaaactggcaaatgttgtgagaaagatgatgactaattcacaagacacagagatttctttcacaatcactggggacaacactaagtggaatgaaaatcaaaatcctcgaatgttcctggcgatgattacatatatcaccagaaatcaacccgagtggttcagaaacatcctgagcatggcacccataatgttctcaaacaaaatggcaagactagggaaagggtacatgttcgagagtaaaagaatgaagattcgaacacaaataccagcagaaatgctagcaagcattgacctgaagtacttcaatgaatcaacaaagaagaaaattgagaaaataaggcctcttctaatagatggcacagcatcactgagtcctgggatgatgatgggcatgttcaacatgctaagtacggtcttgggagtctcgatactgaatcttggacaaaagaaatacaccaagacaatatactggtgggatgggctccaatcatccgacgattttgctctcatagtgaatgcaccaaaccatgagggaatacaagcaggagtggacagattctacaggacctgcaagttagtgggaatcaacatgagcaaaaagaagtcctatataaataagacagggacatttgaattcacaagctttttttatcgctatggatttgtggctaattttagcatggagctacccagctttggagtgtctggagtaaatgaatcagctgacatgagtattggagtaacagtgataaagaacaacatgataaacaatgaccttggacctgcaacggcccagatggctcttcaactgttcatcaaagactacagatacacatataggtgccataggggagacacacaaattcagacgagaagatcatttgagttaaagaagctgtgggatcaaacccaatcaaaggtagggctattagtatcagatggaggaccaaacttatacaatatacggaatcttcacattcctgaagtctgcttaaaatgggagctaatggatgatgattatcggggaagactttgtaatcccctgaatccctttgtcagtcataaagagattgattctgtaaacaacgctgtggtaatgccagcccatggtccagccaaaagcatggaatatgatgccgttgcaactacacattcctggattcccaagaggaatcgttctattctcaacacaagccaaaggggaattcttgaggatgaacagatgtaccagaagtgctgcaatctattcgagaaatttttccctagcagttcatataggagaccggttggaatttctagcatggtggaggccatggtgtctagggcccggattgatgccagggtcgacttcgagtctggacggatcgggaaggaagagttctctgagatcatgaagatctgttccaccattgaagagctcagacggcaaaaataa

>H1N1_PB1_CY056833_A/New York/6846/2009

atggatgtcaatccgactctacttttcctaaaaattccagcgcaaaatgccataagcaccacattcccttatactggagatcctccatacagccatggaacaggaacaggatacaccatggacacagtaaacagaacacaccaatactcagaaaagggaaagtggacgacaaacacagagactggtgcaccccagctcaacccgattgatggaccactacctgaggataatgaaccaagtgggtatgcacaaacagactgtgttctagaggctatggctttccttgaagaatcccacccaggaatatttgagaattcatgccttgaaacaatggaagttgttcaacaaacaagggtagataaactaactcaaggtcgccagacttatgattggacattaaacagaaatcaaccggcagcaactgcattggccaacaccatagaagtctttagatcgaatggcctaacagctaatgagtcaggaaggctaatagatttcttaaaggatgtaatggaatcaatgaacaaagaggaaatagagataacaacccactttcaaagaaaaaggagagtaagagacaacatgaccaagaagatggtcacgcaaagaacaatagggaagaaaaaacaaagactgaataagagaggctatctaataagagctctgacattaaatacgatgaccaaagatgcagagagaggcaagttaaaaagaagggctatcgcaacacctgggatgcagattagaggtttcgtatactttgttgaaactttagctaggagcatttgcgaaaagcttgaacagtctgggctcccagtagggggcaatgaaaagaaggccaaactggcaaatgttgtgagaaagatgatgactaattcacaagacacagagatttctttcacaatcactggggacaacactaagtggaatgaaaatcaaaatcctcgaatgttcctggcgatgattacatatatcaccagaaatcaacccgagtggttcagaaacatcctgagcatggcacccataatgttctcaaacaaaatggcaagactagggaaagggtacatgttcgagagtaaaagaatgaagattcgaacacaaataccagcagaaatgctagcaagcattgacctgaagtacttcaatgaatcaacaaagaagaaaattgagaaaataaggcctcttctaatagatggcacagcatcactgagtcctgggatgatgatgggcatgttcaacatgctaagtacggtcttgggagtctcgatactgaatcttggacaaaagaaatacaccaagacaatatactggtgggatgggctccaatcatccgacgattttgctctcatagtgaatgcaccaaaccatgagggaatacaagcaggagtggacagattctacaggacctgcaagttagtgggaatcaacatgagcaaaaagaagtcctatataaataagacagggacatttgaattcacaagctttttttatcgctatggatttgtggctaattttagcatggagctacccagctttggagtgtctggagtaaatgaatcagctgacatgagtattggagtaacagtgataaagaacaacatgataaacaatgaccttggacctgcaacggcccagatggctcttcaattgttcatcaaagattacagatacacatataggtgccataggggagacacacaaattcaaacgagaagatcatttgagttaaagaagctgtgggatcaaacccaatcaaaggtagggctattagtatcagatggaggaccaaacttatacaatatacggaatcttcacattcctgaagtctgcttaaaatgggagctaatggatgatgattatcggggaagactttgtaatcccctgaatccctttgtcagtcataaagagattgattctgtaaacaacgctgtggtaatgccagcccatggtccagccaaaagcatggaatatgatgccgttgcaactacacattcctggattcccaagaggaatcgttctattctcaacacaagccaaaggggaattcttgaggatgaacagatgtaccagaagtgctgcaatctattcgagaaatttttccctagcagttcatataggagaccggttggaatttctagcatggtggaggccatggtgtctagggcccggattgatgccagggtcgacttcgagtctggacggatcaagaaagaagagttctctgagatcatgaagatctgttccaccattgaagaactcagacggcaaaaataa

>H1N1_PB1_CY056921_A/San Diego/INS61/2009

atggatgtcaatccgactctacttttcctaaaaattccagcgcaaaatgccataagcaccacattcccttatactggagatcctccatacagccatggaacaggaacaggatacaccatggacacagtaaacagaacacaccaatactcagaaaagggaaagtggacgacaaacacagagactggtgcaccccagctcaacccgattgatggaccactacctgaggataatgaaccaagtgggtatgcacaaacagactgtgttctagaggctatggctttccttgaagaatcccacccaggaatatttgagaattcatgccttgaaacaatggaagttgttcaacaaacaagggtagataaactaactcaaggtcgccagacttatgattggacattaaacagaaatcaaccggcagcaactgcattggccaacaccatagaagtctttagatcgaatggcctaacagctaatgagtcaggaaggctaatagatttcttaaaggatgtaatggaatcaatgaacaaagaggaaatagagataacaacccactttcaaagaaaaaggagagtaagagacaacatgaccaagaagatggtcacgcaaagaacaatagggaagaaaaaacaaagactgaataagagaggctatctaataagagcactgacattaaatacgatgaccaaagatgcagagagaggcaagttaaaaagaagggctatcgcaacacctgggatgcagattagaggtttcgtatactttgttgagactttagctaggagcatttgcgaaaagcttgaacagtctgggctcccagtagggggcaatgaaaagaaggccaaactggcaaatgttgtgagaaagatgatgactaattcacaagacacagagatttctttcacaatcactggggacaacactaagtggaatgaaaatcaaaatcctcgaatgttcctggcgatgattacatatatcaccagaaatcaacccgagtggttcagaaacatcctgagcatggcacccataatgttctcaaacaaaatggcaagactagggaaagggtacatgttcgagagtaaaagaatgaagattcgaacacaaataccagcagaaatgctagcaagcattgacctgaagtacttcaatgaatcaacaaagaagaaaattgagaaaataaggcctcttctaatagatggcacagcatcactgagtcctgggatgatgatgggcatgttcaacatgctaagtacggtcttgggagtctcgatactgaatcttggacaaaagaaatacaccaagacaatatactggtgggatgggctccaatcatccgacgattttgctctcatagtgaatgcaccaaaccatgagggaatacaagcaggagtggacagattctacaggacctgcaagttagtgggaatcaacatgagcaaaaagaagtcctatataaataagacagggacatttgaattcacaagctttttttatcgctatggatttgtggctaattttagcatggagctacccagctttggagtgtctggagtaaatgaatcagctgacatgagtattggagtaacagtgataaagaacaacatgataaacaatgaccttggacctgcaacggcccagatggctcttcaactgttcatcaaagactacagatacacatataggtgccataggggagacacacaaattcagacgagaagatcatttgagttaaagaagctgtgggatcaaacccaatcaaaggtagggctattagtatcagatggaggaccaaacttatacaatatacggaatcttcacattcctgaagtctgcttaaaatgggagctaatggatgatgattatcggggaagactttgtaatcccctgaatccctttgtcagtcataaagagattgattctgtaaacaatgctgtggtaatgccagcccatggtccagccaaaagcatggaatatgatgccgttgcaactacacattcctggattcccaagaggaatcgttctattctcaacacaagccaaaggggaattcttgaggatgaacagatgtaccagaagtgctgcaatctattcgagaaatttttccctagcagttcatataggagaccggttggaatttctagcatggtggaggccatggtgtctagggcccggattgatgccagggtcgacttcgagtctggacggatcgggaaggaagagttctctgagatcatgaagatctgttccaccattgaagaactcagacggcaaaaataa

>H1N1_PB1_CY056953_A/Aarhus/INS82/2009

atggatgtcaatccgactctacttttcctaaaaattccagcgcaaaatgccataagcaccacattcccttatactggagatcctccatacagccatggaacaggaacaggatacaccatggacacagtaaacagaacacaccaatactcagaaaagggaaagtggacgacaaacacagagactggtgcaccccagctcaacccgattgatggaccactacctgaggataatgaaccaagtgggtatgcacaaacagactgtgttctagaggctatggctttccttgaagaatcccacccaggaatatttgagaattcatgccttgaaacaatggaagttgttcaacaaacaagggtagataaactaactcaaggtcgccagacttatgattggacattaaacagaaatcaaccggcagcaactgcattggccaacaccatagaagtctttagatcgaatggcctaacagctaatgagtcaggaaggctaatagatttcttaaaggatgtaatggaatcaatgaacaaagaggaaatagagataacaacccactttcaaagaaaaaggagagtaagagacaacatgaccaagaagatggtcacgcaaagaacaatagggaagaaaaaacaaagactgaataagagaggctatctaataagagcactgacattaaatacgatgaccaaagatgcagagagaggcaagttaaaaagaagggctatcgcaacacctgggatgcagattagaggtttcgtatactttgttgaaactttagctaggagcatttgcgaaaagcttgaacagtctgggctcccagtagggggcaatgaaaagaaggccaaactggcaaatgttgtgagaaagatgatgactaattcacaagacacagagatttctttcacaatcactggggacaacactaagtggaatgaaaatcaaaatcctcgaatgttcctggcgatgattacatatatcaccagaaatcaacccgagtggttcagaaacatcctgagcatggcacccataatgttctcaaacaaaatggcaagactagggaaagggtacatgttcgagagtaaaagaatgaagattcgaacacaaataccagcagaaatgctagcaagcattgacctgaagtacttcaatgaatcaacaaagaagaaaattgagaaaataaggcctcttctaatagatggcacagcatcactgagtcctgggatgatgatgggcatgttcaacatgctaagtacggtcttgggagtctcgatactgaatcttggacaaaagaaatacaccaagacaatatactggtgggatgggctccaatcatccgacgattttgctctcatagtgaatgcaccaaaccatgagggaatacaagcaggagtggacagattctacaggacctgcaagttagtgggaatcaacatgagcaagaagaagtcctatataaataagacaggaacatttgaattcacaagctttttttatcgctatggatttgtggctaattttagcatggagctacccagctttggagtgtctggagtaaatgaatcagctgacatgagtattggagtaacagtgataaagaacaacatgataaacaatgaccttggacctgcaacggcccagatggctcttcaattgttcatcaaagactacagatacacatataggtgccataggggagacacacaaattcagacgagaagatcatttgagttaaagaagctgtgggatcaaacccaatcaaaggtagggctattagtatcagatggaggaccaaacttatacaatatacggaatcttcacattcctgaagtctgcttaaaatgggagctaatggatgatgattatcggggaagactttgtaatcccctgaatccctttgtcagtcataaagagattgattctgtaaacaatgctgtggtaatgccagcccatggtccagccaaaagcatggaatatgatgccgttgcaactacacattcctggattcccaagaggaatcgttctattctcaacacaagccaaaggggaattcttgaggatgaacagatgtaccagaagtgctgcaatctattcgagaaatttttccctagcagttcatataggagaccggttggaatttctagcatggtggaggccatggtgtctagggcccggattgatgccagggtcgacttcgagtctggacggatcaagaaagaagagttctctgagatcatgaagatctgttccaccattgaagaactcagacggcaaaaataa

>H1N1_PB1_CY057009_A/Wisconsin/629-D02367/2009

atggatgtcaatccgactctacttttcctaaaaattccagcgcaaaatgccataagcaccacattcccttatactggagatcctccatacagccatggaacaggaacaggatacaccatggacacagtaaacagaacacaccaatactcagaaaagggaaagtggacgacaaacacagagactggtgcaccccagctcaacccgattgatggaccactacctgaggataatgaaccaagtgggtatgcacaaacagactgtgttctagaggctatggctttccttgaagaatcccacccaggaatatttgagaattcatgccttgaaacaatggaagttgttcaacaaacaagggtagataaactaactcaaggtcgccagacttatgattggacattaaacagaaatcaaccggcagcaactgcattggccaacaccatagaagtctttagatcgaatggcctaacagctaatgagtcaggaaggctaatagatttcttaaaggatgtaatggaatcaatgaacaaagaggaaatagagataacaacccactttcaaagaaaaaggagagtaagagacaacatgaccaagaagatggtcacgcaaagaacaatagggaagaaaaaacaaagactgaataagagaggctatctaataagagcactgacattaaatacgatgaccaaagatgcagagagaggcaagttaaaaagaagggctatcgcaacacctgggatgcagattagaggtttcgtatactttgttgaaactttagctaggagcatttgcgagaagcttgaacagtctgggctcccagtagggggcaatgaaaagaaggccaaactggcaaatgttgtgagaaagatgatgactaattcacaagacacagagatttctttcacaatcactggggacaacactaagtggaatgaaaatcaaaatcctcgaatgttcctggcgatgattacatatatcaccagaaatcaacccgagtggttcagaaacatcctgagcatggcacccataatgttctcaaacaaaatggcaagactagggaaagggtacatgttcgagagtaaaagaatgaagattcgaacacaaataccagcagaaatgctagcaagcattgacctgaagtacttcaatgaatcaacaaagaagaaaattgagaaaataaggcctcttctaatagatggcacagcatcactgagtcctgggatgatgatgggcatgttcaacatgctaagtacggtcttgggagtctcgatactgaatcttggacaaaagaaatacaccaagacaatatactggtgggatgggctccaatcatccgacgattttgctctcatagtgaatgcaccaaaccatgagggaatacaagcaggagtggacagattctacaggacctgcaagttagtgggaatcaacatgagcaaaaagaagtcctatataaataagacagggacatttgaattcacaagctttttttatcgctatggatttgtggctaattttagcatggagctacccagctttggagtgtctggagtaaatgaatcagctgacatgagtattggagtaacagtgataaagaacaacatgataaacaatgaccttggacctgcaacggcccagatggctcttcaattgttcatcaaagactacagatacacatataggtgccataggggagacacacaaattcagacgagaagatcatttgagttaaagaagctgtgggatcaaacccaatcaaaggtagggctattagtatcagatggaggaccaaacttatacaatatacggaatcttcacattcctgaagtctgcttaaaatgggagctaatggatgatgattatcggggaagactttgtaatcccctgaatccctttgtcagtcataaagagattgattctgtaaacaatgctgtggtaatgccagcccatggtccagccaaaagcatggaatatgatgccgttgcaactacacattcctggattcccaagaggaatcgttctattctcaacacaagccaaaggggaattcttgaggatgaacagatgtaccagaagtgctgcaatctattcgagaaatttttccctagcagttcatataggagaccggttggaatttctagcatggtggaggccatggtgtctagggcccggattgatgccagggtcgacttcgagtctggacggatcaagaaagaagagttctctgagatcatgaagatctgttccaccattgaagaactcagacggcaaaaataa

>H1N1_PB1_CY057292_A/New York/5271/2009

atggatgtcaatccgactctacttttcctaaaaattccagcgcaaaatgccataagcaccacattcccttatactggagatcctccatacagccatggaacaggaacaggatacaccatggacacagtaaacagaacacaccaatactcagaaaagggaaagtggacgacaaacacagagactggtgcaccccagctcaacccgattgatggaccactacctgaggataatgaaccaagtgggtatgcacaaacagactgtgttctagaggctatggctttccttgaagaatcccacccaggaatatttgagaattcatgccttgaaacaatggaagttgttcaacaaacaagggtagataaactaactcaaggtcgccagacttatgattggacattaaacagaaatcaaccggcagcaactgcattggccaacaccatagaagtctttagatcgaatggcctaacagctaatgagtcaggaaggctaatagatttcttaaaggatgtaatggaatcaatgaacaaagaggaaatagagataacaacccactttcaaagaaaaaggagagtaagagacaacatgaccaagaagatggtcacgcaaagaacaatagggaagaaaaaacaaagactgaataagagaggctatctaataagagcactgacattaaatacgatgaccaaagatgcagagagaggcaagttaaaaagaagggctatcgcaacacctgggatgcagattagaggtttcgtatactttgttgaaactttagctaggagcatttgcgaaaagcttgaacagtctgggctcccagtagggggcaatgaaaagaaggccaaactggcaaatgttgtgagaaagatgatgactaattcacaagacacagagatttctttcacaatcactggggacaacactaagtggaatgaaaatcaaaatcctcgaatgttcctggcgatgattacatatatcaccagaaatcaacccgagtggttcagaaacatcctgagcatggcacccataatgttctcaaacaaaatggcaagactagggaaagggtacatgttcgagagtaaaagaatgaagattcgaacacaaataccagcagaaatgctagcaagcattgacctgaagtacttcaatgaatcaacaaagaagaaaattgagaaaataaggcctcttctaatagatggcacagcatcactgagtcctgggatgatgatgggcatgttcaacatgctaagtacggtcttgggagtctcgatactgaatcttggacaaaagaaatacaccaagacaatatactggtgggatgggctccaatcatccgacgattttgctctcatagtgaatgcaccaaaccatgagggaatacaagcaggagtggacagattctacaggacctgcaagttagtgggaatcaacatgagcaaaaagaagtcctatataaataagacagggacatttgaattcacaagctttttttatcgctatggatttgtggctaattttagcatggagctacccagctttggagtgtctggagtaaatgaatcagctgacatgagtattggagtaacagtgataaagaacaacatgataaacaatgaccttggacctgcaacggcccagatggctcttcaactgttcatcaaagactacagatacacatataggtgccataggggagacacacaaattcagacgagaagatcatttgagttaaagaagctgtgggatcaaacccaatcaaaggtaggactattagtatcagatggaggaccaaacttatacaatatacggaatcttcacattcctgaagtctgcttaaaatgggagctaatggatgatgattatcggggaagactttgtaatcccctgaatccctttgtcagtcataaagagattgattctgtaaacaatgctgtggtaatgccagcccatggtccagccaaaagcatggaatatgatgccgttgcaactacacattcctggattcccaagaggaatcgttctattctcaacacaagccaaaggggaattcttgaggatgaacagatgtaccagaagtgctgcaatctattcgagaaatttttccctagcagttcatataggagaccggttggaatttctagcatggtggaggccatggtgtctagggcccggattgatgccagggtcgacttcgagtctggacggatcgggaaggaagagttctctgagatcatgaagatctgttccaccattgaagaactcagacggcaaaaataa

>H1N1_PB1_CY057332_A/New York/6072/2009

atggatgtcaatccgactctacttttcctaaaaattccagcgcaaaatgccataagcaccacattcccttatactggagatcctccatacagccatggaacaggaacaggatacaccatggacacagtaaacagaacacaccaatactcagaaaagggaaagtggacgacaaacacagagactggtgcaccccagctcaacccgattgatggaccactacctgaggataatgaaccaagtgggtatgcacaaacagactgtgttctagaggctatggcttttcttgaagaatcccacccaggaatatttgagaattcatgccttgaaacaatggaagttgttcaacaaacaagggtagataaactaactcaaggtcgccagacttatgattggacattaaacagaaatcaaccggcagcaactgcattggccaacaccatagaagtctttagatcgaatggcctaacagccaatgagtcaggaaggctaatagatttcttaaaggatgtaatggaatcaatgaacaaagaggaaatagagataacaacccactttcaaagaaaaaggagagtaagagacaacatgaccaagaagatggtcacgcaaagaacaatagggaagaaaaaacaaagactgaataagagaggctatctaataagagcactgacattaaatacgatgaccaaagatgcagagagaggcaagttaaaaagaagggctatcgcaacacctgggatgcagattagaggtttcgtatactttgttgaaactttagctaggagcatttgcgaaaagcttgaacagtctgggctcccagtagggggcaatgaaaagaaggccaaactggcaaatgttgtgagaaagatgatgactaattcacaagacacagagatttctttcacaatcactggggacaacactaagtggaatgaaaatcaaaatcctcgaatgttcctggcgatgattacatatatcaccagaaatcaacccgagtggttcagaaacatcctgagcatggcacccataatgttctcaaacaaaatggcaagactagggaaagggtacatgttcgagagtaaaagaatgaagattcgaacacaaataccagcagaaatgctagcaagcattgacctgaagtacttcaatgaatcaacaaagaagaaaattgagaaaataaggcctcttctaatagatggcacagcatcactgagtcctgggatgatgatgggcatgttcaacatgctaagtacggtcttgggagtctcgatactgaatcttggacaaaagaaatacaccaagacaatatactggtgggatgggctccaatcatccgacgattttgctctcatagtgaatgcaccaaaccatgagggaatacaagcaggagtggacagattctacagggcctgcaagttagtgggaatcaacatgagcaaaaagaagtcctatataaataaaacagggacatttgaattcacaagctttttttatcgctatggatttgtggctaattttagcatggagctacccagctttggagtgtctggagtaaatgaatcagctgacatgagtattggagtaacagtgataaagaacaacatgataaacaatgaccttggacctgcaacggcccagatggctcttcaactgttcatcaaagactacagatacacatataggtgccataggggagacacacaaattcagacgagaagatcatttgagttaaagaagctgtgggatcaaacccaatcaaaggtagggctattagtatcagatggaggaccaaacttatacaatatacggaatcttcacattcctgaagtctgcttaaaatgggagctaatggatgatgattatcggggaagactttgtaatcccctgaatccctttgtcagtcataaagagattgattctgtaaacaatgctgtggtaatgccagcccatggtccagccaaaagcatggaatatgatgctgttgcaactacacattcctggattcccaagaggaatcgttctattctcaacacaagccaaaggggaattcttgaggatgaacagatgtaccagaagtgctgcaatctattcgagaaatttttccctagcagttcatataggagaccggttggaatttctagcatggtggaggccatggtgtctagggcccggattgatgccagggtcgacttcgagtctggacggatcgggaaggaagagttctctgagatcatgaagatctgttccaccattgaagaactcagacggcaaaaataa

>H1N1_PB1_CY057788_A/Wisconsin/629-D01014/2009

atggatgtcaatccgactctacttttcctaaaaattccagcgcaaaatgccataagcaccacattcccttatactggagatcctccatacagccatggaacaggaacaggatacaccatggacacagtaaacagaacacaccaatactcagaaaagggaaagtggacgacaaacacagagactggtgcaccccagctcaacccgattgatggaccactacctgaggacaatgaaccaagtgggtatgcacaaacagactgtgttctagaggctatggctttccttgaagaatcccacccaggaatatttgagaattcatgccttgaaacaatggaagttgttcaacaaacaagggtagataaactaactcaaggtcgccagacttatgattggacattaaacagaaatcaaccggcagcaactgcattggccaacaccatagaagtctttagatcgaatggcctaacagctaatgagtcaggaaggctaatagatttcttaaaggatgtaatggaatcaatgaacaaagaggaaatagagataacaacccactttcaaagaaaaaggagagtaagagacaacatgaccaagaagatggtcacgcaaagaacaatagggaagaaaaaacaaagactgaataagagaggctatctaataagagcactgacattaaatacgatgaccaaagatgcagagagaggcaagttaaaaagaagggctatcgcaacacctgggatgcagattagaggtttcgtatactttgttgaaactttagctaggagcatttgcgaaaagcttgaacagtctgggctcccagtagggggcaatgaaaagaaggccaaactggcaaatgttgtgagaaagatgatgactaattcacaagacacagagatttctttcacaatcactggggacaacactaagtggaatgaaaatcaaaatcctcgaatgttcctggcgatgattacatatatcaccagaaatcaacccgagtggttcagaaacatcctgagcatggcacccataatgttctcaaacaaaatggcaagactagggaaagggtacatgttcgagagtaaaagaatgaagattcgaacacaaataccagcagaaatgctagcaagcattgacctgaagtacttcaatgaatcaacaaagaagaaaattgagaaaataaggcctcttctaatagatggcacagcatcactgagtcctgggatgatgatgggcatgttcaacatgctaagtacggtcttgggagtctcgatactgaatcttggacaaaagaaatacaccaagacaatatactggtgggatgggctccaatcatccgacgattttgctctcatagtgaatgcaccaaaccatgagggaatacaagcaggagtggacagattctacaggacctgcaagttagtgggaatcaacatgagcaaaaagaagtcctatataaataagacagggacatttgaattcacaagctttttttatcgctatggatttgtggctaattttagcatggagctacccagctttggagtgtctggagtaaatgaatcagctgacatgagtattggagtaacagtgataaagaacaacatgataaacaatgaccttggacctgcaacggcccagatggctcttcaactgttcatcaaagactacagatacacatataggtgccataggggagacacacaaattcagacgagaagatcatttgagttaaagaagctgtgggatcaaacccaatcaaaggtagggctattagtatcagatggaggaccaaacttatacaatatacggaatcttcacattcctgaagtctgcttaaaatgggagctaatggatgatgattatcggggaagactttgtaatcccctgaatccctttgtcagtcataaagagattgattctgtaaacaatgctgtggtaatgccagcccatggtccagccaaaagcatggagtatgatgccgttgcaactacacattcctggattcccaagaggaatcgttctattctcaacacaagccaaaggggaattcttgaggatgaacagatgtaccagaagtgctgcaatctattcgagaaatttttccctagcagttcatataggagaccggttggaatttctagcatggtggaggccatggtgtctagggcccggattgatgccagggtcgacttcgagtctggacggatcgggaaggaagagttctctgagatcatgaagatctgttccaccattgaagaactcagacggcacaaataa

>H1N1_PB1_CY057852_A/Wisconsin/629-D02060/2009

atggatgtcaatccgactctacttttcctaaaaattccagcgcaaaatgccataagcaccacattcccttatactggagatcctccatacagccatggaacaggaacaggatacaccatggacacagtaaacagaacacaccaatactcagaaaagggaaagtggacgacaaacacagagactggtgcaccccagctcaacccgattgatggaccactacctgaggataatgaaccaagtgggtatgcacaaacagactgtgttctagaggctatggctttccttgaagaatcccacccaggaatatttgagaattcatgccttgaaacaatggaagttgttcaacaaacaagggtagataaactaactcaaggtcgccagacttatgattggacattaaacagaaatcaaccggcagcaactgcattggccaacaccatagaagtctttagatcgaatggcctaacagctaatgagtcaggaaggctaatagatttcttaaaggatgtaatggaatcaatgaacaaagaggaaatagagataacaacccactttcaaagaaaaaggagagtaagagacaacatgaccaagaagatggtcacgcaaagaacaatagggaagaaaaaacaaagactgaataagagaggctatctaataagagcactgacattaaatacgatgaccaaagatgcagagagaggcaagttaaaaagaagggctatcgcaacacctgggatgcagattagaggtttcgtatactttgttgaaactttagctaggagcatttgcgaaaagcttgaacagtctgggctcccagtagggggcaatgaaaagaaggccaaactggcaaatgttgtgagaaagatgatgactaattcacaagacacagagatttctttcacaatcactggggacaacactaagtggaatgaaaatcaaaatcctcgaatgttcctggcgatgattacatatatcaccagaaatcaacccgagtggttcagaaacatcctgagcatggcacccataatgttctcaaacaaaatggcaagactagggaaagggtacatgttcgagagtaaaagaatgaagattcgaacacaaataccagcagaaatgctagcaagcattgacctgaggtacttcaatgaatcaacaaagaagaaaattgagaaaataaggcctcttctaatagatggcacagcatcactgagtcctgggatgatgatgggcatgttcaacatgctaagtacggtcttgggagtctcgatactgaatcttggacaaaagaaatacaccaagacaatatactggtgggatgggctccaatcatccgacgattttgctctcatagtgaatgcaccaaaccatgagggaatacaagcaggagtggacagattctacaggacctgcaagttagtgggaatcaacatgagcaaaaagaagtcctatataaataagacagggacatttgaattcacaagctttttttatcgctatggatttgtggctaattttagcatggagctacccagctttggagtgtctggagtaaatgaatcagctgacatgagtattggagtaacagtaataaagaacaacatgataaacaatgaccttggacctgcaacggcccagatggctcttcaattgttcatcaaagactacagatacacatataggtgccataggggagacacacaaattcagacgagaagatcatttgaattaaagaagctgtgggatcaaacccaatcaaaggtagggctattagtatcagatggaggaccaaacttatacaatatacggaatcttcacattcctgaagtctgcttaaaatgggagctaatggatgatgattatcggggaagactttgtaatcccctgaatccctttgtcagtcataaagagattgattctgtaaacaatgctgtggtaatgccagcccatggtccagccaaaagcatggaatatgatgccgttgcaactacacattcctggattcccaagaggaatcgttctattctcaacacaagccaaaggggaattcttgaggatgaacagatgtaccagaagtgctgcaatctattcgagaaatttttccctagcagttcatataggagaccggttggaatttctagcatggtggaggccatggtgtctagggcccggattgatgccagggtcgacttcgagtctggacggatcaagaaagaagagttctctgagatcatgaagatctgttccaccattgaagaactcagacggcaaaaataa

>H1N1_PB1_CY057948_A/Wisconsin/629-D00629/2009

atggatgtcaatccgactctacttttcctaaaaattccagcgcaaaatgccataagcaccacattcccttatactggagatcctccatacagccatggaacaggaacaggatacaccatggacacagtaaacagaacacaccaatactcagaaaagggaaagtggacgacaaacacagagactggtgcaccccagctcaacccgattgatggaccactacctgaggataatgaaccaagtgggtatgcacaaacagactgtgttctagaggctatggctttccttgaagaatcccacccaggaatatttgagaattcatgccttgaaacaatggaagttgttcaacaaacaagggtagataaactaactcaaggtcgccagacttatgattggacattaaacagaaatcaaccggcagcaactgcattggccaacaccatagaagtctttagatcgaatggcctaacagctaatgagtcaggaaggctaatagatttcttaaaggatgtaatggaatcaatgaacaaagaggaaatagagataacaacccactttcaaagaaaaaggagagtaagagacaacataaccaagaagatggtcacgcaaagaacaatagggaagaaaaaacaaagactgaataagagaggctatctaataagagcactgacattaaatacgatgaccaaagatgcagagagaggcaagttaaaaagaagggctatcgcaacacctgggatgcagattagaggtttcgtatactttgttgaaactttagctaggagcatttgcgaaaagcttgaacagtctgggctcccagtagggggcaatgaaaagaaggccaaactggcaaatgttgtgagaaagatgatgactaattcacaagacacagagatttctttcacaatcactggggacaacactaagtggaatgaaaatcaaaatcctcgaatgttcctggcgatgattacatatatcaccagaaatcaacccgagtggttcagaaacatcctgagcatggcacccataatgttctcaaacaaaatggcaaggctagggaaagggtacatgttcgagagtaaaagaatgaagattcgaacacaaataccagcagaaatgctagcaagcattgacctgaagtacttcaatgaatcaacaaagaagaaaattgagaaaataaggcctcttctaatagatggcacagcatcactgagtcctgggatgatgatgggcatgttcaacatgctaagtacggtcttgggagtctcgatactgaatcttggacaaaagaaatacaccaagacagtatactggtgggatgggctccaatcatccgacgattttgctctcatagtgaatgcaccaaaccatgagggaatacaagcaggagtggacagattctacaggacctgcaagttagtgggaatcaacatgagcaaaaagaagtcctatataaataagacagggacatttgaattcacaagctttttttatcgctatggatttgtggctaattttagcatggagctacccagctttggagtgtctggagtaaatgaatcagctgacatgagtattggagtaacagtgataaagaacaacatgataaacaatgaccttggacctgcaacggcccagatggctcttcaattgttcatcaaagactacagatacacatataggtgccataggggagacacacaaattcagacgagaagatcatttgagttaaagaagctgtgggatcaaacccaatcaaaggtagggctattagtatcagatggaggaccaaacttatacaatatacggaatcttcacattcctgaagtctgcttaaaatgggagctaatggatgatgattatcggggaagactttgtaatcccctgaatccctttgtcagtcataaagagattgattctgtaaacaatgctgtggtaatgccagcccatggtccagccaaaagcatggaatatgatgccgttgcaactacacattcctggattcccaagaggaatcgttctattctcaacacaagccaaaggggaattcttgaggatgaacagatgtaccagaagtgctgcaatctattcgagaaatttttccctagcagttcatataggagaccggttggaatttctagcatggtggaggccatggtgtctagggcccggattgatgccagggtcgacttcgagtctggacggatcaagaaagaagagttctctgagatcatgaagatctgttccaccattgaagaactcagacggcaaaaataa

>H1N1_PB1_CY058322_A/Wisconsin/629-S1414/2009

atggatgtcaatccgactctacttttcctaaaaattccagcgcaaaatgccataagcaccacattcccttatactggagatcctccatacagccatggaacaggaacaggatacaccatggacacagtaaacagaacacaccaatactcagaaaagggaaagtggacgacaaacacagagactggtgcaccccagctcaacccgattgatggaccactacctgaggataatgaaccaagtgggtatgcacaaacagactgtgttctagaggctatggctttccttgaagaatcccacccaggaatatttgagaattcatgccttgaaacaatggaagttgttcaacaaacaagggtagataaactaactcaaggtcgccagacttatgattggacattaaacagaaatcaaccggcagcaactgcattggccaacaccatagaagtctttagatcaaatggcctaacagctaatgagtcaggaaggctaatagatttcttaaaggatgtaatggaatcaatgaacaaagaggaaatagagataacaacccactttcaaagaaaaaggagagtaagagacaacatgaccaagaagatggtcacgcaaagaacaatagggaagaaaaaacaaagactgaataagagaggctatctaataagagcactgacattaaatacgatgaccaaagatgcagagagaggcaagttaaaaagaagggctatcgcaacacctgggatgcagattagaggtttcgtatactttgttgaaactttagctaggagcatttgcgaaaagcttgaacagtctgggctcccagtagggggcaatgaaaagaaggccaaactggcaaatgttgtgagaaagatgatgactaattcacaagacacagagatttctttcacaatcactggggacaacactaagtggaatgaaaatcaaaatcctcgaatgttcctggcgatgattacatatatcaccagaaatcaacccgagtggttcagaaacatcctgagcatggcacccataatgttctcaaacaaaatggcaagactagggaaagggtacatgttcgagagtaaaagaatgaagattcgaacacaaataccagcagaaatgctagcaagcattgacctgaagtacttcaatgaatcaacaaagaagaaaattgagaaaataaggcctcttctaatagatggcacagcatcactgagtcctgggatgatgatgggtatgttcaacatgctaagtacggtcttgggagtctcgatactgaatcttggacaaaagaaatacaccaagacaatatattggtgggatgggctccaatcatccgacgattttgctctcatagtgaatgcaccaaaccatgagggaatacaagcaggagtggacagattctacaggacctgcaagttagtgggaatcaacatgagcaaaaagaagtcctatataaataagacagggacatttgaattcacaagctttttttatcgttatggatttgtggctaattttagcatggagctacccagctttggagtgtctggagtaaatgaatcagctgacatgagtattggagtaacagtgataaagaacaacatgataaacaatgaccttggacctgcaacggcccagatggctcttcaattgttcatcaaagactacagatacacatataggtgccataggggggacacacaaattcagacgagaagatcatttgagttaaagaagctgtgggatcaaacccaatcaaaggtagggctattagtatcagatggaggaccaaacttatacaatatacggaatcttcacattcctgaagtctgcttaaaatgggagctaatggatgatgattatcggggaagactttgtaatcccctgaatccctttgtcagtcataaagagattgattctgtaaacaatgctgtggtaatgccagcccatggtccagccaaaagcatggaatatgatgccgttgcaactacacattcctggattcccaagaggaatcgttctattctcaacacaagccaaaggggaattcttgaggatgaacagatgtaccagaagtgctgcaatctattcgagaaatttttccctagcagttcatataggagaccggttggaatttctagcatggtggaggccatggtgtctagggcccggattgatgccagggtcgacttcgagtctggacggatcaagaaagaagagttctctgagatcatgaagatctgttccaccattgaagaactcagacggcaaaaataa

>H1N1_PB1_CY058346_A/Wisconsin/629-D00503/2009

atggatgtcaatccgactctacttttcctaaaaattccagcgcaaaatgccataagcaccacattcccttatactggagatcctccatacagccatggaacaggaacaggatacaccatggacacagtaaacagaacacaccaatactcagaaaagggaaagtggacgacaaacacagagactggtgcaccccagctcaacccgattgatggaccactacctgaggataatgaaccaagtgggtatgcacaaacagactgtgttctagaggctatggctttccttgaagaatcccacccaggaatatttgagaattcatgccttgaaacaatggaagttgttcaacaaacaagggtagataaactaactcaaggtcgccagacttatgattggacattaaacagaaatcaaccggcagcaactgcattggccaacaccatagaagtctttagatcgaatggcctaacagctaatgagtcaggaaggctaatagatttcttaaaggatgtaatggaatcaatgaacaaagaggaaatagagataacaacccactttcaaagaaaaaggagagtaagagacaacatgaccaagaagatggtcacgcaaagaacaatagggaagaaaaaacaaagactgaataagagaggctatctaataagagcactgacattaaatacgatgaccaaagatgcagagagaggcaagttaaaaagaagggctatcgcaacacctgggatgcagattagaggtttcgtatactttgttgaaactttagctaggagcatttgcgaaaagcttgaacagtctgggctcccagtagggggcaatgaaaagaaggccaaactggcaaatgttgtgagaaagatgatgactaattcacaagacacagagatttctttcacaatcactggggacaacactaagtggaatgaaaatcaaaatcctcgaatgttcctggcgatgattacatatatcaccagaaatcaacccgagtggttcagaaacatcctgagcatggcacccataatgttctcaaacaaaatggcaagactagggaaagggtacatgttcgagagtaaaagaatgaagattcgaacacaaataccagcagaaatgctagcaagcattgacctgaggtacttcaatgaatcaacaaagaagaaaattgagaaaataaggcctcttctaatagatggcacagcatcactgagtcctgggatgatgatgggcatgttcaacatgctaagtacggtcttgggagtctcgatactgaatcttggacaaaagaaatacaccaagacaacatactggtgggatgggctccaatcatccgacgattttgctctcatagtgaatgcaccaaaccatgagggaatacaagcaggagtggacagattctacaggacctgcaagttagtgggaatcaacatgagcaaaaagaagtcctatataaataagacagggacatttgaattcacaagctttttttatcgctatggatttgtggctaattttagcatggagctacccagctttggagtgtctggagtaaatgaatcagctgacatgagtattggagtaacagtgataaagaacaacatgataaacaatgaccttggacctgcaacggcccagatggctcttcaattgttcatcaaagactacagatacacatataggtgccataggggagacacacaaattcagacgagaagatcatttgaattaaagaagctgtgggatcaaacccaatcaaaggtagggctattagtatcagatggaggaccaaacttatacaatatacggaatcttcacattcctgaagtctgcttaaaatgggagctaatggatgatgattatcggggaagactttgtaatcccctgaatccctttgtcagtcataaagagattgattctgtaaacaatgctgtggtaatgccagcccatggtccagccaaaagcatggaatatgatgccgttgcaactacacattcctggattcccaagaggaatcgttctattctcaacacaagccaaaggggaattcttgaggatgaacagatgtaccagaagtgctgcaatctattcgagaaatttttccctagcagttcatataggagaccggttggaatttctagcatggtggaggccatggtgtctagggcccggattgatgccagggtcgacttcgagtctggacggatcaagaaagaagagttctctgagatcatgaagatctgttccaccattgaagaactcagacggcaaaaataa

>H1N1_PB1_CY058378_A/Wisconsin/629-D01244/2009

atggatgtcaatccgactctacttttcctaaaaattccagcgcaaaatgccataagcaccacattcccttatactggagatcctccatacagccatggaacaggaacaggatacaccatggacacagtaaacagaacacaccaatactcagaaaagggaaagtggacgacaaacacagagactggtgcaccccagctcaacccgattgatggaccactacctgaggataatgaaccaagtgggtatgcacaaacagactgtgttctagaggctatggctttccttgaagaatcccacccaggaatatttgagaattcatgccttgaaacaatggaagttgttcaacaaacaagggtagacaaactaactcaaggtcgccagacttatgattggacattaaacagaaatcaaccggcagcaactgcattggccaacaccatagaagtctttagatcgaatggcctaacggctaatgagtcaggaaggctaatagatttcttaaaggatgtaatggaatcaatgaacaaagaggaaatagagataacaacccactttcaaagaaaaaggagagtaagagacaacatgaccaagaagatggtcacgcaaagaacaatagggaagaaaaaacaaagactgaataagagaggctatctaataagagcactgacattaaatacgatgaccaaagatgcagagagaggcaagttaaaaagaagggctatcgcaacacctgggatgcagattagaggtttcgtatactttgttgaaactttagctaggagcatttgcgaaaagcttgaacagtctgggctcccagtagggggcaatgaaaagaaggccaaactggcaaatgttgtgagaaagatgatgactaattcacaagacacagagatttctttcacaatcactggggacaacactaagtggaatgaaaatcaaaatcctcgaatgttcctggcgatgattacatatatcaccagaaatcaacccgagtggttcagaaacatcctgagcatggcacccataatgttctcaaacaaaatggcaagactagggaaagggtacatgttcgagagtaaaagaatgaagattcgaacacaaataccagcagaaatgctagcaagcattgacctgaagtacttcaatgaatcaacaaagaagaaaattgagaaaataaggcctcttctaatagatggcacagcatcactgagtcctgggatgatgatgggcatgttcaacatgctaagtacggtcttgggagtctcgatactgaatcttggacaaaagaaatacaccaagacaatatactggtgggatgggctccaatcatccgacgattttgctctcatagtgaatgcaccaaaccatgagggaatacaagcaggagtggacagattctacaggacctgcaagttagtgggaatcaacatgagcaaaaagaagtcctatataaataagacagggacatttgaattcacaagctttttttatcgctatggatttgtggctaattttagcatggagctacccagctttggagtgtctggagtaaatgaatcagctgacatgagtattggagtaacagtgataaagaacaacatgataaacaatgaccttggacctgcaacggcccagatggctcttcaattgttcatcaaagactacagatacacatataggtgccataggggagacacacaaattcagacgagaagatcatttgagttaaagaagctgtgggatcaaacccaatcaaaggtagggctattagtatcagatggaggaccaaacttatacaatatacggaatcttcacattcctgaagtctgcttaaaatgggagctaatggatgatgattatcggggaagactttgtaatcccctgaatccctttgtcagtcataaagagattgattctgtaaacaatgctgtggtaatgccagcccatggtccagccaaaagcatggaatatgatgccgttgcaactacacattcctggattcccaagaggaatcgttctattctcaacacaagccaaaggggaattcttgaggatgaacagatgtaccagaagtgctgcaatctattcgagaaatttttccctagcagttcatataggagaccggttggaatttctagcatggtggaggccatggtgtctagggcccggattgatgccagggtcgacttcgagtctggacggatcaagaaagaagagttctctgagatcatgaagatctgttccaccattgaagaactcagacggcaaaaataa

>H1N1_PB1_CY060644_A/Ontario/314603/2009

atggatgtcaatccgactctacttttcctaaaaattccagcgcaaaatgccataagcaccacattcccttatactggagatcctccatacagccatggaacaggaacaggatacaccatggacacagtaaacagaacacaccaatactcagaaaagggaaagtggacgacaaacacagagactggtgcaccccagctcaacccgattgatggaccactacctgaggataatgaaccaagtgggtatgcacaaacagactgtgttctagaggctatggctttccttgaagaatcccacccaggaatatttgagaattcatgccttgaaacaatggaagttgttcaacaaacaagggtagataaactaactcaaggtcgccagacttatgattggacattaaacagaaatcaaccggcagcaactgcattggccaacaccatagaagtctttagatcgaatggcctaacagctaatgagtcaggaaggctaatagatttcttaaaggatgtaatggaatcaatgaacaaagaggaaatagagataacaacccactttcaaagaaaaaggagagtaagagacaacatgaccaagaagatggtcacgcaaagaacaatagggaagaaaaaacaaagactgaataagagaggctatctaataagagcactgacattaaatacgatgaccaaagatgcagagagaggcaagttaaaaagaagggctatcgcaacacctgggatgcagattagaggtttcgtatactttgttgaaactttagctaggagcatttgcgaaaagcttgaacagtctgggctcccagtagggggcaatgaaaagaaggccaaactggcaaatgttgtgagaaagatgatgactaattcacaagacacagagatttctttcacaatcactggggacaacactaagtggaatgaaaatcaaaatcctcgaatgttcctggcgatgattacatatatcaccagaaatcaacccgagtggttcagaaacatcctgagcatggcacccataatgttctcaaacaaaatggcaagactagggaaagggtacatgttcgagagtaaaagaatgaagattcgaacacaaataccagcagaaatgctagcaagcattgacctgaagtacttcaatgaatcaacaaagaagaaaattgagaaaataaggcctcttctaatagatggcacagcatcactgagtcctgggatgatgatgggcatgttcaacatgctaagtacggtcttgggagtctcgatactgaatcttggacaaaagaaatacaccaagacaatatactggtgggatgggctccaatcatccgacgattttgctctcatagtgaatgcaccaaaccatgagggaatacaagcaggagtggacagattctacaggacctgcaagttagtgggaatcaacatgagcaaaaagaagtcctatataaataagacagggacatttgaattcacaagctttttttatcgctatggatttgtggctaattttagcatggagctacccagctttggagtgtctggagtaaatgaatcagctgacatgagtattggagtaacagtgataaagaacaacatgataaacaatgaccttggacctgcaacggcccagatggctcttcaattgttcatcaaagactacagatacacatataggtgccataggggagacacacaaattcagacgagaagatcatttgagttaaagaagctgtgggatcaaacccaatcaaaggtagggctattagtatcagatggaggaccaaacttatacaatatacggaatcttcacattcctgaagtctgcttaaaatgggagctaatggatgatgattatcggggaagactttgtaatcccctgaatccctttgtcagtcataaagagattgattctgtaaacaatgctgtggtaatgccagcccatggtccagccaaaagcatggaatatgatgccgttgcaactacacattcctggattcccaagaggaatcgttctattctcaacacaagccaaaggggaattcttgaggatgaacagatgtaccagaagtgctgcaatctattcgagaaatttttccctagcagttcatataggagaccggttggaatttctagcatggtggaggccatggtgtctagggcccggattgatgccagggtcgacttcgagtctggacggatcaagaaagaagagttctctgagatcatgaagatctgttccaccattgaagaactcagacggcaaaaataa

>H1N1_PB1_CY060652_A/Ontario/315003/2009

atggatgtcaatccgactctacttttcctaaaaattccagcacaaaatgccataagcaccacattcccttatactggagatcctccatacagccatggaacaggaacaggatacaccatggacacagtaaacagaacacaccaatactcagaaaagggaaagtggacgacaaacacagagactggtgcaccccagctcaacccgattgatggaccactacctgaggataatgaaccaagtgggtatgcacaaacagactgtgttctagaggctatggctttccttgaagaatcccacccaggaatatttgagaattcatgccttgaaacaatggaagttgttcaacaaacaagggtagataaactaactcaaggtcgccagacttatgattggacattaaacagaaatcaaccggcagcaactgcattggccaacaccatagaagtctttagatcgaatggcctaacagctaatgagtcaggaaggctaatagatttcttaaaggatgtaatggaatcaatgaacaaagaggaaatagagataacaacccactttcaaagaaaaaggagagtaagagacaacatgaccaagaagatggtcacgcaaagaacaatagggaagaaaaaacaaagactgaataagagaggctatctaataagagcactgacattaaatacgatgaccaaagatgcagagagaggcaagttaaaaagaagggctatcgcaacacctgggatgcagattagaggtttcgtatactttgttgaaactttagctaggagcatttgcgaaaagcttgaacagtctgggctcccagtagggggcaatgaaaagaaggccaaactggcaaatgttgtgagaaagatgatgactaattcacaagacacagagatttctttcacaatcactggggacaacactaagtggaatgaaaatcaaaatcctcgaatgttcctggcgatgattacatatatcaccagaaatcaacccgagtggttcagaaacatcctgagcatggcacccataatgttctcaaacaaaatggcaagactagggaaagggtacatgttcgagagtaaaagaatgaagattcgaacacaaataccagcagaaatgctagcaagcattgacctgaagtacttcaatgaatcaacaaagaagaaaattgagaaaataaggcctcttctaatagatggcacagcatcactgagtcctgggatgatgatgggcatgttcaacatgctaagtacggtcttgggagtctcgatactgaatcttggacaaaagaaatacaccaagacaatatactggtgggatgggctccaatcatccgacgattttgctctcatagtgaatgcaccaaaccatgagggaatacaagcaggagtggacagattctacaggacctgcaagttagtgggaatcaacatgagcaaaaagaagtcctatataaataagacagggacatttgaattcacaagctttttttatcgctatggatttgtggctaattttagcatggagctacccagctttggagtgtctggagtaaatgaatcagctgacatgagtattggagtaacagtgataaagaacaacatgataaacaatgaccttggacctgcaacggcccagatggctcttcaattgttcatcaaagactacagatacacatataggtgccataggggagacacacaaattcagacgagaagatcatttgagttaaagaagctgtgggatcaaacccaatcaaaggtagggctattagtatcagatggaggaccaaacttatacaatatacggaatcttcacattcctgaagtctgcttaaaatgggagctaatggatgatgattatcggggaagactttgtaatcccctgaatccctttgtcagtcataaagagattgattctgtaaacaatgctgtggtaatgccagcccatggtccagccaaaagcatggaatatgatgccgttgcaactacacattcctggattcccaagaggaatcggtctattctcaacacaagccaaaggggaattcttgaggatgaacagatgtaccagaagtgctgcaatctattcgagaaatttttccctagcagttcatataggagaccggttggaatttctagcatggtggaggccatggtgtctagggcccggattgatgccagggtcgacttcgagtctggacggatcaagaaagaagagttctctgagatcatgaagatctgttccaccattgaagaactcagacggcaaaaataa

>H1N1_PB1_CY060692_A/Ontario/315187/2009

atggatgtcaatccgactctacttttcctaaaaattccagcgcaaaatgccataagcaccacattcccttatactggagatcctccatacagccatggaacaggaacaggatacaccatggacacagtaaacagaacacaccaatactcagaaaagggaaagtggacgacaaacacagagactggtgcaccccagctcaacccgattgatggaccactacctgaggataatgaaccaagtgggtatgcacaaacagactgtgttctagaggctatggctttccttgaagaatcccacccaggaatatttgagaattcatgccttgaaacaatggaagttgttcaacaaacaagggtagataaactaactcaaggtcgccagacttatgattggacattaaacagaaatcaaccggcagcaactgcattggccaacaccatagaagtctttagatcgaatggcctaacagctaatgagtcaggaaggctaatagatttcttaaaggatgtaatggaatcaatgaacaaagaggaaatagagataacaacccactttcaaagaaaaaggagagtaagagacaacatgaccaagaagatggtcacgcaaagaacaatagggaagaaaaaacaaagactgaataagagaggctatctaataagagcactgacattaaatacgatgaccaaagatgcagagagaggcaagttaaaaagaagggctatcgcaacacctgggatgcagattagaggtttcgtatactttgttgaaactttagctaggagcatttgcgaaaagcttgaacagtctgggctcccagtagggggcaatgaaaagaaggccaaactggcaaatgttgtgagaaagatgatgactaattcacaagacacagagatttctttcacaatcactggggacaacactaagtggaatgaaaatcaaaatcctcgaatgttcctggcgatgattacatatatcaccagaaatcaacccgagtggttcagaaacatcctgagcatggcacccataatgttctcaaacaaaatggcaagactagggaaagggtacatgttcgagagtaaaagaatgaagattcgaacacaaataccagcagaaatgctagcaagcattgacctgaagtacttcaatgaatcaacaaagaagaaaattgagaaaataaggcctcttctaatagatggcacagcatcactgagtcctgggatgatgatgggcatgttcaacatgctaagtacggtcttgggagtctcgatactgaatcttggacaaaagaaatacaccaagacaatatactggtgggatgggctccaatcatccgacgattttgctctcatagtgaatgcaccaaaccatgagggaatacaagcaggagtggacagattctacaggacctgcaagttagtgggaatcaacatgagcaaaaagaagtcctatataaataagacagggacgtttgaattcacaagctttttttatcgctatggatttgtggctaattttagcatggagctacccagctttggagtgtctggagtaaatgaatcagctgacatgagtattggagtaacagtgataaagaacaacatgataaacaatgaccttggacctgcaacggcccagatggctcttcaattgttcatcaaagactacagatacacatataggtgccataggggagacacacaaattcagacgagaagatcatttgagctaaagaagctgtgggatcaaacccaatcaaaggtagggctattagtatcagatggaggaccaaacttatacaatatacggaatcttcacattcctgaagtctgcttaaaatgggagctaatggatgatgattatcggggaagactttgtaatcccctgaatccctttgtcagtcataaagagattgattctgtaaacaatgctgtggtaatgccagcccatggtccagccaaaagcatggaatatgatgccgttgcaactacacattcctggattcccaagaggaatcgttctattctcaacacaagccaaaggggaattcttgaggatgaacagatgtaccagaagtgctgcaatctattcgagaaatttttccctagcagttcatataggagaccggttggaatttctagcatggtggaggccatggtgtctagggcccggattgatgccagggtcgacttcgagtctggacggatcaagaaagaagagttctctgagatcatgaagatctgttccaccattgaagaactcagacggcaaaaataa

>H1N1_PB1_CY060849_A/Texas/JMS356/2009

atggatgtcaatccgactctacttttcctaaaaattccagcgcaaaatgccataagcaccacattcccttatactggagatcctccatacagccatggaacaggaacaggatacaccatggacacagtaaacagaacacaccaatactcagaaaagggaaagtggacgacaaacacagagactggtgcaccccagctcaacccgattgatggaccactacctgaggataatgaaccaagtgggtatgcacaaacagactgtgttctagaggctatggctttccttgaagaatcccacccaggaatatttgagaattcatgccttgaaacaatggaagttgttcaacaaacaagggtagataaactaactcaaggtcgccagacttatgattggacattaaacagaaatcaaccggcagcaactgcattggccaacaccatagaagtctttagatcgaatggcctaacagctaatgagtcaggaaggctaatagatttcttaaaggatgtaatggaatcaatgaacaaagaggaaatagagataacaacccactttcaaagaaaaaggagagtaagagacaacatgaccaagaagatggtcacgcaaagaacaatagggaagaaaaaacaaagactgaataagagaggctatctaataagagcactgacattaaatacgatgaccaaagatgcagagagaggcaagttaaaaagaagggctatcgcaacacctgggatgcagattagaggtttcgtatactttgttgaaactttagctaggagcatttgcgaaaagcttgaacagtctgggctcccagtagggggcaatgaaaagaaggccaaactggcaaatgttgtgagaaagatgatgactaattcacaagacacagagatttctttcacaatcactggggacaacactaagtggaatgaaaatcaaaatcctcgaatgttcctggcgatgattacatatatcaccagaaatcaacccgagtggttcagaaacatcctgagcatggcacccataatgttctcaaacaaaatggcaagactagggaaagggtacatgttcgagagtaaaagaatgaagattcgaacacaaataccagcagaaatgctagcaagcattgacctgaagtacttcaatgaatcaacaaagaagaaaattgagaaaataaggcctcttctaatagatggcacagcatcactgagtcctgggatgatgatgggcatgttcaacatgctaagtacggtcttgggagtctcgatactgaatcttggacaaaagaaatacaccaagacagtatactggtgggatgggctccaatcatccgacgattttgctctcatagtgaatgcaccaaaccatgagggaatacaagcaggagtggacagattctacaggacctgcaagttagtgggaatcaacatgagcaaaaagaagtcctatataaataagacagggacatttgaattcacaagctttttttatcgctatggatttgtggctaattttagcatggagctacccagctttggcgtgtctggagtaaatgaatcagctgacatgagtattggagtaacagtgataaagaacaacatgataaacaatgaccttggacctgcaacggcccagatggctcttcaattgttcatcaaagactacagatacacatataggtgccataggggagacacacaaattcagacgagaagatcatttgagttaaagaagctgtgggatcaaacccaatcaaaggtagggctattagtatcagatggaggaccaaacttatacaatatacggaaccttcacattcctgaagtctgcttaaaatgggagctaatggatgatgattatcggggaagactttgtaatcccctgaatccctttgtcagtcataaagagattgattctgtaaacaatgctatggtaatgccagcccatggtccagccaaaagcatggaatatgatgccgttgcaactacacattcctggattcccaagaggaatcgttctattctcaacacaagccaaaggggaattcttgaggatgaacagatgtaccagaagtgctgcaatctattcgagaaatttttccctagcagttcatataggagaccggttggaatttctagcatggtggaggccatggtgtctagggcccggattgatgccagggtcgacttcgagtctggacggatcaagaaagaagagttctctgagatcatgaagatctgttccaccattgaagaactcagacggcaaaaataa

>H1N1_PB1_CY060929_A/Texas/JMS371/2009

atggatgtcaatccgactctacttttcctaaaaattccagcgcaaaatgccataagcaccacattcccttatactggagatcctccatacagccatggaacaggaacaggatacaccatggacacagtaaacagaacacaccaatactcagaaaaaggaaagtggacgacaaacacagagactggtgcaccccagctcaacccgattgatggaccactacctgaggataatgaaccaagtgggtatgcacaaacagactgtgttctagaggctatggctttccttgaagaatcccacccaggaatatttgagaattcatgccttgaaacaatggaagttgttcaacaaacaagggtagataaactaactcaaggtcgccagacttatgattggacattaaacagaaatcaaccggcagcaactgcattggccaacaccatagaagtctttagatcgaatggcctaacagctaatgagtcaggaaggctaatagatttcttaaaggatgtaatggaatcaatgaacaaagaggaaatagagataacaacccactttcaaagaaaaaggagagtaagagacaacatgaccaagaagatggtcacgcaaagaacaatagggaagaaaaaacaaagactgaataagagaggctatctaataagagcactgacattaaatacgatgaccaaagatgcagagagaggcaagttaaaaagaagggctatcgcaacacctgggatgcagattagaggtttcgtatactttgttgaaactttagctaggagcatttgcgaaaagcttgaacagtctgggctcccagtagggggcaatgaaaagaaggccaaactggcaaatgttgtgagaaagatgatgactaattcacaagacacagagatttctttcacaatcactggggacaacactaagtggaatgaaaatcaaaatcctcgaatgttcctggcgatgattacatatatcaccagaaatcaacccgagtggttcagaaacatcctgagcatggcacccataatgttctcaaacaaaatggcaagactagggaaagggtacatgttcgagagtaaaagaatgaagattcgaacacaaataccagcagaaatgctagcaagcattgacctgaagtacttcaatgaatcaacaaagaagaaaattgagaaaataaggcctcttctaatagatggcacagcatcactgagtcctgggatgatgatgggcatgttcaacatgctaagtacggtcttgggagtctcgatactgaatcttggacaaaagaaatacaccaagacaatatactggtgggatgggctccaatcatccgacgattttgctctcatagtgaatgcaccaaaccatgagggaatacaagcaggagtggacagattctacaggacctgcaagttagtgggaatcaacatgagcaaaaagaagtcctatataaataagacagggacatttgaattcacaagctttttttatcgctatggatttgtggctaattttagcatggagctacccagctttggagtgtctggagtaaatgaatcagctgacatgagtattggagtaacagtgataaagaacaacatgataaacaatgaccttggacctgcaacggcccagatggctcttcaattgttcatcaaagactacagatacacatataggtgccataggggagacacacaaattcagacgagaagatcatttgagttaaagaagctgtgggatcaaacccaatcaaagggagggctattagtatcagatggaggaccaaacttatacaatatacggaatcttcacattcctgaagtctgcttaaaatgggagctaatggatgatgattatcggggaagactttgtaatcccctgaatccctttgtcagtcataaagagattgattctgtaaacaatgctgtggtaatgccagcccatggtccagtcaaaagcatggaatatgatgccgttgcaactacacattcctggattcccaagaggaatcgttctattctcaacacaagccaaaggggaattcttgaggatgaacagatgtaccagaagtgctgcaatctattcgagaaatttttccctagcagttcatataggagaccggttggaatttctagcatggtggaggccatggtgtctagggcccggattgatgccagggtcgacttcgagtctggacggatcaagaaagaagagttctctgagatcatgaagatctgctccaccattgaagaactcagacggcaaaaataa

>H1N1_PB1_CY061033_A/Texas/JMS389/2009

atggatgtcaatccgactctacttttcctaaaaattccagcgcaaaatgccataagcaccacattcccttatactggagatcctccatacagccatggaacaggaacaggatacaccatggacacagtaaacagaacacaccaatactcagaaaagggaaagtggacgacaaacacagagactggtgcaccccagctcaacccgattgatggaccactacctgaggataatgaaccaagtgggtatgcacaaacagactgtgttctagaggctatggctttccttgaagaatcccacccaggaatatttgagaattcatgccttgaaacaatggaagttgttcaacaaacaagggtagataaactaactcaaggtcgccagacttatgattggacattaaacagaaatcaaccggcagcaactgcattggccaacaccatagaagtctttagatcgaatggcctaacagctaatgagtcaggaaggctaatagatttcttaaaggatgtaatggaatcaatgaacaaagaggaaatagagataacaacccactttcaaagaaaaaggagagtaagagacaacatgaccaagaagatggtcacgcaaagaacaatagggaagaaaaaacaaagactgaataagagaggctatctaataagagcactgacattaaatacgatgaccaaagatgcagagagaggcaagttaaaaagaagggctatcgcaacacctgggatgcagattagaggtttcgtatactttgttgaaactttagctaggagcatttgcgaaaagcttgaacagtctgggctcccagtagggggcaatgaaaagaaggccaaactggcaaatgttgtgagaaagatgatgactaattcacaagacacagagatttctttcacaatcactggggacaacactaagtggaatgaaaatcaaaatcctcgaatgttcctggcgatgattacatatatcaccagaaatcaacctgagtggttcagaaacatcttgagcatggcacccataatgttctcaaacaaaatggcaagactagggaaagggtacatgttcgagagtaaaagaatgaagattcgaacacaaataccagcagaaatgctagcaagcattgacctgaagtacttcaatgaatcaacaaagaagaaaattgagaaaataaggcctcttctaatagatggcacagcatcactgagtcctgggatgatgatgggcatgttcaacatgctcagtacggtcttgggagtctcgatactgaatcttggacaaaagaaatacaccaagacaatatactggtgggatgggctccaatcatccgacgattttgctctcatagtgaatgcaccaaaccatgagggaatacaagcaggagtggacagattctacaggacctgcaagttagtgggaatcaacatgagcaaaaagaagtcctatataaataagacagggacatttgaattcacaagctttttttatcgctatggatttgtggctaattttagcatggagctacccagctttggagtgtctggagtaaatgaatcagctgacatgagtattggagtaacagtgataaagaacaacatgataaacaatgaccttggacctgcaacggcccaaatggctcttcaactgttcatcaaagactacagatacacatataggtgccataggggagacacacaaattcagacgagaagatcatttgagttaaagaagctgtgggatcaaacccaatcaaaggtagggctactagtatcagatggaggaccaaacttatacaatatacggaatcttcacattcctgaagtctgcttaaaatgggagctaatggatgatgattatcggggaagactttgtaatcccctgaatccctttgtcagtcataaagagattgattctgtaaacaatgctgtggtaatgccagcccatggtccagccaaaagcatggaatatgatgccgttgcaactacacattcctggattcccaagaggaatcgttctattctcaacacaagccaaaggggaattcttgaggatgaacagatgtaccagaagtgctgcaatctattcgagaaatttttccctagcagttcatataggagaccggttggaatttctagcatggtggaggccatggtgtctagggcccggattgatgccagggtcgacttcgagtctggacgaatcgggaaggaagagttctctgagatcatgaagatctgttccaccattgaagaactcagacggcaaaaataa

>H1N1_PB1_CY061161_A/Texas/JMS409/2010

atggatgtcaatccgactctacttttcctaaaaattccagcgcaaaatgccataagcaccacattcccttatactggagatcctccatacagccatggaacaggaacaggatacaccatggacacagtaaacagaacacaccaatactcagaaaagggaaagtggacgacaaacacagagactggtgcaccccagctcaacccgattgatggaccactacctgaggataatgaaccaagtgggtatgcacaaacagactgtgttctagaggctatggctttccttgaagaatcccacccaggaatatttgagaattcatgccttgaaacaatggaagttgttcaacaaacaagggtagataaactaactcaaggtcgccagacttatgattggacattaaacagaaatcaaccggcagcaactgcattggccaacaccatagaagtctttagatcgaatggcctaacagctaatgagtcaggaaggctaatagatttcttaaaggatgtaatggaatcaatgaacaaagaggaaatagagataacaacccactttcaaagaaaaaggagagtaagagacaacatgaccaagaagatggtcacgcaaagaacaatagggaagaaaaaacaaagactgaataagagaggctatctaataagagcactgacattaaatacgatgaccaaagatgcagagagaggcaagttaaaaagaagggctatcgcaacacctgggatgcagattagaggtttcgtatactttgttgaaactttagctaggagcatttgcgaaaagcttgaacagtctgggctcccagtagggggcaatgaaaagaaggccaaactggcaaatgttgtgagaaagatgatgactaattcacaagacacagagatttctttcacaatcactggggacaacactaagtggaatgaaaatcaaaatcctcgaatgttcctggcgatgattacatatatcaccagaaatcaacccgagtggttcagaaacatcctgagcatggcacccataatgttctcaaacaaaatggcaagactagggaaagggtacatgttcgagagtaaaagaatgaagattcgaacacaaataccagcagaaatgctagcaagcattgacctgaagtacttcaatgaatcaacaaagaagaaaattgagaaaataaggcctcttctaatagatggcacagcatcactgagtcctgggatgatgatgggcatgttcaacatgctaagtacggtcttgggagtctcgatactgaatcttggacaaaagaaatacaccaagacaatatactggtgggatgggctccaatcatccgacgattttgctctcatagtgaatgcaccaaaccatgagggaatacaagcaggagtggacagattctacaggacctgcaagttagtgggaatcaacatgagcaaaaagaagtcctatataaataagacagggacatttgaattcacaagctttttttatcgctatggatttgtggctaattttagcatggagctacccagctttggagtgtctggagtaaatgaatcagctgacatgagtattggagtaacagtgataaagaacaacatgataaacaatgaccttggacctgcaacggcccagatggctcttcaactgttcatcaaagactacagatacacatataggtgccataggggggacacacaaattcagacgagaagatcatttgagttaaagaagctgtgggatcaaacccaatcaaaggtagggctattagtatcagatggaggaccaaacttatacaatatacggaatcttcacattcctgaagtctgcttaaaatgggagctaatggatgatgattatcggggaagactttgtaatcccctgaatccctttgtcagtcataaagagattgattctgtaaacaatgctgtggtaatgccagcccatggtccagccaaaagcatggaatatgatgccgttgcaactacacattcctggattcccaagaggaatcgttctattctcaacacaagccaaaggggaattcttgaggatgaacagatgtaccagaagtgctgcaatctattcgagaaatttttccctagcagttcatataggagaccggttggaatttctagcatggtggaggccatggtgtctagggcccggattgatgccagggtcgacttcgagtctggacggatcgggaaggaagagttctctgagatcatgaagatctgttccaccattgaagaactcagacggcaaaaataa

>H1N1_PB1_CY061185_A/Texas/JMS412/2010

atggatgtcaatccgactctacttttcctaaaaattccagcgcaaaatgccataagcaccacattcccttatactggagatcctccatacagccatggaacaggaacaggatacaccatggacacagtaaacagaacacaccaatactcagaaaagggaaagtggacgacaaacacagagactggtgcaccccagctcaacccgattgatggaccactacctgaggataatgaaccaagtgggtatgcacaaacagactgtgttctagaggctatggctttccttgaagaatcccacccaggaatatttgagaattcatgccttgaaacaatggaagttgttcaacaaacaagggtagataaactaactcaaggtcgccagacttatgattggacattaaacagaaatcaaccggcagcaactgcattggccaacaccatagaagtctttagatcgaatggcctaacagctaatgagtcaggaaggctaatagatttcttaaaggatgtaatggaatcaatgaacaaagaggaaatagagataacaacccactttcaaagaaaaaggagagtaagagacaacatgaccaagaagatggtcacgcaaagaacaatagggaagaaaaaacaaagactgaataagagaggctatctaataagagcactgacattaaatacgatgaccaaagatgcagagagaggcaagttaaaaagaagggctatcgcaacacctgggatgcagattagaggtttcgtatactttgttgaaactttagctaggagcatttgcgaaaagcttgaacagtctgggctcccagtagggggcaatgaaaagaaggccaaactggcaaatgttgtgagaaagatgatgactaattcacaagacacagaaatttctttcacaatcactggggacaacactaagtggaatgaaaatcaaaatcctcgaatgttcctggcgatgattacatatatcaccagaaatcaacccgagtggttcagaaacatcttgagcatggcacccataatgttctcaaacaaaatggcaagactagggaaagggtacatgttcgagagtaaaagaatgaagattcgaacacaaataccatcagaaatgctagcaagcattgacctgaagtacttcaatgaatcaacaaagaagaaaattgagaaaataaggcctcttctaatagatggcacagcatcactgagtcctgggatgatgatgggcatgttcaacatgctaagtacggtcttgggagtctcgatactgaatcttggacaaaagaaatacaccaagacaatatactggtgggatgggctccaatcatccgacgattttgctctcatagtgaatgcatcaaaccatgagggaatacaagcaggagtggacagattctacaggacctgcaagttagtgggaatcaacatgagcaaaaagaagtcctatataaataagacagggacatttgaattcacaagctttttttatcgctatggatttgtggctaattttagcatggagctacccagctttggagtgtctggagtaaatgaatcagctgacatgagtattggagtaacagtgataaagaacaacatgataaacaatgaccttggacctgcaacggcccagatggctcttcaactgttcatcaaagactacagatacacatataggtgccataggggagacacacaaattcagacgagaagatcatttgagttaaagaagctgtgggatcaaacccaatcaaaggtagggctattagtatcagatggaggaccaaacttatacaatatacggaatcttcacattcctgaagtctgcttaaaatgggagctaatggatgatgattatcggggaagactttgtaatcccctgaatccctttgtcagtcataaagagattgattctgtaaacaatgctgtggtaatgccagcccatggtccagccaaaagcatggaatatgatgccgttgcaactacacattcctggattcccaagaggaatcgttctattctcaacacaagccaaaggggaattcttgaggatgaacagatgtaccagaagtgctgcaatctattcgagaaatttttccctagcagttcatataggagaccggttggaatttctagcatggtggaggccatggtgtctagggcccggattgatgccagggtcgacttcgagtctggacgaatcgggaaggaagagttctctgagatcatgaagatctgttccaccattgaagaactcagacggcaaaaataa

>H1N1_PB1_CY061281_A/Pensacola/INS107/2009

atggatgtcaatccgactctacttttcctaaaaattccagcgcaaaaagccataagcaccacattcccttatactggagatcctccatacagccatggaacaggaacaggatacaccatggacacagtaaacagaacacaccaatactcagaaaagggaaagtggacaacaaacacagagactggtgcaccccagctcaacccgattgatggaccactacctgaggataatgaaccaagtgggtatgcacaaacagactgtgttttagaggctatggctttccttgaagaatcccacccaggaatatttgagaattcatgccttgaaacaatggaagttgttcaacaaacaagggtagataaactaactcaaggtcgccagacttatgattggacattaaacagaaatcaaccggcagcaactgcattggccaacaccatagaagtctttagatcgaatggcctaacagctaatgagtcaggaaggctaatagatttcttaaaggatgtaatggaatcaatgaacaaagaggaaatagagataacaacccactttcaaagaaaaaggagagtaagagacaacatgaccaagaagatggtcacgcaaagaacaatagggaagaaaaaacaaagactgaataagagaggctatctaataagagcactgacattaaatacgatgaccaaagatgcagagagaggcaagttaaaaagaagggctatcgcaacacctgggatgcagattagaggtttcgtatactttgttgaaactttagctaggagcatttgcgaaaagcttgaacagtctgggctcccagtagggggcaatgaaaagaaggccaaactggcaaatgttgtgagaaagatgatgactaattcacaagacacagagatttctttcacaatcactggggacaacactaagtggaatgaaaatcaaaatcctcgaatgttcctggcgatgattacatatatcaccagaaatcaacccgagtggttcagaaacatcctgagcatggcacccataatgttctcaaacaaaatggcaagactagggaaagggtacatgttcgagagtaaaagaatgaagattcgaacacaaataccagcagaaatgctagcaagcattgacctgaagtacttcaatgaatcaacaaagaagaagattgagaaaataaggcctcttctaatagatggcacagcatcactgagtcctgggatgatgatgggcatgttcaacatgctaagtacggtcttgggagtctcgatactgaatcttggacaaaagaaatacaccaagacaatatactggtgggatgggctccaatcatccgacgattttgctctcatagtgaatgcaccaaaccatgagggaatacaagcaggagtggacagattctacaggacctgcaagttagtgggaatcaacatgagcaaaaagaagtcctatataaataagacagggacatttgaattcacaagctttttttatcgctatggatttgtggctaattttagcatggagctacccagctttggagtgtctggagtaaatgaatcagctgacatgagtattggagtaacagtgataaagaacaacatgataaacaatgaccttggacctgcaacggcccagatggctcttcaattgttcatcaaagactacagatacacatataggtgccataggggagacacacaaattcagacgagaagatcatttgagttaaagaagctgtgggatcaaacccaatcaaaggtagggctattagtatcagatggaggaccaaacttatacaatatacggaatcttcacattcctgaagtctgcttaaaatgggagctaatggatgatgattatcggggaagactttgtaatcccctgaatccctttgtcagtcataaagagattgattctgtaaacaatgctgtggtaatgccagcccatggtccagccaaaagcatggaatatgatgccgttgcaactacacattcctggattcccaagaggaatcgttctattctcaacacaagccaaaggggaattcttgaggatgaacagatgtaccagaagtgctgcaatctattcgagaaatttttccctagcagttcatataggagaccggttggaatttctagcatggtggaggccatggtgtctagggcccggattgatgccagggtcgacttcgagtctggacggatcaagaaagaagagttctctgagatcatgaagatctgttccaccattgaagaactcagacggcaaaaataa

>H1N1_PB1_CY062040_A/New York/0259/2009

atggatgtcaatccgactctacttttcctaaaaattccagcgcaaaatgccataagcaccacattcccctatactggagatcctccatacagccatggaacaggaacaggatacaccatggacacagtaaacagaacacaccaatactcagaaaagggaaagtggacgacaaacacagagactggtgcaccccagctcaacccgattgatggaccactacctgaggataatgaaccaagtgggtatgcacaaacagactgtgttctagaggctatggcttttcttgaagaatcccacccaggaatatttgagaattcatgccttgaaacaatggaagttgttcaacaaacaagggtagataaactaactcaaggtcgccagacttatgattggacattaaacagaaatcaaccggcagcaactgcattggccaacaccatagaagtctttagatcgaatggcctaacagctaatgagtcaggaaggctaatagatttcttaaaggatgtaatggaatcaatgaacaaagaggaaatagagataacaacccactttcaaagaaaaaggagagtaagagacaacatgaccaagaagatggtcacgcaaagaacaatagggaagaaaaaacaaagactgaataagagaggctatctaataagagcactgacattaaatacgatgaccaaagatgcagagagaggcaagttaaaaagaagggctatcgcaacacctgggatgcagattagaggtttcgtatactttgttgaaactttagctaggagcatttgcgaaaagcttgaacagtctgggctcccagtagggggcaatgaaaagaaggccaaactggcaaatgttgtgagaaagatgatgactaattcacaagacacagagatttctttcacaatcactggggacaacactaagtggaatgaaaatcaaaatcctcgaatgttcctggcgatgattacatatatcaccagaaatcaacccgagtggttcagaaacatcctgagcatggcacccataatgttctcaaacaaaatggcaagactagggaaagggtacatgttcgagagtaaaagaatgaagattcgaacacaaataccagcagaaatgctagcaagcattgacctgaaatacttcaatgaatcaacaaagaagaaaattgagaaaataaggcctcttctaatagatggcacagcatcactgagtcctgggatgatgatgggcatgttcaacatgctaagtacggtcttgggagtctcgatactgaatcttggacaaaagaaatacaccaagacaatatactggtgggatgggctccaatcatccgacgattttgctctcatagtgaatgcaccaaaccatgagggaatacaagcaggagtggacagattctacaggacctgcaagttagtgggaatcaacatgagcaaaaagaagtcctatataaataagacagggacatttgaattcacaagctttttttatcgctatgggtttgtggctaattttagcatggagctacccagctttggagtgtctggagtaaatgaatcagctgacatgagtattggagtaacagtgataaagaacaacatgataaacaatgaccttggacctgcaacggcccagatggctcttcaattgttcatcaaagactacagatacacatataggtgccataagggagacacacaaattcagacgagaagatcatttgagttaaagaagctgtgggatcaaacccaatcaaaggtagggctattagtatcagatggaggaccaaacttatacaatatacggaatcttcacattcctgaagtctgcttaaaatgggagctaatggatgatgattatcggggaagactttgtaatcccctgaatccctttgtcagtcataaagagattgattctgtaaacaatgctgtggtaatgccagcccatggtccagccaaaagcatggaatatgatgccgttgcaactacacattcctggaytcccaagaggaatcgttctattctcaacacaagccaaaggggaattcttgaggatgaacagatgtaccagaagtgctgcaatctattcgagaaatttttccctagcagttcatataggagaccggttggaatttctagcatggtggaggccatggtgtctagggcccggattgatgccagggtcgacttcgagtctggacggatcaagaaagaagagttctctgagatcatgaagatctgttccaccattgaagaactcagacggcaaaaataa

>H1N1_PB1_CY062256_A/California/VRDL86/2009

atggatgtcaatccgactctacttttcctaaaaattccagcgcaaaatgccataagcaccacattcccttatactggagatcctccatacagccatggaacaggaacaggatacaccatggacacagtaaacagaacacaccaatactcagaaaagggaaagtggacgacaaacacagagactggtgcaccccagctcaacccgattgatggaccactacctgaggataatgaaccaagtgggtatgcacaaacagactgtgttctagaggctatggctttccttgaagaatcccacccaggaatatttgagaattcatgccttgaaacaatggaagttgttcaacaaacaagggtagataaactaactcaaggtcgccagacttatgattggacattaaacagaaatcaaccggcagcaactgcattggccaacaccatagaagtctttagatcgaatggcctaacagctaatgagtcaggaaggctaatagatttcttaaaggatgtaatggaatcaatgaacaaagaggaaatagagataacaacccactttcaaagaaaaaggagagtaagagacaacatgaccaagaagatggtcacgcaaagaacaatagggaagaaaaaacaaagactgaataagagaggctatctaataagagcactgacattaaatacgatgaccaaagatgcagagagaggcaagttaaaaagaagggctatcgcaacacctgggatgcagattagaggtttcgtatactttgttgaaactttagctaggagcatttgcgaaaagcttgaacagtctgggctcccagtagggggcaatgaaaagaaggccaaactggcaaatgttgtgagaaagatgatgactaattcacaagacacagagatttctttcacaatcactggggacaacactaagtggaatgaaaatcaaaatcctcgaatgttcctggcgatgattacatatatcaccagaaatcaacccgagtggttcagaaacatcctgagcatggcacccataatgttctcaaacaaaatggcaagactagggaaagggtacatgttcgagagtaaaagaatgaagattcgaacacaaataccagcagaaatgctagcaagcattgacctgaagtacttcaatgaatcaacaaagaagaaaattgagaaaataaggcctcttctaatagatggcacagcatcactgagtcctgggatgatgatgggcatgttcaacatgctaagtacggtcttgggagtctcgatactgaatcttggacaaaagaaatacaccaagacaatatactggtgggatgggctccaatcatccgacgattttgctctcatagtgaatgcaccaaatcatgagggaatacaagcaggagtggacagattctacaggacctgcaagttagtgggaatcaacatgagcaaaaagaagtcctatataaataagacagggacatttgaattcacaagctttttttatcgctatggatttgtggctaattttagcatggagctacccagctttggagtgtctggagtaaatgaatcagctgacatgagtattggagtaacagtgataaagaacaacatgataaacaatgaccttggacctgcaacggcccagatggctcttcaactgttcatcaaagactacagatacacatataggtgccataggggagacacacaaattcagacgagaagatcatttgagttaaagaagctgtgggatcaaacccaatcaaaggtagggctattagtatcagatggaggaccaaacttatacaatatacggaatcttcacattcctgaagtctgcttaaaatgggagctaatggatgatgattatcggggaagactttgtaatcccctgaatccctttgtcagtcataaagagattgattctgtaaacaatgctgtggtaatgccagcccatggtccagccaaaagcatggaatatgatgccgttgcaactacacattcctggattcccaagaggaatcgttctatcctcaacacaagccaaaggggaattcttgaggatgaacagatgtaccagaagtgctgcaatctattcgagaaatttttccctagcagttcatataggagaccggttggaatttctagcatggtggaggccatggtgtctagggcccggattgatgccagggtcgacttcgagtctggacggatcgggaaggaagagttctctgagatcatgaagatctgttccaccattgaagaactcagacggcaaaaataa

>H1N1_PB1_HM157256_A/Yaroslavl/IIV-196/2009

atggatgtcaatccgactctacttttcctaaaaattccagcgcaaaatgccataagcaccacattcccttatactggagatcctccatacagccatggaacaggaacaggatacaccatggacacagtaaacagaacacaccaatactcagaaaagggaaagtggacgacaaacacagagactggtgcaccccagctcaacccgattgatggaccactacctgaggataatgaaccaagtgggtatgcacaaacagactgtgttctagaggctatggctttccttgaagaatcccacccaggaatatttgagaattcatgccttgaaacaatggaagttgttcaacaaacaagggtagataaactaactcaaggtcgccagacttatgattggacattaaacagaaatcaaccggcagcaactgcattggccaacaccatagaagtttttagatcgaatggcctaacagctaatgagtcaggaaggctaatagatttcttaaaggatgtaatggaatcaatgaacaaagaggaaatagagataacaacccactttcaaagaaaaaggagagtaagagacaacatgaccaagaagatggtcacgcaaagaacaatagggaagaaaaaacaaagactgaataagagaggctatctaataagagcactgacattaaatacaatgaccaaagatgcagagagaggcaagttaaaaagaagggctatcgcaacacctgggatgcagattagaggtttcgtatactttgttgaaactttagctaggagcatttgcgaaaagcttgaacagtctgggctcccagtagggggcaatgaaaagaaggccaaactggcaaatgttgtgagaaagatgatgactaattcacaagacacagagatttctttcacaattactggggacaacactaagtggaatgaaaatcaaaatcctcgaatgttcctggcgatgattacatatatcaccagaaatcaacccgagtggttcagaaacatcctgagcatggcacccataatgttctcaaacaaaatggcaagactagggaaagggtacatgttcgagagcaaaagaatgaagattcgaacacaaataccagcagaaatgctagcaagcattgacctgaagtacttcaatgaatcaacaaagaagaaaattgagaaaataaggcctcttctaatagatggcacagcatcactgagtcctgggatgatgatgggcatgttcaacatgctaagtacggtcttgggagtctcgatactgaatcttggacaaaagaaatacaccaagacaatatactggtgggatgggctccaatcatccgacgattttgctctcatagtgaatgcaccaaaccatgagggaatacaagcaggagtggacagattctacaggacctgcaagttagtgggaatcaacatgagcaaaaagaagtcctatataaataagacagggacatttgaattcacaagctttttttatcgctatggatttgtggctaattttagcatggagctacccagctttggagtgtctggagtaaatgaatcagctgacatgagtattggagtaacagtgataaagaacaacatgataaacaatgaccttggacctgcaacggcccagatggctcttcaattgttcatcaaagactacagatacacatataggtgccataggggagacacacaaattcagacgagaagatcatttgagttaaagaagctgtgggatcaaacccaatcaaaggtagggctattagtatcagatggaggaccaaacttatacaatatacggaatcttcacattcctgaagtctgcttaaaatgggagctaatggatgatgattatcggggaagactttgtaatcccctgaatccctttgtcagtcataaagagattgattctgtaaacaatgctgtggtaatgccagcccatggtccagccaaaagcatggaatatgatgccgttgcaactacacattcctggattcccaagaggaatcgttctattctcaacacaagccaaaggggaattcttgaggatgaacagatgtaccagaagtgctgcaatctattcgagaaatttttccctagcagttcatataggagaccggttggaatttctagcatggtggaggccatggtgtctagggcccggattgatgccagggtcgacttcgagtctggacggatcaagaaagaagagttctctgagatcatgaagatctgttccaccattgaagaactcagacggcaaaaataa

>H1N1_PB1_CY062857_A/Athens/INS158/2009

atggatgtcaatccgactctacttttcctaaaaattccagcgcaaaatgccataagcaccacattcccttatactggagatcctccatacagccatggaacaggaacaggatacaccatggacacagtaaacagaacacaccaatactcagaaaagggaaagtggacgacaaacacagagactggtgcaccccagctcaacccgattgatggaccactacctgaggataatgaaccaagtgggtatgcacaaacagactgtgttctagaggctatggctttccttgaagaatcccacccaggaatatttgagaattcatgccttgaaacaatggaagttgttcaacaaacaagggtagataaactaactcaaggtcgccagacttatgattggacattaaacagaaatcaaccggcagcaactgcattggccaacaccatagaagtctttagatcgaatggcctaacagctaatgagtcaggaaggctaatagatttcttaaaggatgtaatggaatcaatgaacaaagaggaaatagagataacaacccactttcaaagaaaaaggagagtaagagacaacatgaccaagaagatggtcacgcaaagaacaatagggaagaaaaaacaaagactgaataagagaggctatctaataagagcactgacattaaatacgatgaccaaagatgctgagagaggcaagttaaaaagaagggctatcgcaacacctgggatgcagattagaggtttcgtatactttgttgaaactttagctaggagcatttgcgaaaagcttgaacaatctgggctcccagtagggggcaatgaaaagaaggccaaactggcaaatgttgtgagaaagatgatgactaattcacaagacacagagatttctttcacaatcactggggacaacactaagtggaatgaaaatcaaaatcctcgaatgttcctggcgatgattacatatatcaccagaaatcaacccgagtggttcagaaacatcctgagcatggcacccataatgttctcaaacaaaatggcaagactagggaaagggtacatgttcgagagtaaaagaatgaagattcgaacacaaataccagcagaaatgctagcaagcattgacctgaagtacttcaatgaatcaacaaagaagaaaattgagaaaataaggcctcttctaatagatggcacagcatcactgagtcctgggatgatgatgggcatgttcaacatgctaagtacggtcttgggagtctcgatactgaatcttggacaaaagaaatacaccaagacaatatactggtgggatgggctccaatcatccgacgattttgctctcatagtgaatgcaccaaaccatgagggaatacaagcaggagtggacagattctacaggacctgcaagttagtgggaatcaacatgagcaaaaagaagtcctatataaataagacagggacatttgaattcacaagctttttttatcgctatggatttgtggctaattttagcatggagctacccagctttggagtgtctggagtaaatgaatcagctgacatgagtattggagtaacagtgataaagaacaacatgataaacaatgaccttggacctgcaacggcccagatggctcttcaattgttcatcaaagactacagatacacatataggtgccataggggagacacacaaattcagacgagaagatcatttgagttaaagaagctgtgggatcaaacccaatcaaaggtagggctattagtatcagatgggggaccaaacttatacaatatacggaatcttcacattcctgaagtctgcttaaaatgggagctaatggatgatgattatcggggaagactttgtaatcccctgaatccctttgtcagtcataaagagattgattctgtaaacaatgctgtggtaatgccagcccatggtccagccaaaagcatggaatatgatgccgttgcaactacacattcctggattcccaagaggaatcgttctattctcaacacaagccaaaggggaattcttgaggatgaacagatgtaccagaagtgctgcaatctattcgagaaatttttccctagcagttcatataggagaccggttggaatttctagcatggtggaggccatggtgtctagggcccggattgatgccagggtcgacttcgagtctggacggatcaagaaagaagagttctctgagatcatgaagatctgttccaccattgaagaactcagacggcaaaaataa

>H1N1_PB1_CY063009_A/Tallinn/INS182/2010

atggatgtcaatccgactttacttttcctaaaaattccagcgcaaaatgccataagcaccacattcccttatactggagatcctccatacagccatggaacaggaacaggatacaccatggacacagtaaacagaacacaccaatactcagaaaagggaaagtggacgacaaacacagagactggtgcaccccagctcaacccgattgatggaccactacctgaggataatgaaccaagtgggtatgcacaaacagactgtgttctagaggctatggctttccttgaagaatcccacccaggaatatttgagaattcatgccttgaaacaatggaagttgttcaacaaacaagggtagataaactaactcaaggtcgccagacttatgattggacattaaacagaaatcaaccggcagcaactgcattggccaacaccatagaagtctttagatcgaatggcctaacagctaatgagtcaggaaggctaatagatttcttaaaggatgtaatggaatcaatgaacaaagaggaaatagagataacaacccactttcaaagaaaaaggagagtaagagacaacatgaccaagaagatggtcacgcaaagaacaatagggaagaaaaaacaaagactgaataagagaggctatctaataagagcactgacattaaatacgatgaccaaagatgcagagagaggcaagttaaaaagaagggctatcgcaacacctgggatgcagattagaggtttcgtatactttgttgaaactttagctaggagcatttgcgaaaagcttgaacagtctgggctcccagtagggggcaatgaaaagaaggccaaactggcaaatgttgtgagaaagatgatgactaattcacaagacacagagatttctttcacaatcactggggataacactaagtggaatgaaaatcaaaatcctcgaatgttcctggcgatgattacatatatcaccagaaatcaacccgagtggttcagaaacatcctgagcatggcacccataatgttctcaaacaaaatggcaagactagggaaagggtacatgttcgagagtaaaagaatgaagattcgaacacaaataccagcagaaatgctagcaagcattgacctgaagtacttcaatgaatcaacaaagaagaaaattgagaaaataaggcctcttctaatagatggcacagcatcactgagtcctgggatgatgatgggcatgttcaacatgctaagtacggtcttgggagtctcgatactgaatcttggacaaaagaaatacaccaagacaatatattggtgggatgggctccaatcatccgatgattttgctctcatagtgaatgcaccaaaccatgagggaatacaagcaggagtggacagattctacaggacctgcaagttagtgggaatcaacatgagcaaaaagaagtcctatataaataagacagggacatttgaattcacaagctttttttatcgctatggatttgtggctaattttagcatggagctacccagctttggagtatctggagtaaatgaatcagctgacatgagtattggagtaacagtgataaagaacaacatgataaacaatgaccttggacctgcaacggcccagatggctcttcaattgttcatcaaagactacagatacacatataggtgccataggggggacacacaaattcagacgagaagatcatttgagttaaagaagctgtgggatcaaacccaatcaaaggtagggctattagtatcagatggaggaccaaacttatacaatatacggaatcttcacattcctgaagtttgcttaaaatgggagctaatggatgatgattatcggggaagactttgtaatcccctgaatccctttgtcagtcataaagagattgattctgtaaacaatgctgtggtaatgccagcccatggtccagccaaaagcatggaatatgatgccgtcgcaactacacattcctggattcccaagaggaatcgttctattctcaacacaagccaaaggggaattcttgaggatgaacagatgtaccagaagtgctgcaatctattcgagaaatttttccctagcagttcatataggagaccggttggaatttctagcatggtggaggccatggtgtctagggcccggattgatgccagggtcgacttcgagtctggacggatcaagaaagaagagttctctgagatcatgaagatctgttccaccattgaagaactcagacggcaaaaataa

>H1N1_PB1_CY063025_A/Madrid/INS184/2009

atggatgtcaatccgactctacttttcctaaaaattccagcgcaaaatgccataagcaccacattcccttatactggagatcctccatacagccatggaacaggaacaggatacaccatggacacagtaaacagaacacaccaatactcagaaaagggaaagtggacgacaaacacagagactggtgcaccccagctcaacccgattgatggaccactacctgaggataatgaaccaagtgggtatgcacaaacagactgtgttctagaggctatggctttccttgaagaatcccacccaggaatatttgagaattcatgccttgaaacaatggaagttgttcaacaaacaagggtagataaactaactcaaggtcgccagacttatgattggacattaaacagaaatcaaccggcagcaactgcattggccaacaccatagaagtctttagatcgaatggcctaacagctaatgagtcaggaaggctaatagatttcttaaaggatgtaatggaatcaatgaacaaagaggaaatagagataacaacccactttcaaagaaaaaggagagtaagagacaacatgaccaagaagatggtcacgcaaagaacaatagggaagaaaaaacaaagactgaataagagaggctatctaataagagcactgacattaaatacgatgaccaaagatgcagagagaggcaagttaaaaagaagggctatcgcaacacctgggatgcagattagaggtttcgtatactttgttgaaactttagctaggagcatttgcgaaaagcttgaacagtctgggctcccagtagggggcaatgaaaagaaggccaaactggcaaatgttgtgagaaagatgatgactaattcacaagacacagagatttctttcacaatcactggggacaacactaagtggaatgaaaatcaaaatcctcgaatgttcctggcgatgattacatatatcaccagaaatcaacccgagtggttcagaaacatcctgagcatggcacccataatgttctcaaacaaaatggcaagactagggaaagggtacatgttcgagagtaaaagaatgaagattcgaacacaaataccagcagaaatgctagcaagcattgacctgaagtacttcaatgaatcaacaaagaagaaaattgagaaaataaggcctcttctaatagatggcacagcatcactgagtcctgggatgatgatgggcatgttcaacatgctaagtacggtcttgggagtctcgatactgaatcttggacaaaagaaatacaccaagacaatatactggtgggatgggctccaatcatccgacgattttgctctcatagtgaatgcaccaaaccatgagggaatacaagcaggagtggacagattctacaggacctgcaagttagtgggaatcaacatgagcaaaaagaagtcctatataaataagacagggacatttgaattcacaagctttttttatcgctatgggtttgtggctaattttagcatggagctacccagctttggagtgtctggagtaaatgaatcagctgacatgagtattggagtaacagtgataaagaacaacatgataaacaatgaccttggacctgcaacggcccagatggctcttcaattgttcatcaaagactacagatacacatataggtgccataagggagacacacaaattcagacgagaagatcatttgagttaaagaagctgtgggatcaaacccaatcaaaggtagggctattagtatcagatggaggaccaaacttatacaatatacggaatcttcacattcctgaagtctgcttaaaatgggagctaatggatgatgattatcggggaagactttgtaatcccctgaatccctttgtcagtcataaagagattgattctgtaaacaatgctgtggtaatgccagcccatggtccagccaaaagcatggaatatgatgccgttgcaactacacattcctggattcccaagaggaatcgttctattctaaacacaagccaaaggggaattcttgaggatgaacagatgtaccagaagtgctgcaatctattcgagaaatttttccctagcagttcatataggagaccggttggaatttctagcatggtggaggccatggtgtctagggcccggattgatgccagggtcgacttcgagtctggacggatcaagaaagaagagttctctgagatcatgaagatctgttccaccattgaagaactcagacggcaaaaataa

>H1N1_PB1_CY063097_A/California/VRDL90/2009

atggatgtcaatccgactctacttttcctaaaaattccagcgcaaaatgccataagcaccacattcccttatactggagatcctccatacagccatggaacaggaacaggatacaccatggacacagtaaacagaacacaccaatactcagaaaagggaaagtggacgacaaacacagagactggtgcaccccagctcaacccgattgatggaccactacctgaggataatgaaccaagtgggtatgcacaaacagactgtgttctagaggctatggctttccttgaagaatcccacccaggaatatttgagaattcatgccttgaaacaatggaagttgttcaacaaacaagggtagataaactaactcaaggtcgccagacttatgattggacattaaacagaaatcaaccggcagcaactgcattggccaacaccatagaagtctttagatcgaatggcctaacagctaatgagtcaggaaggctaatagatttcttaaaggatgtaatggaatcaatgaacaaagaggaaatagagataacaacccactttcaaagaaaaaggagagtaagagacaacatgaccaagaagatggtcacgcaaagaacaatagggaagaaaaaacaaagactgaataagagaggctatctaataagagcactgacattaaatacgatgaccaaagatgcagagagaggcaagttaaaaagaagggctatcgcaacacctgggatgcagattagaggtttcgtatactttgttgaaactttagctaggagcatttgcgaaaagcttgaacagtctgggctcccagtagggggcaatgaaaagaaggccaaactggcaaatgttgtgagaaagatgatgactaattcacaagacacagagatttctttcacaatcactggggacaacactaagtggaatgaaaatcaaaatcctcgaatgttcctggcgatgattacatatatcaccagaaatcaacccgagtggttcagaaacaccctgagcatggcacccataatgttctcaaacaaaatggcaagactagggaaagggtacatgttcgagagtaaaagaatgaagattcgaacacaaataccagcagaaatgctagcaagcattgacctgaagtacttcaatgaatcaacaaagaagaaaattgagaaaataaggcctcttctaatagatggcacagcatcactgagtcctgggatgatgatgggcatgttcaacatgctaagtacggtcttgggagtctcgatactgaatcttggacaaaagaaatacaccaagacaatatattggtgggatgggctccaatcatccgacgattttgctctcatagtgaatgcaccaaaccatgagggaatacaagcaggagtggacagattctacaggacctgcaagttagtgggaatcaacatgagcaaaaagaagtcctatataaataagacagggacatttgaattcacaagctttttttatcgctatggatttgtggctaattttagcatggagctacccagctttggagtgtctggagtaaatgaatcagctgacatgagtattggagtaacagtgataaagaacaacatgataaacaatgaccttggacctgcaacggcccagatggctcttcaattgttcatcaaagactacagatacacatataggtgccataggggggacacacaaattcagacgagaagatcatttgagttaaagaagctgtgggatcaaacccaatcaaaggtagggctattagtatcagatggaggaccaaacttatacaatatacggaatcttcacattcctgaagtctgcttaaaatgggagctaatggatgatgattatcggggaagactttgtaatcccctgaatccctttgtcagtcataaagagattgattctgtaaacaatgctgtggtaatgccagcccatggtccagccaaaagcatggaatatgatgccgttgcaactacacattcctggattcccaagaggaatcgttctattctcaacacaagccaaaggggaattcttgaggatgaacagatgtaccagaagtgctgcaatctattcgagaaatttttccctagcagttcatataggagaccggttggaatttctagcatggtggaggccatggtgtctagggcccggattgatgccagggtcgacttcgagtctggacggatcaagaaagaagagttctctgagatcatgaagatctgttccaccattgaagaactcagacggcaaaaataa

>H1N1_PB1_CY063113_A/California/VRDL92/2009

atggatgtcaatccgactctacttttcctaaaaattccagcgcaaaatgccataagcaccacattcccttatactggagatcctccatacagccatggaacaggaacaggatacaccatggacacagtaaacagaacacaccaatactcagaaaagggaaagtggacgacaaacacagagactggtgcaccccagctcaacccgattgatggaccactacctgaggataatgaaccaagtgggtatgcacaaacagactgtgttctagaggctatggctttccttgaagaatcccacccaggaatatttgagaattcatgccttgaaacaatggaagttgttcaacaaacaagggtagataaactaactcaaggtcgccagacttatgattggacattaaacagaaatcaaccggcagcaactgcattggccaacaccatagaagtctttagatcgaatggcctaacagctaatgagtcaggaaggctaatagatttcttaaaggatgtaatggaatcaatgaacaaagaggaaatagagataacaacccactttcaaagaaaaaggagagtaagagacaacatgaccaagaagatggtcacgcaaagaacaatagggaagaaaaaacaaagactgaataagagaggctatctaataagagcattgacattaaatacgatgaccaaagatgcagagagaggcaagttaaaaagaagggctatcgcaacacctgggatgcagattagaggtttcgtatactttgttgaaactttagctaggagcatttgcgaaaagcttgaacagtctgggctcccagtagggggcaatgaaaagaaggccaaactggcaaatgttgtgagaaagatgatgactaattcacaagacacagagatttctttcacaatcactggggacaacactaagtggaatgaaaatcaaaatcctcgaatgttcctggcgatgattacatatatcaccagaaaacaacccgagtggttcagaaacatcctgagcatggcacccataatgttctcaaacaaaatggcaagactagggaaagggtacatgttcgagagtaaaagaatgaagattcgaacacaaataccagcagaaatgctagcaagcattgacctgaagtacttcaatgaatcaacaaagaagaaaattgagaaaataaggcctcttctaatagatggcacagcatcactgagtcctgggatgatgatgggcatgttcaacatgctaagtacggtcttgggagtctcgatactgaatcttggacaaaagaaatacaccaagacaatatactggtgggatgggctccaatcatccgacgattttgctctcatagtgaatgcaccaaaccatgagggaatacaagcaggagtggacagattctacaggatctgcaagttagtgggaatcaacatgagcaaaaagaagtcctatataaataagacagggacatttgaattcacaagctttttttatcgctatggatttgtggctaattttagcatggagctacccagctttggagtgtctggagtaaatgaatcagctgacatgagtattggagtaacagtgataaagaacaacatgataaacaatgaccttggacctgcaacggcccagatggctcttcaattgttcatcaaagactacagatacacatataggtgccataggggagacacacaaattcagacgagaagatcatttgagttaaagaagctgtgggatcaaacccaatcaaagggagggctattagtatcagatggaggaccaaacttatacaatatacggaatcttcacattcctgaagtctgcttaaaatgggagctaatggatgatgattatcggggaagactttgtaatcccctgaatccctttgtcagtcataaagagattgattctgtaaacaatgctgtggtaatgccagcccatggtccagtcaaaagcatggaatatgatgccgttgcaactacacattcctggattcccaagaggaatcgttctattctcaacacaagccaaaggggaattcttgaggatgaacagatgtaccagaagtgctgcaatctattcgagaaatttttccctagcagttcatataggagaccggttggaatttctagcatggtggaggccatggtgtctagggcccggattgatgccagggtcgacttcgagtctggacggatcaagaaagaagagttctctgagatcatgaagatctgctccaccattgaagaactcagacggcaaaaataa

>H1N1_PB1_CY063177_A/California/VRDL102/2009

atggatgtcaatccgactctacttttcctaaaaattccagcgcaaaatgccataagcaccacattcccttatactggagatcctccatacagccatggaacaggaacaggatacaccatggacacagtaaacagaacacaccaatactcagaaaagggaaagtggacgacaaacacagagactggtgcaccccagctcaacccgattgatggaccactacctgaggataatgaaccaagtgggtatgcacaaacagactgtgttctagaggctatggctttccttgaagaatcccacccaggaatatttgagaattcatgccttgaaacaatggaagttgttcaacaaacaagggtagataaactaactcaaggtcgccagacttatgattggacattaaacagaaatcaaccggcagcaactgcattggccaacaccatagaagtctttagatcgaatggcctaacagctaatgagtcaggaaggctaatagatttcttaaaggatgtaatggaatcaatgaacaaagaggaaatagagataacaacccactttcaaagaaaaaggagagtaagagacaacatgaccaagaagatggtcacgcaaagaacaatagggaagaaaaaacaaagactgaataagagaggctatctaataagagcactgacattaaatacgatgaccaaagatgcagagagaggcaagttaaaaagaagggctatcgcaacacctgggatgcagattagaggtttcgtatactttgttgaaactttagctaggagcatttgcgaaaagcttgaacagtctgggctcccagtagggggcaatgaaaagaaggccaaactggcaaatgttgtgagaaagatgatgactaattcacaagacacagagatttctttcacaatcactggggacaacactaagtggaatgaaaatcaaaatcctcgaatgttcctggcgatgattacatatatcaccagaaatcaacccgagtggttcagaaacatcctgagcatggcacccataatgttctcaaacaaaatggcaagactagggaaagggtacatgttcgagagtaaaagaatgaagattcgaacacaaataccagcagaaatgctagcaagcattgacctgaagtacttcaatgaatcaacaaagaagaaaattgagaaaataaggcctcttctaatagatggcacagcatcactgagtcctgggatgatgatgggcatgttcaacatgctaagtacggtcttgggagtctcaatactgaatcttggacaaaagaaatacaccaagacaatatactggtgggatgggctccaatcatccgacgattttgctctcatagtgaatgcaccaaaccatgagggaatacaagcaggagtggacagattctacaggacctgcaagttagtgggaatcaacatgagcaaaaagaagtcctatataaataagacagggacatttgaattcacaagctttttttatcgctatggatttgtggctaattttagcatggagctacccagttttggagtgtctggagtaaatgaatcagctgacatgagtattggagtaacagtgataaagaacaacatgataaacaatgaccttggacctgcaacggcccagatggctcttcaattgttcatcaaagactacagatacacatataggtgccataggggagacacacaaattcagacgagaagatcatttgagttaaagaagctgtgggatcaaacccaatcaaaggtagggctattagtatcagatggaggaccaaacttatacaatatacggaatcttcacattcctgaagtctgcttaaaatgggagctaatggatgatgattatcggggaagactttgtaatcccctgaatccctttgtcagtcataaagagattgattctgtaaacaatgctgtggtaatgccagcccatggtccagccaaaagcgtggaatatgatgccgttgcaactacacattcctggattcccaagaggaatcgttctattctcaacacaagccaaaggggaattcttgaggatgaacagatgtaccagaagtgctgcaatctattcgagaaatttttccctagcagttcatataggagaccggttggaatttctagcatggtggaggccatggtgtctagggcccggattgatgccagggtcgacttcgagtctggacggatcacgaaagaagagttctctgagatcatgaagatctgttccaccattgaagaactcagacggcaaaaataa

>H1N1_PB1_CY063217_A/California/VRDL14/2010

atggatgtcaatccgactctacttttcctaaaaattccagcgcaaaatgccataagcaccacattcccttatactggagatcctccatacagccatggaacaggaacaggatacaccatggacacagtaaacagaacacaccaatactcagaaaagggaaagtggacgacaaacacagagactggtgcaccccagctcaacccgattgatggaccactacctgaggataatgaaccaagtgggtatgcacaaacagactgtgttctagaggctatggctttccttgaagaatcccacccaggaatatttgagaattcatgccttgaaacaatggaagttgttcaacaaacaagggtagataaactaactcaaggtcgccagacttatgattggacattaaacagaaatcaaccggcagcaactgcattggccaacaccatagaagtctttagatcgaatggcctgacagctaatgagtcaggaaggctaatagatttcttaaaggatgtaatggaatcaatgaacaaagaggaaatagagataacaacccactttcaaagaaaaaggagagtaagggacaacatgaccaagaagatggtcacgcaaagaacaatagggaagaaaaaacaaagactgaataagagaggctatctaataagagcactgacattaaatacgatgaccaaagatgcagagagaggcaagttaaaaagaagggctatcgcaacacctgggatgcagattagaggtttcgtatactttgttgaaactttagctaggagcatttgcgaaaagcttgaacagtctgggctcccagtagggggcaatgaaaagaaggccaaactggcaaatgttgtgagaaagatgatgactaattcacaagacacagagatttctttcacaatcactggggacaacactaagtggaatgaaaatcaaaatcctcgaatgttcctggcgatgattacatatatcaccagaaatcaacccgagtggttcagaaacatcctgagcatggcacccataatgttctcaaacaaaatggcaagactagggaaagggtacatgttcgagagtaaaagaatgaagattcgaacgcaaataccagcagaaatgctagcaagcattgacctgaagtacttcaatgaatcaacaaagaagaaaattgagaaaataaggcctcttctaatagatggcacagcatcactgagtcctgggatgatgatgggcatgttcaacatgctaagtacggtcttgggagtctcgatactgaatcttggacaaaagaaatacaccaagacaatatactggtgggatgggctccaatcatccgacgattttgctctcatagtgaatgcaccaaaccatgagggaatacaagcaggagtggacagattctacaggacctgcaagttagtgggaatcaacatgagcaaaaagaagtcctatataaataagacagggacatttgaattcacaagctttttttatcgctatggatttgtggctaattttagcatggagctacccagctttggagtgtctggagtaaatgaatcagctgacatgagtattggagtaacagtgataaagaacaacatgataaacaatgaccttggacctgcaacggcccagatggctcttcaactgttcatcaaagactacagatacacatataggtgccataggggagacacacaaattcagacgagaagatcatttgagttaaagaagctgtgggatcaaacccaatcaaaggtagggctattagtatcagatggaggaccaaacttatacaatatacggaatcttcacattcctgaagtctgcttaaaatgggagctaatggatgatgattatcggggaagactttgtaatcccctgaatccctttgtcagtcataaagagattgattctgtaaacaatgctgtggtaatgccagcccatggtccagccaaaagcatggaatatgatgccgttgcaactacacattcctggattcccaagaggaatcgttctattctcaacacaagccaaaggggaattcttgaggatgaacagatgtaccagaagtgctgcaatctattcgagaaatttttccctagcagttcatataggagaccggttggaatttctagcatggtggaggccatggtgtctagggcccggattgatgccagggtcgacttcgagtctggacggatcgggaaggaagagttctctgagatcatgaagatctgttccaccattgaagaactcagacggcaaaaataa

>H1N1_PB1_CY063297_A/Wisconsin/629-D00292/2009

atggatgtcaatccgactctacttttcctaaaaattccagcgcaaaatgccataagcaccacattcccttatactggagatcctccatacagccatggaacaggaacaggatacaccatggacacagtaaacagaacacaccaatactcagaaaagggaaagtggacgacaaacacagagactggtgcaccccagctcaacccgattgatggaccactacctgaggataatgaaccaagtgggtatgcacaaacagactgtgttctagaggctatggctttccttgaagaatcccacccaggaatatttgagaattcatgccttgaaacaatggaagttgttcaacaaacaagggtagataaactaactcaaggtcgccagacttatgattggacattaaacagaaatcaaccggcagcaactgcattggccaacaccatagaagtctttagatcgaatggcctaacagctaatgagtcaggaaggctaatagatttcttaaaggatgtaatggaatcaatgaacaaagaggaaatagagataacaacccactttcaaagaaaaaggagagtaagagacaacatgaccaagaagatggtcacgcaaagaacaatagggaagaaaaaacaaagactgaataagagaggctatctaataagagcactgacattaaatacaatgaccaaagatgcagagagaggcaagttaaaaagaagggctatcgcaacacctgggatgcagattagaggtttcgtatactttgttgaaactttagctaggagcatttgcgaaaagcttgaacagtctgggctcccagtagggggcaatgaaaagaaggccaaactggcaaatgttgtgagaaagatgatgactaattcacaagacacagaaatttctttcacaatcactggggacaacactaagtggaatgaaaatcaaaatcctcgaatgttcctggcgatgattacatatatcaccagaaatcaacccgagtggttcagaaacatcctgagcatggcacccataatgttctcaaacaaaatggcaagactagggaaagggtacatgttcgagagtaaaagaatgaagattcgaacacaaataccagcagaaatgctagcaagcattgacctgaagtacttcaatgaatcaacaaagaagaaaattgagaaaataaggcctcttctaatagatggcacggcatcactgagtcctgggatgatgatgggcatgttcaacatgctaagtacggtcttgggagtctcgatactgaatcttggacaaaagaaatacaccaagacaatatactggtgggatgggctccaatcatccgacgattttgctctcatagtgaatgcaccaaaccatgagggaatacaagcaggagtggacagattctacaggacctgcaagttagtgggaatcaacatgagcaaaaagaagtcctatataaataagacagggacatttgaattcacaagctttttttatcgctatggatttgtggctaattttagcatggagctacccagctttggagtgtctggagtaaatgaatcagctgacatgagtattggagtaacagtgataaagaacaacatgataaacaatgaccttggacctgcaacggcccagatggctcttcaattgttcatcaaagactacagatacacatataggtgccataggggagacacacaaattcagacgagaagatcatttgagttaaagaagctgtgggatcaaacccaatcaaaggtagggctattagtatcagatggaggaccaaacttatacaatatacggaatcttcacattcctgaagtctgcttaaaatgggagctaatggatgatgattatcggggaagactttgtaatcccctgaatccctttgtcagtcataaagagattgattctgtaaacaatgctgtggtaatgccagcccatggtccagccaaaagcatggaatatgatgccgttgcaactacacattcctggattcccaagaggaatcgttctattctcaacacaagccaaaggggaattcttgaggatgaacagatgtaccagaagtgctgcaatctattcgagaaatttttccctagcagttcatataggagaccggttggaatttctagcatggtggaggccatggtgtctagggcccggattgatgccagggtcgacttcgagtctggacggatcaagaaagaagagttctctgagatcatgaagatctgttccaccattgaagaactcagacggcaaaaataa

>H1N1_PB1_CY063540_A/Boston/148/2009

atggatgtcaatccgactctacttttcctaaaaattccagcgcaaaatgccataagcaccacattcccttatactggagatcctccatacagccatggaacaggaacaggatacaccatggacacagtaaacagaacacaccaatactcagaaaagggaaagtggacgacaaacacagagactggtgcaccccagctcaacccgattgatggaccactacctgaggataatgaaccaagtgggtatgcacaaacagactgtgttctagaggctatggctttccttgaagaatcccacccaggaatatttgagaattcatgccttgaaacaatggaagttgttcaacaaacaagggtagataaactaactcaaggtcgccagacttatgattggacattaaacagaaatcaaccggcagcaactgcattggccaacaccatagaagtctttagatcgaatggcctaacagctaatgagtcaggaaggctaatagatttcttaaaggatgtaatggaatcaatgaacaaagaggaaatagagataacaacccactttcaaagaaaaaggagagtaagagacaacatgaccaagaagatggtcacgcaaagaacaatagggaagaaaaaacaaagactgaataagagaggctatctaataagagcactgacattaaatacgatgaccaaagatgcagagagaggcaagttaaaaagaagggctatcgcaacacctgggatgcagattagaggtttcgtatactttgttgaaactttagctaggagcatttgcgaaaagcttgaacagtctgggctcccagtagggggcaatgaaaagaaggccaaactggcaaatgttgtgagaaagatgatgactaattcacaagacacagagatttctttcacaatcactggggacaacactaagtggaatgaaaatcaaaatcctcgaatgttcctggcgatgattacatatatcaccagaaatcaacccgagtggttcagaaacatcctgagcatggcacccataatgttctcaaacaaaatggcaagactagggaaagggtacatgttcgagagtaaaagaatgaagattcgaacacaaataccggcagaaatgctagcaagcattgacctgaagtacttcaatgaatcaacaaagaagaaaattgagaaaataaggcctcttctaatagatggcacagcatcactgagtcctgggatgatgatgggcatgttcaacatgctaagtacggtcttgggagtctcgatactgaatcttggacaaaagaaatacaccaagacaatatactggtgggatgggctccaatcatccgacgattttgctctcatagtgaatgcaccaaaccatgagggaatacaagcaggagtggacagattctacaggacctgcaagttagtgggaatcaacatgagcaaaaagaagtcctatataaataagacagggacatttgaattcacaagctttttttatcgctatggatttgtggctaattttagcatggagctacccagctttggagtgtctggagtaaatgaatcagctgacatgagtattggagtaacagtgataaagaacaacatgataaacaatgaccttggacctgcaacggcccagatggctcttcaattgttcatcaaagactacagatacacatataggtgccataggggagacacacaaattcagacgagaagatcatttgagttaaagaagctgtgggatcaaacccaatcaaaggtagggctattagtatcagatggaggaccaaacttatacaatatacggaatcttcacattcctgaagtctgcttaaaatgggagctaatggatgatgattatcggggaagactttgtaatcccctgaatccctttgtcagtcataaagagattgattctgtaaacaatgctgtggtaatgccagcccatggtccagccaaaagcatggaatatgatgccgttgcaactacacattcctggattcccaagaggaatcgttctattctcaacacaagccaaaggggaattcttgaggatgaacagatgtaccagaagtgctgcaatctattcgagaaatttttccctagcagttcatataggagaccggttggaatttctagcatggtggaggccatggtgtctagggcccggattgatgccagggtcgacttcgagtctggacggatcaagaaagaagagttctctgagatcatgaagatctgttccaccattgaagaactcagacggcaaaaataa

>H1N1_PB1_CY064474_A/Boston/103/2009

atggatgtcaatccgactctacttttcctaaaaattccagcgcaaaatgccataagcaccacattcccttatactggagatcctccatacagccatggaacaggaacaggatacaccatggacacagtaaacagaacacaccaatactcagaaaagggaaagtggacgacaaacacagagactggtgcaccccagctcaacccgattgatggaccactacctgaggataatgaaccaagtgggtatgcacaaacagactgtgttctagaggctatggctttccttgaagaatcccacccaggaatatttgagaattcatgccttgaaacaatggaagttgttcaacaaacaagggtagataaactaactcaaggtcgccagacttatgattggacattaaacagaaatcaaccggcagcaactgcattggccaacaccatagaagtctttagatcgaatggcctaacagctaatgagtcaggaaggctaatagatttcttaaaggatgtaatggaatcaatgaacaaagaggaaatagagataacaacccactttcaaagaaaaaggagagtaagagacaacatgaccaagaagatggtcacacaaagaacaatagggaagaaaaaacaaagactgaataagagaggctatctaataagagcactgacattaaatacgatgaccaaagatgcagagagaggcaagttaaaaagaagggctatcgcaacacctgggatgcagattagaggtttcgtatactttgttgaaactttagctaggagcatttgcgaaaagcttgaacagtctgggctcccagtagggggcaatgaaaagaaggccaaactggcaaatgttgtgagaaagatgatgactaattcacaagacacagagatttctttcacaatcactggggacaacactaagtggaatgaaaatcaaaatcctcgaatgttcctggcgatgattacatatatcaccagaaatcaacccgagtggttcagaaacatcctgagcatggcacccataatgttctcaaacaaaatggcaagactagggaaagggtacatgttcgagagtaaaagaatgaagattcgaacacaaataccagcagaaatgctagcaagcattgacctgaagtacttcaatgaatcaacaaagaagaaaattgagaaaataaggcctcttctaatagatggcacagcatcactgagtcctgggatgatgatgggcatgttcaacatgctaagtacggtcttgggagtctcgatactgaatcttggacaaaagaaatacaccaagacaatatactggtgggatgggctccaatcatccgacgattttgctctcatagtgaatgcaccaaaccatgagggaatacaagcaggagtggacagattctacaggacctgcaagttagtgggaatcaacatgagcaaaaagaagtcctatataaataagacagggacatttgaattcacaagctttttttatcgctatggatttgtggctaattttagcatggagctacccagctttggagtgtctggagtaaatgaatcagctgacatgagtattggagtaacagtgataaagaacaacatgataaacaatgaccttggacctgcaacggcccagatggctcttcaattgttcatcaaagactacagatacacatataggtgccataggggagacacacaaattcagacgagaagatcatttgagttaaagaagctgtgggatcaaacccaatcaaaggtagggctattagtatcagatggaggaccaaacttatacaatatacggaatcttcacattcctgaagtctgcttaaaatgggagctaatggatgatgattatcggggaagactttgcaatcccctgaatccctttgtcagtcataaagggattgattctgtaaacaatgctgtggtaatgccagcccatggtccagccaaaagcatggaatatgatgccgttgcaactacacattcctggattcccaagaggaatcgttctattctcaacacaagccaaaggggaattcttgaggatgaacagatgtaccagaagtgttgcaatctattcgagaaatttttccctagcagttcatataggagaccggttggaatttctagcatggtggaggccatggtgtctagggcccggattgatgccagggtcgacttcgagtctggacggatcaagaaagaagagttctctgagatcatgaagatctgttccaccattgaagaactcagacggcaaaaataa

>H1N1_PB1_CY064538_A/Boston/120/2009

atggatgtcaatccgactctacttttcctaaaaattccagcgcaaaatgccataagcaccacattcccttatactggagatcctccatacagccatggaacaggaacaggatacaccatggacacagtaaacagaacacaccaatactcagaaaagggaaagtggacgacaaacacagagactggtgcaccccagctcaacccgattgatggaccactacctgaggataatgaaccaagtgggtatgcacaaacagactgtgttctagaggctatggctttccttgaagaatcccacccaggaatatttgagaattcatgccttgaaacaatggaagttgttcaacaaacaagggtagataaactaactcaaggtcgccagacttatgattggacattaaacagaaatcaaccggcagcaactgcattggccaacaccatagaagtctttagatcgaatggcctaacagctaatgagtcaggaaggctaatagatttcttaaaggatgtaatggaatcaatgaacaaagaggaaatagagataacaacccactttcaaagaaaaaggagagtaagagacaacatgaccaagaagatggtcacacaaagaacaatagggaagaaaaaacaaagactaaataagagaggctatctaataagagcactgacattaaatacgatgaccaaagatgcagagagaggcaagttaaaaagaagggctatcgcaacacctgggatgcagattagaggtttcgtatactttgttgaaactttagctaggagcatttgcgaaaagcttgaacagtctgggctcccagtagggggcaatgaaaagaaggccaaactggcaaatgttgtgagaaagatgatgactaattcacaagacacagagatttctttcacaatcactggggacaacactaagtggaatgaaaatcaaaatcctcgaatgttcctggcgatgattacatatatcaccagaaatcaacccgagtggttcagaaacatcctgagcatggcacccataatgttctcaaacaaaatggcaagactagggaaagggtacatgttcgagagtaaaagaatgaagattcgaacacaaataccagcagaaatgctagcaagcattgacctgaagtacttcaatgaatcaacaaagaagaaaattgagaaaataaggcctcttctaatagatggcacagcatcactgagtcctgggatgatgatgggcatgttcaacatgctaagtacggtcttgggagtctcgatactgaatcttggacaaaagaaatacaccaagacaatatactggtgggatgggctccaatcatccgacgattttgctctcatagtgaatgcaccaaaccatgagggaatacaagcaggagtggacagattctacaggacctgcaagttagtgggaatcaacatgagcaaaaagaagtcctatataaataagacagggacatttgaattcacaagctttttttatcgctatggatttgtggctaattttagcatggagctacccagctttggagtgtctggagtaaatgaatcagctgacatgagtattggagtaacagtgataaagaacaacatgataaacaatgaccttggacctgcaacggcccagatggctcttcaattgttcatcaaagactacagatacacatataggtgccataggggagacacacaaattcagacgagaagatcatttgagttaaagaagctgtgggatcaaacccaatcaaaggtagggctattagtatcagatggaggaccaaacttatacaatatacggaatcttcacattcctgaagtctgcttaaaatgggagctaatggatgatgattatcggggaagactttgtaatcccctgaatccctttgtcagtcataaagagattgattctgtaaacaatgctgtggtaatgccagcccatggtccagccaaaagcatggaatatgatgccgttgcaactacacattcctggattcccaagaggaatcgttctattctcaacacaagccaaaggggaattcttgaggatgaacagatgtaccagaagtgttgcaatctattcgagaaatttttccctagcagttcatataggagaccggttggaatttctagcatggtggaggccatggtgtctagggcccggattgatgccagggtcgacttcgagtctggacggatcaagaaagaagagttctctgagatcatgaagatctgttccaccattgaagaactcagacggcaaaaataa

>H1N1_PB1_CY064642_A/Boston/134/2009

atggatgtcaatccgactctacttttcctaaaaattccagcgcaaaatgccataagcaccacattcccttatactggagatcctccatacagccatggaacaggaacaggatacaccatggacacagtaaacagaacacaccaatactcagaaaagggaaagtggacgacaaacacagagactggtgcaccccagctcaacccgattgatggaccactacctgaggataatgaaccaagtgggtatgcacaaacagactgtgttctagaggctatggctttccttgaagaatcccacccaggaatatttgagaattcatgccttgaaacaatggaagttgttcaacaaacaagggtagataaactaactcaaggtcgccagacttatgattggacattaaacagaaatcaaccggcagcaactgcattggccaacaccatagaagtctttagatcgaatggcctaacagctaatgagtcaggaaggctaatagatttcttaaaggatgtaatggaatcaatgaacaaagaggaaatagagataacaacccactttcaaagaaaaaggagagtaagagacaacatgaccaagaagatggtcacgcaaagaacaatagggaagaaaaaacaaagactgaataagagaggctatctaataagagcactgacattaaatacgatgaccaaagatgcagagagaggcaagttaaaaagaagggctatcgcaacacctgggatgcagattagaggtttcgtatactttgttgaaactttagctaggagcatttgcgaaaagcttgaacagtctgggctcccagtagggggcaatgaaaagaaggccaaactggcaaatgttgtgagaaagatgatgactaattcacaagacacagagatttctttcacaatcactggggacaacactaagtggaatgaaaatcaaaatcctcgaatgttcctggcgatgattacatatatcaccagaaatcaacccgagtggttcagaaacatcctgagcatggcacccataatgttctcaaacaaaatggcaagactagggaaagggtacatgttcgagagtaaaagaatgaagattcgaacacaaataccagcagaaatgctagcaagcattgacctgaagtacttcaatgaatcaacaaagaagaaaattgagaaaataaggcctcttctaatagatggcacagcatcactgagtcctgggatgatgatgggcatgttcaacatgctaagtacggtcttgggagtctcgatactgaatcttggacaaaagaaatacaccaagacaatatactggtgggatgggctccaatcatccgacgattttgctctcatagtgaatgcaccaaaccatgagggaatacaagcaggagtggacagattctacaggacctgcaagttagtgggaatcaacatgagcaaaaagaagtcctatataaataagacagggacatttgaattcacaagctttttttatcgctatggatttgtggctaattttagcatggagctacccagctttggagtgtctggagtaaatgaatcagctgacatgagtattggagtaacagtgataaagaacaacatgataaacaatgaccttggacctgcaacggcccagatggctcttcaattgttcatcaaagactacagatacacatataggtgccataggggagacacacaaattcagacgagaagatcatttgagttaaagaagctgtgggatcaaacccaatcaaaggtagggctattagtatcagatggaggaccaaacttatacaatatacggaatcttcacattcctgaagtctgcttaaaatgggagctaatggatgatgattatcggggaagactttgtaatcccctgaatccctttgtcagtcataaagagattgattctgtaaacaatgctgtagtaatgccagcccatggtccagccaaaagcatggaatatgatgccgttgcaactacacattcctggattcccaagaggaatcgttctattctcaacacaagccaaaggggaattcttgaggatgaacagatgtaccagaagtgctgcaatctattcgagaaatttttccctagcagttcatataggagaccggttggaatttctagcatggtggaggccatggtgtctagggcccggattgatgccagggtcgacttcgagtctggacggatcaagaaagaagagttctctgagatcatgaagatctgttccaccattgaagaactcagacggcaaaaataa

>H1N1_PB1_CY064778_A/Berlin/INS170/2009

atggatgtcaatccgactctacttttcctaaaaattccagcgcaaaatgccataagcaccacattcccttatactggagatcctccatacagccatggaacaggaacaggatacaccatggacacagtaaacagaacacaccaatactcagaaaagggaaagtggacgacaaacacagagactggtgcaccccagctcaacccgattgatggaccactacctgaggataatgaaccaagtgggtatgcacaaacagactgtgttctagaggctatggctttccttgaagaatcccacccaggaatatttgagaattcatgccttgaaacaatggaagttgttcaacaaacaagggtagataaactaactcaaggtcgccagacttatgattggacattaaacagaaatcaaccggcagcaactgcattggccaacaccatagaagtctttagatcgaatggcctaacagctaatgagtcaggaaggctaatagatttcttaaaggatgtaatggaatcaatgaacaaagaggaaatagagataacaacccactttcaaagaaaaaggagagtaagagacaacatgaccaagaagatggtcacgcaaagaacaatagggaagaaaaaacaaagactgaataagagaggctatctaataagagcactgacattaaatacgatgaccaaagatgcagagagaggcaagttaaaaagaagggctatcgcaacacctgggatgcagattagaggtttcgtatactttgttgaaactttagctaggagcatttgcgaaaagcttgaacagtctgggctcccagtagggggcaatgaaaagaaggccaaactggcaaatgttgtgagaaagatgatgactaattcacaagacacagagatttctttcacaatcactggggacaacactaagtggaatgaaaatcaaaatcctcgaatgttcctggcgatgattacatatatcaccagaaatcaacccgagtggttcagaaacatcctgagcatggcacccataatgttctcaaacaaaatggcaagactagggaaagggtacatgttcgaaagtaaaagaatgaagattcgaacacaaataccagcagaaatgctagcaagcattgacctgaagtacttcaatgaatcaacaaagaagaaaattgagaaaataaggcctcttctaatagatggcacagcatcactgagtcctgggatgatgatgggcatgttcaacatgctaagtacggtcttgggagtctcgatactgaatcttggacaaaagaaatacaccaagacaatatactggtgggatgggctccaatcatccgacgattttgctctcatagtgaatgcaccaaaccatgagggaatacaagcaggagtgaacagattctacaggacctgcaagttagtgggaatcaacatgagcaaaaagaagtcctatataaataagacagggacatttgaattcacaagctttttttatcgctatgggtttgtggctaattttagcatggagctacccagctttggagtgtctggagtaaatgaatcagctgacatgagtattggagtaacagtgataaagaacaacatgataaacaatgaccttggacctgcaacggcccagatggctcttcaattgttcatcaaagactacagatacacatataggtgccataagggagacacacaaattcagacgagaagatcatttgagttaaagaagctgtgggatcaaacccaatcaaaggtagggctattagtatcagatggaggaccaaatttatacaatatacggaatcttcacattcctgaagtctgcttaaaatgggagctaatggatgatgattatcggggaagactttgtaatcccctgaatccctttgtcagtcataaagagattgattctgtaaacaatgctgtggtaatgccagcccatggtccagccaaaagcatggaatatgatgccgttgcaactacacattcctggattcccaagaggaatcgttctattctcaacacaagccaaaggggaattcttgaggatgaacagatgtaccagaagtgctgcaatctattcgagaaatttttccctagcagttcatataggagaccggttggaatttctagcatggtggaggccatggtgtctagggcccggattgatgccagggtcgacttcgagtctggacggatcaagaaagaagagttctctgagatcatgaagatctgttccaccattgaagaactcagacggcaaaaataa

>H1N1_PB1_CY065025_A/New York/3834/2010

atggatgtcaatccgactctacttttcctaaaaattccagcgcaaaatgccataagcaccacattcccttatactggagatcctccatacagccatggaacaggaacaggatacaccatggacacagtaaacagaacacaccaatactcagaaaagggaaagtggacgacaaacacagagactggtgcaccccagctcaacccgattgatggaccactacctgaggataatgaaccaagtgggtatgcacaaacagactgtgttctagaggctatggctttccttgaagaatcccacccaggaatatttgagaattcatgccttgaaacactggaagttgttcaacaaacaagggtagataaactgactcaaggtcgccagacttatgattggacattaaacagaaatcaaccggcagcaactgcattggccaacaccatagaagtctttagatcgaatggcctaacagctaatgagtcaggaaggctaatagatttcttaaaggatgtaatggaatcaatgaacaaagaggaaatagagataacaacccactttcaaagaaaaaggagagtaagagacaacatgaccaagaagatggtcacgcaaagaacaatagggaagaaaaaacaaagactgaataagagaggctatctaataagagcactgacattaaacacgatgaccaaagatgcagagagaggcaagttaaaaagaagggctatcgcaacacctgggatgcagattagaggtttcgtatactttgttgaaactttagctaggagcatttgcgaaaagcttgaacagtctgggctcccagtagggggcaatgaaaagaaggccaaattggcaaatgttgtgagaaagatgatgactaattcacaagacacagagatttctttcacaatcactggggacaacactaagtggaatgaaaatcaaaatcctcgaatgttcctggcgatgattacatatatcaccagaaatcaacccgagtggttcagaaacatcctgagcatggcacccataatgttctcaaacaaaatggcaagactagggaaagggtacatgttcgagagtaaaagaatgaagattcgaacacaaataccagcagaaatgctagcaagcattgacctgaagtacttcaatgaatcaacaaagaagaaaattgagaaaataaggcctcttctaatagatggcacagcatcactgagtcctgggatgatgatgggcatgttcaacatgctaagtacggtcttgggagtctcgatactgaatcttggacaaaagaaatacaccaagacaatatactggtgggatgggctccaatcatccgacgattttgctctcatagtgaatgcaccaaaccatgagggaatacaagcaggagtggacagattctacaggacctgcaagttagtgggaatcaacatgagcaaaaagaagtcctatataaataagacagggacatttgaattcacaagctttttttatcgctatggatttgtggctaattttagcatggagctacccagctttggagtgtctggagtaaatgaatcagctgacatgagtattggagtaacagtgataaagaacaacatgataaacaatgaccttggacctgcaacggcccagatggctcttcaattgttcatcaaagactacagatacacatataggtgccataggggagacacacaaattcagacgagaagatcatttgagttaaagaagctgtgggatcaaacccaatcaaaggtagggctattagtatcagatggaggaccaaacttatacaatatacggaatcttcacattcctgaagtctgcttaaaatgggagctaatggatgatgattatcggggaagactttgtaatcccctgaatccctttgtcagtcataaagagattgattctgtaaacaatgctgtggtaatgccagcccatggtccagccaaaagcatggaatatgatgccgttgcaactacacattcctggattcccaagaggaatcgttctattctcaacacaagccaaaggggaattcttgaggatgaacagatgtaccagaagtgctgcaatctattcgagaaatttttccctagcagttcatataggagaccggttggaatttctagcatggtggaggccatggtgtctagggcccggattgatgccagggtcgacttcgagtctggacggatcaagaaagaagagttctctgagatcatgaagatctgttccaccattgaagaactcagacggcaaaaataa

>H1N1_PB1_CY065033_A/New York/3835/2010

atggatgtcaatccgactctacttttcctaaaaattccagcgcaaaatgccataagcaccacattcccttatactggagatcctccatacagccatggaacaggaacaggatacaccatggacacagtaaacagaacacaccaatactcagaaaagggaaagtggacgacaaacacagagactggtgcaccccagctcaacccgattgatggaccactacctgaggataatgaaccaagtgggtatgcacaaacagactgtgttctagaggctatggctttccttgaagaatcccacccaggaatatttgagaattcatgccttgaaacaatggaagttgttcaacaaacaagggtagataaactaactcaaggtcgccagacttatgattggacattaaacaggaatcaaccggcagcaactgcattggccaacaccatagaagtctttagatcgaatggcctaacagctaatgagtcaggaaggctaatagatttcttaaaggatgtaatggaatcaatgaacaaagaggaaatagagataacaacccactttcaaagaaaaaggagagtaagagacaacatgaccaagaagatggtcacgcaaagaacaatagggaagaaaaaacaaagactgaataagagaggctatctaataagagcactgacattaaatacgatgaccaaagatgcagagagaggcaagttaaaaagaagggctatcgcaacacctgggatgcagattagaggtttcgtatactttgttgaaactttagctaggagcatttgcgaaaagcttgaacagtctgggctcccagtagggggcaatgaaaagaaggccaaactggcaaatgttgtgagaaagatgatgactaattcacaagacacagagatttctttcacaatcactggggacaacactaagtggaatgaaaatcaaaatcctcgaatgttcctggcgatgattacatatatcaccagaaatcaacccgagtggttcagaaacatcctgagcatggcacccataatgttctcaaacaaaatggcaagactagggaaagggtacatgttcgagagtaaaagaatgaagattcgaacacaaataccagcagaaatgctagcaagcattgacctgaagtacttcaatgaatcaacaaagaagaaaattgagaaaataaggcctcttctaatagatggcacagcatcactgagtcctgggatgatgatgggcatgttcaacatgctaagtacggtcttgggagtctcgatactgaatcttggacaaaagaaatacaccaagacaatatactggtgggatgggctccaatcatccgacgattttgctctcatagtgaatgcaccaaaccatgagggaatacaagcaggagtggacagattctacaggacctgcaagttagtgggaatcaacatgagcaaaaagaagtcctatataaataagacagggacatttgaattcacaagctttttttatcgctatgggtttgtggctaattttagcatggagctacccagctttggagtgtctggagtaaatgaatcagctgacatgagtattggagtaacagtgataaagaacaacatgataaacaatgaccttggacctgcaacggcccagatggctcttcaattgttcatcaaagactacagatacacatataggtgccataagggagacacacaaattcagacgagaagatcatttgagttaaagaagctgtgggaccaaacccaatcaaaggtagggctattagtatcagatggaggaccaaacttatacaatatacggaatcttcacattcctgaagtctgcttaaaatgggagctaatggatgatgattatcggggaagactttgtaatcccctgaatccctttgtcagtcataaagagattgattctgtaaacaatgctgtggtaatgccagcccatggtccagccaaaagcatggaatatgatgccgttgcaactacacattcctggattcccaagaggaatcgttctattctcaacacaagccaaaggggaattcttgaggatgaacagatgtaccagaagtgctgcaatctattcgagaagtttttccctagcagttcatataggagaccggttggaatttctagcatggtggaggccatggtgtctagggcccggattgatgccagggtcgacttcgagtctggacggatcaagaaagaagagttctctgagatcatgaagatctgttccaccattgaagaactcagacggcaaaaataa

>H1N1_PB1_HM569697_A/Argentina/19656/2009

atggatgtcaatccgactctacttttcctaaaaattccagcgcaaaatgccataagcaccacattcccttatactggagatcctccatacagccatggaacaggaacaggatacaccatggacacagtaaacagaacacaccaatactcagaaaagggaaagtggacgacaaacacagagactggtgcaccccagctcaacccgattgatggaccactacctgaggataatgaaccaagtgggtatgcacaaacagactgtgttctagaggctatggctttccttgaagaatcccacccaggaatatttgagaattcatgccttgaaacaatggaagttgttcaacaaacaagggtagataaactaactcaaggtcgccagacttatgattggacattaaacagaaatcaaccggcagcaactgcattggccaacaccatagaagtctttagatcgaatggcctaacagctaatgagtcaggaaggctaatagatttcttaaaggatgtaatggaatcaatgaacaaagaggaaatagagataacaacccactttcaaagaaaaaggagagtaagagacaacatgaccaagaagatggtcacgcaaagaacaatagggaagaaaaaacaaagactgaataagagaggctatctaataagagcactgacattaaatacgatgaccaaagatgcagagagaggcaagttaaaaagaagggctatcgcaacacctgggatgcagattagaggtttcgtatactttgttgaaactttagctaggagcatttgcgaaaagcttgaacagtctgggctcccagtagggggcaatgaaaagaaggccaaactggcaaatgttgtgagaaagatgatgactaattcacaagacacagagatttctttcacaatcactggggacaacactaagtggaatgaaaatcaaaatcctcgaatgttcctggcgatgattacatatatcaccagaaatcaacccgagtggttcagaaacatcctgagcatggcacccataatgttctcaaacaaaatggcaagactagggaaagggtacatgttcgagagtaaaagaatgaagatccgaacacaaataccagcagaaatgctagcaagcattgacctgaagtacttcaatgactcaacaaagaagaaaattgagaaaataaggcctcttctaatagatggcacagcatcactgagtcctgggatgatgatgggcatgttcaacatgctaagtacggtcttgggagtctcgatactgaatcttggacaaaagaaatacaccaagacaatatactggtgggatgggctccaatcatccgacgattttgctctcatagtgaatgcaccaaaccatgagggaatacaagcaggagtggacagattctacaggacctgcaagttagtgggaatcaacatgagcaaaaagaagtcctatataaataagacagggacatttgaattcacaagctttttttatcgctatggatttgtggctaattttagcatggagctacccagctttggagtgtctggagtaaatgaatcagctgacatgagtattggagtaacagtgataaagaacaacatgataaacaatgaccttggacctgcaacggcccagatggctcttcaattgttcatcaaagactacagatacacatataggtgccataggggagacacacaaattcagacgagaagatcatttgagttaaagaagctgtgggatcaaacccaatcaaaggtagggctattagtatcagatggaggaccaaacttatacaatatacggaatcttcacattcctgaagtctgcttaaaatgggagctaatggatgatgattatcggggaagactttgtaatcccctgaatccctttgtcagtcataaagagattgattctgtaaacaatgctgtggtaatgccagcccatggtccagccaaaagcatggaatatgatgccgttgcaactacacattcctggattcccaagaggaatcgttctattctcaacacaagccaaaggggaattcttgaggatgaacagatgtaccagaagtgctgcaatctattcgagaaatttttccctagcagttcatataggagaccggttggaatttctagcatggtggaggccatggtgtctagggcccggattgatgccagggtcgacttcgagtctggacggatcaagaaagaagagttctctgagatcatgaagatctgttccaccattgaagaactcagacggcaaaaataa

>H1N1_PB1_HM567678_A/England/349/2009

atggatgtcaatccgactctacttttcctaaaaattccagcgcaaaatgccataagcaccacattcccttatactggagatcctccatacagccatggaacaggaacaggatacaccatggacacagtaaacagaacacaccaatactcagaaaagggaaagtggacgacaaacacagagactggtgcaccccagctcaacccgattgatggaccactacctgaggataatgaaccaagtgggtatgcacaaacagactgtgttctagaggctatggctttccttgaagaatcccacccaggaatatttgagaattcatgccttgaaacaatggaagttgttcaacaaacaagggtagataaactaactcaaggtcgccagacttatgattggacattaaacagaaatcaaccggcagcaactgcattggccaacaccatagaagtctttagatcgaatggcctaacagctaatgagtcaggaaggctaatagatttcttaaaggatgtaatggaatcaatgaacaaagaggaaatagagataacaacccactttcaaagaaaaaggagagtaagagacaacatgaccaagaagatggtcacgcaaagaacaatagggaagaaaaaacaaagactgaataagagaggctatctaataagagcactgacattaaatacgatgaccaaagatgcagagagaggcaagttaaaaagaagggctatcgcaacacctgggatgcagattagaggtttcgtatactttgttgaaactttagctaggagcatttgcgaaaagcttgaacagtctgggctcccagtagggggcaatgaaaagaaggccaaactggcaaatgttgtgagaaagatgatgactaattcacaagacacagagatttctttcacaatcactggggacaacactaagtggaatgaaaatcaaaatcctcgaatgttcctggcgatgattacatatatcaccagaaatcaacccgagtggttcagaaacatcctgagcatggcacccataatgttctcaaacaaaatggcaagactagggaaagggtacatgttcgagagtaaaagaatgaagattcgaacacaaataccagcagaaatgctagcaagcattgacctgaagtacttcaatgaatcaacaaagaagaaaattgagaaaataaggcctcttctaatagatggcacagcatcactgagtcctgggatgatgatgggcatgttcaacatgctaagtacggtcttgggagtctcgatactgaatcttggacaaaagaaatacaccaagacaatatactggtgggatgggctccaatcatccgacgattttgctctcatagtgaatgcaccaaaccatgagggaatacaagcaggagtggacagattctacaggacctgcaagttagtgggaatcaacatgagcaaaaagaagtcctatataaataagacagggacatttgaattcacaagctttttttatcgctatggatttgtggctaattttagcatggagctacccagctttggagtgtctggagtaaatgaatcagctgacatgagtattggagtaacagtgataaagaacaacatgataaacaatgaccttggacctgcaacggcccagatggctcttcaattgttcatcaaagactacagatacacatataggtgccataggggagacacacaaattcagacgagaagatcatttgagttaaagaagctgtgggatcaaacccaatcaaaggcagggctattagtatcagatggaggaccaaacttatacaatatacggaatcttcacattcctgaagtctgcttaaaatgggagctaatggatgatgattatcggggaagactttgtaatcccctgaatccctttgtcagtcataaagagattgattctgtaaacaatgctgtggtaatgccagcccatggtccagccaaaagcatggaatatgatgccgttgcaactacacattcctggattcccaagaggaatcgttctattctcaacacaagccaaaggggaattcttgaggatgaacagatgtaccagaagtgctgcaatctattcgagaaatttttccctagcagttcatataggagaccggttggaatttctagcatggtggaggccatggtgtctagggcccggattgatgccagggtcgacttcgagtctggacggatcaagaaagaagagttctctgagatcatgaagatctgttccaccattgaagaactcagacggcaaaaataa

>H1N1_PB1_HM567750_A/England/377/2009

atggatgtcaatccgactctacttttcctaaaaattccagcgcaaaatgccataagcaccacattcccttatactggagatcctccatacagccatggaacaggaacaggatacaccatggacacagtaaacagaacacaccaatactcagaaaagggaaagtggacgacaaacacagagactggtgcaccccagctcaacccgattgatggaccactacctgaggataatgaaccaagtgggtatgcacaaacagactgtgttctagaggctatggctttccttgaagaatcccacccaggaatatttgagaattcatgccttgaaacaatggaagttgttcaacaaacaagggtagataaactaactcaaggtcgccagacttatgattggacattaaacagaaatcaaccggcagcaactgcattggccaacaccatagaagtctttagatcgaatggcctaacagctaatgagtcaggaaggctaatagatttcttaaaggatgtaatggaatcaatgaacaaagaggaaatagagataacaacccactttcaaagaaaaaggagagtaagagacaacatgaccaaaaagatggtcacgcaaagaacaatagggaagaaaaaacaaagactgaataagagaggctatctaataagagcactgacattaaatacgatgaccaaagatgcagagagaggcaagttaaaaagaagggctatcgcaacacctgggatgcagattagaggtttcgtatactttgttgaaactttagctaggagcatttgcgaaaagcttgaacagtctgggctcccagtagggggcaatgaaaagaaggccaaactggcaaatgttgtgagaaagatgatgactaattcacaagacacagagatttctttcacaatcactggggacaacactaagtggaatgaaaatcaaaatcctcgaatgttcctggcgatgattacatatatcaccagaaatcaacccgagtggttcagaaacatcctaagcatggcacccataatgttctcaaacaaaatggcaagactagggaaagggtacatgttcgagagtaaaagaatgaagattcgaacacaaataccagcagaaatgctagcaagcattgacctgaagtacttcaatgaatcaacaaagaagaaaattgagaaaataaggcctcttctaatagatggcacagcatcactgagtcctgggatgatgatgggcatgttcaacatgctaagtacggtcttgggagtctcgatactgaatcttggacaaaagaaatacaccaagacaatatactggtgggatgggctccaatcatccgacgattttgctctcatagtgaatgcaccaaaccatgagggaatacaagcaggagtggacagattctacaggacctgcaagttagtgggaatcaacatgagcaaaaagaagtcctatataaataagacagggacatttgaattcacaagctttttttatcgctatggatttgtggctaattttagcatggagctacccagctttggagtgtctggagtaaatgaatcagctgacatgagtattggagtaacagtgataaagaacaacatgataaacaatgaccttggacctgcaacggcccagatggctcttcaattgttcatcaaagactacagatacacatataggtgccataggggagacacacaaattcagacgagaagatcatttgagttaaagaagctgtgggatcaaacccaatcaaaggcagggctattagtatcagatggaggaccaaacttatacaatatacggaatcttcacattcctgaagtctgcttaaaatgggagctaatggatgatgattatcggggaagactttgtaatcccctgaatccctttgtcagtcataaagagattgattctgtaaacaatgctgtggtaatgccagcccatggtccagccaaaagcatggaatatgatgccgttgcaactacacattcctggattcccaagaggaatcgttctattctcaacacaagccaaaggggaattcttgaggatgaacagatgtaccagaagtgctgcaatctattcgagaaatttttccctagcagttcatataggagaccggttggaatttctagcatggtggaggccatggtgtctagggcccggattgatgccagggtcgacttcgagtctggacggatcaagaaagaagagttctctgagatcatgaagatctgttccaccattgaagaactcagacggcaaaaataa

>H1N1_PB1_HM567950_A/England/857/2009

atggatgtcaatccgactctacttttcctaaaaattccagcgcaaaatgccataagcaccacattcccttatactggagatcctccatacagccatggaacaggaacaggatacaccatggacacagtgaacagaacacaccaatactcagaaaagggaaagtggacgacaaacacagagactggtgcaccccagctcaacccgattgatggaccactacctgaggataatgaaccaagtgggtatgcacaaacagactgtgttctagaggctatggctttccttgaagaatcccatccaggaatatttgagaattcatgccttgaaacaatggaagttgttcaacaaacaagggtagataaactaactcaaggtcgccagacttatgattggacattaaacagaaatcaaccggcagcaactgcattggccaacaccatagaagtctttagatcgaatggcctaacagctaatgagtcaggaaggctaatagatttcttaaaggatgtaatggaatcaatgaacaaagaggaaatagagataacaacccactttcaaagaaaaaggagagtaagagacaacatgaccaagaagatggtcacgcaaagaacaatagggaagaaaaaacaaagactgaataagagaggctatctaataagagcactgacattaaatacgatgaccaaagatgcagagagaggcaagttaaaaagaagggctatcgcaacacctgggatgcagattagaggtttcgtatactttgttgaaactttagctaggagcatttgcgaaaagcttgaacagtctgggctcccagtagggggcaatgaaaagaaggccaaactggcaaatgttgtgagaaagatgatgactaattcacaagacacagagatttctttcacaatcactggggacaacactaagtggaatgaaaatcaaaatcctcgaatgttcctggcgatgattacatatatcaccagaaatcaacccgagtggttcagaaacatcctgagcatggcacccataatgttctcaaacaaaatggcaagactagggaaagggtacatgttcgagagtaaaagaatgaagattcgaacacaaataccagcagaaatgctagcaagcattgacctgaagtacttcaatgaaccaacaaagaagaaaattgagaaaataaggcctcttctaatagatggcacagcatcactgagtccggggatgatgatgggcatgttcaacatgctaagtacggtcttgggagtctcgatactgaatcttggacaaaagaaatacaccaagacaatatactggtgggatgggctccaatcatccgacgattttgctctcatagtgaatgcaccaaaccatgagggaatacaagcaggagtggacagattctacaggacctgcaagttagtgggaatcaacatgagcaaaaagaagtcctatataaataagacagggacatttgaattcacaagctttttttatcgctatggatttgtggctaattttagcatggagctacccagctttggagtgtctggagtaaatgaatcagctgacatgagtattggagtaacagtgataaagaacaacatgataaacaatgaccttggacctgcaacggcccagatggctcttcaattgttcatcaaagactacagatacacatataggtgccataggggagacacacaaattcagacgagaagatcatttgagttaaagaagctgtgggatcaaacccaatcaaaggtagggctattagtatcagatggaggaccaaacttatacaatatacggaatcttcacattcctgaagtctgcttaaaatgggagctaatggatgatgattatcggggaagactttgtaatcccctgaatccctttgtcagtcataaagagattgattctgtaaacaatgctgtggtaatgccagcccatggtccagccaaaagcatggaatatgatgccgttgcaactacacattcctggatccccaagaggaatcgttctattctcaacacaagccaaaggggaattcttgaggatgaacagatgtaccagaagtgctgcaatctattcgagaaatttttccctagcagttcatataggagaccggttggaatttctagcatggtggaggccatggtgtctagggcccggattgatgccagggtcgacttcgagtctggacggatcaagaaagaagagttctctgagatcatgaagatctgttccaccattgaagaactcagacggcaaaaataa

>H1N1_PB1_HM567998_A/England/900/2009

atggatgtcaatccgactctacttttcctaaaaattccagcgcaaaatgccataagcaccacattcccttatactggagatcctccatacagccatggaacaggaacaggatacaccatggacacagtaaacagaacacaccaatactcagaaaagggaaagtggacgacaaacacagagactggtgcaccccagctcaacccgattgatggaccactacctgaggataatgaaccaagtgggtatgcacaaacagactgtgttctagaggctatggctttccttgaagaatcccacccaggaatatttgagaattcatgccttgaaacaatggaagttgttcaacaaacaagggtagataaactaactcaaggtcgccagacttatgattggacattaaacagaaatcaaccggcagcaactgcgttggccaacaccatagaagtctttagatcgaatggcctaacagctaatgagtcaggaaggctaatagatttcttaaaggatgtaatggaatcaatgaacaaagaggaaatagagataacaacccactttcaaagaaaaaggagagtaagagacaacatgaccaagaagatggtcacgcaaagaacaatagggaagaaaaaacaaagactgaataagagaggctatctaataagagcactgacattaaatacaatgaccaaagatgcagagagaggcaagttaaaaagaagggctatcgcaacacctgggatgcagattagaggtttcgtatactttgttgaaactttagctaggagcatttgcgaaaagcttgaacagtctgggctcccagtagggggcaatgaaaagaaggccaaactggcaaatgttgtgagaaagatgatgactaattcacaagacacagagatttctttcacaatcactggggacaacactaagtggaatgaaaatcaaaatcctcgaatgttcctggcgatgattacatatatcaccagaaatcaacccgagtggttcagaaacatcctgagcatggcacccataatgttctcaaacaaaatggcaagactagggaaagggtacatgttcgagagtaaaagaatgaagattcgaacacaaataccagcagaaatgctagcaagcattgacctgaagtacttcaatgaatcaacaaagaagaaaattgagaaaataaggcctcttctaatagatggcacagcatcactgagtcctgggatgatgatgggcatgttcaacatgctaagtacggtcttgggagtctcgatactgaatcttggacaaaagaaatacaccaagacaatatactggtgggatgggctccaatcatccgacgattttgctctcatagtgaatgcaccaaaccatgagggaatacaagcaggagtggacagattctacaggacctgcaagttagtgggaatcaacatgagcaaaaagaagtcctatataaataagacagggacatttgaattcacaagctttttttatcgctatggatttgtggctaattttagcatggagctacccagctttggagtgtctggagtaaatgaatcagctgacatgagtattggagtaacagtgataaagaacaacatgataaacaatgaccttggacctgcaacggcccagatggctcttcaactgttcatcaaagactacagatacacatataggtgccataggggagacacacaaattcagacgagaagatcatttgagttaaagaagctgtgggatcaaacccaatcaaaggtagggctattagtatcagatggaggaccaaacttatacaatatacggaatcttcacattcctgaagtctgcttaaaatgggagctaatggatgatgattatcggggaagactttgtaatcccctgaatccctttgtcagtcataaagagattgattctgtaaacaatgctgtggtaatgccagcccatggtccagccaaaagcatggaatatgatgccgttgcaactacacattcctggattcccaagaggaatcgttccattctcaacacaagccaaaggggaattcttgaggatgaacagatgtaccagaagtgctgcaatctattcgagaaatttttccctagcagttcatataggagaccggttggaatttctagcatggtggaggccatggtgtctagggcccggattgatgccagggtcgacttcgagtctggacggatcgggaaggaagagttctctgagatcatgaagatctgttccaccattgaagaactcagacggcaaaaataa

>H1N1_PB1_HM568046_A/England/950/2009

atggatgtcaatccgactctacttttcctaaaaattccagcgcaaaatgccataagcaccacattcccttatactggagatcctccatacagccatgggacaggaacaggatacaccatggacacagtaaacagaacacaccaatactcagaaaagggaaagtggacgacaaacacagagactggtgcaccccagctcaacccgattgatggaccactacctgaggataatgaaccaagtgggtatgcacaaacagactgtgttctagaggctatggctttccttgaagaatcccacccaggaatatttgagaattcatgccttgaaacaatggaagttgttcaacaaacaagggtagataaactaactcaaggtcgccagacttatgattggacattaaacagaaatcaaccggcagcaactgcattggccaacaccatagaagtctttagatcgaatggcctaacagctaatgagtcaggaaggctaatagatttcttaaaggatgtaatggaatcaatgaacaaagaggaaatagagataacaacccacttccaaagaaaaaggagagtaagagacaacatgaccaagaagatggtcacgcaaagaacaatagggaagaaaaaacaaagactgaataagagaggctatctaataagagcactgacattaaatacgatgaccaaagatgcagagagaggcaagttaaaaagaagggctatcgcaacacctgggatgcagattagaggtttcgtatactttgttgaaactttagctaggagcatttgcgaaaagcttgaacagtctgggctcccagtagggggcaatgaaaagaaggccaaactggcaaatgttgtgagaaagatgatgactaattcacaagacacagagatttctttcacaatcactggggacaacactaagtggaatgaaaatcaaaatcctcgaatgttcctggcgatgattacatatatcaccagaaatcaacccgagtggttcagaaacatcctgagcatggcacccataatgttctcaaacaaaatggcaagactagggaaagggtacatgttcgagagtaaaagaatgaagattcgaacacaaataccagcagaaatgctagcaagcattgacctgaagtacttcaatgaatcaacaaagaagaaaattgagaaaataaggcctcttctaatagatggcacagcatcactgagtcctgggatgatgatgggcatgttcaacatgctaagtacggtcttgggagtctcgatactgaatcttggacaaaagaaatacaccaagacaatatactggtgggatgggctccaatcatccgacgattttgctctcatagtgaatgcaccaaaccatgagggaatacaagcaggagtggacagattctacaggacctgcaagttagtgggaatcaacatgagcaaaaagaagtcctatataaataagacagggacatttgaattcacaagctttttttatcgctatggatttgtggctaattttagcatggagctacccagctttggagtgtctggaataaatgaatcagctgacatgagtattggagtaacagtgataaagaacaacatgataaacaatgaccttggacctgcaacggcccagatggctcttcaattgttcatcaaagactacagatacacatataggtgccataggggagacacacaaattcagacgagaagatcatttgagttaaagaagctgtgggatcaaacccaatcaaaggtagggctattagtatcagatggaggaccaaacttatacaatatacggaatcttcacattcctgaagtctgcttaaaatgggagctaatggatgatgattatcgggggagactttgtaatcccctgaatccctttgtcagtcataaagagattggttctgtaaacaatgctgtggtaatgccagcccatggtccagccaaaagcatggaatatgatgccgttgcaactacacattcctggattcccaagaggaatcgttctattctcaacacaagccaaaggggaattcttgaggatgaacagatgtaccagaagtgctgcaatctattcgagaaatttttccctagcagttcatataggagaccggttggaatttctagcatggtggaggccatggtgtctagggcccggattgatgccagggtcgacttcgagtctggacggatcaagaaagaagagttctctgagatcatgaagatctgttccaccattgaagaactcagacggcaaaaataa

>H1N1_PB1_CY065886_A/Netherlands/1093/2009

atggatgtcaatccgactctacttttcctaaaaattccagcgcaaaatgccataagcaccacattcccttatactggagatcctccatacagccatggaacaggaacaggatacaccatggacacagtaaacagaacacaccaatactcagaaaagggaaagtggacgacaaacacagagactggtgcaccccagctcaacccgattgatggaccactacctgaggataatgaaccaagtgggtatgcacaaacagactgtgttctagaggctatggctttccttgaagaatcccacccaggaatatttgagaattcatgccttgaaacaatggaagttgttcaacaaacaagggtagataaactaactcaaggtcgccagacttatgattggacattaaacagaaatcaaccggcagcaactgcattggccaacaccatagaagtctttagatcgaatggcctaacagctaatgagtcaggaaggctaatagatttcttaaaggatgtaatggaatcaatgaacaaagaggaaatagagataacaacccactttcaaagaaaaaggagagtaagagacaacatgaccaagaagatggtcacgcaaagaacaatagggaagaaaaaacaaagactgaataagagaggctatctaataagagcactgacattaaatacgatgaccaaagatgcagagagaggcaagttaaaaagaagggctatcgcaacacctgggatgcagattagaggtttcgtatactttgttgaaactttagctaggagcatttgcgaaaagcttgaacagtctgggctcccagtagggggcaatgaaaagaaggccaaactggcaaatgttgtgagaaagatgatgactaattcacaagacacagagatttctttcacaatcactggggacaacactaagtggaatgaaaatcaaaatcctcgaatgttcctggcgatgattacatatatcaccagaaatcaacccgagtggttcagaaacatcctgagcatggcacccataatgttctcaaacaaaatggcaagactagggaaagggtacatgttcgagagtaaaagaatgaagattcgaacacaaataccagcagaaatgctagcaagcattgacctgaagtacttcaatgaatcaacaaagaagaaaattgagaaaataaggcctcttctaatagatggcacagcatcactgagtcctgggatgatgatgggcatgttcaacatgctaagtacggtcttgggagtctcgatactgaatcttggacaaaagaaatacaccaagacaatatactggtgggatgggctccaatcatccgacgattttgctctcatagtgaatgcaccaaaccatgagggaatacaagcaggagtggacagattctacaggacctgcaagttagtgggaatcaacatgagcaaaaagaagtcctatataaataagacagggacatttgaattcacaagctttttttatcgctatggatttgtggctaattttagcatggagctacccagctttggagtgtctggagtaaatgaatcagctgacatgagtattggagtaacagtgataaagaacaacatgataaacaatgaccttggacctgcaacggcccagatggctcttcaattgttcatcaaagactacagatacacatataggtgccataggggagacacacaaattcagacgagaagatcatttgagttaaagaagctgtgggatcaaacccaatcaaaggtagggctattagtatcagatggaggaccaaacttatacaatatacggaatcttcacattcctgaagtctgcttaaaatgggagctaatggatgatgattatcggggaagactttgtaatcccctgaatccctttgtcagtcataaagagattgattctgtaaacaatgctgtggtaatgccagcccatggtccagccaaaagcatggaatatgatgccgttgcaactacacattcctggattcccaaaaggaatcgttctattctcaacacaagccaaaggggaattcttgaggatgaacagatgtaccagaagtgctgcaatctattcgagaaatttttccctagcagttcatataggagaccggttggaatttctagcatggtggaggccatggtgtctagggcccggattgatgccagggtcgacttcgagtctggacggatcaagaaagaagagttctctgagatcatgaagatctgttccaccattgaagaactcagacggcaaaaataa

>H1N1_PB1_CY065902_A/Netherlands/2822/2009

atggatgtcaatccgactctacttttcctaaaaattccagcgcaaaatgccataagcaccacattcccttatactggagatcctccatacagccatggaacaggaacaggatacaccatggacacagtaaacagaacacaccaatactcagaaaagggaaagtggacgacaaacacagagactggtgcaccccagctcaacccgattgatggaccactacctgaggataatgaaccaagtgggtatgcacaaacagactgtgttctagaggctatggctttccttgaagaatcccacccaggaatatttgagaattcatgccttgaaacaatggaagttgttcaacaaacaagggtagataaattaactcaaggtcgccagacttatgattggacattaaacagaaatcaaccggcagcaactgcattggccaacaccatagaagtctttagatcgaatggcctaacagctaatgagtcaggaaggctaatagatttcttaaaggatgtaatggaatcaatgaacaaagaggaaatagagataacaacccactttcaaagaaaaaggagagtaagagacaacatgaccaagaagatggtcacgcaaagaacaatagggaagaaaaaacaaagactgaataagagaggctatctaataagagcactgacattaaatacgatgaccaaagatgcagagagaggcaagttaaaaagaagggctatcgcaacacctgggatgcagattagaggtttcgtatactttgttgaaactttagctaggagcatttgcgaaaagcttgaacagtctgggctcccagtagggggcaatgaaaagaaggccaaactggcaaatgttgtgagaaagatgatgactaattcacaagacacagagatttctttcacaatcactggggacaacactaagtggaatgaaaatcaaaatcctcgaatgttcctggcgatgattacatatatcaccagaaatcaacccgagtggttcagaaacatcctgagcatggcacccataatgttctcaaacaaaatggcaagactagggaaagggtacatgttcgagagtaaaagaatgaagattcgaacacaaataccagcagaaatgctagcaagcattgacctgaagtacttcaatgaatcaacaaagaagaaaattgagaaaataaggcctcttctaatagatggcacagcatcactgagtcctgggatgatgatgggcatgttcaacatgctaagtacggtcttgggagtctcgatactgaatcttggacaaaagaaatacaccaagacaatatactggtgggatgggctccaatcatccgacgattttgctctcatagtgaatgcaccaaaccatgagggaatacaagcaggagtggacagattctacaggacctgcaagttagtgggaatcaacatgagcaaaaagaagtcctatataaataagacagggacatttgaattcacaagctttttttatcgctatggatttgtggctaattttagcatggagctacccagctttggagtgtctggagtaaatgaatcagctgacatgagtattggagtaacagtgataaagaacaacatgataaacaatgaccttggacctgcaacggcccagatggctcttcaattgttcatcaaagactacagatacacatataggtgccataggggagacacacaaattcagacgagaagatcatttgagttaaagaagctgtgggatcaaacccaatcaaaggtagggctattagtatcagatggaggaccaaacttatacaatatacggaatcttcacattcctgaagtctgcttaaaatgggagctaatggatgatgattatcggggaagactttgtaatcccctgaatccctttgtcagtcataaagagattgattctgtaaacaatgctgtggtaatgccagcccatggtccagccaaaagcatggaatatgatgccgttgcaactacacattcctggattcccaagaggaatcgttctattctcaacacaagccaaaggggaattcttgaggatgaacagatgtaccagaagtgctgcaatctattcgagaaatttttccctagcagttcatataggagaccggttggaatttctagcatggtggaggccatggtgtctagggcccggattgatgccagggtcgacttcgagtctggacggatcaagaaagaagagttctctgagatcatgaagatctgttccaccattgaagaactcagacggcaaaaataa

>H1N1_PB1_CY065942_A/Guam/NHRC0002/2009

atggatgtcaatccgactctacttttcctaaaaattccagcgcaaaatgccataagcaccacattcccttatactggagatcctccatacagccatggaacaggaacaggatacaccatggacacagtaaacagaacacaccaatactcagaaaagggaaagtggacgacaaacacagagactggtgcaccccagctcaacccgattgatggaccactacctgaggataatgaaccaagtgggtatgcacaaacagactgtgttctagaggctatggctttccttgaagaatcccacccaggaatatttgagaattcatgccttgaaacaatggaagttgttcaacaaacaagggtagataaactaactcaaggtcgccagacttatgattggacattaaacagaaatcaaccggcagcaactgcattggccaacaccatagaagtctttagatcgaatggcctaacagctaatgagtcaggaaggctaatagatttcttaaaggatgtaatggaatcaatgaacaaagaggaaatagagataacaacccactttcaaagaaaaaggagagtaagagacaacatgaccaagaagatggtcacgcaaagaacaatagggaagaaaaaacaaagactgaataagagaggctatctaataagagcactgacattaaatacgatgaccaaagatgcagagagaggcaagttaaaaagaagggctatcgcaacacctgggatgcagattagaggtttcgtatactttgttgaaactttagctaggagcatttgcgaaaagcttgaacagtctgggctcccagtagggggcaatgaaaagaaggccaaactggcaaatgttgtgagaaagatgatgactaactcacaagacacagagatttctttcacaatcactggggacaacactaagtggaatgaaaatcaaaatcctcgaatgttcctggcgatgattacatatatcaccagaaatcaacccgagtggttcagaaacatcctgagcatggcacccataatgttctcaaacaaaatggcaagactagggaaagggtacatgttcgagagtaaaagaatgaagattcgaacacaaataccagcagaaatgctagcaagcattgacctgaagtacttcaatgaatcaacaaagaagaaaattgagaaaataaggcctcttctaatagatggcacagcatcactgagtcctgggatgatgatgggcatgttcaacatgctaagtacggtcttgggagtctcgatactgaatcttggacaaaagaaatacaccaagacaatatactggtgggatgggctccaatcatccgacgattttgctctcatagtgaatgcaccaaaccatgagggaatacaagcaggagtggacagattctacaggacctgcaagttagtgggaatcaacatgagcaaaaagaagtcctatataaataagacagggacatttgaattcacaagctttttttatcgctatggatttgtggctaattttagcatggagctacccagctttggagtgtctggagtaaatgaatcagctgacatgagtattggagtaacagtgataaagaacaacatgataaacaatgaccttggacctgcaacggcccagatggctcttcaattgttcatcaaagactacagatacacatataggtgccataggggagacacacaaattcagacgagaagatcatttgagttaaagaagctgtgggatcaaacccaatcaaaggtagggctattagtatcagatggaggaccaaacttatacaatatacggaatcttcacatccctgaagtctgcttaaaatgggagctaatggatgatgattatcggggaagactttgtaatcccctgaacccctttgtcagtcataaagagattgattctgtaaacaatgctgtggtaatgccagcccatggtccagccaaaagcatggaatatgatgccgttggaactacacattcctggattcccaagaggaatcgttctattctcaacacaagccaaaagggaattcttgaggatgaacagatgtaccagaagtgctgcaatctattcgagaaatttttccctagcagttcatataggagaccggttggaatttctagcatggtggaggccatggtgtctagggcccggattgatgccagggtcgacttcgagtctggacggatcaagaaagaagagttctctgagatcatgaagatctgttccaccattgaagaactcagacggcaaaaataa

>H1N1_PB1_CY066413_A/California/VRDL129/2009

atggatgtcaatccgactctacttttcctaaaaattccagcgcaaaatgccataagcaccacattcccttatactggagatcctccatacagccatggaacaggaacaggatacaccatggacacagtaaacagaacacaccaatactcagaaaagggaaaatggacgacaaacacagagactggtgcaccccagctcaacccgattgatggaccactacctgaggataatgaaccaagtgggtatgcacaaacagattgtgttctagaggctatggctttccttgaagaatcccacccaggaatatttgagaattcatgccttgaaacaatggaagttgttcaacaaacaagagtagataaactaactcaaggtcgccagacttatgattggacattaaacagaaatcaaccggcagcaactgcattggccaacaccatagaagtctttagatcgaatggcctaacagctaatgagtcaggaaggctaatagatttcttaaaggatgtaatggaatcaatgaacaaagaggaaatagagataacaacccactttcaaagaaaaaggagagtaagagacaacatgaccaagaagatggtcacgcaaagaacaatagggaagaaaaaacaaagactgaataagagaggctatctaataagagcactgacattaaatacgatgaccaaagatgcagagagaggcaagttaaaaagaagggctatcgcaacacctgggatgcagattagaggtttcgtatactttgttgaaactttagctaggagcatttgcgaaaagcttgaacagtccgggctcccagtagggggcaatgaaaagaaggccaaactggcaaatgttgtgagaaagatgatgactaattcacaagacacagagatttctttcacaatcactggggacaacactaagtggaatgaaaatcaaaatcctcgaatgttcctggcgatgattacatatatcaccagaaatcaacccgagtggttcagaaacatcctgagcatggcacccataatgttctcaaacaaaatggcaagactagggaaagggtacatgttcgagagtaaaagaatgaagattcgaacacaaataccagcagaaatgctagcaagcattgacctgaagtacttcaatgaatcaacaaagaagaaaattgagaaaataaggcctcttctaatagatggcacagcatcactgagtcctgggatgatgatgggcatgttcaacatgctaagtacggtcttgggagtctcgatactgaatcttggacaaaagaaatacaccaagacaatatactggtgggatgggctccaatcatccgacgattttgctctcatagtgaatgcaccaaaccatgagggaatacaagcaggagtggacagattctacaggacctgcaagttagtgggaatcaacatgagcaaaaagaagtcctatataaataagacagggacatttgaattcacaagctttttttatcgctatggatttgtggctaattttagcatggagctacccagctttggagtgtctggagtaaatgaatcagccgacatgagtattggagtaacagtgataaagaacaacatgataaacaatgaccttggacctgcaacggcccagatggctcttcaattgttcatcaaagactacagatacacatataggtgccataggggagacacacaaattcagacgagaagatcatttgagttaaagaagctgtgggatcaaacccaatcaaagggagggctattagtatcagatggaggaccaaacttatacaatatacggaatcttcacattcctgaagtctgcttaaaatgggagctaatggatgatgattatcggggaagactttgtaatcccctgaatccctttgtcagtcataaagagattgattctgtaaacaatgctgtggtaatgccagcccatggtccagtcaaaagcatggaatatgatgccgttgcaactacacattcctggattcccaagaggaatcgttctattctcaacacaagccaaaggggaattcttgaggatgaacagatgtaccagaagtgctgcaatctattcgagaaatttttccctagcagttcatataggagaccggttggaatttctagcatggtggaggccatggtgtctagggcccggattgatgccagggtcgacttcgagtctggacggatcaagaaagaagagttctctgagatcatgaagatctgctccaccattgaagaactcagacggcaaaaataa

>H1N1_PB1_CY066501_A/California/VRDL8/2010

atggatgtcaatccgactctacttttcctaaaaattccagcgcaaaatgccataagcaccacattcccttatactggagatcctccatacagccatggaacaggaacaggatacaccatggacacagtaaacagaacacaccaatactcagaaaagggaaagtggacgacaaacacagagactggtgcaccccagctcaacccgattgatggaccactacctgagggtaatgaaccaagtgggtatgcacaaacagactgtgttctagaggctatggctttccttgaagaatcccacccaggaatatttgagaattcatgccttgaaacaatggaagttgttcaacaaacaagggtagataaattaactcaaggtcgccagacttatgattggacattaaacagaaatcaaccggcagcaactgcattggccaacaccatagaagtctttagatcgaatggcctaacagctaatgagtcaggaaggctaatagatttcttaaaggatgtaatggaatcaatgaacaaagaggaaatagagataacaacccactttcaaagaaaaaggagagtaagagacaacatgaccaagaagatggtcacgcaaagaacaatagggaagaaaaaacaaagactgaataagagaggctatctaataagagcactgacattaaatacgatgaccaaagatgcagagagaggcaagttaaaaagaagggctatcgcaacacctgggatgcagattagaggtttcgtatactttgttgaaactttagctagaagcatttgcgaaaagcttgaacagtctgggctcccagtagggggcaatgaaaagaaggccaaactggcaaatgttgtgagaaagatgatgactaattcacaagacacagagatttctttcacaatcactggggacaacactaaatggaatgaaaatcaaaatcctcgaatgttcctggcgatgattacatatatcaccagaaatcaacccgagtggttcagaaacatcttgagcatggcacccataatgttctcaaacaaaatggcaagactagggaaagggtacatgttcgagagtaaaagaatgaagattcgaacacaaataccagcagaaatgctagcaagcattgacctgaagtacttcaatgaatcaacaaagaagaaaattgagaaaataaggcctcttttaatagatggcacagcatcactgagtcctgggatgatgatgggcatgttcaacatgctaagtacggtcttgggagtctcgatactgaatcttggacaaaagaaatacaccaagacaatatactggtgggatgggctccaatcatccgacgattttgctctcatagtgaatgcaccaaaccatgagggaatacaagcaggagtggacagattctacaggacctgcaagttagtgggaatcaacatgagcaaaaagaagtcctatataaataagacagggacatttgaattcacaagctttttttatcgctatggatttgtggctaattttagcatggagctacccagctttggagtgtctggagtaaatgaatcagctgacatgagtattggagtaacagtgataaagaacaacatgataaacaatgaccttggacctgcaacggcccagatggctcttcaactgttcatcaaagactacagatacacatataggtgccataggggagacacacaaattcagacgagaagatcatttgagttaaagaagctgtgggatcaaacccaatcaaaggtagggctattagtatcagatggaggaccaaacttatacaatatacggaatcttcacattcctgaagtctgcttaaaatgggagctaatggatgatgattatcggggaagactttgtaatcccctgaatccctttgtcagtcataaagagattgattctgtaaacaatgctgtggtaatgccagcccatggtccagccaaaagcatggaatatgatgccgttgcaactacacattcctggattcccaagagaaatcgttctattctcaacacaagccaaaggggaattcttgaggatgaacagatgtaccagaagtgctgcaatctattcgagaaatttttccctagcagttcatataggagaccggttggaatttctagcatggtggaggccatggtgtctagggcccggattgatgccagggtcgacttcgagtctggacggatcgggaaggaagagttctctgagatcatgaagatctgttccaccattgaagaactcagacggcaaaaataa

>H1N1_PB1_CY066621_A/Athens/INS274/2009

atggatgtcaatccgactctacttttcctaaaaattccagcgcaaaatgccataagcaccacattcccttatactggagatcctccatacagccatggaacaggaacaggatacaccatggacacagtaaacagaacacaccaatactcagaaaagggaaagtggacgacaaacacagagactggtgcaccccagctcaacccgattgatggaccactacctgaggataatgaaccaagtgggtatgcacaaacagactgtgttctagaggctatggctttccttgaagaatcccacccaggaatatttgagaattcatgccttgaaacaatggaagttgttcaacaaacaagggtagataaactaactcaaggtcgccagacttacgattggacattaaacagaaatcaaccggcagcaactgcattggccaacaccatagaagtctttagatcgaatggcctaacagctaatgagtcaggaaggctaatagatttcttaaaggatgtaatggaatcaatgaacaaagaggaaatagagataacaacccactttcaaagaaaaaggagagtaagagacaacatgaccaagaagatggtcacgcaaagaacaatagggaagaaaaaacaaagactgaataagagaggctatctaataagagcactgacattaaatacgatgaccaaagatgcagagagaggcaagttaaaaagaagggctatcgcaacacctgggatgcagattagaggtttcgtatactttgttgaaactttagctaggagcatttgcgaaaagcttgaacagtctgggctcccagtagggggcaatgaaaagaaggccaaactggcaaatgttgtgagaaagatgatgactaattcacaagacacagagatttctttcacaatcactggggacaacactaagtggaatgaaaatcaaaatcctcgaatgttcctggcgatgattacatatatcaccagaaatcaacccgagtggttcagaaacatcctgagcatggcacccataatgttctcaaacaaaatggcaagactagggaaagggtacatgttcgagagtaaaagaatgaagattcgaacacaaataccagcagaaatgctagcaagcattgacctgaagtacttcaatgaatcaacaaagaagaaaattgagaaaataaggcctcttctaatagatggcacagcatcactgagtcctggaatgatgatgggcatgttcaacatgctaagtacggtattgggagtctcgatactgaatcttggacaaaagaaatacaccaagacaatatactggtgggatgggctccaatcatccgacgattttgctctcatagtgaatgcaccaaaccatgagggaatacaagcaggagtggacagattctacaggacctgcaagttagtgggaatcaacatgagcaaaaagaagtcctatataaataagacagggacatttgaattcacaagctttttttatcgctatggatttgtggctaattttagcatggagctacccagctttggagtgtctggagtaaatgaatcagctgacatgagtattggagtaacagtgataaagaacaacatgataaacaatgaccttggacctgcaacggcccagatggctcttcaattgttcatcaaagactacagatacacatataggtgccataggggagacacacaaattcagacgagaagatcatttgagttaaagaaactgtgggatcaaacccaatcaaaggtagggctattagtatcagatggaggaccaaacttatacaatatacggaatcttcacattcctgaagtctgcttaaaatgggagctaatggatgatgattatcggggaagactttgtaatcccctgaatccctttgtcagtcataaagagattgattctgtaaacaatgctgtggtaatgccagcccatggcccagccaaaagcatggaatatgatgctgttgcaactacacattcctggattcccaagaggaatcgttctattctcaacacaagccaaaggggaattcttgaggatgaacagatgtaccagaagtgctgcaatctattcgagaaatttttccctagcagttcatataggagaccggttggaatttctagcatggtggaggccatggtgtctagggcccggattgatgccagggtcgacttcgagtctggacggatcaagaaagaagagttctctgagatcatgaagatctgttccaccattgaagaactcagacggcaaaaataa

>H1N1_PB1_CY066661_A/Madrid/INS281/2009

atggatgtcaatccgactctacttttcctaaaaattccagcgcaaaatgccataagcaccacattcccttatactggagatcctccatacagccatggaacaggaacaggatacaccatggacacagtaaacagaacacaccaatactcagaaaagggaaagtggacgacaaacacagagactggtgcaccccagctcaacccgattgatggaccactacctgaggataatgaaccaagtgggtatgcacaaacagactgtgttctagaggctatggctttccttgaagaatcccacccaggaatatttgagaattcatgccttgaaacaatggaagttgttcaacaaacaagggtagataaactaactcaaggtcgccagacttatgattggacattaaacagaaatcaaccggcagcaactgcattggccaacaccatagaagtctttagatcgaatggcctaacagccaatgagtcaggaaggctaatagatttcttaaaggatgtaatggaatcaatgaacaaagaggaaatagagataacaacccactttcaaagaaaaaggagagtaagagacaacatgaccaagaagatggtcacgcaaagaacaatagggaagaaaaaacaaagactgaataagagaggctatctaataagagcactgacattaaatacgatgaccaaagatgcagagagaggcaagttaaaaagaagggctatcgcaacacctgggatgcagattagaggtttcgtatactttgttgaaactttagctaggagcatttgcgaaaagcttgaacagtctgggctcccagtagggggcaatgaaaagaaggccaaactggcaaatgttgtgagaaagatgatgactaattcacaagacacagagatttctttcacaatcactggggacaacactaagtggaatgaaaatcaaaatcctcgaatgttcctggcgatgattacatatatcaccagaaatcaacccgagtggttcagaaacatcctgagcatggcacccataatgttctcaaacaaaatggcaagactagggaaagggtacatgttcgagagtaaaagaatgaagattcgaacacaaataccagcagaaatgctagcaagcattgacctgaagtacttcaatgaatcaacaaagaagaaaattgagaaaataaggcctcttctaatagatggcacagcatcgctgagtcctgggatgatgatgggcatgttcaacatgctaagtacggtcttgggagtctcgatactgaatcttggacaaaagaaatacaccaagacaatatactggtgggatgggctccaatcatccgacgattttgctctcatagtgaatgcaccaaaccatgagggaatacaagcaggagtggacagattctacaggacctgcaagttagtgggaatcaacatgagcaaaaagaagtcctatataaataagacagggacatttgaattcacaagctttttttatcgctatggatttgtggctaattttagcatggagctacccagctttggagtgtctggagtaaatgaatcagctgacatgagtattggagtaacagtgataaagaacaacatgataaacaatgaccttggacctgcaacggcccagatggctcttcaattgttcatcaaagactacagatacacatataggtgccataggggagacacacaaattcagacgagaagatcatttgagttaaagaagctgtgggatcaaacccaatcaaaggtagggctattagtatcagatggaggaccaaacttatacaatatacggaatcttcacattcctgaagtctgcttaaaatgggagctaatggatgatgattatcggggaagactttgtaatcccctgaatccctttgtcagtcataaagagattgattctgtaaacaatgctgtggtaatgccaacccatggtccagccaaaagcatggaatatgatgccgttgcaactacacattcctggattcccaagaggaatcgttctattctcaacacaagccaaaggggaattcttgaggatgaacagatgtaccagaagtgctgcaatctattcgagaaatttttccctagcagttcatataggagaccggttggaatttctagcatggtggaggccatggtgtctagggcccggattgatgccagggtcgacttcgagtctggacggatcaagaaagaagagttctctgagatcatgaagatctgttccaccattgaagaactcagacggcaaaaataa

>H1N1_PB1_CY067005_A/Munich/INS246/2009

atggatgtcaatccgactctacttttcctaaaaattccagcgcaaaatgccataagcaccacattcccttatactggagatcctccatacagccatggaacaggaacaggatacaccatggacacagtaaacagaacacaccaatactcagaaaagggaaagtggacgacaaacacagagactggtgcaccccagctcaacccgattgatggaccactacctgaggataatgaaccaagtgggtatgcacaaacagactgtgttctagaggctatggctttccttgaagaatcccacccaggaatatttgagaattcatgccttgaaacaatggaagttgttcaacaaacaagggtagataaactaactcaaggtcgccagacttatgattggacattaaacagaaatcaaccggcagcaactgcattggccaacaccatagaagtctttagatcgaatggcctaacagctaatgagtcaggaaggctaatagatttcttaaaggatgtaatggaatcaatgaacaaagaggaaatagagataacaacccactttcaaagaaaaaggagagtaagagacaacatgaccaagaagatggtcacgcaaagaacaatagggaagaaaaaacaaagactgaataagagaggctatctaataagagcactgacattaaatacgatgaccaaagatgcagagagaggcaagttaaaaagaagggctatcgcaacacctgggatgcagattagaggtttcgtatactttgttgaaactttagctaggagcatttgcgaaaagcttgaacagtctgggctcccagtagggggcaatgaaaagaaggccaaactggcaaatgttgtgagaaagatgatgactaattcacaagacacagagatttctttcacaatcactggggacaacactaagtggaatgaaaatcaaaatcctcgaatgttcctggcgatgattacatatatcaccagaaatcaacccgagtggttcagaaacatcctgagcatggcacccataatgttctcaaacaaaatggcaagactagggaaagggtacatgttcgagagtaaaagaatgaagattcgaacacaaataccagcagaaatgctagcaagcattgacctgaagtacttcaatgaatcaacaaagaagaaaattgagaaaataaggcctcttctaatagatggcacagcatcactgagtcctgggatgatgatgggcatgttcaacatgctaagtacggtcttgggagtctcgatactgaatcttggacaaaagaaatacaccaagacaatatactggtgggatgggctccaatcatccgacgattttgctctcatagtgaatgcaccaaaccatgagggaatacaagcaggagtggacagattctacaggacctgcaagttagtgggaatcaacatgagcaaaaagaagtcctatataaataagacagggacatttgaattcacaagctttttttatcgctatggatttgtggctaattttagcatggagctacccagctttggagtgtctggagtaaatgaatcagctgacatgagtattggagtaacagtgataaagaacaacatgataaacaatgaccttggacctgcaacggcccagatggctcttcaattgttcatcaaagactacagatacacatatagatgccataggggagacacacaaattcagacgagaagatcatttgagttaaagaagctgtgggatcaaacccaatcaaaggtagggctattagtatcagatggaggaccaaacttatacaatatacggaatcttcacattcctgaagtctgcttaaaatgggagctaatggatgatgattatcggggaagactttgtaatcccctgaatccctttgtcagtcataaagagattgattctgtaaacaatgctgtggtaatgccagcccatggtccagccaaaagcatggaatatgatgccgttgcaactacacattcctggattcccaagaggaatcgttctattctcaacacaagccaaaggggaattcttgaggatgaacagatgtaccagaagtgctgcaatctattcgagaaatttttccctagcagttcatataggagaccggttggaatttctagcatggtggaggccatggtgtctagggcccggattgatgccagggttgacttcgagtctggacggatcaagaaagaagagttctctgagatcatgaagatctgttccaccattgaagaactcagacggcaaaaataa

>H1N1_PB1_CY067125_A/Athens/INS266/2009

atggatgtcaatccgactctacttttcctaaaaattccagcgcaaaatgccataagcaccacattcccttatactggagatcctccatacagccatggaacaggaacaggatacaccatggacacagtaaacagaacacaccaatactcagaaaagggaaagtggacgacaaacacagagactggtgcaccccagctcaacccgattgatggaccactacctgaggataatgaaccaagtgggtatgcacaaacagactgtgttctagaggctatggctttccttgaagaatcccacccaggaatatttgagaattcatgccttgaaacaatggaagttgttcaacaaacaagggtagataaactaactcaaggtcgccagacttatgattggacattaaacagaaatcaaccggcagcaactgcattggccaacaccatagaagtctttagatcgaatggcctaacagctaatgagtcaggaaggctaatagatttcttaaaggatgtaatggaatcaatgaacaaagaggaaatagagataacaacccactttcaaagaaaaaggagagtaagagacaacatgaccaagaagatggtcacgcaaagaacaatagggaagaaaaaacaaagactgaataagagaggctatctaataagagcactgacattaaatacgatgaccaaagatgcagagagaggcaagttaaaaagaagggctatcgcaacacctgggatgcagattagaggtttcgtatactttgttgaaactttagctaggagcatttgcgaaaagcttgaacaatctgggctcccagtagggggcaatgaaaagaaggccaaactggcaaatgttgtgagaaagatgatgactaattcacaagacacagagatttctttcacaatcactggggacaacactaagtggaatgaaaatcaaaatcctcgaatgttcctggcgatgattacatatatcaccagaaatcaacccgagtggttcagaaacatcctgagcatggcacccataatgttctcaaacaaaatggcaagactagggaaagggtacatgttcgagagtaaaagaatgaagattcgaacacaaataccagcagaaatgctagcaagcattgacctgaagtacttcaatgaatcaacaaagaagaaaattgagaaaataaggcctcttctaatagatggcacagcatcactgagtcctgggatgatgatgggcatgttcaacatgctaagtacggtcttgggagtctcgatactgaatcttggacaaaagaaatacaccaagacaatatactggtgggatgggctccaatcatccgacgattttgctctcatagtgaatgcaccaaaccatgagggaatacaagcaggagtggacagattctacaggacctgcaagttagtgggaatcaacatgagcaaaaagaagtcctatataaataagacagggacatttgaattcacaagctttttttatcgctatggatttgtggctaattttagcatggagctacccagctttggagtgtctggagtaaatgaatcagctgacatgagtattggagtaacagtgataaagaacaacatgataaacaatgaccttggacctgcaacggcccagatggctcttcaattgttcatcaaagactacagatacacatataggtgccataggggagacacacaaattcagacgagaagatcatttgagttaaagaagctgtgggatcaaacccaatcaaaggtagggctattagtatcagatggaggaccaaacttatacaatatacggaatcttcacattcctgaagtctgcttaaaatgggagctaatggatgatgattatcggggaagactttgtaatcccctgaatccctttgtcagtcataaagagattgattctgtaaacaatgctgtggcaatgccagcccatggtccagccaaaagcatggaatatgatgccgttgcaactacacattcctggattcccaagaggaatcgttctattctcaacacaagccaaaggggaattcttgaggatgaacagatgtaccagaagtgctgcaatctattcgagaaatttttccctagcagttcatataggagaccggttggaatttctagcatggtggaggccatggtgtctagggcccggattgatgccagggtcgacttcgagtctggacggatcaagaaagaagagttctctgagatcatgaagatctgttccaccattgaagaactcagacggcaaaaataa

>H1N1_PB1_CY068928_A/Guam/NHRC0004/2009

atggatgtcaatccgactctacttttcctaaaaattccagcgcaaaatgccataagcaccacattcccttatactggagatcctccatacagccatggaacaggaacaggttacaccatggacacagtaaacagaacacaccaatactcagaaaagggaaagtggacgacaaacacagagactggtgcaccccagctcaacccgattgatggaccactacctgaggatgatgaaccaagtgggtatgcacaaacagactgtgttctagaggctatggctttccttgaagaatcccacccaggaatatttgagaattcatgccttgaaacaatggaagttgttcaacaaacaagggtagataaactaactcaaggtcgccagacttatgattggacattaaacagaaatcaaccggcagcaactgcattggccaacaccatagaagtctttagatcgaatggcctaacagctaatgagtcaggaaggctaatagatttcttaaaggatgtaatggaatcaatgaacaaagaggaaatagagataacaacccactttcaaagaaaaaggagagtaagagacaacatgaccaagaagatggtcacacaaagaacaatagggaagaaaaaacaaagactgaataagagaggctatctaataagagcactgacattaaatacgatgaccaaagatgcagagagaggcaagttaaaaagaagggctatcgcaacacctgggatgcagattagaggtttcgtatactttgttgaaactttagctaggagcatttgcgaaaagcttgaacagtctgggctcccagtagggggcaatgaaaagaaggccaaactggcaaatgttgtgagaaagatgatgactaattcacaagacacagagatttctttcacaatcactggggacaacactaagtggaatgaaaatcaaaatcctcgaatgttcctggcgatgattacatatatcaccagaaatcaacccgagtggttcagaaacatcctgagcatggcacccataatgttctcaaacaaaatggcaagactagggaaagggtacatgttcgagagtaaaagaatgaagattcgaacacaaataccagcagaaatgctagcaagcattgacctgaagtacttcaatgaatcaacaaagaagaaaattgagaaaataaggcctcttctaatagatggcacagcatcactgagtcctgggatgatgatgggcatgttcaacatgctaagtacggtcttgggagtctcgatactgaatcttggacaaaagaaatacaccaagacaatatactggtgggatgggctccaatcatccgacgattttgctctcatagtgaatgcaccaaaccatgagggaatacaagcaggagtggacagattctacaggacctgcaagttagtggggatcaacatgagcaaaaagaagtcctatataaataagacagggacatttgaattcacaagctttttttatcgctatggatttgtggctaattttagcatggagctacccagctttggagtgtctggaataaatgaatcagctgacatgagtattggagtaacagtgataaagaacaacatgataaacaatgaccttggacctgcaacggcccagatggctcttcaattgttcatcaaagactacagatacacatataggtgccataggggagacacacaaattcagacgagaagatcatttgagttaaagaagctgtgggatcaaacccaatcaaaggtagggctattagtatcagatggaggaccaaacttatacaatatacggaatcttcacattcctgaagtctgcttaaaatgggagctaatggatgatgattatcggggaagactttgtaatcccctgaatccctttgtcagtcataaagagattgattctgtaaacaatgctgtggtaatgccagcccatggtccagccaaaagcatggaatatgatgccgttgcaactacacattcctggattcccaagaggaatcgttctattctcaacacaagccaaaggggaattcttgaggatgaacagatgtaccagaagtgttgcaatctattcgagaaatttttccctagcagttcatataggagaccggttggaatttctagcatggtggaggccatggtgtctagggcccggattgatgccagggtcgacttcgagtctggacggatcaagaaagaagagttctctgagatcatgaagatctgttccaccattgaagaactcagacggcaaaaataa

>H1N1_PB1_CY068992_A/Guam/NHRC0013/2009

atggatgtcaatccgactctacttttcctaaaaattccagcgcaaaatgccataagcaccacattcccttatactggagatcctccatacagccatggaacaggaacaggatacaccatggacacagtaaacagaacacaccaatactcagaaaagggaaagtggacgacaaacacagagactggtgcaccccagctcaacccgattgatggaccactacctgaggataatgaaccaagtgggtatgcacaaacagactgtgttctagaggctatggctttccttgaagaatcccacccaggaatatttgagaattcatgccttgaaacaatggaaattgttcaacaaacaagggtggataaactaactcaaggtcgccagacttatgattggacattaaacagaaatcaaccggcagcaactgcattggccaacaccatagaagtctttagatcgaatggcctaacagctaatgaatcaggaaggctaatagatttcttaaaggatgtaatggaatcaatgaacaaagaggaaatagagataacaacccactttcaaagaaaaaggagagtaagagacaacatgaccaagaagatggtcacgcaaagaacaatagggaagaaaaaacaaagactgaataagagaggctatctaataagagcactgacattaaatacgatgaccaaagatgcagagagaggcaagttaaaaagaagggctatcgcaacacctgggatgcagattagaggtttcgtatactttgttgaaactttagctaggagcatttgcgaaaagcttgaacaatctgggctcccagtagggggcaatgaaaagaaggccaaactggcaaatgttgtgagaaagatgatgactaattcacaagacacagagatttctttcacaatcactggggacaacactaagtggaatgaaaatcaaaatcctcgaatgttcctggcgatgattacatatatcaccagaaatcaacccgagtggttcagaaacatcctgagcatggcacccataatgttctcaaacaaaatggcaagactagggaaagggtacatgttcgagagtaaaagaatgaagattcgaacacaaataccagcagaaatgctagcaagcattgacctgaagtacttcaatgaatcaacaaagaaaaaaattgagaaaataaggcctcttctaatagatggcacagcatcactgagtccagggatgatgatgggcatgttcaacatgctaagtacggtcttgggagtctcgatactgaatcttggacaaaagaaatacaccaagacaatatactggtgggatgggctccaatcatccgacgattttgctctcatagtgaatgcaccaaaccatgagggaatacaagcaggagtggacagattctacaggacctgcaagttagtgggaatcaacatgagcaaaaagaagtcctatataaataagacagggacatttgaattcacaagctttttttatcgctatggatttgtggctaattttagcatggagctacccagctttggagtgtctggagtaaatgaatcagctgacatgagtattggagtaacagtgataaagaacaacatgataaacaatgaccttggacctgcaacggcccagatggctcttcaattgttcatcaaagactacagatacacatataggtgccataggggagacacacaaattcagacgagaagatcatttgagttaaagaagctgtgggatcaaacccaatcaaaggtagggctattagtatcagatggaggaccaaacttatacaatatacggaatcttcacattcctgaagtctgcttaaaatgggagctaatggatgatgattatcggggaagactttgtaatcccctgaatccctttgtcagtcataaagagattgattctgtaaacaatgctgtggtaatgccagcccatggtccagccaaaagcatggaatatgatgccgttgcaactacacattcctggattcccaagaggaatcgttctattctcaacacaagccaaaggggaattcttgaggatgaacagatgtaccagaagtgctgcaatctattcgagaaatttttccctagcagttcatataggagaccggttggaatttctagcatggtggaggccatggtgtctagggcccggattgatgccagggtcgacttcgagtctggacggatcaagaaagaagagttctctgagatcatgaagatctgttccaccattgaagaactcagacggcaaaaataa

>H1N1_PB1_CY069064_A/Guam/NHRC0022/2009

atggatgtcaatccgactctacttttcctaaaaattccagcgcaaaatgccataagcaccacattcccttatactggagatcctccatacagccatggaacaggaacaggatacaccatggacacagtaaacagaacacaccaatactcagaaaagggaaagtggacgacaaacacagagactggtgcaccccagctcaacccgattgatggaccactacctgaggataatgaaccaagtgggtatgcacaaacagactgtgttctagaggctatggctttccttgaagaatcccacccaggaatatttgagaattcatgccttgaaacaatggaagttgttcaacaaacaagggtagataaactaactcaaggtcgccagacttatgattggacattaaacagaaatcaaccggcagcaactgcattggccaacaccatagaagtctttagatcgaatggcctaacagctaatgagtcaggaaggctaatagatttcttaaaggatgtaatggaatcaatgaacaaagaggaaatagagataacaacccactttcaaagaaaaaggagagtaagagacaacatgaccaagaagatggtcacgcaaagaacaatagggaagaaaaaacaaagactgaataagagaggctatctaataagagcactgacattaaatacgatgaccaaagatgcagagagaggcaagttaaaaagaagggctatcgcaacacctgggatgcagattagaggtttcgtatactttgttgaaactttagctaggagcatttgcgaaaagcttgaacagtctgggctcccagtagggggcaatgaaaagaaggccaaactggcaaatgttgtgagaaagatgatgactaattcacaagacacagagatttctttcacaatcactggggacaacactaagtggaatgaaaatcaaaatcctcgaatgttcctggcgatgattacatatatcaccagaaatcaacccgagtggttcagaaacatcctgagcatggcacccataatgttctcaaacaaaatggcaagactagggaaagggtacatgttcgagagtaaaagaatgaagattcgaacacaaataccagcagaaatgctagcgagcattgacctgaagtacttcaatgaatcaacaaagaagaaaattgagaaaataaggcctcttctaatagatggcacagcatcactgagtcctgggatgatgatgggcatgttcaacatgctaagtacggtcttgggagtctcgatactgaatcttggacaaaagaaatacaccaagacaatatactggtgggatgggctccaatcatccgacgattttgctctcatagtgaatgcaccaaaccatgagggaatacaagcaggagtggacagattctacaggacctgcaagttagtgggaatcaacatgagcaaaaagaagtcctatataaataagacagggacatttgaattcacaagctttttttatcgctatggatttgtggctaattttagcatggagctacccagctttggagtgtctggagtaaatgaatcagctgacatgagtattggagtaacagtgataaagaacaacatgataaacaatgaccttggacctgcaacggcccagatggctcttcaattgttcatcaaagactacagatacacatataggtgccataggggagacacacaaattcagacgagaagatcatttgagttaaagaagctgtgggatcaaacccaatcaaaggtagggctattagtatcagatggaggaccaaacttatacaatatacggaatcttcacattcctgaagtctgcttaaaatgggagctaatggatgatgattatcggggaagactttgtaatcccctgaatccctttgtcagtcataaagagattgattctgtaaacaatgctgtggtaatgccagcccatggtccagccaaaagcatggaatatgatgccgttgcaactacacattcctggattcccaagaggaatcgttctattctcaacacaagccaaaggggaattcttgaggatgaacagatgtaccagaagtgctgcaatctattcgagaaatttttccctagcagttcatataggagaccggttggaatttctagcatggtggaggccatggtgtctagggcccggattgatgccagggtcgacttcgagtctggacggatcaagaaagaagagttctctgagatcatgaagatctgttccaccattgaagaactcagacggcaaaaataa

>H1N1_PB1_CY069483_A/Louisiana/WRAIR1170P/2009

atggatgtcaatccgacattacttttcttaaaagtgccagcacaaaatgctataagcacaacttttccttatactggtgaccctccttacagccatgggacaggaacagggtacaccatggatacagtcaataggacacaccagtactcagaaagaggaagatggacaaaaaataccgaaacgggagcaccgcaacttaacccaattgatggtcccttaccggaagacaatgaaccaagtggctatgcccaaacagattgtgtattagaagcaatggctttccttgaagaatcccatcccggtatctttgaaaactcttgtattgaaacaatggaggttgttcaacaaacaagggtggacaaactgacacaaggcagacagacctatgactggactctaaataggaaccagcctgctgccacagcattggcaaacactatagaagtattcagatcaaacggcctcatagcaaatgaatctggaaggctaatagacttccttaaagatgtaatggagtcgatggacagagacgaagtagaggtcacaactcattttcaaagaaagaggagagtgagagacaatgtaactaaaaaaatggtgacccaaagaacaataggcaaaaagaaacataaattagacaaaagaagttacctaattagggcattaaccctgaacacaatgaccaaagatgctgagagggggaaactaaaacgcagagcaattgcaaccccaggaatgcaaataagggggtttgtatactttgttgagacactggcaagaagcatatgtgaaaagcttgaacaatcaggattgccagttggaggaaatgagaagaaagcaaagttagcaaatgttgtaaggaagatgatgaccaactcccaggacactgaaatttctttcaccataaccggagacaacacaaaatggaacgaaaatcaaaaccctagaatgttcttggccatgatcacatatataaccaaaaatcagcctgaatggttcagaaatattctaagtattgctccaataatgttttcaaacaagatggcgagactaggtaaggggtacatgtttgaaagcaagagtatgaaactgagaactcaaatacctgcagagatgctagccaacatagacttgaaatatttcaatgattcaactaaaaagaaaattgaaaaaatccgaccattattaatagatggaactgcatcattgagtcctggaatgatgatgggcatgttcaatatgttgagcaccgtcttgggcgtctccattctgaatcttgggcaaaagagatacaccaagactacttactggtgggatggtcttcaatcatctgatgattttgctttgattgtgaacgcacccaactatgcaggaattcaagctggagttgacaggttctatcgaacctgtaagctgctcggaattaatatgagcaaaaagaaatcttacataaacagaacaggtacctttgaattcacgagctttttctatcgttatgggtttgttgccaatttcagcatggagcttcctagttttggggtgtctggggtcaatgaatctgcagacatgagtattggagtcactgtcatcaaaaacaatatgataaacaatgaccttggcccagcaactgctcaaatggcccttcagttatttataaaagattacaggtacacttatcgatgccaccgaggtgacacacaaatacaaacccgaagatcatttgagataaagaaactatgggaccaaacccgctcaaaagctggactgttggtctctgatggaggccccaatttgtataacattagaaatctccatattcctgaagtttgcttgaaatgggagttgatggatgaggattaccaggggcgtttatgcaacccattaaacccgtttgtcagccataaagagattgaatcagtgaacagtgcagtgataatgccggcacatggtccagccaaaaatatggagtatgacgctgttgcaacaacacactcctgggtccccaaaagaaatcgatccattttgaacacgagccaaagggggatacttgaagatgagcaaatgtatcagaggtgctgcaatttatttgaaaaattcttcccaagtagctcatacagaagaccagttggaatatccagtatggtagaggctatggtctcaagagcccgaattgatgcacggattgatttcgaatctggaaggataaagaaagaggaatttgctgagatcatgaagatctgttccaccattgaagacctcagacggcaaaaatga

>H1N1_PB1_CY069507_A/Japan/WRAIR1230P/2009

atggatgtcaatccgacattactcttcttaaaagtgccagcacaaaatgctataagcacaacttttccttatactggtgaccctccttacagccatgggacaggaacagggtacaccatggatacagtcaacaggacacaccagtactcagaaagaggaagatggacaaaaaataccgaaacgggagcaccgcaacttaacccaattgatggtcccttaccggaagacaatgaaccaagtggctatgcccaaacagattgtgtattagaagcaatggctttccttgaagaatcccatcccggtatctttgaaaactcttgtattgaaacaatggaggttgttcaacaaacaagggtggacaaactgacacaaggcagacagacctatgactggactctaaataggaaccagcctgctgccacagcattggcaaacactatagaagtattcagatcaaacggcctcatagcaaatgaatctgggaggctaatagacttccttaaagatgtaatggagtcgatggacagaggcgaagtagaggtcacaactcattttcaaagaaagaggagagtgagagacaatgtaactaaaaaaatggtgacccaaagaacaataggcaaaaagaaacataaattagacaaaagaagttacctaattagggcattaaccctgaacacaatgaccaaagatgctgagagggggaaactaaaacgcagagcaattgcaaccccaggaatgcaaataagggggtttgtatactttgttgagacactggcaagaagcatatgtgaaaagcttgaacaatcaggattgccagttggaggaaatgagaagaaagcaaagttagcaaatgttgtaaggaagatgatgaccaactcccaggacactgaaatttctttcaccataaccggagataacacaaaatggaacgaaaatcaaaaccctagaatgttcttggccatgatcacatatataaccaaaaatcagcctgaatggttcagaaatattctaagtattgctccaataatgttttcaaacaagatggcgagactaggcaaggggtacatgtttgaaagcaagagtatgaaactgagaactcaaatacctgcagagatgctagccaacatagacttgaaatatttcaatgattcaactaaaaagaaaattgaaaaaatccgaccattattaatagatggaactgcatcattgagtcctggaatgatgatgggcatgttcaatatgttgagcaccgtcttgggtgtctccattctgaatcttgggcaaaagagatacaccaagactacttactggtgggatggtcttcaatcgtctgatgattttgctttgattgtgaacgcacccaactatgcaggaattcaagctggagttgacaggttttatcgaacctgtaagctgctcggaattaatatgagcaaaaagaagtcttacataaacagaacaggtacctttgaattcacgagctttttctatcgttatgggtttgttgccaatttcagcatggagcttcctagttttggggtgtctggggtcaatgaatctgcagacatgagtattggagtcactgtcatcaaaaacaatatgataaacaatgaccttggcccagcaactgctcaaatggcccttcagttatttatcaaagattacaggtacacttatcgatgccaccgaggtgacacacaaatacaaacccggagatcatttgagataaagaaactatgggaccaaacccgctcaaaagctggactgttggtctctgatggaggccccaatttgtataacattagaaatctccatattcctgaagtttgcttgaaatgggagttgatggatgaggattaccaggggcgtttatgcaacccattaaacccttttgtcagccataaagagattgaatctgtgaacagtgcagtgataatgccggcacatggtccagccaaaaatatggagtatgacgctgttgcaacaacacactcctgggtccccaaaagaaatcgatccattttgaacacgagccaaagggggatacttgaagatgagcaaatgtatcagaggtgctgcaatttatttgaaaaattcttcccaagtagctcatacagaagaccagttggaatatccagtatggtagaggctatggtctcaagagcccgaattgatgcacggattgatttcgaatctggaaggataaagaaagaggaatttgctgagatcatgaagatctgttccaccattgaagacctcagacggcaaaaatga

>H1N1_PB1_CY070102_A/Peru/WRAIR1311P/2007

atggatgtcaatccgacattacttttcttaaaagtgccagcacaaaatgctataagcacaacttttccttatactggtgaccctccttacagccatgggacaggaacaggatacaccatggatacagtcaacaggacacaccagtactcagaaaggggaagatggacaaaaaataccgaaactggagcaccgcaacttaacccaattgatggtccactaccggaagacaatgaaccaagtggctatgcccaaacagattgtgtattagaagcaatggctttccttgaagaatcccatcccggtatctttgaaaactcttgtattgaaacaatagaggttgttcagcaaacaagggtggacaaactgacacaaggcagacagacctatgactggactctaaataggaaccagcctgctgccacagcattggcaaacactatagaagtattcagatcaaacggcctcatagcaaatgaatctgggaggctaatagacttccttaaagatgtaatggagtcgatggacagaggcgaagtagaggtcacaactcattttcaaagaaagaggagagtgagagacaatgtaactaaaaaaatggtgacccaaagaacaataggcaaaaagaaacataaattagacaaaagaagttacctaattagggcattaaccctgaacacaatgaccaaagatgctgagagggggaaactaaaacgcagagcaattgcaaccccaggaatgcaaataagggggtttgtatactttgttgagacactggcaagaagcatatgtgaaaagcttgaacaatcaggattgccagttggaggaaatgagaagaaagcaaagttagcaaatgttgtaaggaagatgatgaccaactcccaggacactgaaatttctttcaccataaccggagataacacaaaatggaacgaaaatcaaaaccctagaatgtttttggccatgatcacatatataaccaaaaatcagcctgaatggttcagaaatgttctaagtattgctccaataatgttttcaaacaagatggcgagactaggtaaggggtacatgtttgaaagcaagagtatgaaactgagaactcaaatacctgcagagatgctagctaacatagatttgaaatatttcaatgattcaactaaaaagaaaattgaaaaaatccgaccattattaatagatggaactgcatcattgagtcctggaatgatgatgggcatgttcaatatgttgagcaccgtcttgggcgtctccattctgaatcttgggcaaaagagatacaccaagactacttactggtgggatggtcttcaatcgtctgatgattttgctttgattgtgaatgcacccaactatgcaggaattcaagctggagttgacaggttttatcgaacctgtaagctgctcggaattaatatgagcaaaaagaagtcttacataaacagaacaggtacctttgaattcacgagctttttctatcgttatgggtttgttgccaatttcagcatggagctccctagttttggggtgtctgggatcaatgaatctgcagacatgagtattggagtcactgtcatcaaaaacaatatgataaacaatgaccttggcccagcaactgctcaaatggcccttcagttatttataaaagactacaggtacacttatcgatgccaccgaggtgacacacaaatacaaacccggagatcatttgagataaagaaactatgggaccaaacccgctcaaaagctgggctgttggtctctgatggaggccccaatttatataacattagaaacctccatattcctgaagtttgcttgaaatgggagttgatggatgaggattaccaggggcgtttatgcaacccattaaacccgtttgtcagccataaagagattgaatcagtgaacagtgcagtgataatgctggcacatggtccagccaaaaatatggagtatgacgctgttgcaacaacacactcctgggtccccaaaagaaatcgatccattttgaacacgagccaaagggggatacttgaagatgagcaaatgtatcagaggtgctgcaatttatttgaaaaattcttcccaagtagctcatacagaagaccagttggaatatccagtatggcagaggctatggtctcaagagcccgaattgatgcacggattgatttcgaatctggaaggataaagaaagaggaatttgctgagatcatgaagatctgttccaccattgaagacctcagacggcaaaaatga

>H1N1_PB1_CY071253_A/Madrid/INS407/2009

atggatgtcaatccgactctacttttcctaaaaattccagcgcaaaatgccataagcaccacattcccttatactggagatcctccatacagccatggaacaggaacaggatacaccatggacacagtaaacagaacacaccaatactcagaaaagggaaagtggacgacaaacacagagactggtgcaccccagctcaacccgattgatggaccactacctgaggataatgaaccaagtgggtatgcacaaacagactgtgttctagaggctatggctttccttgaagaatcccacccaggaatatttgagaattcatgccttgaaacaatggaagttgttcaacaaacaagggtagataaactaactcaaggtcgccagacttatgattggacattaaacagaaatcaaccggcagcaacagcattggccaacaccatagaagtctttagatcgaatggcctaacagctaatgagtcaggaaggctaatagatttcttaaaggatgtaatggaatcaatgaacaaagaggaaatagagataacaacccactttcaaagaaaaaggagagtaagagacaacatgaccaagaagatggtcacgcaaagaacaatagggaagaaaaaacaaagactgaataagagaggctatctaataagagcactgacattaaatacgatgaccaaagatgcagagagaggcaagttaaaaagaagggctatcgcaacacctgggatgcagattagaggtttcgtatactttgttgaaactttagctaggagcatttgcgaaaagcttgaacagtctgggctcccagtagggggcaatgaaaagaaggccaaactggcaaatgttgtgagaaagatgatgactaattcacaagacacagagatttctttcacaatcactggggacaacactaagtggaatgaaaatcaaaatcctcgaatgttcctggcgatgattacatatatcaccagaaatcaacccgagtggttcagaaacatcctgagcatggcacccataatgttctcaaacaaaatggcaagactagggaaagggtacatgttcgagagtaaaagaatgaagattcgaacacaaataccagcagaaatgctagcaagcattgacctgaagtacttcaatgaatcaacaaagaagaaaattgagaaaataaggcctcttctaatagatggcacagcatcactgagtcctgggatgatgatgggcatgttcaacatgctaagtacggtcttgggagtctcgatactgaatcttggacaaaagaaatacaccaagacaatatactggtgggatgggctccaatcatccgacgattttgctctcatagtgaatgcaccaaaccatgagggaatacaagcaggagtggacagattctacaggacctgcaagttagtgggaatcaacatgagcaaaaagaagtcctatataaataagacagggacatttgaattcacaagctttttttatcgctatggatttgtggctaattttagcatggagctacccagctttggagtgtctggagtaaatgaatcagctgacatgagtattggagtaacagtgataaagaacaacatgataaacaatgaccttggacctgcaacggcccagatggctcttcaattgttcatcaaagactacagatacacatataggtgccataggggagacacacaaattcagacgagaagatcatttgagttaaagaagctgtgggatcaaacccaatcaaaggtagggctattagtatcagatggaggaccaaacttatacaatatacggaatcttcacattcctgaagtctgcttaaaatgggagctaatggatgatgattatcggggaagactttgtaatcccctgaatccctttgtcagtcataaagagattgattctgtaaacaatgctgtggtaatgccagcccatggtccagccaaaagcatggaatatgatgccgttgcaactacacattcctggattcccaagaggaatcgttctattctcaacacaagccaaaggggaattcttgaggatgaacagatgtaccagaagtgctgcaatctattcgagaaatttttccctagcagttcatataggagaccggttggaatttctagcatggtggaggccatggtgtctagggcccggattgatgccagggtcgacttcgagtctggacggatcaagaaagaagagttctctgagatcatgaagatctgttccaccattgaagaactcagacggcaaaaataa

>H1N1_PB1_CY071285_A/Athens/INS413/2010

atggatgtcaatccgactctacttttcctaaaaattccagcgcaaaatgccataagcaccacattcccttatactggagatcctccatacagccatggaacaggaacaggatacaccatggacacagtaaacagaacacaccaatactcagaaaagggaaagtggacgacaaacacagagactggtgcaccccagctcaacccgattgatggaccactacctgaggataatgaaccaagtgggtatgcacaaacagactgtgttctagaggctatggctttccttgaagaatcccacccaggaatatttgagaattcatgccttgaaacaatggaagttgttcaacaaacaagggtagataaactaactcaaggtcgccagacttatgattggacattaaacagaaatcaaccggcagcaactgcattggccaacaccatagaagtctttagatcgaatggcctaacagctaatgagtcaggaaggctaatagatttcttaaaggatgtaatggaatcaatgaacaaagaggaaatagagataacaacccactttcaaagaaaaaggagagtaagagacaacatgaccaagaagatggtcacgcaaagaacaatagggaagaaaaaacaaagactgaataagagaggctatctaataagagcactgacattaaatacgatgaccaaagatgcagagagaggcaagttaaaaagaagggctatcgcaacacctgggatgcagattagaggtttcgtatactttgttgaaactttagctaggagcatttgcgaaaagcttgaacagtctgggctcccagtagggggcaatgaaaagaaggccaaactggcaaatgttgtgagaaagatgatgactaattcacaagacacagagatttctttcacaatcactggggacaacactaagtggaatgaaaatcaaaatcctcgaatgttcctggcgatgattacatacatcaccagaaatcaacccgagtggttcagaaacatcctgagcatggcacccataatgttctcaaacaaaatggcaagactagggaaagggtacatgttcgagagtaaaagaatgaagattcgaacacaaataccagcagaaatgctagcaagcattgacctgaagtacttcaatgaatcaacaaagaagaaaattgagaaaataaggcctcttctaatagatggcacagcatcactgagtcctgggatgatgatgggcatgttcaacatgctaagtacggtcttgggagtctcgatactgaatcttggacaaaagaaatacaccaagacaatatactggtgggatgggctccaatcatccgacgattttgctctcatagtgaatgcaccaaaccatgagggaatacaagcaggagtggacagattctacaggacctgcaagttagtgggaatcaacatgagcaaaaagaagtcctatataaataagacagggacatttgaattcacaagctttttttatcgctatgggtttgtggctaattttagcatggagctacccagctttggagtgtctggagtaaatgaatcagccgacatgagtattggagtaacagtgataaagaacaacatgataaacaatgaccttggacctgcaacggcccagatggctcttcaattgttcatcaaagactacagatacacatataggtgccataagggagacacacaaattcagacgagaagatcatttgagttaaagaaactgtgggatcaaacccaatcaaaggtagggctattagtatcagatggaggaccaaacttatacaatatacggaatcttcacattcctgaagtctgcttaaaatgggagctaatggatgatgattatcggggaagactttgtaatcccctgaatccctttgtcagtcataaagagattgattctgtaaacaatgctgtggtaatgccagcccatggtccagccaaaagcatggaatatgatgccgttgcaactacacattcctggattcccaagaggaatcgttctattctcaacacaagccaaaggggaattcttgaggatgaacagatgtaccagaagtgctgcaatctattcgagaaatttttccctagcagttcatataggagaccggttggaatttctagcatggtggaggccatggtgtctagggcccggattgatgccagggtcgacttcgagtctggacggatcaagaaagaagagttctctgagatcatgaagatctgttccaccattgaagaactcagacggcaaaaataa

>H1N1_PB1_CY072236_A/Frankfurt/INS303/2009

atggatgtcaatccgactctacttttcctaaaaattccagcgcaaaatgccataagcaccacattcccttatactggagatcctccatacagccatggaacaggaacaggatacaccatggacacagtaaacagaacacaccaatactcagaaaagggaaagtggacgacaaacacagagactggtgcaccccagctcaacccgattgatggaccactacctgaggataatgaaccaagtgggtatgcacaaacagactgtgttctagaggctatggctttccttgaagaatcccacccgggaatatttgagaattcatgccttgaaacaatggaagttgttcaacaaacaagggtagataaactaactcaaggtcgccagacttatgattggacattaaacagaaatcaaccggcagcaactgcattggccaacaccatagaagtctttagatcgaatggcctaacagctaatgagtcaggaaggctaatagatttcttaaaggatgtaatggaatcaatgaacaaagaggaaatagagataacaacccactttcaaagaaaaaggagagtaagagacaacatgaccaagaagatggtcacgcaaagaacaatagggaagaaaaaacaaagactgaataagagaggctatctaataagagcactgacattaaatacgatgaccaaagatgcagagagaggcaagttaaaaagaagggctatcgcaacacctgggatgcagattagaggtttcgtatactttgttgaaactttagctaggagcatttgcgaaaagcttgaacagtctgggctcccagtagggggcaatgaaaagaaggccaaactggcaaatgttgtgagaaagatgatgactaattcacaagacacagagatttctttcacaatcactggggacaacactaagtggaatgaaaatcaaaatcctcgaatgttcctggcgatgattacatatatcaccagaaatcaacccgagtggttcagaaacatcctgagcatggcacccataatgttctcaaacaaaatggcaagactagggagagggtacatgttcgagagtaaaagaatgaagattcgaacacaaataccagcagaaatgctagcaagcattgacctgaagtacttcaatgaatcaacaaagaagaaaattgagaaaataaggcctcttctaatagatggcacagcatcactgagtcctgggatgatgatgggcatgttcaacatgctaagtacagtcttgggagtctcgatactgaaccttggacaaaagaaatacaccaagacaatatactggtgggatgggctccaatcatccgacgattttgctctcatagtgaatgcaccaaaccatgaggggatacaagcaggagtggacagattctacaggacctgcaagttagtgggaatcaacatgagcaaaaagaagtcctatataaataaaacagggacatttgaattcacaagctttttttatcgctatggatttgtggctaattttagcatggagctacccagctttggagtgtctggagtaaatgaatcagcggacatgagtattggagtaacagtgataaagaacaacatgataaacaatgaccttggacctgcaacggcccagatggctcttcaattgttcatcaaagactacagatacacatacaggtgccataggggagacacacaaattcagacgagaagatcatttgagttaaagaagctgtgggatcaaacccaatcaaaggtagggctattagtatcagatggaggaccaaacttatacaatatacggaatcttcatattcctgaagtctgcttaaaatgggagctaatggatgatgattatcggggaagactttgtaatcccctgaatccctttgtcagtcataaagagattgattctgtaaacaatgctgtggtaatgccagcccatggtccagccaaaagcatggaatatgatgccgttgcaactacacattcctggattcccaagaggaatcgttctattctcaacacaagccaaaggggaattcttgaggatgaacagatgtaccagaagtgctgcaatctattcgataaatttttccctagcagttcatataggagaccggttggaatttctagcatggtggaggccatggtgtctagggcccggattgatgccagggtcgacttcgagtctggacggatcaagaaagaagagttctctgagatcatgaagatctgttccaccattgaagaactcagacggcaaaaataa

>H1N1_PB1_CY072292_A/Warsaw/INS313/2009

atggatgtcaatccgactctacttttcctaaaaattccagcgcaaaatgccataagcaccacattcccttatactggagatcctccatacagccatggaacaggaacaggatacaccatggacacagtaaacagaacacaccaatactcagaaaaaggaaagtggacgacaaacacagagactggtgcaccccagctcaacccgattgatggaccactacctgaggataatgaaccaagtgggtatgcacaaacagactgtgttctagaggctatggctttccttgaagaatcccacccaggaatatttgagaattcatgccttgaaacaatggaagttgttcaacaaacaagggtagataaactaactcaaggtcgccagacttatgattggacattaaacagaaatcaaccggcagcaactgcattggccaacaccatagaagtctttagatcgaatggcctaacagctaatgagtcaggaaggctaatagatttcttaaaggatgtaatggaatcaatgaacaaagaggaaatagagataacaacccactttcaaagaaaaaggagagtaagagacaacatgaccaaaaagatggtcacgcaaagaacaatagggaagaaaaaacaaagactgaataagagaggctatctaataagagcactgacattaaatacgatgaccaaagatgcagagagaggcaagttaaaaagaagggctatcgcaacacctgggatgcagattagaggtttcgtatactttgttgaaactttagctaggagcatttgcgaaaagcttgaacagtctgggctcccagtagggggcaatgaaaagaaggccaaactggcaaatgttgtgagaaagatgatgactaattcacaagacacagagatttctttcacaatcactggggacaacactaagtggaatgaaaatcaaaatcctcgaatgttcctggcgatgattacatatatcaccagaaatcaacccgagtggttcagaaacatcctgagcattgcacccataatgttctcaaacaaaatggcaagactagggaaagggtacatgttcgagagtaaaagaatgaagattcgaacacaaataccagcagaaatgctagcaagcattgacctgaagtacttcaatgaatcaacaaagaagaaaattgagaaaataaggcctcttctaatagatggcacagcatcactgagtcctgggatgatgatgggcatgttcaacatgctaagtacggtcttgggagtctcgatactgaatcttggacaaaagaaatacaccaagacaatatactggtgggatgggctccaatcatccgacgattttgctctcatagtgaatgcaccaaaccatgagggaatacaagcaggagtggacagattctacaggacctgcaagttagtgggaatcaacatgagcaaaaagaagtcctatataaataagacagggacatttgaattcacaagctttttttatcgctatggatttgtggctaattttagcatggagctacccagctttggagtgtctggagtaaatgaatcagctgacatgagtattggagtaacagtgataaagaacaacatgataaacaatgaccttggacctgcaacggcccagatggctcttcaattgttcatcaaagactacagatacacatataggtgccataggggagacacacaaattcagacgagaagatcatttgagttaaagaagctgtgggatcaaacccaatcaaaggtagggctattagtatcagatggaggaccaaacttatacaatatacggaatcttcacattcctgaagtctgcttaaaatgggagctaatggatgatgattatcggggaagactttgtaatcccctgaatccctttgtcagtcataaagagattgattctgtaaacaatgctgtggtaatgccagcccatggtccagccaaaagcatggaatatgatgccgttgcaactacacattcctggattcccaagaggaatcgttctattctcaacacaagccaaaggggaattcttgaggatgaacagatgtaccagaagtgctgcaatctattcgagaaatttttccctagcagttcatataggagaccggttggaatttctagcatggtggaggccatggtgtctagggcccggattgatgccagggtcgacttcgagtctggacggatcaagaaagaagagttctctgagatcatgaagatctgttccaccattgaagaactcagacggcaaaaataa

>H1N1_PB1_CY072572_A/Managua/4197.01/2009

atggatgtcaatccgactctacttttcctaaaaattccagcgcaaaatgccataagcaccacattcccttatactggagatcctccatacagccatggaacaggaacaggatacaccatggacacagtaaacagaacacaccaatactcagaaaagggaaagtggacgacaaacacagagactggtgcaccccagctcaacccgattgatggaccactacctgaggataatgaaccaagtgggtatgcacaaacagactgtgttctagaggctatggctttccttgaagaatcccacccgggaatatttgagaattcatgccttgaaacaatggaagttgttcaacaaacaagggtagataaactaactcaaggtcgccagacttatgattggacattaaacagaaatcaaccggcagcaactgcattggccaacaccatagaagtctttagatcgaatggcctaacagctaatgaatcaggaaggctaatagatttcttaaaggatgtaatggaatcaatgaacaaagaggaaatagagataacaacccactttcaaagaaaaaggagagtaagagacaacatgaccaagaagatggtcacgcaaagaacaatagggaagaaaaaacaaagactgaataagagaggctatctaataagagcactgacattaaatacgatgaccaaagatgcagagagaggcaagttaaaaagaagggctatcgcaacacctgggatgcagattagaggtttcgtatactttgttgaaactttagctaggagcatttgcgaaaagcttgaacagtctgggctcccagtagggggcaatgaaaagaaggccaaactggcaaatgttgtgagaaagatgatgactaattcacaagacacagagatttctttcacaatcactggggacaacactaagtggaatgaaaatcaaaatcctcgaatgttcctggcgatgattacatatatcaccagaaatcaacccgagtggttcagaaacatcctgagcatggcacccataatgttctcaaacaaaatggcaagactagggagagggtacatgttcgagagtaaaagaatgaagattcgaacacaaataccagcagaaatgctagcaagcattgacctgaagtacttcaatgaatcaacaaagaagaaaattgagaaaataaggcctcttctaatagatggcacagcatcactgagtcctgggatgatgatgggcatgttcaacatgctaagtacggtcttgggagtctcgatactgaatctcggacaaaagaaatacaccaagacaatatactggtgggatgggctccaatcatccgacgattttgctctcatagtgaatgcaccaaaccatgagggaatacaagcaggagtggacagattctacaggacctgcaagttagtgggaatcaacatgagcaaaaagaagtcctatataaataagacagggacatttgaattcacaagctttttttatcgctatggatttgtggctaattttagcatggagctacccagctttggagtgtctggagtaaatgaatcagctgacatgagtattggagtaacagtgataaagaacaacatgataaacaatgaccttggacctgcaacggcccagatggctcttcaattgttcatcaaagactacagatacacatataggtgccataggggagacacacaaattcagacgagaagatcatttgagttaaagaagctgtgggatcaaacccaatcaaaggtagggctattagtatcagatggaggaccaaacttatacaatatacggaatcttcacattcctgaagtctgcttaaaatgggagctaatggatgatgattatcggggaagactttgtaatcccctgaatccctttgtcagtcataaagagattgattctgtaaacaatgctgtggtaatgccagcccatggtccagccaaaagcatggaatatgatgccgttgcaactacacattcctggattcccaagaggaatcgttctattctcaacacaagccaaaggggaattcttgaggatgaacagatgtaccagaagtgctgcaatctattcgagaaatttttccctagcagttcatataggagaccggttggaatttctagcatggtggaggccatggtgtctagggcccggattgatgccagggtcgacttcgagtctggacggatcaagaaagaagagttctctgagatcatgaagatctgttccaccattgaagaactcagacggcaaaaataa

>H1N1_PB1_CY072796_A/Managua/1984.02/2009

atggatgtcaatccgactctacttttcctaaaaattccagcgcaaaatgccataagcaccacattcccttatactggagatcctccatacagccatggaacaggaacaggatacaccatggacacagtaaatagaacacaccaatactcagaaaagggaaagtggacgacaaacacagagactggtgcaccccagctcaacccgattgatggaccactacctgaggataatgaaccaattgggtatgcacaaacagactgtgttctagaggctatggctttccttgaagaatcccacccaggaatatttgagaattcatgccttgaaacaatggaagttgttcaacaaacaagagtagataaactaactcaaggtcgccagacttatgattggacattaaacagaaatcaaccggcagcaactgcattggccaacaccatagaagtctttagatcgaatggcctaacagctaatgagtcaggaaggctaatagatttcttaaaggatgtaatggaatcaatgaacaaagaggaaatagagataacaacccactttcaaagaaaaaggagagtaagagacaacatgaccaagaagatggtcacgcaaagaacaatagggaagaaaaaacaaagactgaataagagaggctatctaataagagcactgacattaaatacgatgaccaaagatgcagagagaggcaagttaaaaagaagggctatcgcaacacctgggatgcagattagaggtttcgtatactttgttgaaactttagctaggagcatttgcgaaaagcttgaacagtctgggctcccagtagggggcaatgaaaagaaggccaaactggcaaatgttgtgagaaagatgatgactaattcacaagacacagagatttctttcacaatcactggggacaacactaagtggaatgaaaatcaaaatcctcgaatgttcctggcgatgattacatatatcaccagaaatcaacccgagtggttcagaaacatcctgagcatggcacccataatgttctcaaacaaaatggcaagactagggaaagggtacatgttcgagagtaaaagaatgaagattcgaacacaaataccagcagaaatgctagcaagcattgacctgaagtacttcaatgaatcaacaaagaagaaaattgagaaaataaggcctcttctaatagatggcacagcatcactgagtcctgggatgatgatgggcatgttcaacatgctaagtacggtcttgggagtctcgatactgaatcttggacaaaagaaatacaccaagacaatatactggtgggatgggctccaatcatccgacgattttgctctcatagtgaatgcaccaaaccatgagggaatacaagcaggagtggacagattctacaggacctgcaagttagtgggaatcaacatgagcaaaaagaagtcctatataaataagacagggacatttgaattcacaagctttttttatcgctatggatttgtggctaattttagcatggagctacccagctttggagtgtctggagtaaatgaatcagctgacatgagtattggagtaacagtgataaagaacaacatgataaacaatgaccttggacctgcaacggcccagatggctcttcaattgttcatcaaagactacagatacacatataggtgccataggggagacacacaaattcagacgagaagatcatttgagttaaagaagctgtgggatcaaacccaatcaaaggtagggctattagtatcagatggaggaccaaacttatacaatatacggaatcttcacattcctgaagtctgcttaaaatgggagctaatggatgatgattatcggggaagactttgtaatcccctgaatccctttgtcagtcataaagagattgattctgtaaacaatgctgtggtaatgccagctcatggtccagccaaaagcatggaatatgatgccgttgcaactacacattcctggattcccaagaggaatcgttctattctcaacacaagccaaaggggaattcttgaggatgaacagatgtaccagaagtgctgcaatctattcgagaaatttttccctagcagttcatataggagaccggttggaatttctagcatggtggaggccatggtgtctagggcccggattgatgccagggtcgacttcgagtctggacggatcaagaaagaagagttctctgagatcatgaagatctgttccaccattgaagaactcagacggcaaaaataa

>H1N1_PB1_CY072924_A/Managua/4972.02/2009

atggatgtcaatccgactctacttttcctaaaaattccagcgcaaaatgccataagcaccacattcccttatactggagatcctccatacagccatggaacaggaacaggatacaccatggacacagtaaacagaacacaccaatactcagaaaagggaaagtggacgacaaacacagagactggtgcaccccagctcaacccgattgatggaccactacctgaggataatgaaccaagtgggtatgcacaaacagactgtgttctagaggctatggctttccttgaagaatcccacccaggaatatttgagaattcatgccttgaaacaatggaagttgttcaacaaacaagggtagataaactaactcaaggtcgccagacttatgattggacattaaacagaaatcaaccggcagcaactgcattggccaacaccatagaagtctttagatcgaatggcctaacagctaatgagtcaggaaggctaatagatttcttaaaggatgtaatggaatcaatgaacaaagaggaaatagagataacaacccactttcaaagaaaaaggagagtaagagacaacatgaccaagaagatggtcacgcaaagaacaatagggaagaaaaaacaaagactgaataagagaggctatctaataagagcactgacattaaatacgatgaccaaagatgcagagagaggcaagttaaaaagaagggctatcgcaacacctgggatgcagattagaggtttcgtatactttgttgaaactttagctaggagcatttgcgaaaagcttgaacagtctgggctcccagtagggggcaatgaaaagaaggccaaactggcaaatgttgtgagaaagatgatgactaattcacaagacacagagatttctttcacaatcactggggacaacactaagtggaatgaaaatcaaaatcctcgaatgttcctggcgatgattacatatatcaccagaaatcaacccgagtggttcagaaacatcctgagcatggcacccataatgttctcaaacaaaatggcaagactagggaaagggtacatgttcgagagtaaaagaatgaagattcgaacacaaataccagcagaaatgctagcaagcattgacctgaagtacttcaatgaatcaacaaagaagaaaattgagaaaataaggcctcttctaatagatggcacagcatcactgagtcctgggatgatgatgggcatgttcaacatgctaagtacggtcttgggagtctcgatactgaatcttggacaaaagaaatacaccaagacaatatactggtgggatgggctccaatcatccgacgattttgctctcatagtgaatgcaccaaaccatgagggaatacaagcaggagtggacagattctacaggacctgcaagttagtgggaatcaacatgagcaaaaagaagtcctatataaataagacagggacatttgaattcacaagctttttttatcgctatggatttgtggctaattttagcatggagctacccagctttggagtgtctggagtaaatgaatcagctgacatgagtattggagtaacagtgataaagaacaacatgataaacaatgaccttggacctgcaacggcccagatggctcttcaattgttcatcaaagactacagatacacatataggtgccataggggggacacacaaattcagacgagaagatcatttgagttaaagaagctgtgggatcaaacccaatcaaaggtagggctattagtatcagatggaggaccaaacttatacaatatacggaatcttcacattcctgaagtctgcttaaaatgggagctaatggatgatgattatcggggaagactttgtaatcccctgaatccctttgtcagtcataaagagattgattctgtaaacaatgctgtggtaatgccagcccatggtccagccaaaagcatggaatatgatgccgttgcaactacacattcctggattcccaagaggaatcgttctattctcaacacaagccaaaggggaattcttgaggatgaacagatgtaccagaagtgctgcaatctattcgagaaatttttccctagcagttcatataggagaccggttggaatttctagcatggtggaggccatggtgtctagagcccggattgatgccagggtcgacttcgagtctggacggatcaagaaagaagagttctctgagatcatgaagatctgttccaccattgaagaactcagacggcaaaaataa

>H1N1_PB1_CY073012_A/Athens/INS330/2009

atggatgtcaatccgactctacttttcctaaaaattccagcgcaaaatgccataagcaccacattcccttatactggagatcctccatacagccatggaacaggaacaggatacaccatggacacagtaaacagaacacaccaatactcagaaaagggaaagtggacgacaaacacagagactggtgcaccccagctcaacccgattgatggaccactacctgaggataatgaaccaagtgggtatgcacaaacagactgtgttctagaggctatggctttccttgaagaatcccacccaggaatatttgagaattcatgccttgaaacaatggaagttgttcaacaaacaagggtagataaactaactcaaggtcgccagacttatgattggacattaaacagaaatcaaccggcagcaactgcattggccaacaccatagaagtctttagatcgaatggcctaacagctaatgagtcaggaaggctaatagatttcttaaaggatgtaatggaatcaatgaacaaagaggaaatagagataacaacccactttcaaagaaaaaggagagtaagagacaacatgaccaagaagatggtcacgcaaagaacaatagggaagaaaaaacaaagactgaataagagaggctatctaataagagcactgacattaaatacgatgaccaaagatgcagagagaggcaagttaaaaagaagggctatcgcaacacctgggatgcagattagaggtttcgtatactttgttgaaactttagctaggagcatttgcgaaaagcttgaacaatctgggctcccagtagggggcaatgaaaagaaggccaaactggcaaatgttgtgagaaagatgatgactaattcacaagacacagagatttctttcacaatcactggggacaacactaagtggaatgaaaatcaaaatcctcgaatgttcctggcgatgattacatatatcaccagaaatcaacccgagtggttcagaaacatcctgagcatggcacccataatgttctcaaataaaatggcaagactagggaaagggtacatgttcgagagtaaaagaatgaagattcgaacacaaataccagcagaaatgctagcaagcattgacctgaagtacttcaatgaatcaacaaagaagaaaattgagaaaataaggcctcttctaatagatggcacagcatcactgagtcctgggatgatgatgggcatgttcaacatgctaagtacggtcttgggagtctcgatactgaatcttggacaaaagaaatacaccaagacaatatactggtgggatgggctccaatcatccgacgattttgctctcatagtgaatgcaccaaaccatgagggaatacaagcaggagtggacagattctacaggacctgcaagttagtgggaatcaacatgagcaaaaagaagtcctatataaataagacagggacatttgaattcacaagctttttttatcgctatggatttgtggctaattttagcatggagctacccagctttggagtgtctggagtaaatgaatcagctgacatgagtattggagtaacagtgataaagaacaacatgataaacaatgaccttggacctgcaacggcccagatggctcttcaattgttcatcaaagactacagatacacatataggtgccataggggagacacacaaattcagacgagaagatcatttgagttaaagaagctgtgggatcaaacccaatcaaaggtagggctattagtatcagatggaggaccaaacttatacaatatacggaatcttcacattcctgaagtctgcttaaaatgggagctaatggatgatgattatcggggaagactttgtaatcccctgaatccctttgtcagtcataaagagattgattctgtaaacaatgctgtggcaatgccagcccatggtccagccaaaagcatggaatatgatgccgttgcaactacacattcctggattcccaagaggaatcgttctattctcaacacaagccaaaggggaattcttgaggatgaacagatgtaccagaagtgctgcaatctattcgagaaatttttccctagcagttcatataggagaccggttggaatttctagcatggtggaggccatggtgtctagggcccggattgatgccagggtcgacttcgagtctggacggatcaagaaagaagagttctctgagatcatgaagatctgttccaccattgaagaactcagacggcaaaaataa

>H1N1_PB1_CY073132_A/Mexico City/WRAIR1779T/2009

atggatgtcaatccgactctacttttcctaaaaattccagcgcaaaatgccataagcaccacattcccttatactggagatcctccatacagccatggaacaggaacaggatacaccatggacacagtaaacagaacacaccaatactcagaaaagggaaagtggacgacaaacacagagactggtgcaccccagctcaacccgattgatggaccactacctgaggataatgaaccaagtgggtatgcacaaacagactgtgtcctggaggctatggctttccttgaagaatcccacccaggaatatttgagaattcatgccttgaaacaatggaagttgttcaacaaacaagggtagataaactaactcaaggtcgccagacttatgattggacattaaacagaaatcaaccggcagcaactgcattggccaacaccatagaagtctttagatcgaatggcctaacagctaatgagtcaggaaggctaatagatttcttaaaggatgtaatggaatcaatgaacaaagaggaaatagagataacaacccactttcaaagaaaaaggagagtaagagacaacatgaccaagaagatggtcacgcaaagaacaatagggaagaaaaaacaaagactgaataagagaggctatctaataagagcactgacattaaatacgatgaccaaagatgcagagagaggcaagttaaaaagaagagctatcgcaacacctgggatgcagattagaggtttcgtatactttgttgaaactttagctaggagcatttgcgaaaagcttgaacagtctgggctcccagtagggggcaatgaaaagaaggccaaactggcaaatgttgtgagaaagatgatgactaattcacaagacacagagatttctttcacaatcactggggacaacactaagtggaatgaaaatcaaaatcctcgaatgttcctggcgatgattacatatatcaccagaaatcaacccgagtggttcagaaacatcctgagcatggcacccataatgttctcaaacaaaatggcaagactagggaaagggtacatgttcgagagtaaaagaatgaagattcgaacacaaataccagcagaaatgctagcaagcattgacctgaagtacttcaatgaatcaacaaagaagaaaattgagaaaataaggcctcttctaatagatggcacagcatcactgagtcctgggatgatgatgggcatgttcaacatgctaagtacggtcttgggagtctcgatactgaatcttggacaaaagaaatacaccaagacaatatactggtgggatgggctccaatcatccgacgattttgctctcatagtgaatgcaccaaaccatgagggaatacaagcaggagtggacagattctacaggacctgcaagttagtgggaatcaacatgagcaaaaagaagtcctatataaataagacagggacatttgaattcacaagctttttttatcgctatggatttgtggctaattttagcatggagctacccagctttggagtgtctggagtaaatgaatcagctgacatgagtattggagtaacagtgataaagaacaacatgataaacaatgaccttggacctgcaacggcccagatggctcttcaattgttcatcaaagactacagatacacatataggtgccataggggagacacacaaattcagacgagaagatcatttgagttaaagaagctgtgggatcaaacccaatcaaagggagggctattagtatcagatggaggaccaaacttatacaatatacggaatcttcacattcctgaagtctgcttaaaatgggagctaatggatgatgattatcggggaagactttgtaatcccctgaatccctttgtcagtcataaagagattgattctgtaaacaatgctgtggtaatgccagcccatggtccagtcaaaagcatggaatatgatgccgttgcaactacacattcctggattcccaagaggaatcgttctattctcaacacaagccaaaggggaattcttgaggatgaacagatgtaccagaagtgctgcaatctattcgagaaatttttccctagcagttcatataggagacctgttggaatttctagcatggtggaggccatggtgtctagggcccggattgatgccagggttgacttcgagtctggacggatcaagaaagaagagttctctgagatcatgaagatctgctccaccattgaagaactcagacggcaaaaataa

>H1N1_PB1_CY073156_A/Lagos/WRAIR1982N/2009

atggatgtcaatccgactctacttttcctaaaaattccagcgcaaaatgccataagcaccacattcccttatactggagatcctccatacagccatggaacaggaacaggatacaccatggacacagtaaacagaacacaccaatactcagaaaagggaaagtggacgacaaacacagagactggtgcaccccagctcaacccgattgatggaccactacctgaggataatgaaccaagtgggtatgcacaaacagactgtgtcctggaggctatggctttccttgaagaatcccacccaggaatatttgagaattcatgccttgaaacaatggaagttgttcaacaaacaagggtagataaactaactcaaggtcgccagacttatgattggacattaaacagaaatcaaccggcagcaactgcattggccaacaccatagaagtctttagatcgaatggcctaacagctaatgagtcaggaaggctaatagatttcttaaaggatgtaatggaatcaatgaacaaagaggaaatagagataacaacccactttcaaagaaaaaggagagtaagagacaacatgaccaagaagatggtcacgcaaagaacaatagggaagaaaaaacaaagactgaataagagaggctatctaataagagcactgacattaaatacgatgaccaaagatgcagagagaggcaagttaaaaagaagggctatcgcaacacctgggatgcagattagaggtttcgtatactttgttgaaactttagctaggagcatttgcgaaaagcttgaacagtctgggctcccagtagggggcaatgaaaagaaagccaaattggcaaatgtggtgagaaagatgatgactaattcacaagacacagagatttctttcacaatcactggggacaacactaagtggaatgaaaatcaaaatcctcgaatgttcctggcgatgattacatatatcaccagaaatcaacccgagtggttcagaaacatcctgagcatggcacccataatgttctcaaacaaaatggcaagactagggaaagggtacatgttcgagagtaaaagaatgaagattcgaacacaaataccagcagaaatgctagcaagcattgacctgaaatacttcaatgaatcaacaaagaagaaaattgagaaaataaggcctcttctaatagatggcacagcatcactgagtcctgggatgatgatgggcatgttcaacatgctaagtacggtcttgggagtctcgatactgaatcttggacaaaagaaatacaccaagacaatatattggtgggatgggctccaatcatccgacgattttgctctcatagtgaatgcaccaaaccatgagggaatacaagcaggagtggacagattctacaggacctgcaagttagtgggaatcaacatgagcaaaaagaagtcctatataaataagacagggacatttgaattcacaagctttttttatcgctatggatttgtggctaattttagcatggagctacccagctttggagtgtctggagtaaatgaatcagctgacatgagtattggagtaacagtgataaagaacaacatgataaacaatgaccttggacctgcaacggcccagatggctcttcaattgttcatcaaagactacagatacacatataggtgccataggggggacacacaaattcagacgagaagatcatttgagttaaagaagctgtgggatcaaacccaatcaaaggtagggctattagtatcagatggaggaccaaacttatacaatatacggaatcttcacattcctgaagtctgcttaaaatgggagctaatggatgatgattatcggggaagactttgtaatcccctgaatccctttgtcagtcataaagagattgattctgtaaacaatgctgtggtaatgccagcccatggcccagccaaaagcatggaatatgatgccgttgcaactacacattcctggattcccaagaggaatcgttctattctcaacacaagccaaaggggaattcttgaggatgaacagatgtaccagaagtgctgcaatctattcgagaaatttttccctagcagttcatataggagaccggttggaatttctagcatggtggaggccatggtgtctagggcccggattgatgccagggtcgacttcgagtctggacggatcaagaaagaagagttctctgagatcatgaagatctgttccaccattgaagaactcagacggcaaaaataa

>H1N1_PB1_CY073575_A/Managua/5230.01/2009

atggatgtcaatccgactctacttttcctaaaaattccagcgcaaaatgccataagcaccacattcccttatactggagatcctccatacagccatggaacaggaacaggatacaccatggacacagtaaacagaacacaccaatactcagaaaagggaaagtggacgacaaacacagagactggtgcaccccagctcaacccgattgatggaccactacctgaggataatgaaccaagtgggtatgcacaaacagactgtgttctagaggctatggctttccttgaagaatcccacccgggaatatttgagaattcatgccttgaaacaatggaagttgttcaacaaacaagggtagataaactaactcaaggtcgccagacttatgattggacattaaacagaaatcaaccggcagcaactgcattggccaacaccatagaagtctttagatcgaatggcctaacagctaatgagtcaggaaggctaatagatttcttaaaggatgtaatggaatcaatgaacaaagaggaaatagagataacaacccactttcaaagaaaaaggagagtaagagataacatgaccaagaagatggtcacgcaaagaacaatagggaagaaaaaacaaagactgaataagagaggctatctaataagagcactgacattaaatacgatgaccaaagatgcagagagaggcaagttaaaaagaagggctatcgcaacacctgggatgcagattagaggtttcgtatactttgttgaaactttagctaggagcatttgcgaaaagcttgaacagtctgggctcccagtagggggcaatgaaaagaaggccaaactggcaaatgttgtgagaaagatgatgactaattcacaagacacagagatttctttcacaatcactggggacaacactaagtggaatgaaaatcaaaatcctcgaatgttcctggcgatgattacatatatcaccagaaatcaacccgagtggttcagaaacatcctgagcatggcacccataatgttctcaaacaaaatggcaagactagggagagggtacatgttcgagagtaaaagaatgaagattcgaacacaaataccagcagaaatgctagcaagcattgacctgaagtacttcaatgaatcaacaaagaagaaaattgagaaaataaggcctcttctaatagatggcacagcatcactgagtcctgggatgatgatgggcatgttcaacatgctaagtacggtcttgggagtctcgatactgaatctcggacaaaagaaatacaccaagacaatatactggtgggatgggctccaatcatccgacgattttgctcttatagtgaatgcaccaaaccatgagggaatacaagcaggagtggacagattctacaggacctgcaagttagtgggaatcaacatgagcaaaaagaagtcctatataaataagacagggacatttgaattcacaagctttttttatcgctatggatttgtggctaattttagcatggagctacccagctttggagtgtctggagtaaatgaatcagctgacatgagtattggagtaacagtgataaagaacaacatgataaacaatgaccttggacctgcaacggcccagatggctcttcaattgttcatcaaagactacagatacacatataggtgccataggggagacacacaaattcagacgagaagatcatttgagttaaagaagctgtgggatcaaacccaatcaaaggtagggctattagtatcagatggaggaccaaacttatacaatatacggaatcttcacattcctgaagtctgcttaaaatgggagctaatggatgatgattatcggggaagactttgtaatcccctgaatccctttgtcagtcataaagagattgattctgtaaacaatgctgtggtaatgccagcccatggtccagccaaaagcatggaatatgatgccgttgcaactacacattcctggattcccaagaggaatcgttctattctcaacacaagccaaaggggaattcttgaggatgaacagatgtaccagaagtgctgcaatctattcgagaaatttttccctagcagttcatataggagaccggttggaatttctagcatggtggaggccatggtgtctagggcccggattgatgccagggtcgacttcgagtctggacggatcaagaaagaagagttctctgagatcatgaagatctgttccaccattgaagaactcagacggcaaaaataa

>H1N1_PB1_CY073631_A/Managua/5595.03/2009

atggatgtcaatccgactctacttttcctaaaaattccagcgcaaaatgccataagcaccacattcccttatactggagatcctccatacagccatggaacaggaacaggatacaccatggacacagtaaacagaacacaccaatactcagaaaagggaaagtggacgacaaacacagagactggtgcaccccagctcaacccgattgatggaccactacctgaggataatgaaccaagtgggtatgcacaaacagactgtgttctagaggctatggctttccttgaagaatcccacccgggaatatttgagaattcatgccttgaaacaatggaagttgttcaacaaacaagggtagataaactaactcaaggtcgccagacttatgattggacattaaacagaaatcaaccggcagcaactgcattggccaacaccatagaagtctttagatcgaatggcctaacagctaatgagtcaggaaggctaatagatttcttaaaggatgtaatggaatcaatgaacaaagaggaaatagagataacaacccactttcaaagaaaaaggagagtaagagacaacatgaccaagaagatggtcacgcaaagaacaatagggaagaaaaaacaaagactgaataagagaggctatctaataagagcactgacattaaatacgatgaccaaagatgcagagagaggcaagttaaaaagaagggctatcgcaacacctgggatgcagattagaggtttcgtatactttgttgaaactttagctaggagcatttgcgaaaagcttgaacagtctgggctcccagtagggggcaatgaaaagaaggccaaactggcaaatgttgtgagaaagatgatgactaattcacaagacacagagatttctttcacaatcactggggacaacactaagtggaatgaaaatcaaaatcctcgaatgttcctggcgatgattacatatatcaccagaaatcaacccgagtggttcagaaacatcctgagcatggcacccataatgttctcaaacaaaatggcaagactagggagagggtacatgttcgagagtaaaagaatgaagattcgaacacaaataccagcagaaatgctagcaagcattgacctgaagtacttcaatgaatcaacaaagaagaaaattgagaaaataaggcctcttctaatagatggcacagcatcactgagtcctgggatgatgatgggcatgttcaacatgctaagtacggtcttgggagtctcgatactgaatcttggacaaaagaaatacaccaagacaatatactggtgggatgggctccaatcatccgacgattttgctctcatagtgaatgcaccaaaccatgagggaatacaagcaggagtggacagattctacaggacctgcaagttagtgggaatcaacatgagcaaaaagaagtcctatataaataagacagggacatttgaattcacaagctttttttatcgctatggatttgtggctaattttagcatggagctatccagctttggagtgtctggagtaaatgaatcagctgacatgagtattggagtaacagtgataaagaacaacatgataaacaatgaccttggacctgcaacggcccagatggctcttcaattgttcatcaaagactacagatacacatataggtgccataggggagacacacaaattcagacgagaagatcatttgagttaaagaagctgtgggatcaaacccaatcaaaggtagggctattagtatcagatggaggaccaaacttatacaatatacggaatcttcacattcctgaagtctgcttaaaatgggagctaatggatgatgattatcggggaagactttgtaatcccctgaatccctttgtcagtcataaagaggttgattctgtaaacaatgctgtggtaatgccagcccatggtccagccaaaagcatggaatatgatgccgttgcaactacacattcctggattcccaagaggaatcgttctattctcaacacaagccaaaggggaattcttgaggatgaacagatgtaccagaagtgctgcaatctattcgagaaatttttccctagcagttcatataggagaccggttggaatttctagcatggtggaggccatggtgtctagggcccggattgatgccagggtcgacttcgagtctggacggatcaagaaagaagagttctctgagatcatgaagatctgttccaccattgaagaactcagacggcaaaaataa

>H1N1_PB1_CY073747_A/Copenhagen/INS433/2009

atggatgtcaatccgactctacttttcctaaaaattccagcgcaaaatgccataagcaccacattcccttatactggagatcctccatacagccatggaacaggaacaggatacaccatggacacagtaaacagaacacaccaatactcagaaaagggaaagtggacgacaaacacagagactggtgcaccccagctcaacccgattgatggaccactacctgaggataatgaaccaagtgggtatgcacaaacagactgtgttctagaggctatggctttccttgaagaatcccacccaggaatatttgagaattcatgccttgaaacaatggaagttgttcaacaaacaagggtagataaattaactcaaggtcgccagacttatgattggacattaaacagaaatcaaccggcagcaactgcattggccaacaccatagaagtctttagatcgaatggcctaacagctaatgagtcaggaaggctaatagatttcttaaaggatgtaatggaatcaatgaacaaagaggaaatagagataacaacccactttcaaagaaaaaggagagtaagagacaacatgaccaagaagatggtcacgcaaagaacaatagggaagaaaaaacaaagactgaataagagaggctatctaataagagcactgacattaaatacgatgaccaaagatgcagagagaggcaagttaaaaagaagggctatcgcaacacctgggatgcagattagaggtttcgtatactttgttgaaactttagctaggagcatttgcgaaaagcttgaacagtctgggctcccagtagggggcaatgaaaagaaggccaaactggcaaatgttgtgagaaagatgatgactaattcacaagacacagagatttctttcacaatcactggggacaacactaagtggaatgaaaatcaaaatcctcgaatgttcctggcgatgattacatatatcaccagaaatcaacccgagtggttcagaaacatcctgagcatggcacccataatgttctcaaacaaaatggcaagactggggaaagggtacatgttcgagagtaaaagaatgaagattcgaacacaaataccagcagaaatgctagcaagcattgacctgaagtacttcaatgaatcaacaaagaagaaaattgagaaaataaggcctcttctaatagatggcacagcatcactgagtcctgggatgatgatgggcatgttcaacatgctaagtacggtcttgggagtctcgatactgaatcttggacaaaagaaatacaccaagacaatatactggtgggatgggctccaatcatccgacgattttgctctcatagtgaatgcaccaaaccatgagggaatacaagcaggagtggacagattctacaggacctgcaagttagtgggaatcaacatgagcaaaaagaagtcctatataaataagacagggacatttgaattcacaagctttttttatcgctatggatttgtggctaattttagcatggagctacccagctttggagtgtctggagtaaatgaatcagctgacatgagtattggaataacagtgataaagaacaacatgataaacaatgaccttggacctgcaacggcccagatggctcttcaattgttcatcaaagactacagatacacatataggtgccataggggagacacacaaattcagacgagaagatcatttgagttaaagaagctgtgggatcaaacccaatcaaaggtagggctattagtatcagatggaggaccaaacttatacaatatacggaatcttcacattcctgaagtctgcttaaaatgggagctaatggatgatgattatcggggaagactttgtaatcccctgaatccctttgtcagtcataaagagattgattctgtaaacaatgctgtggtaatgccagcccatggtccagccaaaagcatggaatatgatgccgttgcaactacacattcctggattcccaagaggaatcgttctattctcaacacaagccaaaggggaattcttgaggatgaacagatgtaccagaagtgctgcaatctattcgagaaatttttccctagcagttcatataggagaccggttggaatttctagcatggtggaggccatggtgtctagggcccggattgatgccagggtcgacttcgagtctggacggatcaagaaagaagagttctctgagatcatgaagatctgttccaccattgaagaactcagacggcaaaaataa

>H1N1_PB1_CY074169_A/California/VRDL213/2009

atggatgtcaatccgacattacttttcttaaaagtgccagcacaaaatgctataagcacaacttttccttatactggtgaccctccttacagccatgggacaggaacagggtacaccatggatacagtcaacaggacacaccagtactcagaaagaggaagatggacaaaaaataccgaaacgggagcaccgcaacttaacccaattgatggtcccttaccggaagacaatgaaccaagtggctatgcccaaacagattgtgtattagaagcaatggctttccttgaagaatcccatcccggtatctttgaaaactcttgtattgaaacaatggaggttgttcaacaaacaagggtggacaaactgacacaaggcagacagacctatgactggactctaaataggaaccagcctgctgccacagcattggcaaacactatagaagtattcagattaaacggcctaatagcaaatgaatctgggaggctaatagacttccttaaagatgtaatggagtcgatggacagaggcgaagtagaggtcacaactcattttcaaagaaagaggagagtgagagacaatgtaactaagaaaatggtgacccaaagaacaataggcaaaaagaaacataaattagacaaaagaagttacctaattagggcattaaccctgaacacaatgaccaaagatgctgagagggggaaactaaaacgcagagcaattgcaacccctggaatgcaaataagggggtttgtatactttgttgagacactggcaagaagcatatgtgaaaagcttgaacaatcaggattgccagttggaggaaatgagaagaaagcaaagttagcaaatgttgtaaggaagatgatgaccaactcccaggacactgaaatttctttcactataaccggagataacacaaaatggaacgaaaatcaaaaccctagaatgttcttggccatgatcacatatataaccaaaaatcagcctgaatggttcagaaatattctaagtattgctccaataatgttttcaaacaagatggcgagactaggtaaggggtacatgtttgaaagcaagagtatgaaactgagaactcaaatacctgcagagatgctagccaacatagacttgaaatatttcaatgattcaactaaaaagaaaattgaaaaaatccgaccattattaatagatggaactgcatcattgagtcctgggatgatgatgggcatgttcaatatgttgagcaccgtcttgggcgtctccattctgaatcttgggcaaaagagatacaccaagactacttactggtgggatggtcttcaatcgtctgatgattttgctttgattgtgaacgcacccaactatgcaggaattcaagctggagttgacaggttttatcgaacctgtaagctgctcggaattaatatgagcaaaaagaagtcttacataaacagaacaggtacctttgaattcacgagctttttctatcgttatgggtttgttgccaatttcagcatggagcttcctagttttggggtgtcaggggtcaatgaatctgcagacatgagtattggagtcactgtcatcaaaaacaatatgataaacaatgaccttggcccagcaactgctcaaatggcccttcagttatttataaaagattacaggtacacttatcgatgccaccgaggtgacacacaaatacaaacccggagatcatttgagataaagaaactatgggaccaaacccgctcaaaagctggactgttggtctctgatggaggccccaatttgtataacattagaaatctccatattcctgaagtttgcttgaaatgggagttgatggatgaggattaccaggggcgtttatgcaacccattaaacccgtttgtcagccataaagagattgaatcagtgaacagtgcagtgataatgccggcacatggtccagccaaaaatatagagtatgacgctgttgcaacaacacactcctgggtccccaaaagaaatcgatccattttgaacacgagccaaagggggatacttgaagatgagcaaatgtatcagaggtgctgcaatttatttgaaaaattcttcccaagtagctcatacagaagaccagttggaatatccagtatggtagaggctatggtctcaagagcccgaattgatgcacggattgatttcgaatctggaaggataaagaaagaggaatttgctgagatcatgaagatctgttccaccattgaagacctcagacggcaaaaatga

>H1N1_PB1_CY074281_A/California/VRDL232/2009

atggatgtcaatccgacattacttttcttaaaagtgccagcacaaaatgctataagcacaacttttccttatactggtgaccctccttacagccatgggacaggaacagggtacaccatggatacagtcaacaggacacaccagtactcagaaagaggaagatggacaaaaaataccgaaacgggagcaccgcaacttaacccaattgatggtcccttaccggaagacaatgaaccaagtggctatgcccaaacagattgtgtattagaagcaatggctttccttgaagaatcccatcccggtatctttgaaaactcttgtattgaaacaatggaggttgttcaacaaacaagggtggacaaactgacacaaggcagacagacctatgactggactctaaataggaaccagcctgctgccacagcattggcaaacactatagaagtattcagatcaaacggcctcatagcaaatgaatctgggaggctaatagacttccttaaagatgtaatggaatcaatggacagaggcgaagtagaggtcacaactcattttcaaagaaagaggagagtgagagacaatgtaactaaaaaaatggtgacccaaagaacaataggcaaaaagaaacataaattagacaaaagaagttacctaattagggcattaaccctgaacacaatgaccaaagatgctgagagggggaaactaaaacgcagagcaattgcaaccccaggaatgcaaataagggggtttgtatactttgttgagacactggcaagaagcatatgtgaaaagcttgaacaatcaggattgccagttggaggaaatgagaagaaagcaaagttagcaaatgttgtaaggaagatgatgaccaactcccaggacactgaaatttctttcaccataaccggagataacacaaaatggaacgaaaatcaaaaccctagaatattcttggccatgatcacatatataaccaaaaatcagcctgaatggttcagaaatattctaagtattgctccaataatgttttcaaacaagatggcgagactaggcaaggggtacatgtttgaaagcaagagtatgaaactgagaactcaaatacctgcagagatgctagccaacatagacttgaaatatttcaatgattcaactaaaaagaaaattgaaaaaatccgaccattattaatagatggaactgcatcattgagtcctggaatgatgatgggcatgttcaatatgttgagcaccgtcttgggcgtctccattctgaatcttgggcaaaagagatacaccaagactacttactggtgggatggtcttcaatcgtctgatgattttgctttgattgtgaacgcacccaactatgcaggaattcaagctggagttgacaggttttatcgaacctgtaagctgctcggaattaatatgagcaaaaagaagtcttacataaacagaacaggtacctttgaattcacgagctttttctatcgttatgggtttgttgccaatttcagcatggaacttccaagttttggggtgtctggggtcaatgaatctgcagacatgagtattggagtcactgtcatcaaaaacaatatgataaacaatgaccttggcccagcaactgctcaaatggcccttcagttatttatcaaagattacaggtacacttatcgatgccaccgaggtgacacacaaatacaaacccggagatcatttgagataaagaaactatgggaccaaacccgctcaaaagctggactgttggtctctgatggagggcccaatttgtataacattagaaatctccatattcctgaagtttgcttgaaatgggagttgatggatgaggattaccaggggcgtttatgcaacccattaaacccgtttgtcagccataaagagattgaatctgtgaacagtgcagtgataatgccggcacatggtccagccaaaaatatggagtatgacgctgttgcaacaacacactcctgggtccccaaaagaaatcgatccattttgaacacgagccaaagggggatacttgaagatgagcaaatgtatcagaggtgctgcaatttatttgaaaaattcttcccaagtagctcatacagaagaccagtcggaatatccagtatggtagaggctatggtctcaagagcccgaattgatgcacggattgatttcgaatctggaaggataaagaaagaggaatttgctgagatcatgaagatctgttccaccattgaagacctcagacggcaaaaatga

>H1N1_PB1_CY074337_A/California/VRDL245/2009

atggatgtcaatccgacattacttttcttaaaagtgccagcacaaaatgctataagcacaacttttccttatactggtgaccctccttacagccatgggacaggaacagggtacaccatggatacagtcaacaggacacaccagtactcagaaagaggaagatggacaaaaaataccgaaacgggagcaccgcaacttaacccaattgatggtcccttaccggaagacaatgaaccaagtggctatgcccaaacagattgtgtattagaagcaatggctttccttgaagaatcccatcccggtatctttgaaaactcttgtattgaaacaatggaggttgttcaacaaacaagggtggacaaactgacacaaggcagacagacctatgactggactctaaataggaaccagcctgctgccacagcattggcaaacactatagaagtattcagatcaaacggcctcatagcaaatgaatctgggaggctaatagacttccttaaagatgtaatggagtcgatggacagaggcgaagtagaggtcacaactcattttcaaagaaagaggagagtgagagacaatgtaactaagaaaatggtgacccaaagaacaataggcaaaaagaaacataaattagacaaaagaagttacctaattagggcattaaccctgaacacaatgaccaaagatgctgagagggggaaactaaaacgcagagcaatcgcaacccctggaatgcaaataagggggtttgtatactttgttgagacactggcaagaagcatatgtgaaaagcttgaacaatcaggattgccagttggaggaaatgagaagaaagcaaagttagcaaatgttgtaaggaagatgatgaccaactcccaggacactgaaatttctttcaccataaccggagataacacaaaatggaacgaaaatcaaaaccctagaatgttcttggccatgatcacatatataaccaaaaatcagcctgaatggttcagaaatattctaagtattgctccaataatgttttcaaacaagatggcgagactaggtaaggggtacatgtttgaaagcaagagtatgaaactgagaactcaaatacctgcagagatgctagccaacatagacttgaaatatttcaatgattcaactaaaaagaaaattgaaaaaatccgaccattattaatagatggaactgcatcattgagtcctgggatgatgatgggcatgttcaatatgttgagcaccgtcttgggcgtctccattctgaatcttgggcaaaagagatacaccaagactacttactggtgggatggtcttcaatcgtctgatgattttgctttgattgtgaacgcacccaactatgcaggaattcaagctggagttgacaggttttatcgaacctgtaagctgctcggaattaatatgagcaaaaagaagtcttacataaacagaacaggtacctttgaattcacgagctttttctatcgttatgggtttgttgccaatttcagcatggagcttcctagttttggggtgtcaggggtcaatgaatctgcagacatgagtattggagtcactgtcatcaaaaacaatatgataaacaatgaccttggcccagcaactgctcaaatggcccttcagttatttataaaagattacaggtacacttatcgatgccaccgaggtgacacacaaatacaaacccggagatcatttgagataaagaaactatgggaccaaacccgctcaaaagctggactgttggtctctgatggaggccccaatttgtataacattagaaatctccatattcctgaagtttgcttgaaatgggagttgatggatgaggattaccaggggcgtttatgcaacccattaaacccgtttgtcagccataaagagattgaatcagtgaacagtgcagtgataatgccggcacatggtccagccaaaaatatggagtatgacgctgttgcaacaacacactcctgggtccccaaaagaaatcgatccattttgaacacgagccaaagggggatacttgaagatgagcaaatgtatcagaggtgctgcaatttatttgaaaaattcttcccaagtagctcatacagaagaccagttggaatatccagtatggtagaggctatggtctcaagagcccgaattgatgcacggattgatttcgaatctggaaggataaagaaagaggaatttgctgagatcatgaagatctgttccaccattgaagacctcagacggcaaaaatga

>H1N1_PB1_CY074409_A/California/VRDL270/2009

atggatgtcaatccgacattacttttcttaaaagtgccagcacaaaatgctataagcacaacttttccttatactggtgaccctccttacagccatgggacaggaacagggtacaccatggatacagtcaacaggacacaccagtactcagaaagaggaagatggacagaaaataccgaaacgggagcaccgcaacttaacccaattgatggtcccttaccggaagacaatgaaccaagtggctatgcccaaacagattgtgtattagaagcaatggctttccttgaagaatcccatcccggtatctttgaaaactcttgtattgaaacaatggaggttgttcaacaaacaagggtggacaaactgacacaaggcagacagacctatgactggactctaaataggaaccagcctgctgccacagcattggcaaacactatagaagtattcagatcaaacggcctcatagcaaatgaatctgggaggctaatagacttccttaaagatgtaatggagtcgatggacagaggcgaagtagaggtcacaacacattttcaaagaaagaggagagtgagagacaatgtaactaaaaaaatggtgacccaaagaacaataggcaaaaagaaacataaattagacaaaagaagttacctgattagggcattaaccctgaacacaatgaccaaagatgctgagagggggaaactaaaacgcagagcaattgcaaccccaggaatgcaaataagggggtttgtatactttgttgagacactggcaagaagcatatgtgaaaagcttgaacaatcaggattgccagttggaggaaatgagaagaaagcaaagttagcaaatgttgtaaggaagatgatgaccaactcccaggacactgaaatttctttcaccataaccggagataacacaaaatggaacgaaaatcaaaaccctagaatgttcttggccatgatcacatatataaccaaaaatcagcctgaatggttcagaaatattctaagtattgctccaataatgttttcaaacaagatggcgagactaggtaaggggtacatgtttgaaagcaagagtatgaaactgagaactcaaatacctgcagagatgctagccaacatagacttgaaatatttcaatgattcaactaaaaagaaaattgaaaaaatccgaccattattaatagatggaactgcatcattgagtcctggaatgatgatgggcatgttcaatatgttgagcaccgtcttgggcgtctccatcctgaatcttgggcaaaagagatacaccaagactacttactggtgggatggtcttcaatcgtctgatgattttgctttgattgtgaatgcacccaactatgcaggaattcaagctggagttgacaggttttatcgaacctgtaagctgctcggaattaatatgagcaaaaagaagtcttacataaacagaacaggtacctttgaattcacgagctttttctatcgttatgggtttgttgctaatttcagcatggagcttcctagttttggggtgtctggggtcaatgaatctgcagacatgagtattggagtcactgtcatcaaaaacaatatgataaacaatgaccttggcccagcaactgctcaaatggcccttcagttatttataaaagattacaggtacacttatcgatgccaccgaggtgacacacaaatacaaacccggagatcatttgagataaagaaactatgggaccaaacccgctcaaaagctgggctgttggtctctgatggaggccccaatttgtataacattagaaatctccatatccctgaagtktgtttgaaatgggagttgatggatgaggattaccaggggcgtttatgcaacccattaaacccgtttgtcagccataaagagattgaatcagtgaacaatacagtgataatgccggcacatggtccagccaaaaatatggagtatgacgctgttgcaacaacacactcctgggtccccaaaagaaatcgatccattttgaacacgagccaaagggggatacttgaagatgagcaaatgtatcagaggtgctgcaatttatttgaaaaattcttcccaagtagctcatacagaagaccagttggaatatccagtatggtagaggctatggtctcaagagcccgaattgatgcacggattgatttcgaatctggaaggataaagaaagaggaatttgctgagatcatgaagatctgttccaccattgaagacctcagacggcaaaaatga

>H1N1_PB1_CY074665_A/California/VRDL380/2009

atggatgtcaatccgacattacttttcttaaaagtgccagcacaaaatgctataagcacaacttttccttatactggtgaccctccttacagccatgggacaggaacagggtacaccatggatacagtcaacaggacacatcagtactcagaaagaggaagatggacaaaaaataccgaaacgggagcaccgcaacttaacccaattgatggtcccttaccggaagacaatgaaccaagtggctatgcccaaacagattgtgtattagaagcaatggctttccttgaagaatcccaccccggtatctttgaaaactcttgtattgaaacaatggaggttgttcaacaaacaagggtggacaaactgacacaaggcagacagacctatgactggactctaaataggaaccagcctgctgccacagcattggcaaacactatagaagtattcagatcaaacggcctcatagcaaatgaatctggaaggctaatagacttccttaaagatgtaatggagtcgatggacagaggcgaagtagaggtcacaactcattttcaaagaaagaggagagtgagagacaatgtaactaaaaaaatggtgacccaaagaacaataggcaaaaagaaacataaattagacaaaagaagttacctaattagggcattaaccctgaatacaatgaccaaagatgctgagagggggaaactaaaacgcagagcaattgcaaccccaggaatgcaaataagggggtttgtatactttgttgagacactggcaagaagcatatgtgaaaagcttgaacaatcaggattgccagttggaggaaatgagaagaaagcaaagttagcaaatgttgtaaggaagatgatgactaactcccaggacactgaaatttctttcaccataaccggagacaacacaaaatggaacgaaaatcaaaaccctagaatgttcttggccatgatcacatatataaccaaaaatcagcctgaatggttcagaaatattctaagtattgctccaataatgttttcaaacaagatggcgagactaggtaaggggtacatgtttgaaagcaagagtatgaaactgagaactcaaatacctgcagagatgctagccaacatagacttgaaatatttcaatgattcaactaaaaagaaaattgaaaaaatccgaccattattaatagatggaactgcatcattgagtcctggaatgatgatgggcatgttcaatatgttgagcaccgtcttgggcgtctccattctgaatcttgggcaaaagagatacaccaagactacttactggtgggatggtcttcaatcgtctgatgattttgctttgattgtgaacgcacccaactatgcaggaattcaagctggagttgacaggttttatcgaacctgtaagctgctcggaattaatatgagcaaaaagaagtcttacataaacagaacaggtacctttgaattcacgagctttttctatcgttatgggtttgttgccaatttcagcatggagcttcctagttttggggtgtctggggtcaatgaatctgcagacatgagtattggagtcactgtcatcaaaaacaatatgataaacaatgaccttggcccagcaactgctcaaatggcccttcagttatttataaaagattacaggtacacttatcgatgccaccgaggtgacacacaaatacaaacccgaagatcatttgagataaagaaactatgggaccaaacccgctcaaaagctggactgttggtctctgatggaggccccaatttgtataacattagaaatctccatattcctgaagtttgcttgaaatgggagttgatggatgaggattaccaggggcgtttatgcaacccattaaacccgtttgtcagccataaagagattgaatcagtgaacagtgcagtgataatgccggcacatggtccagccaaaaatatggagtatgacgctgttgcaacaacacactcctgggtccccaaaagaaatcgatccattttgaacacgagccaaagggggatacttgaagatgagcaaatgtatcagaggtgctgcaatttatttgaaaaattcttcccaagtagctcatacagaagaccagttggaatatccagtatggtagaggctatggtctcaagagcccgaattgatgcacggattgacttcgaatctggaaggataaagaaagaggaatttgctgagatcatgaagatctgttccaccattgaagacctcagacggcaaaaatga

>H1N1_PB1_HQ291967_A/Taiwan/10103/2008

atggatgtcaatccgacattacttttcttaaaagtgccagcacaaaatgctataagcacaacttttccttatactggtgaccctccttacagccatgggacaggaacagggtacaccatggatacagtcaacaggacacaccagtactcagaaagaggaagatggacaaaaaataccgaaacgggagcaccgcaacttaacccaattgatggtcccttaccggaagacaatgaaccaagtggctatgcccaaacagattgtgtattagaagcaatggctttccttgaagaatcccatcccggtatctttgaaaactcttgtattgaaacaatggaggttgttcaacaaacaagggtggacaaactgacacaaggcagacagacctatgactggactctaaatagaaaccagcctgctgccacagcattggcaaacactatagaagtattcagatcaaacggcctcatagcaaatgaatctgggaggctaatagacttccttaaagatgtaatggagtcgatggacagaggcgaagtagaggtcacaactcattttcaaagaaagaggagagtgagagacaatgtaactaaaaaaatggtgacccaaagaacaataggcaaaaagaaacataaattagacaaaagaagttacctaattagggcattaaccctgaacacaatgaccaaagatgctgagagggggaaactaaaacgcagagcaattgcaaccccaggaatgcaaataagggggtttgtatactttgttgagacactggcaagaagcatatgtgaaaagcttgaacaatcaggattgccagttggaggaaatgagaagaaagcaaagttagcaaatgttgtaaggaagatgacgaccaactcccaggacactgaaatttctttcaccataaccggagataacacaaaatggaacgaaaatcaaaaccctagaatgttcttggccatgatcacatacataaccaaaaatcagcctgaatggttcagaaatattctaagtattgctccaataatgttttcaaacaagatggcgagactaggtaaggggtacatgtttgaaagcaagagtatgaaactgagaactcaaatacctgcagagatgctagccaacatagacttgaaatatttcaatgattcaactaaaaagaaaattgaaaaaatccgaccattattaatagatggaactgcatcattgagtcctggaatgatgatgggcatgttcaatatgttgagcaccgtcttgggcgtctccattctgaatcttgggcaaaagagatacaccaagactacttactggtgggatggtcttcaatcgtctgatgattttgctttgattgtgaacgcacccaactatgcaggaattcaagctggagttgacaggttttatcgaacctgtaagctgctcggaattaatatgagcaaaaagaagtcttacataaacagaacaggtacctttgaattcacgagctttttctatcgttatgggtttgttgccaatttcagcatggagcttcctagttttggggtgtctggggtcaatgaatctgcagacatgagtattggagtcactgtcatcaaaaacaatatgataaacaatgaccttggcccagcaactgctcaaatggcccttcagttatttataaaagattacaggtacacttatcgatgccaccgaggtgacacacaaatacaaacccggagatcatttgagataaagaagctatgggaccaaacccgctcaaaagctgggctgttggtctctgatggaggccccaatttgtataacattagaaatctccatattcctgaagtttgcttgaaatgggagttgatggatgaggattaccaggggcgtttatgcaacccattaaacccgtttgtcagccataaagagattgaatcagtgaacaatgcagtgataatgccggcacatggtccagccaaaaatatggagtatgacgctgttgcaacaacacactcctgggtccccaaaagaaatcgatccattttgaacacgagccaaagggggatacttgaagatgagcaaatgtatcagaggtgctgcaatttatttgaaaaattcttcccaagtagctcatacagaagaccagttggaatatccagtatggtagaggctatggtctcaagagcccgaattgatgcacggattgatttcgaatctggaaggataaagaaagaggaatttgctgagatcatgaagatctgttccaccattgaagacctcagacggcaaaaatga

>H1N1_PB1_CY075081_A/Athens/INS416/2010

atggatgtcaatccgactctacttttcctaaaaattccagcgcaaaatgccataagcaccacattcccttatactggagatcccccatacagccatggaacaggaacaggatacaccatggacacagtaaacagaacacaccaatactcagaaaagggaaagtggacgacaaacacagagactggtgcaccccagctcaacccgattgatggaccactacctgaggataatgaaccaagtgggtatgcacaaacagactgtgttctagaggctatggctttccttgaagaatcccacccaggaatatttgagaattcatgccttgaaacaatggaagttgttcaacaaacaagggtagataaactaactcaaggtcgccagacttatgattggacattaaacagaaatcaaccggcagcaactgcattggccaacaccatagaagtctttagatcgaatggcctaacagctaatgagtcaggaaggctaatagatttcttaaaggatgtaatggaatcaatgaacaaagaggaaatagagataacaacccactttcaaagaaaaaggagagtaagagacaacatgaccaagaagatggtcacgcaaagaacaatagggaagaaaaaacaaagactgaataagagaggctatctaataagagcactgacattaaatacgatgaccaaagatgcagagagaggcaagttaaaaagaagggctatcgcaacacctgggatgcagattagaggtttcgtatactttgttgaaactttagctaggagcatttgcgaaaagcttgaacagtctgggctcccagtagggggcaatgaaaagaaggccaaactggcaaatgttgtgagaaagatgatgactaattcacaagacacagagatttctttcacaatcactggggacaacactaagtggaatgaaaatcaaaatcctcgaatgttcctggcgatgattacatatatcaccagaaatcaacccgagtggttcagaaacatcctgagcatggcacccataatgttctcaaacaaaatggcaagactagggaaagggtacatgttcgagagtaaaagaatgaagattcgaacacaaataccagcagaaatgctagcaagcattgacctgaagtacttcaatgaatcaacaaagaagaaaattgagaaaataaggcctcttctaatagatggcacagcatcactgagtcctggaatgatgatgggcatgttcaacatgctaagtacggtcttgggagtctcgatactgaatcttggacaaaagaaatacaccaagacaatatactggtgggatgggctccaatcatccgacgattttgctctcatagtgaatgcaccaaaccatgagggaatacaagcaggagtggacagattctacaggacctgcaagttagtgggaatcaacatgagcaaaaagaagtcctatataaataagacagggacatttgaattcacaagctttttttatcgctatggatttgtggctaattttagcatggagctacccagctttggagtgtctggagtaaatgaatcagctgacatgagtattggagtaacagtgataaagaacaacatgataaacaatgaccttggacctgcaacggcccagatggctcttcaattgttcatcaaagactacagatacacatataggtgccataggggagacacacaaattcagacgagaagatcatttgagttaaagaagctgtgggatcaaacccaatcaaaggtagggctattagtatcagatggaggaccaaacttatacaatatacggaatcttcacattcctgaagtctgcttaaaatgggagctaatggatgatgattatcggggaagactttgtaatcccctgaatccctttgtcagtcataaagagattgattctgtaaacaatgctgtggtaatgccagcccatggtccagccaaaagcatggaatatgatgccgttgcaactacacattcctggattcccaagaggaatcgttctattctcaacacaagccaaaggggaattcttgaggatgaacagatgtaccagaagtgctgcaatctattcgagaaatttttccctagcagttcatataggagaccggttggaatttctagcatggtggaggccatggtgtctagggcccggattgatgccagggtcgacttcgagtctggacggatcaagaaagaagagttctctgagatcatgaagatctgttccaccattgaagaactcagacggcaaaaataa

>H1N1_PB1_CY075393_A/Chile/3760/2009

atggatgtcaatccgactctacttttcctaaaaattccagcgcaaaatgccataagcaccacattcccttatactggagatcctccatacagccatggaacaggaacaggatacaccatggacacagtaaacagaacacaccaatactcagaaaagggaaagtggacgacaaacacagagactggtgcaccccagctcaacccgattgatggaccactacctgaggataatgaaccaagtgggtatgcacaaacagactgtgttctagaggctatggctttccttgaagaatcccacccaggaatatttgagaattcatgccttgaaacaatggaagttgttcaacaaacaagggtagataaactaactcaaggtcgccagacttatgattggacattaaacagaaatcaaccggcagcaactgcattggccaacaccatagaagtctttagatcgaatgacctaacagctaatgagtcaggaaggctaatagatttcttaaaggatgtaatggaatcaatgaacaaagaggaaatagagataacaacccactttcaaagaaaaaggagagtaagagacagcatgaccaagaagatggtcacgcaaagaacaatagggaagaaaaaacaaagactgaataagagaggctatctaataagagcactgacattaaatacgatgaccaaagatgcagagagaggcaagttaaaaagaagggctatcgcaacacctgggatgcagattagaggtttcgtatactttgttgaaactttagctaggagcatttgcgaaaagcttgaacagtctgggctcccagtagggggcaatgaaaagaaggccaaactggcaaatgttgtgagaaagatgatgaccaattcacaagacacagagatttctttcacaatcactggggacaacactaagtggaatgaaaatcaaaatcctcgaatgttcctggcgatgattacatatatcaccagaaatcaacccgagtggttcagaaacatcctgagcatggcacccataatgttctcaaacaaaatggcaagactagggaaagggtacatgttcgagagtaaaagaatgaagattcgaacacaaataccagcagaaatgctagcaagcattgacctgaagtacttcaatgaatcaacaaagaagaaaattgagaaaataaggcctcttctaatagatggcacagcatcactgagtcctgggatgatgatgggcatgttcaacatgctaagtacggtcttgggagtctcgatactgaatcttggacaaaagaaatacaccaagacaatatactggtgggatgggctccaatcatccgacgattttgctctcatagtgaatgcaccaaaccatgagggaatacaagcaggagtggacagattctacaggacctgcaagttagtgggaatcaacatgagcaaaaagaagtcctatataaataagacagggacatttgaattcacaagctttttttatcgctatggatttgtggctaattttagcatggagctacccagctttggagtgtctggagtaaatgaatcagctgacatgagtattggagtaacagtgataaagaacaacatgataaacaatgaccttggacctgcaacggcccagatggctcttcaattgttcatcaaagactacagatacacatataggtgccataggggagacacacaaattcagacgagaagatcatttgagttaaagaagctgtgggatcaaacccaatcaaaggtagggctattagtatcagatggaggaccaaacttatacaatatacggaatcttcacattcctgaagtctgcttaaaatgggagctaatggatgatgattatcggggaagactttgtaatcccctgaatccctttgtcagtcataaagagattgattctgtaaacaatgctgtggtaatgccagcccatggtccagccaaaagcatggaatatgatgccgttgcaactacacattcctggattcccaagaggaatcgttctattctcaacacaagccaaaggggaattcttgaggatgaacaaatgtaccagaagtgctgcaatctattcgagaaatttttccctagcagttcatataggagaccggttggaatttctagcatggtggaggccatggtgtctagggcccggattgatgccagggtcgacttcgagtctggacggatcaagaaagaagagttctctgagatcatgaagatctgttccaccattgaagaactcagacggcaaaaataa

>H1N1_PB1_HQ695918_A/Taiwan/14/2009

atggatgtcaatccgactctacttttcctaaaaattccagcgcaaaatgccataagcaccacattcccttatactggagatcctccatacagccatggaacaggaacaggatacaccatggacacagtaaacagaacacaccaatactcagaaaagggaaagtggacgacaaacacagagactggtgcaccccagctcaacccgattgatggaccactacctgaggataatgaaccaagtgggtatgcacaaacagactgtgttctagaggctatggctttccttgaagaatcccacccaggaatatttgagaattcatgccttgaaacaatggaagttgttcaacaaacaagggtagataaactaactcaaggtcgccagacttatgattggacattaaacagaaatcaaccggcagcaactgcattggccaacaccatagaagtctttagatcgaatggcctaacagctaatgagtcaggaaggctaatagatttcttaaaggatgtaatggaatcaatgaacaaagaggaaatagagataacaacccactttcaaagaaaaaggagagtaagagacaacatgaccaagaagatggtcacgcaaagaacaatagggaagaaaaaacaaagactgaataagagaggctatctaataagagcactgacattaaatacgatgaccaaagatgcagaaagaggcaagttaaaaagaagggctatcgcaacacctgggatgcaaattagaggtttcgtatactttgttgaaactttagctaggagcatttgcgaaaagcttgaacagtctgggctcccagtagggggcaatgaaaagaaggccaaactggcaaatgttgtgggaaagatgatgactaattcacaagacacagagatttctttcacaatcactggggacaacactaagtggaatgaaaatcaaaatcctcgaatgttcctggcgatgattacatatatcaccagaaatcaacccgagtggttcaaaaacatcctgagcatggcacccataatgttctcaaacaaaatggcgagactagggaaagggtacatgtttgagagtaaaagaatgaagattcgaacacaaataccagcagaaatgctagcaagcattgacctgaagtacttcaatgaatcaacaaagaagaaaattgagaaaataaggcctcttctaatagatggcacagcatcactgagtcctgggatgatgatgggcatgttcaacatgctaagtacggtcttgggagtctcgatactgaatcttggacaaaagaaatacaccaagacaatatactggtgggatgggctccaatcatccgacgattttgctctcatagtgaatgcaccaaaccatgagggaatacaagcaggagtggacagattctacaggacctgcaagttagtgggaatcaacatgagcaaaaagaagtcctatataaataagacagggacatttgaattcacaagctttttttatcgctatggatttgtggctaattttagcatggagctacccagctttggagtgtctggagtaaatgaatcagctgacatgagtattggagtaacagtgataaagaacaacatgataaacaatgaccttggacctgcaacggcccagatggctcttcaattgttcatcaaagactacagatacacatataggtgccataggggagacacacaaattcagacgagaagatcatttgagttaaagaagctgtgggatcaaacccaatcaaaggtagggctattagtatcagatggaggaccaaacttatacaatatacggaatcttcacattcctgaagtctgcttaaaatgggagctaatggatgatgattatcggggaagactttgtaatcccctgaatccctttgtcagtcataaagagattgattctgtaaacaatgctgtggtaatgccagcccatggtccagccaaaagcatggaatatgatgccgttgcaactacacattcctggattcccaagaggaatcgttctattctcaacacaagccaaaggggaattcttgaggatgaacagatgtaccagaagtgctgcaatctattcgagaaatttttccctagcagttcatataggagaccggttggaatttctagcatggtggaggccatggtgtctagggcccggattgatgccagggtcgacttcgagtctggacggatcaagaaagaagagttctctgagatcatgaagatctgttccaccattgaagaactcagacggcaaaaataa

>H1N1_PB1_HQ712181_A/Brisbane/2015/2009

atggatgtcaatccgactctacttttcctaaaaattccagcgcaaaatgccataagcaccacattcccttatactggagatcctccatacagccatggaacaggaacaggatacaccatggacacagtaaacagaacacaccaatactcagaaaagggaaagtggacgacaaacacagagactggtgcaccccagctcaacccgattgatggaccactacctgaggataatgaaccaagtgggtatgcacaaacagactgtgttctagaggctatggctttccttgaagaatcccacccaggaatatttgagaattcatgccttgaaacaatggaagttgttcaacaaacaagggtagataaactaactcaaggtcgccagacttatgattggacattaaacagaaatcaaccggcagcaactgcattggccaacaccatagaagtctttagatcgaatggcctaacagctaatgagtcaggaaggctaatagatttcttaaaggatgtaatggaatcaatgaacaaagaggaaatagagataacaacccactttcaaagaaaaaggagagtaagagacaacatgaccaagaagatggtcacgcaaagaacaatagggaagaaaaaacaaagactgaataagagaggctatctaataagagcactgacattaaatacgatgaccaaagatgcagagagaggcaagttaaaargaagggctatcgcaacacctgggatgcagattagaggtttcgtatactttgttgaaactttagctaggagcatttgcgaaaagcttgaacagtctgggctcccagtagggggcaatgaaaagaaggccaaactggcaaatgttgtgagaaagatgatgactaattcacaagacacagagatttctttcacaatcactggggacaacactaagtggaatgaaaatcaaaatcctcgaatgttcctggcgatgattacatatatcaccagaaatcaacccgagtggttcagaaacatcctgagcatggcacccataatgttctcaaacaaaatggcaagactagggaaagggtacatgttcgagagtaaaagaatgaagattcgaacacaaataccagcagaaatgctagcaagcattgacctgaagtacttcaatgaatcaacaaagaaaaaaattgagaaaataaggcctcttctaatagatggcacagcatcactgagtcctgggatgatgatgggcatgttcaacatgctaagtacggtcttgggagtctcgatactgaatcttggacaaaagaaatacaccaagacaatatactggtgggatgggctccaatcatccgacgattttgctctcatagtgaatgcaccaaaccatgagggaatacaagcaggagtggacagattctacaggacctgcaagttagtgggaatcaacatgagcaaaaagaagtcctatataaataagacagggacatttgaattcacaagctttttttatcgctatggatttgtggctaattttagcatggagctacccagctttggagtgtctggagtaaatgaatcagctgacatgagtattggagtaacagtgataaagaacaacatgataaacaatgaccttggacctgcaacggcccagatggctcttcaattgttcatcaaagactacagatacacatataggtgccataggggagacacacaaattcagacgagaagatcatttgagttaaagaagctgtgggatcaaacccaatcaaaggtrgggctattagtatcagatggaggaccaaacttatacaatatacggaatcttcacattcctgaagtctgcttaaaatgggagctaatggatgatgactatcggggaagactttgtaatcccctgaatccctttgtcagtcataaagagattgattctgtaaacaatgctgtggtaatgccagcccatggtccagccaaaagcatggaatatgatgccgttgcaactacacattcctggattcccaagaggaatcgttctattctcaacacaagccaaaggggaattcttgaggatgaacagatgtaccagaagtgctgcaatctattcgagaaatttttccctagcagttcatataggagaccggttggaatttctagcatggtggaggccatggtgtctagggcccggattgatgccagggtcgacttcgagtctggacggatcaagaaagaagagttctctgagatcatgaagatctgttccaccattgaagaactcagacggcaaaaataa

>H1N1_PB1_CY073994_A/Argentina/HNRG107/2009

atggatgtcaatccgactctacttttcctaaaaattccagcgcaaaatgccataagcaccacattcccttatactggagatcctccatacagccatggaacaggaacaggatacaccatggacacagtaaacagaacacaccaatactcagaaaagggaaagtggacgacaaacacagagactggtgcaccccagctcaacccgattgatggaccactacctgaggataatgaaccaagtgggtatgcacaaacagactgtgttctagaggctatggctttccttgaagaatcccacccaggaatatttgagaattcatgccttgaaacaatggaagttgttcaacaaacaagggtagataaactaactcaaggtcgccagacttatgattggacattaaacagaaatcaaccggcagcaactgcattggccaacaccatagaagtctttagatcgaatggcctaacagctaatgagtcaggaaggctaatagatttcttaaaggatgtaatggaatcaatgaacaaagaggaaatagagataacaacccactttcaaagaaaaaggagagtaagagacaacatgaccaagaagatggtcacgcaaagaacaatagggaagaaaaaacaaagactgaataagagaggctatctaataagagcactgacattaaatacgatgaccaaagatgcagagagaggcaagttaaaaagaagggctatcgcaacacctgggatgcagattagaggtttcgtatactttgttgaaactttagctaggagcatttgcgaaaagcttgaacagtctgggctcccagtagggggcaatgaaaagaaggccaaactggcaaatgttgtgagaaagatgatgactaattcacaagacacagagatttctttcacaatcactggggacaacactaagtggaatgaaaatcaaaatcctcgaatgttcctggcgatgattacatatatcaccagaaatcaacccgagtggttcagaaacatcctgagcatggcacccataatgttctcaaacaaaatggcaagactagggaaagggtacatgttcgagagtaaaagaatgaagattcgaacacaaataccagcagaaatgctagcaagcattgacctgaagtacttcaatgaatcaacaaagaagaaaattgagaaaataaggcctcttctaatagatggcacagcatcactgagtcctgggatgatgatgggcatgttcaacatgctaagtacggtcttgggagtctcgatactgaatcttggacaaaagaaatacaccaagacaatatactggtgggatgggctccaatcatccgacgattttgctctcatagtgaatgcaccaaaccatgagggaatacaagcaggagtggacagattctacaggacctgcaagttagtgggaatcaacatgagcaaaaagaagtcctatataaataagacagggacatttgaattcacaagctttttttatcgctatggatttgtggctaattttagcatggagctacccagctttggagtgtctggagtaaatgaatcagctgacatgagtattggagtaactgtgataaagaacaacatgataaacaatgaccttggacctgcaacggcccagatggctcttcaattgttcatcaaagactacagatacacatataggtgccataggggagacacacaaattcagacgagaagatcatttgagttaaagaagctgtgggatcaaacccaatcaaaggtagggctattagtatcagatggaggaccaaacttatacaatatacggaatcttcacattcctgaagtctgcttaaaatgggagctaatggatgatgattatcggggaagactttgtaatcccctgaatccctttgtcagtcataaagagattgattctgtaaacaatgctgtggtaatgccagcccatggtccagccaaaagcatggaatatgatgccgttgcaactacacattcctggattcccaagaggaatcgttctattctcaacacaagccaaaggggaattcttgaggatgaacagatgtaccagaagtgctgcaatctattcgagaaatttttccctagcagttcatataggagaccggttggaatttctagcatggtggaggccatggtgtctagggcccggattgatgccagggtcgacttcgagtctggacggatcaagaaagaagagttctctgagatcatgaagatctgttccaccattgaagaactcagacggcaaaaataa

>H1N1_PB1_CY080572_A/Ulaanbaatar/190/2011

atggatgtcaatccgactctacttttcctaaaaattccagcgcaaaatgccataagcaccacattcccttatactggagatcctccatacagccatggaacaggaacaggatacaccatggacacagtaaacaggacacaccaatactcagaaaagggaaagtggacgacaaacacagagactggtgcaccccagctcaacccgattgatggaccactacctgaggataatgaaccaagtgggtatgcacaaacagactgtgttctagaggctatggctttccttgaagaatcccacccaggaatatttgagaattcatgccttgaaacaatggaagttgttcaacaaacaagggtagataaactgactcaaggtcgccagacttatgattggacattaaacagaaatcaaccggcagcaactgcattggccaacaccatagaagtctttagatcgaatggcctaacagctaatgagtcaggaaggctaatagatttcttaaaggatgtaatggaatcaatgaacaaagaggaaatagagataacaacccactttcaaagaaaaaggagagtaagagacaacatgaccaagaagatggtcacgcaaagaacaatagggaagaaaaaacagagactgaataagagaggctatctaataagagcactgacattaaatacgatgaccaaagatgcagagagaggcaagttaaaaagaagggctatcgcaacacctgggatgcagattagaggtttcgtatactttgttgaaactttagctaggagcatttgcgaaaagcttgaacagtctgggctcccagtggggggcaatgaaaagaaggccaaactggcaaatgttgtgagaaagatgatgactaattcacaagacacagagatttctttcacaatcactggggacaacactaagtggaatgaaaatcaaaatcctcgaatgttcctggcgatgattacatatatcaccagaaatcaacccgagtggtttagaaacatcctgagcatggcacccataatgttctcaaacaaaatggcaagactagggaaagggtacatgttcgagagtaaaagaatgaagattcgaacacaaataccagcagaaatgctagcaagcattgacctgaagtacttcaatgaatcaacaaagaagaaaattgagaaaataaggcctcttttaatggatggcacagcatcactgagtcctgggatgatgatgggcatgttcaacatgctaagtacggtcttgggagtctcgatactgaatcttggacaaaagaaatacaccaagacaacatactggtgggatgggctccaatcatccgacgattttgctctcatagtgaatgcaccaaaccatgagggaatacaagcaggagtggacagattctacaggacctgcaagttagtgggaatcaacatgagcaaaaagaagtcttatataaataagacagggacatttgaattcacaagctttttttatcgctatggatttgtggctaattttagcatggagctacccagctttggagtgtctggagtaaatgaatcagctgacatgagtattggagtaacagtgataaagaacaacatgataaacaatgaccttggacctgcaacggcccagatggctcttcaattgttcatcaaagactacaggtacacatataggtgccataggggagacacacaaattcagacgagaagatcatttgagttaaagaagctgtgggatcaaacccaatcaaaggtagggctattagtatcagatggaggaccaaacttatacaatatacggaatcttcacattcctgaagtctgcttaaaatgggagctaatggatgatgattatcggggaagactttgtaatcccctgaatccctttgtaagtcataaggagattgattctgtaaacaatgctgtggtaatgccagcccatggtccagccaaaagcatggaatatgatgccgttgcaactacacattcctggattcccaagaggaatcgttctattctcaacacaagccaaaggggaattcttgaggatgaacagatgtaccagaagtgctgcaatctattcgagaaatttttccctagcagttcatataggagaccggttggaatttctagcatggtggaggccatggtgtctagggcccggattgatgccagggtcgactttgagtctggacggatcaagaaagaagagttctctgagatcatgaagatctgttccaccattgaagaactcagacggcaaaaataa

>H1N1_PB1_CY080751_A/Boston/33/2009

atggatgtcaatccgacattacttttcttaaaagtgccagcacaaaatgctataagcacaacttttccttatactggtgaccctccttacagccatgggacaggaacagggtacaccatggatacagtcaacaggacacaccagtactcagaaagaggaagatggacaaaaaataccgaaacgggagcaccgcaacttaacccaattgatggtcccttaccggaagacaatgaaccaagtggctatgcccaaacagattgtgtattagaagcaatggctttccttgaagaatcccatcccggtatctttgaaaactcttgtattgaaacaatggaggttgttcaacaaacaagggtggacaaactgacacaaggcagacagacctatgactggactctaaataggaaccagcctgctgccacagcattggcaaacactatagaagtattcagatcaaacggcctcatagcaaatgaatctggaaggctaatagacttccttaaagatgtaatggagtcgatggacagaggcgaagtagaggtcacaactcattttcaaagaaagaggagagtgagagacaatgtaactaaaaaaatggtgacccaaagaacaataggcaaaaagaaacataaattagacaaaagaagttacctaattagggcattaaccctgaacacaatgaccaaagatgctgagagggggaaactaaaacgcagagcaattgcaaccccaggaatgcaaataagggggtttgtatactttgttgagacactggcaagaagcatatgtgaaaagcttgaacaatcaggattgccagttggaggaaatgagaagaaagcaaagttagcaaatgttgtaaggaagatgatgaccaactcccaggacactgaaatttctttcaccataaccggagacaacacaaaatggaacgaaaatcaaaaccctagaatgttcttggccatgatcacatatataaccaaaaatcagcctgaatggttcagaaatattctaagtattgctccaataatgttttcaaacaagatggcgagactaggtaaggggtacatgtttgaaagcaagagtatgaaactgagaactcaaatacctgcagagatgctagccaacatagacttgaaatatttcaatgattcaactaaaaagaaaattgaaaaaatccgaccattattaatagatggaactgcatcattgagtcctggaatgatgatgggcatgttcaatatgttgagcaccgtattgggcgtctccattctgaatcttgggcaaaagagatacaccaagactacttactggtgggatggtcttcaatcgtctgatgattttgctttaattgtgaacgcacccaactatgcaggaattcaagctggagttgacaggttttatcgaacctgtaagctgctcggaattaatatgagcaaaaagaagtcttacataaacagaacaggtacctttgaattcacgagctttttctatcgttatgggtttgttgccaatttcagcatggagcttcctagttttggggtgtctggggtcaatgaatctgcagacatgagtattggagtcactgtcatcaaaaacaatatgataaacaatgaccttggcccagcaactgctcaaatggcccttcagttatttataaaagattacaggtacacttatcgatgccaccgaggtgacacacaaatacaaacccgaagatcatttgagataaagaaactatgggaccaaacccgctcaaaagctggactgttggtctctgatggaggccccaatttgtataacattagaaatctccatattcctgaagtttgcttgaaatgggagttgatggatgaggattaccaggggcgtttatgcaacccattaaacccgtttgtcagccataaagagattgaatcagtgaacagtgcagtgataatgccggcacatggtccagccaaaaatatggagtatgacgctgttgcaacaacacactcctgggtccccaaaagaaatcgatccattttgaacacgagccaaagggggatacttgaagatgagcaaatgtatcagaggtgctgcaatttatttgaaaaattcttcccaagtagctcatacagaagaccagttggaatatccagtatggtagaggctatggtctcaagagcccgaattgatgcacggattgatttcgaatctggaaggataaagaaagaggaatttgctgagatcatgaagatctgttccaccattgaagacctcagacggcaaaaatga

>H1N1_PB1_CY080767_A/Boston/36/2009

atggatgtcaatccgacattacttttcttaaaagtgccagcacaaaatgctataagcacaacttttccttatactggtgaccctccttacagccatgggacaggaacagggtacaccatggatacagtcaacaggacacaccagtactcagaaagaggaagatggacaaaaaataccgaaacgggagcaccgcaacttaacccaattgatggtcccttaccggaagacaatgaaccaagtggctatgcccaaacagattgtgtattagaagcaatggctttccttgaagaatcccatcccggtatctttgaaaactcttgtattgaaacaatggaggttgttcaacaaacaagggtggacaaactgacacaaggcagacagacctatgactggactctaaataggaaccagcctgctgccacagcattggcaaacactatagaagtattcagatcaaacggcctcatagcaaatgaatctggaaggctaatagacttccttaaagatgtaatggagtcgatggacagaggcgaagtagaggtcacaactcattttcaaagaaagaggagagtgagagacaatgtaactaaaaaaatggtgacccaaagaacaataggcaaaaagaaacataaattagacaaaagaagttacctaattagggcattaaccctgaacacaatgaccaaagatgctgagagggggaaactaaaacgcagagcaattgcaaccccaggaatgcaaataagggggtttgtatactttgttgagacactggcaagaagcatatgtgaaaagcttgaacaatcaggattgccagttggaggaaatgagaagaaagcaaagttagcaaatgttgtaaggaagatgatgaccaactcccaggacactgaaatttctttcaccataaccggagacaacacaaaatggaacgaaaatcaaaaccctagaatgttcttggccatgatcacatatataaccaaaaatcagcctgaatggttcagaaatattctaagtattgctccaataatgttttcaaacaagatggcgagactaggtaaggggtacatgtttgaaagcaagagtatgaaactgagaactcaaatacctgcagagatgctagccaacatagacttgaaatatttcaatgattcaactaaaaagaaaattgaaaaaatccgaccattattaatagatggaactgcatcattgagtcctggaatgatgatgggcatgttcaatatgttgagcaccgtcttgggcgtctccattctgaatcttgggcaaaagagatacaccaagactacttactggtgggatggtcttcaatcgtctgatgattttgctttgattgtgaacgcacccaactatgcaggaattcaagctggagttgacaggttttatcgaacctgtaagctgctcggaattaatatgagcaaaaagaagtcttacataaacagaacaggtacctttgaattcacgagctttttctatcgttatgggtttgttgccaatttcagcatggagcttcctagttttggggtgtctggggtcaatgaatctgcagacatgagtattggagtcactgtcatcaaaaacaatatgataaacaatgaccttggcccagcaactgctcaaatggcccttcagttatttataaaagattacaggtacacttatcgatgccaccgaggtgacacacaaatacaaacccgaagatcatttgagataaagaaactatgggaccaaacccgctcaaaagctggactgttggtctctgatggaggccccaatttgtataacattagaaatctccatattcctgaagtttgcttgaaatgggagttgatggatgaggattaccaggggcgtttatgcaacccattaaacccgtttgtcagccataaagagattgaatcggtgaacagtgcagtgataatgccggcacatggtccagccaaaaatatggagtatgacgctgttgcaacaacacactcctgggtccccaaaagaaatcgatccattttgaacacgagccaaagggggatacttgaagatgagcaaatgtatcagaggtgctgcaatttatttgaaaaattcttcccaagtagctcatacagaagaccagttggaatatccagtatggtagaggctatggtctcaagagcccgaattgatgcacggattgatttcgaatctggaaggataaagaaagaggaatttgctgagatcatgaagatctgttccaccattgaagacctcagacggcaaaaatga

>H1N1_PB1_CY080871_A/Boston/60/2009

atggatgtcaatccgacattacttttcttaaaagtgccagcacaaaatgctataagcacaacttttccttatactggtgaccctccttacagccatgggacaggaacagggtacaccatggatacagtcaacaggacacaccagtactcagaaagaggaagatggacaaaaaataccgaaacgggagcaccgcaacttaacccaattgatggtcccttaccggaagacaatgaaccaagtggctatgcccaaacagattgtgtattagaagcaatggctttccttgaagaatcccatcccggtatctttgaaaactcttgtattgaaacaatggaggttgttcaacaaacaagggtggacaaactgacacaaggcagacagacctatgactggactctaaataggaaccagcctgctgccacagcattggcaaacactatagaagtattcagatcaaacggcctcatagcaaatgaatctggaaggctaatagacttccttaaagatgtaatggagtcgatggacagaggcgaagtagagatcacaactcattttcaaagaaagaggagagtgagagacaatgtaactaaaaaaatggtgacccaaagaacaataggcaaaaagaaacataaattagacaaaagaagttacctaattagggcattaaccctgaacacaatgaccaaagatgctgagagggggaaactaaaacgcagagcaattgcaaccccaggaatgcaaataagggggtttgtatactttgttgagacactggcaagaagcatatgtgaaaagcttgaacaatcaggattgccagttggaggaaatgagaagaaagcaaagttagcaaatgttgtaaggaagatgatgaccaactcccaggacactgaaatttctttcaccataaccggagacaacacaaaatggaacgaaaatcaaaaccctagaatgttcttggccatgatcacatatataaccaaaaatcagcctgaatggttcagaaatattctaagtattgctccaataatgttttcaaacaagatggcgagactaggtaaggggtacatgtttgaaagcaagagtatgaaactgagaactcaaatacctgcagagatgctagccaacatagacttgaaatatttcaatgattcaactaaaaagaaaattgaaaaaatccgaccattattaatagatggaactgcatcattgagtcctggaatgatgatgggcatgttcaatatgttgagcaccgtattgggcgtctccattctgaatcttgggcaaaagagatacaccaagactacttactggtgggatggtcttcaatcgtctgatgattttgctttaattgtgaacgcacccaactatgcaggaattcaagctggagttgacaggttttatcgaacctgtaagctgctcggaattaatatgagcaaaaagaagtcttacataaacagaacaggtacctttgaattcacgagctttttctatcgttatgggtttgttgccaatttcagcatggagcttcctagttttggggtgtctggggtcaatgaatctgcagacatgagtattggagtcactgtcatcaaaaacaatatgataaacaatgaccttggcccagcaactgctcaaatggcccttcagttatttataaaagattacaggtacacttatcgatgccaccgaggtgacacacaaatacaaacccgaagatcatttgagataaagaaactatgggaccaaacccgctcaaaagctggactgttggtctctgatggaggccccaatttgtataacattagaaatctccatattcctgaagtttgcttgaaatgggagttgatggatgaggattaccaggggcgtttatgcaacccattaaacccgtttgtcagccataaagagattgaatcagtgaacagtgcagtgataatgccggcacatggtccagccaaaaatatggagtatgacgctgttgcaacaacacactcctgggtccccaaaagaaatcgatccattttgaacacgagccaaagggggatacttgaagatgagcaaatgtatcagaggtgctgcaatttatttgaaaaattcttcccaagtagctcatacagaagaccagttggaatatccagtatggtagaggctatggtctcaagagcccgaattgatgcacggattgatttcgaatctggaaggataaagaaagaggaatttgctgagatcatgaagatctgttccaccattgaagacctcagacggcaaaaatga

>H1N1_PB1_CY080999_A/Boston/89/2009

atggatgtcaatccgacattacttttcttaaaagtgccagcacaaaatgctataagcacaacttttccttatactggtgaccctccttacagccatgggacaggaacagggtacaccatggatacagtcaacaggacacaccagtactcagaaagaggaagatggacaaaaaataccgaaacgggagcaccgcaacttaacccaattgatggtcccttaccggaagacaatgaaccaagtggctatgcccaaacagattgtgtattagaagcaatggctttccttgaagaatcccatcccggtatctttgaaaactcttgtattgaaacaatggaggttgttcaacaaacaagagtggacaaactgacacaaggcagacagacctatgactggactctaaataggaaccagcctgctgccacagcattggcaaacactatagaagtattcagatcaaacggcctcatagcaaatgaatctggaaggctaatagacttccttaaagatgtaatggagtcgatggacagaggcgaagtagaggtcacaactcattttcaaagaaagaggagagtgagagacaatgtaactaaaaaaatggtgacccaaagaacaataggcaaaaagaaacataaattagacaaaagaagttacctaattagggcattaaccctgaacacaatgaccaaagatgctgagagggggaaactaaaacgcagagcaattgcaaccccaggaatgcaaataagggggtttgtatactttgttgagacactggcaagaagcatatgtgaaaagcttgaacaatcaggattgccagttggaggaaatgagaagaaagcaaagttagcaaatgttgtaaggaagatgatgaccaactcccaggacactgaaatttctttcaccataaccggagacaacacaaaatggaacgaaaatcaaaaccctagaatgttcttggccatgatcacatatataaccaaaaatcagcctgaatggttcagaaatattctaagtattgctccaataatgttttcaaacaagatggcgagactaggtaaggggtacatgtttgaaagcaagagtatgaaactgagaactcaaatacctgcagagatgctagccaacatagacttgaaatatttcaatgattcaactaaaaagaaaattgaaaaaatccgaccattattaatagatggaactgcatcattgagtcctggaatgatgatgggcatgttcaatatgttgagcaccgtattgggcgtctccattctgaatcttgggcaaaagagatacaccaagactacttactggtgggatggtcttcaatcgtctgatgattttgctttaattgtgaacgcacccaactatgcaggaattcaagctggagttgacaggttttatcgaacctgtaagctgctcggaattaatatgagcaaaaagaagtcttacataaacagaacaggtacctttgaattcacgagctttttctatcgttatgggtttgttgccaatttcagcatggagcttcctagttttggggtgtctggggtcaatgaatctgcagacatgagtattggagtcactgtcatcaaaaacaatatgataaacaatgaccttggcccagcaactgctcaaatggcccttcagttatttataaaagattacaggtacacttatcgatgccaccgaggtgacacacaaatacaaacccgaagatcatttgagataaagaaactatgggaccaaacccgctcaaaagctggactgttggtctctgatggaggccccaatttgtataacattagaaatctccatattcctgaagtttgcttgaaatgggagttgatggatgaggattaccaggggcgtttatgcaacccattaaacccgtttgtcagccataaagagattgaatcagtgaacagtgcagtgataatgccggcacatggtccagccaaaaatatggagtatgacgctgttgcaacaacacactcctgggtccccaaaagaaatcgatccattttgaacacgagccaaagggggatacttgaagatgagcaaatgtatcagaggtgctgcaatttatttgaaaaattcttcccaagtagctcatacagaagaccagttggaatatccagtatggtagaggctatggtctcaagagcccgaattgatgcacggattgatttcgaatctggaaggataaagaaagaggaatttgctgagatcatgaagatctgttccaccattgaagacctcagacggcaaaaatga

>H1N1_PB1_CY083070_A/Bogota/WRAIR0435T/2009

atggatgtcaatccgactctacttttcctaaaaattccagcgcaaaatgccataagcaccacattcccttatactggagatcctccatacagccatggaacaggaacaggatacaccatggacacagtaaacagaacacaccaatactcagaaaagggaaagtggacgacaaacacagagactggtgcaccccagctcaacccgattgatggaccactacctgaggataatgaaccaagtgggtatgcacaaacagactgtgttctagaggctatggctttccttgaagaatcccacccaggaatatttgagaattcatgccttgaaacaatggaagttgttcaacaaacaagagtagataaactaactcaaggtcgccagacttatgattggacattaaacagaaatcaaccggcagcaactgcattggccaacaccatagaagtctttagatcgaatggcctaacagctaatgagtcaggaaggctaatagatttcttaaaggatgtaatggaatcaatgaacaaagaggaaatagagataacaacccactttcaaagaaaaaggagagtaagagacaacatgaccaagaagatggtcacgcaaagaacaatagggaagaaaaaacaaagactgaataagagaggctatctaataagagcactgacattaaatacgatgaccaaagatgcagagagaggcaagttaaaaagaagggctatcgcaacacctgggatgcagattagaggtttcgtatactttgttgaaactttagctaggagcatttgcgaaaagcttgaacagtctgggctcccagtagggggcaatgaaaagaaggccaaactggcaaatgttgtgagaaagatgatgactaattcacaagacacagagatttctttcacaatcactggggacaacactaagtggaatgaaaatcaaaatcctcgaatgttcctggcgatgattacatatatcaccagaaatcaacccgagtggttcagaaacatcctgagcatggcacccataatgttctcaaacaaaatggcaagactagggaaagggtacatgttcgagagtaaaagaatgaagattcgaacacaaataccagcagaaatgctagcaagcattgacctgaagtacttcaatgaatcaacaaagaagaaaattgagaaaataaggcctcttctaatagatggcacagcatcactgagtcctgggatgatgatgggcatgttcaacatgctaagtacggtcttgggagtctcgatactgaatcttggacaaaagaaatacaccaagacaatatactggtgggatgggctccaatcatccgacgattttgctctcatagtgaatgcaccaaaccatgagggaatacaagcaggagtggacagattctacaggacctgcaagttagtgggaatcaacatgagcaaaaagaagtcctatataaataagacagggacatttgaattcacaagctttttttatcgctatggatttgtggctaattttagcatggagctacccagctttggagtgtctggagtaaatgaatcagctgacatgagtattggagtaacagtgataaagaacaacatgataaacaatgaccttggacctgcaacggcccagatggctcttcaattgttcatcaaagactacagatacacatataggtgccataggggagacacacaaattcagacgagaagatcatttgagttaaagaagctgtgggatcaaacccaatcaaaggtagggctattagtatcagatggaggaccaaacttatacaatatacggaatcttcacattcctgaagtctgcttaaaatgggagctaatggatgatgattatcggggaagactttgtaatcccctgaatccctttgtcagtcataaagagattgattctgtaaacaatgctgtggtaatgccagcccatggtccagccaaaagcatggaatatgatgccgttgcaactacacattcctggattcccaagaggaatcgttctattctcaacacaagccaaaggggaattcttgaggatgaacagatgtaccagaaatgctgcaatctattcgagaaatttttccctagcagttcatataggagaccggttggaatttctagcatggtggaggccatggtgtctagggcccggattgatgccagggtcgacttcgagtctggacggatcaagaaagaagagttctctgagatcatgaagatctgttccaccattgaagaactcagacggcaaaaataa

>H1N1_PB1_CY083461_A/Lima/WRAIR1688P/2009

atggatgtcaatccgactctacttttcctaaaaattccagcgcaaaatgccataagcaccacattcccttatactggagatcctccatacagccatggaacaggaacaggatacaccatggacacagtaaacagaacacaccaatactcagaaaagggaaagtggacgacaaacacagagactggtgcaccccagctcaacccgattgatggaccactacctgaggataatgaaccaagtgggtatgcacaaacagactgtgttctagaggctatggctttccttgaagaatcccacccaggaatatttgagaattcatgccttgaaacaatggaagttgttcaacaaacaagggtagataaactaactcaaggtcgccagacttatgattggacattaaacagaaatcaaccggcagcaactgcattggccaacaccatagaagtctttagatcgaatggcctaacagctaatgagtcaggaaggctaatagatttcttaaaggatgtaatggaatcaatgaacaaagaggaaatagagataacaacccactttcaaagaaaaaggagagtaagagacaacatgaccaagaagatggtcacgcaaagaacaatagggaagaaaaaacaaagactgaataagagaggctatctaataagagcactgacattaaatacgatgaccaaagatgcagagagaggcaagttaaaaagaagggctatcgcaacacctgggatgcagattagaggtttcgtatactttgttgaaactttagctaggagcatttgcgaaaagcttgaacagtctgggctcccagtagggggcaatgaaaagaaggccaaactggcaaatgttgtgagaaagatgatgactaattcacaagacacagagatttctttcacaatcactggggacaacactaagtggaatgaaaatcaaaatcctcgaatgttcctggcgatgattacatatatcaccagaaatcaacccgagtggttcagaaacatcctgagcatggcacccataatgttctcaaacaaaatggcaagactagggaaagggtacatgttcgagagtaaaagaatgaagattcgaacacaaataccagcagaaatgctagcaagcattgacctgaagtacttcaatgaatcaacaaagaagaaaattgagaaaataaggcctcttctaatagatggcacagcatcactgagtcctgggatgatgatgggcatgttcaacatgctaagtacggtcttgggagtctcgatactgaatcttggacaaaagaaatacaccaagacaatatactggtgggatgggctccaatcatccgacgattttgctctcatagtgaatgcaccaaaccatgagggaatacaagcaggagtggacagattctacaggacctgcaagttagtgggaatcaacatgagcaaaaagaagtcctatataaataagacagggacatttgaattcacaagctttttttatcgctatggatttgtggctaattttagcatggagctacccagctttggagtgtctggagtaaatgaatcagctgacatgagtattggagtaacagtgataaagaacaacatgataaacaatgaccttggacctgcaacggcccagatggctcttcaattgttcatcaaagactacagatacacatataggtgccataggggagacacacaaattcagacgagaagatcatttgagttaaagaagctgtgggatcaaacccaatcaaaggtagggctattagtatcagatggaggaccaaacttatacaatatacggaatcttcacattcctggtcatagcttaaaatgggagctaatggatgatgattatcggggaagactttgtaatcccctgaatccctttgtcagtcataaagagattgattctgtaaacaatgctgtggtaatgccagcccatggtccagccaaaagcatggaatatgatgccgttgcaactacacattcctggattcccaagaggaatcgttctattctcaacacaagccaaaggggaattcttgaggatgaacagatgtaccagaagtgctgcaatctattcgagaaatttttccctagcagttcatataggagaccggttggaatttctagcatggtggaggccatggtgtctagggcccggattgatgccagggtcgacttcgagtctggacggatcaagaaagaagagttctctgagatcatgaagatctgttccaccattgaagaactcagacggcaaaaataa

>H1N1_PB1_CY083477_A/Chicago/WRAIR1690P/2009

atggatgtcaatccgactctacttttcctaaaaattccagcgcaaaatgccataagcaccacattcccttatactggagatcctccatacagccatggaacaggaacaggatacaccatggacacagtaaacagaacacaccaatactcagaaaagggaaagtggacgacaaacacagagactggtgcaccccagctcaacccgattgatggaccactacctgaggataatgaaccaagtgggtatgcacaaacagactgtgttctagaggctatggctttccttgaagaatcccacccgggaatatttgagaattcatgccttgaaacaatggaagttgttcaacaaacaagggtagataaactaactcaaggtcgccagacttatgattggacattaaacagaaatcaaccggcagcaactgcattggccaacaccatagaagtctttagatcgaatggcctaacagctaatgagtcaggaaggctaatagatttcttaaaggatgtaatggaatcaatgaacaaagaggaaatagagataacaacccactttcaaagaaaaaggagagtaagagacaacatgaccaagaagatggtcacgcaaagaacaatagggaagaaaaaacaaagactgaataagagaggctatctaataagagcactgacattaaatacgatgaccaaagatgcagagagaggcaagttaaaaagaagggctatcgcaacacctgggatgcagattagaggtttcgtatactttgttgaaactttagctaggagtatttgcgaaaagcttgaacagtctgggctcccagtagggggcaatgaaaagaaggccaaactggcaaatgttgtgagaaagatgatgactaattcacaagacacagagatttctttcacaatcactggggacaacactaagtggaatgaaaatcaaaatcctcgaatgttcctggcgatgattacatatatcaccagaaatcaacccgagtggttcagaaacatcctgagcatggcacccataatgttctcaaacaaaatggcaagactagggaaagggtacatgttcgagagtaaaagaatgaagattcgaacacaaataccagcagaaatgctagcaagcattgacctgaagtacttcaatgaatcaacaaagaagaaaattgagaaaataaggcctcttctaatagatggcacagcatcactgagtcctgggatgatgatgggcatgttcaacatgctaagtacagtcttgggagtctcgatactgaaccttggacaaaagaaatacaccaagacaatatactggtgggatgggctccaatcatccgacgattttgctctcatagtgaatgcaccaaaccatgaggggatacaagcaggagtggacagattctacaggacctgcaagttagtgggaatcaacatgagcaaaaagaagtcctatataaataagacagggacatttgaattcacaagctttttttatcgctatggatttgtggctaattttagcatggagctacccagctttggagtgtctggagtaaatgaatcagctgacatgagtattggagtaacagtgataaagaacaacatgataaacaatgaccttggacctgcaacggcccagatggctcttcaattgttcatcaaagactacagatacacatacaggtgccataggggagacacacaaattcagacgagaagatcatttgagttaaagaagctgtgggatcaaacccaatcaaaggtagggctattagtatcagatggaggaccaaacttatacaatatacggaatcttcacattcctgaagtctgcttaaaatgggagctaatggatgatgattatcggggaagactttgtaatcccctgaatccctttgtcagtcataaagagattgattctgtaaacaatgctgtggtaatgccagcccatggtccagccaaaagcatggaatatgatgccgttgcaactacacattcctggattcccaagaggaatcgttctattctcaacacaagccaaaggggaattcttgaggatgaacagatgtaccagaagtgctgcaatctattcgagaaatttttccctagcagttcatataggagaccggttggaatttctagcatggtggaggccatggtgtctagggcccggattgatgccagggtcgacttcgagtctggacggatcaagaaagaagagttctctgagatcatgaagatctgttccaccattgaagaactcagacggcaaaaataa

>H1N1_PB1_CY083667_A/San Diego/INS41/2009

atggatgtcaatccgactctacttttcctaaaaattccagcgcaaaatgccataagcaccacattcccttatactggagatcctccatacagccatggaacaggaacaggatacaccatggacacagtaaacagaacacaccaatactcagaaaagggaaagtggacgacaaacacagagactggtgcaccccagctcaacccgattgatggaccactacctgaggataatgaaccaagtgggtatgcacaaacagactgtgttctagaggctatggctttccttgaagaatcccacccaggaatatttgagaattcatgccttgaaacaatggaagttgttcaacaaacaagggtagataaactaactcaaggtcgccagacttatgattggacattaaacagaaatcaaccggcagcaactgcattggccaacaccatagaagtctttagatcgaatggcctaacagctaatgagtcaggaaggctaatagatttcttaaaggatgtaatggaatcaatgaacaaagaggaaatagagataacaacccactttcaaagaaaaaggagagtaagagacaacatgaccaagaagatggtcacgcaaagaacaatagggaagaaaaaacaaagactgaataagagaggctatctaataagagcactgacattaaatacgatgaccaaagatgcagagagaggcaagttaaaaagaagggctatcgcaacacctgggatgcagattagaggtttcgtatactttgttgaaactttagctaggagcatttgcgaaaagcttgaacagtctgggctcccagtagggggcaatgaaaagaaggccaaactggcaaatgttgtgagaaagatgatgactaattcacaagacacagagatttctttcacaatcactggggacaacactaagtggaatgaaaatcaaaatcctcgaatgttcctggcgatgattacatatatcaccagaaatcaaccagagtggttcagaaacatcctgagcatggcacccataatgttctcaaacaaaatggcaagactagggaaagggtacatgttcgagagtaaaagaatgaagattcgaacacaaataccagcagaaatgctagcaagcattgacctgaagtacttcaatgaatcaacaaagaggaaaattgagaaaataaggcctcttctaatagatggcacagcatcactgagtcctgggatgatgatgggcatgttcaacatgctaagtacggtcttgggagtctcgatactgaatcttggacaaaagaaatacaccaagacaatatattggtgggatgggctccaatcatccgacgattttgctctcatagtgaatgcaccaaaccatgagggaatacaagcaggagtggacagattctacaggacctgcaagttagtgggaatcaacatgagcaaaaagaagtcctatataaataagacagggacatttgaattcacaagctttttttatcgctatggatttgtggctaattttagcatggagctacccagctttggagtgtctggagtaaatgaatcagctgacatgagtattggagtaacagtgataaagaacaacatgataaacaatgaccttggacctgcaacggcccagatggctcttcaattgttcatcaaagactacagatacacatataggtgccataggggggacacacaaattcagacgagaaggtcatttgagttaaagaagctgtgggatcaaacccaatcaaaggtagggctattagtatcagatggaggaccaaacttatacaatatacggaatcttcacattcctgaagtctgcttaaaatgggagctaatggatgatgattatcggggaagactttgtaatcccctgaatccctttgtcagtcataaagagattgattctgtaaacaatgctgtggtaatgccagcccatggtccagccaaaagcatggaatatgatgccgttgcaactacacattcctggattcccaagaggaatcgttctattctcaacacaagccaaaggggaattcttgaggatgaacagatgtaccagaagtgctgcaatctattcgagaaatttttccctagcagttcatataggagaccggttggaatttctagcatggtggaggccatggtgtctagggcccggattgatgccagggtcgacttcgagtctggacggatcaagaaagaagagttctctgagatcatgaagatctgttccaccattgaagaactcagacggcaaaaataa

>H1N1_PB1_CY083860_A/Pensacola/INS39/2009

atggatgtcaatccgactctacttttcctaaaaattccagcgcaaaatgccataagcaccacattcccttatactggagatcctccatacagccatggaacaggaacaggatacaccatggacacagtaaacagaacacaccaatactcagaaaagggaaagtggacgacaaacacagagactggtgcaccccagctcaacccgattgatggaccactacctgaggataatgaaccaagtgggtatgcacaaacagactgtgttctagaggctatggctttccttgaagaatcccacccaggaatatttgagaattcatgccttgaaacaatggaagttgttcaacaaacaagggtagataaactaactcaaggtcgccagacttatgattggacattaaacagaaatcaaccggcagcaactgcattggccaacaccatagaagtctttagatcgaatggcctaacagctaatgagtcaggaaggctaatagatttcttaaaggatgtaatggaatcaatgaacaaagaggaaatagagataacaacccactttcaaagaaaaaggagagtaagagacaacatgaccaagaagatggtcacgcaaagaacaatagggaagaaaaaacaaagactgaataagagaggctatctaataagagcactgacattaaatacgatgaccaaagatgcagagagaggcaagttaaaaagaagggctatcgcaacacctgggatgcagattagaggtttcgtatactttgttgaaactttagctaggagcatttgcgaaaagcttgaacagtctgggctcccagtagggggcaatgaaaagaaggccaaactggcaaatgttgtgagaaagatgatgactaattcacaagacacagagatttctttcacaatcactggggacaacactaagtggaatgaaaatcaaaatcctcgaatgttcctggcgatgattacatatatcaccagaaatcaacccgagtggttcagaaacatcctgagcatggcacccataatgttctcaaacaaaatggcaagactagggaaagggtacatgttcgagagtaaaagaatgaagattcgaacacaaataccagcagaaatgctagcaagcattgacctgaagtacttcaatgaatcaacaaagaagaaaattgagaaaataaggcctcttctaatagatggcacagcatcactgagtcctgggatgatgatgggcatgttcaacatgctaagtacggtcttgggagtctcgatactgaatcttggacaaaagaaatacaccaagacaatatattggtgggatgggctccaatcatccgacgattttgctctcatagtgaatgcaccaaaccatgagggaatacaagcaggagtggacagattctacaggacctgcaagttagtgggaatcaacatgagcaaaaagaagtcctatataaataagacagggacatttgaattcacaagctttttttatcgctatggatttgtggctaatttcagcatggagctacccagctttggagtgtctggagtaaatgaatcagctgacatgagtattggagtaacagtgataaagaacaacatgataaacaatgaccttggacctgcaacggcccaaatggctcttcaattgttcatcaaagactacagatacacatataggtgccataggggagacacacaaattcagacgagaagatcatttgagttaaagaagctgtgggatcaaacccaatcaaaggtagggctattagtatcagatggaggaccaaacttatacaatatacggaatcttcacattcctgaagtctgcttaaaatgggagctaatggatggtgattatcggggaagactttgtaatcccctgaatccctttgtcagtcataaagagattgattctgtaaacaatgctgtggtaatgccagcccatggtccagccaaaagcatggaatatgatgccgttgcaactacacattcctggattcccaagaggaatcgttctattctcaacacaagccaaaggggaattcttgaggatgaacagatgtaccagaagtgctgcaatctattcgagaaatttttccctagcagttcatataggagaccggttggaatttctagcatggtggaggccatggtgtctagggcccggattgatgccagggtcgacttcgagtctggacggatcaagaaagaagagttctctgagatcatgaagatctgttccaccattgaagaactcagacggcaaaaataa

>H1N1_PB1_CY083916_A/Aalborg/INS132/2009

atggatgtcaatccgactctacttttcctaaaaattccagcgcaaaatgccataagcaccacattcccttatactggagatcctccatacagccatggaacaggaacaggatacaccatggacacagtaaacagaacacaccaatactcagaaaagggaaagtggacgacaaacacagagactggtgcaccccagctcaacccgattgatggaccactacctgaggataatgaaccaagtgggtatgcacaaacagactgtgttctagaggctatggctttccttgaagaaacccacccaggaatatttgagaattcatgccttgaaacaatggaagttgttcaacaaacaagggtagataaactaactcaaggtcgccagacttatgattggacattaaacagaaatcaaccggcagcaactgcattggccaacaccatagaagtctttagatcgaatggcctaacagctaatgagtcaggaaggctaatagatttcttaaaggatgtaatggaatcaatgaacaaagaggaaatagagataacaacccactttcaaagaaaaaggagagtaagagacaacatgaccaagaagatggtcacgcaaagaacaatagggaagaaaaaacaaagactgaataagagaggctatctaataagagcactgacattaaatacgatgaccaaagatgcagagagaggcaagttaaaaagaagggctatcgcaacacctgggatgcagattagaggtttcgtatactttgttgaaactttagctaggagcatttgcgaaaagcttgaacagtctgggctcccagtagggggcaatgaaaagaaggccaaactggcaaatgttgtgagaaagatgatgactaattcacaagacacagagatttctttcacaatcactggggacaacactaagtggaatgaaaatcaaaatcctcgaatgttcctggcgatgattacatatatcaccagaaatcaacccgagtggttcagaaacatcctgagcatggcacccataatgttctcaaacaaaatggcaagactagggaaagggtacatgttcgagagtaaaagaatgaagattcgaacacaaataccagcagaaatgctagcaagcattgacctgaagtacttcaatgaatcaacaaagaagaaaattgagaaaataaggcctcttctaatagatggcacagcatcactgagtcctgggatgatgatgggcatgttcaacatgctaagtacggtattgggagtctcgatactgaatcttggacaaaagaaatacaccaagacaatatactggtgggatgggctccaatcatccgacgattttgctctcatagtgaatgcaccaaaccatgagggtatacaagcaggagtggacagattctacaggacctgcaagttagtgggaatcaacatgagcaaaaagaagtcctatataaataagacagggacatttgaattcacaagctttttttatcgctatggatttgtggctaattttagcatggagctacccagctttggagtgtctggagtaaatgaatcagctgacatgagtattggagtaacagtgataaagaacaacatgataaacaatgaccttggacctgcaacggcccagatggctcttcaattgttcatcaaagactacagatacacatataggtgccataggggagacacacaaattcagacgagaagatcatttgagttaaagaagctgtgggatcaaacccaatcaaaggtagggctattagtatcagatggaggaccaaacttatacaatatacggaatcttcacattcctgaagtctgcttaaaatgggagctaatggatgatgattatcggggaagactttgtaatcccctgaatccctttgtcagtcataaagagattgattctgtaaacaatgctgtggtaatgccagcccatggtccagccaaaagcatggaatatgatgccgttgcaactacacattcctggattcccaagaggaatcgttctattctcaacacaagccaaaggggaattcttgaggatgaacagatgtaccagaagtgctgcaatctattcgagaaatttttccctagcagttcatataggagaccggttggaatttctagcatggtggaggccatggtgtctagggcccggattgatgccagggtcgacttcgagtctggacggatcaagaaagaagagttctctgagatcatgaagatctgttccaccattgaagaactcagacggcaaaaataa

>H1N1_PB1_CY088619_A/Frankfurt/INS93/2009

atggatgtcaatccgactctacttttcctaaaaattccagcgcaaaatgccataagcaccacattcccttatactggagatcctccatacagccatggaacaggaacaggatacaccatggacacagtaaacagaacacaccaatactcagaaaagggaaagtggacgacaaacacagagactggtgcaccccagctcaacccgattgatggaccactacctgaggataatgaaccaagtgggtatgcacaaacagactgtgttctagaggctatggctttccttgaagaatcccacccaggaatatttgagaattcatgccttgaaacaatggaagttgttcaacaaacaagggtagataaactaactcaaggtcgccagacttatgattggacattaaacagaaatcaaccggcagcaactgcattggccaacaccatagaagtctttagatcgaatggcctaacagctaatgagtcaggaaggctaatagatttcttaaaggatgtaatggaatcaatgaacaaagaggaaatagagataacaacccactttcaaagaaaaaggagagtaagagacaacatgaccaagaagatggtcacgcaaagaacaatagggaagaaaaaacaaagactgaataagagaggctatctaataagagcactgacattaaatacgatgaccaaagatgcagagagaggcaagttaaaaagaagggctatcgcaacacctgggatgcagattagaggtttcgtatactttgttgaaactttagctaggagcatttgcgaaaagcttgaacagtctgggctcccagtagggggcaatgaaaagaaggccaaactggcaaatgttgtgagaaagatgatgactaattcacaagacacagagatttctttcacaatcactggggacaacactaagtggaatgaaaatcaaaatcctcgaatgttcctggcgatgattacatatatcaccagaaatcaacccgagtggttcagaaacatcctgagcatggcacccataatgttctcaaacaaaatggcaagactagggaaagggtacatgttcgagagtaaaagaatgaagattcgaacacaaataccagcagaaatgctagcaagcattgacctgaagtacttcaatgaatcaacaaagaagaaaattgagaaaataaggcctcttctaatagatggcacagcatcactgagtcctgggatgatgatgggcatgttcaacatgctaagtacggtcttgggagtctcgatactgaatcttggacaaaagaaatacaccaagacaatatactggtgggatgggctccaatcatccgacgattttgctctcatagtgaatgcaccaaaccatgagggaatacaagcaggagtggacagattctacaggacctgcaagttagtgggaatcaacatgagcaaaaagaagtcctatataaataagacagggacatttgaattcacaagctttttttatcgctatggatttgtggctaattttagcatggagctacccagctttggagtgtctggagtaaatgaatcagctgacatgagtattggagtaacagtgataaagaacaacatgataaacaatgaccttggacctgcaacggcccagatggctcttcaattgttcatcaaagactacagatacacatataggtgccataagggagacacacaaattcagacgagaagatcatttgagttaaagaagctgtgggatcaaacccaatcaaaggtagggctattagtatcagatggaggaccaaacttatacaatatacggaatcttcacattcctgaagtctgcttaaaatgggagctaatggatgatgattatcggggaagactttgtaatcccctgaatccctttgtcagtcataaagagattgattctgtaaacaatgctgtggtaatgccagcccatggtccagccaaaagcatggaatatgatgccgttgcaactacacattcctggattcccaagaggaatcgttctattctcaacacaagccaaaggggaattcttgaggatgaacagatgtaccagaagtgctgcaatctattcgagaaatttttccctagcagttcatataggagaccggttggaatttctagcatggtggaggccatggtgtctagggcccggattgatgccagggtcgacttcgagtctggacggatcaagaaagaagagttctctgagatcatgaagatctgttccaccattgaagaactcagacggcaaaaataa

>H1N1_PB1_CY089225_A/Boston/630/2009

atggatgtcaatccgactctacttttcctaaaaattccagcgcaaaatgccataagcaccacattcccttatactggagatcctccatacagccatggaacaggaacaggatacaccatggacacagtaaacagaacacaccaatactcagaaaagggaaagtggacgacaaacacagagactggtgcaccccagctcaacccgattgatggaccactacctgaggataatgaaccaagtgggtatgcacaaacagactgtgttctagaggctatggctttccttgaagaatcccacccaggaatatttgagaattcatgccttgaaacaatggaagttgttcaacaaacaagggtagataaactaactcaaggtcgccagacttatgattggacattaaacagaaatcaaccggcagcaactgcattggccaacaccatagaagtctttagatcgaatggcctaacagctaatgagtcaggaaggctaatagatttcttaaaggatgtaatggaatcaatgaacaaagaggaaatagagataacaacccactttcaaagaaaaaggagagtaagagacaacatgaccaagaagatggtcacgcaaagaacaatagggaagaaaaaacaaagactgaataagagaggctatctaataagagcactgacattaaatacgatgaccaaagatgcagagagaggcaagttaaaaagaagggctatcgcaacacctgggatgcagattagaggtttcgtatactttgttgaaactttagctaggagcatttgcgaaaagcttgaacagtctgggctcccagtagggggcaatgaaaagaaggccaaactggcaaatgttgtgagaaagatgatgactaattcacaagacacagagatttctttcacaatcactggggacaacactaagtggaatgaaaatcaaaatcctcgaatgttcctggcgatgattacatatatcaccagaaatcaacccgagtggttcagaaacatcctgagcatggcacccataatgttctcaaacaaaatggcaagactagggaaagggtacatgttcgagagtaaaagaatgaagattcgaacacaaataccagcagaaatgctagcaagcattgacctgaagtacttcaatgaatcaacaaagaagaaaattgagaaaataaggcctcttctaatagatggcacagcatcactgagtcctgggatgatgatgggcatgttcaacatgctaagtacggtcttgggagtctcgatactgaatcttggacaaaagaaatacaccaagacaatatactggtgggatgggctccaatcatccgacgattttgctctcatagtgaatgcaccaaaccatgagggaatacaagcaggagtggacagattctacaggacctgcaagttagtgggaatcaacatgagcaaaaagaagtcctatataaataagacagggacatttgaattcacaagctttttttatcgctatgggtttgtggctaattttagcatggagctacccagctttggagtgtctggagtaaatgaatcagctgacatgagtattggagtaacagtgataaagaacaacatgataaacaatgaccttggacctgcaacggcccagatggctcttcaattgttcatcaaagactacagatacacatataggtgccataagggagacacacaaattcagacgagaagatcatttgagttaaagaagctgtgggatcaaacccaatcaaaggtagggctattagtatcagatggaggaccaaacttatacaatatacgaaatcttcacattcctgaagtctgcttaaaatgggagctaatggatgatgattatcggggaagactttgtaatcccctgaatccctttgtcagtcataaagagattgattctgtaaacaatgctgtggtaatgccagcccatggtccagccaaaagcatggaatatgatgctgttgcaactacacattcctggattcccaagaggaatcgttctattctcaacacaagccaaaggggaattcttgaggatgaacagatgtaccagaagtgctgcaatctattcgagaaatttttccctagcagttcatataggagaccggttggaatttctagcatggtggaggccatggtgtctagggcccggattgatgccagggtcgacttcgagtctggacggatcaagaaagaagagttctctgagatcatgaagatctgttccaccattgaagaactcagacggcaaaaataa

>H1N1_PB1_CY089313_A/Boston/673/2009

atggatgtcaatccgactctacttttcctaaaaattccagcgcaaaatgccataagcaccacattcccttatactggagatcctccatacagccatggaacaggaacaggatacaccatggacacagtaaacagaacacaccaatactcagaaaggggaaagtggacgacaaacacagagactggtgcaccccagctcaacccgattgatggaccactacctgaggataatgaaccaagtgggtatgcacaaacagattgtgttctagaggctatggctttccttgaagaatcccacccaggaatatttgagaattcatgccttgaaacaatggaagttgttcaacaaacaagggtagataaactaactcaaggtcgccagacttatgattggacattaaacagaaatcaaccggcagcaactgcattggccaacaccatagaagtctttagatcgaatggcctaacagctaatgagtcaggaaggctaatagatttcttaaaggatgtaatggaatcaatgaacaaagaggaaatagagataacaacccactttcaaagaaaaaggagagtaagagacaacatgaccaagaagatggtcacgcaaagaacaatagggaagaaaaaacaaagactgaataagagaggctatctaataagagcactgacattaaatacgatgaccaaagatgcagagagaggcaagttaaaaagaagggctatcgcaacacctgggatgcagattagaggtttcgtatactttgttgaaactttagctaggagcatttgcgaaaagcttgaacagtctgggctcccagtagggggcaatgaaaagaaggccaaactggcaaatgttgtgagaaagatgatgactaattcacaagacacagagatttctttcacaatcactggggacaacactaagtggaatgaaaatcaaaatcctcgaatgttcctggcgatgattacatatatcaccagaaatcaacccgagtggttcagaaacatcctgagcatggcacccataatgttctcaaacaaaatggcaagactagggaaagggtacatgttcgagagtaaaagaatgaagattcgaacacaaataccagcagaaatgctagcaagcattgacctgaagtacttcaatgaatcaacaaagaagaaaattgagaaaataaggcctcttctaatagatggcacagcatcactgagtcctgggatgatgatgggcatgttcaacatgctaagtacggtcttgggagtctcgatactgaatcttggacaaaagaaatacaccaagacaatatactggtgggatgggctccaatcatccgacgattttgctctcatagtgaatgcaccaaaccatgagggaatacaagcaggagtggacagattctacaggacctgcaagttagtgggaatcaacatgagcaaaaagaagtcctatataaataagacagggacatttgaattcacaagctttttttatcgctatggatttgtggctaattttagcatggagctacccagctttggagtgtctggagtaaatgaatcagctgacatgagtattggagtaacagtgataaagaacaacatgataaacaatgaccttggacctgcaacggcccagatggctcttcaactgttcatcaaagactacagatacacatataggtgccataggggagacacacaaattcagacgagaagatcatttgagttaaagaagctgtgggatcaaacccaatcaaaggtagggctattagtatcagatggaggaccaaacttatacaatatacggaatcttcacattcctgaagtctgcttaaaatgggagctaatggatgatgattatcggggaagactttgtaatcccctgaatccctttgtcagtcataaagagattgattctgtaaacaatgctgtggtaatgccagcccatggtccagccaaaagcatggaatatgatgccgttgcaactacacattcctggattcccaagaggaatcgttctattctcaacacaagccaacggggaattcttgaggatgaacagatgtaccagaagtgctgcaatctattcgagaaatttttccctagcagttcatataggagaccggttggaatttctagcatggtggaggccatggtgtctagggcccggattgatgccagggtcgacttcgagtctggacggatcgggaaggaagagttctctgagatcatgaagatctgttccaccattgaagaactcagacggcaaaaataa

>H1N1_PB1_CY089715_A/Boston/19/2009

atggatgtcaatccgacattacttttcttaaaagtgccagcacaaaatgctataagcacaacttttccttatactggtgaccctccttacagccatgggacaggaacagggtacaccatggatacagtcaayaggacacaccagtactcagaaagaggaagatggacaaaaaataccgaaacgggagcaccgcaacttaacccaattgatggycccttaccggaagacaatgaaccaagtggctatgcccaaacagattgtgtattagaagcaatggctttccttgaagaatcccatcccggtatctttgaaaactcttgtattgaaacaatggaggttgttcaacaaacaagggtggacaaactgacacaaggcagacagacctatgactggactctaaataggaaccagcctgctgccacagcattggcaaacactatagaagtattcagatcaaacggcctcatagcaaatgaatctggaaggctaatagacttccttaaagatgtaatggagtcgatggacagaggcgaagtagaggtcacaactcattttcaaagaaagaggagagtgagagacaatgtaactaaaaaaatggtgacccaaagaacaataggcaaaaagaaacataaattagacaaaagaagttacctaattagggcattaaccctgaacacaatgaccaaagatgctgagagggggaaactaaaacgcagagcaattgcaaccccaggaatgcaaataagggggtttgtatactttgttgagacactggcaagaagcatatgtgaaaagcttgaacaatcaggattgccagttggaggaaatgagaagaaagcaaagttagcaaatgttgtaaggaagatgatgaccagctcccaggacactgaaatttctttcaccataaccggagacaacacaaaatggaacgaaaatcaaaaccctagaatgttcttggccatgatcacatatataaccaaaaatcagcctgaatggttcagaaatattctaagtattgctccaataatgttttcaaacaagatggcgagactaggtaaggggtacatgtttgaaagcaagagtatgaaactgagaactcaaatacctgcagagatgctagccaacatagacttgaaatatttcaatgattcaactaaaaagaaaattgaaaaaatccgaccattattaatagatggaactgcatcattgagtcctggaatgatgatgggcatgttcaatatgttgagcaccgtcttgggcgtctccattctgaatcttgggcaaaagagatacaccaagactacttactggtgggatggtcttcaatcgtctgatgattttgctttaattgtgaacgcacccaactatgcaggaattcaagctggagttgacaggttttatcgaacctgtaagctgctcggaattaatatgagcaaaaagaagtcttacataaacagaacaggtacctttgaattcacgagctttttctatcgttatgggtttgttgccaatttcagcatggagcttcctagttttggggtgtctggggtcaatgaatctgcagacatgagtattggagtcactgtcatcaaaaacaatatgataaacaatgaccttggcccagcaactgctcaaatggcccttcagttatttataaaagattacaggtacacttatcgatgccaccgaggtgacacacaaatacaaacccgaagatcatttgagataaagaaactatgggatcaaacccgctcaaaagctggactgttggtctctgatggaggccccaatttgtataacattagaaatctccatattcctgaagtttgcttgaaatgggagttgatggatgaggattaccaggggcgtttatgcaacccattaaacccgtttgtcagccataaagagattgaatcagtgaacagtgcagtgataatgccggcacatggtccagccaaaaatatggagtatgacgctgttgcaacaacacactcctgggtccccaaaagaaatcgatccattttgaacacgagccaaagggggatacttgaagatgagcaaatgtatcagaggtgctgcaatttatttgaaaaattcttcccaagtagctcatacagaagaccagttggaatatccagtatggtagaggctatggtctcaagagcccgaattgatgcacggattgatttcgaatctggaaggataaagaaagaggaatttgctgagatcatgaagatctgttccaccattgaagacctcagacggcaaaaatga

>H1N1_PB1_HQ853556_A/KOL/611/2007

atggatgtcaatccgactctactgttcataaaggttccagcgcaaaatgccataagcacaacattcccttatactggagatcctccatacagccatgggacaggaacagggtacaccatggacacagtcaacagaacacaccaatattcagagaaggggaagtggacgacaaatacagaaactggggcaccccaactcaacccaattgatggaccactacctgaggataatgaaccaagtggatatgcacaaacagactgtgtcctggaggctatggccttccttgaagaatcccacccaggtatctttgagaactcatgccttgaaacaatggaagccgttcaacagacaagggtggacagactaacccaaggtcgccagacttatgattggacattaaacagaaatcaaccggcagcaactgcattagccaacaccatagaagtttttagatcgaacggattaacagctaatgaatcaggaaggctaatagatttcctcaaggatgtgatggaatcaatggataaagaggaaatggagataacaacacactttcaaagaaaaaggagagtaagggacaacatgaccaagaaaatggtcacacaaagaacaatagggaagaaaaaacaaagagtgaataagagaggctacctaataagagctttgacattgaacacgatgaccaaagatgcagagagaggcaaattaaaaagaagggctattgcaacacccgggatgcaaattagagggttcgtgtacttcgttgaaactttagctagaagcatttgcgaaaagcttgaacagtctggacttccggttgggggtaatgaaaagaaggccaaactggcaaatgttgtgagaaaaatgatgactaattcacaagacacagagctttctttcacaatcactggggacaacactaagtggaatgaaaatcaaaaccctcgaatgtttttggcgatgattacatacatcacaaaaaatcaacctgagtggttcagaaacatcctgagcatcgcaccaataatgttctcaaacaaaatggcaagactgggaaaaggatacatgttcgagagtaagagaatgaagctccggacacaaatacctgcagaaatgctagcaagcattgacctgaagtatttcaatgaatcaacaaggaagaaaattgagaaaataaggcctcttctaatagatggcacagcatcattgagccctggaatgatgatgggcatgttcaacatgctaagtacggttttaggagtctcgatactgaatcttggacaaaagaaatacaccaagacaacatactggtgggatgggctccaatcctccgacgattttgccctcatagtgaatgcaccaaatcatgagggaatacaagcaggagtggatagattctacaggacctgcaagttagtgggaatcaacatgagcaaaaagaagtcctatataaataaaacagggacatttgaattcactagctttttttatcgatatggatttgtggctaattttagcatggagctgcccagttttggagtgtctggaataaacgagtcagctgatatgagcattggagtaacagtgataaagaacaacatgataaacaatgaccttggaccagcaacagcccaaatggctctccaattgttcatcaaagattacagatacacatataggtgccatagaggagacacacaaatccagacgagaagatcattcgagctaaagaagctgtgggatcaaacccaatcaagggcaggactattggtatcagatgggggaccaaacttatacaatatccggaatcttcacatccctgaagtctgcttaaagtgggagctgatggatgagaattatcggggaagactttgtaatcccctgaacccctttgtcagccataaagaaattgagtctgtaaacaatgctgtagtaatgccagcccatggtccggccaaaagtatggaatatgatgccgttgcaactacacactcctggattcccaagaggaaccgctctattctaaacacaagccaaaggggaattcttgaggatgaacagatgtaccagaagtgctgcaacttgttcgagaaatttttccctagtagttcatataggagaccgattggaatttctagcatggtggaggccatggtgtctagggcccggattgatgccagaattgacttcgaatctggacggattaagaaggaagagttctctgagatcatgaagatctgttccaccattgaagaactcagacggcaaaaataa

>H1N1_PB1_CY092191_A/Australia/17/2009

atggatgtcaatccgactctacttttcctaaaaattccagcgcaaaatgccataagcaccacattcccttatactggagatcctccatacagccatggaacaggaacaggatacaccatggacacagtaaacagaacacaccaatactcagaaaagggaaagtggacgacaaacacagagactggtgcaccccagctcaacccgattgatggaccactacctgaggataatgaaccaagtgggtatgcacaaacagactgtgttctagaggctatggctttccttgaagaatcccacccaggaatatttgagaattcatgccttgaaacaatggaagttgttcaacaaacaagggtagataaactaactcaaggtcgccagacttatgattggacattaaacagaaatcaaccggcagcaactgcattggccaacaccatagaagtttttagatcgaatggcctaacagctaatgagtcaggaaggctaatagatttcttaaaggatgtaatggaatcaatgaacaaagaggaaatagagataacaacccactttcaaagaaaaaggagagtaagagacaacatgaccaagaagatggtcacgcaaagaacaatagggaagaaaaaacaaagactgaataagagaggctatctaataagagcactgacattaaatacgatgaccaaagatgcagagagaggcaagttaaaaagaagggctatcgcaacacctgggatgcagattagaggtttcgtatactttgttgaaactttagctaggagcatttgcgaaaagcttgaacagtctgggctcccagtagggggcaatgaaaagaaggccaaactggcaaatgttgtgagaaagatgatgactaattcacaagacacagagatttctttcacaatcactggggacaacactaagtggaatgaaaatcaaaatcctcgaatgttcctggcgatgattacatatatcaccagaaatcaacccgagtggttcagaaacatcctgagcatggcacccataatgttctcaaacaaaatggcaagactagggaaagggtacatgttcgagagtaaaagaatgaagattcgaacacaaataccagcagaaatgctagcaagcattgacctgaagtacttcaatgaatcaacaaagaagaaaattgagaaaataaggcctcttctaatagatggcacagcatcactgagtcctgggatgatgatgggcatgttcaacatgctaagtacggtcttgggagtctcgatactgaatcttggacaaaagaaatacaccaagacaatatactggtgggatgggctccaatcatccgacgattttgctctcatagtgaatgcaccaaaccatgagggaatacaagcaggagtggacagattctacaggacctgcaagttagtgggaatcaacatgagcaaaaagaagtcctatataaataagacagggacatttgaattcacaagctttttttatcgctatggatttgtggctaattttagcatggagctacccagctttggagtgtctggagtaaatgaatcagctgacatgagtattggagtaacagtgataaagaacaacatgataaacaatgaccttggacctgcaacggcccagatggctcttcaattgttcatcaaagactacagatacacatataggtgccataggggagacacacaaattcagacgagaagatcatttgagttaaagaagctgtgggatcaaacccaatcaaaggtagggctattagtatcagatggaggaccaaacttatacaatatacggaatcttcacattcctgaagtctgcttaaaatgggagctaatggatgatgattatcggggaagactttgtaatcccctgaatccctttgtcagtcataaagagattgattctgtaaacaatgctgtggtaatgccagcccatggtccagccaaaagcatggaatatgatgccgttgcaactacacattcctggattcccaagaggaatcgttctattctcaacacaagccaaaggggaattcttgaggatgaacagatgtaccagaagtgctgcaatctattcgagaaatttttccctagcagttcatataggagaccggttggaatttctagcatggtggaggccatggtgtctagggcccggattgatgccagggtcgacttcgagtctggacggatcaagaaagaagagttctctgagatcatgaagatctgttccaccattgaagaactcagacggcaaaaataa

>H1N1_PB1_CY092351_A/California/VRDL191/2009

atggatgtcaatccgacattacttttcttaaaagtgccagcacaaaatgctataagcacaacttttccttatactggtgaccctccttacagccatgggacaggaacagggtacaccatggatacagtcaacaggacacatcagtactcagaaagaggaagatggacaaaaaataccgaaacgggagcaccgcaacttaacccaattgatggtcccttaccggaagacaatgaaccaagtggctatgcccaaacagattgtgtattagaagcaatggctttccttgaagaatcccatcccggtatctttgaaaactcttgtattgaaacaatggaggttgttcaacaaacaagggtggacaaactgacacaaggcagacagacctatgactggactctaaataggaaccagcctgctgccacagcattggcaaacactatagaagtattcagatcaaacggcctcatagcaaatgaatctggaaggctaatagacttccttaaagatgtaatggagtcgatggacagaggcgaagtagaggtcacaactcattttcaaagaaagaggagagtgagagacaatgtaactaaaaaaatggtgacccaaagaacaataggcaaaaagaaacataaattagacaaaagaagttacctaattagggcattaaccctgaatacaatgaccaaagatgctgagagggggaaactaaaacgcagagcaattgcaaccccaggaatgcaaataagggggtttgtatactttgttgagacactggcaagaagcatatgtgaaaagcttgaacaatcaggattgccagttggaggaaatgagaagaaagcaaagttagcaaatgttgtaaggaagatgatgaccaactcccaggacactgaaatttctttcaccataaccggagacaacacaaaatggaacgaaaatcaaaaccctagaatgttcttggccatgatcacatatataaccaaaaatcagcctgaatggttcagaaatattctaagtattgctccaataatgttttcaaacaagatggcgagactaggtaaggggtacatgtttgaaagcaagagtatgaaactgagaactcaaatacctgcagagatgctagccaacatagacttgaaatatttcaatgattcaactaaaaagaaaattgaaaaaatccgaccattattaatagatggaactgcatcattgagtcctggaatgatgatgggcatgttcaatatgttgagcaccgtcttgggcgtctccattctgaatcttgggcaaaagagatacaccaagactacttactggtgggatggtcttcaatcgtctgatgattttgctttgattgtgaacgcacccaactatgcaggaattcaagctggagttgacaggttttatcgaacctgtaagctgctcggaattaatatgagcaaaaagaagtcttacataaacagaacaggtacctttgaattcacgagctttttctatcgttatgggtttgttgccaatttcagcatggagcttcctagttttggggtgtctggggtcaatgaatctgcagacatgagtattggagtcactgtcatcaaaaacaatatgataaacaatgaccttggcccagcaactgctcaaatggcccttcagttatttataaaagattacaggtacacttatcgatgccaccgaggtgacacacaaatacaaacccgaagatcatttgagataaagaaactatgggaccaaacccgctcaaaagctggactgttggtctctgatggaggccccaatttgtataacattagaaatctccatattcctgaagtttgcttgaaatgggagttgatggatgaggattaccaggggcgtttatgcaacccattaaacccgtttgtcagccataaagagattgaatcagtgaacagtgcagtgataatgccggcacatggtccagccaaaaatatggagtatgacgctgttgcaacaacacactcctgggtccccaaaagaaatcgatccattttgaacacgagccaaagggggatacttgaagatgagcaaatgtatcagaggtgctgcaatttatttgaaaaattcttcccaagtagctcatacagaagaccagttggaatatccagtatggtagaggctatggtctcaagagcccgaattgatgcacggattgacttcgaatctggaaggataaagaaagaggaatttgctgagatcatgaagatctgttccaccattgaagacctcagacggcaaaaatga

>H1N1_PB1_CY092516_A/Sydney/DD3-12/2010

atggatgtcaatccgactctacttttcctaaaaattccagcgcaaaatgccataagcaccacattcccttatactggagatcctccatacagccatggaacaggaacaggatacaccatggacacagtaaatagaacacaccaatactcagaaaagggaaagtggacgacaaacacagagactggtgcaccccagctcaacccgattgatggaccactacctgaggataatgaaccaagtgggtatgcacaaacagactgtgttctagaggctatggctttccttgaagaatcccacccaggaatatttgagaattcatgccttgaaacaatggaagttgttcaacaaacaagggtagacaaactaactcaaggtcgccagacttatgattggacattaaacagaaatcaaccggcagcaactgcattggccaacaccatagaagtctttagatcgaatggcctaacagctaatgagtcaggaagactaatagatttcttaaaggatgtaatggaatcaatgaacaaagaggaaatagagataacaacccactttcaaagaaaaaggagagtaagagacaacatgaccaagaagatggtcacgcaaagaacaatagggaagaaaaaacaaagactgaataagagaggctatctaataagagcactgacattaaatacgatgaccaaagatgcagagagaggcaagttaaaaagaagggctatcgcaacacctgggatgcagattagaggtttcgtatactttgttgaagctttagctaggagcatttgcgaaaagcttgaacagtctgggctcccagtagggggcaatgaaaagaaggccaaactggcaaatgttgtgagaaagatgatgactaattcacaagacacagagatttctttcacaatcactggggacaacactaagtggaatgaaaatcaaaatcctcgaatgttcctggcaatgattacatatatcaccagaaatcaacccgagtggttcagaaacatcctgagcatggcacccataatgttctcaaacaaaatggcaagactagggaaagggtacatgttcgagagtaaaagaatgaagattcgaacacaaataccagcagaaatgctagcaagcattgacctgaagtacttcaatgaatcaacgaagaagaaaattgagaaaataaggcctcttctaatagatggcacagcatcactgagtcctgggatgatgatgggcatgttcaacatgctaagtacggtcttgggagtctcgatactgaatcttggacaaaagaaatacaccaagacaatatactggtgggatgggctccaatcatccgacgattttgctctcatagtgaatgcaccaaaccatgagggaatacaagcaggagtggacagattctacaggacctgcaagttagtgggaatcaacatgagcaaaaagaagtcctatataaataagacagggacatttgaattcacaagctttttttatcgctatggatttgtggctaattttagcatggagctacccagctttggagtgtctggagtaaatgaatcagctgacatgagtattggagtaacagtgataaagaacaacatgataaacaatgaccttggacctgcaacggcccagatggctcttcaattgttcatcaaagactacagatacacatataggtgccataggggagacacacaaattcagacgagaagatcatttgagttaaagaagctgtgggatcaaacccaatcaaaggtagggctattagtatcagatggaggaccaaacttatacaatatacggaaccttcacattcctgaagtctgcttaaaatgggagctaatggatgatgattatcggggaagactttgtaatcccctgaatccctttgtcagtcataaagagattgattctgtaaacaatgctgtggtaatgccagcccatggtccagtcaaaagcatggaatatgatgccgttgcaactacacattcctggattcccaagaggaatcgttctattctcaacacaagccaaaggggaattcttgaggatgaacagatgtaccagaagtgctgcaatctattcgagaaatttttccctagcagttcatataggagaccggttggaatttctagcatggtggaggccatggtgtctagggcccggattgatgccagggtcgacttcgagtctggacggatcaagaaagaagagttctctgagatcatgaagatctgttccaccattgaagaactcagacggcaaaaataa

>H1N1_PB1_CY092556_A/Sydney/DD3-17/2010

atggatgtcaatccgactctacttttcctaaaaattccagcgcaaaatgccataagcaccacattcccttatactggagatcctccatacagccatggaacaggaacaggatacaccatggacacagtaaacagaacacaccaatactcagaaaagggaaagtggacgacaaacacagagactggtgcaccccagctcaacccgattgatggaccactacctgaggataatgaaccaagtgggtatgcacaaacagactgtgttctagaggctatggctttccttgaagaatcccacccaggaatatttgagaattcatgccttgaaacaatggaagttgttcaacaaacaagggtagataaactaactcaaggtcgccagacttatgattggacattaaacagaaatcaaccggcagcaactgcattggccaacaccatagaagtctttagatcgaatggcctaacagctaatgagtcaggaaggctaatagatttcttaaaggatgtaatggaatcaatgaacaaagaggaaatagagataacaacccactttcaaagaaaaaggagagtaagagacaacatgaccaagaagatggtcacgcaaagaacaatagggaagaaaaaacaaagactgaataagagaggctatctaataagagcactgacattaaatacgatgaccaaagatgcagaaagaggcaagttaaaaagaagggctatcgcaacacctgggatgcagattagaggtttcgtatactttgttgaaactttagctaggagcatttgcgaaaagcttgaacagtctgggctcccagtagggggcaatgaaaagaaggccaaactggcaaatgttgtgagaaagatgatgactaattcacaagacacagagatttctttcacaatcactggggacaacactaagtggaatgaaaatcaaaatcctcgaatgttcctggcgatgattacatatatcaccagaaatcaacccgagtggttcagaaacatcctgagcatggcacccataatgttctcaaacaaaatggcaagactagggaaagggtacatgttcgagagtaaaagaatgaagattcgaacacaaataccagcagaaatgctagcaagcattgacctgaagtacttcaatgaatcaacaaagaagaaaattgagaaaataaggcctcttttaatagatggcacagcatcactgagtcctgggatgatgatgggcatgttcaacatgctaagtacggtcttgggagtctcgatactgaatcttggacaaaagaaacacaccaagacaatatactggtgggatgggctccaatcatccgacgattttgctctcatagtgaatgcaccaaaccatgagggaatacaagcaggagtggacagattctacaggacctgcaagttagtgggaatcaacatgagcaaaaagaagtcctatataaataagacagggacatttgaattcacaagctttttttatcgctatggatttgtggctaattttagcatggagctacccagctttggagtgtctggagtaaatgaatcagctgacatgagtattggagtaacagtgataaagaacaacatgataaacaatgaccttggacctgcaacggcccagatggctcttcaattgttcatcaaagactacagatacacatataggtgccataggggagacacacaaattcagacgagaagatcatttgagttaaagaagctgtgggatcaaacccaatcaaaggtagggctattagtatcagatggaggaccaaacttatacaatatacggaatcttcacattcctgaagtctgcttaaaatgggagctaatggatgatgattatcggggaagactttgtaatcccctgaatccctttgtaagtcataaagagattgattctgtaaacaatgctgtggtaatgccagcccatggtccagccaaaagcatggaatatgatgccgttgcaactacacattcctggattcccaagaggaatcgttctattctcaacacaagccaaaggggaattcttgaggatgaacagatgtaccagaagtgctgcaatctattcgagaaatttttccctagcagttcatataggagaccggttggaatttctagcatggtggaggccatggtgtctagggcccggattgatgccagggttgactttgagtctggacggatcaagaaagaagagttctctgagatcatgaagatctgctccaccattgaagaactcagacggcaaaaataa

>H1N1_PB1_CY092588_A/Sydney/DD3-25/2010

atggatgtcaatccgactctacttttcctaaaaattccagcgcaaaatgccataagcaccacattcccttatactggagatcctccatacagccatggaacaggaacaggatacaccatggacacagtaaacagaacacaccaatactcagaaaagggaaagtggacgacaaacacagagactggtgcaccccagctcaacccgattgatggaccactacctgaggataatgaaccaagtgggtatgcacaaacagactgtgttctagaggctatggctttccttgaagaatcccacccaggaatatttgagaattcatgccttgaaacaatggaagttgttcaacaaacaagggtagataaactaactcaaggtcgccagacttatgattggacattaaacagaaatcaaccggcagcaactgcattggccaacaccatagaagtctttagatcgaatggcctaacagctaatgagtcaggaaggctaatagatttcttaaaggatgtaatggaatcaatgaacaaagaggaaatagagataacaacccactttcaaagaaaaaggagagtaagagacaacatgaccaagaagatggtcacgcaaagaacaatagggaagaaaaaacaaagactgaataagagaggctatctaataagagcactgacattaaatacgatgaccaaagatgcagagagaggcaagttaaaaagaagggctatcgcaacacctgggatgcagattagaggtttcgtatactttgttgaaactttagctaggagcatttgcgaaaagcttgaacagtctgggctcccagtagggggcaatgaaaagaaggccaaactggcaaatgttgtgagaaagatgatgactaattcacaagacacagagatttctttcacaatcactggggacaacactaagtggaatgaaaatcaaaatcctcgaatgttcctggcgatgattacatatatcaccagaaatcaacccgagtggttcagaaacatcctgagcatggcacccataatgttctcaaacaaaatggcaagactagggaaagggtacatgttcgagagtaaaagaatgaagattcgaacacaaataccagcagaaatgctagcaagcattgacctgaagtacttcaatgaatcaacaaagaagaaaattgagaaaataaggcctcttttaatagatggcacagcatcactgagtcctgggatgatgatgggcatgttcaacatgctaagtacggtcttgggagtctcgatactgaatcttggacaaaagaaatacaccaagacaatatactggtgggatgggctccaatcatccgacgattttgctctcatagtgaatgcaccaaaccatgaggggatacaagcaggagtggacagattctacaggacctgcaagttagtgggaatcaacatgagcaaaaagaagtcctatataaataagacagggacatttgaattcacaagctttttttatcgctatggatttgtggctaattttagcatggagctacccagctttggagtgtctggagtaaatgaatcagctgacatgagtattggagtaacagtgataaagaacaacatgataaacaatgaccttggacctgcaacggcccagatggctcttcaattgttcatcaaagactacagatacacatataggtgccataggggagacacacaaattcagacgagaagatcatttgagttaaagaagctgtgggatcaaacccaatcaaaggtagggctattagtatcagatggaggaccaaacttatacaatatacggaatcttcacattcctgaagtctgcttaaaatgggagctaatggatgatgattatcggggaagactttgtaatcccctgaatccctttgtaagtcataaagagattgattctgtaaacaatgctgtggtaatgccagcccatggtccagccaaaagcatggagtatgatgccgttgcaactacacattcctggattcccaagaggaatcgttctattctcaacacaagccaaaggggaattcttgaggatgaacagatgtaccagaagtgctgcaatctattcgagaaatttttccctagcagttcatataggagaccggttggaatttctagcatggtggaggccatggtgtctagggcccggattgatgccagggtcgactttgagtctggacggatcaagaaagaagagttctctgagatcatgaagatctgttccaccattgaagaactcagacggcaaaaataa

>H1N1_PB1_JN187228_A/Taiwan/842/2010

atggatgtcaatccgactctacttttcctaaaaattccagcgcaaaatgccataagcaccacattcccttatactggagatcctccatacagccatggaacaggaacaggatacaccatggacacagtaaacagaacacaccaatactcagaaaagggaaagtggacgacaaacacagagactggtgcaccccagctcaacccgattgatggaccactacctgaggataatgaaccaagtgggtatgcacaaacagactgtgttctagaggctatggctttccttgaagaatcccacccaggaatatttgagaattcatgccttgaaacaatggaagttgttcaacaaacaagggtagataaactgactcaaggtcgccagacttatgattggacattaaacagaaatcaaccggcagcaactgcattggccaacaccatagaagtctttagatcgaatggcctaacagctaatgagtcaggaaggctaatagatttcttaaaggatgtaatggaatcaatgaacaaagaggaaatagagataacaacccactttcaaagaaaaaggagagtaagagacaacatgaccaagaagatggtcacgcaaagaacaatagggaagaaaaaacagagactgaataagagaggctatctaataagagcactgacattaaatacgatgaccaaagatgcagagagaggcaagttaaaaagaagggctatcgcaacacctgggatgcagattagaggtttcgtatactttgttgaaactttagctaggagcatttgcgaaaagcttgaacagtctgggctcccagtagggggcaatgaaaagaaggccaaactggcaaatgttgtgagaaagatgatgactaattcacaagacacagagatttctttcacaatcactggggacaacactaagtggaatgaaaatcaaaatcctcgaatgttcctggcgatgattacatatatcaccagaaatcaacccgagtggtttagaaacatcctgagcatggcacccataatgttctcaaacaaaatggcaagactagggaaagggtacatgttcgagagtaaaagaatgaagattcgaacacaaataccagcagaaatgctagcaagcattgacctgaagtacttcaatgaatcaacaaagaagaaaattgagaaaataaggcctcttttaatggatggcacagcatcactgagtcctgggatgatgatgggcatgttcaacatgctaagtacggtcttgggagtctcgatactgaatcttggacaaaagaaatacaccaagacaacatactggtgggatgggctccaatcatccgacgattttgctctcatagtgaatgcaccaaaccatgagggaatacaagcaggagtggacagattctacaggacctgcaagttagtgggaatcaacatgagcaaaaagaagtcctatataaataagacagggacatttgaattcacaagctttttttatcgctatggatttgtggctaattttagcatggagctacccagctttggagtgtctggagtaaatgaatcagctgacatgagtattggagtaacagtgataaagaacaacatgataaacaatgaccttggacctgcaacggcccagatggctcttcaattgttcatcaaagactacagatacacatataggtgccataggggagacacacaaattcagacgagaagatcatttgagttaaagaagctgtgggatcaaacccaatcaaaggtagggctattagtatcagatggaggaccaaacttatacaatatacggaatcttcacattcctgaagtctgcttaaaatgggagctaatggatgatgattatcggggaagactttgtaatcccctgaatccctttgtaagtcataaggagattgattctgtaaacaatgctgtggtaatgccagcccatggtccagccaaaagcatggaatatgatgccgttgcaactacacattcctggattcccaagaggaatcgttctattctcaacacaagccaaaggggaattcttgaggatgaacagatgtaccagaagtgctgcaatctattcgagaaatttttccctagcagttcatataggagaccggttggaatttctagcatggtggaggccatggtgtctagggcccggattgatgccagggtcgactttgagtctggacggatcaagaaagaagagttctctgagatcatgaagatctgttccaccattgaagaactcagacggcaaaaataa

>H1N1_PB1_CY093147_A/Sydney/DD3-20/2010

atggatgtcaatccgactctacttttcctaaaaattccagcgcaaaatgccataagcaccacattcccttatactggagatcctccatacagccatggaacaggaacaggatacaccatggacacagtaaacagaacacaccaatactcagaaaagggaaagtggacgacaaacacagagactggtgcaccccagctcaacccgattgatggaccactacctgaggataatgaaccaagtgggtatgcacaaacagactgtgttctagaggctatggctttccttgaagaatcccacccaggaatatttgagaattcatgccttgaaacaatggaagttgttcaacaaacaagggtagataaactaactcaaggtcgccagacttatgattggacattaaacagaaatcaaccggcagcaactgcattggccaacaccatagaagtctttagatcgaatggcctaacagctaatgagtcaggaaggctaatagatttcttaaaggatgtaatggaatcaatgaacaaagaggaaatagagataacaacccactttcaaagaaaaaggagagtaagagacaacatgaccaagaagatggtcacgcaaagaacaatagggaagaaaaaacaaagactgaataagagaggctatctaataagagcactgacattaaatacgatgaccaaagatgcagagagaggcaagttaaaaagaagggctatcgcaacacctgggatgcagattagaggtttcgtatactttgttgaagctttagctaggagcatttgcgaaaagcttgaacagtctgggctcccagtaggaggcaatgaaaagaaggccaaactggcaaatgttgtgagaaagatgatgactaattcacaagacacagagatttctttcacaatcactggggacaacactaagtggaatgaaaatcaaaatcctcgaatgttcctggcaatgattacatatatcaccagaaatcaacccgagtggttcagaaacatcctgagcatggcacccataatgttctcaaacaaaatggcaagactagggaaagggtacatgttcgagagtaaaagaatgaagattcgaacacaaataccagcagaaatgctagcaagcattgacctgaagtacttcaatgaatcaacgaagaagaaaattgagaaaataaggcctcttctaatagatggcacagcatcactgagtcctgggatgatgatgggcatgttcaacatgctaagtacggtcttgggagtctcgatactgaatcttggacaaaagaaatacaccaagacaatatactggtgggatgggctccaatcatccgacgattttgctctcatagtgaatgcaccaaaccatgagggaatacaagcaggagtggacagattctacaggacctgcaagttagtgggaatcaacatgagcaaaaagaagtcctatataaataagacagggacatttgaattcacaagctttttttatcgctatggatttgtggctaattttagcatggagctacccagctttggagtgtctggagtaaatgaatcagctgacatgagtattggagtaacagtgataaagaacaacatgataaacaatgaccttggacctgcaacggcccagatggctcttcaattgttcatcaaagactacagatacacatataggtgccataggggagacacacaaattcagacgagaagatcatttgagttaaagaagctgtgggatcaaacccaatcaaaggtagggctattagtatcagatggaggaccaaacttatacaatatacggaatcttcacattcctgaagtctgcttaaaatgggagctaatggatgatgattatcggggaagactttgtaatcccctgaatccctttgtcagtcataaagagattgattctgtaaacaatgctgtggtaatgccagcccatggtccagtcaaaagcatggaatatgatgccgttgcaactacacattcctggattcccaagaggaatcgttctattctcaacacaagccaaaggggaattcttgaggatgaacagatgtaccagaagtgctgcaatctattcgagaaatttttccctagcagttcatataggagaccggttggaatttctagcatggtggaggccatggtgtctagggcccggattgatgccagggtcgacttcgagtctggacggatcaagaaagaagagttctctgagatcatgaagatctgctccaccattgaagaactcagacggcaaaaataa

>H1N1_PB1_CY093187_A/Chile/94/2010

atggatgtcaatccgactctacttttcctaaaaattccagcgcaaaatgccataagcaccacattcccttatactggagatcctccatacagccatggaacaggaacaggatacaccatggacacagtaaacagaacacaccaatactcagaaaagggaaagtggacgacaaacacagagactggtgcaccccagctcaacccgattgatggaccactaccagaggataatgaaccaagtgggtatgcacaaacagactgtgttctagaggctatggctttccttgaagaatcccacccaggaatatttgagaattcatgccttgaaacaatggaagttgttcaacaaacaagggtagataaactaactcaaggtcgccagacttatgattggacattaaacagaaatcaaccggcagcaactgcattggccaacaccatagaagtctttagatcgaatggcctaatagctaatgagtcaggaaggctaatagatttcttaaaggatgtaatggaatcaatgaacaaagaggaaatagagataacaacccactttcaaagaaaaaggagagtaagagacaacatgaccaagaagatggtcacgcaaagaacaatagggaagaaaaaacaaagactgaataagagaggctatctaataagagcactgacattaaatacgatgaccaaagatgcagagagaggcaagttaaaaagaagggctatcgcaacacctgggatgcagattagaggtttcgtatactttgttgaaactttagctaggagcatttgcgaaaagcttgaacagtctgggctcccagtagggggcaatgaaaagaaggccaaactggcaaatgttgtgagaaagatgatgactaattcacaagacacagagatttctttcacaatcactggggacaacactaagtggaatgaaaatcaaaatcctcgaatgttcctggcgatgattacatatatcaccagaaatcaacccgagtggttcagaaacatcctgagcatggcacccataatgttctcaaacaaaatggcaagactagggaaagggtacatgttcgagagtaaaagaatgaagattcgaacacaaataccagcagaaatgctagcaagcattgacctgaagtacttcaatgaatcaacaaagaagaaaattgagaaaataaggcctcttctaatagatggcacagcatcactgagtcctgggatgatgatgggcatgttcaacatgctaagtacggtcttgggagtctcgatactgaatcttggacaaaagaaatacaccaagacaatatactggtgggatgggctccaatcatccgacgattttgctctcatagtgaatgcaccaaaccatgagggaatacaagcaggagtggacagattctacaggacctgcaagttagtgggaatcaacatgagcaaaaagaagtcctatataaataagacagggacatttgaattcacaagctttttttatcgctatggatttgtggctaattttagcatggagctacccagctttggagtgtctggagtaaatgaatcagctgacatgagtattggagtaacagtgataaagaacaacatgataaacaatgaccttggacctgcaacggcccagatggctcttcaattgttcatcaaagactacagatacacatataggtgccataggggagacacacaaattcagacgagaagatcatttgagttaaagaagctgtgggatcaaacccaatcaaagggagggctattagtatcagatggaggaccaaacttatacaatatacggaatcttcacattcctgaagtctgcttaaaatgggagctaatggatgatgattatcggggaagactttgtaatcccctgaatccctttgtcagtcataaagagattgattctgtaaacaatgctgtggtaatgccagcccatggtccagtcaaaagcatggaatatgatgccgttgcaactacacattcctggattcccaagaggaatcgttctattctcaatacaagccaaaggggaattcttgaggatgaacagatgtaccagaagtgctgcaatctattcgagaaatttttccctagcagttcatataggagaccggttggaatttctagcatggtggaggccatggtgtctagggcccggatcgatgccagggtcgacttcgagtctggacggatcaagaaagaagagttctctgagatcatgaagatctgctccaccattgaagaactcagacggcaaaaataa

>H1N1_PB1_CY093195_A/Chile/106/2010

atggatgtcaatccgactctacttttcctaaaaattccagcgcaaaatgccataagcaccacattcccttatactggagatcctccatacagccatggaacaggaacaggatacaccatggacacagtaaacagaacacaccaatactcagaaaagggaaagtggacgacaaacacagagactggtgcaccccagctcaacccgattgatggaccactacctgaggataatgaaccaagtgggtatgcacaaacagactgtgttctagaggctatggctttccttgaagaatcccacccaggaatatttgagaattcatgccttgaaacaatggaagttgttcaacaaacaagggtagataaactaactcaaggtcgccagacttatgattggacattaaacagaaatcaaccggcagcaactgcattggccaacaccatagaagtctttagatcgaatggcctaacagctaatgagtcaggaaggctaatagatttcttaaaggatgtaatggaatcaatgaacaaagaggaaatagagataacaacccactttcaaagaaaaaggagagtaagagacaacatgaccaagaagatggtcacgcaaagaacaatagggaagaaaaaacaaaggctgaataagagaggctatctaataagagcactgacattaaatacgatgaccaaagatgcagagagaggcaagttaaaaagaagggctatcgcaacacctgggatgcagattagaggtttcgtatactttgttgaagctttagctaggagcatttgcgaaaagcttgaacagtctgggctcccagtagggggcaatgaaaagaaggccaaactggcaaatgttgtgagaaagatgatgactaattcacaagacacagagatttctttcacaatcactggggacaacactaagtggaatgaaaatcaaaatcctcgaatgttcctggcaatgattacatatatcaccagaaatcaacccgagtggttcagaaacatcctgagcatggcacccataatgttctcaaacaaaatggcaagactagggaaagggtacatgttcgagagtaaaagaatgaagattcgaacacaaataccagcagaaatgctagcaagcattgacctgaagtacttcaatgaatcaacgaagaagaaaattgagaaaataaggcctcttctaatagatggcacagcatcactgagtcctgggatgatgatgggcatgttcaacatgctaagtacggtcttgggagtctcgatactgaatcttggacaaaagaaatacaccaagacaatatactggtgggatggactccaatcatccgacgattttgctctcatagtgaatgcaccaaaccatgagggaatacaagcaggagtggacagattctacaggacctgcaagttagtgggaatcaacatgagcaaaaagaagtcctatataaataagacagggacatttgaattcacaagctttttttatcgctatggatttgtggctaattttagcatggagctacccagctttggagtgtctggagtaaatgaatcagctgacatgagtattggagtaacagtgataaagaacaacatgataaacaatgaccttggacctgcaacggcccagatggctcttcaattgttcatcaaagactacagatacacatataggtgccataggggagacacacaaattcagacgagaagatcatttgagttaaagaagctgtgggatcaaacccaatcaaaggtagggctattagtatcagatggaggaccaaacttatacaatatacggaatcttcacattcctgaagtctgcttaaaatgggagctaatggatgatgattatcggggaagactttgtaatcccctgaatccctttgtcagtcataaagagattgattctgtaaacaatgctgtggtaatgccagcccatggtccagtcaaaagcatggaatatgatgccgttgcaactacacattcctggattcccaagaggaatcgttctattctcaacacaagccaaaggggaattcttgaggatgaacagatgtaccagaagtgctgcaatctattcgagaaatttttccctagcagttcatataggagaccggttggaatttctagcatggtggaggccatggtgtctagggcccggattgatgccagggtcgacttcgagtctggacggatcaagaaagaagagttctctgagatcatgaagatctgttccaccattgaagaactcagacggcaaaaataa

>H1N1_PB1_CY096240_A/North Fitzroy/INS469/2010

atggatgtcaatccgactctacttttcctaaaaattccagcgcaaaatgccataagcaccacattcccttatactggagatcctccatacagccatggaacaggaacaggatacaccatggacacagtaaacagaacacaccaatactcagaaaagggaaagtggacgacaaacacagagactggtgcaccccagctcaacccgattgatggaccactacctgaggataatgaaccaagtgggtatgcacaaacagactgtgttctagaggctatggctttccttgaagaatcccacccaggaatatttgagaattcatgccttgaaacaatggaagttgttcaacaaacaagggtagataaactaactcaaggtcgccagacttatgattggacattaaacagaaatcaaccggcagcaactgcattggccaacaccatagaagtctttagatcgaatggcctaacagctaatgagtcaggaaggctaatagatttcttaaaggatgtaatggaatcaatgaacaaagaggaaatagagataacaacccactttcaaagaaaaaggagagtaagagacaacatgaccaagaagatggtcacgcaaagaacaatagggaagaaaaaacaaagactgaataagagaggctatctaataagagcactaacattaaatacgatgaccaaagatgcagagagaggcaagttaaaaagaagggctatcgcaacacctgggatgcagattagaggtttcgtatactttgttgaagctttagctaggagcatttgcgaaaagcttgaacagtctgggctcccagtagggggcaatgaaaagaaggccaaactggcaaatgttgtgagaaagatgatgactaattcacaagacacagagatttctttcacaatcactggggacaacactaagtggaatgaaaatcaaaatcctcgaatgttcctggcaatgattacatatatcaccagaaatcaacccgagtggttcagaaacatcctgagcatggcacccataatgttctcaaacaaaatggcaagactagggaaagggtacatgttcgagagtaaaagaatgaagattcgaacacaaataccagcagaaatgctagcaagcattgacctgaagtacttcaatgaatcaacgaagaaaaaaattgagaaaataaggcctcttctaatagatggcacagcatcactgagtcctgggatgatgatgggcatgttcaacatgctaagtacggtcttgggagtctcgatactgaatcttggacaaaagaaatacaccaagacaatatactggtgggatgggctccaatcatccgacgattttgctctcatagtgaatgcaccaaaccatgagggaatacaagcaggagtggacagattctacaggacctgcaagttagtgggaatcaacatgagcaaaaagaagtcctatataaataagacagggacatttgaattcacaagctttttttatcgctatggatttgtggctaattttagcatggagctacccagctttggagtgtctggagtaaatgaatcagctgacatgagtattggagtaacagtgataaagaacaacatgataaacaatgaccttggacctgcaacggcccagatggctcttcaattgttcatcaaagactacagatatacatataggtgccataggggagacacacaaattcagacgagaagatcatttgagttaaagaagctgtgggatcaaacccaatcaaaggtagggctattagtatcagatggaggaccaaacttatacaatatacggaatcttcacattcctgaagtctgcttaaaatgggagctaatggatgatgattatcggggaagactttgtaatcccctgaatccctttgtcagtcataaagagattgattctgtaaacaatgctgtggtaatgccagcccatggtccagtcaaaagcatggaatatgatgccgttgcaactacacattcctggattcccaagaggaatcgttctattctcaacacaagccaaaggggaattcttgaggatgaacagatgtaccagaagtgctgcaatctattcgagaaatttttccctagcagttcatataggagaccggttggaatttctagcatggtggaggccatggtgtctagggcccggattgatgccagggtcgacttcgagtctggacggatcaagaaagaagagttctctgagatcatgaagatctgttccaccattgaagaactcagacggcaaaaataa

>H1N1_PB1_CY096296_A/Bangkok/INS477/2010

atggatgtcaatccgactctacttttcctaaaaattccagcgcaaaatgccataagcaccacattcccttatactggagatcctccatacagccatggaacaggaacaggatacaccatggacacagtaaacagaacacaccaatactcagaaaagggaaagtggacgacaaacacagagactggtgcaccccagctcaacccgattgatggaccactacctgaggataatgaaccaagtgggtatgcacaaacagactgtgttctagaggctatggctttccttgaagaatcccacccaggaatatttgagaattcatgccttgaaacaatggaagttgttcaacaaacaagggtagataaactgactcaaggtcgccagacttatgattggacattaaacagaaatcaaccggcagcaactgcattggccaacaccatagaagtctttagatcgaatggcctaacagctaatgagtcaggaaggctaatagatttcttaaaggatgtaatggaatcaatgaacaaagaggaaatagagataacaacccactttcaaagaaaaaggagagtaagagacaacatgaccaagaagatggtcacgcaaagaacaatagggaagaaaaaacagagactgaataagagaggctatctaataagagcactgacattaaatacgatgaccaaagatgcagagagaggcaagttaaaaagaagggctatcgcaacacctgggatgcagattagaggtttcgtatactttgttgaaactttagctaggagcatttgcgaaaagcttgaacagtctgggctcccagtagggggcaatgaaaagaaggccaaactggcaaatgttgtgagaaagatgatgactaattcacaagacacagagatttctttcacaatcactggggacaacactaagtggaatgaaaatcaaaatcctcgaatgttcctggcgatgattacatatatcaccagaaatcaacccgagtggtttagaaacatcctgagcatggcacccataatgttctcaaacaaaatggcaagactagggaaagggtacatgttcgagagtaaaagaatgaagattcgaacacaaataccagcagaaatgctagcaagcattgacctgaagtacttcaatgaatcaacaaagaagaaaattgagaaaataaggcctcttttaatggatggcacagcatcactgagtcctgggatgatgatgggcatgttcaacatgctaagtacggtcttgggagtctcgatactgaatcttggacaaaagaaatacaccaagacaacatactggtgggatgggctccaatcatccgacgattttgctctcatagtgaatgcaccaaaccatgagggaatacaagcaggagtggacagattctacaggacctgcaagttagtgggaatcaacatgagcaaaaagaagtcctatataaataagacagggacatttgaattcacaagctttttttatcgctatggatttgtggctaattttagcatggagctacccagctttggagtgtctggagtaaatgaatcagctgacatgagtattggagtaacagtgataaagaacaacatgataaacaatgaccttggacctgcaacggcccagatggctcttcaattgttcatcaaagactacagatacacatataggtgccataggggagacacacaaattcagacgagaagatcatttgagttaaagaagctgtgggatcaaacccaatcaaaggtagggctattagtatcagatggaggaccaaacttatacaatatacggaatcttcacattcctgaagtctgcttaaaatgggagctaatggatgatgattatcggggaagactttgtaatcccctgaatccctttgtaagtcataaggagattgattctgtaaacaatgctgtggtaatgccagcccatggtccagccaaaagcatggaatatgatgccgttgcaactacacattcctggattcccaagaggaatcgttctattctcaacacaagccaaaggggaattcttgaggatgaacagatgtaccagaagtgctgcaatctattcgagaaatttttccctagcagttcatataggagaccggttggaatttctagcatggtggaggccatggtgtctagggcccggattgatgccagggtcgactttgagtctggacggatcaagaaagaagagttctctgagatcatgaagatctgttccaccattgaagaactcagacggcaaaaataa

>H1N1_PB1_CY096488_A/Bangkok/INS510/2010

atggatgtcaatccgactctacttttcctaaaaattccagcgcaaaatgccataagcaccacattcccttatactggagatcctccatacagccatggaacaggaacaggatacaccatggacacagtaaacagaacacaccaatactcagaaaagggaaagtggacgacaaacacagagactggtgcaccccagctcaacccgattgatggaccactacctgaggataatgaaccaagtgggtatgcacaaacagactgtgttctagaggctatggctttccttgaagaatcccacccaggaatatttgagaattcatgccttgaaacaatggaagttgttcaacaaacaagggtagataaactgactcaaggtcgccagacttatgattggacattaaacagaaatcaaccggcagcaactgcattggccaacaccatagaagtctttagatcaaatggcctaacagctaatgagtcaggaaggctaatagatttcttaaaggatgtaatggaatcaatgaacaaagaggaaatagagataacaacccactttcaaagaaaaaggagagtaagagacaacatgaccaagaagatggtcacgcaaagaacaatagggaagaaaaaacagagactgaataagagaggctatctaataagagcactgacattaaatacgatgaccaaagatgcagagagaggcaagttaaaaagaagggctatcgcaacacctgggatgcagattagaggtttcgtatactttgttgaaactttagctaggagcatttgcgaaaagcttgaacagtctggactcccagtagggggcaatgaaaagaaggccaaactggcaaatgttgtgagaaagatgatgactaattcacaagacacagagatttctttcacaatcactggggacaacactaagtggaatgaaaatcaaaatcctcgaatgttcctggcgatgattacatatatcaccagaaatcaacccgagtggtttagaaacatcctgagcatggcacccataatgttctcaaacaaaatggcaagactagggaaagggtacatgttcgagagtaaaagaatgaagattcgaacacaaataccagcagaaatgctagcaagcattgacctgaagtacttcaatgaatcaacaaagaaaaaaattgagaaaataaggcctcttttaatggatggcacagcatcactgagtcctgggatgatgatgggcatgttcaacatgctaagtacggtcttgggagtctcgatactgaatcttggacaaaagaaatacaccaagacaatatactggtgggatgggctccaatcatccgacgattttgctctcatagtgaatgcaccaaaccatgagggaatacaagcaggagtggacagattctacaggacctgcaagttagtgggaatcaacatgagcaaaaagaagtcctatataaataagacagggacatttgaattcacaagctttttttatcgctatggatttgtggctaattttagcatggagctacccagctttggagtgtctggagtaaatgaatcagctgacatgagtattggagtaacagtgataaagaacaacatgataaacaatgaccttggacctgcaacggcccagatggctcttcaattgttcatcaaagactacagatacacatataggtgccataggggagacacacaaattcagacgagaagatcatttgagttaaagaagctgtgggatcaaacccaatcaaaggtagggctattagtatcagatggaggaccaaacttatacaatatacggaatcttcacattcctgaagtctgcttaaaatgggagctaatggatgatgattatcggggaagactttgtaatcccctgaatccctttgtaagtcataaggagattgattctgtaaacaatgctgtggtaatgccagcccatggtccagccaaaagcatggaatatgatgccgttgcaactacacattcctggattcccaagaggaatcgttctattctcaacacaagccaaaggggaattcttgaggatgaacagatgtaccagaagtgctgcaatctattcgagaaatttttccctagcagttcatataggagaccggttggaatttctagcatggtggaggccatggtgtctagggcccggattgatgccagggtcgactttgagtctggacggatcaagaaagaagagttctctgagatcatgaagatctgttccaccattgaagaactcagacggcaaaaataa

>H1N1_PB1_CY096535_A/Bangkok/INS518/2010

atggatgtcaatccgactctacttttcctaaaaattccagcgcaaaatgccataagcaccacattcccttatactggagatcctccatacagccatggaacaggaacaggatacaccatggacacagtaaacagaacacaccaatactcagaaaagggaaagtggacgacaaacacagagactggtgcaccccagctcaacccgattgatggaccactacctgaggataatgaaccaagtgggtatgcacaaacagactgtgttctagaggctatggctttccttgaagaatcccacccaggaatatttgagaattcatgccttgaaacaatggaagttgttcaacaaacaagggtagataaactaactcaaggtcgccagacttatgattggacattaaacagaaatcaaccggcagcaactgcattggccaacaccatagaagtctttagatcgaatggcctaacagctaatgagtcaggaaggctaatagatttcttaaaggatgtaatggaatcaatgaacaaagaggaaatagagataacaacccattttcaaagaaaaaggagagtaagagacaacatgaccaagaagatggtcacgcaaagaacaatagggaagaaaaaacaaagactgaataagagaggctatctaataagagcactgacattaaatacgatgaccaaagatgcagagagaggcaagttaaaaagaagggctatcgcaacacctgggatgcagattagaggtttcgtatactttgttgaaactttagctaggagcatttgcgaaaagcttgaacagtctgggctcccagtagggggcaatgaaaagaaggccaaactggcaaatgttgtgagaaagatgatgactaattcacaagacacagagatttctttcacaatcactggggacaacactaagtggaatgaaaatcaaaatcctcgaatgttcctggcgatgattacatatatcaccagaaatcaacccgagtggttcagaaacatcctgagcatggcacctataatgttctcaaacaaaatggcaagactagggaaagggtacatgttcgagagtaaaaggatgaagattcgaacacaaataccagcagaaatgctagcaagcattgacctgaagtacttcaatgaatcaacaaagaagaaaattgagaaaataaggcctcttttaatagatggcacagcatcactgagtccagggatgatgatgggcatgttcaacatgctaagtacggtcttgggagtctcgatactgaatcttggacaaaagaaatacaccaagacaatatactggtgggatgggctccaatcatccgacgattttgctctcatagtgaatgcaccaaaccatgagggaatacaagcaggagtggacagattctacaggacctgcaagttagtgggaatcaacatgagcaaaaagaagtcttatataaataagacagggacatttgaattcacaagctttttttatcgctatggatttgtggctaattttagcatggagctacccagctttggagtgtctggagtaaatgaatcagctgacatgagtattggagtaacagtgataaagaacaacatgataaacaatgaccttggacctgcaacggcccagatggctcttcaattgttcatcaaagactacagatacacatataggtgccataggggagacacacaaattcagacgagaagatcatttgagttaaagaagctttgggatcaaacccaatcaaaggtagggctattagtatcagatggaggaccaaacttatacaatatacggaatcttcacattcctgaagtctgcttaaaatgggagctaatggatgatgattatcggggaagactttgtaatcccctgaatccctttgtaagtcataaagagattgattctgtaaacaatgctgtggtaatgccagcccatggtccagccaaaagcatggaatatgatgccgttgcaactacacattcctggattcccaagaggaatcgttctattctcaacacaagccaaaggggaattcttgaggatgaacagatgtaccagaagtgctgcaatctattcgagaaatttttccctagcagttcatataggagaccggttggaatttctagcatggtggaggccatggtgtctagggcccggattgatgccagggtcgactttgagtctggacggatcaagaaagaagagttctctgagatcatgaagatctgttccaccattgaagaactcagacggcaaaaataa

>H1N1_PB1_CY096725_A/Singapore/276/2009

atggatgtcaatccgactctacttttcctaaaaattccagcgcaaaatgccataagcaccacattcccttatactggagatcctccatacagccatggaacaggaacaggatacaccatggacacagtaaacagaacacaccaatactcagaaaagggaaagtggacgacaaacacagagactggtgcaccccagctcaacccgattgatggaccactacctgaggataatgaaccaagtgggtatgcacaaacagactgtgttctagaggctatggctttccttgaagaatcccacccaggaatatttgagaattcatgccttgaaacaatggaagttgttcaacaaacaagggtagataaactaactcaaggtcgccagacttatgattggacattaaacagaaatcaaccggcagcaactgcattggccaacaccatagaagtctttagatcgaatggcctaacagctaatgagtcaggaaggctaatagatttcttaaaggatgtaatggaatcaatgaacaaagaggaaatagagataacaacccactttcaaagaaaaaggagagtaagagacaacatgaccaagaagatggtcacgcaaagaacaatagggaagaaaaaacaaagactgaataagagaggctatctaataagagcactgacattaaatacgatgaccaaagatgcagagagaggcaagttaaaaagaagggctatcgcaacacctgggatgcagattagaggtttcgtatactttgttgaaactttagctaggagcatttgcgaaaagcttgaacagtctgggctcccagtagggggcaatgaaaagaaggccaaactggcaaatgttgtgagaaagatgatgactaattcacaagacacagagatttctttcacaatcactggggacaacactaagtggaatgaaaatcaaaatcctcgaatgttcctggcgatgattacatatatcaccagaaatcaacccgagtggttcagaaacatcctgagcatggcacccataatgttctcaaacaaaatggcaagactagggaaagggtacatgttcgagagtaaaagaatgaagattcgaacacaaataccagcagaaatgctagcaagcattgacctgaagtacttcaatgaatcaacaaagaagaaaattgagaaaataaggcctcttctaatagatggcacagcatcactgagtcctgggatgatgatgggcatgttcaacatgctaagtacggtcttgggagtctcgatactgaatcttggacaaaagaaatacaccaagacaatatactggtgggatgggctccaatcatccgacgattttgctctcatagtgaatgcaccaaaccatgagggaatacaagcaggagtggacagattctacaggacctgcaagttagtgggaatcaacatgagcaaaaagaagtcctatataaataagacagggacatttgaattcacaagctttttttatcgctatggatttgtggctaattttagcatggagctacccagctttggagtgtctggagtaaatgaatcagctgacatgagtattggagtaacagtgataaagaacaacatgataaacaatgaccttggacctgcaacggcccagatggctcttcaattgttcatcaaagactacagatacacatataggtgccataggggagacacacaaattcagacgagaagatcatttgagttaaagaagctgtgggatcaaacccaatcaaaggtagggctattagtatcagatggaggaccaaacttatacaatatacggaatcttcacattcctgaagtctgcttaaaatgggagctaatggatgatgattatcggggaagactttgtaatcccctgaatccctttgtcagtcataaagagattgattctgtaaacaatgctgtggtaatgccagcccatggtccagccaaaagcatggaatatgatgccgttgcaactacacattcctggattcccaagaggaatcgttctattctcaacacaagccaaaggggaattcttgaggatgaacagatgtaccagaagtgctgcaatctattcgagaaatttttccctagcagttcatataggagaccggttggaatttctagcatggtggaggccatggtgtctagggcccggattgatgccagggtcgacttcgagtctggacggatcaagaaagaagagttctctgagatcatgaagatctgttccaccattgaagaactcagacggcaaaaataa

>H1N1_PB1_CY088658_A/Ngp/NIV14725/2009

atggatgtcaatccgactctacttttcctaaaaattccagcgcaaaatgccataagcaccacattcccttatactggagatcctccatacagccatggaacaggaacaggatacaccatggacacagtaaacagaacacaccaatactcagaaaagggaaagtggacgacaaacacagagactggtgcaccccagctcaacccgattgatggaccactacctgaggataatgaaccaagtgggtatgcacaaacagactgtgttctagaggctatggctttccttgaagaatcccacccaggaatatttgagaattcatgccttgaaacaatggaagttgttcaacaaacaagggtagataaactaactcaaggtcgccagacttatgattggacattaaacagaaatcaaccggcagcaactgcattggccaacaccatagaagtctttagatcgaatggcctaacagctaatgagtcaggaaggctaatagatttcttaaaggatgtaatggaatcaatgaacaaagaggaaatagagataacaacccactttcaaagaaaaaggagagtaagagacaacatgaccaagaagatggtcacgcaaagaacaatagggaagaaaaaacaaagactgaataagagaggctatctaataagagcactgacattaaatacgatgaccaaagatgcagagagaggcaagttaaaaagaagggctatcgcaacacctgggatgcagattagaggtttcgtatactttgttgaaactttagctaggagcatttgcgaaaagcttgaacagtctgggctcccagtagggggcaatgaaaagaaggccaaactggcaaatgttgtgagaaagatgatgactaattcacaagacacagagatttctttcacaatcactggggacaacactaagtggaatgaaaatcaaaatcctcgaatgttcctggcgatgaatacatatatcaccagaaatcaacccgagtggttcagaaacatcctgagcatggcacccataatgttctcaaacaaaatggcaagactagggaaagggtacatgttcgagagtaaaagaatgaagattcgaacacaaataccagcagaaatgctagcaagcattgacctgaagtacttcaatgaatcaacaaagaagaaaattgagaaaataaggcctcttctaatagatggcacagcatcactgagtcctgggatgatgatgggcatgttcaacatgctaagtacggtcttgggagtctcgatactgaatcttggacaaaagaaatacaccaagacaatatactggtgggatgggctcccatcatccgacgattttgctctcatagtgaatgcaccaaaccatgagggaatacaagcaggagtggacagattctacaggacctgcaaattagtgggaatcaacatgagcaaaaggaagtcctatataaataagacagggacatttgaattcacaagctttttttatcgctatggatttgtggccaatttcagcatggagctacccagctttggagtgtctggagtaaatgaatcagctgacatgagtattggagtaacagtgataaagaacaacatgataaacaatgaccttggacctgcaacggcccagatggctcttcaattgttcatcaaagactacagatacacatataggtgccataggggagacacacaaattcagacgagaagatcatttgagttaaagaagctgtgggatcaaacccaatcaaaggtagggctattagtatcagatggaggaccaaacttatacaatatacggaatcttcacattcctgaagtctgcttaaaatgggagctaatggatgatgattatcggggaagactttgtaatcccctgaatccctttgtcagtcataaagagattgattctgtaaacaatgctgttgtaatgccagcccatggtccagccaaaagcatggaatatgatgccgttgcaactacacattcctggattcccaagaggaatcgttctattctcaacacaagccaaaggggaattcttgaggatgaacagatgtaccagaagtgctgcaatctattcgagaaatttttccctagcagttcatataggagaccggttggaatttctagcatggtggaggccatggtgtctagggcccggattgatgccagggtcgacttcgagtctggacggatcaagaaagaagagttctctgagatcatgaagatctgttccaccattgaagaactcagacggcaaaaataa

>H1N1_PB1_CY097835_A/District of Columbia/WRAIR0309/2010

atggatgtcaatccgactctacttttcctaaaaattccagcgcaaaatgccataagcaccacattcccttatactggagatcctccatacagccatggaacgggaacaggatacaccatggacacagtaaacagaacacaccaatactcagaaaagggaaagtggacgacaaacacagagactggtgcaccccagctcaacccgattgatggaccactacctgaggataatgaaccaagtgggtatgcacaaacagactgtgttctagaggctatggctttccttgaagaatcccacccaggaatatttgagaattcatgccttgaaacaatggaagttgttcaacaaacaagggtagataaactaactcaaggtcgccagacttatgattggacattaaacagaaatcaaccggcagcaactgcattggccaacaccatagaagtctttagatcgaatggcctaacagctaatgagtcaggaaggctaatagatttcttaaaggatgtaatggaatcaatgaacaaagaggaaatagagataacaacccactttcaaagaaaaaggagagtaagagacaacatgaccaagaagatggtcacgcaaagaacaatagggaagaaaaaacaaagactgaataagagaggctatctaataagagcactgacattaaatacgatgaccaaagatgcagagagaggcaagttaaaaagaagggctatcgcaacacctgggatgcagattagaggtttcgtatactttgttgaaactttagctaggagcatttgcgaaaagcttgaacagtctgggctcccagtagggggcaatgaaaagaaggccaaactggcaaatgttgtgagaaagatgatgactaattcacaagacacagagatttctttcacaatcactggagacaacactaagtggaatgaaaatcaaaatcctcgaatgttcctggcgatgattacatatatcaccagaaatcaacccgagtggttcagaaacatcctgagcatggcacccataatgttctcaaacaaaatggcaagactagggaaagggtacatgttcgagagtaaaagaatgaagattcgaacacaaataccagcagaaatgctagcaagcattgacctgaagtacttcaatgaatcaacaaagaagaaaattgagaaaataaggcctcttttaatagatggcacagcatcactgagtcctgggatgatgatgggcatgttcaacatgctaagtacggtcttgggagtctcgatactgaatcttggacaaaagaaatacaccaagacaatatactggtgggatgggctccaatcatccgacgattttgctctcatagtgaatgcaccaaaccatgagggaatacaagcaggagtggacagattctacaggacctgcaagttagtgggaatcaacatgagcaaaaagaagtcctatataaataagacagggacatttgaattcacaagctttttttatcgctatggatttgtggctaattttagcatggagctacccagctttggagtgtctggagtaaatgaatcagctgacatgagtattggagtaacagtgataaagaacaacatgataaacaatgaccttgggcctgcaacggcccagatggctcttcaattgttcatcaaagactacagatacacatataggtgccataggggagacacacaaattcagacgagaagatcatttgagttaaagaaactgtgggatcaaacccaatcaaaggtagggctattagtatcagatggaggaccaaatctatacaatatacggaatcttcacattcctgaagtctgcttaaaatgggagctaatggatgatgattatcggggaagactttgtaatcccctgaatccctttgtaagtcataaagagattgattctgtaaacaatgctgtggtaatgccagcccatggtccagccaaaagcatggagtatgatgccgttgcaactacacattcctggattcccaagaggaatcgttctattctcaacacaagccaaaggggaattcttgaggatgaacagatgtaccagaagtgctgcaatctattcgagaaatttttccctagcagttcatataggagaccggttggaatttctagcatggtggaggccatggtgtctagggcccggattgatgccagggtcgactttgagtctggacggatcaagaaagaagagttctctgagatcatgaagatctgttccaccattgaagaactcagacggcaaaaataa

>H1N1_PB1_CY083110_A/Quito/WRAIR0617N/2009

atggatgtcaatccgactctacttttcctaaaaattccagcgcaaaatgccataagcaccacattcccttatactggagatcctccatacagccatggaacaggaacaggatacaccatggacacagtaaacagaacacaccaatactcagaaaagggaaagtggacgacaaacacagagactggtgcaccccagctcaacccgattgatggaccactacctgaggataatgaaccaagtgggtatgcacaaacagactgtgttctagaggctatggctttccttgaagaatcccacccaggaatatttgagaattcatgccttgaaacaatggaagttgttcaacaaacaagggtagataaactaactcaaggtcgccagacttatgattggacattaaacagaaatcaaccggcagcaactgcattggccaacaccatagaagtctttagatcgaatggcctaacagctaatgagtcaggaaggctaatagatttcttaaaggatgtaatggaatcaatgaacaaagaggaaatagagataacaacccactttcaaagaaaaaggagagtaagagacaacatgaccaagaagatggtcacgcaaagaacaatagggagtaaaaaacaaagactgaataagagaggctatctaataagagcactgacattaaatacgatgaccaaagatgcagagagaggcaagttaaaaagaagggctatcgcaacacctgggatgcagattagaggtttcgtatactttgctgaaactttagctaggagcatttgcgaaaagcttgaacagtctgggctcccagtagggggcaatgaaaagaaggccaaattggcaaatgttgtgagaaagatgatgactaattcacaagacacagagatttctttcacaatcactggggacaacactaagtggaatgaaaatcaaaatcctcgaatgttcctggcgatgattacatatatcaccagaaatcaacccgagtggttcagaaacatcctgagcatggcacccataatgttctcaaacaaaatggcaagactagggaaagggtacatgttcgagagtaaaagaatgaagattcgaacacaaataccagcagaaatgctagcaagcattgacctgaagtacttcaatgaatcaacaaagaagaaaattgagaaaataaggcctcttctaatagatggcacagcatcactgagtcctgggatgatgatgggcatgttcaacatgctaagtacggtcttgggagtctcgatactgaatcttggacaaaagaaatacaccaagacaatatactggtgggatgggctccaatcatccgacgattttgctctcatagtgaatgcaccaaaccatgagggaatacaagcaggagtggacagattctacaggacctgcaagttagtgggaatcaacatgagcaagaagaagtcctatataaataagacagggacatttgaattcacaagctttttttatcgctatggatttgtggctaattttagcatggagctacccagctttggagtgtctggagtaaatgaatcagctgacatgagtattggagtaacagtgataaagaacaacatgataaacaatgaccttggacctgcaacggcccagatggctcttcaattgttcatcaaagactacagatacacatataggtgccataggggagacacacaaattcagacgagaagatcatttgagttaaagaagctgtgggatcaaacccaatcaaaggtagggctattagtatcagatggaggaccaaacttatacaatatacggaatcttcacattcctgaagtctgcttaaaatgggagctaatggatgatgattatcggggaagactttgtaatcccctgaatccctttgtcagtcataaagagattgattctgtaaacaatgctgtggtaatgccagcccatggtccagccaaaagcatggaatatgatgccgttgcaactacacattcctggattcccaagaggaatcgttctattctcaacacaagccaaaggggaattcttgaggatgaacagatgtaccagaagtgctgcaatctattcgagaaatttttccctagcagttcatataggagaccggttggaatttctagcatggtggaggccatggtgtctagggcccggattgatgccagggtcgacttcgagtctggacggatcaagaaagaagagttctctgagatcatgaagatctgttccaccattgaagaactcagacggcaaaaataa

>H1N1_PB1_CY097882_A/South Carolina/WRAIR1645P/2009

atggatgtcaatccgactctacttttcctaaaaattccagcgcaaaatgccataagcaccacattcccttatactggagatcctccatacagccatggaacaggaacaggatacaccatggacacagtaaacagaacacaccaatactcagaaaagggaaagtggacgacaaacacagagactggtgcaccccagctcaacccgattgatggaccactacctgaggataatgaaccaagtgggtatgcacaaacagactgtgttctagaggctatggctttccttgaagaatcccacccaggaatatttgagaattcatgccttgaaacaatggaagttgttcaacaaacaagggtagataaactaactcaaggtcgccagacttatgattggacattaaacagaaatcaaccggcagcaactgcattggccaacaccatagaagtctttagatcgaatggcctaacagctaatgagtcaggaaggctaatagatttcttaaaggatgtaatggaatcaatgaacaaagaggaaatagagataacaacccactttcaaagaaaaaggagagtaagagacaacatgaccaagaagatggtcacgcaaagaacaatagggaagaaaaaacaaagactgaataagagaggctatctaataagagcactgacattaaatacgatgaccaaagatgcagagagaggcaagttaaaaagaagggctatcgcaacacctgggatgcagattagaggtttcgtatactttgttgaaactttagctaggagcatttgcgaaaagcttgaacagtctgggctcccagtagggggcaatgaaaagaaggccaaactggcaaatgttgtgagaaagatgatgactaattcacaagacacagagatttctttcacaatcactggggacaacactaagtggaatgaaaatcaaaatcctcgaatgttcctggcgatgattacatatatcaccagaaatcaacccgagtggttcagaaacatcctgagcatggcacccataatgttctcaaacaaaatggcaagactagggaaagggtacatgttcgagagtaaaagaatgaagattcgaacacaaataccagcagaaatgctagcaagcattgacctgaagtacttcaatgaatcaacaaagaagaaaattgagaaaataaggcctcttctaatagatggcacagcatcactgagtcctgggatgatgatgggcatgttcaacatgctaagtacggtcttgggagtctcgatactgaatcttggacaaaagaaatacaccaagacaatatactggtgggatgggctccaatcatccgacgattttgctctcatagtgaatgcaccaaaccatgagggaatacaagcaggagtggacagattctacaggacctgcaagttagtgggaatcaacatgagcaaaaagaagtcctatataaataagacagggacatttgaattcacaagctttttttatcgctatggatttgtggctaattttagcatggagctacccagctttggagtgtctggagtaaatgaatcagctgacatgagtattggagtaacagtgataaagaacaacatgataaacaatgaccttggacctgcaacggcccagatggctcttcaattgttcatcaaagactacagatacacatataggtgccataggggagacacacaaattcagacgagaagatcatttgagttaaagaagctgtgggatcaaacccaatcaaaggtagggctattagtatcagatggaggaccaaacttatacaatatacggaatcttcacattcctgaagtctgcttaaaatgggagctaatggatgatgattatcggggaagactttgtaatcccctgaatccctttgtcagtcataaagagattgattctgtaaacaatgctgtggtaatgccagcccatggtccagccaaaagcatggaatatgatgtcgttgcaactacacattcctggattcccaagaggaatcgttctattctcaacacaagccaaaggggaattcttgaggatgaacagatgtaccagaagtgctgcaatctattcgagaaatttttccctagcagttcatataggagaccggttggaatttcaagcatggtggaggccatggtgtctagggcccggattgatgccagggtcgacttcgagtctggacggatcaagaaagaagagttctctgagatcacgaagatctgttccaccattgaagaactcagacggcaaaaataa

>H1N1_PB1_CY097994_A/Budapest/WRAIR3794T/2011

atggatgtcaatccgactctacttttcctaaaaactccagcgcaaaatgccataagcaccacattcccttatactggagatcctccatacagccatggaacaggaacaggatacaccatggacacagtgaacagaacacaccaatactcagaaaagggaaagtggacgacaaacacagagactggtgcaccccagctcaacccgattgatggaccactacctgaggataatgaaccaagtgggtatgcacaaacagactgtgttctagaggctatggctttccttgaagaatcccacccagggatatttgagaattcatgccttgaaacaatggaagttgttcaacaaacaagggtagataaactaactcaaggtcgccagacttatgattggacattaaacagaaatcaaccggcagcaactgcattggccaacaccatagaagtctttagatcgaatggcctaacagctaatgagtcaggcaggctaatagatttcttaaaggatgtaatggaatcaatgaacaaagaggaaatagagataacaacccactttcaaagaaaaaggagagtaagagacaacatgaccaagaagatggtcacgcaaagaacaatagggaagaaaaaacaaagactgaataagagaggctatctaataagagcactgacattaaatacgatgaccaaagatgcagagagaggcaagttaaaaagaagggctatcgcaacacctgggatgcagattagaggtttcgtatactttgttgaaactttagctaggagcatttgcgaaaagcttgaacagtctgggctcccagtagggggcaatgaaaagaaggccaaactggcaaatgttgtgagaaagatgatgactaattcacaagacacagagatttctttcacaatcactggggacaacactaagtggaatgaaaatcaaaatcctcgaatgttcctggcgatgattacatatatcaccagaaatcaacccgagtggttcagaaacatcctgagcatggcacccataatgttctcaaacaaaatggcaagactagggaaagggtacatgttcgagagtaaaagaatgaagattcgaacacaaataccagcagaaatgctagcaagcattgacctgaagtacttcaatgaatcaacaaagaagaaaattgagaaaataaggcctcttttaatagatggcacagcatcactgagtcctgggatgatgatgggcatgttcaacatgctaagtacggtcttgggagtctcgatactgaatcttggacaaaagaaatacaccaagacaatatactggtgggatgggctccaatcatccgacgattttgctctcatagtgaatgcaccaaaccatgagggaatacaagcaggagtggacagattctacaggacctgcaagttagtgggaatcaacatgagcaaaaagaagtcctatataaataagacagggacatttgaattcacaagctttttttatcgctatggatttgtggctaattttagcatggagctacccagctttggagtgtctggagtaaatgaatcagctgacatgagtattggagtaacagtgataaagaacaacatgataaacaatgaccttggacctgcaacggcccagatggctcttcaattgttcatcaaagactacagatacacatataggtgccataggggagacacacaaattcagacgagaagatcatttgagttaaagaagctgtgggatcaaacccaatcaaaggtagggctattagtatcagatggaggaccaaacttatacaatatacggaatcttcacattcctgaagtctgcttaaaatgggagctaatggatgatgattatcggggaagactttgtaatcccctgaatccctttgtaagtcataaagagattgattctgtaaacaatgctgtggtaatgccagcccatggtccagccaaaagcatggaatatgatgccgttgcaactacacattcctggattcccaagaggaatcgttctattctcaacacaagccaaaggggaattcttgaggatgaacagatgtaccagaagtgctgcaatctattcgagaaatttttccctagcagttcatataggagaccggttggaatttctagcatggtggaggccatggtgtctagggcccggattgatgccagggtcgactttgagtctggacgggtcaagaaagaagagttctctgagatcatgaagatctgttccaccattgaagaactcagacggcaaaaataa

>H1N1_PB1_CY098002_A/Prague/WRAIR4146N/2011

atggatgtcaatccgactctacttttcctaaaaattccagcgcaaaatgccataagcaccacattcccttatactggagatcctccatacagccatggaacaggaacaggatacaccatggacacagtgaacagaacacaccaatactcagaaaagggaaagtggacgacaaacacagagactggtgcaccccagctcaacccgattgatggaccactacctgaggataatgaaccaagtgggtatgcacaaacagactgtgttctagaggctatggctttccttgaagaatcccacccaggaatatttgagaattcatgccttgaaacaatggaagttgttcaacaaacaagggtagataaactaactcaaggtcgccagacttatgattggacattaaacagaaatcaaccggcagcaactgcattggccaacaccatagaagtctttagatcgaatggcctaacagctaatgagtcaggcaggctaatagatttcttaaaggatgtaatggaatcaatgaacaaagaggaaatagagataacaacccactttcaaagaaaaaggagagtaagagacaacatgaccaagaagatggtcacgcaaagaacaatagggaagaaaaaacaaagactgaataagagaggctatctaataagagcactgacattaaatacgatgaccaaagatgcagagagaggcaagttaaaaagaagggctatcgcaacacctgggatgcagattagaggtttcgtatactttgttgaaactttagctaggagcatttgcgaaaagcttgaacagtctgggctcccagtagggggcaatgaaaagaaagccaaattggcaaatgttgtgagaaagatgatgactaattcacaagacacagagatttctttcacaatcactggggacaacactaagtggaatgaaaatcaaaatcctcgaatgttcctggcgatgattacatatatcaccagaaatcaacccgagtggttcagaaacatcctgagcatggcacccataatgttctcaaacaaaatggcaagactagggaaagggtacatgttcgagagtaaaagaatgaagattcgaacacaaataccagcagaaatgctagcaagcattgacctgaagtacttcaatgaatcaacaaagaagaaaattgagaaaataaggcctcttttaatagatggcacagcatcactgagtcctgggatgatgatgggcatgttcaacatgctaagtacggtcttgggagtctcgatactgaatcttggacaaaagaaatacaccaagacaatatactggtgggatgggctccaatcatccgacgattttgctctcatagtgaatgcaccaaaccatgagggaatacaagcaggagtggacagattctacaggacctgcaagttagtgggaatcaacatgagcaaaaagaagtcctatataaataagacagggacatttgaattcacaagctttttttatcgctatggatttgtggctaattttagcatggagctacccagctttggagtgtctggagtaaatgaatcagctgacatgagtattggagtaacagtgataaagaacaacatgataaacaatgaccttggacctgcaacggcccagatggctcttcaattgttcatcaaagactacagatacacatataggtgccataggggagacacacaaattcagacgagaagatcatttgagttaaagaagctgtgggatcaaacccaatcaaaggtagggctattagtatcagatggaggaccaaacttatacaatatacggaatcttcacattcctgaagtctgcttaaaatgggagctaatggatgatgattatcggggaagactttgtaatcccctgaatccctttgtaagtcataaagagattgattctgtaaacaatgctgtggtaatgccagcccatggtccagccaaaagcatggaatatgatgccgttgcaactacacattcctggattcccaagaggaatcgttctattctcaacacaagccaaaggggaattcttgaggatgaacagatgtaccagaagtgctgcaatctattcgagaaatttttccctagcagttcatataggagaccggttggaatttctagcatggtggaggccatggtgtctagggcccggattgatgccagggtcgactttgagtctggacggatcaagaaagaagagttctctgagatcatgaagatctgttccaccattgaagaactcagacggcaaaaataa

>H1N1_PB1_CY098136_A/Bangkok/INS488/2010

atggatgtcaatccgactctacttttcctaaaaattccagcgcaaaatgccataagcaccacattcccttatactggagatcctccatacagccatggaacaggaacaggatacaccatggacacagtaaacagaacacaccaatactcagaaaagggaaagtggacgacaaacacagagactggtgcaccccagctcaacccgattgatggaccactacctgaggataatgaaccaagtgggtatgcacaaacagactgtgttctagaggctatggctttccttgaagaatcccacccaggaatatttgagaattcatgccttgaaacaatggaagttgttcaacaaacaagggtagataaactgactcaaggtcgccagacttatgattggacattaaacagaaatcaaccggcagcaactgcattggccaacaccatagaagtctttagatcgaatggcctaacagctaatgagtcaggaaggctaatagatttcttaaaggatgtaatggaatcaatgaacaaagaggaaatagagataacaacccactttcaaagaaaaaggagagtaagagacaacatgaccaagaagatggtcacgcaaagaacaatagggaagaaaaaacagagactgaataagagaggctatctaataagagcactgacattaaatacgatgaccaaagatgcagagagaggcaagttaaaaagaagggctatcgcaacacctgggatgcagattagaggtttcgtatactttgttgaaactttagctaggagcatttgcgaaaagcttgaacagtctgggctcccagtagggggcaatgaaaagaaggccaaactggcaaatgttgtgagaaagatgatgactaattcacaagacacagagatttctttcacaatcactggggacaacactaagtggaatgaaaatcaaaatcctcgaatgttcctggcgatgattacatatatcaccagaaatcaacccgagtggtttagaaacatcctgagcatggcacccataatgttctcaaacaaaatggcaagactagggaaagggtacatgttcgagagtaaaaggatgaagattcgaacacaaataccagcagaaatgctagcaagcattgacctgaagtacttcaatgaatcaacaaagaagaaaattgagaaaataaggcctcttttaatggatggcacagcatcactgagtcctgggatgatgatgggcatgttcaacatgctaagtacggtcttgggagtctcgatactgaatcttggacaaaagaaatacaccaagacaatatactggtgggatgggctccaatcatccgacgattttgctctcatagtgaatgcaccaaaccatgagggaatacaagcaggggtggacagattctacaggacctgcaagttagtgggaatcaacatgagcaaaaagaagtcctatataaataagacagggacatttgaattcacaagctttttttatcgctatggatttgtggctaattttagcatggagctacccagctttggagtgtctggagtaaatgaatcagctgacatgagtattggagtaacagtgataaagaacaacatgataaacaatgaccttggacctgcaacggcccagatggctcttcaattgttcatcaaagactacagatacacatataggtgccataggggagacacacaaattcagacgagaagatcatttgagttaaagaagctgtgggatcaaacccaatcaaaggtagggctattagtatcagatggaggaccaaacttatacaatatacggaatcttcacattcctgaagtctgcttaaaatgggagctaatggatgatgattatcggggaagactttgtaatcccctgaatccctttgtaagtcataaggagattgattctgtaaacaatgctgtggtaatgccagcccatggtccagccaaaagcatggaatatgatgccgttgcaactacacattcctggattcccaagaggaatcgttctattctcaacacaagccaaaggggaattcttgaggatgaacagatgtaccagaagtgctgcaatctattcgagaaatttttccctagcagttcatataggagaccggttggaatttctagcatggtggaggccatggtgtctagggcccggattgatgccagggtcgactttgagtctggacggatcaagaaagaagagttctctgagatcatgaagatctgttccaccattgaagaactcagacggcaaaaataa

>H1N1_PB1_CY073925_A/Mexico/UASLP-005/2008

atggatgtcaatccgacattacttttcttaaaagtgccagcacaaaatgctataagcacaacttttccttatactggtgaccctccttacagccatgggacaggaacagggtacaccatggatacagtcaacaggacacaccagtactcagaaagaggaagatggacaaaaaataccgaaactggagcaccgcaacttaacccaattgatggtccactaccggaagacaatgaaccaagtggctatgcccaaacagattgtgtattagaagcaatggctttccttgaagaatcccatcccggtatctttgaaaactcttgtattgaaacaatggaggttgttcagcaaacaagggtggacaaactgacacaaggcagacagacctatgactggactctaaataggaaccagcctgctgccacagcattggcaaacactatagaagtattcagatcaaacggcctcatagcaaatgaatctgggaggctaatagacttccttaaagatgtaatggagtcgatggacagaggcgaagtagaggtcacaactcattttcaaagaaagaggagagtgagagacaatgtaactaaaaaaatggtgacccaaagaacaataggcaaaaagaaacataaattagacaaaagaagttacctaattagggcattaaccctgaacacaatgaccaaagatgctgagagggggaaactaaaacgcagagcaattgcaaccccaggaatgcaaataagggggtttgtatactttgttgagacactggcaagaagcatatgtgaaaagcttgaacaatcaggattgccagttggaggaaatgagaagaaagcaaagttagcaaatgtagtaaggaagatgatgaccaactcccaggacactgaaatttctttcaccataaccggagataacacaaaatggaacgaaaatcaaaaccctagaatgttcttggccatgatcacatatataaccaaaaatcagcctgaatggttcagaaatattctaagtattgctccaataatgttttcaaacaagatggcgagactaggtaaggggtacatgtttgaaagcaagagtatgaaactgagaactcaaatacctgcagagatgctagccaacatagatttgaaatatttcaatgattcaactaaaaagaaaattgaaaaaatccgaccattattaatagatggaactgcatcattgagtcctggaatgatgatgggcatgttcaatatgttgagcaccgtcttgggcgtctccattctgaatcttgggcaaaagagatacaccaagactacttactggtgggatggtcttcaatcgtctgatgattttgctttgattgtgaatgcacccaactatgcaggaatacaagctggagttgacaggttttatcgaacctgtaagctgctcggaattaatatgagcaaaaagaagtcttacataaacagaacaggtacccttgaattcacgagctttttctatcgttatgggtttgttgccaatttcagcatggagcttcctagttttggggtgtctggggtcaatgaatctgcagacatgagtattggagtcactgtcatcaaaaacaatatgataaacaatgaccttggcccagcaactgctcaaatggcccttcagttatttataaaagattacaggtacacttatcgatgccaccgaggtgacacacaaatacaaacccggagatcatttgagataaagaaactatgggaccaaacccgctcaaaagctgggctgttggtctctgatggaggccccaatttatataacattagaaatctccatattcctgaagtttgcttgaaatgggagttgatggatgaggattaccaggggcgtttatgcaacccattaaacccgtttgtcagccataaagagattgaatcagtgaacaatgcagtgataatgccggcacatggtccagccaaaaatatggagtatgacgctgttgcaacaacacactcctgggtccccaaaagaaatcgatccattttgaacacgagccaaagggggatacttgaagatgagcaaatgtatcagaggtgctgcaatttatttgaaaaattcttcccaagtagctcatacagaagaccagttggaatatccagtatggtagaggctatggtctcaagagcccgaattgatgcacggattgatttcgaatctggaaggataaagaaagaggaatttgctgagatcatgaagatctgttccaccattgaagacctcagacggcaaaaatga

>H1N1_PB1_JN714506_A/Volgograd/CRIE-DMV/2011

atggatgtcaatccgactctacttttcctaaaaattccagcgcaaaatgctataagcaccacattcccttatactggagatcctccatacagccatggaacaggaacaggatacaccatggacacagtaaacagaacacaccaatactcagaaaagggaaagtggacgacaaacacagagactggtgcaccccagctcaacccgattgatggaccactacctgaggataatgaaccaagtgggtatgcacaaacagactgtgttctagaggctatggctttccttgaagaatcccacccaggaatatttgagaattcatgccttgaaacaatggaagttgttcaacaaacaagggtagataaactaactcaaggtcgccagacttatgattggacattaaacagaaatcaaccggcagcaactgcattggccaacaccatagaagtctttagatcgaatggcctaacagctaatgagtcaggaaggctaatagattttttaaaggatgtaatggaatcaatgaacaaagaggaaatagagataacaacccactttcaaagaaaaaggagagtaagagacaacatgaccaagaagatggtcacgcaaagaacaatagggaagaaaaaacaaagactgaataagagaggctatctaataagagcactgacattaaatacgatgaccaaagatgcagagagaggcaagttaaaaagaagggctatcgcaacacctgggatgcagattagaggtttcgtatactttgttgaagctttagctaggagcatttgcgaaaagcttgaacagtctgggctcccagtagggggcaatgaaaagaaggccaaactggcaaatgttgtgagaaagatgatgactaattcacaagacacagagatttctttcacaatcactggggacaacactaagtggaatgaaaatcaaaatccccgaatgttcctggcaatgattacatatatcaccagaaatcaacccgagtggttcagaaacatcctgagcatggcacccataatgttctcaaacaaaatggcaagactagggaaagggtacatgttcgagagtaaaagaatgaagattcgaacacaaataccagcagaaatgctagcaagcattgacctgaagtacttcaatgaatcaacgaagaagaaaattgagaaaataaggcctcttctaatagatggcacagcatcactgagtcctgggatgatgatgggcatgttcaacatgctaagtacggtcttgggagtctcgatactgaatcttggacaaaagaaatacaccaagacaatatactggtgggatgggctccaatcatccgacgattttgctctcatagtgaatgcaccaaaccatgagggaatacaagcaggagtggacagattctacaggacctgcaagttagtgggaatcaacatgagcaaaaagaaatcctatataaataagacagggacatttgaattcacaagctttttttatcgctatggatttgtggctaattttagcatggagctacccagctttggagtgtctggagtaaatgaatcagctgacatgagtattggagtaacagtgataaagaacaacatgataaacaatgaccttggacctgcaacggcccagatggctcttcaattgttcatcaaagactacagatacacatataggtgccataggggagacacacaaattcagacgagaagatcatttgagttaaagaagctgtgggatcaaacccaatcaaaggtagggctattagtatcagatggaggaccaaacttatacaatatacggaatcttcacattcctgaagtctgcttaaaatgggagctaatggatgatgattatcggggaagactttgtaatcccctgaatccctttgtcagtcataaagagattgattctgtaaacaatgctgtggtaatgccagcccatggtccagtcaaaagcatggaatatgatgccgttgcaactacacattcctggattcccaagaggaatcgttctattctcaacacaagccaaaggggaattcttgaggatgaacagatgtaccagaagtgctgcaatctattcgagaaatttttccctagcagttcatataggagaccggttggaatttctagcatggtggaggccatggtgtctagggcccggattgatgccagggtcgacttcgagtctggacggataaagaaagaagagttctctgagatcatgaagatctgttccaccattgaagaactcagacggcaaaaataa

>H1N1_PB1_CY065260_A/England/XFL00137/2009

atggatgtcaatccgactctacttttcctaaaaattccagcgcaaaatgccataagcaccacattcccttatactggagatcctccatacagccatggaacaggaacaggatacaccatggacacagtaaacagaacacaccaatactcagaaaagggaaagtggacgacaaacacagagactggtgcaccccagctcaacccgattgatggaccactacctgaggataatgaaccaagtgggtatgcacaaacagactgtgttctagaggctatggctttccttgaagaatcccacccaggaatatttgagaattcatgccttgaaacaatggaagttgttcaacaaacaagggtagataaactaactcaaggtcgccagacttatgattggacattaaacagaaatcaaccggcagcaactgcattggccaacaccatagaagtctttagatcgaatggcctaacagctaatgagtcaggaaggctaatagatttcttaaaggatgtaatggaatcaatgaacaaagaggaaatagagataacaacccactttcaaagaaaaaggagagtaagagacaacatgaccaagaagatggtcacgcaaagaacaatagggaagaaaaaacaaagactgaataagagaggctatctaataagagcactgacattaaatacgatgaccaaagatgcagagagaggcaagttaaaaagaagggctatcgcaacacctgggatgcagattagaggtttcgtatactttgttgaaactttagctaggagcatttgcgaaaagcttgaacagtctgggctcccagtagggggcaatgaaaagaaggccaaactggcaaatgttgtgagaaagatgatgactaattcacaagacacagagatttctttcacaatcactggggacaacactaagtggaatgaaaatcaaaatcctcgaatgttcctggcgatgattacatatatcaccagaaatcaacccgagtggttcagaaacatcctgagcatggcacccataatgttctcaaacaaaatggcaagactagggaaagggtacatgttcgagagtaaaagaatgaagattcgaacacaaataccagcagaaatgctagcaagcattgacctgaagtacttcaatgaatcaacaaagaagaaaattgagaaaataaggcctcttctaatagatggcacagcatcactgagtcctgggatgatgatgggcatgttcaacatgctaagtacggtcttgggagtctcgatactgaatcttggacaaaagaaatacaccaagacagtatactggtgggatgggctccaatcatccgacgattttgctctcatagtgaatgcaccaaaccatgagggaatacaagcaggagtggacagattctacaggacctgcaagttagtgggaatcaacatgagcaaaaagaagtcctatataaataagacagggacatttgaattcacaagctttttttatcgctatggatttgtggctaattttagcatggagctacccagctttggagtgtctggagtaaatgaatcagctgacatgagtattggagtaacagtgataaagaacaacatgataaacaatgaccttggacctgcaacggcccagatggctcttcaattgttcatcaaagactacagatacacatataggtgccataggggagacacacaaattcagacgagaagatcatttgagttaaagaagctgtgggatcaaacccaatcaaaggtagggctattagtatcagatggaggaccaaacttatacaatatacggaatcttcacattcctgaagtctgcttaaaatgggagctaatggatgatgattatcggggaagactttgtaatcccctgaatccctttgtcagtcataaagagattgattctgtaaacaatgctgtggtaatgccagcccatggtccagccaaaagcatggaatatgatgccgttgcaactacacattcctggattcccaagaggaatcgttctattctcaacacaagccaaaggggaattcttgaggatgaacagatgtaccagaagtgctgcaatctattcgagaaatttttccctagcagttcatataggagaccggttggaatttctagcatggtggaggccatgatgtctagggcccggattgatgccagggtcgacttcgagtctggacggatcaagaaagaagagttctctgagatcatgaagatctgttccaccattgaagaactcagacggcaaaaataa

>H1N1_PB1_CY065644_A/England/402/2009

atggatgtcaatccgactctacttttcctaaaaattccagcgcaaaatgccataagcaccacattcccttatactggagatcctccatacagccatggaacaggaacaggatacaccatggacacagtaaacagaacacaccaatactcagaaaagggaaagtggacgacaaacacagagactggtgcaccccagctcaacccgattgatggaccactacctgaggataatgaaccaagtgggtatgcacaaacagactgtgttctagaggctatggctttccttgaagaatcccacccaggaatatttgagaattcatgccttgaaacaatggaagttgttcaacaaacaagggtagataaactaactcaaggtcgccagacttatgattggacattaaacagaaatcaaccggcagcaactgcattggccaacaccatagaagtctttagatcgaatggcctaacagctaatgagtcaggaaggctaatagatttcttaaaggatgtaatggaatcaatgaacaaagaggaaatagagataacaacccactttcaaagaaaaaggagagtaagagacaacatgaccaagaagatggtcacgcaaagaacaatagggaagaaaaaacaaagactgaataagagaggctatctaataagagcactgacattaaatacgatgaccaaagatgcagagagaggcaagttaaaaagaagggctatcgcaacacctgggatgcagattagaggtttcgtatactttgttgaaactttagctaggagcatttgcgaaaagcttgaacagtctgggctcccagtagggggcaatgaaaagaaggccaaactggcaaatgttgtgagaaagatgatgactaattcacaagacacagagatttctttcacaatcactggggacaacactaagtggaatgaaaatcaaaatcctcgaatgttcctggcgatgattacatatatcaccagaaatcaacccgagtggttcagaaacatcctgagcatggcacccataatgttctcaaacaaaatggcaagactagggaaagggtacatgttcgagagtaaaagaatgaagattcgaacacaaataccagcagaaatgctagcaagcattgacctgaagtacttcaatgaatcaacaaagaagaaaattgagaaaataaggcctcttctaatagatggcacagcatcactgagtcctgggatgatgatgggcatgttcaacatgctaagtacggtcttgggagtctcgatactgaatcttggacaaaagaaatacaccaagacaatatactggtgggatgggctccaatcatccgacgattttgctctcatagtgaatgcaccaaaccatgagggaatacaagcaggagtggacagattctacaggacctgcaagttagtgggaatcaacatgagcaaaaagaagtcctatataaataagacagggacatttgaattcacaagctttttttatcgctatggatttgtggctaattttagcatggagctacccagctttggagtgtctggagtaaatgaatcagctgacatgagtattggagtaacagtgataaagaacaacatgataaacaatgaccttggacctgcaacggcccagatggctcttcaattgttcatcaaagactacagatacacatataggtgccataggggagacacacaaattcagacgagaagatcatttgagttaaagaagctgtgggatcaaacccaatcaaaggtagggctattagtatcagatggaggaccaaacttatacaatatacggaatcttcacattcctgaagtctgcttaaaatgggaactaatggatgatgattatcggggaagactttgtaatcccctgaatccctttgtcagtcataaagagattgattctgtaaacaatgctgtggtaatgccagcccatggtccagccaaaagcatggaatatgatgccgttgcaactacacattcctggattcccaagaggaatcgttctattctcaacacaagccaaaggggaattcttgaggatgaacagatgtaccagaagtgctgcaatctattcgagaaatttttccctagcagttcatataggagaccggttggaatttctagcatggtggaggccatggtgtctagggcccggattgatgccagggtcgacttcgagtctggacggatcaagaaagaagagttctctgagatcatgaagatctgttccaccattgaagaactcagacggcaaaaataa

>H1N1_PB1_CY095876_A/Hubei/76/2009

atggatgtcaatccgactctacttttcctaaaaattccagcgcaaaatgccataagcaccacattcccttatactggagatcctccatacagccacggaacaggaacaggatacaccatggacacagtaaacagaacacaccaatactcagaaaagggaaagtgggcgacaaacacggagactggtgcaccccagctcaacccgattgatggaccactacctgaggataatgaaccaagtgggtatgcacaaacagactgtgttctagaggctatggctttccttgaagaatcccacccaggaatatttgagaattcatgccttgaaacaatggaagttgttcaacaaacaagggtagataaactaactcaaggttgccagacttatgattggacattaaacagaaatcaaccggcagcaactgcattggccgacaccatagaagtctttagatcgaatggcctaacagctaatgagtcaggaaggctaatagatttcttaaaggatgtaatggaatcaatgaacaaagaggaaatagagataacaacccactttcaaagaaaaaggagagtaagagacaacatgaccaagaagatggtcacgcaaagaacaatagggaagaaaaaacaaagactgaataagagaggctatctaataagagcactgacattaaatacgatgaccaaagatgcagagagaggcaagttaaaaagaagggctatcgcaacacctgggatgcagattagaggtttcgtatactttgttgaaactttagctaggagcatttgcgaaaagcttgaacagtctgggctcccagtagggggcaatgaaaagaaggccaaactggcaaatgttgtgagaaagatgatgactaattcacaagacacagagatttctttcacaatcaccggggacaacactaagtggaatgaaaatcaaaatcctcgaatgttcctggcgatgattacatatatcaccagaaatcaacccgagtggttcagaaacatcctgagcatggcacccataatgttctcaaacaaaatggcaagactagggaaagggtacatgttcgagagtagaagaatgaagattcgaacacaaataccagcagaaatgctagcaagcattgacctgaagtacttcaatgaatcaacaaagaagaaaattgagaaaataaggcctcttctaatagatggcacagcatcactgagtcctgggatgatgatgggcatgttcaacatgctaagtacggtcttgggagtctcgatactgaatcttggacaaaagaaatacaccaagacaatatactggtgggatgggctccaatcatccgacgattttgctctcatagtgaatgcaccaaaccatgagggaatacaagcaggagtggacagattctacaggacctgcaagttagtgggaatcaacatgagcaaaaagaagtcctatataaataagacagggacatttgaattcacaagctttttttatcgctatggatttgtggctaattttagcatggagctacccagctttggagtgtctggagtaaatgaatcagctgacatgagtattggagtaacagtgataaagaacaacatgataaacaatgaccttggacctgcaacggcccagatggctcttcaattgttcatcaaagactacagatacacatataggtgccataggggagacacacaaattcagacgagaagatcatttgagttaaagaagctgtgggatcaaacccaatcaaaggtagggctattagtatcagatggaggaccaaacttatacaatataccgaatcttcacattcctgaagtctgcttaaaatgggagctaatggatgatgattatcggggaagactttgtaatcccctgaatccctttgtcagtcataaagagattgattctgtaaacaatgctgtggtaatgccagcccatggtccagccaaaagcatggaatatgatgccgttgcaactacacattcctggattcccaagaggaatcgttctattctcaacacaagccaaaggggaattcttgaggatgaacagatgtaccagaagtgctgcaatctattcgagaaatttttccctagcagttcatataggagaccggttggaatttctagcatggtggaggccatggtgtctagggcccggattgatgccagggtcgacttcgagtctggacggatcaagaaagaagagttctctgagatcatgaagatctgttccaccattgaagaactcagacggcaaaaataa

>H1N1_PB1_CY095904_A/Zhejiang/8/2009

atggatgtcaatccgactctacctttcctaaaaattccagcgcaaaatgccataagcaccacattcccttatactggagatcctccatacagccatggaacaggaacaggatacaccatggacacagtaaacagaacacaccaatactcagaaaagggaaagtggacgacaaacacagagactggtgcaccccagctcaacccgattgatggaccactacctgaggataatgaaccaagtgggtatgcacaaacagactgtgttctagaggctatggctttccttgaagaatcccacccaggaatatttgagaattcatgccttgaaacaatggaagttgttcaacaaacaagggtagataaactaactcaaggtcgccagacttatgattggacattaaacagaaatcaaccggcagcaactgcattggccaacaccatagaagtctttagatcgaatggcctaacagctaatgagtcaggaaggctaatagatttcttaaaggatgtaatggaatcaatgaacaaagaggaaatagagataacaacccactttcaaagaaaaaggagagtaagagacaacatgaccaagaagatggtcacgcaaagaacaatagggaagaaaaaacaaagactgaataagagaggctatctaataagagcactgacattaaatacgatgaccaaagatgcagagagaggcaagttaaaaagaagggctatcgcaacacctgggatgcagattagaggtttcgtatactttgttgaaactttagctaggagcatttgcgaaaagcttgaacagtctgggctcccagtagggggcaatgaaaagaaggccaaactggcaaatgttgtgagaaagatgatgactaattcacaagacacagagatttctttcacaatcactggggacaacactaagtggaatgaaaatcaaaatcctcgaatgttcctggcgatgattacatatatcaccagaaatcaacccgaatggttcagaaacatcctgagcatggcacccataatgttctcaaacaaaatggcaagactagggaaagggtacatgttcgagagtaaaagaatgaagattcgaacacaaataccagcagaaatgctagcaagcattgacctgaagtacttcaatgaatcaacaaagaagaaaattgagaaaataaggcctcttctaatagatggcacagcatcactgagtcctgggatgatgatgggcatgttcaacatgctaagtacggtcttgggagtctcgatactgaatcttggacaaaagaaatacaccaagacaatatactggtgggatgggctccaatcatccgacgattttgctctcatagtgaatgcaccaaaccatgagggaatacaagcaggagtggacagattctacaggacctgcaagttagtgggaatcaacatgagcaaaaagaagtcctatataaataagacagggacatttgaattcacaagctttttttatcgctatggatttgtggctaattttagcatggagctacccagctttggagtgtctggagtaaatgaatcagctgacatgagtattggagtaacagtgataaagaacaacatgataaacaatgaccttggacctgcaacggcccagatggctcttcaattgttcatcaaagactacagatacacatataggtgccataggggagacacacaaattcagacgagaagatcatttgagttaaagaagctgtgggatcaaacccaatcaaaggtagggctattagtatcagatggaggaccaaacttatacaatatacggaatcttcacattcctgaagtctgcttaaaatgggagctaatggatgatgactatcggggaagactttgtaatcccctgaatccctttgtcagtcataaagagattgattctgtaaacaatgctgtggtaatgccagcccatggtccagccaaaagcatggaatatgatgccgttgcaactacacattcctggattcccaagaggaatcgttctattctcaacacaagccaaaggggaattcttgaggatgaacagatgtaccagaagtgccgcaatctattcgagaaatttttccctagcagttcatataggagaccggttggaatttctagcatggtggaggccatggtgtctagggcccggattgatgccagggtcgacttcgagtctggacggatcaagaaagaagagttctctgagatcatgaagatctgctccaccattgaagaactcagacggcaaaaataa

>H1N1_PB1_JQ173162_A/Helsinki/P18/2009

atggatgtcaatccgactctacttttcctaaaaattccagcgcaaaatgccataagcaccacattcccttatactggagatcctccatacagccatggaacaggaacaggatacaccatggacacagtaaacagaacacaccaatactcagaaaagggaaagtggacgacaaacacagagactggtgcaccccagctcaacccgattgatggaccactacctgaggataatgaaccaagtgggtatgcacaaacagactgtgttctagaggctatggctttccttgaagaatcccacccaggaatattcgagaattcatgccttgaaacaatggaagttgttcaacaaacaagggtagataaactaactcaaggtcgccagacttatgattggacattaaacagaaatcaaccggcagcaactgcattggccaacaccatagaagtctttagatcgaatggcctaacagctaatgagtcaggaaggctaatagatttcttaaaggatgtaatggaatcaatgaacaaagaggaaatagagataacaacccactttcaaagaaaaaggagagtaagagacaacatgaccaagaagatggtcacgcaaagaacaatagggaagaaaaaacaaagactgaataagagaggctatctaataagagcactgacattaaatacgatgaccaaagatgcagagagaggcaagttaaaaagaagggctatcgcaacacctgggatgcagattagaggtttcgtatactttgttgaaactttagctaggagcatttgcgaaaagcttgaacagtctgggctcccagtagggggcaatgaaaagaaggccaaactggcaaatgttgtgagaaagatgatgactaattcacaagacacagagatttctttcacaatcactggggacaacactaagtggaatgaaaatcaaaatcctcgaatgttcctggcgatgattacatatatcaccagaaatcaacccgagtggttcagaaacatcctgagcatggcacccataatgttctcaaacaaaatggcaagactagggaaagggtacatgttcgagagtaaaagaatgaagattcgaacacaaataccagcagaaatgctagcaagcattgacctgaagtacttcaatgaatcaacaaagaagaaaattgagaaaataaggcctcttctaatagatggcacagcatcattgagtcctgggatgatgatgggcatgttcaacatgctaagtacggtcttgggagtctcgatactgaatcttggacaaaagaaatacaccaagacaatatactggtgggatgggctccaatcatccgacgattttgctctcatagtgaatgcaccaaaccatgagggaatacaagcaggagtggacagattctacaggacctgcaagttagtgggaatcaacatgagcaaaaagaagtcctatataaataagacagggacatttgaattcacaagctttttttatcgctatggatttgtggctaattttagcatggagctacccagctttggagtgtctggagtgaatgaatcagctgacatgagtgttggagtaacagtgataaagaacaacatgataaacaatgaccttggacctgcaacggcccagatggctctccaattgttcatcaaagactacagatacacatataggtgccatargggagacacacaaattcagacgagaagatcatttgagttaaagaagctgtgggatcaaacccaatcaaaggtagggctattagtatcagatggaggaccaaacttatacaatatacggaatcttcacattcctgaagtctgcttaaaatgggagctaatggatgatgattatcggggaagactttgtaatcccctgaatccctttgtcagtcataaagagattgattctgtaaacaatgctgtggtaatgccagcccatggtccagccaaaagcatggaatatgatgccgttgcaactacacattcctggattcccaagaggaatcgttctattctcaacacaagccaaaggggaattcttgaggatgaacagatgtaccagaagtgctgcaatctattcgagaaatttttccctagcagttcatataggagaccggttggaatttctagcatggtggaggccatggtgtctagggcccggattgatgccagggtcgacttcgagtctggacggatcaagaaagaagagttctctgagatcatgaagatctgttccaccattgaagaactcagacggcaaaaatag

>H1N1_PB1_CY103986_A/HaNoi/2253/2001

atggatgtcaatccgacattacttttcttaaaagtgccagcacaaaatgctataagcacaacttttccttatactggtgaccccccttacagccatgggacaggaacaggatacaccatggatacagtcaacaggacacatcagtactcagaaagaggaagatggacaaaaaataccgaaactggagcaccgcaactcaacccaattgatgggccactaccagaagacaatgaaccaagtggctatgcccaaacagattgtgtattagaagcaatggctttccttgaagaatcccatcctggtatttttgaaaactcttgtattgaaacaatggaggttgttcagcaaacaagggtggacaaactgacacaaggcagacagacctatgactggactctaaataggaaccagcctgctgccacagcattggccaacactatagaagtattcagatcaaacggcctcatagcaaatgaatctgggaggctaatagacttccttaaagatgtaatggagtcgatggacagagacgaagtagaggtcacaactcattttcagagaaagaggagggtgagagacaatgtaactaaaaaaatggtgacccaaagaacaataggcaaaaagaaacataaattagacaaaagaagttacctaattagggcattaaccctgaacacaatgaccaaagatgctgagagggggaaactaaaacgcagagcaattgcaaccccaggaatgcaaataagggggtttgtatactttgttgagacactggcaagaagcatatgtgaaaagcttgaacaatcagggttgccagttggaggaaatgagaagaaagcaaagttagcaaatgttgtaaggaagatgatgaccaactcccaggacactgaaatttctttcaccatcactggagataacacaaaatggaacgaaaatcaaaaccctagaatgttcttggccatgatcacatatataaccaaaaatcagcctgaatggttcagaaatattctaagtattgctccaataatgttttcaaacaaaatggcgagactaggtaaggggtacatgtttgaaagcaagagtatgaaactgagaactcaaatacctgcagagatgctagccaacatagatttgaaatatttcaatgattcaactaaaaagaaaattgaaaaaatccgaccattattaatagatggaactgcatcattgagtcctggaatgatgatgggcatgttcaatatgttaagcaccgtcttgggcgtctccattctgaatcttgggcaaaagagatacaccaagactacttactggtgggatggtcttcaatcgtctgatgattttgctctgattgtgaatgcacccaactatgcaggaattcaagctggagttgacaggttttatcgaacctgtaagctgctcggaattaatatgagcaaaaagaagtcttacataaacagaacaggtacctttgaattcacgagctttttctatcgttatgggtttgttgccaatttcagcatggagcttcctagttttggggtgtctggggtcaatgaatctgcagacatgagtattggagtcactgtcatcaaaaacaatatgataaacaatgaccttggcccagcaactgctcaaatggcccttcagttatttataaaagattacaggtacacgtatcgatgccacagaggtgacacacaaatacaaacccggagatcatttgagataaagaaactatgggaccaaacccgctccaaagctgggctgttggtctctgatggaggccccaatttatataacattagaaatctccatattcctgaagtctgcttgaaatgggagttgatggatgaggattaccaggggcgtttatgcaacccattgaacccgtttgtcagtcataaagagattgaatcagtgaacaatgcagtgatgatgccggcacatggtccagccaaaaatatggagtatgacgctgttgcaacaacacactcctgggttcccaaaaggaatcgatccattttgaacacgagccaaagggggatacttgaggatgagcaaatgtatcagaggtgctgcaatttatttgaaaaattcttcccaagtagctcatacagaagaccagttggaatatccagtatggtagaggctatggtttccagagcccgaattgatgcacggattgatttcgaatctggaaggataaagaaagaggaattcactgagatcatgaagacctgttccaccattgaagacctcagacggcaaaaatag

>H1N1_PB1_CY104010_A/HaNoi/2546/2002

atggatgtcaatccgacattacttttcttaaaagtgccagcacaaaatgctataagcacaacttttccttatactggtgaccccccttacagccatgggacaggaacaggatacaccatggatacagtcaacaggacacatcagtactcagaaagagggagatggacaaaaaataccgaaactggagcaccgcaactcaacccaattgatgggccactaccagaagacaatgaaccaagtggctatgcccaaacagattgtgtattagaagcaatggctttccttgaagaatcccatcctggtatttttgaaaactcttgtattgaaacaatggaggttgttcagcaaacaagggtggacaaactgacacaaggcagacagacctatgactggactctaaataggaaccagcctgctgccacagcattggccaacactatagaagtattcagatcaaacggcctcatagcaaatgaatctgggaggctaatagacttccttaaagatgtaatggagtcgatggacagagacgaagtagaggtcacaactcattttcagagaaagaggagagtgagagacaatgtaactaaaaaaatggtgacccaaagaacaataggcaaaaagaaacataaattagacaaaagaagttacctaattagggcattaaccctgaacacaatgaccaaagatgctgagagggggaaactaaaacgcagagcaattgcaaccccaggaatgcaaataagggggtttgtatactttgttgagacactggcaagaagcatatgtgaaaagcttgaacaatcagggttgccagttggaggaaatgagaagaaagcaaagttagcaaatgttgtaaggaagatgatgaccaactcccaggacactgaaatttctttcaccatcactggagataacacaaaatggaacgaaaatcaaaaccctagaatgttcttggccatgatcacatatataaccaaaaatcagcctgaatggttcagaaatattctaagtattgctccaataatgttttcaaacaaaatggcgagactaggtaaggggtacatgtttgaaagcaagagtatgaaactgagaactcaaatacctgcagagatgctagccaacatagatttgaaatatttcaatgattcaactaaaaagaaaattgaaaaaatccgaccattattaatagatggaactgcatcattgagtcctggaatgatgatgggaatgttcaatatgttaagcaccgtcttgggcgtttccattctgaatcttgggcaaaagagatacaccaagactacttactggtgggatggtcttcaatcgtctgatgattttgctctgattgtgaatgcacccaactatgcaggaattcaagctggagttgacaggttttatcgaacctgtaagctgctcggaattaatatgagcaaaaagaagtcttacataaacagaacaggtacctttgaattcacgagctttttctatcgttatgggtttgttgccaatttcagcatggagcttcctagttttggggtgtctggggtcaatgaatctgcagacatgagtattggagtcactgtcatcaaaaacaatatgataaacaatgaccttggcccagcaactgctcaaatggcccttcagttatttataaaagattacaggtacacgtatcgatgccacagaggtgacacacaaatacaaacccggagatcatttgagataaagaaactatgggaccaaacccgctccaaagctgggctgttggtctctgatggaggccccaatttatataacattagaaatctccatattcctgaagtctgcttgaaatgggagttgatggatgaggattaccaggggcgtttatgcaacccattgaacccgtttgtcagtcataaagagattgaatcagtgaacaatgcagtgatgatgccggcacatggtccagccaaaaatatggagtatgacgctgttgcaacaacacactcctgggttcccaaaaggaatcgatccattttgaacacgagccaaagggggatacttgaggatgagcaaatgtatcagaggtgctgcaatttatttgaaaaattcttcccaagtagctcatacagaagaccagttggaatatccagtatggtagaggctatggtttccagagcccgaattgatgcacggattgatttcgaatctggaaggataaagaaagaggaattcactgagatcatgaagacctgttccaccattgaagacctcagacggcaaaaatag

>H1N1_PB1_CY104314_A/TayNguyen/TN161/2006

atggatgtcaatccgacattacttttcttaaaagtgccagcacaaaatgctataagcacaacttttccttatactggtgaccctccttacagccatgggacaggaacagggtacacaatggatacagtcaacaggacacatcagtactcagaaagaggaagatggacaaagaataccgaaactggagcaccgcaactcaacccaattgatgggccactaccagaagacaatgaaccaagtggctatgcccaaacagattgtgtattagaagcaatggctttccttgaagaatcccatcctggcatttttgaaaactcttgtattgaaacaatggaggttgttcagcaaacaagggtggacaaactgacacaaggcagacagacctatgactggactctaaataggaaccagcctgctgccacagcattggccaacactatagaagtattcagatcaaacggcctcatagcaaatgaatctgggaggctaatagacttccttaaagatgtaatggagtcgatggacagagacgaagtagaggtcacaactcattttcagagaaagaggagagtgagagacaatgtaactaaaaaaatggtgacccaaagaacaataggcaaaaagaaacataaattagacaaaaggagttacctaattagagcattaaccctgaacacaatgaccaaggatgctgagagggggaaactaaaacgcagagcaattgcaaccccaggaatgcaaataagggggtttgtatactttgttgagacactggcaagaagcatatgtgaaaagcttgaacaatcagggttgcccgttggagggaacgagaagaaagcaaagttagcaaatgttgtaaggaagatgatgaccaactcccaggacactgaaatttctttcaccatcactggagataacacaaaatggaacgaaaatcaaaaccctagaatgtttttggccatgatcacatatataaccagaaatcagcctgaatggttcagaaatattctaagtattgctccaataatgttttcaaacaaaatggcgagactaggtaaggggtacatgtttgaaagcaagagtatgaaactgagaactcaaatacctgcagagatactagccgacatagatttgaaatatttcaatgagtcaactaaaaagaaaattgaaaaaatccgaccattattaatagatggaactgcatcattgagccctggaatgatgatgggaatgttcaatatgttaagcactgtcttgggcgtctccattctgaatcttgggcaaaagagatacaccaagaccacttactggtgggatggtcttcaatcgtctgatgattttgctctgatagtgaatgcgcccaactatgcaggaattcaagctggagttgacaggttttatcgaacctgtaagctgctcggaattaatatgagcaaaaagaagtcttacataaataggacaggtacctttgaattcacgagctttttctatcgttatgggtttgttgccaatttcagcatggagcttcctagttttggggtgtctggggtcaatgaatctgcagacatgagtattggagtcactgttatcaaaaacaatatggtaaacaatgaccttggcccagcaactgctcaaatggcccttcagttatttataaaagattacagatacacgtatcgatgccacagaggtgacacacaaatacaaacccggagatcatttgagataaagaaattatgggaccaaacccgctccaaagctgggctgttggtctctgatggaggccccaatctatataacattagaaatctccatattcctgaagtctgcttgaaatgggagttgatggatgaggattaccaggggcgtttatgcaacccattgaacccgtttgtcagtcataaagagattgaatcagtgaacaatgcagtgatgatgccggcacatggtccagccaaaaatatggagtatgacgctgttgcaacaacacattcctgggttcccaaaaggaatcgatccattttgaacacgagccaaagggggatacttgaggatgagcaaatgtatcagaggtgctgcaatttatttgaaaaattcttcccaagtagctcatacagaagaccagttgggatatccagtatggtagaggctatgatttccagagcccgaattgatgcacggattgatttcgaatctggaaggataaagaaagaggaattcgctgagatcatgaagacctgttccaccattgaagacctcagacggcaaaaatag

>H1N1_PB1_CY104788_A/HaNoi/HN756/2003

atggatgtcaatccgacattacttttcttaaaagtgccagcacaaaatgctataagcacaacttttccttatactggtgaccccccttacagccatgggacaggaacaggatacaccatggatacagtcaacaggacacatcagtactcagaaagagggagatggacaaaaaataccgaaactggagcaccgcaactcaacccaattgatgggccactaccagaagacaatgaaccaagtggctatgcccaaacagattgtgtattagaagcaatggctttccttgaagaatcccatcctggtatttttgaaaactcttgtattgaaacaatggaggttgttcagcaaacaagggtggacaaactgacacaaggcagacagacctatgactggactctaaataggaaccagcctgctgccacagcattggccaacactatagaagtattcagatcaaacggcctcatagcaaatgaatctgggaggctaatagacttccttaaagatgtaatggagtcgatggacagagacgaagtagaggtcacaactcattttcagagaaagaggagagtgagagacaatgtaactaaaaaaatggtgacccaaagaacaataggcaaaaagaaacataaattagacaaaagaagttacctaattagggcattaaccctgaacacaatgaccaaagatgctgagagggggaaactaaaacgcagagcaattgcaaccccaggaatgcaaataagggggtttgtatactttgttgagacactggcaagaagcatatgtgaaaagcttgaacaatcagggttgccaattggaggaaatgagaagaaagcaaagttagcaaatgttgtaaggaagatgatgaccaactcccaggacactgaaatttctttcaccatcactggagataacacaaaatggaacgaaaatcaaaaccctagaatgttcttggccatgatcacatatataaccaaaaatcagcctgaatggttcagaaatattctaagtattgctccaataatgttttcaaacaaaatggcgagactaggtaaggggtacatgtttgaaagcaagagtatgaaactgagaactcaaatacctgcagagatgctagccaacatagatttgaaatatttcaatgattcaactaaaaagaaaattgaaaaaatccgaccattattaatagatggaactgcatcattgagtcctggaatgatgatgggaatgttcaatatgttaagcaccgtcttgggcgtttccattctgaatcttgggcaaaagagatacaccaagactacttactggtgggatggtcttcaatcgtctgatgattttgctctgattgtgaatgcacccaactatgcaggaattcaagctggagttgacaggttttatcgaacctgtaagctgctcggaattaatatgagcaaaaagaagtcttacataaacagaacaggtacctttgaattcacgagctttttctatcgttatgggtttgttgccaatttcagcatggagcttcctagttttggggtgtctggggtcaatgaatctgcagacatgagtattggagtcactgtcatcaaaaacaatatgataaacaatgaccttggcccagcaactgctcaaatggcccttcagttatttataaaagattacaggtacacgtatcgatgccacagaggtgacacacaaatacaaacccggagatcatttgagataaagaaactatgggaccaaacccgctccaaagctgggctgttggtctctgatggaggccccaatttatataacattagaaatctccatattcctgaagtctgcttgaaatgggagttgatggatgaggattaccaggggcgtttatgcaacccattgaacccgtttgtcagtcataaagagattgaatcagtgaacaatgcagtgatgatgccggcacatggtccagccaaaaatatggagtatgacgctgttgcaacaacacactcctgggttcccaaaaggaatcgatccattttgaacacgagccaaagggggatacttgaggatgagcaaatgtatcagaggtgctgcaatttatttgaaaaattcttcccaagtagctcatacagaagaccagttggaatatccagtatggtagaggctatggtttccagagcccgaattgatgcacggattgatttcgaatctggaaggataaagaaagaggaattcactgagatcatgaagacctgttccaccattgaagacctcagacggcaaaaatag

>H1N1_PB1_CY104844_A/HaNoi/Q421/2006

atggatgtcaatccgacattacttttcttaaaagtgccagcacaaaatgctataagcacaacttttccttatactggtgaccctccttacagccatgggacaggaacagggtacacaatggatacagtcaacaggacacatcagtactcagaaagaggaagatggacaaagaataccgaaactggagcaccgcaactcaacccaattgatgggccactaccagaagacaatgaaccaagtggctatgcccaaacagattgtgtattagaagcaatggctttccttgaagaatcccatcctggcatttttgaaaactcttgtattgaaacaatggaggttgttcagcaaacaagggtggacaaactgacacaaggcagacagacctatgactggactctaaatagaaaccagcctgctgccacagcattggccaacactatagaagtattcagatcaaacggcctcatagcaaatgaatctgggaggctaatagacttccttaaagatgtaatggagtcgatggacagagacgaagtagaggtcacaactcattttcagagaaagaggagagtgagagacaatgtaactaaaaaaatggtgacccaaagaacaataggcaaaaagaaacataaattagacaaaaggagttacctaattagagcattaaccctgaacacaatgaccaaagatgctgagagggggaaactaaaacgcagagcaattgcaaccccaggaatgcaaataagggggtttgtatactttgttgagacactggcaagaagcatatgtgaaaagcttgaacaatcagggttgcccgttggagggaacgagaagaaagcaaagttagcaaatgttgtaaggaagatgatgaccaactcccaggacactgaaatttctttcaccatcactggagataacacaaaatggaacgaaaatcaaaaccctagaatgtttttggccatgatcacatatataaccagaaatcagcctgaatggttcagaaatattctaagtattgctccaataatgttttcaaacaaaatggcgagactaggtaaggggtacatgtttgaaagcaagagtatgaaactgagaactcaaatacctgcagagatactagccgacatagatttgaaatatttcaatgagtcaaccaaaaagaaaattgaaaaaatccgaccattattaatagatggaactgcatcattgagccctggaatgatgatgggaatgttcaatatgttaagcactgtcttgggcgtctccattctgaatcttgggcaaaagagatacaccaagaccacttactggtgggatggtcttcaatcgtctgatgattttgctctgatagtgaatgcgcccaactatgcaggaattcaagctggagttgacaggttttatcgaacctgtaagctgctcggaattaatatgagcaaaaagaagtcttacataaataggacaggcacctttgaattcacgagctttttctatcgttatgggtttgttgccaatttcagcatggagcttcctagttttggggtgtctggggtcaatgaatctgcagacatgagtattggagtcactgttatcaaaaacaatatgataaacaatgaccttggcccagcaactgctcaaatggcccttcagttatttataaaagattacagatacacgtatcgatgccacagaggtgacacacaaatacaaacccggagatcatttgagataaagaaattatgggaccaaacacgctccaaagctgggctgttggtctctgatggaggccccaatctatataacattagaaatctccatattcctgaagtctgcttgaaatgggagttgatggatgaggattaccaggggcgtttatgcaacccattgaacccgtttgtcagtcataaagagattgaatcagtgaacaatgcagtgatgatgccggcacatggtccagccaaaaatatggagtatgacgctgttgcaacaacacattcctgggttcccaaaaggaatcgatccattttgaacacgagccaaagggggatacttgaggatgagcaaatgtatcagaggtgctgcaatttattcgaaaaattcttcccaagtagctcatacagaagaccagttgggatatccagtatggtagaggctatgatttccagagcccgaattgatgcacggattgatttcgaatctggaaggataaagaaagaggaattcgctgagatcatgaagacctgttccaccattgaagacctcagacggcaaaaatag

>H1N1_PB1_CY105156_A/HaNoi/TX233/2008

atggatgtcaatccgacattacttttcttaaaagtgccagcacaaaatgctataagcacaacttttccttatactggtgaccctccttacagccatgggacaggaacagggtacaccatggatacagtcaacaggacacaccagtactcagaaagaggaagatggacaaaaaataccgaaacgggagcaccgcaacttaacccaattgatggtcccttaccggaagacaatgaaccaagtggctatgcccaaacagattgtgtattagaagcaatggctttccttgaagaatcccatcccggtatctttgaaaactcttgtattgaaacaatggaggttgttcaacaaacaagggtggacaaactgacacaaggcagacaaacctatgactggactctaaataggaaccagcctgctgccacagcattggcaaacactatagaagtattcagatcaaacggcctcatagcaaatgaatctgggaggctaatagacttccttaaagatgtaatggagtcgatggacagaggcgaagtagaggtcacaactcattttcaaagaaagaggagagtgagagacaatgtaactaaaaaaatggtgacccaaagaacaataggcaaaaagaaacataaattagacaaaagaagttacctaattagggcattaaccctgaacacaatgaccaaagatgctgagagggggaaactaaaacgcagagcaattgcaaccccaggaatgcaaataagggggtttgtatactttgttgagacactggcaagaagcatatgtgaaaagcttgaacaatcaggattgccagttggaggaaatgagaagaaagcaaagttagcaaatgttgtaaggaagatgatgaccaactcccaggacactgaaatttctttcaccataaccggagataacacaaaatggaacgaaaatcaaaaccctagaatgttcttggccatgatcacatatataaccaaaaatcagcctgaatggttcagaaatattctaagtattgctccaataatgttttcaaacaagatggcgagactaggtaaggggtacatgtttgaaagcaagagtatgaaactgagaactcaaatacctgcagagatgctagccaacatagacttgaaatatttcaatgattcaactaaaaagaaaattgaaaaaatccgaccattattaatagatggaactgcatcattgagtcctggaatgatgatgggcatgttcaatatgttgagcaccgtcttgggcgtctccattctgaatcttgggcaaaagagatacaccaagactacttactggtgggatggtcttcaatcgtctgatgattttgctttgattgtgaacgcacccaactatgcaggaattcaagctggagttgacaggttttatcgaacctgtaagctgctcggaattaatatgagcaaaaagaagtcttacataaacagaacaggtacctttgaattcacgagctttttctatcgttatggatttgttgccaatttcagcatggagcttcctagttttggggtgtctggggtcaatgaatctgcagacatgagtattggagtcactgtcatcaaaaacaatatgataaacaatgaccttggcccagcaactgctcaaatggcccttcagttatttataaaagattacaggtacacttatcgatgccaccgaggtgacacacaaatacaaacccggagatcatttgagataaagaaactatgggaccaaacccgctcaaaagctgggctgttggtctctgatggaggccccaatttgtataacattagaaatctccatattcctgaagtttgcttgaaatgggagttgatggatgaggattaccaggggcgtttatgcaacccattaaacccgtttgtcagccataaagagattgaatcagtgaacaatgcagtgataatgccggcacatggtccagccaaaaatatggagtatgacgctgttgcaacaacacactcctgggtccccaaaagaaatcgatccattttgaacacgagccaaagggggatacttgaagatgagcaaatgtatcagaggtgctgcaatttgtttgaaaaattcttcccaagtagctcatacagaagaccagttggaatatccagtatggtagaggctatggcctcaagagcccgaattgatgcacggattgatttcgaatctggaaggataaagaaagaggaatttgctgagatcatgaagatctgttccaccattgaagacctcagacggcaaaaatga

>H1N1_PB1_CY105164_A/HoChiMinh/HCM395/2006

atggatgtcaatccgacattacttttcttaaaagtgccagcacaaaatgctataagcacaacttttccttatactggcgaccctccttacagccatgggacaggaacaggatacaccatggatacagtcaacaggacacatcagtactcagaaagaggaagatggacaaaaaataccgaaactggagcacctcaactcaacccaattgatgggccactaccagaagacaatgaaccaagtggctatgcccaaacagattgtgtattagaagcaatggctttccttgaagagtcccatcctggtatttttgaaaactcttgtattgaaacaatggaggttgttcagcaaacaagggtggacaaactgacacaaggcagacagacctatgactggactttaaataggaaccagcctgctgccacagctttggcaaacactatagaagtattcagatcaaacggcctcatagcaaatgaatctgggaggctaatagacttccttaaagatgtaatggagtcgatggacagagacgaagtagaggtcacaactcattttcaaagaaagaggagagtgagagacaatgtaactaaaaaaatggtgacccaaagaacaataggcaaaaagaaacataaattagacaaaagaagttacctaattagggcattaaccctgaacacaatgaccaaagatgctgagagggggaaactaaaacgcagagcaattgcaaccccaggaatgcaaataagggggttcgtatactttgttgagacactggcaagaagcatatgtgaaaagcttgaacaatcagggttgccagttggaggaaatgagaagaaagcaaagttagcaaatgttgtaagaaagatgatgaccaactcccaggacactgaaatttctttcaccatcactggagataacacaaaatggaacgaaaatcaaaaccctagaatgttcttggccatgatcacatatataaccaaaaatcagcctgaatggttcagaaacattctaagtattgctccaataatgttttcaaacaagatggcgagactaggtaaggggtacatgtttgaaagcaagagtatgaaactgagaactcaaatacctgcagagatgctagccaacatagatttgaaatatttcaatgattcaactaaaaagaaaattgaaaaaatccgaccattattaatagatggaactgcatcattgagtcctggaatgatgatgggcatgttcaatatgttaagcaccgtcttgggcgtctccattctgaatcttgggcaaaagagatacaccaagactacttactggtgggatggtcttcaatcgtctgatgattttgctttgattgtgaatgcacccaactatgcaggaattcaagctggagttgacaggttttatcgaacctgtaagctgctcggaattaatatgagcaaaaagaaatcttacataaacagaacaggtacctttgaattcacgagctttttctatcgttatgggtttgttgccaatttcagcatggagcttcctagttttggggtgtctggggttaatgaatctgcagacatgagtattggagtcactgttatcaaaaacaatatgataaacaatgaccttggcccagcaactgctcaaatggcccttcagttattcataaaagattacaggtacacttatcgatgccacagaggtgacacacaaatacaaacccggagatcatttgagataaagaaactatgggaccaaacccgctccaaagctgggctgttggtctctgatggaggccccaatttatataacattagaaatctccatattcctgaagtctgcttgaagtgggagttgatggatgaggattaccaggggcgtttatgcaacccattgaacccgtttgtcagccataaagagattgaatcagtgaacaatgcagtgataatgccggcacatggtccagccaaaaatatggagtatgacgctgttgcaacaacacactcctgggtccccaaaagaaaccgatccattttgaacacgagccaaagggggatacttgaagatgagcaaatgtatcagaggtgctgcaatttatttgaaaaattcttcccaagtagctcatacagaagaccagttggaatatccagtatggtagaggctatggtttcaagagcccgaattgatgcacggattgatttcgaatctggaaggataaagaaagaggaattcgctgagatcatgaagacctgttccaccattgaagacctcagacggcaaaaatag

>H1N1_PB1_JQ409124_A/Helsinki/Vi1/2009

atggatgtcaatccgactctacttttcctaaaaattccagcgcaaaatgccataagcaccacattcccttatactggagatcctccatacagccatggaacaggaacaggatacaccatggacacagtaaacagaacacaccaatactcagaaaagggaaagtggacgacaaacacagagactggtgcaccccagctcaacccgattgatggaccactacctgaggataatgaaccaagtgggtatgcacaaacagactgtgttctagaggctatggctttccttgaagaatcccacccaggaatatttgagaattcatgccttgaaacaatggaagttgttcaacaaacaagggtagataaactaactcaaggtcgccagacttatgattggacattaaacagaaatcaaccggcagcaactgcattggccaacaccatagaagtctttagatcgaatggcctaacagctaatgagtcaggaaggctaatagatttcttaaaggatgtaatggaatcaatgaacaaagaggaaatagagataacaacccactttcaaagaaaaaggagagtaagagacaacatgaccaagaagatggtcacgcaaagaacaatagggaagaaaaaacaaagactgaataagagaggctatctaataagagcactgacattaaatacgatgaccaaagatgcagagagaggcaagttaaaaagaagggctatcgcaacacctgggatgcagattagaggtttcgtatactttgttgaaactttagctaggagcatttgcgaaaagcttgaacagtctgggctcccagtagggggcaatgaaaagaaggccaaactggcaaatgttgtgagaaagatgatgactaattcacaagacacagagatttctttcacaatcactggggacaacactaagtggaatgaaaatcaaaatcctcgaatgttcctggcgatgattacatatatcaccagaaatcaacccgagtggttcagaaacatcctgagcatggcacccataatgttctcaaacaaaatggcaagactagggaaagggtacatgttcgagagtaaaagaatgaagattcgaacacaaataccagcagaaatgctagcaagcattgacctgaagtacttcaatgaatcaacaaagaagaaaattgagaaaataaggcctcttctaatagatggcacagcatcactgagtcctgggatgatgatgggcatgttcaacatgctaagtacggtcttgggagtctcgatactgaatcttggacaaaagaaatacaccaagacaatatactggtgggatgggctccaatcatccgacgattttgctctcatagtgaatgcaccaaaccatgagggaatacaagcaggagtggacagattctacaggacctgcaagttagtgggaatcaacatgagcaaaaagaagtcctatataaataagacagggacatttgaattcacaagctttttttatcgctatggatttgtggctaattttagcatggagctacccagctttggagtgtctggagtaaatgaatcagctgacatgagtattggagtaacagtgataaagaacaacatgataaacaatgaccttggacctgcaacggcccagatggctcttcaattgttcatcaaagactacagatacacatataggtgccatargggagacacacaaattcagacgagaagatcatttgagttaaagaagctgtgggatcaaacccaatcaaaggtagggctattagtatcagatggaggaccaaacttatacaatatacggaatcttcacattcctgaagtctgcttaaaatgggagctaatggatgatgattatcggggaagactttgtaatcccctgaatccctttgtcagtcataaagagattgattctgtaaacaatgctgtggtaatgccagcccatggtccagccaaaagcatggaatatgatgccgttgcaactacacattcctggattcccaagaggaatcgttctattctcaacacaagccaaaggggaattcttgaggatgaacagatgtaccagaagtgctgcaatctattcgagaaatttttccctagcagttcatataggagaccggttggaatttctagcatggtggaggccatggtgtctagggcccggattgatgccagggtcgacttcgagtctggacggatcaagaaagaagagttctctgagatcatgaagatctgttccaccattgaagaactcagacggcaaaaataa

>H1N1_PB1_CY047793_A/Argentina/7937/2009

atggatgtcaatccgactctacttttcctaaaaattccagcgcaaaatgccataagcaccacattcccttatactggagatcctccatacagccatggaacaggaacaggatacaccatggacacagtaaacagaacacaccaatactcagaaaagggaaagtggacgacaaacacagagactggtgcaccccagctcaacccgattgatggaccactacctgaggataatgaaccaagtgggtatgcacaaacagactgtgttctagaggctatggctttccttgaagaatcccatccaggaatatttgagaattcatgccttgaaacaatggaagttgttcaacaaacaagggtagataaactaactcaaggtcgccagacttatgattggacattaaacagaaatcaaccggcagcaactgcattggccaacaccatagaagtctttagatcgaatggcctaacagctaatgagtcaggaaggctaatagatttcttaaaggatgtaatggaatcaatgaacaaagaggaaatagagataacaacccactttcaaagaaaaaggagagtaagagacaacatgaccaagaagatggtcacgcaaagaacaatagggaagaaaaaacaaagactgaataagagaggctatctaataagagcactgacattaaatacgatgaccaaagatgcagagagaggcaagttaaaaagaagggctatcgcaacacctgggatgcagattagaggtttcgtatactttgttgaaactttagctaggagcatttgcgaaaagcttgaacagtctgggctcccagtagggggcaatgaaaagaaggccaaactggcaaatgttgtgagaaagatgatgactaattcacaagacacagagatttctttcacaatcactggggacaacactaagtggaatgaaaatcaaaatcctcgaatgttcctggcgatgattacatatatcaccagaaatcaacccgagtggttcagaaacatcctgagcatggcacccataatgttctcaaacaaaatggcaagactagggaaagggtacatgttcgagagtaaaagaatgaagattcgaacacaaataccagcagaaatgctagcaagcattgacctgaagtacttcaatgaatcaacaaagaagaaaattgagaaaataaggcctcttctaatagatggcacagcatcactgagtcctgggatgatgatgggcatgttcaacatgctaagtacggtcttgggagtctcgatactgaatcttggacaaaagaaatacaccaagacaatatactggtgggatgggctccaatcatccgacgattttgctctcatagtgaatgcaccaaaccatgagggaatacaagcaggagtggacagattctacaggacctgcaagttagtgggaatcaacatgagcaaaaagaagtcctatataaataagacagggacatttgaattcacaagctttttttatcgctatggatttgtggctaattttagcatggagctacccagctttggagtgtctggagtaaatgaatcagctgacatgagtattggagtaacagtgataaagaacaacatgataaacaatgaccttggacctgcaacggcccagatggctcttcaattgttcatcaaagactacagatacacatataggtgccataggggagacacacaaattcagacgagaagatcatttgagttaaagaagctgtgggatcaaacccaatcaaaggtagggctattagtatcagatggaggaccaaacttatacaatatacggaatcttcacattcctgaagtctgcttaaaatgggagctaatggatgatgattatcggggaagactttgtaatcccctgaatccctttgtcagtcataaagagattgattctgtaaacaatgctgtggtaatgccagcccatggtccagccaaaagcatggaatatgatgccgttgcaactacacattcctggattcccaagaggaatcgttctattctcaacacaagccaaaggggaattcttgaggatgaacagatgtaccagaagtgctgcaatctattcgagaaatttttccctagcagttcatataggagaccggttggaatttctagcatggtggaggccatggtgtctagggcccggattgatgccagggtcgacttcgaatctggacggatcaagaaagaagagttctctgagatcatgaagatctgttccaccattgaagaactcagacggcaaaaataa

>H1N1_PB1_CY047921_A/Argentina/9579/2009

atggatgtcaatccgactctacttttcctaaaaattccagcgcaaaatgccataagcaccacattcccttatactggagatcctccatacagccatggaacaggaacaggatacaccatggacacagtaaacagaacacaccaatactcagaaaagggaaagtggacgacaaacacagagactggtgcaccccagctcaacccgattgatggaccactacctgaggataatgaaccaagtgggtatgcacaaacagactgtgttctagaggctatggctttccttgaagaatcccacccaggaatatttgagaattcatgccttgaaacaatggaagttgttcaacaaacaagggtagataaactaactcaaggtcgccagacttatgattggacattaaacagaaatcaaccggcagcaactgcattggccaacaccatagaagtctttagatcgaatggcctaacagctaatgagtcaggaaggctaatagatttcttaaaggatgtaatggaatcaatgaacaaagaggaaatagagataacaacccactttcaaagaaaaaggagagtaagagacaacatgaccaagaagatggtcacgcaaagaacaatagggaagaaaaaacaaagactgaataagagaggctatctaataagagcactgacattaaatacgatgaccaaagatgcagagagaggcaagttaaaaagaagggctatcgcaacacctgggatgcagattagaggtttcgtatactttgttgaaactttagctaggagcatttgcgaaaagcttgaacagtctgggctcccagtagggggcaatgaaaagaaggccaaactggcaaatgttgtgagaaagatgatgactaattcacaagacacagagatttctttcacaatcactggggacaacactaagtggaatgaaaatcaaaatcctcgaatgttcctggcgatgattacatatatcaccagaaatcaacccgagtggttcagaaacatcctgagcatggcacccataatgttctcaaacaaaatggcaagactagggaaagggtacatgttcgagagtaaaagaatgaagattcgaacacaaataccagcagaaatgctagcaagcattgacctgaagtacttcaatgaatcaacaaagaagaaaattgagaaaataaggcctcttctaatagatggcacagcatcactgagtcctgggatgatgatgggcatgttcaacatgctaagtacggtcttgggagtctcgatactgaatcttggacaaaagaaatacaccaagacaatatactggtgggatgggctccaatcatccgacgattttgctctcatagtgaatgcaccaaaccatgagggaatacaagcaggagtggacagattctataggacctgcaagttagtgggaatcaacatgagcaaaaagaagtcctatataaataagacagggacatttgaattcacaagctttttttatcgctatggatttgtggctaattttagcatggagctacccagctttggagtgtctggagtaaatgaatcagctgacatgagtattggagtaacagtgataaagaacaacatgataaacaatgaccttggacctgcaacggcccagatggctcttcaattgttcatcaaagactacagatacacatataggtgccataggggagacacacaaattcagacgagaagatcatttgagttaaagaagctgtgggatcaaacccaatcaaaggtagggctattagtatcagatggaggaccaaacttatacaatatacggaatcttcacattcctgaagtctgcttaaaatgggagctaatggatgatgattatcggggaagactttgtaatcccctgaatccctttgtcagtcataaagagattgattctgtaaacaatgctgtggtaatgccagcccatggtccagccaaaagcatggaatatgatgccgttgcaactacacattcctggattcccaagaggaatcgttctattctcaacacaagccaaaggggaattcttgaggatgaacagatgtaccagaagtgctgcaatctattcgagaaatttttccctagcagttcatataggagaccggttggaatttctagcatggtggaggccatggtgtctagggcccggattgatgccagggtcgacttcgagtctggacggatcaagaaagaagagttctctgagatcatgaagatctgttccaccattgaagaactcagacggcaaaaataa

>H1N1_PB1_CY047953_A/Argentina/9705/2009

atggatgtcaatccgactctacttttcctaaaaattccagcgcaaaatgccataagcaccacattcccttatactggagatcctccatacagccatggaacaggaacaggatacaccatggacacagtaaacagaacacaccaatactcagaaaagggaaggtggacgacaaacacagagactggtgcaccccagctcaacccgattgatggaccactacctgaggataatgaaccaagtgggtatgcacaaacagactgtgttctagaggctatggctttccttgaagaatcccacccaggaatatttgagaattcatgccttgaaacaatggaagttgttcaacaaacaagggtagataaactaactcaaggtcgccagacttatgattggacattaaacagaaatcaaccggcagcaactgcattggccaacaccatagaagtctttagatcgaatggcctaacagctaatgagtcaggaaggctaatagatttcttaaaggatgtaatggaatcaatgaacaaagaggaaatagagataacaacccactttcaaagaaaaaggagagtaagagacaacatgaccaagaagatggtcacgcaaagaacaatagggaagaaaaaacaaagactgaataagagaggctatctaataagagcactgacattaaatacgatgaccaaagatgcagagagaggcaagttaaaaagaagggctatcgcaacacctgggatgcagattagaggtttcgtatactttgttgaaactttagctaggagcatttgcgaaaagcttgaacagtctgggctcccagtagggggcaatgaaaagaaggccaaactggcaaatgttgtgagaaagatgatgactaattcacaagacacagagatttctttcacaatcactggggacaacactaagtggaatgaaaatcaaaatcctcgaatgttcctggcgatgattacatatatcaccagaaatcaacccgagtggttcagaaacatcctgagcatggcacccataatgttctcaaacaaaatggcaagactagggaaagggtacatgttcgagagtaaaagaatgaagattcgaacacaaataccagcagaaatgctagcaagcattgacctgaagtacttcaatgaatcaacaaagaagaaaattgagaaaataaggcctcttctaatagatggcacagcatcactgagtcctgggatgatgatgggcatgttcaacatgctaagtacggtcttgggagtctcgatactgaatcttggacaaaagaaatacaccaagacaatatactggtgggatgggctccaatcatccgacgattttgctctcatagtgaatgcaccaaaccatgagggaatacaagcaggagtggacagattctacaggacctgcaagttagtgggaatcaacatgagcaaaaagaagtcctatataaataagacagggacatttgaattcacaagctttttttatcgctatggatttgtggctaattttagcatggagctacccagctttggagtgtctggagtaaatgaatcagctgacatgagtattggagtaacagtgataaagaacaacatgataaacaatgaccttggacctgcaacggcccagatggctcttcaattgttcatcaaagactacagatacacatataggtgccataagggagacacacaaattcagacgagaagatcatttgagttaaagaagctgtgggatcaaacccaatcaaaggtagggctattagtatcagatggaggaccaaacttatacaatatacggaatcttcacattcctgaagtctgcttaaaatgggagctaatggatgatgattatcggggaagactttgtaatcccctgaatccctttgtcagtcataaagagattgattctgtaaacaatgctgtggtaatgccagcccatggtccagccaaaagcatggaatatgatgccgttgcaactacacattcctggattcccaagaggaatcgttctattctcaacacaagccaaaggggaattcttgaggatgaacagatgtaccagaagtgctgcaatctattcgagaaatttttccctagcagttcatataggagaccggttggaatttctagcatggtggaggccatggtgtctagggcccggattgatgccagggtcgacttcgagtctggacggatcaagaaagaagagttctctgagatcatgaagatctgttccaccattgaagaactcagacggcaaaaataa

>H1N1_PB1_JQ431376_A/Reunion/0148-6-M1E/2009

atggatgtcaatccgactctacttttcctaaaaattccagcgcaaaatgccataagcaccacattcccttatactggagatcctccatacagccatggaacaggaacaggatacaccatggacacagtaaacagaacacaccaatactcagaaaagggaaagtggacgacaaacacagagactggtgcaccccagctcaacccgattgatggaccactacctgaggataatgaaccaagtgggtatgcacaaacagactgtgttctggaggctatggctttccttgaagaatcccacccaggaatatttgagaattcatgccttgaaacaatggaagttgttcaacaaacaagggtagataaactaactcaaggtcgccagacttatgattggacattaaacagaaatcaaccggcagcaactgcattggccaacaccatagaagtctttagatcgaatggcctaacagctaatgagtcaggaaggctaatagatttcttaaaggatgtaatggaatcaatgaacaaagaggaaatagagataacaacccactttcaaagaaaaaggagagtaagagacaacatgaccaagaagatggtcacgcaaagaacaatagggaagaaaaaacaaagactgaataagagaggctatctaataagagcactgacattaaatacgatgaccaaagatgcagagagaggcaagttaaaaagaagggctatcgcaacacctgggatgcagattagaggtttcgtatactttgttgaaactttagctaggagcatttgcgaaaagcttgaacagtctgggctcccagtagggggcaatgaaaagaaggccaaactggcaaatgttgtgagaaagatgatgactaattcacaagacacagagatttctttcacaatcactggggacaacactaagtggaatgaaaatcaaaatcctcgaatgttcctggcgatgattacatatatcaccagaaatcaacccgagtggttcagaaacatcctgagcatggcacccataatgttctcaaacaaaatggcaagactagggaaagggtacatgttcgagagtaaaagaatgaagattcgaacacaaataccagcagaaatgctagcaagcattgacctgaagtacttcaatgaatcaacaaagaagaaaattgagaaaataaggcctcttctaatagatggcacagcatcactgagtcctgggatgatgatgggcatgttcaacatgctaagtacggtcttgggagtctcgatactgaatcttggacaaaagaaatacaccaagacaatatactggtgggatgggctccaatcatccgacgattttgctctcatagtgaatgcaccaaaccatgagggaatacaagcaggagtggacagattctacaggacctgcaagttagtgggaatcaacatgagcaaaaagaagtcctatataaataagacagggacatttgaattcacaagctttttttatcgctatggatttgtggctaattttagcatggagctacccagctttggagtgtctggagtaaatgaatcagctgacatgagtattggagtaacagtgataaagaacaacatgataaacaatgaccttggacctgcaacggcccagatggctcttcaattgttcatcaaagactacagatacacatataggtgccataggggagacacacaaattcagacgagaagatcatttgagttaaagaagctgtgggatcaaacccaatcaaaggtagggctattagtatcagatggaggaccaaacttatacaatatacggaatcttcacattcctgaagtctgcttaaaatgggagctaatggatgatgattatcggggaagactttgtaatcccctgaatccctttgtcagtcataaagagattgattctgtaaacaatgctgtggtaatgccagcccatggtccagccaaaagcatggaatatgatgccgttgcaactacacattcctggattcccaagaggaatcgttctattctcaacacaagccaaaggggaattcttgaggatgaacagatgtaccagaagtgctgcaatctattcgagaaatttttccctagcagttcatataggagaccggttggaatttctagcatggtggaggccatggtgtctagggcccggattgatgccagggtcgacttcgagtctggacggatcaagaaagaagagttctctgagatcatgaagatctgttccaccattgaagaactcagacggcaaaaataa

>H1N1_PB1_HQ263272_A/Bethesda/NIH106-D0/2009

atggatgtcaatccgactctacttttcctaaaaattccagcgcaaaatgccataagcaccacattcccttatactggagatcctccatacagccatggaacaggaacaggatacaccatggacacagtaaacagaacacaccaatactcagaaaagggaaagtggacgacaaacacagagactggtgcaccccagctcaacccgattgatggaccactacctgaggataatgaaccaagtgggtatgcacaaacagactgtgttctagaggctatggctttccttgaagaatcccacccaggaatatttgagaattcatgccttgaaacaatggaagttgttcaacaaacaagggtagataaactaactcaaggtcgccagacttatgattggacattaaacagaaatcaaccggcagcaactgcattggccaacaccatagaagtctttagatcgaatggcctaacagctaatgagtcaggaaggctaatagatttcttaaaggatgtaatggaatcaatgaacaaagaggaaatagagataacaacccactttcaaagaaaaaggagagtaagagacaacatgaccaagaagatggtcacgcaaagaacaatagggaagaaaaaacaaagactgaataagagaggctatctaataagagcactgacattaaatacgatgaccaaagatgcagagagaggcaagttaaaaagaagagctatcgcaacacctgggatgcagattagaggtttcgtatactttgttgaaactttagctaggagcatttgcgaaaagcttgaacagtctgggctcccagtagggggcaatgaaaagaaggccaaactggcaaatgttgtgagaaagatgatgactaattcacaagacacagagatttctttcacaatcactggggacaacactaagtggaatgaaaatcaaaatcctcgaatgttcctggcgatgattacatatatcaccagaaatcaacccgagtggttcagaaacatcctgagcatggcacccataatgttctcaaacaaaatggcaagactagggaaagggtacatgttcgagagtaaaagaatgaagattcgaacacaaataccagcagaaatgctagcaagcattgacctgaagtacttcaatgaatcaacaaagaagaaaattgagaaaataaggcctcttctaatagatggcacagcatcactgagtcctgggatgatgatgggcatgttcaacatgctaagtacggtcttgggagtctcgatactgaatcttggacaaaagaaatacaccaagacaatatactggtgggatgggctccaatcatccgacgattttgctctcatagtgaatgcaccaaaccatgagggaatacaagcaggagtggacagattctacaggacctgcaagttagtgggaatcaacatgagcaaaaagaagtcctatataaataagacagggacatttgaattcacaagctttttttatcgctatggatttgtggctaattttagcatggagctacccagctttggagtgtctggagtaaatgaatcagctgacatgagtattggagtaacagtgataaagaacaacatgataaacaatgaccttggacctgcaacggcccagatggctcttcaactgttcatcaaagactacagatacacatataggtgccataggggagacacacaaattcagacgagaagatcatttgagttaaagaagctgtgggatcaaacccaatcaaaggtagggctattagtatcagatggaggaccaaacttatacaatatacggaatcttcacattcctgaagtctgcttaaaatgggagctaatggatgatgattatcggggaagactttgtaatcccctgaatccctttgtcagtcataaagagattgattctgtaaacaatgctgtggtaatgccagcccatggtccagccaaaagcatggaatatgatgccgttgcaactacacattcctggattcccaagaggaatcgttctattctcaacacaagccaaaggggaattcttgaggatgaacagatgtaccagaagtgctgcaatctattcgagaaatttttccctagcagttcatataggagaccggttggaatttctagcatggtggaggccatggtgtctagggcccggattgatgccagggtcgacttcgagtctggacggatcgggaaggaagagttctctgagatcatgaagatctgctccaccattgaagaactcagacggcaaaaataa

>H1N1_PB1_CY119128_A/Malaysia/15042/1998

atggatgtcaatccgaccttacttttcttaaaagtgccagcacaaaatgctataagcacaacttttccttatactggagaccctccttacagccatgggacaggaacagggtacaccatggatacagtcaacaggacacatcagtactcagaaagaggaagatggacaaaaaataccgaaactggagcaccgcaactcaacccaattgatgggccactaccaaaagacaatgaaccaagtggctatgcccaaacagattgtgtattagaagcaatggctttccttgaggaatcccatcctggtatttttgaaaactcttgtattgaaacaatggaggttgttcagcaaacaagggtggacaaactgacacaaggcagacagacctatgactggactctaaatagaaaccagcctgctgccacagcattggccaacactatagaagtgttcagatcaaacggtctcatagcaaatgaatctgggaggctaatagacttccttaaagatgtaatggagtcgatgaacagagacgaggtagagatcacaactcattttcagagaaagaggagagtgagagacaatgtaactaaaaaaatggtgacccaaagaacaataggcaaaaagaaacataaattagacaaaagaagttacctaattagggcactaaccctgaacacaatgaccaaagatgctgagagggggaaactaaaacgtagagcaattgcaaccccaggaatgcaaataagggggtttgtatactttgttgagacactggcaaggagcatatgtgaaaagcttgaacaatcagggttgccagttggaggaaatgaaaagaaagcaaagttagcaaatgttgtaaggaagatgatgaccaactcccaggacactgaaatttctttcaccatcactggagataacacaaaatggaacgaaaatcaaaaccctagaatgttcttggccatgatcacatatataaccaaaaatcagcctgaatggttcagaaatattctaagtattgctccaataatgttttcaaacaaaatggcgagactaggtaaggggtacatgtttgaaagcaagagtatgaaactgagaactcaaatacctgcagagatgctagccaacatagatttgaaatatttcaatgattcaacaaaaaagaaaattgaaaaaatccggccattattaatagatggaactgcatcattgagtcctggaatgatgatgggcatgttcaatatgttaagcactgtcttgggcgtctccattctgaatcttgggcaaaagagatacaccaagactacttactggtgggatggtcttcaatcgtctgatgattttgctctgattgtgaatgcacccaactatgcaggaattcaggctggagttgacaggttttatcgaacctgtaagctgctcgggattaatatgagcaaaaagaagtcttacataaacagaacaggtacctttgaattcacgagctttttctatcgttatgggtttgttgccaatttcagcatggagcttcctagttttggggtgtctggggtcaatgagtctgcagacatgagtattggagtcactgtcatcaaaaacaatatgataaacaatgaccttggcccagcaactgctcaaatggcccttcagttatttatcaaagattacagatacacgtatcgatgccacagaggtgacacacaaatacaaacccggagatcatttgagataaagaaactatgggaccaaacccgctccaaagctgggctgttagtctctgatggaggccccaatttatataacattagaaatctccacattcctgaagtctgcttgaaatgggagttgatggatgaggattaccaggggcgtttatgcaacccattgaacccgtttgtcagtcataaagagattgaatcagtgaacaatgcagtgatgatgccggcacatggtccagccaaaaatatggagtatgacgctgttgcaacaacacactcctgggttcccaaaaggaatcgatccattttgaatacgagccaaagggggatacttgaggatgagcaaatgtatcagaggtgctgcagtttatttgaaaaattcttcccaagtagctcatacagaagaccagttggaatatccagtatggtagaggctatggtttccagagcccgaattgatgcacggattgatttcgaatctggaaggataaagaaagaggagttcgctgagatcatgaagacctgttctaccattgaagacctcagacggcaaaaatag

>H1N1_PB1_CY119328_A/Malaysia/2156486/2009

atggatgtcaatccgacattacttttcttaaaagtgccagcacaaaatgctataagcacaacttttccttatactggtgaccctccttacagccatgggacaggaacagggtacaccatggatacagtcaacaggacacaccagtactcagaaagaggaagatggacagaaaataccgaaacgggagcaccgcaacttaacccaattgatggtcccttaccggaagacaatgaaccaagtggctatgcccaaacagattgtgtattagaagcaatggctttccttgaagaatcccatcctggtatctttgaaaactcttgtattgaaacaatggaggttgttcaacaaacaagggtggacaaattgacacaaggcagacagacctatgactggactctaaataggaaccagcctgctgccacagcattggcaaacactatagaagtattcagatcaaacggcctcatagcaaatgaatctgggaggctaatagacttccttaaagatgtaatggagtcgatggacagaggcgaagtagaggtcacaacacattttcaaagaaagaggagagtgagagacaatgtaactaaaaaaatggtgacccaaagaacaataggcaaaaagaaacataaattagacaaaagaagttacctgattagggcattaaccctgaacacaatgaccaaagatgctgagagggggaaactaaaacgcagagcaattgcaaccccaggaatgcaaataagggggtttgtatactttgttgagacactggcaagaagcatatgtgaaaagcttgaacaatcaggattgccagttggaggaaatgagaagaaagcaaagttagcaaatgttgtaaggaagatgatgaccaactcccaggacactgaaatttctttcaccataaccggagataacacaaaatggaacgaaaatcaaaaccctagaatgttcttggccatgatcacatatataaccaaaaatcagcctgaatggttcagaaatattctaagtattgctccaataatgttttcaaacaagatggcgagactaggtaaggggtacatgtttgaaagcaagagtatgaaactgagaactcaaatacctgcagagatgctagccaacatagacttgaaatatttcaatgattcaactaaaaagaagattgaaaaaatccgaccattattaatagatggaactgcatcattgagtcctggaatgatgatgggcatgttcaatatgttgagcaccgtcttgggcgtctccattctgaatcttgggcaaaagagatacaccaagactacttactggtgggatggtcttcaatcgtctgatgattttgctttgattgtgaacgcacccaactatgcaggaattcaagctggagttgacaggttttatcgaacctgtaagctgctcggaattaatatgagcaaaaagaagtcttacataaacagaacaggtacctttgaattcacgagctttttctatcgttatgggtttgttgctaatttcagcatggagcttcctagttttggggtgtctggggtcaatgaatctgcagacatgagtattggagtcactgtcatcaaaaacaatatgataaacaatgaccttggcccagcaactgctcaaatggcccttcagttatttataaaagattacaggtacacttatcgatgccaccgaggtgacacacaaatacaaacccggagatcatttgagataaagaaactatgggaccaaacccgctcaaaagctgggctgttggtctctgatggaggccccaatttgtataacattagaaatctccatattcctgaagtttgcttgaaatgggagttgatggatgaggattaccaggggcgtttatgcaacccattaaacccgtttgtcagccataaagagattgaatcagtgaacaatgcagtgataatgccggcacatggtccagccaaaaatatggagtatgacgctgttgcaacaacacactcctgggtccccaaaagaaatcgatccattttgaacacgagccaaagggggatacttgaagatgagcaaatgtatcagaggtgctgcaatttatttgaaaaattcttcccaagtagctcatacagaagaccagttggaatatccagtatggtagaggctatggtctcaagagcccgaattgatgcacggattgatttcgaatctggaaggataaagaaagaggaatttgctgagatcataaagatctgttccaccattgaagacctcagacggcaaaaatga

>H1N1_PB1_CY119416_A/Malaysia/2089841/2009

atggatgtcaatccgactctacttttcctaaaaattccagcgcaaaatgccataagcaccacattcccttatactggagatcctccatacagccatggaacaggaacaggatacaccatggacacagtaaacagaacacaccaatactcagaaaagggaaagtggacgacgaacacagagactggtgcaccccagctcaacccgattgatggaccactacctgaggataatgaaccaagtgggtatgcacaaacagactgtgttctagaggctatggctttccttgaagaatcccacccaggaatatttgagaattcatgccttgaaacaatggaagttgttcaacaaacaagggtagataaactaactcaaggtcgccagacttatgattggacattaaacagaaatcaaccggcagcaactgcattggccaacaccatagaagtctttagatcgaatggcctaacagctaatgagtcaggaaggctaatagatttcttaaaggatgtaatggaatcaatgaacaaagaggaaatagagataacaacccactttcaaagaaaaaggagagtaagagacaacatgaccaagaagatggtcacgcaaagaacaatagggaagaaaaaacaaagactgaataagagaggctatctaataagagcactgacattaaatacgatgaccaaagatgcagagagaggcaagttaaaaagaagggctatcgcaacacctgggatgcagattagaggtttcgtatactttgttgaaactttagctaggagcatttgcgaaaagcttgaacagtctgggctcccagtagggggcaatgaaaagaaggccaaactggcaaatgttgtgagaaagatgatgactaattcacaagacacagagatttctttcacaatcactggggacaacactaagtggaatgaaaatcaaaatcctcgaatgttcctggcgatgattacatatatcaccagaaatcaacccgagtggttcagaaacatcctgagcatggcacccataatgttctcaaacaaaatggcaagactagggaaagggtacatgttcgagagtaaaagaatgaagattcgaacacaaataccagcagaaatgctagcaagcattgacctgaagtacttcaatgaatcaacaaagaagaaaattgagaaaataaggcctcttctaatagatggcacagcatcactgagtcctgggatgatgatgggcatgttcaacatgctaagtacggtcttgggagtctcgatactgaatcttggacaaaagaaatacaccaagacaatatactggtgggatgggctccaatcatccgacgattttgctctcatagtgaatgcaccaagccatgagggaatacaagcaggagtggacagattctacaggacctgcaagttagtgggaatcaacatgagcaaaaagaagtcctatataaataagacagggacatttgaattcacaagctttttttatcgctatggatttgtggctaattttagcatggagctacccagctttggagtgtctggagtaaatgaatcagctgacatgagtattggagtaacagtgataaagaacaacatgataaacaatgaccttggacctgcaacggcccagatggctcttcaattgttcatcaaagactacagatacacatataggtgccataggggagacacacaaattcagacgagaagatcatttgagttaaagaagctgtgggatcaaacccaatcaaaggtagggctattagtatcagatggaggaccaaacttatacaatatacggaatcttcacattcctgaagtctgcttaaaatgggagctaatggatgatgattatcggggaagactttgtaatcccctgaatccctttgtcagtcataaagagattgattctgtaaacaatgctgtggtaatgccagcccatggtccagccaaaagcatggaatatgatgccgttgcaactacacattcctggattcccaagaggaatcgttctattctcaacacaagccaaaggggaattcttgaggatgaacagatgtaccagaagtgctgcaatctattcgagaaatttttccctagcagttcatataggagaccggttggaatttctagcatggtggaggccatggtgtctagggcccggattgatgccagggtcgacttcgagtctggacggatcaagaaagaagagttctctgagatcatgaagatctgttccaccattgaagaactcagacggcaaaaataa

>H1N1_PB1_CY119440_A/Malaysia/2098044/2009

atggatgtcaatccgactctacttttcctaaaaattccagcgcaaaatgccataagcaccacattcccttatactggagatcctccatacagccatggaacaggaacaggatacaccatggacacagtaaacagaacacaccaatactcagaaaagggaaagtggacgacaaacacagagactggtgcaccccagctcaacccgattgatggaccactacctgaggataatgaaccaagtgggtatgcacaaacagactgtgttctagaggctatggctttccttgaagaatcccacccaggaatatttgagaattcatgccttgaaacaatggaagttgttcaacaaacaagggtagataaactaactcaaggtcgccagacttatgattggacattaaacagaaatcaaccggcagcaactgcattggccaacaccatagaagtctttagatcgaatggcctaacagctaatgagtcaggaaggctaatagatttcttaaaggatgtaatggaatcaatgaacaaagaggaaatagagataacaacccactttcaaagaaaaaggagagtaagagacaacatgaccaagaagatggtcacgcaaagaacaatagggaagaaaaaacaaagactgaataagagaggctatctaataagagcactgacattaaatacgatgaccaaagatgcagagagaggcaagttaaaaagaagggctatcgcaacacctgggatgcagattagaggtttcgtatactttgttgaaactttagctaggagcatttgcgaaaagcttgaacagtctgggctcccagtagggggcaatgaaaagaaggccaaactggcaaatgttgtgagaaagatgatgactaattcacaagacacagagatttctttcacaatcactggggacaacactaagtggaatgaaaatcaaaatcctcgaatgttcctggcgatgattacatatatcaccagaaatcaacccgagtggttcagaaacatcctgagcatggcacccataatgttctcaaacaaaatggcaagactagggaaagggtacatgtttgagagtaaaagaatgaagattcgaacacaaataccagcagaaatgctagcaagcattgacctgaagtacttcaatgaatcaacaaagaagaaaattgagaaaataaggcctcttctaatagatggcacagcatcactgagtcctgggatgatgatgggcatgttcaacatgctaagtacggtcttgggagtctcgatactgaatcttggacaaaagaaatacaccaagacaatatactggtgggatgggctccaatcatccgacgattttgctctcatagtgaatgcaccaaaccatgagggaatacaagcaggagtggacagattctacaggacctgcaagttagtgggaatcaacatgagcaaaaagaagtcctatataaataagacagggacatttgaattcacaagctttttttatcgctatggatttgtggctaattttagcatggagctacccagctttggagtatctggagtaaatgaatcagctgacatgagtattggagtaacagtgataaagaacaacatgataaacaatgaccttggacctgcaacggcccagatggctcttcaattgttcatcaaagactacagatacacatataggtgccataggggagacacacaaattcagacgagaagatcatttgagttaaagaagctgtgggatcaaacccaatcaaaggtagggctattagtatcagatggaggaccaaacttatacaatatacggaatcttcacattcctgaagtctgcttaaaatgggagctaatggatgatgattatcggggaagactttgtaatcccctgaatccctttgtcagtcataaagagattgattctgtaaacaatgctgtggtaatgccagcccatggtccagccaaaagcatggaatatgatgccgttgcaactacacattcctggattcccaagaggaatcgttctattctcaacacaagccaaaggggaattcttgaggatgaacagatgtaccagaagtgctgcaatctattcgagaaatttttccctagcagttcatataggagaccggttggaatttctagcatggtggaggccatggtgtctagggcccggattgatgccagggtcgacttcgagtctggacggatcaagaaagaagagttctctgagatcatgaagatctgttccaccattgaagaactcagacggcaaaaataa

>H1N1_PB1_CY119456_A/Malaysia/2097717/2009

atggatgtcaatccgactctacttttcctaaaaattccagcgcaaaatgccataagcaccacattcccttatactggagatcctccatacagccatggaacaggaacaggatacaccatggacacagtaaacagaacacaccaatactcagaaaagggaaagtggacgacaaacacagagactggtgcaccccagctcaacccgattgatggaccactacctgaggataatgaaccaagtgggtatgcacaaacagactgtgttctagaggctatggctttccttgaagaatcccacccaggaatatttgagaattcatgccttgaaacaatggaagttgttcaacaaacaagggtagataaactaactcaaggtcgccagacttatgattggacattaaacagaaatcaaccggcagcaactgcattggccaacaccatagaagtctttagatcgaatggcctaacagctaatgagtcaggaaggctaatagatttcttaaaggatgtaatggaatcaatgaacaaagaggaaatagagataacaacccactttcaaagaaaaaggagagtaagagacaacatgaccaagaagatggtcacgcaaagaacaatagggaagaaaaaacaaagactgaataagagaggctatctaataagagcactgacattaaatacgatgaccaaagatgcagagagaggcaagttaaaaagaagggctatcgcaacacctgggatgcagattagaggtttcgtatactttgttgaaactttagctaggagcatttgcgaaaagcttgaacagtctgggctcccagtagggggcaatgaaaagaaggccaaactggcaaatgttgtgagaaagatgatgactaattcacaagacacagagatttctttcacaatcactggggacaacactaagtggaatgaaaatcaaaatcctcgaatgttcctggcgatgattacatatatcaccagaaatcaacccgagtggttcagaaacatcctgagcatggcacccataatgttctcaaacaaaatggcaagactagggaaagggtacatgttcgagagtaaaagaatgaagattcgaacacaaataccagcagaaatgctagcaagcattgacctgaagtacttcaatgaatcaacaaagaagaaaattgagaaaataaggcctcttctaatagatggcacagcatcactgagtcctgggatgatgatgggcatgttcaacatgctaagtacggtcttgggagtctcgatactgaatcttggacaaaagaaatacaccaagacaatatactggtgggatgggctccaatcatccgacgattttgctctcatagtgaatgcaccaaaccatgagggaatacaagcaggagtggacagattctacaggacctgcaagttagtgggaatcaacatgagcaaaaagaagtcctatataaataagacagggacatttgaattcacaagctttttttatcgctatggatttgtggctaattttagcatggagctacccagctttggagtgtctggagtaaatgaatcagctgacatgagtattggagtaacagtgataaagaacaacatgataaacaatgaccttggacctgcaacggcccagatggctcttcaattgttcatcaaagactacagatacacatataggtgccataggggagacacacaaattcagacgagaagatcatttgagttaaagaagctgtgggatcaaacccaatcaaaggtagggctattagtatcagatggaggaccaaacttatacaatatacggaatcttcacattcctgaagtctgcttaaaatgggagctaatggatgatgattatcggggaagactttgtaatcccctgaatccctttgtcagtcataaagagattgattctgtaaacaatgctgtggtaatgccagcccacggtccagccaaaagcatggaatatgatgccgttgcaactacacattcctggattcccaagaggaatcgttctattctcaacacaagccaaaggggaattcttgaggatgaacagatgtaccagaagtgctgcaatctattcgagaaatttttccctagcagttcatataggagaccggttggaatttctagcatggtggaggccatggtgtctagggcccggattgatgccagggtcgacttcgagtctggacggatcaagaaagaagagttctctgagatcatgaagatctgttccaccattgaagaactcagacggcaaaaataa

>H1N1_PB1_CY120098_A/Hong Kong/H090-779-V20/2009

atggatgtcaatccgactctacttttcctaaaaattccagcgcaaaatgccataagcaccacattcccttatactggagatcctccatacagccatggaacaggaacaggatacaccatggacacagtaaacagaacacaccaatactcagaaaagggaaagtggacgacaaacacagagactggtgcaccccagctcaacccgattgatggaccactacctgaggataatgaaccaagtgggtatgcacaaacagactgtgttctagaggctatggctttccttgaagaatcccacccaggaatatttgagaattcatgccttgaaacaatggaagttgttcaacaaacaagggtagataaactaactcaaggtcgccagacttatgattggacattaaacagaaatcaaccggcagcaactgcattggccaacaccatagaagtctttagatcgaatggcctaacagctaatgagtcaggaaggctgatagatttcttaaaggatgtaatggaatcaatgaacaaagaggaaatagagataacaacccactttcaaagaaaaaggagagtaagagacaacatgaccaagaagatggtcacacaaagaacaatagggaagaaaaaacaaagactgaataagagaggctatctaataagagcactgacattaaatacgatgaccaaagatgcagagagaggcaagttaaaaagaagggctatcgcaacacctgggatgcagattagaggtttcgtatactttgttgaaactttagctaggagcatttgcgaaaagcttgaacagtctgggctcccagtagggggcaatgaaaagaaggccaaactggcaaatgttgtgagaaagatgatgactaattcacaagacacagagatttctttcacaatcactggggacaacactaagtggaatgaaaatcaaaatcctcgaatgttcctggcgatgattacatatatcaccagaaatcaacccgagtggttcagaaacatcctgagcatggcacccataatgttctcaaacaaaatggcaagactagggaaagggtacatgttcgagagtaaaagaatgaagattcgaacacaaataccagcagaaatgctagcaagcattgacctgaagtacttcaatgaatcaacaaagaagaaaattgagaaaataaggcctcttctaatagatggcacagcatcactgagtcctgggatgatgatgggcatgttcaacatgctaagtacggtcttgggagtctcgatactgaatcttggacaaaagaaatacaccaagacaatatactggtgggatgggctccaatcatccgacgattttgctctcatagtgaatgcaccaaaccatgagggaatacaagcaggagtggacagattctacaggacctgcaagttagtgggaatcaacatgagcaaaaagaagtcctatataaataagacagggacatttgaattcacaagctttttttatcgctatggatttgtggctaattttagcatggagctacccagctttggagtgtctggagtaaatgaatcagctgacatgagtattggagtaacagtgataaagaacaacatgataaacaatgaccttggacctgcaacggcccagatggctcttcaattgttcatcaaagactacagatacacatataggtgccataggggagacacacaaattcagacgagaagatcatttgagttaaagaagctgtgggatcaaacccaatcaaaggtagggctattagtatcagatggaggaccaaacttatacaatatacggaatcttcacattcctgaagtctgcttaaaatgggagctaatggatgatgattatcggggaagactttgtaatcccctgaatccctttgtcagtcataaagagattgattctgtaaacaatgctgtggtaatgccagcccatggtccagccaaaagcatggaatatgatgccgttgcaactacacattcctggattcccaagaggaatcgttctattctcaacacaagccaaaggggaattcttgaggatgaacagatgtaccagaagtgttgcactctattcgagaaatttttccctagcagttcatataggagaccggttggaatttctagcatggtggaggccatggtgtctagggcccggattgatgccagggtcgacttcgagtctggacggatcaagaaagaagagttctctgagatcatgaagatctgttccaccattgaagaactcagacggcaaaaataa

>H1N1_PB1_CY123065_A/Singapore/GP3175/2009

atggatgtcaatccgactctacttttcctaaaaattccagcgcaaaatgccataagcaccacattcccttatactggagatcctccatacagccatggaacaggaacaggatacaccatggacacagtaaacagaacacaccaatactcagaaaagggaaaatggacgacaaacacagagactggtgcaccccagctcaacccgattgatggaccactacctgaggataatgaaccaagtgggtatgcacaaacagactgtgttctagaggctatggctttccttgaagaatcccacccaggaatatttgagaattcatgccttgaaacaatggaagttgttcaacaaacaagggtagataaactaactcaaggtcgccagacttatgattggacattaaacagaaatcaaccggcagcaactgcattggccaacaccatagaagtctttagatcgaatggcctaacagctaatgagtcaggaaggctaatagatttcttaaaggatgtaatggaatcaatgaacaaagaggaaatagagataacaacccactttcaaagaaaaaggagagtaagagacaacatgaccaagaagatggtcacgcaaagaacaatagggaagaaaaaacaaagactgaataagagaggctatctaataagagcactgacattaaatacgatgaccaaagatgcagagagaggcaagttaaaaagaagggctatcgcaacacctgggatgcagattagaggtttcgtatactttgttgaaactttagctaggagcatttgcgaaaagcttgaacagtctgggctcccagtagggggcaatgaaaagaaggccaaactggcaaatgttgtgagaaagatgatgactaattcacaagacacagagatttctttcacaatcactggggacaacactaagtggaatgaaaatcaaaatcctcgaatgttcctggcgatgattacatatatcaccagaaatcaacccgagtggttcagaaacatcctgagcatggcacccataatgttctcaaacaaaatggcaagactagggaaagggtacatgttcgagagtaaaagaatgaagattcgaacacaaataccagcagaaatgctagcaagcattgacctgaagtacttcaatgaatcaacaaagaagaaaattgagaaaataaggcctcttctaatagatggcacagcatcactgagtcctgggatgatgatgggcatgttcaacatgctaagtacggtcttgggagtctcgatactgaatctcggacaaaagaaatacaccaagacaatatactggtgggatgggctccaatcatccgacgattttgctctcatagtgaatgcaccaaaccatgagggaatacaagcaggagtggacagattctacaggacctgcaagttagtgggaatcaacatgagcaaaaagaagtcctatataaataagacagggacatttgaattcacaagctttttttatcgctatggatttgtggctaattttagcatggagctacccagctttggagtgtctggagtaaatgaatcagctgacatgagtattggagtaacagtgataaagaacaacatgataaacaatgaccttggacctgcaacggcccagatggctcttcaattgttcatcaaagactacagatacacatataggtgccataggggagacacacaaattcagacgagaagatcatttgagttaaagaagctgtgggatcaaacccaatcaaaggtagggctattagtatcagatggaggaccaaacttatacaacatacggaatcttcacattcctgaagtctgcttaaaatgggagctaatggatgatgattatcggggaagactttgtaatcccctgaatccctttgtcagtcataaagagattgattctgtaaacaatgctgtggtaatgccagcccatggtccagccaaaagcatggaatatgatgccgttgcaactacacattcctggattcccaagaggaatcgttctattctcaacacaagccaaaggggaattcttgaggatgaacagatgtaccagaagtgctgcaatctattcgagaaatttttccctagcagttcatataggagaccggttggaatttctagcatggtggaggccatggtgtctagggcccggattgatgccagggtcgacttcgagtctggacggatcaagaaagaagagttctctgagatcatgaagatctgttccaccattgaagaactcagacggcaaaaataa

>H1N1_PB1_CY123073_A/Singapore/GP3178/2009

atggatgtcaatccgactctacttttcctaaaaattccagcgcaaaatgccataagcaccacattcccttatactggagaccctccatacagccatggaacaggaacaggatacaccatggacacagtaaacagaacacaccaatactcagaaaagggaaagtggacgacaaacacagagactggtgcaccccagctcaacccgattgatggaccactacctgaggataatgaaccaagtgggtatgcacaaacagactgtgttctagaggctatggctttccttgaagaatcccacccaggaatatttgagaattcatgccttgaaacaatggaagttgttcaacaaacaagggtagataaactaactcaaggtcgccagacttatgattggacattgaacagaaatcaaccggcagcaactgcattggccaacaccatagaagtctttagatcgaatggcctaacagctaatgagtcaggaaggctaatagatttcttaaaggatgtaatggaatcaatgaacaaagaggaaatagagataacaacccactttcaaagaaaaaggagagtaagagacaacatgaccaagaagatggtcacgcaaagaacaatagggaagaaaaaacaaagactgaataagagaggctatctaataagagcactgacattaaatacgatgaccaaagatgcagagagaggcaagttaaaaagaagggctatcgcaacacctgggatgcagattagaggtttcgtatactttgttgaaactttagctaggagcatttgcgaaaagcttgaacagtctgggctcccagtagggggcaatgaaaagaaggccaaactggcaaatgttgtgagaaagatgatgactaattcacaagacacagagatttctttcacaatcactggggacaacactaagtggaatgaaaatcaaaatcctcgaatgttcctggcgatgattacatatatcaccagaaatcaacccgagtggttcagaaacatcctgagcatggcacccataatgttctcaaacaaaatggcaagactagggaaagggtacatgttcgagagtaaaagaatgaagattcgaacacaaataccagcagaaatgctagcaagcattgacctgaagtacttcaatgaatcaacaaagaagaaaattgagaaaataaggcctcttctaatagatggcacagcatcactgagtcctgggatgatgatgggcatgttcaacatgctaagtacggtcttgggagtctcgatactgaatcttggacaaaagaaatacaccaagacaatatactggtgggatgggctccaatcatccgacgattttgctctcatagtgaatgcaccaaaccatgagggaatacaagcaggagtggacagattctacaggacctgcaagttagtgggaatcaacatgagcaaaaagaagtcctatataaataagacagggacatttgaattcacaagctttttttatcgctatggatttgtggctaattttagcatggagctacccagctttggagtgtctggagtaaatgaatcagctgacatgagtattggagtaacagtgataaagaacaacatgataaacaatgaccttggacctgcaacggcccagatggctcttcaattgttcatcaaagactacagatacacatataggtgccataggggagacacacaaattcagacgagaagatcatttgagttaaagaaactgtgggatcaaacccaatcaaaggtagggctattagtatcagatggaggaccaaacttatacaatatacggaatcttcacattcctgaagtctgcttaaaatgggagctaatggatgatgattatcggggaagactttgtaatcccctgaatccctttgtcagtcataaagagattgattctgtaaacaatgctgtggtaatgccagcccatggtccagccaaaagcatggaatatgatgccgttgcaactacacattcctggattcccaagaggaatcgttctattctcaacacaagccaaaggggaattcttgaggatgaacagatgtaccagaagtgctgcaatctattcgagaaatttttccctagcagttcatataggagaccggttggaatttctagcatggtggaggccatggtgtctagggcccggattgatgccagggtcgacttcgagtctggacggatcaagaaagaagagttctctgagatcatgaagatctgttccaccattgaagaactcagacggcaaaaataa

>H1N1_PB1_CY123715_A/Singapore/ON202/2009

atggatgtcaatccgactctacttttcctaaaaattccagcgcaaaatgccataagcaccacattcccttatactggagatcctccatacagccatggaacaggaacaggatacaccatggacacagtaaacagaacacaccaatactcagaaaagggaaagtggacgacaaacacagagactggtgcaccccagctcaacccgattgatggaccactacctgaggataatgaaccaagtgggtatgcacaaacagactgtgttctagaggctatggctttccttgaagaatcccacccaggaatatttgagaattcatgccttgaaacaatggaagttgttcaacaaacaagggtagataaactaactcaaggtcgccagacttatgattggacattaaacagaaatcaaccggcagcaactgcattggccaacaccatagaagtctttagatcgaatggcctaacagctaatgagtcaggaaggctaatagatttcttaaaggatgtaatggaatcaatgaacaaagaggaaatagagataacaacccactttcaaagaaaaaggagagtaagagacaacatgaccaagaagatggtcacgcaaagaacaatagggaagaaaaaacaaagactgaataagagaggctatctaataagagcactgacattaaatacgatgaccaaagatgcagagagaggcaagttaaaaagaagggctatcgcaacacctgggatgcagattagaggtttcgtatactttgttgaaactttagctaggagcatttgcgaaaagcttgaacagtctgggctcccagtagggggcaatgaaaagaaggccaaactggcaaatgttgtgagaaagatgatgactaattcacaagacacagagatttctttcacaatcactggggacaacactaagtggaatgaaaatcaaaatcctcgaatgttcctggcgatgattacatatatcaccagaaatcaacccgagtggttcagaaacatcctgagcatggcacccataatgttctcaaacaaaatggcaagactagggaaagggtacatgttcgagagtaaaagaatgaagattcgaacacaaataccagcagaaatgctagcaagcattgacctgaagtacttcaatgaatcaacaaagaagaaaattgagaaaataaggcctcttctaatagatggcacagcatcactgagtcctgggatgatgatgggcatgttcaacatgctaagtacggtcttgggagtctcgatactgaatcttggacaaaagaaatacaccaagacaatatactggtgggatgggctccaatcatccgacgattttgctctcatagtgaatgcaccaaaccatgagggaatacaagcaggagtggacagattctacaggacctgcaagttagtgggaatcaacatgagcaaaaagaagtcctatataaataagacagggacatttgaattcacaagctttttttatcgctatggatttgtggctaattttagcatggagctacccagctttggagtgtctggagtaaatgaatcagctgacatgagtattggagtaacagtgataaagaacaacatgataaacaatgaccttggacctgcaacggcccagatggctcttcaattgttcatcaaagactacagatacacatataggtgccataggggagacacacaaattcagacgagaagatcatttgagttaaagaagctgtgggatcaaacccaatcaaaggtagggctattagtatcagatggaggaccaaacttatacaatatacggaatcttcacatccctgaagtctgcttaaaatgggagctaatggatgatgattatcggggaagactttgtaatcccctgaatccctttgtcagtcataaagagattgattctgtaaacaatgctgtggtaatgccagcccatggtccagccaaaagcatggaatatgatgccgttgcaactacacattcctggattcccaagaggaatcgttctattctcaacacaagccaaaggggaattcttgaggatgaacagatgtaccagaagtgctgcaatctattcgagaaatttttccctagcagttcatataggagaccggttggaatttctagcatggtggaggccatggtgtctagggcccggattgatgccagggtcgacttcgagtctggacggatcaagaaagaagagttctctgagatcatgaagatctgttccaccattgaagaactcagacggcaaaaataa

>H1N1_PB1_CY124105_A/Singapore/ON809/2009

atggatgtcaatccgactctacttttcctaaaaattccagcgcaaaatgccataagcaccacattcccttatactggagatcctccatacagccatggaacaggaacaggatacaccatggacacagtaaacagaacacaccaatactcagaaaagggaaagtggacgacaaacacagagactggtgcaccccagctcaacccgattgatggaccactacctgaggataatgaaccaagtgggtatgcacaaacagactgtgttctagaggctatggctttccttgaagaatcccacccaggaatatttgagaattcatgccttgaaacaatggaagttgttcaacaaacaagggtagataaactaactcaaggtcgccagacttatgattggacattaaacagaaatcaaccggcagcaactgcattggccaacaccatagaagtctttagatcgaatggcctaacagctaatgagtcaggaaggctaatagatttcttaaaggatgtaatggaatcaatgaacaaagaggaaatagagataacaacccactttcaaagaaaaaggagagtaagagacaacatgaccaagaagatggtcacgcaaagaacaatagggaagaaaaaacaaagactgaataagagaggctatctaataagagcactgacattaaatacgatgaccaaagatgcagagagaggcaagttaaaaagaagggctatcgcaacacctgggatgcagattagaggtttcgtatactttgttgaaactttagctaggagcatttgcgaaaagcttgaacagtctgggctcccagtagggggcaatgaaaagaaggccaaactggcaaatgttgtgagaaagatgatgactaattcacaagacacagagatttctttcacaatcactggggacaacactaagtggaatgaaaatcaaaatcctcgaatgttcctggcgatgattacatatatcaccagaaatcaacccgagtggttcagaaacatcctgagcatggcacccataatgttctcaaacaaaatggcaagactagggaaagggtacatgttcgagagtaaaagaatgaagattcgaacacaaataccagcagaaatgctagcaagcattgacctgaagtacttcaatgaatcaacaaagaagaaaattgagaaaataaggcctcttctaatagatggcacagcatcactgagtcctgggatgatgatgggcatgttcaacatgctaagtacggtcttgggagtctcaatactgaatcttggacaaaagaaatacaccaagacaatatactggtgggatgggctccaatcatccgacgattttgctctcatagtgaatgcaccaaaccatgagggaatacaagcaggagtggacagattctacaggacctgcaagttagtgggaatcaacatgagcaaaaagaagtcctatataaataagacagggacatttgaattcacaagctttttttatcgctatggatttgtggctaattttagcatggagctacccagctttggagtgtctggagtaaatgaatcagctgacatgagtattggagtaacagtgataaagaacaacatgataaacaatgaccttggacctgcaacggcccagatggctcttcaattgttcatcaaagactacagatacacatataggtgccataggggagacacacaaattcagacgagaagatcatttgagttaaagaagctgtgggatcaaacccaatcaaaggtagggctattagtatcagatggaggaccaaacttatacaatatacggaatcttcacatccctgaagtctgcttaaaatgggagctaatggatgatgattatcggggaagactttgtaatcccctgaatccatttgtcagtcataaagagattgattctgtaaacaatgctgtggtaatgccagcccatggtccagccaaaagcatggaatatgatgccgttgcaactacacattcctggattcccaagaggaatcgttctattctcaacacaagccaaaggggaattcttgaggatgaacagatgtaccagaagtgctgcaatctattcgagaaatttttccctagcagttcatataggagaccggttggaatttctagcatggtggaggccatggtgtctagggcccggattgatgccagggtcgacttcgagtctggacggatcaagaaagaagagttctctgagatcatgaagatctgttccaccattgaagaactcagacggcaaaaataa

>H1N1_PB1_CY124542_A/Singapore/GP414/2011

atggatgtcaatccgactctacttttcctaaaaattccagcacaaaatgccataagcaccacattcccttatactggagatcctccatacagccatggaacaggaacaggatacaccatggacacagtaaacagaacacaccaatactcagaaaagggaaagtggacaacaaacacagagactggtgcaccccagctcaacccgattgatggaccactacctgaggataatgaaccaagtgggtatgcacaaacagactgtgttctagaggctatggctttccttgaagaatcccacccaggaatatttgagaattcatgccttgaaacaatggaagttgttcaacaaacaagggtagataaactgactcaaggtcgccagacttatgattggacattaaacagaaatcaaccggcagcaactgcattggccaacaccatagaagtctttagatcgaatgacctaacagctaatgagtcaggaaggctaatagatttcttaaaggatgtaatggaatcaatgaacaaagaggaaatagagataacaacccactttcaaagaaaaaggagagtaagagacaacatgaccaagaagatggtcacgcaaagaacaatagggaagaaaaaacagagactgaataagagaggctatttaataagagcactgacattaaatacgatgaccaaagatgcagagagaggcaagttaaaaagaagggctatcgcaacacctgggatgcagattagaggtttcgtatactttgttgaaactttagctaggagcatttgcgaaaagcttgaacagtctgggctcccagtagggggcaatgaaaagaaggccaaactggcaaatgttgtgagaaagatgatgactaattcacaagacacagagatttctttcacaatcactggggacaacactaagtggaatgaaaatcaaaatcctcgaatgttcctggcgatgattacatatatcaccagaaatcaacccgagtggtttagaaacatcctgagcatggcacccataatgttctcaaacaaaatggcaagactagggaaagggtacatgttcgagagtaaaagaatgaagattcgaacacaaataccagcagaaatgctagcaagcattgacctgaagtacttcaatgaatcaacaaagaagaaaattgagaaaataaggcctcttttaatggatggcacagcatcactgagtcctgggatgatgatgggcatgttcaacatgctaagtacggtcttgggagtctcgatactgaatcttggacaaaagaaatacaccaagacaacatactggtgggatgggctccaatcatccgacgattttgctctcatagtgaatgcaccaaaccatgagggaatacaagcaggagtgaacagattctacaggacctgcaagttagtgggaatcaacatgagcaaaaagaagtcctatataaataagacagggacatttgaattcacaagctttttttatcgctatggatttgtggctaattttagcatggagctacccagctttggagtgtctggagtaaatgaatcagctgacatgagtattggagtaacagtgataaagaacaacatgataaacaatgaccttggacctgcaacggcccagatggctcttcaattgttcatcaaagactacagatacacatataggtgccataggggagacacacaaattcagacgagaagatcatttgagttaaagaagctgtgggatcaaacccaatcaaaggtagggctattagtatcagatggaggaccaaacttatacaatatacggaatcttcacattcctgaagtctgcttaaaatgggagctaatggatgatgattatcggggaagactttgtaatcccctgaatccctttgtaagtcataaggagattgattctgtaaacaatgctgtggtaatgccagcccatggtccagccaaaagcatggaatatgatgccgttgcaactacacattcctggattcccaagaggaatcgttctattctcaacacaagccaaaggggaattcttgaggatgaacagatgtaccagaagtgctgcaatctattcgagaaatttttccctagcagttcatataggagaccggttggaatttctagcatggtggaggccatggtgtctagggcccggattgatgccagggtcgactttgagtctggacggatcaagaaagaagagttctctgagatcatgaagatctgttccaccattgaagaactcagacggcaaaaataa

>H1N1_PB1_CY124566_A/Singapore/GP496/2011

atggatgtcaatccgactctacttttcctaaaaattccagcgcaaaatgccataagcaccacattcccttatactggagatcctccatacagccatggaacaggaacaggatacaccatggacacagtaaacagaacacaccaatactcagaaaagggaaagtggacgacaaacacagagactggtgcaccccagctcaacccgattgatggaccactacctgaggataatgaaccaagtgggtatgcacaaacagactgtgttctagaggctatggctttccttgaagaatcccacccaggaatatttgagaattcatgccttgaaacaatggaagctgttcaacaaacaagggtagataaactaactcaaggtcgccagacttatgattggacattaaacagaaatcaaccggcagcaactgcattggccaacaccatagaagtctttagatcgaatggcctaacagctaatgagtcaggaaggctaatagatttcttaaaggatgtaatggaatcaatgaacaaagaggaaatagagataacaacccactttcaaagaaaaaggagagtaagagacaacatgaccaagaagatggtcacgcaaagaacaatagggaagaaaaaacaaagactgaataagagaggctatctaataagagcattgacattaaatacgatgaccaaagatgcagagagaggcaagttaaaaagaagggctatcgcaacacctgggatgcagattagaggtttcgtatactttgttgaaactttagctaggagcatttgcgaaaagcttgaacagtctgggctcccagtagggggcaatgaaaagaaggccaaactggcaaatgttgtgagaaagatgatgactaattcacaagacacagagatttctttcacaatcactggggacaacactaagtggaatgaaaatcaaaatcctcgaatgttcctggcgatgattacatatatcaccagaaatcaacccgagtggttcagaaacatcctgagcatggcacccataatgttctcaaacaaaatggcaagactagggaaagggtacatgttcgagagtaaaagaatgaagattcgaacacaaattccagcagaaatgctagcaagcattgacctgaaatacttcaatgaatcaacaaggaagaaaattgagaaaataaggcctcttctaatagatggcacagcatcactgagtcctgggatgatgatgggcatgttcaacatgctaagtacggtcttgggagtctcgatactgaatcttggacaaaagaaatacaccaagacaacatactggtgggatgggctccaatcatccgacgattttgctctcatagtgaatgcaccaaaccatgagggaatacaagcaggagtggacagattctacaggacctgcaagttagtgggaatcaacatgagcaaaaagaagtcatatataaataaaacagggacatttgaattcacaagctttttttatcgctatggatttgtggctaattttagcatggagctacccagctttggagtgtctggagtaaatgaatcagctgacatgagtattggagtaacagtgataaagaacaacatgataaacaatgaccttggacctgcaacggcccagatggctcttcaattgttcatcaaagactacagatacacatataggtgccataggggagacacacaaattcagacgagaagatcatttgagttaaaaaagctgtgggatcaaacccaatcaaaggtagggctattagtatcagatggaggaccaaacttatacaatatacggaatcttcacattcctgaagtctgcttaaaatgggagctaatggatgatgattaccggggaagactttgtaatcccctgaatccctttgtcagtcataaagagattgattctgtaaacaatgccgtggtaatgccagcccatggtccagccaaaagcatggaatatgatgccgttgcaactacacattcctggattcccaagaggaatcgttctattctcaacacaagccaaaggggaattcttgaggatgaacagatgtaccagaagtgctgcaatctattcgagaaatttttccctagcagttcatataggagaccggttggaatttctagcatggtggaggccatggtgtctagggcccggattgatgccagggtcgacttcgagtctggacggatcaagaaagaagagttctctgagatcatgaagatctgttccaccattgaagaactcagacggcaaaaataa

>H1N1_PB1_CY124676_A/Singapore/GP908/2011

atggatgtcaatccgactctacttttcctaaaaattccagcgcaaaatgccataagcaccacattcccttatactggagatcctccatacagccatggaacaggaacaggatacaccatggacacagtaaacagaacacaccaatactcagaaaagggaaaatggacgacaaacacagagactggtgcaccccagctcaacccgattgatggaccactacctgaggataatgaaccaagtgggtatgcacaaacagactgtgttctagaggctatggctttccttgaagaatcccacccaggaatatttgagaattcatgccttgaaacaatggaagttgttcaacaaacaagggtagataaactgactcaaggtcgccagacttatgattggacattaaatagaaatcaaccggcagcaactgcattggccaacaccatagaagtctttagatcgaatggcctaacagctaatgagtcaggaaggctaatagatttcctaaaggatgtaatggaatcaatgaacaaagaagaaatagagataacaacccactttcaaagaaaaaggagagtaagagacaacatgaccaagaagatggtcacgcaaaggacaatagggaagaaaaaacagagactgaataagagaggctatctaataagagcactgacattaaatacaatgaccaaagatgcagagagaggcaagttaaaaagaagggctatcgcaacacctgggatgcagattagaggtttcgtatactttgttgaaactttagctaggagcatttgcgaaaagcttgaacagtctgggctcccagtagggggcaatgaaaagaaggccaaactggcaaatgttgtgagaaagatgatgactaattcacaagacacagagatttctttcacaatcactggggacaacactaagtggaatgaaaatcaaaatcctcgaatgttcctggcgatgattacatatatcaccagaaatcaacccgagtggtttagaaacatcctgagcatggcacccataatgttctcaaacaaaatggcaagactagggaaagggtacatgttcgagagtaaaagaatgaagattcgaacacaaataccagcagaaatgctagcaagcattgacctgaagtacttcaatgaatcaacaaagaaaaaaattgagaaaataaggcctcttttaatggatggcacagcatcactgagtcctgggatgatgatgggcatgttcaacatgctaagtacagtcttgggagtctcgatactgaatcttggacaaaagaaatacaccaagacaacatactggtgggatgggctccaatcatccgacgattttgctctcattgtgaatgcaccaaaccatgagggaatacaagcaggagtggacagattctacaggacctgcaagttagtgggaatcaacatgagcaaaaagaagtcctatataaataagacagggacatttgaattcacaagctttttttatcgctatggatttgtggctaattttagcatggagctacccagctttggagtgtctggagtaaatgaatcagctgacatgagtattggagtaacagtgataaagaacaacatgataaacaatgaccttggacctgcaacggcccagatggctcttcaattgttcatcaaagactacagatacacatataggtgccataggggagacacacaaattcagacgagaagatcatttgagttaaagaagctgtgggatcaaacccaatcaaaggtagggctattagtatcagatggaggaccaaacttatacaatatacggaatcttcacattcctgaagtctgcttaaaatgggagctaatggatgatgattatcggggaagactttgtaatcccctgaatccctttgtaagtcataaggagattgattctgtaaacaatgctgtggtaatgccagcccatggtccagccaaaagcatggaatatgatgccgttgcaactacacattcctggattcccaagaggaatcgttctattctcaacacaagccaaaggggaattcttgaggatgaacagatgtaccagaagtgctgcaatctattcgagaaatttttccctagcagttcatataggagaccggttggaatttctagcatggtggaggccatggtgtctagggcccggattgatgccagggtcgactttgagtctggacggatcaagaaagaagagttctctgagatcatgaagatctgttccaccattgaagaactcagacggcaaaaataa

>H1N1_PB1_CY124692_A/Singapore/GP924/2011

atggatgtcaatccgactctacttttcctaaaaattccagcgcaaaatgccataagcaccacattcccttatactggagatcctccatacagccatggaacaggaacaggatacaccatggacacagtaaacagaacacaccaatactcagaaaagggaaagtggacgacaaacacagagactggtgcaccccagctcaacccgattgatggaccactacctgaggataatgaaccaagtgggtatgcacaaacagactgtgttctagaggctatggctttccttgaagaatcccacccaggaatatttgagaattcatgccttgaaacaatggaagttgttcaacaaacaagggtagataaactgactcaaggtcgccagacttatgattggacattaaacagaaatcaaccggcagcaactgcattggccaacaccatagaagtctttagatcgaatggcctaacagctaatgagtcaggaaggctaatagatttcttaaaggatgtaatggaatcaatgaacaaagaggagatagagataacaacccactttcaaagaaaaaggagagtaagagacaacatgaccaagaagatggtcacgcaaagaacaatagggaagaaaaaacagagactgaataagagagactatctaataagagcactgacattaaatacgatgaccaaagatgcagagagaggcaagttaaaaagaagggctatcgcaacacctgggatgcagattagaggtttcgtatactttgttgaaactttagctaggagcatttgcgaaaagcttgaacagtctgggctcccagtagggggcaatgaaaagaaggccaaactggcaaatgttgtgagaaagatgatgactaattcacaagacacagagatttctttcacaatcactggggacaacactaagtggaatgaaaatcaaaatcctagaatgttcctggcgatgattacatatatcaccagaaatcaacccgagtggtttagaaacatcctgagcatggcacccataatgttctcaaacaaaatggcaagactagggaaagggtacatgttcgagagtaaaagaatgaagattcgaacacaaataccagcagaaatgctagcaagcattgacctgaagtacttcaatgaatcaacaaagaagaaaattgagaaaataaggcctcttttaatggatggcacagcatcactgagtcctgggatgatgatgggcatgttcaacatgctaagtacggtcttgggagtctcgatactgaatcttggacaaaagaaatacaccaagacaacatactggtgggatgggctccaatcatccgacgattttgctctcatagtgaatgcaccaaaccatgagggaatacaagcaggagtggacagattctacaggacctgcaagttagtgggaatcaacatgagcaaaaagaagtcctatataaataagacagggacatttgaattcacaagctttttttatcgctatggatttgtggctaattttagcatggagctacccagctttggagtgtctggagtaaatgaatcagctgacatgagtattggagtaacagtgataaagaacaacatgataaacaatgaccttggacctgcaacggcccagatggctcttcaattgttcatcaaagactacagatacacatataggtgccataggggagacacacaaattcagacgagaagatcatttgagttaaagaagctgtgggatcaaacccaatcaaaggtagggctattagtatcagatggaggaccaaacttatacaatatacggaatcttcacattcctgaagtctgcttaaaatgggagctaatggatgatgattatcggggaagactttgtaatcccctgaatccctttgtaagtcataaggagattgattctgtaaacaatgctgtggtaatgccagcccatggtccagccaaaagcatggaatatgatgccgttgcaactacacattcctggattcccaagaggaatcgttctattctcaacacaagccaaaggggaattcttgaggatgaacagatgtaccagaagtgctgcaatctattcgagaaatttttccctagcagttcatataggagaccggttggaatttctagcatggtggaggccatggtgtctagggcccggattgatgccagggtcgactttgagtctggacggatcaagaaagaagagttctctgagatcatgaagatctgttccaccattgaagagctcagacggcaaaaataa

>H1N1_PB1_CY124799_A/Singapore/TT198/2011

atggatgtcaatccgactctacttttcctaaaaattccagcacaaaatgccataagcaccacattcccttatactggagatcctccatacagccatggaacaggaacaggatacaccatggacacagtaaacagaacacaccaatactcagaaaagggaaagtggacaacaaacacagagactggtgcaccccagctcaacccgattgatggaccactacctgaggataatgaaccaagtgggtatgcacaaacagactgtgttctagaggctatggctttccttgaagaatcccacccaggaatatttgagaattcatgccttgaaacaatggaagttgttcaacaaacaagggtagataaactgactcaaggtcgccagacttatgattggacattaaacagaaatcaaccggcagcaactgcattggccaacaccatagaagtctttagatcgaatgacctaacagctaatgagtcaggaaggctaatagatttcttaaaggatgtaatggaatcaatgaacaaagaggaaatagagataacaacccactttcaaagaaaaaggagagtaagagacaacatgaccaagaagatggtcacgcaaagaacaatagggaagaaaaaacagagactgaataagagaggctatctaataagagcactgacattaaatacgatgaccaaagatgcagagagaggcaagttaaaaagaagggctatcgcaacacctgggatgcagattagaggtttcgtatactttgttgaaactttagctaggagcatttgcgaaaagcttgaacagtctgggctcccagtagggggcaatgaaaagaaggccaaactggcaaatgttgtgagaaagatgatgactaattcacaagacacagagatttctttcacaatcactggggacaacactaagtggaatgaaaatcaaaatcctcgaatgttcctggcgatgattacatatatcaccagaaatcaacccgagtggtttagaaacatcctgagcatggcacccataatgttctcaaacaaaatggcaagactagggaaagggtacatgttcgagagtaaaagaatgaagattcgaacacaaataccagcagaaatgctagcaagcattgacctgaagtacttcaatgaatcaacaaagaagaaaattgagaaaataaggcctcttttaatggatggcacagcatcactgagtcctgggatgatgatgggcatgttcaacatgctaagtacggtcttgggagtctcgatactgaatcttggacaaaagaaatacaccaagacaacatactggtgggatggactccaatcatccgacgattttgctctcatagtgaatgcaccaaaccatgagggaatacaagcaggagtggacagattctacaggacctgcaagttagtgggaatcaacatgagcaaaaagaagtcctatataaataagacagggacatttgaattcacaagctttttttatcgctatggatttgtggctaattttagcatggagctacccagctttggagtgtctggagtaaatgaatcagctgacatgagtattggagtaacagtgataaagaacaacatgataaacaatgaccttggacctgcaacggcccagatggctcttcaattgttcatcaaagactacagatacacatataggtgccataggggagacacacaaattcagacgagaagatcatttgagttaaagaagctgtgggatcaaacccaatcaaaggtagggctattagtatcagatggaggaccaaacttatacaatatacggaatcttcacattcctgaagtctgcttaaaatgggagctaatggatgatgattatcggggaagactttgtaatcccctgaatccctttgtaagtcataaggagattgattctgtaaacaatgctgtggtaatgccagcccatggtccagccaaaagcatggaatatgatgccgttgcaactacacattcctggattcccaagaggaatcgttctattctcaacacaagccaaaggggaattcttgaggatgaacagatgtaccagaagtgctgcaatctattcgagaaatttttccctagcagttcatataggagaccggttggaatttctagcatggtggaggccatggtgtctagggcccggattgatgccagggtcgactttgagtctggacggatcaagaaagaagagttctctgagatcatgaagatctgttccaccattgaagaactcagacggcaaaaataa

>H1N1_PB1_AB704420_A/Gunma/262/2009

atggatgtcaatccgactctacttttcctaaaaattccagcacaaaatgccataagcaccacattcccttatactggagatcctccatacagccatggaacaggaacaggatacaccatggacacagtaaacagaacacaccaatactcagaaaagggaaagtggacgacaaacacagagactggtgcaccccagctcaacccgattgatggaccactacctgaggataatgaaccaagtgggtatgcacaaacagactgtgttctagaggctatggctttccttgaagaatcccacccaggaatatttgagaattcatgccttgaaacaatggaagttgttcaacaaacaagggtagataaactaactcaaggtcgccagacttatgattggacattaaacagaaatcaaccggcagcaactgcattggccaacaccatagaagtctttagatcgaatggcctaacagctaatgagtcaggaaggctaatagattttttaaaggatgtaatggaatcaatgaacaaagaggaaatagagataacaacccactttcaaagaaaaaggagagtaagagacaacatgaccaaaaagatggtcacgcaaagaacaatagggaagaaaaaacaaagactgaataagagaggctatctaataagagcactgacattaaatacgatgaccaaagatgcagagagaggcaagttaaaaagaagggctatcgcaacacctgggatgcagattagaggtttcgtatactttgttgaaactttagctaggagcatttgcgaaaagcttgaacagtctgggctcccagtagggggcaatgaaaagaaggccaaactggcaaatgttgtgagaaagatgatgactaattcacaagacacagagatttctttcacaatcactggggacaacactaagtggaatgaaaatcaaaatcctcgaatgttcctggcgatgattacatatatcaccagaaatcaacccgagtggttcagaaacatcctgagcatggcacccataatgttctcaaacaaaatggcaagactagggaaagggtacatgttcgagagtaaaaggatgaagattcgaacacaaataccagcagaaatgctagcaagcattgacctgaagtacttcaatgaatcaacaaagaagaaaattgagaaaataaggcctcttctaatagatggcacagcatcactgagtcctgggatgatgatgggcatgttcaacatgctaagtacggtcttgggagtctcgatactgaatcttggacaaaagaaatacaccaagacaatatactggtgggatgggctccaatcatccgacgattttgctctcatagtgaatgcaccaaaccatgagggaatacaagcaggagtggacagattctacaggacctgcaagttagtgggaatcaacatgagcaaaaagaagtcctatataaataagacagggacatttgaattcacaagctttttttatcgctatggatttgtggctaattttagcatggagctacccagctttggagtgtctggagtaaatgaatcagctgacatgagtattggagtaacagtgataaagaacaacatgataaacaatgaccttggacctgcaacggcccagatggctcttcaattgttcatcaaagactacagatacacatataggtgccataggggagacacacaaattcagacgagaagatcatttgagttaaagaagctgtgggatcaaacccaatcaaaggtagggctattagtatcagatggaggaccaaacttatacaatatacggaatcttcacattcctgaagtctgcttaaaatgggagctaatggatgatgattatcggggaagactttgtaatcccctgaatccctttgtcagtcataaagagattgattctgtaaacaatgctgtggtaatgccagcccatggtccagccaaaagcatggaatatgatgccgttgcaactacacattcctggattcccaagaggaatcgttctattctcaacacaagccaaaggggaattcttgaggatgaacagatgtaccagaagtgctgcaatctattcgagaaatttttccctagcagttcatataggagaccggttggaatttctagcatggtggaggccatggtgtctagggcccggattgatgccagggtcgacttcgagtctggacggatcaagaaagaagagttctctgagatcatgaagatctgttccaccattgaagaactcagacggcaaaaataa

>H1N1_PB1_AB704436_A/Gunma/267/2009

atggatgtcaatccgactctacttttcctaaaaattccagcgcaaaatgccataagcaccacattcccttatactggagatcctccatacagccatggaacaggaacaggatacaccatggacacagtaaacagaacacaccaatactcagaaaagggaaagtggacgacaaacacagagactggtgcaccccagctcaacccgattgatggaccactacctgagaataatgaaccaagtgggtatgcacaaacagactgtgttctagaggctatggctttccttgaagaatcccacccaggaatatttgagaattcatgccttgaaacaatggaagttgttcaacaaacaagggtagataaactaactcaaggtcgccagacttatgattggacattaaacagaaatcaaccggcagcaactgcattggccaacaccatagaagtctttagatcgaatggcctaacagctaatgagtcaggaaggctaatagatttcttaaaggatgtaatggaatcaatgaacaaagaggaaatagagataacaacccactttcaaagaaaaaggagagtaagagacaacatgaccaagaagatggtcacgcaaagaacaatagggaagaaaaaacaaagactgaataagagaggctatctaataagagcactgacattaaatacgatgaccaaagatgcagagagaggcaagttaaaaagaagggctattgcaacacctgggatgcagattagaggtttcgtatactttgttgaaactttagctaggagcatttgcgaaaagcttgaacagtctgggctcccagtagggggcaatgaaaagaaggccaaactggcaaatgttgtgagaaaaatgatgactaattcacaagacacagagatttctttcacaatcactggggacaacactaagtggaatgaaaatcaaaatcctcgaatgttcctggcgatgattacatatatcaccagaaatcaacccgagtggttcagaaacatcctgagcatggcacccataatgttctcaaacaaaatggcaagactagggaaagggtacatgttcgagagtaaaagaatgaagattcgaacacaaataccagcagaaatgctagcaagcattgacctgaagtacttcaatgaatcaacaaagaagaaaattgagaaaataacgcctcttctaatagatggcacagcatcactgagtcctgggatgatgatgggcatgttcaacatgctaagtacggtcttgggagtctcgatactgaatcttggacaaaagaaatacaccaagacagtatactggtgggatgggctccaatcatccgacgattttgctctcatagtaaatgcaccaaaccatgagggaatacaagcaggagtggacagattctacaggacctgcaagttagtgggaatcaacatgagcaaaaagaagtcctatataaataagacagggacatttgaattcacaagctttttttatcgctatggatttgtggctaattttagcatggagctacccagctttggagtgtctggagtaaatgaatcagctgacatgagtattggagtaacagtgataaagaacaacatgataaacagtgaccttggacctgcaacggcccagatggctcttcaattgttcataaaagactacagatacacatataggtgccataggggagacacacaaattcagacgagaagatcatttgagttaaagaagctgtgggatcaaacccaatcaaaggtagggctattagtatcagatggaggaccaaacttatacaatatacggaatcttcacattcctgaagtctgcttaaaatgggagctaatggatgatgattatcggggaagactttgtaatcccctgaatccctttgtcagtcataaagagattgattctgtaaacaatgctgtggtaatgccagcccatggtccagccaaaagcatggaatatgatgccgttgcaactacacattcctggattcccaagaggaatcgttctattctcaacacaagccaaaggggaattcttgaggatgaacagatgtaccagaagtgctgcaatctattcgagaaatttttccctagcagttcatataggagaccggttggaatttctagcatggtggaggccatggtgtctagggcccggattgatgccagggtcgacttcgagtctggacggatcaagaaagaagagttctctgagatcatgaagatctgttccaccattgaagaactcagacggcaaaaataa

>H1N1_PB1_CY125114_A/Bangkok/163/2000

atggatgtcaatccgaccttacttttcttaaaagtgccagcacaaaatgctataagcacaacttttccttatactggtgaccctccttacagccatgggacaggaacagggtacaccatggatacagtcaacaggacacatcagtactcagaaagaggaagatggacaaaaaataccgaaactggagcaccgcaactcaacccaattgatgggccactaccaaaagacaatgaaccaagtggctatgcccaaacagattgtgtattagaagcaatggctttccttgaggaatcccatcctggtatttttgaaaactcttgtattgaaacaatggaggttgttcagcaaacaagggtggacaaactgacacaaggcagacagacctatgactggaccctaaatagaaaccagcctgctgccacagcattggccaacactatagaagtgttcagatcaaacggcctcatagcaaatgaatctgggaggctaatagacttccttaaagatgtaatggagtcgatggacagagacgaagtagagatcacaactcattttcagagaaagaggagagtgagagacaatgtaactaaaaaaatggtgacccaaagaacaataggcaaaaagaaacataaattagacaaaagaagttacctaattagggcattaaccctgaacacaatgaccaaagatgctgagagggggaaactaaaacgtagagcaattgcaaccccaggaatgcaaataagggggtttgtatactttgttgagacactggcaagaagcatatgtgaaaagcttgaacaatcagggttgccagttgggggaaatgaaaagaaagcaaagttagcaaatgttgtaaggaagatgatgaccaactcccaggacactgaaatttctttcaccatcactggagataacacaaaatggaacgaaaatcaaaaccctagaatgtttttggccatgatcacatatataaccaaaaatcagcctgaatggttcagaaatattctaagtattgctccaataatgttttcaaacaaaatggcgagactaggtaaggggtacatgtttgaaagcaagagtatgaaactgagaactcaaatacctgcagagatgctagccaacatagatttgaaatatttcaatgattcaactaaaaagaaaattgaaaaaatccggccattattaatagatggaactgcatcattgagtcctggaatgatgatgggcatgttcaatatgttaagcaccgtcttgggcgtctccattctgaatcttgggcaaaagagatacaccaagactacttactggtgggatggtcttcaatcgtctgatgattttgctctgattgtgaatgcacccaactatgcaggaattcaagctggagttgacaggttttatcgaacctgtaagctgctcggaattaatatgagcaaaaagaagtcctacataaacagaacaggtacctttgaattcacgagctttttctatcgttatgggtttgttgccaatttcagcatggagcttcctagttttggggtgtctggggtcaatgagtctgcagacatgagtattggagtcactgtcatcaaaaacaatatgataaacaatgaccttggcccagcaactgctcaaatggcccttcagttatttatcaaagattacaggtacacgtatcgatgccacagaggtgacacacaaatacaaacccggagaacatttgagataaagaaactatgggaccaaacccgctccaaagctgggctgttggtctctgatggaggccccaatttatataacattagaaatctccatattcctgaagtctgcttgaaatgggagttgatggatgaggattaccaggggcgtttatgcaacccattgaacccgtttgtcagtcataaagagattgaatcagtgaacaatgcagtgatgatgccggcacacggtccagccaaaaatatggagtatgacgctgttgcaacaacacactcctgggttcccaaaaggaatcgatccattttgaatacgagccaaagggggatacttgaggatgagcaaatgtatcagaagtgctgcaatttatttgaaaaattcttcccaagtagctcatacagaagaccagttggaatatccagtatggtagaggctatggtttccagagcccgaattgatgcacggattgatttcgaatctggaaggataaagaaagaggaattcgctgagatcatgaagacctgttccaccattgaagacctcagacggcaaaaatag

>H1N1_PB1_CY125154_A/Singapore/14/2001

atggatgtcaatccgacattacttttcttaaaagtgccagcacaaaatgctataagcaccacttttccttatactggtgaccctccttacagccatgggacaggaacagggtacaccatggatacagtcaacaggacacatcagtactcagaaagaggaagatggacaaaaaacaccgaaactggagcaccgcaactcaacccaattgatgggccactaccagaagacaatgaaccaagtggctatgcccaaacagattgtgtattagaagcaatggctttccttgaagaatcccatcctggtatttttgaaaactcttgtattgaaacaatggaggttgttcagcaaacaagggtggacaaactgacacaaggcagacagacctatgactggactctaaataggaaccagcctgctgccacagcattggccaacactatagaagtattcagatcaaacggcctcatagcaaatgaatctgggaggctaatagacttcctcaaagatgtaatggagtcgatggacagagacgaagtagaggtcacaactcattttcagagaaagaggagagtgagagacaatgtaactaaaaaaatggtgacccaaagaacaataggcaaaaagaaacataaattagacaaaaggagttacctaattagagcattaaccctgaacacaatgaccaaagatgctgagagggggaaactaaaacgcagagcaattgcaaccccaggaatgcaaataagggggtttgtatactttgttgagacactggcaagaagcatatgtgaaaagcttgaacaatcagggttgccagttggaggaaatgagaagaaagcaaagttagcaaatgttgtaaggaagatgatgaccaactcccaggacactgaaatttctttcaccatcactggagataacacaaaatggaacgaaaatcaaaaccctagaatgtttttggccatgatcacatatataaccaaaaatcagcctgaatggttcagaaatattctaagtattgctccaataatgttttcaaacaaaatggcgagactaggtaaggggtacatgtttgaaagcaagagtatgaaactgagaactcaaatacctgcagagatactagccgacatagatttgaaatatttcaatgagtcaactaaaaagaaaattgaaaaaatccgaccattattaatagatggaactgcatcattgagtcctggaatgatgatgggcatgttcaatatgttaagcactgtcttgggcgtctccattctgaatcttgggcaaaagagatacaccaagactacttactggtgggatggtcttcaatcgtctgatgattttgctctgatagtgaatgcgcccaactatgcaggaattcaagctggagttgacaggttttatcgaacctgtaagctgctcggaattaatatgagcaaaaagaagtcttacataaacaggacaggtacctttgaattcacgagctttttctatcgctatgggtttgttgcaaatttcagcatggagcttcctagttttggggtgtctggggtcaatgaatctgcagacatgagtattggagtcactgtcatcaaaaacaatatgataaacaatgaccttggcccagcaactgctcaaatggcccttcagttatttataaaagattacagatacacgtatcgatgccacagaggtgacacacaaatacaaacccagagatcatttgagataaagaaattatgggaccaaacccgctccaaagctgggctgttggtctctgatggaggccccaatttatataacattagaaatctccatattcctgaagtctgcttgaaatgggagttgatggatgaggattaccaggggcgtttatgcaacccattgaacccgtttgtcagtcataaagagattgaatcagtgaacaatgcagtgatgatgccggcacatggtccagccaaaaatatggagtatgacgctgttgcaacaacacactcctgggttcccaaaaggaatcgatccattttgaacacgagccaaagggggatacttgaggatgagcaaatgtatcagagatgctgcaatttatttgaaaaattcttcccaagtagctcatacagaagaccagttgggatatccagtatggtagaggctatggtttccagagcccgaattgatgcacggattgatttcgaatctggaaggataaagaaagaggaattcgctgagatcatgaagacctgttccaccattgaagacctcagacggcaaaaatag

>H1N1_PB1_JX308991_A/Singapore/GP329/2010

atggatgtcaatccgactctacttttcctaaaaattccagcgcaaaatgccataagcaccacattcccttatactggagatcctccatacagccatggaacaggaacaggatacaccatggacacagtaaacagaacacaccaatactcagaaaagggaaagtggacgacaaacacagagactggtgcaccccagctcaacccgattgatggaccactacctgaggataatgaaccaagtgggtatgcacaaacagactgtgttctagaggctatggctttccttgaagaatcccacccaggaatatttgagaattcatgccttgaaacaatggaagttgttcaacaaacaagggtagataaactaactcaaggtcgccagacttatgattggacattaaacagaaatcaaccggcagcaactgcattggccaacaccatagaagtctttagatcgaatggcctaacagctaatgagtcaggaaggctaatagatttcttaaaggatgtaatggaatcaatgaacaaagaggaaatagagataacaacccactttcaaagaaaaaggagagtaagagacaacatgaccaagaagatggtcacgcaaagaacaatagggaagaaaaaacaaagactgaataagagaggctatctaataagagcactgacattaaatacgatgaccaaagatgcagagagaggcaagttaaaaagaagggctatcgcaacacctgggatgcagattagaggtttcgtatactttgttgaagctttagctaggagcatttgcgaaaagcttgaacagtctgggctcccagtagggggcaatgaaaagaaggccaaactggcaaatgttgtgagaaagatgatgactaattcacaagacacagagatttctttcacaatcactggggacaacactaagtggaatgaaaatcaaaatcctcgaatgttcctggcaatgattacatatatcaccagaaatcaacccgagtggttcagaaacatcctgagcatggcacccataatgttctcaaacaaaatggcaagactagggaaagggtacatgttcgagagtaaaagaatgaagattcgaacacaaataccagcagaaatgctagcaagcattgacctgaagtacttcaatgaatcaacgaagaagaaaattgagaaaataaggcctcttctaatagatggcacagcatcactgagtcctgggatgatgatgggcatgttcaacatgctaagtacggtcttgggagtctcgatactgaatcttggacaaaagaaatacaccaagacaatatactggtgggatgggctccaatcatccgacgattttgctctcatagtgaatgcaccaaaccatgagggaatacaagcaggagtggacagattctacaggacctgcaagttagtgggaatcaacatgagcaaaaagaagtcctatataaataagacagggacatttgaattcacaagctttttttatcgctatggatttgtggctaattttagcatggagctacccagctttggagtgtctggagtaaatgaatcagctgacatgagtattggagtaacagtgataaagaacaacatgataaacaatgaccttggacctgcaacggcccagatggctcttcaattgttcatcaaagactacagatacacatataggtgccataggggagacacacaaattcagacgagaagatcatttgagttaaagaagctgtgggatcaaacccaatcaaaggtagggctattagtatcagatggaggaccaaacttatacaatatacggaatcttcacattcctgaagtctgcttaaaatgggagctaatggatgatgattatcggggaagactttgtaatcccctgaatccctttgtcagtcataaagagattgattctgtaaacaatgctgtggtaatgccagcccatggtccagtcaaaagcatggaatatgatgccgttgcaactacacattcctggattcccaagaggaatcgttctattctcaacacaagccaaaggggaattcttgaggatgaacagatgtaccagaagtgctgcaatctattcgagaaatttttccctagcagttcatataggagaccggttggaatttctagcatggtggaggccatggtgtctagggcccggattgatgccagggtcgacttcgagtctggacggatcaagaaagaagagttctctgagatcatgaagatctgttccaccattgaagaactcagacggcaaaaataa

>H1N1_PB1_JX309221_A/Singapore/GP1035/2010

atggatgtcaatccgactctacttttcctaaaaattccagcgcaaaatgccataagcaccacattcccttatactggagatcctccatacagccatggaacaggaacaggatacaccatggacacagtaaacagaacacaccaatattcagaaaagggaaagtggacgacaaacacagagactggtgcaccccagctcaacccgattgatggaccactacctgaggataatgaaccaagtgggtatgcacaaacagactgtgttctagaggctatggctttccttgaagaatcccacccaggaatatttgagaattcatgccttgaaacaatggaagttgttcaacaaacaagggtagataaactaactcaaggtcgccagacttatgattggacattaaacagaaatcaaccggctgcaactgcattggccaacaccatagaagtctttagatcgaatggcctaacagctaatgagtcaggaaggctaatagatttcttaaaggatgtaatggaatcaatgaacaaagaggaaatagagataacaacccactttcaaagaaaaaggagagtaagagacaacatgaccaagaagatggtcacgcaaagaacaatagggaagaaaaaacaaagactgaataagagaggctatctgataagagcactgacattaaatacgatgaccaaagatgcagagagaggcaagttaaaaagaagggctatcgcaacacctgggatgcagattagaggtttcgtatactttgttgaagctttagctaggagcatttgcgaaaagcttgaacagtctgggctcccagtagggggcaatgaaaagaaggccaaactggcaaatgttgtgagaaagatgatgactaattcacaagacacagagatttctttcacaatcactggggacaacactaagtggaatgaaaatcaaaatcctcgaatgttcctggcaatgattacatatatcaccagaaatcaacccgagtggttcagaaacatcctgagcatggcacccataatgttttcaaacaaaatggcaagactagggaaagggtacatgttcgagagtaaaagaatgaagattcgaacacaaataccagcagaaatgctagcaagcattgacctgaagtacttcaatgaatcaacgaagaagaaaattgagaaaataaggcctcttctaatagatggcacagcatcactgagtcctgggatgatgatgggcatgttcaacatgctaagtacggtcttgggagtctcgatactgaatcttggacaaaagaaatacaccaagacaatatactggtgggatgggctccaatcatccgacgattttgctctcatagtgaatgcaccaaaccatgagggaatacaagcaggagtggacagattctacaggacctgcaagttagtgggaatcaacatgagcaaaaagaagtcctatataaataagacagggacatttgaattcacaagctttttttatcgctatggatttgtggctaattttagcatggagctacccagctttggagtgtctggagtaaatgaatcagctgacatgagtattggagtaacagtgataaagaacaacatgataaacaatgaccttggacctgcaacggcccagatggctcttcaattgttcatcaaagactacagatacacatataggtgccataggggagacacacaaattcagacgagaagatcatttgagttaaagaagctgtgggatcaaacccaatcaaaggtagggctattagtatcagatggaggaccaaacttatacaatatacggaatcttcacattcctgaagtctgcttaaaatgggagctaatggatgatgattatcggggaagactttgtaatcccctgaatccctttgtcagtcataaagagattgattctgtaaacaatgctgtggtaatgccagcccatggtccagtcaaaagcatggaatatgatgccgttgcaactacacattcctggattcccaagaggaatcgttctattctcaacacaagccaaaggggaattcttgaggatgaacagatgtaccagaagtgctgcaatctattcgagaaatttttccctagcagttcatataggagaccggttggaatttctagcatggtggaggccatggtgtctagggcccggattgatgccagggtcgacttcgagtctggacggatcaagaaagaagagttctctgagatcatgaagatctgttccaccattgaagaactcagacggcaaaaataa

>H1N1_PB1_JX309368_A/Singapore/GP1212/2010

atggatgtcaatccgactctacttttcctaaaaattccagcgcaaaatgccataagcaccacattcccttatactggagatcctccatacagccatggaacaggaacaggatacaccatggacacagtaaacagaacacaccaatactcagaaaagggaaagtggacgacaaacacagagactggtgcaccccagctcaacccgattgatggaccactacctgaggataatgaaccaagtgggtatgcacaaacagactgtgttctggaggctatggctttccttgaagaatcccacccaggaatatttgagaattcatgccttgaaacaatggaagttgttcaacaaacaagggtagataaactaactcaaggtcgccagacttatgattggacattaaatagaaatcaaccggcagcaactgcattggccaacaccatagaagtctttagatcgaatggcctaacagctaatgagtcaggaaggctaatagatttcttaaaggatgtaatggaatcaatgaacaaagaggaaatagagataacaacccactttcaaagaaaaaggagagtaagagacaacatgaccaagaagatggtcacgcaaagaacaatagggaagaaaaaacaaagactgaataagagaggctatctaataagagcactgacattaaatacgatgaccaaagatgcagagagaggcaagttaaaaagaagggctatcgcaacacctgggatgcagattagaggtttcgtatactttgttgaaactttagctaggagcatttgcgaaaagcttgaacagtctgggctcccagtagggggcaatgaaaagaaggccaaactggcaaatgttgtgagaaagatgatgactaattcacaagacacagagatttctttcacaatcactggggacaacactaagtggaatgaaaatcaaaatcctcgaatgttcctggcaatgattacatatatcaccagaaatcaacccgagtggttcagaaacatcctgagcatggcacccataatgttctcaaacaaaatggcaagactagggaaagggtacatgttcgagagtaaaagaatgaagattcgaacacaaataccagcagaaatgctagcaagcattgacctgaagtacttcaatgaatcaacaaagaaaaaaattgagaaaataaggcctcttctaatagatggcacagcatcactgagtcctgggatgatgatgggcatgttcaacatgctaagtacggtcttgggagtctcgatactgaatcttggacaaaagaaatacaccaagacaacatactggtgggatgggctccaatcatccgacgattttgctctcatagtgaatgcaccaaaccatgagggaatacaagcaggagtggacagattctacaggacctgcaagttagtgggaatcaacatgagcaaaaagaagtcctatataaataagacagggacatttgaattcacaagctttttttatcgctatggatttgtggctaattttagcatggagctacccagctttggagtgtctggagtaaatgaatcagctgacatgagtattggagtaacagtgataaagaacaacatgataaacaatgaccttggacctgcaacggcccagatggctcttcaattgttcatcaaagactacagatacacatataggtgccataggggagacacacaaattcagacgagaagatcatttgagttaaagaagctgtgggatcaaacccaatcaaaggtagggctattagtatcagatggaggaccaaacttatacaatatacggaatcttcacattcctgaagtctgcttaaaatgggagctaatggatgatgattatcggggaagactttgtaatcccctgaatccctttgtcagtcataaagagattgattctgtaaacaatgctgtggtaatgccagcccatggtccagccaaaagcatggaatatgatgccgttgcaactacacattcctggattcccaagaggaatcgttctattctcaacacaagccaaaggggaattcttgaggatgaacagatgtaccagaagtgctgcaatctattcgagaaatttttccctagcagttcatataggagaccggttggaatttctagcatggtggaggccatggtgtctagggcccggattgatgccagggtcgacttcgagtctggacggatcaagaaagaagagttctctgagatcatgaagatctgttccaccattgaagaactcagacggcaaaaataa

>H1N1_PB1_JX309520_A/Singapore/SS35/2010

atggatgtcaatccgactctacttttcctaaaaattccagcgcaaaatgccataagcaccacattcccttatactggagatcctccatacagccatggaacaggaacaggatacaccatggacacagtaaacagaacacaccaatactcagaaaagggaaagtggacgacaaacacagagactggtgcaccccagctcaacccgattgatggaccactacctgaggataatgaaccaagtgggtatgcacaaacagactgtgttctagaggctatggctttccttgaagaatcccacccaggaatatttgagaattcatgccttgaaacaatggaagttgttcaacaaacaagggtagataaactaactcaaggtcgccagacttatgattggacattaaacagaaatcaaccggcagcaactgcattggccaacaccatagaagtctttagatcgaatggcctaacagctaatgagtcaggaaggctaatagatttcttaaaggatgtaatggaatcaatgaacaaagaggaaatagagataacaacccactttcaaagaaaaaggagagtaagagacaacatgaccaagaagatggtcacgcaaagaacaatagggaagaaaaaacaaagactgaataagagaggctatctaataagagcactgacattaaatacgatgaccaaagatgcagagagaggcaagttaaaaagaagggctatcgcaacacctgggatgcagattagaggtttcgtatactttgttgaagctttagctaggagtatttgcgaaaagcttgaacagtctgggctcccagtagggggcaatgaaaagaaggccaaactggcaaatgttgtgagaaagatgatgactaattcacaagacacagagatttctttcacaatcactggggacaacactaagtggaatgaaaatcaaaatcctcgaatgttcctggcaatgattacatatatcaccagaaatcaacccgagtggttcagaaacatcctgagcatggcacccataatgttctcaaacaaaatggcaagactagggaaagggtacatgttcgagagtaaaagaatgaagattcgaacacaaataccagcagaaatgctagcaagcattgacctgaagtacttcaatgaatcaacgaagaagaaaattgagaaaataaggcctcttctaatagatggcacagcatcactgagtcctgggatgatgatgggcatgttcaacatgctaagtacggtcttgggagtctcgatactgaatcttggacaaaagaaatacaccaagacaatatactggtgggatgggctccaatcatccgacgattttgctctcatagtgaatgcaccaaaccatgagggaatacaagcaggagtggacagattctacaggacctgcaagttagtgggaatcaacatgagcaaaaagaagtcctatataaataagacagggacatttgaattcacaagctttttttatcgctatggatttgtggctaattttagcatggagctacccagctttggagtgtctggagtaaatgaatcagctgacatgagtattggagtaacagtgataaagaacaacatgataaacaatgaccttggacctgcaacggcccagatggctcttcaattgttcatcaaagactacagatacacatataggtgccataggggagacacacaaattcagacgagaagatcatttgagttaaagaagctgtgggatcaaacccaatcaaaggtagggctattagtatcagatggaggaccaaacttatacaatatacggaatcttcacattcctgaagtctgcttaaaatgggagctaatggatgatgattatcggggaagactttgtaatcccctgaatccctttgtcagtcataaagagattgattctgtaaacaatgctgtggtaatgccagcccatggtccagtcaaaagcatggaatatgatgccgttgcaactacacattcctggattcccaagaggaatcgttctattctcaacacaagccaaaggggaattcttgaggatgaacagatgtaccagaagtgctgcaatctattcgagaaatttttccctagcagttcatataggagaccggttggaatttctagcatggtggaggccatggtgtctagggcccggattgatgccagggtcgacttcgagtctggacggatcaagaaagaagagttctctgagatcatgaagatctgttccaccattgaagaactcagacggcaaaaataa

>H1N1_PB1_JX309750_A/Singapore/GP3760/2010

atggatgtcaatccgactctacttttcctgaaaattccagcgcaaaatgccataagcaccacattcccttatactggagatcctccatacagccatggaacaggaacaggatacaccatggacacagtaaacagaacacaccaatactcagaaaagggaaagtggacgacaaacacagagactggtgcaccccagctcaacccgattgatggaccactacctgaggataatgaaccaagtgggtatgcacaaacagactgtgttctagaggctatggctttccttgaagaatcccacccaggaatatttgagaattcatgccttgaaacaatggaagttgttcaacaaacaagggtagataaactaactcaaggtcgccagacttatgattggacattaaacagaaatcagccggcagcaacggcattggccaacaccatagaagtcttcagatcgaatggcctaacagctaatgagtcaggaaggctaatagatttcttaaaggatgtaatggaatcaacgaacaaagaggaaatagagataacaacccactttcaaagaaaaaggagagtaagagacaacatgaccaagaagatggtcacgcaaagaacaatagggaagaaaaaacaaaaactgaataagagaggctatctaataagagcactgacattaaatacgatgaccaaagatgcagagagaggcaagttaaaaagaagggctatcgcaacacctgggatgcagattagaggtttcgtatactttgttgaaactttagctaggagcatttgcgaaaagcttgaacagtctgggctcccagtagggggcaatgaaaagaaggccaaactggcaaatgttgtgagaaagatgatgactaattcacaagacacagagatttctttcacaatcactggggacaacactaagtggaatgaaaatcaaaatcctcgaatgttcctggcgatgattacatatatcaccagaaatcaacccgaatggttcagaaacatcctgagcatggcacccataatgttctcaaacaaaatggcaagactagggaaagggtacatgttcgagagtaaaagaatgaagattcgaacacaaataccagcagaaatgctagcaagcattgacctgaagtacttcaatgaatcaacaaagaagaaaattgagaaaataaggcctcttctaatagatggcacagcatcactgagtcctgggatgatgatgggcatgttcaacatgctaagtacggtcttgggagtctcgatactgaatcttggacaaaagaaatacaccaagacagtatactggtgggatgggctccaatcatccgacgattttgctctcatagtgaatgcaccaaaccatgagggaatacaagcaggagtggacagattctacaggacctgcaagttagtgggaattaacatgagcaaaaagaagtcctatataaataagacagggacatttgaattcacaagctttttttatcgctatggatttgtggctaattttagcatggagctacccagctttggagtgtctggagtaaatgaatcagctgacatgagtattggagtaacagtgataaagaacaacatgataaacaatgaccttggacctgcaacggcccagatggctcttcaattgttcatcaaagactacagatacacatataggtgccataggggagacacacaaattcagacaagaagatcatttgagttaaagaagctgtgggatcaaacccaatcaaaggtagggctattagtatcagatggagggccaaacttatacaatatacggaatcttcacattcctgaagtctgcttaaaatgggagctaatggatgatgattatcggggaagactttgtaatcccctgaatccctttgtcagtcataaagagattgattctataaacaatgctgtggtaatgccagcccatggtccagtcaaaagcatggaatatgatgccgttgcaactacacattcctggattcccaagaggaatcgttctattctcaacacaagccaaaggggaattcttgaggatgaacagatgtaccagaagtgctgcaatctattcgagaaatttttccctagcagttcatataggagaccggttggaatttctagcatggtggaggccatggtgtctagggcccggattgatgccagggttgacttcgagtctggacggatcaagaaagaagagttctctgagatcatgaagatctgttccaccattgaagaactcagacggcaaaaataa

>H1N1_PB1_JX309774_A/Singapore/GP4183/2010

atggatgtcaatccgactctacttttcctgaaaattccagcgcaaaatgccataagcaccacattcccttatactggagatcctccatacagccatggaacaggaacaggatacaccatggacacagtaaacagaacacaccaatactcagaaaagggaaagtggacgacaaacacagagactggagcaccccagctcaacccgattgatggaccactacctgaggataatgaaccaagtgggtatgcacaaacagactgtgttctagaggctatggctttccttgaagaatcccacccaggaatatttgagaattcatgccttgaaacaatggaagttgttcaacaaacaagggtagataaactaactcaaggtcgccagacttatgattggacattaaacagaaatcaaccggcagcaactgcattggccaacaccatagaagtctttagatcgaatggcctaacagctaatgagtcaggaaggctaatagatttcttaaaggatgtaatggaatcaatgaacaaagaggaaatagagataacaacccactttcaaagaaaaaggagagtaagagacaacatgaccaagaagatggtcacgcaaagaacaatagggaagaaaaaacaaaaactgaataagagaggctatctaataagagcactgacattaaatacgatgaccaaagatgcagagagaggcaagttaaaaagaagggctatcgcaacacctgggatgcagattagaggtttcgtatactttgttgaaactttagctaggagcatctgcgaaaagcttgaacagtctgggctcccagtagggggcaatgaaaagaaggccaaactggcaaatgttgtgagaaagatgatgactaattcacaagacacagagatttctttcacaatcactggggacaacactaagtggaatgaaaatcaaaatcctcgaatgtttctggcgatgattacatatatcaccagaaatcaacccgagtggttcagaaacatcctgagcatggcacccataatgttctcaaacaaaatggcaagactagggaaagggtacatgttcgagagtaaaagaatgaagattcgaacacaaataccagcagaaatgctagcaagcattgacctgaagtacttcaatgaatcaacaaagaagaaaattgagaaaataaggcctcttctaatagatggcacagcatcactgagtcctgggatgatgatgggcatgttcaacatgctaagtacggtcttgggagtctcgatactgaatcttggacaaaagaaatacaccaagacagtatactggtgggatgggctccaatcatccgacgattttgctctcatagtgaatgcaccaaaccatgagggaatacaagcaggagtggacagattctacaggacctgcaagttagtgggaatcaacatgagcaaaaagaagtcctatataaataagacagggacatttgaattcacaagctttttttatcgctatggatttgtggctaattttagcatggagctacccagctttggagtgtctggagtaaatgaatcagctgacatgagtattggagtaacagtgataaagaataacatgataaacaatgaccttggacctgcaacggcccagatggctcttcaattgttcatcaaagactacagatacacatataggtgccataggggagacacacaaattcagacgagaagatcatttgagttaaagaagctgtgggatcaaacccaatcaaaggtagggctattagtatcagatggaggaccaaacttatacaatatacggaatcttcacattcctgaagtctgcttaaaatgggagctaatggatgatgattatcggggaagactttgtaatcccctgaatccctttgtcagtcataaagagattgattctgtaaacaatgctgtggtaatgccagcccatggtccagtcaaaagcatggaatatgatgccgttgcaactacacattcctggattcccaagaggaatcgttctattctcaacacaagccaaaggggaattcttgaggatgaacagatgtaccagaagtgctgcaatctattcgagaaatttttccctagcagttcatataggagaccggttggaatttctagcatggtggaggccatggtgtctagggcccggattgatgccagggtcgacttcgagtctggacggatcaagaaagaagagttctctgagatcatgaagatctgttccaccattgaagaactcagacggcaaaaataa

>H1N1_PB1_JX309798_A/Singapore/GP4138/2010

atggatgtcaatccgactctacttttcctgaaaattccagcgcaaaatgccataagcaccacattcccttatactggagatcctccatacagccatggaacaggaacaggatacaccatggacacagtaaacagaacacaccaatactcagaaaagggaaaatggacgacaaacacagagactggtgcaccccagctcaacccgattgatggaccactacctgaggataatgaaccaagtgggtatgcacaaacagactgtgttctagaggctatggctttccttgaagaatcccacccaggaatatttgagaattcatgccttgaaacaatggaagttgttcaacaaacaagggtagataaactaactcaaggtcgccagacttatgattggacattaaacagaaatcaaccggcagcaactgcattggccaacaccatagaagtctttagatcgaatggcctaacagctaatgagtcaggaaggctaatagatttcttaaaggatgtaatggaatcaatgaacaaagaggaaatagagataacaacccactttcaaagaaaaaggagagtaagagacaacatgaccaagaagatggtcacgcaaagaacaatagggaagaaaaaacaaaaactgaataagagaggctatctaataagagcactgacgttaaatacgatgaccaaagatgcagagagaggcaagttaaaaagaagggctatcgcaacacctgggatgcagattagaggtttcgtatactttgttgaaactttagctaggagcatttgcgaaaagcttgaacagtctgggctcccagtagggggcaatgaaaagaaggccaaactggcaaatgttgtgagaaagatgatgactaattcacaagacacagagatttctttcacaatcactggggacaacactaagtggaatgaaaatcaaaatcctcgaatgttcctggcgatgattacatatatcaccagaaatcaacccgagtggttcagaaacatcctgagcatggcacccataatgttctcaaacaaaatggcaagactagggaaagggtacatgttcgagagtaaaagaatgaagattcgaacacaaataccagcagaaatgctagcaagcattgacctgaagtacttcaatgaatcaacaaagaagaaaattgagaaaataaggcctcttctaatagatggcacagcatcactgagtcctgggatgatgatgggcatgttcaacatgctaagtacggtcttgggagtctcgatactgaatcttggacaaaagaaatacaccaagacaatatactggtgggatgggctccaatcatccgacgattttgctctcatagtgaatgcaccaaaccatgagggaatacaagcaggagtggacagattctacaggacctgcaagttagtgggaatcaacatgagcaaaaagaagtcctatataaataagacagggacatttgaattcacaagctttttttatcgctatggatttgtggctaattttagcatggagctacccagctttggagtgtctggagtaaatgaatcagctgacatgagtattggagtaacagtgataaagaacaacatgataaacaatgaccttggacctgcaacggcccagatggctcttcaattgttcatcaaagactacagatacacatataggtgccataggggagacacacaaattcagacgagaagatcatttgagttaaagaagctgtgggatcaaacccaatcaaaggtagggctattagtatcagatggaggaccaaacttatacaatatacggaatcttcacattcctgaagtctgcttaaaatgggagctgatggatgatgattatcggggaagactttgtaatcccctgaatccctttgtcagtcataaagagattgattctgtaaacaatgctgtggtaatgccagcccatggtccagtcaaaagcatggaatatgatgccgttgcaactacacattcctggattcccaagaggaatcgttctattctcaacacaagccaaaggggaattcttgaggatgaacagatgtatcagaagtgctgcaatctattcgagaaatttttccctagcagttcatataggagaccggttggaatttctagcatggtggaggccatggtgtctagggcccggattgatgccagggtcgacttcgagtctggacggatcaagaaagaagagttctctgagatcatgaagatctgttccaccattgaagaactcagacggcaaaaataa

>H1N1_PB1_JX309982_A/Singapore/KK734/2010

atggatgtcaatccgactctacttttcctaaaaattccagcgcaaaatgccataagcaccacattcccttatactggagatcctccatacagccatggaacaggaacaggatacaccatggacacagtaaacagaacacaccaatactcagaaaagggaaggtggacgacaaacacagagactggtgcaccccagctcaacccgattgatggaccactacctgaggataatgaaccaagtgggtatgcacaaacagactgtgttctagaggctatggctttccttgaagaatcccacccaggaatatttgagaattcatgccttgaaacaatggaagctgttcaacaaacaagggtagataaactaactcaaggtcgccagacttatgattggacattgaacagaaatcaaccggcagcaactgcattggccaacaccatagaagtctttagatcgaatggcctaacagctaatgagtcaggaaggctaatagatttcttaaaggatgtaatggaatcaatgaacaaagaggaaatagagataacaacccactttcaaagaaaaaggagagtaagagacaacatgaccaagaagatggtcacgcaaagaacaataggaaagaaaaagcaaagactgaataagagaggctatctaataagagcactgacattaaatacgatgaccaaagatgcagagagaggcaagttaaaaagaagggctatcgcaacacctgggatgcagattagaggtttcgtatactttgttgaaactttagctaggagcatttgcgaaaagcttgaacagtctgggctcccagtagggggcaatgaaaagaaggccaaactggcaaatgttgtgagaaagatgatgactaattcacaagacacagagatttctttcacaatcactggggacaacactaagtggaatgaaaatcaaaatcctcgaatgttcctggcgatgattacatatatcaccagaaatcaacccgagtggttcagaaacatcctgagcatggcacccataatgttctcaaacaaaatggcaagactagggaaagggtacatgttcgagagtaaaagaatgaagattcgaacacaaattccagcagaaatgctagcaagcattgacctgaagtacttcaatgaatcaacaaagaggaaaattgagaaaataaggcctcttctaatagatggcacagcatcactgagtcctgggatgatgatgggcatgttcaacatgctaagtacggtcttgggagtctcgatactgaatcttggacaaaagaaatacaccaagacaacatactggtgggatgggctccaatcatccgacgattttgctctcatagtgaatgcaccaaaccatgagggaatacaagcaggagtggacagattctacaggacctgcaagttagtgggaatcaacatgagcaaaaagaagtcatatataaataaaacagggacatttgaattcacaagctttttttatcgctatggatttgtggctaattttagcatggagctacccagctttggagtgtctggagtaaatgaatcagctgacatgagtattggagtaacagtgataaagaacaacatgataaacaatgaccttggacctgcaacggcccagatggctcttcaattgttcatcaaagactacagatacacatataggtgccataggggagacacacaaattcagacgagaagatcatttgagttaaaaaagctgtgggatcaaacccaatcaaaggtagggctattagtatcagatggaggaccaaacttatacaatatacggaatcttcacattcctgaagtctgcttaaaatgggagctaatggatgatgattatcggggaagactttgcaatcccctgaatccctttgtcagtcataaagagattgattctgtaaacaatgctgtggtaatgccagcccatggtccagccaaaagcatggaatatgatgccgttgcaactacacattcctggattcccaagaggaatcgttctattctcaacacaagccaaaggggaattcttgaggatgaacagatgtaccagaagtgctgcaatctattcgagaaatttttccctagcagttcatataggagaccggttggaatttctagcatggtggaggccatggtgtctagggcccggattgatgccagggtcgacttcgagtctggacggatcaagaaagaagagttctctgagatcatgaagatctgttccaccattgaagaactcagacggcaaaaataa

>H1N1_PB1_CY069787_A/England/222/2009

atggatgtcaatccgactctacttttcctaaaaattccagcgcaaaatgccataagcaccacattcccttatactggagatcctccatacagccatggaacaggaacaggatacaccatggacacagtaaacagaacacaccaatactcagaaaagggaaaatggacgacaaacacagagactggtgcaccccagctcaacccgattgatggaccactacctgaggataatgaaccaagtgggtatgcacaaacagactgtgttctagaggctatggctttccttgaagaatcccacccgggaatatttgagaattcatgccttgaaacaatggaagttgttcaacaaacaagggtagataaactaactcaaggtcgccagacttatgattggacattaaacagaaatcaaccggcagcaactgcattggccaacaccatagaagtctttagatcgaatggcctaacagctaatgagtcaggaaggctaatagatttcttaaaggatgtaatggaatcaatgaacaaagaggaaatagagataacaacccactttcaaagaaaaaggagagtaagagacaacatgaccaagaagatggtcacgcaaagaacaatagggaagaaaaaacaaagactgaataagagaggctatctaataagagcactgacattaaatacgatgaccaaagatgcagagagaggcaagttaaaaagaagggctatcgcaacacctgggatgcagattagaggtttcgtatactttgttgaaactttagctaggagcatttgcgaaaagcttgaacagtctgggctcccagtagggggcaatgaaaagaaggccaaactggcaaatgttgtgagaaagatgatgactaattcacaagacacagagatttctttcacaatcactggggacaacactaagtggaatgaaaatcaaaatcctcgaatgttcctggcgatgattacatatatcaccagaaatcaacccgagtggttcagaaacatcctgagcatggcacccataatgttctcaaacaaaatggcaagactagggagagggtacatgttcgagagtaaaagaatgaagattcgaacacaaataccagcagaaatgctagcaagcattgacctgaagtacttcaatgaatcaacaaagaagaaaattgagaaaataaggcctcttctaatagatggcacagcatcactgagtcctgggatgatgatgggcatgttcaacatgctaagtacggtcttgggagtctcgatactgaatcttggacaaaagaaatacaccaagacaatatactggtgggatgggctccaatcatccgacgattttgctctcatagtgaatgcaccaaaccatgagggaatacaagcaggagtggacagattctacaggacctgcaagttagtgggaatcaacatgagcaaaaagaagtcctatataaataagacagggacatttgaattcacaagctttttttatcgctatggatttgtggctaattttagcatggagctacccagctttggagtgtctggagtaaatgaatcagctgacatgagtattggagtaacagtgataaagaacaacatgataaacaatgaccttggacctgcaacggcccagatggctcttcaattgttcatcaaagactacagatacacatataggtgccataggggagacacacaaattcagacgagaagatcatttgagttaaagaagctgtgggatcaaacccaatcaaaggtagggctattagtatcagatggaggaccaaacttatacaatatacggaatcttcacattcctgaagtctgcttaaaatgggagctaatggatgatgattatcggggaagactttgtaatcccctgaatccctttgtcagtcataaagagattgattctgtaaacaatgctgtggtaatgccagcccatggtccagccaaaagcatggaatatgatgccgttgcaactacacattcctggattcccaagaggaatcgttctattctcaacacaagccaaaggggaattcttgaggatgaacagatgtaccagaagtgctgcaatctattcgagaaatttttccctagcagttcatataggagaccggttggaatttctagcatggtggaggccatggtgtctagggcccggattgatgccagggtcgacttcgagtctggacggatcaagaaagaagagttctctgagatcatgaagatctgttccaccattgaagaactcagacggcaaaaataa

>H1N1_PB1_CY069883_A/England/383/2009

atggatgtcaatccgactctacttttcctaaaaattccagcgcaaaatgccataagcaccacattcccttatactggagatcctccatacagccatggaacaggaacaggatacaccatggacacagtaaacagaacacaccaatactcagaaaagggaaagtggacgacaaacacagagactggtgcaccccagctcaacccgattgatggaccactacctgaggataatgaaccaagtgggtatgcacaaacagactgtgttctagaggctatggctttccttgaagaatcccacccaggaatatttgagaattcatgccttgaaacaatggaagttgttcaacaaacaagggtagataaactaactcaaggtcgccagacttatgattggacattaaacagaaatcaaccggcagcaactgcattggccaacaccatagaagtctttagatcgaatggcctaacagctaatgagtcaggaaggctaatagatttcttaaaggatgtaatggaatcaatgaacaaagaggaaatagagataacaacccactttcaaagaaaaaggagagtaagagacaacatgaccaagaagatggtcacgcaaagaacaatagggaagaaaaaacaaagactgaataagagaggctatctaataagagcactgacattaaatacgatgaccaaagatgcagagagaggcaagttaaaaagaagggctatcgcaacacctgggatgcagattagaggtttcgtatactttgttgaaactttagctaggagcatttgcgaaaagcttgaacagtctgggctcccagtagggggcaatgaaaagaaggccaaactggcaaatgttgtgagaaagatgatgactaattcacaagacacagagatttctttcacaatcactggggacaacactaagtggaatgaaaatcaaaatcctcgaatgttcctggcgatgattacatatatcaccagaaatcaacccgagtggttcagaaacatcctgagcatggcacccataatgttctcaaacaaaatggcaagactagggaaagggtacatgttcgagagtaaaagaatgaagattcgaacacaaataccagcagaaatgctagcaagcattgacctgaagtacttcaatgaatcaacaaagaagaaaattgagaaaataaggcctcttctaatagatggcacagcatcactgagtcctgggatgatgatgggcatgttcaacatgctaagtacggtcttgggagtctcgatactgaatcttggacaaaagaaatacaccaagacaatatactggtgggatgggctccaatcatccgacgattttgctctcatagtgaatgcaccaaaccatgagggaatacaagcaggagtggacagattctacaggacctgcaagttagtgggaatcaacatgagcaaaaagaagtcctatataaataagacagggacatttgaattcacaagctttttttatcgctatggatttgtggctaattttagcatggagctacccagctttggagtgtctggagtaaatgaatcagctgacatgagtattggagtaacagtgataaagaacaacatgataaacaatgaccttggacctgcaacggcccagatggctcttcaattgttcatcaaagactacagatacacatataggtgccataggggagacacacaaattcagacgagaagawcatttgagttaaagaagctgtgggatcaaacccaatcaaaggcagggctattagtatcagatggaggaccaaacttatacaatatacggaatcttcacattcctgaagtctgcttaaaatgggagctaatggatgatgattatcggggaagactttgtaatcccctgaatccctttgtcagtcataaagagattgattctgtaaacaatgctgtggtaatgccagcccatggtccagccaaaagcatggaatatgatgccgttgcaactacacattcctggattcccaagaggaatcgttctattctcaacacaagccaaaggggaattcttgaggatgaacagatgtaccagaagtgctgcaatctattcgagaaatttttccctagcagttcatataggagaccggttggaatttctagcatggtggaggccatggtgtctagggcccggattgatgccagggtcgacttcgagtctggacggatcaagaaagaagagttctctgagatcatgaagatctgttccaccattgaagaactcagacggcaaaaataa

>H1N1_PB1_JX625788_A/England/05000088/2010

atggatgtcaatccgactctacttttcctaaaaattccagcgcaaaatgccataagcaccacattcccttatactggagatcctccatacagccatggaacaggaacaggatacaccatggacacagtaaacagaacacaccaatactcagaaaagggaaagtggacgacaaacacagagactggtgcaccccagctcaacccgattgatggaccactacctgaggataatgaaccaagtgggtatgcacaaacagactgtgttctagaggctatggctttccttgaagaatcccacccaggaatatttgagaattcatgccttgaaacaatggaagttgttcaacaaacaagggtagataaactgactcaaggtcgccagacttatgattggacattaaacagaaatcaaccggcagcaactgcattggccaacaccatagaagtctttagatcgaatgacctaacagctaatgagtcaggaaggctaatagatttcttaaaggatgtaatggaatcaatgaacaaagaggaaatagagataacaacccactttcaaagaaaaaggagagtaagagacaacatgaccaagaagatggtcacgcaaagaacaatagggaagaaaaaacagagactgaataagagaggctatctaataagagcactgacattaaatacgatgaccaaagatgcagagagaggcaagttaaaaagaagggctatcgcaacacctgggatgcagattagaggtttcgtatactttgttgaaactttagctaggagcatttgcgaaaagcttgaacagtctgggctcccagtagggggcaatgaaaagaaggccaaactggcaaatgttgtgagaaagatgatgactaattcacaagacacagagatttctttcacaatcactggggacaacactaagtggaatgaaaatcaaaatcctcgaatgttcctggcgatgattacatatatcaccagaaatcaacccgagtggtttagaaacatcctgagcatggcacccataatgttctcaaacaaaatggcaagactagggaaagggtacatgttcgagagtaaaagaatgaagattcgaacacaaataccagcagaaatgctagcaagcattgacctgaagtacttcaatgaatcaacaaagaagaaaattgagaaaataaggcctcttttaatggatggcacagcatcactgagtcctgggatgatgatgggcatgttcaacatgctaagtacggtcttgggagtctcgatactgaatcttggacaaaagaaatacaccaagacaacatactggtgggatgggctccaatcatccgacgattttgctctcatagtgaatgcaccaaatcatgagggaatacaagcaggagtggacagattctacaggacctgcaagttagtgggaatcaacatgagcaaaaagaagtcctatataaataagacaggtacatttgaattcacaagctttttttatcgctatggatttgtggctaattttagcatggagctacccagctttggagtgtctggagtaaatgaatcagctgacatgagtattggagtaacagtgataaagaacaacatgataaacaatgaccttggacctgcaacggcccagatggctcttcaattgttcatcaaagactacagatacacatataggtgccataggggagacacacaaattcagacgagaagatcatttgagttaaagaagctgtgggatcaaacccaatcaaaggtagggctattagtatcagatggaggaccaaacttatacaatatacggaatcttcacattcctgaagtctgcttaaaatgggagctaatggatgatgattatcggggaagactttgtaatcccctgaatccctttgtaagtcataaggagattgattctgtaaacaatgctgtgataatgccagcccatggtccagccaaaagcatggaatatgatgccgttgcaactacacattcctggattcccaagaggaatcgttctattctcaacacaagccaaaggggaattcttgaggatgaacagatgtaccagaagtgctgcaatctattcgagaaatttttccctagcagttcatataggagaccggttggaatttctagcatggtggaggccatggtgtctagggcccggattgatgccagggtcgactttgagtctggacggatcaagaaagaagagttctctgagatcatgaagatctgttccaccattgaagaactcagacggcaaaaataa

>H1N1_PB1_JX625772_A/England/04940476/2010

atggatgtcaatccgactctacttttcctaaaaattccagcgcaaaatgccataagcaccacattcccttatactggagatcctccatacagccatggaacaggaacaggatacaccatggacacagtaaacagaacacaccaatactcagaaaagggaaagtggacgacaaacacagagactggtgcaccccagctcaacccgattgatggaccactacctgaggataatgaaccaagtgggtatgcacaaacagactgtgttctagaggctatggctttccttgaagaatcccacccaggaatatttgagaattcatgccttgaaacaatggaagttgttcaacaaacaagggtagataaactaactcaaggtcgccagacttatgattggacattaaacagaaatcaaccggcagcaactgcattggccaacaccatagaagtctttagatcgaatggcctaacagctaatgagtcaggaaggctaatagatttcttaaaggatgtaatggaatcaatgaacaaagaggaaatagagataacaacccactttcaaagaaaaaggagagtaagagacaacatgaccaagaagatggtcacgcaaagaacaatagggaagaaaaaacaaagactgaataagagaggctatctaataagagcactgacattgaatacgatgaccaaagatgcagagagaggcaagttaaaaagaagggctatcgcaacacctgggatgcagattagaggtttcgtatactttgttgaaactttagctaggagcatttgcgaaaagcttgaacagtctgggctcccagtagggggcaatgaaaagaaggccaaactggcaaatgttgtgagaaagatgatgactaattcacaagacacagagatttctttcacaatcactggggacaacactaagtggaatgaaaaccaaaatcctcgaatgttcctggcgatgattacatatatcaccagaaatcaacccgagtggttcagaaacatcctgagcatggcacccataatgttctcaaacaaaatggcaagactagggaaagggtacatgttcgagagtaaaagaatgaagattcgaacgcaaataccagcagaaatgctagcaagcattgacctgaagtacttcaatgaatcaacaaagaagaaaattgagaaaataaggcctcttttaatagatggcacagcatcactgagtcctgggatgatgatgggcatgttcaacatgctaagtacggtcttgggagtctcgatactgaatcttggacaaaagaaatacaccaagacaatatactggtgggatgggctccaatcatccgacgattttgctctcatagtgaatgcaccaaaccatgagggaatacaagcaggagtggacagattttacaggacctgcaagttagtgggaatcaacatgagcaaaaagaagtcctatataaataagacagggacatttgaattcacaagctttttttatcgctatggatttgtggctaattttagcatggagctacccagctttggagtgtctggagtaaatgaatcagctgacatgagtattggagtaacagtgataaagaacaacatgataaacaatgaccttggacctgcaacggcccagatggctcttcaattgttcatcaaagactacagatacacatataggtgccataggggagacacacaaattcagacgagaagatcatttgagttaaagaagctgtgggatcaaacccaatcaaaggtagggctattagtatcagatggaggaccaaacttatacaatatacggaatcttcacattcctgaagtctgcttaaaatgggagctaatggatgatgactatcggggaagactttgtaatcccctgaatccctttgtaagtcataaagagattgattctgtaaacaatgctgtgataatgccagctcatggtccagccaaaagcatggaatatgatgccgttgcaactacacattcctggattcccaagaggaatcgttctattctcaacacaagccaaaggggaattcttgaggatgaacagatgtaccagaagtgctgcaatctattcgagaaatttttccctagcagttcatataggagaccggttggaatttctagcatggtggaggccatggtgtctagggcccggattgatgccagggtcgactttgagtctggacggatcaagaaagaagagttctctgagatcatgaagatctgttccaccattgaagaactcagacggcaaaaataa

>H1N1_PB1_JX625660_A/England/04640543/2010

atggatgtcaatccgactctacttttcctaaaaattccagcgcaaaatgccataagcaccacattcccttatactggagatcctccatacagccatggaacaggaacaggatacaccatggacacagtaaacagaacacaccaatactcagaaaagggaaagtggacgacaaacacagagactggtgcaccccagctcaacccgattgatggaccactacctgaggataatgaaccaagtgggtatgcacaaacagactgtgttctagaggctatggctttccttgaagaatcccacccaggaatatttgagaattcatgccttgaaacaatggaagttgttcaacaaacaagggtagataaactaactcaaggtcgccagacttatgattggacattaaacagaaatcaaccggcagcaactgcattggccaacaccatagaagtctttagatcgaatggcctaacagctaatgagtcaggaaggctaatagatttcttaaaggatgtaatggaatcaatgaacaaagaggaaatagagataacaacccactttcaaagaaaaaggagagtaagagacaacatgaccaagaagatggtcacgcaaagaacaatagggaagaaaaaacaaagactgaataagagaggctatctaataagagcactgacattaaatacgatgaccaaagatgcagagagaggcaagttaaaaagaagggctatcgcaacacctgggatgcagattagaggtttcgtatactttgttgaaactttagctaggagcatttgcgaaaagcttgaacagtctgggctcccagtagggggcaatgaaaagaaggccaaactggcaaatgttgtgagaaagatgatgactaattcacaagacacagagatttctttcacaatcactggggacaacactaagtggaatgaaaaccaaaatcctcgaatgttcctggcgatgattacatatatcaccagaaatcaacccgagtggttcagaaacatcctgagcatggcacccataatgttctcaaacaaaatggcaagactagggaaagggtacatgttcgagagtaaaagaatgaagattcgaacgcaaataccagcagaaatgctagcaagcattgacctgaagtacttcaatgaatcaacaaagaagaaaattgagaaaataaggcctcttttaatagatggcacagcatcactgagtcctgggatgatgatgggcatgttcaacatgctaagtacggtcttgggagtctcgatactgaatcttggacaaaagaaatacaccaagacaatatactggtgggatgggctccaatcatccgacgattttgctctcatagtgaatgcaccaaaccatgagggaatacaagcaggagtggacagattttacaggacctgcaagttagtgggaatcaacatgagcaaaaagaagtcctatataaataagacagggacatttgaattcacaagctttttttatcgctatggatttgtggctaattttagcatggagctacccagctttggagtgtctggagtaaatgaatcagctgacatgagtattggagtaacagtgataaagaacaacatgataaacaatgaccttggacctgcaacggcccagatggctcttcaattgttcatcaaagactacagatacacatataggtgccataggggagacacacaaattcagacgagaagatcatttgagttaaagaagctgtgggatcaaacccaatcaaaggtagggctattagtatcagatggaggaccaaacttatacaatatacggaatcttcacattcctgaagtctgcttaaaatgggagctaatggatgatgactatcggggaagactttgtaatcccctgaatccctttgtaagtcataaagagattgattctgtaaacaatgctgtgataatgccagctcatggtccagccaaaagcatggaatatgatgccgttgcaactacacattcctggattcccaagagaaatcgttctattctcaacacaagccaaaggggaattcttgaggatgaacagatgtaccagaagtgctgcaatctattcgagaaatttttccctagcagttcatataggagaccggttggaatttctagcatggtggaggccatggtgtctagggcccggattgatgccagggtcgactttgagtctggacggatcaagaaagaagagttctctgagatcatgaagatctgttccaccattgaagaactcagacggcaaaaataa

>H1N1_PB1_JX625558_A/England/94560024/2009

atggatgtcaatccgactctacttttcctaaaaattccagcgcaaaatgccataagcaccacattcccttatactggagatcctccatacagccatggaacaggaacaggatacaccatggacacagtaaacagaacacaccaatactcagaaaagggaaagtggacgacaaacacagagactggtgcaccccagctcaacccgattgatggaccactacctgaggataatgaaccaagtgggtatgcacaaacagactgtgttctagaggctatggctttccttgaagaatcccacccaggaatatttgagaattcatgccttgaaacaatggaagttgttcaacaaacaagggtagataaactaactcaaggtcgccagacttatgattggacattaaacagaaatcaaccggcagcaactgcattggccaacaccatagaagtctttagatcgaatggcctaacagctaatgagtcaggaaggctaatagatttcttaaaggatgtaatggaatcaatgaacaaagaggaaatagagataacaacccactttcaaagaaaaaggagagtaagagacaacatgaccaagaagatggtcacgcaaagaacaatagggaagaaaaaacaaagactgaataagagaggctatctaataagagcactgacattaaatacgatgaccaaagatgcagagagaggcaagttaaaaagaagggctatcgcaacacctgggatgcagattagaggtttcgtatactttgttgaaactttagctaggagcatttgcgaaaagcttgaacagtctgggctcccagtagggggcaatgaaaagaaggccaaactggcaaatgttgtgagaaagatgatgactaattcacaagacacagagatttctttcacaatcactggggacaacactaagtggaatgaaaatcaaaatcctcgaatgttcctggcgatgattacatatatcaccagaaatcaacccgagtggttcagaaacatcctgagcatggcacccataatgttctcaaacaaaatggcaagactagggaaagggtacatgttcgagagtaaaagaatgaagattcgaacacaaataccagcagaaatgctagcaagcattgacctgaagtacttcaatgaatcaacaaagaagaaaattgagaaaataaggcctcttttaatagatggcacagcatcactgagtcctgggatgatgatgggcatgttcaacatgctaagtacggtcttgggagtctcgatactgaatcttggacaaaagaaatacaccaagacaatatactggtgggatgggctccaatcatccgacgattttgctctcatagtgaatgcaccaaaccatgagggaatacaagcaggagtggacagattctacaggacctgcaagttagtgggaatcaacatgagcaaaaagaagtcctatataaataagacagggacatttgaattcacaagctttttttatcgctatggatttgtggctaattttagcatggagctacccagctttggagtgtctggagtaaatgaatcagctgacatgagtattggagtaacagtgataaagaacaacatgataaacaatgaccttggacctgcaacggcccagatggctcttcaattgttcatcaaagactacagatacacatataggtgccataggggagacacacaaattcagacgagaagatcatttgagttaaagaagctgtgggatcaaacccaatcaaaggtagggctattagtatcagatggaggaccaaacttatacaatatacggaatcttcacattcctgaagtctgcttaaaatgggagctaatggatgatgattatcggggaagactttgtaatcccctgaatccctttgtcagtcataaagagattgattctgtaaacaatgctgtggtaatgccagcccatggtccagccaaaagcatggaatatgatgccgttgcaactacacattcctggattcccaagaggaatcgttctattctcaacacaagccaaaggggaattcttgaggatgaacagatgtaccagaagtgctgcaatctattcgagaaatttttccctagcagttcatataggagaccggttggaatttctagcatggtggaggccatggtgtctagggcccggattgatgccagggtcgacttcgagtctggacggatcaagaaagaagagttctctgagatcatgaagatctgttccaccattgaagaactcagacggcaaaaataa

>H1N1_PB1_CY128169_A/Viet Nam/13032011/2009

atggatgtcaatccgactctacttttcctaaaaattccagcgcaaaatgccataagcaccacattcccttatactggagatcctccatacagccatggaacaggaacaggatacaccatggacacagtaaacagaacacaccaatactcagaaaagggaaagtggacgacaaacacagagactggtgcaccccagctcaacccgattgatggaccactacctgaggataatgaaccaagtgggtatgcacaaacagactgtgttctagaggctatggctttccttgaagaatcccacccaggaatatttgagaattcatgccttgaaacaatggaagttgttcaacaaacaagggtagataaactaactcaaggtcgccagacttatgattggacattaaacagaaatcaaccggcagcaactgcattggccaacaccatagaagtctttagatcgaatggcctaacagctaatgagtcaggaaggctaatagatttcttaaaggatgtaatggaatcaatgaacaaagaggaaatagagataacaacccactttcaaagaaaaaggagagtaagagacaacatgaccaagaagatggtcacgcaaagaacaatagggaagaaaaaacaaagactgaataagagaggctatctaataagagcactgacattaaatacgatgaccaaagatgcagagagaggcaagttaaaaagaagggctatcgcaacacctgggatgcagattagaggtttcgtatactttgttgaaactttagctaggagcatttgcgaaaagcttgaacagtctgggctcccagtagggggcaatgaaaagaaggccaaactggcaaatgttgtgagaaagatgatgactaattcacaagacacagagatttctttcacaatcactggggacaacactaagtggaatgaaaatcaaaatcctcgaatgttcctggcgatgattacatatatcaccagaaatcaacccgagtggttcagaaacatcctgagcatggcacccataatgttctcaaacaaaatggcaagactagggaaagggtacatgttcgagagtaaaagaatgaagattcgaacacaaataccagcagaaatgctagcaagcattgacctgaagtacttcaatgaatcaacaaagaagaaaattgagaaaataaggcctcttctaatagatggcacagcatcactgagtcctgggatgatgatgggcatgttcaacatgctaagtacggtcttgggagtctcgatactgaatcttggacaaaagaaatacaccaagacagtatactggtgggatgggctccaatcatccgacgattttgctctcatagtgaatgcaccaaaccatgagggaatacaagcaggagtggacagattctacaggacctgcaagttagtgggaatcaacatgagcaaaaagaagtcctatataaataagacagggacatttgaattcacaagctttttttatcgctatggatttgtggctaattttagcatggagctacccagctttggagtgtctggagtaaatgaatcagctgacatgagtattggagtaacagtgataaagaacaacatgataaacaatgaccttggacctgcaacggcccagatggctcttcaattgttcatcaaagactacagatacacatataggtgccataggggagacacacaaattcagacgagaagatcatttgagttaaagaagctgtgggatcaaacccaatcaaaggtagggctattagtatcagatggaggaccaaacttatacaatatacggaatcttcacattcctgaagtctgcttaaaatgggagctaatggatgatgattatcggggaagactttgtaatcccctgaatccctttgtcagtcataaagagattgattctgtaaacaatgctgtggtaatgccagcccatggtccagccaaaagcatggaatatgatgccgttgcaactacacattcctggattcccaagaggaatcgttctattctcaacacaagccaaaggggaattcttgaggatgaacagatgtaccagaagtgctgcaatctattcgagaaatttttccctagcagttcatataggagaccggttggaatttctagcatggtggaggccatggtatctagggcccggattgatgccagggtcgacttcgagtctggacggatcaagaaagaagagttctctgagatcatgaagatctgttccaccattgaagaactcagacggcaaaaataa

>H1N1_PB1_CY128377_A/Viet Nam/11032001/2009

atggatgtcaatccgactctacttttcctaaaaattccagcgcaaaatgccataagcaccacattcccttatactggagatcctccatacagccatggaacaggaacaggatacaccatggacacagtaaacagaacacaccaatactcagaaaagggaaagtggacgacaaacacagagactggtgcaccccagctcaacccgattgatggaccactacctgaggataatgaaccaagtgggtatgcacaaacagactgtgttctagaggctatggctttccttgaagaatcccacccaggaatatttgagaattcatgccttgaaacaatggaagttgttcaacaaacaagggtagataaactaactcaaggtcgccagacttatgattggacattaaacagaaatcaaccggcagcaactgcattggccaacaccatagaagtctttagatcgaatggcctaacagctaatgagtcaggaaggctaatagatttcttaaaggatgtaatggaatcaatgaacaaagaggaaatagagataacaacccactttcaaagaaaaaggagagtaagagacaacatgaccaagaagatggtcacgcaaagaacaatagggaagaaaaaacaaaaactgaataagagaggctatctaataagagcactgacattaaatacgatgaccaaagatgcagaaagaggcaagttaaaaagaagggctatcgcaacacctgggatgcagattagaggtttcgtatactttgttgaaactttagctaggagcatttgcgaaaagcttgaacagtctgggctcccagtagggggcaatgaaaagaaggccaaactggcaaatgttgtgagaaagatgatgactaattcacaagacacagagatttctttcacaatcactggggacaacactaagtggaatgaaaatcaaaatcctcgaatgttcctggcgatgattacatatatcaccagaagtcaacccgagtggttcagaaacatcctgagcatggcacccataatgttctcaaacaaaatggcaagactagggaaagggtacatgttcgagagtaaaagaatgaagattcgaacacaaataccagcagaaatgctagcaagcattgacctgaagtacttcaatgaatcaacaaagaagaaaattgagaaaataaggcctcttctaatagatggcacagcatcactgagtcctgggatgatgatgggcatgttcaacatgctaagtacggtcctgggagtctcgatactgaatcttggacaaaagaaatacaccaagacaatatactggtgggatgggctccaatcatccgacgattttgctctcatagtgaatgcaccaaaccatgagggaatacaagcaggagtggacagattctacaggacctgcaagttagtgggaatcaacatgagcaaaaagaagtcctatataaataagacagggacatttgaattcacaagctttttttatcgctatggatttgtggctaattttagcatggagctacccagctttggagtgtctggagtaaatgaatcagctgacatgagtattggagtaacagtgataaagaacaacatgataaacaatgaccttggacctgcaacggcccagatggctcttcaattgttcatcaaagactacagatacacatataggtgccataggggagacacacaaattcagacgagaagatcatttgagttaaagaagctgtgggatcaaacccaatcaaaggtagggctattagtatcagatggaggaccaaacttatacaatatacggaatcttcacattcctgaagtctgcttaaaatgggagctaatggatgatgattatcggggaagactttgtaatcccctgaatccctttgtcggtcataaagagattgattctgtaaacaatgctgtggtaatgccagcccatggtccagccaaaagcatggaatatgatgccgttgcaactacacattcctggattcccaagaggaatcgttctattctcaacacaagccaaaggggaattcttgaggatgaacagatgtaccagaagtgctgcaatctattcgagaaatttttccctagcagttcctataggagaccggttggaatttctagcatggtggaggccatggtgtctagggcccggattgatgccagggtcgacttcgagtctggacggatcaagaaagaagagttctctgagatcatgaagatctgttccaccattgaagaactcagacggcaaaaataa

>H1N1_PB1_CY128433_A/Viet Nam/12032005/2009

atggatgtcaatccgactctacttttcctaaaaattccagcgcaaaatgccataagcaccacattcccttatactggagatcctccatacagccatggaacaggaacaggatacaccatggacacagtaaacagaacacaccaatactcagaaaagggaaagtggacgacaaacacagagactggtgcaccccagctcaacccgattgatggaccactacctgaggataatgaaccaagtgggtatgcacaaacagactgtgttctagaggctatggctttccttgaagaatcccacccaggaatatttgagaattcatgccttgaaacaatggaagttgttcaacaaacaagggtagataaactaactcaaggtcgccagacttatgattggacattaaacagaaatcaaccggcagcaactgcattggccaacaccatagaagtctttagatcgaatggcctaacagctaatgagtcaggaaggctaatagatttcttaaaggatgtaatggaatcaatgaacaaagaggaaatagagataacaacccactttcaaagaaaaaggagagtaagagacaacatgaccaagaagatggtcacgcaaagaacaatagggaagaaaaaacaaagactgaataagagaggctatctaataagagcactgacattaaatacgatgaccaaagatgcagagagaggcaagttaaaaagaagggctatcgcaacacctgggatgcagattagaggtttcgtatactttgttgaaactttagctaggagcatttgcgaaaagcttgaacagtctgggctcccagtagggggcaatgaaaagaaggccaaactggcaaatgttgtgagaaaaatgatgactaattcacaagacacagagatttctttcacaatcactggggacaacactaagtggaatgaaaatcaaaatcctcgaatgttcctggcgatgattacatatatcaccagaaatcaacccgagtggttcagaaacatcctgagcatggcacccataatgttctcaaacaaaatggcaagactagggaaagggtacatgttcgagagtaaaagaatgaagattcgaacacaaataccagcagaaatgctagcaagcattgacctgaagtacttcaatgaatcaacaaagaagaaaattgagaaaataaggcctcttctaatagatggcacagcatcactgagtcctgggatgatgatgggcatgttcaacatgctaagtacggtcttgggagtctcgatactgaatcttggacaaaagaaatacaccaagacagtatactggtgggatgggctccaatcatccgacgattttgctctcatagtgaatgcaccaaaccatgagggaatacaagcaggagtggacagattctacaggacctgcaagttagtgggaatcaacatgagcaaaaagaagtcctatataaataagacagggacatttgaattcacaagctttttttatcgctatggatttgtggctaattttagcatggagctacccagctttggagtgtctggagtaaatgaatcagctgacatgagtattggagtaacagtgataaagaacaacatgataaacaatgaccttggacctgcaacggcccagatggctcttcaattgttcatcaaagactatagatacacatataggtgccataggggagacacacaaattcagacgagaagatcatttgagttaaagaagctgtgggatcaaacccagtcaaaggtagggctattagtatcagatggaggaccaaacttatacaatatacggaatcttcacattcctgaagtctgcttaaaatgggagctaatggatgatgattatcggggaagactttgtaatcccctgaatccctttgtcagtcataaagagattgattctgtaaacaatgctgtggtaatgccagcccatggtccagccaaaagcatggaatatgatgccgttgcaactacacattcctggattcccaagaggaatcgttctattctcaacacaagccaaaggggaattcttgaggatgaacagatgtatcagaagtgctgcaacctattcgagaaatttttccctagcagttcatataggagaccggttggaatttctagcatggtggaggccatggtgtctagggcccggattgatgccagggtcgacttcgagtctggacggatcaagaaagaagagttctctgagatcatgaagatctgttccaccattgaagaactcagacggcaaaaataa

>H1N1_PB1_CY100446_A/Mexico City/IBT22/2009

atggatgtcaatccgactctacttttcctaaaaattccagcgcaaaatgccataagcaccacattcccttatactggagatcctccatacagccatggaacaggaacaggatacaccatggacacagtaaacagaacacaccaatactcagaaaagggaaagtggacgacaaacacagagactggtgcaccccagctcaacccgattgatggaccactaccwgaggataatgaaccaagtgggtatgcacaaacagactgtgttctagaggctatggctttccttgaagaatcccacccaggaatatttgagaattcatgccttgaaacaatggaagttgttcaacaaacaagggtagataaactaactcaaggtcgccagacttatgattggacattaaacagaaatcaaccggcagcaactgcattggccaacaccatagaagtctttagatcgaatggcctaacagctaatgagtcaggaaggctaatagatttcttaaaggatgtaatggaatcaatgaacaaagaggaaatagagataacaacccactttcaaagaaaaaggagagtaagagacaacatgaccaagaagatggtcacgcaaagaacaatagggaagaaaaaacaaagactgaataagagaggctatctaataagagcactgacattaaatacgatgaccaaagatgcagagagaggcaagttaaaaagaagggctatcgcaacacctgggatgcagattagaggtttcgtatactttgttgaaactttagctaggagcatttgcgaaaagcttgaacagtctgggctcccagtagggggcaatgaaaagaaggccaaactggcaaatgttgtgagaaagatgatgactaattcacaagacacagagatttctttcacaatcactggggacaacactaagtggaatgaaaatcaaaatcctcgaatgttcctggcgatgattacatatatcaccagaaatcaacccgagtggttcagaaacatcctgagcatggcacccataatgttctcaaacaaaatggcaagactagggaaagggtacatgttcgagagtaaaagaatgaagattcgaacacaaataccagcagaaatgctagcaagcattgacctgaagtacttcaatgaatcaacaaagaagaaaattgagaaaataaggcctcttctaatagatggcacagcatcactgagtcctgggatgatgatgggcatgttcaacatgctaagtacggtcttgggagtctcgatactgaatcttggacaaaagaaatacaccaagacaatatactggtgggatgggctccaatcatccgacgattttgctctcatagtgaatgcaccaaaccatgagggaatamaagcaggagtggacagattctacaggacctgcaagttagtgggaatcaacatgagcaaaaagaagtcctatataaataagacagggacatttgaattcacaagctttttttatcgctatggatttgtggctaattttagcatggagctacccagctttggagtgtctggagtaaatgaatcagctgacatgagtattggagtaacagtgataaagaacaacatgataaacaatgaccttggacctgcaacggcccagatggctcttcaattgttcatcaaagactacagatacacatataggtgccataggggagacacacaaattcagacgaraagatcatttgagttaaagaagctgtgggatcaaacccaatcaaaggtagggctattagtatcagatggaggaccaaacttatacaatatacggaatcttcacattcctgaagtctgcttaaaatgggagctaatggatgatgattatcggggaagactttgtaatcccctgaatccctttgtcagtcataaagagattgattctgtaaacaatgctgtggtaatgccagcccatggtccagccaaaagcatggaatatgatgccgttgcaactacacattcttggattcccaagaggaatcgttctattctcaacacaagccaaaggggaattcttgaggatgaacagawgtaccagaagtgctgcaatctattcgagaaatttttccctagcagttcatataggagaccggttggaatttctagcatggtggaggccatggtgtctagggcccggattgatgccagggtcgacttcgagtctggacggatcaagaaagaagagttctctgagatcatgaagatctgttccaccattgaagaactcagacggcaaaaataa

>H1N1_PB1_CY129628_A/Munich/INS539/2011

atggatgtcaatccgactctacttttcctaaaaattccagcgcaaaatgccataagcaccacattcccttatactggagatcctccatacagccatggaacaggaacaggatacaccatggacacagtgaacagaacacaccaatactcagaaaagggaaagtggacgacaaacacagagactggtgcaccccagctcaacccgattgatggaccactacctgaggataatgaaccaagtgggtatgcacaaacagactgtgttctagaggctatggctttccttgaagaatcccacccaggaatatttgagaattcatgccttgaaacaatggaagttgttcaacaaacaagggtagataaactaactcaaggtcgccagacttatgattggacattaaacagaaatcaaccggcagcaactgcattggccaacaccatagaagtctttagatcgaatggcctaacagctaatgagtcaggcaggctaatagatttcttaaaagatgtaatggaatcaatgaacaaagaggaaatagagataacaacccactttcaaagaaaaaggagagtaagagacaacatgaccaagaagatggtcacgcaaagaacaatagggaagaaaaaacaaagactgaataagagaggctatctaataagagcactgacattaaatacgatgaccaaagatgcagagagaggcaagttaaaaagaagggctatcgcaacacctgggatgcagattagaggtttcgtatactttgttgaaactttagctaggagcatttgcgaaaagcttgaacagtctgggctcccagtagggggcaatgaaaagaaggccaaactggcaaatgttgtgagaaagatgatgactaattcacaagacacagagatttctttcacaatcactggggacaacactaagtggaatgaaaatcaaaatcctcgaatgttcctggcgatgattacatatatcaccagaaatcaacccgagtggttcagaaacatcctgagcatggcacccataatgttctcaaacaaaatggcaagactagggaaagggtacatgttcgagagtaaaagaatgaagattcgaacacaaataccagcagaaatgctagcaagcattgacctgaagtacttcaatgaatcaacaaagaagaaaattgagaaaataaggcctcttttaatagatggcacagcatcactgagtcctgggatgatgatgggcatgttcaacatgctaagtacggtcttgggagtctcgatactgaatcttggacaaaagaaatacaccaagacaatatactggtgggatgggctccaatcatccgacgattttgctctcatagtgaatgcaccaaaccatgagggaatacaagcaggagtggacagattctacaggacctgcaagttagtgggaatcaacatgagcaaaaagaagtcctatataaataagacagggacatttgaattcacaagctttttttatcgctatggatttgtggctaattttagcatggagctacccagctttggagtgtctggagtaaatgaatcagctgacatgagtattggagtaacagtgataaagaacaacatgataaacaatgaccttggacctgcaacggcccagatggctcttcaattgttcatcaaagactacagatacacatataggtgccataggggagacacacaaattcagacgagaagatcatttgagttaaagaagctgtgggatcaaacccaatcaaaggtagggctattagtatcagatggaggaccaaacttatacaatatacggaatcttcacattcctgaagtctgcttaaaatgggagctaatggatgatgattatcggggaagactttgtaatcccctgaatccctttgtaagtcataaagagattgattctgtaaacaatgctgtggtaatgccagcccatggtccagccaaaagcatggaatatgatgccgttgcaactacacattcctggattcccaagaggaatcgttctattctcaacacaagccaaaggggaattcttgaggatgaacagatgtaccagaagtgctgcaatctattcgagaaatttttccctagcagttcatataggagaccggttggaatttctagcatggtggaggccatggtgtctagggcccggattgatgccagggtcgactttgagtctggacggatcaagaaagaagagttctctgagatcatgaagatctgttccaccattgaagaactcagacggcaaaaataa

>H1N1_PB1_CY129868_A/Springfield/INS598/2011

atggatgtcaatccgactctacttttcctaaaaattccagcgcaaaatgccataagcaccacattcccttatactggagatcctccatacagccatggaacaggaacaggatacaccatggacacagtaaacagaacacaccaatactcagaaaagggaaagtggacgacaaacacagagactggtgcaccccagctcaacccgattgatggaccactacctgaggataatgaaccaagtgggtatgcacaaacagactgtgttctagaggctatggctttccttgaagaatcccacccaggaatatttgagaattcatgccttgaaacaatggaagttgttcaacaaacaagggtagataaactaactcaaggtcgccagacttatgattggacattaaacagaaatcaaccggcagcaactgcattggccaacaccatagaagtctttagatcaaatgacctaacagctaatgagtcaggaaggctaatagatttcttaaaggatgtaatggaatcaatgaacaaagaggaaatagagataacaacccactttcaaagaaaaaggagagtaagagacaacatgaccaagaagatggtcacgcaaagaacaatagggaagaaaaaacaaagactgaataagagaggctatctaataagagcactgacattaaatacgatgaccaaagatgcagagagaggcaagttaaaaagaagggctatcgcaacacctgggatgcagattagaggtttcgtatactttgttgaaactttagctaggagcatttgtgaaaagcttgaacagtctgggctcccagtagggggcaatgaaaagaaggccaaactggcaaatgttgtgagaaagatgatgactaattcacaagacacagagatttctttcacaatcactggggacaacactaagtggaatgaaaatcaaaatcctcgaatgttcctggcgatgattacatacatcaccagaaatcaacccgagtggttcagaaacatcctgagcatggcacccataatgttctcaaacaaaatggcaagactagggaaagggtacatgttcgagagtaaaagaatgaagattcgaacacaaataccagcagaaatgctagcaagcattgacctgaagtacttcaatgaatcaacaaagaagaaaattgagaaaataaggcctcttctaatagatggcacagcatcactgagtcctgggatgatgatgggcatgttcaacatgctaagtacggtcttgggagtctcgatactgaatcttggacaaaagaaatacaccaagacaatatactggtgggatgggctccaatcatccgacgattttgctctcatagtgaatgcaccaaaccatgagggaatacaagcaggagtggacagattctacaggacctgcaagttagtgggaatcaacatgagcaaaaagaagtcctatataaataagacagggacatttgaattcacaagctttttttatcgctatggatttgtggctaattttagcatggagctacccagctttggagtgtctggagtaaatgaatcagctgacatgagtattggagtaacagtgataaagaacaacatgataaacaatgaccttggacctgcaacggcccagatggcacttcaattgttcatcaaagactacagatacacatataggtgccataggggagacacacaaatccagacgagaagatcatttgagttaaagaagctgtgggatcaaacccaatcaaagggagggctattagtatcagatggaggaccaaacttatacaatatacggaatcttcacattcctgaagtctgcttaaaatgggagctaatggatgatgattatcggggaagactttgtaatcccctgaatccctttgtcagtcataaagagattgattctgtaaacaatgctgtggtaatgccagcccatggtccagtcaaaagcatggaatatgatgccgttgcaactacacattcctggattcccaagaggaatcgttctattctcaacacaagccaaaggggaattcttgaggatgaacagatgtaccagaagtgctgcaatctattcgagaaatttttccctagcagttcatataggagaccggttggaatttctagcatggtggaggccatggtgtctagggcccggattgatgccagggtcgacttcgaatctggacggatcaagaaagaagagttctctgagatcatgaagatctgctccaccattgaagaactcagacggcaaaaataa

>H1N1_PB1_CY130124_A/New Jersey/8/1976

atggacgtcaatccgactttgcttttcttgaaagtgccagcacaaaatgctataagcacaacgtttccttacactggagaccccccttacagccatgggacaggaacaggatacaccatggatactgtgaacagaacacatcagtactcagaaagaggaaaatggacaataaacaccgaaactggggcaccacaactaaatccaattgatggaccgctgccagaggacaatgaaccaactggttatgctcaaacagactgcgtattggaagcaatggcgttcctcgagaaatcccatcccggcatttttgagaactcatgtcttgaaacgatggaagttgttcagcaaacacgggtggacaaactaacgcaaggtcggcaaactttcgattggactctgaataggaaccagcctgctgcaacagcattggccaacacaatagaagtgttcagattgaacggcctcacggccagtgaatctggaagactcatagacttcctaaaggatgttatggaatcaatggacaaagaagaaatggaaatcgtaactcatttccagagaaaaaggagagtgagggacaatatgactaagaaaatggtgacgcaaagaacaataggtaaaaagaaacagaagttaaataaaagaagctatctaattagagcattgaccttgaacacaatgacaaaagatgctgaaagaggaaaattgaaaagaagagcaattgccaccccagggatgcaaatacggggattcgtatatttcgttgagacactagcgagaagtatttgtgaaaaacttgaacaatcaggactgccagttggaggcaatgaaaagaaagccaaattggcaaatgttgtacgaaaaatgatgaccaattctcaggacactgaactctcctttactatcactggagataataccaaatggaatgaaaatcagaatcctcgaatgtttttagctatgattacatacataaccagaaatcagcccgaatggttcagaaatgttctaagtgttgctccaataatgttctcgaacaagatggctagattgggtaaaggatacatgtttgagagtaaaaacatgaagcttaggactcagatacctgcagagatgctatcaggcattgacctgaggtattttaatgattcaactaggaaaaaaattgaaaaaatccgaccgctcttaatagagggagctgtttctttaagccctggaatgatgatgggtatgtttaacatgttaagcactgtgttgggtgtttccattcttaaccttggacaaaaggagtacacgaagactgcttactggtgggacggtctccaatcgtctgatgattttgctctgattgtgaacgcacccaattatgaagggattcaagcaggagttgacaggttttatcgaacctgtaagctactcggaattaatatgagcaaaaagaaatcttacataaatagaacaggcacttttgaattcacaagctttttctatcgttatggatttgttgccaatttcagcatggagcttcctagttttggagtgtctggaattaatgagtctgcagacatgagtattggagttactgtcatcaaaaacaacatgataaacaatgatcttggaccagcaacagcacaaatggctctccagttgttcatcaaagattaccgatacacgtatcgctgccacaggggtgatactcaaatacaaactcgaaggtcatttgaaataaagaaattgtgggatcaaacccgttctaaggctggattgttggtttccgatggaggccccaatttatataatatcaggaatctccacattcctgaagtctgcttgaagtgggaattaatggataaagattaccaggggcgattgtgcaatccgctaaacccatttgtcagccataaagagattgaatcagtgaataatgcagtagttatgccatcacatggtccagccaaagccatggaatacgacgctgttgccacaacgcactcctgggttcctaaaagaaatcgatccatcttgaacacaagccagagaggaatacttgaagacgagcaaatgtaccaaaaatgctgtaacctatttgaaaagttcttccctagcagctcatataggagaccagttgggatttccagtatggtggaggccatggtttccagggcccgaatcgatgcacgaattgattttgaatctggaaggataaagaaagaggatttcgctgagatcatgaagatctgttccaccattgaagatctcagacggcaaaagtag

>H1N1_PB1_CY134495_A/Boston/DOA30/2011

atggatgtcaatccgactctacttttcctaaaaattccagcgcaaaatgccataagcaccacattcccttatactggagatcctccatacagccatggaacaggaacaggatacaccatggacacagtaaacagaacacaccaatactcagaaaagggaaagtggacgacaaacacagagactggtgcaccccagctcaacccgattgatggaccactacctgaggataatgaaccaagtgggtatgcacaaacagactgtgttctagaggctatggctttccttgaagaatcccacccaggaatatttgagaattcatgccttgaaacaatggaagttgttcaacaaacaagggtagataaactgactcaaggtcgccagacttatgattggacattaaacagaaatcaaccggcagcaactgcattggccaacaccatagaagtctttagatcgaatgacctaacagctaatgagtcaggaaggctaatagatttcttaaaggatgtaatggaatcaatgaacaaagaggaaatagagataacaacccactttcaaagaaaaaggagagtaagagacaacatgaccaagaagatggtcacgcaaagaacaatagggaagaaaaaacagagactgaataagagaggctatctaataagagcactgacattaaatacgatgaccaaagatgcagagagaggcaagttaaaaagaagggctatcgcaacacctgggatgcagattagaggtttcgtatactttgttgaaactttagctaggagcatttgcgaaaagcttgaacagtctgggctcccagtagggggcaatgaaaagaaggccaaactggcaaatgttgtgagaaagatgatgactaattcacaagacacagagatttctttcacaatcactggggacaacactaagtggaatgaaaatcaaaatcctcgaatgttcctggcgatgattacatatatcaccagaaatcaacccgagtggtttagaaacatcctgagcatggcacccataatgttctcaaacaaaatggcaagactagggaaagggtacatgttcgagagtaaaagaatgaagattcgaacacaaataccagcagaaatgctagcaagcattgacctgaagtacttcaatgaatcaacaaagaagaaaattgagaaaataaggcctcttttaatggatggcacagcatcactgagccctgggatgatgatgggcatgttcaacatgctaagtacggtcttgggagtctcgatactgaatcttggacaaaagaaatacaccaagacaacatactggtgggatgggctccaatcatccgacgattttgctctcatagtgaatgcaccaaatcatgagggaatacaagcaggagtggacagattctacaggacctgcaagttagtgggaatcaacatgagcaaaaagaagtcctatataaataagacagggacatttgaattcacaagctttttttatcgctatggatttgtggctaattttagcatggagctacccagctttggagtgtctggagtaaatgaatcagctgacatgagtattggagtaacagtgataaagaacaacatgataaacaatgaccttggacctgcaacggcccagatggctcttcaattgttcatcaaagactacagatacacatataggtgccataggggagacacacaaattcagacgagaagatcatttgagttaaagaagctgtgggatcaaacccaatcaaaggtagggctattagtatcagatggaggaccaaacttatacaatatacggaatcttcacattcctgaagtctgcttaaaatgggagctaatggatgatgattatcggggaagactttgtaatcccctgaatccctttgtaagtcataaggagattgattctgtaaacaatgctgtggtaatgccagcccatggtccagccaaaagcatggaatatgatgccgttgcaactacacattcctggattcccaagaggaatcgttctattctcaacacaagccaaaggggaattcttgaggatgaacagatgtaccagaagtgctgcaatctattcgagaaatttttccctagcagttcatataggagaccggttggaatttctagcatggtggaggccatggtgtctagggcccggattgatgccagggtcgactttgagtctggacggatcaagaaagaagagttctctgagatcatgaagatctgttccaccattgaagaactcagacggcaaaaataa

>H1N1_PB1_CY134503_A/Boston/DOA34/2011

atggatgtcaacccgactctacttttcctgaaaattccagcgcaaaatgccataagcaccacattcccttatactggagatcctccatacagccatggaacaggaacaggatacaccatggacacagtaaacagaacacaccaatactcagaaaagggaaaatggacgacaaacacagagactggtgcaccccagctcaacccgattgatggaccactacctgaggataatgaaccaagtgggtatgcacaaacagactgtgttctagaggctatggctttccttgaagaatcccacccaggaatatttgagaattcatgccttgaaacaatggaagttgttcaacaaacaagggtagataaactaactcaaggtcgccagacttatgattggacattaaacagaaatcagccggcagcaactgcattggccaacaccatagaagtctttagatcgaatggcctaacagctaatgagtcaggaaggctaatagatttcttaaaggatgtaatggaatcaatgaacaaagaggaaatagagataacaacccactttcaaagaaaaaggagagtaagagacaacatgaccaagaagatggtcacgcaaagaacaatagggaagaaaaaacaaaaactaaataagagaggctatctaataagggcactgacattaaatacgatgaccaaagatgcagagagaggcaagttaaaaagaagggctatcgcaacacctgggatgcagattagaggtttcgtatactttgttgaaactttagctaggagcatttgcgaaaagcttgaacagtctgggctcccagtagggggcaatgaaaagaaggccaaactggcaaatgttgtgagaaagatgatgactaattcacaagacacagagatttctttcacaatcactggggacaacactaagtggaatgaaaatcaaaatcctcgaatgttcctggcgatgattacatatatcaccagaaatcaacccgaatggttcagaaacatcctgagcatggcacccataatgttctcaaacaaaatggcaagactagggaaagggtacatgttcgagagtaaaaaaatgaagattcgaacacaaataccagcagaaatgctagcaagcattgacctgaagtacttcaatgaatcaacaaagaagaaaattgagaaaataaggcctcttctaatagatggcacagcatcactgagtcctgggatgatgatgggcatgttcaacatgctaagtacggtcttgggagtctcgatactgaatcttggacaaaagaaatacaccaagacagtatactggtgggatgggctccaatcatccgatgattttgccctcatagtgaatgcaccaaaccatgagggaatacaagcaggagtggacagattctacaggacctgcaagttagtgggaattaacatgagcaaaaagaagtcctatataaataagacagggacatttgaattcacaagctttttttatcgctatggatttgtggctaattttagcatggagctacccagctttggagtgtctggagtaaatgaatcagctgacatgagtattggagtaacagtgataaagaacaacatgataaacaatgaccttggacctgcaacggcccagatggctcttcaattgttcatcaaagactacagatacacatataggtgccataggggagacacacaaattcagacgagaagatcatttgagttaaagaagctgtgggatcaaacccaatcaaaggtagggctattagtatcagatggaggaccaaacttatacaatatacggaatcttcacattcctgaagtctgcttaaaatgggagctaatggatgatgattatcggggaagactttgtaatcccctgaatccctttgtcagtcataaagagattgattctgtaaacaatgctgtggtaatgccagcccatggtccagtcaaaagcatggaatatgatgccgttgcaactacacattcctggattcccaagaggaatcgttctattctcaacacaagccaaaggggaattcttgaggatgaacagatgtaccagaagtgctgcaatctattcgagaaatttttccctagcagttcatataggagaccggttggaatttctagcatggtggaggccatggtgtctagggcccggattgatgccagggttgacttcgagtctggacggatcaagaaagaagagttctctgagatcatgaagatctgttccaccattgaagaactcagacggcaaaaataa

>H1N1_PB1_CY134575_A/Boston/DOA49/2011

atggatgtcaatccgactctacttttcctaaaaattccagcgcaaaatgccataagcaccacattcccttatactggagatcctccatacagccatggaacaggaacaggatacaccatggacacagtaaacagaacacaccaatactcagaaaagggaaagtggacgacaaacacagagactggtgcaccccagctcaacccgattgatggaccactacctgaggatgatgaaccaagtgggtatgcacaaacagactgtgttctagaggctatggctttccttgaagaatcccacccaggaatatttgagaattcatgccttgaaacaatggaagttgttcaacaaacaagggtagataaactaactcaaggtcgccagacttatgattggacattaaacagaaatcaaccggcagcaactgcattggccaacaccatagaagtctttagatcaaatgacctaacagctaatgagtcaggaaggctaatagatttcttaaaggatgtaatggaatcaatgaacaaagaggaaatagagataacaacccactttcaaagaaaaaggagagtaagagacaacatgaccaagaagatggtcacgcaaagaacaatagggaagaaaaaacaaagactgaataagagaggctatctaataagagcactgacattaaatacgatgaccaaagatgcagagagaggcaagttaaaaagaagggctatcgcaacacctgggatgcagattagaggtttcgtatactttgttgaaactttagctaggagcatttgcgaaaagcttgaacagtctgggctcccagtagggggcaatgaaaagaaggccaaactggcaaatgttgtgagaaagatgatgactaattcacaagacacagagatttctttcacaatcactggggacaacactaagtggaatgaaaatcaaaatcctcgaatgttcctggcgatgattacatacatcaccagaaatcaacccgagtggttcagaaacatcctgagcatggcacccataatgttctcaaacaaaatggcaagactagggaaagggtacatgttcgagagtaaaagaatgaagattcgaacacaaataccagcagaaatgctagcaagcattgacctgaagtacttcaatgaatcaacaaagaagaaaattgagaaaataaggcctcttctaatagatggcacagcatcactgagtcctgggatgatgatgggcatgttcaacatgctaagtacggtcttgggagtctcgatactgaatcttggacaaaagaaatacaccaagacaatatactggtgggatgggctccaatcatccgatgattttgctctcatagtgaatgcgccaaaccatgagggaatacaagcaggagtggacagattctacaggacctgcaagttagtgggaatcaacatgagcaaaaagaagtcctatataaataagacagggacatttgaattcacaagctttttttatcgctatggatttgtggctaattttagcatggagctacccagctttggagtgtctggagtaaatgaatcagctgacatgagtattggagtaacagtgataaagaacaacatgataaacaatgaccttggacctgcaacggcccagatggcacttcaattgttcatcaaagactacagatacacatataggtgccataggggagacacacaaatccagacgagaagatcatttgagttaaagaagctgtgggatcaaacccaatcaaagggagggctattagtatcagatggaggaccaaacttatacaatatacggaatcttcacgttcctgaagtctgcttaaaatgggagctaatggatgatgattatcggggaagactttgtaatcccctgaatccctttgtcagtcataaagagattgattctgtaaacaatgctgtggtaatgccagcccatggtccagtcaaaagcatggaatatgatgccgttgcaactacacattcctggattcccaagaggaatcgttctattctcaacacaagccaaaggggaattcttgaggatgaacagatgtaccagaagtgctgcaatctattcgagaaatttttccctagcagttcatataggagaccggttggaatttctagcatggtggaggccatggtgtctagggcccggattgatgccagggtcgacttcgaatctggacggatcaagaaagaagagttctctgagatcatgaagatctgctccaccattgaagaactcagacggcaaaaataa

>H1N1_PB1_CY135122_A/Texas/JMM_53/2012

atggatgtcaatccgactctacttttcctaaaaattccagcacaaaatgccataagcaccacattcccttatactggagatcctccatacagccatggaacaggaacaggatacaccatggacacagtaaacagaacacaccaatactcagaaaagggaaagtggacaacaaacacagagactggtgcaccccagctcaacccgattgatggaccactacctgaggataatgaaccaagtgggtatgcacaaacagactgtgttctagaggctatggctttccttgaagaatcccacccaggaatatttgagaattcatgccttgaaacaatggaagttgttcaacaaacaagagtagataaactgactcaaggtcgccagacttatgattggacattaaacagaaatcaaccggcagcaactgcattggccaacaccatagaagtctttagatcgaatgacctaacagctaacgagtcaggaaggctaatagatttcttaaaggatgtaatggaatcaatgaacaaagaggaaatagagataacaacccactttcaaagaaaaaggagagtaagagacaacatgaccaaaaagatggtcacgcaaagaacaatagggaagaaaaaacagagactgaataagagaggctatctaataagagcactgacattaaatacgatgaccaaagatgcagagagaggcaagttaaaaagaagggctatcgcaacacctgggatgcagattagaggtttcgtatactttgttgaaactttagctaggagcatttgcgaaaagcttgaacagtctggactcccagtagggggcaatgaaaagaaggccaaactggcaaatgttgtgagaaagatgatgactaattcacaagatacagagatttctttcacaatcactggggacaacactaagtggaatgaaaatcaaaatcctcgaatgttcctggcgatgattacatatatcaccagaaatcaacccgagtggtttagaaacatcctgagcatggcacccataatgttctcaaataaaatggcaagactagggaaagggtacatgttcgagagtaaaagaatgaagattcgaacacaaataccagcagaaatgctagcaagcattgatctgaagtacttcaatgaatcaacaaagaagaaaattgagaaaataaggcctcttttaatggatggcacagcatcactgagtcctgggatgatgatgggcatgttcaacatgctaagtacggtcttgggagtctcgatactgaatcttggacaaaagaaatacaccaagacaacatactggtgggatgggctccaatcatccgacgattttgctctcatagtgaatgcaccaaaccatgagggaatacaagcaggagtggacagattctacaggacctgcaagttagtgggaatcaacatgagcaaaaagaagtcctatataaataagacagggacatttgaattcacaagctttttttatcgctatggatttgtggctaattttagcatggagctacccagctttggagtgtctggagtaaatgaatcagctgacatgagtattggagtaacagtgataaagaacaacatgataaacaatgaccttggacctgcaacggcccagatggctcttcaattgttcatcaaagactacagatacacatataggtgccataggggagacacacaaattcagacgagaagatcatttgagttaaagaagctgtgggatcaaacccaatcaaaggtagggctattagtatcagatggaggaccaaacttatacaatatacggaatcttcacattcctgaagtctgcttaaaatgggagctaatggatgatgattatcggggaagactttgtaatcccctgaatccctttgtaagtcataaggagattgattctgtaaacaatgctgtggtaatgccagcccatggtccagccaaaagcatggaatatgatgccgttgcaactacacattcctggattcccaagaggaatcgttctattctcaacacaagccaaaggggaattcttgaggatgaacagatgtaccagaagtgttgcaatctattcgagaaatttttccctagcagttcatataggagaccggttggaatttctagcatggtggaggccatggtatctagggccaggattgatgccagggtcgactttgagtctggacggatcaagaaagaagagttctcagagatcatgaagatctgttccaccattgaagaactcagacggcaaaaataa

>H1N1_PB1_KC620384_A/Giessen/6/2009

atggatgtcaatccgactctacttttcctaaaaattccagcgcaaaatgccataagcaccacattcccttatactggagatcctccatacagccatggaacaggaacaggatacaccatggacacagtaaacagaacacaccaatactcagaaaagggaaagtggacgacaaacacagagactggtgcaccccaactcaacccgattgatggaccactacctgaggataatgaaccaagtgggtatgcacaaacagactgtgttctagaggctatggctttccttgaagaatcccacccaggaatatttgagaattcatgccttgaaacaatggaagttgttcaacaaacaagggtagataaactaactcaaggtcgccagacttatgattggacattaaacagaaatcaaccggcagcaactgcattggccaacaccatagaagtctttagatcgaatggcctaacagctaatgagtcaggaaggctaatagatttcttaaaggatgtaatggaatcaatgaacaaagaggaaatagagataacaacccactttcaaagaaaaaggagagtaagagacaacatgaccaagaagatggtcacgcaaagaacaatagggaagaaaaaacaaagactgaataagagaggctatctaataagagcactgacattaaatacgatgaccaaagatgcagagagaggcaagttaaaaagaagggctatcgcaacacctgggatgcagattagaggtttcgtatactttgttgaaactttagctaggagcatttgcgaaaagcttgaacagtctgggctcccagtagggggcaatgaaaagaaggccaaactggcaaatgttgtgagaaagatgatgactaattcacaagacacagagatttctttcacaatcactggagacaacactaagtggaatgaaaatcaaaatcctcgaatgttcctggcgatgattacatatatcaccagaaatcaacccgagtggttcagaaacatcctgagcatggcacccataatgttctcaaacaaaatggcaagactagggaaagggtacatgttcgagagtaaaagaatgaagattcgaacacaaataccagcagaaatgctagcaagcattgacctgaagtacttcaatgaatcaacaaagaagaaaattgagaaaataaggcctcttctaatagatggcacagcatcactgagtcctgggatgatgatgggcatgttcaacatgctaagtacggtcttgggagtctcgatactgaatcttggacaaaagaaatacaccaagacaatatactggtgggatgggctccaatcatccgacgattttgctctcatagtgaatgcaccaaaccatgagggaatacaagcaggagtggacagattctacaggacctgcaagttagtgggaatcaacatgagcaaaaagaagtcctatataaataagacagggacatttgaattcacaagctttttttatcgctatggatttgtggctaattttagcatggagctacccagctttggagtgtctggagtaaatgaatcagctgacatgagtattggagtaacagtgataaagaacaacatgataaacaatgaccttggacctgcaacggcccagatggctcttcaattgttcatcaaagactacagatacacatataggtgccataggggagacacacaaattcagacgagaagatcatttgagttaaagaagctgtgggatcaaacccaatcaaaggtagggctattagtatcagatggaggaccaaacttatacaatatacggaatcttcacattcctgaagtctgcttaaaatgggagctaatggatgatgattatcggggaagactttgtaatcccctgaatccctttgtcagtcataaagagattgattctgtaaacaatgctgtggtaatgccagcccatggtccagccaaaagcatggaatatgatgccgttgcaactacacattcctggattcccaagaggaatcgttctattctcaacacaagccaaaggggaattcttgaggatgaacagatgtaccagaagtgctgcaatctattcgagaaatttttccctagcagttcatataggagaccggttggaatttctagcatggtggaggccatggtgtctagggcccggattgatgccagggtcgacttcgagtctggacggatcaagaaagaagagttctctgagatcatgaagatctgttccaccattgaagaactcagacggcaaaaataa

>H1N1_PB1_KC780032_A/Wisconsin/67/2009

atggatgtcaatccgactctacttttcctaaaaattccagcgcaaaatgccataagcaccacattcccttatactggagatcctccatacagccatggaacaggaacaggatacaccatggacacagtaaacagaacacaccaatactcagaaaagggaaagtggacgacaaacacagagactggtgcaccccagctcaacccgattgatggaccactacctgaggataatgaaccaagtgggtatgcacaaacagactgtgttctagaggctatggctttccttgaagaatcccacccaggaatatttgagaattcatgccttgaaacaatggaagttgttcaacaaacaagggtagataaactaactcaaggtcgccagacttatgattggacattaaacagaaatcaaccggcagcaactgcattggccaacaccatagaagtctttagatcgaatggcctaacagctaatgagtcaggaaggctaatagatttcttaaaggatgtaatggaatcaatgaacaaagaggaaatagagataacaacccactttcaaagaaaaaggagagtaagagacaacatgaccaagaagatggtcacgcaaagaacaatagggaagaaaaaacaaagactgaataagagaggctatctaataagagcactgacattaaatacgatgaccaaagatgcagagagaggcaagttaaaaagaagggctatcgcaacacctgggatgcagattagaggtttcgtatactttgttgaaactttagctaggagcatttgcgaaaagcttgaacagtctgggctcccagtagggggcaatgaaaagaaggccaaactggcaaatgttgtgagaaagatgatgactaattcacaagacacagagatttctttcacaatcactggggacaacactaagtggaatgaaaatcaaaatcctcgaatgttcctggcgatgattacatatatcaccagagatcaacccgagtggttcagaaacatcctgagcatggcacccataatgttctcaaacaaaatggcaagactagggaaagggtacatgttcgagagtaaaagaatgaagattcgaacacaaataccagcagaaatgctagcaagcattgacctgaagtacttcaatgaatcaacaaagaagaaaattgagaaaataaggcctcttctaatagatggcacagcatcactgagtcctgggatgatgatgggcatgttcaacatgctaagtacggtcttgggagtctcgatactgaatcttggacaaaagaaatacaccaagacaatatactggtgggatgggctccaatcatccgacgattttgctctcatagtgaatgcaccaaaccatgagggaatacaagcaggagtggacagattctacaggacctgcaagttagtgggaatcaacatgagcaaaaagaagtcctatataaataagacagggacatttgaattcacaagctttttttatcgctatggatttgtggctaattttagcatggagctacccagctttggagtgtctggagtaaatgaatcagctgacatgagtattggagtaacagtgataaagaacaacatgataaacaatgaccttggacctgcaacggcccagatggctcttcaattgttcatcaaagactacagatacacatataggtgccataggggagacacacaaattcagacgagaagatcatttgagttaaagaagctatgggatcaaacccaatcaaaggtagggctattagtatcagatggaggaccaaacttatacaatatacggaatcttcacattcctgaagtctgcttaaaatgggagctaatggatgatgattatcggggaagactttgtaaccccctgaatccctttgtcagtcataaagagattgattctgtaaacaatgctgtggtaatgccagcccatggtccagccaaaagcatggaatatgatgccgttgcaactacacattcctggattcccaagaggaatcgttctattctcaacacaagccaaaggggaattcttgaggatgaacagatgtaccagaagtgctgcaatctattcgagaaatttttccctagcagttcatataggagaccggttggaatttctagcatggtggaggccatggtgtctagggcccggattgatgccagggtcgacttcgagtctggacggatcaagaaagaagagttctctgagatcatgaagatctgttccaccattgaagaactcagacggcaaaaataa

>H1N1_PB1_KC780339_A/New York/102/2009

atggatgtcaatccgactctacttttcctaaaaattccagcgcaaaatgccataagcaccacattcccttatactggagatcctccatacagccatggaacaggaacaggatacaccatggacacagtaaacagaacacaccaatactcagaaaagggaaagtggacgacaaacacagagactggtgcaccccagctcaacccgattgatggaccactacctgaggataatgaaccaagtgggtatgcacaaacagactgtgttctagaggctatggctttccttgaagaatcccacccaggaatatttgagaattcatgccttgaaacaatagaagttgttcaacaaacaagggtagataaactaactcaaggtcgccagacttatgattggacattaaacagaaatcaaccggcagcaactgcattggccaacaccatagaagtctttagatcgaatggcctaacagctaatgagtcaggaaggctaatagatttcttaaaggatgtaatggaatcaatgaacaaagaggaaatagagataacaacccactttcaaagaaaaaggagagtaagagacaacatgaccaagaagatggtcacgcaaagaacaatagggaagaaaaaacaaagactgaataagagaggctatctaataagagcactgacattgaatacgatgaccaaagatgcagagagaggcaagttaaaaagaagggctatcgcaacacctgggatgcagattagaggtttcgtatactttgttgaaactttagctaggagcatttgcgaaaagcttgaacagtctgggctcccagtagggggcaatgaaaagaaggccaaactggcaaatgttgtgagaaagatgatgactaattcacaagacacagagatttctttcacaatcactggggacaacactaagtggaatgaaaatcaaaatcctcgaatgttcctggcgatgattacatatatcaccagaaatcaacccgagtggttcagaaacatcctgagcatggcacccataatgttctcaaacaaaatggcaagactagggaaagggtacatgttcgagagtaaaagaatgaagattcgaacacaaataccagcagaaatgctagcaagcattgacctgaagtacttcaatgaatcaacaaagaagaaaattgagaaaataaggcctcttctaatagatggcacagcatcactgagtcctgggatgatgatgggcatgttcaacatgctaagtacggtcttgggagtctcgatactgaatcttggacaaaagaaatacaccaagacaatatactggtgggatgggctccaatcatccgacgattttgctctcatagtgaatgcaccaaaccatgagggaatacaagcaggagtggacagattctacaggacctgcaagttagtgggaatcaacatgagcaaaaagaagtcctatataaataagacagggacatttgaattcacaagctttttttatcgctatggatttgtggctaattttagcatggagctacccagctttggagtgtctggagtaaatgaatcagctgacatgagtattggagtaacagtgataaagaacaacatgataaacaatgaccttggacctgcaacggcccagatggctcttcaattgttcatcaaagactacagatacacatataggtgccataggggagacacacaaattcagacgagaagatcatttgagttaaagaagctgtgggatcaaacccaatcaaaggtagggctattagtatcagatggaggaccaaacttatacaatatacggaatcttcacattcctgaagtctgcttaaaatgggagctaatggatgattattatcggggaagactttgtaatcccctgaatccctttgtcagtcataaagagattgattctgtaaacaatgctgtggtaatgccagcccatggtccagccaaaagcatggaatatgatgccgttgcaactacacattcctggattcccaagaggaatcgttctattctcaacacaagccaaaggggaattcttgaggatgaacagatgtaccagaagtgctgcaatctattcgagaaatttttccctagcagttcatataggagaccggttggaatttctagcatggtggaggccatggtgtctagggcccggattgatgccagggtcgacttcgagtctggacggatcaagaaagaagagttctctgagatcatgaagatctgttccaccattgaagaactcagacggcaaaaataa

>H1N1_PB1_KC781507_A/Utah/22/2009

atggatgtcaatccgactctacttttcctaaaaattccagcgcaaaatgccataagcaccacattcccttatactggagatcctccatacagccatggaacaggaacaggatacaccatggacacagtaaacagaacacaccaatactcagaaaagggaaagtggacgacaaacacagagactggtgcaccccagctcaacccgattgatggaccactacctgaggataatgaaccaagtgggtatgcacaaacagactgtgttctagaggctatggctttccttgaagaatcccacccaggaatatttgagaattcatgccttgaaacaatggaagttgttcaacaaacaagggtagataaactaactcaaggtcgccagacttatgattggacattaaacagaaatcaaccggcagcaactgcattggccaacaccatagaagtctttagatcgaatggcctaacagctaatgagtcaggaaggctaatagatttcttaaaggatgtaatggaatcaatgaacaaagaggaaatagagataacaacccactttcaaagaaaaaggagagtaagagacaacatgaccaagaagatggtcacgcaaagaacaatagggaagaaaaaacaaagactgaataagagaggctatctaataagagcactgacattaaatacgatgaccaaagatgcagagagaggcaagttaaaaagaagggctatcgcaacacctgggatgcagattagaggtttcgtatactttgttgaaactttagctaggagcatttgcgaaaagcttgaacagtctgggctcccagtagggggcaatgaaaagaaggccaaactggcaaatgttgtgagaaagatgatgactaattcacaagacacagagatttctttcacaatcactggggacaacactaagtggaatgaaaatcaaaatcctcgaatgttcctggcgatgattacatatatcaccagaaatcaacccgagtggttcagaaacatcctgagcatggcacccataatgttctcaaacaaaatggcaagactagggaaagggtacatgttcgagagtaaaagaatgaagattcgaacacaaataccagcagaaatgctagcaagcattgacctgaagtacttcaatgaatcaacaaagaagaaaattgagaaaataaggcctcttctaatagatggcacagcatcactgagtcctgggatgatgatgggcatgttcaacatgctaagtacggtcttgggagtctcgatactgaatcttggacaaaagaaatacaccaagacaatatattggtgggatgggctccaatcatccgacgattttgctctcatagtgaatgcaccaaaccatgagggaatacaagcaggagtggacagattctacaggacctgcaagttagtgggaatcaacatgagcaaaaagaagtcctatataaataagacagggacatttgaattcacaagctttttttatcgctatggatttgtggctaattttagcatggagctacccagctttggagtgtctggagtaaatgaatcagctgacatgagtattggagtaacagtgataaagaacaacatgataaacaatgaccttggacctgcaacggcccagatggctcttcaattgttcatcaaagactacagatacacatataggtgccataggggggacacacaaattcagacgagaagatcatttgagttaaagaagctgtgggatcaaacccaatcaaaggtagggctattagtatcagatggaggaccaaacttatacaatatacggaatcttcacattcctgaagtctgcttaaaatgggagctaatggatgatgattatcggggaagactttgtaatcccctgaatcccttggtcagtcataaagagattgattctgtaaacaatgctgtggtaatgccagcccatggtccagccaaaagcatggaatatgatgccgttgcaactacacattcctggattcccaagaggaatcgttctattctcaacacaagccaaaggggaattcttgaggatgaacagatgtaccagaagtgctgcaatctattcgagaaatttttccctagcagttcatataggagaccggttggaatttctagcatggtggaggccatggtgtctagggcccggattgatgccagggtcgacttcgagtctggacggatcaagaaagaagagttctctgagatcatgaagatctgttccaccattgaagaactcagacggcaaaaataa

>H1N1_PB1_KC781715_A/New York/98/2009

atggatgtcaatccgactctacttttcctaaaaattccagcgcaaaatgccataagcaccacattcccttatactggagatcctccatacagccatggaacaggaacaggatacaccatggacacagtaaatagaacacaccaatactcagaaaagggaaagtggacgacaaacacagagactggtgcaccccagctcaacccgattgatggaccactacctgaggataatgaaccaagtgggtatgcacaaacagactgtgttctagaggctatggctttccttgaagaatcccacccaggaatatttgagaattcatgccttgaaacaatggaagttgttcaacaaacaagggtagataaactaactcaaggtcgccagacttatgattggacattaaacagaaatcaaccggcagcaactgcattggccaacaccatagaagtctttagatcgaatggcctaacagctaatgagtcaggaaggctaatagatttcttaaaggatgtaatggaatcaatgaacaaagaggaaatagagataacaacccactttcaaagaaaaaggagagtaagagacaacatgaccaagaagatggtcacgcaaagaacaatagggaagaaaaaacaaagactgaataagagaggctatctaataagagcactgacattaaatacgatgaccaaagatgcagagagaggcaagttaaaaagaagggctatcgcaacacctgggatgcagattagaggtttcgtatactttgttgaaactttagctaggagcatttgcgaaaagcttgaacagtctgggctcccagtagggggcaatgaaaagaaggccaaactggcaaatgttgtgagaaagatgatgactaattcacaagacacagagatttctttcacaatcactggggacaacactaagtggaatgaaaatcaaaatcctcgaatgttcctggcgatgattacatatatcaccagaaatcaacccgagtggttcagaaacatcctgagcatggcacccataatgttctcaaacaaaatggcaagactagggaaagggtacatgttcgagagtaaaagaatgaagattcgaacacaaataccagcagaaatgctagcaagcattgacctgaagtacttcaatgaatcaacaaagaagaaaattgagaaaataaggcctcttctaatagatggcacagcatcactgagtcctgggatgatgatgggcatgttcaacatgctaagtacggtcttgggagtctcgatactgaatcttggacaaaagaaatacaccaagacaatatactggtgggatgggctccaatcatccgacgattttgctctcatagtgaatgcaccaaaccatgagggaatacaagcaggagtggacagattctacaggacctgcaagttagtgggaatcaacatgagcaaaaagaagtcctatataaataagacagggacatttgaattcacaagctttttttatcgctatggatttgtggctaattttagcatggagctacccagctttggagtgtctggagtaaatgaatcagctgacatgagtattggagtaacagtgataaagaacaacatgataaacaatgaccttggacctgcaacggcccagatggctcttcaattgttcatcaaagactacagatacacatataggtgccataggggagacacacaaattcagacgagaagatcatttgagttaaagaagctgtgggatcaaacccaatcaaaggtagggctattagtatcagatggaggaccaaacttatacaatatacggaatcttcacattcctgaagtctgcttaaaatgggagctaatggatgatgattatcggggaagactttgtaatcccctgaatccctttgtcagtcataaagagattgattctgtaaacaatgctgtggtaatgccagcccatggtccagccaaaagcatggaatatgatgccgttgcaactacacattcctggattcccaagaggaatcgttctattctcaacacaagccaaaggggaattcttgaggatgaacagatgtaccagaagtgctgcaatctattcgagaaatttttccctagcagttcatataggagaccggttggaatttctagcatggtggaggccatggtgtctagggcccggattgatgccagggtcgacttcgagtctggacggatcaagaaagaagagttctctgagatcatgaagatctgttccaccattgaagaactcagacggcaaaaataa

>H1N1_PB1_KC782247_A/Minnesota/40/2009

atggatgtcaatccgactctacttttcctaaaaattccagcgcaaaatgccataagcaccacattcccttatactggagatcctccatacagccatggaacaggaacaggatacaccatggacacagtaaacagaacacaccaatactcagaaaagggaaagtggacgacaaacacagagactggtgcaccccagctcaacccgattgatggaccactacctgaggataatgaaccaagtgggtatgcacaaacagactgtgttctagaggctatggctttccttgaagaatcccacccaggaatatttgagaattcatgccttgaaacaatggaagttgttcaacaaacaagggtagataaactaactcaaggtcgccagacttatgattggacattaaacagaaatcaaccggcagcaactgcattggccaacaccatagaagtctttagatcgaatggcctaacagctaatgagtcaggaaggctaatagatttcttaaaggatgtaatggaatcaatgaacaaagaggaaatagagataacaacccactttcaaagaaaaaggagagtaagagacaacatgaccaagaagatggtcacgcaaagaacaatagggaagaaaaaacaaagactgaataagagaggctatctaataagagcactgacattaaatacgatgaccaaagatgcagagagaggcaagttaaaaagaagggctatcgcaacacctgggatgcagattagaggtttcgtatacttcgttgaaactttagctaggagcatttgcgaaaagcttgaacagtctgggctcccagtagggggcaatgaaaagaaggccaaactggcaaatgttgtgagaaagatgatgactaattcacaagacacagagatttctttcacaatcactggggacaacactaagtggaatgaaaatcaaaatcctcgaatgttcctggcgatgattacatatatcaccagaaatcaacccgagtggttcagaaacatcctgagcatggcacccataatgttctcaaacaaaatggcaaggctagggaaagggtacatgttcgagagtaaaagaatgaagattcgaacacaaataccagcagaaatgctagcaagcattgacctgaagtacttcaatgaatcaacaaagaagaaaattgagaaaataaggcctcttctaatagatggcacagcatcactgagtcctgggatgatgatgggcatgttcaacatgctaagtacggtcttgggagtctcgatactgaatcttggacaaaagaaatacaccaagacagtatactggtgggatgggctccaatcatccgacgattttgctctcatagtgaatgcaccaaaccatgagggaatacaagcaggagtggacagattctacaggacctgcaagttagtgggaatcaacatgagcaaaaagaagtcctatataaataagacagggacatttgaattcacaagctttttttatcgctatggatttgtggctaattttagcatggagctacccagctttggagtgtctggagtaaatgaatcagctgacatgagtattggagtaacagtgataaagaacaacatgataaacaatgaccttggacctgcaacggcccagatggctcttcaattgttcatcaaagactacagatacacatataggtgccataggggagacacacaaattcagacgagaagatcacttgagttaaagaagctgtgggatcaaacccaatcaaaggtagggctattagtatcagatggaggaccaaacttatacaatatacggaatcttcacattcctgaagtctgcttaaaatgggagctaatggatgatgattatcggggaagactttgtaatcccctgaatccctttgtcagtcataaagagattgattctgtaaacaatgctgtggtaatgccagcccatggtccagccaaaagcatggaatatgatgccgttgcaactacacattcctggattcccaagaggaatcgttctattctcaacacaagccaaaggggaattcttgaggatgaacagatgtaccagaagtgctgcaatctattcgagaaatttttccctagcagttcatataggagaccggttggaatttctagcatggtggaggccatggtgtctagggcccggattgatgccagggtcgacttcgagtctggacggatcaagaaagaagagttctctgagatcatgaagatctgttccaccattgaagaactcagacggcaaaaataa

>H1N1_PB1_KC881913_A/New Mexico/04/2011

atggatgtcaatccgactctacttttcctaaaaattccagcgcaaaatgccataagcaccacattcccttatactggagatcctccatacagccatggaacaggaacaggatacaccatggacacagtaaacagaacacaccaatactcagaaaagggaaagtggacgacaaacacggagactggtgcaccccagctcaacccgattgatggaccactacctgaggataatgaaccaagtgggtatgcacaaacagactgtgttctagaggctatggctttcctagaagaatcccacccaggaatatttgagaattcatgccttgaaacaatggaagttgttcaacaaacaagggtagataaactaactcaaggtcgccagacttatgattggacattaaacagaaatcaaccggcagcaactgcattggccaacaccatagaagtctttagatcgaatggcctaacagctaatgagtcaggaaggctaatagatttcttaaaggatgtaatggaatcaatgaacaaagaggaaatagagataacaacccactttcaaagaaaaaggagagtaagagacaacatgaccaagaagatggtcacgcaaagaacaataggaaagaaaaaacaaagactgaataagagaggctatctaataagagcactgacattaaatacgatgaccaaagatgcagagagaggcaagttaaaaagaagggctatcgcaacacctgggatgcagattagaggtttcgtatactttgttgaaactttagctaggagcatttgcgaaaagcttgaacagtctgggctcccagtagggggcaatgaaaagaaggccaaactggcaaatgttgtgagaaagatgatgactaattcacaagacacagagatttctttcacaatcactggggacaacactaagtggaatgaaaatcaaaatcctcgaatgttcctggcgatgattacatatatcaccagaaatcaacccgagtggttcagaaacatcctgagcatggcacccataatgttctcaaacaaaatggcaagactagggaaagggtacatgttcgagagtaaaagaatgaagattcgaacacaaataccagcagaaatgctagcaagcattgacctgaagtacttcaatgaatcaacaaagaagaaaattgagaaaataaggcctcttctaatagatggcacagcatcactgagtcctgggatgatgatgggcatgttcaacatgctaagtacggtcttgggagtctcgatactgaatcttggacaaaagaaatacaccaagacaatatactggtgggatgggctccaatcatccgacgattttgctctcatagtgaatgcaccaaaccatgagggaatacaagcaggagtggacagattctacaggacctgcaagttagtgggaatcaacatgagcaaaaagaagtcctatataaataagacagggacatttgaattcacaagctttttttatcgctatggatttgtggctaattttagcatggagctacccagctttggggtgtctggagtaaatgaatcagctgacatgagtattggagtaacagtaataaagaacaacatgataaacaatgaccttggacctgcaacggcccagatggctcttcaattgttcatcaaagactacagatacacatataggtgccataggggagacacacaaattcagacgagaagatcatttgagttaaagaagctgtgggatcaaacccaatcaaagatagggctattagtatcagatggaggaccaaacttatacaacatacggaatcttcacattcctgaagtctgcttaaaatgggagctaatggatgatgattatcggggaagactttgtaatcccctgaatccctttgtcagtcataaagagattgattctgtaaacaatgctgtggtaatgccagcccatggtccagccaaaagcatggaatatgatgccgttgcaactacacattcctggattcccaagaggaatcgttctattctcaacacaagccaaaggggaattcttgaggatgaacagatgtaccagaagtgctgcaatctattcgagaaatttttccctagcagttcatataggagaccggttggaatttctagcatggtggaggccatggtgtctagggctcggattgatgccagggtcgacttcgagtctggacggatcaagaaagaagagttctctgagatcatgaagatctgttccaccattgaagaactcagacggcaaaaataa

>H1N1_PB1_KC882265_A/California/20/2011

atggatgtcaatccgactctacttttcctaaaaattccagcgcaaaatgccataagcaccacattcccttatactggagatcctccatacagccatggaacaggaacaggatacaccatggacacagtaaacagaacacaccaatactcagaaaagggaaagtggacgacaaacacagagactggtgcaccccagctcaacccgattgatggaccactacctgaggataatgaaccaagtgggtatgcacaaacagactgtgttctagaggctatggctttccttgaagaatcccacccaggaatatttgagaattcatgccttgaaacaatggaagctgttcaacaaacaagggtagataaactaactcaaggtcgccagacttatgattggacattaaacagaaatcaaccggcagcaactgcattggccaacaccatagaggtctttagatcgaatggcctaacagctaatgagtcaggaaggctaatagatttcttaaaggatgtaatggaatcaatgaacaaagaggaaatagagataacaacccactttcaaagaaaaaggagagtaagagacaacatgaccaagaagatggtcactcaaagaacaatagggaagaaaaaacaaagactaaataagagaggctatctaataagagcactgacattaaatacgatgaccaaagatgcagagagaggcaagttaaaaagaagggctatcgcaacacctgggatgcagattagaggtttcgtatactttgttgaaactttagctaggagcatttgcgaaaagcttgaacagtctgggctcccagtagggggcaatgaaaagaaggccaaactggcaaatgttgtgagaaagatgatgactaattcacaagacacagagatttctttcacaatcactggggacaacactaagtggaatgaaaatcaaaatcctcgaatgttcctggcgatgattacatatatcaccagaaatcaacccgagtggttcagaaacatccttagcatggcacccataatgttctcaaacaaaatggcaaggctagggaaagggtacatgttcgagagtaaaagaatgaagattcgaacacaaattccagcagaaatgctagcaagcattgacctgaagtacttcaatgaatcaacaaagaagaaaattgagaaaataaggcctcttctaatagatggcacagcatcactgagtcctgggatgatgatgggcatgttcaacatgctaagtacggtcttgggagtctcgatactgaatcttggacaaaagaaatacaccaagacaacatactggtgggatgggctccaatcatccgacgattttgctctcatagtgaatgcaccaaaccatgagggaatacaagcaggagtggacagattctacaggacctgcaagttagtgggaatcaacatgagcaaaaagaagtcatatataaataaaacagggacatttgaattcacaagctttttttatcgctatggatttgtggctaattttagcatggagctacccagctttggagtgtctggagtaaatgaatcagctgacatgagtattggagtaacagtgataaagaacaacatgataaacaatgaccttggacctgcaacggcccagatggctcttcaattgttcatcaaagactacagatacacatataggtgccataggggagacacacaaattcagacgagaagatcatttgagttaaaaaagctgtgggatcaaacccaatcaaaggtagggctattagtatcagatggaggaccaaacttatacaatatacggaatcttcacattcctgaagtctgcttaaaatgggagctaatggatgatgattatcggggaagactttgtaatcccctgaatccctttgtcagtcataaagagattgattctgtaaacaatgctgtggtaatgccagcccatggtccagccaaaagcatggaatatgatgccgttgcaactacacattcctggattcccaagaggaatcgttctattctcaacacaagccaaaggggaattcttgaggatgaacagatgtaccagaagtgctgcaatctattcgagaaatttttccctagcagttcatataggagaccggttggaatttctagcatggtggaggccatggtgtctagggcccggattgatgccagggtcgacttcgagtctggacggatcaagaaagaagagttctctgagatcataaagatctgttccaccattgaagaactcagacggcaaaaataa

>H1N1_PB1_KC891539_A/Texas/22/2012

atggatgtcaatccgactctacttttcctaaaaattccagcgcaaaatgccataagcatcacattcccttatactggagatcctccatacagccatggaacaggaacaggatacaccatggacacagtaaacagaacacaccaatactcagaaaagggaaagtggacgaccaacacagagactggtgcaccccagctcaacccgattgatggaccactacctgaggataacgaaccaagtgggtatgcacaaacagactgtgtcctagaggctatggctttccttgaagaatcccacccaggaatatttgagaattcatgtcttgaaacaatggaagttgttcaacaaacaagagtagataaactgactcaaggtcgccagacttatgattggacattaaacagaaatcaaccggcagcaactgcattggccaacaccatagaagtctttagatcgaatggcctaacagctaatgagtcaggaaggctagtagatttcttaaaggatgtaatggaatcaatgaacaaagaggaaatagagataacaacccactttcaaagaaaaaggagagtaagagacaacatgaccaagaagatggtcacgcaaagaacaatagggaagaaaaaacagagactgaataagagaggctatctaataagagcactgacattaaatacgatgaccaaagatgcagagagaggcaagttaaaaagaagggctatcgcaacacctgggatgcagattagaggtttcgtatactttgttgaaactttagctaggagcatttgcgaaaagcttgaacagtctgggctcccagtagggggcaatgaaaagaaggccaaactggcaaatgttgtgagaaagatgatgactaattcacaagacacagagatttctttcacaatcactggggacaacactaagtggaatgaaaatcaaaatcctcgaatgttcctggcgatgattacatatatcaccaaaaatcaacccgagtggtttagaaacatcctgagcatggcacccataatgttctcaaacaaaatggcaagactagggaaagggtacatgttcgagagtaaaagaatgaagattcgaacacaaataccagcagaaatgctagcaagcattgacctgaagtacttcaatgaatcaacaaagaagaaaattgagaaaataaggcctcttttaatggatggcacagcatcactgagtcctgggatgatgatgggcatgttcaacatgctaagtacggtcttgggagtctcgatactgaatcttggacaaaagaaatacaccaagacaacatactggtgggatgggctccaatcatccgacgattttgctctcatagtgaatgcaccaaaccatgagggaatacaagcaggagtggacagattctacaggacctgcaagttagtgggaatcaacatgagcaaaaagaagtcctatataaataagacagggacatttgaattcacaagctttttttatcgctatggatttgtggctaattttagcatggagctacccagctttggagtgtctggagtaaatgaatcagctgacatgagtattggagtaacagtgataaagaacaacatgataaacaatgaccttggacctgcaacagcccagatggctcttcaattgttcatcaaagactacagatacacatataggtgccataggggagacacacaaattcagacgagaagatcatttgagttaaagaagctgtgggatcaaacccaatcaaaggtagggctattagtatcagatggaggaccaaacttatacaatatacggaatcttcacattcctgaagtctgcttaaaatgggagctaatggatgatgattatcggggaagactttgtaatcccctgaatccctttgtaagtcataaggagattgattctgtaaacaatgctgtggtaatgccagcccatggtccagccaaaagcatggaatatgatgccgttgcaactacacattcctggattcccaagaggaatcgttctattctcaacacaagccaaaggggaattcttgaggatgaacagatgtaccagaagtgctgcaatctattcgagaaatttttccctagcagttcatataggagaccggttggaatttctagcatggtggaggccatggtgtctagggcccggattgatgccagggtcgactttgagtctggacggataaagaaagaagagttctctgagatcatgaagatctgttccaccattgaagaactcagacggcaaaaataa

>H1N1_PB1_CY146783_A/Hickox/JY2/1940

atggatgtcaatccgaccttacttttcttaaaagtgccagcacaaaatgctataagcacaacttttccttatactggagaccctccttacagccatggaacaggaacaggatacaccatggatactgtcaacaggacacatcagtactcagaaaggggaaggtggacaacaaacaccgaaactggagcaccacaactcaacccgattgatggaccactgccagaagacaatgaaccaagtggttatgcccaaacagattgtgtattggaagcaatggctttccttgaggaatcccatcctggtatttttgaaaactcgtgtattgagacaatggaggttgttcagcaaacacgagtggacaaactgacacagggccgacagacctatgactggactctaaatagaaaccagcctgctgcaacagcattggccaacacaatagaagtgttcagatcaaatggtctcacggccaatgaatctggaaggctaatagacttccttaaggatgtaatggaatcaatggacaaagaagaaatggagatcacaactcattttcagagaaagagacgagtgagagacaatgtgactaagaaaatggtgacacagagaacaataggtaaaaggaagcagagattgaacaaaagaagttatctaattagagcattgaccctgaacacaatgaccaaagatgctgagagagggaagctaaaacgaagagcaattgcaaccccagggatgcaaataagagggtttgtatattttgttgagacattggcaagaagtatatgtgaaaaacttgaacaatctggattaccagttggaggcaatgagaagaaagcaaagttggcaaatgttgtaaggaagatgatgaccaattctcaggacactgaaatttctttcaccatcactggagataacaccaaatggaacgaaaatcagaaccctaggatgtttttggccatgatcacatatatgaccagaaatcagccagaatggttcagaaatgttctaagtattgctccaataatgttctcaaacaaaatggcgagactaggaaagggatacatgtttgaaagcaagagtatgaaacttagaactcaaatacctgcagaaatgctagcaaacatcgatttgaagtatttcaatgattcaacaagaaagaagattgaaagaatccgaccactcttaatagatgggactgcatcactgagccctggtatgatgatgggcatgttcaatatgttaagcactgtattgggcgtctccatcctgaatcttggacaaaagagatacaccaagactacttactggtgggatggtcttcaatcttctgatgattttgcgctgattgtgaatgcacccaatcatgaaggaattcaagccggagttgacaggttttatcgaacctgtaagttactcggaatcaatatgagcaagaaaaagtcttacataaacagaacaggtacatttgaattcacgagctttttctatcgttatgggtttgttgccaatttcagcatggagcttcccagttttggggtgtctggaatcaacgagtctgcagacatgagtattggagtcactgtcatcaaaaacaatatgataaacaatgatcttggtccagcaacagctcaaatggcccttcagttgttcatcaaagattacaggtacacatatcgatgccatagaggtgacacacaaatacaaacccgaagatcatttgaaataaagaaactgtgggagcaaacccgttccaaagctggtctgctggtctccgacggaggcccaaatttatacaacattagaaatcttcacattcctgaagtctgcttgaaatgggaattgatggatgaagattaccaggggcgtttatgcaacccactgaacccatttgtcagtcataaagagattgaatcagtgaacaatgcagtgatgatgccggcacatggcccagccaaaaacatggaatatgatgctgttgcaacaacacactcctggatccccaaaagaaatcggtccatcttgaatacgagccagagaggaatacttgaagatgaacaaatgtatcaaaggtgctgcaatttgtttgaaaaattcttccccagcagttcatacagaagaccagtcggaatatccagtatggtggaggctatggtttcaagagcccgaattgatgcacggattgacttcgaatcaggaaggataaagaaagaggagttcactgagatcatgaagatctgttccaccattgaagaactcagacggcaaaaatag

>H1N1_PB1_CY146791_A/Malaysia/JY2/1954

atggatgtcaatccgaccttacttttcttaaaagtgccagcacaaaatgctataagcacaactttcccttatactggagaccctccttacagccatgggacaggaacagggtacaccatggatactgtcaacaggacacatcagtactcagaaaggggaagatggacaaaaaacaccgaaactggagcaccacaactcaacccaattgatgggccactaccagaagacaatgaaccaagtggttatgcccaaacagattgtgtattagaagcaatggctttccttgaggaatcccatcctggtatttttgaaaactcatgtattgaaacgatggagattgttcagcaaacacgagtggacaagctaacacaaggccgacagacctatgactggactctgaatagaaaccagcctgctgctacagcattggccaacacaatagaagtgttcagatcaaatggcctcatggcaaatgaatctggaaggctaatagactttcttaaggatgtaatggagtcaatggacaaagaagaaatggagatcacaactcattttcagagaaagagacgagtgagggacaatgtgactaagaaaatggtgacacaaagaacaataggcaaaaagaaacagagattaaacaaaagtagttatctaattagggcattgaccctgaacacaatgaccaaagatgctgagagagggaaactaaaaagaagagcaattgcaaccccagggatgcaaataagggggtttgtatactttgttgagacactggcaagaagtatatgtgaaaaacttgaacaatcaggattgccagttggaggaaatgaaaagaaagcaaagttggcaaatgttgtaaggaagatgatgaccaattctcaggacactgaaatttctttcaccatcactggagataacaccaaatggaacgagaatcagaaccctagaatgtttttggccatgatcacttacataaccagaaatcagcccgaatggttcagaaatattctaagtattgctccaataatgttctcaaacaaaatggcgagactaggtaagggttacatgtttgagagcaagagtatgaaacttagaacgcaaatacctgcagagatgatagcaaacatcgatttgaagtatttcaatgattcaacaagaaagaaaattgaaaaaatccggccgctcttaatagatgggactgcatcactgagccctggaatgatgatgggtatgttcaatatgttaagcactgtattgggcgtctccatcctgaatctcggacaaaagagatacaccaagactacttactggtgggacggtcttcaatcatctgatgattttgctctgattgtgaatgcacccaatcatgcaggaattcaagctggagttgacaggttttatcgaacctgtaaactgctcggaatcaatatgagcaagaaaaagtcttacataaacagaacaggtacatttgaattcacgagctttttctatcgttatgggtttgttgccaatttcagcatggagcttcccagttttggggtatctgggatcaacgagtctgcagacatgagtattggggtcactgtcatcaaaaacaatatgataaacaatgatcttggtccagcaaccgctcaaatggcccttcagttattcatcaaagattacaggtacacgtatcgatgccacagaggtgacacacaaatacaaacccgaagatcatttgaaataaataaactatgggagcaaacccgttccaaagctgggctgctggtctccgatggaggtcccaatttatataacattagaaatctccacattcctgaagtatgcttgaaatgggaattgatggatgaggattaccaggggcgtttatgcaacccattgaacccatttgtcagtcataaagagattgaatcagtgaacaatgcagtggtgatgccggcacatggtccagccaaaaacatggagtatgatgctgtagcaacaacacactcctgggtccccaaaagaaatcgatccatcttgaatacgagccaaaggggaatacttgaagatgaacaaatgtatcaaaggtgctgcaatttatttgaaaaattcttccccagcagttcatacagaaggccagttggaatatccagtatggtggaggctatggtttccagagcccgaattgatgcacggattgatttcgaatctggaaggataaagaaagaggatttcactgagatcatgaagatctgttccaccattgaagaactcagacggcaaaaatag

>H1N1_PB1_CY147388_A/Texas/36-JY2/1991

atggatgtcaatccgaccttacttttcttaaaagtgccagcacaaaatgctataagcacaactttcccctatactggagaccctccttacagccatgggacagggacagggtacaccatggatacagtcaacaggacacatcagtactcagaaagaggaagatggacaaaaaataccgaaactggagcaccgcaactcaacccgattgatgggccactaccaaaagacaatgaaccaagtggctatgcccaaacagattgtgtattagaagcaatggctttccttgaggaatcccatcctggtatttttgaaaactcgtgtattgaaacaatggaggttgttcagcaaacaagagtggacaaactgacacaaggcagacagacctatgactggactctaaatagaaaccagcctgctgccacagcattggccaacactatagaagtgttcagatcaaacggcctcatagcaaatgaatctgggaggctaatagatttccttaaagatgtaatggagtcaatggacagagaagaagtagagatcacaactcattttcagagaaagagaagagtgagagacaatgtaactaaaaaaatggtgacacaaagaacaataggcaaaaagaaacataaattggacaaaagaagttatctaattagggcattaaccctgaacacaatgaccaaagatgctgagagggggaaactaaaacgtagagcaattgcaaccccaggaatgcagataagggggtttgtatactttgttgagacactggcaaggagcatatgtgaaaagcttgaacaatcagggttgccagttggaggaaatgagaagaaagcaaagttagcaaatgttgtaaggaagatgatgaccaactcccaggacactgaaatttctttcaccatcactggagataacacaaaatggaacgaaaatcagaaccctagaatgtttttggctatgatcacatatataaccagaaatcagcctgaatggttcagaaacattctaagtattgctccaataatgttttcaaacaaaatggcgagactaggtaaggggtacatgtttgaaagcaagagtatgaaactgagaactcaaatacctgcagaaatgctagccaacatagatttgaaatatttcaatgattcaacaaaaaagaaaattgaaaaaatccggccactattaatagatggaactgcatcactgagccctggaatgatgatgggcatgttcaatatgttaagcactgtcttgggcgtctccatcctgaatcttggacaaaagagatacaccaagactacttactggtgggatggtcttcaatcgtctgatgattttgctctgattgtgaatgcacccaactatgcaggaattcaagctggagttgacaggttttatcgaacctgtaagctgctcggaattaatatgagcaaaaagaagtcttacataaacagaacaggtacttttgaattcacgagctttttctatcgttatgggtttgttgccaatttcagcatggaacttcctagttttggggtgtctggggtcaatgagtctgcagacatgagtattggagtcactgtcatcaaaaacaatatgataaacaatgatcttggtccagcaactgctcaaatggcccttcagttattcatcaaagattacaggtacacgtatcgatgccacagaggtgacacacaaatacaaacccggagatcatttgagataaagaaactgtgggaccaaacccgctccaaagctgggctgttggtctctgatggaggccccaatttatataacattagaaatctccacattcctgaagtctgcttgaaatgggagttgatggatgaggattaccaggggcgtttatgcaatccattgaacccgtttgtcagtcataaagaaattgaatcagtgaacaatgcagtgatgatgccggcacatggtccagccaaaaatatggagtatgacgctgttgcaacaacacactcctgggtccccaaaaggaatcgatccattttgaatacgagccaaaaaggaatacttgaggatgagcaaatgtatcaaaggtgctgcaatttatttgaaaaattcttcccaagcagctcatacagaagaccagttggaatatccagtatggtagaggccatggtttccagagcccgaattgatgcacggattgatttcgaatctggaaggataaagaaagaggagttcgctgagatcatgaagacctgttccaccattgaagacctcagacggcaaaaatag

>H1N1_PB1_KF280701_A/India/P121778/2012

atggatgtcaatccgactctacttttcctaaaaattccagcgcaaaatgccataagcaccacattcccttatactggagatcctccatacagccatggaacaggaacaggatacaccatggacacagtaaacagaacacaccaatactcagaaaagggaaagtggacgacaaacacagagactggtgcaccccagctcaacccaattgatggaccactacctgaggataatgaaccaagtgggtatgcacaaacagactgtgttctagaggctatggctttcctagaagaatcccacccaggaatatttgagaattcatgccttgaaacaatggaagttgttcaacaaacaagggtagataaactgactcaaggtcgccagacttatgattggacattaaacagaaatcaaccggcagcaactgcattggccaacaccatagaagtctttagatcgaatgacctaacagctaatgagtcaggaaggctaatagatttcttaaaggatgtaatggaatcaatgaacaaagaggaaatagagataacaacccactttcaaagaaaaaggagagtaagagacaacatgaccaagaagatggtcacgcaaagaacaatagggaagaaaaaacagagactgaataagagaggctatctaataagagcactgacattaaacacgatgaccaaagatgcagagagaggcaagttaaaaagaagggctatcgcaacacctgggatgcagattagaggtttcgtatactttgttgaaactttagctaggagcatttgcgaaaagcttgaacagtctgggctcccagtagggggcaatgaaaagaaggccaaactggcaaatgttgtgagaaagatgatgactaattcacaagacacagagatttctttcacaatcactggggacaacactaagtggaatgaaaatcaaaatcctcgaatgttcctggcgatgattacatatatcaccagaaatcaacccgagtggtttagaaacatcctgagcatggcacccataatgttctcaaacaagatggcaagactagggaaagggtacatgttcgagagtaaaagaatgaagattcgaacacaaataccagcagaaatgctagcaagcattgacctgaagtacttcaatgaatcaacaaagaagaaaattgagaaaataaggcctcttttaatggatggcacagcatcactgagtcctgggatgatgatgggcatgttcaacatgctaagtacggtcttgggagtctcgatactgaatcttggacaaaagaaatacaccaagacaacatactggtgggatgggctccaatcatccgacgattttgctctcatagtgaatgcaccaaaccatgagggaatacaagcaggagtggacagattctacaggacctgcaagttagtgggaattaacatgagcaaaaagaagtcctatataaataagacagggacatttgaattcacaagctttttttatcgctatggatttgtggctaattttagcatggagctacccagctttggagtgtctggagtaaatgaatcagctgacatgagtattggagtaacagtgataaagaacaacatgataaacaatgaccttggacctgcaacggcccagatggctcttcaattgttcatcaaagactacagatacacatataggtgccataggggagacacacaaattcagacgagaagatcatttgagttaaagaagctgtgggatcaaacccaatcaaaggtagggctattagtatcagatggaggaccaaacttatacaatatacggaatcttcacattcctgaagtctgcttaaaatgggagctaatggatgatgattatcggggaagactttgtaatcccctgaatccctttgtaagtcataaagagattgattctgtaaacaatgctgtggtaatgccagcccatggtccagtcaaaagcatggaatatgatgccgttgcaactacacattcctggattcccaagaggaatcgttctattctcaacacaagccaaaggggaattcttgaggatgaacagatgtaccagaagtgctgcaatctattcgagaaatttttccctagcagttcatataggagaccggttggaatttctagcatggtggaggccatggtgtctagggcccggattgatgccagggtcgactttgagtctggacggatcaagaaagaagagttctctgagatcatgaagatctgttccaccattgaagaactcagacggcaaaaataa

>H1N1_PB1_CY147793_A/Mexico/24038/2009

atggatgtcaatccgactctacttttcctaaaaattccagcgcaaaatgccataagcaccacattcccttatactggagatcctccatacagccatggaacaggaacaggatacaccatggacacagtaaacagaacacaccaatactcagaaaagggaaagtggacgacaaacacagagactggtgcaccccagctcaacccgattgatggaccactacctgaggataatgaaccaagtgggtatgcacaaacagactgtgttctagaggctatggctttccttgaagaatcccacccaggaatatttgagaattcatgccttgaaacaatggaagttgttcaacaaacaagggtagataaactaactcaaggtcgccagacttatgattggacattaaacagaaatcaaccggcagcaactgcattggccaacaccatagaagtctttagatcgaatggcctaacagctaatgagtcaggaaggctaatagatttcttaaaggatgtaatggaatcaatgaacaaagaggaaatagagataacaacccactttcaaagaaaaaggagagtaagagacaacatgaccaagaagatggtcacgcaaagaacaatagggaagaaaaaacaaagactgaataagagaggctatctaataagagcactgacattaaatacgatgaccaaagatgcagagagaggcaagttaaaaagaagggctatcgcaacacctgggatgcagattagaggtttcgtatactttgttgaaactttagctaggagcatttgcgaaaagcttgaacagtctgggctcccagtagggggcaatgaaaagaaggccaaactggcaaatgttgtgagaaagatgatgactaattcacaagacacagagatttctttcacaatcactggggacaacactaagtggaatgaaaatcaaaatcctcgaatgttcctggcgatgattacatatatcaccagaaatcaacccgagtggttcagaaacatcctgagcatggcacccataatgttctcaaacaaaatggcaagactagggaaagggtacatgttcgagagtaaaagaatgaagattcgaacacaaataccagcagaaatgctagcaagcattgacctgaagtacttcaatgaatcaacaaagaagaaaattgagaaaataaggcctcttctaatagatggcacagcatcactgagtcctgggatgatgatgggcatgttcaacatgctaagtacggtcttgggagtttcgatactgaatcttggacaaaagaaatacaccaagacaatatactggtgggatgggctccaatcatccgacgattttgctctcatagtgaatgcaccaaaccatgagggaatacaagcaggagtggacagattctacaggacctgcaagttagtgggaatcaacatgagcaaaaagaagtcctatataaataagacagggacatttgaattcacaagctttttttatcgctatggatttgtggctaattttagcatggagctacccagctttggagtgtctggagtaaatgaatcagctgacatgagtattggagtaacagtgataaagaacaacatgataaacaatgaccttggacctgcaacggcccagatggctcttcaattgttcatcaaagactacagatacacatataggtgccataggggagacacacaaattcagacgagaagatcatttgagttaaagaagctgtgggatcaaacccaatcaaaggtagggctattagtatcagatggaggaccaaacttatacaatatacggaatcttcacattcctgaagtctgcttaaaatgggagctaatggatgatgattatcggggaagactttgtaatcccctgaatccctttgtcagtcataaagagattgattctgtaaacaatgctgtggtaatgccagcccatggtccagccaaaagcatggaatatgatgccgttgcaactacacattcctggattcccaagaggaatcgttctattctcaacacaagccaaaggggaattcttgaggatgaacagatgtaccagaagtgctgcaatctattcgagaaatttttccctagcagttcatataggagaccggttggaatttctagcatggtggaggccatggtgtctagggcccggattgatgccagggtcgacttcgagtctggacggatcaagaaagaagagttctctgagatcatgaagatctgttccaccattgaagaactcagacggcaaaaataa

>H1N1_PB1_KF411338_A/Qingdao/F162/2011

atggatgtcaatccgactctacttttcctaaaaattccagcacaaaatgccataagcaccacattcccttatactggagatcctccatacagccatggaacaggaacaggatacaccatggacacagtaaacagaacacaccaatactcagaaaagggaaagtggacaacaaacacagagactggtgcaccccagctcaacccgattgatggaccactacctgaggataatgaaccaagtgggtatgcacaaacagactgtgttctagaggctatggctttccttgaagaatcccacccaggaatatttgagaattcatgccttgaaacaatggaagttgttcaacaaacaagggtagataaactgactcaaggtcgccagacttatgattggacattaaacagaaatcaaccggcagcaactgcattggccaacaccatagaagtctttagatcgaatgacctaacagctaatgagtcaggaaggctaatagatttcttaaaggatgtaatggaatcaatgaacaaagaggaaatagagataacaacccactttcaaagaaaaaggagagtaagagacaacatgaccaagaagatggtcacgcaaagaacaatagggaagaaaaaacagagactgaataagagaggctatctaataagagcactgacattaaatacgatgaccaaagatgcagagagaggcaagttaaaaagaagggctatcgcaacacctgggatgcagattagaggtttcgtatactttgttgaaactttagctaggagcatttgcgaaaagcttgaacagtctgggctcccagtagggggcaatgaaaagaaggccaaactggcaaatgttgtgagaaagatgatgactaattcacaagacacagagatttctttcacaatcactggggacaacactaagtggaatgaaaatcaaaatcctcgaatgttcctggcgatgattacatatatcaccagaaatcaacccgagtggtttagaaacatcctgagcatggcacccataatgttctcaaacaaaatggcaagactagggaaagggtacatgttcgagagtaaaagaatgaagattcgaacacaaataccagcagaaatgctagcaagcattgacctgaagtacttcaatgaatcaacaaagaagaaaattgagaaaataaggcctcttttaatggatggcacagcatcactgagtcctgggatgatgatgggcatgttcaacatgctaagtacggtcttgggagtctcgatactgaatcttggacaaaagaaatacaccaagacaacatactggtgggatgggctccaatcatccgacgattttgctctcatagtgaatgcaccaaaccatgagggaatacaagcaggagtggacagattctacaggacctgcaagttagtgggaatcaacatgagcaaaaagaagtcctatataaataagacagggacatttgaattcacaagctttttttatcgctatggatttgtggctaattttagcatggagctacccagctttggagtgtctggagtaaatgaatcagctgacatgagtattggagtaacagtgataaagaacaacatgataaacaatgaccttggacctgcaacggcccagatggctcttcaattgttcatcaaagactacagatacacatataggtgccataggggagacacacaaattcagacgagaagatcatttgagttaaagaagctgtgggatcaaacccaatcaaaggtagggctattagtatcagatggaggaccaaacttatacaatatacggaatcttcacattcctgaagtctgcttaaaatgggagctaatggatgatgattatcggggaagactttgtaatcccctgaatccctttgtaagtcataaggagattgattctgtaaacaatgctgtggtaatgccagcccatggtccagccaaaagcgtggaatatgatgccgttgcaactacacattcctggattcccaagaggaatcgttctattctcaacacaagccaaaggggaattcttgaggatgaacagatgtaccagaagtgctgcaatctattcgagaaatttttccctagcagttcatataggagaccggttggaatttctagcatggtggaggccatggtgtctagggcccggattgatgccagggtcgactttgagtctggacggatcaagaaagaagagttttctgagatcatgaagatctgttccaccattgaagaactcagacggcaaaaataa

>H1N1_PB1_KF560292_A/Helsinki/1973/2012

atggatgtcaatccgactctacttttcctaaaaattccagcacaaaatgccataagcaccacattcccttatactggagatcctccatacagccatggaacaggaacaggatacaccatggacacagtaaacagaacacaccaatactcagaaaagggaaagtggacaacaaacacagagactggtgcaccccagctcaacccgattgatggaccactacctgaggataatgaaccaagtgggtatgcacaaacagattgtgttctagaggctatggctttccttgaagaatcccacccaggaatatttgagaattcatgccttgaaacaatggaagttgttcaacaaacaagggtagataaactgactcaaggtcgccagacttatgattggacattaaacagaaaccaaccggcagcaactgcattggccaacaccatagaagtctttagatcgaatgacctaacagccaacgagtcaggaaggctaatagatttcttaaaggatgtaatggaatcaatgaacaaagaggaaatagagataacaacccactttcaaagaaaaaggagagtaagagacaacatgaccaagaagatggtcacgcaaagaacaatagggaagaaaaaacagagactgaataagagaggctatctaataagagcactgacattaaatacgatgaccaaagatgcagagagaggcaagttaaaaagaagggctatcgcaacacctgggatgcagattagaggtttcgtatactttgttgaaactttagctaggagcatttgcgaaaagcttgaacagtctggactcccagtagggggcaatgaaaagaaggccaaactggcaaatgttgtgagaaagatgatgactaattcacaagatacagagatttctttcacaatcactggggacaacactaagtggaatgaaaatcaaaatcctcgaatgttcctggcgatgattacatatatcaccagaaatcaacccgagtggtttagaaacatcctgagcatggcacccataatgttctcaaataaaatggcaagactagggaaagggtacatgttcgagagtaaaagaatgaagattcgaacacaaataccagcagaaatgctagcaagcattgatctgaagtacttcaatgaatcaacaaagaaaaaaattgagaaaataaggcctcttttaatggatggcacagcatcactgagccctgggatgatgatgggcatgttcaacatgctaagtacggtcttgggagtctcgatactgaatcttggacaaaagaaatacaccaagacaacatactggtgggatgggctccaatcatccgacgattttgctctcatagtgaatgcaccaaaccatgagggaatacaagcaggagtggacagattctacaggacctgcaagttagtgggaatcaacatgagcaaaaagaagtcctatataaataagacagggacatttgaattcacaagctttttttatcgctatggatttgtggctaattttagcatggagctacccagctttggagtgtctggagtaaatgaatcagctgacatgagtattggagtaacagtgataaagaacaacatgataaacaatgaccttggacctgcaacggcccagatggctcttcaattgttcatcaaagactacagatacacatataggtgccataggggagacacacaaattcagacgagaagatcatttgagttaaagaagctgtgggatcaaacccaatcaaaggtagggctattaatatcagatggaggaccaaacttatacaatatacggaatcttcacattcctgaagtctgcttaaaatgggagctaatggatgatgattatcggggaagactttgtaatcccctgaatccctttgtaagtcataaggagattgattctgtaaacaatgctgtggtaatgccagcccatggtccagccaaaagcatggaatatgatgccgttgcaactacacattcctggattcccaagaggaatcgttctattctcaacacaagccaaaggggaattcttgaggatgaacagatgtaccagaagtgttgcaatctattcgagaaatttttccctagcagttcatataggagaccggttggaatttctagcatggtggaggccatggtatctagggcccggattgatgccagggtcgactttgagtctggacggatcaagaaagaagagttctctgagatcatgaagatctgttccaccattgaagaactcagacggcaaaaataa

>H1N1_PB1_KF560228_A/Helsinki/994/2013

atggatgtcaatccgactctacttttcctaaaaattccagcacaaaatgccataagcaccacattcccttatactggagatcctccatacagccatggaacaggaacaggatacaccatggacacagtaaacagaacacaccaatactcagaaaagggaaagtggacaacaaacacagagactggtgcaccccagctcaacccgattgatggaccactacctgaggataatgaaccaagtgggtatgcacaaacagactgtgttctagaggctatggctttccttgaagaatcccacccaggaatatttgagaattcatgccttgaaacaatggaagttgttcaacaaacaagggtagataaactgactcaaggtcgccagacttatgattggacattaaacagaaatcaaccggcagcaactgcattggccaacaccatagaagtctttagatcgaatgacctaacagctaacgagtcaggaaggctaatagatttcttaaaggatgtaatggaatcaatgaacaaagaggaaatagagataacaacccactttcaaagaaaaagaagagtaagagacaacatgaccaagaagatggtcacgcaaagaacaatagggaagaaaaaacagagactgaataagagaggctatctaataagagcactgacattaaatacgatgaccaaagatgcagagagaggcaagttaaaaagaagggctatcgcaacacctgggatgcagattagaggtttcgtatactttgttgaaactttagctaggagcatttgcgaaaagcttgaacagtctggactcccagtagggggcaatgaaaagaaggccaaattggcaaatgttgtgagaaagatgatgactaattcacaagatacagagatttctttcacaatcactggggacaacactaagtggaatgaaaatcaaaatcctcgaatgttcctggcgatgattacatatatcaccagaaatcaacccgagtggtttagaaacatcctgagcatggcacccataatgttctcaaataaaatggcaagactagggaaagggtacatgttcgagagtaaaagaatgaagattcgaacacaaataccagcagaaatgctagcaagcattgatctgaagtacttcaatgaatcaacaaagaagaaaattgagaaaataaggcctcttttaatggatggcacagcatcactgagtcctgggatgatgatgggcatgttcaacatgctaagtacggtcttgggagtctcgatactgaatcttggacaaaagaaatacaccaagacaacatactggtgggatgggctccaatcatccgacgattttgctctcatagtgaatgcaccaaaccatgagggaatacaagcaggagtggacagattctacaggacctgcaagttagtgggaatcaacatgagcaaaaagaagtcctatataaataagacagggacatttgaattcacaagctttttttatcgctatggatttgtggctaattttagcatggagctacccaactttggagtgtctggagtaaatgaatcagctgacatgagtattggagtaacagtgataaagaacaacatgataaacaatgaccttggacctgcaacggcccagatggctcttcaattgttcatcaaagactacagatacacatataggtgccataggggagacacacaaattcagacgagaagatcatttgagttaaagaagctgtgggatcaaacccaatcaaaggtagggctattagtatcagatggaggaccaaacttatacaatatacggaatcttcacattcctgaagtctgcttaaaatgggagctaatggatgatgattatcggggaagactttgtaatcccctgaatccctttgtaagtcataaggagattgattctgtaaacaatgctgtggtaatgccagcccatggtccagccaaaagcatggaatatgatgccgttgcaactacacattcctggattcccaagaggaatcgttctattctcaacacaagccaaaggggaattcttgaggatgaacagatgtaccagaagtgttgcaatctattcgagaaatttttccctagcagttcatataggagaccggttggaatttctagcatggtggaggccatggtatctagggcccggattgatgccagggtcgactttgagtctggacggatcaagaaagaagagttctcagagatcatgaagatctgttccaccattgaagaactcagacggcaaaaataa

>H1N1_PB1_KF560012_A/Helsinki/753/2013

atggatgtcaatccgactctacttttcctaaaaattccagcgcaaaatgccataagcaccacattcccttatactggagatcctccatacagccatggaacaggaacaggatacaccatggacacagtaaacagaacacaccaatactcagaaaagggaaagtggacgacaaacacagagactggtgcaccccagctcaacccgattgatggaccactacctgaggataatgaaccaagtgggtatgcacaaacagactgtgttctagaggctatggctttccttgaagaatcccacccaggaatatttgagaattcatgccttgaaacaatggaagttgttcaacaaacaagggtagataaactaactcaaggtcgccagacttatgattggacattaaacagaaatcaaccggcagcaactgcattggccaacaccatagaagtctttagatcgaatggcctaacagctaatgagtcaggaaggctaatagatttcttaaaggatgtaatggaatcaatgaacaaagaggaaatagagataacaacccactttcaaagaaaaaggagagtaagagacaacatgaccaagaagatggtcacgcaaagaacaatagggaagaaaaaacaaagactgaataagagaggctatctaataagagcactgacattaaatacgatgaccaaagatgcagagagaggcaagttaaaaagaagggctatcgcaacacctgggatgcagattagaggtttcgtatactttgttgaaactttagctaggagcatttgcgaaaagcttgaacagtctgggctcccagtagggggcaatgaaaagaaggccaaactggcaaatgttgtgagaaagatgatgactaattcacaagatacagagatttctttcacaatcactggggacaacactaagtggaatgaaaatcaaaatcctcgaatgttcctggcgatgattacatatatcaccagaaatcaacccgagtggttcagaaacatcctgagcatggcacccataatgttctcaaacaaaatggcaagactagggaaagggtacatgttcgagagtaaaagaatgaagattcgaacacaaataccagcagaaatgctagcaagcattgacctgaagtacttcaatgaatcaacaaagaagaaaattgagaaaataaggcctcttctaatagatggcacagcatcactgagtcctgggatgatgatgggcatgttcaacatgctaagtacggtcttgggagtctcgatactgaatcttggacaaaagaaatacaccaagacaacatactggtgggatgggctccaatcatccgacgattttgctctcatagtgaatgcaccaaaccatgagggaatacaagcaggagtggacagattctacaggacctgcaagttagtgggaatcaacatgagcaaaaagaagtcctatataaataagacagggacatttgaattcacaagctttttttatcgctatggatttgtggctaattttagcatggagctacccagctttggagtgtctggagtaaatgaatcagctgacatgagtattggagtaacagtgataaagaacaacatgataaacaatgaccttggacctgcaacggcccagatggctcttcaattgttcatcaaagactacagatacacatataggtgccataggggagacacacaaattcagacgagaagatcatttgagttaaagaagctgtgggatcaaacccaatcaaaggtagggctattagtatcagatggaggaccaaacttatacaatatacggaatcttcacattcctgaagtctgcttaaaatgggagctaatggatgatgattatcggggaagactttgtaatcccctgaatccctttgtcagtcataaagagattgattctgtaaacaatgctgtggtaatgccagcccatggtccagccaaaagcatggaatatgatgccgttgcaactacacattcctggattcccaagaggaatcgttctattctcaacacaagccaaaggggaattcttgaggatgaacagatgtaccagaagtgttgcaatctattcgagaaatttttccctagcagttcatataggagaccggttggaatttctagcatggtggaggccatggtatctagggcccggattgatgccagggtcgacttcgagtctggacggatcaagaaagaagagttctctgagatcatgaagatctgttccaccattgaagaactcagacggcaaaaataa

>H1N1_PB1_KF559996_A/Helsinki/737/2013

atggatgtcaatccgactctacttttcctaaaaattccagcacaaaatgccataagcaccacattcccttatactggagatcctccatacagccatggaacaggaacaggatacaccatggacacagtaaacagaacacaccaatactcagaaaagggaaagtggacaacaaacacagagactggtgcaccccagctcaacccgattgatggaccactacctgaggataatgaaccaagtgggtatgcacaaacagactgtgttctagaggctatggctttccttgaagaatcccacccaggaatatttgagaattcatgccttgaaacaatggaagttgttcaacaaacaagggtagataaactgactcaaggtcgccagacttatgattggacattaaacagaaatcaaccggcagcaactgcattggccaacaccatagaagtctttagatcgaatgacctaacagctaacgagtcaggaaggctaatagatttcttaaaggatgtaatggaatcaatgaacaaagaggaaatagagataacaacccactttcaaagaaaaaggagagtaagagacaacatgaccaagaagatggtcacgcaaagaacaatagggaagaaaaaacagagactgaataagagaggctatctaataagagcactgacattaaatacgatgaccaaagatgcagagagaggcaagttaaaaagaagggctatcgcaacacctgggatgcagattagaggtttcgtatactttgttgaaactttagctaggagcatttgcgaaaagcttgaacagtctgggctcccagtagggggcaatgaaaagaaggccaaactggcaaatgttgtgagaaagatgatgactaattcacaagatacagagatttctttcacaatcactggggacaacactaagtggaatgaaaatcaaaatcctcgaatgttcctggcgatgattacatatatcaccagaaatcaacccgagtggtttagaaacatcctgagcatggcacccataatgttctcaaataaaatggcaagactagggaaagggtacatgttcgagagtaaaagaatgaagattcgaacacaaataccagcagaaatgctagcaagcattgatctgaagtacttcaatgaatcaacaaagaagaaaattgagaaaataaggcctcttctaatagatggcacagcatcactgagtcctgggatgatgatgggcatgttcaacatgctaagtacggtcttgggagtctcgatactgaatcttggacaaaagaaatacaccaagacaacatactggtgggatgggctccaatcatccgacgattttgctctcatagtgaatgcaccaaaccatgagggaatacaagcaggagtggacagattctacaggacctgcaagttagtgggaatcaacatgagcaaaaagaagtcctatataaataagacagggacatttgaattcacaagctttttttatcgctatggatttgtggctaattttagcatggagctacccagctttggagtgtctggagtaaatgaatcagctgacatgagtattggagtaacagtgataaagaacaacatgataaacaatgaccttggacctgcaacggcccagatggctcttcaattgttcatcaaagactacagatacacatataggtgccataggggagacacacaaattcagacgagaagatcatttgagttaaagaagctgtgggatcaaacccaatcaaaggtagggctattagtatcagatggaggaccaaacttatacaatatacggaatcttcacattcctgaagtctgcttaaaatgggagctaatggatgatgattatcggggaagactttgtaatcccctgaatccctttgtaagtcataaagagattgattctgtaaacaatgctgtggtaatgccagcccatggtccagccaaaagcatggaatatgatgccgttgcaactacacattcctggattcccaagaggaatcgttctattctcaacacaagccaaaggggaattcttgaggatgaacagatgtaccagaagtgctgcaatctattcgagaaatttttccctagcagttcatataggagaccggttggaatttctagcatggtggaggccatggtatctagggcccggattgatgccagggtcgactttgagtctggacggatcaagaaagaagagttctctgagatcatgaagatctgttccaccattgaagaactcagacggcaaaaataa

>H1N1_PB1_KF559980_A/Helsinki/715/2013

atggatgtcaatccgactctacttttcctaaaaattccagcacaaaatgccataagcaccacattcccttatactggagatcctccatacagccatggaacaggaacaggatacaccatggacacagtaaacagaacacaccaatactcagaaaagggaaagtggacaacaaacacagagactggtgcaccccagctcaacccgattgatggaccactacctgaggataatgaaccaagtgggtatgcacaaacagactgtgttctagaggctatggctttccttgaagaatcccacccaggaatatttgagaattcatgccttgaaacaatggaagttgttcaacaaacaagggtagataaactgactcaaggtcgccagacttatgattggacattaaacagaaatcaaccggcagcaactgcattggccaacaccatagaagtctttagatcgaatgacctaacagctaacgagtcaggaaggctagtagatttcttaaaggatgtaatggaatcaatgaacaaagaggaaatagagataacaacccactttcaaagaaaaaggagagtaagagacaacatgaccaagaagatggtcacgcaaagaacaatagggaagaaaaaacagagactgaataagagaggctatctaataagagcactgacattaaatacgatgaccaaagatgcagagagaggcaagttaaaaagaagggctatcgcaacacctgggatgcagattagaggtttcgtatactttgttgaaattttagctaggagcatttgcgaaaagcttgaacagtctggactcccagtagggggcaatgaaaagaaggccaaactggcaaatgttgtgagaaagatgatgactaattcacaagatacagagatttctttcacaatcactggggacaacactaagtggaatgaaaatcaaaatcctcgaatgttcctggcgatgattacatatatcaccagaaatcaacccgagtggtttagaaacatcctgagcatggcacccataatgttctcaaataaaatggcaagactagggaaagggtacatgttcgagagtaaaagaatgaagattcgaacacaaataccagcagaaatgctagcaagcattgatctgaagtacttcaatgaatcaacaaagaagaaaattgagaaaataaggcctcttttaatggatggcacagcatcactgagtcctgggatgatgatgggcatgttcaacatgctaagtacggtcttgggagtctcgatactgaatcttggacaaaagaaatacaccaagacaacatactggtgggatgggctccaatcatccgacgattttgctctcatagtgaatgcaccaaaccatgagggaatacaagcaggagtggacagattctacaggacctgcaagttagtgggaatcaacatgagcaaaaagaagtcctatataaataagacagggacatttgaattcacaagctttttttatcgctatggatttgtggctaattttagcatggagctacccagctttggagtgtctggagtaaatgaatcagctgacatgagtattggagtaacagtgataaagaacaacatgataaacaatgaccttggacctgcaacggcccagatggctcttcaattgttcatcaaagactacaggtacacatataggtgccataggggagacacacaaattcagacgagaagatcatttgagttaaagaagctgtgggatcaaacccaatcaaaggtagggctattagtatcagatggaggaccaaacttatacaatatacggaatcttcacattcctgaagtctgcttaaaatgggagctaatggatgatgattatcggggaagactttgtaatcccctgaatccctttgtaagtcataaggagattgattctgtaaacaatgctgtggtaatgccagcccatggtccagccaaaagcatggaatatgatgcagttgcaactacacattcctggattcccaagaggaatcgttctattctcaacacaagccaaaggggaattcttgaggatgaacagatgtaccagaagtgttgcaatctattcgagaaatttttccctagcagttcatataggagaccggttggaatttctagcatggtggaggccatggtatctagggcccggattgatgccagggtcgactttgagtctggacggatcaagaaagaagagttctctgagatcatgaagatctgttccaccattgaagaactcagacggcaaaaataa

>H1N1_PB1_KF559964_A/Helsinki/694/2013

atggatgtcaatccgactctacttttcctaaaaattccagcacaaaatgccataagcaccacattcccttatactggagatcctccatacagccatggaacaggaacaggatacaccatggacacagtaaacagaacacaccaatactcagaaaagggaaagtggacaacaaacacagagactggtgcaccccagctcaacccgattgatggaccactacctgaggataatgaaccaagtgggtatgcacaaacagactgtgttctagaggctatggctttccttgaagaatcccacccaggaatatttgagaattcatgccttgaaacaatggaagttgttcaacaaacaagggtagataaactgactcaaggtcgccagacttatgattggacattaaacagaaatcaaccggcagcaactgcattggccaacaccatagaagtctttagatcgaatgacctaacagctaacgagtcaggaaggctaatagatttcttaaaggatgtaatggaatcaatgaacaaagaggaaatagagataacaacccactttcaaagaaaaaggagagtaagagacaacatgaccaagaagatggtcacgcaaagaacaatagggaagaaaaaacagagactgaataagagaggctatctaataagagcactgacattaaatacgatgaccaaagatgcagagagaggcaagttaaaaagaagggctatcgcaacacctgggatgcagattagaggtttcgtatactttgttgaaactttagctaggagcatttgcgaaaagcttgaacagtctggactcccagtagggggcaatgaaaagaaggccaaactggcaaatgttgtgagaaagatgatgactaattcacaagatacagagatttctttcacaatcactggggacaacactaagtggaatgaaaatcaaaatcctcgaatgttcctggcgatgattacatatatcaccagaaatcaacccgagtggtttagaaacatcctgagcatggcacccataatgttctcaaataaaatggcaagactagggaaagggtacatgttcgagagtaaaagaatgaagattcgaacacaaataccagcagaaatgctagcaagcattgatctgaagtacttcaatgaatcaacaaagaagaaaattgagaaaataaggcctcttttaatagatggcacagcatcactgagtcctgggatgatgatgggcatgttcaacatgctaagtacggtcttgggagtctcgatactgaatcttggacaaaagaaatacaccaagacaacatactggtgggatgggctccaatcatccgacgattttgctctcatagtgaatgcaccaaaccatgagggaatacaagcaggagtggacagattctacaggacctgcaagttagtgggaatcaacatgagcaaaaagaagtcctatataaataagacagggacatttgaattcacaagctttttttatcgctatggatttgtggctaattttagcatggagctacccagctttggagtgtctggagtaaatgaatcagctgacatgagtattggagtaacagtgataaagaacaacatgataaacaatgaccttggacctgcaacggcccagatggctcttcaattgttcatcaaagactacagatacacatataggtgccataggggagacacacaaattcagacgagaagatcatttgagttaaagaagctgtgggatcaaacccaatcaaaggtagggctattagtatcagatggaggaccaaacttatacaatatacggaatcttcacattcctgaagtctgcttaaaatgggagctaatggatgatgattatcggggaagactttgtaatcccctgaatccctttgtaagtcataaggagattgattctgtaaacaatgctgtggtaatgccagcccatggtccagccaaaagcatggaatatgatgccgttgcaactacacattcctggattcccaagaggaatcgttctattctcaacacaagccaaaggggaattcttgaggatgaacagatgtaccagaagtgttgcaatctattcgagaaatttttccctagcagttcatataggagaccggttggaatttctagcatggtggaggccatggtatctagggcccggattgatgccagggtcgactttgagtctggacggatcaagaaagaagagttctctgagatcatgaagatctgttccaccattgaagaactcagacggcaaaaataa

>H1N1_PB1_KF559700_A/Helsinki/492/2013

atggatgtcaatccgactctacttttcctaaaaattccagcacaaaatgccataagcaccacattcccttatactggagatcctccatacagccatggaacaggaacaggatacaccatggacacagtaaacagaacacaccaatactcagaaaagggaaagtggacaacaaacacagagactggtgcaccccagctcaacccgattgatggaccactacctgaggataatgaaccaagtgggtatgcacaaacagactgtgttctagaggctatggctttccttgaagaatcccacccaggaatatttgagaattcatgccttgaaacaatggaagttgttcaacaaacaagggtagataaactgacacaaggtcgccagacttatgattggacattaaacagaaatcaaccggcagcaactgcattggccaacaccatagaagtctttagatcgaatgacctaacagctaacgagtcaggaaggctaatagatttcttaaaggatgtaatggaatcaatgaacaaagaggaaatagagataacaacccactttcaaagaaaaaggagagtaagagacaacatgaccaagaagatggtcacgcaaagaacaatagggaagaaaaaacagagactgaataagagaggctatctaataagagcactgacattaaatacgatgaccaaagatgcagagagaggcaagttaaaaagaagggctatcgcaacacctgggatgcagattagaggtttcgtatactttgttgaaactttagctaggagcatttgcgaaaagcttgaacagtctggactcccagtagggggcaatgaaaagaaggccaaactggcaaatgttgtgagaaagatgatgaccaattcacaagatacagagatttctttcacaatcactggggacaacactaagtggaatgaaaatcaaaatcctcgaatgttcctggcgatgattacatatatcaccagaaatcaacccgagtggtttagaaacatcctgagcatggcacccataatgttctcaaataaaatggcaagactagggaaagggtacatgttcgagagtaaaagaatgaagattcgaacacaaataccagcagaaatgctagcaagcattgatctgaagtacttcaatgaatcaacaaagaagaaaattgagaaaataaggcctcttttaatggatggcacagcatcactgagtcctgggatgatgatgggcatgttcaacatgctaagtacggtcttgggagtctcgatactgaatcttggacaaaagaaatacaccaagacaacatactggtgggatgggctccaatcatccgacgattttgctctcatagtgaatgcaccaaaccatgagggaatacaagcaggagtggacagattctacaggacctgcaagttagtgggaatcaacatgagcaaaaagaagtcctatataaataagacagggacatttgaattcacaagctttttttatcgctatggatttgtggctaattttagcatggagctacccagctttggagtgtctggagtaaatgaatcagctgacatgagtattggagtaacagtgataaagaacaacatgataaacaatgaccttggacctgcaacggcccagatggctcttcaattgttcatcaaagactacagatacacatataggtgccataggggagacacacaaattcagacgagaagatcatttgagttaaagaagctgtgggatcaaacccaatcaaaggtagggctattagtatcagatggaggaccaaacttatacaatatacggaatcttcacattcctgaagtctgcttaaaatgggagctaatggatgatgattatcggggaagactttgtaatcccctgaatccctttgtaagtcataaggagattgattctgtaaacaatgctgtggtaatgccagcccatggtccagccaaaagcatggaatatgatgccgttgcaactacacattcctggattcccaagaggaatcgttctattctcaacacaagccaaaggggaattcttgaggatgaacagatgtaccagaagtgttgcaatctattcgagaaatttttccctagcagttcatataggagaccggttggaatttctagcatggtggaggccatggtatctagggcccggattgatgccagggtcgactttgagtctggacggatcaagaaagaagagttctcagagatcatgaagatctgttccaccattgaagaactcagacggcaaaaataa

>H1N1_PB1_KF559516_A/Helsinki/330/2013

atggatgtcaatccgactctacttttcctaaaaattccagcacaaaatgccataagcaccacattcccttatactggagatcctccatacagccatggaacaggaacaggatacaccatggacacagtaaacagaacacaccaatactcagaaaagggaaagtggacaacaaacacagagactggtgcaccccagctcaacccgattgatggaccactacctgaggataatgaaccaagtgggtatgcacaaacagactgtgttctagaggctatggctttccttgaagaatcccacccaggaatatttgagaattcatgccttgaaacaatggaagttgttcaacaaacaagggtagataaactgactcaaggtcgccagacttatgattggacattaaacagaaatcaaccggcagcaactgcattggccaacaccatagaagtctttagatcgaatgacctaacagctaacgagtcaggaaggctaatagatttcttaaaggatgtaatggaatcaatgaacaaagaggaaatagagataacaacccactttcaaagaaaaaggagagtaagagacaacatgaccaagaagatggtcacgcaaagaacaatagggaagaaaaaacagagactgaataagagaggctatctaataagagcactgacattaaatacaatgaccaaagatgcagagagaggcaagttaaaaagaagggctatcgcaacacctgggatgcagattagaggtttcgtatactttgttgaaactttagctaggagcatttgcgaaaagcttgaacagtctggactcccagtagggggcaatgaaaagaaggccaaactggcaaatgttgtgagaaagatgatgactaattcacaagatacagagatttctttcacaatcactggggacaacactaagtggaatgaaaatcaaaatcctcgaatgttcctggcgatgattacatatatcaccagaaatcaacccgagtggtttagaaacatcctgagcatggcacccataatgttctcaaataaaatggcaagactagggaaagggtacatgttcgagagtaaaagaatgaagattcgaacacaaataccagcagaaatgctagcaagcattgatctgaagtacttcaatgaatcaacaaagaagaaaattgagaaaataaggcctcttttaatggatggcacagcatcactgagtcctgggatgatgatgggcatgttcaacatgctaagtacggtcttgggagtctcgatactgaatcttggacaaaagaaatacaccaagacaacatactggtgggatgggctccaatcatccgacgattttgctctcatagtgaatgcaccaaaccatgagggaatacaagcaggagtggacagattctacaggacctgcaagttagtgggaatcaacatgagcaaaaagaagtcctatataaataagacagggacatttgaattcacaagctttttttatcgctatggatttgtggctaattttagcatggagctacccagctttggagtgtctggagtaaatgaatcagctgacatgagtattggagtaacagtgataaagaacaacatgataaacaatgaccttggacctgcaacggcccagatggctcttcaattgttcatcaaagactacagatacacatataggtgccataggggagacacacaaattcagacgagaagatcatttgagttaaagaagctgtgggatcaaacccaatcaaaggtagggctattagtatcagatggaggaccaaacttatacaatatacggaatcttcacattcctgaagtctgcttaaaatgggagctaatggatgatgattatcggggaagactttgtaatcccctgaatccctttgtaagtcataaggagattgattctgtaaacaatgctgtggtaatgccagcccatggtccagccaaaagcatggaatatgatgccgttgcaactacacattcctggattcccaagaggaatcgttctattctcaacacaagccaaaggggaattcttgaggatgaacagatgtaccagaagtgttgcaatctattcgagaaatttttccctagcagttcatataggagaccggttggaatttctagcatggtggaggccatggtatctagggcccggattgatgccagggtcgactttgagtctggacggatcaagaaagaagagttctctgagatcatgaagatctgttccaccattgaagaactcagacggcaaaaataa

>H1N1_PB1_KF559412_A/Helsinki/141/2013

atggatgtcaatccgactctacttttcctaaaaattccagcacaaaatgccataagcaccacattcccttatactggagatcctccatacagccatggaacaggaacaggatacaccatggacacagtaaacagaacacaccaatactcagaaaagggaaagtggacaacaaacacagagactggtgcaccccagctcaacccgattgatggaccactacctgaggataatgaaccaagtgggtatgcacaaacagactgtgttctagaggctatggctttccttgaagaatcccacccaggaatatttgagaattcatgccttgaaacaatggaagttgttcaacaaacaagggtagataaactgactcaaggtcgccagacttatgattggacattaaacagaaatcaaccggcagcaactgcattggccaacaccatagaagtctttagatcgaatgacctaacagctaacgagtcaggaaggctaatagatttcttaaaggatgtaatggaatcaatgaacaaagaggaaatagagataacaacccactttcaaagaaaaaggagagtaagagacaacatgaccaagaagatggtcacgcaaagaacaatagggaagaaaaaacagagactgaataagagaggctatctaataagagcactgacattaaatacaatgaccaaagatgcagagagaggcaagttaaaaagaagggctatcgcaacacctgggatgcagattagaggtttcgtatactttgttgaaactttagctaggagcatttgcgaaaagcttgaacagtctggactcccagtagggggcaatgaaaagaaggccaaactggcaaatgttgtgagaaagatgatgactaattcacaagatacagagatttctttcacaatcactggggacaacactaagtggaatgaaaatcaaaatcctcgaatgttcctggcgatgattacatatatcaccagaaatcaacccgagtggtttagaaacatcctgagcatggcacccataatgttctcaaataaaatggcaagactagggaaagggtacatgttcgagagtaaaagaatgaagattcgaacacaaataccagcagaaatgctagcaagcattgatctgaagtacttcaatgaatcaacaaagaagaaaattgagaaaataaggcctcttttaatggatggcacagcatcactgagtcctgggatgatgatgggcatgttcaacatgctaagtacggtcttgggagtctcgatactgaatcttggacaaaagaaatacaccaagacaacatactggtgggatgggctccaatcatccgacgattttgctctcatagtgaatgcaccaaaccatgagggaatacaagcaggagtggacagattctacaggacctgcaagttagtgggaatcaacatgagcaaaaagaagtcctatataaataagacagggacatttgaattcacaagctttttttatcgctatggatttgtggctaattttagcatggagctacccagctttggagtgtctggagtaaatgaatcagctgacatgagtattggagtaacagtgataaagaacaacatgataaacaatgaccttggacctgcaacggcccagatggctcttcaattgttcatcaaagactacagatacacatataggtgccataggggagacacacaaattcagacgagaagatcatttgagttaaagaagctgtgggatcaaacccaatcaaaggtagggctattagtatcagatggaggaccaaacttatacaatatacggaatcttcacattcctgaagtctgcttaaaatgggagctaatggatgatgattatcggggaagactttgtaatcccctgaatccctttgtaagtcataaggagattgattctgtaaacaatactgtggtaatgccagcccatggtccagccaaaagcatggaatatgatgccgttgcaactacacattcctggattcccaagaggaatcgttccattctcaacacaagccaaaggggaattcttgaggatgaacagatgtaccagaagtgttgcaatctattcgagaaatttttccctagcagttcatataggagaccggttggaatttctagcatggtggaggccatggtatctagggcccggattgatgccagggtcgactttgagtctggacggatcaagaaagaagagttctctgagatcatgaagatctgttccaccattgaagaactcagacggcaaaaataa

>H1N1_PB1_KF612054_A/Santa Cruz/541/2012

atggatgtcaatccgactttacttttcctaaaaattccagcgcaaaatgccataagcaccacattcccttatactggagatcctccatacagccatggaacaggaacaggatacaccatggacacagtaaacagaacacaccaatactcagaaaagggaaagtggacgacaaacacagagactggtgcaccccagctcaacccgattgatggaccactacctgaggataatgaaccaagtgggtatgcacaaacagactgtgttctagaggctatggctttccttgaagaatcccacccaggaatatttgagaattcatgccttgaaacaatggaggttgttcaacaaacaagggtagataaactaactcaaggtcgccagacttatgattggacattaaacagaaatcaaccggcagcaactgcattggccaacaccatagaagtctttagatcgaatggcctaacagctaatgagtcaggaaggctaatagatttcttaaaggatgtaatggaatcaatgaacaaagaggaaatagagataacaacccactttcaaagaaaaaggagagtaagagacaacatgaccaagaagatggtcacgcaaagaacaatagggaagaaaaaacaaagactgaataagagaggctatctaataagagcactgacattaaatacgatgaccaaagatgcagagagaggcaagttaaaaagaagggctatcgcaacacctgggatgcagattagaggtttcgtatactttgttgaaactttagctaggagcatttgcgaaaagcttgaacagtctgggctcccagtagggggcaatgaaaagaaggccaaactggcaaatgttgtgagaaagatgatgactaattcacaagacacagagatttctttcacaatcactggggacaacactaagtggaatgaaaaccaaaatcctcgaatgttcctggcgatgattacatatatcaccagaaatcaacccgagtggttcagaaacatcctgagcatggcacccataatgttctcaaacaaaatggcaagactagggaaagggtacatgttcgagagtaaaagaatgaagattcgaacgcaaataccagcagaaatgctagcaagcattgacctgaagtacttcaatgaatcaacaaagaagaaaattgagaaaataaggcctcttttaatagatggcacagcatcactgagccctgggatgatgatgggcatgttcaacatgctaagtacggtcttgggagtctcgatactgaatcttggacaaaagaaatacaccaagacaatatactggtgggatgggctccaatcatccgacgattttgctctcatagtgaatgcaccaaaccatgagggaatacaagcaggagtggacagattttacaggacctgcaagttagtgggaatcaacatgagcaaaaagaagtcctatataaataagacagggacatttgaattcacaagctttttttatcgctatggatttgtggctaattttagcatggagctacccagctttggagtgtctggagtaaatgaatcagctgacatgagtattggagtaacagtgataaagaacaacatgataaacaatgaccttggacctgcaactgcccagatggctcttcaattgttcatcaaagactacagatacacatataggtgccataggggagacacacaaattcagacgagaagatcatttgagttaaagaagctgtgggatcaaacccaatcaaaggtagggctattagtatcagatggaggaccaaacttatacaatatacggaatcttcacattcctgaagtctgcttaaaatgggagctaatggatgatgattatcggggaagactctgtaatcccctgaatccctttgtaagtcataaagagattgattctgtaaacaatgctgtgataatgccagctcatggtccagccaaaagcatggaatatgatgccgttgcaactacgcattcctggattcccaagaggaatcgttctattctcaacacaagccaaaggggaattcttgaggatgaacagatgtaccagaagtgctgcaatctattcgagaaatttttccctagcagttcatataggagaccggttggaatttctagcatggtggaggccatggtgtctagggcccggattgatgccagggtcgactttgagtctggacggatcaagaaagaagagttctctgagatcatgaagatctgttccaccattgaagaactcagacggcaaaaataa

>H1N1_PB1_KF612050_A/Santa Cruz/0181/2011

atggatgtcaatccgactctacttttcctaaaaattccagcgcaaaatgccataagcaccacattcccttatactggagatcctccatacagccatggaacaggaacaggatacaccatggacacagtaaacagaacacaccaatactcagaaaagggaaagtggacgacaaacacagagactggtgcaccccagctcaacccgattgatggaccactacctgaggataatgaaccaagtgggtatgcacaaacagactgtgttctagaggctatggctttccttgaagaatcccacccaggaatatttgagaattcatgccttgaaacaatggaggttgttcaacaaacaagggtagataaactaactcaaggtcgccagacttatgattggacattaaacagaaatcaaccggcagcaactgcattggccaacaccatagaagtctttagatcaaatggcctaacagctaatgagtcaggaaggctaatagatttcttaaaggatgtaatggaatcaatgaacaaagaggaaatagagataacaacccactttcaaagaaaaaggagagtaagagacaacatgaccaagaagatggtcactcaaagaacaatagggaagaaaaaacaaagactgaataagagaggctatctaataagagcactgacattaaatacgatgaccaaagatgcagagagaggcaagttaaaaagaagggctatcgcaacacctgggatgcagattagaggtttcgtatactttgttgaaactttagctaggagcatttgcgaaaagcttgaacagtctgggctcccagtagggggcaatgaaaagaaggccaaactggcaaatgttgtgagaaagatgatgactaattcacaagacacagagatttctttcacaatcactggggacaacactaagtggaatgaaaaccaaaatcctcgaatgttcctggcgatgattacatatatcaccagaaatcaacccgagtggttcagaaacatcctgagcatggcacccataatgttctcaaacaaaatggcaagactagggaaagggtacatgttcgagagtaaaagaatgaagattcgaacgcaaataccagcagaaatgctagcaagcattgacctgaagtacttcaatgaatcaacaaagaagaaaattgagaaaataaggcctcttttaatagatggcacagcatcactgagtcctgggatgatgatgggcatgttcaacatgctaagtacggtcttgggagtctcgatactgaatcttggacaaaagaaatacaccaagacaatatactggtgggatgggctccaatcatccgacgattttgctctcatagtgaatgcaccaaaccatgagggaatacaagcaggagtggacagattttacaggacctgcaagttagtgggaatcaacatgagcaaaaagaagtcctatataaataagacagggacatttgaattcacaagctttttttatcgctatggatttgtggctaattttagcatggagctacccagctttggagtgtctggagtaaatgaatcagctgacatgagtattggagtaacagtgataaagaacaacatgataaacaatgaccttggacctgcaacggcccagatggctcttcaattgttcatcaaagactacagatacacatataggtgccataggggagacacacaaattcagacgagaagatcatttgagttaaagaagctgtgggatcaaacccaatcaaaggtagggctattagtatcagatggaggaccaaacttatacaatatacggaatcttcacattcctgaagtctgcttaaaatgggagctaatggatgatgattatcggggaagactttgtaatcccctgaatccctttgtaagtcataaagagattgattctgtaaacaatgctgtgataatgccagctcatggtccagccaaaagcatggaatatgatgccgttgcaactacgcattcctggattcccaagaggaatcgttctattctcaacacaagccaaaggggaattcttgaggatgaacagatgtaccagaagtgctgcaatctattcgagaaatttttccctagcagttcatataggagaccggttggaatttctagcatggtggaggccatggtgtctagggcccggattgatgccagggtcgactttgagtctggacggatcaagaaagaagagttctctgagatcatgaagatctgttccaccattgaagaactcagacggcaaaaataa

>H1N1_PB1_KF746699_A/Kazan/CRIE-03/2013

atggatgtcaatccgactctacttttcctaaaaattccagcgcaaaatgccataagcaccacattcccttatactggagatcctccatacagccatggaacaggaacaggatacaccatggacacagtaaacagaacacaccaatactcagaaaagggaaagtggacaacaaacacagagactggtgcaccccagctcaacccgattgatggaccactacctgaggataatgaaccaagcgggtatgcacaaacagactgtgttctagaggctatggctttccttgaagaatcccacccaggaatatttgagaattcatgccttgaaacaatggaagttgttcaacaaacaagggtagataaactgactcaaggtcgccagacttatgattggacattaaacagaaatcaaccggcagcaactgcattggccaacaccatagaagtctttagatcgaatgacctaacagctaacgagtcaggaaggctaatagatttcttaaaggatgtaatggaatcaatgaacaaagaggaaatagagataacaacccactttcaaagaaaaaggagaataagagacaacatgaccaagaagatggtcacgcaaagaacaatagggaagaaaaaacagagactgaataagagaggctatctaataagagcactgacattaaatacgatgaccaaagatgcagagagaggcaagttaaaaagaagggctatcgcaacacctgggatgcagattagaggtttcgtatactttgttgaaactttagctaggagcatttgcgaaaagcttgaacagtctggactcccagtagggggcaatgaaaagaaggccaaactggcaaatgttgtgagaaagatgatgactaattcacaagatacagagatttctttcacaatcactggggacaacactaagtggaatgaaaatcaaaatcctcgaatgttcctggcgatgattacatatatcaccagaaatcaacccgagtggtttagaaacatcctgagcatggcacccataatgttctcaaataaaatggcaagactagggaaagggtacatgttcgagagtaaaagaatgaagattcgaacacaaataccagcagaaatgctagcaagcattgatctgaagtacttcaatgaatcaacaaagaagaaaattgagaaaataaggcctcttttaatggatggcacagcatcactgagtcctgggatgatgatgggcatgttcaacatgctaagtacggtcttgggagtctcgatactgaatcttggacaaaagaaatacaccaagacaacatactggtgggatgggctccaatcatccgacgattttgctctcatagtgaatgcaccaaaccatgagggaatacaagcaggagtggacagattctacaggacctgcaagttagtgggaatcaacatgagcaaaaagaagtcctatataaataagacagggacatttgaattcacaagctttttttatcgctatggatttgtggctaattttagcatggagctacccagctttggagtgtctggagtaaatgaatcagctgacatgagtattggagtaacagtgataaagaacaacatgataaacaatgaccttggacctgcaacggcccagatggctcttcaattgttcatcaaagactacagatacacatataggtgccataggggagacacacaaattcagacgagaagatcatttgagttaaagaagctgtgggatcaaacccaatcaaaggtagggctattagtatcagatggaggaccaaacttatacaatatacggaatcttcacattcctgaagtctgcttaaaatgggagctaatggatgatgattatcggggaagactttgtaatcccctgaatccctttgtaagtcataaggagattgattctgtaaacaatgctgtggtaatgccagcccatggtccagccaaaagcatggaatatgatgccgttgcaactacacattcctggattcccaagaggaatcgttctattctcaacacaagccaaaggggaattcttgaggatgaacagatgtaccagaagtgttgcaatctattcgagaaatttttccctagcagttcatataggagaccggttggaatttctagcatggtggaggccatgatatctagggcccggattgatgccagggtcgactttgagtctggacggatcaagaaagaagagttctcagagatcatgaagatctgttccaccattgaagaactcagacggcaaaaataa

>H1N1_PB1_KF746708_A/Kazan/CRIE-04/2013

atggatgtcaatccgactctacttttcctaaaaattccagcgcaaaatgccataagcaccacattcccttatactggagatcctccatacagccatggaacaggaacaggatacaccatggacacagtaaacagaacacaccaatactcagaaaagggaaagtggacaacaaacacagagactggtgcaccccagctcaacccgattgatggaccactacctgaggataatgaaccaagcgggtatgcacaaacagactgtgttctagaggctatggctttccttgaagaatcccacccaggaatatttgagaattcatgccttgaaacaatggaagttgttcaacaaacaagggtagataaactgactcaaggtcgccagacttatgattggacattaaacagaaatcaaccggcagcaactgcattggccaacaccatagaagtctttagatcgaatgacctaacagctaacgagtcaggaaggctaatagatttcttaaaggatgtaatggaatcaatgaacaaagaggaaatagagataacaacccactttcaaagaaaaaggagaataagagacaacatgaccaagaagatggtcacgcaaagaacaatagggaagaaaaaacagagactgaataagagaggctatctaataagagcactgacattaaatacgatgaccaaagatgcagagagaggcaagttaaaaagaagggctatcgcaacacctgggatgcagattagaggtttcgtatactttgttgaaactttagctaggagcatttgcgaaaagcttgaacagtctggactcccagtagggggcaatgaaaagaaggccaaactggcaaatgttgtgagaaagatgatgactaattcacaagatacagagatttctttcacaatcactggggacaacactaagtggaatgaaaatcaaaatcctcgaatgttcctggcgatgattacatatatcaccagaaatcaacccgagtggtttagaaacatcctgagcatggcacccataatgttctcaaataaaatggcaagactagggaaagggtacatgttcgagagtaaaagaatgaagattcgaacacaaataccagcagaaatgctagcaagcattgatctgaagtacttcaatgaatcaacaaagaagaaaattgagaaaataaggcctcttttaatggatggcacagcatcactgagtcctgggatgatgatgggcatgttcaacatgctaagtacggtcttgggagtctcgatactgaatcttggacaaaagaaatacaccaagacaacatactggtgggatgggctccaatcatccgacgattttgctctcatagtgaatgcaccaaaccatgagggaatacaagcaggagtggacagattctacaggacctgcaagttagtgggaatcaacatgagcaaaaagaagtcctatataaataagacagggacatttgaattcacaagctttttttatcgctatggatttgtggctaattttagcatggagctacccagctttggagtgtctggagtaaatgaatcagctgacatgagtattggagtaacagtgataaagaacaacatgataaacaatgaccttggacctgcaacagcccagatggctcttcaattgttcatcaaagactacagatacacatataggtgccataggggagacacacaaattcagacgagaagatcatttgagttaaagaagctgtgggatcaaacccaatcaaaggtagggctattagtatcagatggaggaccaaacttatacaatatacggaatcttcacattcctgaagtctgcttaaaatgggagctaatggatgatgattatcggggaagactttgtaatcccctgaatccctttgtaagtcataaggagattgattctgtaaacaatgctgtggtaatgccagcccatggtccagccaaaagcatggaatatgatgccgttgcaactacacattcctggattcccaagaggaatcgttctattctcaacacaagccaaaggggaattcttgaggatgaacagatgtaccagaagtgttgcaatctattcgagaaatttttccctagcagttcatataggagaccggttggaatttctagcatggtggaggccatgatatctagggcccggattgatgccagggtcgactttgagtctggacggatcaagaaagaagagttctcagagatcatgaagatctgttccaccattgaagaactcagacggcaaaaataa

>H1N1_PB1_KF791415_A/Czech Republic/31/2013

atggatgtcaatccgactctacttttcctaaaaattccagcacaaaatgccataagcaccacattcccttatactggagatcctccatacagccatggaacaggaacaggatacaccatggacacagtaaacagaacacaccaatactcagaaaagggaaagtggacaacaaacacagagactggtgcaccccagctcaacccgattgatggaccactacctgaggataatgaaccaagtgggtatgcacaaacagactgtgttctagaggctatggctttccttgaagaatcccacccaggaatatttgagaattcatgccttgaaacaatggaagttgttcaacaaacaagggtagataaactgactcaaggtcgccagacttatgattggacattaaacagaaatcaaccggcagcaactgcattggccaacaccatagaagtctttagatcgaatgacctaacagctaacgagtcaggaaggctaatagatttcttaaaggatgtaatggaatcaatgaacaaagaggaaatagagataacaacccactttcaaagaaaaaggagagtaagagacaacatgaccaagaagatggtcacgcaaagaacaatagggaagaaaaaacagagactgaataagagaggctatctaataagagcactgacattaaatacgatgaccaaagatgcagagagaggcaagttaaaaagaagggctatcgcaacacctgggatgcagattagaggtttcgtatactttgttgaaactttagctaggagcatttgcgaaaagcttgaacagtctggactcccagtagggggcaatgaaaagaaggccaaactggcaaatgttgtgagaaagatgatgactaattcacaagatacagagatttctttcacaatcactggggacaacactaagtggaatgaaaatcaaaatcctcgaatgttcctggcgatgattacatatatcaccagaaatcaacccgagtggtttagaaacatcctgagcatggcacccataatgttctcaaataaaatggcaagactagggaaagggtacatgttcgagagtaaaagaatgaagattcgaacacaaataccagcagaaatgctagcaagcattgatctgaagtacttcaatgaatcaacaaagaagaaaattgagaaaataaggcctcttttaatggatggcacagcatcactgagtcctgggatgatgatgggcatgttcaacatgctaagtacggtattgggagtctcgatactgaatcttggacaaaagaaatacaccaagacaacatactggtgggatgggctccaatcatccgacgattttgctctcatagtgaatgcaccaaaccatgagggaatacaagcaggagtggacagattctacaggacctgcaagttagtgggaatcaacatgagcaaaaagaagtcctatataaataagacagggacatttgaattcacaagctttttttatcgctatggatttgtggctaattttagcatggagctacccagctttggagtgtctggagtaaatgaatcagctgacatgagtattggagtaacagtgataaagaacaacatgataaacaatgaccttggacctgcaacggcccagatggctcttcaattgttcatcaaagactacaggtacacatataggtgccataggggagacacacaaattcagacgagaagatcatttgagttaaagaagctgtgggatcaaacccaatcaaaggtagggctattagtatcagatggaggaccaaacttatacaatatacggaatcttcacattcctgaagtctgcttaaaatgggagctaatggatgatgattatcggggaagactttgtaatcccctgaatccctttgtaagtcataaggagattgattctgtaaacaatgctgtggtaatgccagcccatggtccagccaaaagcatggaatatgatgcagttgcaactacacattcctggattcccaagaggaatcgttctattctcaacacaagccaaaggggaattcttgaggatgaacagatgtaccataagtgttgcaatctattcgagaaatttttccctagcagttcatataggagaccggttggaatttctagcatggtggaggccatggtatctagggcccggattgatgccagggtcgactttgagtctggacggatcaagaaagaagagttctctgagatcatgaagatctgttccaccattgaagaactcagacggcaaaaataa

>H1N1_PB1_KF791471_A/Czech Republic/191/2013

atggatgtcaatccgactctacttttcctaaaaattccagcacaaaatgccataagcaccacattcccttatactggagatcctccatacagccatggaacaggaacaggatacaccatggacacagtaaacagaacacaccaatactcagaaaagggaaagtggacaacaaacacagagactggtgcaccccagctcaacccgattgatggaccactacctgaggataatgaaccaagtgggtatgcacaaacagactgtgttctagaggctatggctttccttgaagaatcccacccaggaatatttgagaattcatgccttgaaacaatggaagttgttcaacaaacaagggtagataaactgactcaaggtcgccagacttatgattggacattaaacagaaatcaaccggcagcaactgcattggccaacaccatagaagtctttagatcgaatgacctaacagctaacgagtcaggaaggctaatagatttcttaaaggatgtaatggaatcaatgaacaaagaggaaatagagataacaacccactttcaaagaaaaaggagagtaagagacaacatgaccaagaagatggtcacgcaaagaacaatagggaagaaaaaacagagactgaataagagaggctatctaataagagccctgacattaaatacgatgaccaaagatgcagagagaggcaagttaaaaagaagggctatcgcaacacctgggatgcagattagaggtttcgtatactttgttgaaactttagctaggagcatttgcgaaaagcttgaacagtctggactcccagtagggggcaatgaaaagaaggccaaactggcaaatgttgtgagaaagatgatgactaattcacaagatacagagatttctttcacaatcactggggacaacactaagtggaatgaaaatcaaaatcctcgaatgttcctggcgatgattacatatatcaccagaaatcaacccgagtggtttagaaacatcctgagcatggcacccataatgttctcaaataaaatggcaagactagggaaagggtacatgttcgagagtaaaagaatgaagattcgaacacaaataccagcagaaatgctagcaagcattgatctgaagtacttcaatgaatcaacaaagaagaaaattgagaaaataaggcctcttttaatggatggcacagcatcactgagtcctgggatgatgatgggcatgttcaacatgctaagtacggtcttgggagtctcgatactgaatcttggacaaaagaaatacaccaagacaacatactggtgggatgggctccaatcatccgacgattttgctctcatagtgaatgcaccaaaccatgagggaatacaagcaggagtggacagattctacaggacctgcaagttagtgggaatcaacatgagcaaaaagaagtcctatataaataagacagggacatttgaattcacaagctttttttatcgctatggatttgtggctaattttagcatggagctacccagctttggagtgtctggagtaaatgaatcagctgacatgagtattggagtaacagtgataaagaacaacatgataaacaatgaccttggacctgcaacggcccagatggctcttcaattgttcatcaaagactacaggtacacatataggtgccataggggagacacacaaattcagacgagaagatcatttgagttaaagaagctgtgggatcaaacccaatcaaaggtagggctattagtatcagatggaggaccaaacttatacaatatacggaatcttcacattcctgaagtctgcttaaaatgggagctaatggatgatgattatcggggaagactttgtaatcccctgaatccctttgtaagtcataaggagattgattctgtaaacaatgctgtggtaatgccagcccatggtccagccaaaagcatggaatatgatgcagttgcaactacacattcctggattcccaagaggaatcgttctattctcaacacaagccaaaggggaattcttgaggatgaacagatgtaccagaagtgttgcaatctattcgagaaatttttccctagcagttcatataggagaccggttggaatttctagcatggtggaggccatggtatctagggcccggattgatgccagggtcgactttgagtctggacggatcaagaaagaagagttctctgagatcatgaagatctgttccaccattgaagaactcagacggcaaaaataa

>H1N1_PB1_KF897783_A/Lyon/52.16/2010

atggatgtcaatccgactctacttttcctgaaaattccagcgcaaaatgccataagcaccacattcccttatactggagatcctccatacagccatggaacaggaacaggatacaccatggacacagtaaacagaacacaccaatactcagaaaagggaaagtggacgacaaacacagagactggtgcaccccagctcaacccgattgatggaccactacctgaggataatgaaccaagtgggtatgcacaaacagactgtgttctagaggctatggctttccttgaagaatcccacccaggaatatttgagaattcatgccttgaaacaatggaagttgttcaacaaacaagggtagataaactaactcaaggtcgccagacttatgattggacattaaacagaaatcaaccggcagcaactgcattggccaacaccatagaagtctttagatcgaatggcctaacagctaatgagtcaggaaggctaatagatttcttaaaggatgtaatggaatcaatgaacaaagaggaaatagagataacaacccactttcaaagaaaaaggagagtaagagacaacatgaccaagaagatggtcacgcaaagaacaatagggaagaaaaaacaaaaactgaataagagaggctatctaataagagcactgacgttaaatacgatgaccaaagatgcagagagaggcaagttaaaaagaagggctatcgcaacacctgggatgcagattagaggtttcgtatactttgttgaaactttagctaggagcatttgcgaaaagcttgaacagtctgggctcccagtagggggcaatgaaaagaaggccaaactggcaaatgttgtgagaaagatgatgactaattcacaagacacagagatttctttcacaatcactggggacaacactaagtggaatgaaaatcaaaatcctcgaatgttcctggcgatgattacatatatcaccagaaatcaacccgagtggttcagaaacatcctgagcatggcacccataatgttctcaaacaaaatggcaagactagggaaagggtacatgttcgagagtaaaagaatgaagattcggacacaaataccagcagaaatgctagcaagcattgacctgaagtacttcaatgaatcaacaaagaagaaaattgagaaaataaggcctcttctaatagatggcacagcatcactgagtcctgggatgatgatgggcatgttcaacatgctaagtacggtcttgggagtctcgatactgaatcttggacaaaagaaatacaccaagacaatatactggtgggatgggctccaatcatccgacgattttgctctcatagtgaatgcaccaaaccatgagggaatacaagcaggagtggacagattctacaggacctgcaagttagtgggaatcaacatgagcaaaaagaagtcctatataaataagacagggacatttgaattcacaagctttttttatcgctatggatttgtggctaattttagcatggagctacccagctttggagtgtctggagtaaatgaatcagctgacatgagtattggagtaacagtgataaagaacaacatgataaacaatgaccttggacctgcaacggcccagatggctcttcaattgttcatcaaagactacagatacacatataggtgccataggggagacacacaaattcagacgagaagatcatttgagttaaagaagctgtgggatcaaacccaatcaaaggtagggctattagtatcagatggaggaccaaacttatacaatatacggaatcttcacattcctgaagtctgcttaaaatgggagctgatggatgatgattatcggggaagactttgtaatcccctgaatccctttgtcagtcataaagagattgattctgtaaacaatgctgtggtaatgccagcccatggtccagtcaaaagcatggaatatgatgccgttgcaactacacattcctggattcccaagaggaatcgttctattctcaacacaagccaaaggggaattcttgaggatgaacagatgtatcagaagtgctgcaatctattcgagaaatttttccctagcagttcatataggagaccggttggaatttctagcatggtggaggccatggtgtctagggcccggattgatgccagggtcgacttcgagtctggacggatcaagaaagaagagttctctgagatcatgaagatctgttccaccattgaagaactcagacggcaaaaataa

>H1N1_PB1_KF897807_A/Lyon/1.12/2011

atggatgtcaatccgactctacttttcctaaaaattccagcgcaaaatgccataagcaccacattcccttatactggagatcctccatacagccatggaacaggaacaggatacaccatggacacagtgaacagaacacaccaatactcagaaaagggaaagtggacgacaaacacagagactggtgcaccccagctcaacccgattgatggaccattacctgaggataatgaaccaagtgggtatgcacaaacagactgtgttctagaggctatggctttccttgaagaatcccacccagggatatttgagaattcatgccttgaaacaatggaagttgttcaacaaacaagggtagataaactaactcaaggtcgccagacttatgattggacattaaacagaaatcaaccggcagcaactgcattggccaacaccatagaagtctttagatcgaatggcctaacagctaatgagtcaggcaggctaatagatttcttaaaggatgtaatggaatcaatgaacagagaggaaatagagataacaacccactttcaaagaaaaaggagagtaagagacaacatgaccaagaagatggtcacgcaaagaacaatagggaagaaaaaacaaagactgaataagagaggctatctaataagagcactgacattaaatacgatgaccaaagatgcagagagaggcaagttaaaaagaagggctatcgcaacacctgggatgcagattagaggtttcgtatactttgttgaaactttagctaggagcatttgcgaaaagcttgaacagtctgggctcccagtagggggcaatgaaaagaaggccaaactggcaaatgttgtgagaaagatgatgactaattcacaagacacagagatttctttcacaatcactggggacaacactaagtggaatgaaaatcaaaatcctcgaatgttcctggcgatgattacatatatcaccagaaatcaacccgagtggttcagaaacatcctgagcatggcacccataatgttctcaaacaaaatggcaagactagggaaagggtacatgttcgagagtaaaagaatgaagattcgaacacaaataccagcagaaatgctagcaagcattgacctgaagtacttcaatgaatcaacaaagaagaaaattgagaaaataaggcctcttttaatagatggcacagcatcactgagtcctgggatgatgatgggcatgttcaacatgctaagtacggtcttgggagtctcgatactgaatcttggacaaaagaaatacaccaagacaatatactggtgggatgggctccaatcatccgacgattttgctctcatagtgaatgcaccaaaccatgagggaatacaagcaggagtggacagattctacaggacctgcaagttagtgggaatcaacatgagcaaaaagaagtcctatataaataaaacagggacatttgaattcacaagctttttttatcgctatggatttgtggctaattttagcatggagctacccagctttggagtgtctggagtaaatgaatcagctgacatgagtattggagtaacagtgataaagaacaacatgataaacaatgaccttggacctgcaacggcccagatggctcttcaattgttcatcaaagactacagatacacatataggtgccataggggagacacacaaattcagacgagaagatcatttgagttaaagaagctgtgggatcaaacccaatcaaaggtagggctattagtatcagatggaggaccaaacttatacaatatacggaatcttcacattcctgaagtctgcttaaaatgggagctaatggatgatgattatcggggaagactttgtaatcccctgaatccctttgtaagtcataaagagattgattctgtaaacaatgctgtggtaatgccagcccatggtccagccaaaagcatggaatatgatgccgttgcaactacacattcctggattccaaagaggaatcgttctattctcaacacaagccaaaggggaattcttgaggatgaacagatgtaccagaagtgctgcaatctattcgagaaatttttccctagcagttcatataggagaccggttggaatttctagcatggtggaggccatggtgtctagggcccggattgatgccagggtcgactttgagtctggacggatcaagaaagaagagttctctgagatcatgaagatctgttccaccattgaagaactcagacggcaaaaataa

>H1N1_PB1_CY172565_A/New York/1052/2006

atggatgtcaatccgacattacttttcttaaaagtgccagcacaaaatgctataagcacaacttttccttatactggtgaccctccttacagccatgggacaggaacagggtacaccatggatacagtcaacaggacacaccagtactcagaaagaggaagatggacaaaaaataccgaaactggagcaccgcaactcaacccaattgatgggccactaccagaagacaatgaaccgagtggctatgcccaaacagattgtgtattagaagcaatggctttccttgaagaatcccatcctggtatctttgaaaactcttgtattgagacaatggaggttgttcagcaaacaagggtggacaaactaacacaaggcagacagacctatgactggactctaaataggaaccagcctgctgccacagcattggcaaacactatagaagtattcagatcaaacggcctcatagcaaatgaatctgggaggctaatagacttccttaaagatgtaatggagtcgatggacatagacgaaatagaggtcacaactcattttcaaagaaagaggagaatgagagacaatgtaactaaaaaaatggtgacccaaagaacaataggcaaaaagaaacataaattagacaaaagaagttacctaattagggcattaaccctgaacacaatgaccaaagatgctgagagagggaaactaaaacgcagagcaattgcaaccccaggaatgcaaataagggggtttgtatactttgttgagacactggcaagaagcatatgtgaaaagcttgaacaatcaggattgccagttggaggaaatgagaagaaagcaaagttagcaaatgttgtaaggaagatgatgaccaactcccaggacactgaaatttctttcaccatcactggagataacacaaaatggaacgaaaatcagaaccctagaatgttcttggccatgatcacatatataaccaaaaatcagcctgaatggttcagaaacattctaagtattgccccaataatgttttcaaacaagatggcgagactaggtaaggggtacatgtttgaaagcaagagcatgaaactgagaactcaaatacctgcagagatactagccaacatagatttgaaatatttcaatgattcaactaaaaagaaaattgaaaaaatccgaccattattaatagatggaactgcatcattgagtcctggaatgatgatgggcatgttcaatatgttgagcaccgtcttgggcgtctccattctgaatcttgggcaaaagagatacaccaagactacttactggtgggatggtcttcaatcgtctgatgattttgctttgattgtgaatgcacccaaccatgcaggaattcaagctggagttgacaggttttatcgaacctgtaagctgctcggaattaatatgagcaaaaagaagtcttacataaacagaacaggtacctttgaattcacgagctttttctatcgttatgggtttgttgccaatttcagcatggagcttcctagttttggggtgtctggggtcaatgaatctgcagacatgagtattggagtcactgtcatcaaaaacaatatgataaacaatgaccttggcccagcaactgctcaaatggcccttcagttatttataaaagattacaggtacacttatcgatgccacagaggtgacacacaaatacaaacccggagatcatttgagataaagaaactatgggaccaaacccgctccaaagctgggctgttggtctctgatggaggccccaatttatataacattagaaatctccatattcctgaagtttgcttgaaatgggagttgatggatgaggattaccaggggcgtttatgcaacccattaaacccgtttgtcagccataaagagattgaatcagtaaacaatgcagtgataatgccggcacatggtccagccaaaaatatggagtatgacgctgttgcaacaacacactcctgggtccccaaaagaaatcgatccattttgaacacgagccaaagggggatacttgaagatgagcaaatgtatcagaggtgctgcaatttatttgaaaaattcttcccaagtagctcatacagaagaccagttggaatatccagtatggtagaggctatggtttcaagagcccgaattgatgcacggattgatttcgaatctggaaggataaagaaagaggaatttgctgagatcatgaagatctgttccaccattgaagacctcagacggcaaaaatag

>H1N1_PB1_CY172637_A/New York/1062/2007

atggatgtcaatccgacattacttttcttaaaagttccagcacaaaatgctataagcacaacttttccttatactggtgaccctccttacagccatgggacaggaacagggtacaccatggatacagtcaacaggacacatcagtactcagaaagaggaagatggacaaaaaataccgaaactggagcaccgcaactcaacccaattgatgggccactaccagaagacaatgaaccaagtggctatgcccaaacagattgtgtattagaagcaatggctttccttgaagaatcccatcctggtatttttgaaaactcctgtattgaaacaatggaggttgttcagcaaacaagggtggacaaactgacacaaggcagacagacctatgactggactctaaataggaaccagcctgctgccacagcattggccaacactatagaagtattcagatcaaacggcctcatagcaaatgaatctgggaggctaatagacttccttaaagatgtaatggagtcgatggacagagacgaagtagaggtcacaactcattttcagagaaagaggagagtgagagacaatgtaactaaaaaaatggtgacccaaagaacaataggcaaaaagaaacataaattagccaaaaggagttacctaattagagcattaaccctgaacacaatgaccaaagatgctgagagggggaaattaaaacgcagagcaattgcaaccccaggaatgcaaataagggggtttgtatattttgttgagacactggcaagaagcatatgtgaaaagcttgaacaatcagggttgccagttggaggaaatgagaagaaagcaaagttagcaaatgttgtaaggaagatgatgaccaactcccaggacactgaaatttctttcaccatcactggagataacacaaaatggaacgaaaatcaaaaccctagaatgtttttggccatgatcacatacataaccaaaaatcagcctgaatggttcagaaatattctaagtattgctccaataatgttttcaaacaaaatggcgagactaggtaaggggtacatgtttgaaagcaagagtatgaaactgagaactcaaatacctgcggagatgctagccgacatagatttgaaatatttcaatgagtcaactaaaaagaaaattgaaaaaattcgaccattattaatagatggaactgcatcattgagtcctggaatgatgatgggcatgttcaatatgttaagcactgtcttgggcgtctccattctgaatcttgggcaaaagagatacaccaagactacttactggtgggatggtcttcaatcgtctgatgattttgctctgatagtgaatgcgcccaactatgctggaattcaagctggagttgacaggttttatcgaacctgtaagctgctcggaattaatatgagcaaaaagaagtcttacataaacaggacaggtacctttgaattcacgagctttttctatcgttatgggtttgttgccaatttcagcatggagcttcctagttttggggtgtctggggtcaatgaatctgcagacatgagtattggagtcactgtcatcaaaaataatatgataaacaatgaccttggcccagcaactgctcaaatggcccttcagttatttataaaagattacagatacacgtatcgatgccacagaggtgacacacaaatacaaacccggagatcatttgagataaagaaattatgggaccaaacccgctccaaagctgggctgttggtttctgatggaggccccaatttatataacattagaaatctccatattcctgaagtctgcttgaaatgggagttgatggatgaagattaccaggggcgtttatgcaacccattgaacccgtttgtcagccataaagagattgaatcagtgaacaatgcagtggtgatgccggcacatggtccagccaaaaatatggagtatgacgctgttgcaacaacacactcctgggttcccaaaaggaatcgatccattttgaacacgagccaaagggggatacttgaggatgagcaaatgtatcagaggtgctgcaatttatttgaaaaattcttcccaagtagctcatacaggagaccagttgggatatccagtatggtagaggctatggtttccagagccagaattgacgcacggattgatttcgaatctggaaggataaagaaagaggaattcgctgagatcatgaagacctgttccaccattgaagacctcagacggcaaaaatag

>H1N1_PB1_CY172941_A/New York/1108/2008

atggatgtcaatccgacattacttttcttaaaagtgccagcacaaaatgctataagcacaacttttccttatactggtgaccctccttacagccatgggacaggaacagggtacaccatggatacagtcaacaggacacaccagtactcagaaagaggaagatggacaaaaaataccgaaacaggagcaccgcaacttaacccaattgatggtcccctaccggaagacaatgaaccaagtggctatgcccaaacagattgtgtattagaagcaatggctttccttgaagaatcccatcccggtatctttgaaaactcttgtattgaaacaatggaggttgttcagcaaacaagggtggacaaactgacacaaggcagacagacctatgactggactctaaataggaaccagcctgctgccacagcattggcaaacactatagaagtattcagatcaaacggcctcatagcaaatgaatctgggaggctaatagacttccttaaagatgtaatggagtcgatggacagaggcgaagtagaggtcacaactcattttcaaagaaagaggagagtgagagacaatgtaactaaaaaaatggtgacccaaagaacaataggcaaaaagaaacataaattagacaaaagaagttacctaattagggcattaaccctgaacacaatgaccaaagatgctgagagggggaaactaaaacgcagagcaattgcaaccccaggaatgcaaataagggggtttgtatactttgttgagacactggcaagaagcatatgtgaaaagcttgaacaatcaggattgccagttggaggaaatgagaagaaagcaaagttagcaaatgttgtaaggaagatgatgaccaactcccaggacactgaaatttctttcaccataaccggagataacacaaaatggaacgaaaatcaaaaccctagaatgttcttggccatgatcacatatataaccaaaaatcagcctgaatggttcagaaatattctaagtattgctccaataatgttttcaaacaagatggcgaggctaggtaaggggtacatgtttgaaagcaagagtatgaaactgagaactcaaatacctgcagagatgctagccaacatagatttgaaatatttcaatgattcaactaaaaagaaaattgaaaaaatccgaccattattaatagatggaactgcatcattgagtcctggaatgatgatgggcatgttcaatatgttgagcaccgtcttgggcgtctccattctgaatcttgggcaaaagagatacaccaagactacttactggtgggatggtcttcaatcgtctgatgattttgctttgattgtgaatgcacccaactatgcaggaattcaagctggagttgacaggttttatcgaacctgtaagctgctcggaattaatatgagcaaaaagaagtcttacataaacagaacaggtacctttgaattcacgagctttttctatcgttatgggtttgttgccaatttcagcatggagcttcctagttttggggtgtctggggtcaatgaatctgcagacatgagtattggggtcactgtcatcaaaaacaatatgataaacaatgaccttggcccagcaactgctcaaatggcccttcagttatttataaaagattacaggtacacttatcgatgccaccgaggtgacacacaaatacaaacccggagatcatttgagataaagaaactatgggaccaaacccgctcaaaagctgggctgttggtctctgatggaggccccaatttatataacattagaaatctccatattcctgaagtttgcttgaaatgggagttgatggatgaggattaccaggggcgtttatgcaacccattaaacccgtttgtcagccataaagagattgaatcagtgaacaatgcagtgataatgccggcacatggtccagccaaaaatatggagtatgacgctgttgcaacaacacactcctgggtccccaaaagaaatcgatccattttgaacacgagccaaagggggatacttgaagatgagcaaatgtatcagaggtgctgcaatttatttgaaaaattcttcccaagtagctcatacagaagaccagttggaatatccagtatggtagaggctatggtctcaagagcccgaattgatgcacggattgatttcgaatctggaaggataaagaaagaggaatttgctgagatcatgaagatctgttccaccattgaagacctcagacggcaaaaatga

>H1N1_PB1_CY170093_A/Boston/YGA_01218/2013

atggatgtcaatccgaccctacttttcctaaaaattccagcacaaaatgccataagcaccacattcccttatactggagatcctccatacagccatggaacaggaacaggatacaccatggacacagtaaacagaacacaccaatactcagaaaggggaaagtggacaacaaacacagagactggtgcaccccagctcaacccgattgatggaccactacctgaggataatgaaccaagtgggtatgcacaaacagactgtgttctagaagctatggctttccttgaagaatcccacccaggaatatttgagaattcatgccttgaaacaatggaagttgttcaacaaacaagggtagataaactgactcaaggtcgccagacttatgattggacattaaacagaaatcaaccggcagcaactgcattagccaacaccatagaagtctttagatcgaatgacctaacagctaacgagtcaggaaggctaatagatttcttaaaggatgtaatggaatcaatgaacaaagaggaaatagagataacaacccactttcaaagaaaaaggagagtaagagacaacatgaccaagaagatggtcacgcaaagaacaatagggaagaaaaaacagagactgaataagagaggctatctaataagagcactgacattaaatacgatgaccaaagatgcagagagaggcaagttaaaaagaagggctatcgcaacacctgggatgcagattagaggtttcgtatactttgttgaaactttagctaggagcatttgcgaaaagcttgaacagtctgggctcccagtagggggcaatgaaaagaaggccaaattggcaaatgttgtgagaaagatgatgactaattcacaagatacagagatttctttcacaatcactggggacaacactaagtggaatgaaaatcaaaatcctcgaatgttcctggcgatgattacatatatcaccagaaatcaacccgagtggtttagaaacatcctgagcatggcacccataatgttctcaaataaaatggcaagactagggaaagggtacatgttcgagagtaaaagaatgaagattcgaacacaaataccagcagaaatgctagcaagcattgatctgaagtacttcaatgaatcaacaaagaagaaaattgagaaaataaggcctcttttaatggatggcacagcatcactgagtcctgggatgatgatgggcatgttcaacatgctaagtacggtcttgggagtctcgatactgaatcttggacaaaagaaatacaccaagacaacatactggtgggatgggctccaatcatccgacgattttgctctcatagtgaatgcaccaaaccatgagggaatacaagcaggagtggacagattctacaggacctgcaagttagtgggaatcaacatgagcaaaaagaagtcctatataaataagacagggacatttgaattcacaagctttttttatcgctatggatttgtggctaattttagcatggagctacccagctttggagtgtctggaataaatgaatcagctgacatgagtattggagtaacagtgataaagaacaacatgataaacaatgaccttggacctgcaacggcccagatggctcttcaattgttcatcaaagactacagatacacatataggtgccatagaggagacacacaaattcagacgagaagatcatttgagttaaagaagctgtgggatcaaacccaatcaaaggtagggctattagtatcagatggaggaccaaacttatacaatatacggaatcttcacattcctgaagtctgcttaaaatgggagctaatggatgatgattatcggggaagactttgtaatcccctgaatccctttgtgagtcataaggagattgactctgtaaacaatgctgtggtaatgccagcccatggtccagccaaaagcatggaatatgatgccgttgcaactacacattcctggattcccaagaggaatcgttctattctcaacacaagccaaaggggaattcttgaggatgaacagatgtaccagaagtgttgcaatctattcgagaaatttttccctagcagttcatataggagaccggttggaatttctagcatggtggaggccatggtgtctagggcccggattgatgccagggtcgactttgagtctggacggatcaagaaagaagagttctctgagatcatgaagatctgttccaccattgaagaactcagacggcaaaaataa

>H1N1_PB1_CY170701_A/Santa Clara/YGA_03033/2013

atggatgtcaatccgactctacttttcctaaaaattccagcacaaaatgccataagcaccacattcccttatactggagatcctccatacagccatggaacaggaacaggatacaccatggacacagtaaacagaacacatcaatactcagaaaagggaaagtggacaacaaacacagagactggtgcaccccagctcaacccgattgatggaccactccctgaggataatgaaccaagtgggtatgcacaaacagactgtgttctagaggctatggctttccttgaagaatcccacccaggaatatttgagaattcatgccttgaaacaatggaagttgttcaacaaacaagggtagacaaactgactcaaggtcgccagacttatgattggacattaaacagaaatcaaccggcagcaactgcattggccaacaccatagaagtatttagatcgaatgacctaacagctaatgagtcaggaaggctaatagatttcttaaaagatgtaatggaatcaatgaacaaagaggaaatagagataacaacccactttcaaagaaaaaggagagtaagagacaacatgaccaagaagatggtcacgcaaagaacaatagggaagaaaaaacagagactgaataagagaggctatctaataagagcactgacattaaatacgatgaccaaagatgcagagagaggcaagttaaaaagaagggctatcgcaacacctgggatgcagattagaggtttcgtatactttgttgaaactttagctaggagcatttgcgaaaagcttgaacagtctgggctcccagtagggggcaatgaaaagaaggccaaactggcaaatgttgtgagaaagatgatgactaattcacaagacacagagatttctttcacaatcactggggacaacactaagtggaatgaaaatcaaaatcctcgaatgttcctggcgatgattacatatatcaccagaaatcaacccgagtggtttagaaacatcctgagcatggcacccataatgttctcaaacaaaatggcaagactagggaaagggtacatgttcgagagtaaaagaatgaagattcgaacacaaataccagcagaaatgctagcaagcattgacctgaagtacttcaatgaatcaacaaagaagaaaattgagaaaataaggcctcttttaatggatggcacagcatcactgagtcctgggatgatgatgggcatgttcaacatgctaagtacggtcttgggagtctcgatactgaatcttggacaaaagaaatacaccaagacaacatactggtgggatgggctccaatcatccgacgattttgctctcatagtgaatgcaccaaactatgagggaatacaagcaggagtggacagattctacaggacctgcaagttagtgggaatcaacatgagcaaaaagaagtcctatataaataagacagggacatttgaattcacaagctttttttatcgctatggatttgtggctaattttagcatggagctacccagctttggagtgtctggggtaaatgaatcagctgacatgagtattggagtaacagtgataaagaacaacatgataaacaatgaccttggacctgcaacggcccagatggctcttcaattgttcatcaaagactacagatacacatataggtgccataggggagacacacaaattcagacgagaagatcatttgagttaaagaagctgtgggatcaaacccaatcaaaggtagggctattagtatcagatggaggaccaaacttatacaatatacggaatcttcacattcctgaagtctgcttaaaatgggagctaatggatgatgattatcggggaagactttgtaatcccctgaatccctttgtaagtcataaggagattgattctgtaaacaatgctgtggtaatgccagcccatggtccagccaaaagcatggaatatgatgccgttgcaactacacattcctggattcccaagaggaatcgttctattctcaacacaagccaaaggggaattcttgaggatgaacagatgtaccagaagtgctgcaatctattcgagaaatttttccctagcagttcatataggagaccggttggaatttctagcatggtggaggccatggtgtctagggcccggattgatgccagggtcgactttgagtctggacggatcaagaaagaagagttctctgagatcatgaagatctgttccaccattgaagaactcagacggcaaaaataa

>H1N1_PB1_CY176544_A/Khon Kaen/INS3_649/2012

atggatgtcaatccgactctacttttcctaaaaattccagcgcaaaatgccataagcaccacattcccttatactggagatcctccatacagccatggaacaggaacaggatacaccatggacacagtaaacagaacacaccaatactcagaaaagggaaagtggacgacaaacacagagactggtgcaccccagctcaacccgattgatggaccactacctgaggataatgaaccaagtgggtatgcacaaacagactgtgttttagaggctatggctttccttgaagaatcccacccaggaatatttgagaattcatgccttgaaacaatagaagttgttcaacaaacaagggtagataaactgactcaaggtcgccagacttatgattggacattaaacagaaatcaaccggcagcaactgcattggccaacaccatagaagtctttagatcgaatggcctaacagctaatgagtcgggaaggctaatagatttcttaaaggatgtaatggaatcaatgaacaaagaggaaatagagataacaacccactttcaaagaaaaaggagagtaagagacaacatgaccaagaagatggtaacgcaaagaacaatagggaagaaaaaacagagactgaataagagaggctatctaataagagcactaacattaaatacgatgaccaaagatgcagagagaggcaagttaaaaagaagggctatcgcaacacctgggatgcagattagaggtttcgtatactttgttgaaactttagctaggagcatttgcgaaaagcttgaacagtctgggctcccagtagggggcaatgaaaagaaggccaagctggcaaatgttgtgagaaagatgatgactaattcacaagacacagagatttctttcacaatcactggggacaacactaagtggaatgaaaatcaaaatcctcgaatgttcctggcgatgattacatatatcaccagagatcaacccgaatggtttagaaacatcctgagcatggcacccataatgttctcaaacaaaatggcacgactagggaaagggtacatgttcgagagtaaaagaatgaagattcgaacacaaataccagcagaaatgctagcaagcattgacctgaagtacttcaatgaatcaacaaagaagaaaattgagaaaataaggcctcttttaatggatggcacagcatcactgagtcctgggatgatgatgggcatgttcaacatgctaagtacggtcttgggagtctcgatactgaatcttggacaaaagaaatacaccaagacaacatactggtgggatgggctccaatcatccgacgattttgccctcatagtgaatgcaccaaatcatgaggggatacaagcaggagtggacagattctacaggacctgcaagttagtgggaatcaacatgagcaaaaagaagtcctatataaataagacagggacatttgaattcacaagctttttttatcgctatggatttgtggctaattttagcatggagctacccagctttggagtgtctggagtaaatgaatcagctgacatgagtattggagtaacagtgataaagaacaacatgataaacaatgaccttggacctgcaacggcccagatggccctccaattgttcatcaaagactacagatacacatataggtgccataggggagacacacaaattcagacgagaagatcatttgagttaaagaagctgtgggatcaaacccaatcaaaggtagggctattagtatcagatggaggaccaaacttatacaatatacggaatcttcacatccctgaagtctgcttaaaatgggagctaatggatgatgattatcggggaagactttgtaatcccctgaatccctttgtaagtcataaggagattgattctgtaaacaatgctgtggtaatgccagcccatggtccagccaaaagcatggaatatgatgccgttgcaacaacacattcctggattcccaagaggaatcgttctattctcaacacaagccaaaggggaattcttgaggatgaacagatgtatcagaagtgctgcaatctattcgagaaatttttccctagcagttcatataggagaccggttggaatttctagcatggtggaggccatggtgtctagggcacgaattgatgccagggtcgactttgagtctggacggatcaagaaagaagagttctctgagatcatgaagatctgttccaccattgaagaactcagacggcaaaaataa

>H1N1_PB1_CY176624_A/Westmead/INS3_661/2011

atggatgtcaatccgactctacttttcctaaaaattccagcgcaaaatgccataagcaccacattcccttatactggagatcctccatacagccatggaacaggaacaggatacaccatggacacagtaaacagaacacaccaatactcagaaaagggaaagtggacgacaaacacagagactggtgcaccccagctcaacccgattgatggaccactacctgaggataatgaaccaagtgggtatgcacaaacagactgtgttctagaggctatggctttccttgaagaatcccacccaggaatatttgagaattcatgccttgaaacaatggaagttgttcaacaaacaagggtagataaactgactcaaggtcgccagacttatgattggacattaaacagaaatcaaccggcagcaactgcattggccaacaccatagaagtctttagatcgaatggcctaacagctaatgagtcaggaaggctaatagatttcttaaaggatgtaatggaatcaatgaacaaagaggaaatagagataacaacccactttcaaagaaaaaggagagtaagagacaacatgaccaagaagatggtcacgcaaagaacaatagggaagaaaaaacagagactgaataagagaggctatctaataagagcactaacattaaatacgatgaccaaagatgcagagagaggcaagttaaaaagaagggctatcgcaacacctgggatgcagattagaggtttcgtatactttgttgaaactttagctaggagcatttgcgaaaagcttgaacagtctgggctcccagtagggggcaatgaaaagaaggccaaactggcaaatgttgtgagaaagatgatgactaattcacaagacacagagatttctttcacaatcactggggacaacactaagtggaatgaaaatcaaaatcctcgaatgttcctggcgatgattacatatatcaccagaaatcaacccgagtggtttagaaacatcctgagcatggcacccataatgttctcaaacaaaatggcaagactagggaaagggtacatgttcgagagtaaaagaatgaagattcgaacacaaataccagcagaaatgctagcaagcattgacctgaagtacttcaatgaatcaacaaagaagaaaattgagaaaataaggcctcttttaatggatggcacagcatcactgagtcctgggatgatgatgggcatgttcaacatgctaagtacggtcttgggagtctcgatactgaatcttggacaaaagaaatacaccaagacaacatactggtgggatgggctccaatcatccgacgattttgccctcatagtgaatgcaccaaatcatgagggaatacaagcaggagtggacagattctacaggacctgcaagttagtgggaatcaacatgagcaaaaagaagtcctatataaataagacagggacatttgaattcacaagctttttttatcgctatggatttgtggctaattttagcatggagctacccagctttggagtgtctggagtaaatgaatcagctgacatgagtattggagtaacagtgataaagaacaacatgataaacaatgaccttggacctgcaacggcccagatggctctccaattgttcatcaaagactacagatacacatataggtgccataggggagacacacaaattcagacgagaagatcatttgagttaaagaagctgtgggatcaaacccaatcaaaggtagggctattagtatcagatggaggaccaaacttatacaatatacggaatcttcacattcctgaagtctgcttaaaatgggagctaatggatgatgattatcggggaagactttgtaatcccctgaatccctttgtaagtcataaggagattgattctgtaaacaatgctgtggtaatgccagcccatggtccagccaaaagcatggaatatgatgccgttgcaactacacattcctggattcccaagaggaatcgttctattctcaacacaagccaaaggggaattcttgaggatgaacagatgtaccagaagtgctgcaatctattcgagaaatttttccctagcagttcatataggagaccggttggaatttctagcatggtggaggccatggtgtctagggcccggattgatgccagggtcgactttgagtctggacggatcaagaaagaagagttctctgagatcatgaagatctgttccaccattgaagaactcagacggcaaaaataa

>H1N1_PB1_CY176712_A/Minneapolis/INS3_675/2012

atggatgtcaatccgactctacttttcctaaaaattccagtgcaaaatgccataagcaccacattcccttatactggagatcctccatacagccatggaacaggaacaggatacaccatggacacagtaaacagaacacaccaatactcagaaaagggaaggtggacgacaaacacagagactggtgcaccccagctcaacccgattgatggaccactacctgaggataatgaaccaagtggatatgcacaaacagactgtgttctagaggctatggctttcctcgaagaatcccacccaggaatatttgagaattcatgccttgaaacaatggaagctgttcaacaaacaagggtagataaactaactcaaggtcgccagacttatgattggacattgaacagaaatcaaccggcagcaactgcattggccaacaccatagaagtctttagatcgaatggcctaacagctaatgagtcaggaaggctaatagatttcttaaaggatgtgatggaatcaatgaacaaagaggaaatagagataacaacccactttcaaagaaaaaggagagtaagagacaacatgaccaagaagatggtcacgcaaagaacaataggaaagaaaaagcaaagattgaataagagaggctatctaataagagcactgacattaaatacgatgaccaaagatgcagagagaggcaagttaaaaagaagggctatcgcaacacctgggatgcagattagaggtttcgtatactttgttgaaactttagccaggagcatttgcgaaaagcttgaacagtctgggctcccagtagggggcaatgaaaagaaggccaaactggcaaatgttgtgagaaagatgatgactaattcacaagacacagagatttctttcacaatcactggggacaacactaagtggaatgaaaatcaaaatcctcgaatgttcctggcgatgattacatatataaccagaaatcaacccgagtggttcagaaacatcctgagcatggcacccataatgttctcaaacaaaatggcaagactagggaaagggtacatgttcgagagtaaaagaatgaagattcgaacacaaattccagcagaaatgctagcaagcattgacctgaagtacttcaatgaatcaacaaagaggaaaattgagaaaataaggcctcttctaatagatggcacagcatcactgagtcctgggatgatgatgggcatgttcaacatgctaagtacggtcttgggagtctcgatactgaatcttggacaaaagaaatacaccaagacaacatactggtgggatgggctccaatcatccgacgattttgctctcatagtgaatgcaccaaaccatgagggaatacaagcaggagtggacagattctacaggacctgcaagttagtgggaatcaacatgagcaaaaagaagtcatatataaataaaacagggacatttgaattcacaagctttttttatcgctatggatttgtggctaattttagcatggagctacccagctttggagtgtctggagtaaatgaatcaactgacatgagtattggagtaacagtgataaagaacaacatgataaacaatgaccttggacctgcaacggcccagatggctcttcaattgttcatcaaagactacagatacacatataggtgccataggggagacacacaaattcagacgagaagatcatttgagttaaaaaagctgtgggatcaaacccaatcaaaggtagggctattagtatcagatggaggaccaaacttatacaatatacggaatcttcacattcctgaagtctgcttaaaatgggagctaatggatgatgattatcggggaagactttgcaatcccctgaatccctttgtcagtcataaagagattgattctgtaaacaatgctgtggtaatgccagcccatggtccagccaaaagcatggaatatgatgccgttgcaactacacattcctggattcccaagaggaatcgttctattctcaacacaagccaaaggggaattcttgaggatgaacagatgtaccagaagtgctgcaatctattcgagaaatttttccctagcagttcatataggagaccggttggaatttctagcatggtggaggccatggtgtctagggcccggattgatgccagggtcgacttcgagtctggacggatcaagaaagaagagttctctgagatcatgaagatctgttccaccattgaagaactcagacggcaaaaataa

>H1N1_PB1_CY176720_A/Dayton/INS3_676/2012

atggatgtcaatccgactctacttttcctaaaaattccagcgcaaaatgccataagcatcacattcccttatactggagatcctccatacagccatggaacaggaacaggatacaccatggacacagtaaacagaacacaccaatactcagaaaagggaaagtggacgacaaacacagagactggtgcaccccagctcaacccgattgatggaccactacctgaggataacgaaccaagtgggtatgcacaaacagactgtgtcctagaggctatggctttccttgaagaatcccacccaggaatatttgagaattcatgccttgaaacaatggaagttgttcaacaaacaagagtagataaactgactcaaggtcgccagacttatgattggacattaaacagaaatcaaccggcagcaactgcattggccaacaccatagaagtctttagatcgaatggcctaacagctaatgagtcaggaaggctagtagatttcttaaaggatgtaatggaatcaatgaacaaagaggaaatagagataacaacccactttcaaagaaaaaggagagtaagagacaacatgaccaagaagatggtcacgcaaagaacaatagggaagaaaaaacagagactgaataagagaggctatctaataagagcactgacattaaatacgatgaccaaagatgcagagagaggcaagttaaaaagaagggctatcgcaacacctgggatgcagattagaggtttcgtatactttgttgaaactttagctaggagcatttgcgaaaagcttgaacagtctgggctcccagtagggggcaatgaaaagaaggccaaactggcaaatgttgtgagaaagatgatgactaattcacaagacacagagatttctttcacaatcactggggacaacactaagtggaatgaaaatcaaaaccctcgaatgttcctggcgatgattacatatatcaccaaaaatcaacccgagtggtttagaaacatcctgagcatggcacccataatgttctcaaacaaaatggcaagactagggaaagggtacatgttcgagagtaaaagaatgaagattcgaacacaaataccagcagaaatgctagcaagcattgacctgaagtacttcaatgaatcaacaaagaagaaaattgagaaaataaggcctcttttaatggatggcacagcatcactgagtcctgggatgatgatgggcatgttcaacatgctaagtacggtcttgggagtctcgatactgaatcttggacaaaagaaatacaccaagacaacatactggtgggatgggctccaatcatccgacgattttgctctcatagtgaatgcaccaaaccatgagggaatacaagcaggagtggacagattctacaggacctgcaagttagtgggaatcaacatgagcaaaaagaagtcctatataaataagacagggacatttgaattcacaagctttttttatcgctatggatttgtggctaattttagcatggagctacccagctttggagtgtctggagtaaatgaatcagctgacatgagtattggagtaacagtgataaagaacaacatgataaacaatgaccttggacctgcaacagcccagatggctcttcaattgttcatcaaagactacagatacacatataggtgccataggggagacacacaaattcagacgagaagatcatttgagttaaagaagctgtgggatcaaacccaatcaaaggtagggctattagtatcagatggaggaccaaacttatacaatatacggaatcttcacattcctgaagtctgcttaaaatgggagctaatggatgatgattatcggggaagactttgtaatcccctgaatccctttgtaagtcataaggagattgattctgtaaacaatgctgtggtaatgccagcccatggtccagccaaaagcatggaatatgatgccgttgcaactacacattcctggattcccaagaggaatcgttctattctcaacacaagccaaaggggaattcttgaggatgaacagatgtaccagaaatgctgcaatctattcgagaaatttttccctagcagttcatataggagaccggttggaatttctagcatggtggaggccatggtgtctagggcccggattgatgccagggtcgactttgagtctggacggatcaagaaagaagagttctctgagatcatgaagatctgttccaccattgaagaactcagacggcaaaaataa

>H1N1_PB1_CY176728_A/Lima/INS3_677/2012

atggatgtcaatccgactctacttttcctaaaaattccagcgcaaaatgccataagcatcacattcccttatactggagatcctccatacagccatggaacaggaacaggatacaccatggacacagtaaacagaacacaccaatactcagaaaagggaaagtggacgacaaacacagagactggtgcaccccagctcaacccgattgatggaccactacctgaggataacgaaccaagtgggtatgcacaaacagactgtgtcctagaggctatggctttccttgaagaatcccacccaggaatatttgagaattcatgccttgaaacaatggaagttgttcaacaaacaagagtagataaactgactcaaggtcgccagacttatgattggacattaaacagaaatcaaccggcagcaactgcattggccaacaccatagaagtctttagatcgaatggcctaacagctaatgagtcaggaaggctagtagatttcttaaaggatgtaatggaatcaatgaacaaagaggaaatagagataacaacccactttcaaagaaaaaggagagtaagagacaacatgaccaagaagatggtcacgcaaagaacaatagggaagaaaaaacagagactgaataagagaggctatctaataagagcactgacattaaatacgatgaccaaagatgcagagagaggcaagttaaaaagaagggctatcgcaacacctgggatgcagattagaggtttcgtatactttgttgaaattttagctaggagcatttgcgaaaagcttgaacagtctgggctcccggtagggggcaatgaaaagaaggccaaactggcaaatgttgtgagaaagatgatgactaattcacaagacacagagatttctttcacaatcactggggacaacactaagtggaatgaaaatcaaaatcctcgaatgttcctggcgatgattacatatatcaccaaaaatcaacccgagtggtttagaaacatcctgagcatggcacccataatgttctcaaacaaaatggcaagactagggaaagggtacatgttcgagagtaaaagaatgaagattcgaacacaaataccagcagaaatgctagcaagcattgacctgaagtacttcaatgaatcaacaaagaagaaaattgagaaaataaggcctcttttaatggatggcacagcatcactgagtcctgggatgatgatgggcatgttcaacatgctaagtacggtcttgggagtctcgatactgaatcttggacaaaagaaatacaccaagacaacatactggtgggatgggctccaatcatccgacgattttgctctcatagtgaatgcaccaaaccatgagggaatacaagcaggagtggacagattctacaggacctgcaagttagtgggtatcaacatgagcaaaaagaagtcctatataaataagacagggacatttgaattcacaagctttttttatcgctatggatttgtggctaattttagcatggagctacccagctttggagtgtctggagtaaatgaatcagctgacatgagtattggagtaacagtgataaagaacaacatgataaacaatgaccttggacctgcaacagcccagatggctcttcaattgttcatcaaagactacagatacacatataggtgccataggggagacacacaaattcagacgagaagatcatttgagttaaagaagctgtgggatcaaacccaatcaaaggtagggctattagtatcagatggaggaccaaacttatacaatatacggaatcttcacattcctgaagtctgcttaaaatgggagctaatggatgatgattatcggggaagactttgtaatcccctgaatccctttgtaagtcataaggagattgattctgtaaacaatgctgtggtaatgccagcccacggtccagccaaaagcatggaatatgatgccgttgcaactacacattcctggattcccaagaggaatcgttctattctcaacacaagccaaaggggaattcttgaggatgaacagatgtaccagaagtgctgcaatctattcgagaaatttttccctagcagttcgtataggagaccggttggaatttctagcatggtggaggccatggtgtctagggcccggattgatgccagggtcgactttgagtctggacggatcaagaaagaagagttctctgagatcatgaagatctgttccaccattgaagaactcagacggcaaaaataa

>H1N1_PB1_CY176440_A/Athens/INS3_698/2011

atggatgtcaatccgactctacttttcctaaaaattccagcgcaaaatgccataagcaccacattcccttatactggagatcctccatacagccatggaacaggaacaggatacaccatggacacagtaaacagaacacaccaatactcagaaaagggaaagtggacgacaaacacagagactggtgcaccccagctcaacccgattgatggaccactaccggaggataatgaaccaagtgggtatgcacaaacagactgtgttctagaggctatggctttccttgaagaatcccacccaggaatatttgagaattcatgccttgaaacaatggaagttgttcaacaaacaagggtagataaactgactcaaggtcgccagacttatgattggacattaaacagaaatcaaccggcagcaactgcattggccaacaccatagaagtctttagatcgaatgacctaacagctaatgagtcaggaaggctaatagatttcttaaaggatgtaatggaatcaatgaacaaagaggaaatagagataacaacccactttcaaagaaaaaggagagtaagagacaacatgaccaagaagatggtcacgcaaagaacaatagggaagaaaaaacagagactgaataagagaggctatctaataagagcactgacattaaatacgatgaccaaagatgcagagagaggcaagttaaaaagaagggctatcgcaacacctgggatgcagattagaggtttcgtatactttgttgaaactttagctaggagcatttgcgaaaagcttgaacagtctgggctcccagtagggggcaatgaaaagaaggccaaactggcaaatgttgtgagaaagatgatgactaattcacaagacacagagatttctttcacaatcactggggacaacactaagtggaatgaaaatcaaaatcctcgaatgttcctggcgatgattacatatatcaccagaaatcaacccgagtggtttagaaacatcctgagcatggcacccataatgttctcaaacaaaatggcaagactagggaaagggtacatgttcgagagtaaaagaatgaagattcgaacacaaataccagcagaaatgctagcaagcattgacctgaagtacttcaatgaatcaacaaagaagaaaattgagaaaataaggcctcttttaatggatggcacagcatcactgagtcctgggatgatgatgggcatgttcaacatgctaagtacggtcttgggagtctcgatactgaatcttggacaaaagaaatacaccaagacaacatactggtgggatgggctccaatcatccgacgattttgctctcatagtgaatgcaccaaatcatgagggaatacaagcaggagtggacagattctacaggacctgcaagttagtgggaatcaacatgagcaaaaagaagtcctatataaataagacagggacatttgaattcacaagctttttttatcgctatggatttgtggctaattttagcatggagctacccagctttggagtgtctggagtaaatgaatcagctgacatgagtattggagtaacagtgataaagaacaacatgataaacaatgaccttggacctgcaacggcccagatggctcttcaattgttcatcaaagactacagatacacatataggtgccataggggagacacacaaattcagacgagaagatcatttgagttaaagaagctgtgggatcaaacccaatcaaaggtagggctattagtatcagatggaggaccaaacttatacaatatacggaatcttcacattcctgaagtctgcttaaaatgggagctaatggatgatgattatcggggaagactttgtaatcccctgaatccctttgtaagtcataaggagattgattctgtaaacaatgctgtgataatgccagcccatggtccagccaaaagcatggaatatgatgccgttgcaactacacattcctggattcccaagaggaatcgttctattctcaacacaagccaaaggggaattcttgaggatgaacagatgtaccagaagtgctgcaatctattcgagaaatttttccctagcagttcatataggagaccggttggaatttctagcatggtggaggccatggtgtctagggcccggattgatgccagggtcgactttgagtctggacggatcaagaaagaagagttctctgagatcatgaagatctgttccaccattgaagaactcagacggcaaaaataa

>H1N1_PB1_CY176907_A/Netherlands/602-cHAP2/2009

atggatgtcaatccgactctacttttcctaaaaattccagcgcaaaatgccataagcaccacattcccttatactggagatcctccatacagccatggaacaggaacaggatacaccatggacacagtaaacagaacacaccaatactcagaaaagggaaagtggacgacaaacacagagactggtgcaccccagctcaacccgattgatggaccactacctgaggataatgaaccaagtgggtatgcacaaacagactgtgttctagaggctatggctttccttgaagaatcccacccaggaatatttgagaattcatgccttgaaacaatggaagttgttcaacaaacaagggtagataaactaactcaaggtcgccagacttatgattggacattaaacagaaatcaaccggcagcaactgcattggccaacaccatagaagtctttagatcgaatggcctaacagctaatgagtcaggaaggctaatagatttcttaaaggatgtaatggaatcaatgaacaaagaggaaatagagataacaacccactttcaaagaaaaaggagagtaagagacaacatgaccaagaagatggtcacgcaaagaacaatagggaagaaaaaacaaagactgaataagagaggctatctaataagagcactgacattaaatacgatgaccaaagatgcagagagaggcaagttaaaaagaagggctatcgcaacacctgggatgcagattagaggtttcgtatactttgttgaaactttagctaggagcatttgcgaaaagcttgaacagtctgggctcccagtagggggcaatgaaaagaaggccaaactggcaaatgttgtgagaaagatgatgactaattcacaagacacagagatttctttcacaatcactggggacaacactaagtggaatgaaaatcaaaatcctcgaatgttcctggcgatgattacatatatcaccagaaatcaacccgagtggttcagaaacatcctgagcatggcacccataatgttctcaaacaaaatggcaagactagggaaagggtacatgttcgagagtaaaagaatgaagattcgaacacaaataccagcagaaatgctagcaagcattgacctgaagtacttcaatgaatcaacaaagaagaaaattgagaaaataaggcctcttctaatagatggcacagcatcactgagtcctgggatgatgatgggcatgttcaacatgctaagtacggtcttgggagtctcgatactgaatcttggacaaaagaaatacaccaagacaatatactggtgggatgggctccaatcatccgacgattttgctctcatagtgaatgcaccaaaccatgagggaatacaagcaggagtggacagattctacaggacctgcaagttagtgggaatcaacatgagcaaaaagaagtcctatataaataagacagggacatttgaattcacaagctttttttatcgctatggatttgtggctaattttagcatggagctacccagctttggagtgtctggagtaaatgaatcagctgacatgagtattggagtaacagtgataaagaacaacatgataaacaatgaccttggacctgcaacggcccagatggctcttcaattgttcatcaaagactacagatacacatataggtgccataggggagacacacaaattcagacgagaagatcatttgagttaaagaagctgtgggatcaaacccaatcaaaggtagggctattagtatcagatggaggaccaaacttatacaatatacggaatcttcacattcctgaagtctgcttaaaatgggagctaatggatgatgattatcggggaagactttgtaatcccctgaatccctttgtcagtcataaagaaattgattctgtaaacaatgctgtggtaatgccagcccatggtccagccaaaagcatggaatatgatgccgttgcaactacacattcctggattcccaagaggaatcgttctattctcaacacaagccaaaggggaattcttgaggatgaacagatgtaccagaagtgctgcaatctattcgagaaatttttccctagcagttcatataggagaccggttggaatttctagcatggtggaggccatggtgtctagggcccggattgatgccagggtcgacttcgagtctggacggatcaagaaagaagagttctctgagatcatgaagatctgttccaccattgaagaactcagacggcaaaaataa

>H1N1_PB1_KJ690461_A/Uganda/MUWRP-069/2009

atggatgtcaatccgactctacttttcctaaaaattccagcgcaaaatgccataagcaccacattcccttatactggagatcctccatacagccatggaacaggaacaggatacaccatggacacagtaaacagaacacaccaatactcagaaaagggaaagtggacgacaaacacagagactggtgcaccccagctcaacccgattgatggaccactacctgaggataatgaaccaagtgggtatgcacaaacagactgtgttcaagaggctatggctttccttgaagaatcccacccgggaatatttgagaattcatgccttgaaacaatggaagttgttcaacaaacaagggaagataaactaactcaaggtcgccagacttatgattggacattaaacagaaatcaaccggcagcaactgcattggccaacaccatagaagtttttagatcgaatggcctaacagctaatgagtcaggaaggctaatagatttcttaaaggatgtaatggaatcaatgaacaaagaggaaatagagataacaacccactttcaaagaaaaaggagagtaagagacaacatgaccaagaagatggtcacgcaaagaacaatagggaagaaaaaacaaagactgaataagagaggctatctaataagagcactgacattaaatacgatgaccaaagatgcagagagaggcaagttaaaaagaagggctattgcaacacctgggatgcagattagaggtttcgtatactttgttgaaactttagctaggagcatttgcgaaaagcttgaacagtctgggctcccagtagggggcaatgaaaagaaggccaaactggcaaatgttgtgagaaagatgatgactaattcacaagacacagagatttctttcacaatcactggggacaacactaagtggaatgaaaatcaaaatcctcgaatgttcctagcgatgattacatatatcaccagaaatcaacccgagtggttcagaaacatcctgagcatggcacccataatgttctcaaacaaaatggcaagactagggaaagggtacatgttcgagagtaaaagaatgaagattcgaacacaaataccagcagaaatgctagcaagcattgacctgaagtacttcaatgaatcaacaaagaagaaaattgagaaaataaggcctcttctaatagatggcacagcatcactgagtcctgggatgatgatgggcatgttcaacatgctaagtacggtcttgggagtctcgatactgaatcttggacaaaagaaatacaccaagacaatatactggtgggatgggctccaatcatccgacgattttgctctcatagtgaatgcaccaaaccatgagggaatacaagcaggagtggacagattctacaggacctgcaagttagtgggaatcaacatgagcaaaaagaagtcctatataaataagacagggacatttgaattcacaagctttttttatcgctatggatttgtggctaattttagcatggagctacccagctttggagtgtctggagtaaatgaatcagctgacatgagtattggagtaacagtgataaagaacaacatgataaacaatgaccttggacctgcaacggcccagatggctcttcaattgttcatcaaagactacagatacacatataggtgccataggggagacacacaaattcagacgagaagatcatttgagttaaagaagctgtgggatcaaacccaatcaaaggtagggctattagtatcagatggaggaccaaacttatacaatatacggaatcttcacattcctgaagtctgcttaaaatgggagctaatggatgatgattatcggggaagactttgtaatcccctgaatccctttgtcagtcataaagagattgattctgtaaacaatgctgtggtaatgccagcccatggtccagccaaaagcatggaatatgatgccgttgcaactacacattcctggattcccaagaggaatcgttctattctcaacacaagccaaaggggaattcttgaggatgaacagatgtaccagaagtgctgcaatctattcgagaaatttttccctagcagttcatataggagaccggttggaatttctagcatggtggaggccatggtgtctagggcccggattgatgccaggatcgacttcgagtctggacggatcaagaaagaagagttctctgagatcatgaagatctgttccaccattgaagaactcagacggcaaaaataa

>H1N1_PB1_KJ690398_A/Uganda/MUWRP-093/2009

atggatgtcaatccgactctacttttcctaaaaattccagcgcaaaatgccataagcaccacattcccttatactggagatcctccatacagccatggaacaggaacaggatacaccatggacacagtaaacagaacacaccaatactcagaaaagggaaagtggacgacaaacacagagactggtgcaccccagctcaacccgattgatggaccactacctgaggataatgaaccaagtgggtatgcacaaacagactgtgttctagaggctatggctttccttgaagaatcccacccaggaatatttgagaattcatgccttgaaacaatggaagttgttcaacaaacaagggtagataaactaactcaaggtcgccagacttatgattggacattaaacagaaatcaaccggcagcaactgcattggccaacaccatagaagtctttagatcgaatggcctaacagctaatgagtcaggaaggctaatagatttcttaaaggatgtaatggaatcaatgaacaaagaggaaatagagataacaacccactttcaaagaaaaaggagagtaagagacaacatgaccaagaagatggtcacgcaaagaacaatagggaagaaaaaacaaagactgaataagagaggctatctaataagagcactgacattaaatacgatgaccaaagatgcagagagaggcaagttaaaaagaagggctatcgcaacacctgggatgcagattagaggtttcgtatactttgttgaaactttagctaggagcatttgcgaaaagcttgaacagtctgggctcccagtagggggcaatgaaaagaaggccaaactggcaaatgttgtgagaaagatgatgactaattcacaagacacagagatttctttcacaatcactggggacaacactaagtggaatgaaaatcaaaatcctcgaatgttcctggcgatgattacatatatcaccagaaatcaacccgagtggttcagaaacatcctgagcatggcacccataatgttctcaaacaaaatggcaagactagggaaagggtacatgttcgagagtaaaagaatgaagattcgaacacaaataccagcagaaatgctagcaagcattgacctgaagtacttcaatgaatcaacaaagaagaaaattgagaaaataaggcctcttctaatagatggcacagcatcactgagtcctgggatgatgatgggcatgttcaacatgctaagtacggtcttgggagtctcgatactgaatcttggacaaaagaaatacaccaagacaatatactggtgggatgggctccaatcatccgacgattttgctctcatagtgaatgcaccaaaccatgagggaatacaagcaggagtggacagattctacaggacctgcaagttagtgggaatcaacatgagcaaaaagaagtcctatataaataagacagggacatttgaattcacaagctttttttatcgctatgggtttgtggctaattttagcatggagctacccagctttggagtgtctggagtaaatgaatcagctgacatgagtattggagtaacagtgataaagaacaacatgataaacaatgaccttggacctgcaacggcccagatggctcttcaattgttcatcaaagactacagatacacatataggtgccataggggagacacacaaattcagacgagaagatcatttgagttaaagaagctgtgggatcaaacccaatcaaaggtagggctattagtatcagatggaggaccaaacttatacaatatacggaatcttcacattcctgaagtctgcttaaaatgggagctaatggatgatgattatcggggaagactttgtaatcccctgaatccctttgtcagtcataaagagattgattctgtaaacaatgctgtggtaatgccagcccatggtccagccaaaagcatggaatatgatgccgttgcaactacacattcctggattcccaagaggaatcgttctattctcaacacaagccaaaggggaattcttgaggatgaacagatgtaccagaaatgctgcaatctattcgagaaatttttccctagcagttcatataggagaccggttggaatttctagcatggtggaggccatggtgtctagggcccggattgatgccagggtcgacttcgagtctggacggatcaagaaagaagagttctctgagatcatgaagatctgttccaccattgaagaactcagacggcaaaaataa

>H1N1_PB1_CY181775_A/Nicaragua/AGA2-28/2011

atggatgtcaatccgactctacttttcctaaaaattccagcgcaaaatgccataagcaccacattcccttatactggagatcctccatacagccatggaacaggaacaggatacaccatggacacagtaaacagaacacaccaatactcagaaaagggaaagtggacgacaaacacagagactggtgcaccccagctcaacccgattgatggaccattacctgaggataatgaaccaagtgggtatgcacaaacagactgtgttctagaggctatggctttccttgaagaatcccacccaggaatatttgagaattcatgccttgaaacaatggaagttgttcaacaaacaagggtagataaactaactcaaggtcgccagacttatgattggacattaaacagaaatcaaccggcagcaactgcattggccaacaccatagaagtctttagatcgaatggcctaacagctaatgagtcaggaaggctaatagatttcttaaaggatgtaatggaatcaatgaacaaagaggaaatagagataacaacccactttcaaagaaaaaggagagtgagagacaacatgaccaagaagatggtcacgcaaagaacaatagggaagaaaaaacaaagactgaataagagaggctatctaataagagcactgacattaaatacgatgaccaaagatgcagagagaggcaagttaaaaagaagggctatcgcaacacctgggatgcagattagaggtttcgtatactttgttgaaactttagctaggagcatttgcgaaaagcttgaacagtctgggctcccagtagggggcaatgaaaaaaaggccaaactggcaaatgttgtgagaaagatgatgactaattcacaagacacagagatttctttcacaatcactggggacaacactaagtggaatgaaaaccaaaatcctcgaatgttcctggcgatgattacatatatcaccagaaatcaacccgagtggttcagaaacatcctgagcatggcacccataatgttctcaaacaaaatggcaagactagggaaagggtacatgttcgagagtaaaagaatgaagattcgaacgcaaataccagcagaaatgctagcaagcattgacctgaagtacttcaatgagtcaacaaagaagaaaattgagaaaataaggcctcttttaatagatggcacagcatcactgagtcctgggatgatgatgggcatgttcaacatgctaagtacggtcttgggagtctcgatactgaatcttggacaaaagaaatacaccaagacaatatactggtgggatgggctccaatcatccgacgattttgctctcatagtgaatgcaccaaaccatgagggaatacaagcaggagtggacagattttacaggacctgcaagttagtgggaatcaacatgagcaaaaagaagtcctatatcaataagacagggacatttgaattcacaagcttcttttatcgctatggatttgtggctaattttagcatggagctacccagctttggagtggctggagtaaatgaatcagctgacatgagtattggagtaacagtgataaagaacaacatgataaacaatgaccttggacctgcaacggcccagatggctcttcaattgttcatcaaagactacagatacacatataggtgccataggggagacacacaaattcagacgagaagatcatttgagttaaagaagctgtgggatcaaacccaatcaaaggtagggctattagtatcagatggaggaccaaacttatacaatatacggaatcttcacattcctgaagtctgcttaaaatgggagctaatggatgatgattatcggggaagactttgtaatcccctgaatccctttgtaagtcataaagagattgattctgtaaacaatgctgtggtaatgccagcccatggtccagccaaaagcatggaatatgatgccgttgcaactacacattcctggattcccaagaggaatcgttctattctcaacacaagccaaaggggaattcttgaggatgaacagatgtaccagaagtgctgcaatctattcgagagatttttccctagcagttcatataggagaccggttggaatttctagcatggtggaggccatggtgtctagggcccggattgatgccagggtcgactttgagtctggacggatcaagaaagaagagttctctgagatcatgaagatctgttccaccattgaagaactcagacggcaaaaataa

>H1N1_PB1_CY183151_A/Houston/JMM_122/2013

atggatgtcaatccgactctacttttcctaaaaattccagcacaaaatgccataagcaccacattcccttatactggagatcctccatacagccatggaacaggaacaggatacaccatggacacagtaaacagaacacatcaatactcagaaaagggaaagtggacaacaaacacagagactggtgcaccccagctcaacccgattgatggaccactacctgaggataatgaaccaagtgggtatgcacaaacagactgtgttctagaggctatggctttccttgaagaatcccacccaggaatatttgagaattcatgtcttgaaacaatggaagttgttcaacaaacaagggtagacaaactgactcaaggtcgccagacttatgattggacattaaacagaaatcaaccggcagcaactgcattggccaacaccatagaagtatttagatcgaatgacctaacagctaatgagtcaggaaggctaatagatttcttaaaggatgtaatggaatcaatgaacaaagaggaaatagagataacaacccactttcaaagaaaaaggagagtaagagacaacatgaccaagaagatggtcacgcaacgaacaatagggaagaaaaaacagagactgaataagagaggctatctaataagagcactgacattaaatacgatgaccaaagatgcagagagaggcaagttaaaaagaagggctatcgcaacacctgggatgcagattagaggtttcgtatactttgttgaaactttagctaggagcatttgcgaaaagcttgaacagtctgggctcccagtagggggcaatgaaaagaaggccaaactggcaaatgttgtgagaaagatgatgactaattcacaagacacagagatttctttcacaatcactggggacaacactaagtggaatgaaaatcaaaatcctcgaatgttcctggcgatgattacatatatcaccagaaatcaacccgagtggtttagaaacatcctgagcatggcacccataatgttctcaaacaaaatggcaagactagggaaagggtacatgttcgagagtaaaagaatgaagattcgaacacaaataccagcagaaatgctagcaagcattgacctgaagtacttcaatgaatcaacaaagaagaaaattgagaaaataaggcctcttttaatggatggcacagcatcactgagtcctgggatgatgatgggcatgttcaacatgctaagtacggtcttgggagtctcgatactgaatcttggacaaaagaaatacaccaagacaacatactggtgggatgggctccaatcatccgacgattttgctctcatagtgaatgcaccaaactatgagggaatacaagcaggagtggacagattctacaggacctgcaagttagtgggaatcaacatgagcaaaaagaagtcctatataaataagacagggacatttgaattcacaagctttttttatcgctatggatttgtggctaattttagcatggagctacccagctttggagtgtctggggtaaatgaatcagctgacatgagtattggagtaacagtgataaagaacaacatgataaacaatgaccttggacctgcaacggcccagatggctcttcaattgttcatcaaagactacagatacacatataggtgccataggggagacacacaaattcagacgagaagatcatttgagttaaagaagctgtgggatcaaacccaatcaaaggtagggctattagtatcagatggaggaccaaacttatacaatatacggaatcttcacattcctgaagtctgcttaaaatgggagctaatggatgatgattatcggggaagactttgtaatcccctgaatccctttgtaagtcataaggagattgattctgtaaacaatgctgtggtaatgccagcccatggtccagccaaaagcatggaatatgatgccgttgcaactacacattcctggattcccaagaggaatcgttctattctcaacacaagccaaaggggaattcttgaggatgaacagatgtaccagaagtgctgcaatctattcgagaaatttttccctagcagttcatataggagaccggttggaatttctagcatggtggaggccatggtgtctagggcccggattgatgccagggtcgactttgagtctggacggatcaagaaagaagagttctctgagatcatgaagatctgttccaccattgaagaactcagacggcaaaaataa

>H1N1_PB1_CY183335_A/Houston/JMM_148/2013

atggatgtcaatccgactctacttttcctaaaaattccagcacaaaatgccataagcaccacattcccttatactggagatcctccatacagccatggaacaggaacaggatacaccatggacacagtaaacagaacacaccaatactcagaaaagggaaagtggacaacaaacacagagactggtgcaccccagctcaacccgattgatggaccactacctgaggataatgaaccaagtgggtatgcacaaacagactgtgttctagaggctatggctttccttgaagaatcccacccaggaatatttgagaattcatgccttgaaacaatggaagttgttcaacaaacaagggtagataaactgactcaaggtcgccagacttatgattggacattaaacagaaatcaaccggcagcaactgcattggccaacaccatagaagtctttagatcgaatgacctaacagctaacgagtcaggaaggctaatagatttcttaaaggatgtaatggaatcaatgaacaaagaggaaatagagataacaacccactttcaaagaaaaaggagagtaagagacaacatgaccaagaagatggtcacgcaaagaacaatagggaagaaaaaacagagactgaataagagaggctatctaataagagcattgacattaaatacaatgaccaaagatgcagagagaggcaagttaaaaagaagggctatcgcaacacctgggatgcagattagaggtttcgtatactttgttgaaactttagctaggagcatttgcgaaaagcttgaacagtctggactcccagtaggaggcaatgaaaagaaggccaaactggcaaatgttgtgagaaagatgatgactaattcacaagatacagagatttctttcacaatcactggggacaacactaagtggaatgaaaatcaaaatcctcgaatgttcctggcgatgattacatatatcaccagaaatcaacccgagtggtttagaaacatcctgagcatggcacccataatgttctcaaataaaatggcaagactagggaaagggtacatgttcgagagtaaaagaatgaagattcgaacacaaataccagcagaaatgctagcaagcattgatctgaagtacttcaatgaatcaacaaagaagaaaattgagaaaataaggcctcttttaatggatggcacagcatcactgagtcctgggatgatgatgggcatgttcaacatgctaagtacggtcttgggagtctcgatactgaatcttggacaaaagaaatacaccaagacaacatactggtgggatgggctccaatcatccgacgattttgctctcatagtgaatgcaccaaaccatgagggaatacaagcaggagtggacagattctacaggacctgcaagttagtgggaatcaacatgagcaaaaagaagtcctatataaataagacagggacatttgaattcacaagctttttttatcgctatggatttgtggctaattttagcatggagctacccagctttggagtgtctggagtaaatgaatcagctgacatgagtattggagtaacagtgataaagaacaacatgataaacaatgaccttggacctgcaacggcccagatggctcttcaattgttcatcaaagactacagatacacatataggtgccataggggagacacacaaattcagacgagaagatcatttgagttaaagaagctgtgggatcaaacccaatcaaaggtagggctattagtatcagatggaggaccaaacttatacaatatacggaatcttcacattcctgaagtctgcttaaaatgggagctaatggatgatgattatcggggaagactttgtaatcccctgaatccctttgtaagtcataaggagattgattctgtaaacaatgctgtggtaatgccagcccatggtccagccaaaagcatggaatatgatgccgttgcaactacacattcctggattcccaagaggaatcgttctattctcaacacaagccaaaggggaattcttgaggatgaacagatgtaccagaagtgttgcaatctattcgagaaatttttccctagcagttcatataggagaccggttggaatttctagcatggtggaggccatggtatctagggcccggattgatgccagggtcgactttgagtctggacggatcaagaaagaagagttctctgagatcatgaagatctgttccaccattgaagaactcagacggcaaaaataa

>H1N1_PB1_CY186073_A/Houston/JMM_155/2013

atggatgtcaatccgactctacttttcctaaaaattccagcacaaaatgccataagcaccacattcccttatactggagatcctccatacagccatggaacaggaacaggatacaccatggacacagtaaacagaacacatcaatactcagaaaagggaaagtggacaacaaacacagagactggtgcaccccagctcaacccgattgatggaccactacctgaggataatgaaccaagtgggtatgcacaaacagactgtgttctagaggctatggctttccttgaagaatcccacccaggaatatttgagaattcatgccttgaaacaatggaagttgttcaacaaacaagggtagacaaactgactcaaggtcgccagacttacgattggacattaaacagaaatcaaccggcagcaactgcattggccaacaccatagaagtatttagatcgaatgacctaacagctaatgagtcaggaaggctaatagatttcttaaaagatgtaatggaatcaatgaacaaagaggaaatagagataacaacccactttcaaagaaaaaggagagtaagagacaacatgaccaagaagatggtcacgcaaagaacaatagggaagaaaaaacagagactgaataagagaggctatctaataagagcactgacattaaatacgatgaccaaagatgcagagagaggcaagttaaaaagaagggctatcgcaacacctgggatgcagattagaggtttcgtatactttgttgaaactttagctaggagcatttgcgaaaagcttgaacagtctgggctcccagtagggggcaatgaaaagaaggccaaactggcaaatgttgtgagaaagatgatgactaattcacaagacacagagatttctttcacaatcactggggacaacactaagtggaatgaaaatcaaaatcctcgaatgttcctggcgatgattacatatatcaccagaaatcaacccgagtggtttagaaacatcctgagcatggcacccataatgttctcaaacaaaatggcaagactagggaaagggtacatgttcgagagtaaaagaatgaagattcgaacacaaataccagcagaaatgctagcaagcattgacctgaagtacttcaatgaatcaacaaagaagaaaattgagaaaataaggcctcttttaatggatggcacagcatcactgagtcctgggatgatgatgggcatgttcaacatgctaagtacggtcttgggagtctcgatactgaatcttggacaaaagaaatacaccaagacaacatactggtgggatgggctccaatcatccgacgattttgctctcatagtgaatgcaccaaactatgagggaatacaagcaggagtggacagattctacaggacctgcaagttagtgggaatcaacatgagcaaaaagaagtcctatataaataagacagggacatttgaattcacaagctttttttatcgctatggatttgtggctaattttagcatggagctacccagctttggagtgtctggggtaaatgaatcagctgacatgagtattggagtaacagtgataaagaacaacatgataaacaatgaccttggacctgcaacggcccagatggctcttcaattgttcatcaaagactatagatacacatataggtgccataggggagacacacaaattcagacgagaagatcatttgagttaaagaagctgtgggatcaaacccaatcaaaggtagggctattagtatcagatggaggaccaaacttatacaatatacggaatcttcacattcctgaagtctgcttaaaatgggagctaatggatggtgattatcggggaagactttgtaatcccctgaatccctttgtaagtcataaggagattgattctgtaaacaatgctgtggtaatgccagcccatggtccagccaaaagcatggaatatgatgccgttgcaactacacattcctggattcccaagaggaatcgttctattctcaacacaagccaaaggggaattcttgaggatgaacagatgtaccagaagtgctgcaatctattcgagaaatttttccctagcagttcatataggagaccggttggaatttctagcatggtggaggccatggtgtctagggcccggattgatgccagggtcgattttgagtctggacggatcaagaaagaagagttctctgagatcatgaagatctgttccaccattgaagaactcagacggcaaaaataa

>H1N1_PB1_KJ856259_A/Sri Lanka/505/2011

atggatgtcaatccgactctacttttcctaaaaattccagcgcaaaatgccataagcaccacattcccttatactggagatcctccatacagccatggaacaggaacaggatacactatggacacagtaaacagaacacaccaatactcagaaaagggaaggtggacgacaaacacagagactggtgcaccccagctcaacccgattgatggaccactacctgaggataatgaaccaagtgggtatgcacaaacagactgtgttctagaggctatggctttccttgaagaatcccacccaggaatatttgagaattcatgccttgaaacaatggaagctgttcaacaaacaagggtagataaactaactcaaggtcgccaaacttatgattggacattgaacagaaatcaaccggcagcaactgcattggccaacaccatagaagtctttagatcgaatggcctaacagctaatgagtcaggaaggctaatagatttcttaaaggatgtaatggaatcaatgaacaaagaggaaatagagataacaacccactttcaaagaaaaaggatagttagagacaacatgaccaagaagatggtcacgcaaagaacaatagggaagaaaaaacaaagactgaataagagaggctatctaataagagcactgacattaaatacgatgaccaaagatgcagagagaggcaagttaaaaagaagggctatcgcaacacctgggatgcagattagaggtttcgtatactttgttgaaactttagctaggagcatttgcgaaaagcttgaacagtctgggctcccagtagggggcaatgaaaagaaggccaaactggcaaatgttgtgagaaagatgatgactaattcacaagacacagagatttctttcacaatcactggggacaacactaagtggaatgaaaatcaaaatcctcgaatgttcctggcgatgattacatatatcaccagaaatcaacccgagtggttcagaaacatccttagcatggcacccataatgttctcaaacaaaatggcaagactagggaaagggtacatgttcgagagtaaaagaatgaagattcgaacacaaattccagcagaaatgctagcaagcattgacctgaagtacttcaatgaatcaacaaagaagaaaattagaaaaataaggcctcttctaatagatggcacagcatcactgagtcctgggatgatgatgggcatgttcaacatgctaagtacggtcttgggagtctcgatactgaatcttggacaaaagaaatacaccaagacaacatactggtgggatgggctccaatcatccgacgattttgctctcatagtgaatgcaccaaaccatgagggaatacaagcaggagtggacagattctacaggacctgcaagttagtgggaatcaacatgagcaaaaagaagtcatatataaataaaacagggacatttgaattcacaagctttttttatcgctatggatttgtggctaattttagcatggagctacccagctttggagtgtctggagtaaatgaatcagctgacatgagtattggagtaacagtgataaagaacaacatgataaacaatgaccttggacctgcaacggcccagatggctcttcaattgttcatcaaagactacagatacacatataggtgccataggggagacacacaaattcagacgagaagatcatttgagttaaaaaagctgtgggatcaaacccaatcaaaggtagggctattagtatcagatggaggaccaaacttatacaatatacggaatcttcacattcctgaagtctgcttaaaatgggagctaatggatgatgattatcggggaagactttgtaatcccctgaatccctttgtcagtcataaagagattgattctgtaaacaatgctgtggtaatgccagcccatggtccagccaaaagcatggaatatgatgccgttgcaactacacattcctggattcccaagaggaatcgttctattctcaacacaagccaaaggggaattcttgaggatgaacagatgtaccagaagtgctgcaatctattcgagaaatttttccctagcagttcatataggagaccggttggaatttctagcatggtggaggccatggtgtctagggcccggattgatgccagggtcgaattcgagtctggacggatcaagaaagaagagttctctgagatcatgaagatctgttccaccattgaagaactcagacggcaaaaataa

>H1N1_PB1_KJ856206_A/Sri Lanka/23127/2010

atggatgtcaatccgactctacttttcctaaaaattccagcgcaaaatgccataagcaccacattcccttatactggagatcctccatacagccatggaacaggaacaggatacaccatggacacagtaaacagaacacaccaatactcagaaaagggaaagtggacgacaaacacagagactggtgcaccccagctcaacccgattgatggaccactacctgaggataatgaaccaagtgggtatgcacaaacagactgtgttctagaggctatggctttccttgaagaatcccacccaggaatatttgagaattcatgccttgaaacaatggaggctgttcaacaaacaagggtagataaactaactcaaggtcgccagacttatgattggacattaaacagaaatcaaccggcagcaactgcattggccaacaccatagaagtctttagatcgaatggcctaacagctaatgagtcaggaaggctaatagatttcttaaaggatgtaatggaatcaatgaacaaagaggaaatagagataacaacccactttcaaagaaaaaggagagtaagagacaacatgaccaagaagatggtcacgcaaagaacaatagggaagaaaaaacaaagactgaataagagaggctatctaataagagcactgacattaaatacgatgaccaaagatgcagagagaggcaagttaaaaagaagggctatcgcaacacctgggatgcagattagaggtttcgtatactttgttgaaactttagctaggagcatttgcgaaaagcttgaacagtctgggctcccagtagggggcaatgaaaagaaggccaaactggcaaatgttgtgagaaagatgatgactaattcacaagacacagagatttctttcacaatcactggggacaacactaagtggaatgaaaatcaaaatcctcgaatgttcctggcgatgattacatatatcaccagaaatcaacccgagtggttcagaaacatccttagcatggcacccataatgttctcaaacaaaatggcaagactagggaaagggtacatgttcgagagtaaaagaatgaagattcgaacacaaattccagcagaaatgctagcaagcattgacctgaagtacttcaatgaatcaacaaagaagaaaattgagaaaataaggcctcttctaatagatggcacagcatcactgagtcctgggatgatgatgggcatgttcaacatgctaagtacggtcttgggagtctcgatactgaatcttggacaaaagaaatacaccaagacaacatactggtgggatgggctccaatcatccgacgattttgctctcatagtgaatgcaccaaaccatgagggaatacaagcaggagtggacagattctacaggacctgcaagttagtgggaatcaacatgagcaaaaagaagtcatatataaataaaacagggacatttgaattcacaagctttttttatcgctatggatttgtggctaattttagcatggagctacccagctttggagtgtctggagtaaatgaatcagctgacatgagtattggagtaacagtgataaagaacaacatgataaacaatgaccttggacctgcaacggcccagatggctcttcaattgttcatcaaagactacagatacacatataggtgccataggggagacacacaaattcagacgagaagatcatttgaggtaaaaaagctgtgggatcaaacccaatcaaaggtagggctattagtatcagatggaggaccaaacttatacaatatacggaatcttcacattcctgaagtctgcttaaaatgggagctaatggatgatgattatcggggaagactttgtaatcccctgaatccctttgtcagtcataaagagattgattctgtaaacaatgctgtggtaatgccagcccatggtccagccaaaagcatggaatatgatgccgttgcaactacacattcctggattcccaagaggaatcgttctattctcaacacaagccaaaggggaattcttgaggatgaacagatgtaccagaagtgctgcaatctattcgagaaatttttccctagtagttcatataggagaccggttggaatttctagcatggtggaggccatggtgtctagggcccggattgatgccagggtcgacttcgagtctggacggatcaagaaagaagagttctctgagatcatgaagatctgttccaccattgaagaactcagacggcaaaaataa

>H1N1_PB1_KJ856120_A/Sri Lanka/20894/2010

atggatgtcaatccgactctacttttcctaaaaattccagcgcaaaatgccataagcaccacattcccttatactggagatcctccatacagccatggaacaggaacaggatacaccatggacacagtaaacagaacacaccaatactcagaaaagggaaagtgggcgacaaacacagagactggtgcaccccagctcaacccgattgatggaccactacctgaggataatgaaccaagtgggtatgcacaaacagactgtgttctagaggctatggctttccttgaagaatcccacccaggaatatttgagaattcatgccttgaaacaatggaagctgttcaacaaacaagggtagataaactaactcaaggtcgccagacttatgattggacattaaacagaaatcaaccggcagcaactgcattggccaacaccatagaagtctttagatcgaatggcctaacagctaatgagtcaggaaggctaatagatttcttaaaggatgtaatggaatcaatgaacaaagaggaaatagagataacaacccactttcaaagaaaaaggagagtaagagacaacatgaccaagaagatggtcacgcaaagaacaatagggaagaaaaaacaaagactgaataagagaggctatctaataagagcactgacattaaatacgatgaccaaagatgcagagagaggcaagttaaaaagaagggctatcgcaacacctgggatgcagattagaggtttcgtatactttgttgaaactttagctaggagcatttgcgaaaagctcgaacagtctgggctcccagtagggggcaatgaaaagaaggccaaactggcaaatgttgtgagaaagatgatgactaattcacaagacacagagatttctttcacaatcactggggacaacactaagtggaatgaaaatcaaaatcctcgaatgttcctggcgatgattacatatatcaccagaaatcaacccgagtggttcagaaacatccttagcatggcacccataatgttctcaaacaaaatggcaagactagggaaagggtacatgttcgagagtaaaagaatgaagattcgaacacaaattccagcagaaatgctagcaagcattgacctgaagtacttcaatgaatcaacaaagaagaaaattgagaaaataaggcctcttctaatagatggcacagcatcactgagtcctgggatgatgatgggcatgttcaacatgctaagtacggtcttgggagtctcgatactgaatcttggacaaaagaaatacaccaagacaacatactggtgggatgggctccaatcatccgacgattttgctctcatagtgaatgcaccaaaccatgggggaatacaagcaggagtggacagattctacaggacctgcaagttagtgggaatcaacatgagcaaaaagaagtcatatataaataaaacagggacatttgaattcacaagctttttttatcgctatggatttgtggctaattttagcatggagctacccagctttggagtgtctggagtaaatgaatcagctgacatgagtattggagtaacagtgataaagaacaacatgataaacaatgaccttggacctgcaacggcccagatggctcttcaattgttcatcaaagactacagatacacatataggtgccataggggagacacacaaattcagacgagaagatcatttgagttaaaaaagctgtgggatcaaacccaatcaaaggtagggctattagtatcagatggaggaccaaacttatacaatatacggaatcttcacattcctgaagtctgcttaaaatgggagctaatggatgatgattatcggggaagactttgtaatcccctgaatccctttgtcagtcataaagagattgattctgtaaacaatgctgtggtaatgccagcccatggtccagccaaaagcatggaatatgatgccgttgcaactacacattcctggattcccaagaggaatcgttctattctcaacacaagccaaaggggaattcttgaggatgaacagatgtaccagaagtgctgcaatctattcgagaaatttttccctagcagttcttataggagaccggttggaatttctagcatggtggaggccatggtgtctagggcccggattgatgccagggtcgacttcgagtctggacggatcaagaaagaagagttctctgagatcatgaagatctgttccaccattgaagaactcagacggcaaaaataa

>H1N1_PB1_KJ856104_A/Sri Lanka/20708/2010

atggatgtcaatccgactctacttttcctaaaaattccagcgcaaaatgccataagcaccacattcccttatactggagatcctccatacagccatggaacaggaacaggatacaccatggacacagtaaacagaacacaccaatactcagaaaagggaaagtgggcgacaaacacagagactggtgcaccccagctcaacccgattgatggaccactacctgaggataatgaaccaagtgggtatgcacaaacagactgtgttctagaggctatggctttccttgaagaatcccacccaggaatatttgagaattcatgccttgaaacaatggaagctgttcaacaaacaagggtagataaactaactcaaggtcgccagacttatgattggacattaaacagaaatcaaccggcagcaactgcattggccaacaccatagaagtctttagatcgaatggcctaacagctaatgagtcaggaaggctaatagatttcttaaaggatgtaatggaatcaatgaacaaagaggaaatagagataacaacccactttcaaagaaaaaggagagtaagagacaacatgaccaagaagatggtcacgcaaagaacaatagggaagaaaaaacaaagactgaataagagaggctatctaataagagcactgacattaaatacgatgaccaaagatgcagagagaggcaagttaaaaagaagggctatcgcaacacctgggatgcagattagaggtttcgtatactttgttgaaactttagctaggagcatttgcgaaaagcttgaacagtctgggctcccagtagggggcaatgaaaagaaggccaaactggcaaatgttgtgagaaagatgatgactaattcacaagacacagagatttctttcacaatcactggggacaacactaagtggaatgaaaatcaaaatcctcgaatgttcctggcgatgattacatatatcaccagaaatcaacccgagtggttcagaaacatccttagcatggcacccataatgttctcaaacaaaatggcaagactagggaaagggtacatgttcgagagtaaaagaatgaagattcgaacacaaattccagcagaaatgctagcaagcattgacctgaagtacttcaatgaatcaacaaagaagaaaattgagaaaataaggcctcttctaatagatggcacagcatcactgagtcctgggatgatgatgggcatgttcaacatgctaagtacggtcttgggagtctcgatactgaatcttggacaaaagaaatacaccaagacaacatactggtgggatgggctccaatcatccgacgattttgctctcatagtgaatgcaccaaaccatgagggaatacaagcaggagtggacagattctacaggacctgcaagttagtgggaatcaacatgagcaaaaagaagtcatatataaataaaacagggacatttaaattcacaagctttttttatcgctatggatttgtggctaattttagcatggagctacccagctttggagtgtctggagtaaatgaatcagctgacatgagtattggagtaacagtgataaagaacaacatgataaacaatgaccttggacctgcaacggcccagatggctcttcaattgttcatcaaagactacagatacacatataggtgccataggggagacacacaaattcagacgagaagatcatttgagttaaaaaagctgtgggatcaaacccaatcaaaggtagggctattagtatcagatggaggaccaaacttatacaatatacggaatcttcacattcctgaagtctgcttaaaatgggagctaatggatgatgattatcggggaagactttgtaatcccctgaatccctttgtcagtcataaagagattgattctgtaaacaatgctgtggtaatgccagcccatggtccagccaaaagcatggaatatgatgccgttgcaactacacattcctggattcccaagaggaatcgttctattctcaacacaagccaaaggggaattcttgaggatgaacagatgtaccagaagtgctgcaatctattcgagaaatttttccctagcagttcatataggagaccggttggaatttctagcatggtggaggccatggtgtctagggcccggattgatgccagggtcgacttcgagtctggacggatcaagaaagaagagttctctgagatcatgaagatctgttccaccattgaagaactcagacggcaaaaataa

>H1N1_PB1_KJ856035_A/Sri Lanka/17773/2010

atggatgtcaatccgactctacttttcctaaaaattccagcgcaaaatgccataagcaccacattcccttatactggagatcctccatacagccatggaacaggaacaggatacaccatggacacagtaaacagaacacaccaatactcagaaaagggaaagtggacgacaaacacagagactggtgcaccccagctcaacccgattgatggaccactacctgaggataatgaaccaagtgggtatgcacaaacagactgtgttctagaggctatggctttccttgaagaatcccacccaggaatatttgagaattcatgccttgaaacaatggaagctgttcaacaaacaagggtagataaactaactcaaggtcgccagacttatgattggacattaaacagaaatcaaccggcagcaactgcattggccaacaccatagaagtctttagatcgaatggcctaacagctaatgagtcaggaaggctaatagatttcttaaaggatgtaatggaatcaatgaacaaagaggaaatagagataacaacccactttcaaagaaaaaggagagtaagagacaacatgaccaagaagatggtcacgcaaagaacaatagggaagaaaaaacaaagactgaataagagaggctatctaataagagcactgacattaaatacgatgaccaaagatgcagagagaggcaagttaaaaagaagggctatcgcaacacctgggatgcagattagaggtttcgtatactttgttgaaactttagctaggagcatttgcgaaaagcttgaacagtctgggctcccagtagggggcaatgaaaagaaggccaaactggcaaatgttgtgagaaagatgatgactaattcacaagacacagagatttctttcacaatcactggggacaacactaagtggaatgaaaatcaaaatcctcgaatgttcctggcgatgattacatatatcaccagaaatcaacccgagtggttcagaaacatccttagcatggcacccataatgttctcaaacaaaatggcaagactagggaaagggtacatgttcgagagtaaaagaatgaagattcgaacacaaattccagcagaaatgctagcaagcattgacctgaagtacttcaatgaatcaacaaagaagaaaattgagaaaataaggcctcttctaatagatggcacagcatcactgagtcctgggatgatgatgggcatgttcaacatgctaagtacggtcttgggagtctcgatactgaatcttggacaaaagaaatacaccaagacaacatactggtgggatgggctccaatcatccgacgattttgctctcatagtgaatgcaccaaaccatgagggaatacaagcaggagtggacagattctacaggacatgcaagttagtgggaatcaacatgagcaaaaagaagtcatatataaataaaacagggacatttgaattcacaagctttttttatcgatatggatttgtggctaattttagcatggagctacccagctttggagtgtctggagtaaatgaatcagatgacatgagtattggagtaacagtgataaagaacaacatgataaacaatgaccttggacctgcaacggcccagatggctcttcaattgttcatcaaagactacagatacacatataggtgccataggggagacacacaaattcagacgagaagatcatttgagttaaaaaagctgtgggatcaaacccaatcaaaggtagggctattagtatcagatggaggaccaaacttatacaatatacggaatcttcacattcctgaagtctgcataaaatgggagctaatggatgatgattatcggggaagactttgtaatcccctgaatccctttgtcagtcataaagagattgattctgtaaacaatgctgtggtaatgccagcccatggtccagccaaaagcatggaatatgatgccgttgcaactacacattcctggattcccaagaggaatcgttctattctcaacacaagccaaaggggaattcttgaggatgaacagatgtaccagaagtgctgcaatctattcgagaaatttttccctagcagttcatataggagaccggttggaatttctagcatggtggaggccatggtgtctagggcccggattgatgccagggtcgacttcgagtctggacggatcaagaaagaagagttctctgagatcatgaagatctgttccaccattgaagaactcagacggcaaaaataa

>H1N1_PB1_KJ880884_A/Czech Republic/3/2014

atggatgtcaatccgactctacttttcctaaaaattccagcacaaaatgccataagcaccacattcccttatactggagatcctccatacagccatggaacaggaacaggatacaccatggacacagtaaacagaacacaccaatactcagaaaagggaaagtggacaacaaacacagagactggtgcaccccagctcaacccgattgatggaccactacctgaggataatgaaccaagtgggtatgcacaaacagactgtgttctagaggctatggctttccttgaagaatcccacccaggaatatttgagaattcatgccttgaaacaatggaagttgttcaacaaacaagggtagataaactgactcaaggtcgccagacttatgattggacattaaacagaaatcaaccggcagcaactgcattggccaacaccatagaagtctttagatcgaatgacctaatagctaacgagtcaggaaggctaatagatttcttaaaggatgtaatggaatcaatgaacaaagaggaaatagagataacaacccactttcaaagaaaaagaagagtaagagacaacatgaccaagaagatggtcacgcaaagaacaataggtaagaaaaaacagagactgaataagagaggctatctaataagagcactgacattaaatacgatgaccaaagatgcagagagaggcaagttaaaaagaagggctatcgcaacacctgggatgcagattagaggtttcgtatactttgttgaaactttagctaggagcatttgcgaaaagcttgaacagtctgggctcccagtagggggcaatgaaaagaaggccaaattggcaaatgttgtgagaaagatgatgactaattcacaagatacagaaatttctttcacaatcactggggacaacactaagtggaatgaaaatcaaaatcctcgaatgttcctggcgatgattacatatatcaccagaaatcaacccgagtggtttagaaacatcctgagcatggcacccataatgttctcaaataaaatggcaagactagggaaagggtacatgttcgagagtaaaagaatgaagattcgaacacaaataccagcagaaatgctagcaaacattgatctgaagtacttcaatgaatcaacaaagaagaaaattgagaaaataaggcctcttttaatggatggcacagcatcactgagtcctgggatgatgatgggcatgttcaacatgctaagtacggtcttgggagtctcgatactgaatcttggacaaaagaaatacaccaagacaacatactggtgggatgggctccaatcatccgacgattttgctctcatagtgaatgcaccaaaccatgaaggaatacaagcaggagtggacagattctacaggacctgcaagttagtgggaatcaacatgagcaaaaagaaatcctatataaataagacagggacatttgaattcacaagctttttttatcgctatggatttgtggctaattttagcatggagctacccagctttggagtgtctggagtaaatgaatcagctgacatgagtattggagtaacagtgataaagaacaacatgataaacaatgaccttggacctgcaacggcccagatggctcttcaattgttcatcaaagactacagatacacatataggtgccataggggagacacacaaattcagacaagaagatcatttgagttaaagaagctatgggatcagacccaatcaaaggtagggctattagtatcagatggaggaccaaacttatacaacatacggaatcttcacattcctgaagtctgcttaaaatgggagctaatggatgatgattatcggggaagactttgtaatcccctgaatccctttgtgagtcataaggagattgattctgtaaacaatgctgtggtaatcccagcccatggtccagccaaaagcatggaatatgatgccgttgcaactacacattcctggattcccaagaggaatcgttctattctcaacacaagccaaaggggaattcttgaggatgaacagatgtaccagaagtgttgcaacctattcgagaaatttttccctagcagttcatataggagaccggttggaatttctagcatggtggaggccatggtgtctagggcccggattgatgccagggtcgactttgagtctggacggatcaagaaagaagagttctctgagatcatgaagatctgttccaccattgaagaactcagacggcaaaaataa

>H1N1_PB1_KM013740_A/Bangkok/SIMI505/2010
[truncated: 57,559,105 more chars]
